# Supplementary material for: Complex‐centric proteome profiling by SEC‐SWATH‐MS
Source: Mol Syst Biol. 2019 Jan 14;15(1):e8438. doi: 10.15252/msb.20188438 (PMC6346213; doi:10.15252/msb.20188438)
Supplement: Supplementary file 1 — Appendix [file MSB-15-e8438-s001.pdf]

Complex-centric proteome profiling by SEC-SWATH-MS

Contents

Appendix Figures and Captions 2

Appendix Figure S1: Properties of the SEC elution profiles. 3

Appendix Figure S2: Protein detection based on independent proteotypic peptides........ 5

Appendix Figure S3: Parameter optimization and benchmarking details........ 7

Appendix Figure S4: Molecular weight calibration and enrichment analysis. 9

Appendix Figure S5: Exemplary feature collapsing and septin complex stoichiometry. 11

Appendix Figure S6: Protein intensity profiles of workflow replicates. 12

Appendix Figure S7: Gaussian deconvolution mixture model 14

Concepts of the *CCprofiler* framework and algorithm 16

SEC-informed data filtering and protein FDR control 16

Target and decoy complex query set generation 16

Elution feature finding 17

Coelution score calculation and statistical FDR control 18

Collapsing of redundant co-elution evidence into unique features 19

References 20

CCprofiler Vignette........ 21

Appendix Figures and Captions

Appendix Figure S1: Properties of the SEC elution profiles.

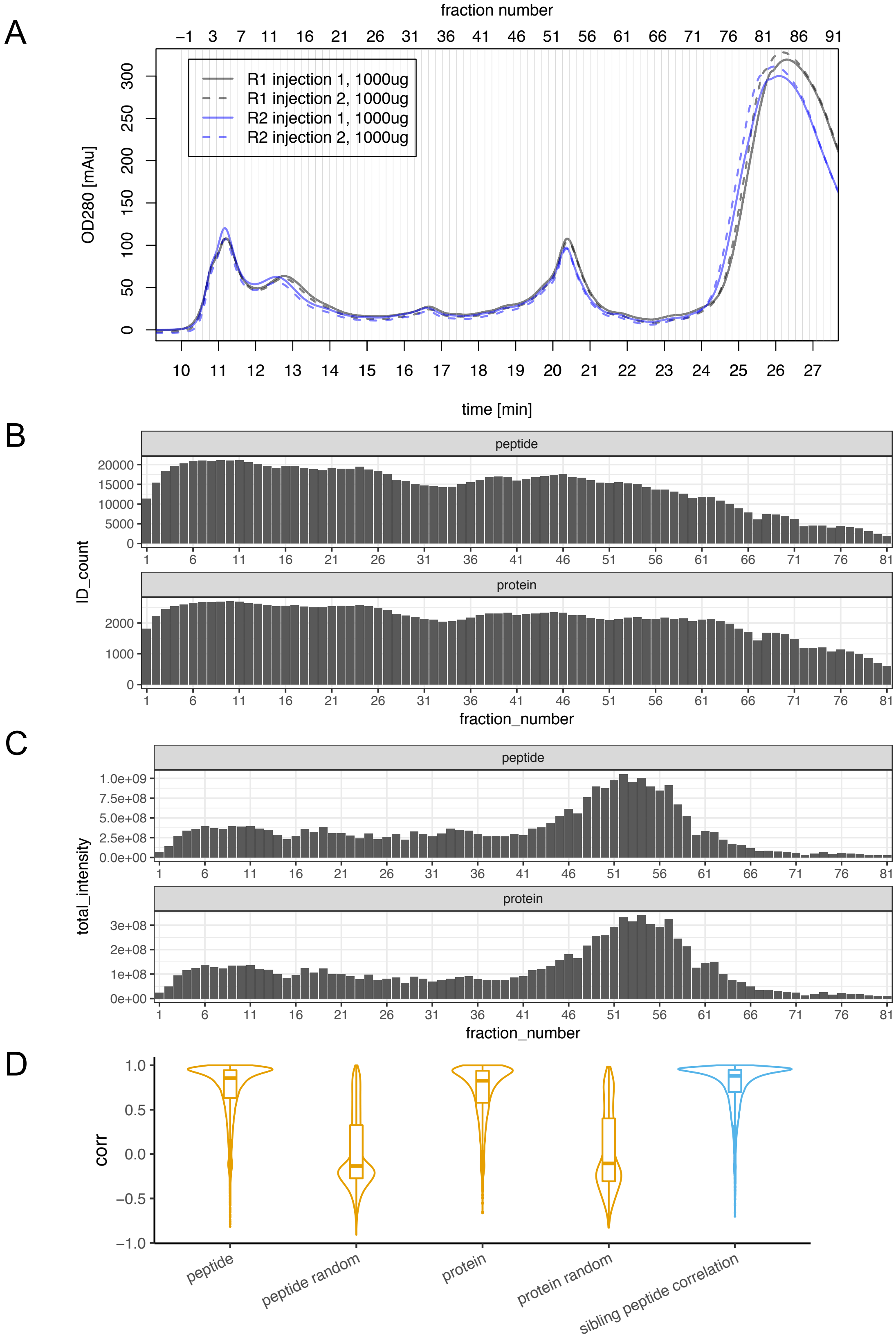

Appendix Figure S1: Properties of the SEC elution profiles. A The OD280 profile along the sampled SEC fractions for both replicates R1 and R2. Fractions collected from two consecutive, well-reproducible separations (injections 1 and 2) of 1000 ug mild lysate each were pooled for downstream analyses. The first fraction that was measured by mass spectrometry for replicate R1 was labeled as fraction 1. Any prior fractions are labeled ≤0. **B** The total number of peptide and protein counts along the SEC fractions after 1% protein FDR filtering in CCprofiler (replicate R1). **C** The total cumulative peptide- and protein-level raw MS intensity profile along the SEC fractions after 1% protein FDR filtering in CCprofiler (replicate R1). **D** Assessment of SEC-SWATH-MS workflow reproducibility based on replicate peptide and protein correlation (fractions 23-46). True replicate correlations are compared to random peptide or protein correlation pairs. The combined sibling peptide correlation distribution is further depicted as a reference point for comparison.

Appendix Figure S2: Protein detection based on independent proteotypic peptides.

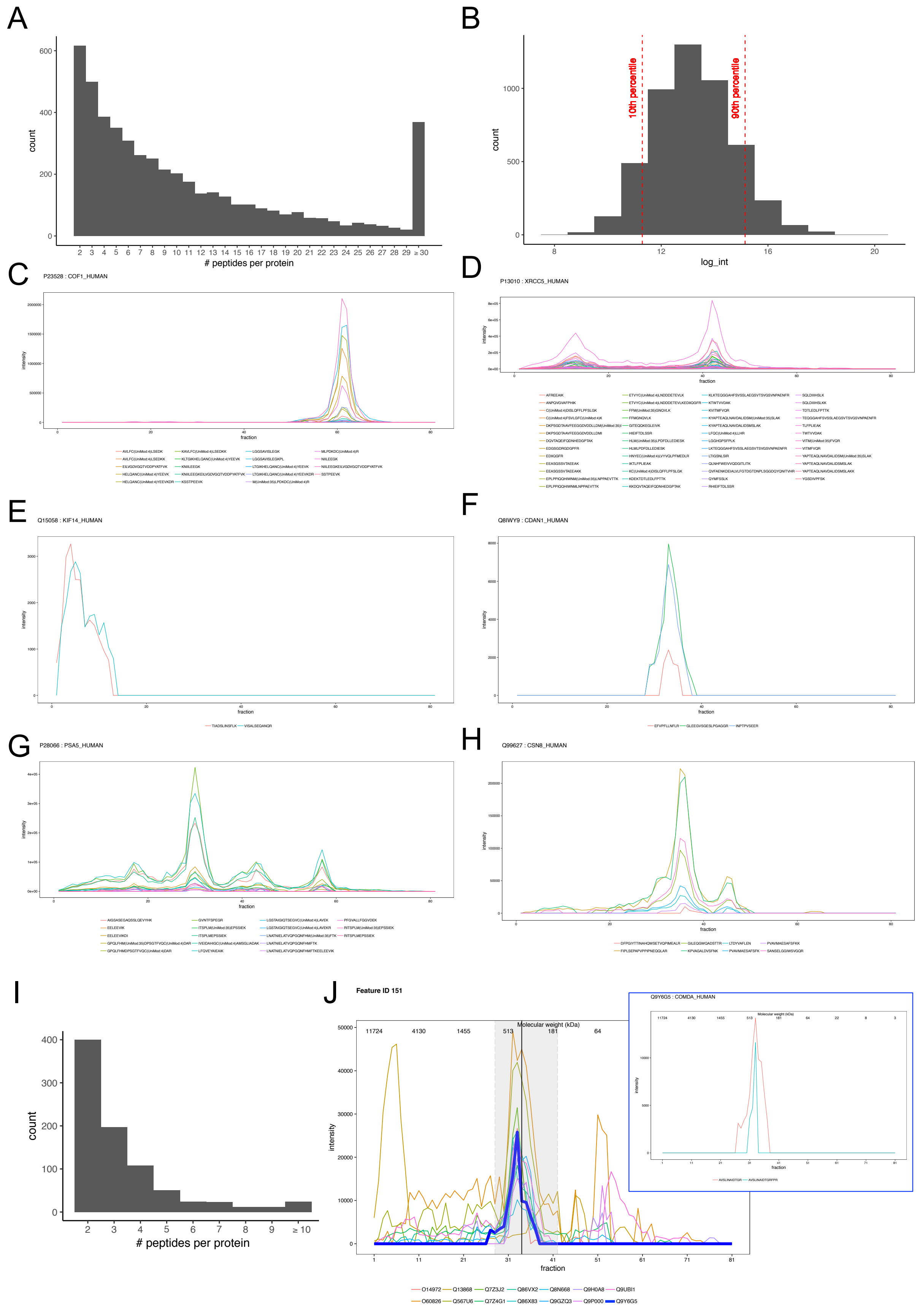

Appendix Figure S2: Protein detection based on independent proteotypic peptides. A Histogram showing number of proteotypic peptides detected per protein. **B** Histogram of the log2 intensities of all detected proteins, as inferred by summing the top two most intense proteotypic peptide signals. Red dashed lines indicate the lowest 10th and highest 10th percentile of intensities. **C** and **D** depict the peptide intensity profiles for two randomly selected proteins form the highest 10th percentile of proteins. **E** and **F** depict the peptide intensity profiles for two randomly selected proteins form the lowest 10th percentile of proteins. **I** Histogram showing number of proteotypic peptides detected per protein, including only proteins for which no protein feature was detected by protein-centric analysis. **J** Protein profiles of a randomly selected collapsed complex elution feature, detecting a protein as co-eluting subunit (left, protein profile highlighted in bold blue) for which no high confidence protein elution feature was detected by protein-centric analysis of its proteotypic peptides' SEC profiles (blue box).

Appendix Figure S3: Parameter optimization and benchmarking details.

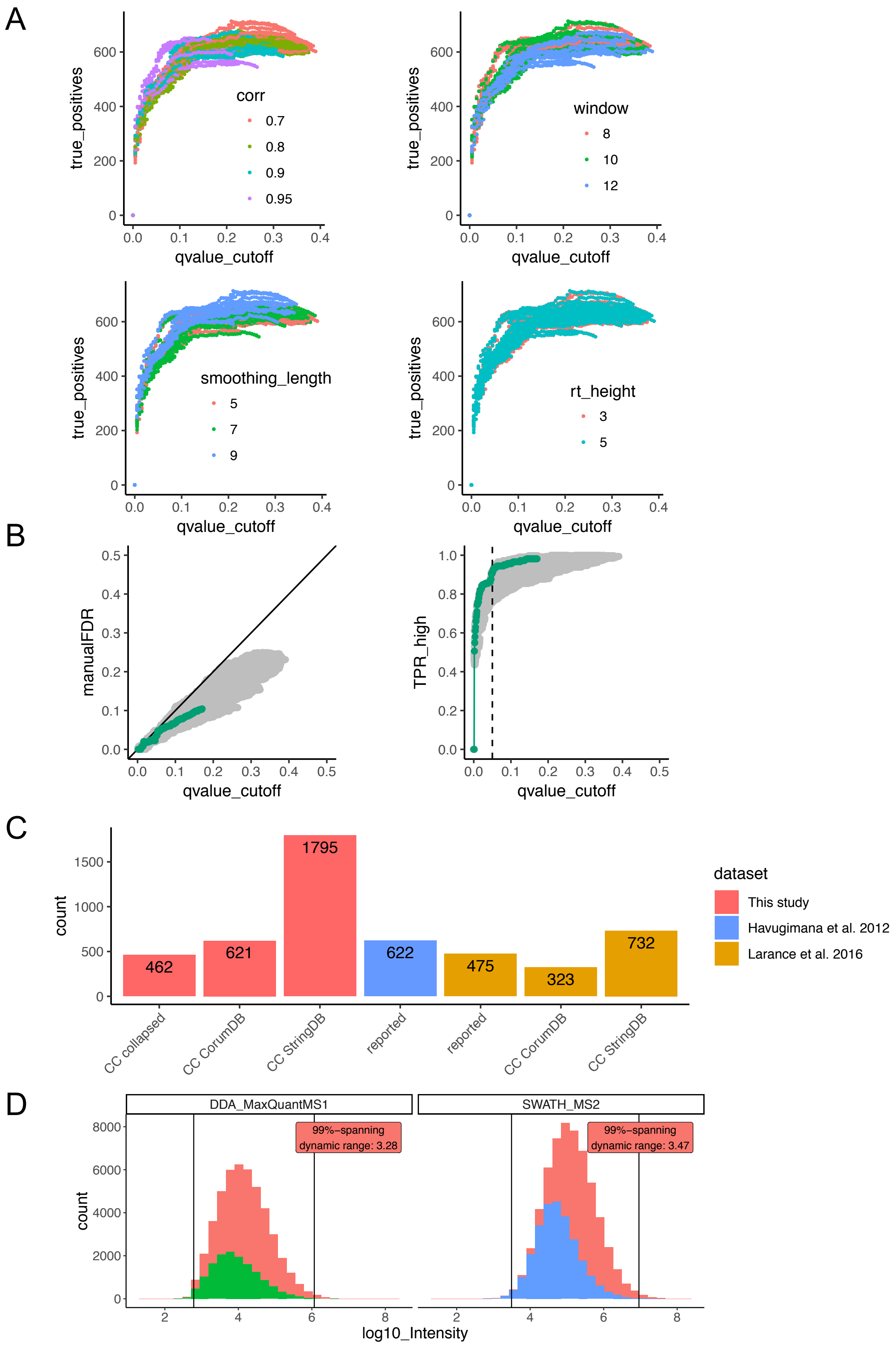

Appendix Figure S3: Parameter optimization and benchmarking details. A Visualization of the impact of different parameters that were tested in the grid search for optimal parameters in complex-centric profiling. Depicted is the number of estimated true positive protein complex subunit co-elution signals over the q-value cutoff (equivalent to the FDR). **B** Visualization of the parameter space evaluated in the grid search with reference to the manual annotation benchmark. The green data points depict the results obtained when employing the optimal parameter set selected in the grid search. **C** Comparison of reported complex numbers based on different datasets and analysis approaches. The reported complexes from this study are shown in red, separating between the results reported after collapsing of the complex signals detected by using CorumDB, BioPlex and StringDB as priors (CC collapsed), using only CorumDB as prior connectivity information (CCorumDB), or only StringDB as prior connectivity information (CC StringDB). The number of complexes reported in the original study by Havugimana et al. (2012) are depicted in blue. Further, numbers of complexes retrieved from the SEC-MS/MS dataset by Larance et al. (2016) are shown in orange. The complexes reported in the original study (Larance et al. 2016 (reported)), as well as the results of complex-centric analysis of their native SEC-MS/MS data using only CorumDB (CC CorumDB) or StringDB (CC StringDB) as prior connectivity information are shown. **D** Histogram of log10 peptide intensities from DDA data quantified based on MS1 signals and DIA data quantified based on MS2 signals. The peptide intensities were calculated by summing peptide intensities across the SEC dimension. For quantitative comparison of the dynamic range of quantification the range spanned by 99 % of the quantitative values derived by both quantification strategies is depicted. Peptides detected with both strategies are depicted in red. Peptides only detected by DDA or DIA are depicted in green and blue respectively. The number of peptides detected in DIA analysis only is significantly more than in DDA only and these DIA-only peptides are also more strongly concentrated than DDA-only peptides in the lowest part of the abundance range. Detection of more low abundance peptides in DIA mode than DDA mode is consistent with the larger estimated global dynamic range (3.47 versus 3.28 orders of magnitude respectively).

Appendix Figure S4: Molecular weight calibration and enrichment analysis.

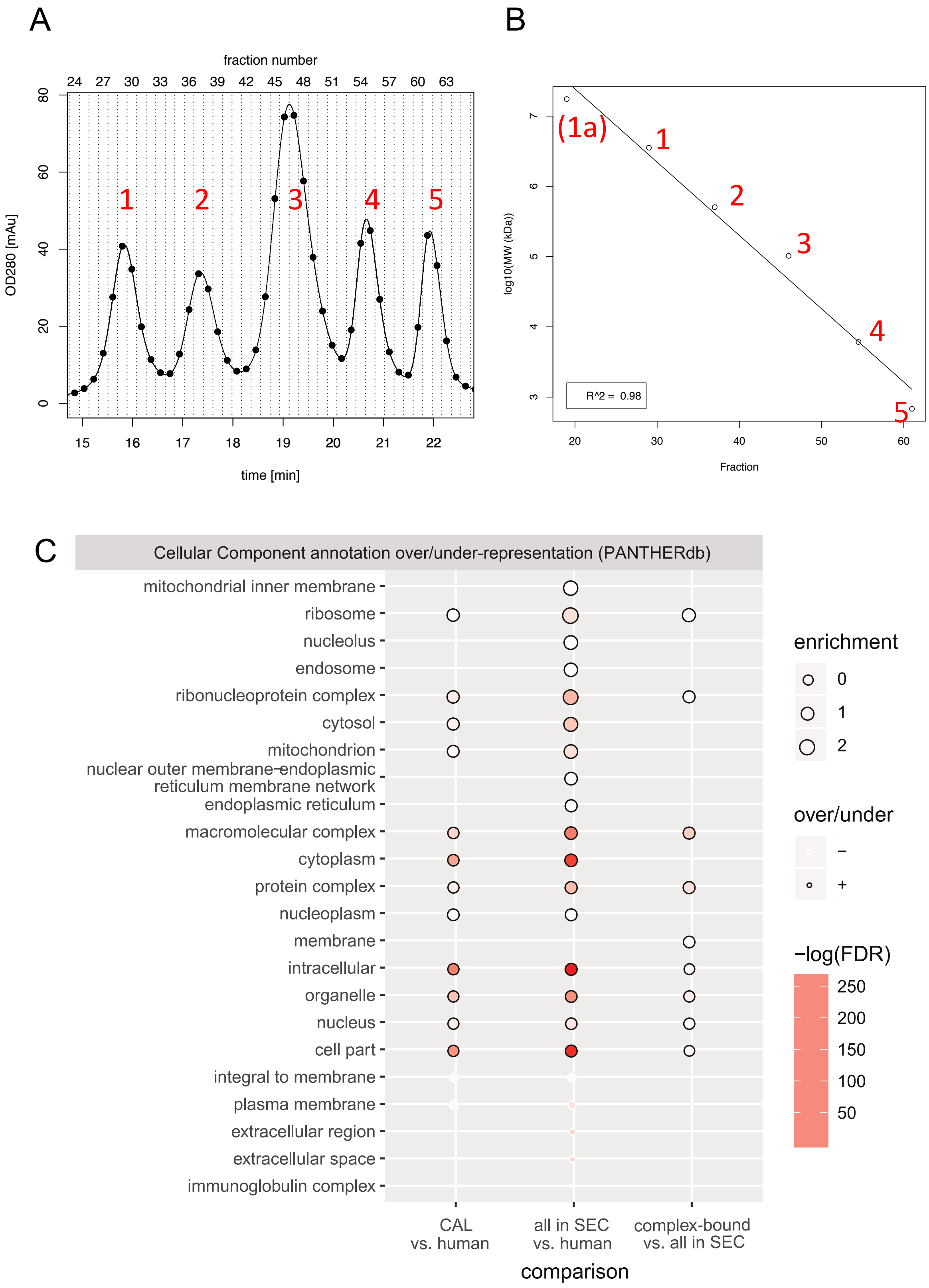

Appendix Figure S4: Molecular weight calibration and enrichment analysis. A OD280 elution profile of 5 standard proteins used for molecular weight calibration. The standard proteins and their molecular weights were: Thyroglobulin tetramer, 1398 kDa (1a); Thyroglobulin dimer, 699 kDa (1); IgA, 300 kDa (2); IgG, 150 kDa (3); Ovalbumin, 44 kDa (4); and Myoglobin, 17 kDa (5). **B** Molecular weight calibration based on the protein standards. **C** Cellular component overrepresentation testing among proteins in the combined assay library of human peptide query parameters (CAL) or those detected in SEC-SWATH-MS (SEC, each tested vs. Full Human Background) or among proteins detected as part of a complex via SEC-SWATH-MS (ComplexBound, tested vs. the set of proteins detected in SEC-SWATH-MS).

Appendix Figure S5: Exemplary feature collapsing and septin complex stoichiometry.

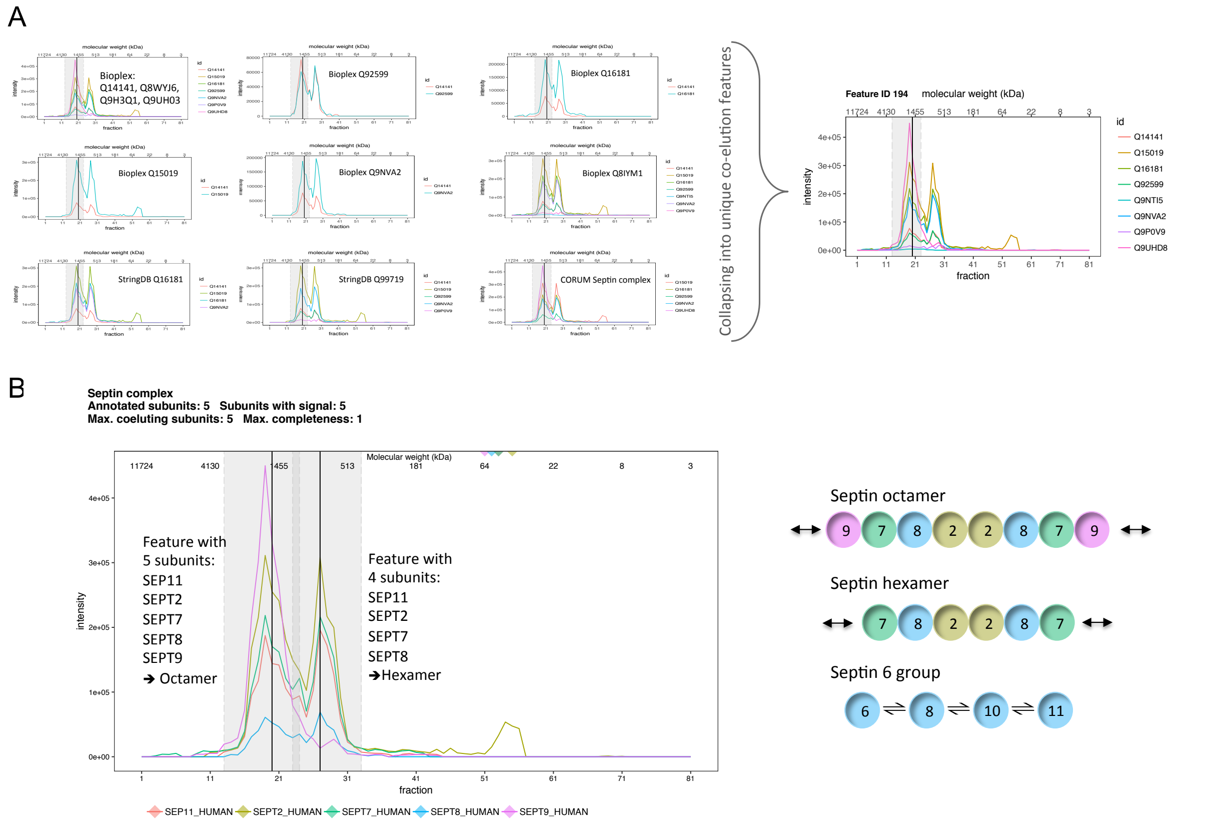

Appendix Figure S5: Exemplary feature collapsing and septin complex stoichiometry. A Example of how redundant and overlapping protein complex elution signals are collapsed into unique complex signals. Left panels, complex features that were detected based on different complex hypotheses queries generated from CORUM, Bioplex or StringDB. Right panel, the product of collapsing the co-elution evidence from the left panels into a non-redundant complex elution signal (feature ID 194). The nomenclature of the complex queries generated from Bioplex and StringDB is based on the UniProtKB identifier of the protein that was used to generate the respective query by selecting all its direct neighbors in the interaction network. The graph on the top left depicts a feature that was detected based on four distinct complex hypotheses generated from the Bioplex interaction network. **B** Example of label-free MS intensity conforming with expected stoichiometry. Protein intensity profiles of the septin protein subunits annotated in CorumDB display two distinct complex elution features in line with two known modi of Septin complex appearance as hexa- and octamer(Neubauer & Zieger, 2017). Obligate core subunits of the assemblies consistently display higher MS intensity compared to the facultative proteins of the septin 6 group (SEPT8 and SEP11) that alternatively occupy one site of the structure and are thus each present only in substoichiometric amounts compared to the obligate core subunits.

Appendix Figure S6: Protein intensity profiles of workflow replicates.

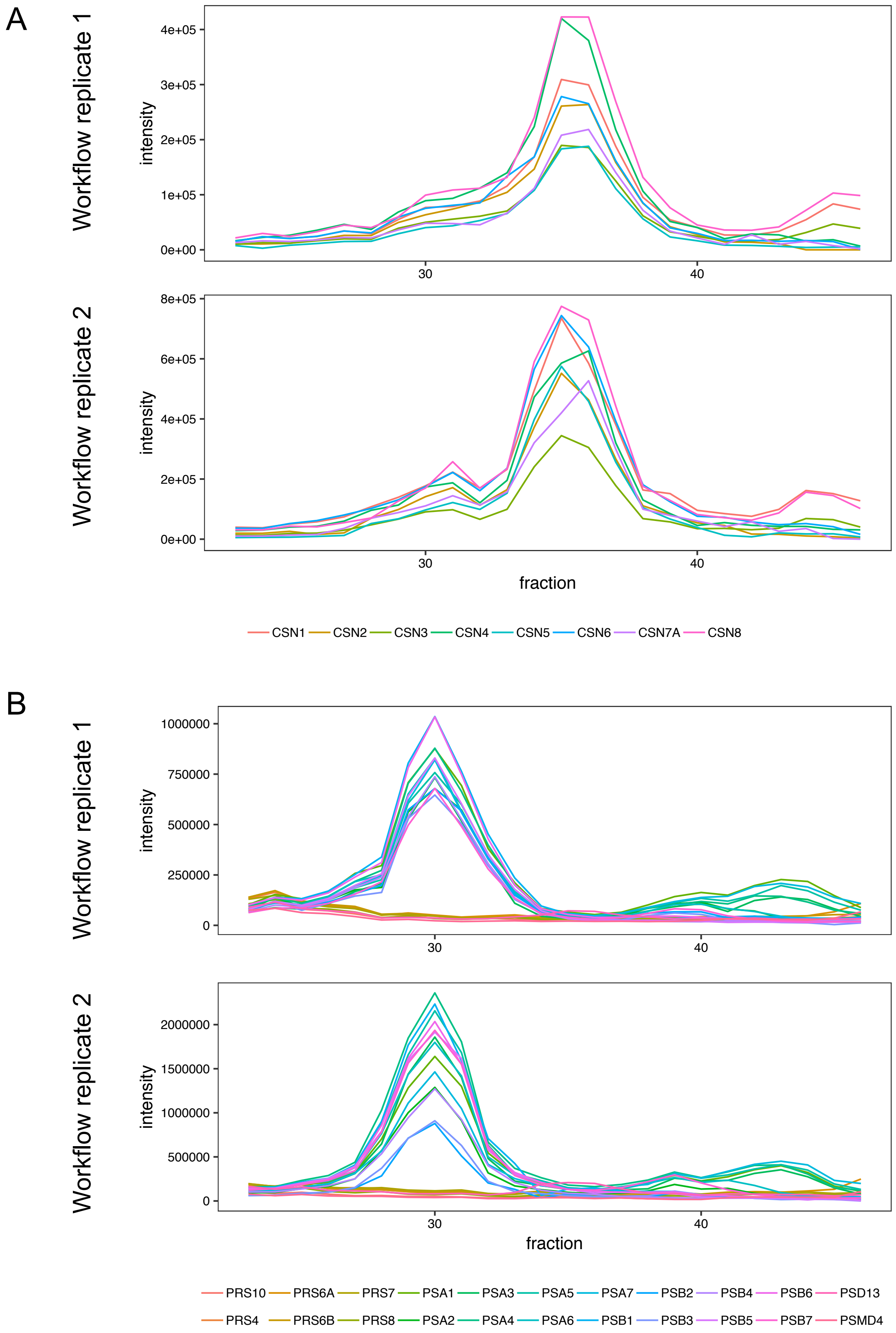

Appendix Figure S6: Protein intensity profiles for all subunits of the COP9 signalosome and proteasome complexes for both workflow replicates. A The protein intensity profiles for the eight canonical subunits of the COP9 signalosome complex in workflow replicate 1 and 2, fractions 23-46. Related to Figure 5. **B** The protein intensity profiles for all proteasome complex members in workflow replicate 1 and 2, fractions 23-46. Related to Figure 6.

Appendix Figure S7: Gaussian deconvolution mixture model

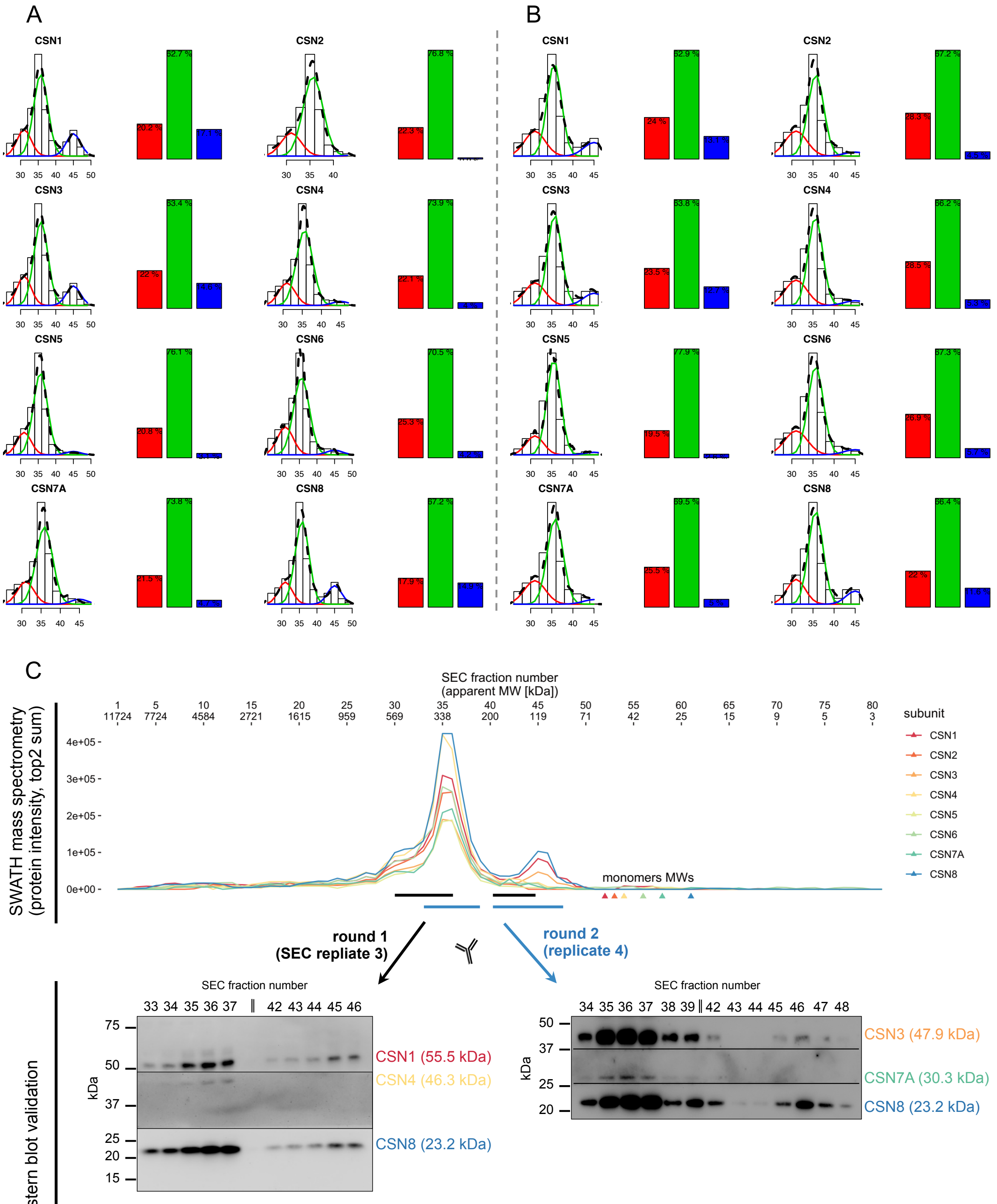

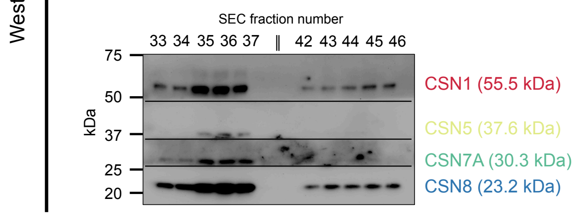

CSN subunit	~340 kDa signal		~120 kDa complex	
	mass spectr.	western blot	mass spectr.	western blot
1	x	x	x	x
2	x	n.d.	-	n.d.
3	x	x	x	x
4	x	x	-	-
5	x	x	-	-
6	x	n.d.	-	n.d.
7(A)	x	x	-	-
8	x	x	x	x

x = peak/bands detected, n.d. = not determined, - = no peak/bands detected

Appendix Figure S7: Estimating protein mass distribution across COP9 Signalosome variants by Gaussian deconvolution. For the 8 subunits of COP9 Signalosome, Gaussian mixture modeling deconstitutes the observed SEC profiles (dashed lines) into the component signals contributed by different underlying complex variants eluting from the SEC column (red, green and blue curves). Mixture models were calculated under restraint of the component means and standard deviations using R/normalmixem. Bar plots give quantitative estimates of protein subunit mass distribution across three manually assigned variants of COP9 Signalosome (CSN holocomplex; green bar and CSN1/3/8; blue bar, compare Figure 5 and CSN holocomplex bound to substrate E3 CRLs, compare Figure 7, red bar). The models suggest a substrate-bound fraction , across the 8 subunits, of of 22 ± 3 % (Replicate 1, Panel **A**) and 25 ± 4 % (Replicate 2, Panel **B**). **C** Western blot validation of distinct elution behavior of holo-CSN (CSN1/3/8) and shared (CSN4/5/7A) subunits. Related to Figure 5 and Figure 7.

Concepts of the *CCprofiler* framework and algorithm

SEC-informed data filtering and protein FDR control

In order to achieve optimal data quality and sensitivity in complex-centric profiling, CCprofiler features upstream data filtering strategies to leverage the conformance of quantitative profiles of sibling peptides and the consistency of analyte retrieval as additional information to validate peptide and protein elution signals in SEC chromatography. Protein (and their fragment peptide) elution signals in SEC-SWATH-MS can be expected to conform to biophysical constraints imposed by size exclusion chromatography and experimental design. First, a valid elution event can be expected to consist of a number of consecutive quantifications as function of the peak capacity of the chromatographic separation. Second, multiple peptides originating from the same protein should display highly similar quantitative elution profiles along the chromatographic dimension, given that the proteins are presumably intact during separation and are only cleaved into peptides for LC-MS/MS analysis thereafter. To ensure maximal sensitivity of analyte retrieval, peptide signals are generated under relatively relaxed FDR and related score cut-off criteria. Then, signals are filtered employing to the SEC-informed criteria while controlling the protein-level error rate based on the target-decoy rationale. We filter the peptide-level data based on SEC-informed filters regarding (i) the length of coherent identification stretches along consecutive SEC fractions and (ii) peptides' quantitative fractionation pattern similarity to those of its sibling peptides (originating from the same parent protein). We monitor the impact of filtering on the decoy-estimated FDR on protein level by the target-decoy approach (TDA)(Choi & Nesvizhskii, 2008) while accounting for the fraction of false targets on the protein level, also referred to as percentage of incorrect targets (PIT(Käll *et al*, 2008)) or [pi0(Storey, 2002)].

Target and decoy complex query set generation

A crucial step in complex-centric proteome profiling is the definition of target complex queries. These can either be provided directly by the user or be generated from a protein-protein interaction (PPI) network. An exemplary set of target complex queries are the complexes represented in CORUM(Ruepp *et al*, 2010), available within the package as corumComplexHypothesesRedundant. If a PPI network should be used for complex query set generation, a table with all binary interactions can be provided to *CCprofiler*. To generate complex queries from the binary interaction table, the shortest path between any two nodes of the interaction network is calculated (*calculatePathlength*) employing network functions from the R/igraph package (https://cran.r-project.org/web/packages/igraph/igraph.pdf). The resulting distance information is used to generate target complex queries *(generateComplexTargets*) based on a maximum distance cutoff, taking each

protein in the network as a complex query seed to which all proteins within a specified distance are added (e.g. max_distance = 1: all proteins with a maximum path length of 1 to the complex query seed are included in the complex query). This procedure results in as many complex queries as there are proteins in the network. Depending on the topology of the input network, this complex query set is likely to be highly redundant. Therefore, a redundancy cutoff can be selected to prevent the generation of multiple redundant complex complex queries (redundancy_cutoff = 0: all identical complex queries are collapsed, redundancy_cutoff = 1: all identical complex queries and complex query subsets are collapsed).

In order to enable an automated error-estimation of the complex-centric feature finding, a decoy model is implemented in *CCprofiler* (*generateComplexDecoys*). Decoy generation is performed taking the target complex queries as well as the pairwise distance information (pathway lengths) into account. For each target complex query, a corresponding decoy complex query of similar size is created. This is done by randomly grouping proteins that all meet the minimum network distance requirement (e.g. min_distance = 2). This prevents an accumulation of true binary interactions within a decoy complex query.

Elution feature finding

During complex feature finding, protein traces are queried for the presence of local co-eluting signals as defined in the complex queries (*findComplexFeatures*). The strategy for complex detection implemented in *CCprofiler* is based on a sliding-window approach. For each complex query, a window of specified length is moved across the fractionation dimension, testing for local co-elution of all proteins defined in the complex query, or subsets thereof. If a group of proteins passes the user defined average protein correlation within a window, as calculated by the default dist function of the R-package proxy (https://cran.r-project.org/web/packages/proxy/proxy.pdf), a complex feature is initiated.

To enable the calculation of correlation values across the entire fractionation range, missing values are replaced by imputed random noise with intensities in the lowest n*th* percentile of the entire protein quantification matrix. Initiated complex features are extended over consecutive windows until the correlation threshold is no longer met. Employing this strategy, multiple complex features can be found per complex query. For each detected complex feature, a peak detection is performed by first summing the intensities of the proteins that are part of the complex feature, followed by Savitzky-Golay smoothing across a specified range of fractions, and final peak detection using the findpeaks function of R/pracma (https://cran.r-project.org/web/packages/pracma/pracma.pdf). Complex features with no detected peak are removed from the result set. After peak detection,

complex features coming from a single complex query are reduced by merging complex features that have a peak apex distance smaller than a defined threshold and overlapping complex subunits. For each resulting merged complex feature, the average pearson correlation of all complex subunits is calculated and the complex stoichiometry is estimated by dividing the peak area of all subunits by the area of the subunit with the lowest intensity, rounding to the next integer. The final result of the *findComplexFeatures* function can potentially contain multiple complex features per queried complex query.

Coelution score calculation and statistical FDR control

The detected complex features from *findComplexFeatures* can contain protein co-elution signals of varying size and correlation. *CCprofiler* scores all these co-elution signals by using the average pearson correlation of all proteins within a feature as initial quality score. However, the quality of a feature must be judged against the chance of random observation of such a co-elution event (E.g. it is more likely that two proteins randomly co-elute, than 10 proteins). Thus, the initial quality score is further corrected for the complex query size and the actual number of proteins that were detected co-eluting within the feature. More specifically, we aim to correct for the chance that the detected number of proteins within a feature are co-eluting by chance given the size of the tested complex query.

In order to estimate the chance of random co-elution, the binomial distribution is leveraged to calculate the chance $P(x)$ of k subunits to randomly co-elute within a feature:

$$P(x = k) = \binom{n-1}{k-1} * p^{k-1} * (1-p)^{n-k} \equiv f(x)$$

Here, *n* is the number of subunits tested within the complex query, k is the number of subunits that were detected as co-eluting within the feature and *p* is the chance for a random peptide to co-elute with the detected feature (see assumptions). The binomial distribution is adjusted by subtracting one from n and k to account for the fact that at least 2 subunits need to be correlating to detect a feature.

The chance to detect the observed, or a more extreme result (i.e. a feature with $\geq k$ subunits co-eluting) can then be estimated by the cumulative distribution:

$$P(x \geq k) = \sum_{x=k}^{n} f(x)$$

Based on this model the initial correlation score can be corrected so that complex queries of different sizes can be compared. To perform the proposed score correction, the following assumptions are made:

i. The chance of a protein to randomly co-elute with a feature is proportional to the inverse correlation of proteins within a detected feature: $p = 1 - average\ feature\ correlation$.
ii. p is independent of other variables, such as the position of the feature along the SEC dimension.

We therefore define the corrected coelution score as:

$$coelution\ score = 1 - \sum_{x=k}^{n} f(x)$$

The coelution score is subsequently converted into empirical p-values and used for q-value estimation based on the *empPvals* and *qvalue* functions within the R/qvalue package (https://www.bioconductor.org/packages/devel/bioc/manuals/qvalue/man/qvalue.pdf). The scores of the decoy features are used as a null distribution. The resulting q-values can be used to filter the coelution features to achieve a target FDR on the data, e.g. if the results are filtered for a q-value smaller than 0.05, the FDR will be 5%.

Collapsing of redundant co-elution evidence into unique features

One important consideration in the complex query driven analysis of protein complexes is that the protein complex queries used for feature finding are usually not fully unique and independent, meaning that there might be some degree of redundancy in subunits that were annotated as co-complex members in multiple different complex queries. One simple example would be that complex query A consists of subunits WXYZ and complex query B consists of subunits VXYZ. If only XYZ are detected as a co-elution group in the data, they will be reported for both complex query A and B. However, since they represent the same signal, the feature collapsing algorithm will merge these two features from complex query A and B to one unique signal XYZ.

Feature collapsing is implemented within *CCprofiler* by first performing hierarchical clustering (*hclust*) of all detected complex features according to the location of the peak apex. Clusters are created based on a specified apex distance (*cutree*). Within each apex cluster, a subunit overlap score (SOscore) is calculated between all feature pairs as follows:

$$SOscore = \begin{cases} 0 & for A_{unique} = 0 \wedge B_{unique} = 0, \\ 1 + \left(A_{unique}/A_{all} * B_{unique}/B_{all}\right) & for A_{unique} \neq 0 \wedge B_{unique} \neq 0. \end{cases}$$

Here, A and B are a detected feature pair that is evaluated. A_{unique} is the number of subunits only detected in feature A (not in feature B), and A_{all} is the number of all subunits detected in A.

B_{unique} is the number of subunits only detected in feature B (not in A), and B_{all} is the number of all subunits detected in B.

This results in a SOscore of zero if feature A and B are identical and a SOscore of one if A or B are a subset of the other. If there is no subunit overlap, the SOscore will be two, and all values between 1 and 2 are a partial overlaps between the subunits of features A and B.

Based on a selected SOscore threshold, features within one apex cluster are collapsed by taking the median peak boundaries and median apex.

References

Choi H & Nesvizhskii AI (2008) False discovery rates and related statistical concepts in mass spectrometry-based proteomics. *J. Proteome Res.* **7:** 47–50

Käll L, Storey JD, MacCoss MJ & Noble WS (2008) Assigning significance to peptides identified by tandem mass spectrometry using decoy databases. *J. Proteome Res.* **7:** 29–34

Neubauer K & Zieger B (2017) The Mammalian Septin Interactome. *Front. Cell Dev. Biol.* **5:** 3

Ruepp A, Waegele B, Lechner M, Brauner B, Dunger-Kaltenbach I, Fobo G, Frishman G, Montrone C & Mewes H-W (2010) CORUM: the comprehensive resource of mammalian protein complexes--2009. *Nucleic Acids Res.* **38:** D497-501

Storey JD (2002) A direct approach to false discovery rates. *J. R. Stat. Soc. Ser. B (Statistical Methodol.* **64:** 479–498

Introduction to the *CCprofiler* package for complex-centric analysis of co-fractionation MS datasets

Isabell Bludau, Moritz Heusel, Robin Hafen, Max Frank, Amir Banaei-Esfahani and Ruedi Aebersold

2018-05-09

Contents

Overview **1**

Preparation **2**
- Installing *CCprofiler* . . . 2
- Input data . . . 2

Standard analysis workflow **3**
- Import of elution profiles to traces object . . . 3
- Quality control and filtering . . . 5
- Protein quantification . . . 6
- Protein feature finding . . . 7
- Complex feature finding . . . 13

Visualization **22**
- Peptide or protein trace visualization . . . 22
- Protein and protein complex elution feature visualization . . . 22
- Visualization of the MS coverage of all tested complex hypotheses . . . 24
- Visualization of fully and partially observed complex hypotheses . . . 26

Parameter optimization **27**
- Protein-level grid search . . . 27
- Complex-level grid search . . . 28

Session information **28**

Overview

In this vignette we present the basic functionalities of the package *CCprofiler* for complex-centric co-fractionation MS data analysis. Based on prior protein interaction data and quantitative fractionation profiles, *CCprofiler* extracts information on the presence and composition of protein-protein complexes in biological samples that have been analyzed by co-fractionation-MS (also termed protein correlation profiling, PCP). Performance is optimal in combination with high accuracy and consistency SWATH/DIA mass spectrometry. The vignette covers the example data in the package that can serve as template to format new datasets, a typical analysis workflow, as well as display of functions to visualize and inspect data and to optimize the processing parameters for a given co-fractionation-MS dataset.

Preparation

Installing *CCprofiler*

```
require(devtools)
install_github("CCprofiler/CCprofiler")
library('CCprofiler')
```

Input data

Quantitative co-fractionation MS data

CCprofiler is generically applicable to data sets generated by co-fractionation MS techniques. The main input for *CCprofiler* is a quantitative peptide- or protein-level matrix in long or wide format. There are a variety of computational processing steps upstream of *CCprofiler* that are necessary to convert raw MS files to a matrix with quantitative values of the detected peptides or proteins across all acquired fractions. The necessary information are the protein_id and/or peptide_id, filename and intensity. The exemplary data set used in this vignette is a subset of the peptide-level quantitative matrix generated from HEK293 cells analyzed by SEC-SWATH-MS (Heusel and Bludau et. al.). A subset of the quantitative data in long format is available within this package as `examplePCPdataLong`.

```
pcpData <- examplePCPdataLong
head(pcpData, n = 2)
```

```
##    protein_id        peptide_id            filename intensity
## 1:     P11021       MKETAEAYLGK heuselm_J130730_011     12188
## 2:     P12956 EVAALC(UniMod:4)R heuselm_J130730_011      4310
```

Fraction annotation table

To import the data into the *CCprofiler* container format, an annotation table mapping the biochemical fraction numbers to the respective file name is required. It is important that the fractions start at 1 and consecutively increase by 1 unit until reaching the total number of fractions. An example annotation file is available as `exampleFractionAnnotation`.

```
fractionAnnotation <- exampleFractionAnnotation
head(fractionAnnotation, n = 2)
```

```
##               filename fraction_number
## 1: heuselm_J130729_001               1
## 2: heuselm_J130729_003               2
```

Molecular weight calibration (optional)

In some co-fractionation techniques, such as size-exclusion chromatography, a log-linear relationship between the fraction dimension and molecular weight (MW) can be assumed. In such cases a MW calibration is possible by leveraging the elution information of standard proteins with known MWs run separately on the fractionation device. The inferred apparent molecular weight can then be used to judge a protein's likely assembly state given its monomeric molecular weight and observed elution fraction (~ apparent molecular weight). Monomer weights can be added to the

data by the annotation functions (see below). An example MW calibration table is available as `exampleCalibrationTable`.

```
calibrationTable <- exampleCalibrationTable
calibrationTable
```

```
##    std_weights_kDa std_elu_fractions
## 1:            1398              19.0
## 2:             699              29.0
## 3:             300              37.0
## 4:             150              46.0
## 5:              44              54.5
## 6:              17              61.0
```

```
calibration = calibrateMW(calibrationTable)
```

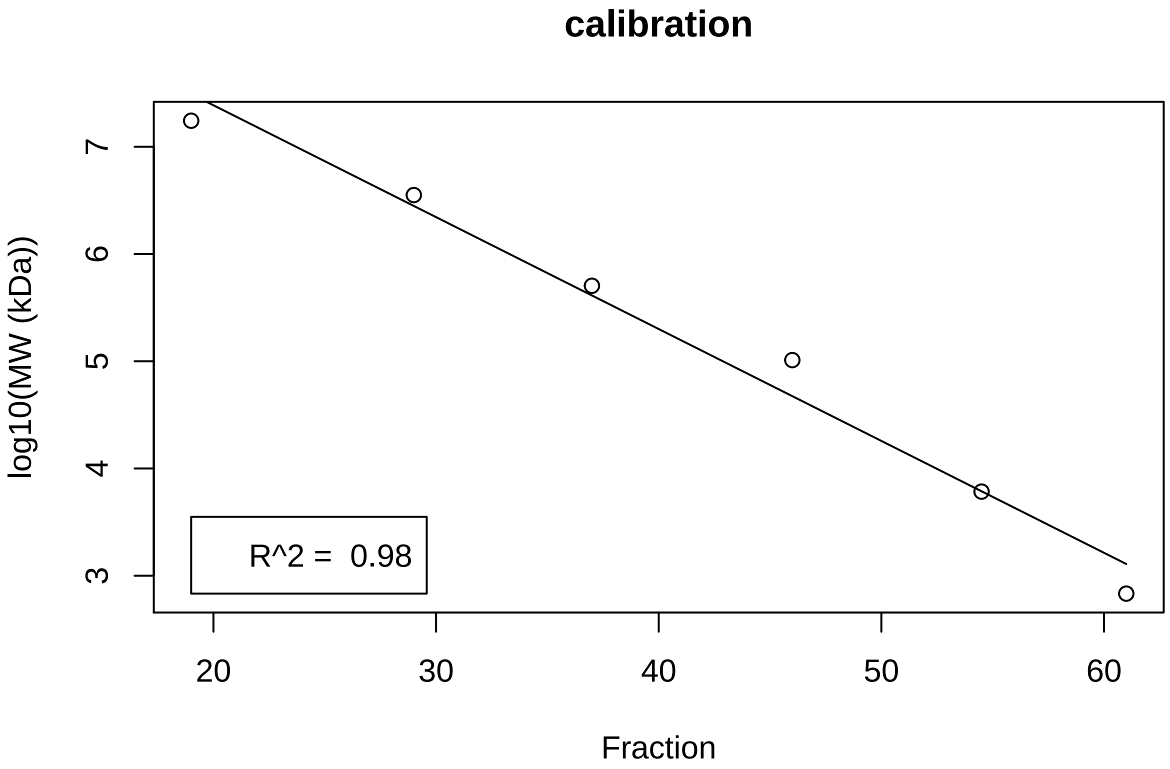

Standard analysis workflow

Import of elution profiles to traces object

The first step in any analysis with *CCprofiler* is the import of a quantitative peptide- or protein-level matrix in long or wide format that is converted to an object of class "traces". The traces data structure is a list containing four items:

- A traces data.table containing quantitative profiles of the peptides or proteins. Each row represents a peptide or protein while the columns are the consecutive fractions. The final, last column specifies the identifiers of the row (peptide or protein ids).
- A trace_type character specifying whether the quantitative profiles (traces) are of type "peptide" or "protein".

- A trace_annotation data.table containing additional information for each peptide or protein, e.g. the molecular weight of a protein.
- A fraction annotation data.table containing additional information for each fraction (column of the traces data.table), e.g. the raw MS filename of each fraction.

The trace annotation can be extended with additional information from databases such as UniProt (`exampleTraceAnnotation`). Finally each fraction can be mapped to its estimated molecular weight as determined by a calibration function. Although helpful for biological interpretation of the results, the general functionalities of *CCprofiler* do not depend on the additional trace and molecular weight annotation steps.

```
## Import quantitative PCP data into a traces object
pepTraces <- importPCPdata(input_data = pcpData,
                           fraction_annotation = fractionAnnotation,
                           rm_decoys = FALSE)

## Trace annotation table
traceAnnotation <- exampleTraceAnnotation
head(traceAnnotation, n = 2)
```

```
##     Entry Entry name   Status
## 1: P30450 1A26_HUMAN reviewed
## 2: P10314 1A32_HUMAN reviewed
##                                                                           Protein names
## 1: HLA class I histocompatibility antigen, A-26 alpha chain (MHC class I antigen A*26)
## 2: HLA class I histocompatibility antigen, A-32 alpha chain (MHC class I antigen A*32)
##    Gene names             Organism Length   Mass
## 1: HLA-A HLAA Homo sapiens (Human)    365 41,062
## 2: HLA-A HLAA Homo sapiens (Human)    365 41,048
```

```
## Annotate traces with information from uniprot
pepTraces <- annotateTraces(traces = pepTraces,
                            trace_annotation = traceAnnotation,
                            traces_id_column = "protein_id",
                            trace_annotation_id_column = "Entry",
                            trace_annotation_mass_column = "Mass",
                            uniprot_mass_format = TRUE,
                            replace_whitespace = TRUE)

## Annotate traces with molecular weight calibration (if applicable)
pepTraces <- annotateMolecularWeight(examplePeptideTraces,
                                     calibration)

## Inspect resulting annotated traces object
summary(pepTraces)
```

```
## $metrics
##  No. of Traces No. of Targets  No. of Decoys       % Decoys
##           1889           1612            277             15
##
## $type
## [1] "peptide"
##
## $annotations
##  [1] "protein_id"    "id"            "Entry_name"    "Status"
##  [5] "Protein_names" "Gene_names"    "Organism"      "Length"
##  [9] "Mass"          "protein_mw"
##
## $fraction_count
```

```
## [1] 81
```

Quality control and filtering

CCprofiler includes several options to filter for low-quality data points and noisy traces in the data. The first option is to filter the data points within a peptide trace based on consecutive identifications. A minimum stretch length of 3 means that intensity values that are not embedded in a row of at least 3 consecutive identifications are removed (i.e. set to zero). A second filter based on sibling peptide correlation calculates the mean sibling peptide correlation of each peptide to its sibling peptides. Peptides with a low sibling peptide correlation can be removed either by a fixed cutoff or by selecting an FDR cutoff that should be reached after the filtering.

```
## Filter by 3 consecutive ids
pepTraces_cons <- filterConsecutiveIdStretches(traces = pepTraces,
                                               min_stretch_length = 3)
## Filter by 0.2 sibling peptide correlation
## Note that instead of a fixed "absolute_spcCutoff" an fdr cutoff can be specified.
## The sibling peptide correlation cutoff will then be chosen to result in the specified
## FDR as estimated by decoy counting.
pepTraces_cons_sib <- filterBySibPepCorr(traces = pepTraces_cons,
                                         fdr_cutoff = NULL,
                                         absolute_spcCutoff = 0.2,
                                         plot = TRUE)
```

```
## Sibling peptide correlation not yet calculated for this dataset
## Calculating SibPepCorr(spc)...

## No fdr_cutoff provided...Using absulte_spcCutoff: 0.2

## Proteins remaining in dataset: 75
```

```
summary(pepTraces_cons_sib)
```

```
## $metrics
##  No. of Traces No. of Targets  No. of Decoys       % Decoys
##        1388.00        1384.00           4.00           0.29
##
## $type
## [1] "peptide"
##
## $annotations
##  [1] "protein_id"    "id"            "Entry_name"    "Status"
##  [5] "Protein_names" "Gene_names"    "Organism"      "Length"
##  [9] "Mass"          "protein_mw"    "SibPepCorr"    "DECOY"
##
## $fraction_count
## [1] 81
##
## $SibPepCorr_summary
##    Min. 1st Qu.  Median    Mean 3rd Qu.    Max.
##  0.2006  0.7827  0.8673  0.8203  0.9126  0.9809
```

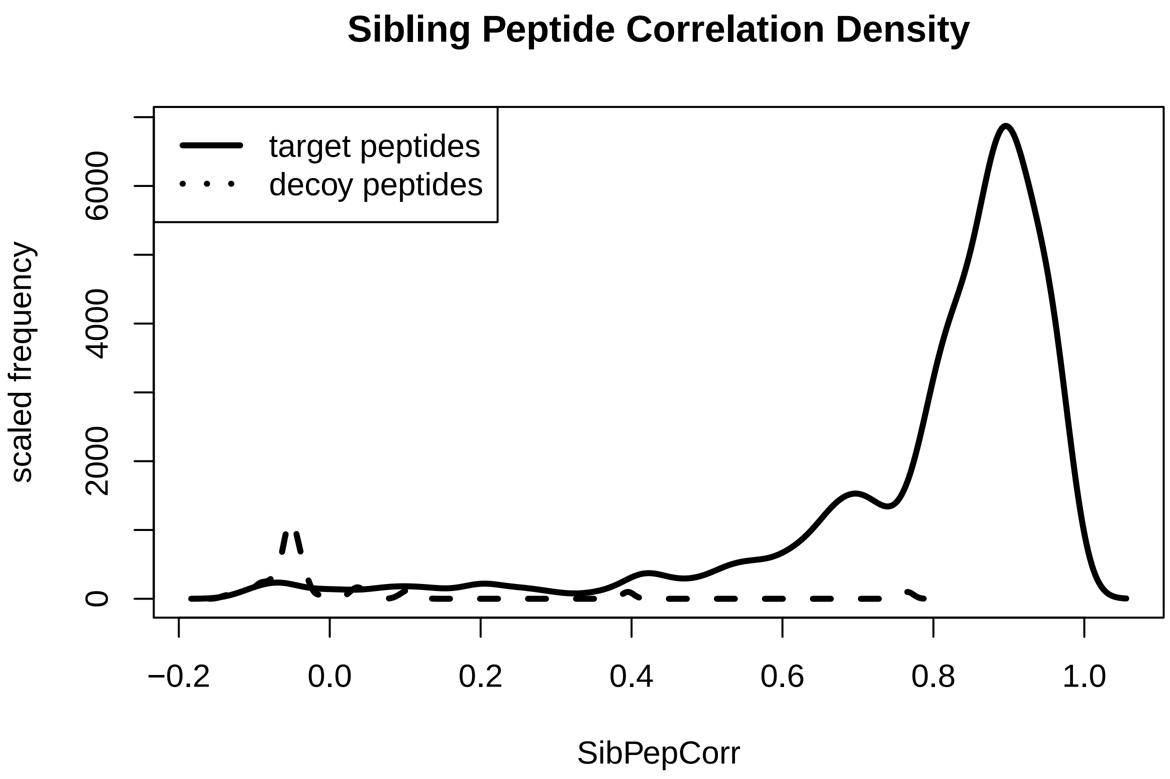

Protein quantification

The peptide traces can be used to infer protein-level traces. *CCprofiler* implements a topN strategy for summarizing peptides to a protein trace. Decoys should typically be removed at this stage.

```
protTraces <- proteinQuantification(pepTraces_cons_sib,
                                    topN = 2,
                                    keep_less = FALSE,
                                    rm_decoys = TRUE)
```

```
## 4 decoys removed
```

```
summary(protTraces)
```

```
## $metrics
##  No. of Traces No. of Targets  No. of Decoys       % Decoys
##             75             75              0              0
##
## $type
## [1] "protein"
##
## $annotations
##  [1] "id"                         "protein_id"
##  [3] "Entry_name"                 "Status"
##  [5] "Protein_names"              "Gene_names"
##  [7] "Organism"                   "Length"
##  [9] "Mass"                       "protein_mw"
## [11] "DECOY"                      "SibPepCorr_protein_mean"
## [13] "n_peptides"                 "quant_peptides_used"
##
## $fraction_count
## [1] 81
```

Protein feature finding

CCprofiler can be employed to automatically detect protein elution peaks along the SEC dimension. Protein features are detected based on local correlation among the proteolytic peptides' quantitative profiles. Peptides are grouped by parent protein identifier; no external prior knowledge is required. Protein feature finding consists of two consecutive steps: (i) co-elution signal detection followed by (ii) statistical scoring, FDR estimation and filtering. To support basic control for random effects, decoy protein queries with randomized peptide-protein mapping are employed.

Protein-centric detection of protein elution features

Protein features are determined by a sliding window strategy, where all peptides of a protein are tested for local profile correlation. If a subset of the peptides within a protein correlate better then the specified cutoff, a protein feature is initiated, followed by peak detection within the regions of high correlation. Here we perform the protein feature finding only on a subset of all proteins because this can take some time. In order to be able to estimate an error-rate, protein decoys can be generated on the fly during the feature detection step by randomly assigning peptides to a protein decoy (`useRandomDecoyModel = TRUE`).

```
testProteins = unique(pepTraces_cons_sib$trace_annotation$protein_id)[1:20]
peptideTracesSubset = subset(pepTraces_cons_sib,
                             trace_subset_ids = testProteins,
                             trace_subset_type = "protein_id")
proteinFeatures <- findProteinFeatures(traces = peptideTracesSubset,
                                       useRandomDecoyModel = TRUE)
```

```
head(proteinFeatures, n = 2)
```

```
##    protein_id protein_name
## 1:     O00161       O00161
## 2:     O00161       O00161
##                                                                                    subunits_annotated
## 1: AHQITDESLESTRR;IEEGLDQINK;ILGLAIESQDAGIK;ITNDAREDEMEENLTQVGSILGNLK;TITMLDEQK;TTWGDGGENSPC(UniMod:4)NVVSK
## 2: AHQITDESLESTRR;IEEGLDQINK;ILGLAIESQDAGIK;ITNDAREDEMEENLTQVGSILGNLK;TITMLDEQK;TTWGDGGENSPC(UniMod:4)NVVSK
##    n_subunits_annotated                    subunits_detected
## 1:                    6 ITNDAREDEMEENLTQVGSILGNLK;TITMLDEQK
## 2:                    6           IEEGLDQINK;ILGLAIESQDAGIK
##    n_subunits_detected completeness left_pp right_pp apex  apex_mw   area
## 1:                   2    0.3333333       2       16   11 4130.110  51536
## 2:                   2    0.3333333      44       58   51   63.608 132058
##    peak_corr monomer_sec monomer_mw in_complex
## 1: 0.5673578          61     23.354       TRUE
## 2: 0.9402879          61     23.354       TRUE
```

Note: On a full dataset, protein elution feature finding is a high computational effort. Therefore, we implemented parallelization of this step to speed up processing.

```
proteinFeatures <- findProteinFeatures(traces = peptideTracesSubset,
                                       useRandomDecoyModel = TRUE,
                                       n_cores = 10,
                                       parallelized = TRUE)
```

Statistical scoring, FDR estimation and filtering

The resulting protein features can be scored based on the co-elution characteristics (average peptide correlation) of the peptides within each protein feature. The co-elution score is further statistcally corrected for the number of correlating peptides out of all the MS detectable peptides. The statistically corrected co-elution score can be used to estimate q-values for each protein peak group. Filtering the protein peak groups based on a specified q-value cutoff then achieves a final result table at specified FDR (a q-value cutoff of 0.05 results in a final table with 5% FDR).

```
proteinFeaturesScored <- calculateCoelutionScore(proteinFeatures)
qvalueProteinFeaturesScored <- calculateQvalue(proteinFeaturesScored)
```

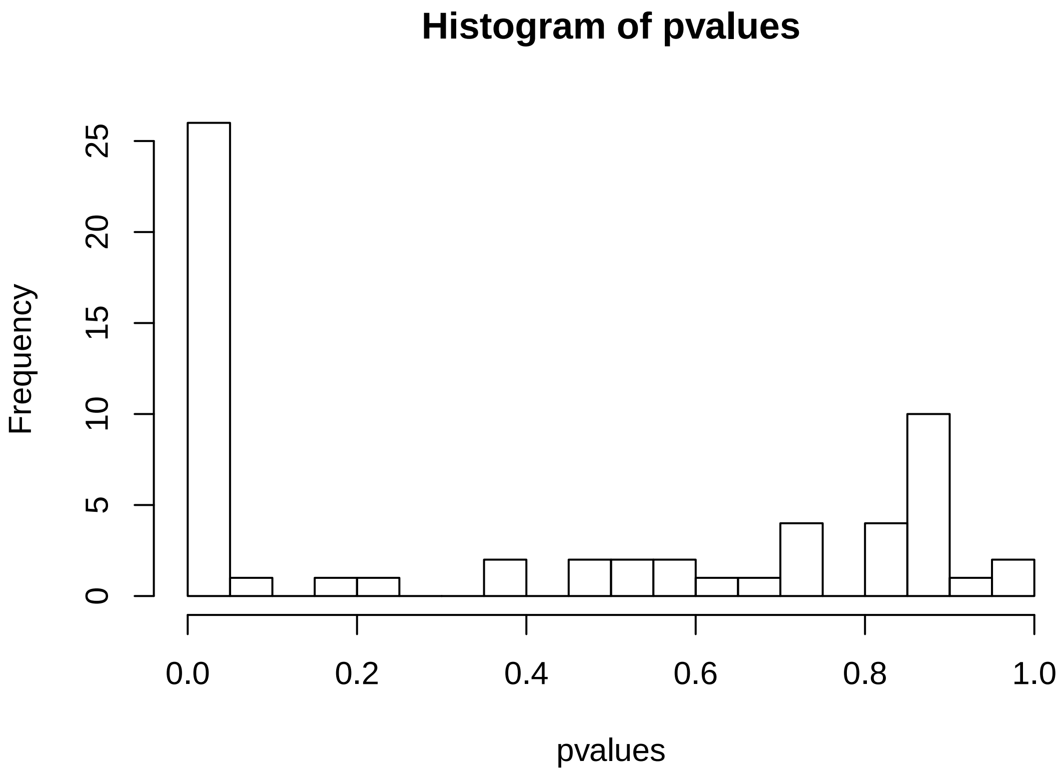

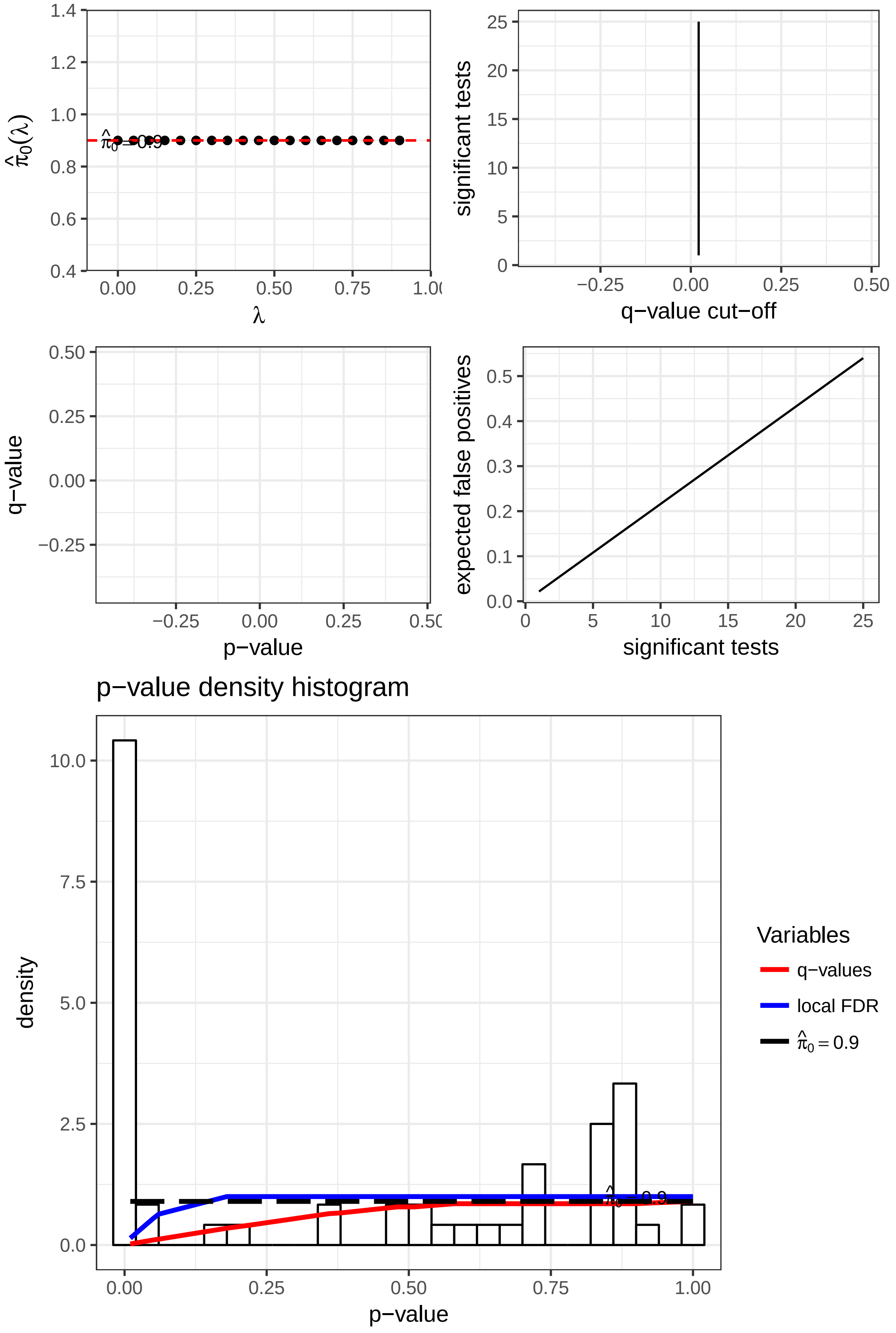

$\hat{\pi}_0(\lambda)$
λ
$\hat{\pi}_0 = 0.9$
significant tests
q−value cut−off
q−value
p−value
expected false positives
significant tests
p−value density histogram
density
p−value
Variables
q−values
local FDR
$\hat{\pi}_0 = 0.9$

```
qvalueProteinFeaturesScoredStats <- qvaluePositivesPlot(qvalueProteinFeaturesScored)
```

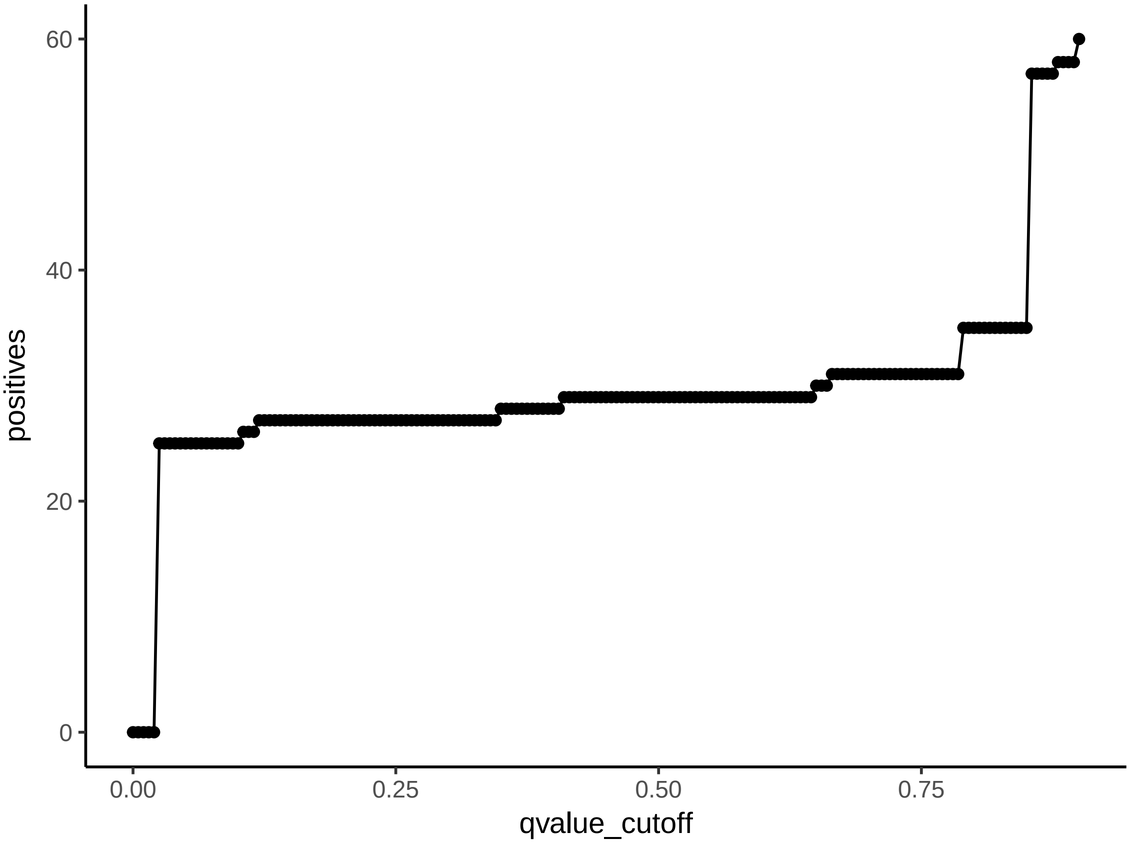

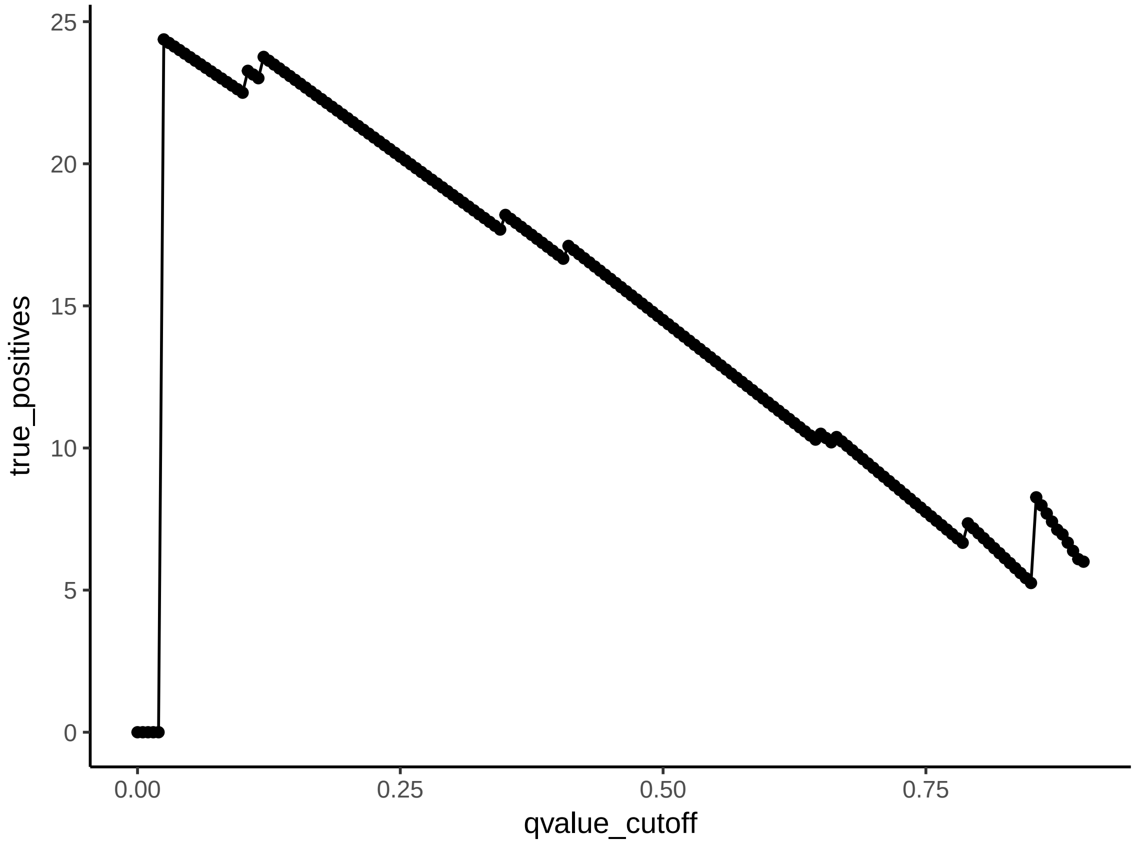


```
proteinFeaturesFiltered <- subset(qvalueProteinFeaturesScored, qvalue <= 0.05)
summarizeFeatures(proteinFeaturesFiltered)
```

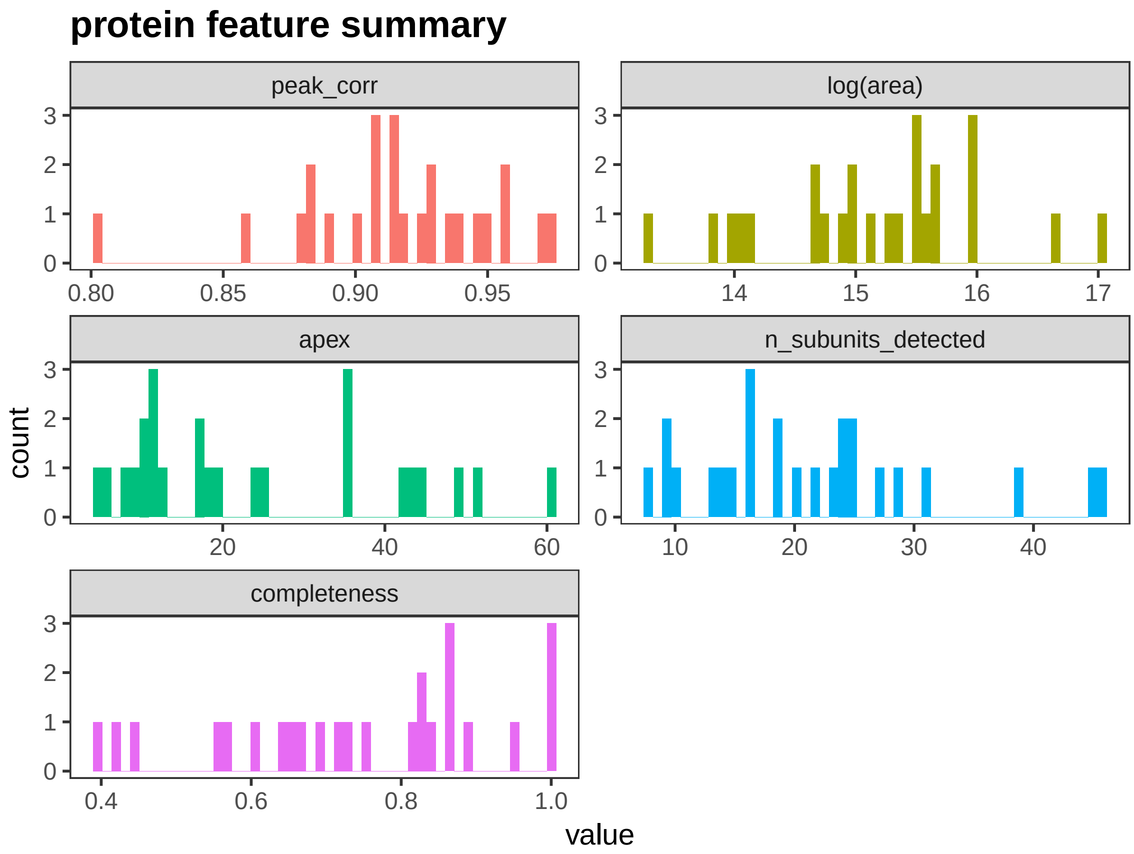
protein feature summary
peak_corr
log(area)
apex
n_subunits_detected
completeness
count
value
0
1
2
3
0.80
0.85
0.90
0.95
14
15
16
17
20
40
60
10
20
30
40
0.4
0.6
0.8
1.0

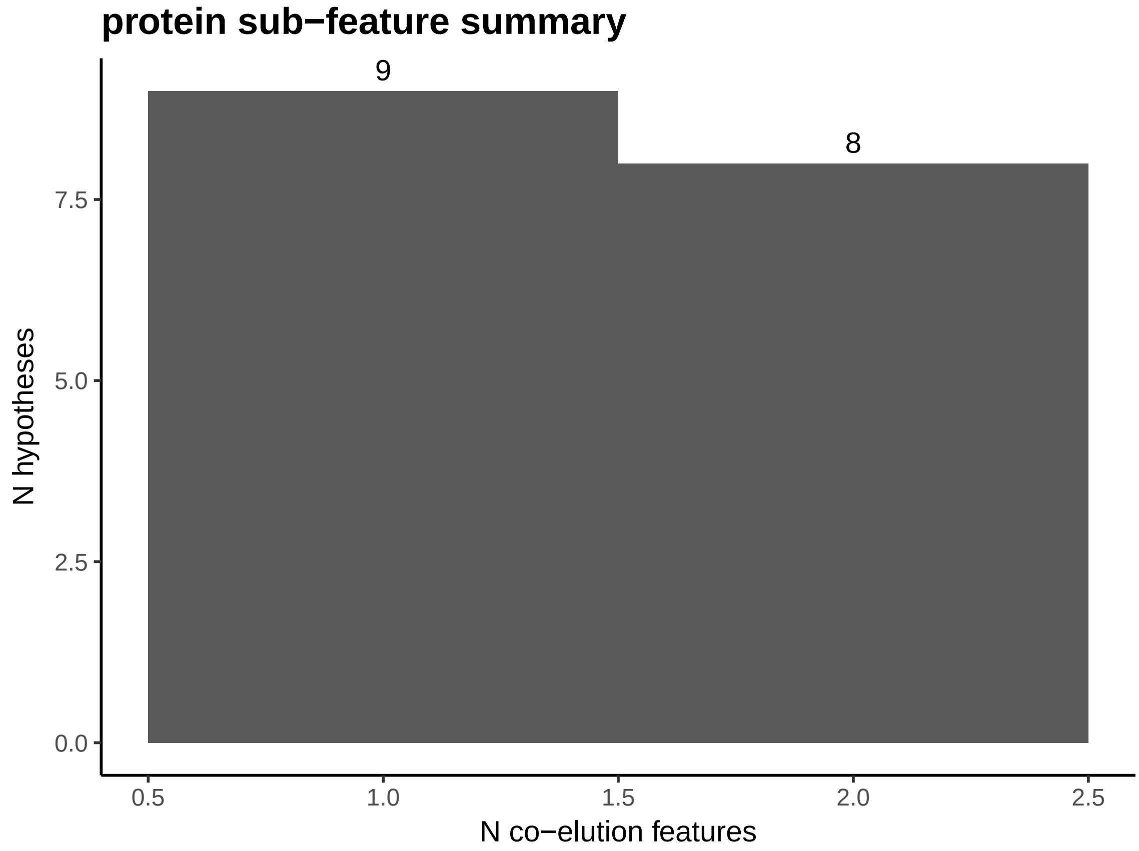
protein sub−feature summary
9
8
N hypotheses
N co−elution features
0.0
2.5
5.0
7.5
0.5
1.0
1.5
2.0
2.5

```
## $type
## [1] "protein"
##
## $totalFeatures
## [1] 25
##
## $totalConfirmedHypotheses
## [1] 17
##
## $totalHypothesesWithMultipleFeatures
## [1] 8
##
## $summaryFeatureCount
##    Min. 1st Qu.  Median    Mean 3rd Qu.    Max.
##   1.000   1.000   1.000   1.471   2.000   2.000
##
## $summaryCorrelation
##    Min. 1st Qu.  Median    Mean 3rd Qu.    Max.
##  0.8012  0.8997  0.9134  0.9152  0.9379  0.9729
##
## $summaryArea
##     Min.  1st Qu.   Median     Mean  3rd Qu.     Max.
##   575600  2347000  4377000  5437000  6417000 24300000
##
## $summaryNsubunitsAnnotated
##    Min. 1st Qu.  Median    Mean 3rd Qu.    Max.
##   11.00   19.00   27.00   30.16   41.00   52.00
##
## $summaryNsubunitsWithSignal
## Length  Class   Mode
##      0   NULL   NULL
##
## $summaryNsubunitsDetected
##    Min. 1st Qu.  Median    Mean 3rd Qu.    Max.
##    8.00   15.00   20.00   21.76   25.00   46.00
##
## $summaryCompleteness
##    Min. 1st Qu.  Median    Mean 3rd Qu.    Max.
##  0.3947  0.6429  0.7500  0.7448  0.8654  1.0000
```

Complex feature finding

Complex feature finding represents the central step of complex-centric analysis using *CCprofiler*. Based on prior protein interaction data and quantitative fractionation profiles, CCprofiler detects groups or subgroups of locally co-eluting proteins, indicating the presence of protein-protein complexes in the biological sample. Target complex queries are supplemented with decoy complex queries to support error control of the reported results. The result is a table summarizing the presence and composition of protein-protein complexes in the biological sample analyzed.

Complex query generation

In general the complex feature detection is very similar to the protein feature detection with the difference that complex hypotheses have to be generated at the beginning. This includes both target and decoy complex hypotheses for error estimation.

Preparing target complex queries

There are two options for protein complex target generation in *CCprofiler*: (i) loading of full complex models for direct use as queries (2 or more subunits, e.g. from CORUM) or (ii) loading a protein-protein interaction network from which target complex hypotheses can be extracted. The use of concrete complex hypotheses requires loading a complex query table in the following format:

```
complexHypotheses <- exampleComplexHypotheses
head(complexHypotheses)
```

```
##    complex_id                             complex_name protein_id
## 1:          1                        BCL6-HDAC4 complex     P41182
## 2:          1                        BCL6-HDAC4 complex     P56524
## 3: 10;157;167 13S condensin complex;Condensin I complex     O95347
## 4: 10;157;167 13S condensin complex;Condensin I complex     Q15003
## 5: 10;157;167 13S condensin complex;Condensin I complex     Q15021
## 6: 10;157;167 13S condensin complex;Condensin I complex     Q9BPX3
```

Alternatively target complex hypotheses can be generated from a binary protein interaction network by grouping each proteins with their neighbors. In this example, we first generate a binary interaction network from the example complexes included with the package generated above. In a real world example users could for example load data from Bioplex or StringDB. The distance between any protein pair in the network is then calculated and used for generating network-based targets.

```
binaryHypotheses <- generateBinaryNetwork(complexHypotheses)
pathLength <- calculatePathlength(binaryHypotheses)
networkTargets <- generateComplexTargets(pathLength)
head(networkTargets)
```

```
##    complex_id                     complex_name protein_id
## 1:     P41182                    P41182_P56524     P03886
## 2:     P41182                    P41182_P56524     P13010
## 3:     P41182                    P41182_P56524     P41182
## 4:     P41182                    P41182_P56524     P56524
## 5:     Q9BPX3 Q9BPX3_Q15021_O95347_Q15003_Q9NTJ3     O00161
## 6:     Q9BPX3 Q9BPX3_Q15021_O95347_Q15003_Q9NTJ3     O00267
```

Preparing decoy complex queries

Decoy complex queries are generated based on the target complex query set and its underlying network structure. The minimum distance specifies the minimal number of edges between any two proteins within any generated decoy complex query. It is important that the interacton network based on the targets is large enough to generate a random decoy set that does not overlap with the target complex queries. We recommend complex query sets of at least 1000 targets for the decoy based approach.

```
corumTargetsPlusDecoys <- generateComplexDecoys(target_hypotheses=corumComplexHypotheses,
                                                dist_info=pathLength,
                                                min_distance = 2,
                                                append=TRUE)
```

Complex-centric detection of protein co-elution features

Protein complex features are determined similar to the protein features described above. First, a sliding window strategy is applied, where all proteins of a protein complex hypothesis are tested for

local profile correlation. If a subset of the proteins within a protein complex hypothesis correlate better then the specified cutoff, a protein complex feature is initiated, followed by peak detection within the regions of high correlation.

```
complexFeatures <- findComplexFeatures(traces=protTraces,
                                       complex_hypothesis = complexHypotheses)
```

Sometimes it might is beneficial to only report the 'best' (largest numer of co-eluting protein subunits) detected complex co-elution peak group.

```
complexFeaturesBest <- getBestFeatures(complexFeatures)
```

Additionally, complex (and also protein) feature results can be filtered by different criteria. For example, if information abount the molecular weight of each measured fraction is available, one can filter out complex features that elute at a molecular weight that is lower than a certain threshold with respect to the expected monomer elution fraction (based on the molecular weights of the protein monomer molecular weights).

```
complexFeaturesFilteredMW <- filterFeatures(complexFeatures,
                                            complex_ids = NULL,
                                            protein_ids = NULL,
                                            min_feature_completeness = NULL,
                                            min_hypothesis_completeness = NULL,
                                            min_subunits = NULL,
                                            min_peak_corr = NULL,
                                            min_monomer_distance_factor = 2
                                            )
```

Statistical scoring, FDR estimation and filtering

The resulting protein complex features can be scored based on the co-elution characteristics (average protein subunit correlation) of the proteins within each protein complex feature. The co-elution score is further statistcally corrected for the number of correlating proteins out of all the proteins in the protein complex hypothesis. The statistically corrected co-elution score can be used to estimate q-values for each protein complex peak group. Filtering the protein complex peak groups based on a specified q-value cutoff then achieves a final result table at specified FDR (a q-value cutoff of 0.05 results in a final table with 5% FDR).

```
complexFeaturesScored <- calculateCoelutionScore(complexFeatures)
qvalueComplexFeaturesScored <- calculateQvalue(complexFeaturesScored)
```

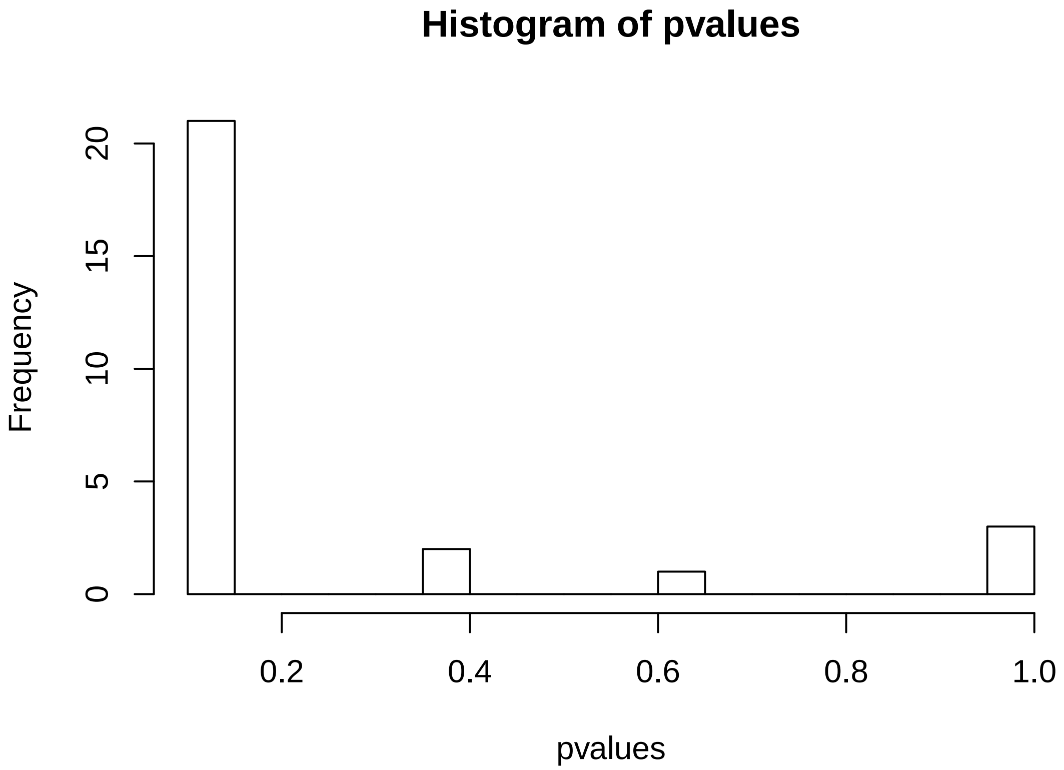
Histogram of pvalues
Frequency
0
5
10
15
20
0.2
0.4
0.6
0.8
1.0
pvalues

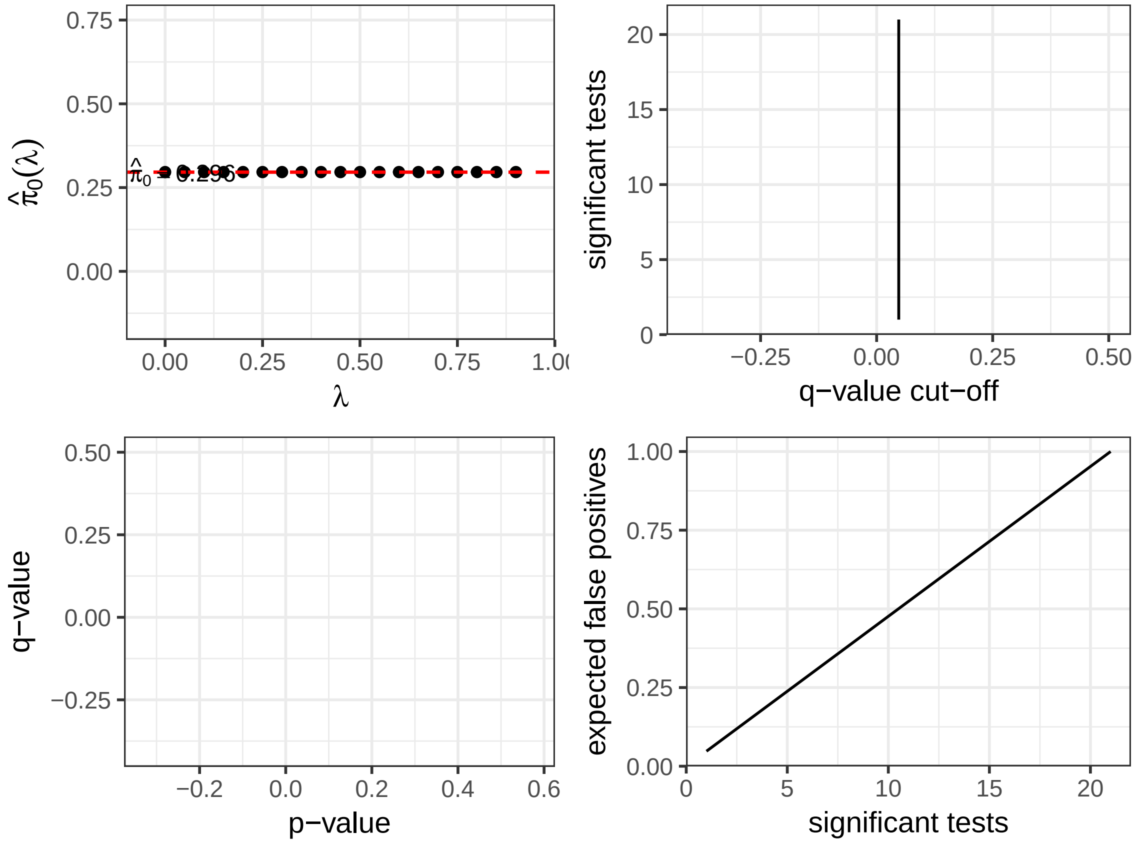
0.75
0.50
0.25
0.00
0.00
0.25
0.50
0.75
λ
significant tests
20
15
10
5
0
−0.25
0.00
0.25
0.50
q−value cut−off
q−value
0.50
0.25
0.00
−0.25
−0.2
0.0
0.2
0.4
0.6
p−value
expected false positives
1.00
0.75
0.50
0.25
0.00
0
5
10
15
20
significant tests

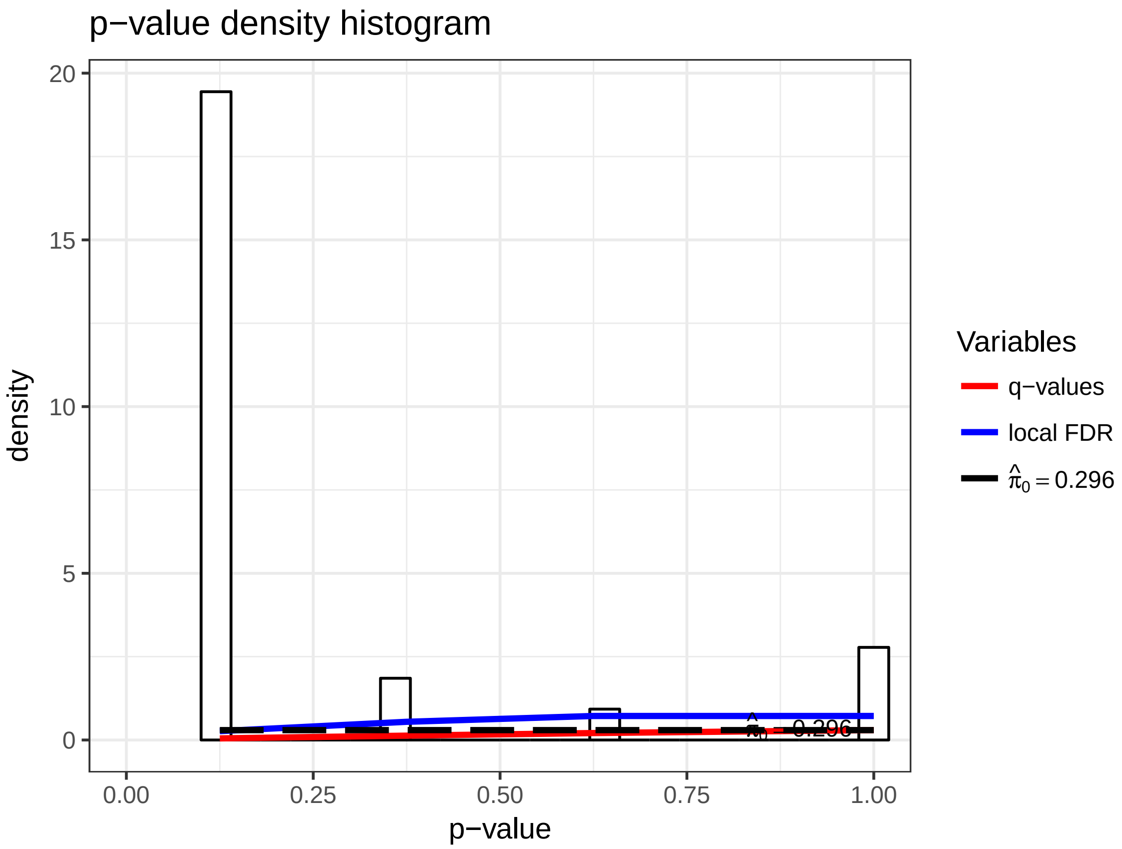


```
head(qvalueComplexFeaturesScored, n = 2)
```

```
##    complex_id                                    complex_name
## 1: 10;157;167 13S condensin complex;Condensin I complex
## 2:       1187                              ESCRT-II complex
##                 subunits_annotated n_subunits_annotated
## 1: O95347;Q15003;Q15021;Q9BPX3;Q9NTJ3                    5
## 2:               Q86VN1;Q96H20;Q9BRG1                    3
##               subunits_with_signal n_subunits_with_signal
## 1: O95347;Q15003;Q15021;Q9BPX3;Q9NTJ3                      5
## 2:               Q86VN1;Q96H20;Q9BRG1                      3
##                   subunits_detected n_subunits_detected completeness
## 1: O95347;Q15003;Q15021;Q9BPX3;Q9NTJ3                   5            1
## 2:               Q86VN1;Q96H20;Q9BRG1                   3            1
##    left_sw right_sw  sw_score left_pp right_pp apex  apex_mw    area
## 1:       1       17 0.9835337       1       16   10 4584.298 4110258
## 2:      31       66 0.9822610      42       62   49   78.368 2918071
##    peak_corr                      total_intensity
## 1: 0.9592516 1011999;575369;678807;911587;932496
## 2: 0.9825221              764020;557916;1588423
##                                                        intensity_ratio
## 1: 1.75886952546974;1;1.17977680410311;1.58435195500627;1.62069211236615
## 2:                                   1.36941761842285;1;2.84706479111551
##    stoichiometry_estimated                             monomer_mw
## 1:               2;1;1;2;2 135.656;82.563;157.182;114.334;147.182
## 2:                   1;1;3                   43.817;28.864;20.748
##       monomer_sec complex_mw_estimated complex_sec_estimated sec_diff
## 1: 44;49;42;45;43             1034.089                    24       14
## 2:       55;59;62              134.925                    44        5
##     mw_diff coelution_score decoy pvalue     qvalue
```

```
## 1: 3550.209       0.9999972     0  0.125 0.04761905
## 2:   56.557       0.9996945     0  0.125 0.04761905
```

```
qvalueComplexFeaturesScoredStats <- qvaluePositivesPlot(qvalueComplexFeaturesScored)
```

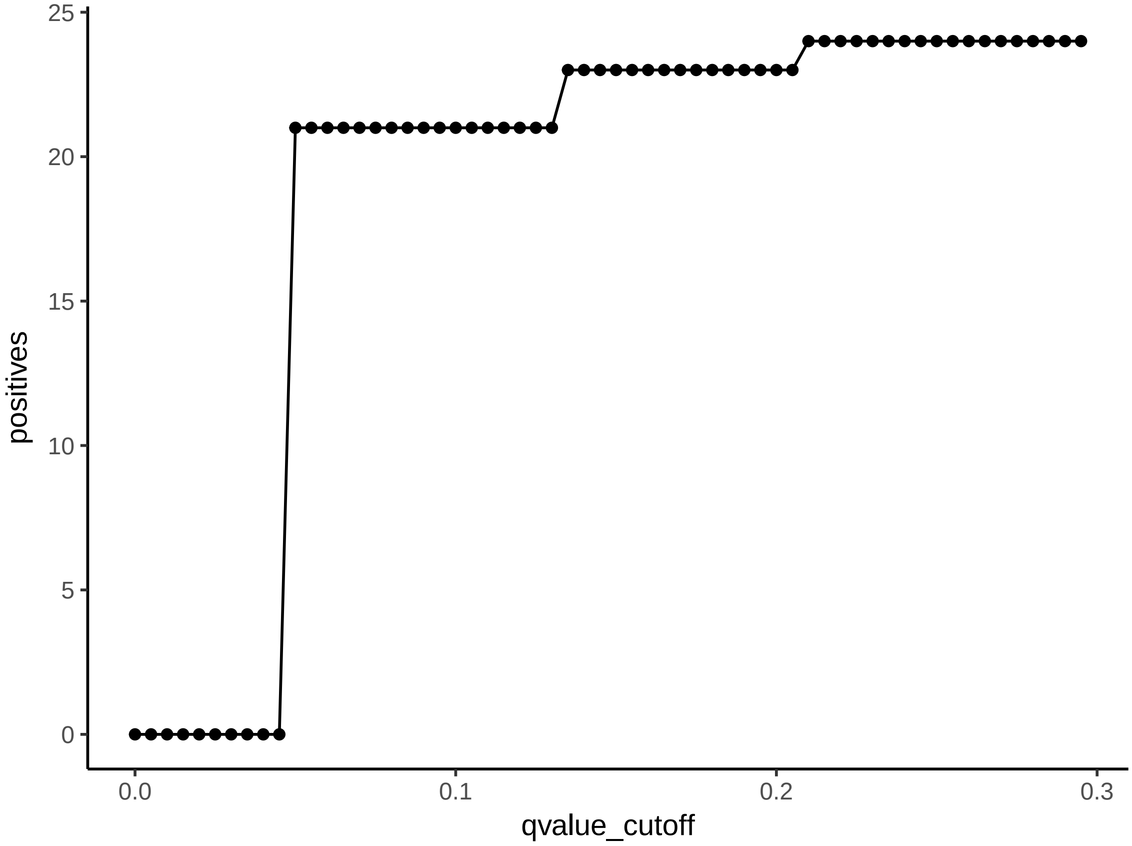

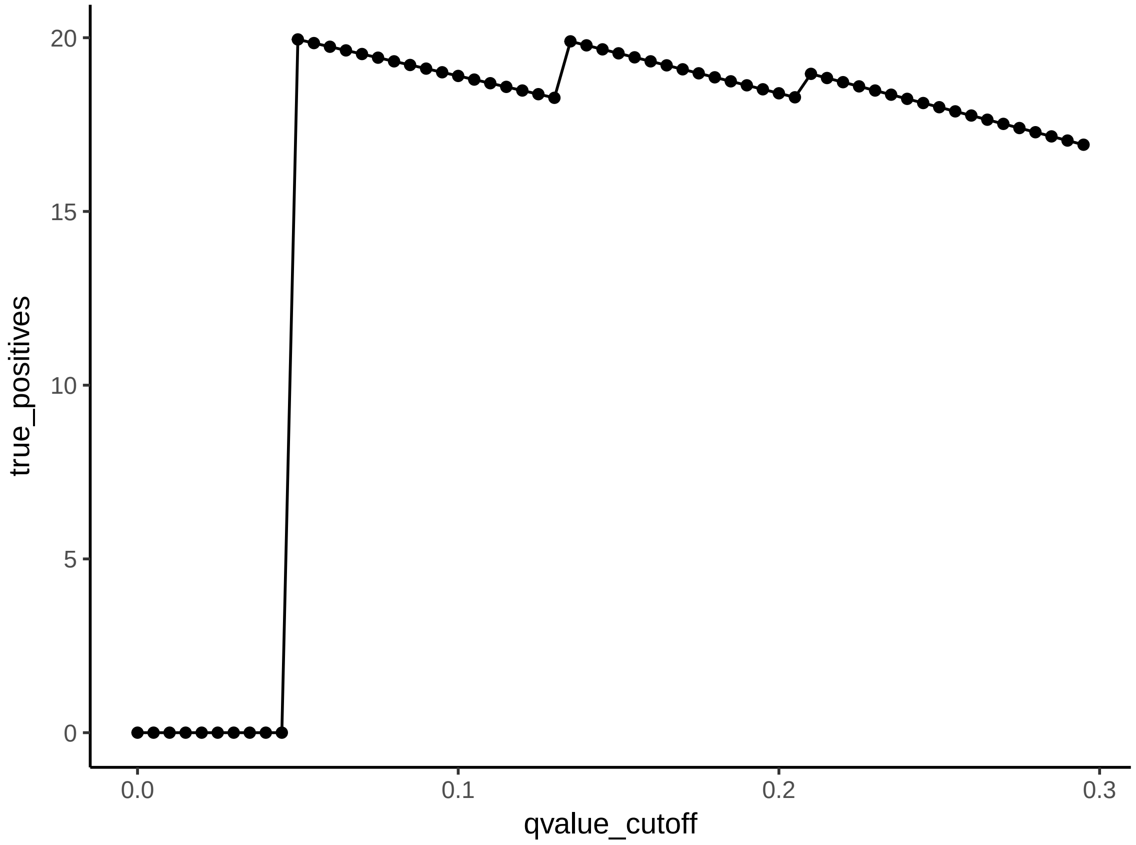


```
complexFeaturesFiltered <- subset(qvalueComplexFeaturesScored, qvalue <= 0.05)
summarizeFeatures(complexFeaturesFiltered)
```

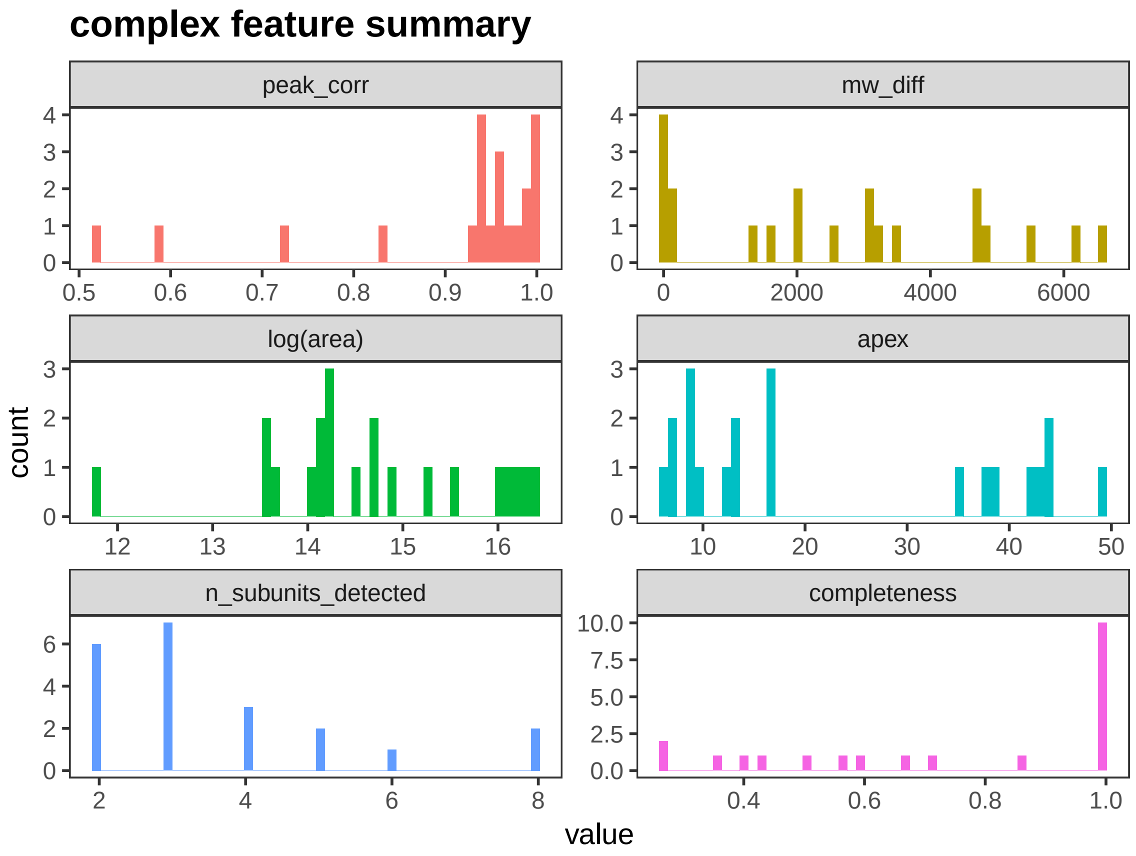
complex feature summary
peak_corr
mw_diff
log(area)
apex
n_subunits_detected
completeness
count
value
0.5
0.6
0.7
0.8
0.9
1.0
0
2000
4000
6000
12
13
14
15
16
10
20
30
40
50
2
4
6
8
0.4
0.6
0.8
1.0
0
1
2
3
4
10.0
7.5
5.0
2.5
0.0

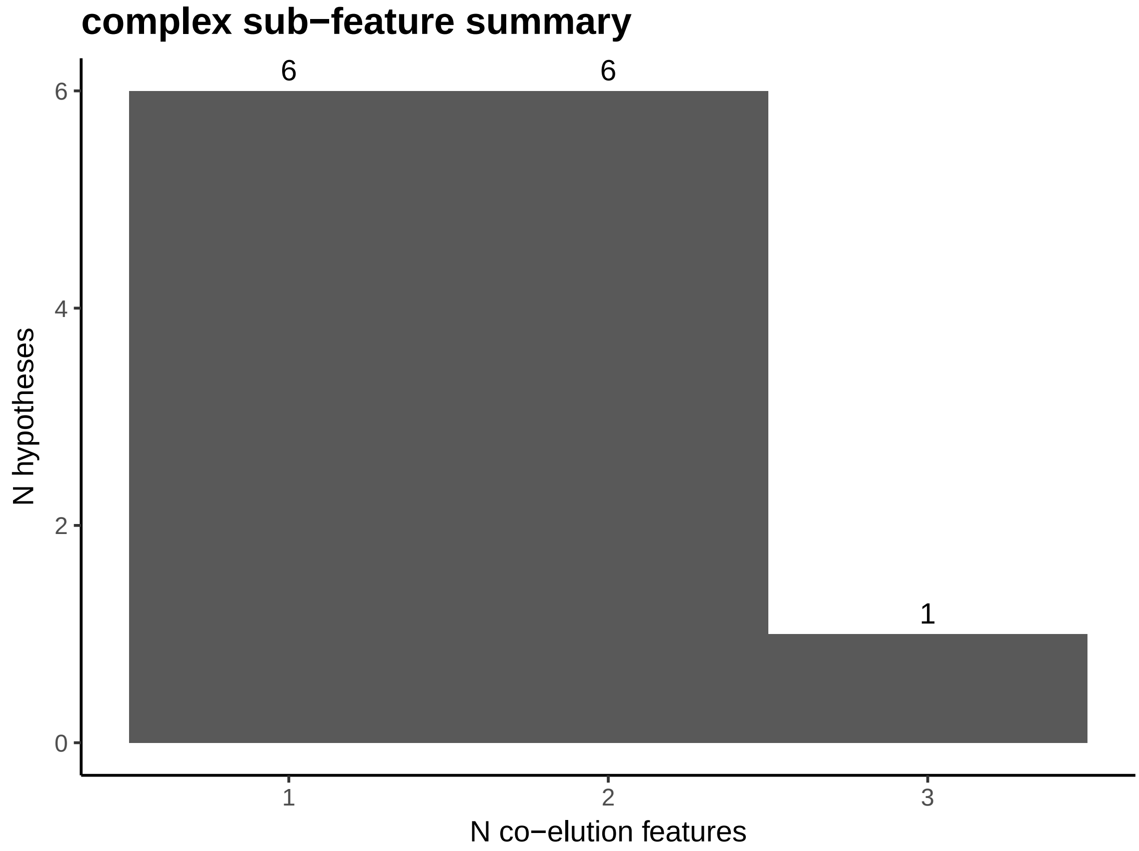
complex sub−feature summary
N hypotheses
N co−elution features
6
6
1
0
2
4
6
1
2
3

```
## $type
## [1] "complex"
##
## $totalFeatures
## [1] 21
##
## $totalConfirmedHypotheses
## [1] 13
##
## $totalHypothesesWithMultipleFeatures
## [1] 7
##
## $summaryFeatureCount
##    Min. 1st Qu.  Median    Mean 3rd Qu.    Max.
##   1.000   1.000   2.000   1.615   2.000   3.000
##
## $summaryCorrelation
##    Min. 1st Qu.  Median    Mean 3rd Qu.    Max.
##  0.5158  0.9376  0.9574  0.9076  0.9855  0.9954
##
## $summaryArea
##     Min.  1st Qu.   Median     Mean  3rd Qu.     Max.
##   126000  1329000  1944000  4031000  5775000 12740000
##
## $summaryMWdiff
##    Min. 1st Qu.  Median    Mean 3rd Qu.    Max.
##   10.12  152.00 2563.00 2639.00 4739.00 6592.00
##
## $summaryNsubunitsAnnotated
##    Min. 1st Qu.  Median    Mean 3rd Qu.    Max.
##   2.000   3.000   6.000   5.714   7.000  11.000
##
## $summaryNsubunitsWithSignal
##    Min. 1st Qu.  Median    Mean 3rd Qu.    Max.
##   2.000   3.000   5.000   5.048   6.000  10.000
##
## $summaryNsubunitsDetected
##    Min. 1st Qu.  Median    Mean 3rd Qu.    Max.
##   2.000   2.000   3.000   3.667   4.000   8.000
##
## $summaryCompleteness
##    Min. 1st Qu.  Median    Mean 3rd Qu.    Max.
##  0.2727  0.5000  0.8571  0.7451  1.0000  1.0000
```

Complex feature collapsing

Notably, *CCprofiler* is able to retrieve co-elution evidence from full protein complex queries but also subsets thereof. Due to partial overlap of the protein sets grouped as complex queries, the same co-elution signal can be retrieved as evidence for multiple different queries. Thus, we collapse the initial results to truly unique signals with regard to (i), subunit composition and (ii), resolution in the chromatographic dimension.

```
complexFeaturesUnique <- getUniqueFeatureGroups(complexFeaturesFiltered,
                                                rt_height = 0,
                                                distance_cutoff = 1.25)
complexFeaturesCollapsed <- callapseByUniqueFeatureGroups(complexFeaturesUnique,
                                                          rm_decoys = TRUE)
```

Visualization

CCprofiler offers multiple functions to visualize the data and results.

Peptide or protein trace visualization

Traces objects in CCproiler can be plotted at different stages during data processing to get a feeling for the data structure and chromatographic beaviour of different peptides or proteins.

```
plot(protTraces,
     legend = FALSE)
```

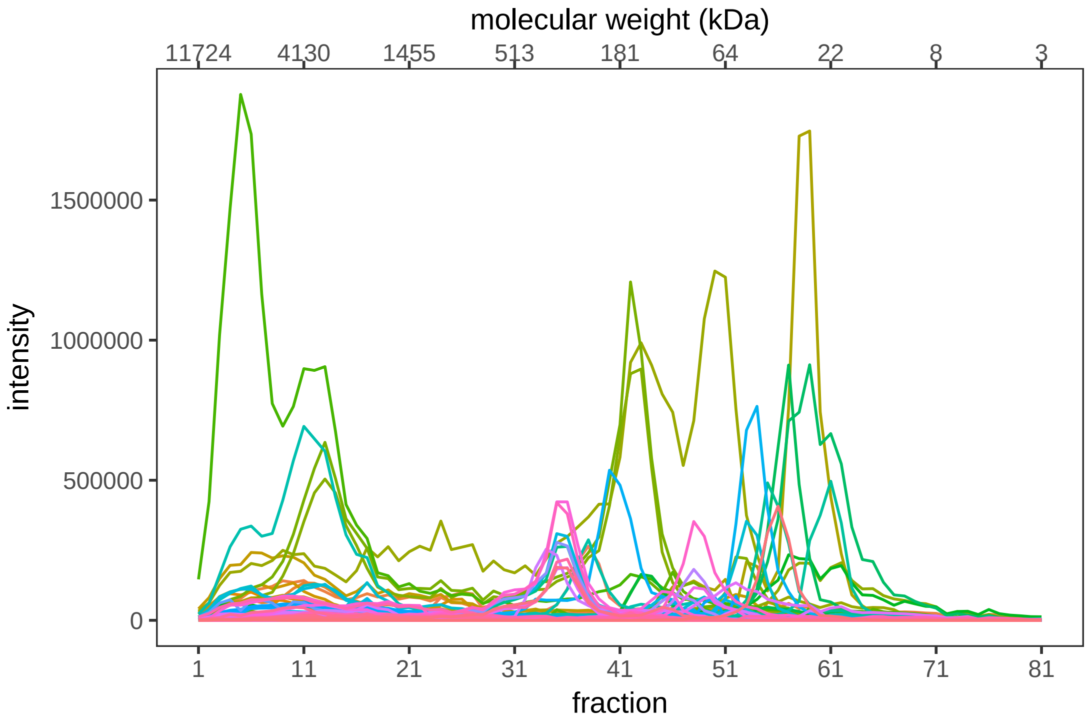

Protein and protein complex elution feature visualization

Detected protein features (peptide co-elution peak groups) and/or protein complex features (protein subunit co-elution peak groups) can be visualized with `plotFeatures`.

```
plotFeatures(feature_table = proteinFeaturesFiltered,
             traces = pepTraces_cons_sib,
             feature_id = "P61201",
             calibration = calibration,
             peak_area = TRUE,
             legend = FALSE)
```

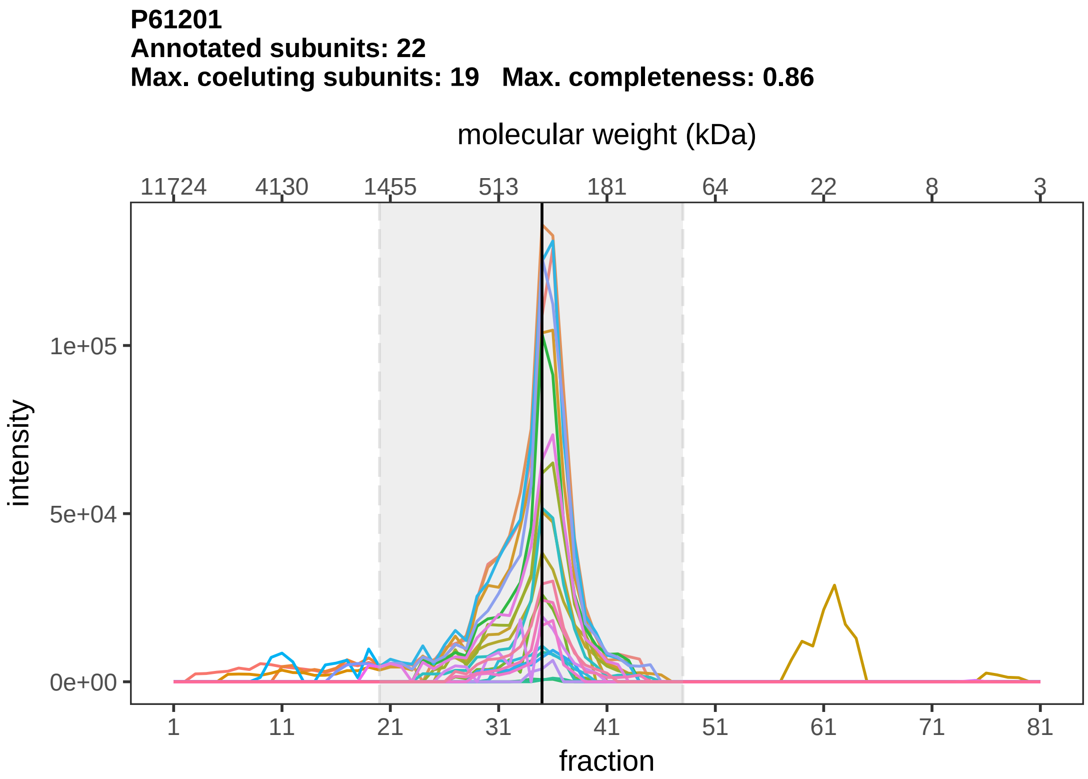


```r
plotFeatures(feature_table = complexFeaturesFiltered,
             traces = protTraces,
             feature_id = "1187",
             annotation_label="Entry_name",
             calibration = calibration,
             peak_area = TRUE)
```

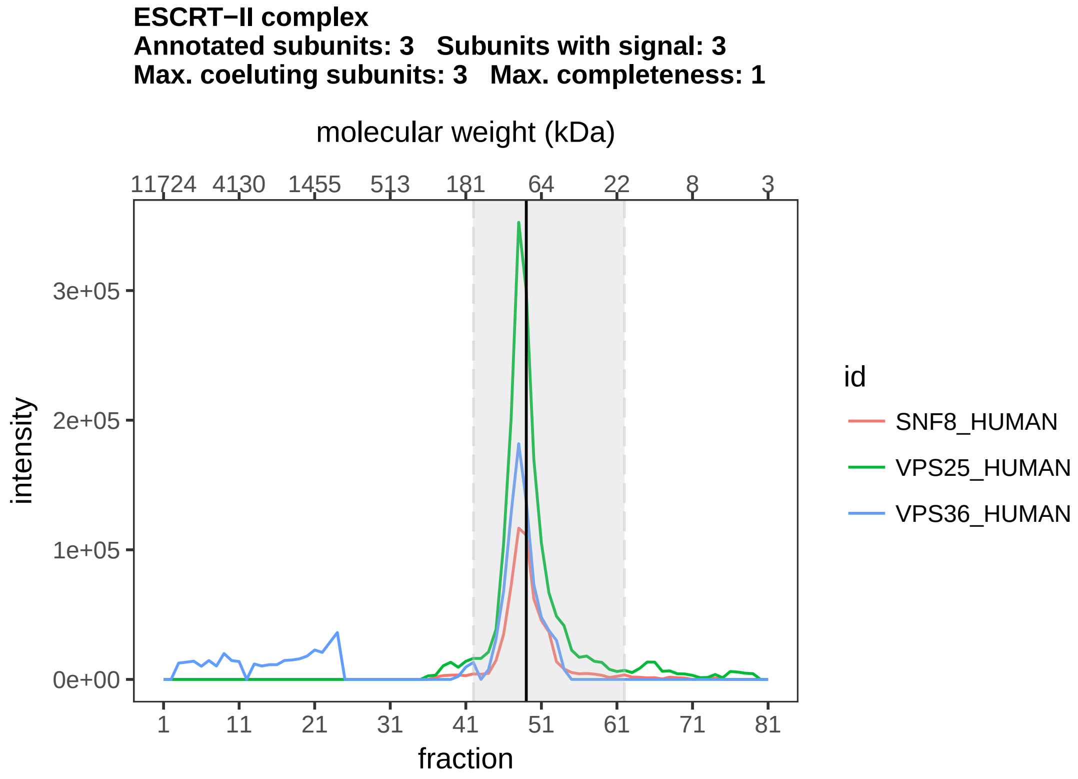

Visualization of the MS coverage of all tested complex hypotheses

The MS coverage of all tested complex hypotheses can be inspected to gain an impression of how well your hypothesis set is represented my the MS measurements without taing co-elution information into account.

```
plotSummarizedMScoverage(hypotheses = complexHypotheses,
                         protTraces = protTraces)
```

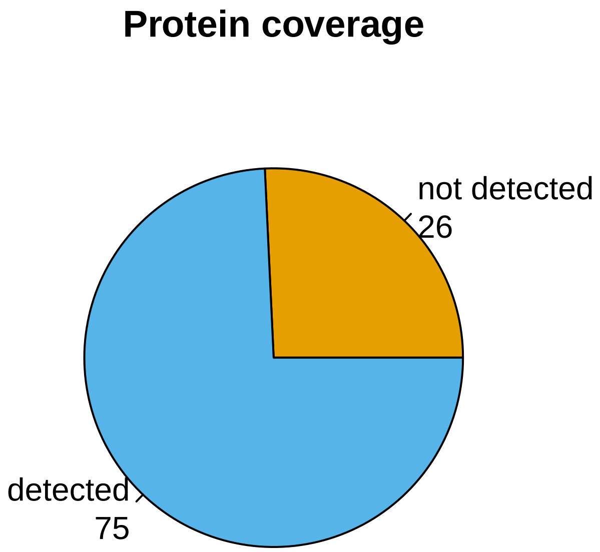
Protein coverage
not detected
26
detected
75

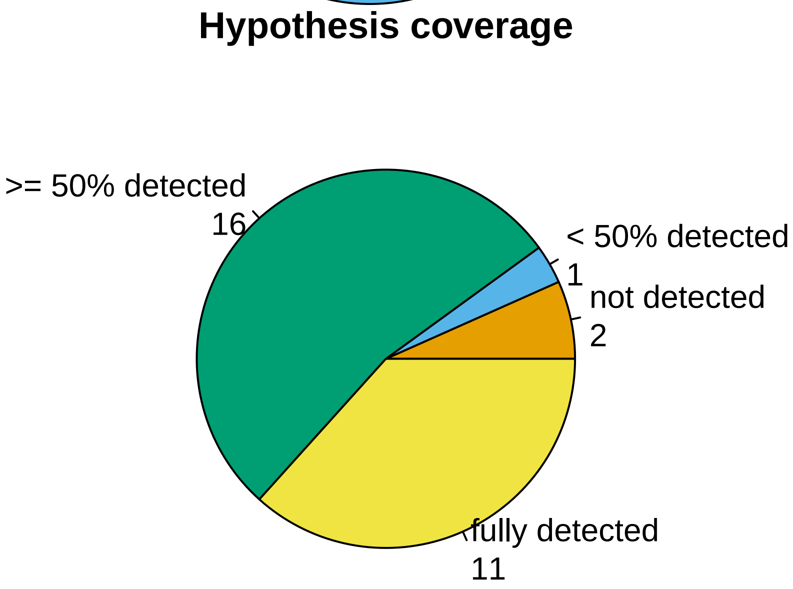
Hypothesis coverage
>= 50% detected
16
< 50% detected
1
not detected
2
fully detected
11

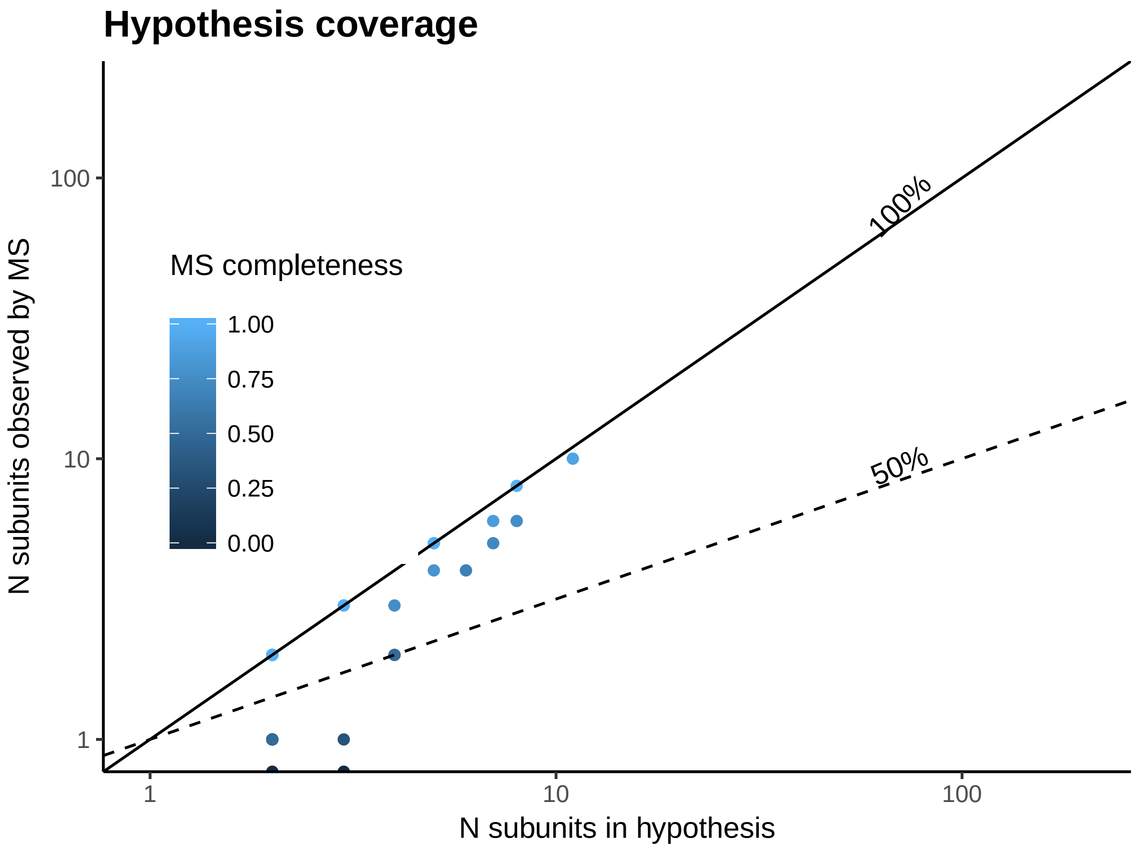

Visualization of fully and partially observed complex hypotheses

```
plotSummarizedComplexes(complexFeatures = complexFeaturesFiltered,
                        hypotheses = complexHypotheses,
                        protTraces = protTraces)
```

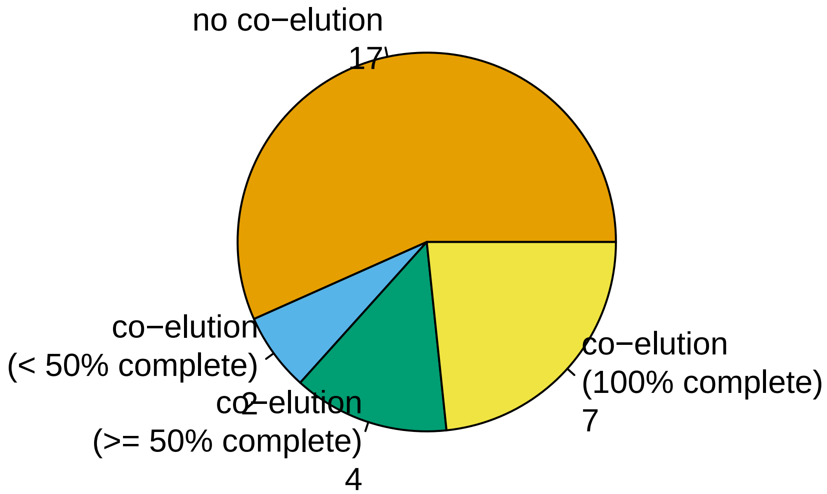

```
plotComplexCompletenessScatter(complexFeatures = complexFeaturesFiltered)
```

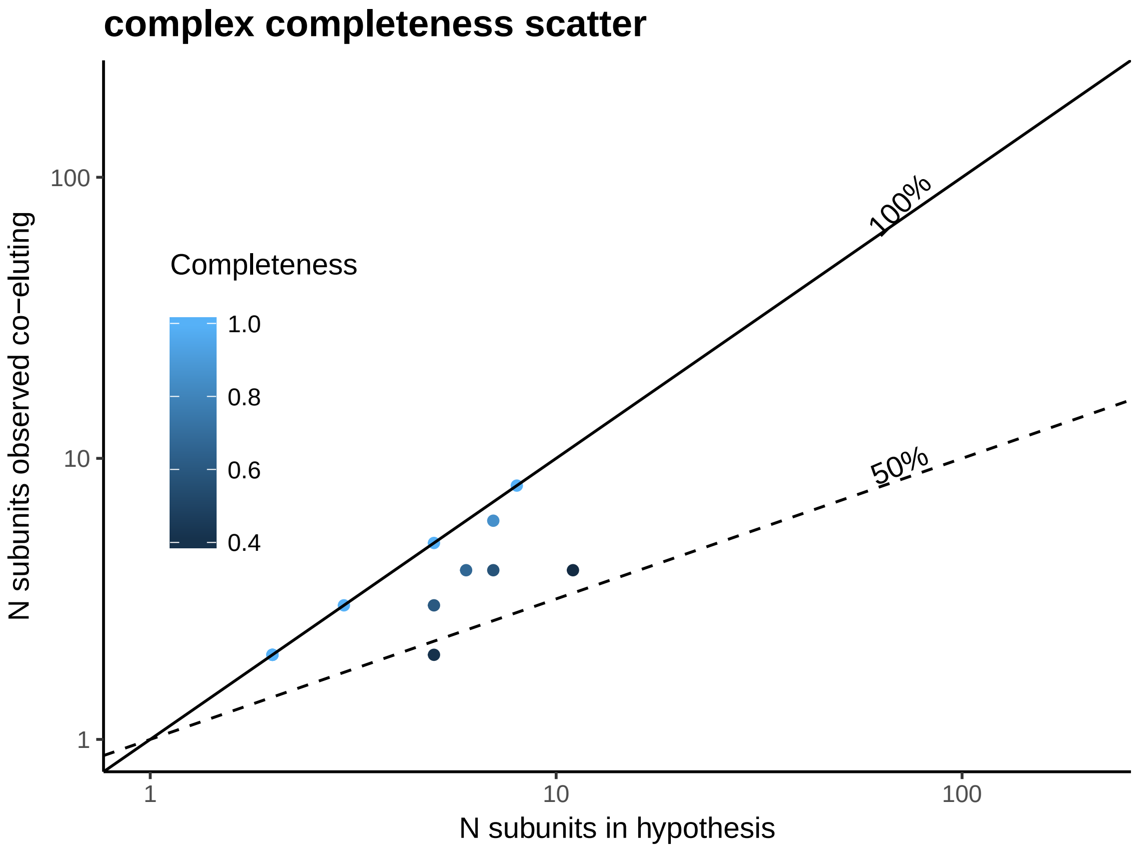

Parameter optimization

A grid search can be performed to determine an optimal set of parameters for the protein- and/or complex-centric proteome profiling workflow. This optimal parameter set depends mostly on the co-fractionation characteristics and MS setup.

Protein-level grid search

```
proteinFeatures_grid <- performProteinGridSearch(traces = peptideTracesSubset,
                                                 corrs = c(0.5,0.9),
                                                 windows = c(10),
                                                 smoothing = c(7),
                                                 rt_heights = c(4),
                                                 n_cores = 2)

proteinFeatures_scoredData <- lapply(proteinFeatures_grid, calculateCoelutionScore)
proteinFeatures_qvalueData <- lapply(proteinFeatures_scoredData, calculateQvalue, plot=F)
proteinFeatures_stats <- qvaluePositivesPlotGrid(proteinFeatures_qvalueData)
proteinFeatures_bestStats <- getBestQvalueParameters(proteinFeatures_stats, FDR_cutoff = 0.05)
proteinFeatures_bestStats
```

Complex-level grid search

```
complexFeatures_grid <- performComplexGridSearch(traces = protTraces,
                                                 complex_hypothesis = complexHypotheses,
                                                 corrs = c(0.9,0.95),
                                                 windows = c(10),
                                                 smoothing = c(7),
                                                 rt_heights = c(4),
                                                 n_cores = 2
                                                 )

complexFeatures_scoredData <- lapply(complexFeatures_grid, calculateCoelutionScore)
complexFeatures_qvalueData <- lapply(complexFeatures_scoredData, calculateQvalue, plot=F)
complexFeatures_stats <- qvaluePositivesPlotGrid(complexFeatures_qvalueData)
complexFeatures_bestStats <- getBestQvalueParameters(complexFeatures_stats, FDR_cutoff = 0.05)
complexFeatures_bestStats
```

Session information

```
## Session info -------------------------------------------------------------

##  setting  value
##  version  R version 3.2.1 (2015-06-18)
##  system   x86_64, darwin13.4.0
##  ui       X11
##  language (EN)
##  collate  en_US.UTF-8
##  tz       Europe/Zurich
##  date     2018-05-09

## Packages -----------------------------------------------------------------

##  package    * version  date       source
##  backports    1.1.1    2017-09-25 CRAN (R 3.2.5)
##  base       * 3.2.1    2015-06-18 local
##  CCprofiler * 0.1      2018-05-09 Github (CCprofiler/CCprofiler@725f71e)
##  codetools    0.2-15   2016-10-05 CRAN (R 3.2.5)
##  colorspace   1.3-2    2016-12-14 CRAN (R 3.2.5)
##  compiler     3.2.1    2015-06-18 local
##  curl         3.0      2017-10-06 CRAN (R 3.2.5)
##  data.table * 1.10.4-3 2017-10-27 CRAN (R 3.2.5)
##  datasets   * 3.2.1    2015-06-18 local
##  devtools   * 1.13.4   2017-11-09 CRAN (R 3.2.5)
##  digest       0.6.12   2017-01-27 CRAN (R 3.2.5)
##  doSNOW     * 1.0.15   2017-09-29 CRAN (R 3.2.5)
##  evaluate     0.10.1   2017-06-24 CRAN (R 3.2.5)
##  foreach    * 1.4.3    2015-10-13 CRAN (R 3.2.0)
##  ggplot2    * 2.2.1    2016-12-30 CRAN (R 3.2.5)
##  git2r        0.19.0   2017-07-19 CRAN (R 3.2.5)
##  gmp        * 0.5-13.1 2017-03-10 CRAN (R 3.2.5)
##  graphics   * 3.2.1    2015-06-18 local
##  grDevices  * 3.2.1    2015-06-18 local
##  grid         3.2.1    2015-06-18 local
##  gridExtra  * 2.3      2017-09-09 CRAN (R 3.2.5)
##  gtable       0.2.0    2016-02-26 CRAN (R 3.2.3)
##  htmltools    0.3.6    2017-04-28 CRAN (R 3.2.5)
##  httr         1.3.1    2017-08-20 CRAN (R 3.2.5)
```

```
## igraph     * 1.1.2    2017-07-21 CRAN (R 3.2.5)
## iterators  * 1.0.8    2015-10-13 CRAN (R 3.2.0)
## knitr        1.17     2017-08-10 CRAN (R 3.2.5)
## labeling     0.3      2014-08-23 CRAN (R 3.2.0)
## lazyeval     0.2.1    2017-10-29 CRAN (R 3.2.5)
## magrittr     1.5      2014-11-22 CRAN (R 3.2.0)
## memoise      1.1.0    2017-04-21 CRAN (R 3.2.5)
## methods    * 3.2.1    2015-06-18 local
## munsell      0.4.3    2016-02-13 CRAN (R 3.2.3)
## parallel   * 3.2.1    2015-06-18 local
## pkgconfig    2.0.1    2017-03-21 CRAN (R 3.2.5)
## plyr         1.8.4    2016-06-08 CRAN (R 3.2.5)
## pracma     * 2.0.7    2017-06-21 CRAN (R 3.2.5)
## proxy      * 0.4-16   2016-06-25 CRAN (R 3.2.5)
## quadprog     1.5-5    2013-04-17 CRAN (R 3.2.0)
## qvalue       2.2.2    2016-01-08 Bioconductor
## R6           2.2.2    2017-06-17 CRAN (R 3.2.1)
## Rcpp         0.12.13  2017-09-28 CRAN (R 3.2.5)
## reshape    * 0.8.7    2017-08-06 CRAN (R 3.2.5)
## reshape2     1.4.2    2016-10-22 CRAN (R 3.2.5)
## rlang        0.1.4    2017-11-05 CRAN (R 3.2.5)
## rmarkdown    1.8      2017-11-17 CRAN (R 3.2.5)
## Rmpfr      * 0.6-1    2016-11-15 CRAN (R 3.2.5)
## rprojroot    1.2      2017-01-16 CRAN (R 3.2.5)
## scales       0.5.0    2017-08-24 CRAN (R 3.2.5)
## snow       * 0.4-2    2016-10-14 CRAN (R 3.2.5)
## splines      3.2.1    2015-06-18 local
## stats      * 3.2.1    2015-06-18 local
## stringi      1.1.6    2017-11-17 CRAN (R 3.2.5)
## stringr      1.2.0    2017-02-18 CRAN (R 3.2.1)
## tibble       1.3.4    2017-08-22 CRAN (R 3.2.5)
## tools        3.2.1    2015-06-18 local
## utils      * 3.2.1    2015-06-18 local
## withr        2.1.0    2017-11-01 CRAN (R 3.2.5)
## yaml         2.1.14   2016-11-12 CRAN (R 3.2.5)
```
