# Supplementary material for: Complex‐centric proteome profiling by SEC‐SWATH‐MS
Source: Mol Syst Biol. 2019 Jan 14;15(1):e8438. doi: 10.15252/msb.20188438 (PMC6346213; doi:10.15252/msb.20188438)
Supplement: Supplementary file 6 — Dataset EV5 [file MSB-15-e8438-s006.zip › feature_plots_corum/1005.pdf]

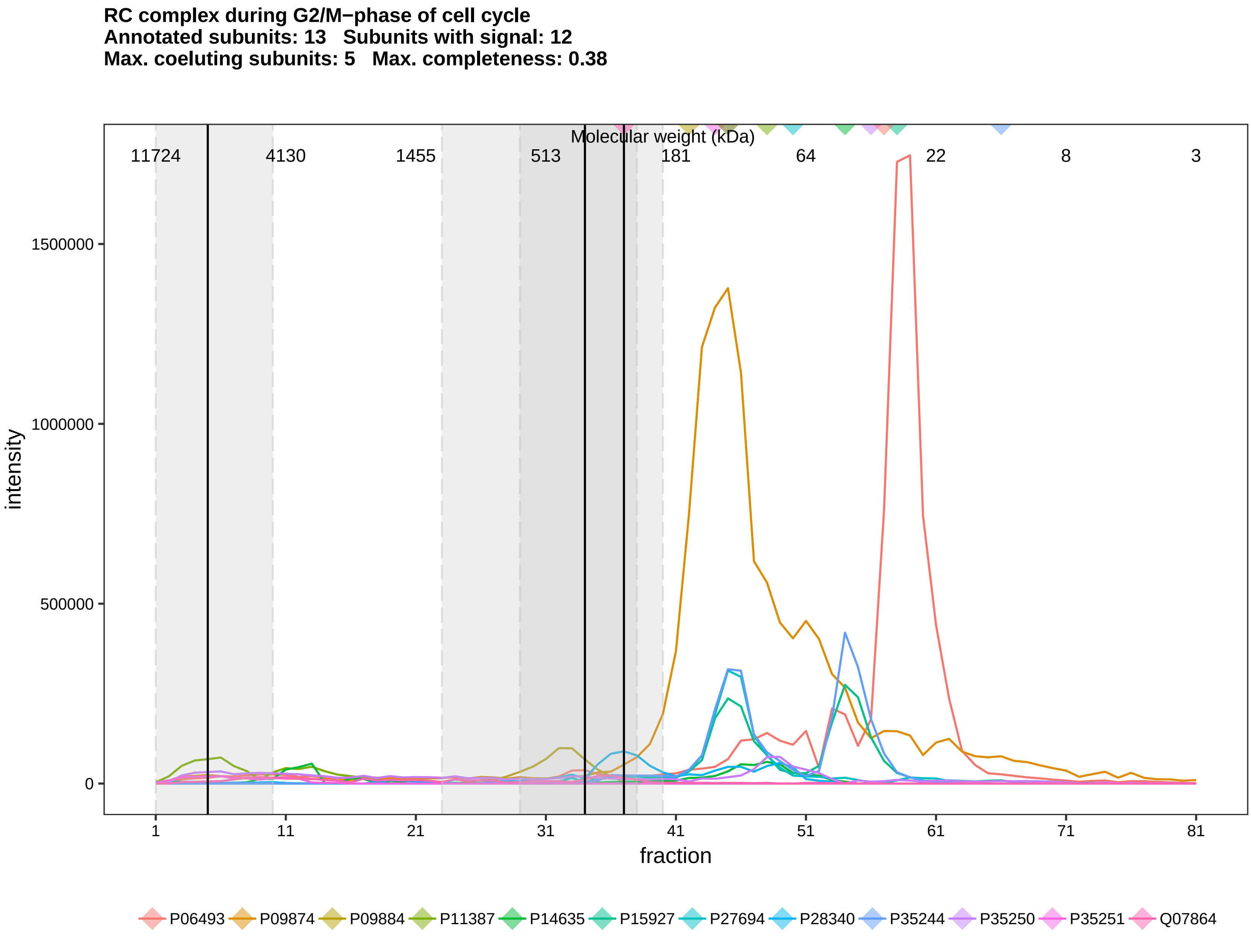

RC complex during G2/M−phase of cell cycle
Annotated subunits: 13 Subunits with signal: 12
Max. coeluting subunits: 5 Max. completeness: 0.38
Molecular weight (kDa)
11724
4130
1455
513
181
64
22
8
3
intensity
1500000
1000000
500000
0
fraction
1
11
21
31
41
51
61
71
81
P06493
P09874
P09884
P11387
P14635
P15927
P27694
P28340
P35244
P35250
P35251
Q07864
