# Supplementary material for: Complex‐centric proteome profiling by SEC‐SWATH‐MS
Source: Mol Syst Biol. 2019 Jan 14;15(1):e8438. doi: 10.15252/msb.20188438 (PMC6346213; doi:10.15252/msb.20188438)
Supplement: Supplementary file 6 — Dataset EV5 [file MSB-15-e8438-s006.zip › feature_plots_corum/103.pdf]

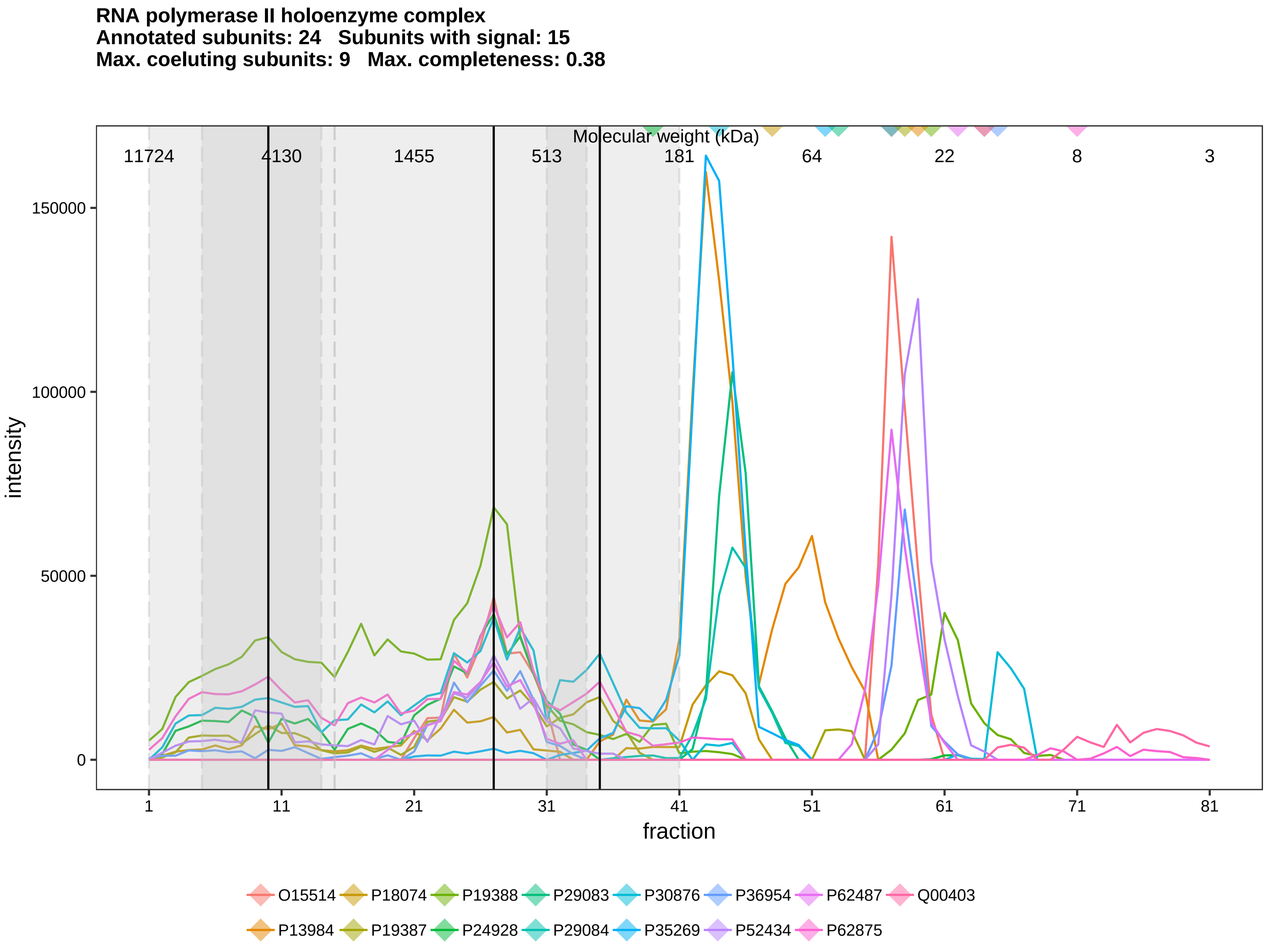
RNA polymerase II holoenzyme complex
Annotated subunits: 24 Subunits with signal: 15
Max. coeluting subunits: 9 Max. completeness: 0.38
Molecular weight (kDa)
11724
4130
1455
513
181
64
22
8
3
intensity
150000
100000
50000
0
fraction
1
11
21
31
41
51
61
71
81
O15514
P18074
P19388
P29083
P30876
P36954
P62487
Q00403
P13984
P19387
P24928
P29084
P35269
P52434
P62875
