# Supplementary material for: Complex‐centric proteome profiling by SEC‐SWATH‐MS
Source: Mol Syst Biol. 2019 Jan 14;15(1):e8438. doi: 10.15252/msb.20188438 (PMC6346213; doi:10.15252/msb.20188438)

eIF3 complex (EIF3S6, EIF3S5, EIF3S4, EIF3S3, EIF3S6IP, EIF3S2, EIF3S9, EIF3S12, EIF3S10, EIF3S8, EIF3S1, EIF3S7, PCID
Annotated subunits: 13 Subunits with signal: 13
Max. coeluting subunits: 12 Max. completeness: 0.92

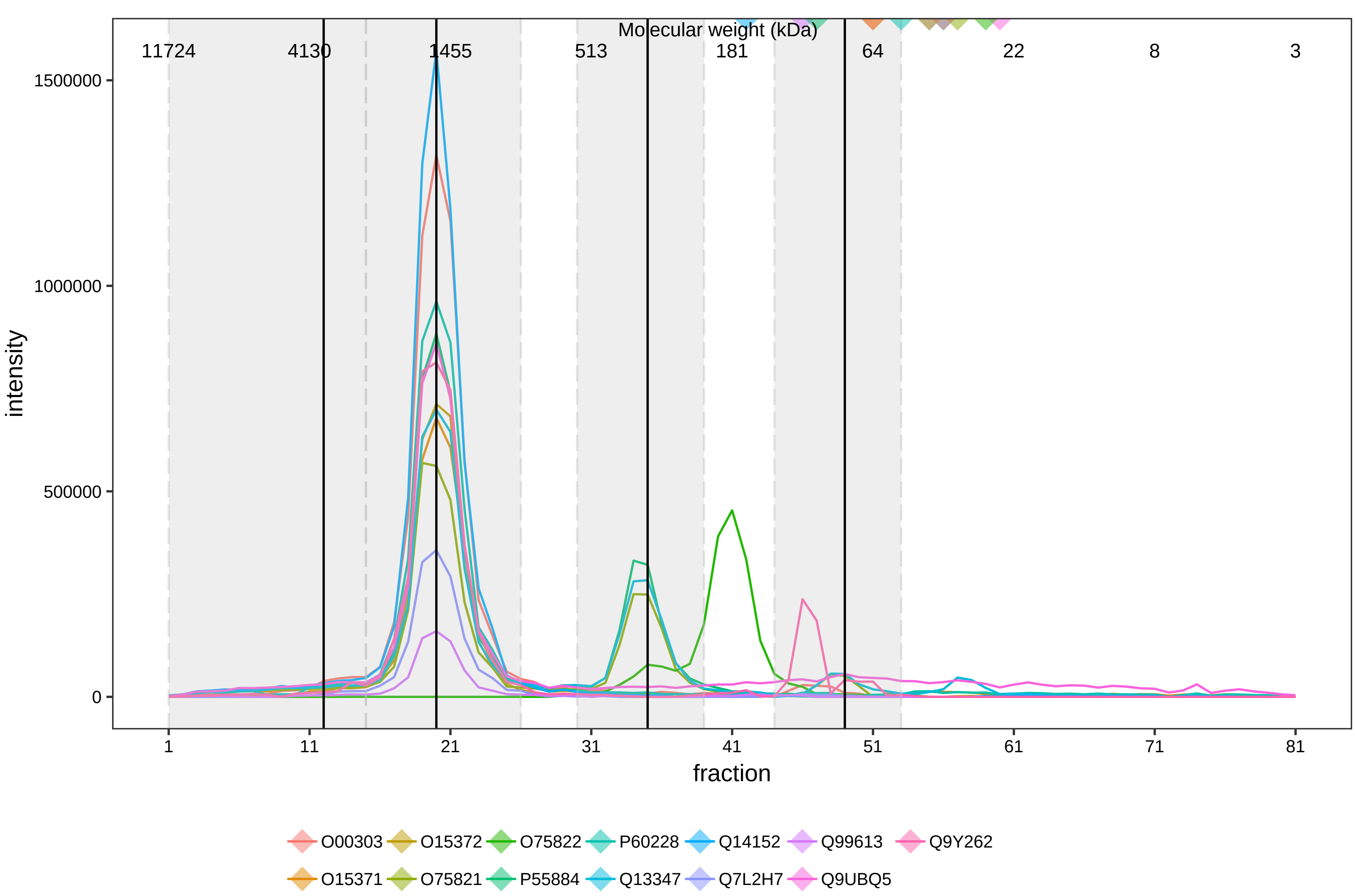

Supplement: Supplementary file 6 — Dataset EV5 [file MSB-15-e8438-s006.zip › feature_plots_corum/1097.pdf]
