# Supplementary material for: Complex‐centric proteome profiling by SEC‐SWATH‐MS
Source: Mol Syst Biol. 2019 Jan 14;15(1):e8438. doi: 10.15252/msb.20188438 (PMC6346213; doi:10.15252/msb.20188438)
Supplement: Supplementary file 6 — Dataset EV5 [file MSB-15-e8438-s006.zip › feature_plots_corum/1143.pdf]

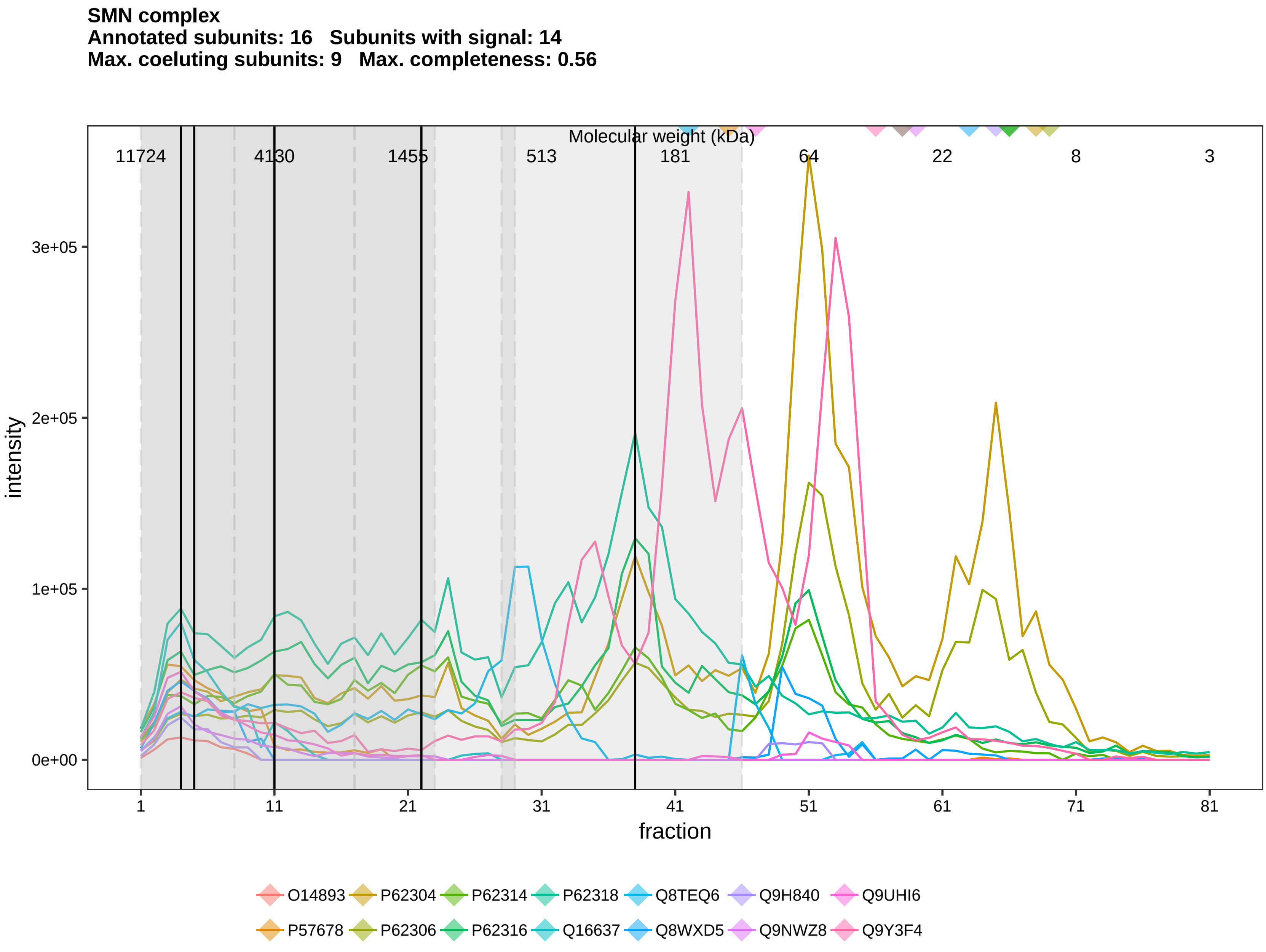

SMN complex
Annotated subunits: 16 Subunits with signal: 14
Max. coeluting subunits: 9 Max. completeness: 0.56
Molecular weight (kDa)
11724
4130
1455
513
181
64
22
8
3
intensity
3e+05
2e+05
1e+05
0e+00
fraction
1
11
21
31
41
51
61
71
81
O14893
P62304
P62314
P62318
Q8TEQ6
Q9H840
Q9UHI6
P57678
P62306
P62316
Q16637
Q8WXD5
Q9NWZ8
Q9Y3F4
