# Supplementary material for: Complex‐centric proteome profiling by SEC‐SWATH‐MS
Source: Mol Syst Biol. 2019 Jan 14;15(1):e8438. doi: 10.15252/msb.20188438 (PMC6346213; doi:10.15252/msb.20188438)
Supplement: Supplementary file 6 — Dataset EV5 [file MSB-15-e8438-s006.zip › feature_plots_corum/1237.pdf]

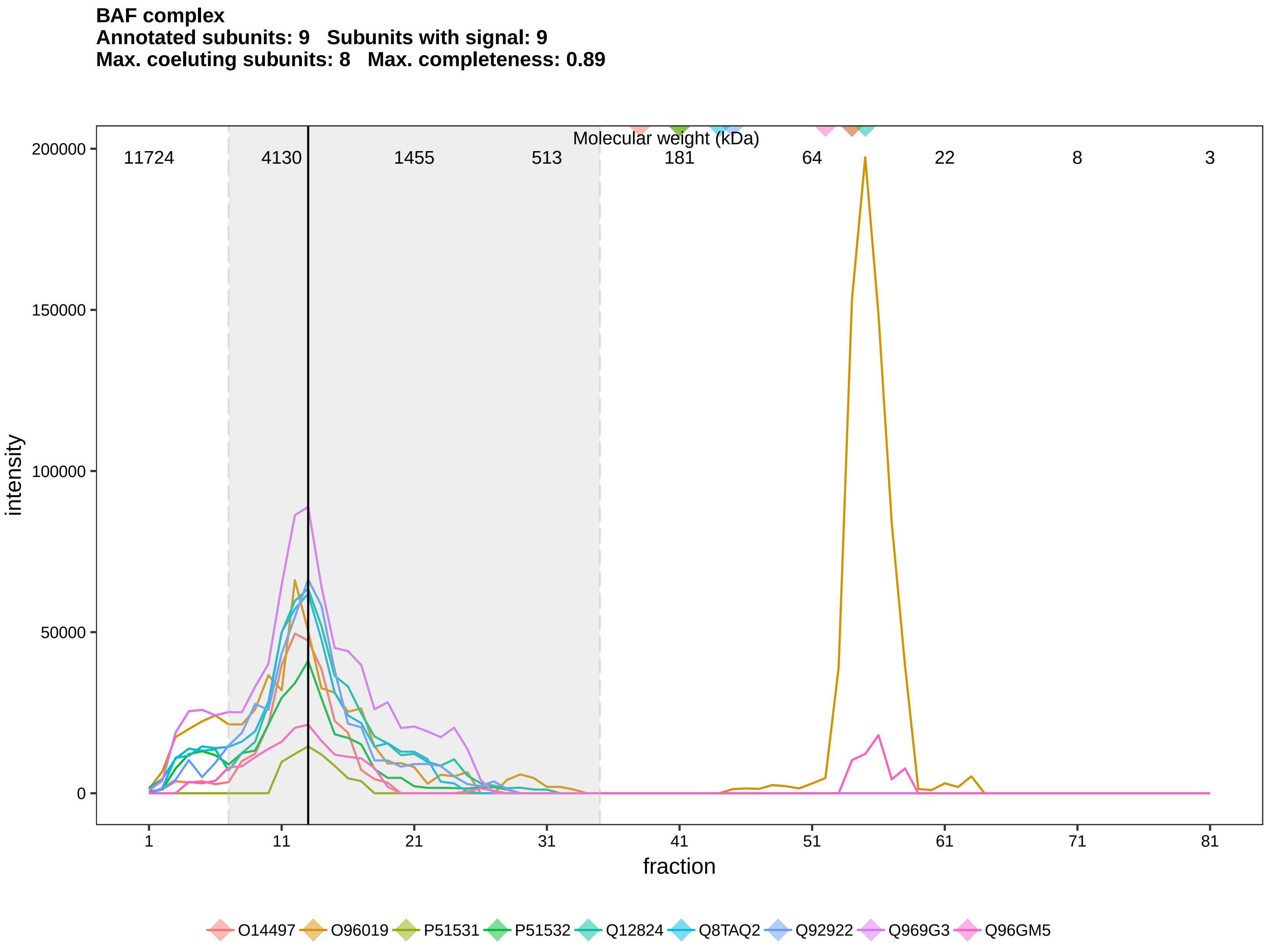
BAF complex
Annotated subunits: 9 Subunits with signal: 9
Max. coeluting subunits: 8 Max. completeness: 0.89
Molecular weight (kDa)
11724
4130
1455
513
181
64
22
8
3
200000
150000
100000
50000
0
intensity
1
11
21
31
41
51
61
71
81
fraction
O14497
O96019
P51531
P51532
Q12824
Q8TAQ2
Q92922
Q969G3
Q96GM5
