# Supplementary material for: Complex‐centric proteome profiling by SEC‐SWATH‐MS
Source: Mol Syst Biol. 2019 Jan 14;15(1):e8438. doi: 10.15252/msb.20188438 (PMC6346213; doi:10.15252/msb.20188438)

PBAF complex (Polybromo− and BAF containing complex);PBAF complex (Polybromo− and BAF containing complex)−1
Annotated subunits: 9   Subunits with signal: 8
Max. coeluting subunits: 6   Max. completeness: 0.67

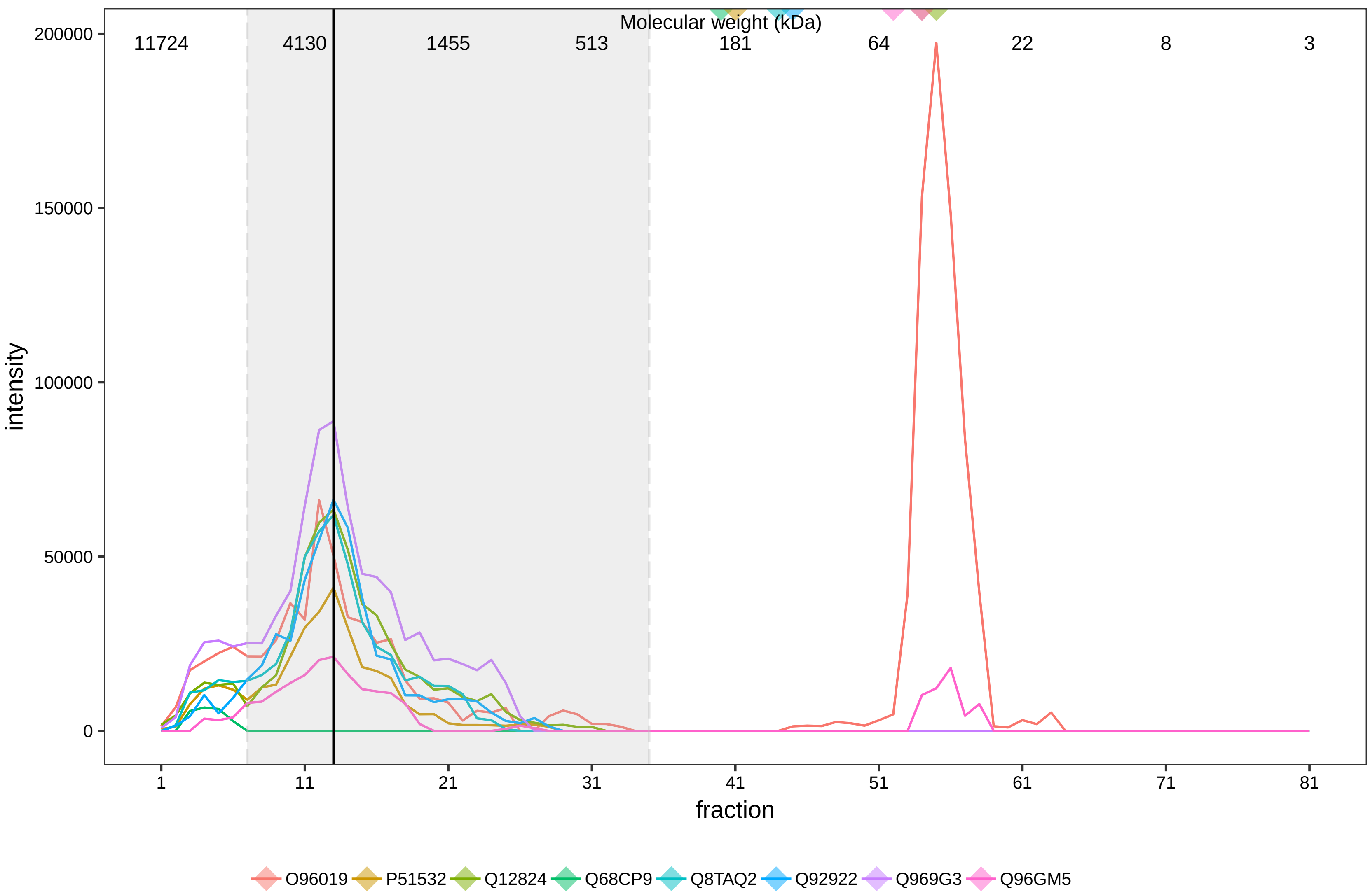

Supplement: Supplementary file 6 — Dataset EV5 [file MSB-15-e8438-s006.zip › feature_plots_corum/1238;556;565-1.pdf]
