# Supplementary material for: Complex‐centric proteome profiling by SEC‐SWATH‐MS
Source: Mol Syst Biol. 2019 Jan 14;15(1):e8438. doi: 10.15252/msb.20188438 (PMC6346213; doi:10.15252/msb.20188438)
Supplement: Supplementary file 6 — Dataset EV5 [file MSB-15-e8438-s006.zip › feature_plots_corum/1257.pdf]

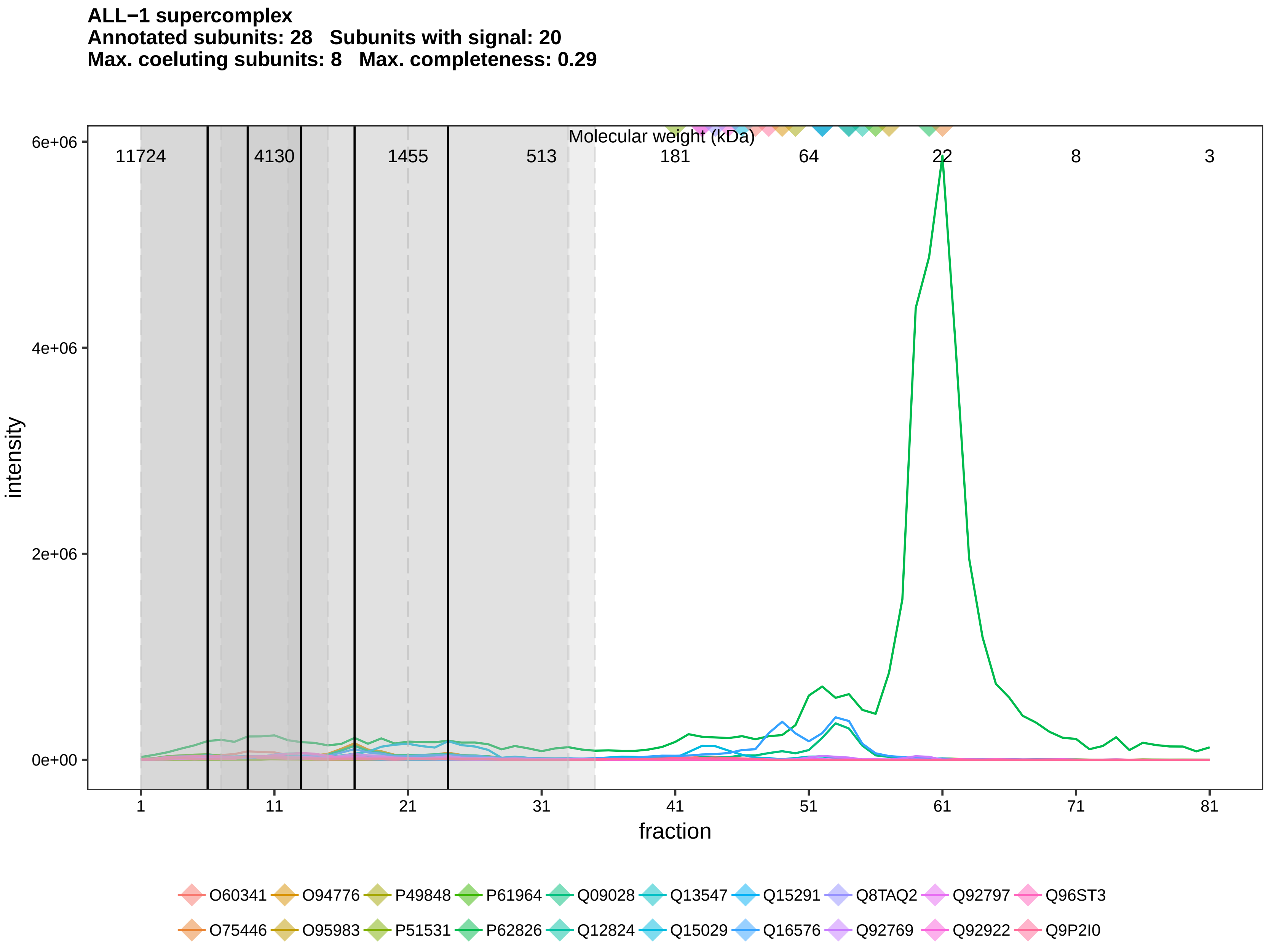
ALL−1 supercomplex
Annotated subunits: 28 Subunits with signal: 20
Max. coeluting subunits: 8 Max. completeness: 0.29
Molecular weight (kDa)
11724
4130
1455
513
181
64
22
8
3
6e+06
4e+06
2e+06
0e+00
intensity
1
11
21
31
41
51
61
71
81
fraction
O60341
O94776
P49848
P61964
Q09028
Q13547
Q15291
Q8TAQ2
Q92797
Q96ST3
O75446
O95983
P51531
P62826
Q12824
Q15029
Q16576
Q92769
Q92922
Q9P2I0
