# Supplementary material for: Complex‐centric proteome profiling by SEC‐SWATH‐MS
Source: Mol Syst Biol. 2019 Jan 14;15(1):e8438. doi: 10.15252/msb.20188438 (PMC6346213; doi:10.15252/msb.20188438)
Supplement: Supplementary file 6 — Dataset EV5 [file MSB-15-e8438-s006.zip › feature_plots_corum/1332.pdf]

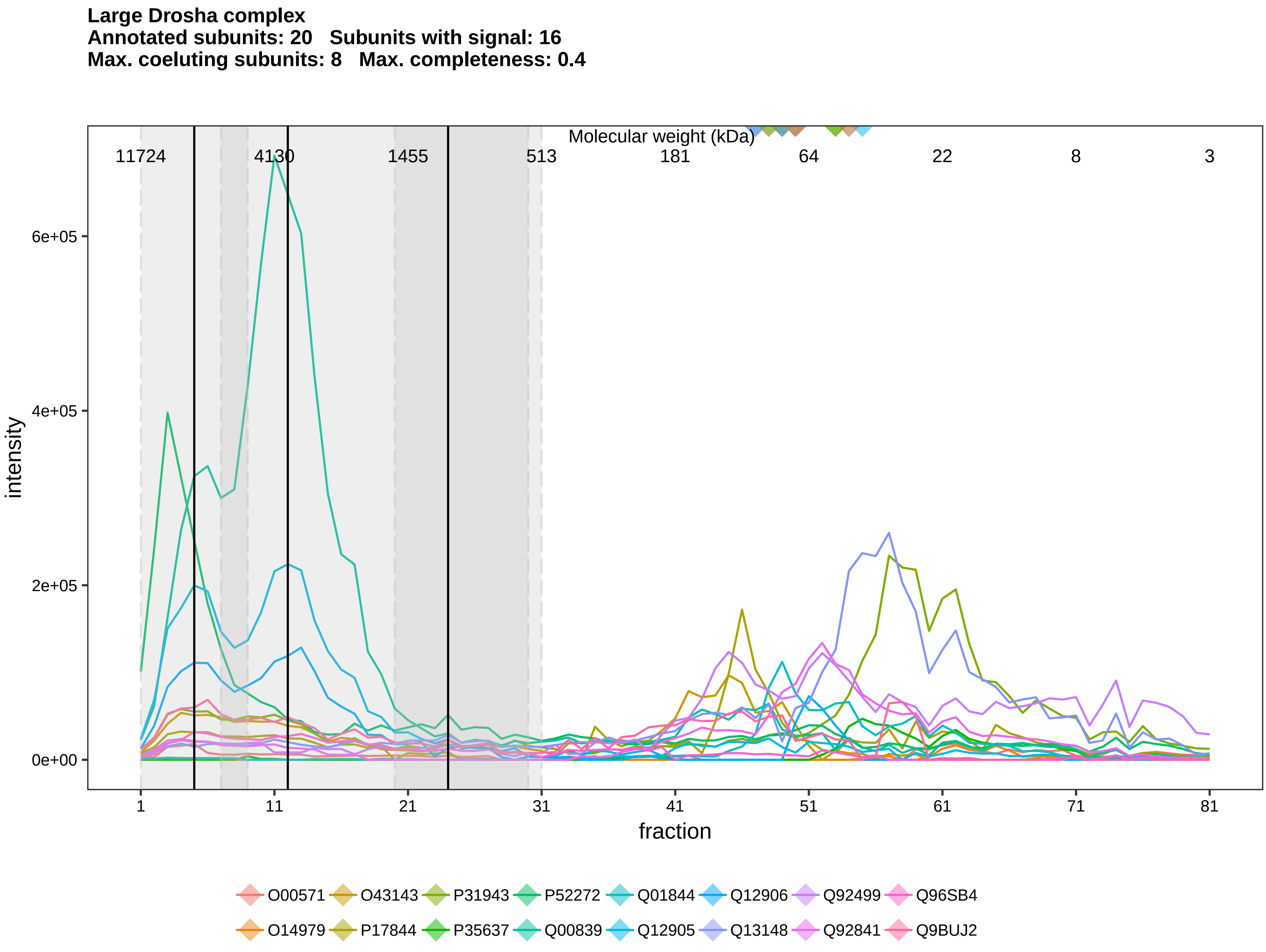

Large Drosha complex
Annotated subunits: 20   Subunits with signal: 16
Max. coeluting subunits: 8   Max. completeness: 0.4
Molecular weight (kDa)
11724
4130
1455
513
181
64
22
8
3
6e+05
4e+05
2e+05
0e+00
intensity
1
11
21
31
41
51
61
71
81
fraction
O00571
O43143
P31943
P52272
Q01844
Q12906
Q92499
Q96SB4
O14979
P17844
P35637
Q00839
Q12905
Q13148
Q92841
Q9BUJ2
