# Supplementary material for: Complex‐centric proteome profiling by SEC‐SWATH‐MS
Source: Mol Syst Biol. 2019 Jan 14;15(1):e8438. doi: 10.15252/msb.20188438 (PMC6346213; doi:10.15252/msb.20188438)
Supplement: Supplementary file 6 — Dataset EV5 [file MSB-15-e8438-s006.zip › feature_plots_corum/191.pdf]

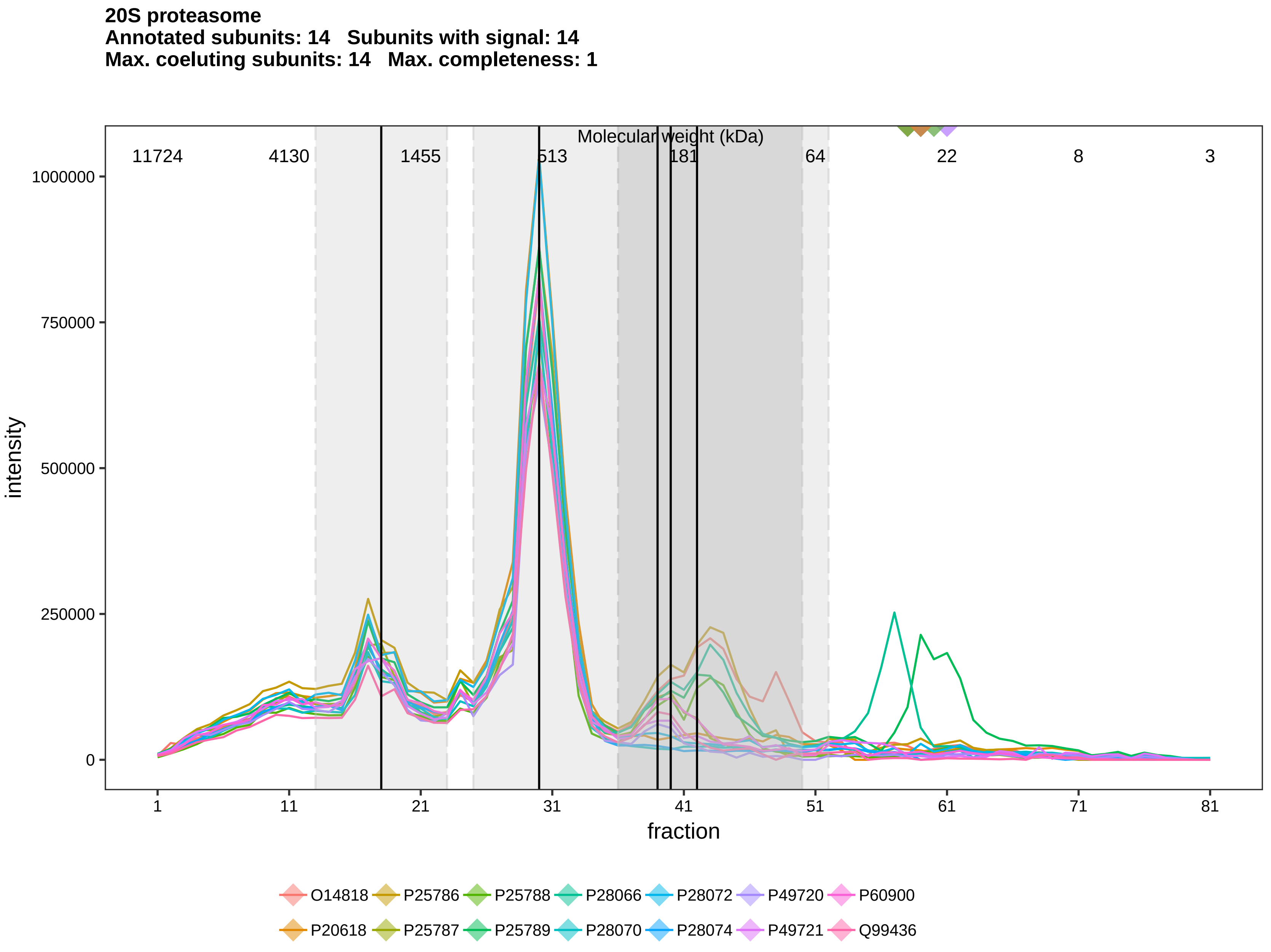

20S proteasome
Annotated subunits: 14   Subunits with signal: 14
Max. coeluting subunits: 14   Max. completeness: 1
Molecular weight (kDa)
11724
4130
1455
513
181
64
22
8
3
intensity
1000000
750000
500000
250000
0
fraction
1
11
21
31
41
51
61
71
81
O14818
P25786
P25788
P28066
P28072
P49720
P60900
P20618
P25787
P25789
P28070
P28074
P49721
Q99436
