# Supplementary figures and images for: Complex‐centric proteome profiling by SEC‐SWATH‐MS (part 1 of 3)
Source: Mol Syst Biol. 2019 Jan 14;15(1):e8438. doi: 10.15252/msb.20188438 (PMC6346213; doi:10.15252/msb.20188438)

**Annotated subunits: 10   Subunits with signal: 8**  
**Max. coeluting subunits: 5   Max. completeness: 0.5**

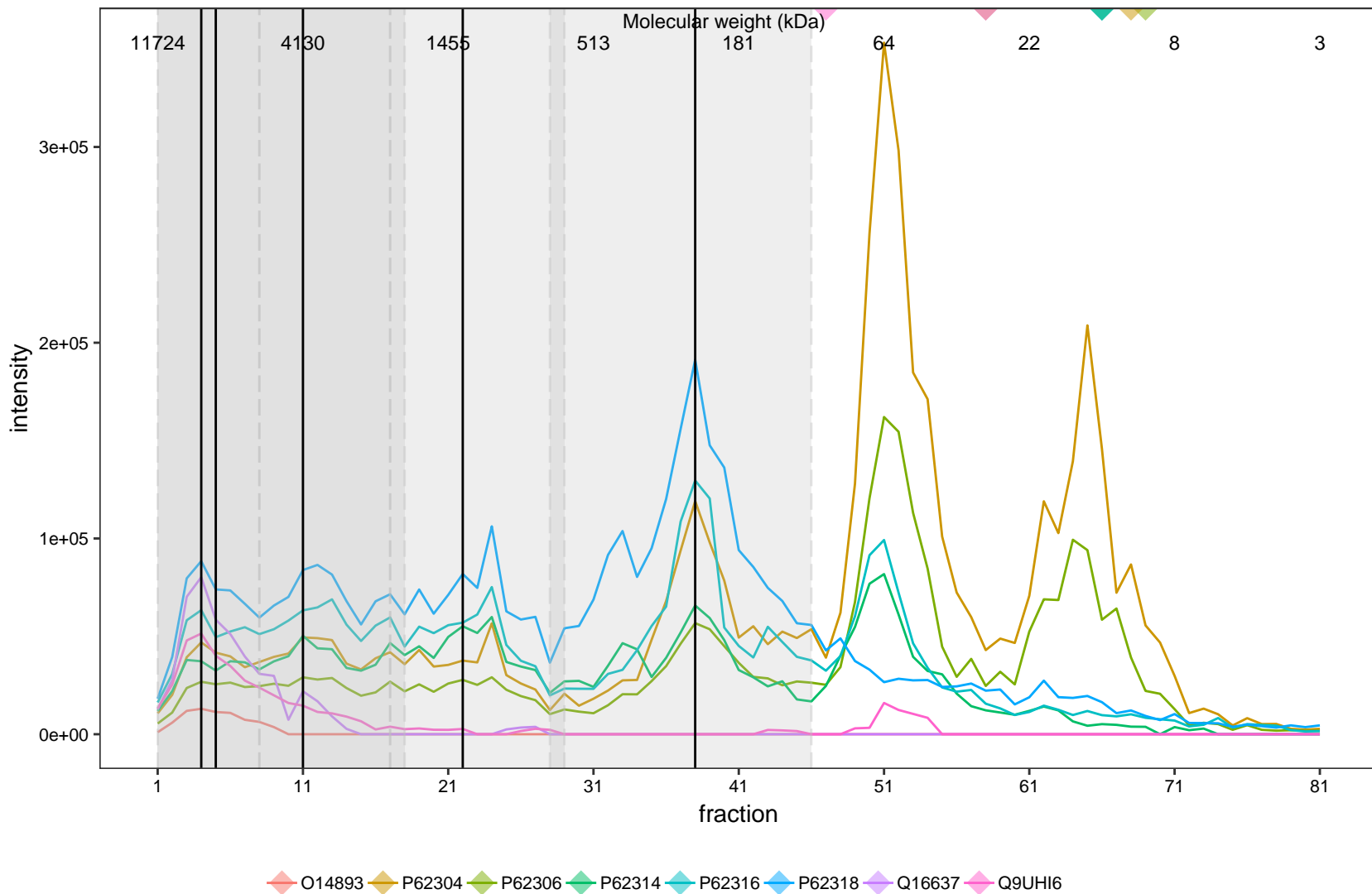

Supplement: Supplementary file 6 — Dataset EV5 [file MSB-15-e8438-s006.zip › feature_plots_corum/1142.pdf]

# E2F-6 complex

Annotated subunits: 12 Subunits with signal: 5

Max. coeluting subunits: 2 Max. completeness: 0.17

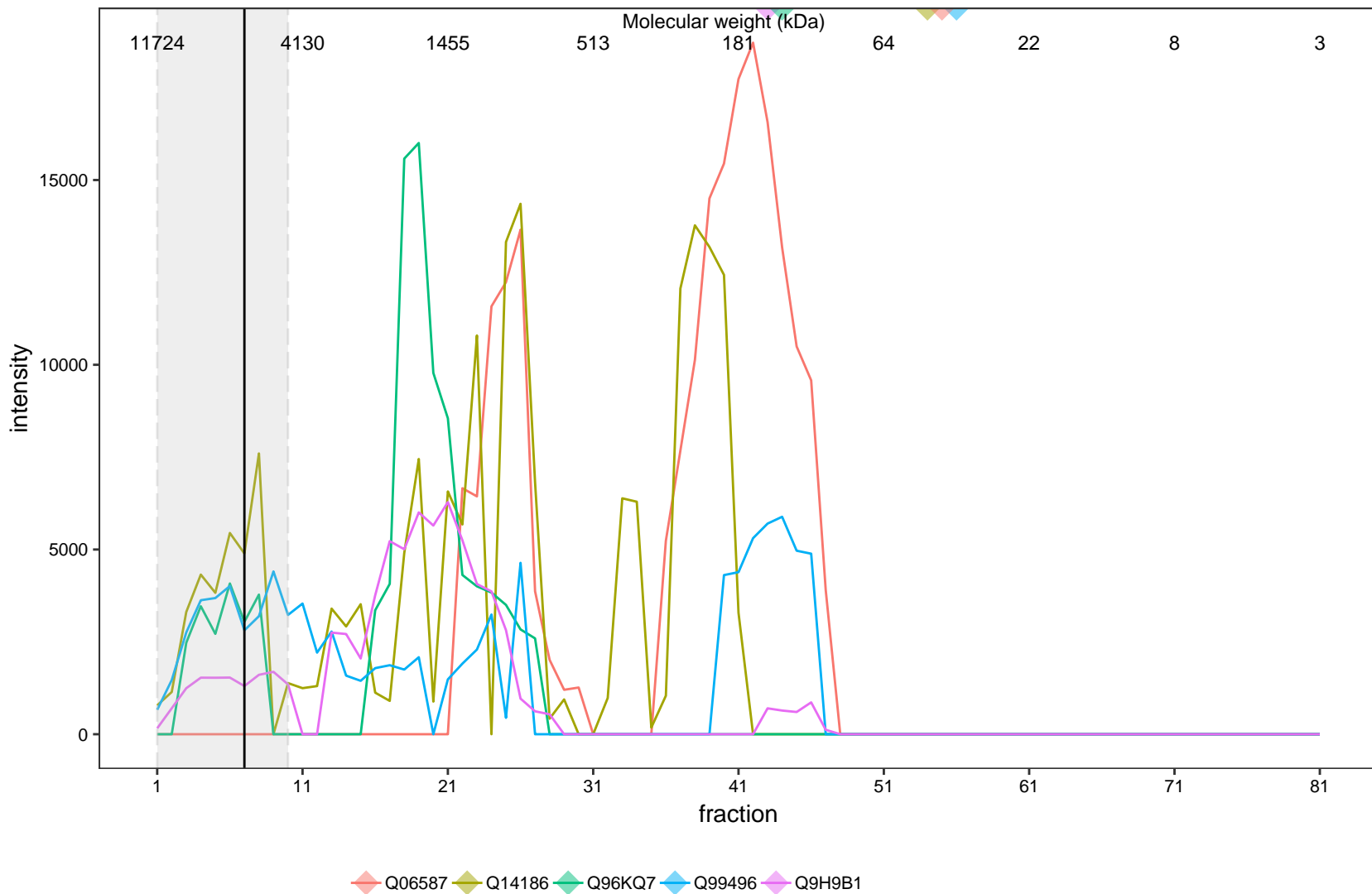

Supplement: Supplementary file 6 — Dataset EV5 [file MSB-15-e8438-s006.zip › feature_plots_corum/1194.pdf]

**Annotated subunits: 8   Subunits with signal: 8**  
**Max. coeluting subunits: 7   Max. completeness: 0.88**

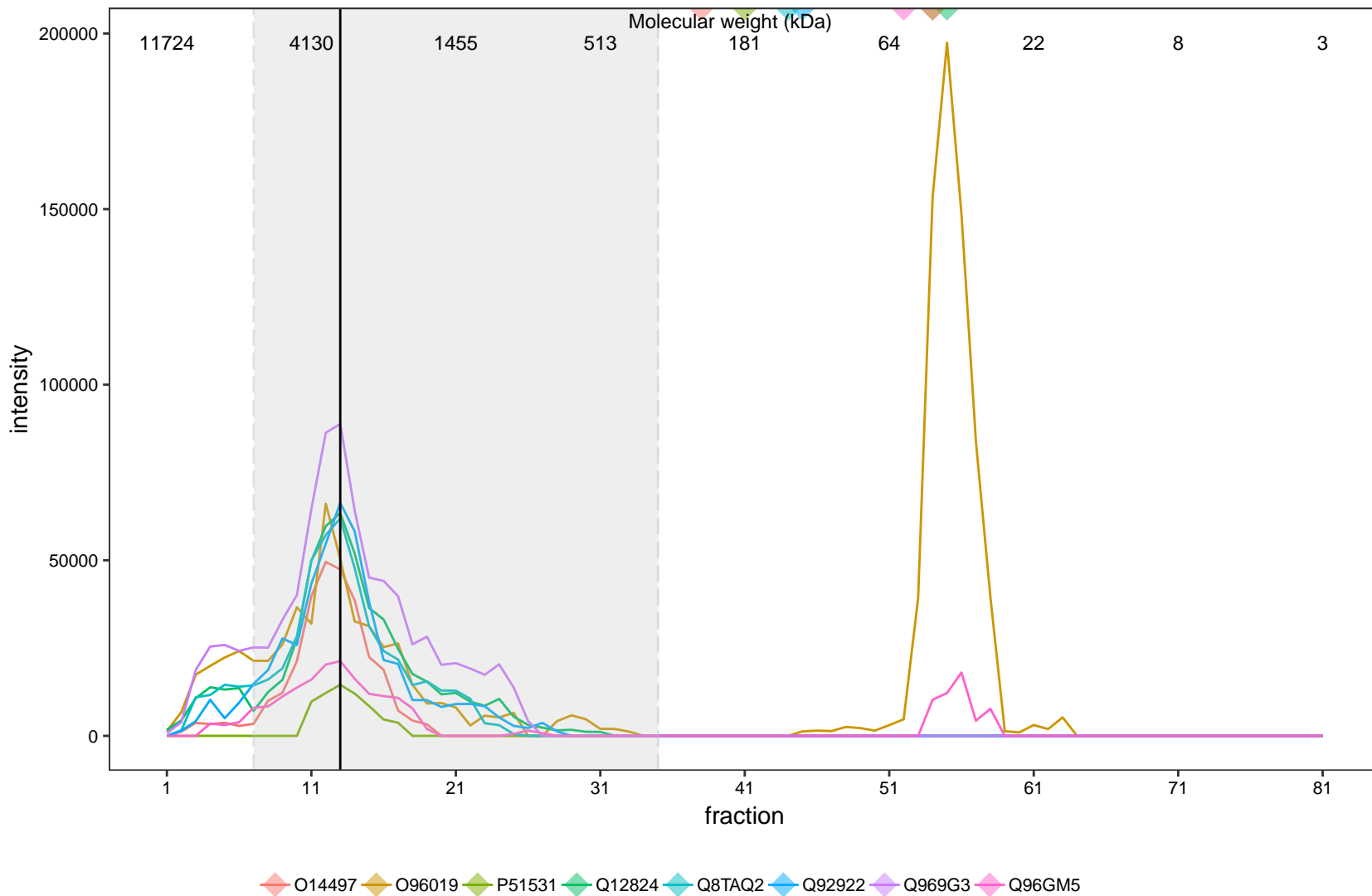

Supplement: Supplementary file 6 — Dataset EV5 [file MSB-15-e8438-s006.zip › feature_plots_corum/1251-1;564-1.pdf]

**Annotated subunits: 8   Subunits with signal: 8**  
**Max. coeluting subunits: 5   Max. completeness: 0.62**

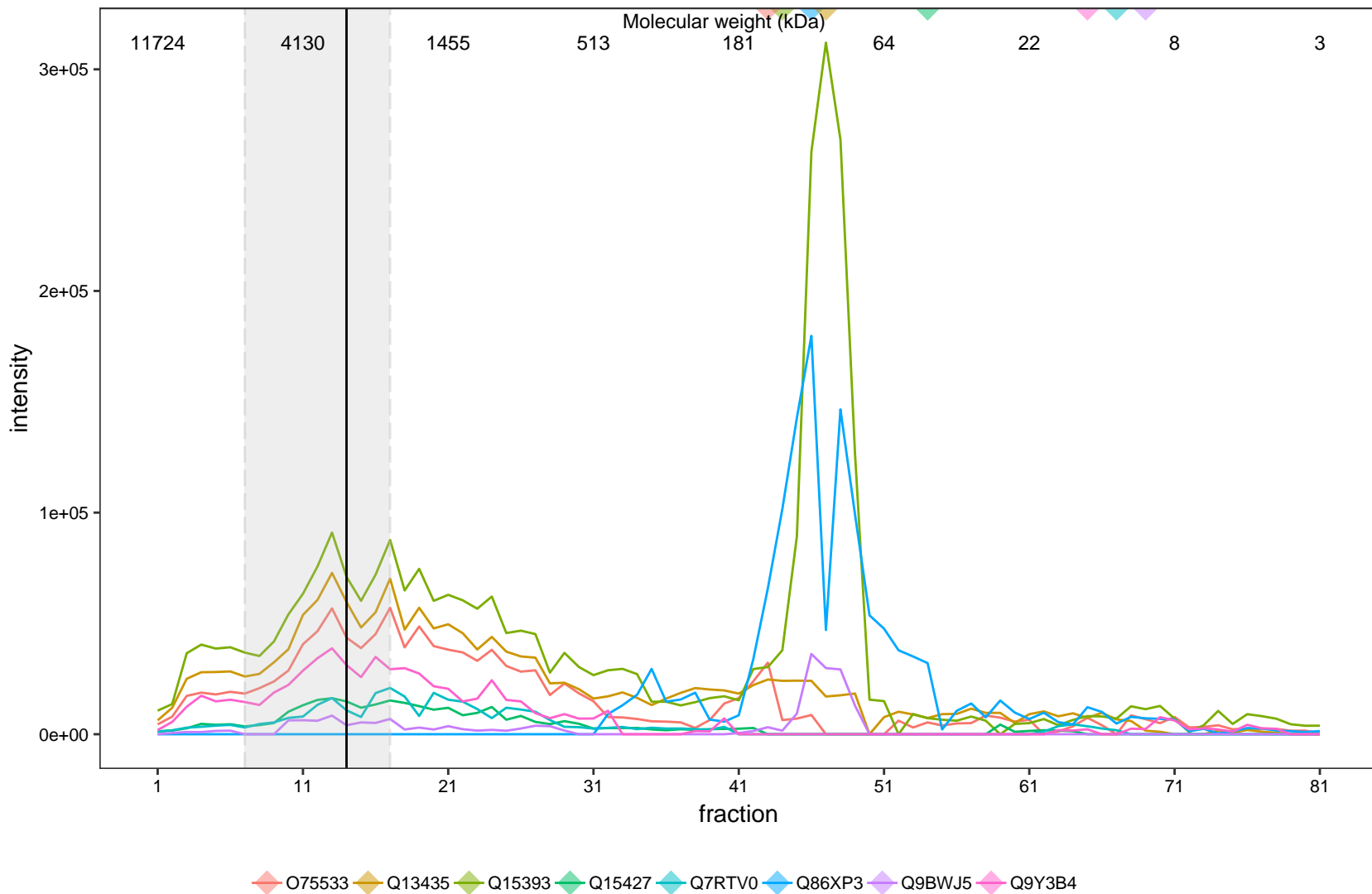

Supplement: Supplementary file 6 — Dataset EV5 [file MSB-15-e8438-s006.zip › feature_plots_corum/1737.pdf]

A1A519

Annotated subunits: 14 Subunits with signal: 7

Max. coeluting subunits: 4 Max. completeness: 0.29

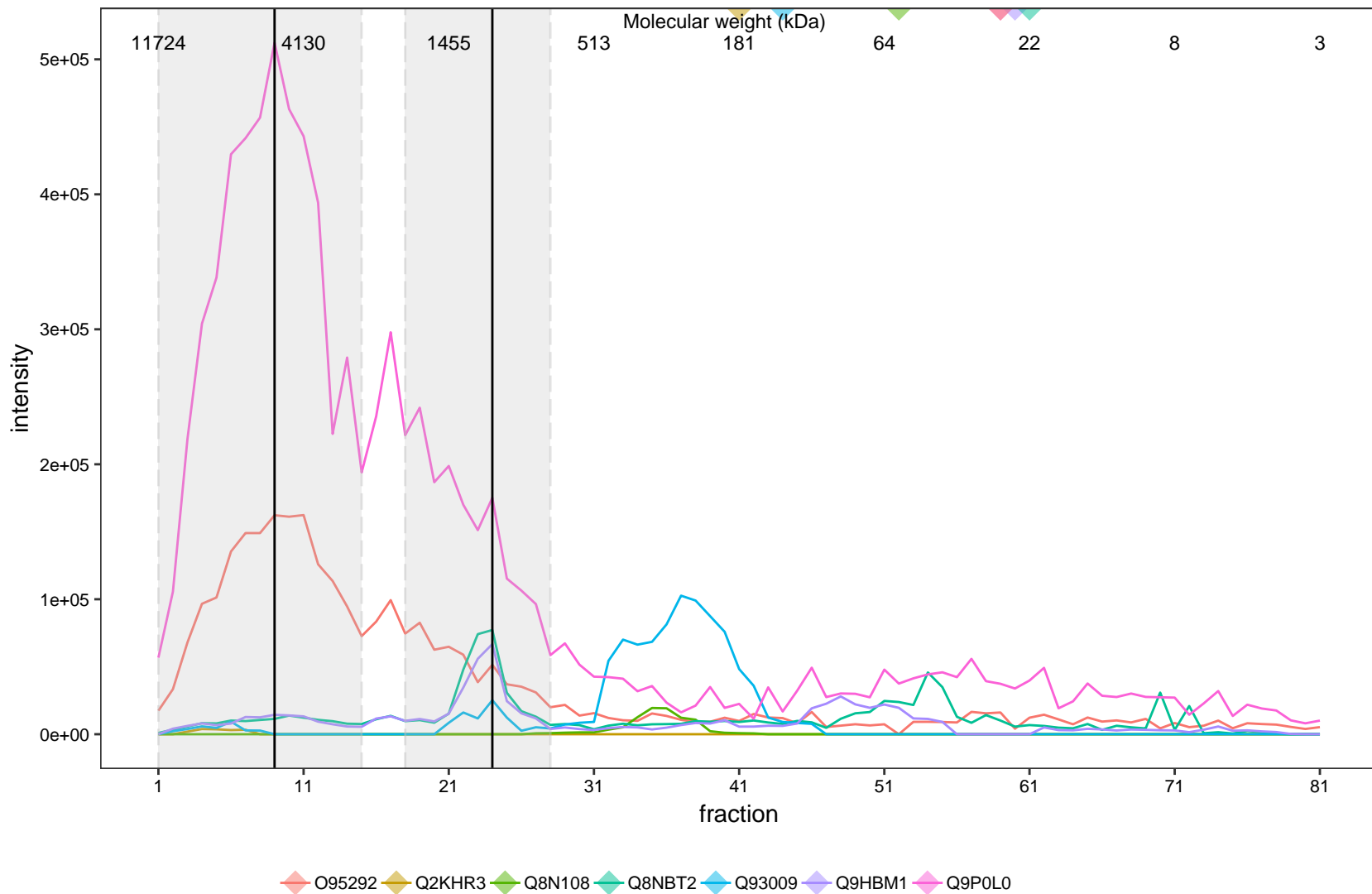

Supplement: Supplementary file 7 — Dataset EV6 [file MSB-15-e8438-s007.zip › feature_plots_bioplex/A1A519.pdf]

# A2RRP1

Annotated subunits: 7 Subunits with signal: 7

Max. coeluting subunits: 3 Max. completeness: 0.43

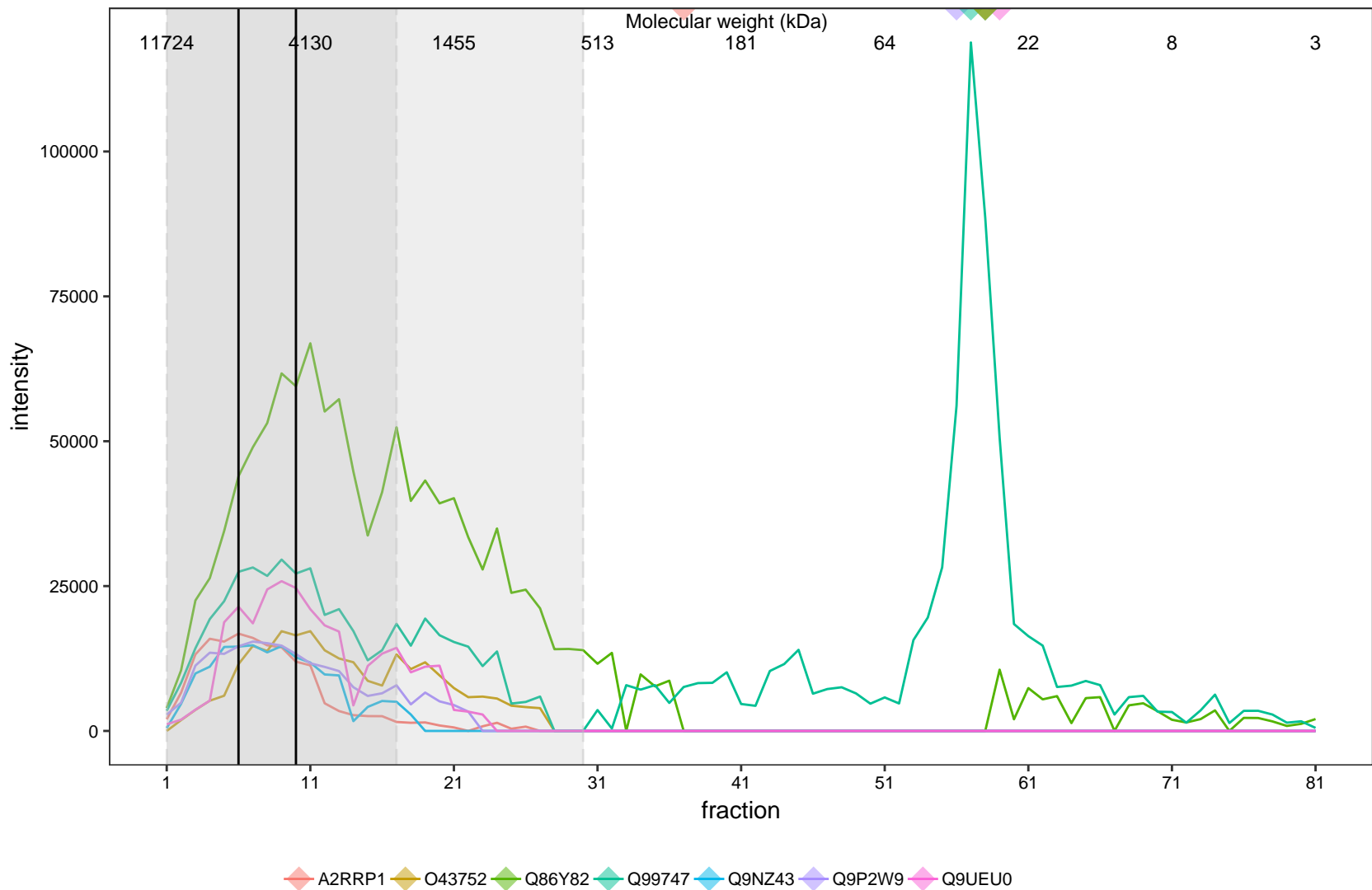

Supplement: Supplementary file 7 — Dataset EV6 [file MSB-15-e8438-s007.zip › feature_plots_bioplex/A2RRP1.pdf]

A2RU30  
Annotated subunits: 10 Subunits with signal: 6  
Max. coeluting subunits: 2 Max. completeness: 0.2

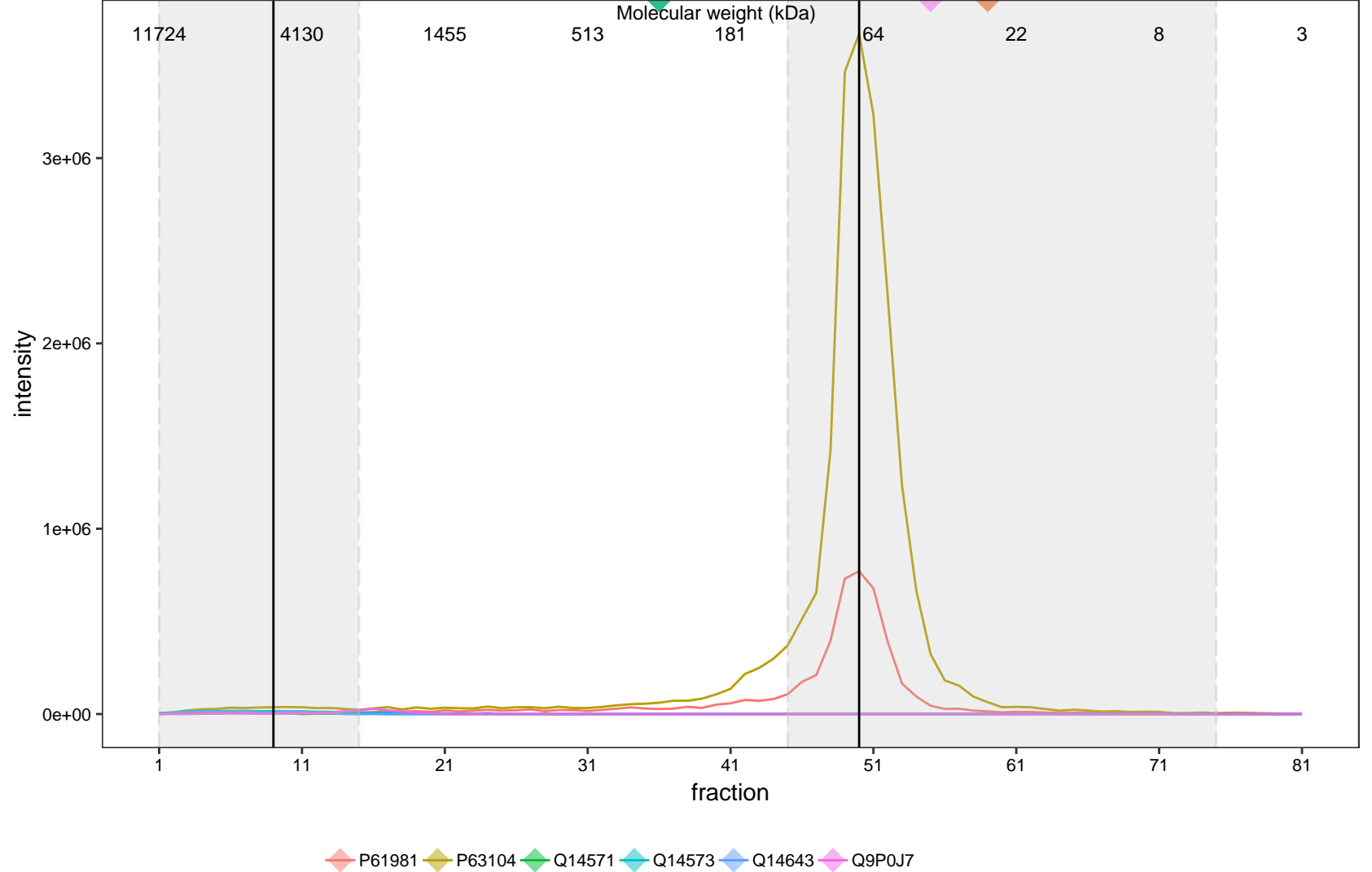

Supplement: Supplementary file 7 — Dataset EV6 [file MSB-15-e8438-s007.zip › feature_plots_bioplex/A2RU30.pdf]

A5YKK6  
Annotated subunits: 7   Subunits with signal: 3  
Max. coeluting subunits: 2   Max. completeness: 0.29

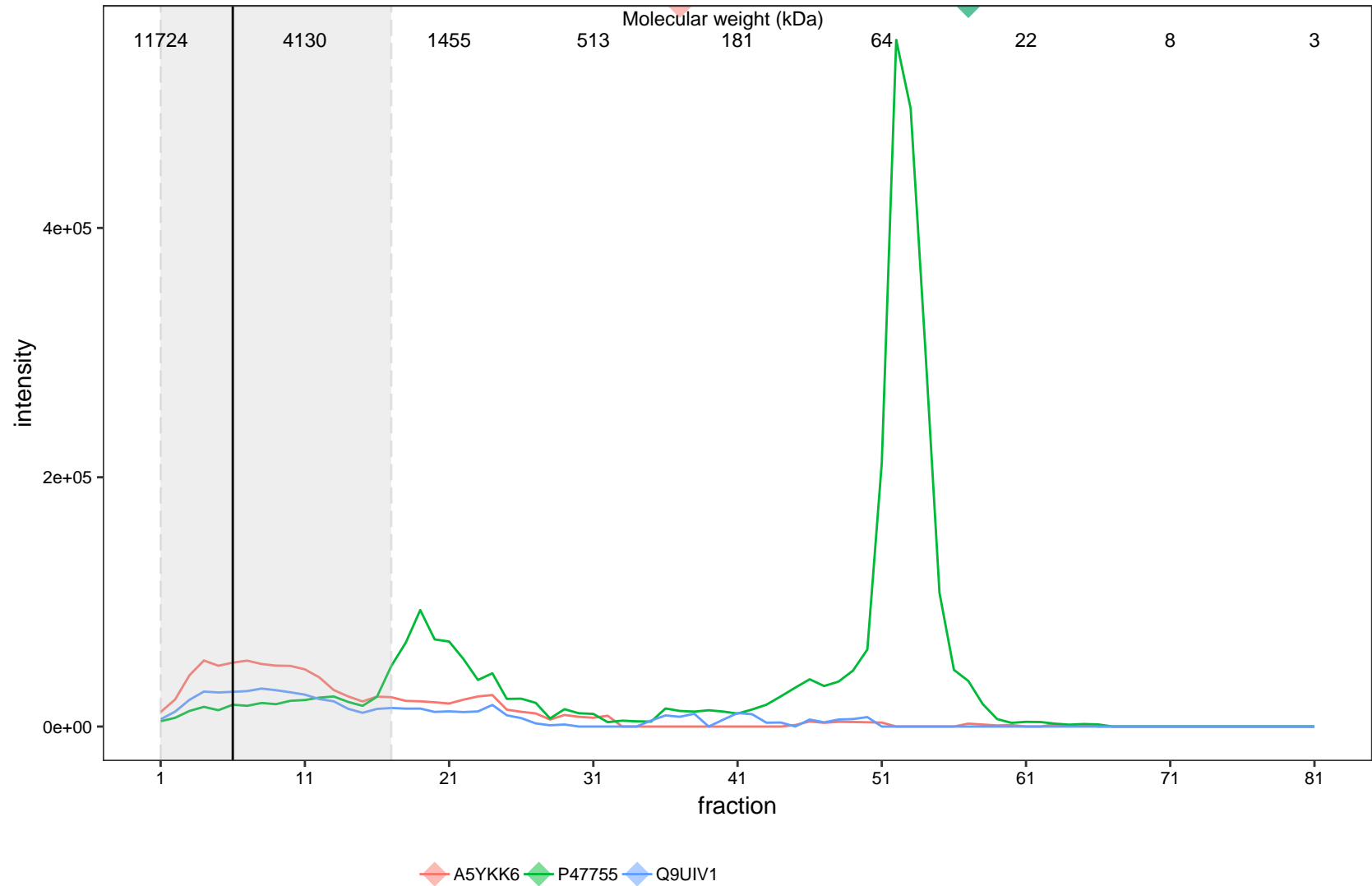

Supplement: Supplementary file 7 — Dataset EV6 [file MSB-15-e8438-s007.zip › feature_plots_bioplex/A5YKK6.pdf]

# A6NHL2

Annotated subunits: 9 Subunits with signal: 6

Max. coeluting subunits: 6 Max. completeness: 0.67

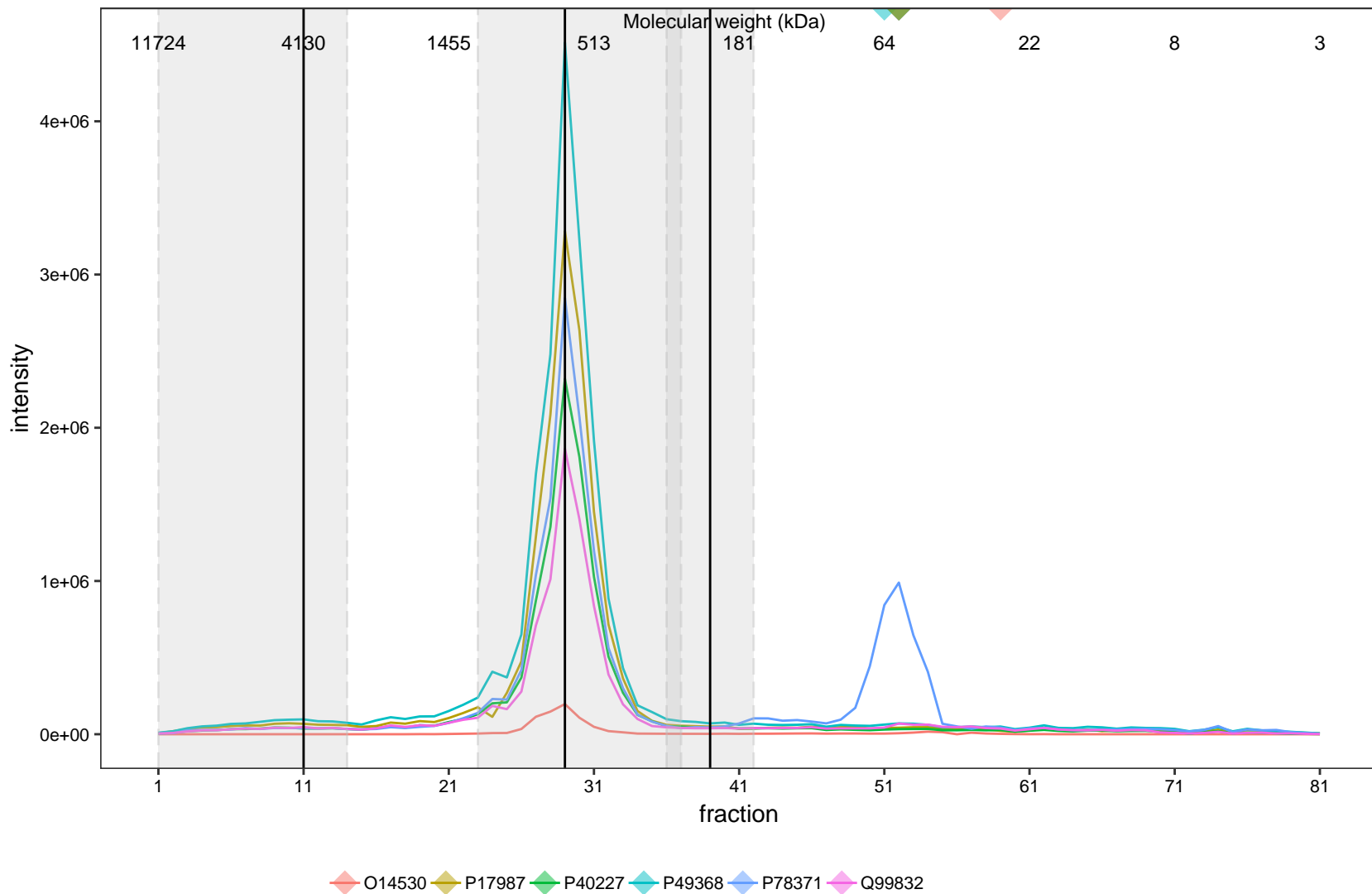

Supplement: Supplementary file 7 — Dataset EV6 [file MSB-15-e8438-s007.zip › feature_plots_bioplex/A6NHL2.pdf]

**A8MW92**  
**Annotated subunits: 15   Subunits with signal: 11**  
**Max. coeluting subunits: 10   Max. completeness: 0.67**

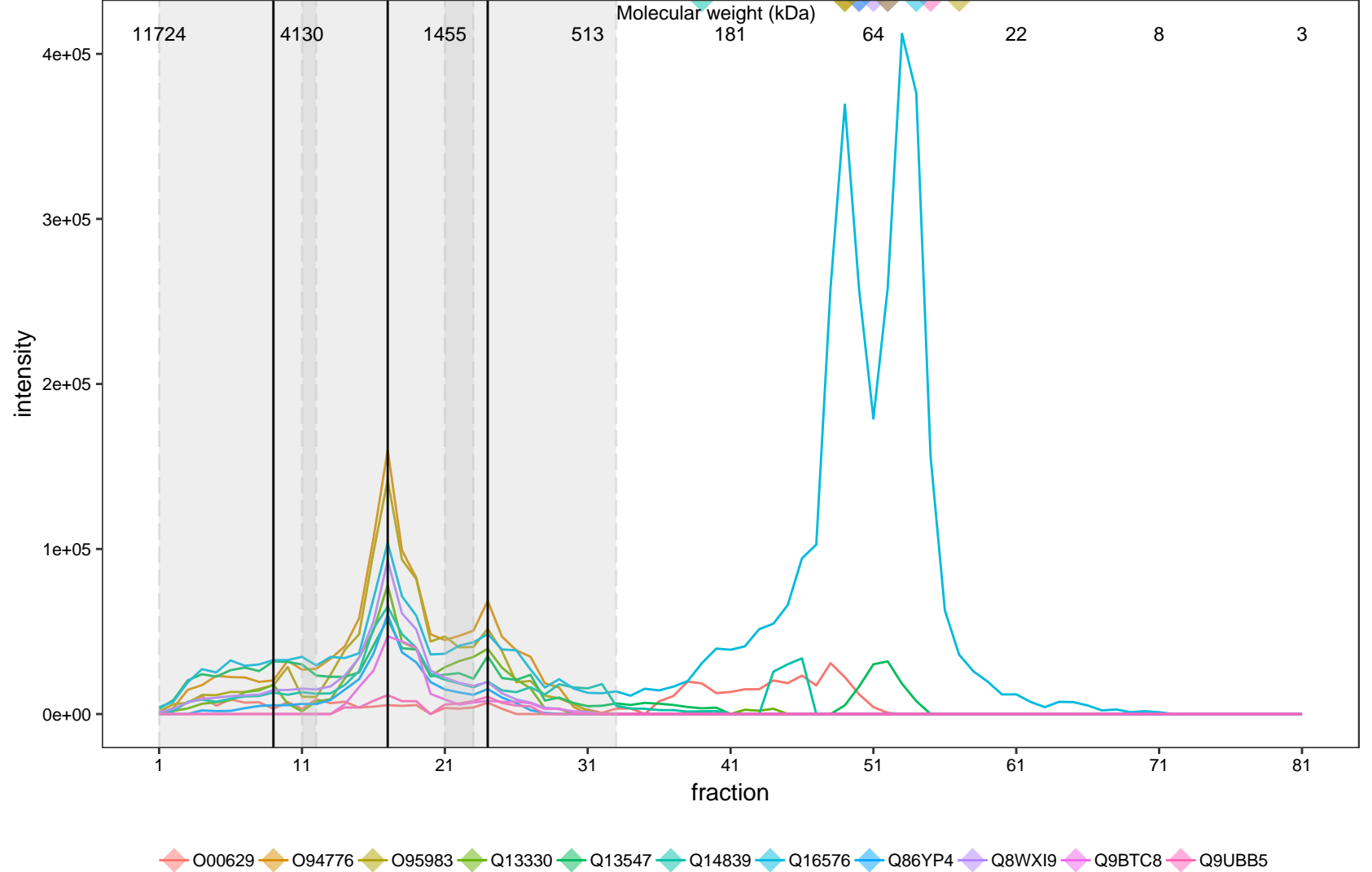

Supplement: Supplementary file 7 — Dataset EV6 [file MSB-15-e8438-s007.zip › feature_plots_bioplex/A8MW92.pdf]

# B0FP48

Annotated subunits: 14 Subunits with signal: 5

Max. coeluting subunits: 4 Max. completeness: 0.29

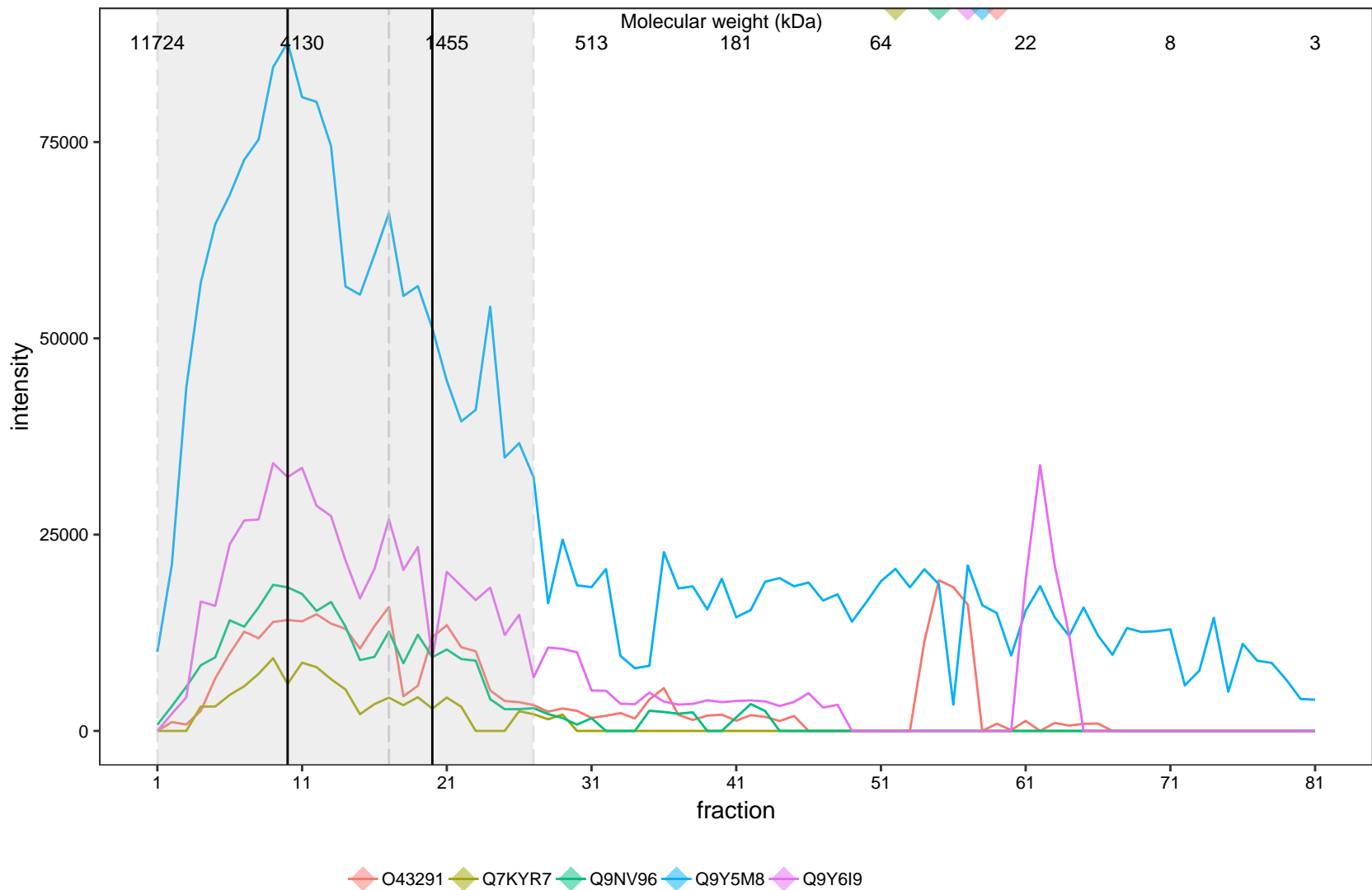

Supplement: Supplementary file 7 — Dataset EV6 [file MSB-15-e8438-s007.zip › feature_plots_bioplex/B0FP48.pdf]

**B4DTM2**  
Annotated subunits: 4   Subunits with signal: 2  
Max. coeluting subunits: 2   Max. completeness: 0.5

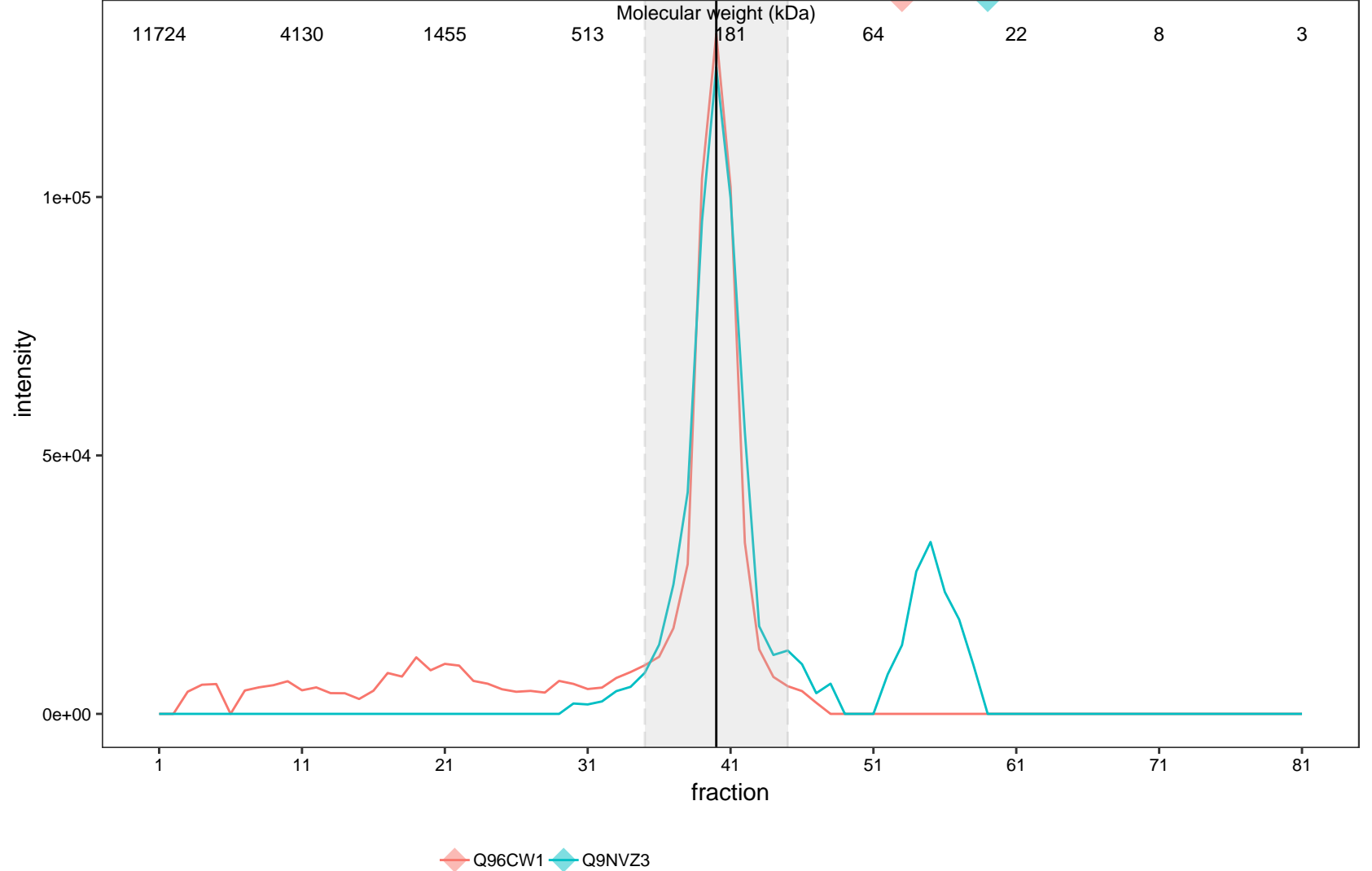

Supplement: Supplementary file 7 — Dataset EV6 [file MSB-15-e8438-s007.zip › feature_plots_bioplex/B4DTM2.pdf]

**B5ME19**  
Annotated subunits: 5   Subunits with signal: 4  
Max. coeluting subunits: 4   Max. completeness: 0.8

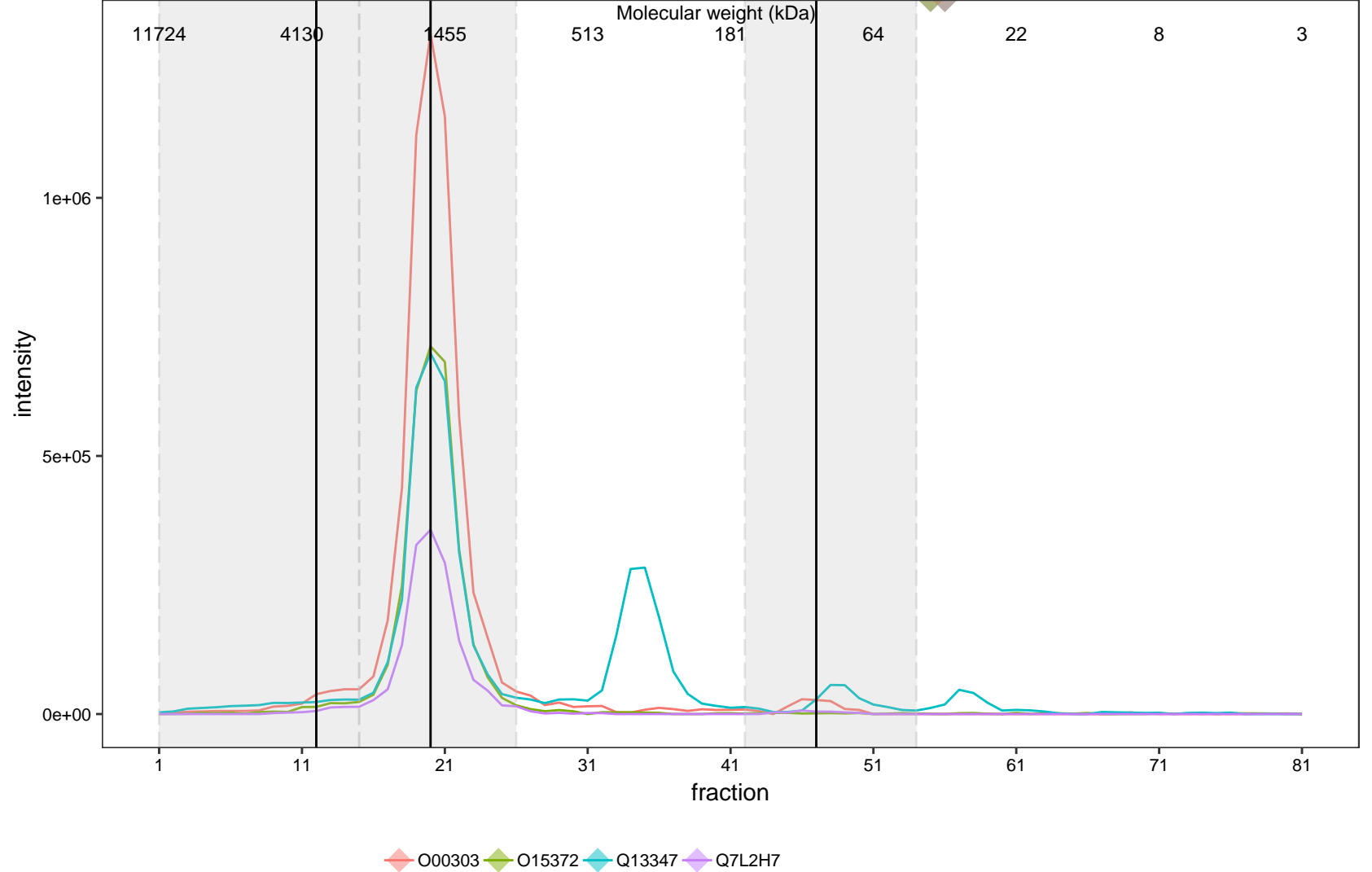

Supplement: Supplementary file 7 — Dataset EV6 [file MSB-15-e8438-s007.zip › feature_plots_bioplex/B5ME19.pdf]

C9JP52  
Annotated subunits: 3   Subunits with signal: 2  
Max. coeluting subunits: 2   Max. completeness: 0.67

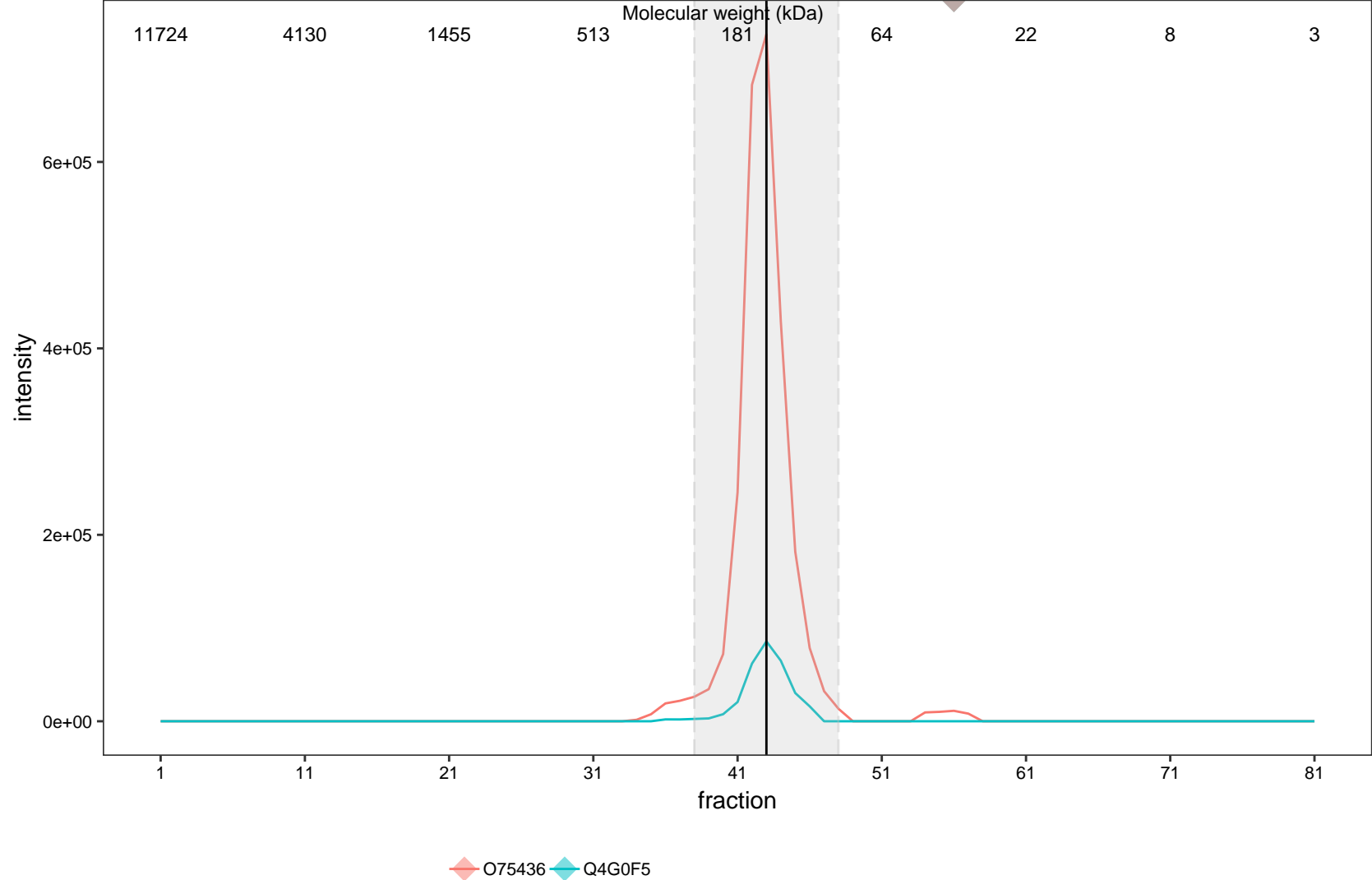

Supplement: Supplementary file 7 — Dataset EV6 [file MSB-15-e8438-s007.zip › feature_plots_bioplex/C9JP52.pdf]

D6RGH0  
Annotated subunits: 14   Subunits with signal: 7  
Max. coeluting subunits: 4   Max. completeness: 0.29

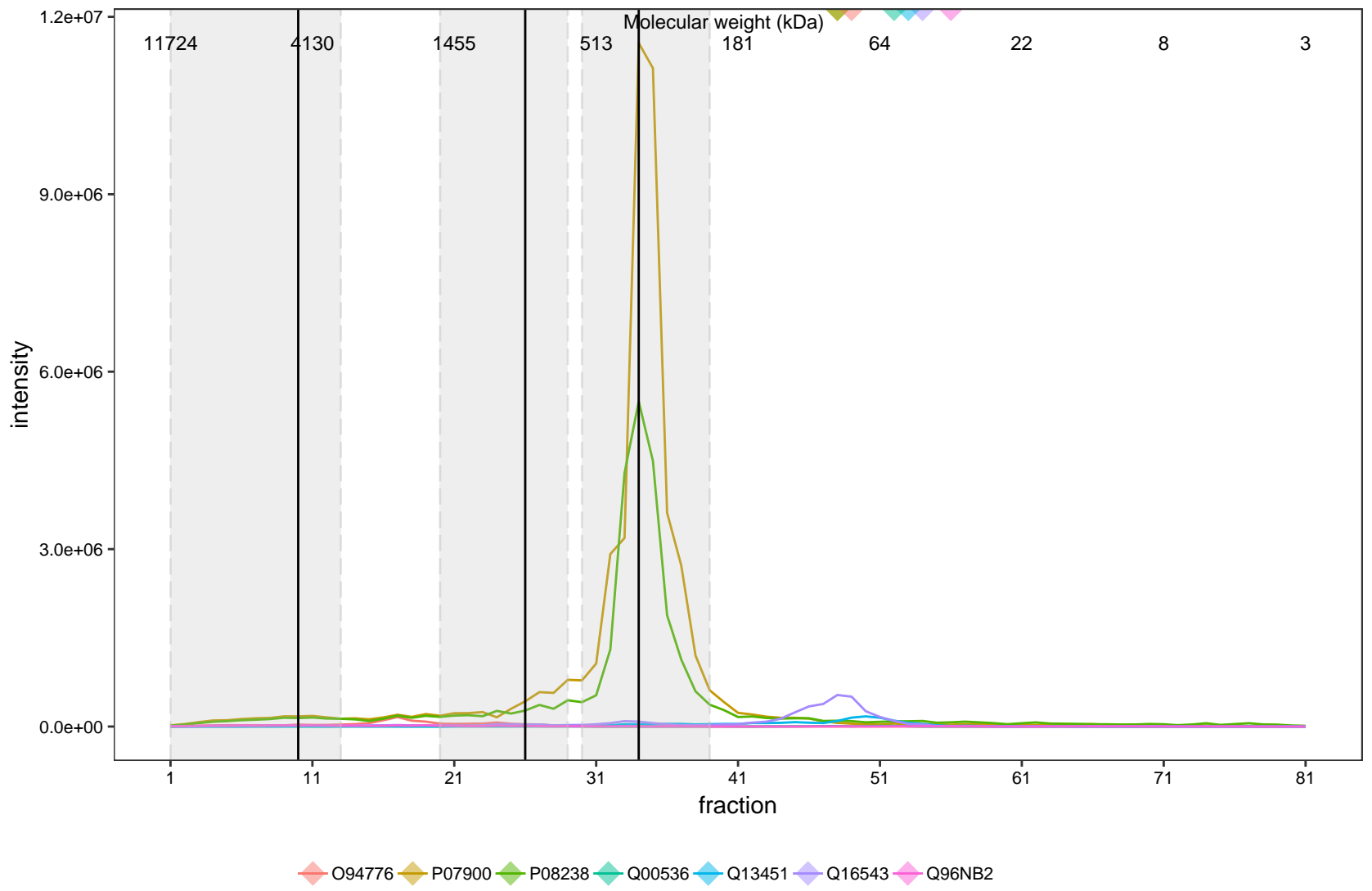

Supplement: Supplementary file 7 — Dataset EV6 [file MSB-15-e8438-s007.zip › feature_plots_bioplex/D6RGH0.pdf]

E9PD40  
Annotated subunits: 7   Subunits with signal: 4  
Max. coeluting subunits: 3   Max. completeness: 0.43

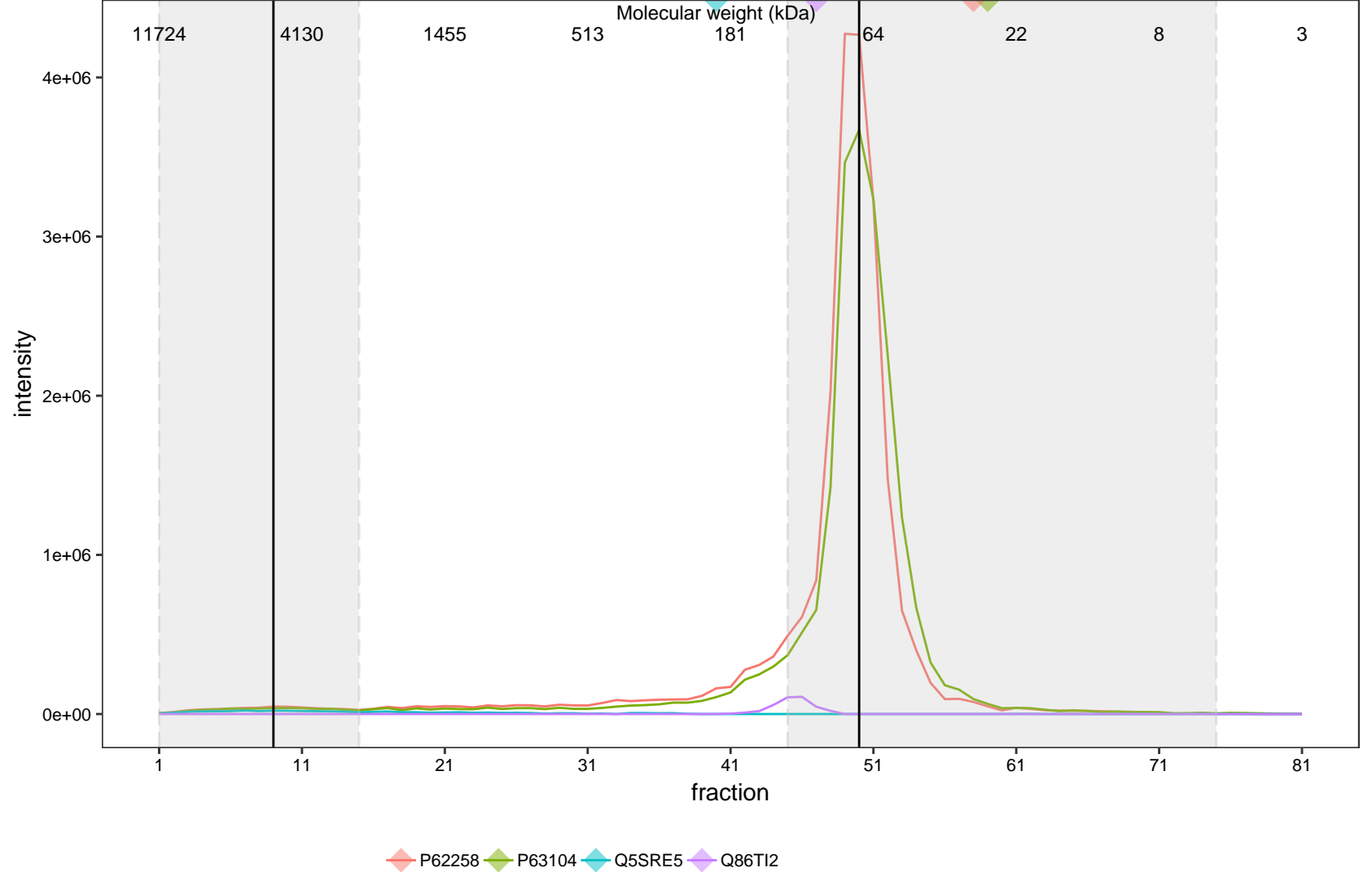

Supplement: Supplementary file 7 — Dataset EV6 [file MSB-15-e8438-s007.zip › feature_plots_bioplex/E9PD40.pdf]

# E9PFU9

Annotated subunits: 11 Subunits with signal: 8

Max. coeluting subunits: 4 Max. completeness: 0.36

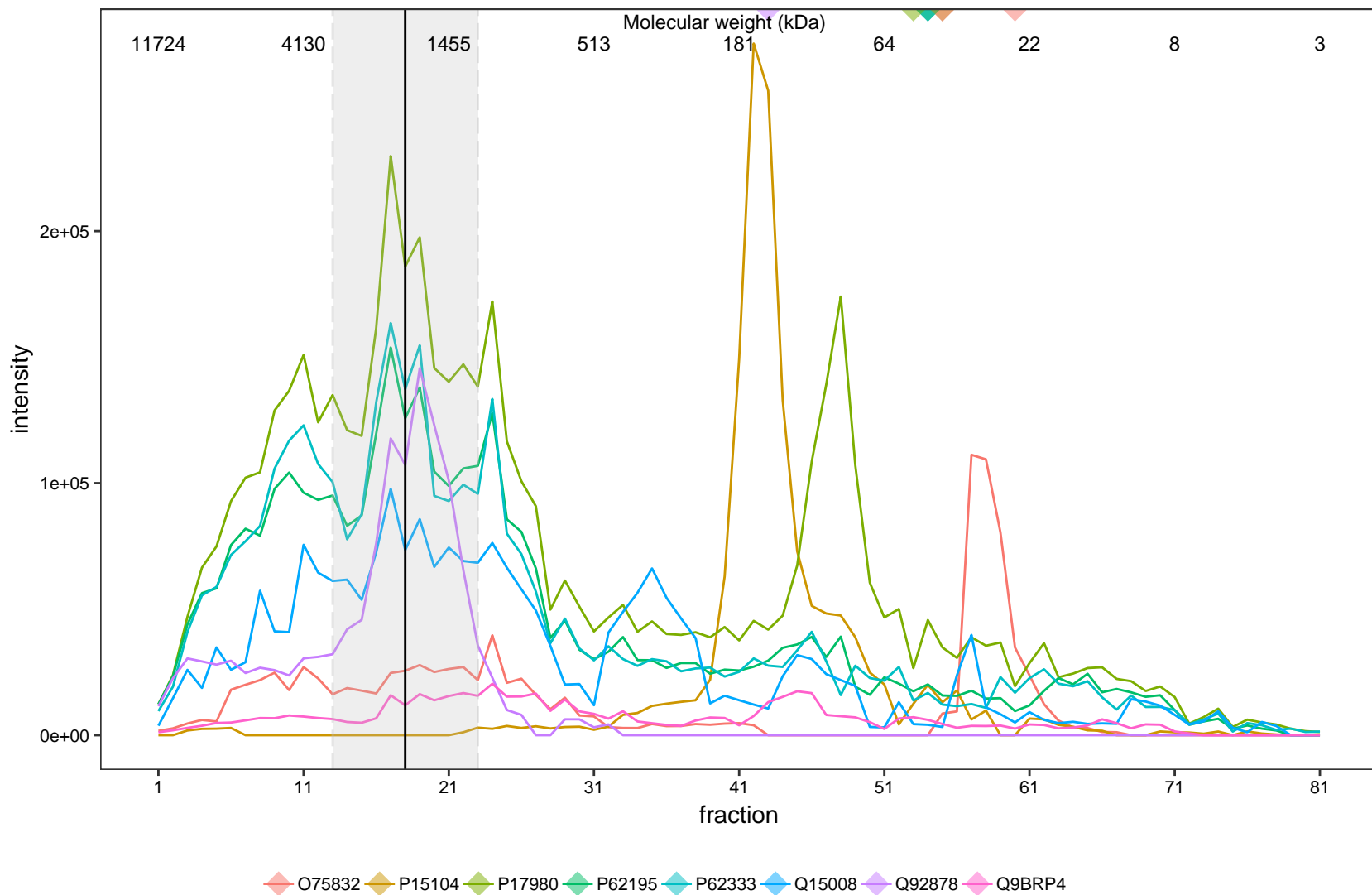

Supplement: Supplementary file 7 — Dataset EV6 [file MSB-15-e8438-s007.zip › feature_plots_bioplex/E9PFU9.pdf]

F5H1R9  
Annotated subunits: 10 Subunits with signal: 5  
Max. coeluting subunits: 2 Max. completeness: 0.2

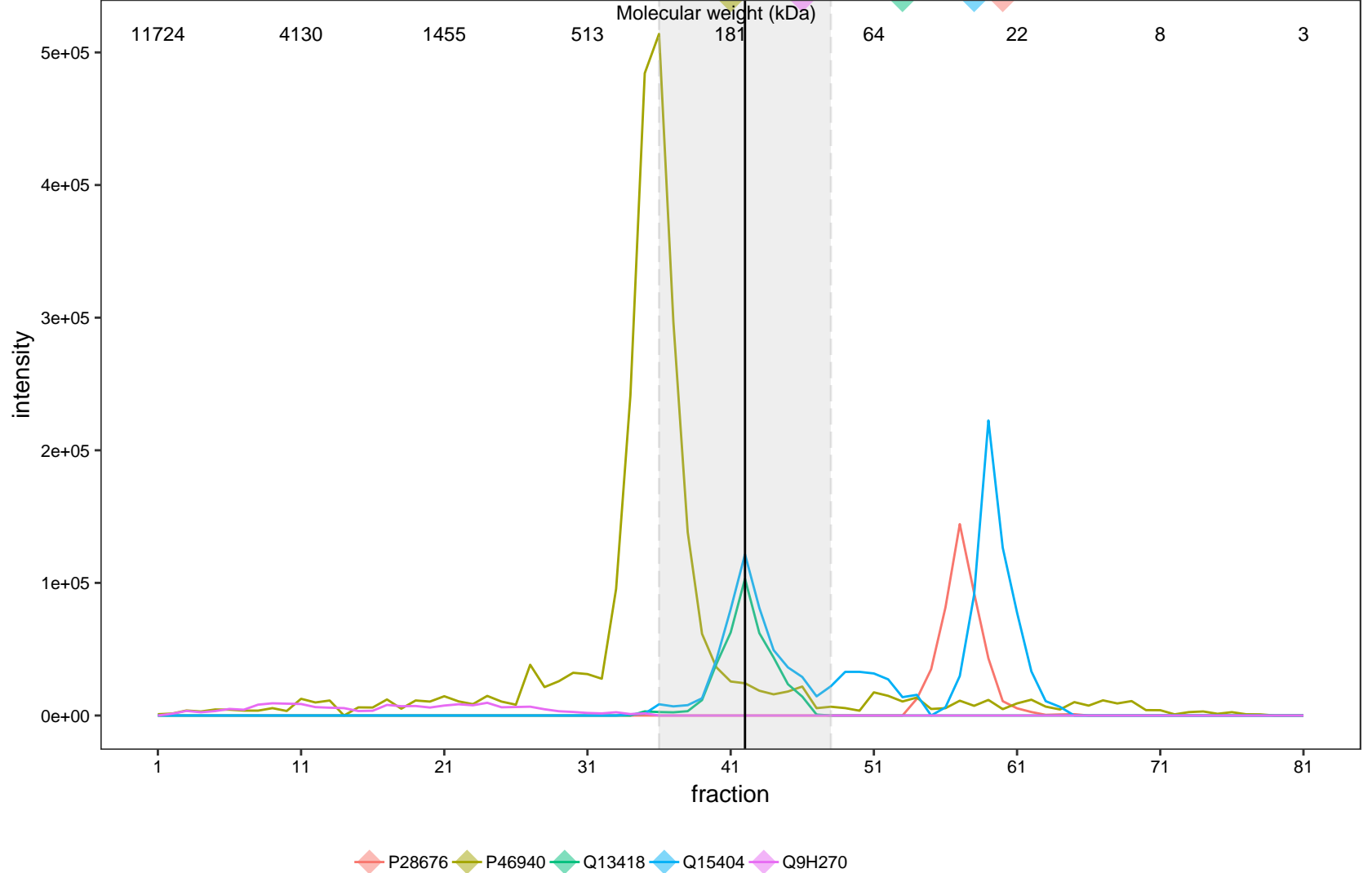

Supplement: Supplementary file 7 — Dataset EV6 [file MSB-15-e8438-s007.zip › feature_plots_bioplex/F5H1R9.pdf]

O00151  
Annotated subunits: 7   Subunits with signal: 5  
Max. coeluting subunits: 2   Max. completeness: 0.29

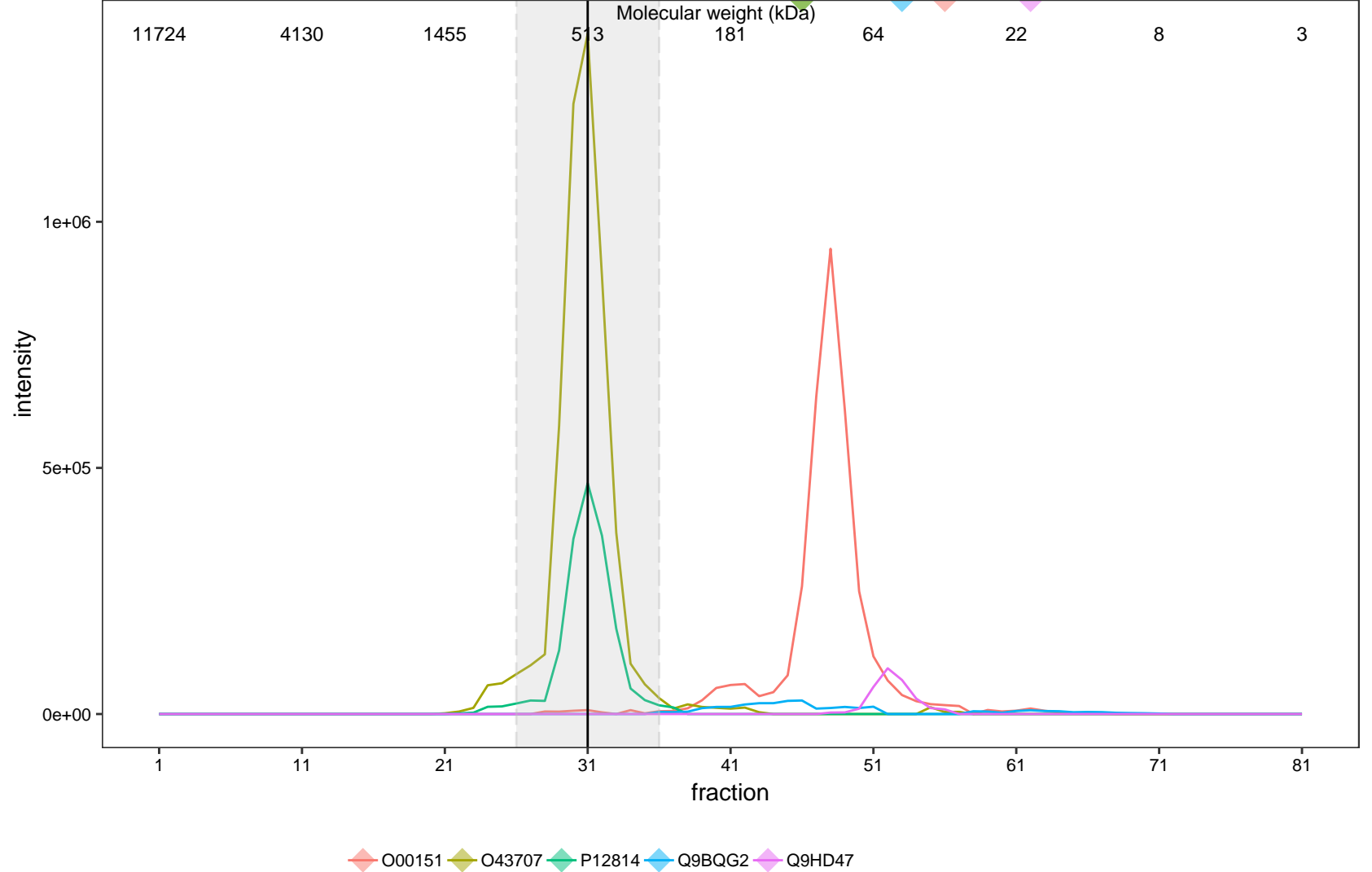

Supplement: Supplementary file 7 — Dataset EV6 [file MSB-15-e8438-s007.zip › feature_plots_bioplex/O00151.pdf]

**O00161**

**Annotated subunits: 6 Subunits with signal: 4**

**Max. coeluting subunits: 4 Max. completeness: 0.67**

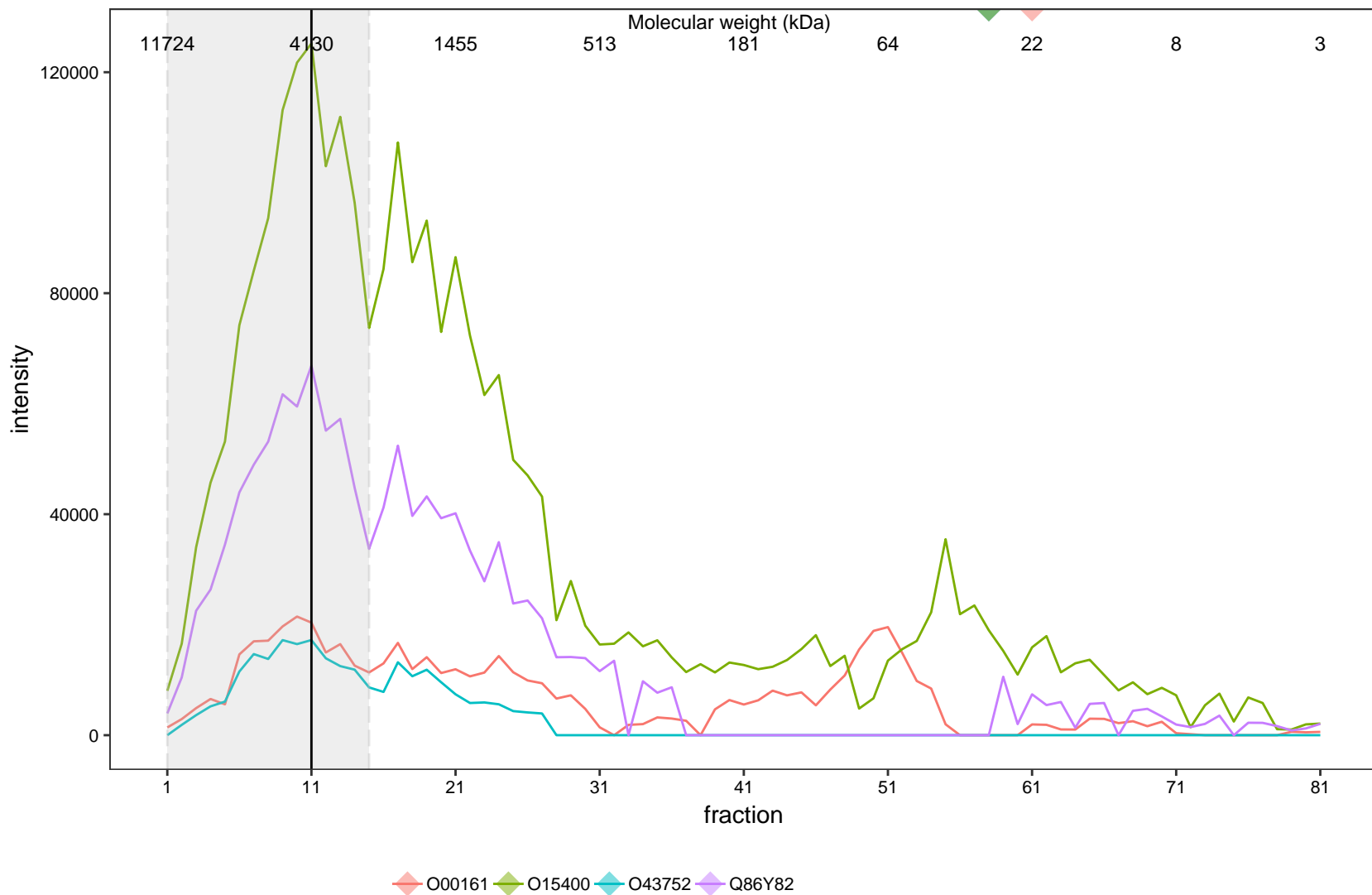

Supplement: Supplementary file 7 — Dataset EV6 [file MSB-15-e8438-s007.zip › feature_plots_bioplex/O00161.pdf]

**O00217**

**Annotated subunits: 4 Subunits with signal: 4**

**Max. coeluting subunits: 4 Max. completeness: 1**

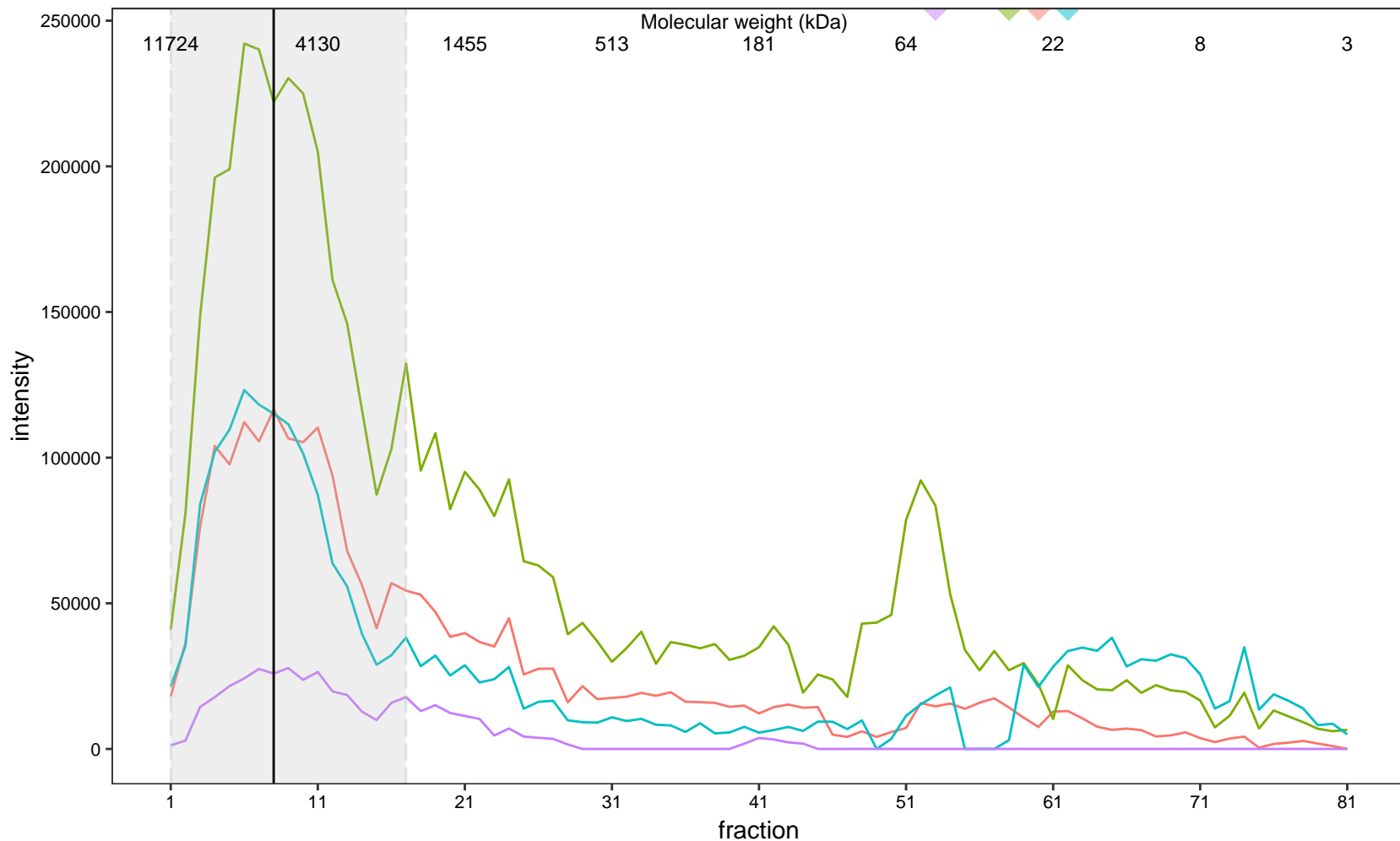

◊ O00217 ◊ O75489 ◊ P51970 ◊ Q9BQ95

Supplement: Supplementary file 7 — Dataset EV6 [file MSB-15-e8438-s007.zip › feature_plots_bioplex/O00217.pdf]

**O00231**  
**Annotated subunits: 10   Subunits with signal: 7**  
**Max. coeluting subunits: 4   Max. completeness: 0.4**

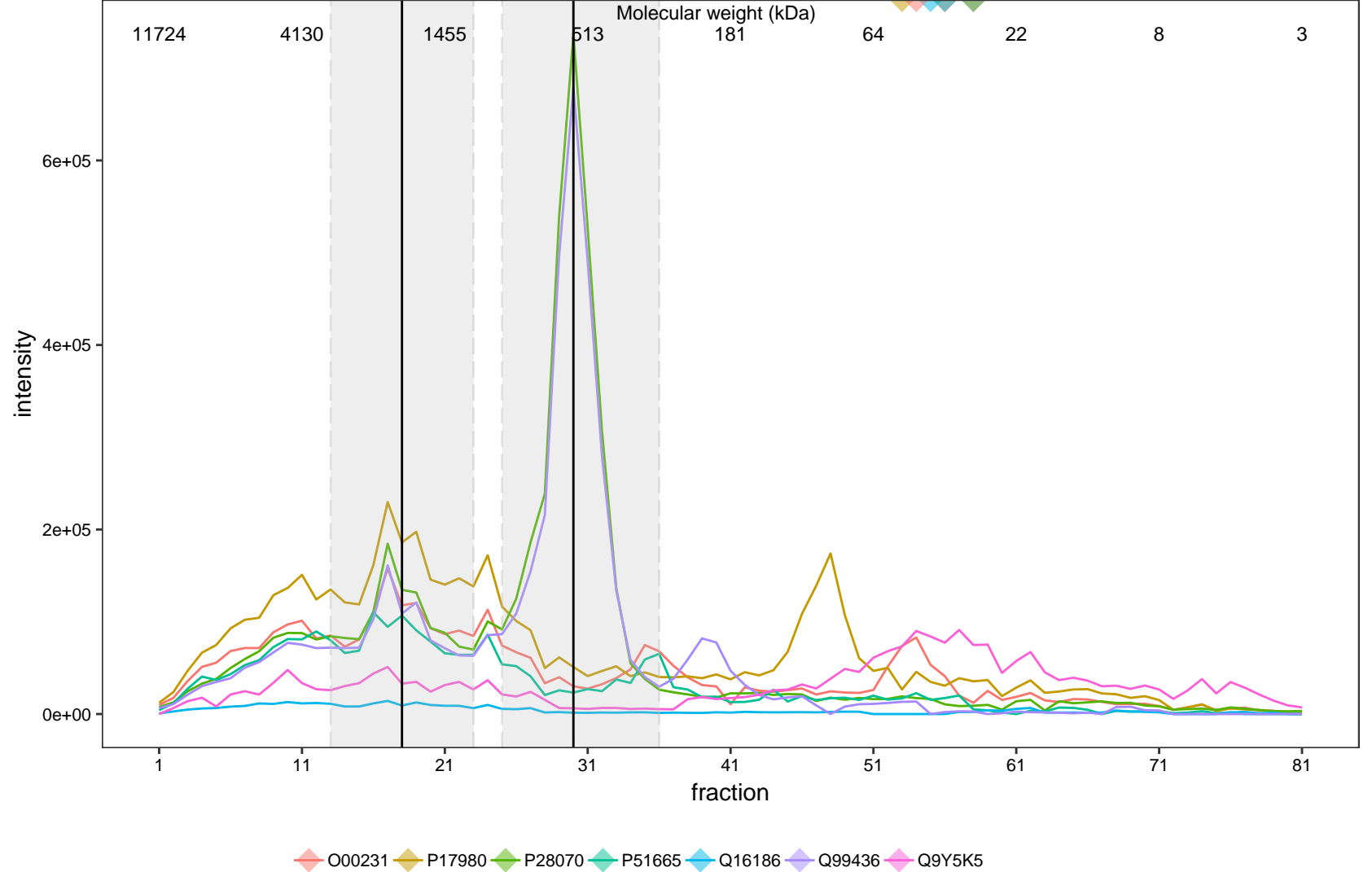

Supplement: Supplementary file 7 — Dataset EV6 [file MSB-15-e8438-s007.zip › feature_plots_bioplex/O00231.pdf]

**O00232**

**Annotated subunits: 10 Subunits with signal: 7**

**Max. coeluting subunits: 3 Max. completeness: 0.3**

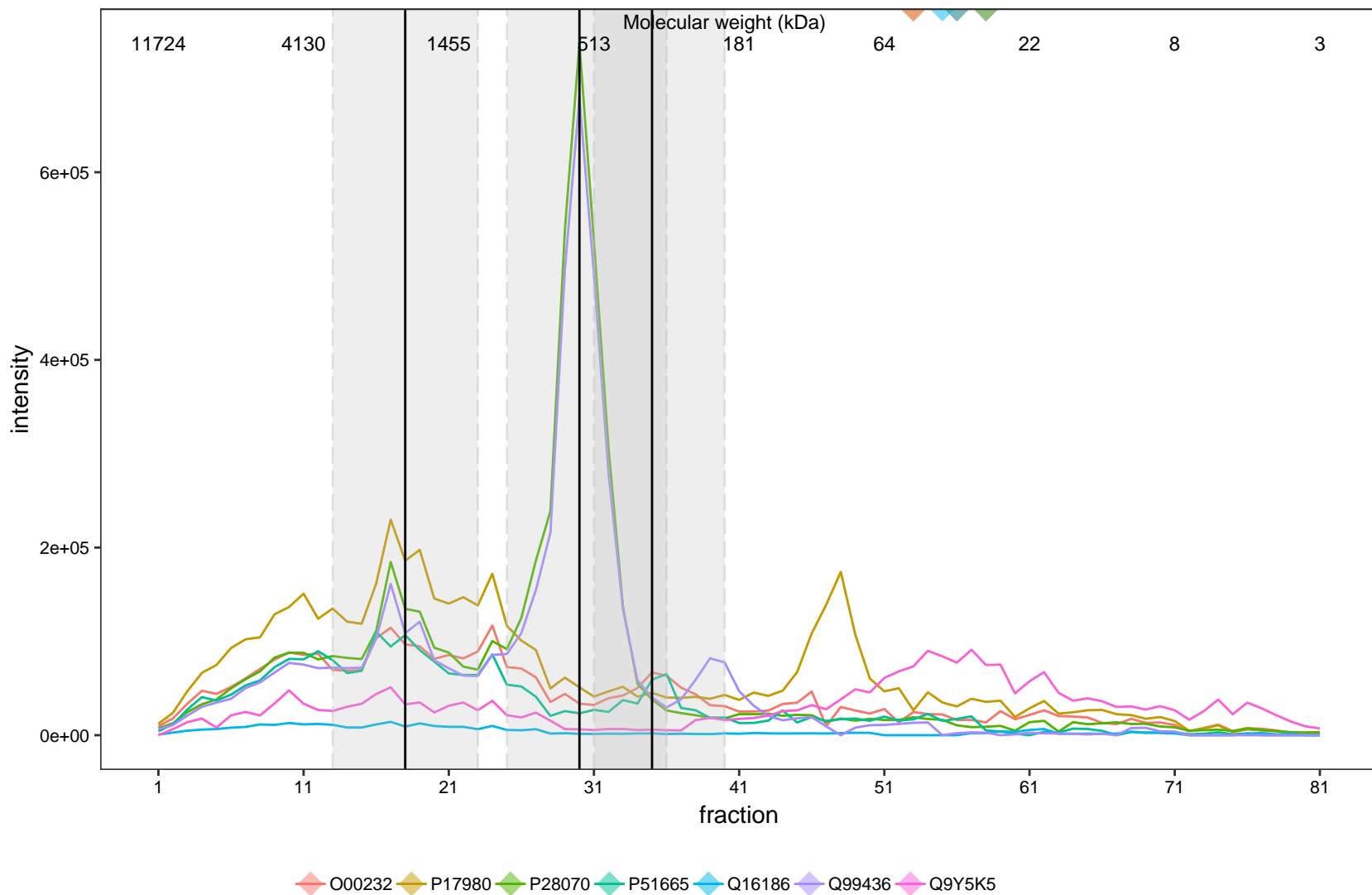

Supplement: Supplementary file 7 — Dataset EV6 [file MSB-15-e8438-s007.zip › feature_plots_bioplex/O00232.pdf]

**O00233**

**Annotated subunits: 4 Subunits with signal: 4**

**Max. coeluting subunits: 2 Max. completeness: 0.5**

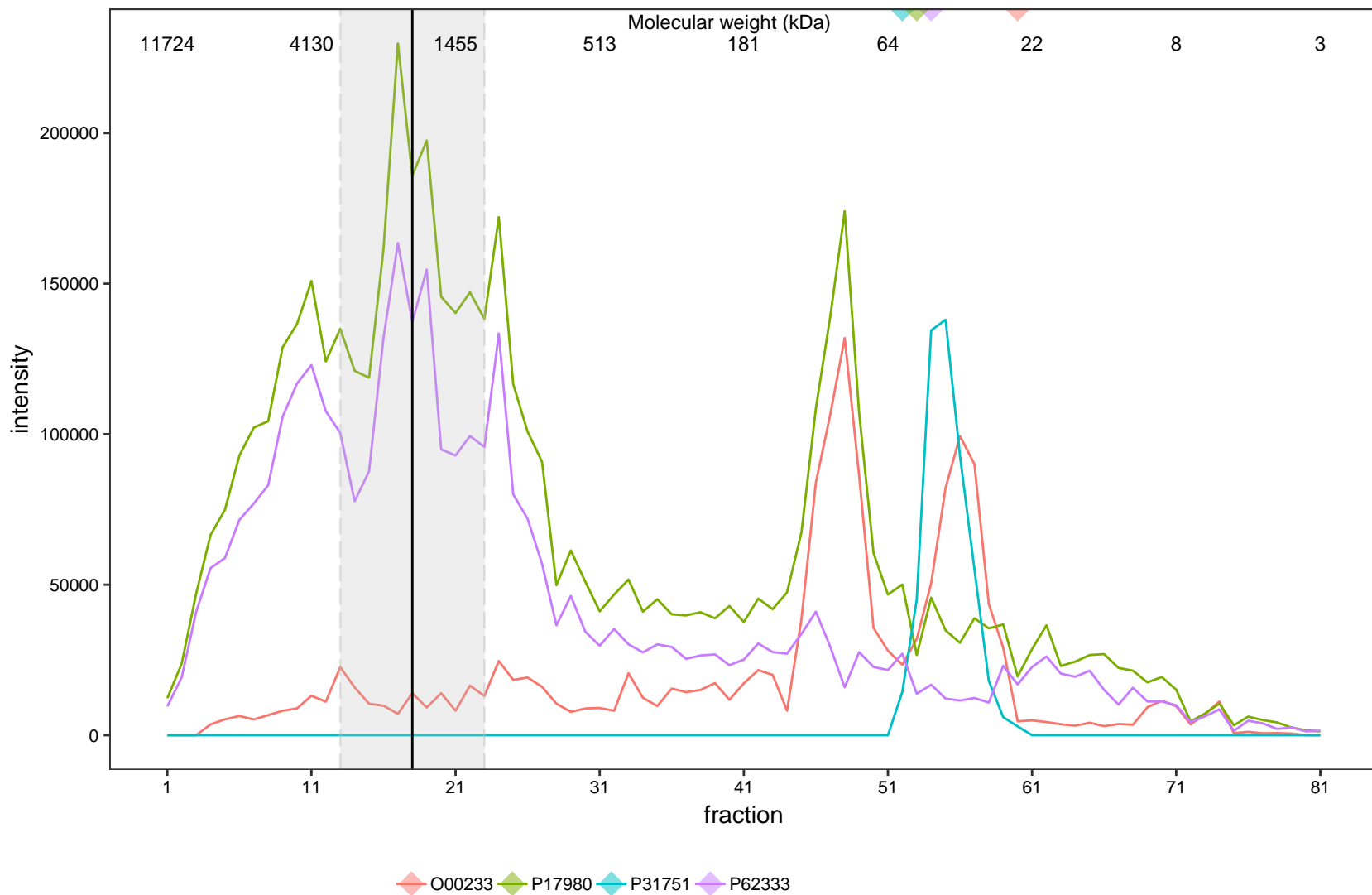

Supplement: Supplementary file 7 — Dataset EV6 [file MSB-15-e8438-s007.zip › feature_plots_bioplex/O00233.pdf]

**O00273**

**Annotated subunits: 4 Subunits with signal: 3**

**Max. coeluting subunits: 2 Max. completeness: 0.5**

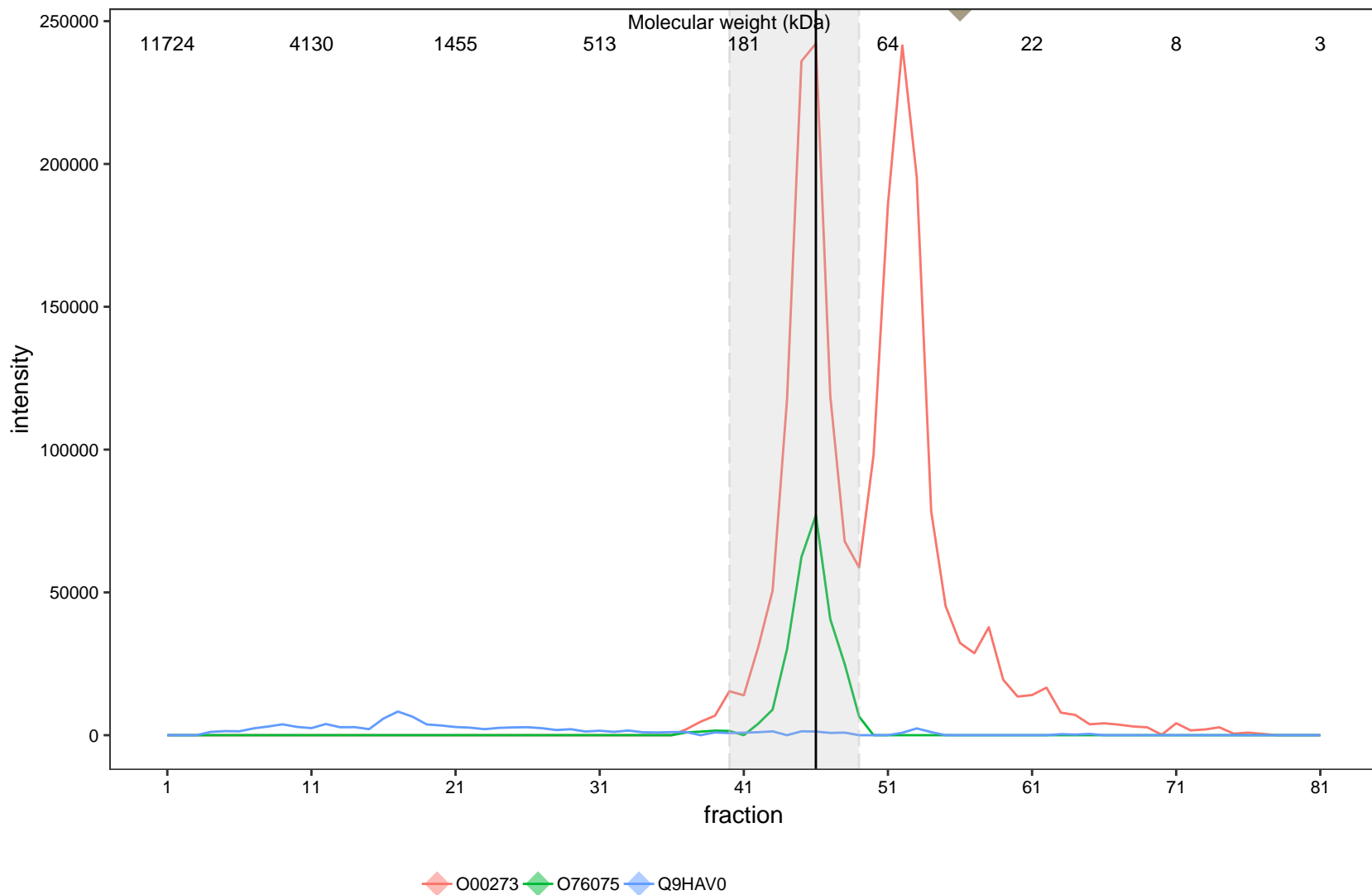

Supplement: Supplementary file 7 — Dataset EV6 [file MSB-15-e8438-s007.zip › feature_plots_bioplex/O00273.pdf]

O00303

Annotated subunits: 23 Subunits with signal: 17

Max. coeluting subunits: 13 Max. completeness: 0.57

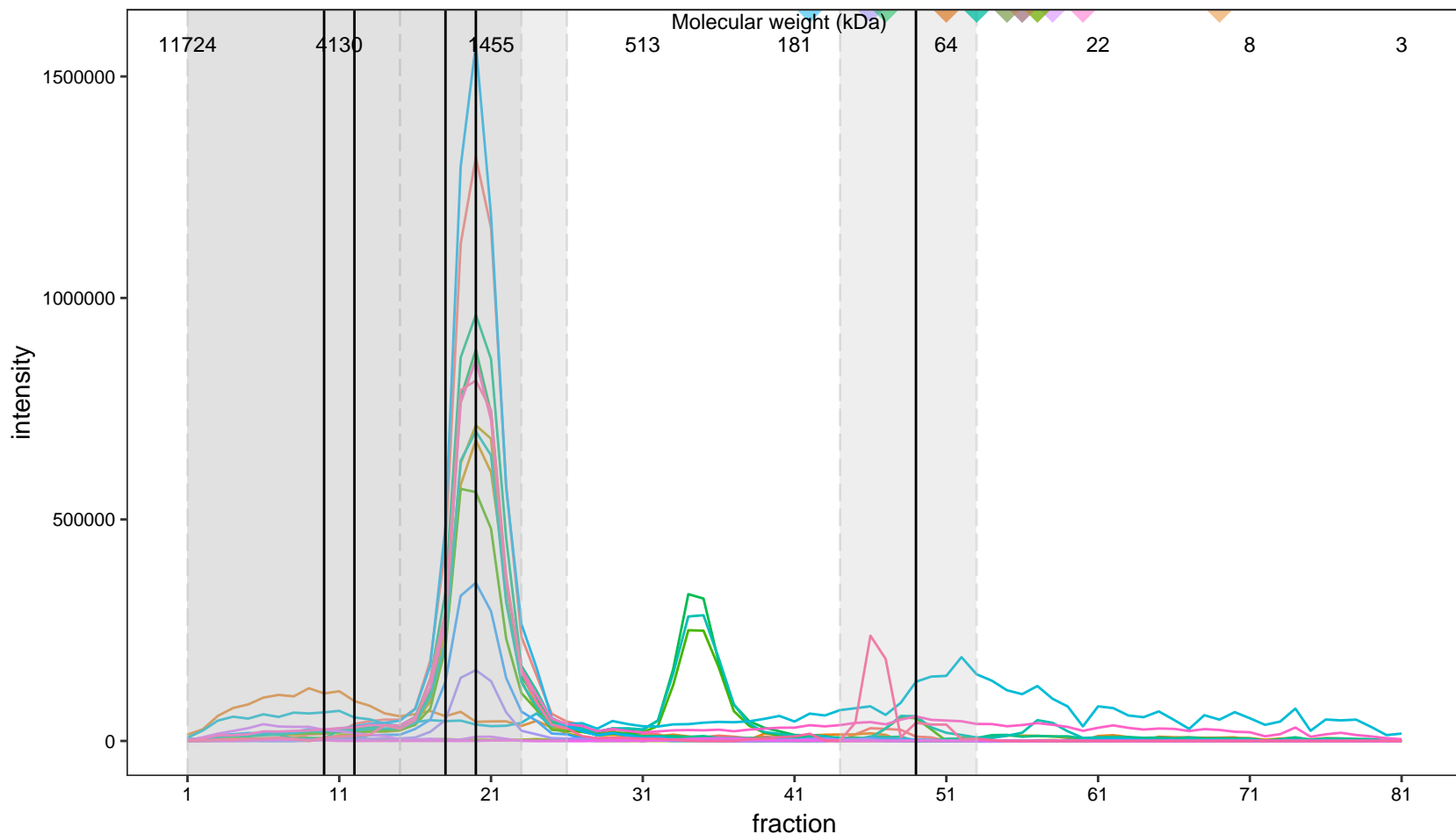

Supplement: Supplementary file 7 — Dataset EV6 [file MSB-15-e8438-s007.zip › feature_plots_bioplex/O00303.pdf]

**O00399**  
**Annotated subunits: 10   Subunits with signal: 4**  
**Max. coeluting subunits: 4   Max. completeness: 0.4**

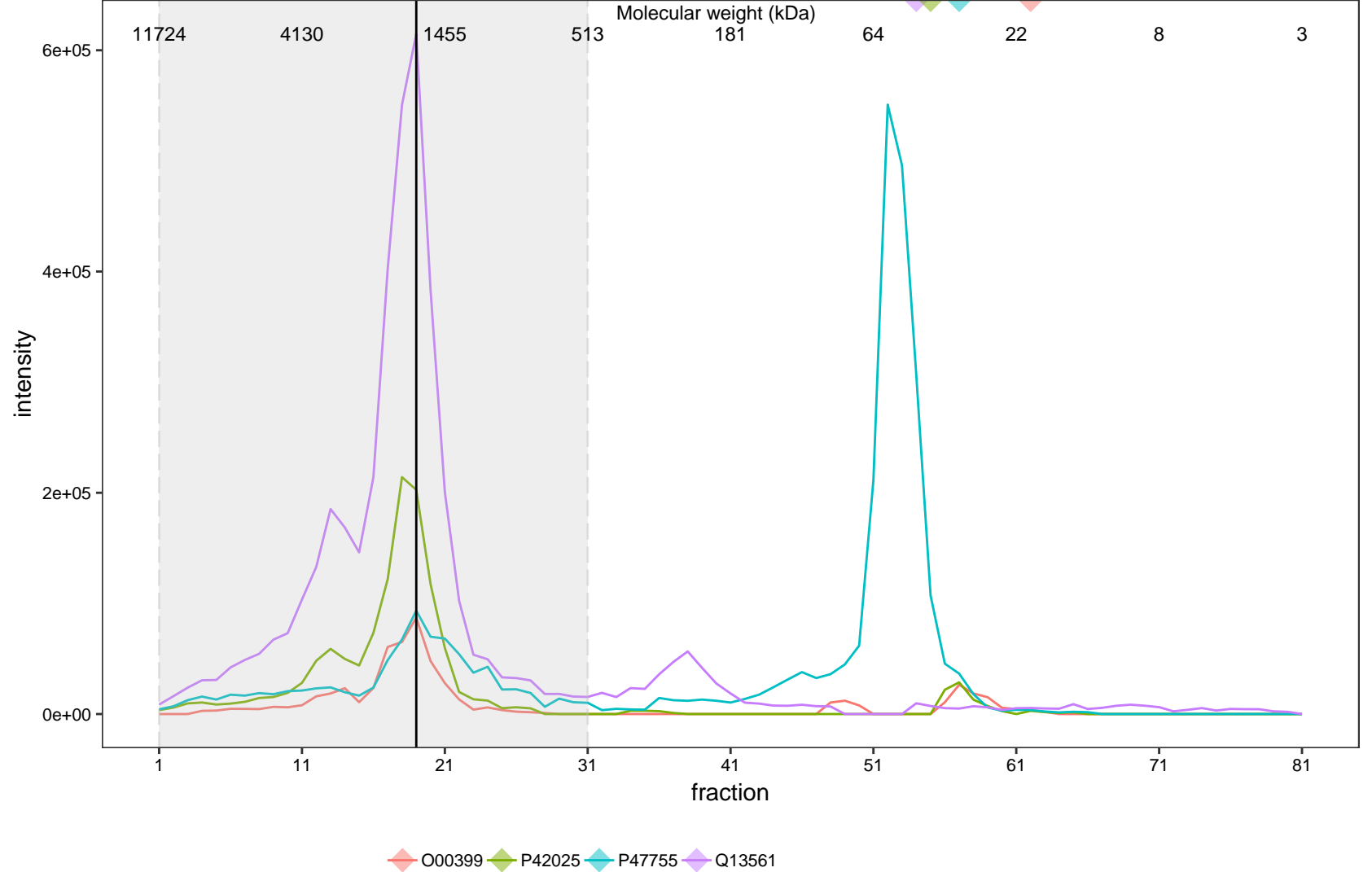

Supplement: Supplementary file 7 — Dataset EV6 [file MSB-15-e8438-s007.zip › feature_plots_bioplex/O00399.pdf]

**O14497**

**Annotated subunits: 6 Subunits with signal: 5**

**Max. coeluting subunits: 5 Max. completeness: 0.83**

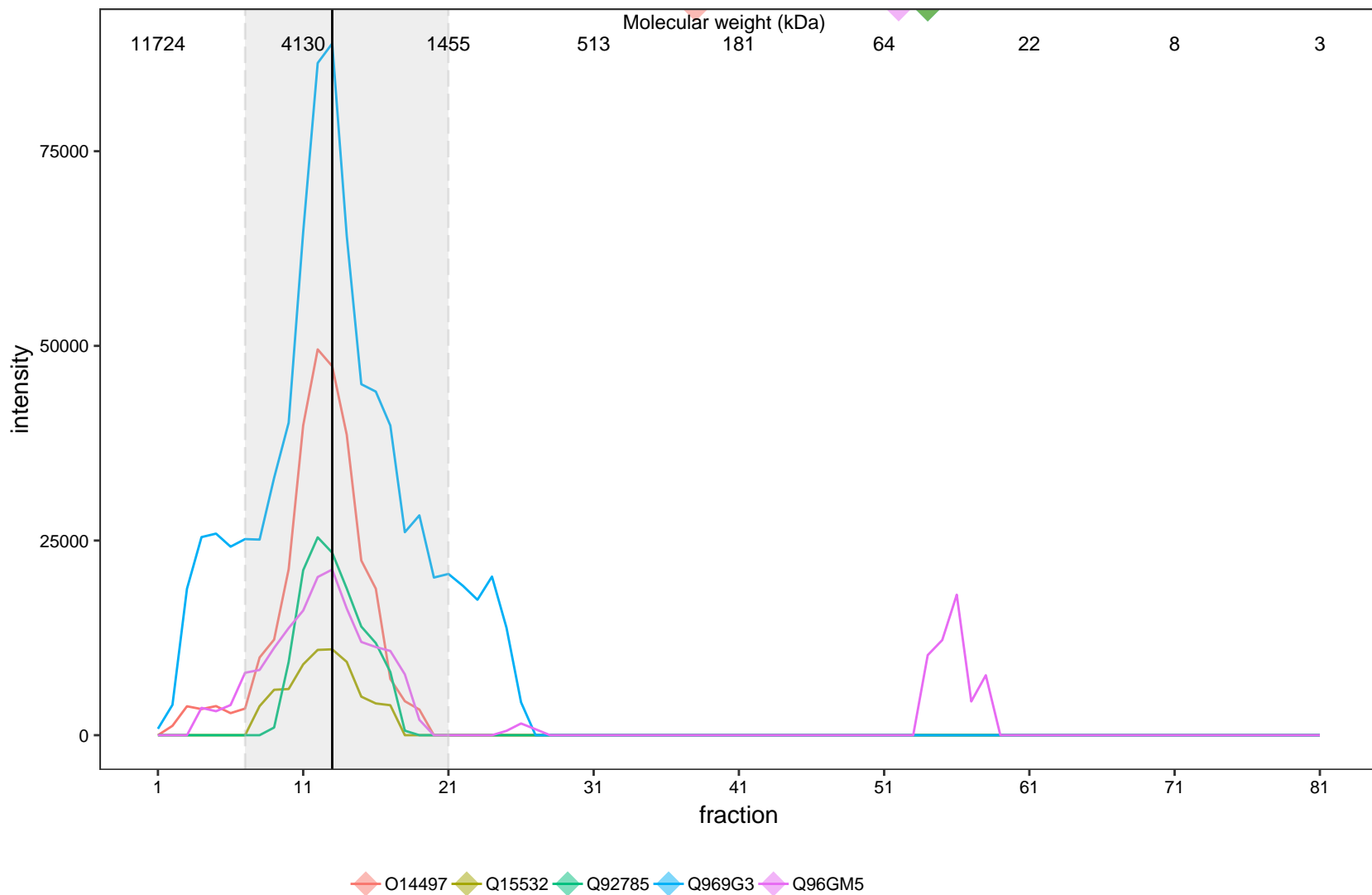

Supplement: Supplementary file 7 — Dataset EV6 [file MSB-15-e8438-s007.zip › feature_plots_bioplex/O14497.pdf]

O14519  
Annotated subunits: 5   Subunits with signal: 3  
Max. coeluting subunits: 3   Max. completeness: 0.6

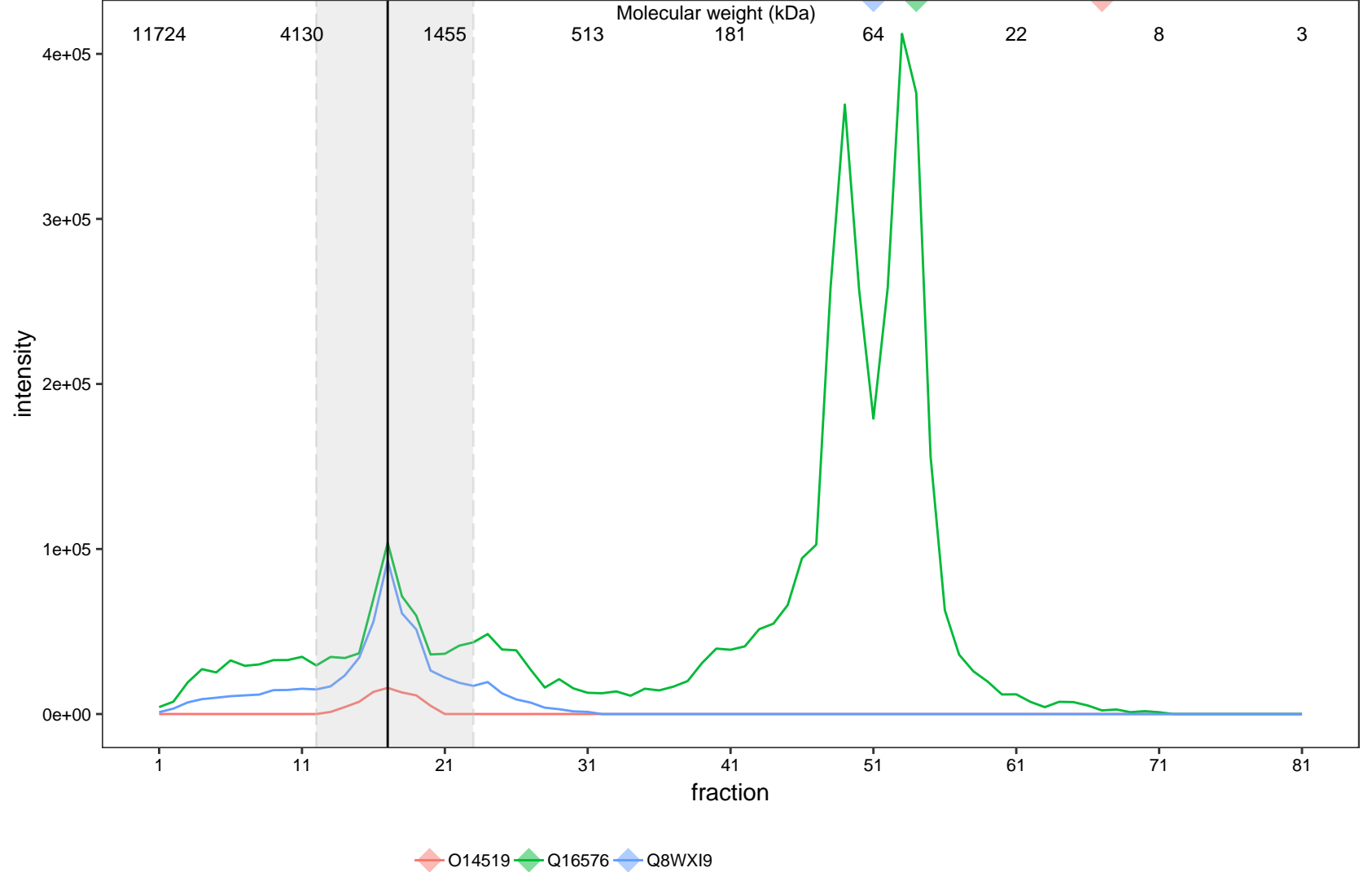

Supplement: Supplementary file 7 — Dataset EV6 [file MSB-15-e8438-s007.zip › feature_plots_bioplex/O14519.pdf]

**O14530**

**Annotated subunits: 14 Subunits with signal: 5**

**Max. coeluting subunits: 4 Max. completeness: 0.29**

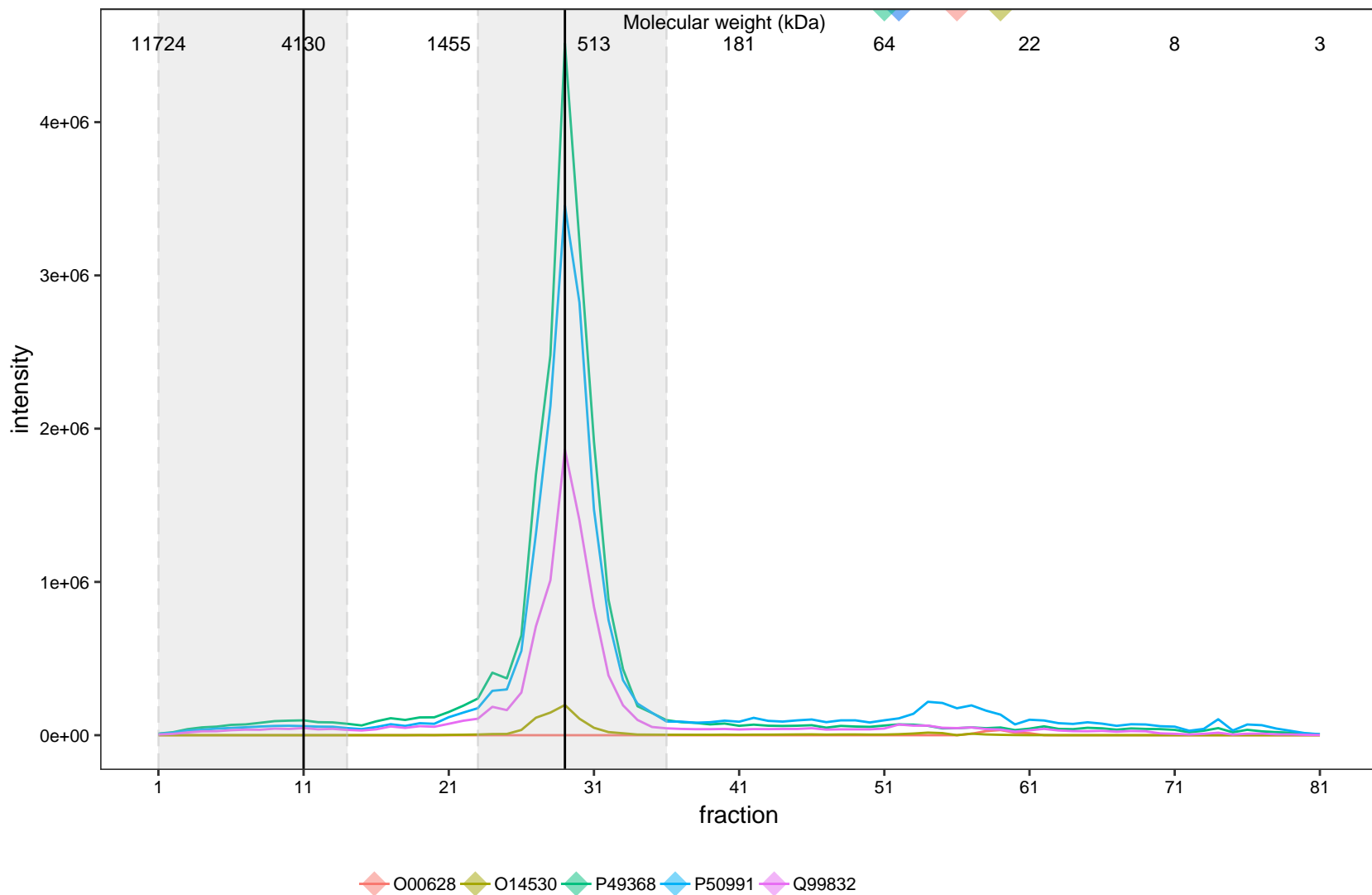

Supplement: Supplementary file 7 — Dataset EV6 [file MSB-15-e8438-s007.zip › feature_plots_bioplex/O14530.pdf]

**O14548**

**Annotated subunits: 2 Subunits with signal: 2**

**Max. coeluting subunits: 2 Max. completeness: 1**

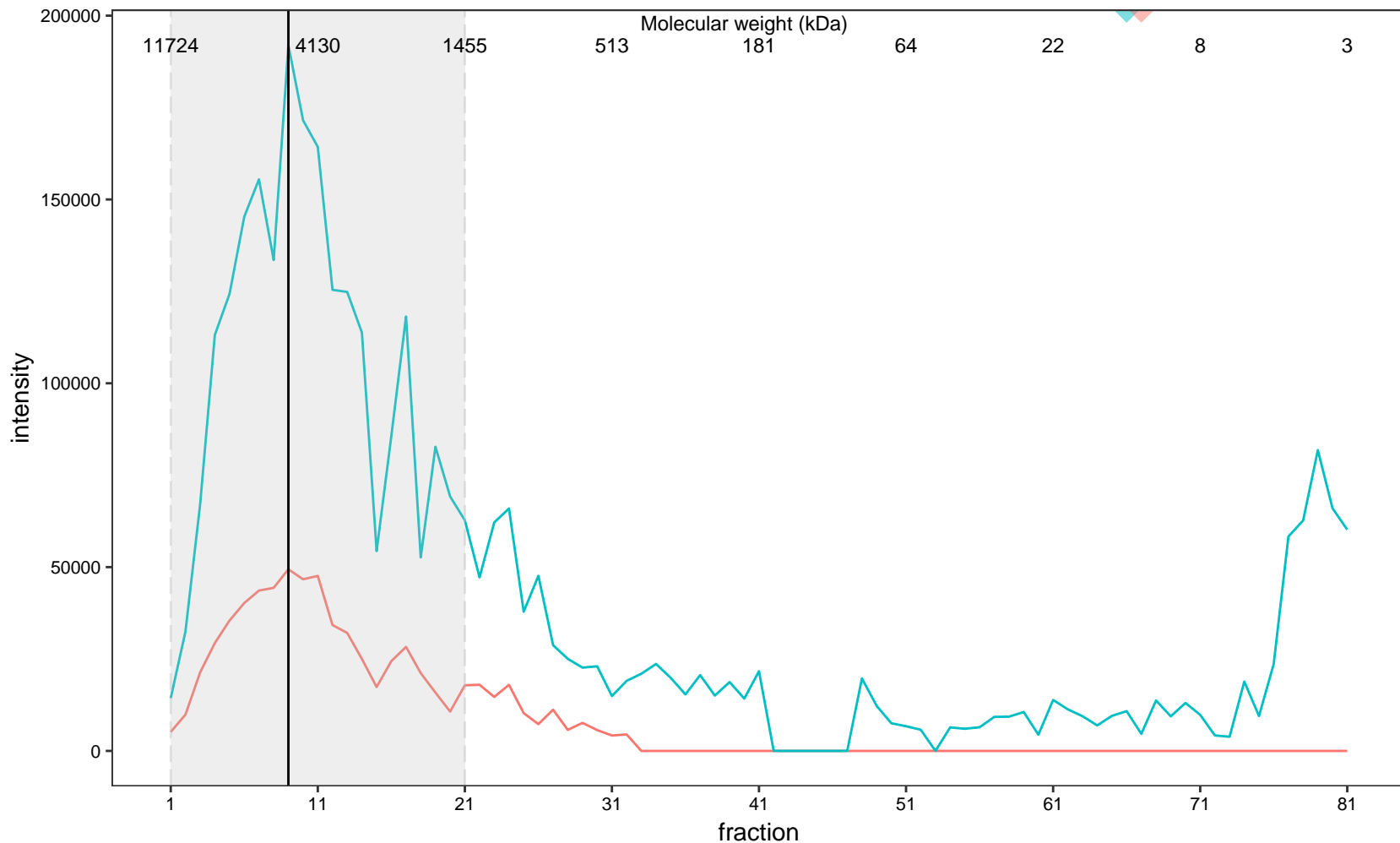

— O14548 — P14927

Supplement: Supplementary file 7 — Dataset EV6 [file MSB-15-e8438-s007.zip › feature_plots_bioplex/O14548.pdf]

**O14653**

**Annotated subunits: 5 Subunits with signal: 4**

**Max. coeluting subunits: 4 Max. completeness: 0.8**

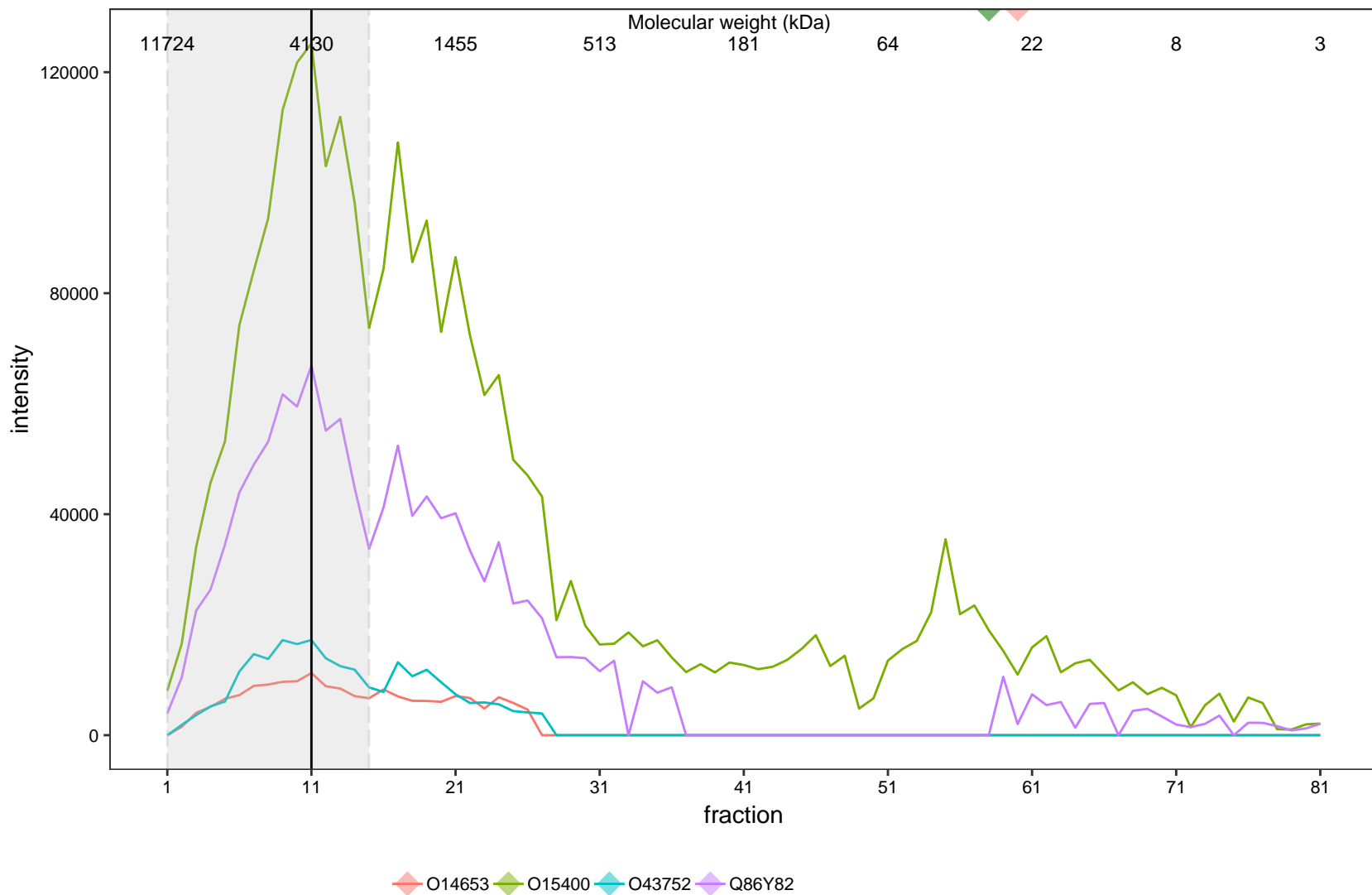

Supplement: Supplementary file 7 — Dataset EV6 [file MSB-15-e8438-s007.zip › feature_plots_bioplex/O14653.pdf]

**O14686**

**Annotated subunits: 2 Subunits with signal: 2**

**Max. coeluting subunits: 2 Max. completeness: 1**

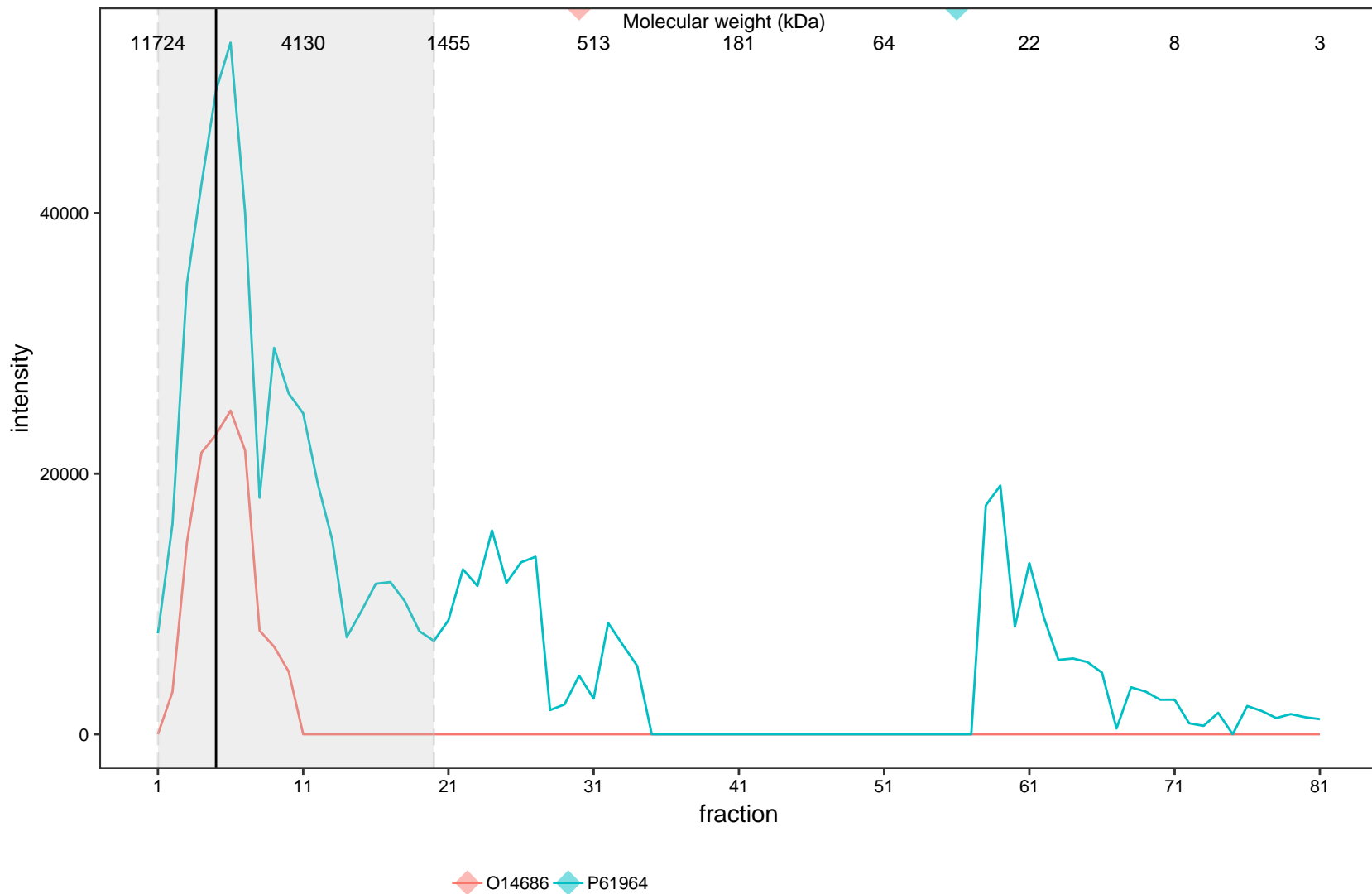

Supplement: Supplementary file 7 — Dataset EV6 [file MSB-15-e8438-s007.zip › feature_plots_bioplex/O14686.pdf]

O14775

Annotated subunits: 9 Subunits with signal: 5

Max. coeluting subunits: 3 Max. completeness: 0.33

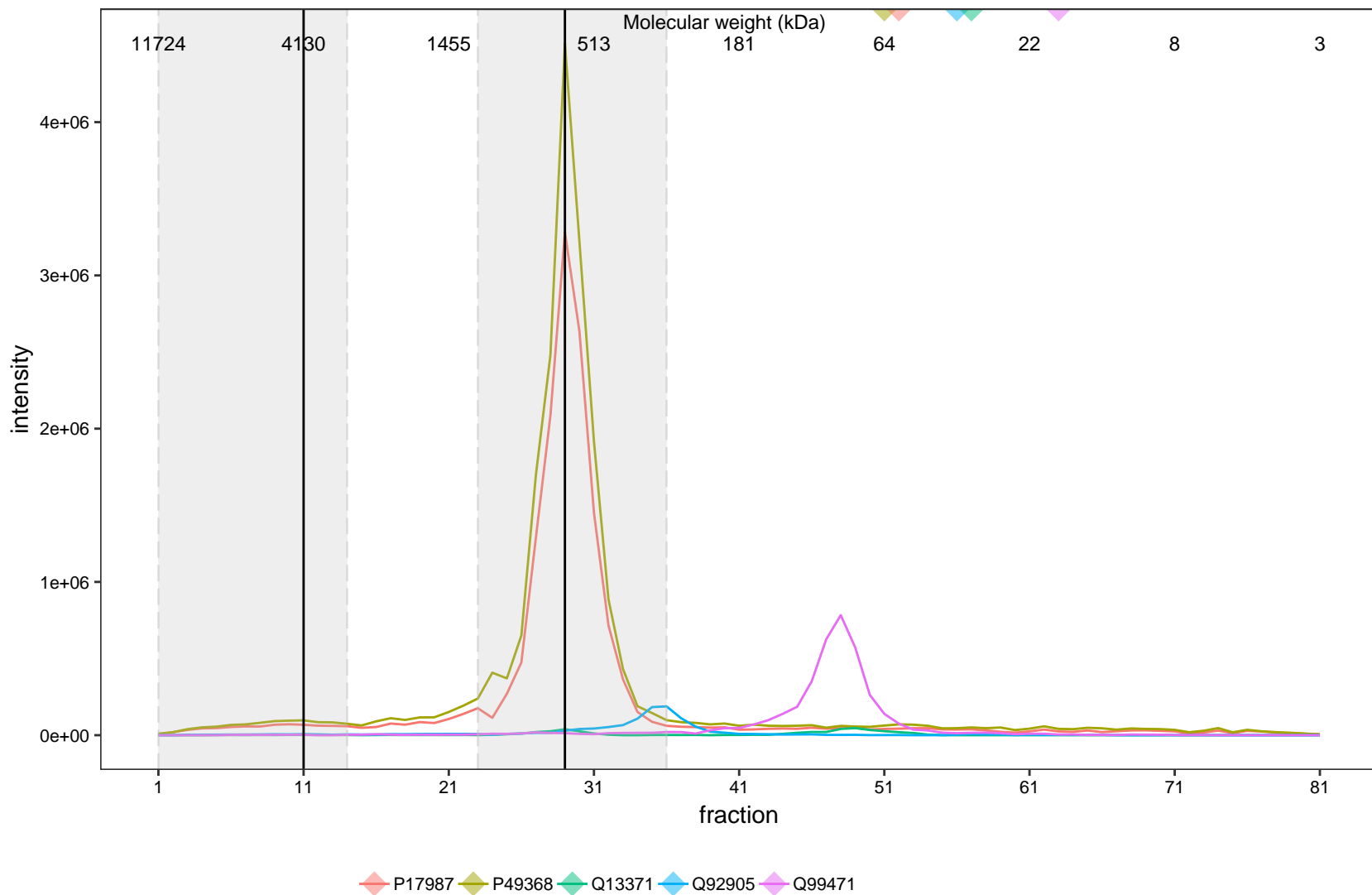

Supplement: Supplementary file 7 — Dataset EV6 [file MSB-15-e8438-s007.zip › feature_plots_bioplex/O14775.pdf]

**O14777**

**Annotated subunits: 9   Subunits with signal: 6**

**Max. coeluting subunits: 3   Max. completeness: 0.33**

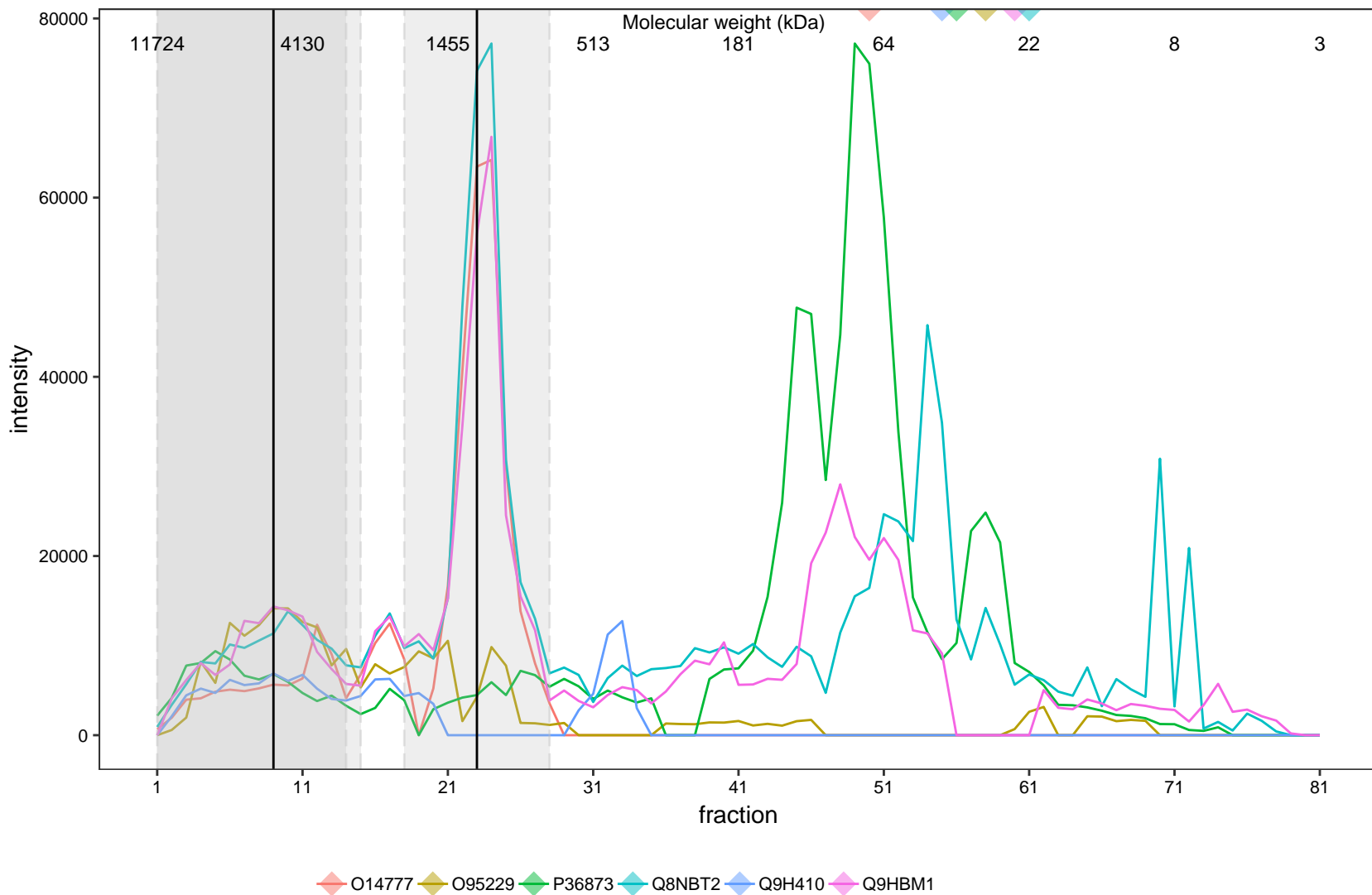

Supplement: Supplementary file 7 — Dataset EV6 [file MSB-15-e8438-s007.zip › feature_plots_bioplex/O14777.pdf]

**O14818**

**Annotated subunits: 5 Subunits with signal: 4**

**Max. coeluting subunits: 4 Max. completeness: 0.8**

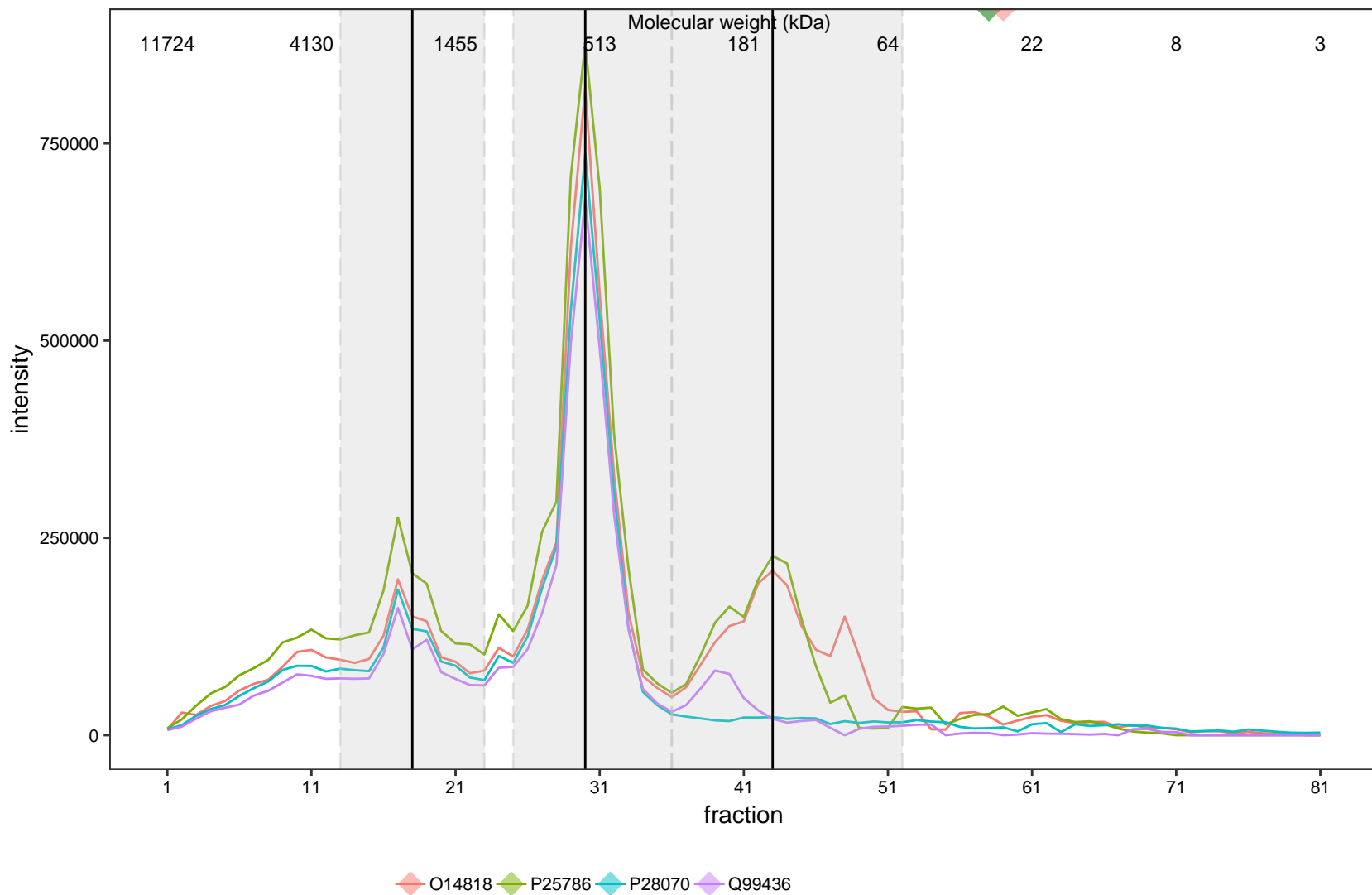

Supplement: Supplementary file 7 — Dataset EV6 [file MSB-15-e8438-s007.zip › feature_plots_bioplex/O14818.pdf]

**O14924**

**Annotated subunits: 3 Subunits with signal: 2**

**Max. coeluting subunits: 2 Max. completeness: 0.67**

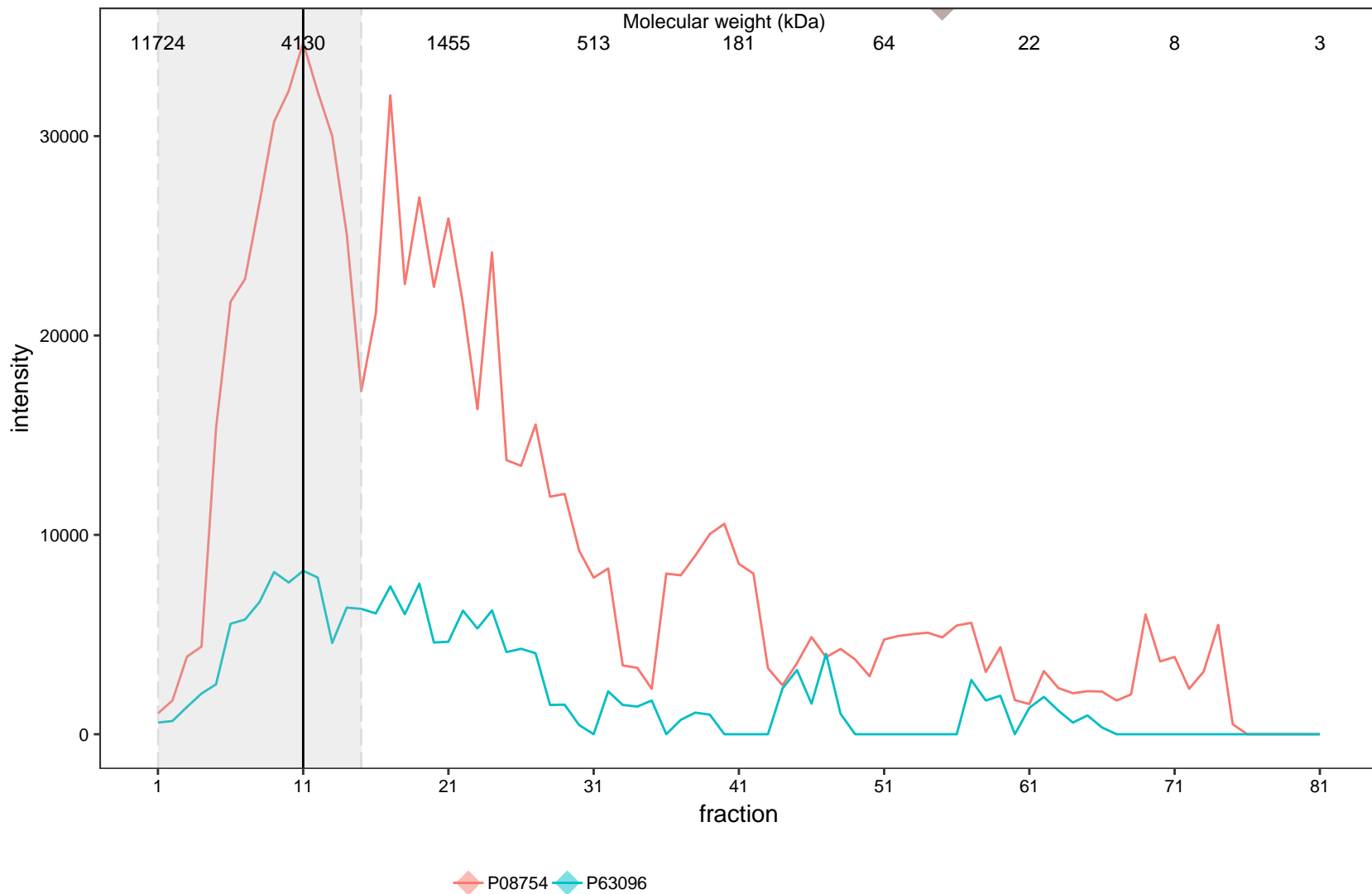

Supplement: Supplementary file 7 — Dataset EV6 [file MSB-15-e8438-s007.zip › feature_plots_bioplex/O14924.pdf]

**O14939**

**Annotated subunits: 8 Subunits with signal: 4**

**Max. coeluting subunits: 4 Max. completeness: 0.5**

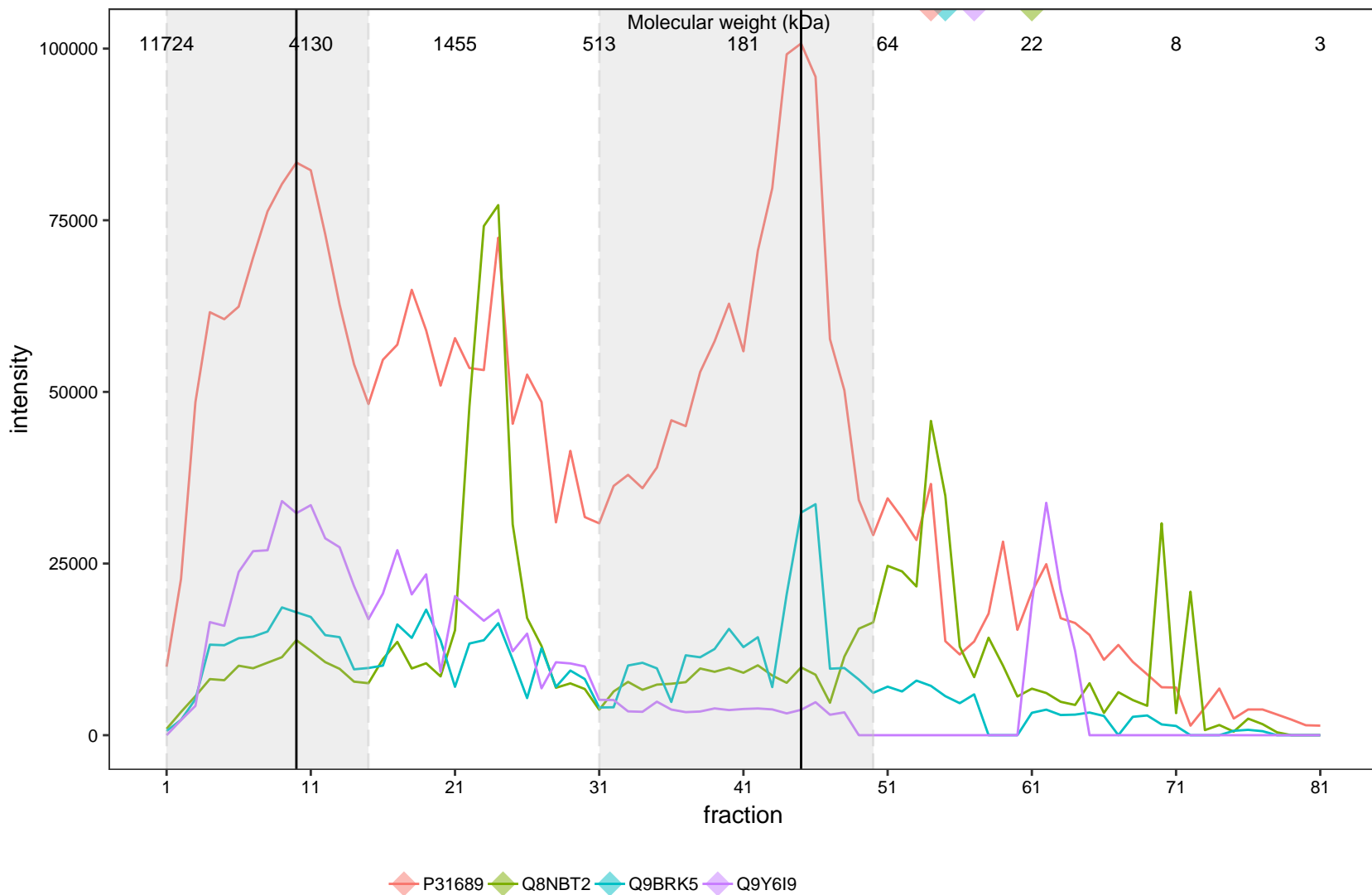

Supplement: Supplementary file 7 — Dataset EV6 [file MSB-15-e8438-s007.zip › feature_plots_bioplex/O14939.pdf]

O14949  
Annotated subunits: 6   Subunits with signal: 5  
Max. coeluting subunits: 3   Max. completeness: 0.5

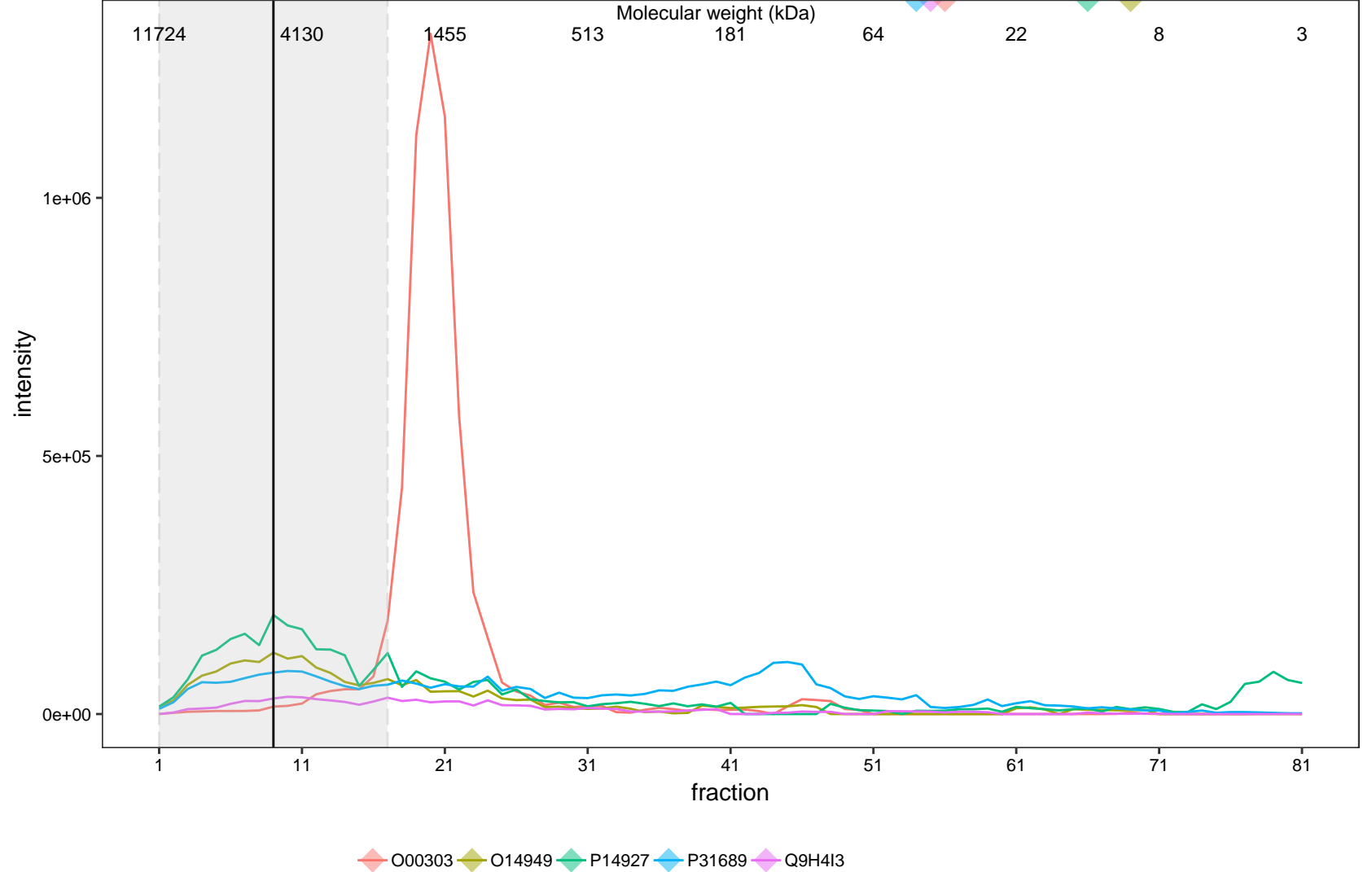

Supplement: Supplementary file 7 — Dataset EV6 [file MSB-15-e8438-s007.zip › feature_plots_bioplex/O14949.pdf]

**O14972**

**Annotated subunits: 3 Subunits with signal: 3**

**Max. coeluting subunits: 3 Max. completeness: 1**

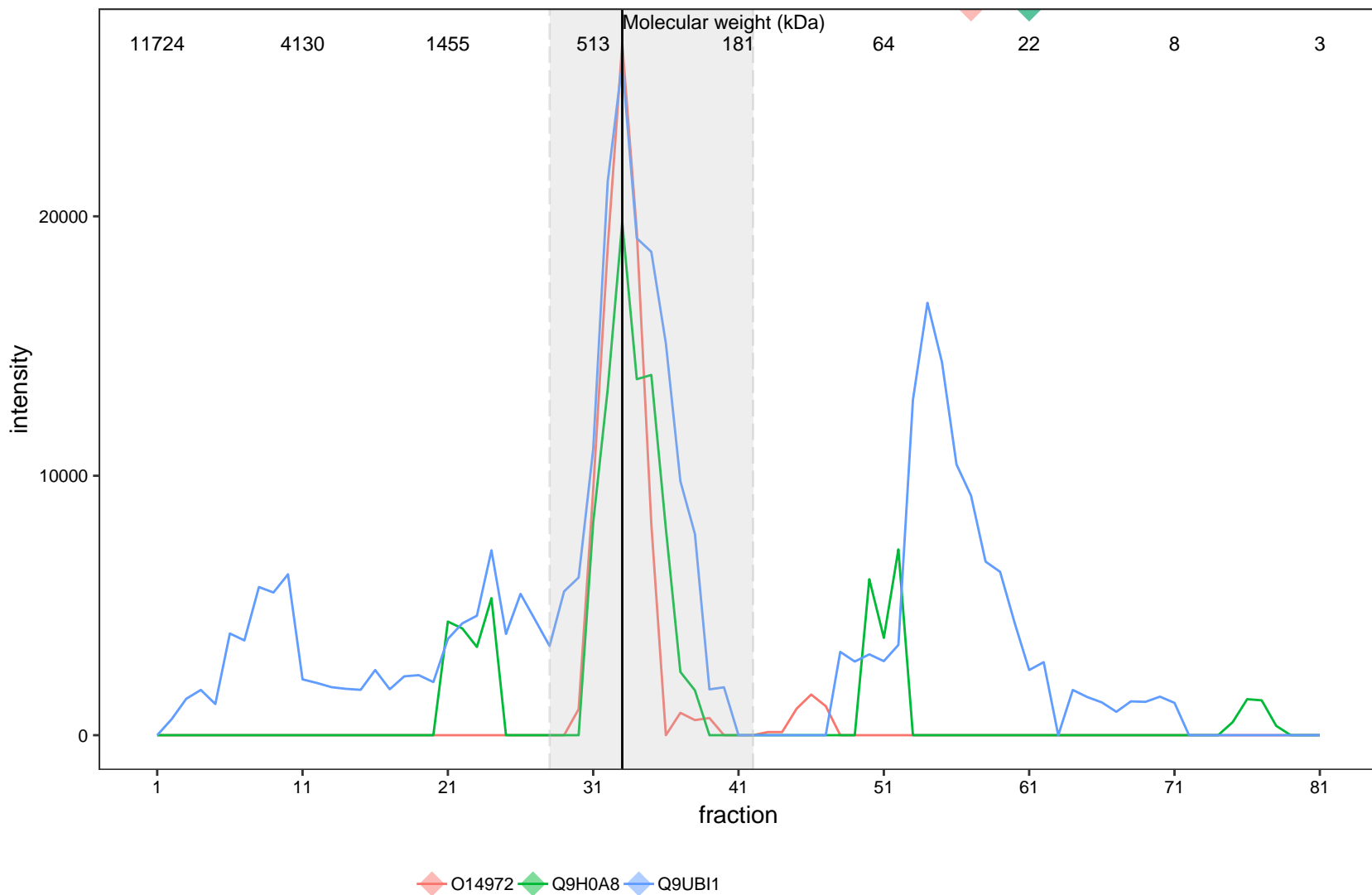

Supplement: Supplementary file 7 — Dataset EV6 [file MSB-15-e8438-s007.zip › feature_plots_bioplex/O14972.pdf]

O14977  
Annotated subunits: 10   Subunits with signal: 7  
Max. coeluting subunits: 5   Max. completeness: 0.5

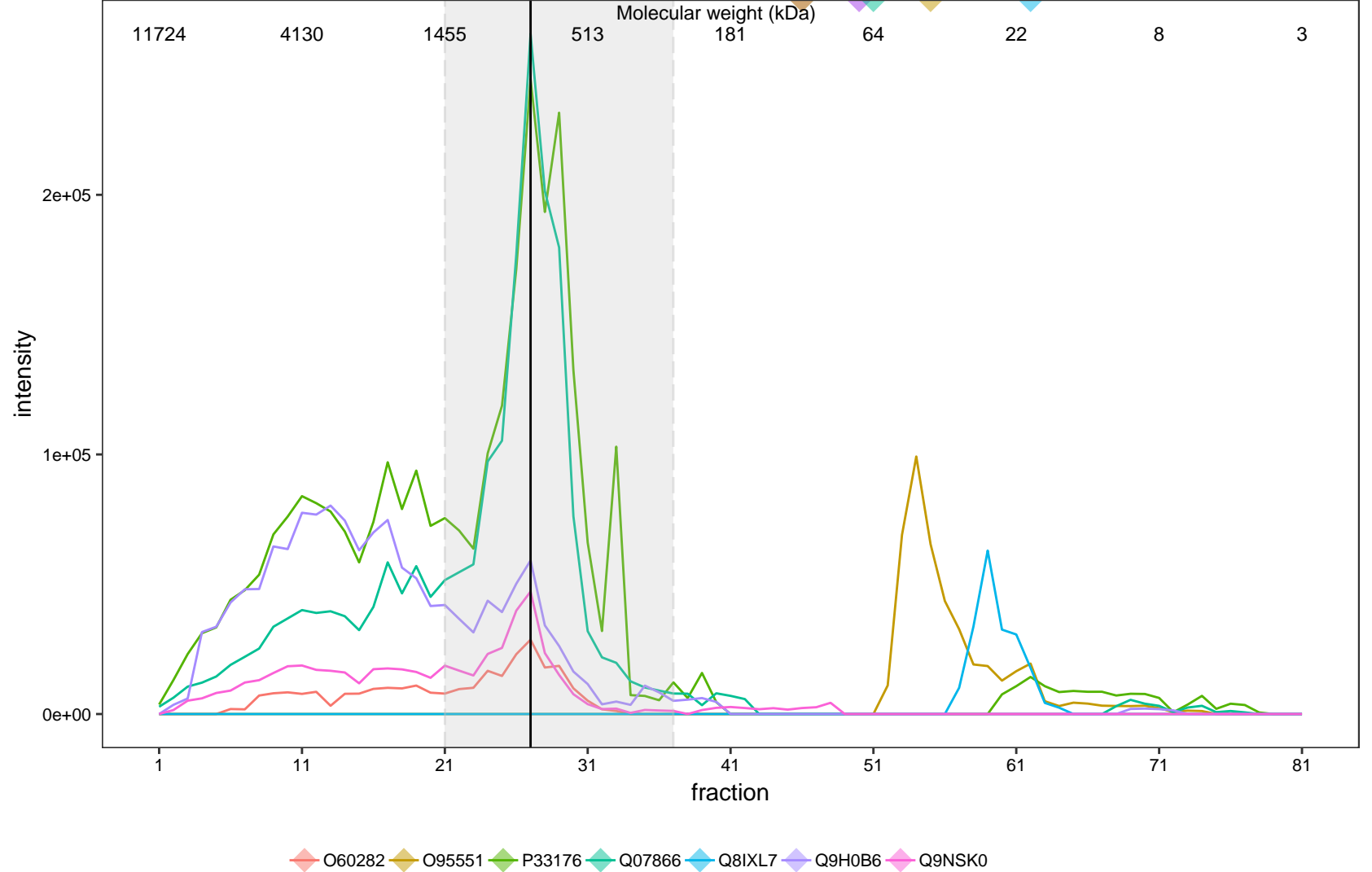

Supplement: Supplementary file 7 — Dataset EV6 [file MSB-15-e8438-s007.zip › feature_plots_bioplex/O14977.pdf]

O15042  
Annotated subunits: 6   Subunits with signal: 4  
Max. coeluting subunits: 2   Max. completeness: 0.33

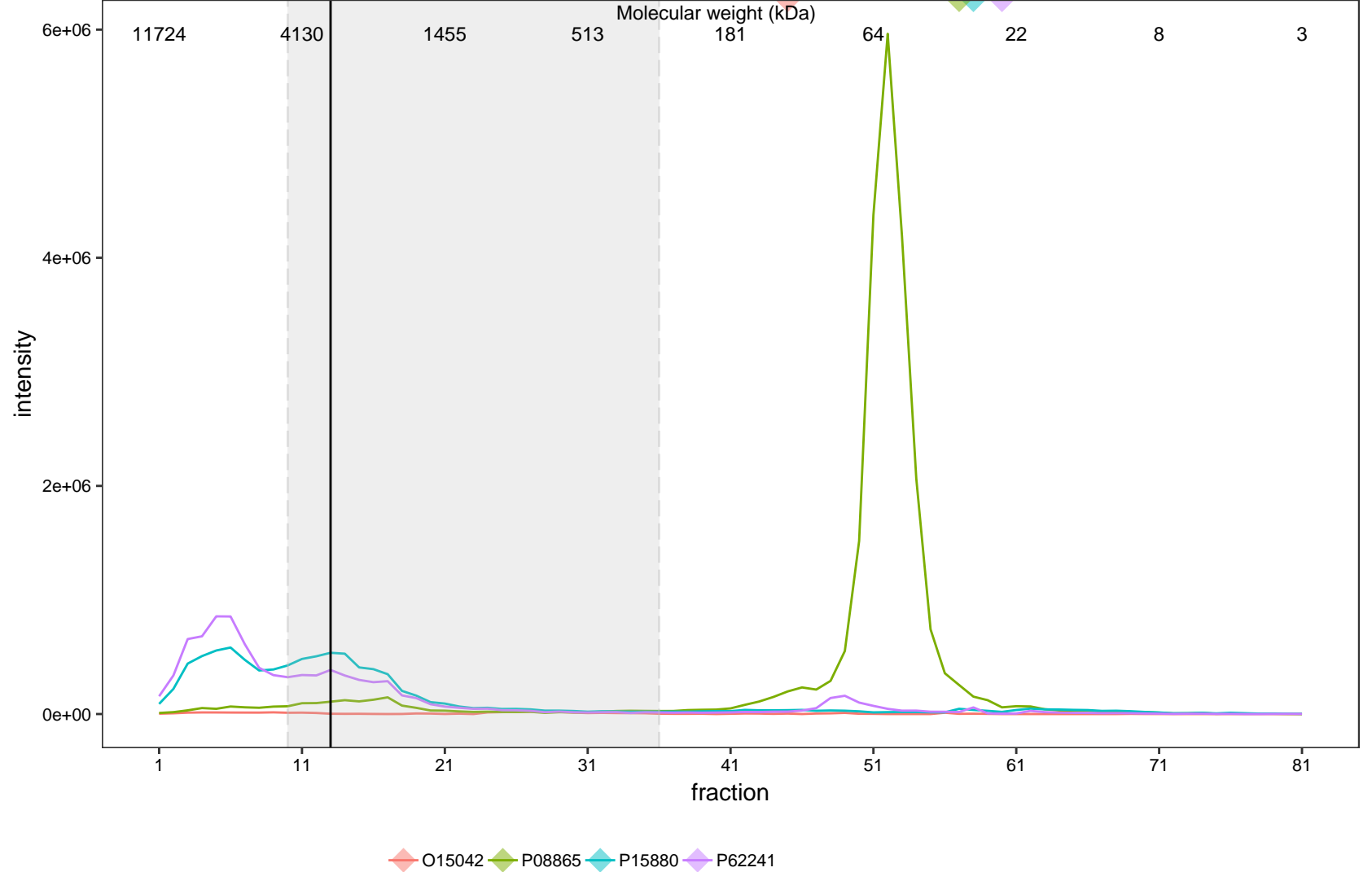

Supplement: Supplementary file 7 — Dataset EV6 [file MSB-15-e8438-s007.zip › feature_plots_bioplex/O15042.pdf]

**O15084**

**Annotated subunits: 9 Subunits with signal: 4**

**Max. coeluting subunits: 2 Max. completeness: 0.22**

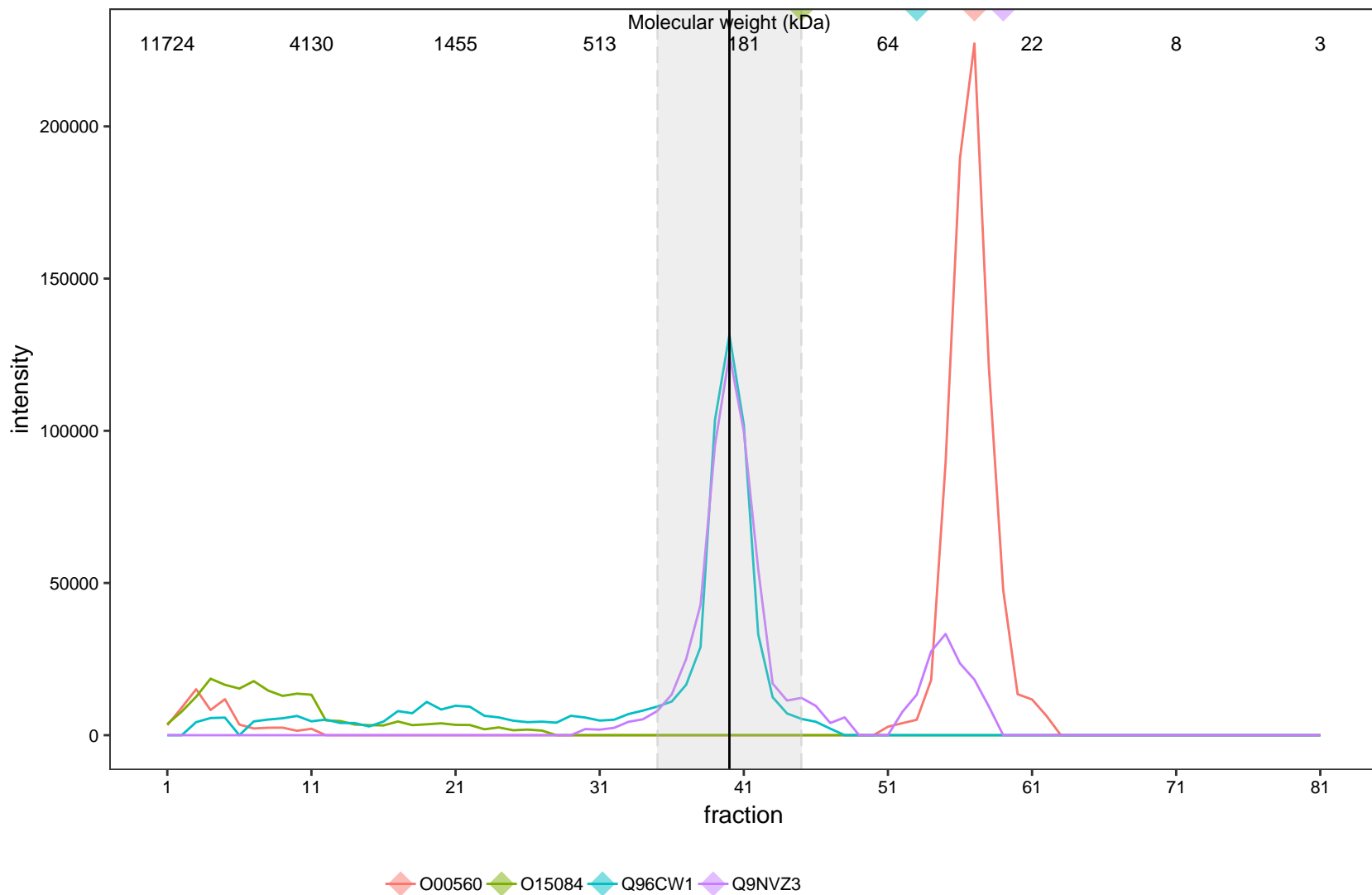

Supplement: Supplementary file 7 — Dataset EV6 [file MSB-15-e8438-s007.zip › feature_plots_bioplex/O15084.pdf]

**O15118**  
**Annotated subunits: 3   Subunits with signal: 2**  
**Max. coeluting subunits: 2   Max. completeness: 0.67**

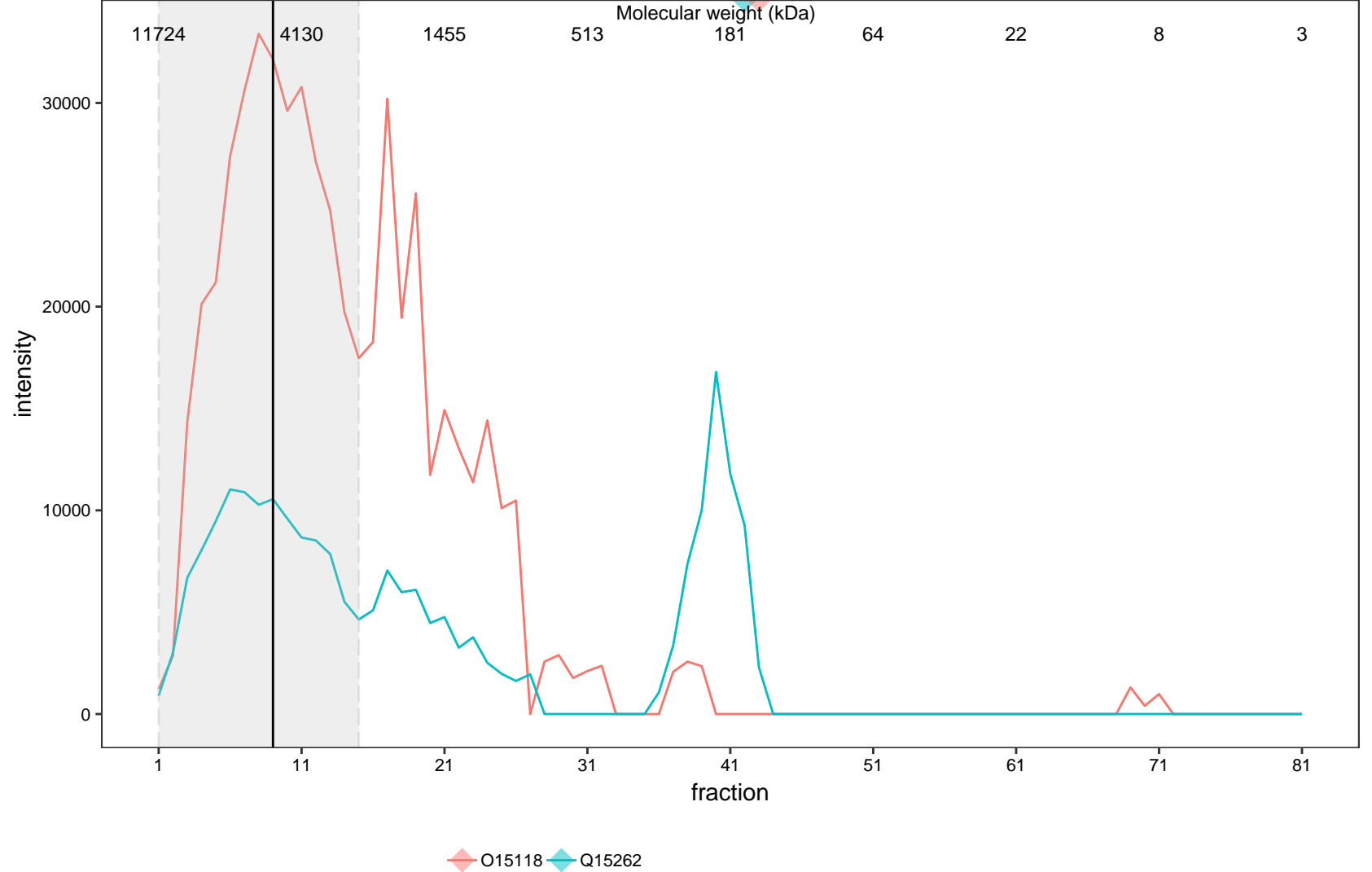

Supplement: Supplementary file 7 — Dataset EV6 [file MSB-15-e8438-s007.zip › feature_plots_bioplex/O15118.pdf]

O15126  
Annotated subunits: 10   Subunits with signal: 4  
Max. coeluting subunits: 4   Max. completeness: 0.4

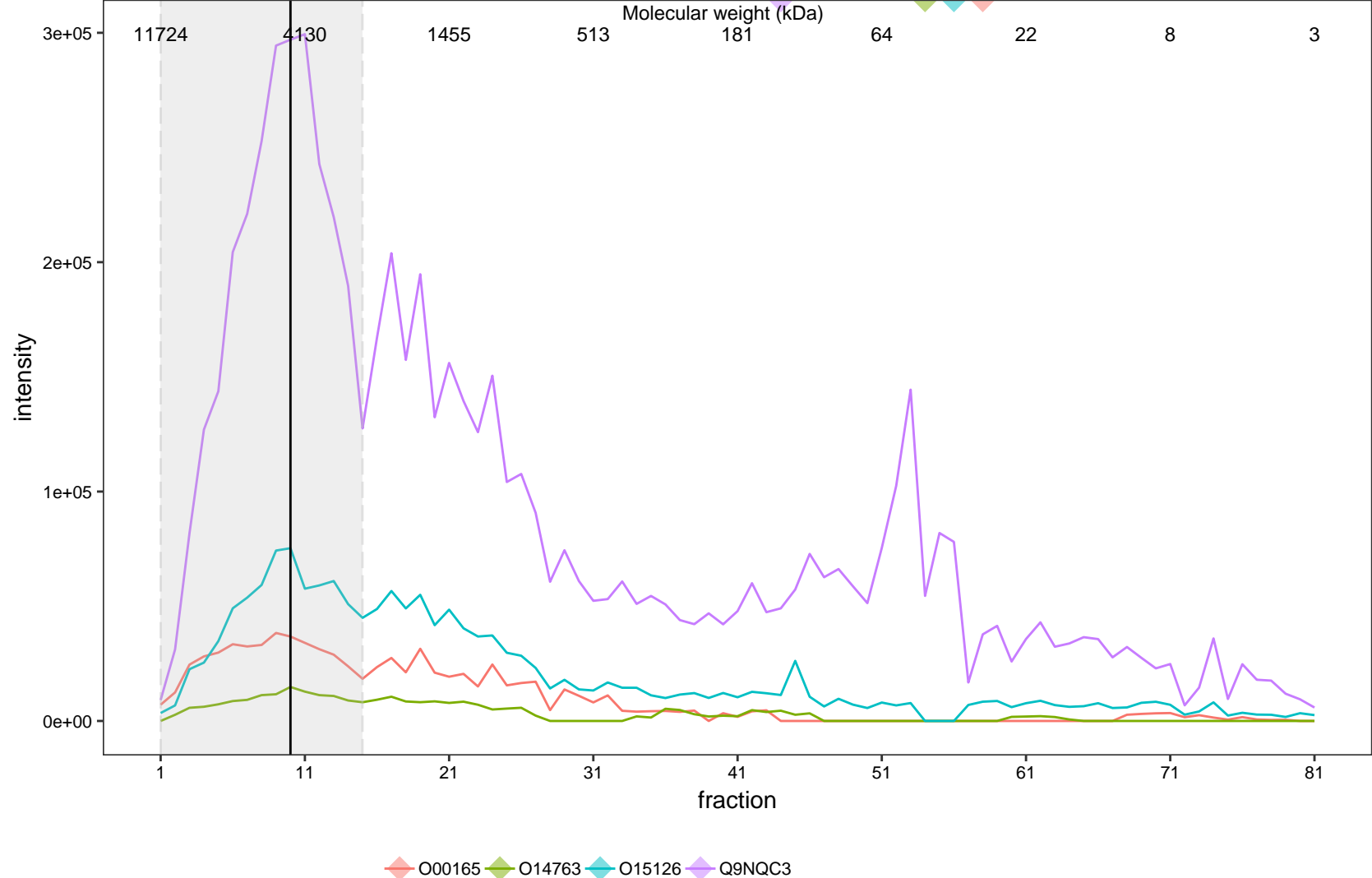

Supplement: Supplementary file 7 — Dataset EV6 [file MSB-15-e8438-s007.zip › feature_plots_bioplex/O15126.pdf]

O15127  
Annotated subunits: 9   Subunits with signal: 2  
Max. coeluting subunits: 2   Max. completeness: 0.22

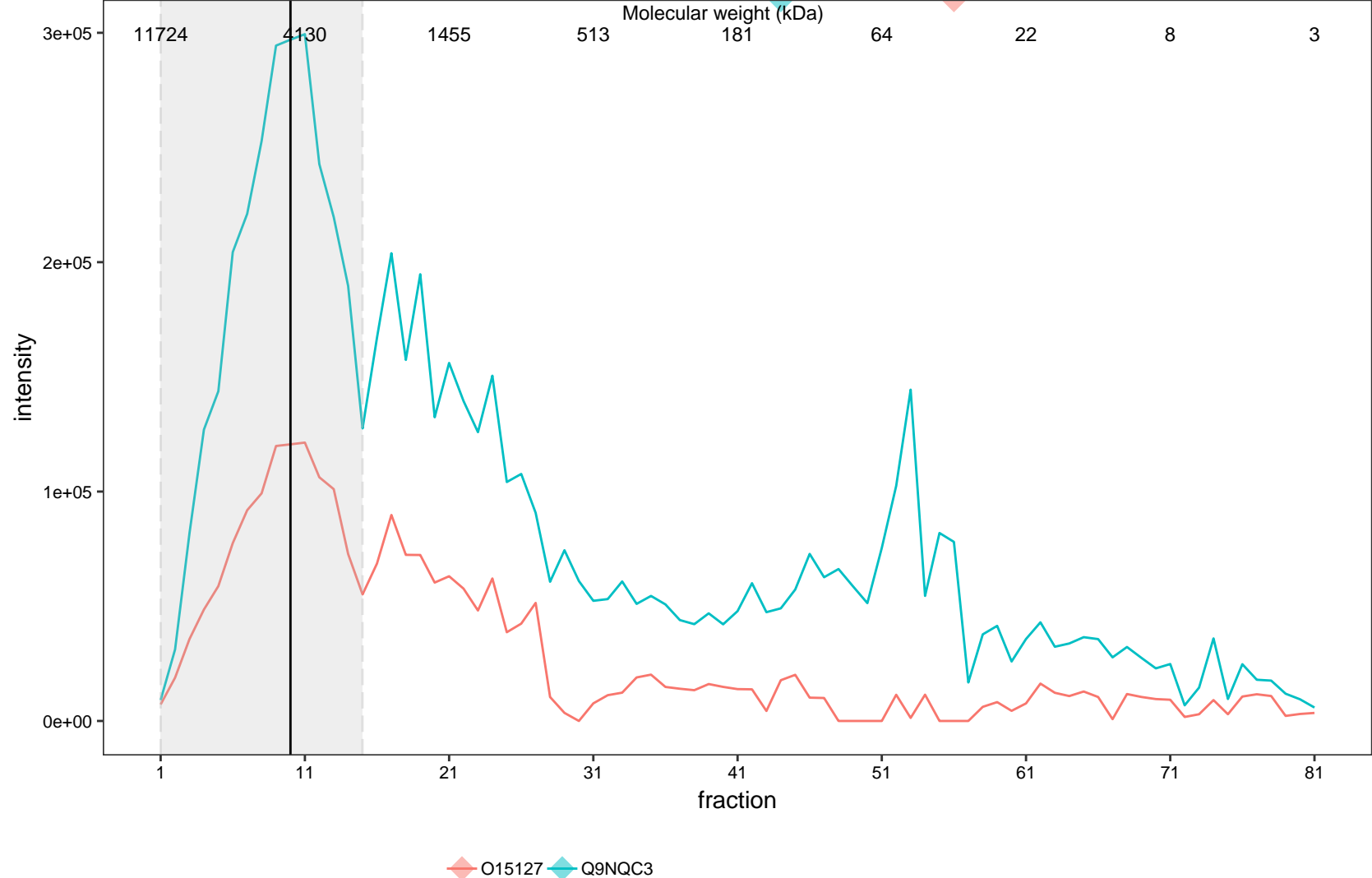

Supplement: Supplementary file 7 — Dataset EV6 [file MSB-15-e8438-s007.zip › feature_plots_bioplex/O15127.pdf]

O15143

Annotated subunits: 11 Subunits with signal: 9

Max. coeluting subunits: 8 Max. completeness: 0.73

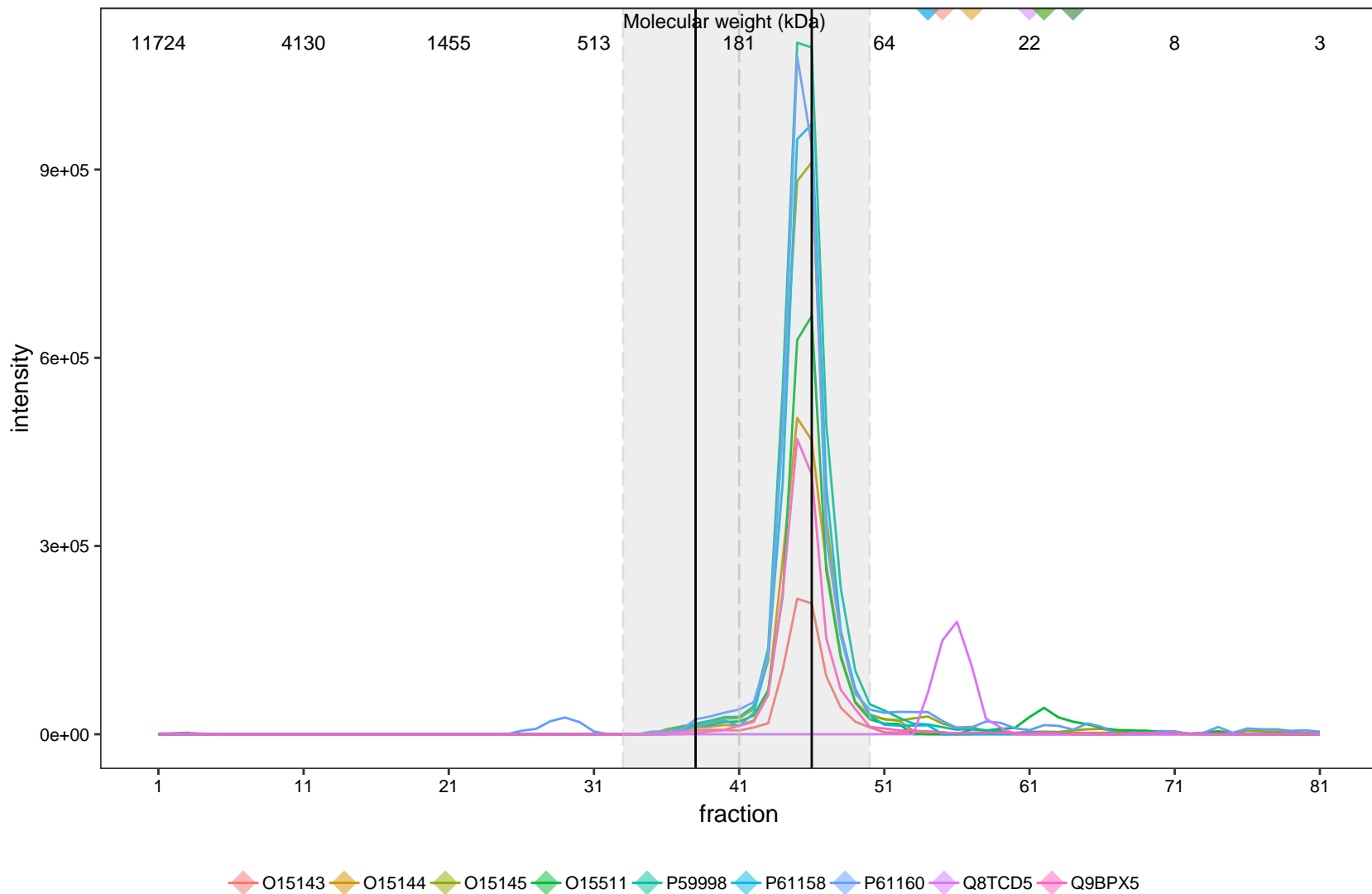

Supplement: Supplementary file 7 — Dataset EV6 [file MSB-15-e8438-s007.zip › feature_plots_bioplex/O15143.pdf]

**O15144**

**Annotated subunits: 4 Subunits with signal: 4**

**Max. coeluting subunits: 4 Max. completeness: 1**

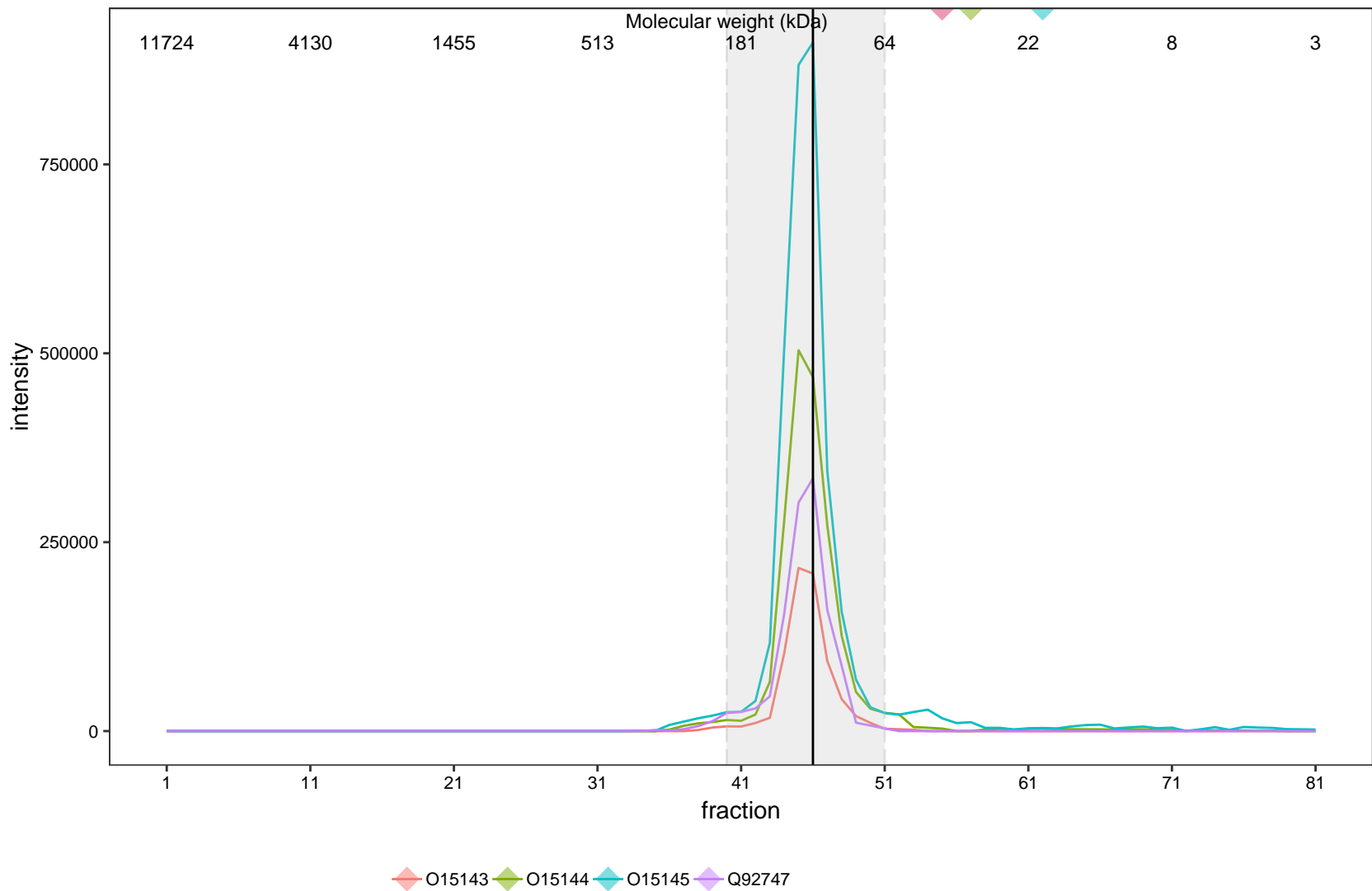

Supplement: Supplementary file 7 — Dataset EV6 [file MSB-15-e8438-s007.zip › feature_plots_bioplex/O15144.pdf]

**O15318**

**Annotated subunits: 2 Subunits with signal: 2**

**Max. coeluting subunits: 2 Max. completeness: 1**

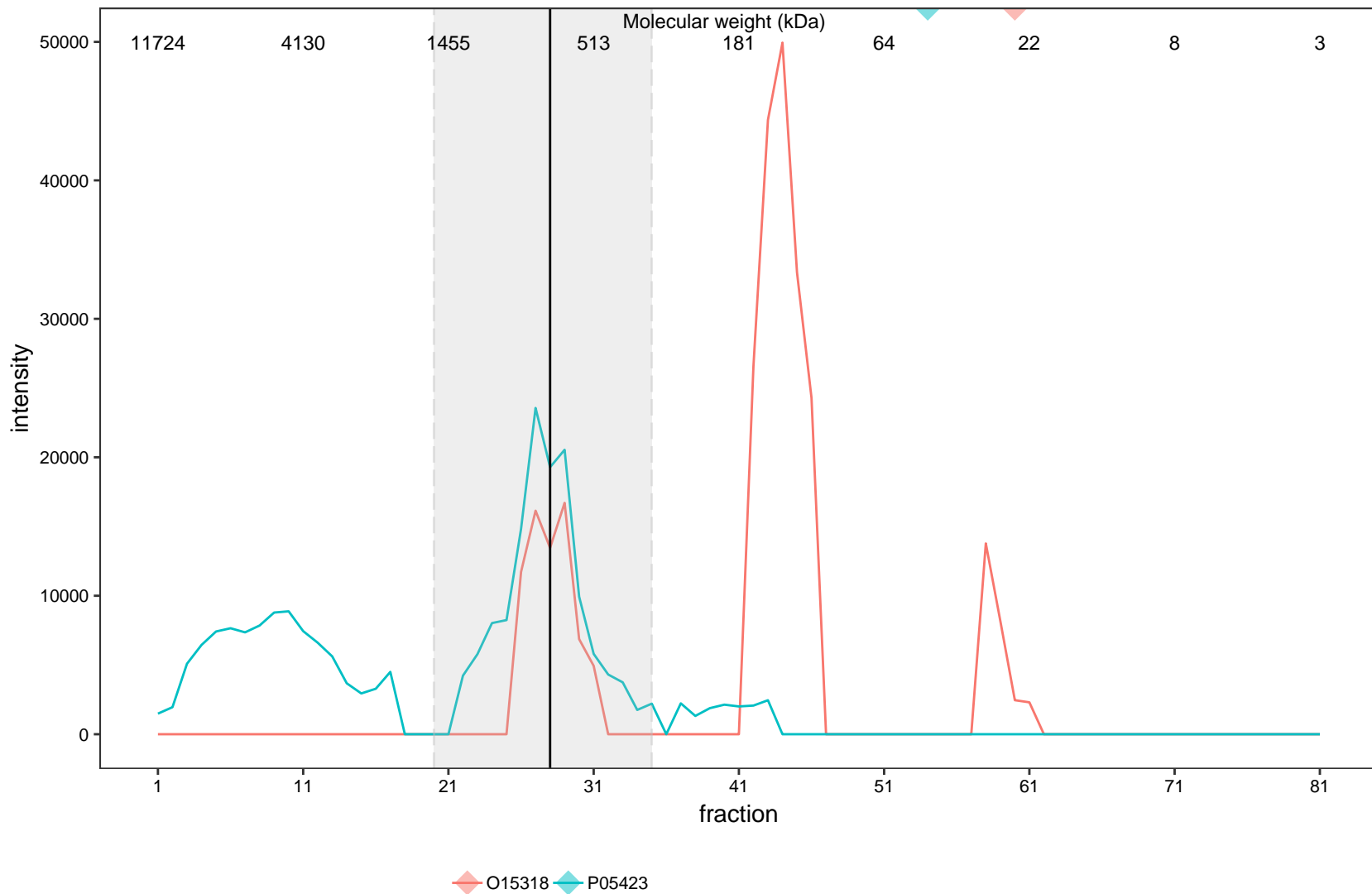

Supplement: Supplementary file 7 — Dataset EV6 [file MSB-15-e8438-s007.zip › feature_plots_bioplex/O15318.pdf]

O15371

Annotated subunits: 6 Subunits with signal: 6

Max. coeluting subunits: 6 Max. completeness: 1

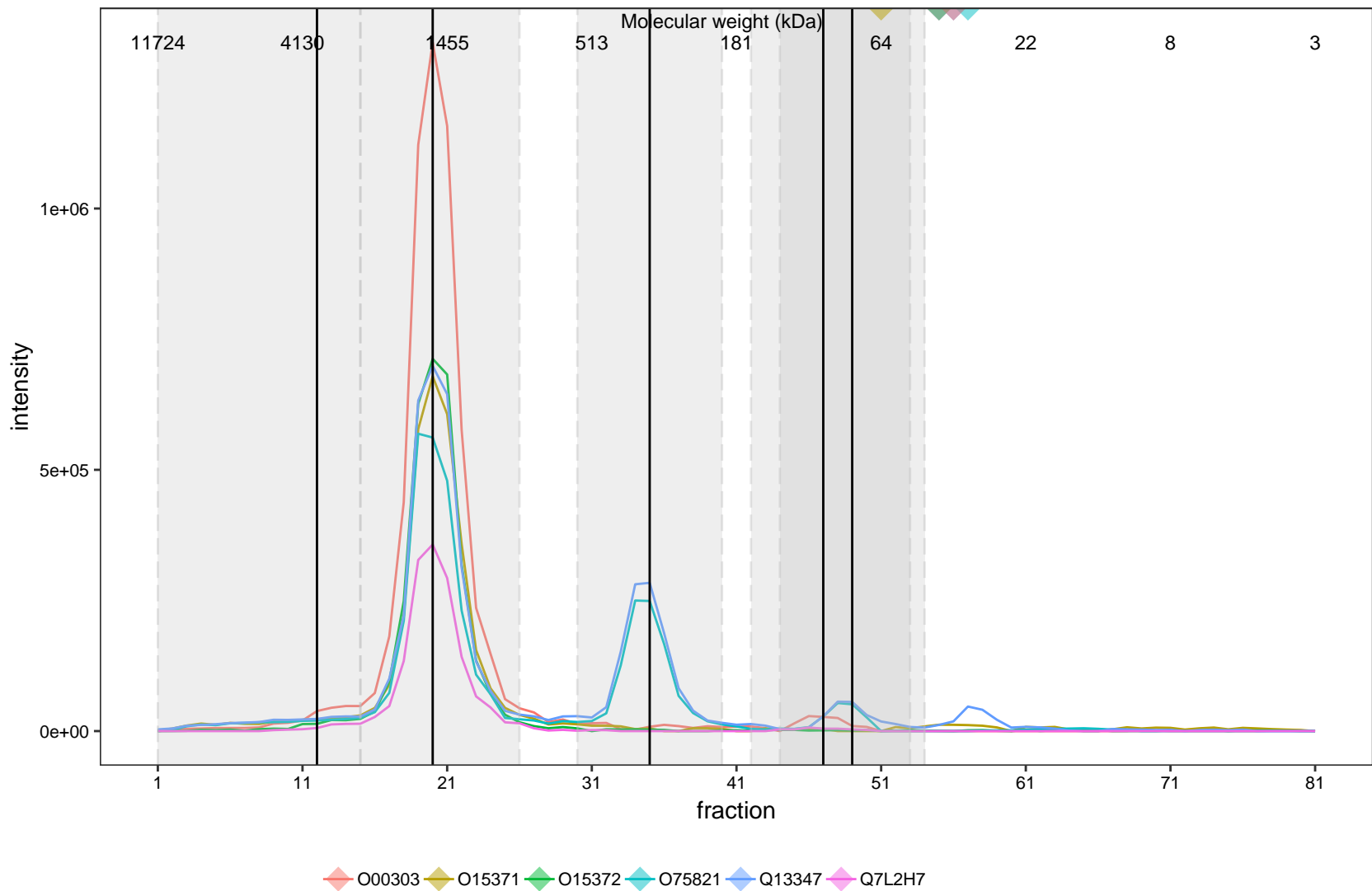

Supplement: Supplementary file 7 — Dataset EV6 [file MSB-15-e8438-s007.zip › feature_plots_bioplex/O15371.pdf]

O15442

Annotated subunits: 22 Subunits with signal: 12

Max. coeluting subunits: 4 Max. completeness: 0.18

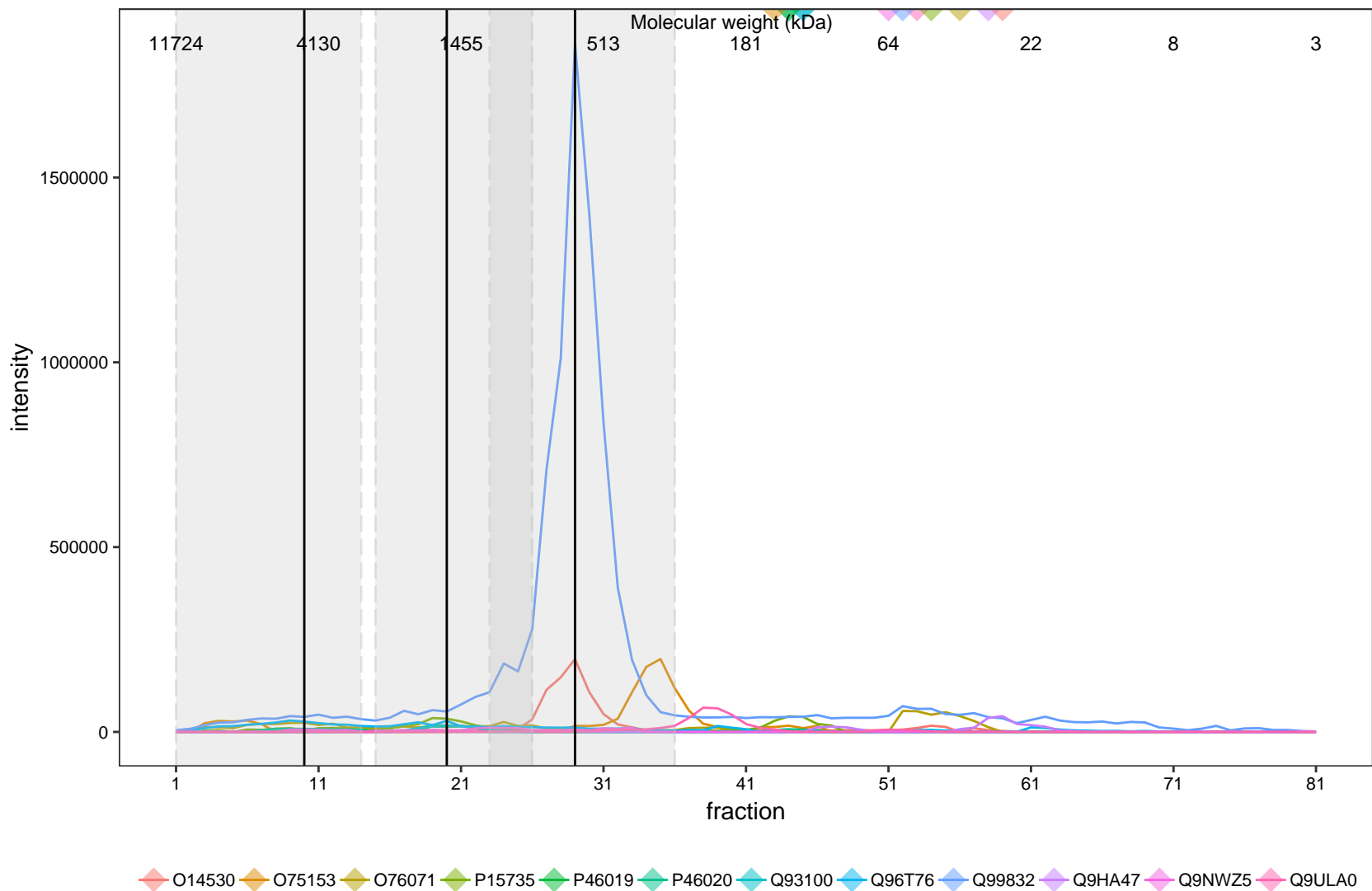

Supplement: Supplementary file 7 — Dataset EV6 [file MSB-15-e8438-s007.zip › feature_plots_bioplex/O15442.pdf]

**O15460**

**Annotated subunits: 4   Subunits with signal: 3**

**Max. coeluting subunits: 2   Max. completeness: 0.5**

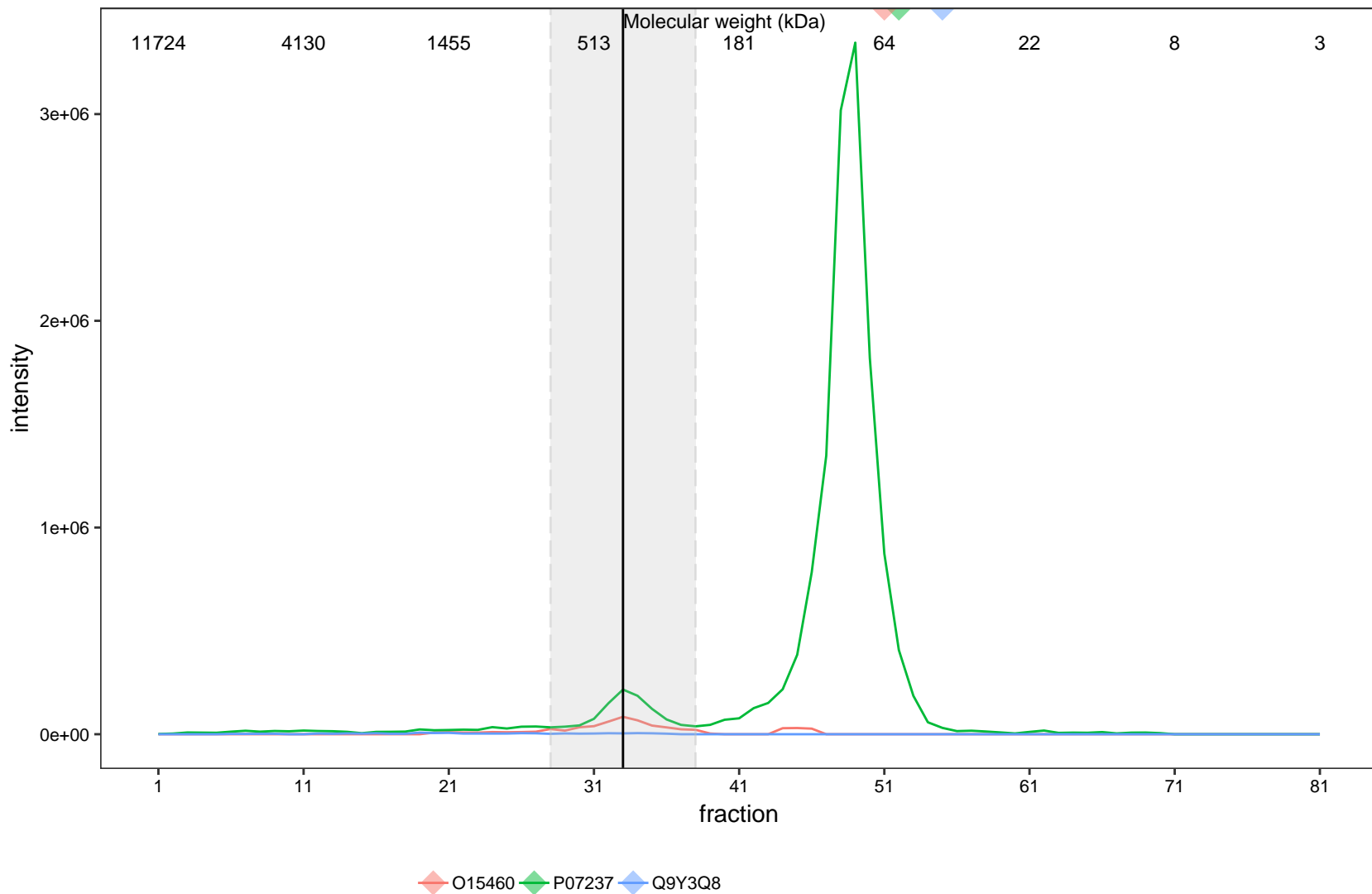

Supplement: Supplementary file 7 — Dataset EV6 [file MSB-15-e8438-s007.zip › feature_plots_bioplex/O15460.pdf]

**O15498**

**Annotated subunits: 9 Subunits with signal: 6**

**Max. coeluting subunits: 2 Max. completeness: 0.22**

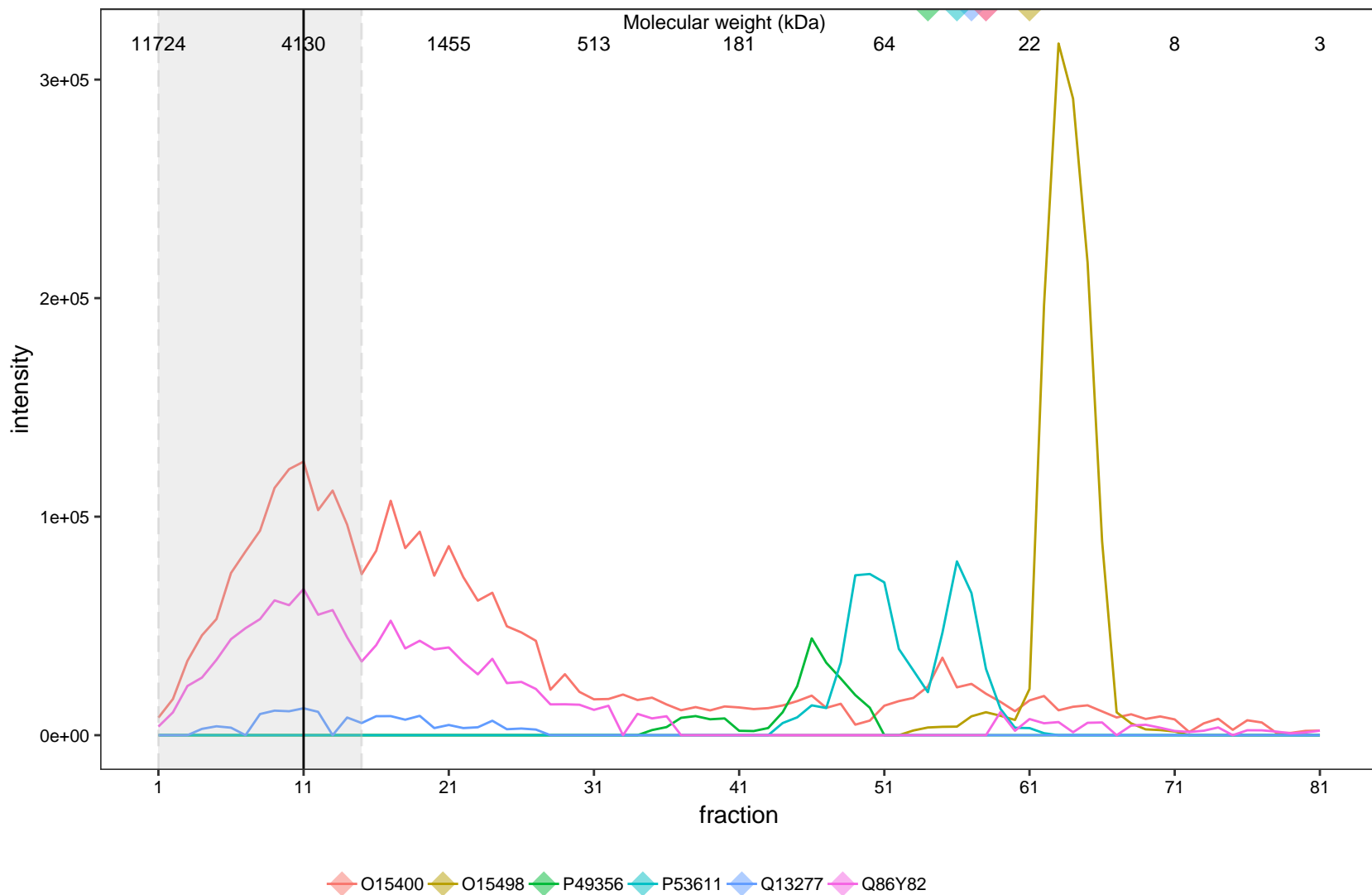

Supplement: Supplementary file 7 — Dataset EV6 [file MSB-15-e8438-s007.zip › feature_plots_bioplex/O15498.pdf]

O15511

Annotated subunits: 4 Subunits with signal: 4

Max. coeluting subunits: 4 Max. completeness: 1

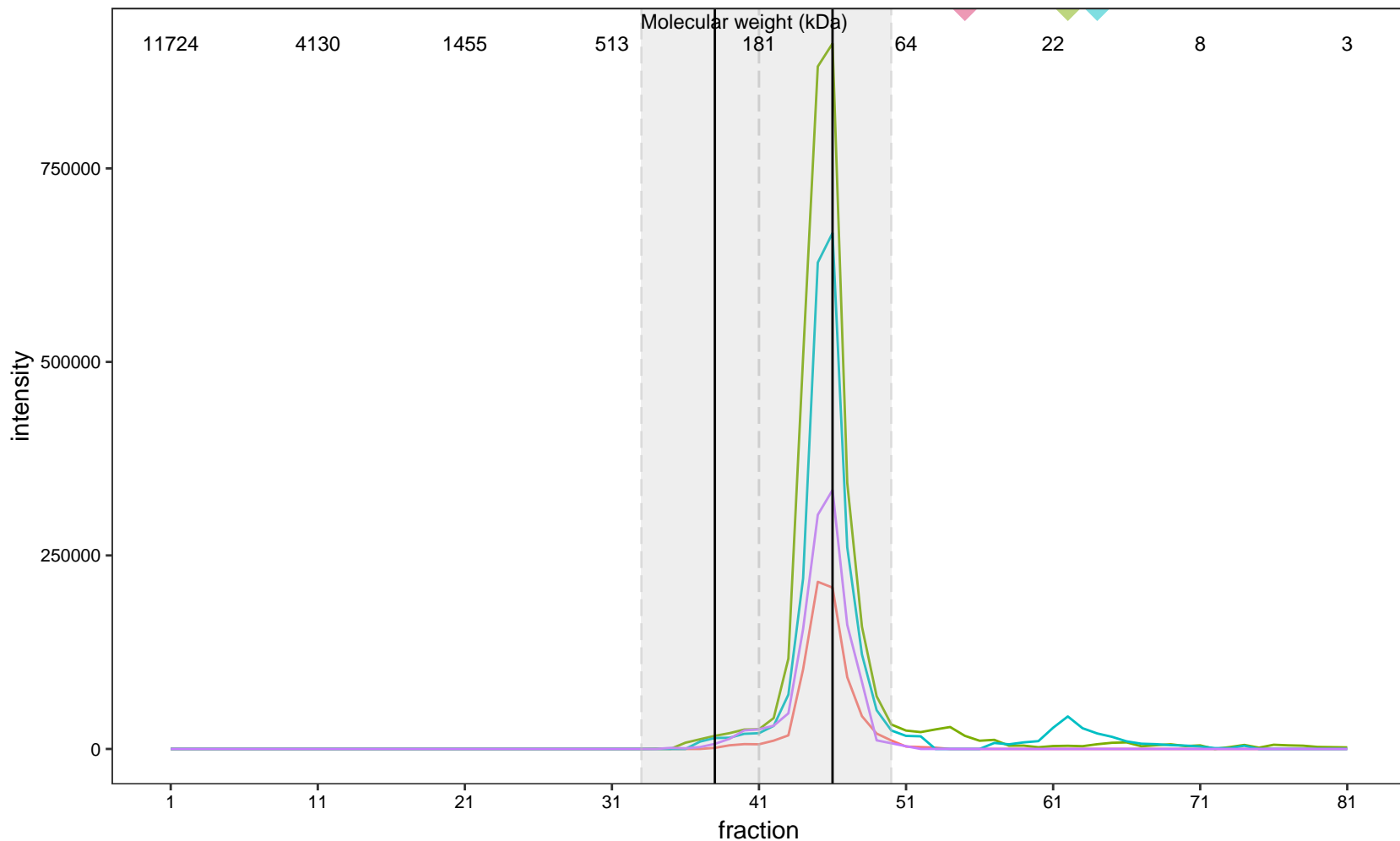

O15143 O15145 O15511 Q92747

Supplement: Supplementary file 7 — Dataset EV6 [file MSB-15-e8438-s007.zip › feature_plots_bioplex/O15511.pdf]

**O15514**

**Annotated subunits: 6 Subunits with signal: 4**

**Max. coeluting subunits: 2 Max. completeness: 0.33**

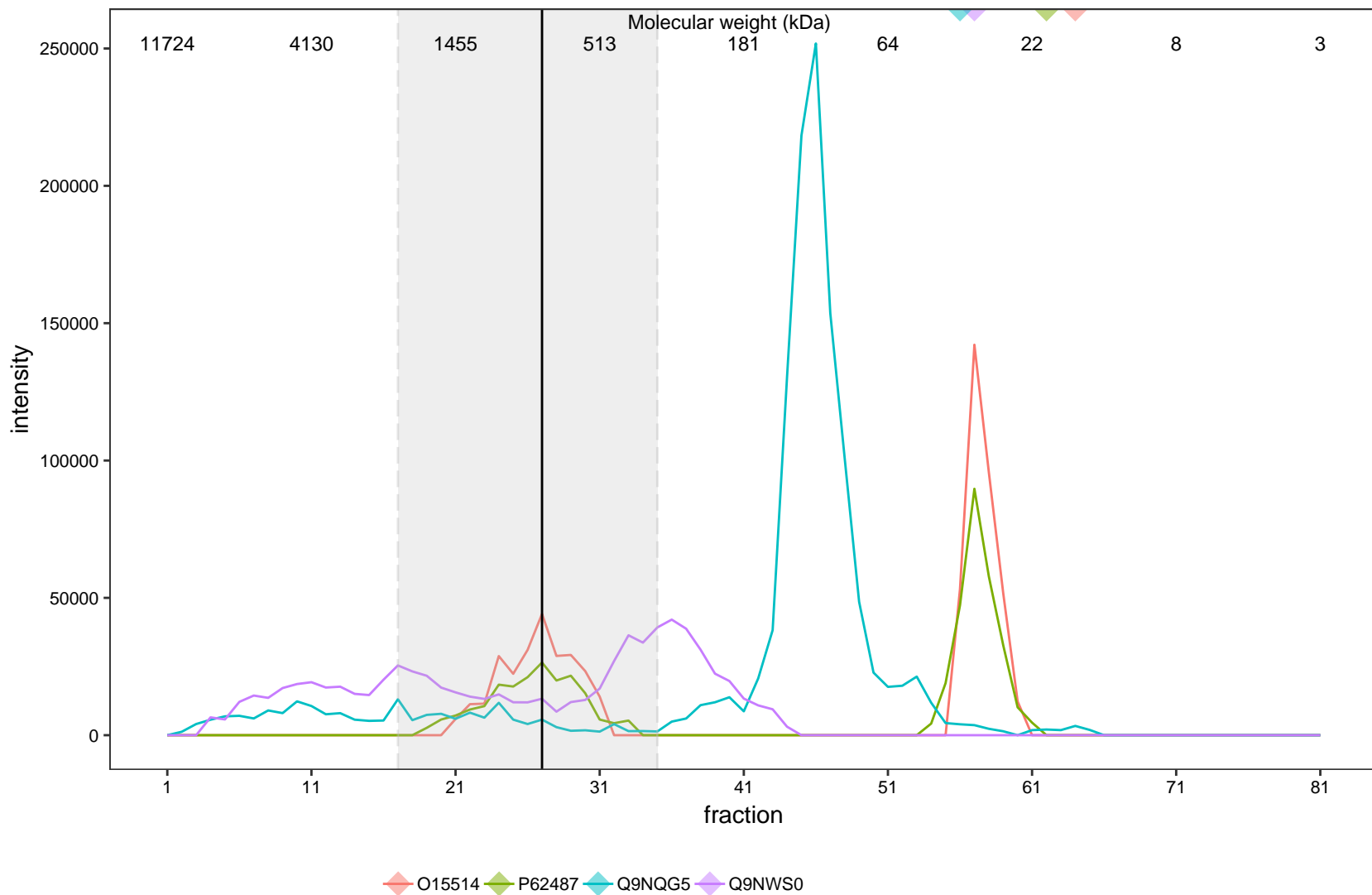

Supplement: Supplementary file 7 — Dataset EV6 [file MSB-15-e8438-s007.zip › feature_plots_bioplex/O15514.pdf]

**O43149**  
**Annotated subunits: 16 Subunits with signal: 7**  
**Max. coeluting subunits: 2 Max. completeness: 0.12**

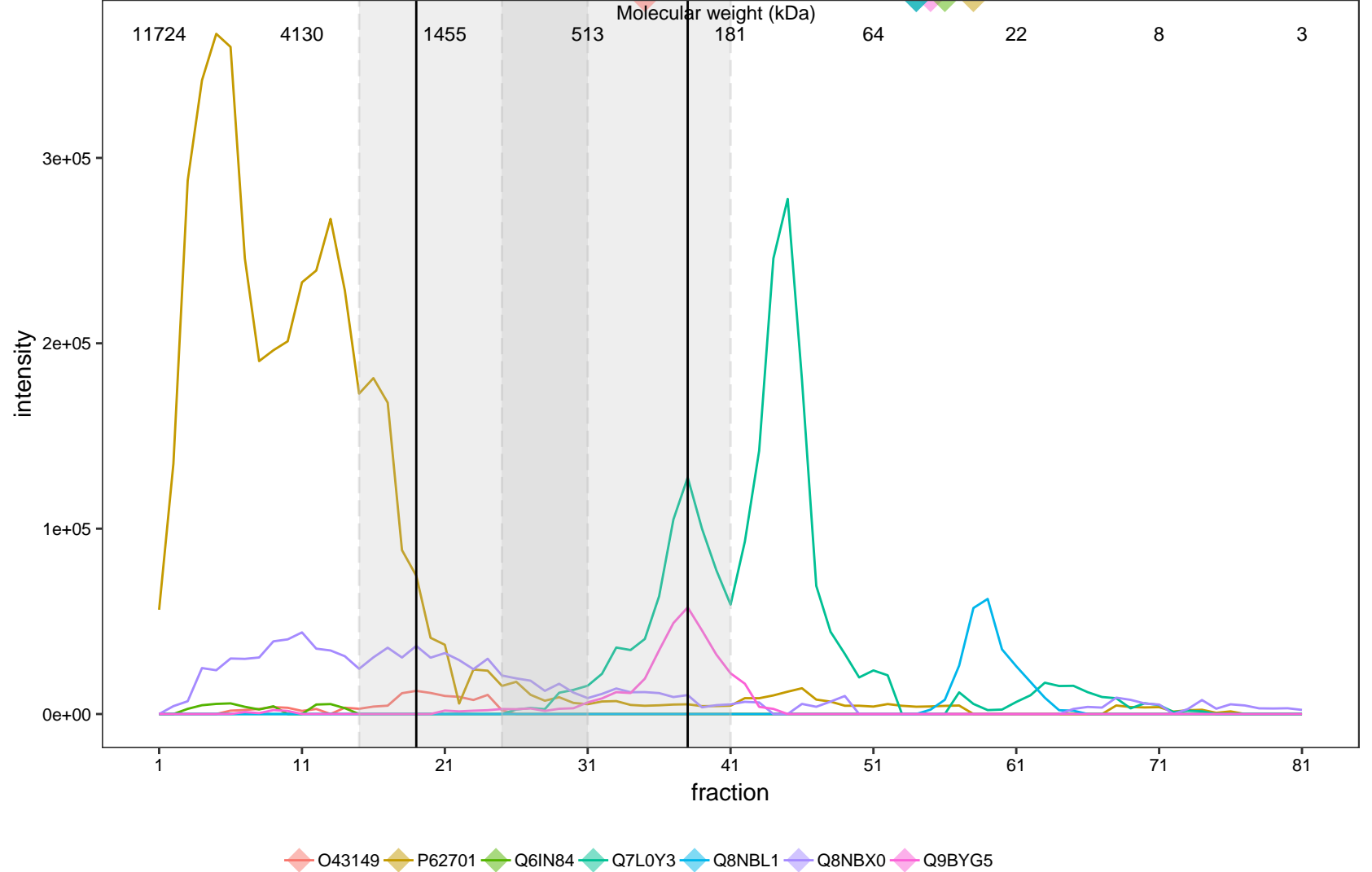

Supplement: Supplementary file 7 — Dataset EV6 [file MSB-15-e8438-s007.zip › feature_plots_bioplex/O43149.pdf]

**O43159**

**Annotated subunits: 8 Subunits with signal: 6**

**Max. coeluting subunits: 3 Max. completeness: 0.38**

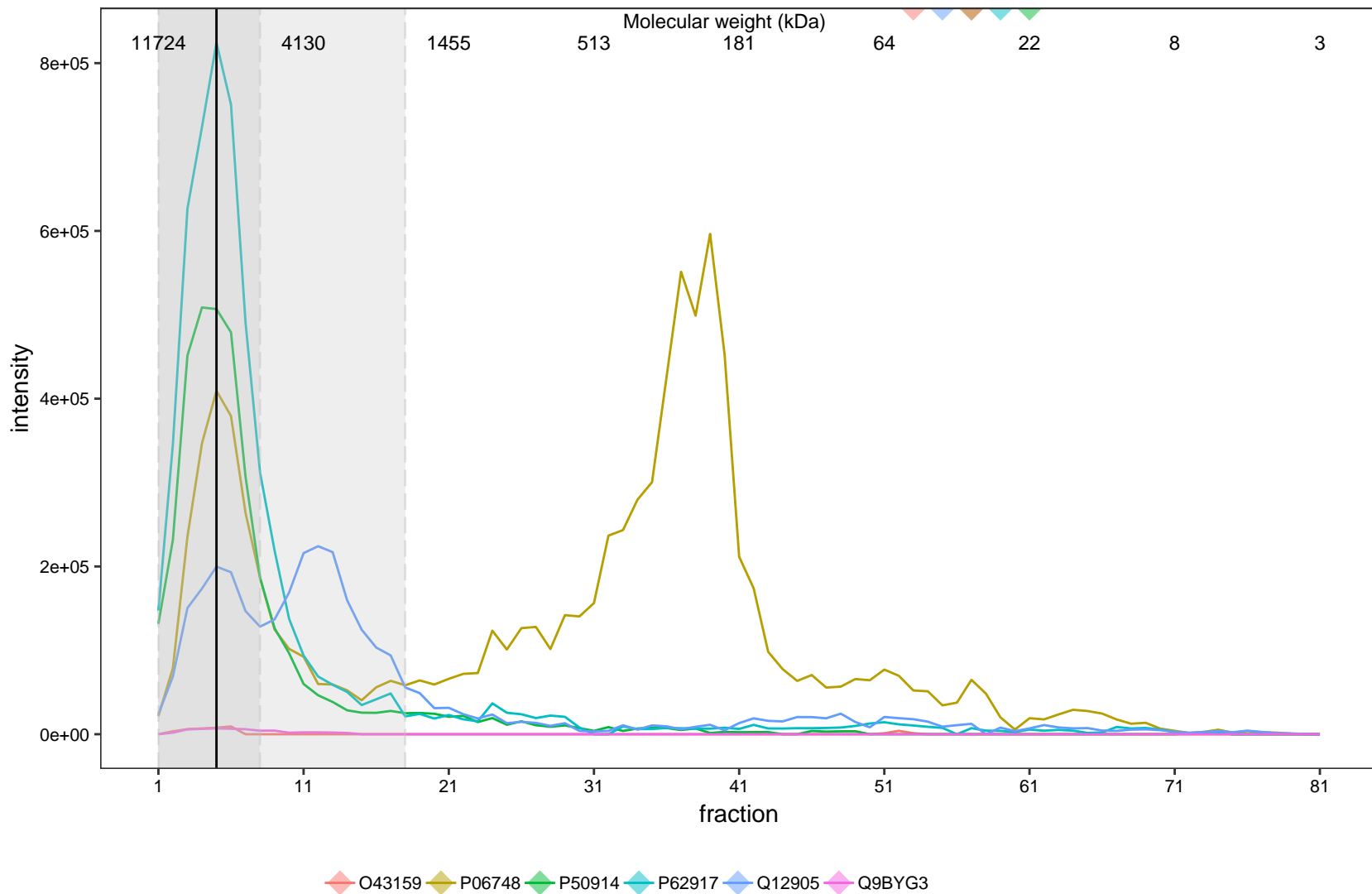

Supplement: Supplementary file 7 — Dataset EV6 [file MSB-15-e8438-s007.zip › feature_plots_bioplex/O43159.pdf]

**O43181**

**Annotated subunits: 5 Subunits with signal: 3**

**Max. coeluting subunits: 3 Max. completeness: 0.6**

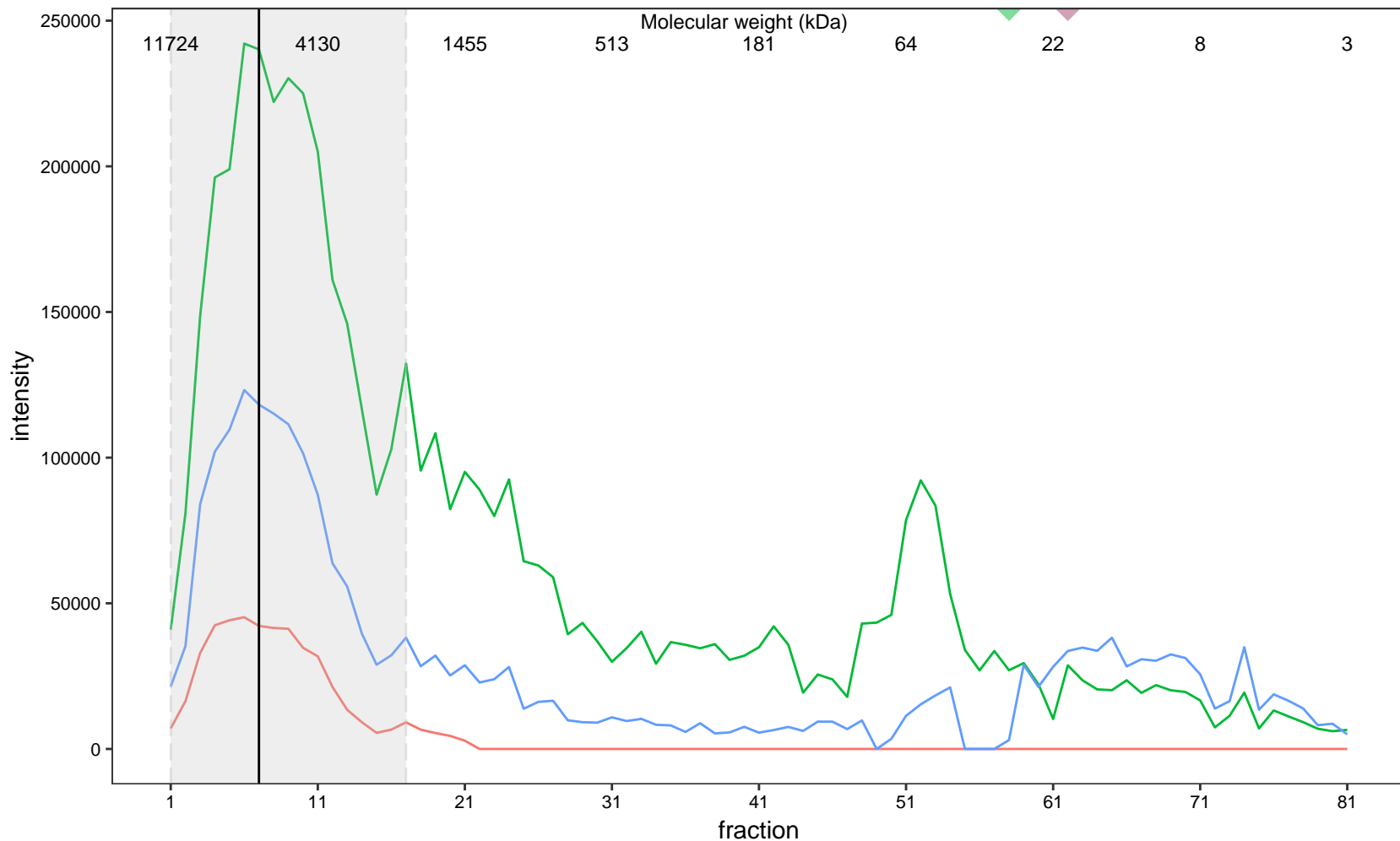

◊ O43181 ◊ O75489 ◊ P51970

Supplement: Supplementary file 7 — Dataset EV6 [file MSB-15-e8438-s007.zip › feature_plots_bioplex/O43181.pdf]

**O43264**

**Annotated subunits: 6 Subunits with signal: 4**

**Max. coeluting subunits: 3 Max. completeness: 0.5**

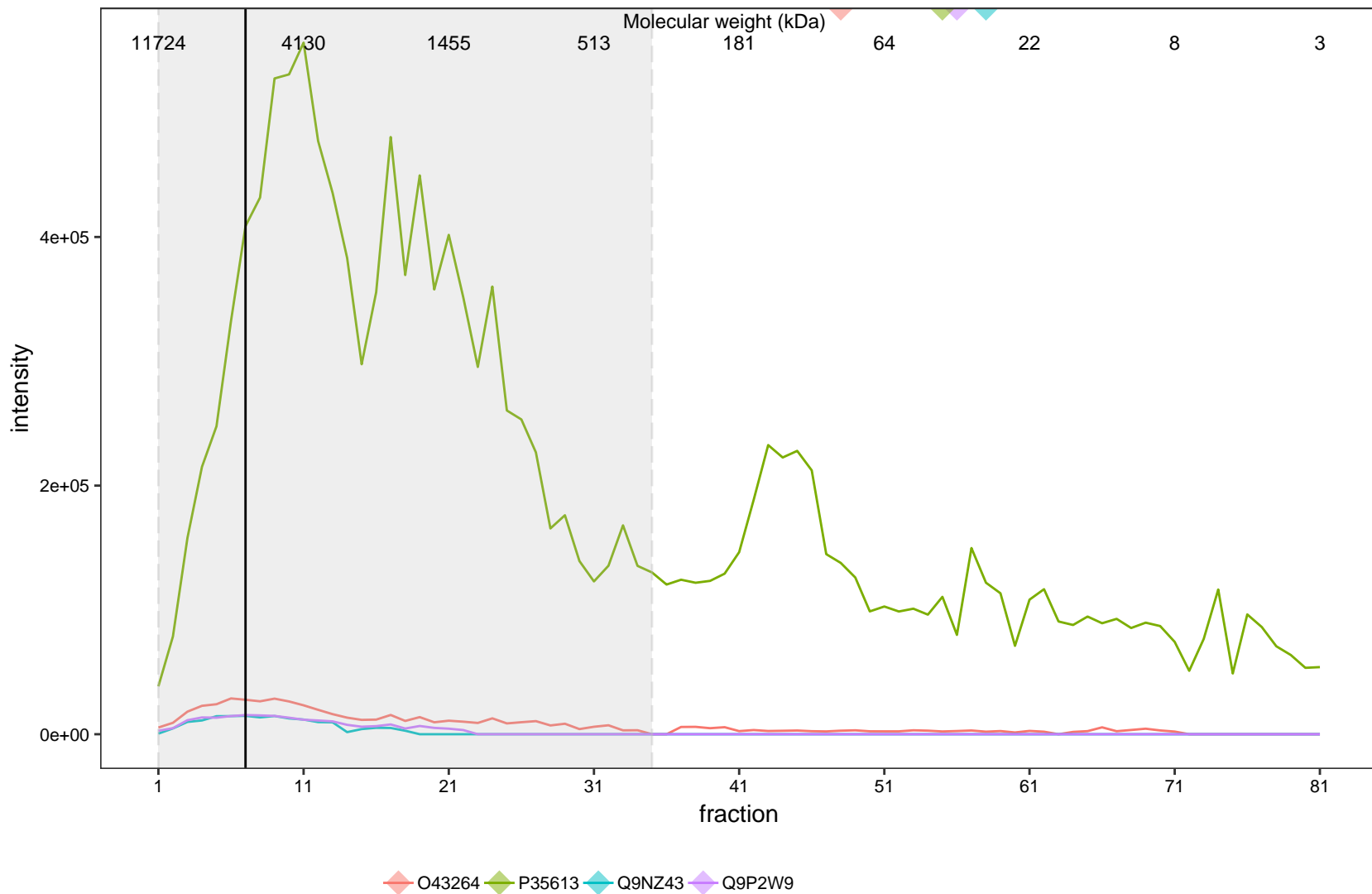

Supplement: Supplementary file 7 — Dataset EV6 [file MSB-15-e8438-s007.zip › feature_plots_bioplex/O43264.pdf]

**O43292**

**Annotated subunits: 10 Subunits with signal: 2**

**Max. coeluting subunits: 2 Max. completeness: 0.2**

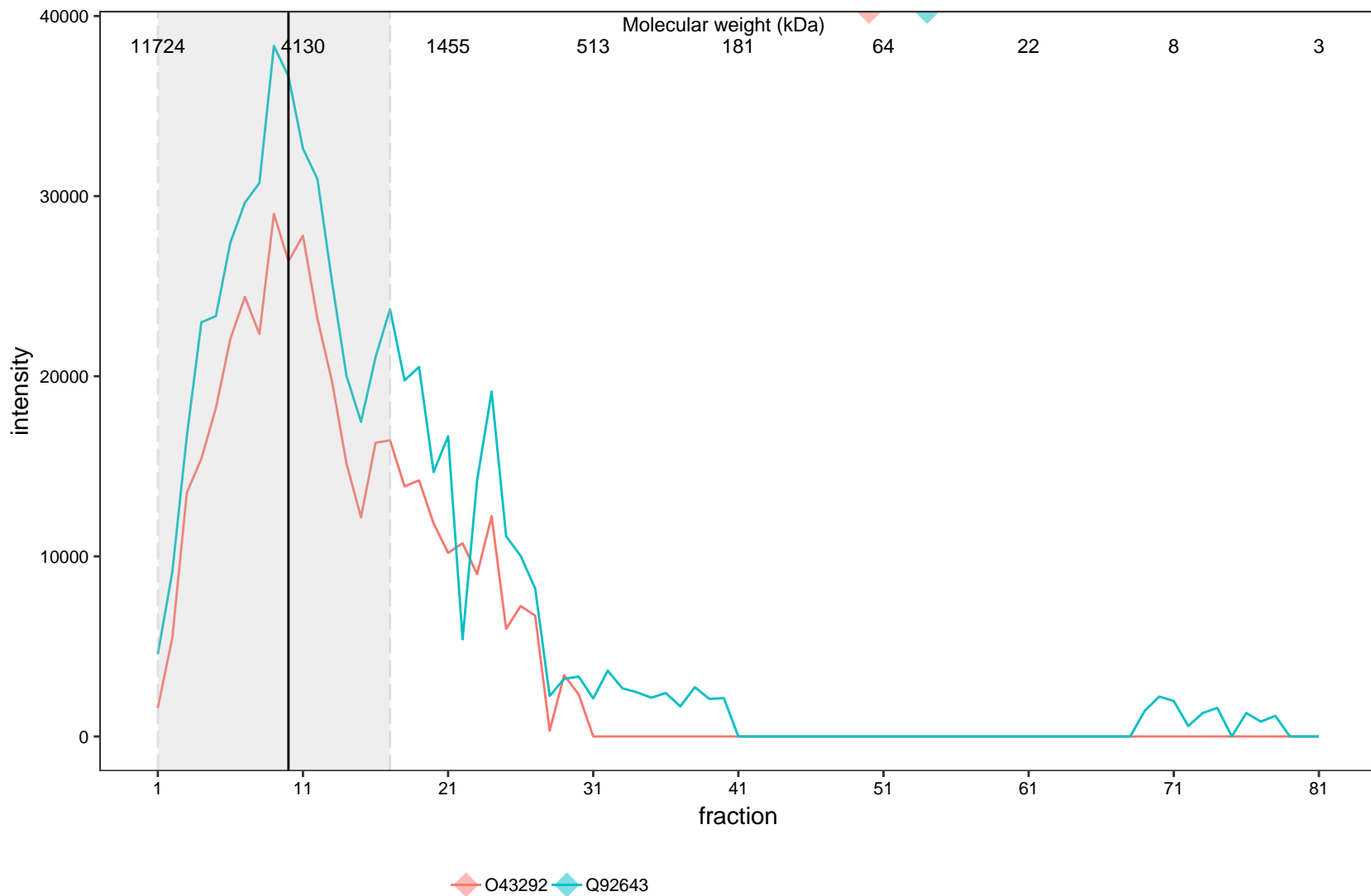

Supplement: Supplementary file 7 — Dataset EV6 [file MSB-15-e8438-s007.zip › feature_plots_bioplex/O43292.pdf]

**O43390**  
**Annotated subunits: 5   Subunits with signal: 4**  
**Max. coeluting subunits: 2   Max. completeness: 0.4**

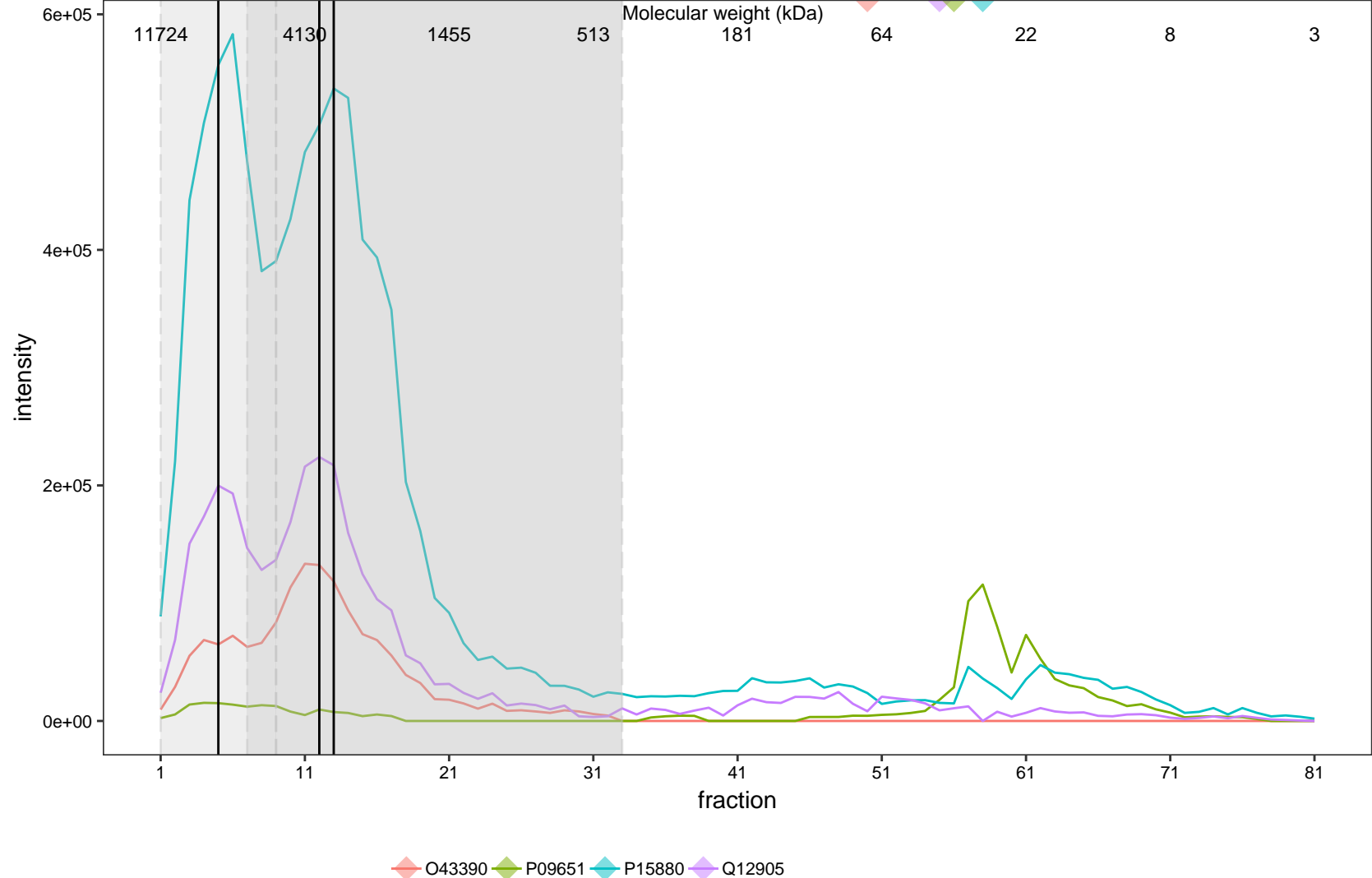

Supplement: Supplementary file 7 — Dataset EV6 [file MSB-15-e8438-s007.zip › feature_plots_bioplex/O43390.pdf]

**O43399**

**Annotated subunits: 4 Subunits with signal: 4**

**Max. coeluting subunits: 2 Max. completeness: 0.5**

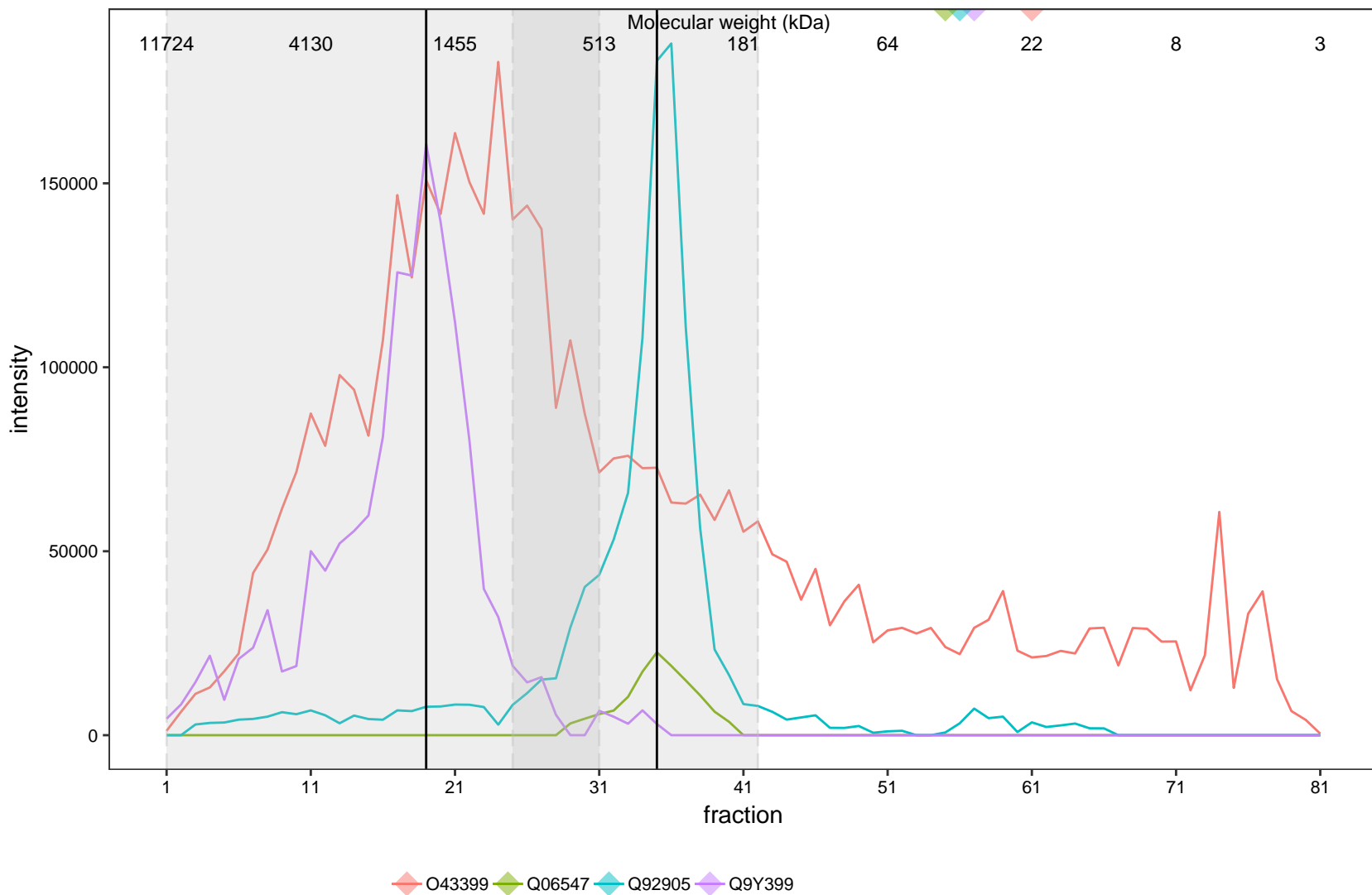

Supplement: Supplementary file 7 — Dataset EV6 [file MSB-15-e8438-s007.zip › feature_plots_bioplex/O43399.pdf]

**O43402**

**Annotated subunits: 7 Subunits with signal: 2**

**Max. coeluting subunits: 2 Max. completeness: 0.29**

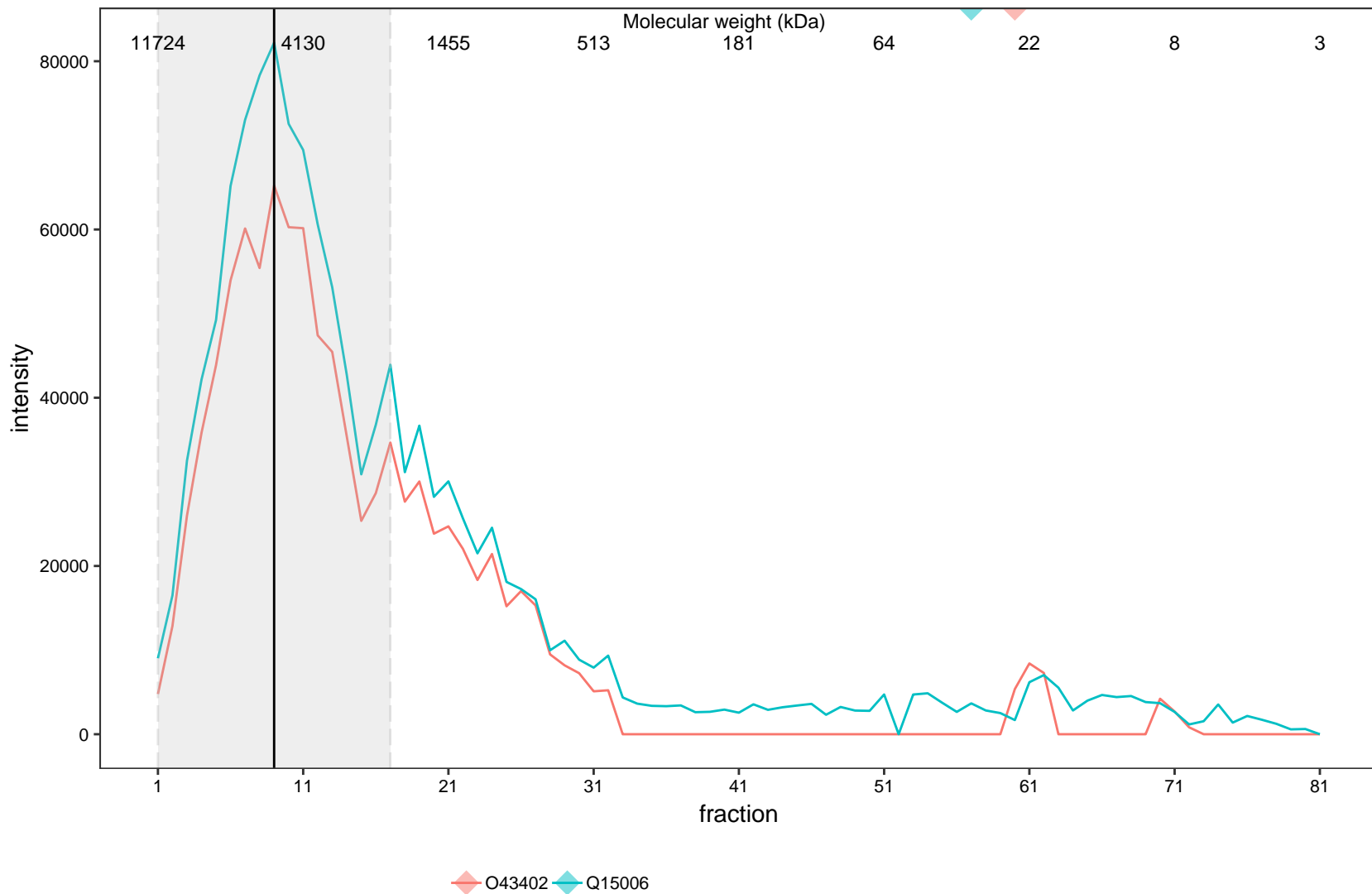

Supplement: Supplementary file 7 — Dataset EV6 [file MSB-15-e8438-s007.zip › feature_plots_bioplex/O43402.pdf]

**O43432**

**Annotated subunits: 10 Subunits with signal: 7**

**Max. coeluting subunits: 4 Max. completeness: 0.4**

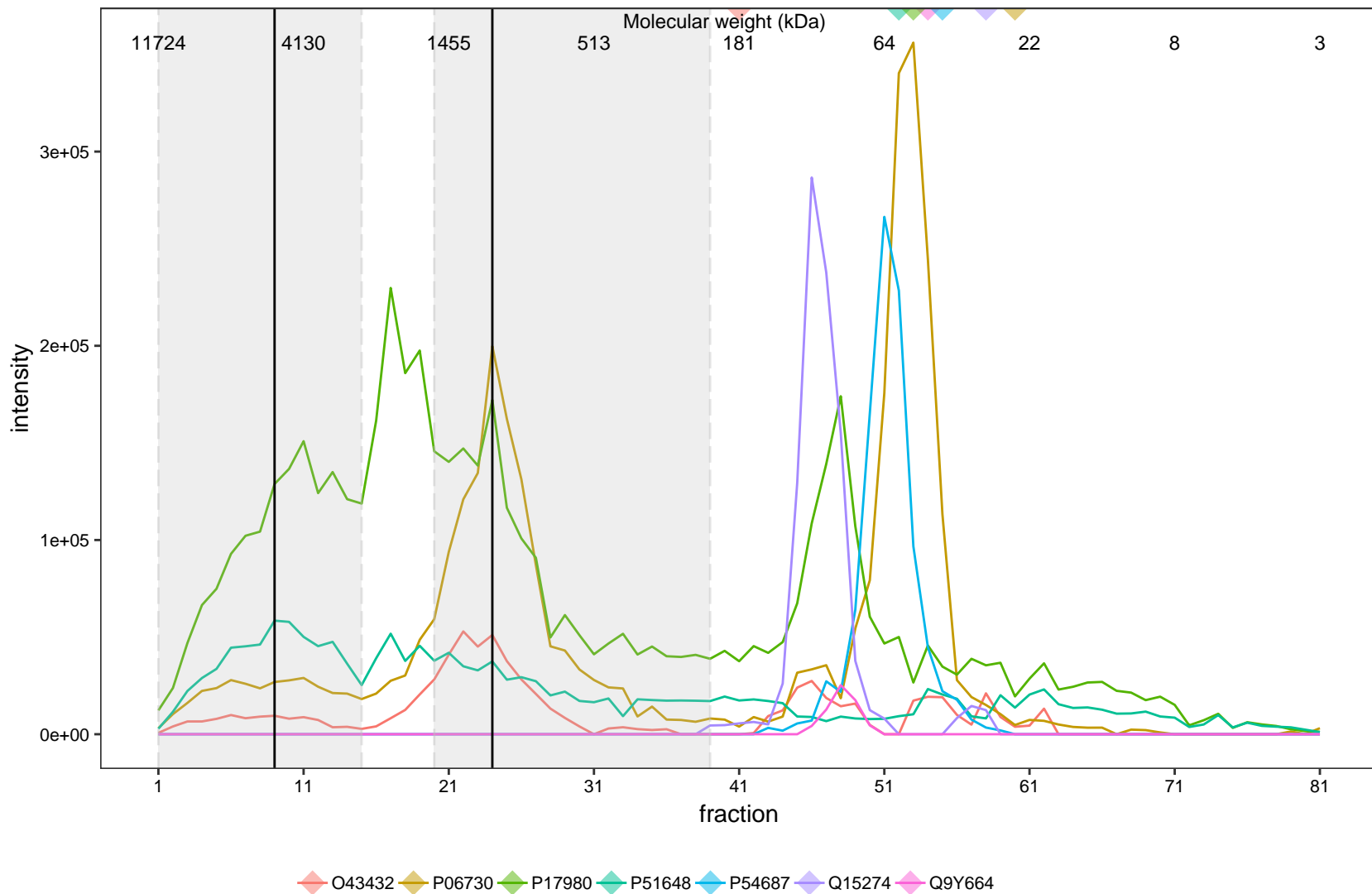

Supplement: Supplementary file 7 — Dataset EV6 [file MSB-15-e8438-s007.zip › feature_plots_bioplex/O43432.pdf]

O43566

Annotated subunits: 3 Subunits with signal: 2

Max. coeluting subunits: 2 Max. completeness: 0.67

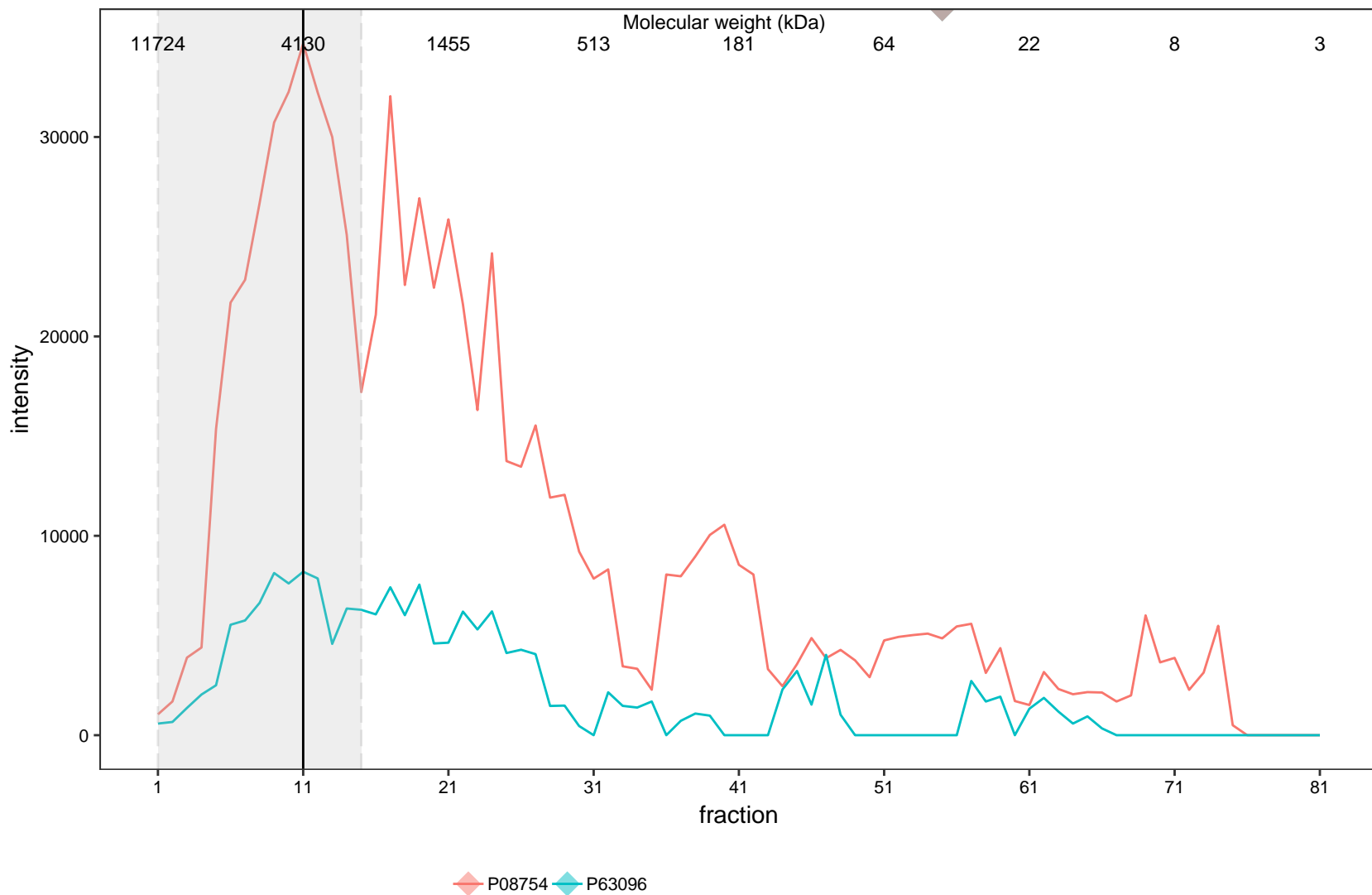

Supplement: Supplementary file 7 — Dataset EV6 [file MSB-15-e8438-s007.zip › feature_plots_bioplex/O43566.pdf]

**O43617**

**Annotated subunits: 6 Subunits with signal: 2**

**Max. coeluting subunits: 2 Max. completeness: 0.33**

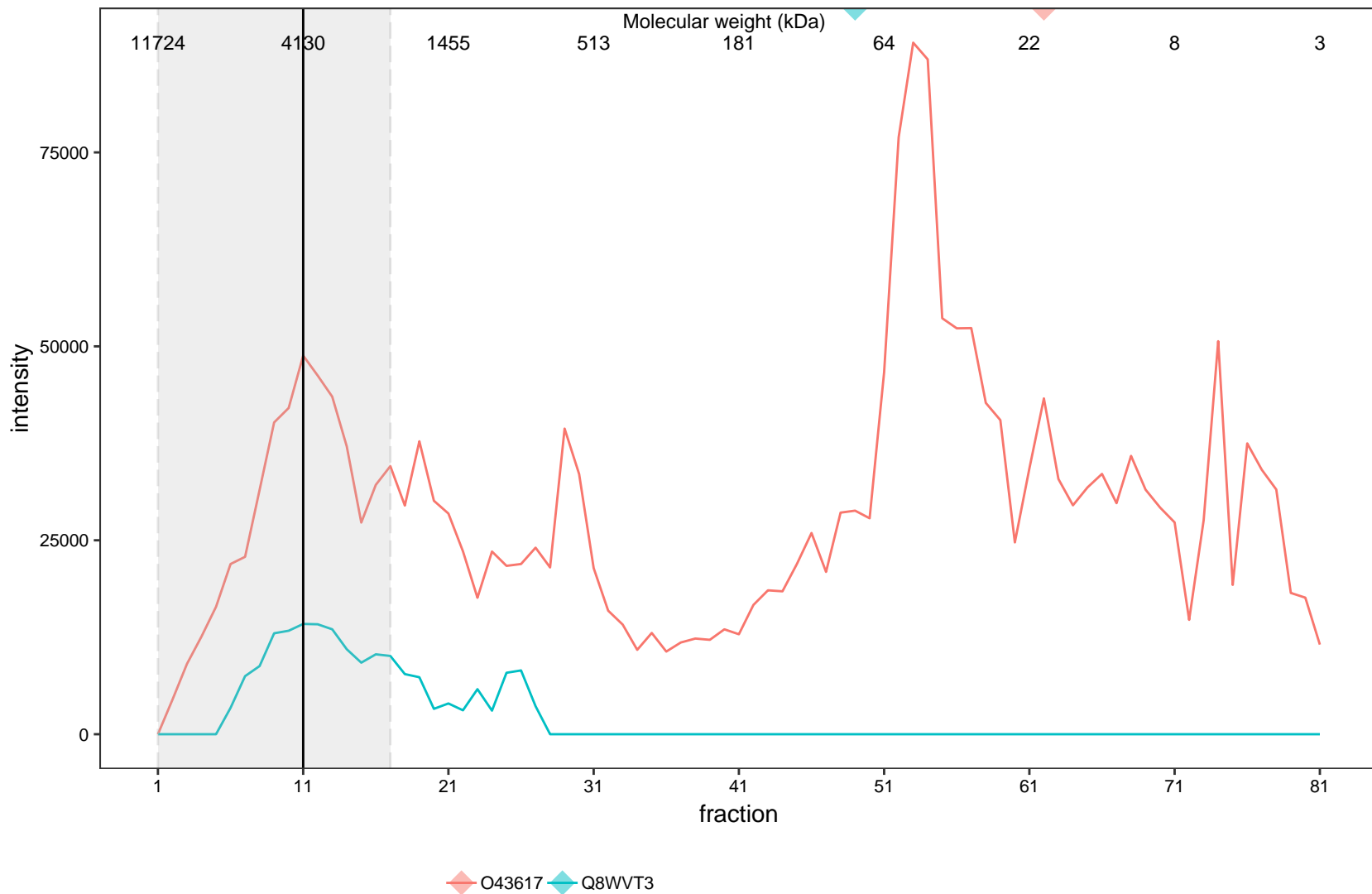

Supplement: Supplementary file 7 — Dataset EV6 [file MSB-15-e8438-s007.zip › feature_plots_bioplex/O43617.pdf]

O43670  
Annotated subunits: 2 Subunits with signal: 2  
Max. coeluting subunits: 2 Max. completeness: 1

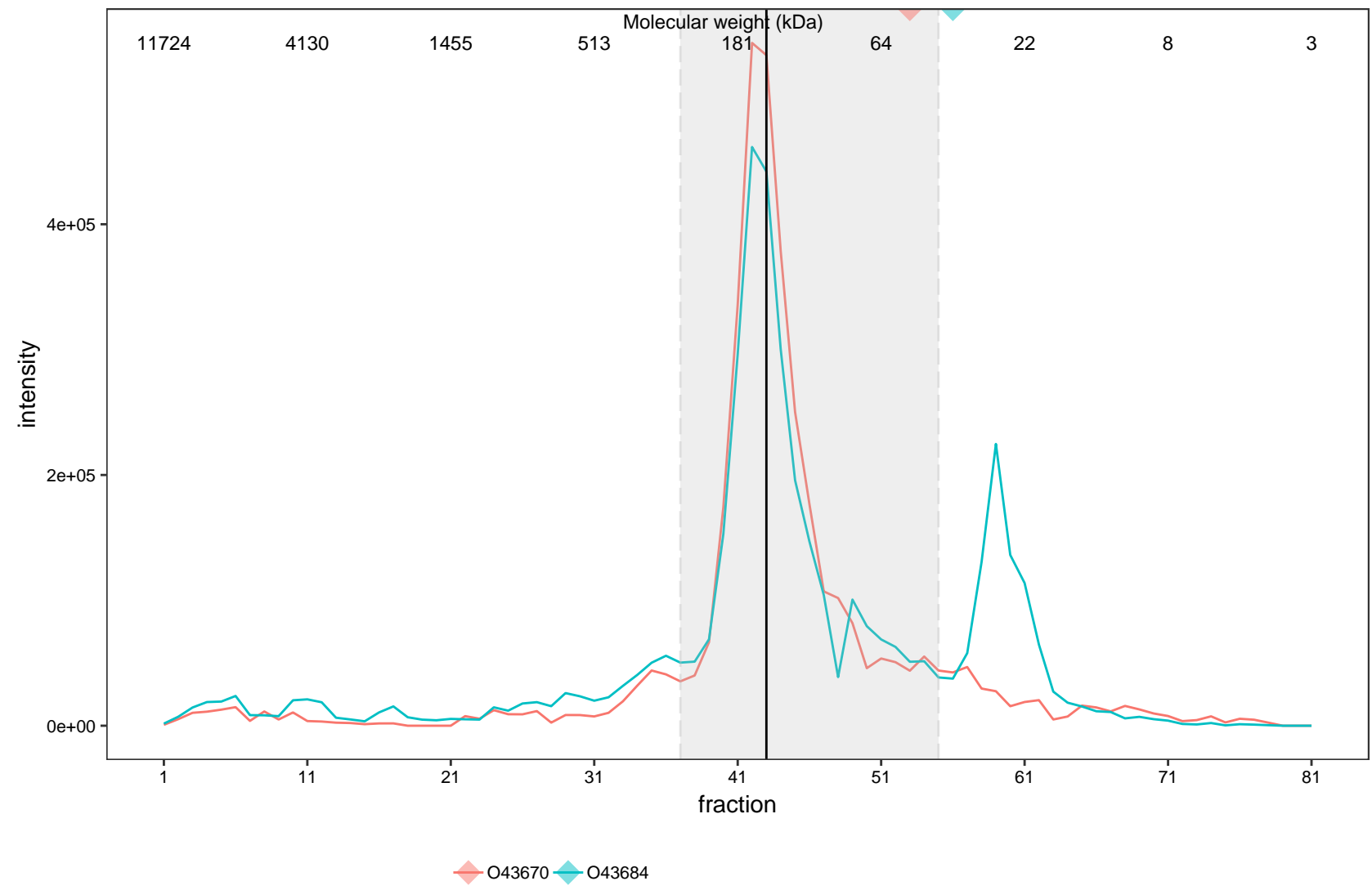

Supplement: Supplementary file 7 — Dataset EV6 [file MSB-15-e8438-s007.zip › feature_plots_bioplex/O43670.pdf]

**O43674**

**Annotated subunits: 5 Subunits with signal: 4**

**Max. coeluting subunits: 4 Max. completeness: 0.8**

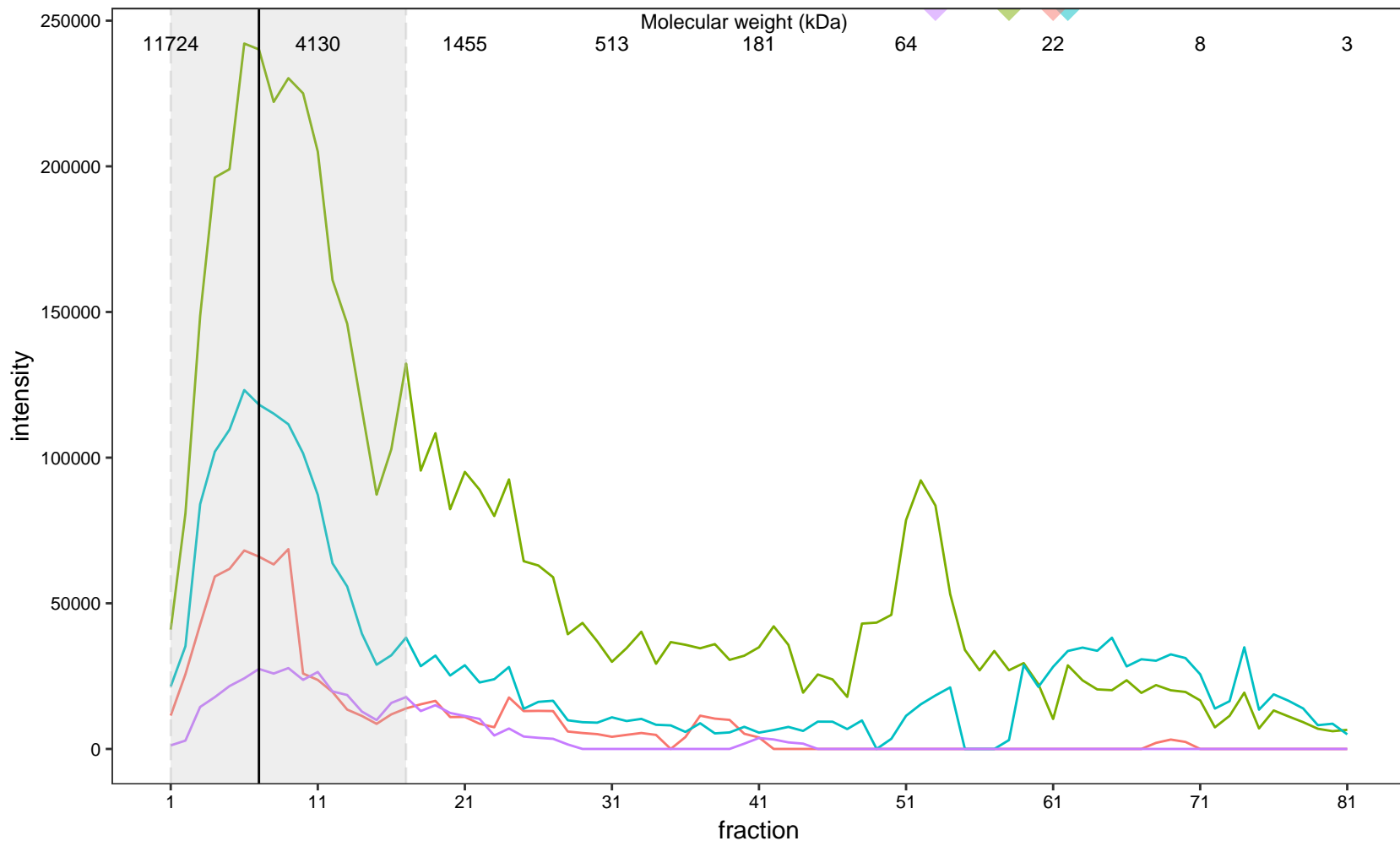

◈ O43674 ◈ O75489 ◈ P51970 ◈ Q9BQ95

Supplement: Supplementary file 7 — Dataset EV6 [file MSB-15-e8438-s007.zip › feature_plots_bioplex/O43674.pdf]

**O43676**

**Annotated subunits: 5 Subunits with signal: 3**

**Max. coeluting subunits: 3 Max. completeness: 0.6**

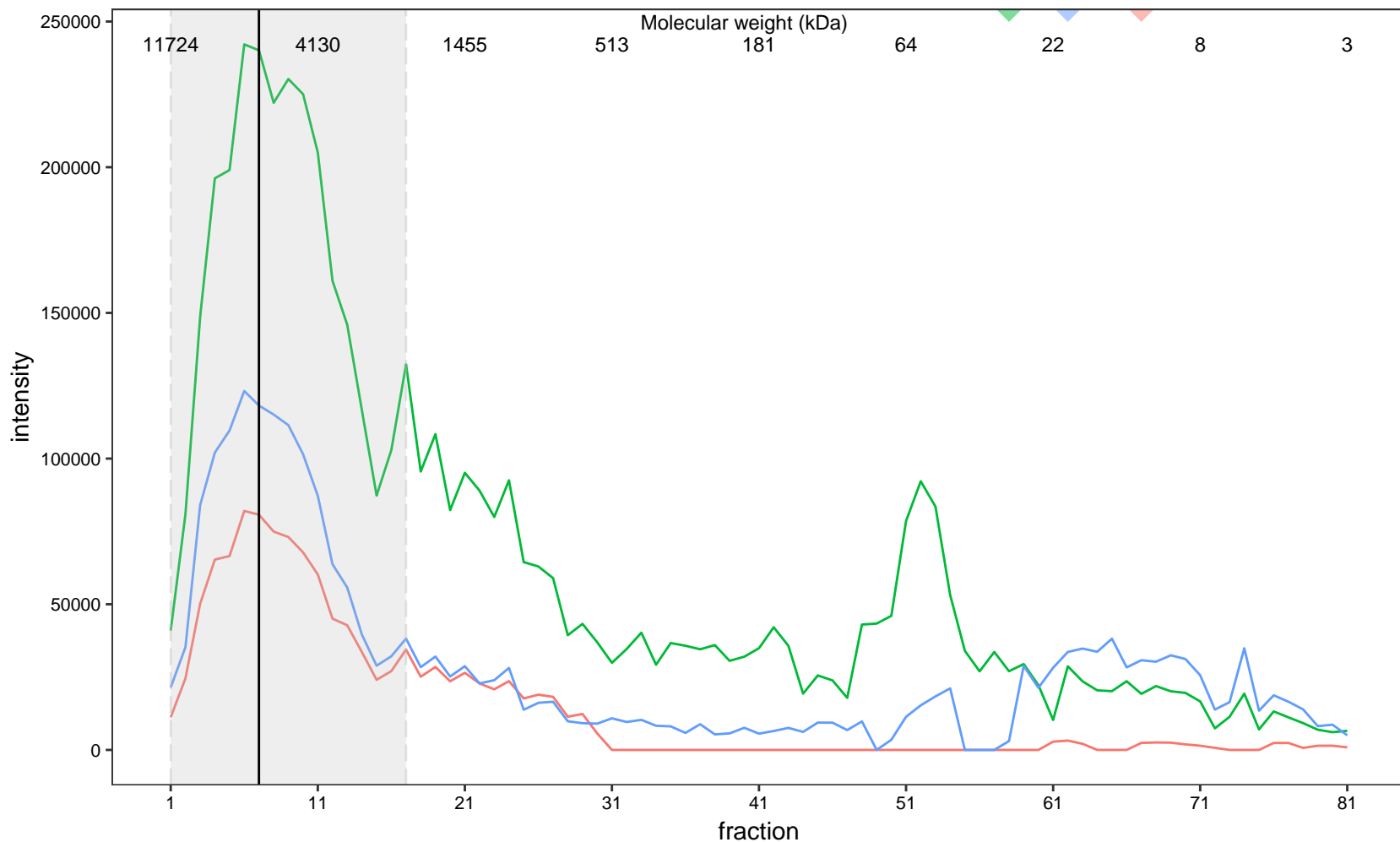

◊ O43676 ◊ O75489 ◊ P51970

Supplement: Supplementary file 7 — Dataset EV6 [file MSB-15-e8438-s007.zip › feature_plots_bioplex/O43676.pdf]

O43681  
Annotated subunits: 4   Subunits with signal: 3  
Max. coeluting subunits: 2   Max. completeness: 0.5

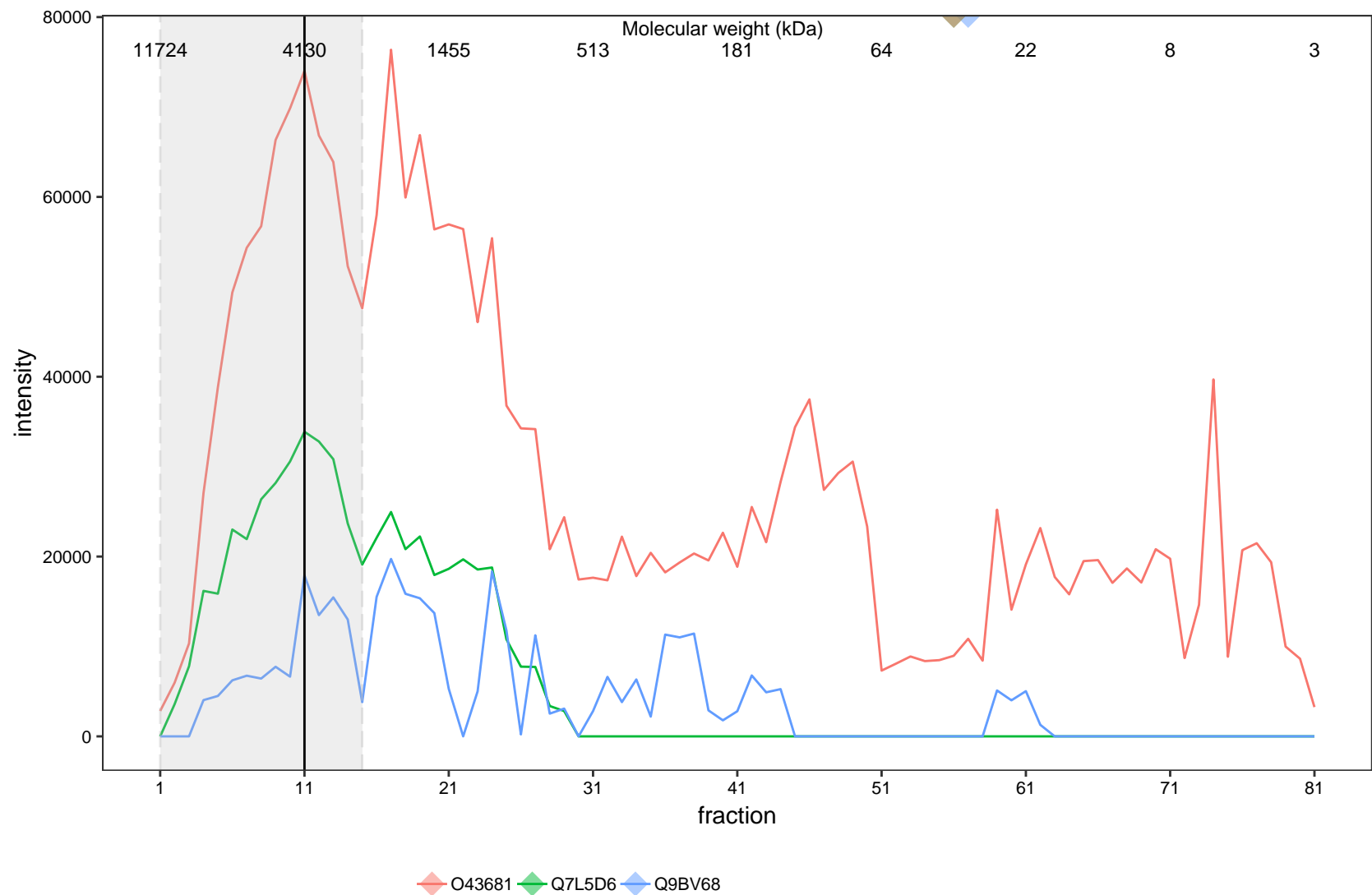

Supplement: Supplementary file 7 — Dataset EV6 [file MSB-15-e8438-s007.zip › feature_plots_bioplex/O43681.pdf]

**O43747**

**Annotated subunits: 5 Subunits with signal: 4**

**Max. coeluting subunits: 2 Max. completeness: 0.4**

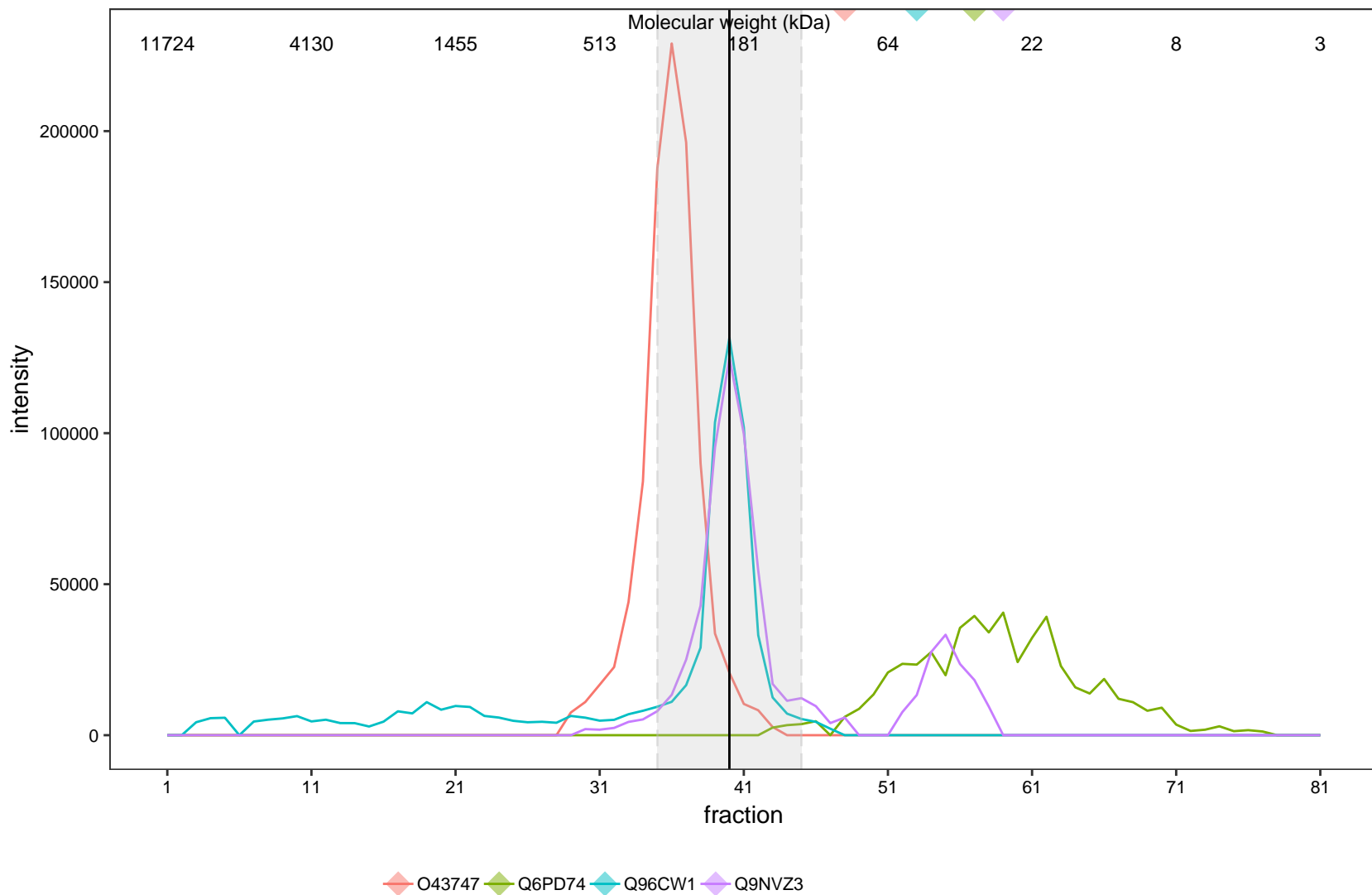

Supplement: Supplementary file 7 — Dataset EV6 [file MSB-15-e8438-s007.zip › feature_plots_bioplex/O43747.pdf]

**O43759**

**Annotated subunits: 2 Subunits with signal: 2**

**Max. coeluting subunits: 2 Max. completeness: 1**

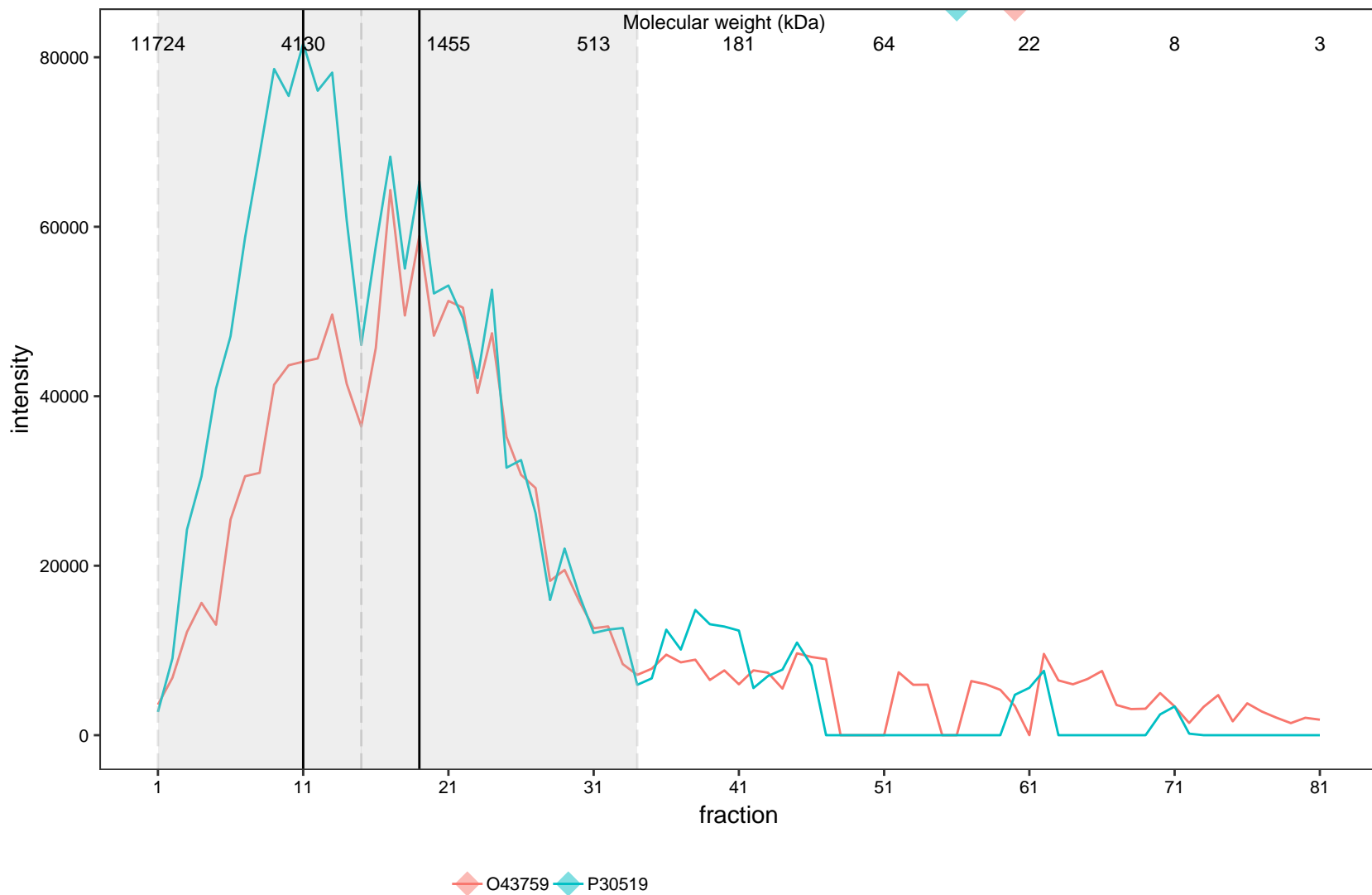

Supplement: Supplementary file 7 — Dataset EV6 [file MSB-15-e8438-s007.zip › feature_plots_bioplex/O43759.pdf]

**O43765**

**Annotated subunits: 2 Subunits with signal: 2**

**Max. coeluting subunits: 2 Max. completeness: 1**

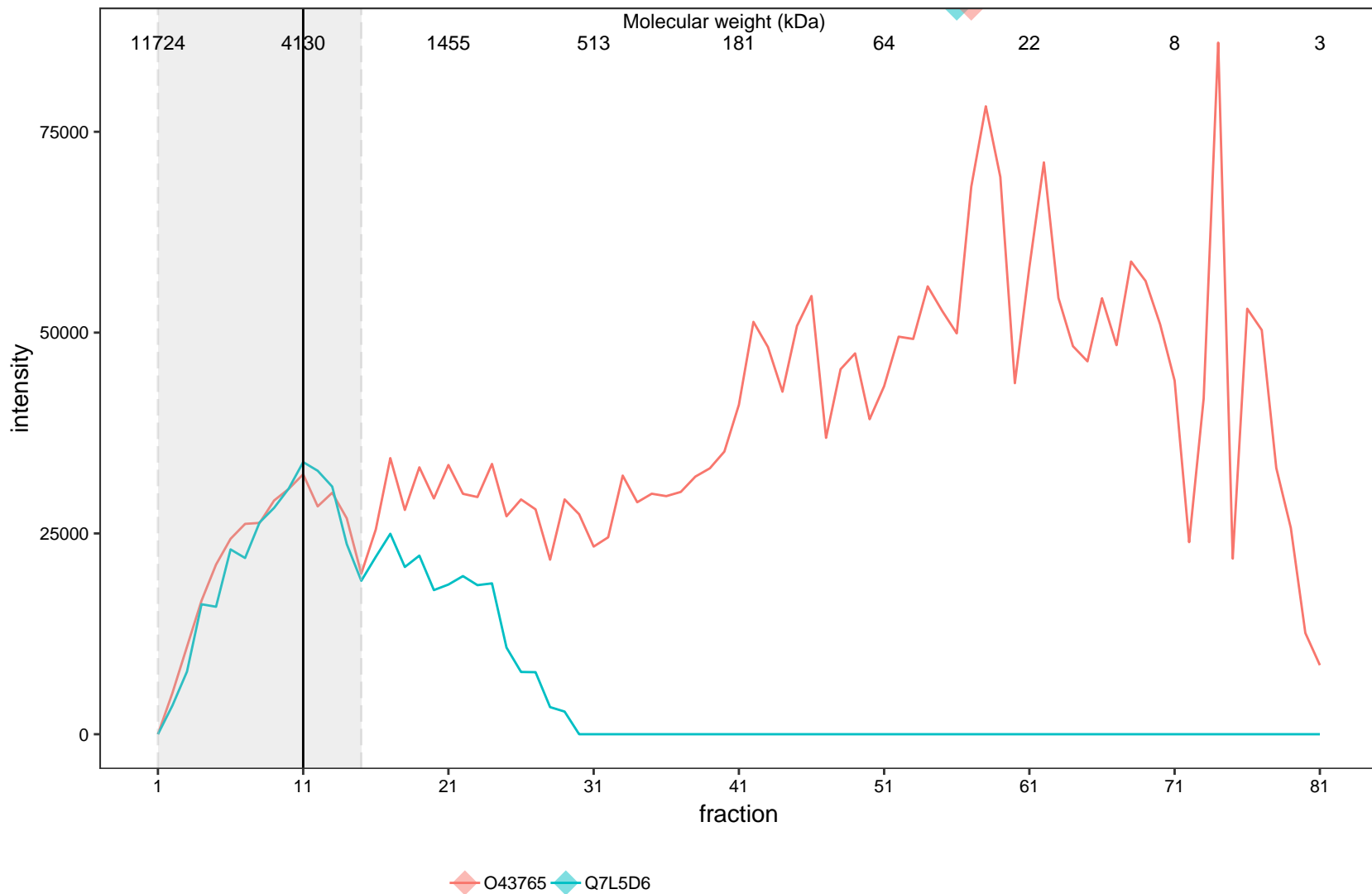

Supplement: Supplementary file 7 — Dataset EV6 [file MSB-15-e8438-s007.zip › feature_plots_bioplex/O43765.pdf]

O43813  
Annotated subunits: 10   Subunits with signal: 5  
Max. coeluting subunits: 4   Max. completeness: 0.4

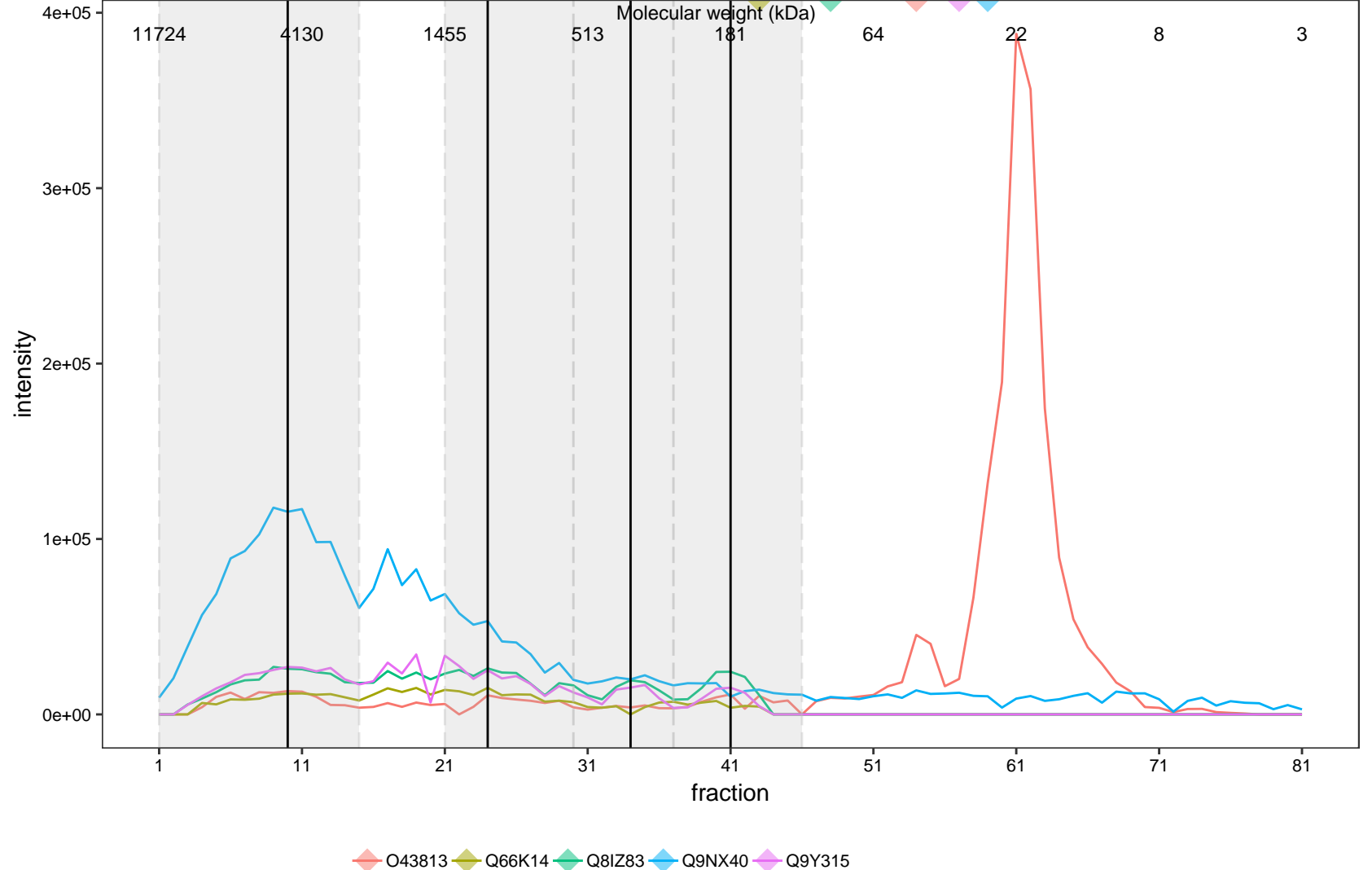

Supplement: Supplementary file 7 — Dataset EV6 [file MSB-15-e8438-s007.zip › feature_plots_bioplex/O43813.pdf]

O43837  
Annotated subunits: 5   Subunits with signal: 4  
Max. coeluting subunits: 3   Max. completeness: 0.6

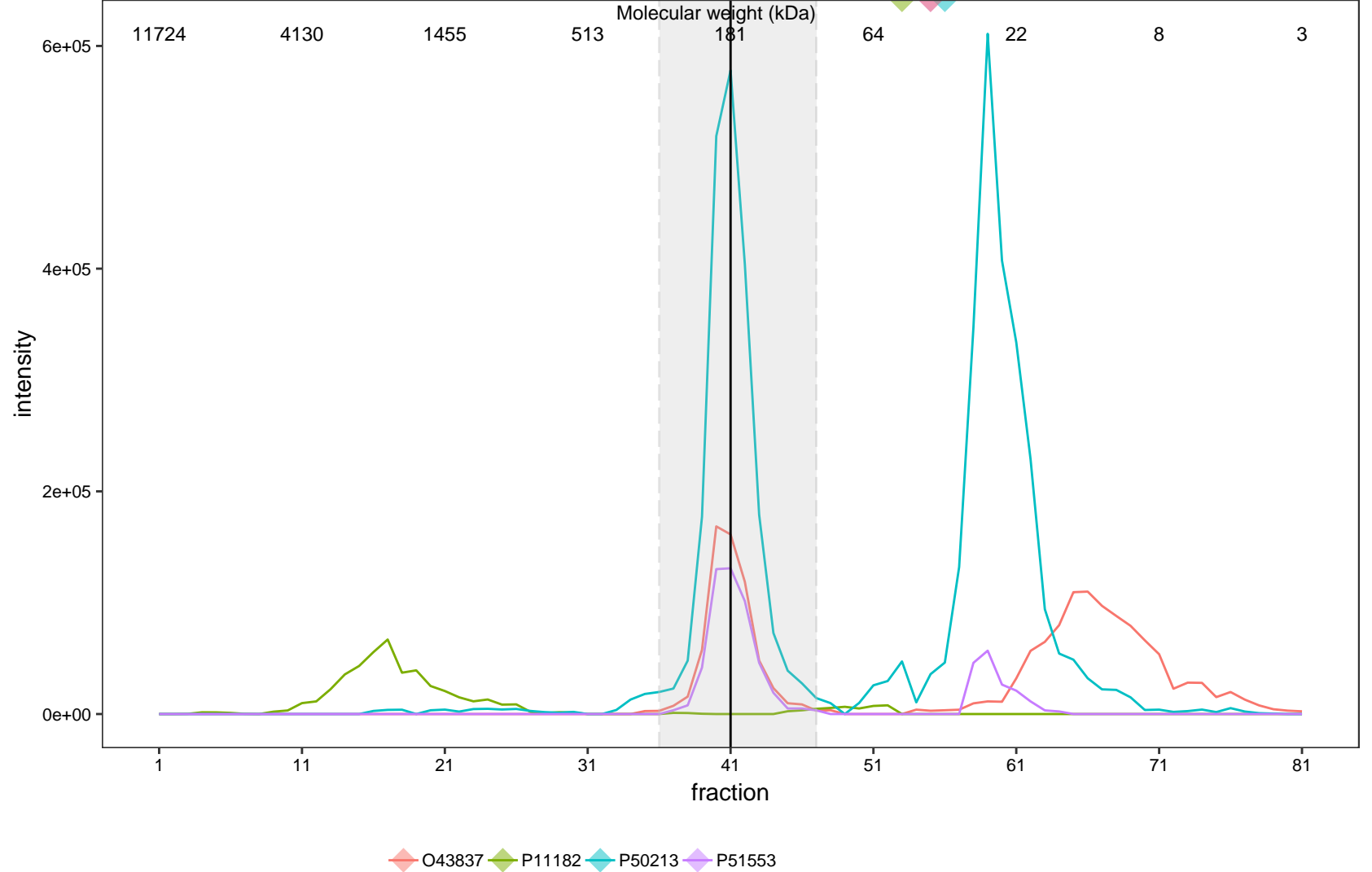

Supplement: Supplementary file 7 — Dataset EV6 [file MSB-15-e8438-s007.zip › feature_plots_bioplex/O43837.pdf]

**O60262**

**Annotated subunits: 6   Subunits with signal: 3**

**Max. coeluting subunits: 3   Max. completeness: 0.5**

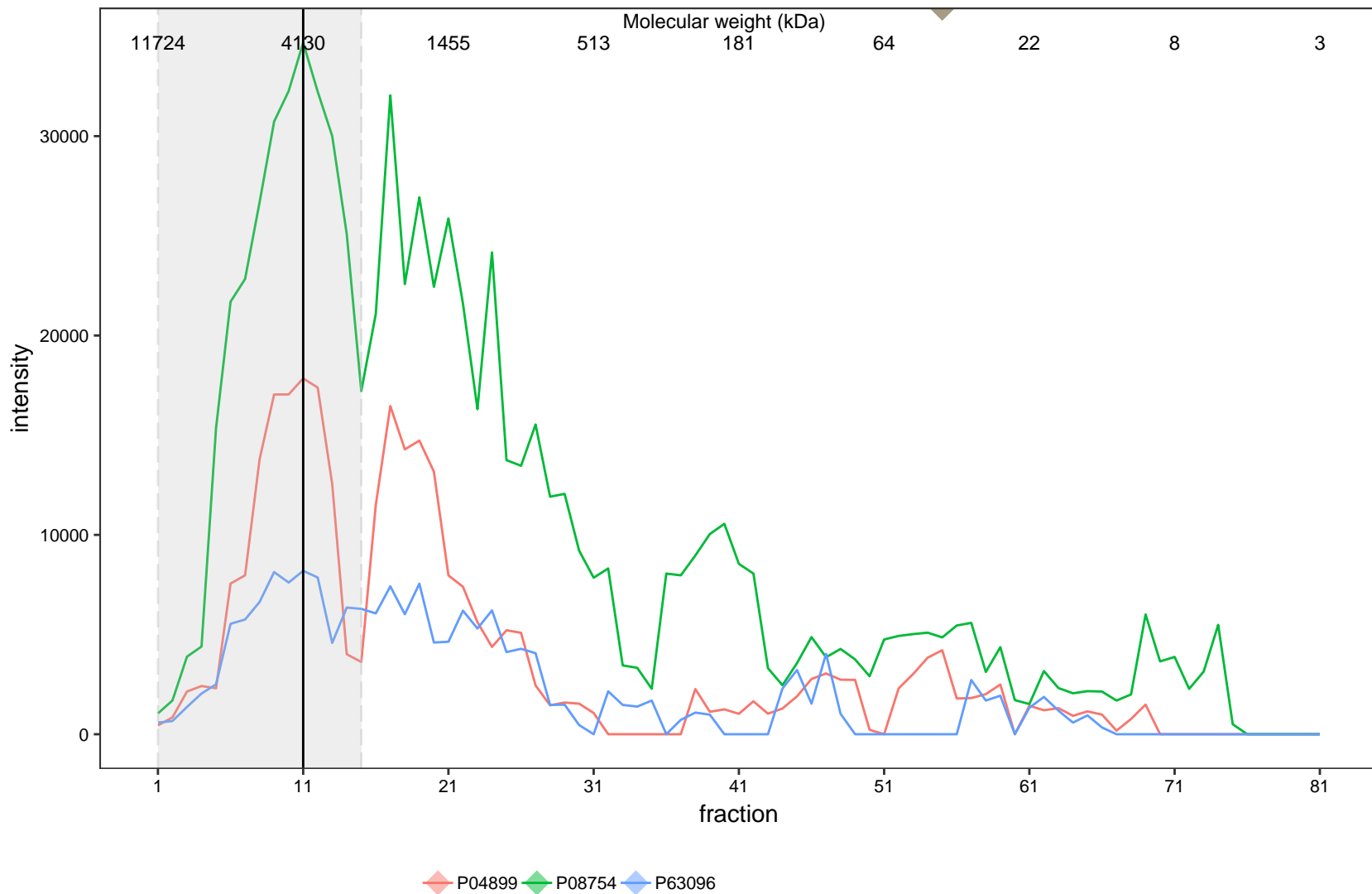

Supplement: Supplementary file 7 — Dataset EV6 [file MSB-15-e8438-s007.zip › feature_plots_bioplex/O60262.pdf]

**O60287**

**Annotated subunits: 4 Subunits with signal: 3**

**Max. coeluting subunits: 3 Max. completeness: 0.75**

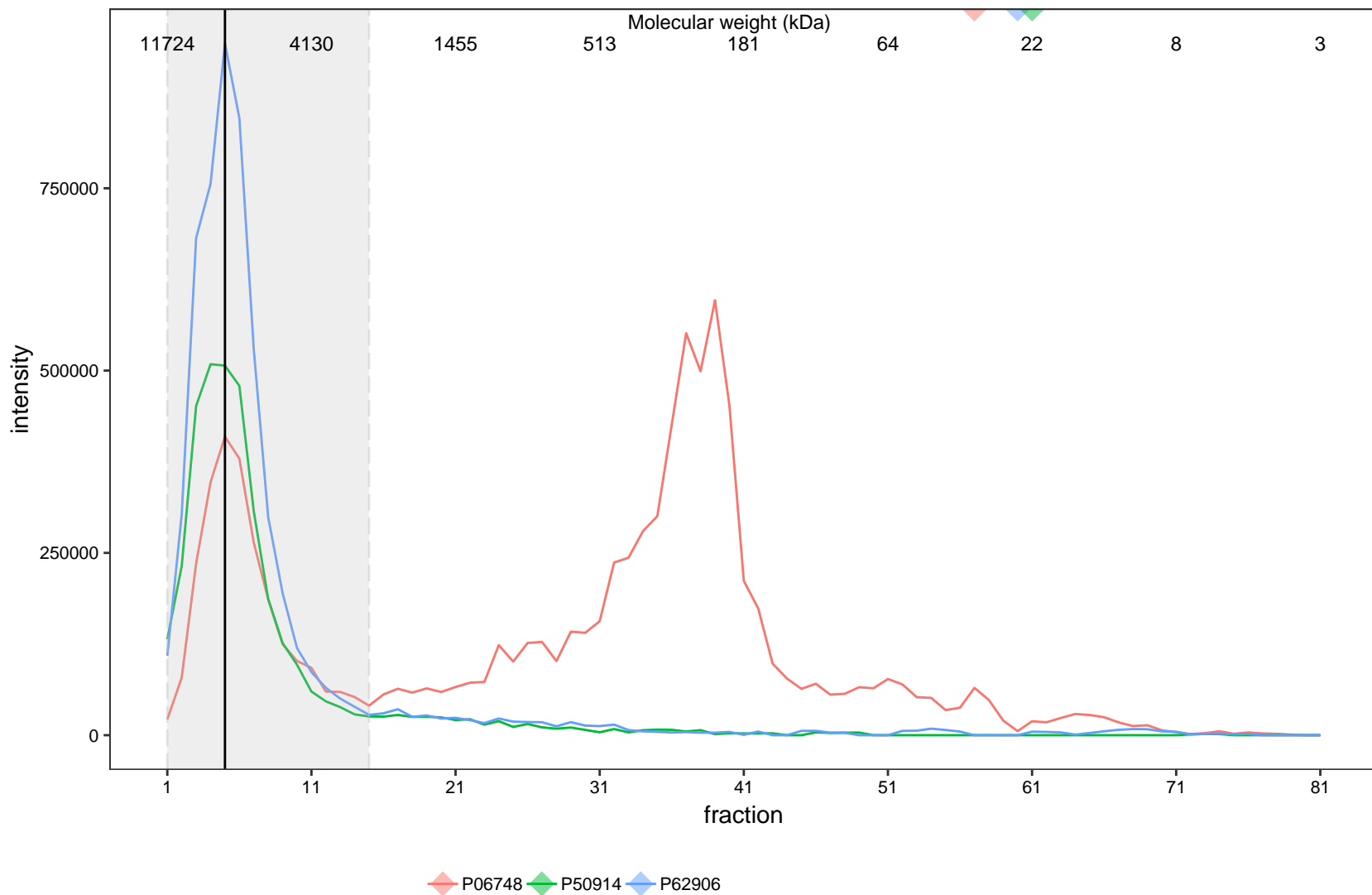

Supplement: Supplementary file 7 — Dataset EV6 [file MSB-15-e8438-s007.zip › feature_plots_bioplex/O60287.pdf]

**O60344**

**Annotated subunits: 6 Subunits with signal: 3**

**Max. coeluting subunits: 2 Max. completeness: 0.33**

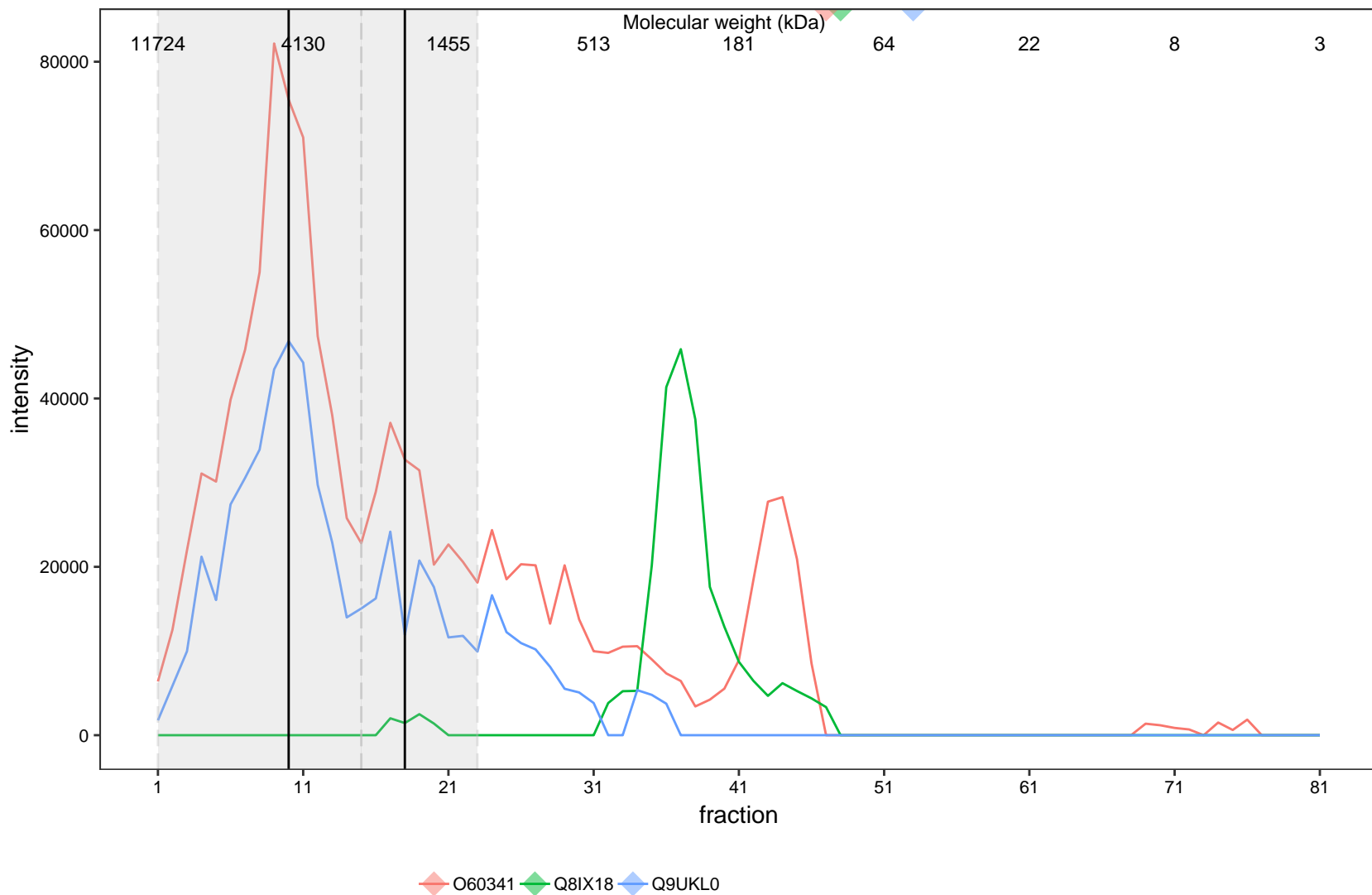

Supplement: Supplementary file 7 — Dataset EV6 [file MSB-15-e8438-s007.zip › feature_plots_bioplex/O60344.pdf]

O60478

Annotated subunits: 7 Subunits with signal: 5

Max. coeluting subunits: 4 Max. completeness: 0.57

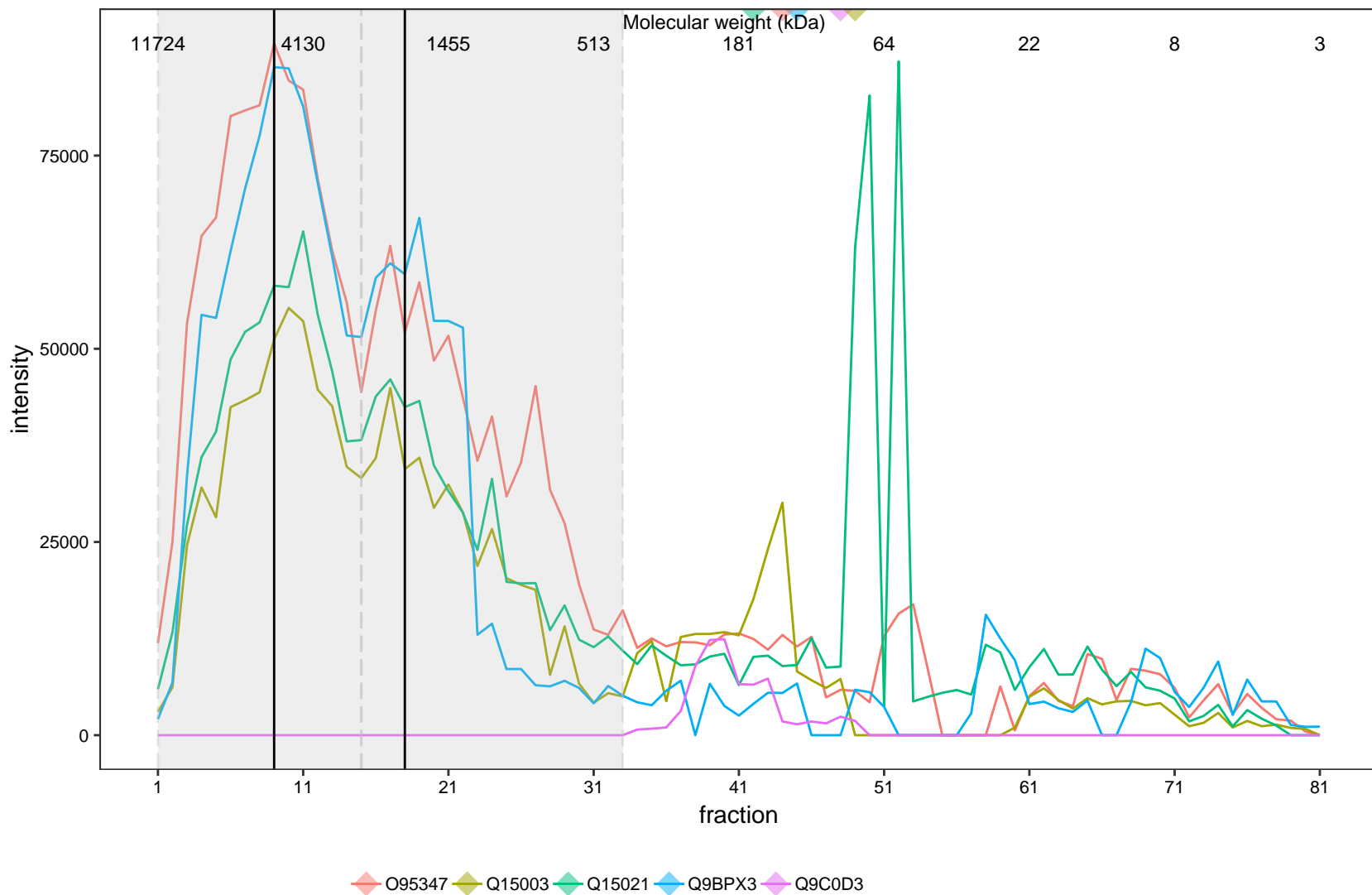

Supplement: Supplementary file 7 — Dataset EV6 [file MSB-15-e8438-s007.zip › feature_plots_bioplex/O60478.pdf]

**O60499**

**Annotated subunits: 9 Subunits with signal: 6**

**Max. coeluting subunits: 4 Max. completeness: 0.44**

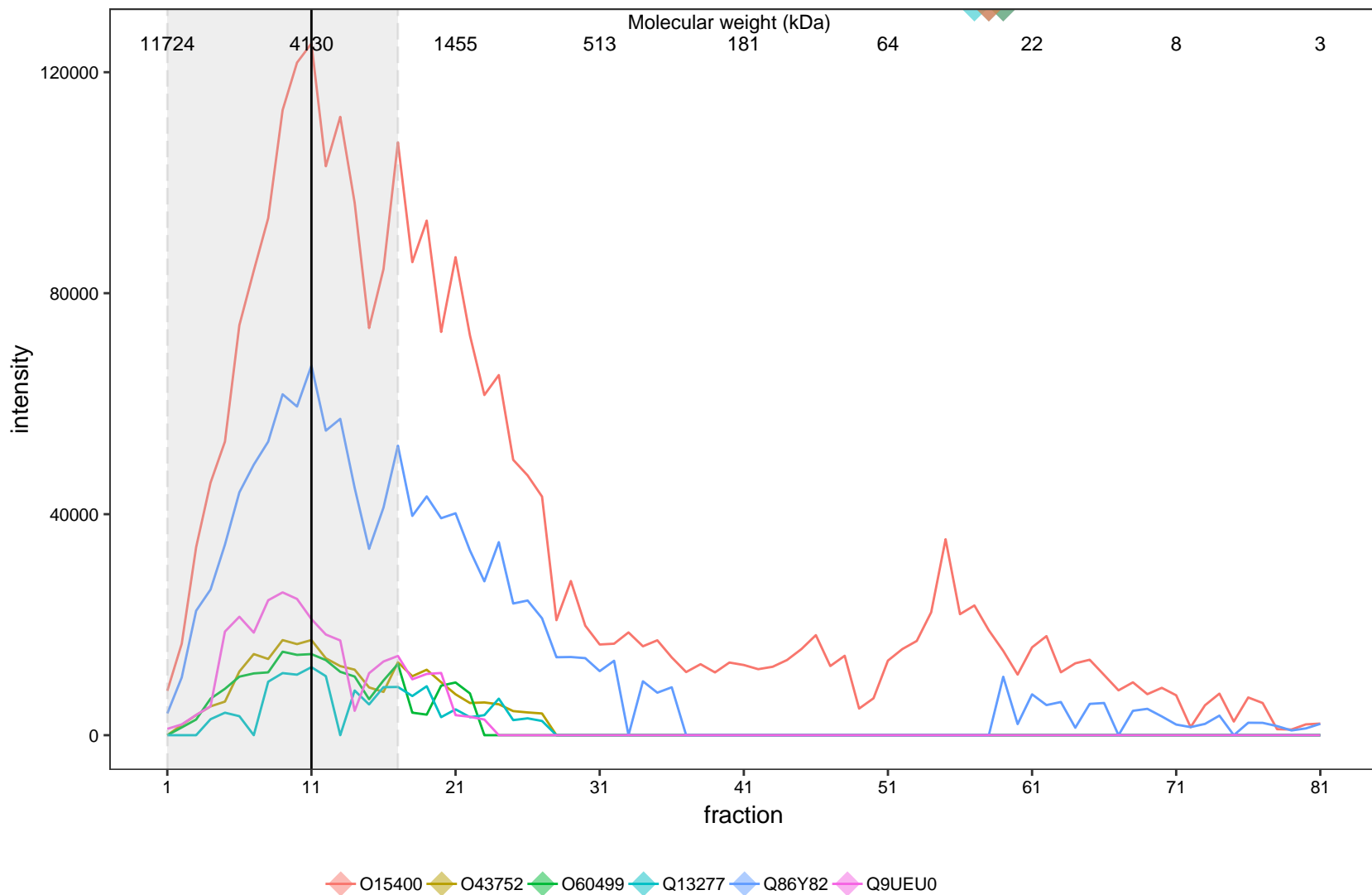

Supplement: Supplementary file 7 — Dataset EV6 [file MSB-15-e8438-s007.zip › feature_plots_bioplex/O60499.pdf]

O60512  
Annotated subunits: 22   Subunits with signal: 8  
Max. coeluting subunits: 6   Max. completeness: 0.27

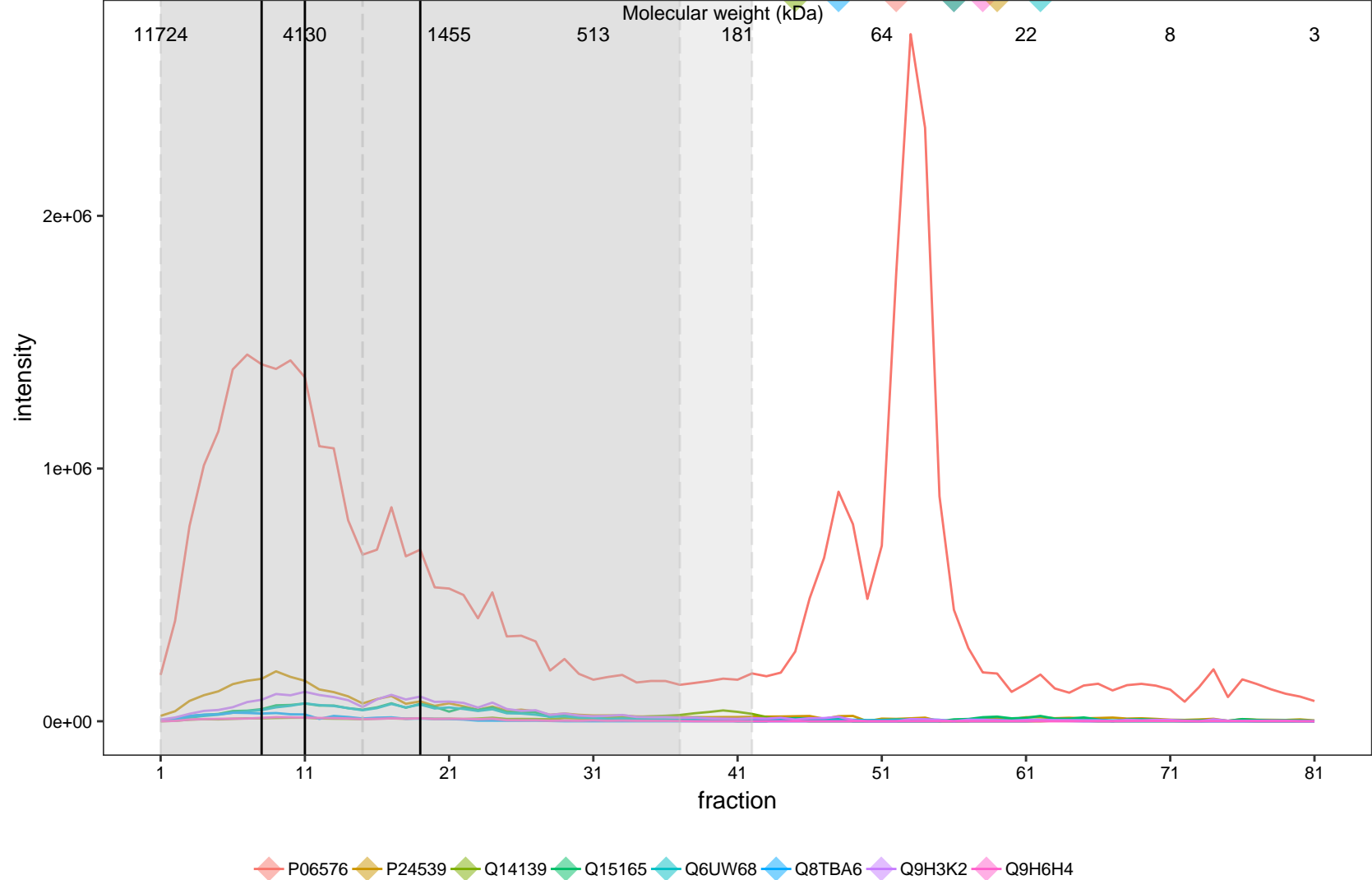

Supplement: Supplementary file 7 — Dataset EV6 [file MSB-15-e8438-s007.zip › feature_plots_bioplex/O60512.pdf]

**O60563**

**Annotated subunits: 9 Subunits with signal: 5**

**Max. coeluting subunits: 4 Max. completeness: 0.44**

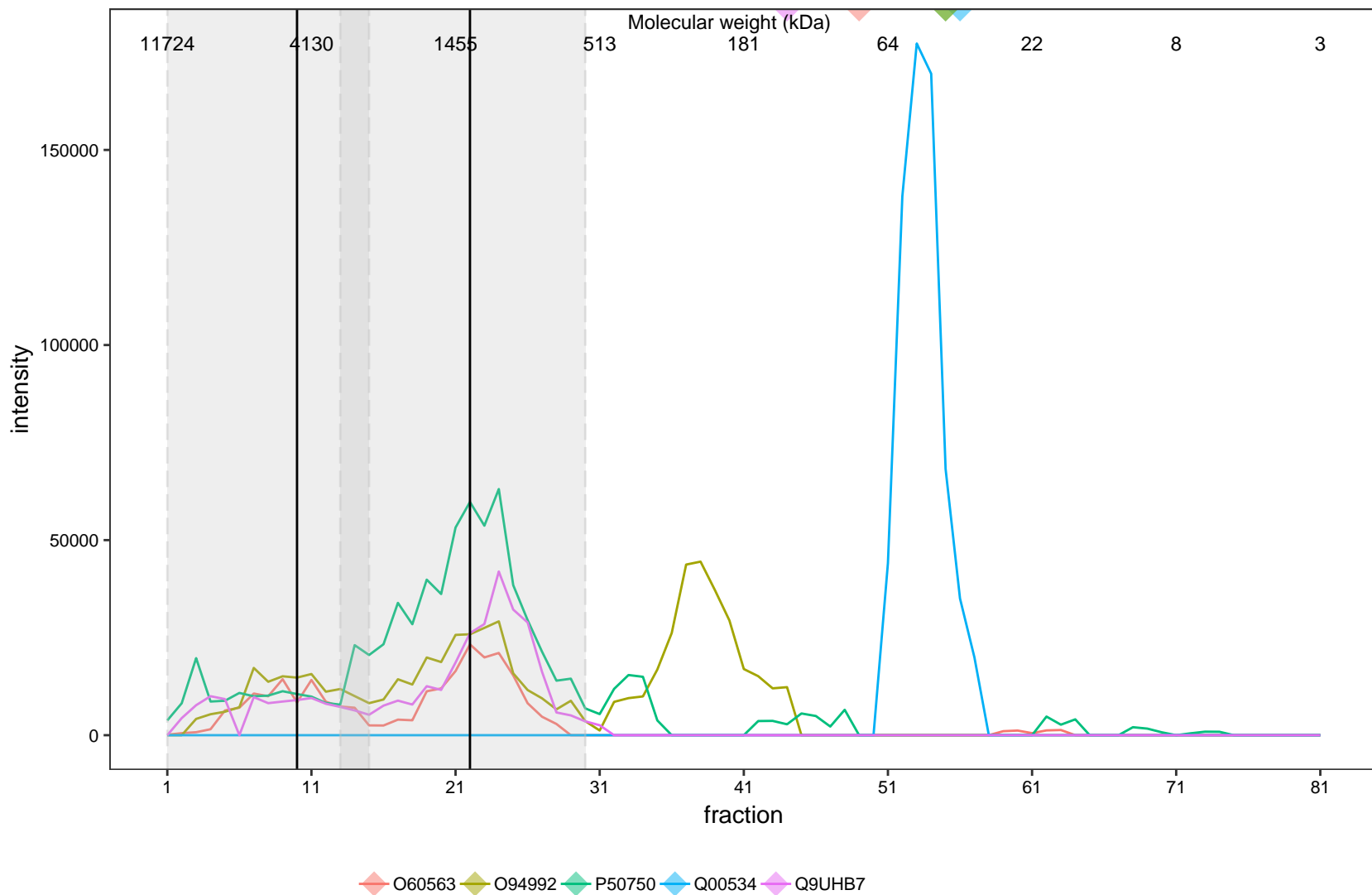

Supplement: Supplementary file 7 — Dataset EV6 [file MSB-15-e8438-s007.zip › feature_plots_bioplex/O60563.pdf]

O60645  
Annotated subunits: 3 Subunits with signal: 3  
Max. coeluting subunits: 2 Max. completeness: 0.67

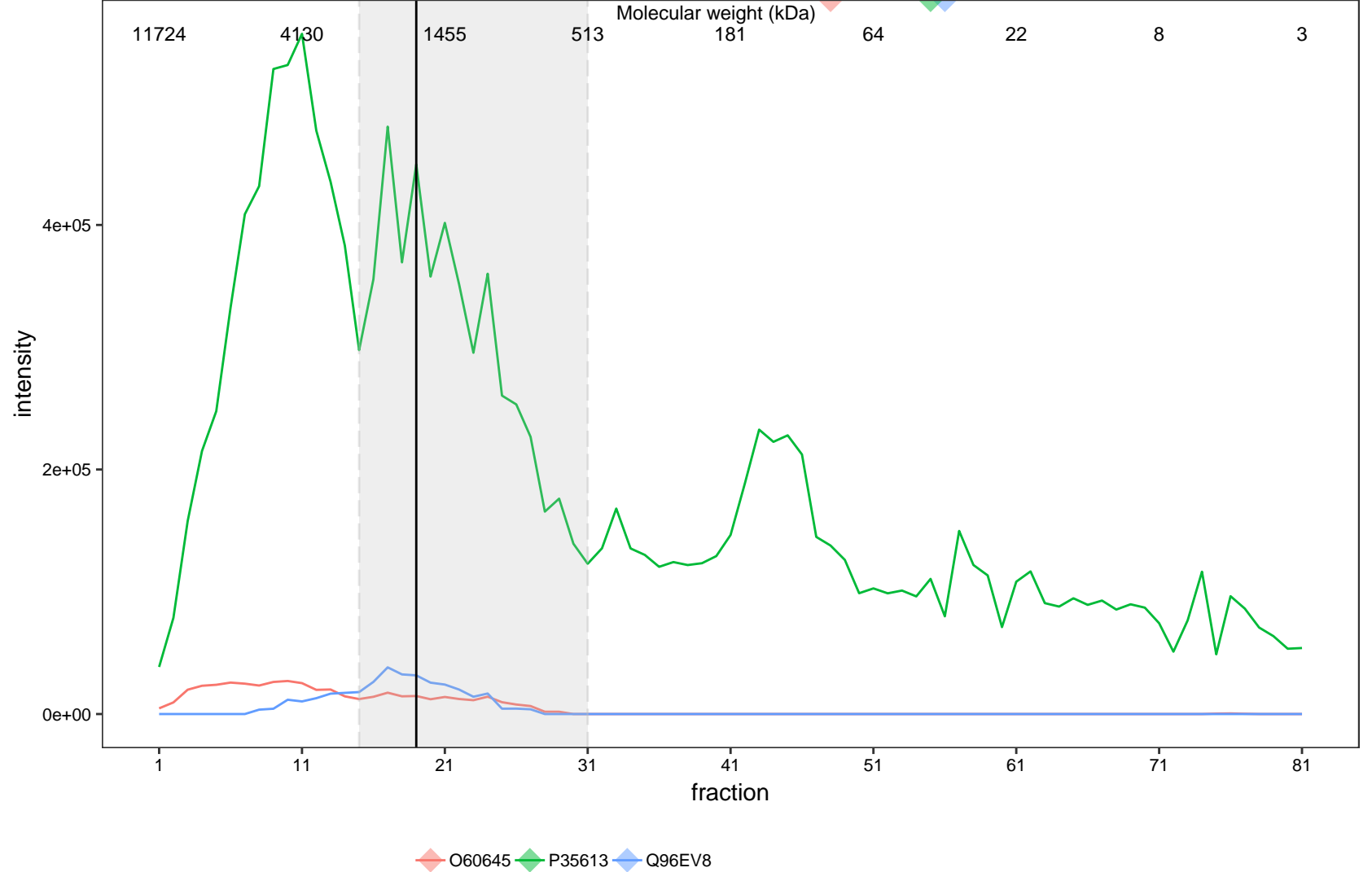

Supplement: Supplementary file 7 — Dataset EV6 [file MSB-15-e8438-s007.zip › feature_plots_bioplex/O60645.pdf]

**O60783**

**Annotated subunits: 9   Subunits with signal: 5**

**Max. coeluting subunits: 3   Max. completeness: 0.33**

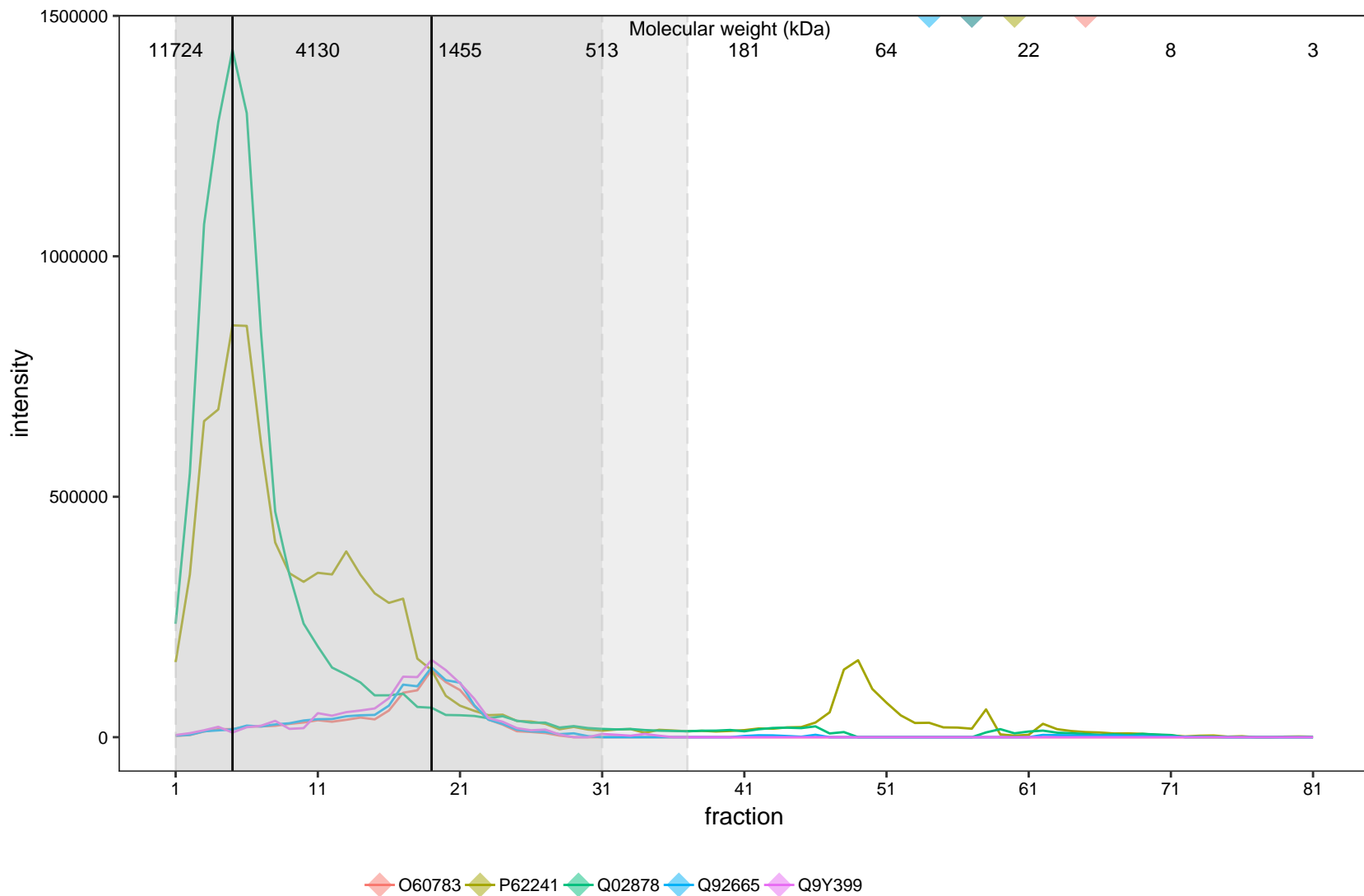

Supplement: Supplementary file 7 — Dataset EV6 [file MSB-15-e8438-s007.zip › feature_plots_bioplex/O60783.pdf]

**O60826**

**Annotated subunits: 9 Subunits with signal: 6**

**Max. coeluting subunits: 3 Max. completeness: 0.33**

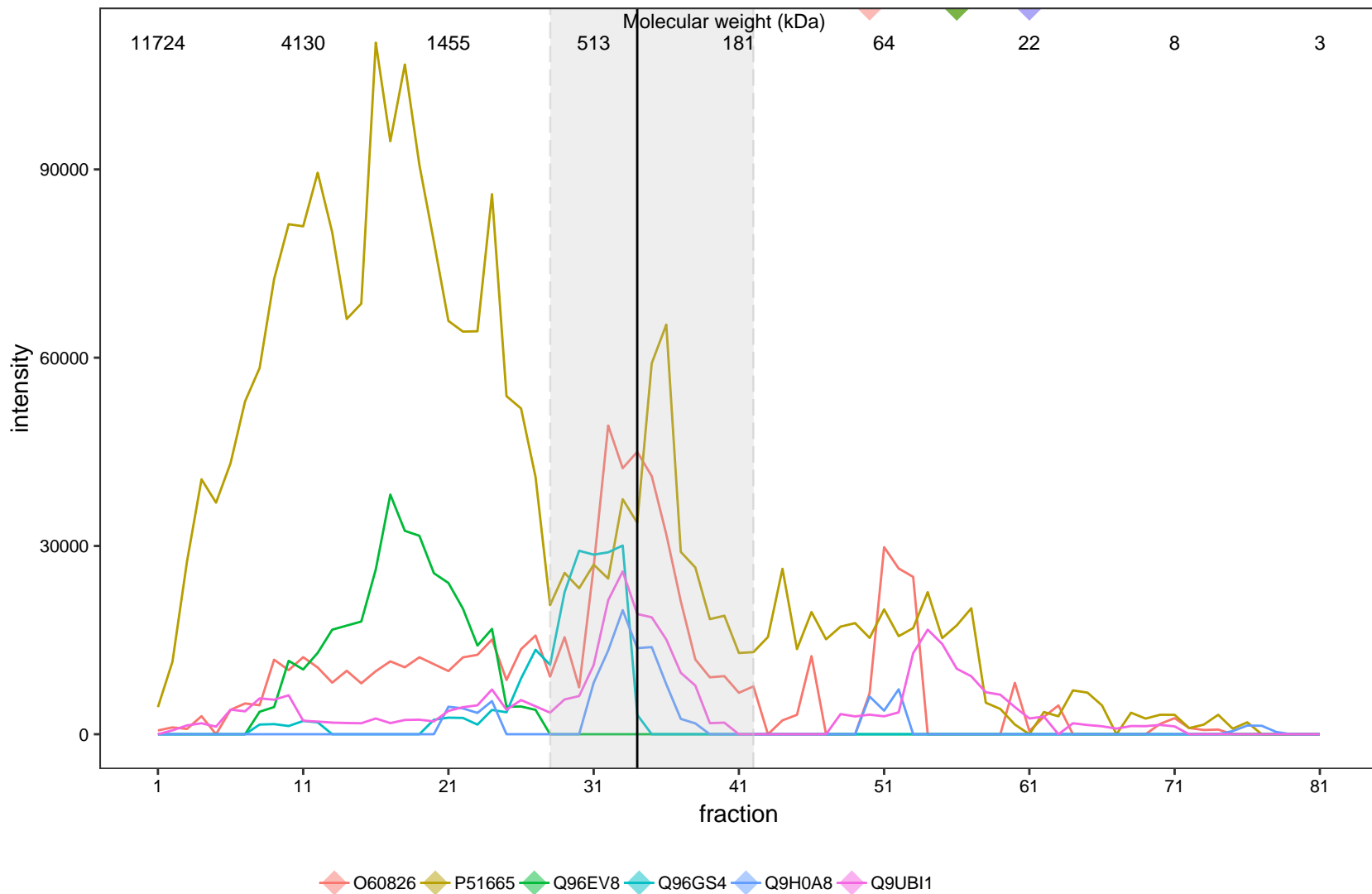

Supplement: Supplementary file 7 — Dataset EV6 [file MSB-15-e8438-s007.zip › feature_plots_bioplex/O60826.pdf]

O60831  
Annotated subunits: 10   Subunits with signal: 5  
Max. coeluting subunits: 4   Max. completeness: 0.4

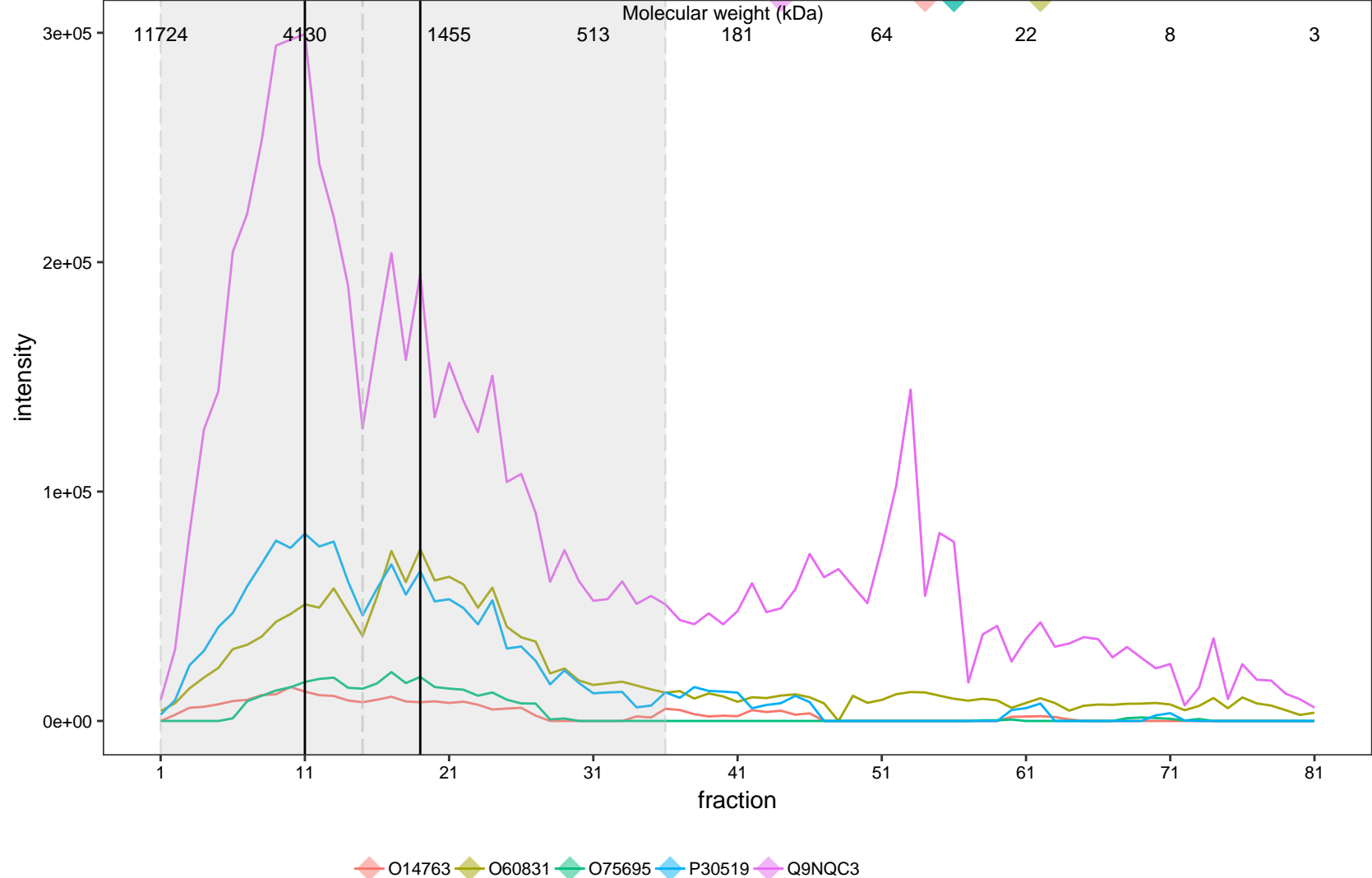

Supplement: Supplementary file 7 — Dataset EV6 [file MSB-15-e8438-s007.zip › feature_plots_bioplex/O60831.pdf]

**O75155**  
Annotated subunits: 10   Subunits with signal: 5  
Max. coeluting subunits: 3   Max. completeness: 0.3

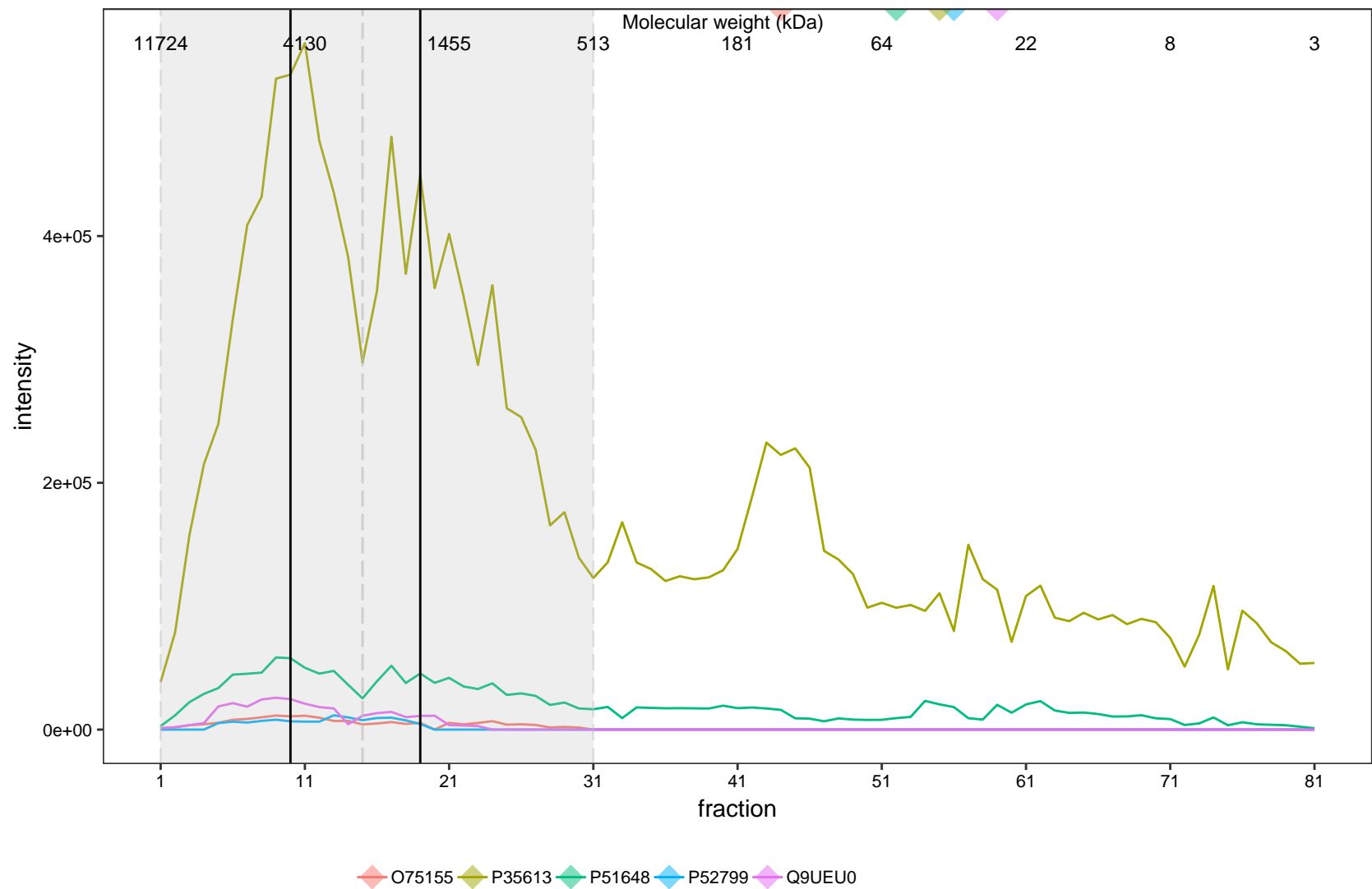

Supplement: Supplementary file 7 — Dataset EV6 [file MSB-15-e8438-s007.zip › feature_plots_bioplex/O75155.pdf]

**O75177**  
**Annotated subunits: 5   Subunits with signal: 3**  
**Max. coeluting subunits: 3   Max. completeness: 0.6**

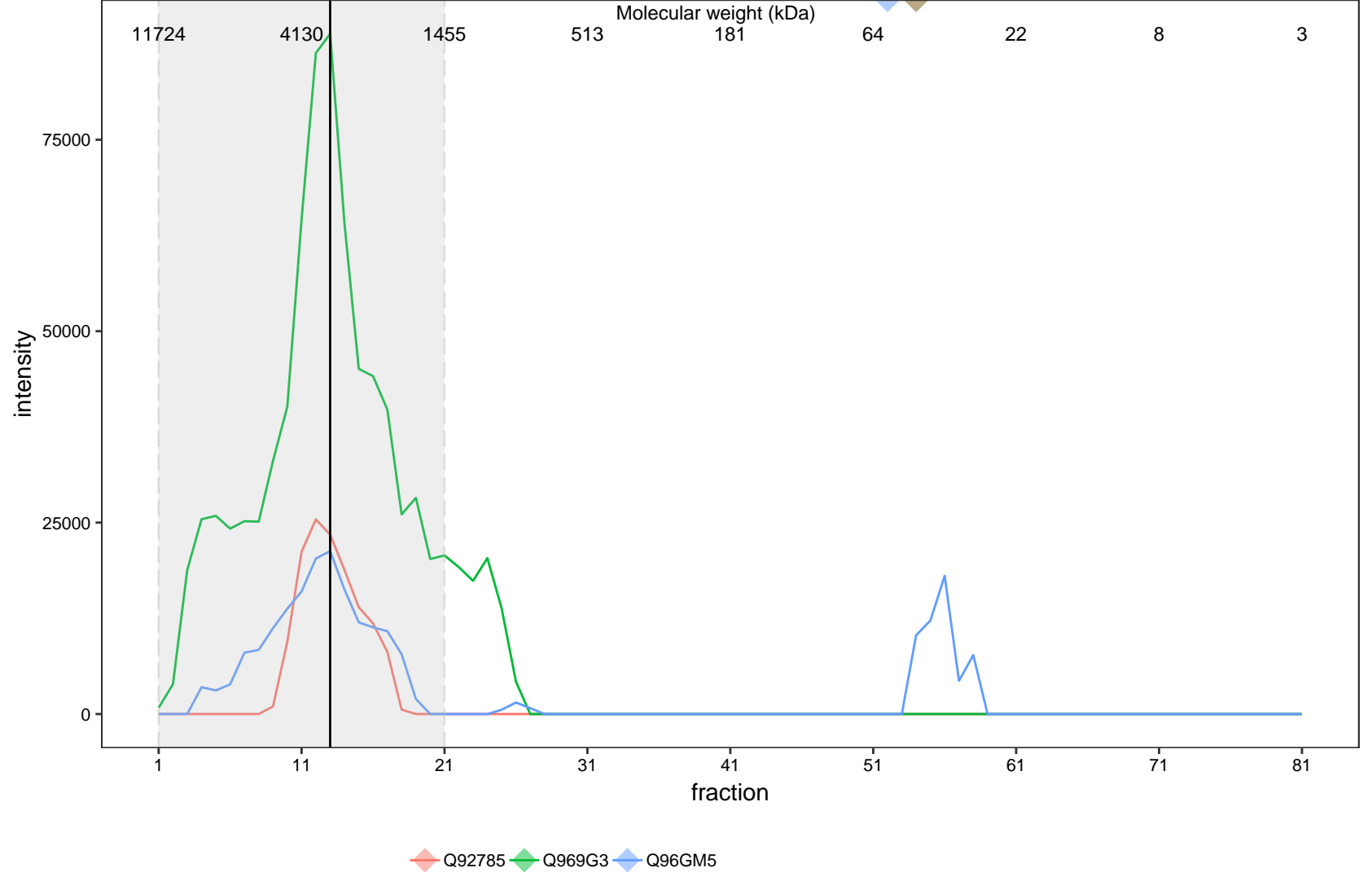

Supplement: Supplementary file 7 — Dataset EV6 [file MSB-15-e8438-s007.zip › feature_plots_bioplex/O75177.pdf]

**O75251**

**Annotated subunits: 3 Subunits with signal: 3**

**Max. coeluting subunits: 3 Max. completeness: 1**

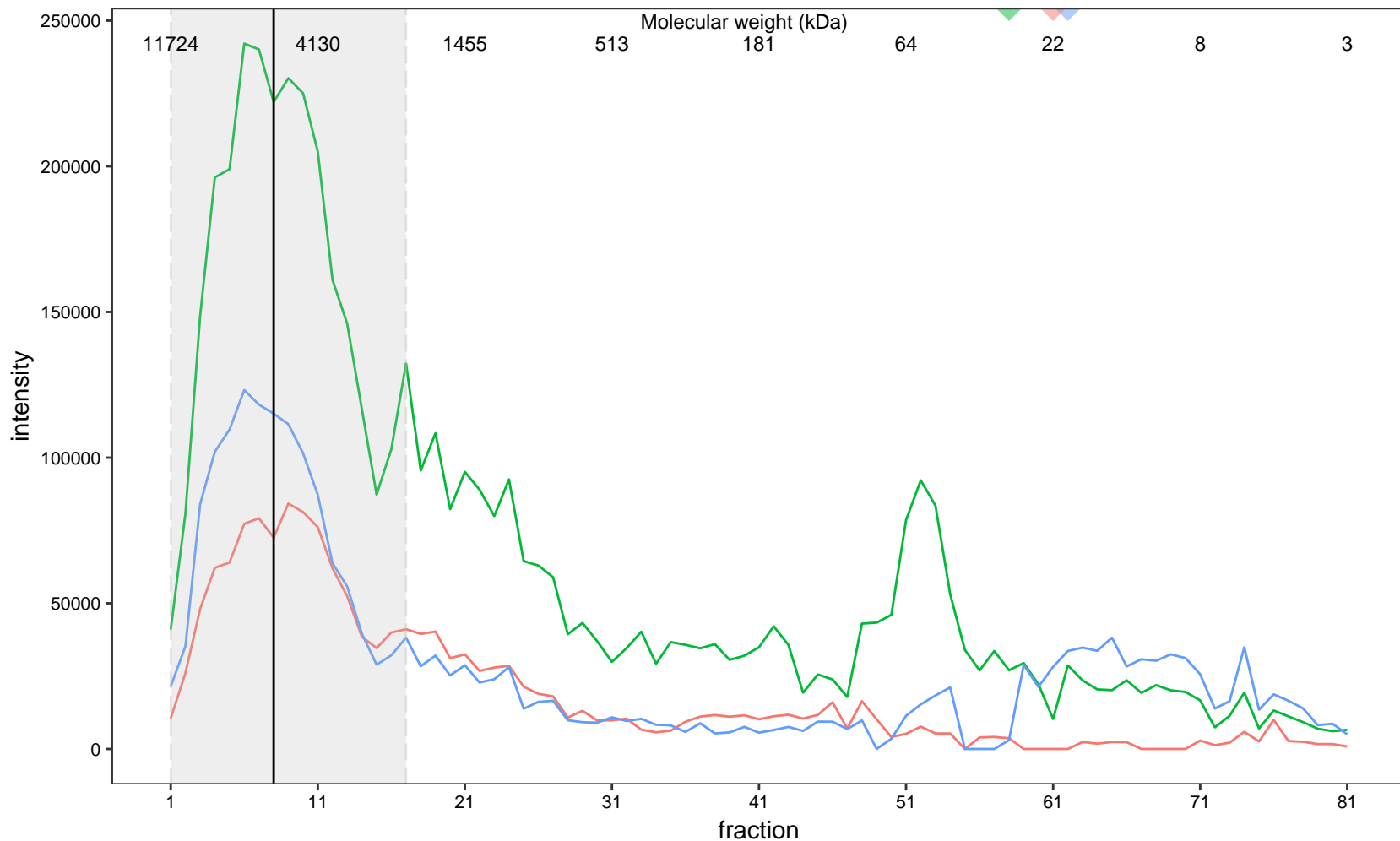

◊ O75251 ◊ O75489 ◊ P51970

Supplement: Supplementary file 7 — Dataset EV6 [file MSB-15-e8438-s007.zip › feature_plots_bioplex/O75251.pdf]

**O75306**

**Annotated subunits: 4 Subunits with signal: 4**

**Max. coeluting subunits: 4 Max. completeness: 1**

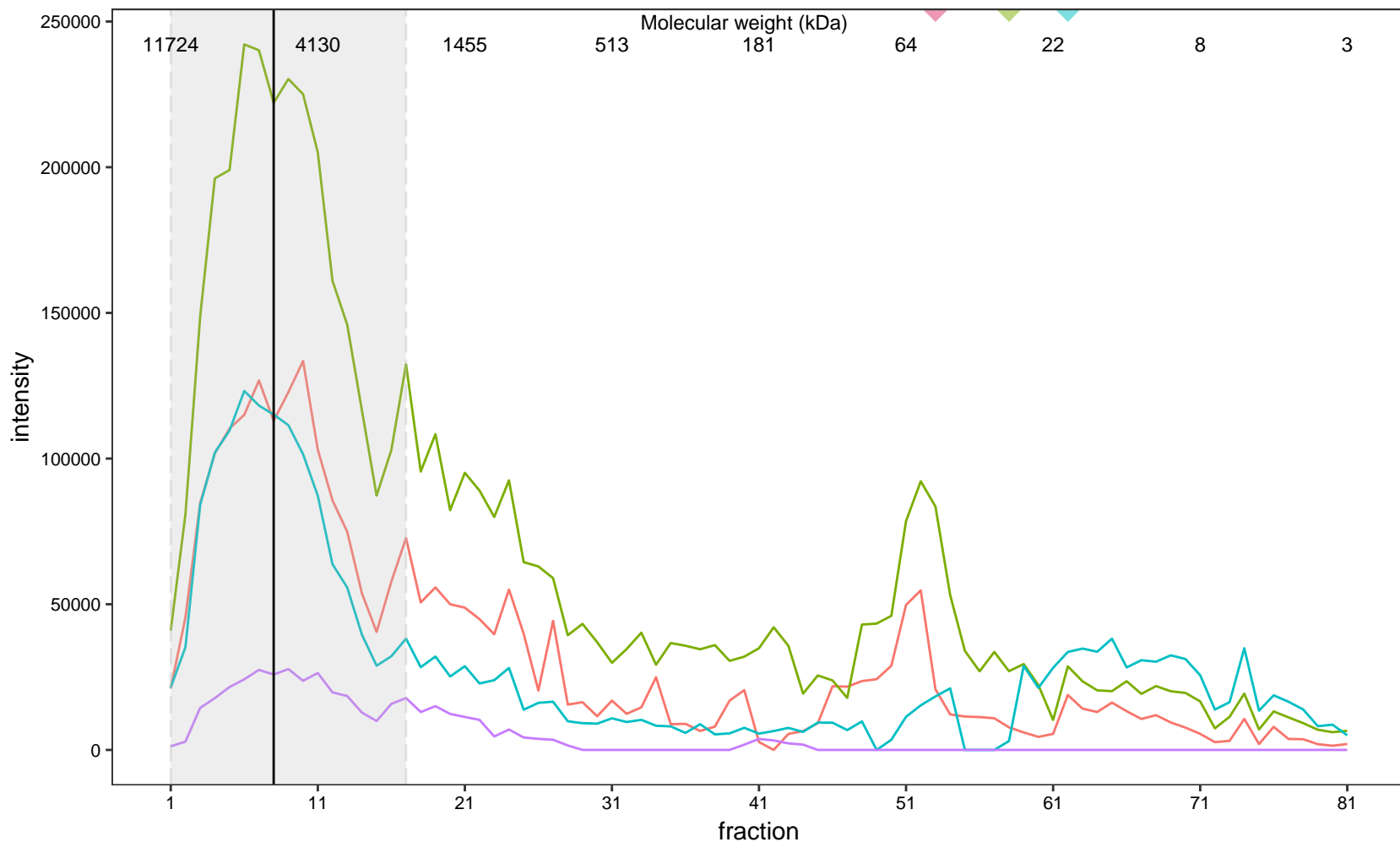

◊ O75306 ◊ O75489 ◊ P51970 ◊ Q9BQ95

Supplement: Supplementary file 7 — Dataset EV6 [file MSB-15-e8438-s007.zip › feature_plots_bioplex/O75306.pdf]

**O75379**

**Annotated subunits: 7 Subunits with signal: 5**

**Max. coeluting subunits: 4 Max. completeness: 0.57**

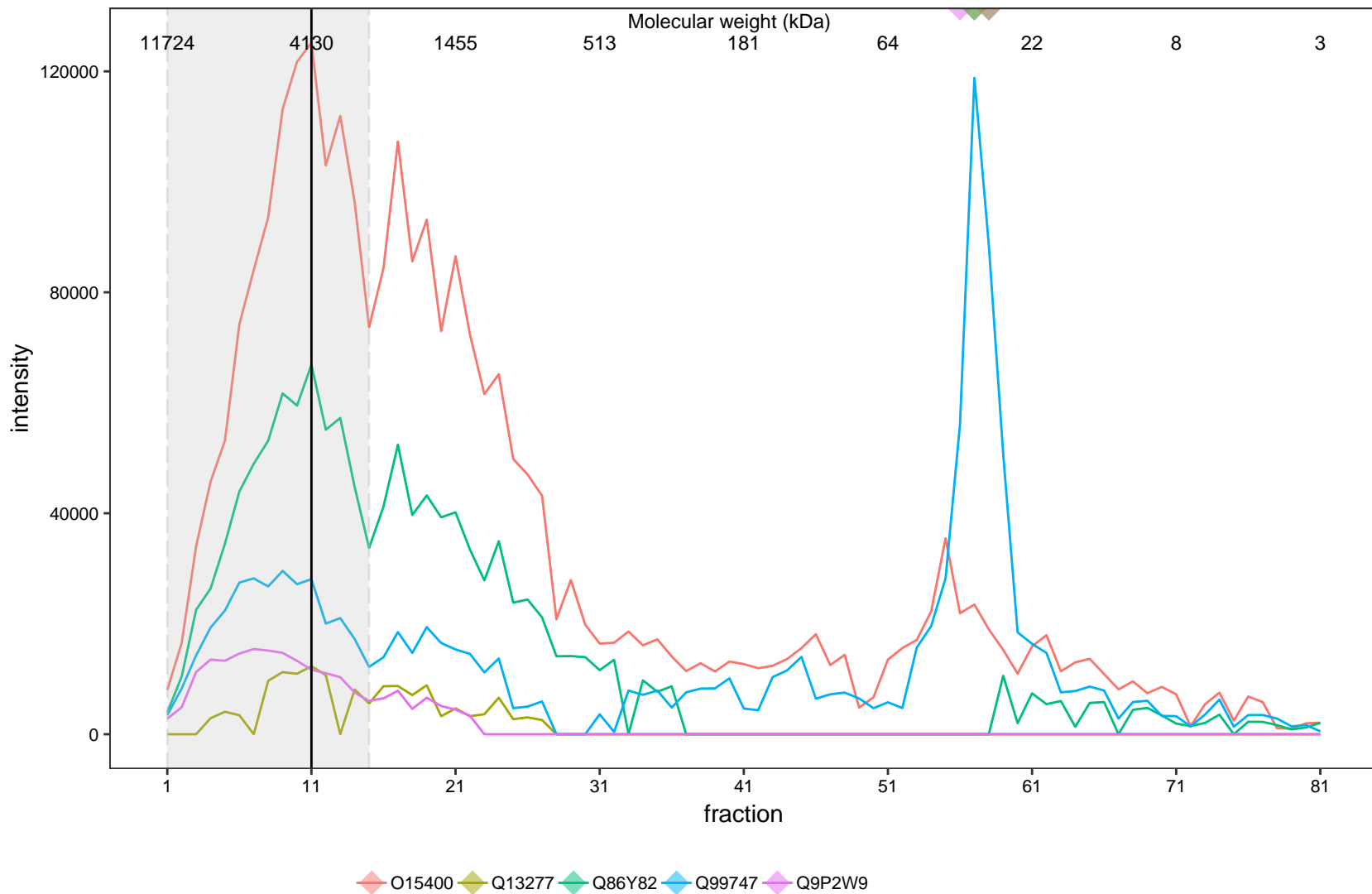

Supplement: Supplementary file 7 — Dataset EV6 [file MSB-15-e8438-s007.zip › feature_plots_bioplex/O75379.pdf]

**O75380**

**Annotated subunits: 9 Subunits with signal: 6**

**Max. coeluting subunits: 3 Max. completeness: 0.33**

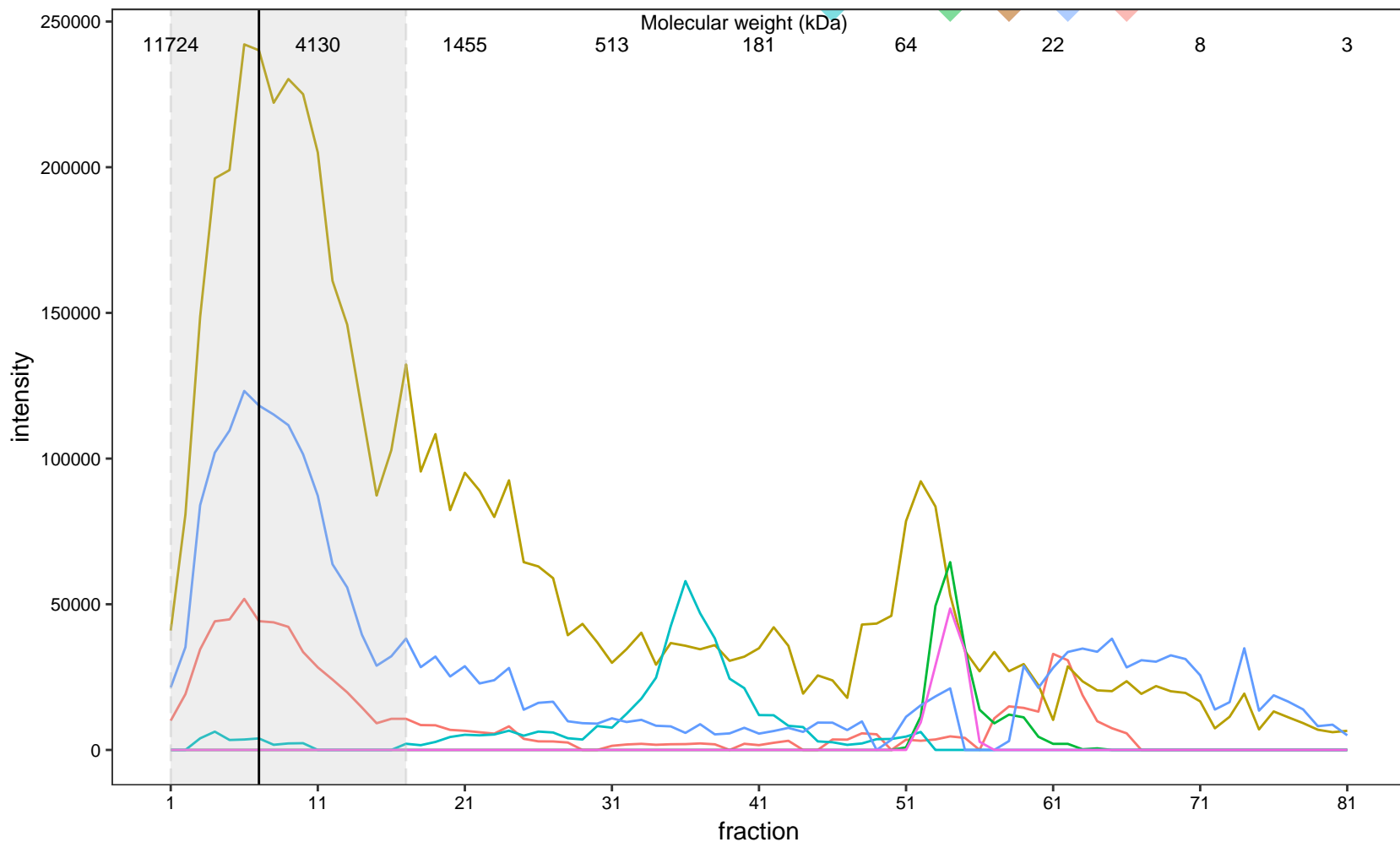

◊ O75380 ◊ O75489 ◊ O75600 ◊ P49916 ◊ P51970 ◊ Q96EF6

Supplement: Supplementary file 7 — Dataset EV6 [file MSB-15-e8438-s007.zip › feature_plots_bioplex/O75380.pdf]

**O75394**

**Annotated subunits: 4 Subunits with signal: 3**

**Max. coeluting subunits: 3 Max. completeness: 0.75**

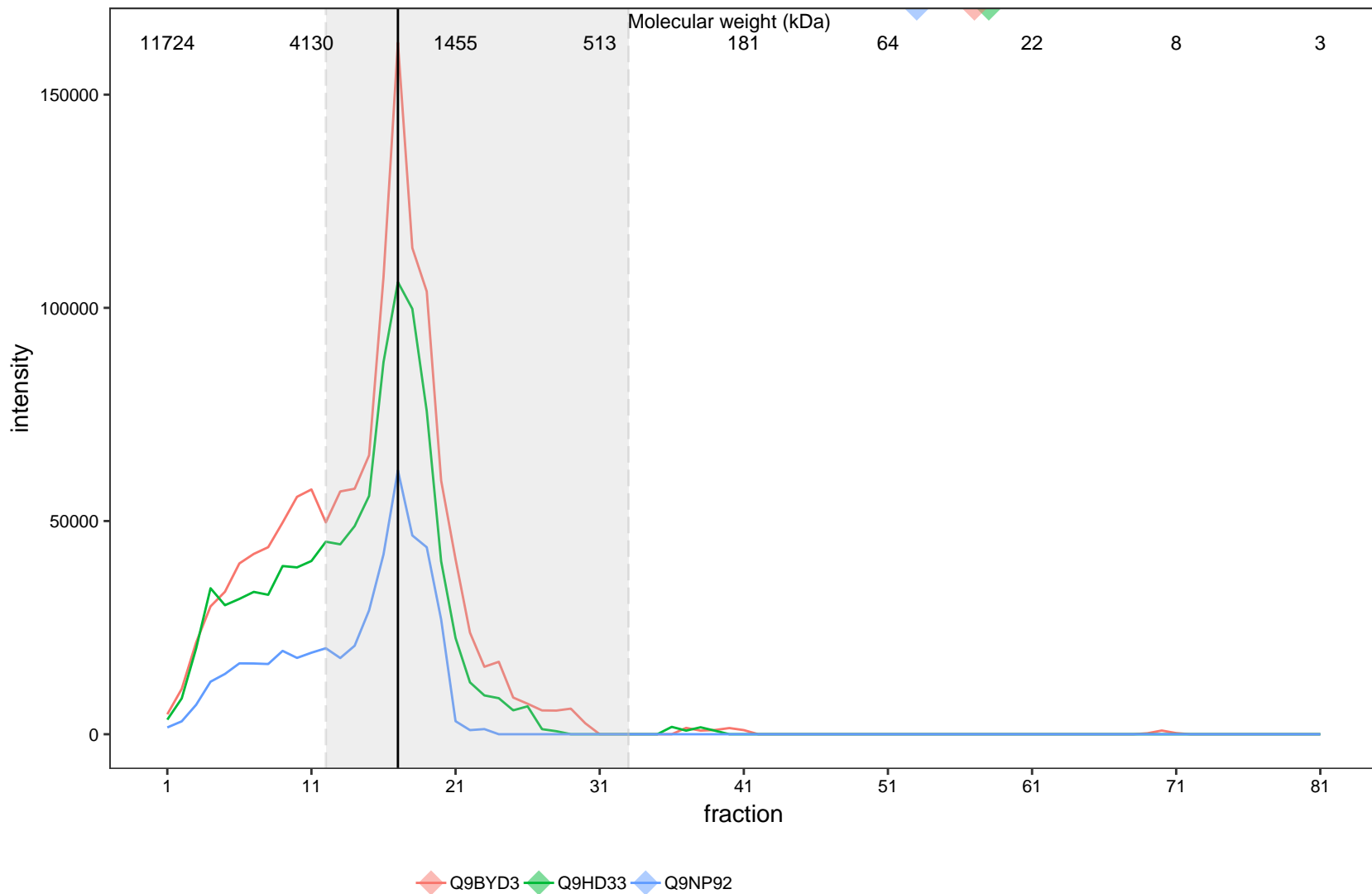

Supplement: Supplementary file 7 — Dataset EV6 [file MSB-15-e8438-s007.zip › feature_plots_bioplex/O75394.pdf]

**O75410**

**Annotated subunits: 3 Subunits with signal: 3**

**Max. coeluting subunits: 2 Max. completeness: 0.67**

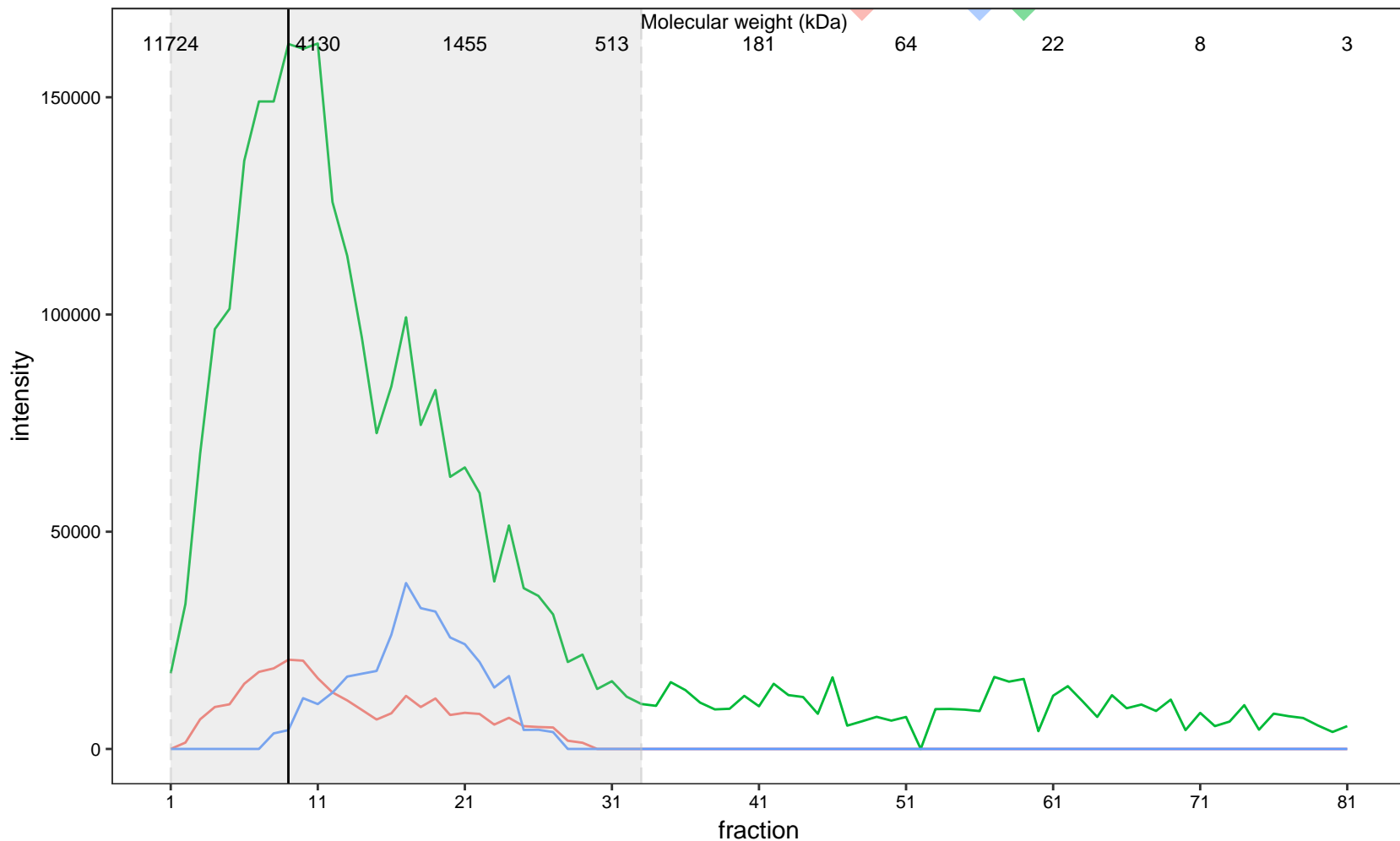

Supplement: Supplementary file 7 — Dataset EV6 [file MSB-15-e8438-s007.zip › feature_plots_bioplex/O75410.pdf]

**O75438**

**Annotated subunits: 4 Subunits with signal: 4**

**Max. coeluting subunits: 4 Max. completeness: 1**

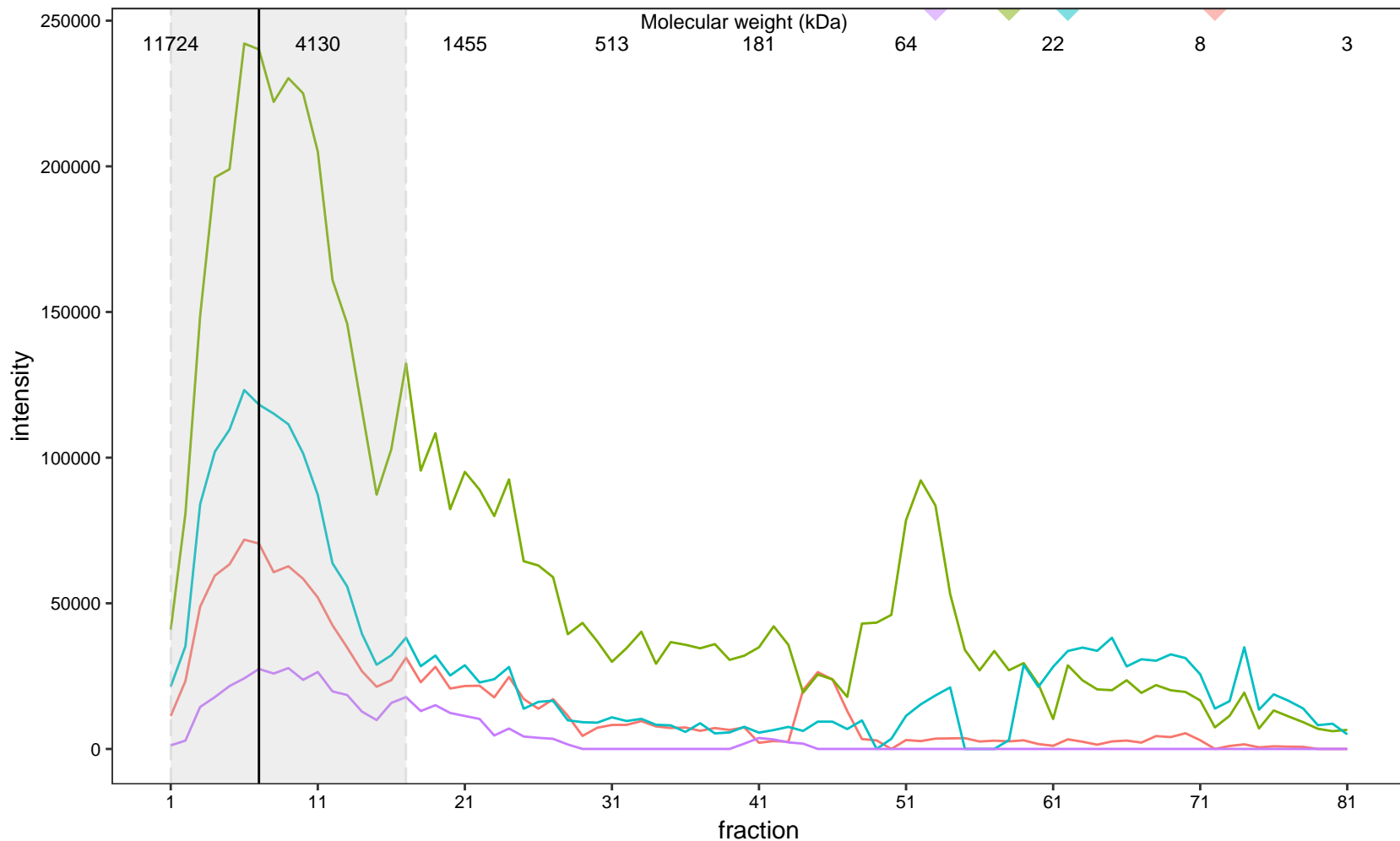

◈ O75438 ◈ O75489 ◈ P51970 ◈ Q9BQ95

Supplement: Supplementary file 7 — Dataset EV6 [file MSB-15-e8438-s007.zip › feature_plots_bioplex/O75438.pdf]

**O75446**  
**Annotated subunits: 2   Subunits with signal: 2**  
**Max. coeluting subunits: 2   Max. completeness: 1**

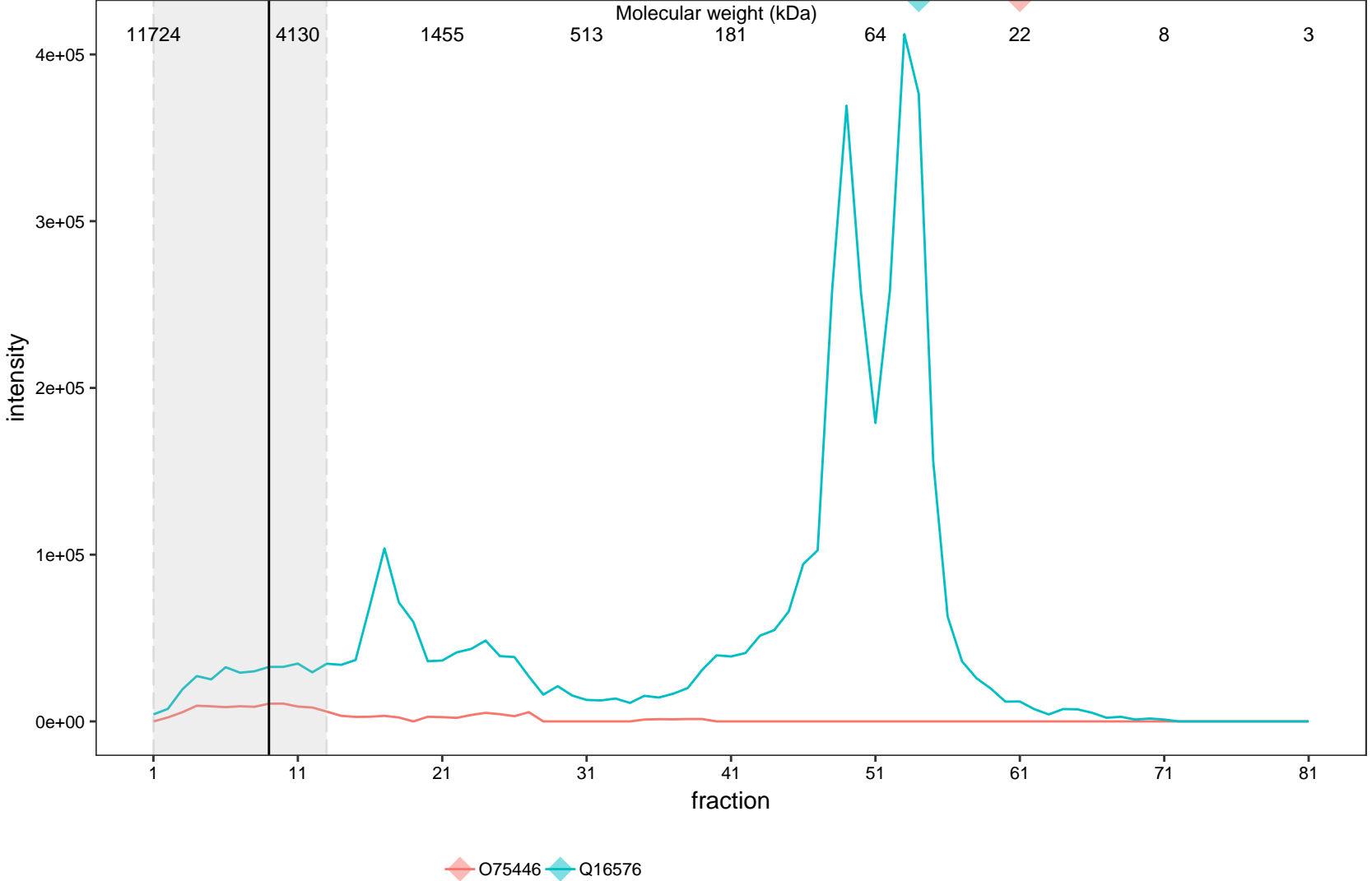

Supplement: Supplementary file 7 — Dataset EV6 [file MSB-15-e8438-s007.zip › feature_plots_bioplex/O75446.pdf]

**O75467**

**Annotated subunits: 9   Subunits with signal: 3**

**Max. coeluting subunits: 2   Max. completeness: 0.22**

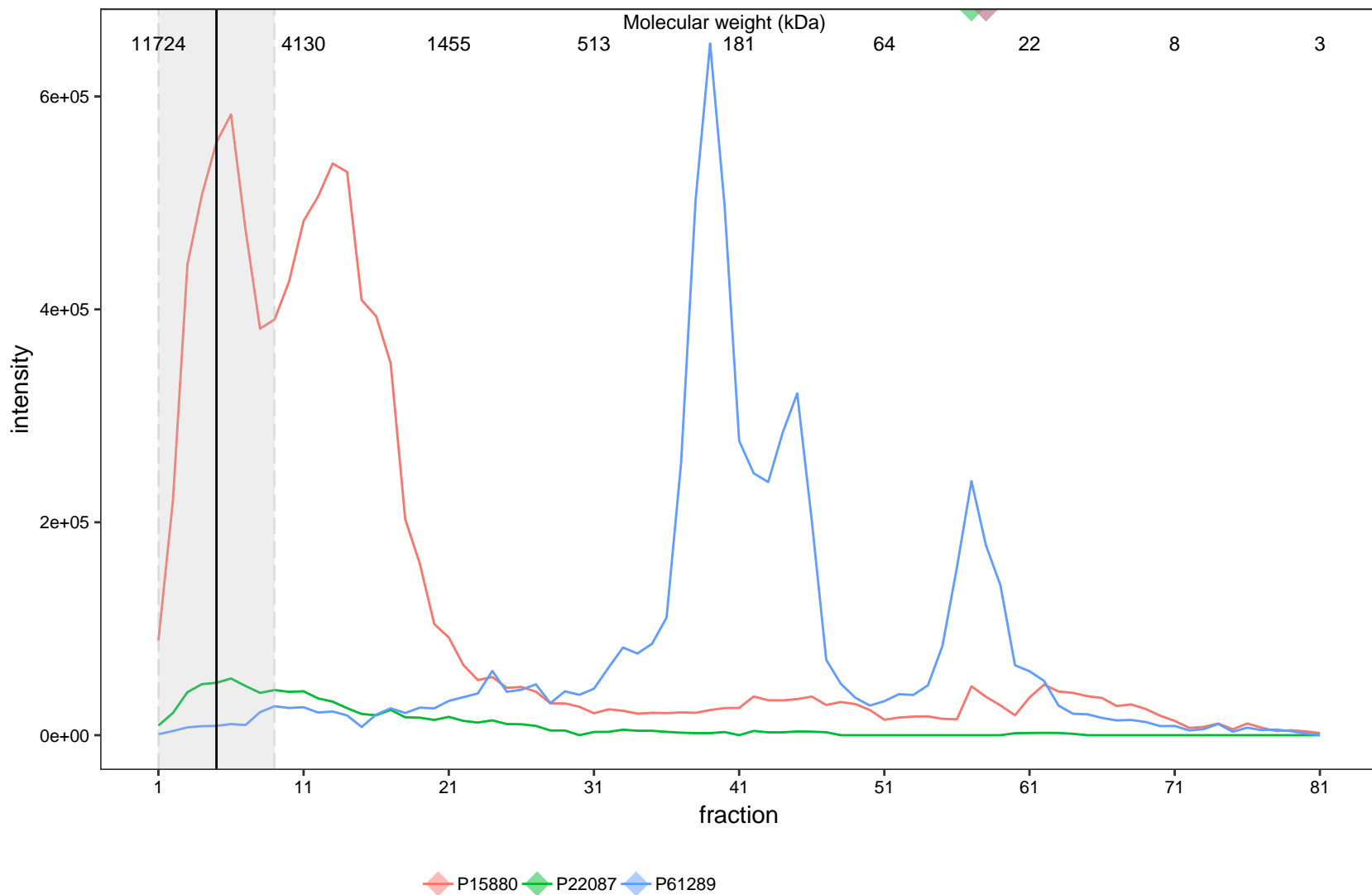

Supplement: Supplementary file 7 — Dataset EV6 [file MSB-15-e8438-s007.zip › feature_plots_bioplex/O75467.pdf]

O75533  
Annotated subunits: 10   Subunits with signal: 6  
Max. coeluting subunits: 2   Max. completeness: 0.2

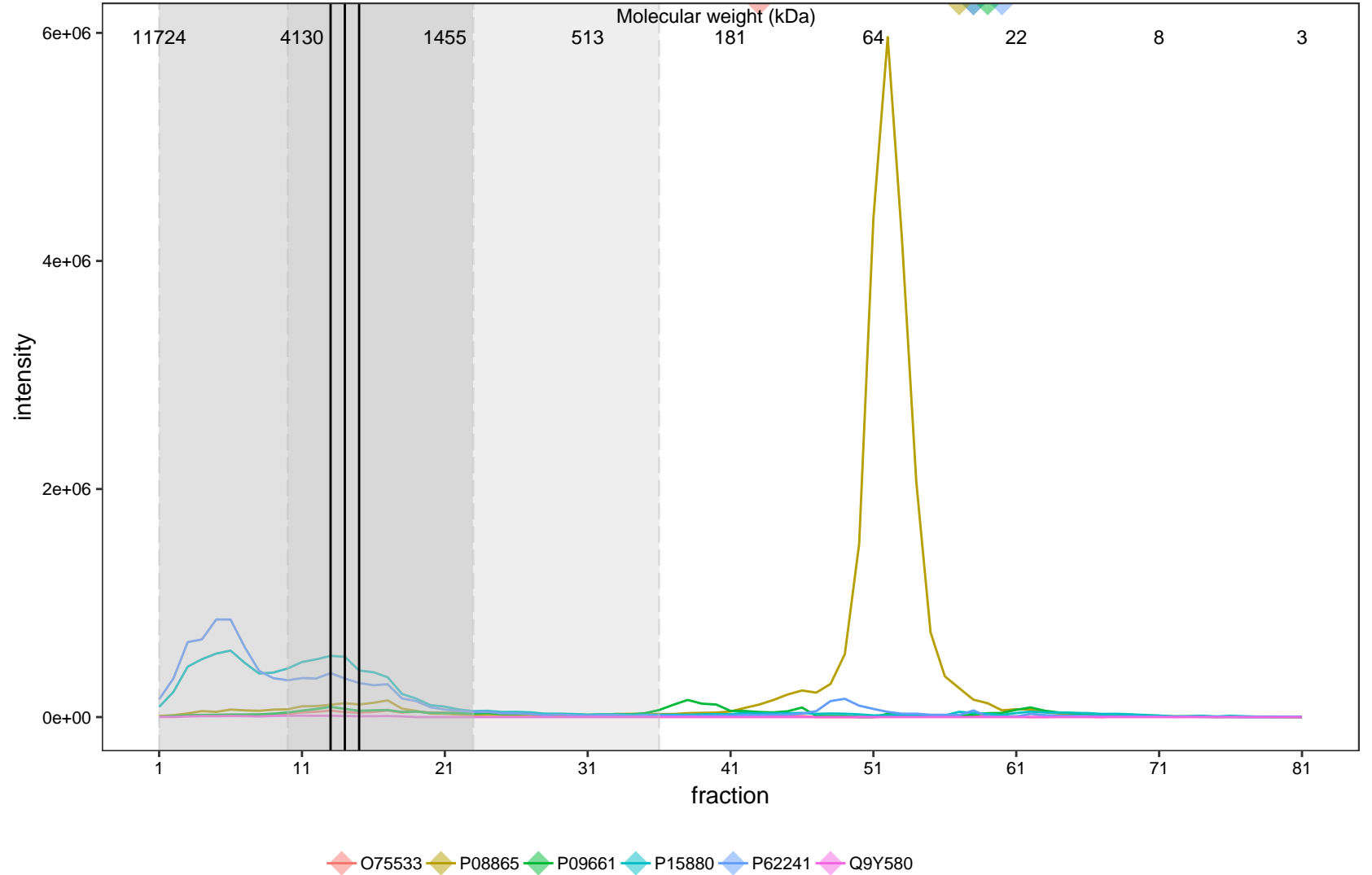

Supplement: Supplementary file 7 — Dataset EV6 [file MSB-15-e8438-s007.zip › feature_plots_bioplex/O75533.pdf]

**O75607**  
Annotated subunits: 2   Subunits with signal: 2  
Max. coeluting subunits: 2   Max. completeness: 1

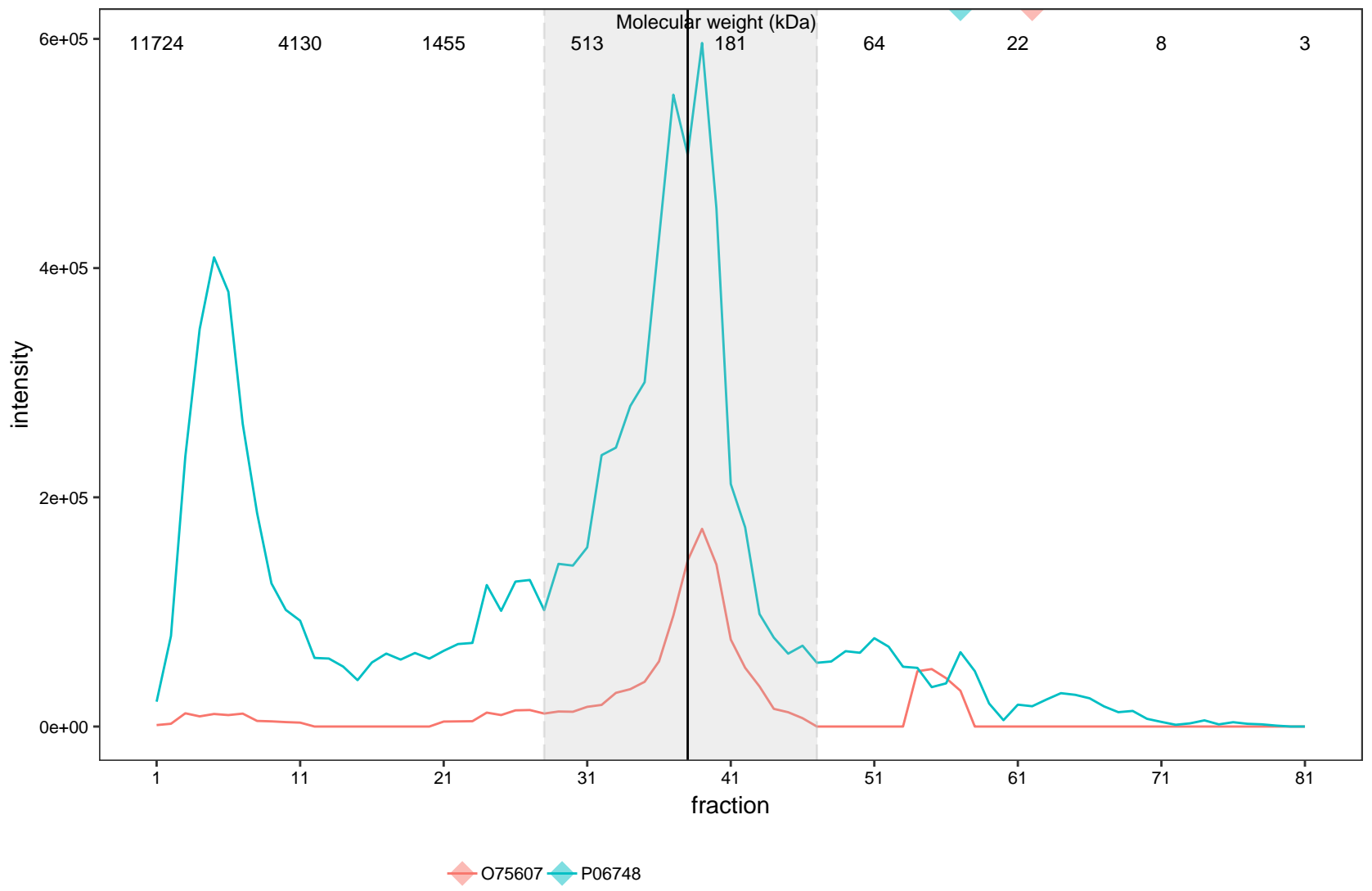

Supplement: Supplementary file 7 — Dataset EV6 [file MSB-15-e8438-s007.zip › feature_plots_bioplex/O75607.pdf]

**O75683**

**Annotated subunits: 9 Subunits with signal: 5**

**Max. coeluting subunits: 4 Max. completeness: 0.44**

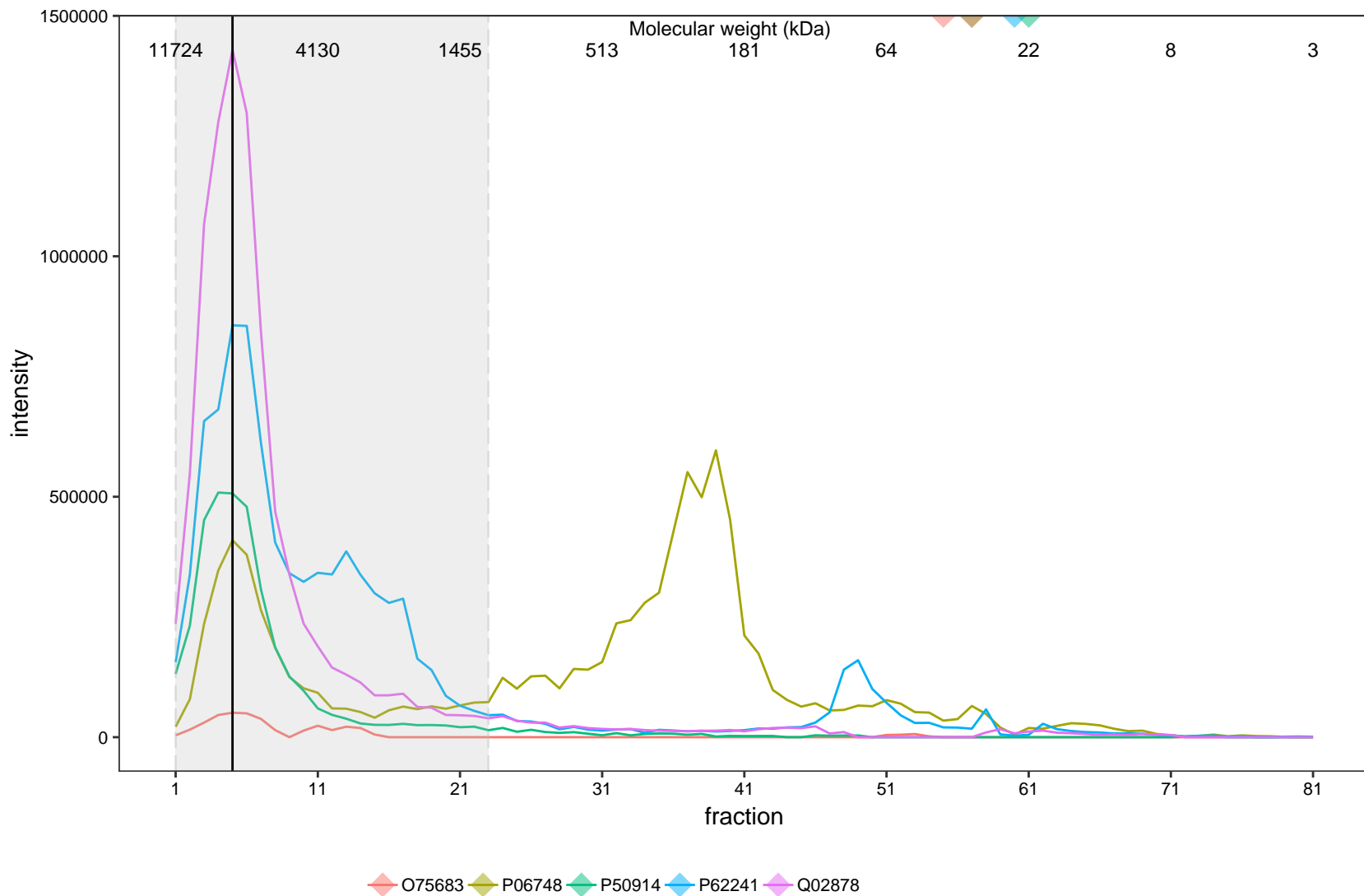

Supplement: Supplementary file 7 — Dataset EV6 [file MSB-15-e8438-s007.zip › feature_plots_bioplex/O75683.pdf]
