# Supplementary material for: Complex‐centric proteome profiling by SEC‐SWATH‐MS
Source: Mol Syst Biol. 2019 Jan 14;15(1):e8438. doi: 10.15252/msb.20188438 (PMC6346213; doi:10.15252/msb.20188438)
Supplement: Supplementary file 7 — Dataset EV6 [file MSB-15-e8438-s007.zip › feature_plots_bioplex/A2RU67.pdf]

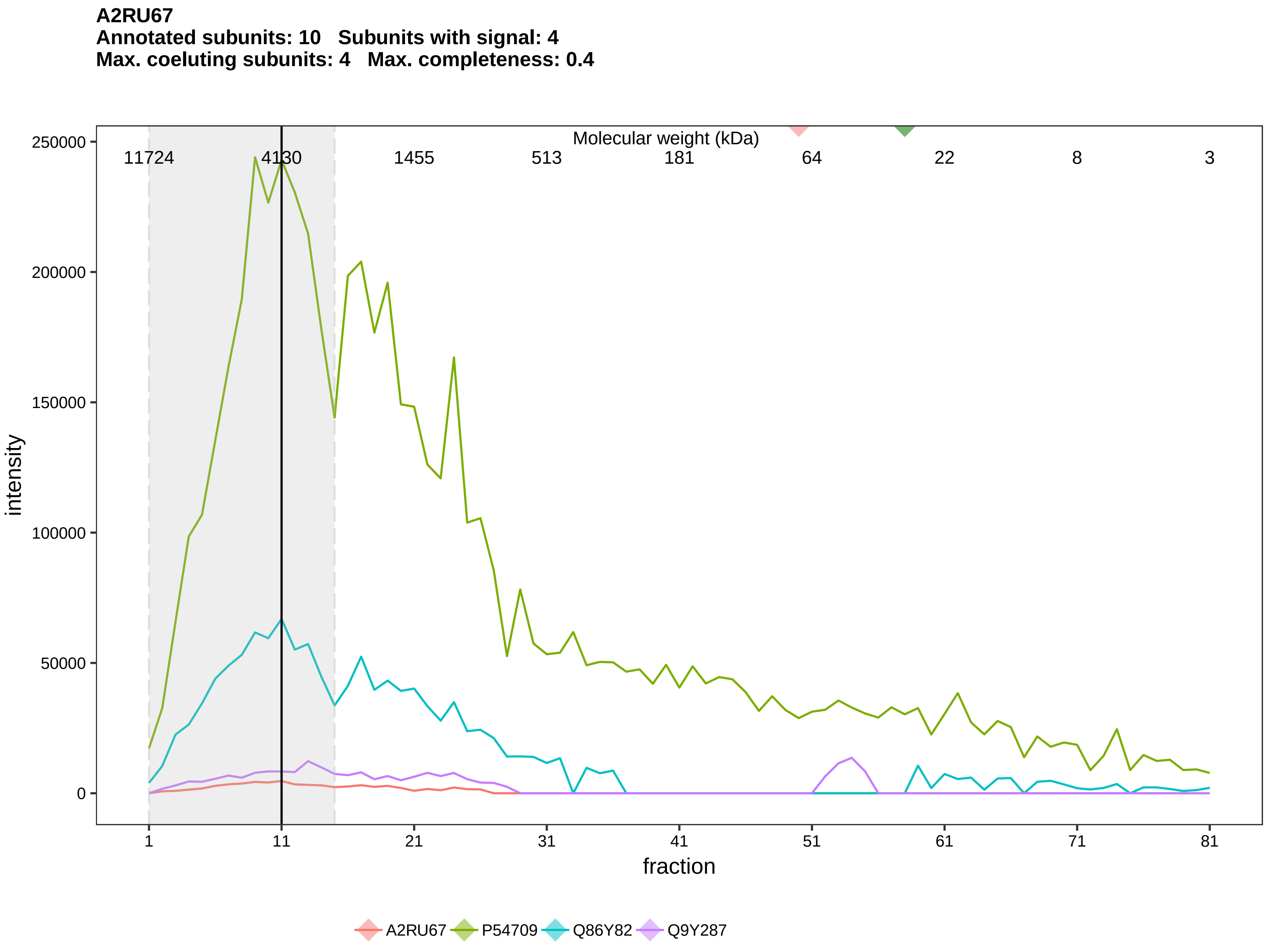
A2RU67
Annotated subunits: 10 Subunits with signal: 4
Max. coeluting subunits: 4 Max. completeness: 0.4
Molecular weight (kDa)
11724
4130
1455
513
181
64
22
8
3
250000
200000
150000
100000
50000
0
intensity
1
11
21
31
41
51
61
71
81
fraction
A2RU67
P54709
Q86Y82
Q9Y287
