# Supplementary material for: Complex‐centric proteome profiling by SEC‐SWATH‐MS
Source: Mol Syst Biol. 2019 Jan 14;15(1):e8438. doi: 10.15252/msb.20188438 (PMC6346213; doi:10.15252/msb.20188438)
Supplement: Supplementary file 7 — Dataset EV6 [file MSB-15-e8438-s007.zip › feature_plots_bioplex/O00165.pdf]

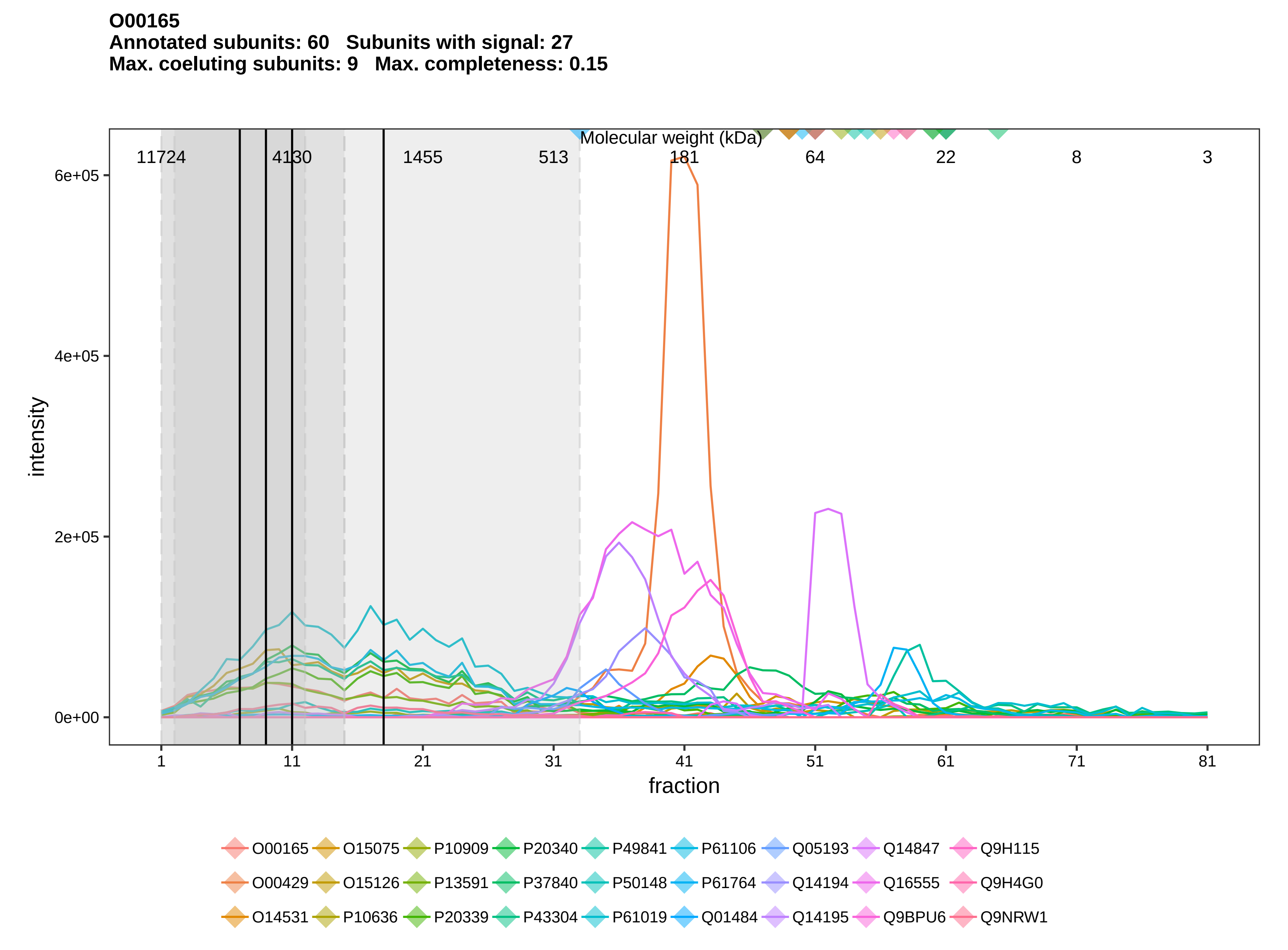
O00165
Annotated subunits: 60   Subunits with signal: 27
Max. coeluting subunits: 9   Max. completeness: 0.15
Molecular weight (kDa)
11724
4130
1455
513
181
64
22
8
3
intensity
6e+05
4e+05
2e+05
0e+00
fraction
1
11
21
31
41
51
61
71
81
O00165
O00429
O14531
O15075
O15126
P10636
P10909
P13591
P20339
P20340
P37840
P43304
P49841
P50148
P61019
P61106
P61764
Q01484
Q05193
Q14194
Q14195
Q14847
Q16555
Q9BPU6
Q9H115
Q9H4G0
Q9NRW1
