# Supplementary material for: Complex‐centric proteome profiling by SEC‐SWATH‐MS
Source: Mol Syst Biol. 2019 Jan 14;15(1):e8438. doi: 10.15252/msb.20188438 (PMC6346213; doi:10.15252/msb.20188438)
Supplement: Supplementary file 7 — Dataset EV6 [file MSB-15-e8438-s007.zip › feature_plots_bioplex/O00560.pdf]

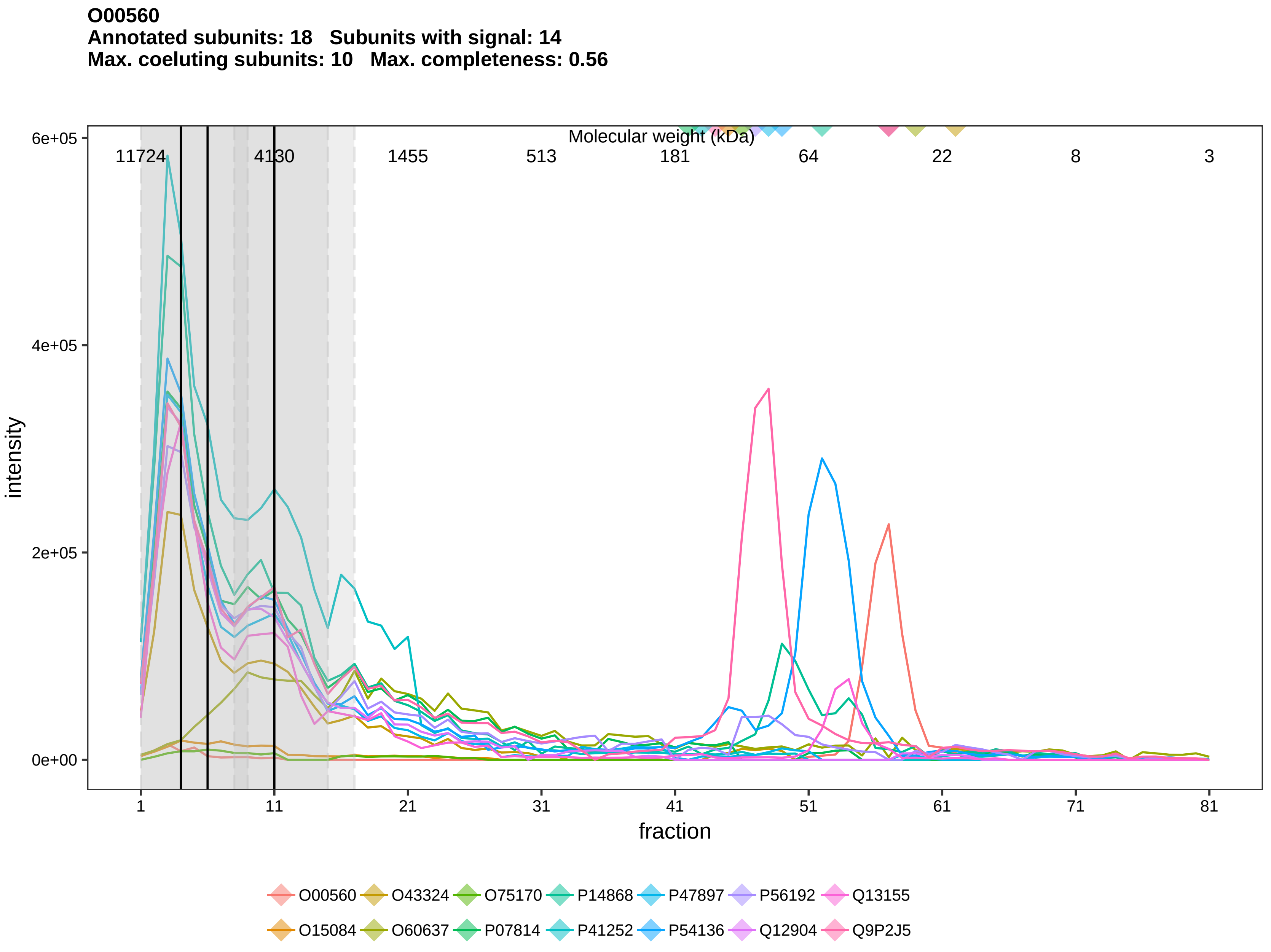

O00560
Annotated subunits: 18 Subunits with signal: 14
Max. coeluting subunits: 10 Max. completeness: 0.56
Molecular weight (kDa)
11724
4130
1455
513
181
64
22
8
3
6e+05
4e+05
2e+05
0e+00
intensity
1
11
21
31
41
51
61
71
81
fraction
O00560
O43324
O75170
P14868
P47897
P56192
Q13155
O15084
O60637
P07814
P41252
P54136
Q12904
Q9P2J5
