# Supplementary material for: Complex‐centric proteome profiling by SEC‐SWATH‐MS
Source: Mol Syst Biol. 2019 Jan 14;15(1):e8438. doi: 10.15252/msb.20188438 (PMC6346213; doi:10.15252/msb.20188438)
Supplement: Supplementary file 7 — Dataset EV6 [file MSB-15-e8438-s007.zip › feature_plots_bioplex/O00628.pdf]

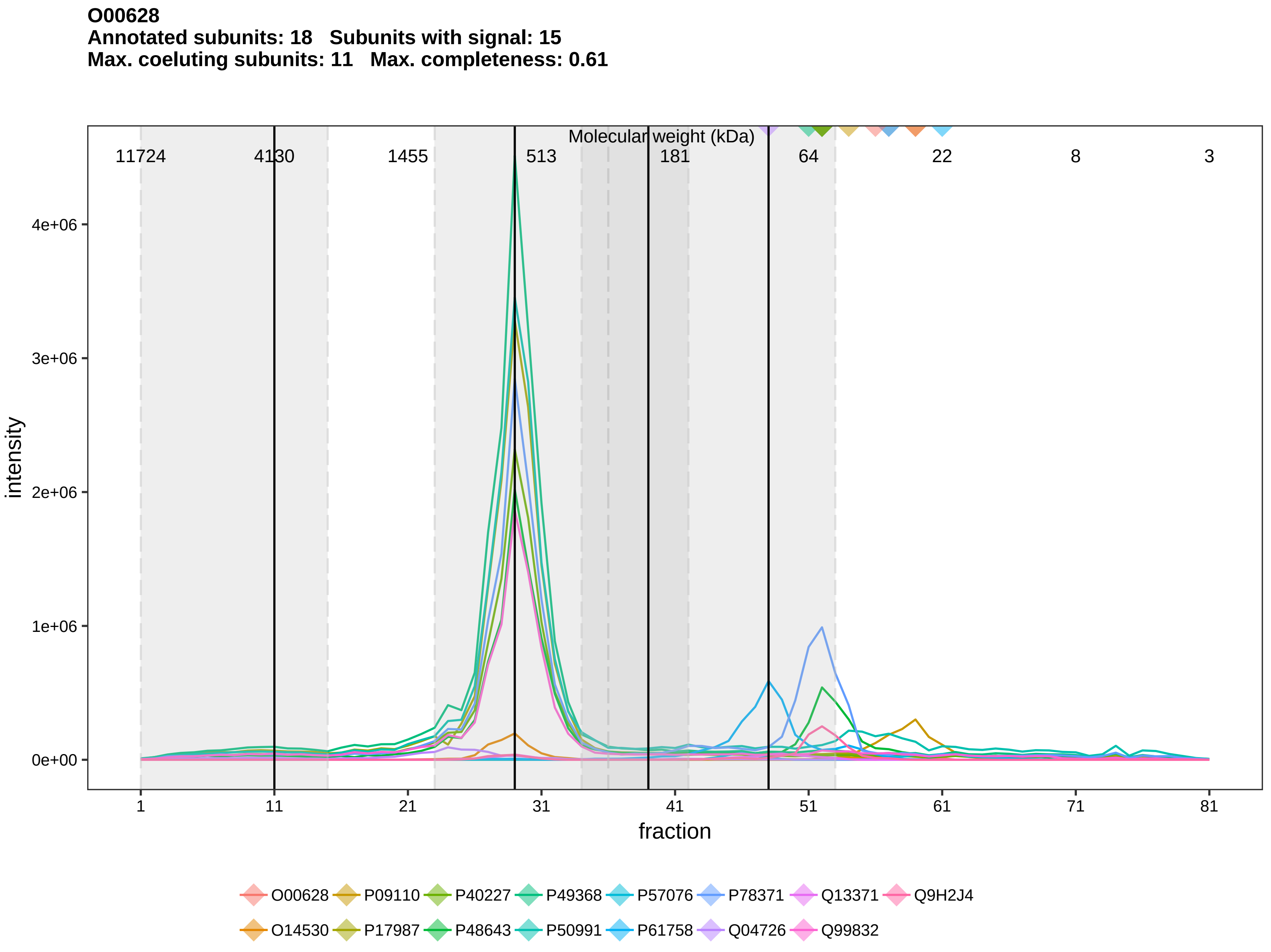
O00628
Annotated subunits: 18 Subunits with signal: 15
Max. coeluting subunits: 11 Max. completeness: 0.61
Molecular weight (kDa)
11724
4130
1455
513
181
64
22
8
3
4e+06
3e+06
2e+06
1e+06
0e+00
intensity
1
11
21
31
41
51
61
71
81
fraction
O00628
P09110
P40227
P49368
P57076
P78371
Q13371
Q9H2J4
O14530
P17987
P48643
P50991
P61758
Q04726
Q99832
