# Supplementary material for: Complex‐centric proteome profiling by SEC‐SWATH‐MS
Source: Mol Syst Biol. 2019 Jan 14;15(1):e8438. doi: 10.15252/msb.20188438 (PMC6346213; doi:10.15252/msb.20188438)
Supplement: Supplementary file 7 — Dataset EV6 [file MSB-15-e8438-s007.zip › feature_plots_bioplex/O14763.pdf]

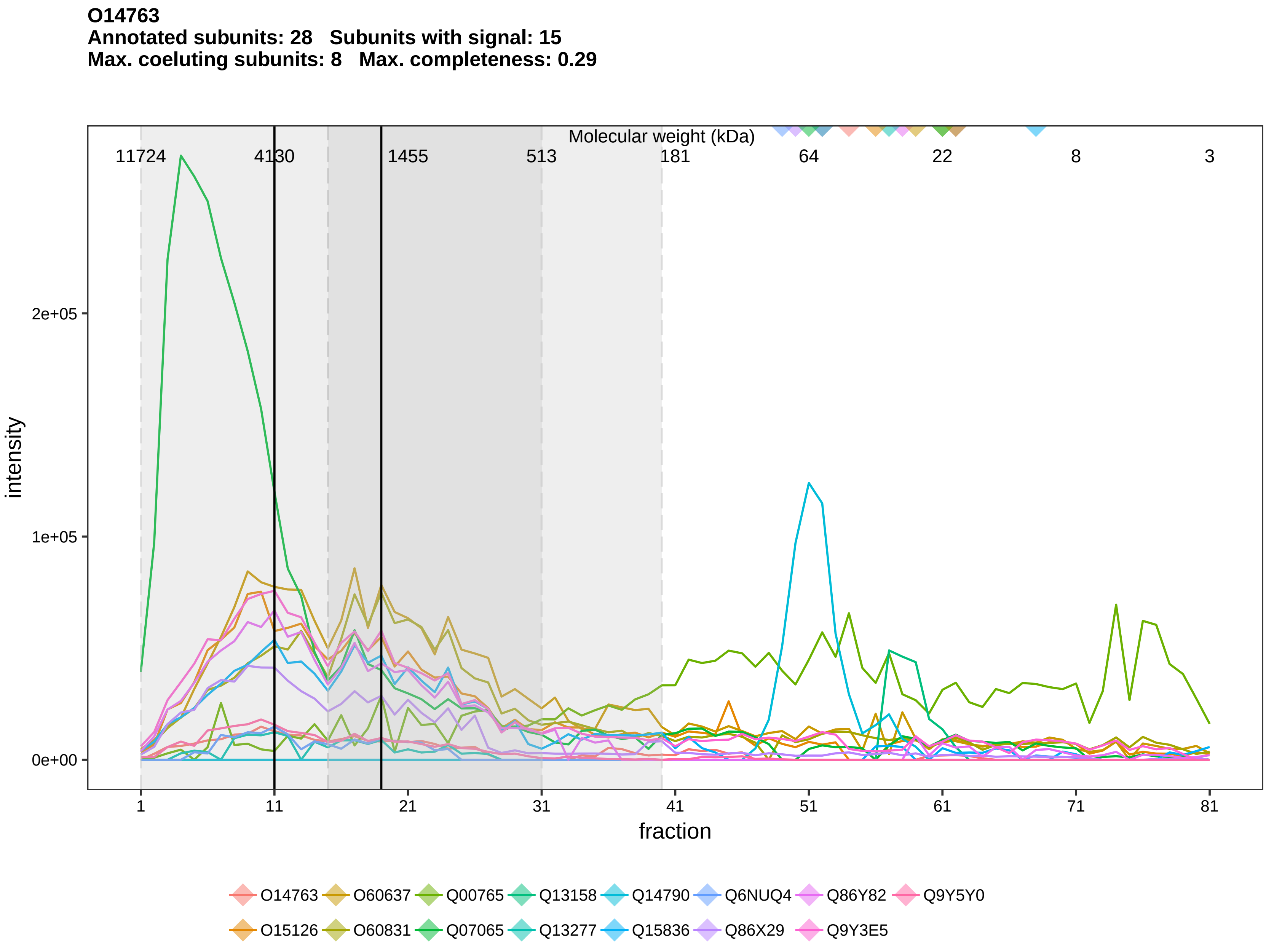
O14763
Annotated subunits: 28 Subunits with signal: 15
Max. coeluting subunits: 8 Max. completeness: 0.29
Molecular weight (kDa)
11724
4130
1455
513
181
64
22
8
3
intensity
2e+05
1e+05
0e+00
fraction
1
11
21
31
41
51
61
71
81
O14763
O60637
Q00765
Q13158
Q14790
Q6NUQ4
Q86Y82
Q9Y5Y0
O15126
O60831
Q07065
Q13277
Q15836
Q86X29
Q9Y3E5
