# Supplementary material for: Complex‐centric proteome profiling by SEC‐SWATH‐MS
Source: Mol Syst Biol. 2019 Jan 14;15(1):e8438. doi: 10.15252/msb.20188438 (PMC6346213; doi:10.15252/msb.20188438)
Supplement: Supplementary file 7 — Dataset EV6 [file MSB-15-e8438-s007.zip › feature_plots_bioplex/O75489.pdf]

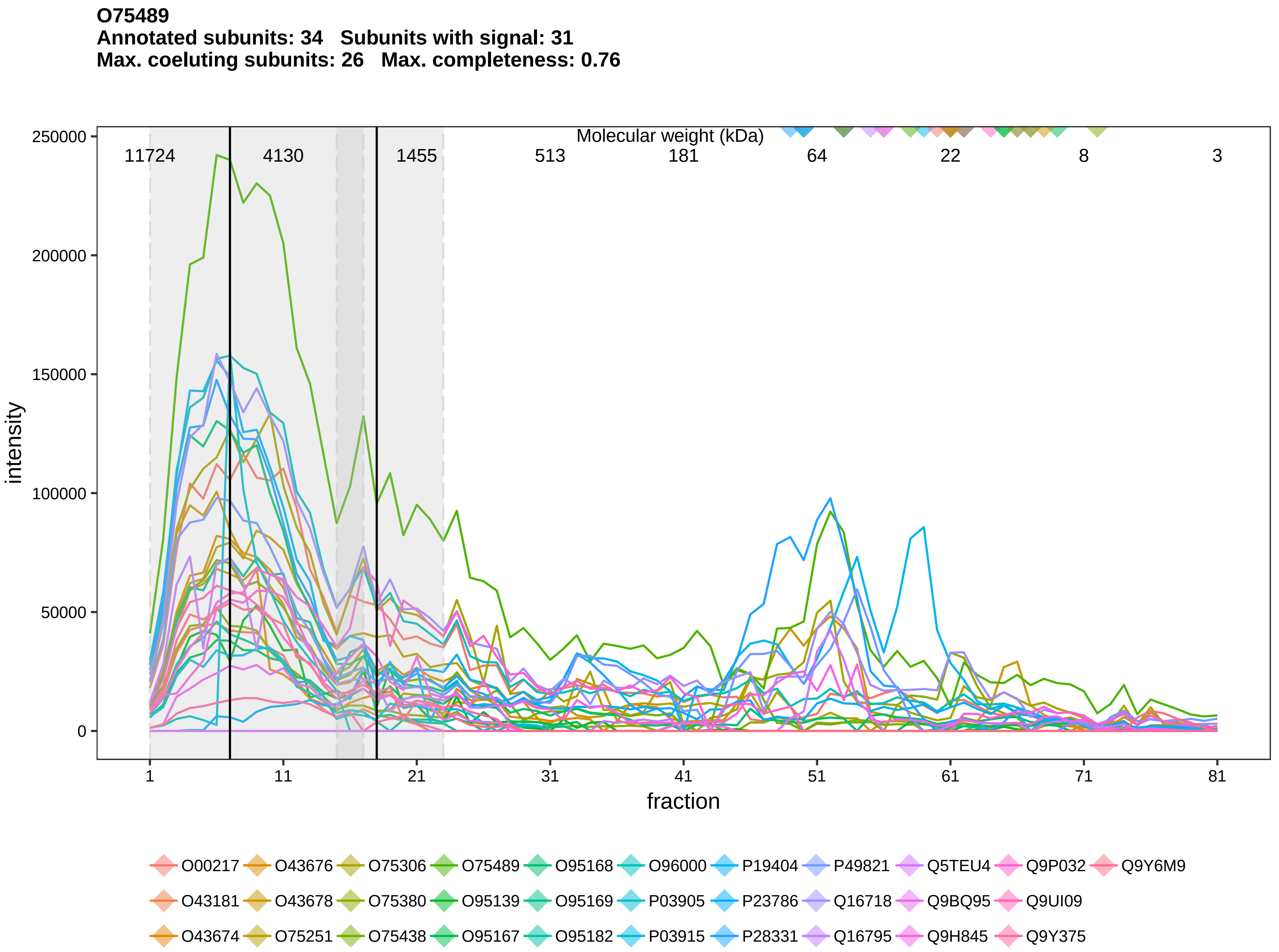
O75489
Annotated subunits: 34 Subunits with signal: 31
Max. coeluting subunits: 26 Max. completeness: 0.76
Molecular weight (kDa)
11724
4130
1455
513
181
64
22
8
3
intensity
250000
200000
150000
100000
50000
0
fraction
1
11
21
31
41
51
61
71
81
O00217
O43181
O43674
O43676
O43678
O75251
O75306
O75380
O75438
O75489
O95139
O95167
O95168
O95169
O95182
O96000
P03905
P03915
P19404
P23786
P28331
P49821
Q16718
Q16795
Q5TEU4
Q9BQ95
Q9H845
Q9P032
Q9UI09
Q9Y375
Q9Y6M9
