# Supplementary material for: Complex‐centric proteome profiling by SEC‐SWATH‐MS
Source: Mol Syst Biol. 2019 Jan 14;15(1):e8438. doi: 10.15252/msb.20188438 (PMC6346213; doi:10.15252/msb.20188438)
Supplement: Supplementary file 7 — Dataset EV6 [file MSB-15-e8438-s007.zip › feature_plots_bioplex/O75643.pdf]

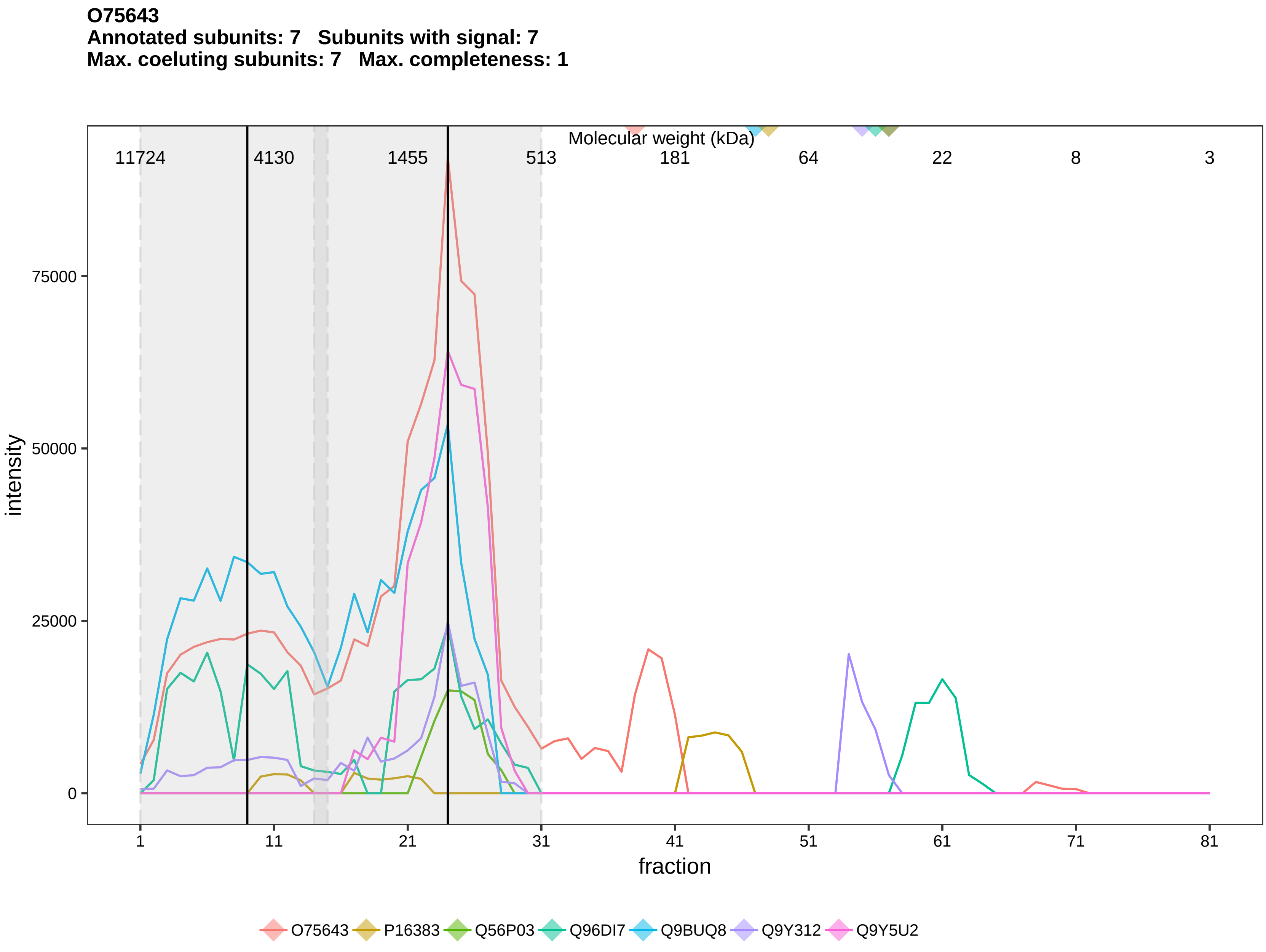
O75643
Annotated subunits: 7   Subunits with signal: 7
Max. coeluting subunits: 7   Max. completeness: 1
Molecular weight (kDa)
11724
4130
1455
513
181
64
22
8
3
intensity
75000
50000
25000
0
fraction
1
11
21
31
41
51
61
71
81
O75643
P16383
Q56P03
Q96DI7
Q9BUQ8
Q9Y312
Q9Y5U2
