# Supplementary material for: Complex‐centric proteome profiling by SEC‐SWATH‐MS
Source: Mol Syst Biol. 2019 Jan 14;15(1):e8438. doi: 10.15252/msb.20188438 (PMC6346213; doi:10.15252/msb.20188438)
Supplement: Supplementary file 7 — Dataset EV6 [file MSB-15-e8438-s007.zip › feature_plots_bioplex/O75695.pdf]

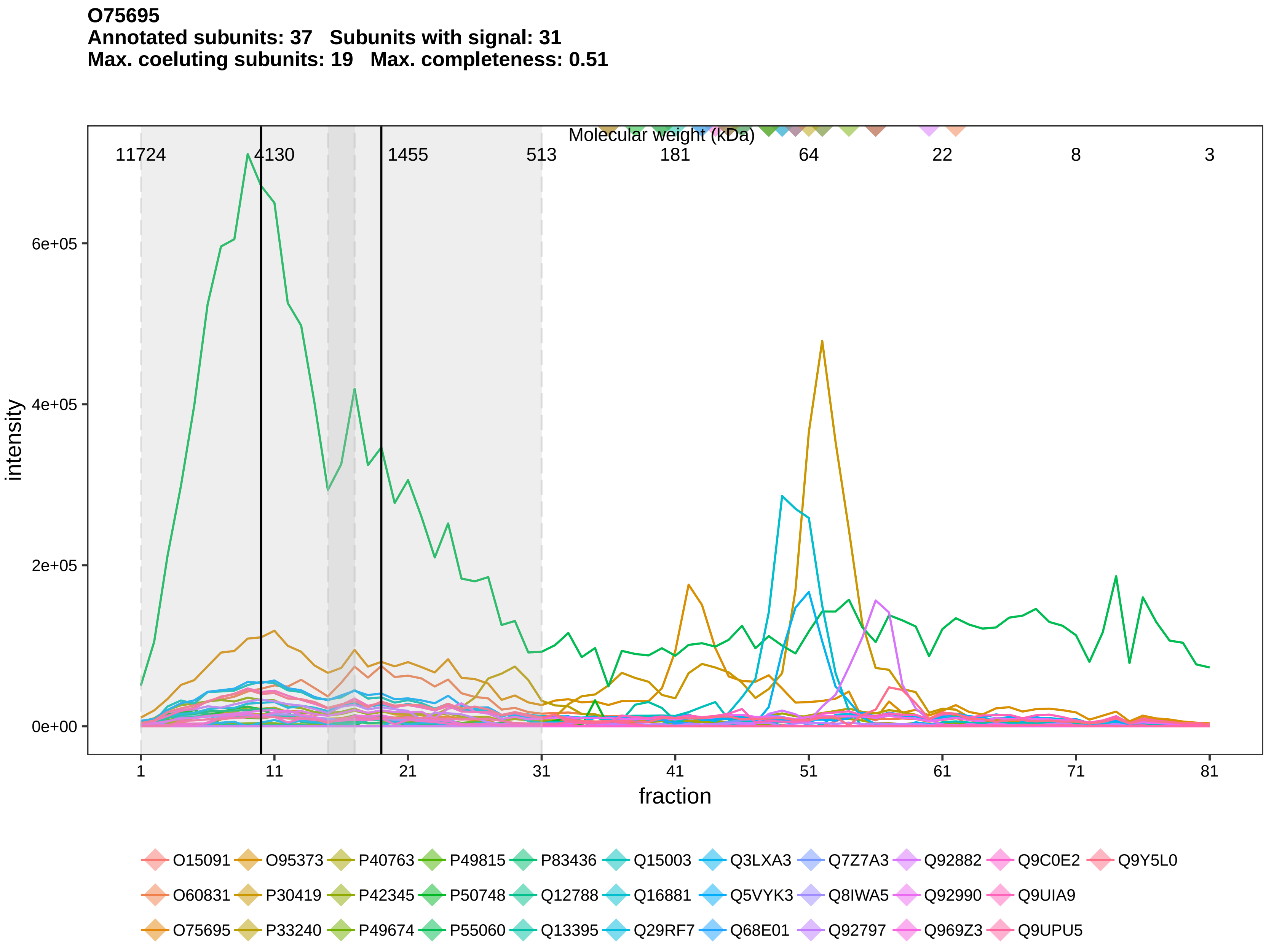
O75695
Annotated subunits: 37 Subunits with signal: 31
Max. coeluting subunits: 19 Max. completeness: 0.51
Molecular weight (kDa)
11724
4130
1455
513
181
64
22
8
3
intensity
6e+05
4e+05
2e+05
0e+00
1
11
21
31
41
51
61
71
81
fraction
O15091
O95373
P40763
P49815
P83436
Q15003
Q3LXA3
Q7Z7A3
Q92882
Q9C0E2
Q9Y5L0
O60831
P30419
P42345
P50748
Q12788
Q16881
Q5VYK3
Q8IWA5
Q92990
Q9UIA9
O75695
P33240
P49674
P55060
Q13395
Q29RF7
Q68E01
Q92797
Q969Z3
Q9UPU5
