# Supplementary material for: Complex‐centric proteome profiling by SEC‐SWATH‐MS
Source: Mol Syst Biol. 2019 Jan 14;15(1):e8438. doi: 10.15252/msb.20188438 (PMC6346213; doi:10.15252/msb.20188438)
Supplement: Supplementary file 7 — Dataset EV6 [file MSB-15-e8438-s007.zip › feature_plots_bioplex/O75791.pdf]

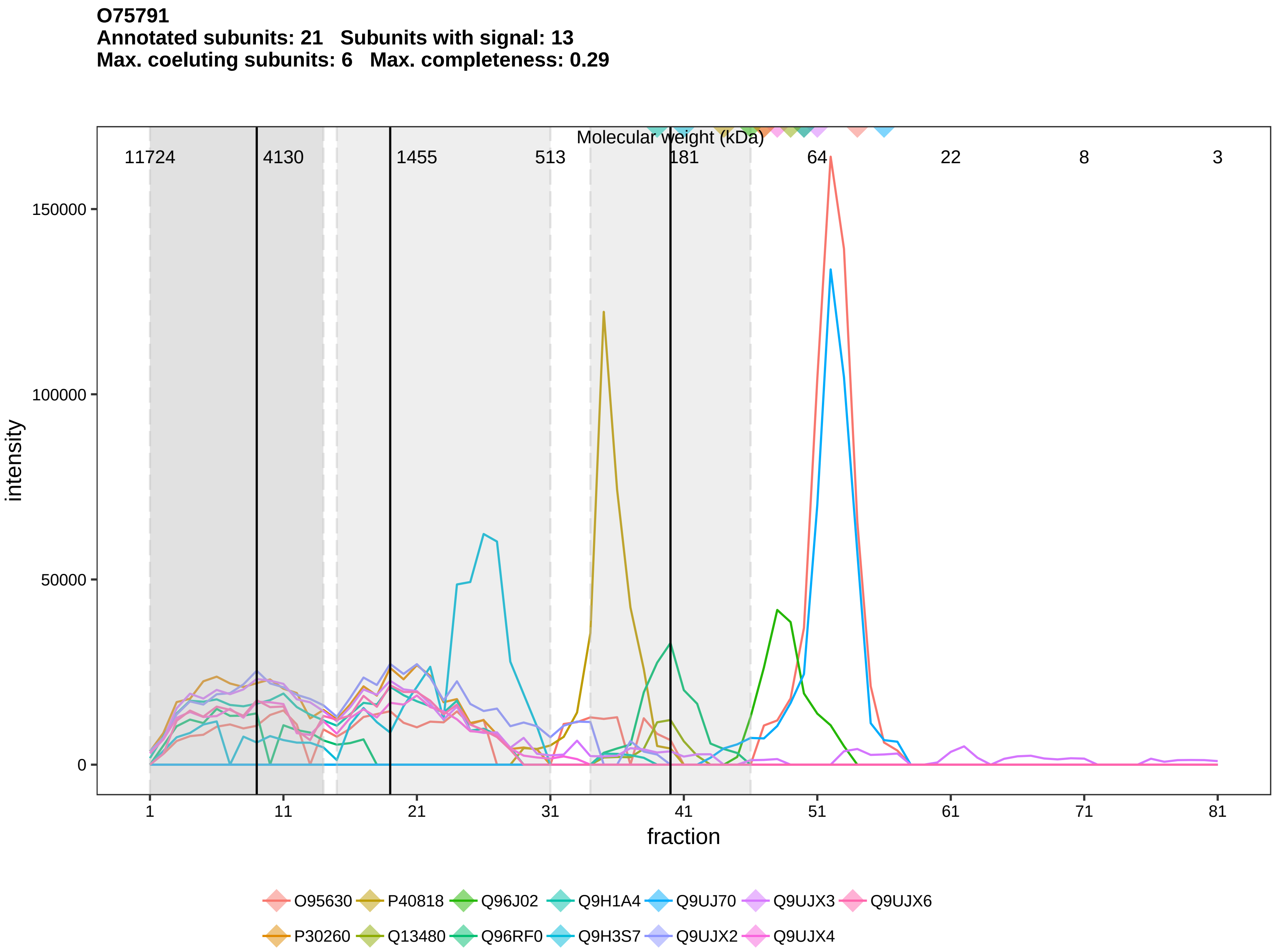
O75791
Annotated subunits: 21 Subunits with signal: 13
Max. coeluting subunits: 6 Max. completeness: 0.29
Molecular weight (kDa)
11724
4130
1455
513
181
64
22
8
3
intensity
150000
100000
50000
0
1
11
21
31
41
51
61
71
81
fraction
O95630
P40818
Q96J02
Q9H1A4
Q9UJ70
Q9UJX3
Q9UJX6
P30260
Q13480
Q96RF0
Q9H3S7
Q9UJX2
Q9UJX4
