# Supplementary material for: Complex‐centric proteome profiling by SEC‐SWATH‐MS
Source: Mol Syst Biol. 2019 Jan 14;15(1):e8438. doi: 10.15252/msb.20188438 (PMC6346213; doi:10.15252/msb.20188438)
Supplement: Supplementary file 7 — Dataset EV6 [file MSB-15-e8438-s007.zip › feature_plots_bioplex/O94766.pdf]

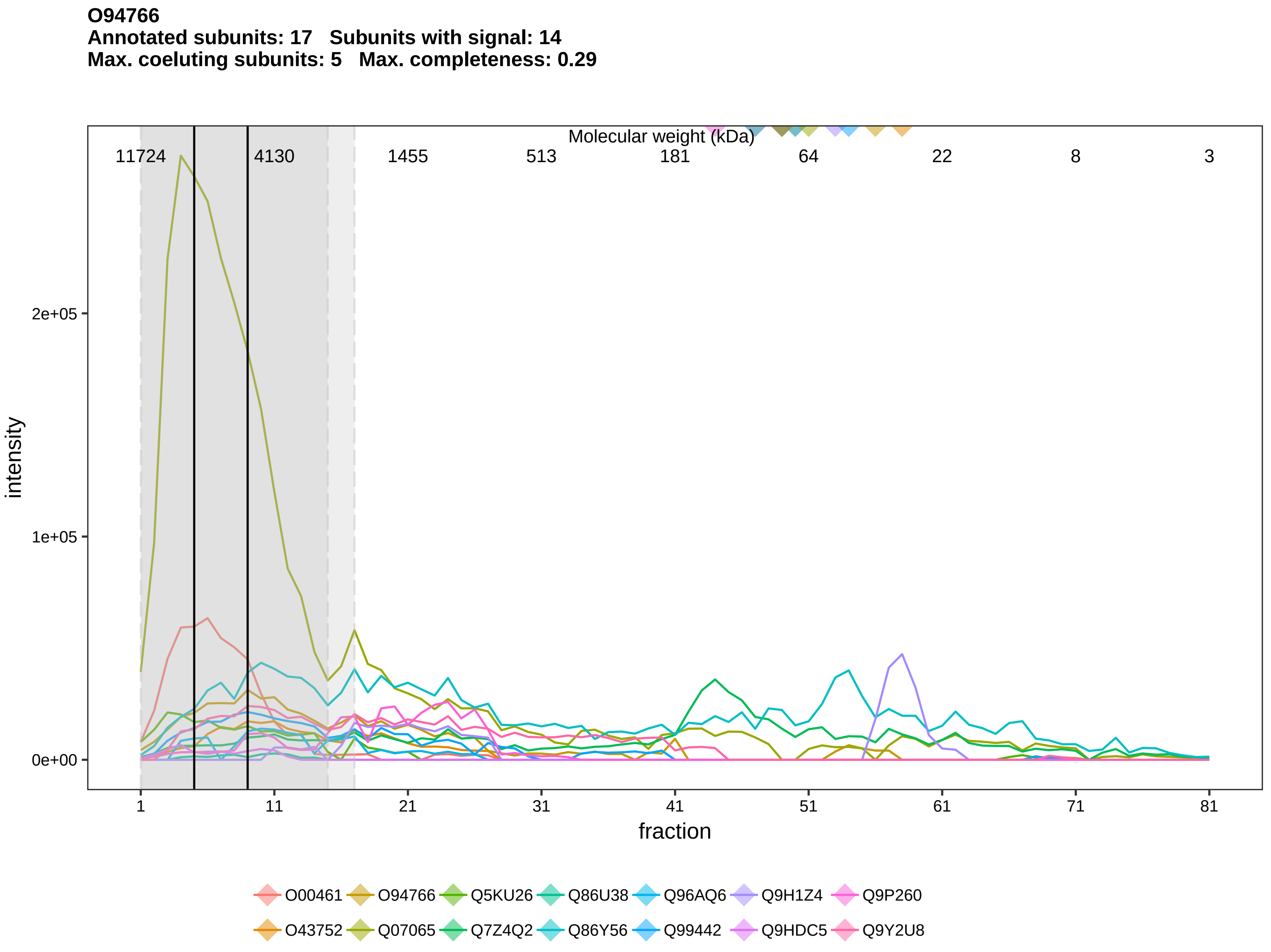
O94766
Annotated subunits: 17 Subunits with signal: 14
Max. coeluting subunits: 5 Max. completeness: 0.29
Molecular weight (kDa)
11724
4130
1455
513
181
64
22
8
3
intensity
2e+05
1e+05
0e+00
1
11
21
31
41
51
61
71
81
fraction
O00461
O94766
Q5KU26
Q86U38
Q96AQ6
Q9H1Z4
Q9P260
O43752
Q07065
Q7Z4Q2
Q86Y56
Q99442
Q9HDC5
Q9Y2U8
