# Supplementary material for: Complex‐centric proteome profiling by SEC‐SWATH‐MS
Source: Mol Syst Biol. 2019 Jan 14;15(1):e8438. doi: 10.15252/msb.20188438 (PMC6346213; doi:10.15252/msb.20188438)
Supplement: Supplementary file 7 — Dataset EV6 [file MSB-15-e8438-s007.zip › feature_plots_bioplex/O95721.pdf]

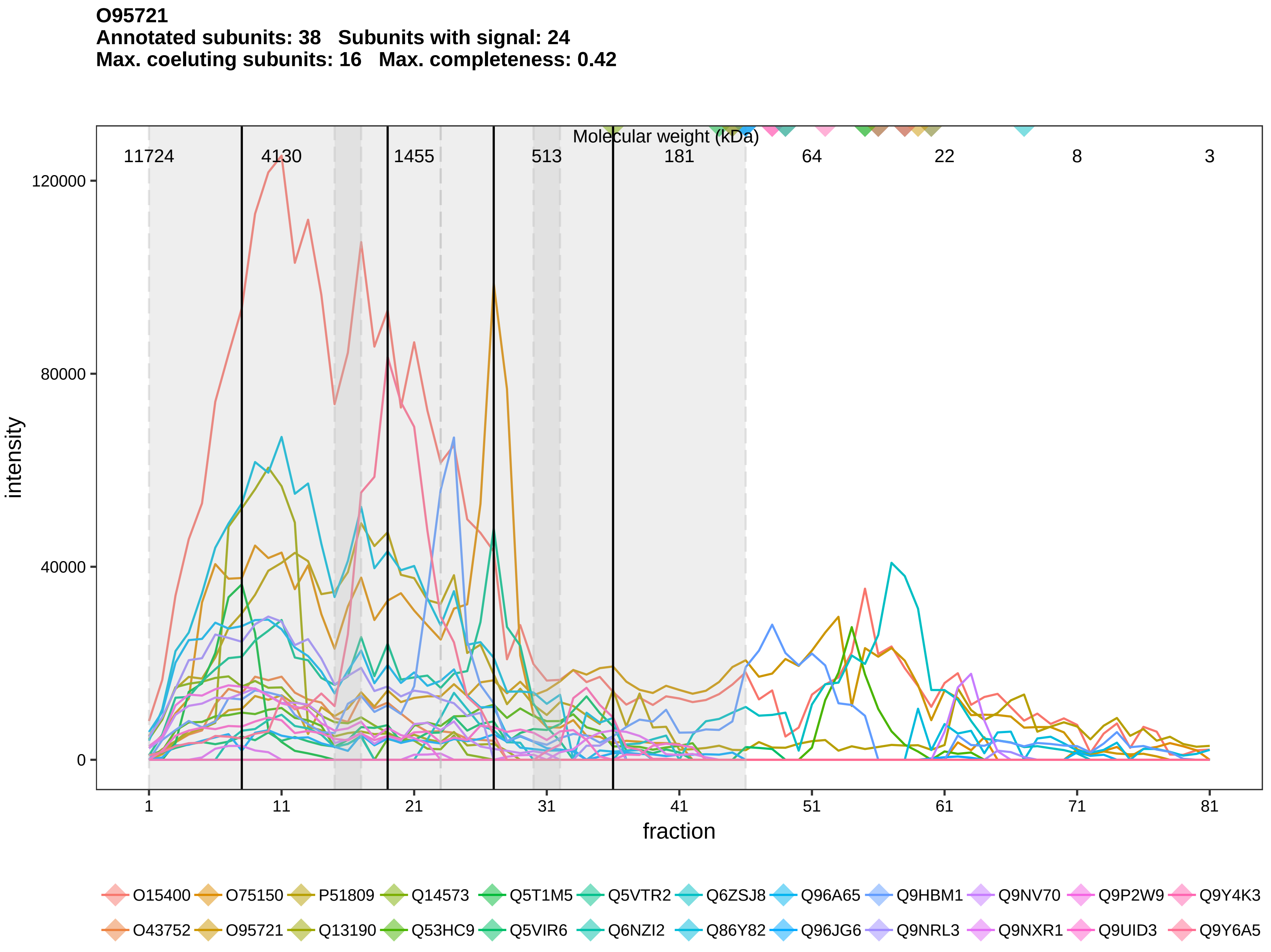
O95721
Annotated subunits: 38 Subunits with signal: 24
Max. coeluting subunits: 16 Max. completeness: 0.42
Molecular weight (kDa)
11724
4130
1455
513
181
64
22
8
3
intensity
120000
80000
40000
0
1
11
21
31
41
51
61
71
81
fraction
O15400
O75150
P51809
Q14573
Q5T1M5
Q5VTR2
Q6ZSJ8
Q96A65
Q9HBM1
Q9NV70
Q9P2W9
Q9Y4K3
O43752
O95721
Q13190
Q53HC9
Q5VIR6
Q6NZI2
Q86Y82
Q96JG6
Q9NRL3
Q9NXR1
Q9UID3
Q9Y6A5
