# Supplementary material for: Complex‐centric proteome profiling by SEC‐SWATH‐MS
Source: Mol Syst Biol. 2019 Jan 14;15(1):e8438. doi: 10.15252/msb.20188438 (PMC6346213; doi:10.15252/msb.20188438)
Supplement: Supplementary file 7 — Dataset EV6 [file MSB-15-e8438-s007.zip › feature_plots_bioplex/O95922.pdf]

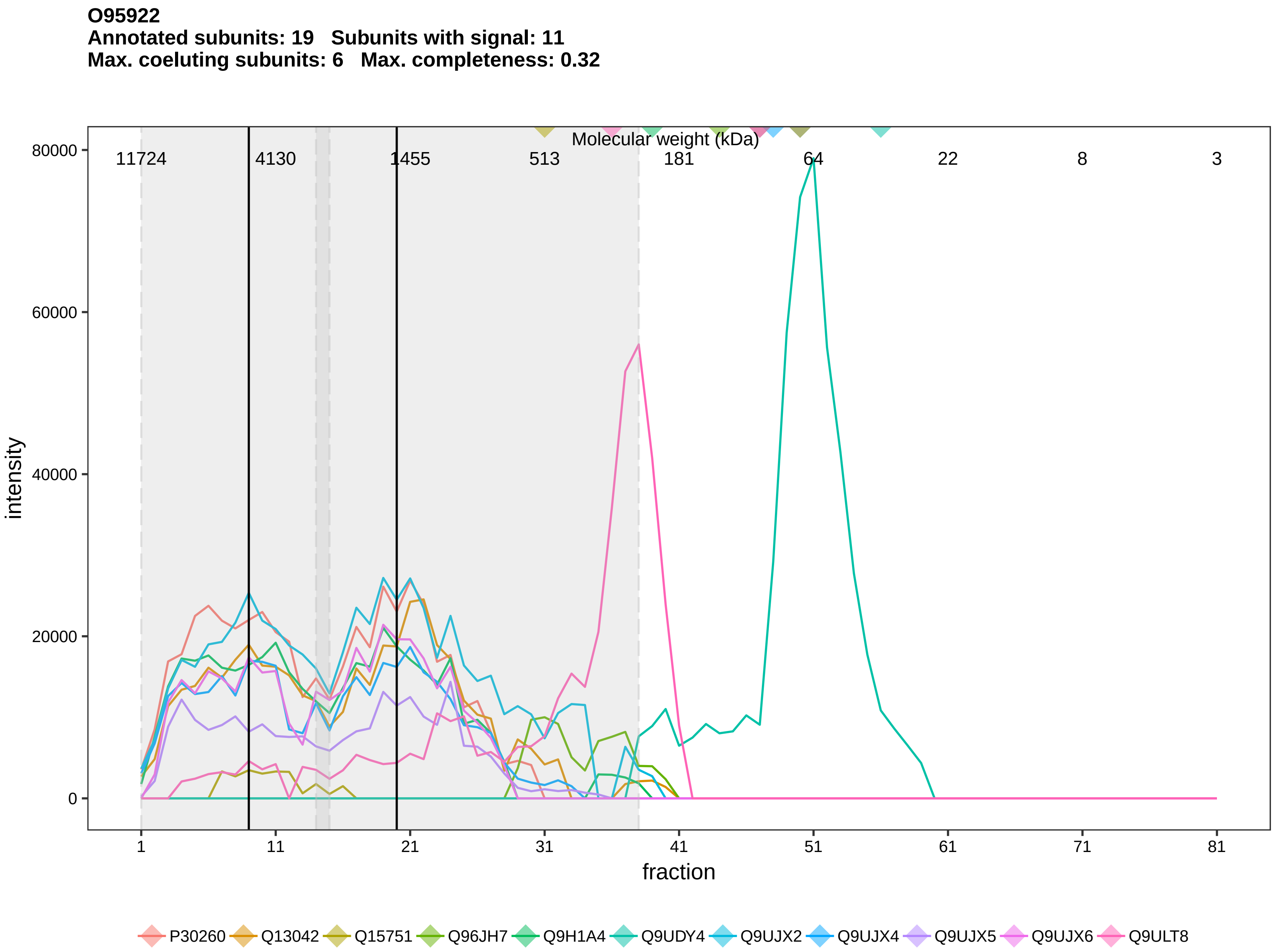

O95922
Annotated subunits: 19 Subunits with signal: 11
Max. coeluting subunits: 6 Max. completeness: 0.32
Molecular weight (kDa)
11724
4130
1455
513
181
64
22
8
3
intensity
80000
60000
40000
20000
0
1
11
21
31
41
51
61
71
81
fraction
P30260
Q13042
Q15751
Q96JH7
Q9H1A4
Q9UDY4
Q9UJX2
Q9UJX4
Q9UJX5
Q9UJX6
Q9ULT8
