# Supplementary material for: Complex‐centric proteome profiling by SEC‐SWATH‐MS
Source: Mol Syst Biol. 2019 Jan 14;15(1):e8438. doi: 10.15252/msb.20188438 (PMC6346213; doi:10.15252/msb.20188438)
Supplement: Supplementary file 7 — Dataset EV6 [file MSB-15-e8438-s007.zip › feature_plots_bioplex/O95988.pdf]

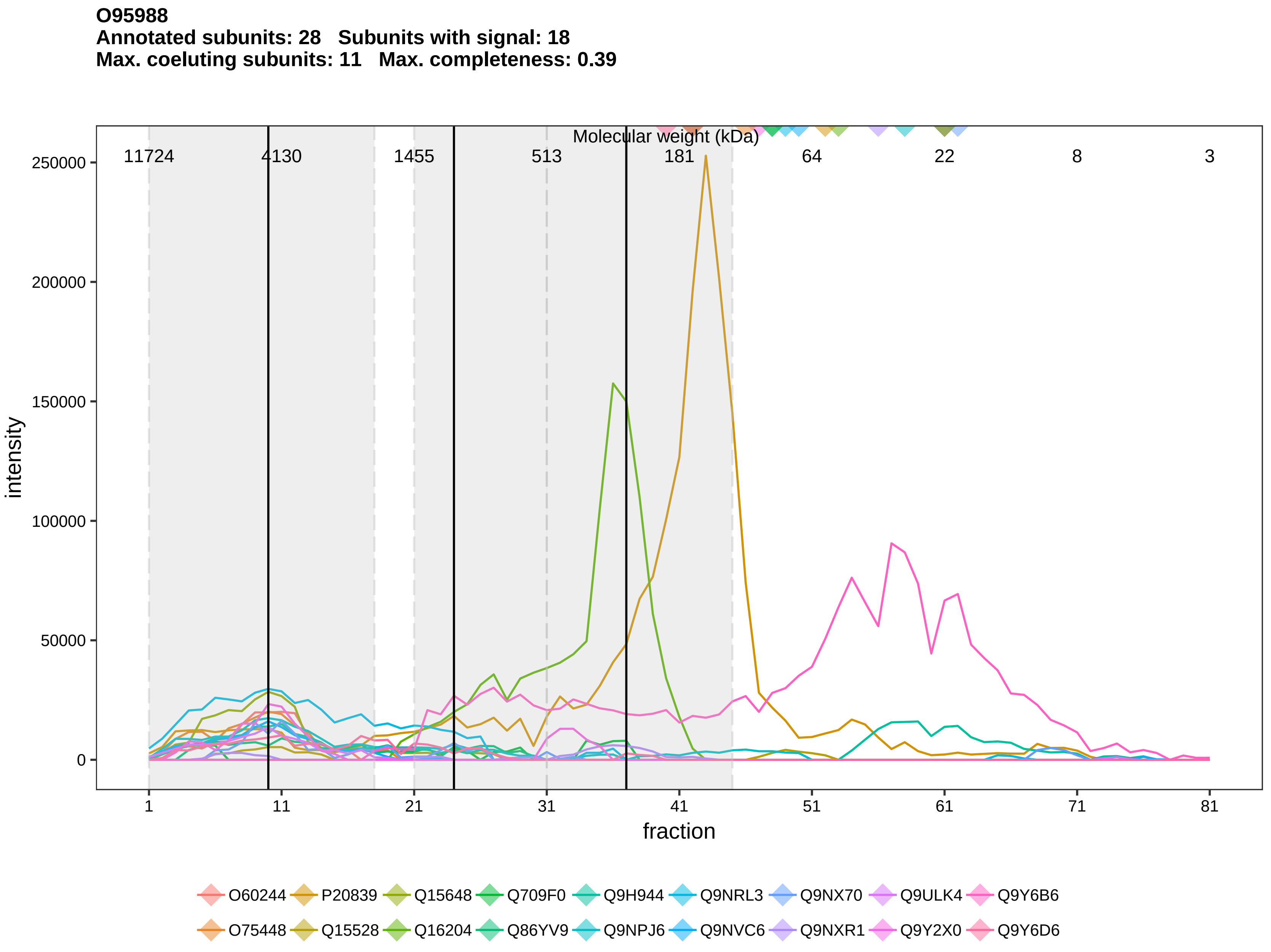
O95988
Annotated subunits: 28 Subunits with signal: 18
Max. coeluting subunits: 11 Max. completeness: 0.39
Molecular weight (kDa)
11724
4130
1455
513
181
64
22
8
3
intensity
250000
200000
150000
100000
50000
0
fraction
1
11
21
31
41
51
61
71
81
O60244
P20839
Q15648
Q709F0
Q9H944
Q9NRL3
Q9NX70
Q9ULK4
Q9Y6B6
O75448
Q15528
Q16204
Q86YV9
Q9NPJ6
Q9NVC6
Q9NXR1
Q9Y2X0
Q9Y6D6
