# Supplementary material for: Complex‐centric proteome profiling by SEC‐SWATH‐MS
Source: Mol Syst Biol. 2019 Jan 14;15(1):e8438. doi: 10.15252/msb.20188438 (PMC6346213; doi:10.15252/msb.20188438)
Supplement: Supplementary file 7 — Dataset EV6 [file MSB-15-e8438-s007.zip › feature_plots_bioplex/P06748.pdf]

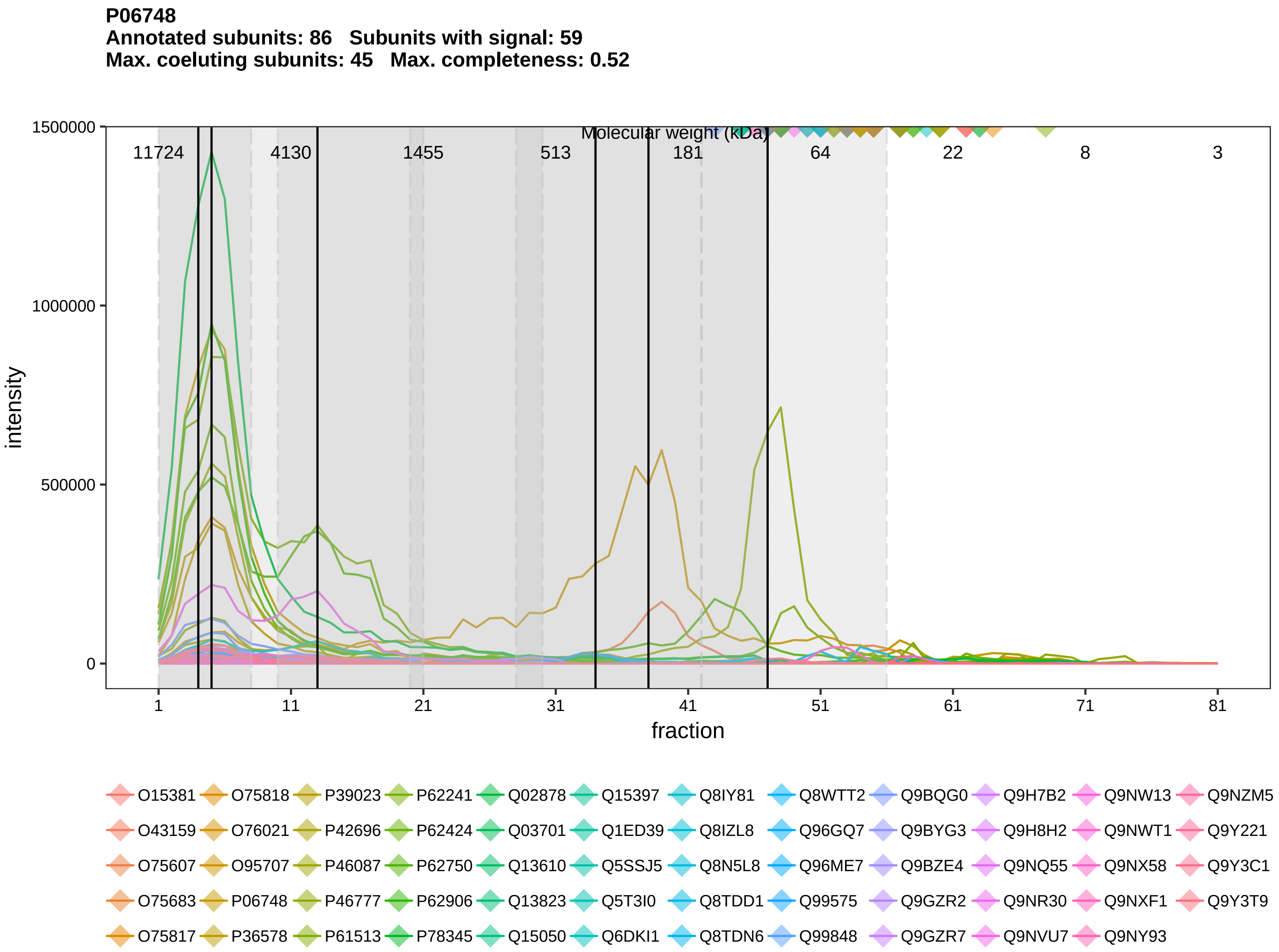

P06748
Annotated subunits: 86 Subunits with signal: 59
Max. coeluting subunits: 45 Max. completeness: 0.52
Molecular weight (kDa)
11724
4130
1455
513
181
64
22
8
3
1500000
1000000
500000
0
intensity
1
11
21
31
41
51
61
71
81
fraction
O15381
O75818
P39023
P62241
Q02878
Q15397
Q8IY81
Q8WTT2
Q9BQG0
Q9H7B2
Q9NW13
Q9NZM5
O43159
O76021
P42696
P62424
Q03701
Q1ED39
Q8IZL8
Q96GQ7
Q9BYG3
Q9H8H2
Q9NWT1
Q9Y221
O75607
O95707
P46087
P62750
Q13610
Q5SSJ5
Q8N5L8
Q96ME7
Q9BZE4
Q9NQ55
Q9NX58
Q9Y3C1
O75683
P06748
P46777
P62906
Q13823
Q5T3I0
Q8TDD1
Q99575
Q9GZR2
Q9NR30
Q9NXF1
Q9Y3T9
O75817
P36578
P61513
P78345
Q15050
Q6DKI1
Q8TDN6
Q99848
Q9GZR7
Q9NVU7
Q9NY93
