# Supplementary figures and images for: Complex‐centric proteome profiling by SEC‐SWATH‐MS (part 2 of 3)
Source: Mol Syst Biol. 2019 Jan 14;15(1):e8438. doi: 10.15252/msb.20188438 (PMC6346213; doi:10.15252/msb.20188438)

**O75787**

**Annotated subunits: 16 Subunits with signal: 11**

**Max. coeluting subunits: 7 Max. completeness: 0.44**

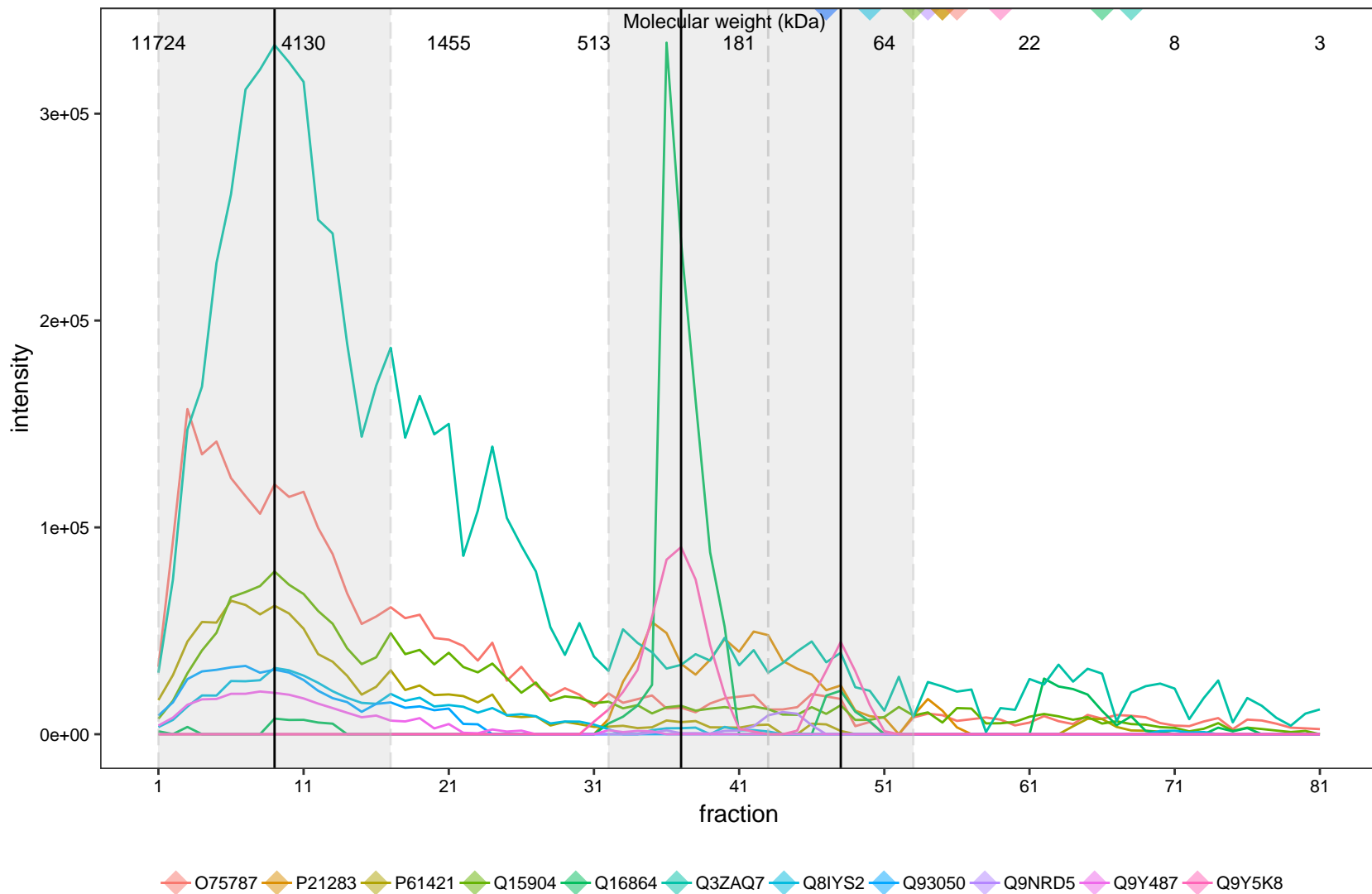

Supplement: Supplementary file 7 — Dataset EV6 [file MSB-15-e8438-s007.zip › feature_plots_bioplex/O75787.pdf]

O75818  
Annotated subunits: 5   Subunits with signal: 3  
Max. coeluting subunits: 2   Max. completeness: 0.4

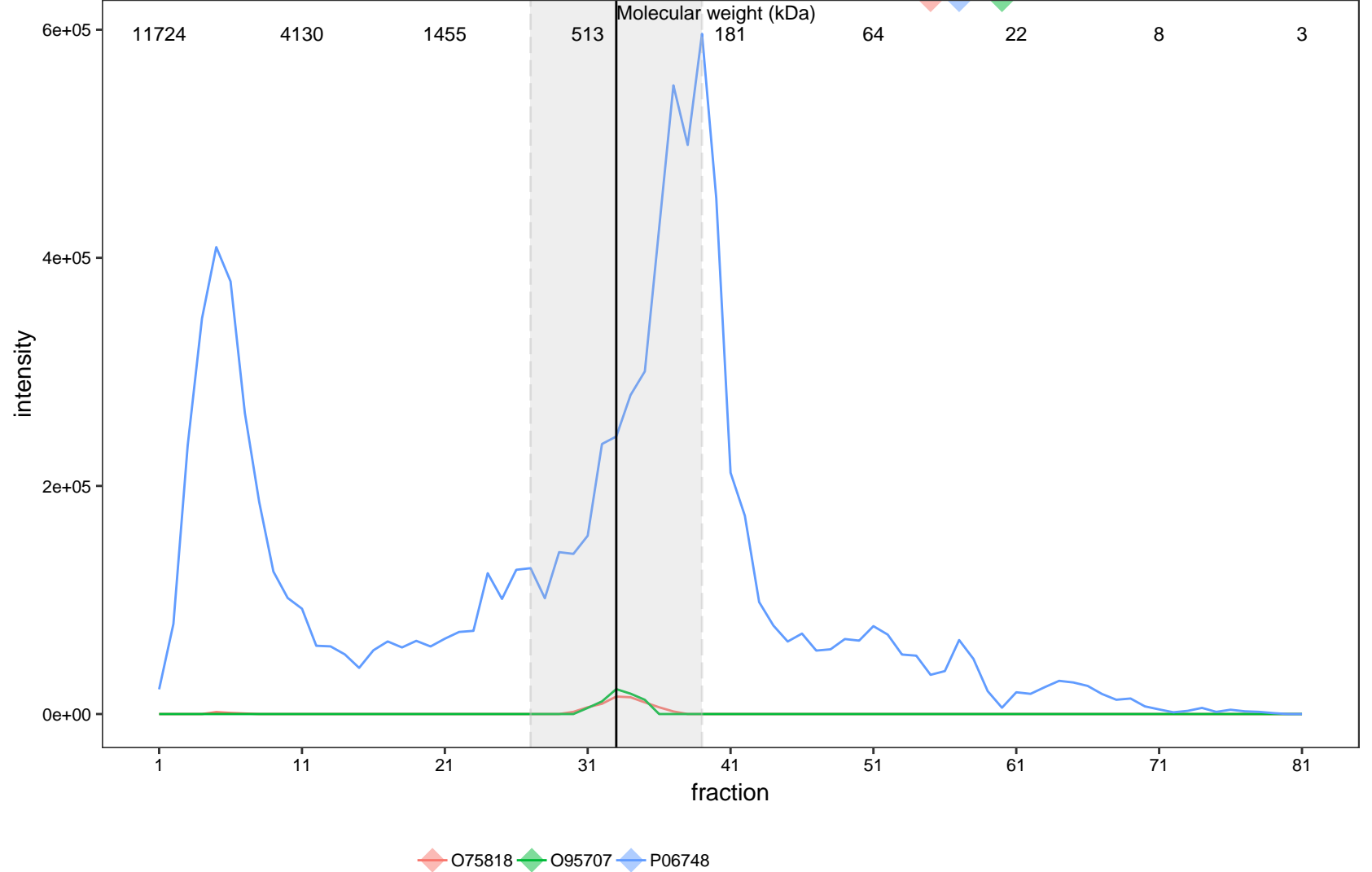

Supplement: Supplementary file 7 — Dataset EV6 [file MSB-15-e8438-s007.zip › feature_plots_bioplex/O75818.pdf]

O75821

Annotated subunits: 11 Subunits with signal: 11

Max. coeluting subunits: 11 Max. completeness: 1

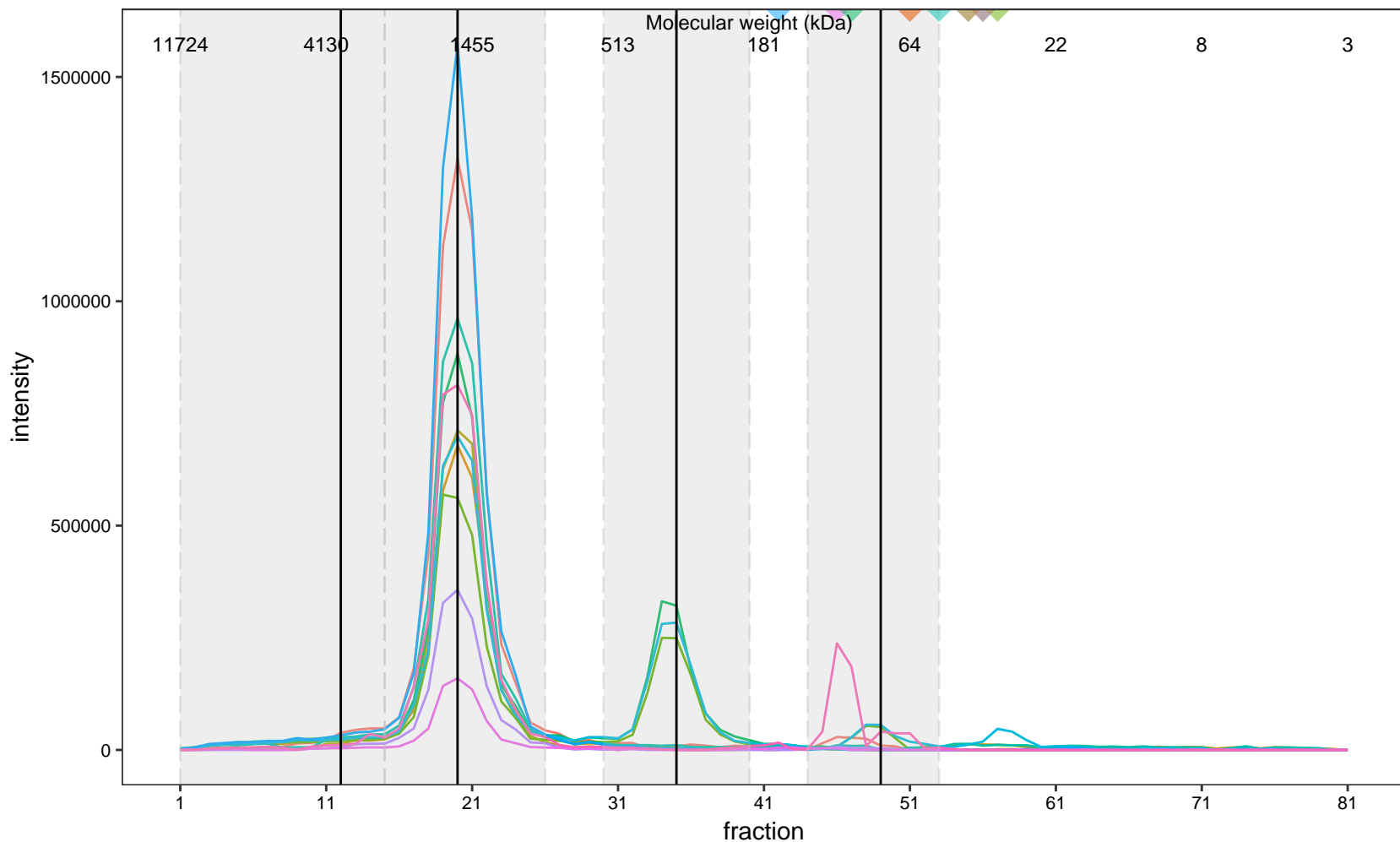

Supplement: Supplementary file 7 — Dataset EV6 [file MSB-15-e8438-s007.zip › feature_plots_bioplex/O75821.pdf]

**O75915**

**Annotated subunits: 12 Subunits with signal: 4**

**Max. coeluting subunits: 4 Max. completeness: 0.33**

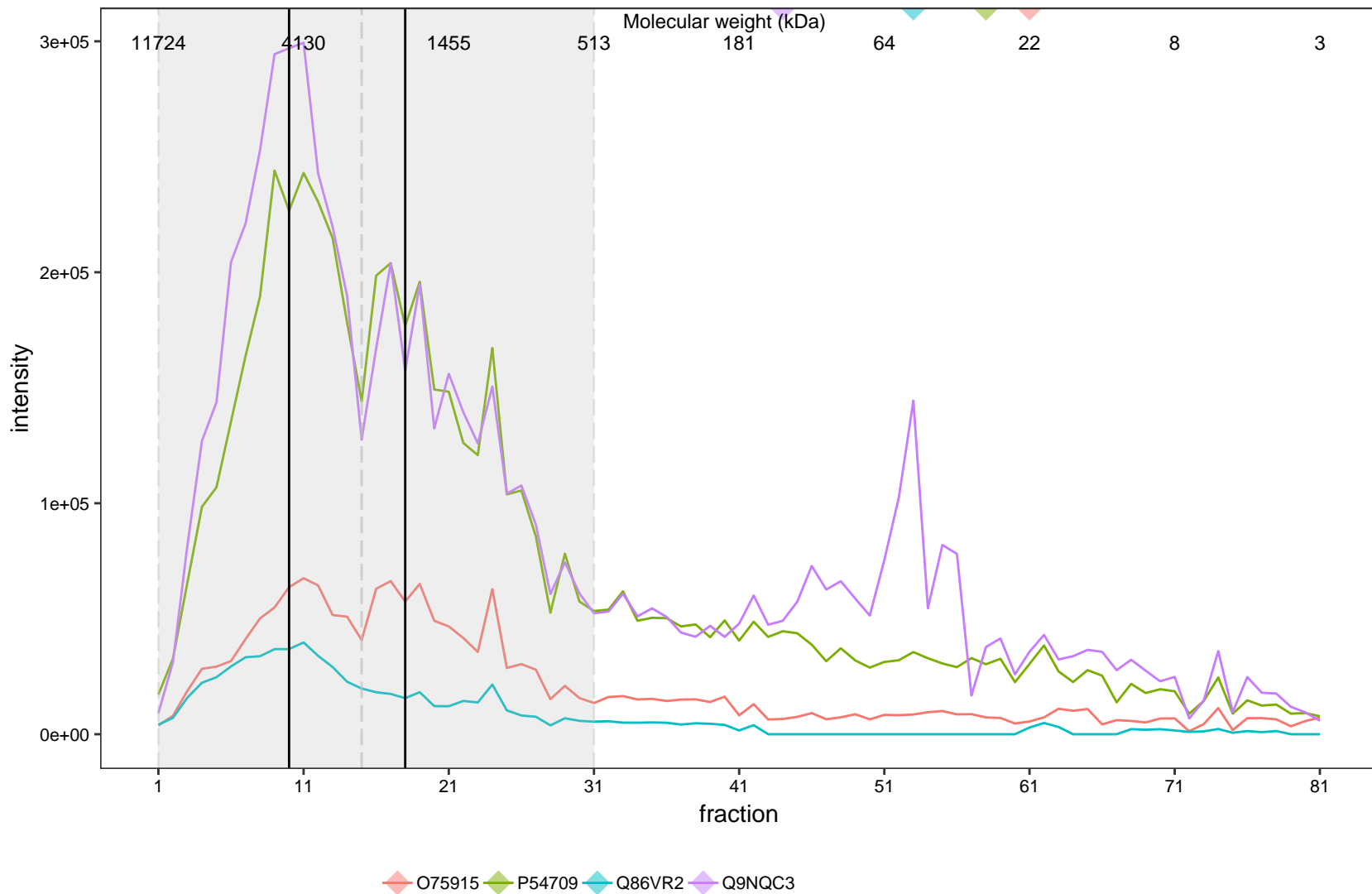

Supplement: Supplementary file 7 — Dataset EV6 [file MSB-15-e8438-s007.zip › feature_plots_bioplex/O75915.pdf]

**O75935**  
**Annotated subunits: 11   Subunits with signal: 4**  
**Max. coeluting subunits: 4   Max. completeness: 0.36**

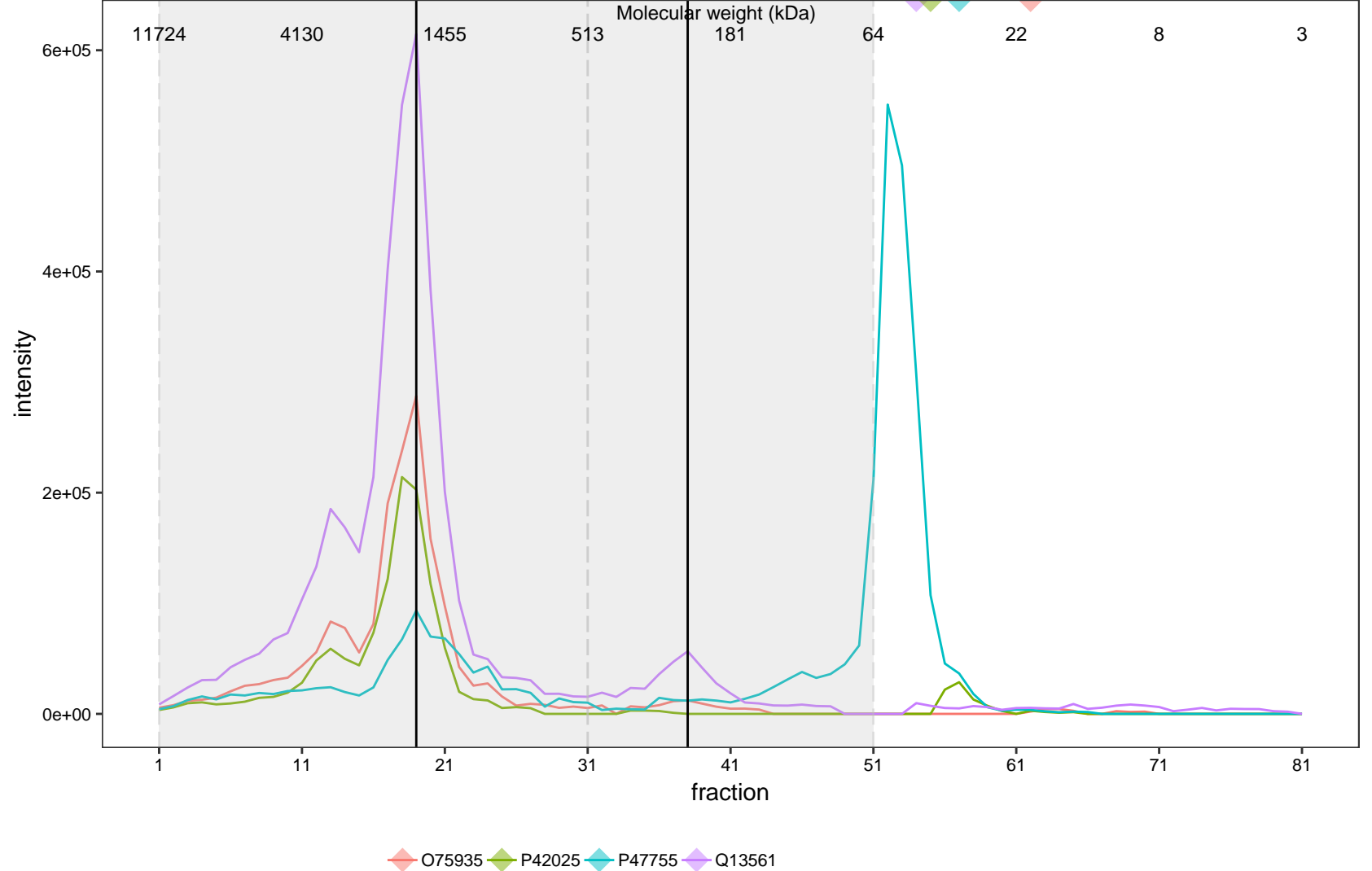

Supplement: Supplementary file 7 — Dataset EV6 [file MSB-15-e8438-s007.zip › feature_plots_bioplex/O75935.pdf]

**O75947**

**Annotated subunits: 2 Subunits with signal: 2**

**Max. coeluting subunits: 2 Max. completeness: 1**

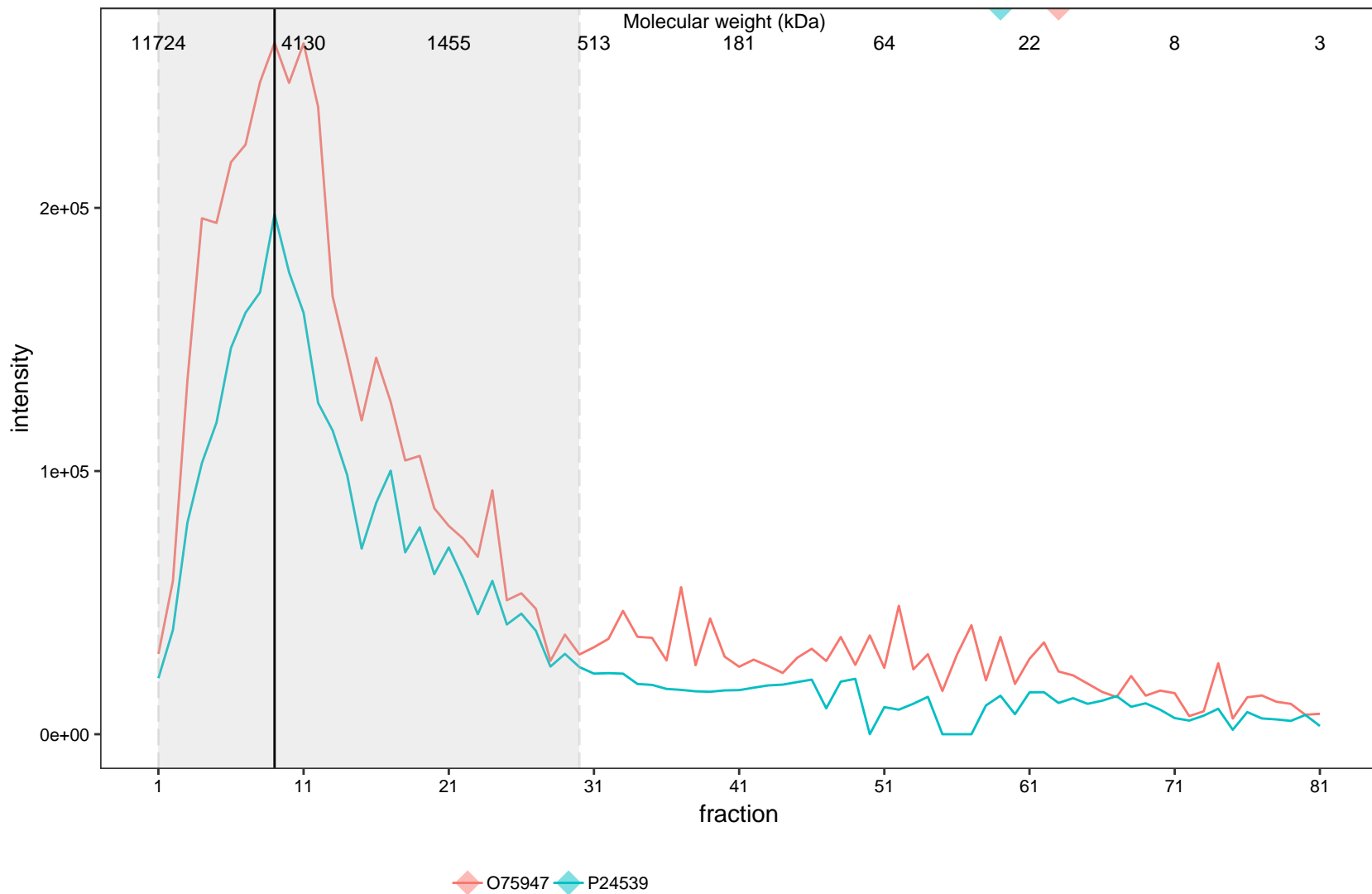

Supplement: Supplementary file 7 — Dataset EV6 [file MSB-15-e8438-s007.zip › feature_plots_bioplex/O75947.pdf]

**O76075**

**Annotated subunits: 3 Subunits with signal: 2**

**Max. coeluting subunits: 2 Max. completeness: 0.67**

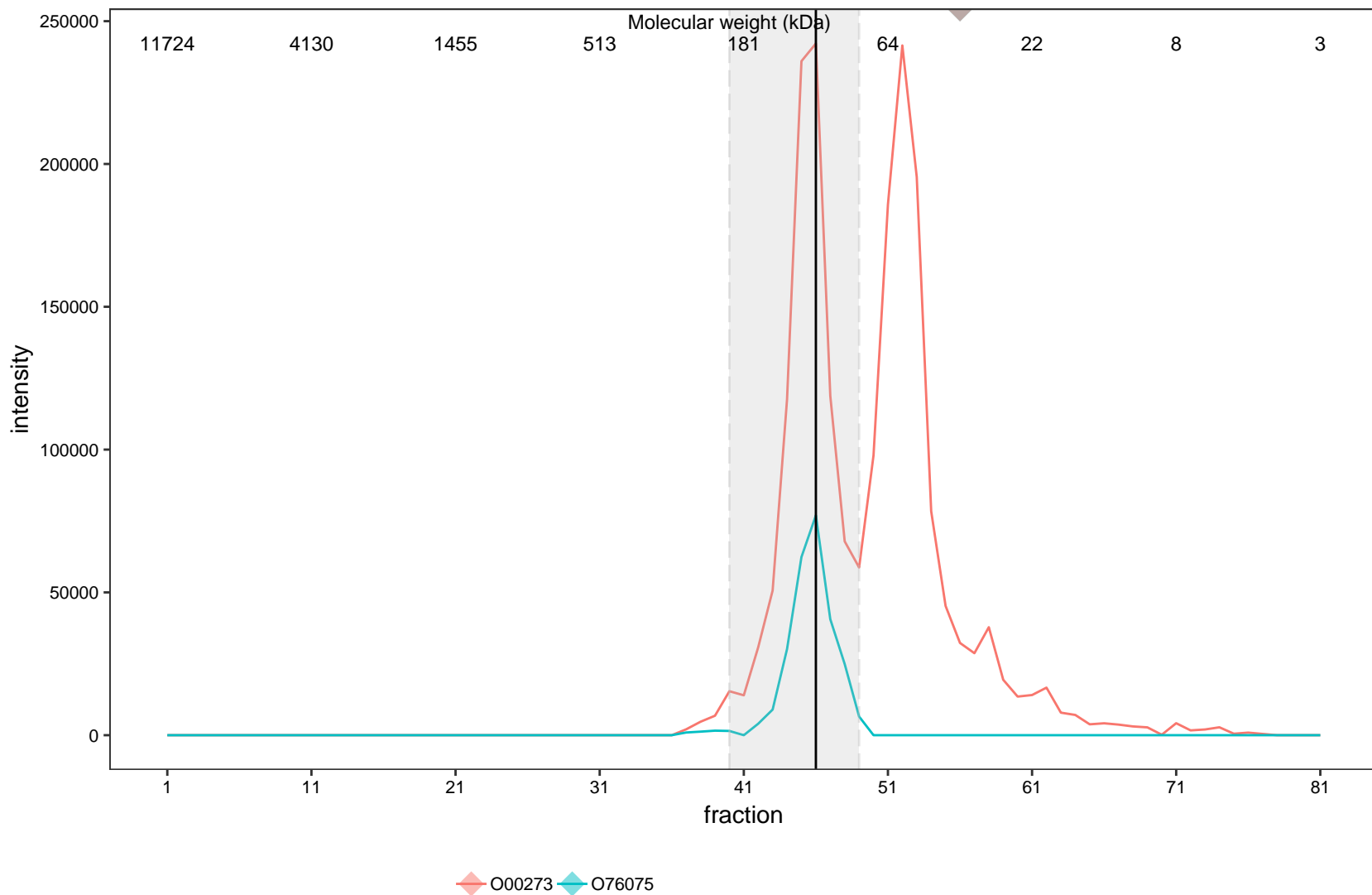

Supplement: Supplementary file 7 — Dataset EV6 [file MSB-15-e8438-s007.zip › feature_plots_bioplex/O76075.pdf]

**O76094**

**Annotated subunits: 3 Subunits with signal: 3**

**Max. coeluting subunits: 2 Max. completeness: 0.67**

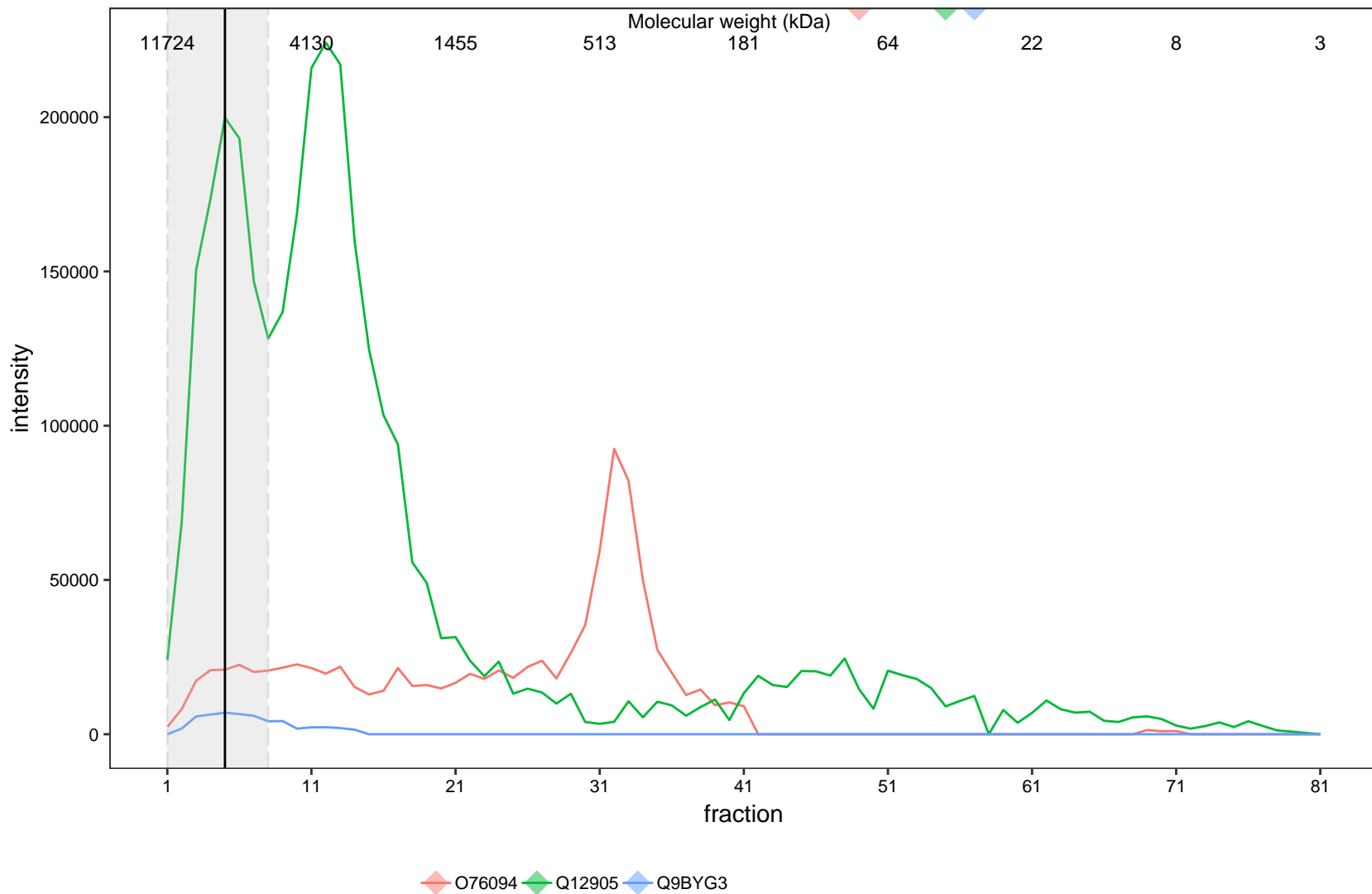

Supplement: Supplementary file 7 — Dataset EV6 [file MSB-15-e8438-s007.zip › feature_plots_bioplex/O76094.pdf]

**O94776**  
**Annotated subunits: 9   Subunits with signal: 3**  
**Max. coeluting subunits: 3   Max. completeness: 0.33**

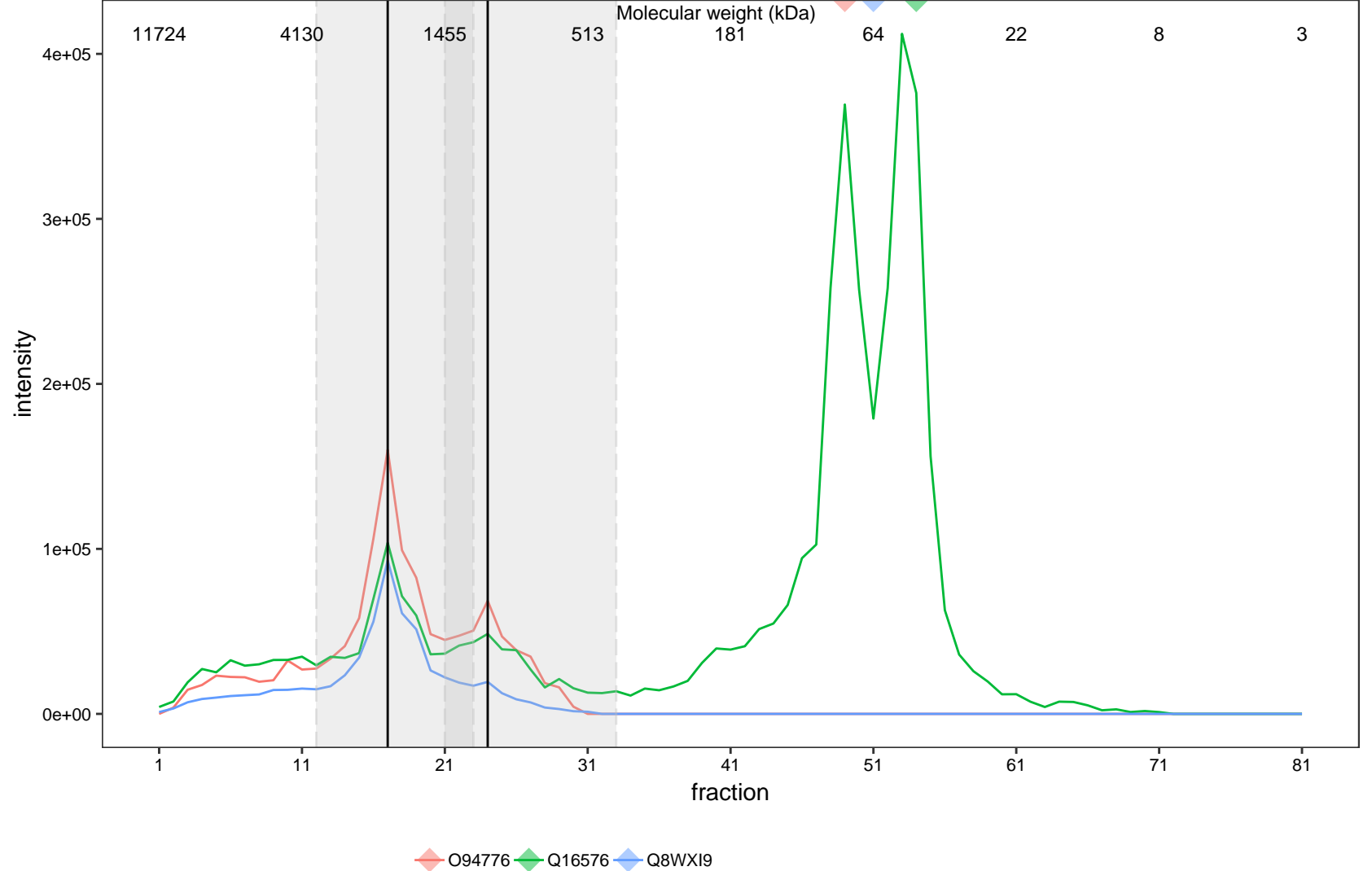

Supplement: Supplementary file 7 — Dataset EV6 [file MSB-15-e8438-s007.zip › feature_plots_bioplex/O94776.pdf]

**O94805**

**Annotated subunits: 8 Subunits with signal: 4**

**Max. coeluting subunits: 4 Max. completeness: 0.5**

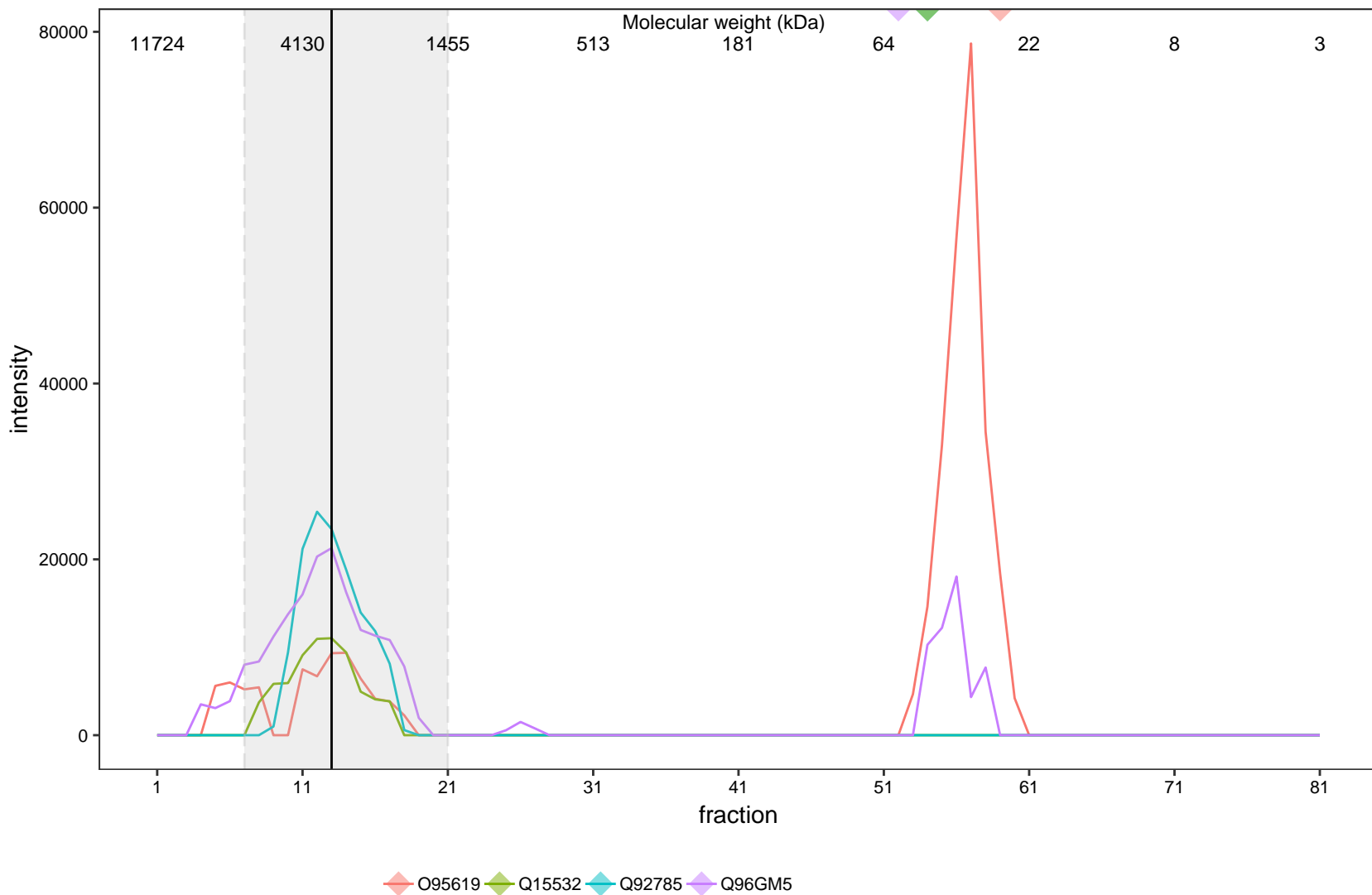

Supplement: Supplementary file 7 — Dataset EV6 [file MSB-15-e8438-s007.zip › feature_plots_bioplex/O94805.pdf]

**O94874**  
**Annotated subunits: 4   Subunits with signal: 3**  
**Max. coeluting subunits: 3   Max. completeness: 0.75**

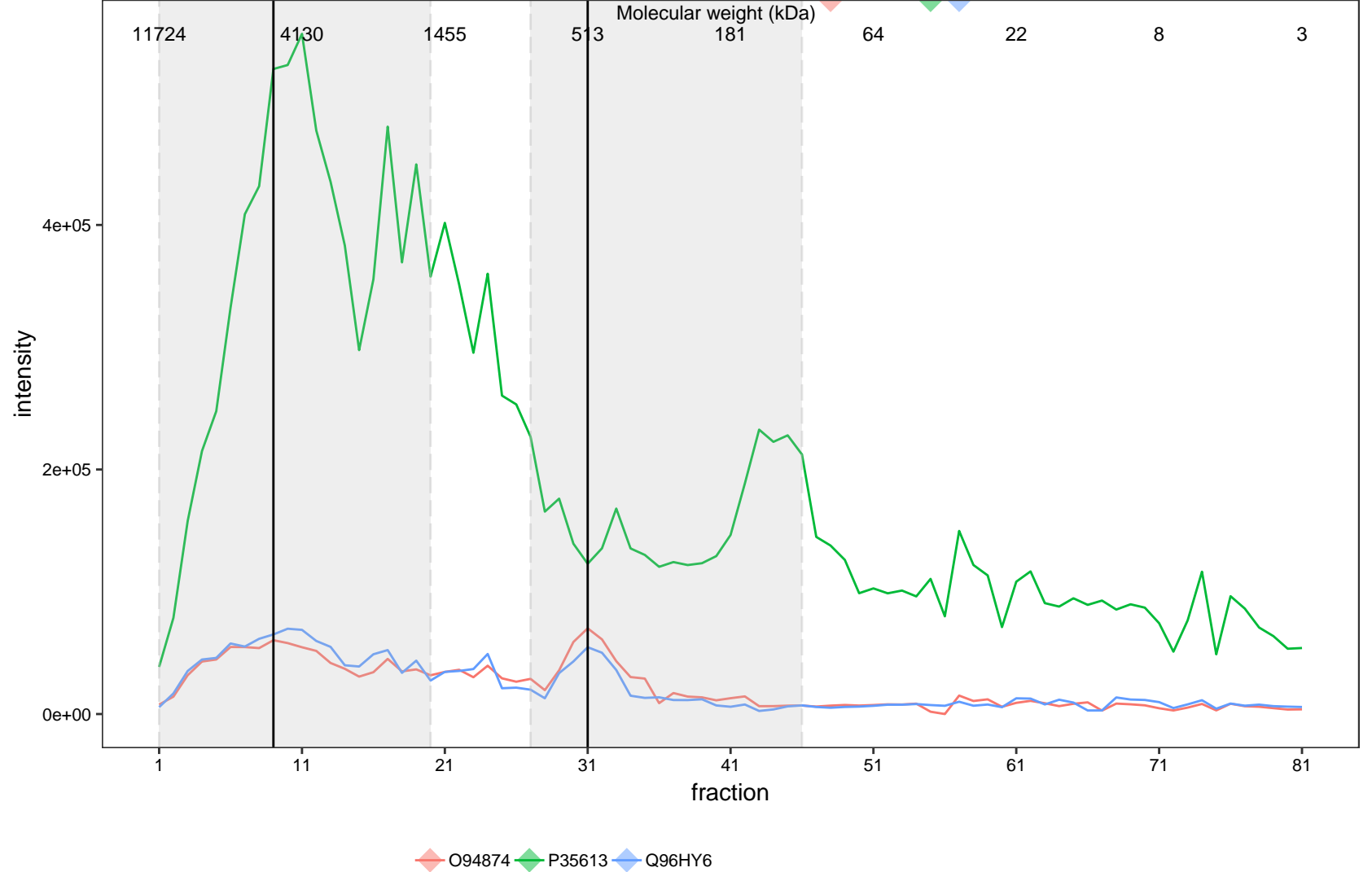

Supplement: Supplementary file 7 — Dataset EV6 [file MSB-15-e8438-s007.zip › feature_plots_bioplex/O94874.pdf]

**O94925**

**Annotated subunits: 14 Subunits with signal: 9**

**Max. coeluting subunits: 2 Max. completeness: 0.14**

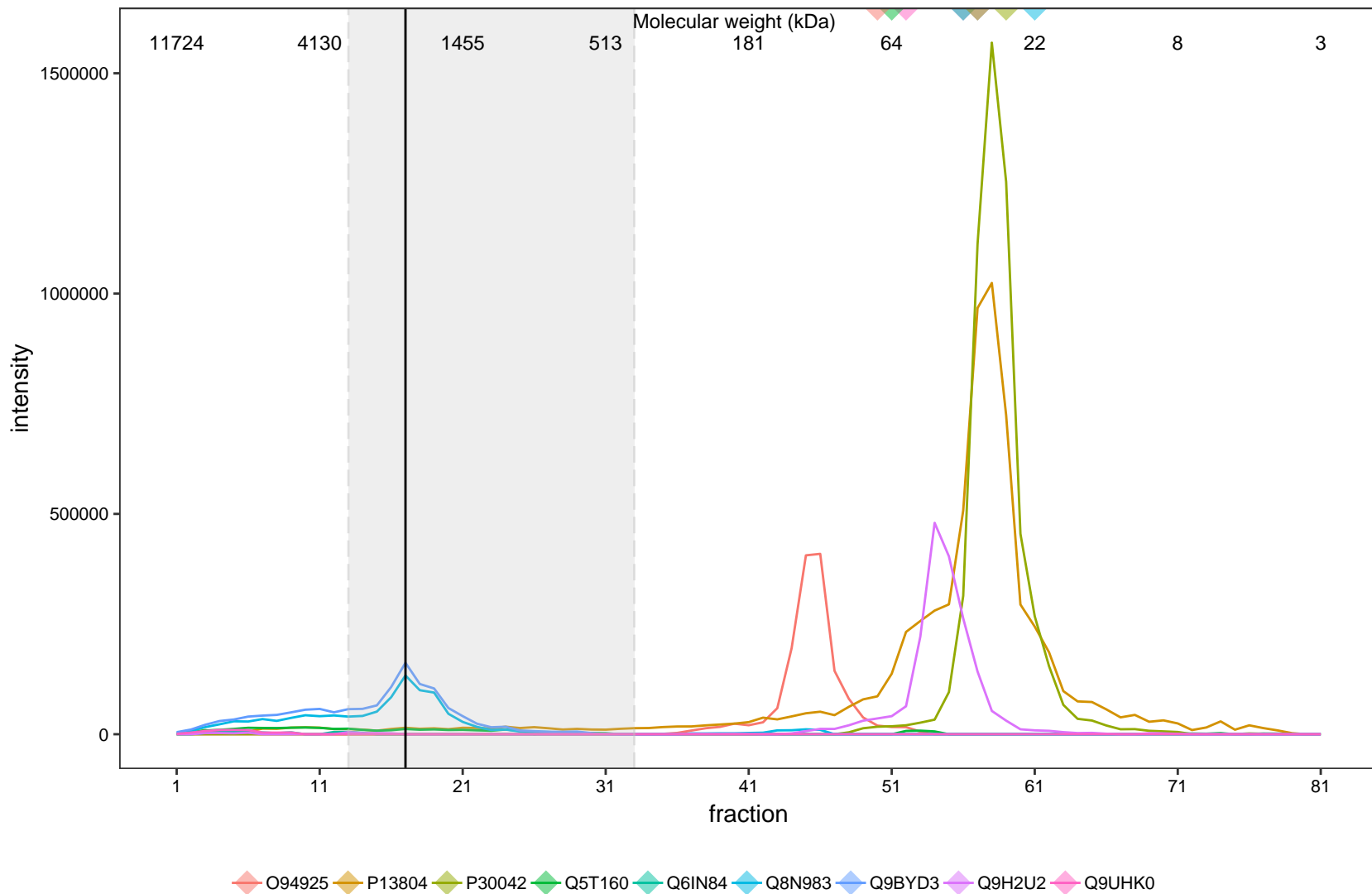

Supplement: Supplementary file 7 — Dataset EV6 [file MSB-15-e8438-s007.zip › feature_plots_bioplex/O94925.pdf]

**O94979**  
**Annotated subunits: 4   Subunits with signal: 4**  
**Max. coeluting subunits: 2   Max. completeness: 0.5**

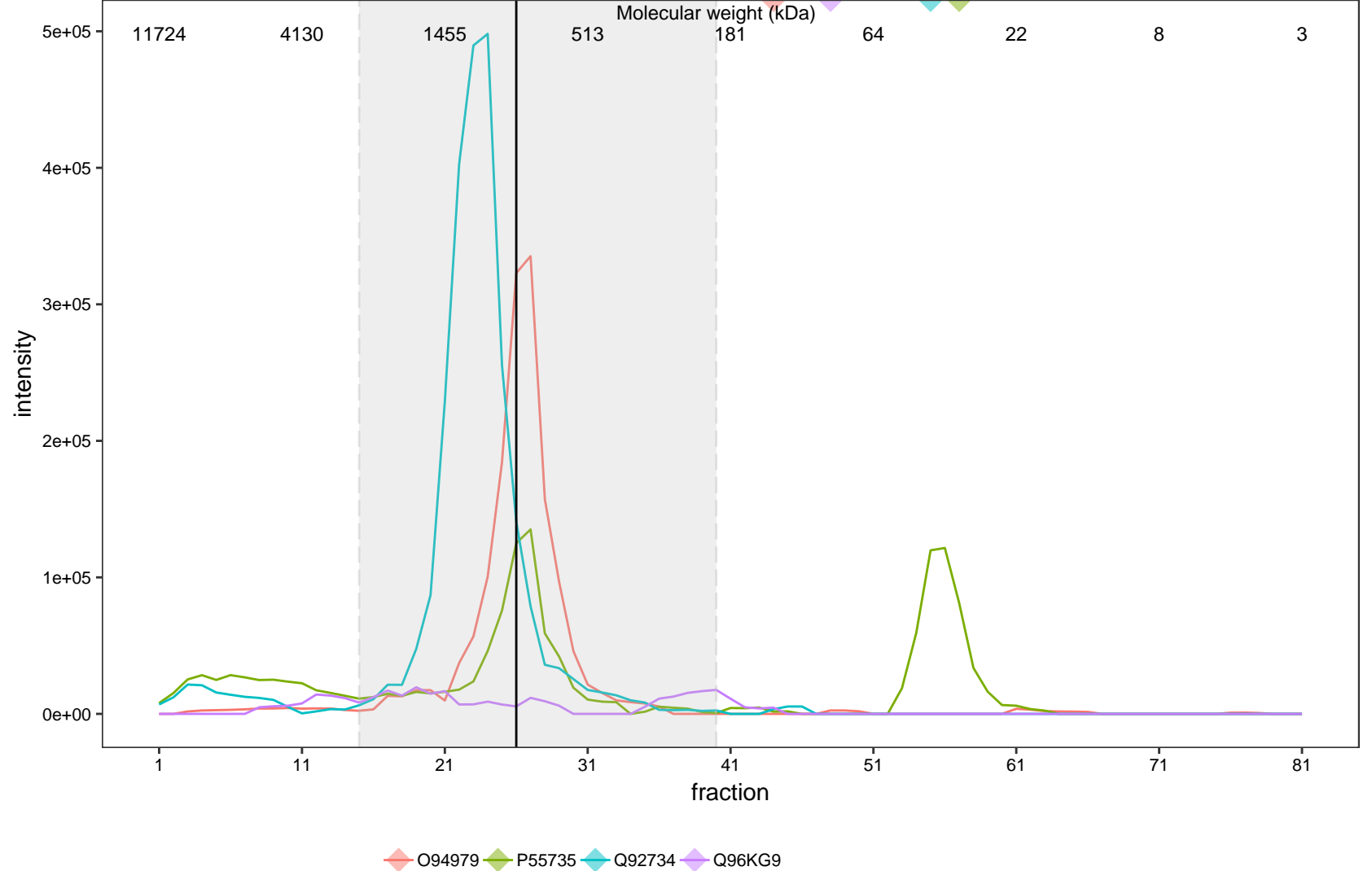

Supplement: Supplementary file 7 — Dataset EV6 [file MSB-15-e8438-s007.zip › feature_plots_bioplex/O94979.pdf]

**O95059**  
**Annotated subunits: 3   Subunits with signal: 2**  
**Max. coeluting subunits: 2   Max. completeness: 0.67**

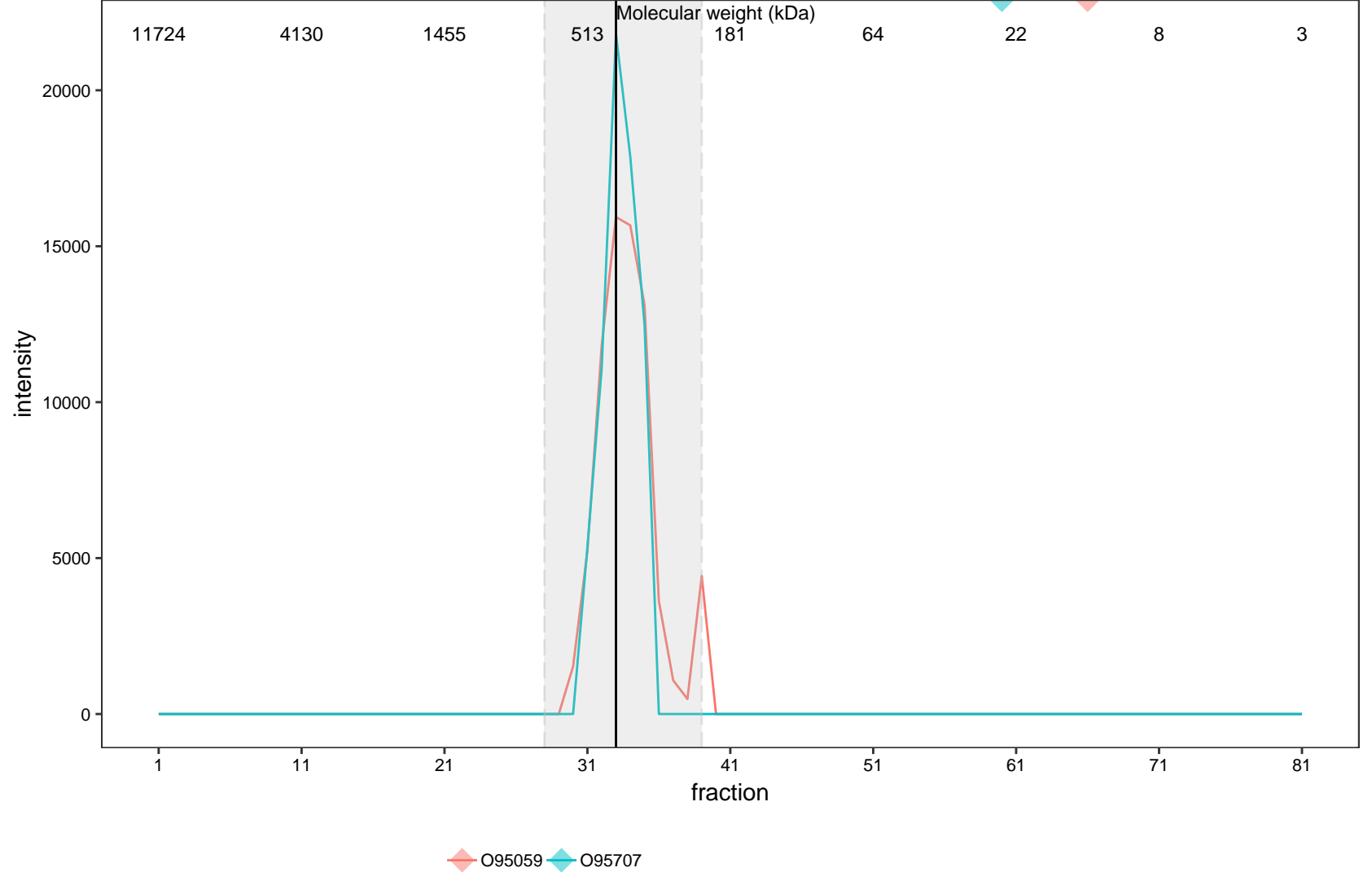

Supplement: Supplementary file 7 — Dataset EV6 [file MSB-15-e8438-s007.zip › feature_plots_bioplex/O95059.pdf]

O95067

Annotated subunits: 6 Subunits with signal: 5

Max. coeluting subunits: 3 Max. completeness: 0.5

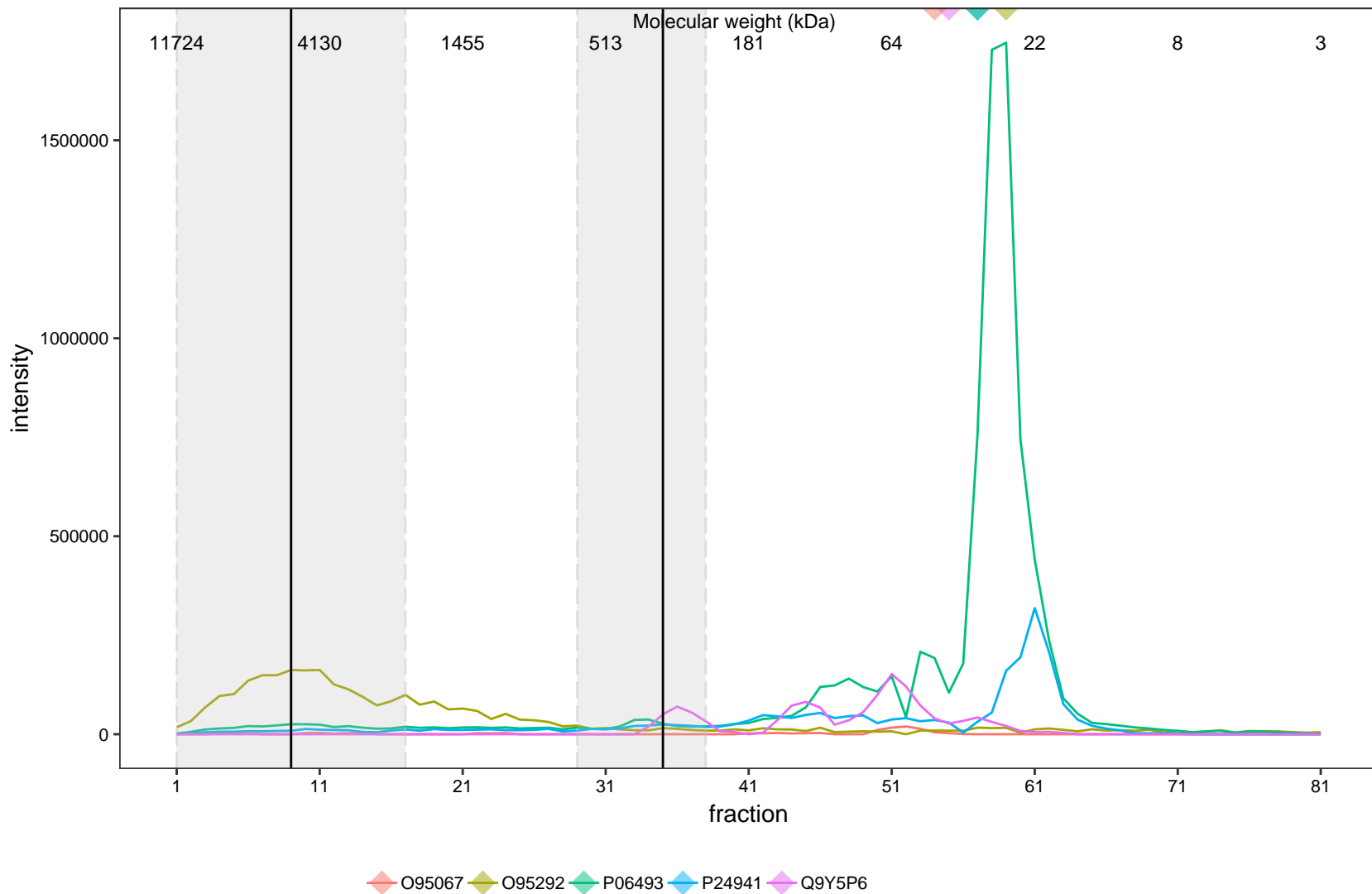

Supplement: Supplementary file 7 — Dataset EV6 [file MSB-15-e8438-s007.zip › feature_plots_bioplex/O95067.pdf]

**O95139**

**Annotated subunits: 3 Subunits with signal: 3**

**Max. coeluting subunits: 2 Max. completeness: 0.67**

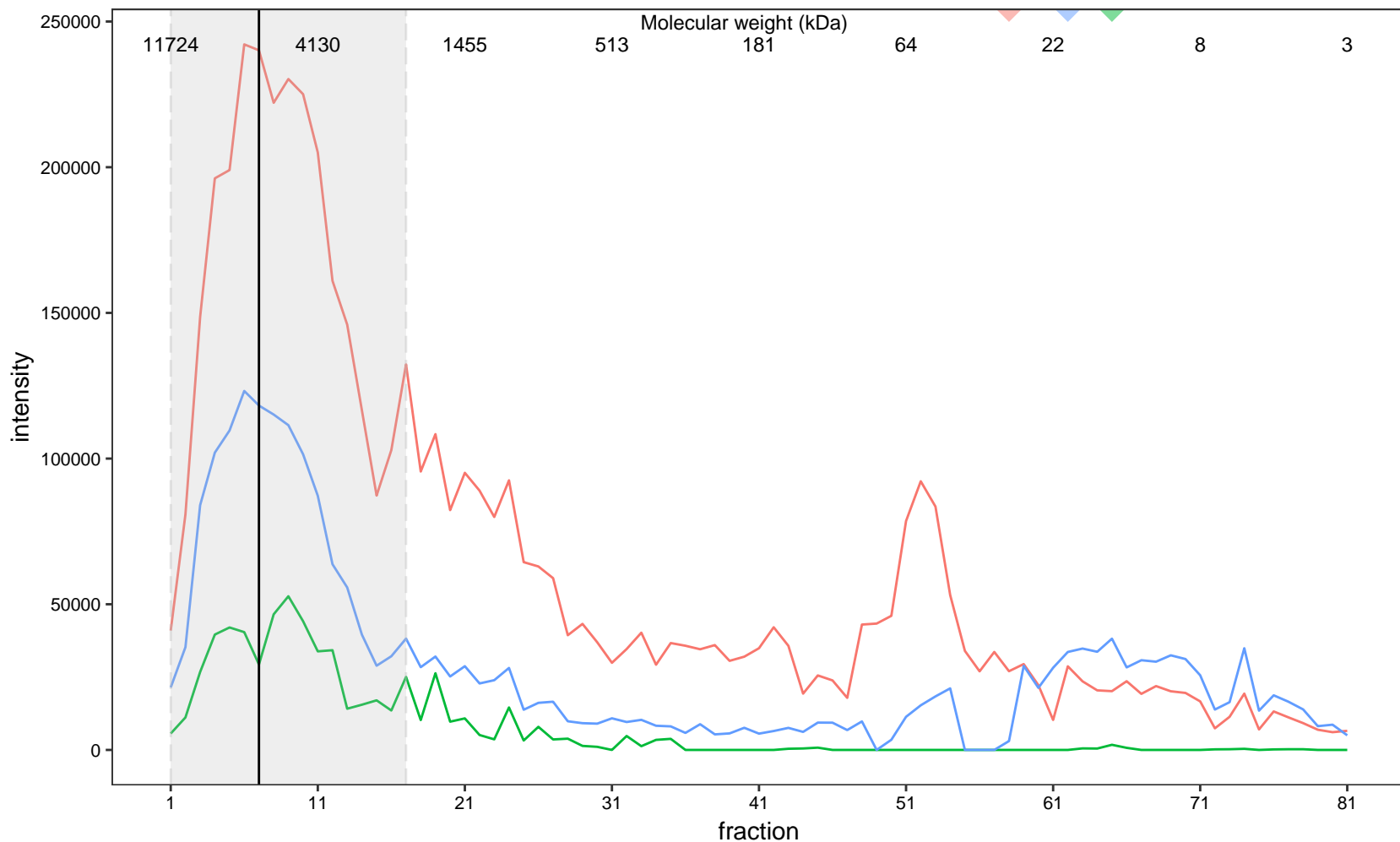

◊ O75489 ◊ O95139 ◊ P51970

Supplement: Supplementary file 7 — Dataset EV6 [file MSB-15-e8438-s007.zip › feature_plots_bioplex/O95139.pdf]

**O95167**

**Annotated subunits: 9 Subunits with signal: 4**

**Max. coeluting subunits: 4 Max. completeness: 0.44**

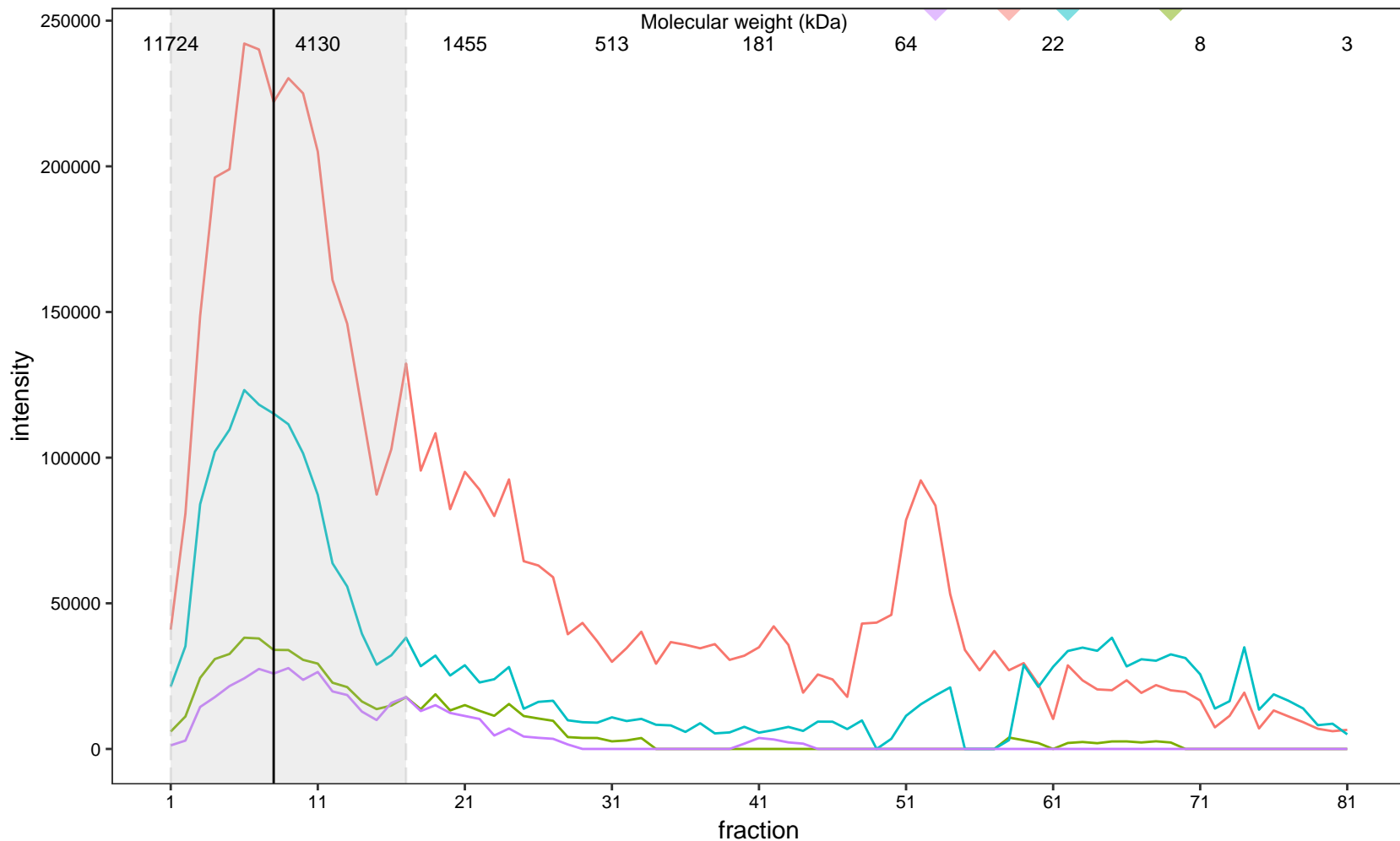

◊ O75489 ◊ O95167 ◊ P51970 ◊ Q9BQ95

Supplement: Supplementary file 7 — Dataset EV6 [file MSB-15-e8438-s007.zip › feature_plots_bioplex/O95167.pdf]

**O95168**

**Annotated subunits: 4 Subunits with signal: 3**

**Max. coeluting subunits: 3 Max. completeness: 0.75**

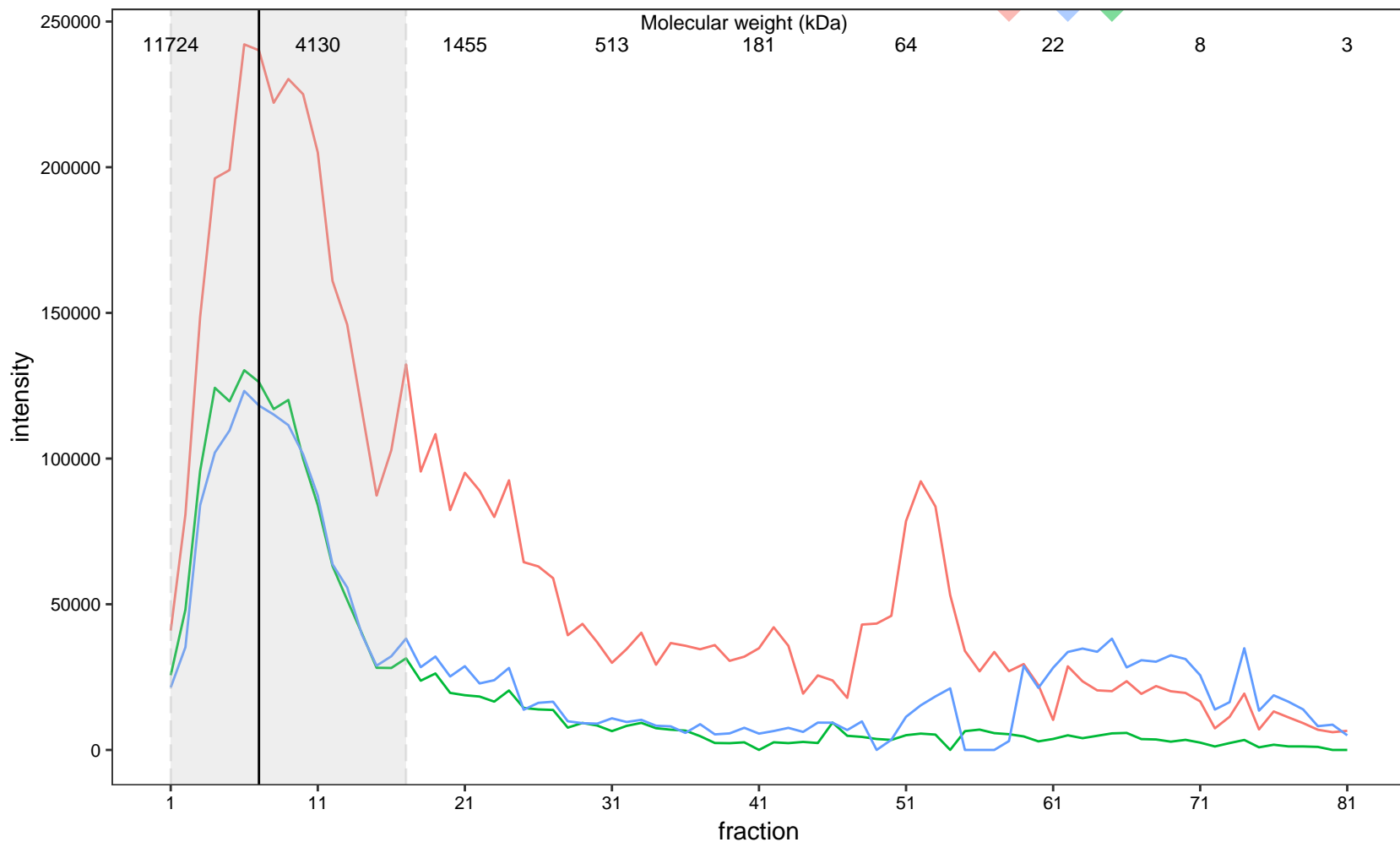

◊ O75489 ◊ O95168 ◊ P51970

Supplement: Supplementary file 7 — Dataset EV6 [file MSB-15-e8438-s007.zip › feature_plots_bioplex/O95168.pdf]

**O95169**

**Annotated subunits: 8 Subunits with signal: 5**

**Max. coeluting subunits: 5 Max. completeness: 0.62**

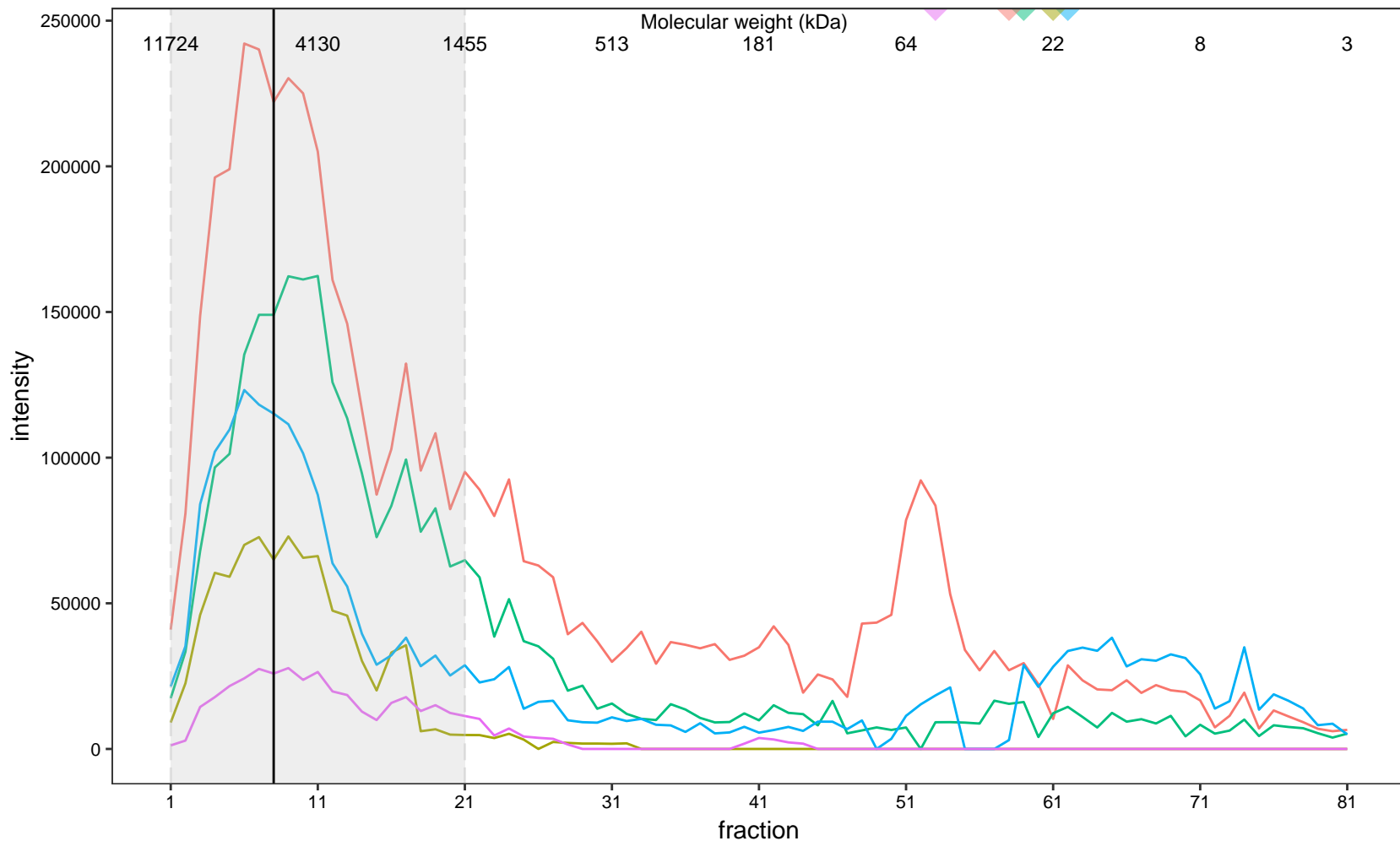

Supplement: Supplementary file 7 — Dataset EV6 [file MSB-15-e8438-s007.zip › feature_plots_bioplex/O95169.pdf]

**O95182**

**Annotated subunits: 8 Subunits with signal: 5**

**Max. coeluting subunits: 5 Max. completeness: 0.62**

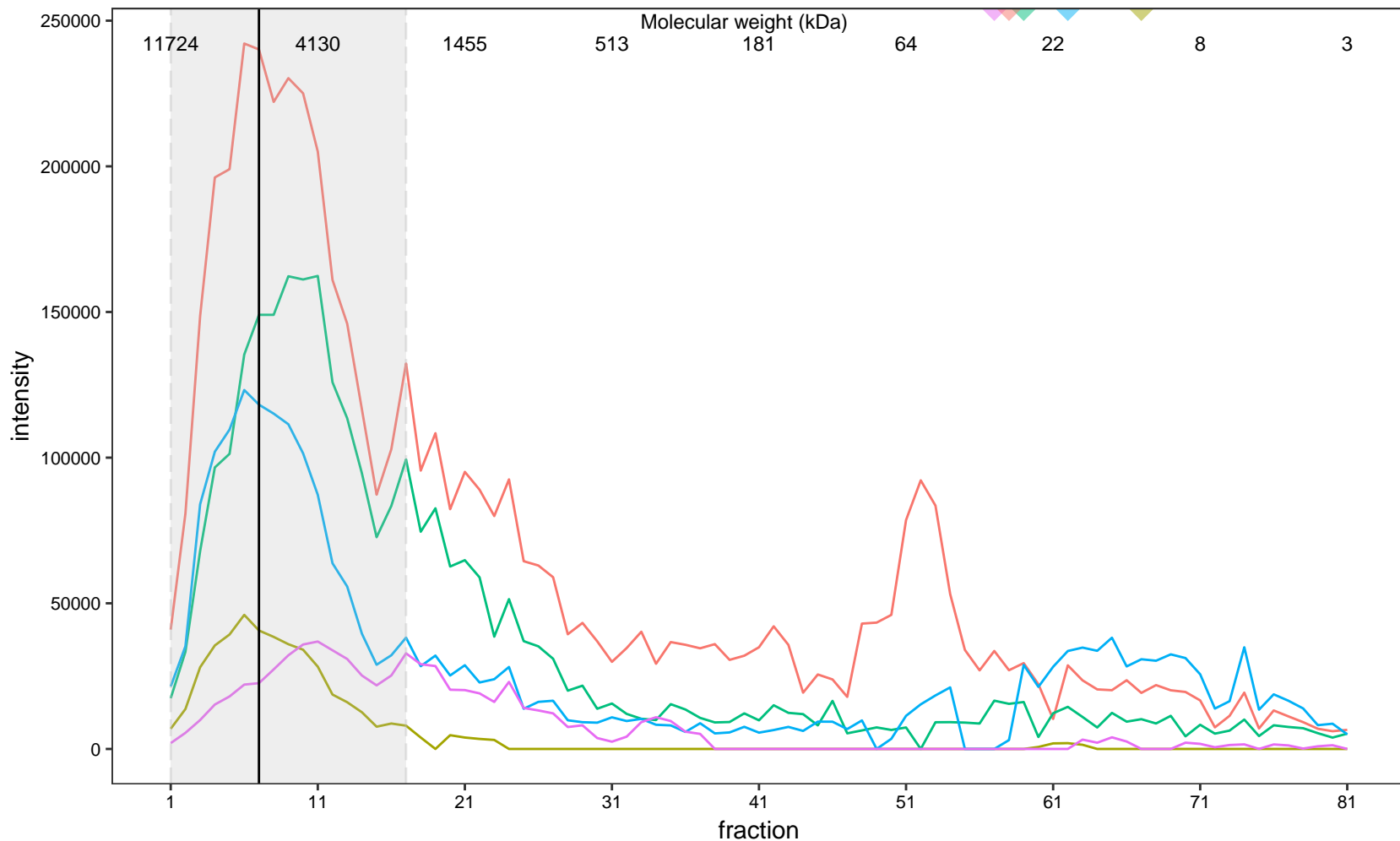

Supplement: Supplementary file 7 — Dataset EV6 [file MSB-15-e8438-s007.zip › feature_plots_bioplex/O95182.pdf]

**O95249**

**Annotated subunits: 7 Subunits with signal: 5**

**Max. coeluting subunits: 3 Max. completeness: 0.43**

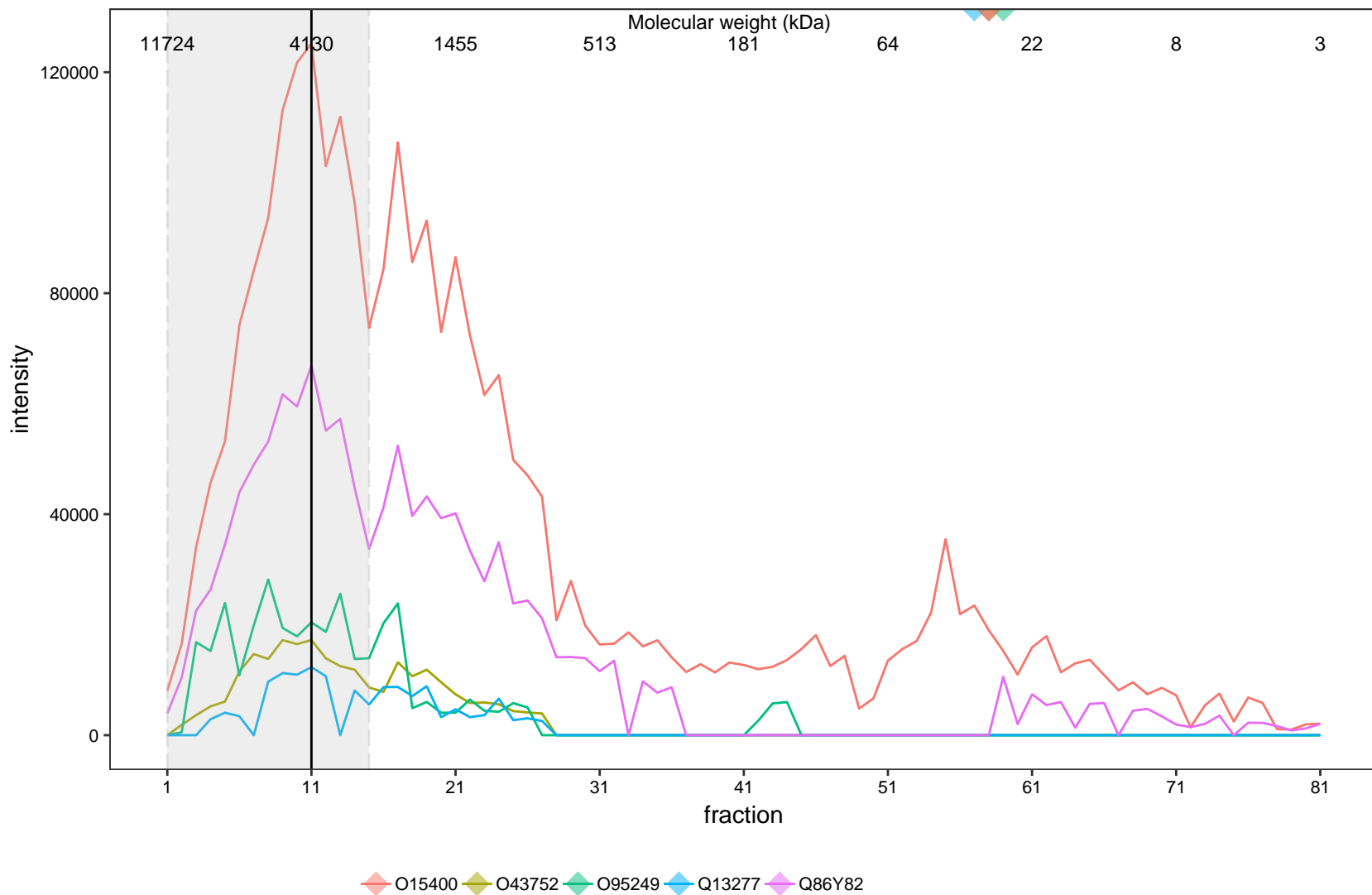

Supplement: Supplementary file 7 — Dataset EV6 [file MSB-15-e8438-s007.zip › feature_plots_bioplex/O95249.pdf]

**O95400**

**Annotated subunits: 4 Subunits with signal: 4**

**Max. coeluting subunits: 4 Max. completeness: 1**

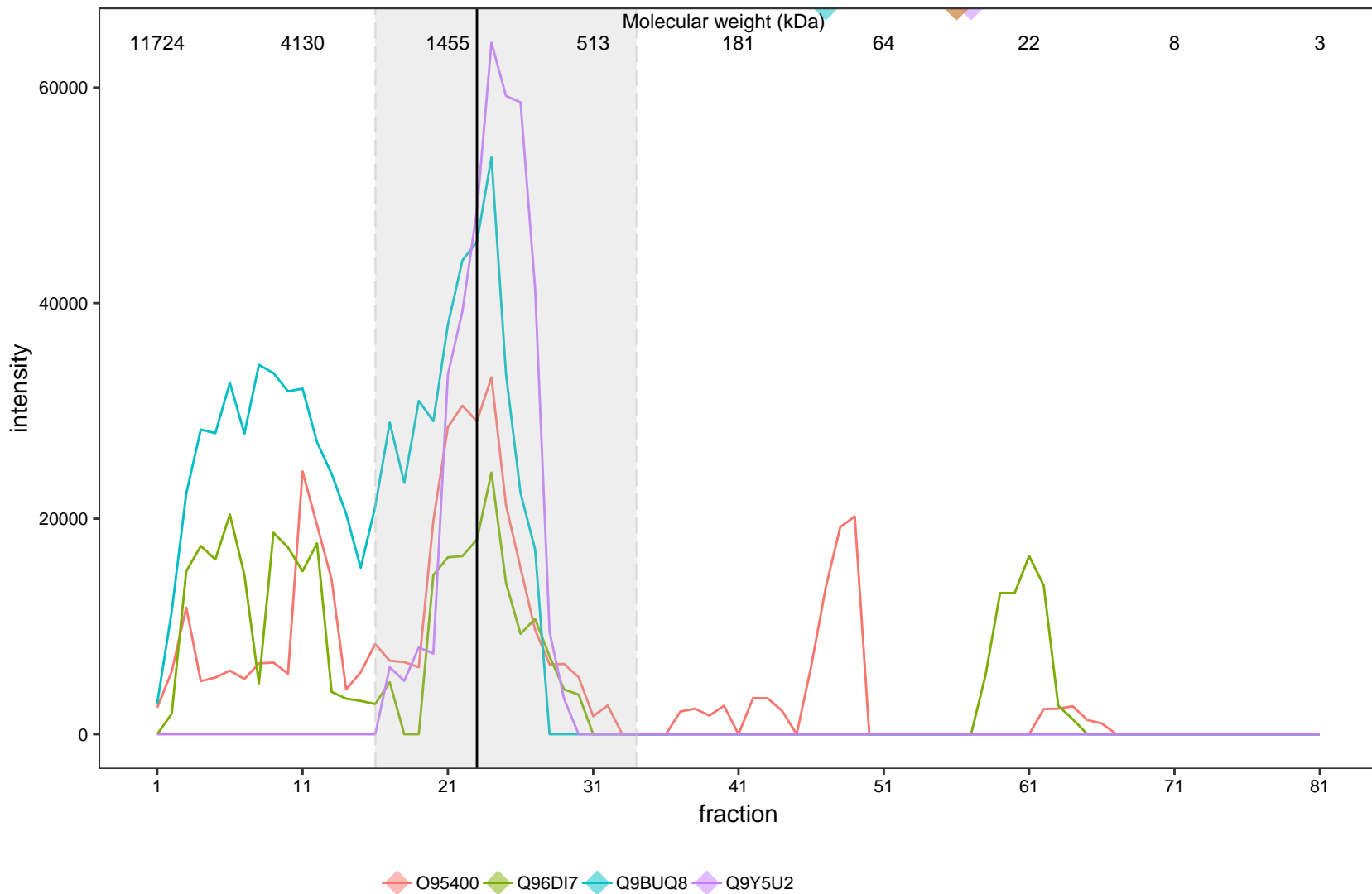

Supplement: Supplementary file 7 — Dataset EV6 [file MSB-15-e8438-s007.zip › feature_plots_bioplex/O95400.pdf]

**O95707**  
**Annotated subunits: 17   Subunits with signal: 9**  
**Max. coeluting subunits: 8   Max. completeness: 0.47**

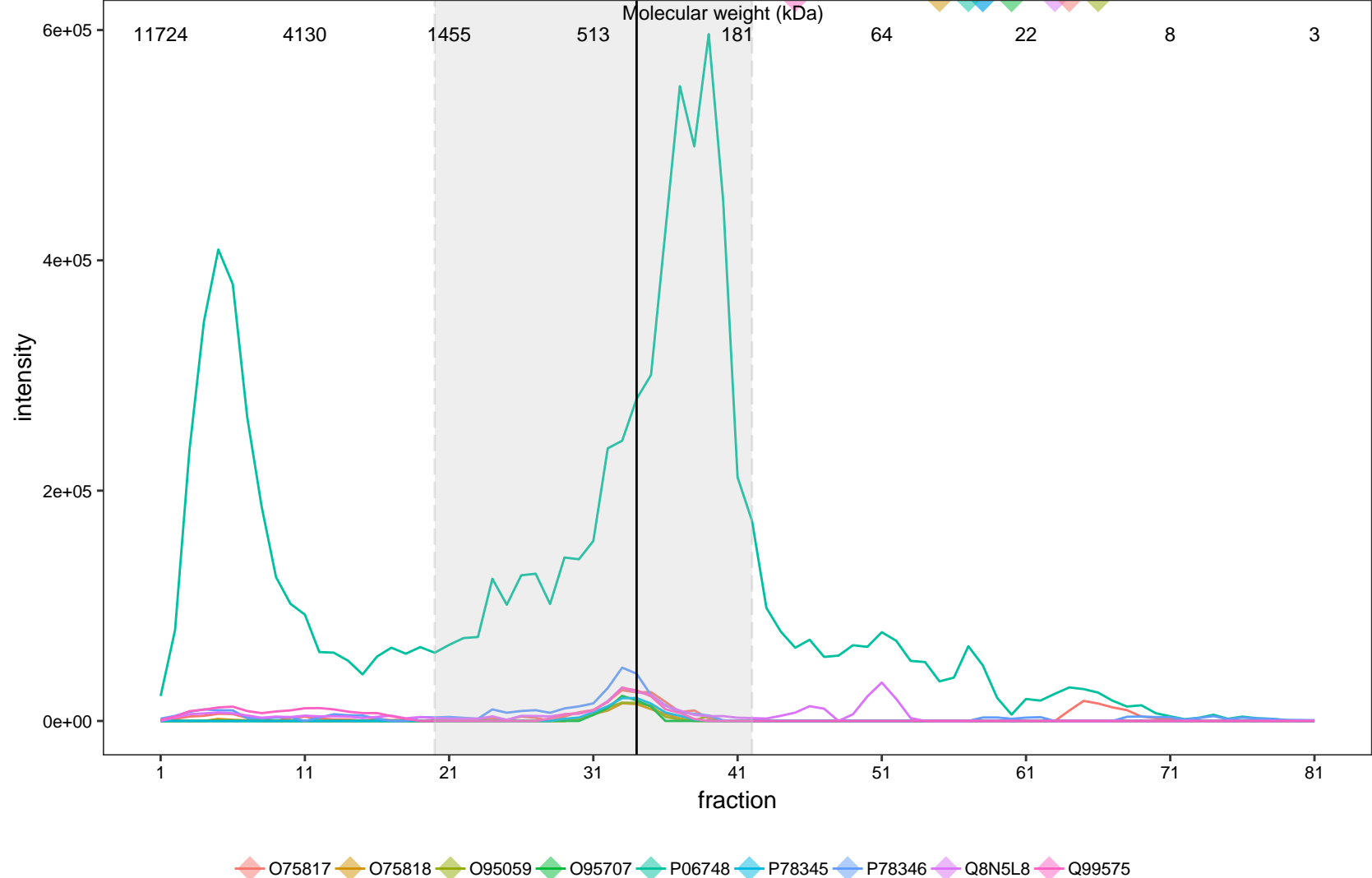

Supplement: Supplementary file 7 — Dataset EV6 [file MSB-15-e8438-s007.zip › feature_plots_bioplex/O95707.pdf]

**O95793**  
**Annotated subunits: 10   Subunits with signal: 5**  
**Max. coeluting subunits: 3   Max. completeness: 0.3**

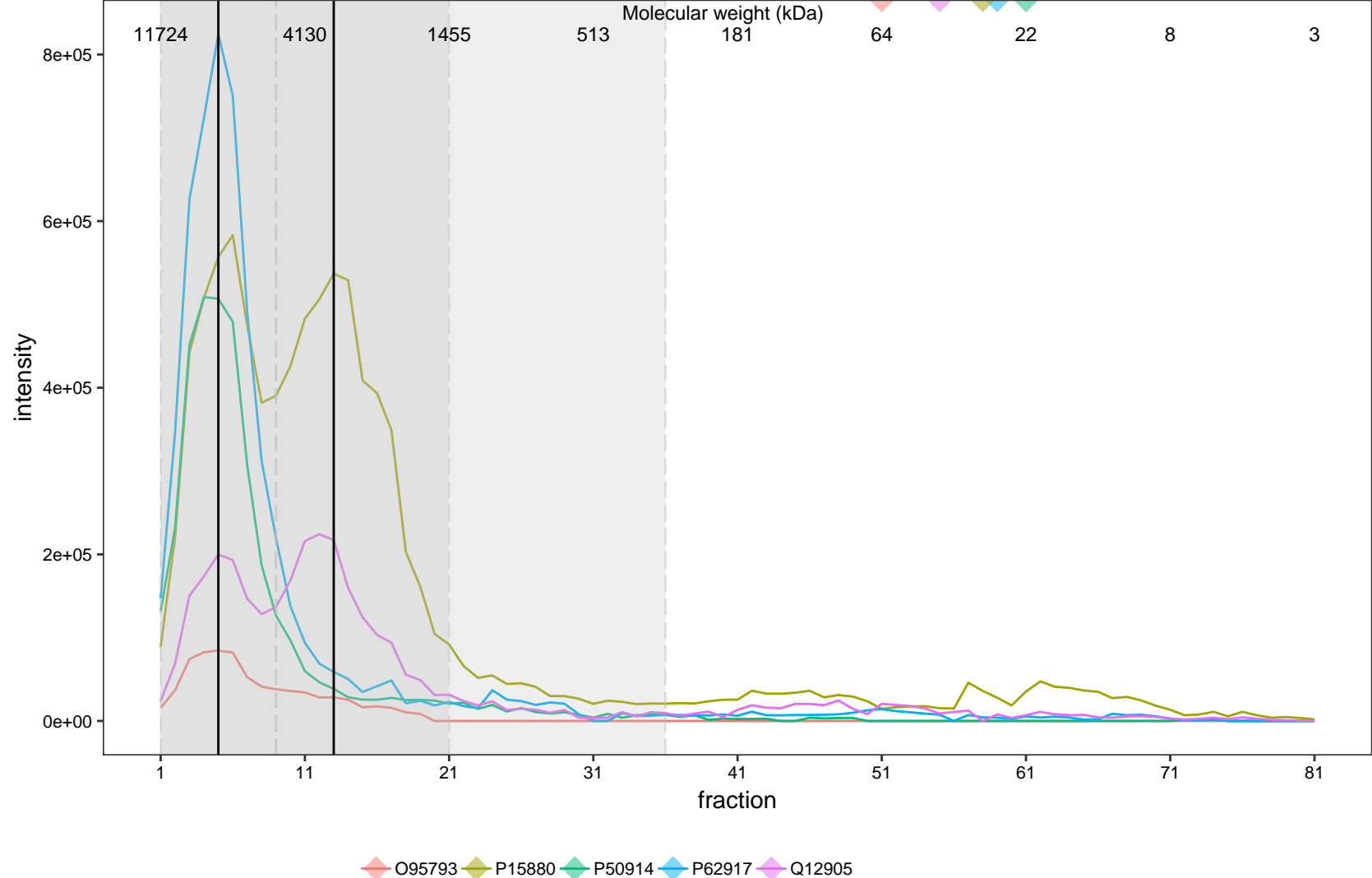

Supplement: Supplementary file 7 — Dataset EV6 [file MSB-15-e8438-s007.zip › feature_plots_bioplex/O95793.pdf]

**O95825**

**Annotated subunits: 3 Subunits with signal: 3**

**Max. coeluting subunits: 2 Max. completeness: 0.67**

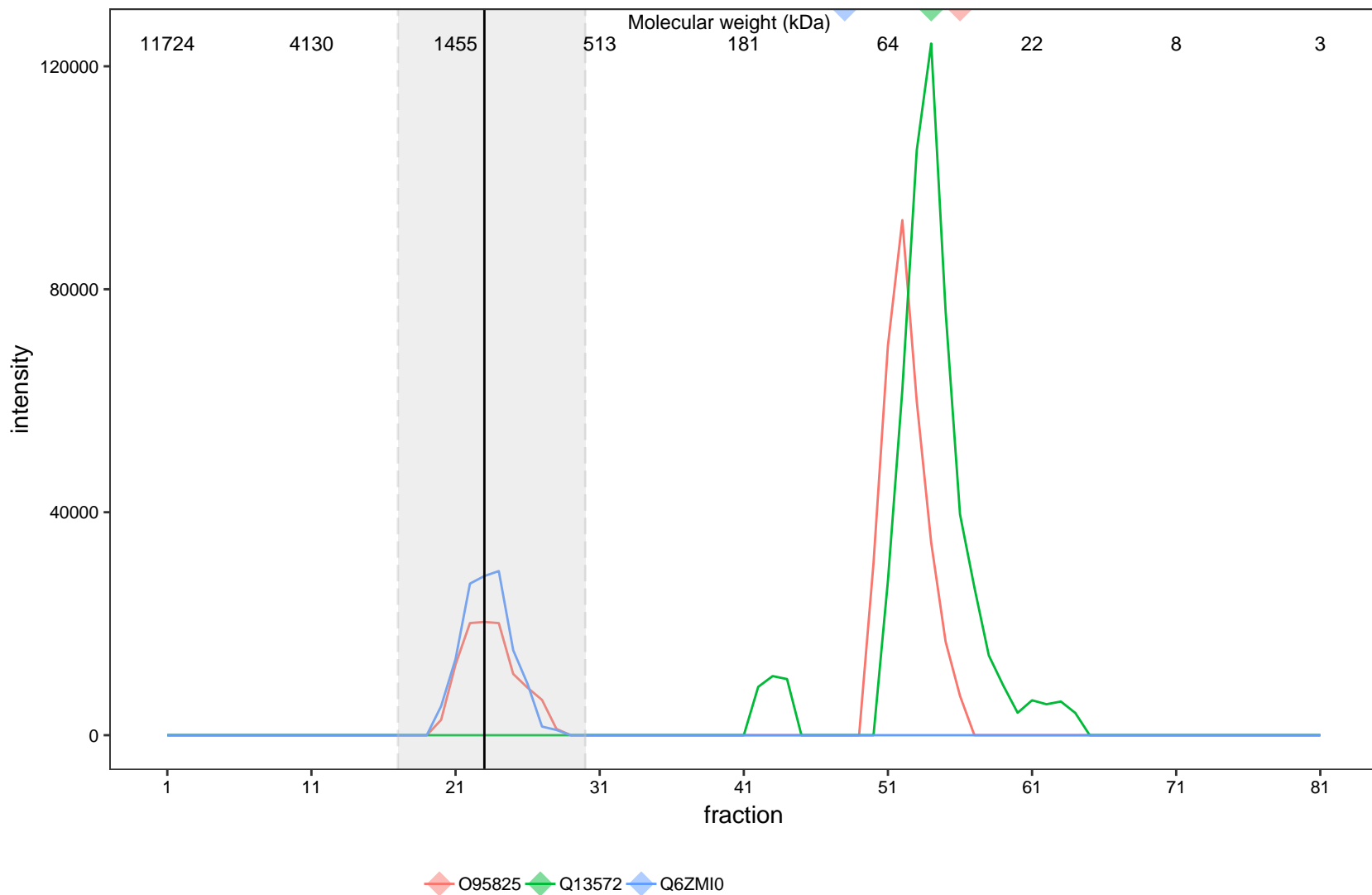

Supplement: Supplementary file 7 — Dataset EV6 [file MSB-15-e8438-s007.zip › feature_plots_bioplex/O95825.pdf]

**O95863**

**Annotated subunits: 10 Subunits with signal: 6**

**Max. coeluting subunits: 4 Max. completeness: 0.4**

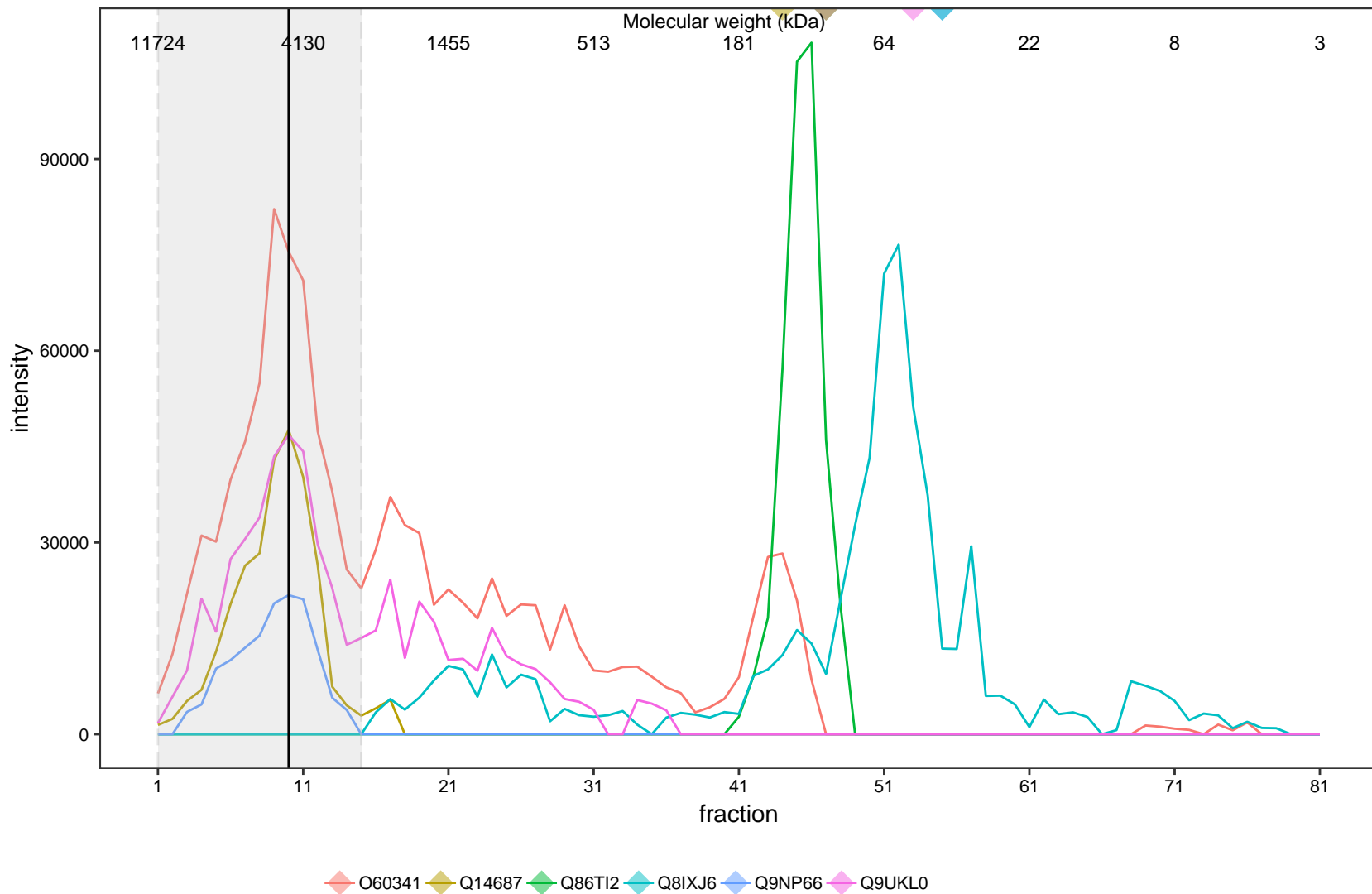

Supplement: Supplementary file 7 — Dataset EV6 [file MSB-15-e8438-s007.zip › feature_plots_bioplex/O95863.pdf]

**O95864**

**Annotated subunits: 3 Subunits with signal: 2**

**Max. coeluting subunits: 2 Max. completeness: 0.67**

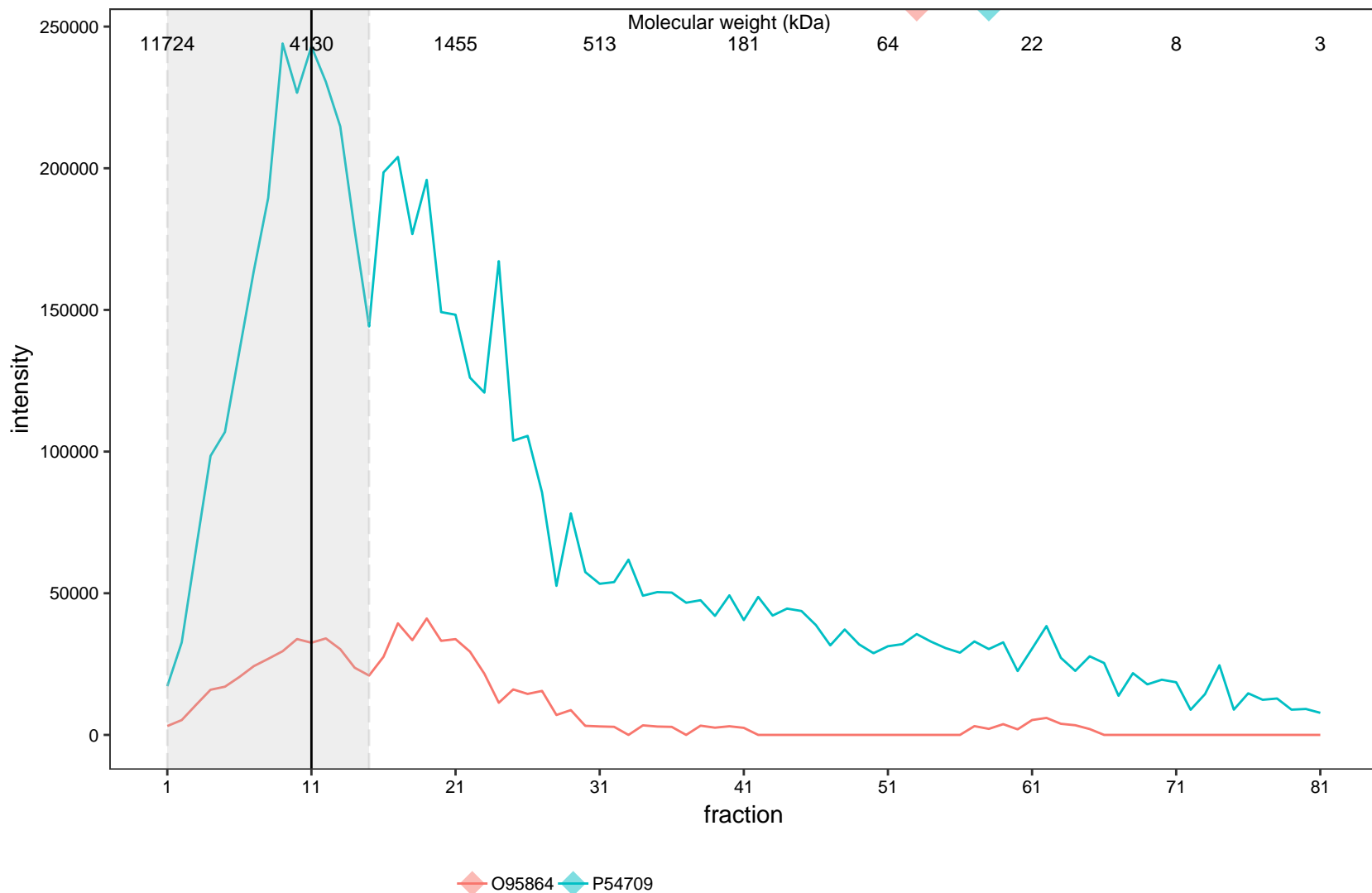

Supplement: Supplementary file 7 — Dataset EV6 [file MSB-15-e8438-s007.zip › feature_plots_bioplex/O95864.pdf]

**O95983**  
**Annotated subunits: 8   Subunits with signal: 3**  
**Max. coeluting subunits: 3   Max. completeness: 0.38**

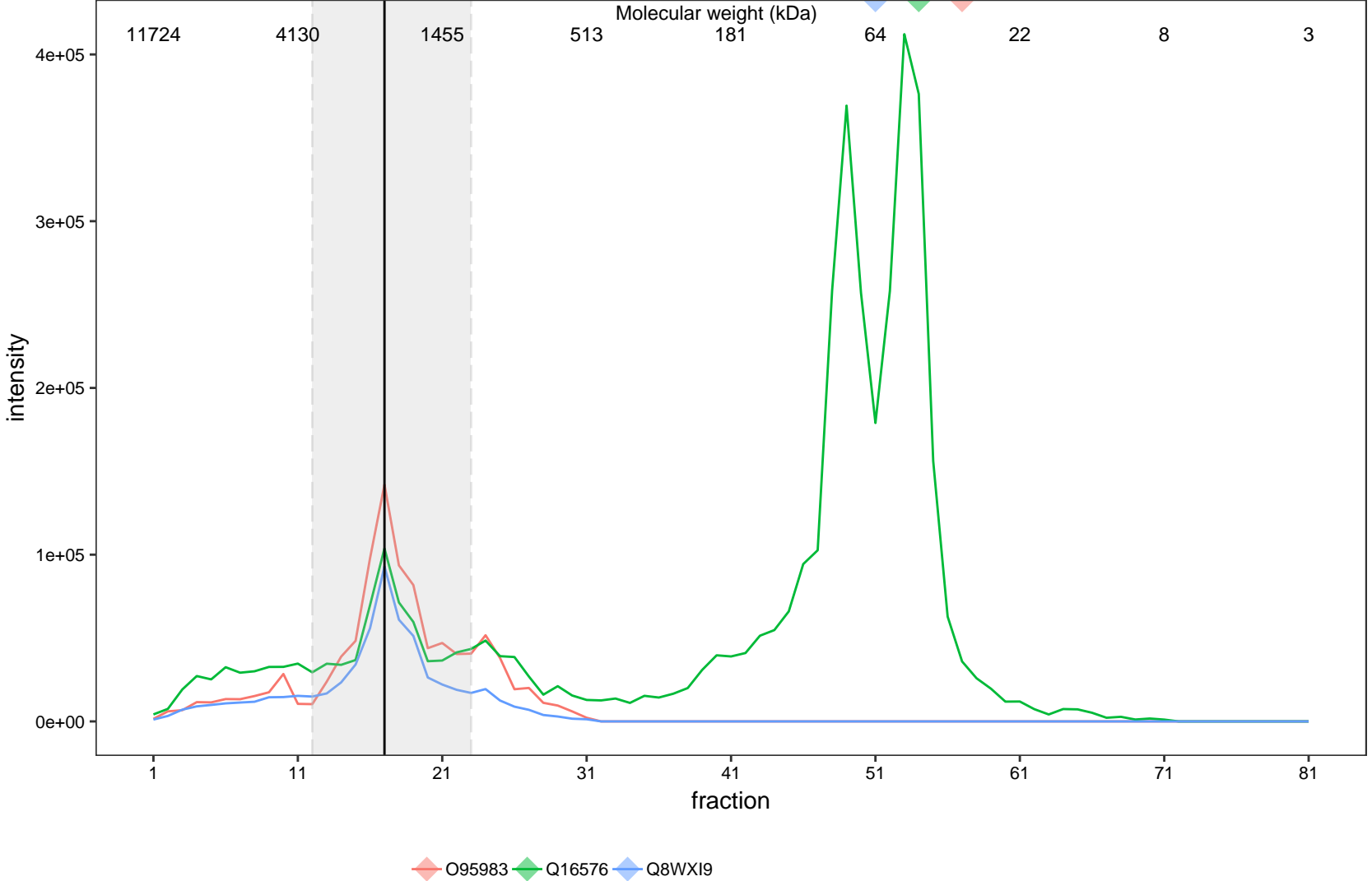

Supplement: Supplementary file 7 — Dataset EV6 [file MSB-15-e8438-s007.zip › feature_plots_bioplex/O95983.pdf]

**O96000**

**Annotated subunits: 2 Subunits with signal: 2**

**Max. coeluting subunits: 2 Max. completeness: 1**

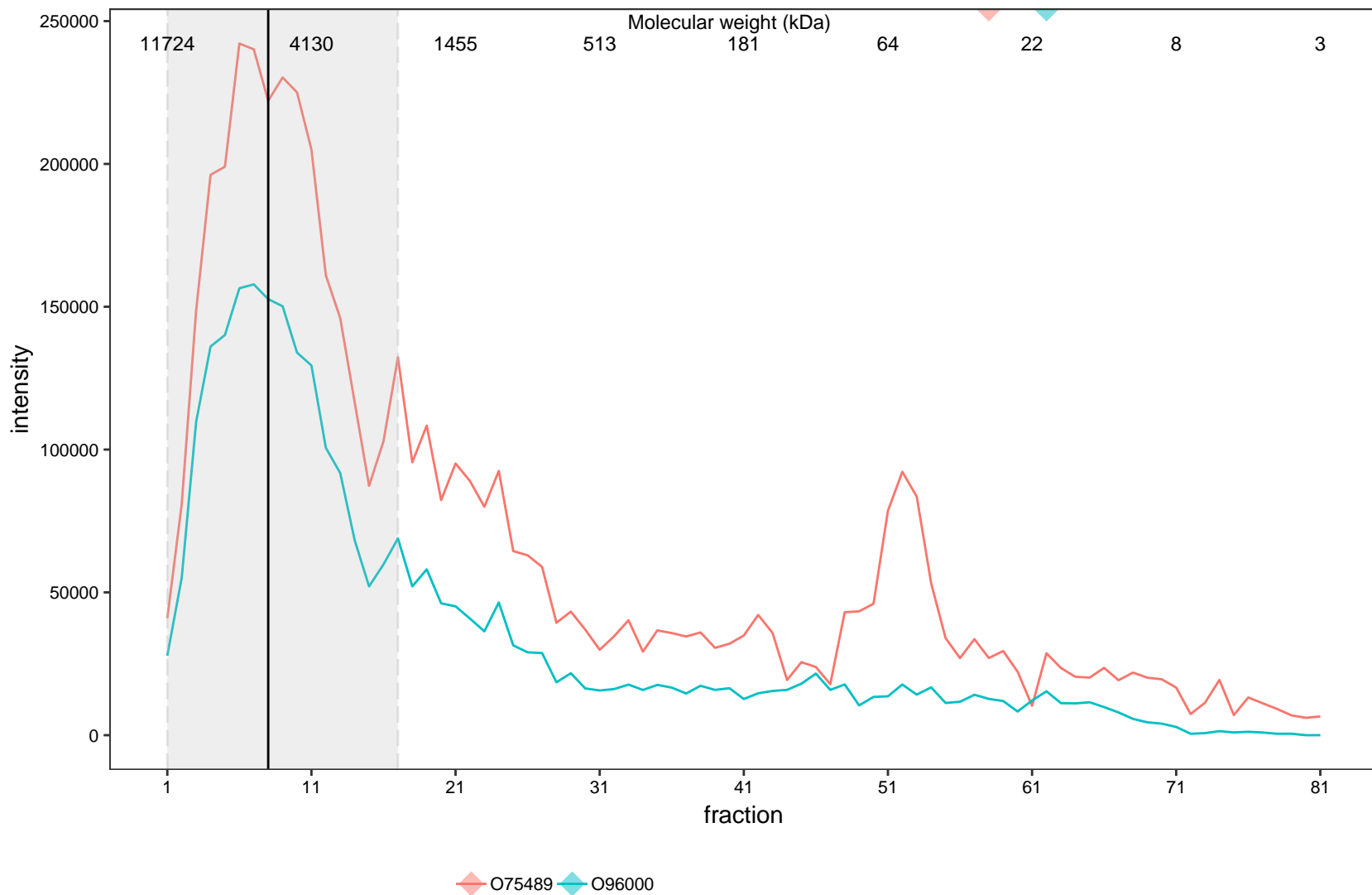

Supplement: Supplementary file 7 — Dataset EV6 [file MSB-15-e8438-s007.zip › feature_plots_bioplex/O96000.pdf]

**O96005**

**Annotated subunits: 2 Subunits with signal: 2**

**Max. coeluting subunits: 2 Max. completeness: 1**

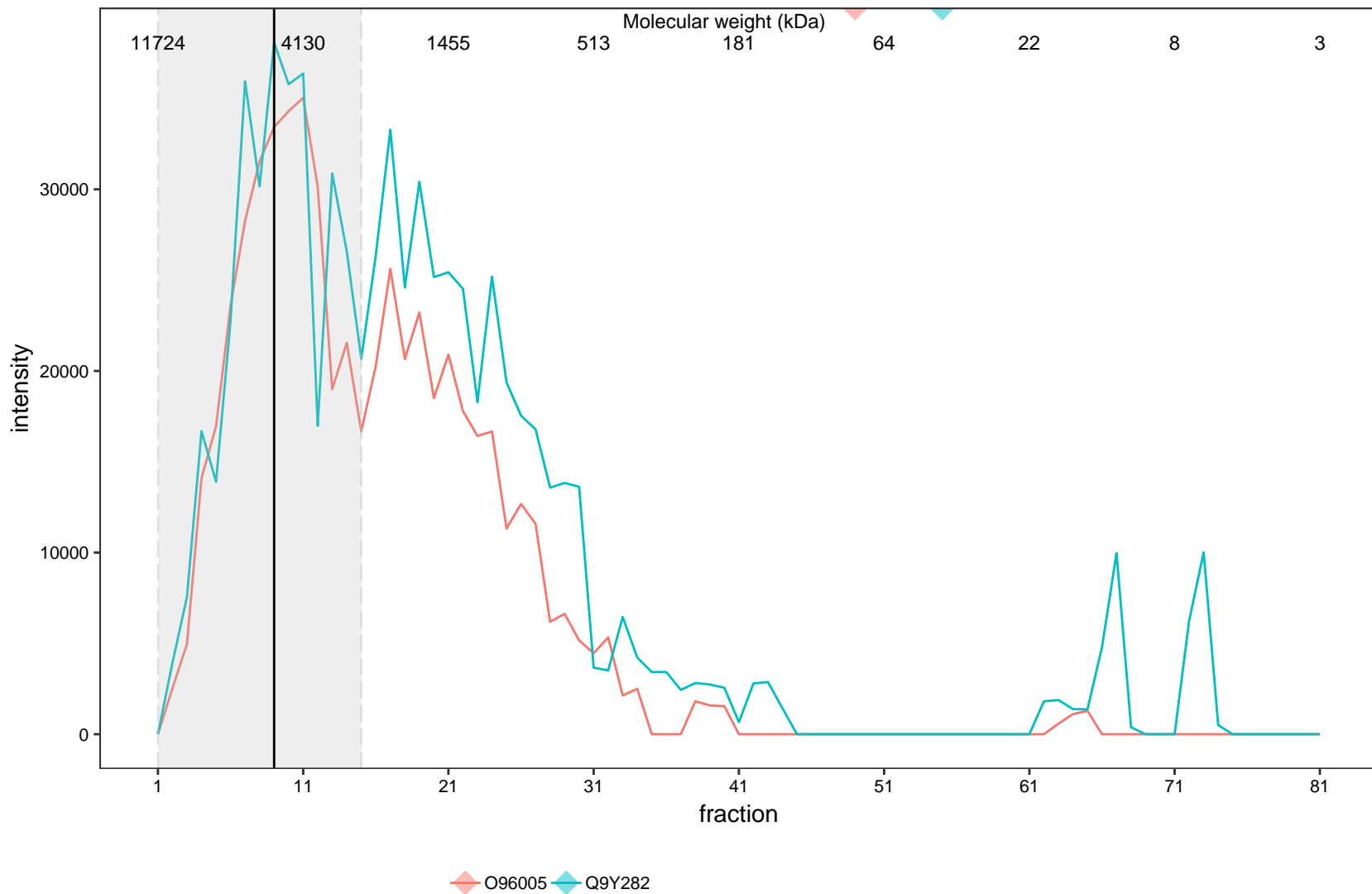

Supplement: Supplementary file 7 — Dataset EV6 [file MSB-15-e8438-s007.zip › feature_plots_bioplex/O96005.pdf]

O96028  
Annotated subunits: 6   Subunits with signal: 2  
Max. coeluting subunits: 2   Max. completeness: 0.33

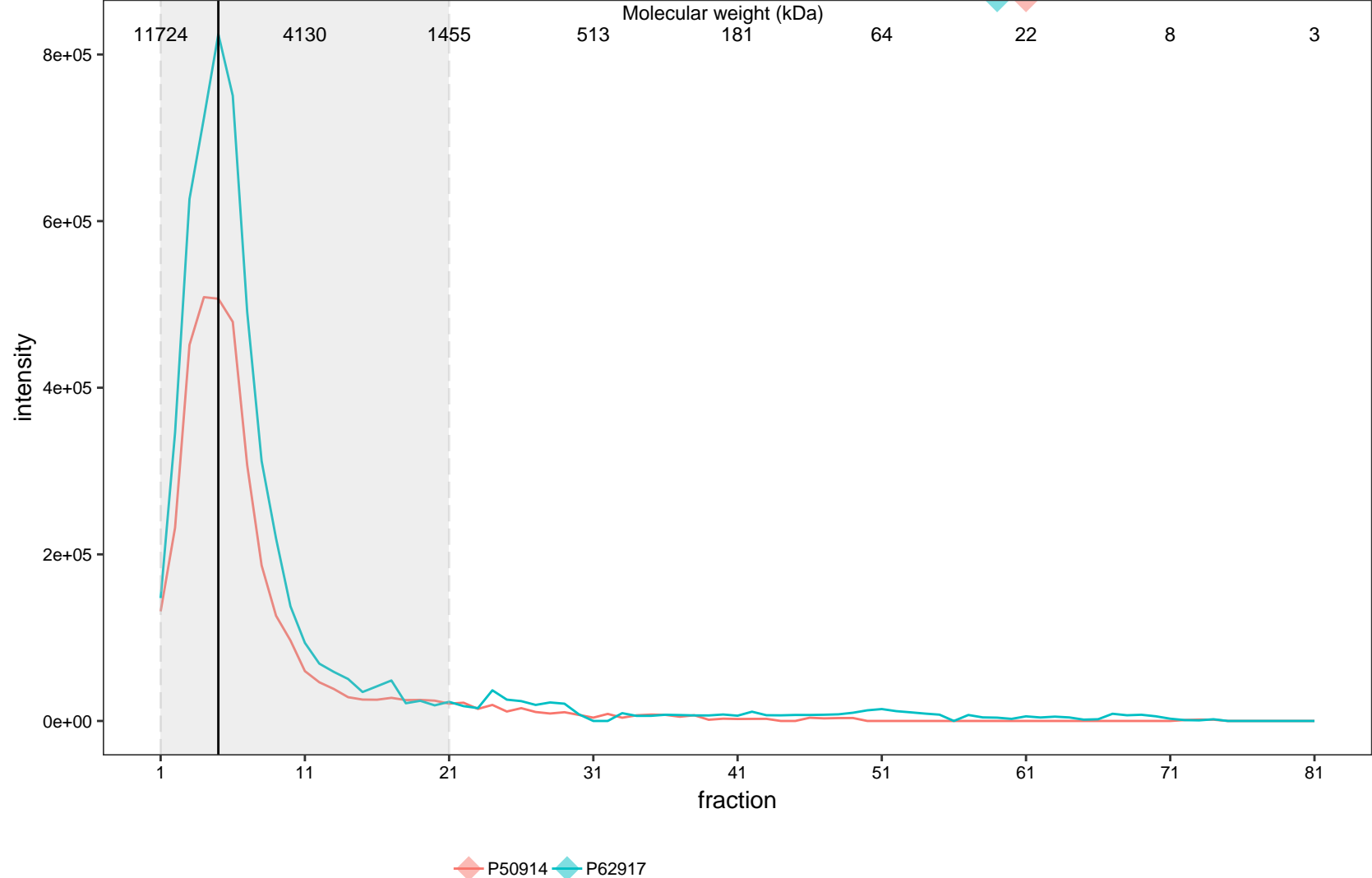

Supplement: Supplementary file 7 — Dataset EV6 [file MSB-15-e8438-s007.zip › feature_plots_bioplex/O96028.pdf]

**P00387**

**Annotated subunits: 8 Subunits with signal: 4**

**Max. coeluting subunits: 3 Max. completeness: 0.38**

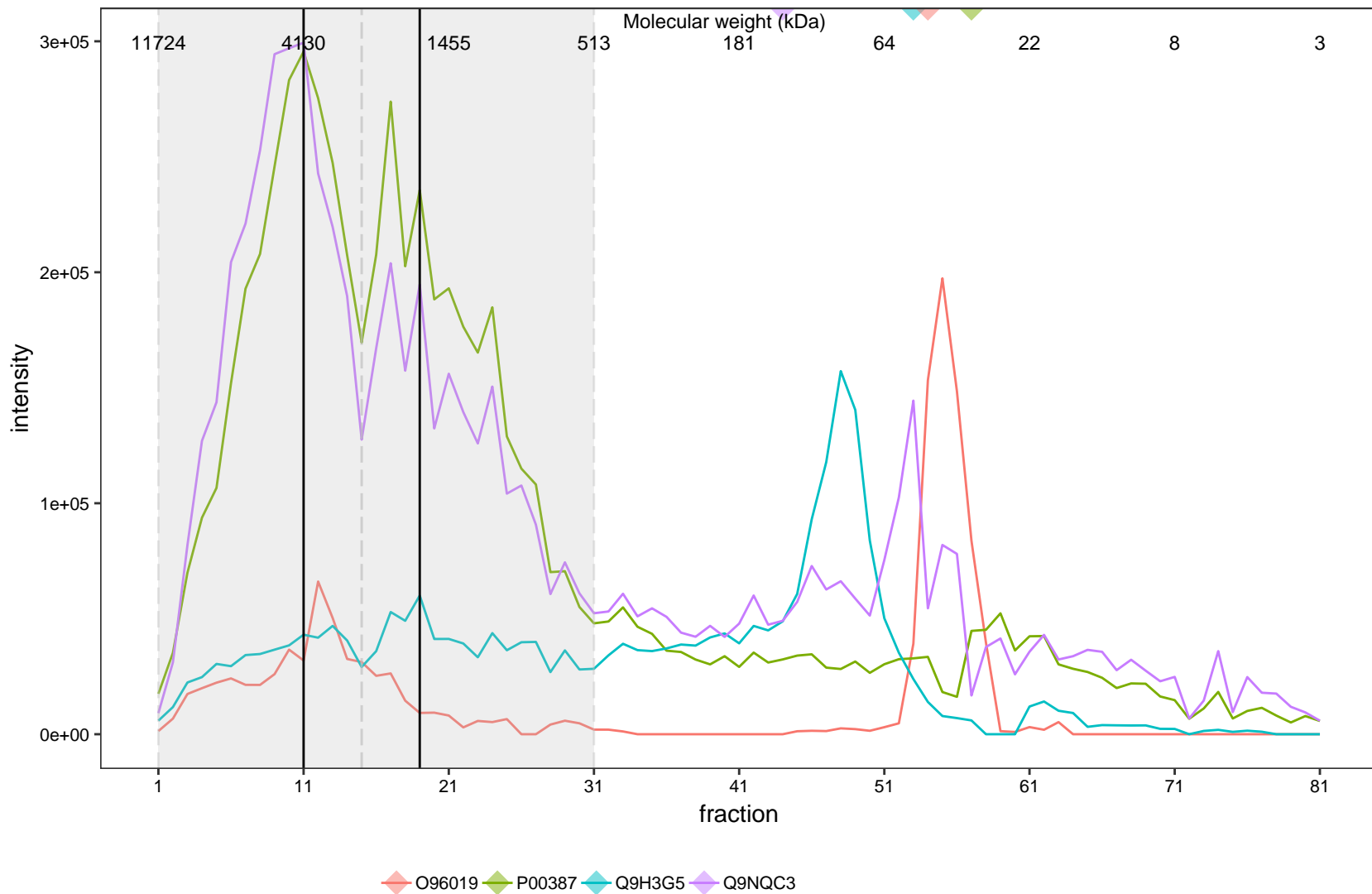

Supplement: Supplementary file 7 — Dataset EV6 [file MSB-15-e8438-s007.zip › feature_plots_bioplex/P00387.pdf]

**P01210**

**Annotated subunits: 9   Subunits with signal: 6**

**Max. coeluting subunits: 5   Max. completeness: 0.56**

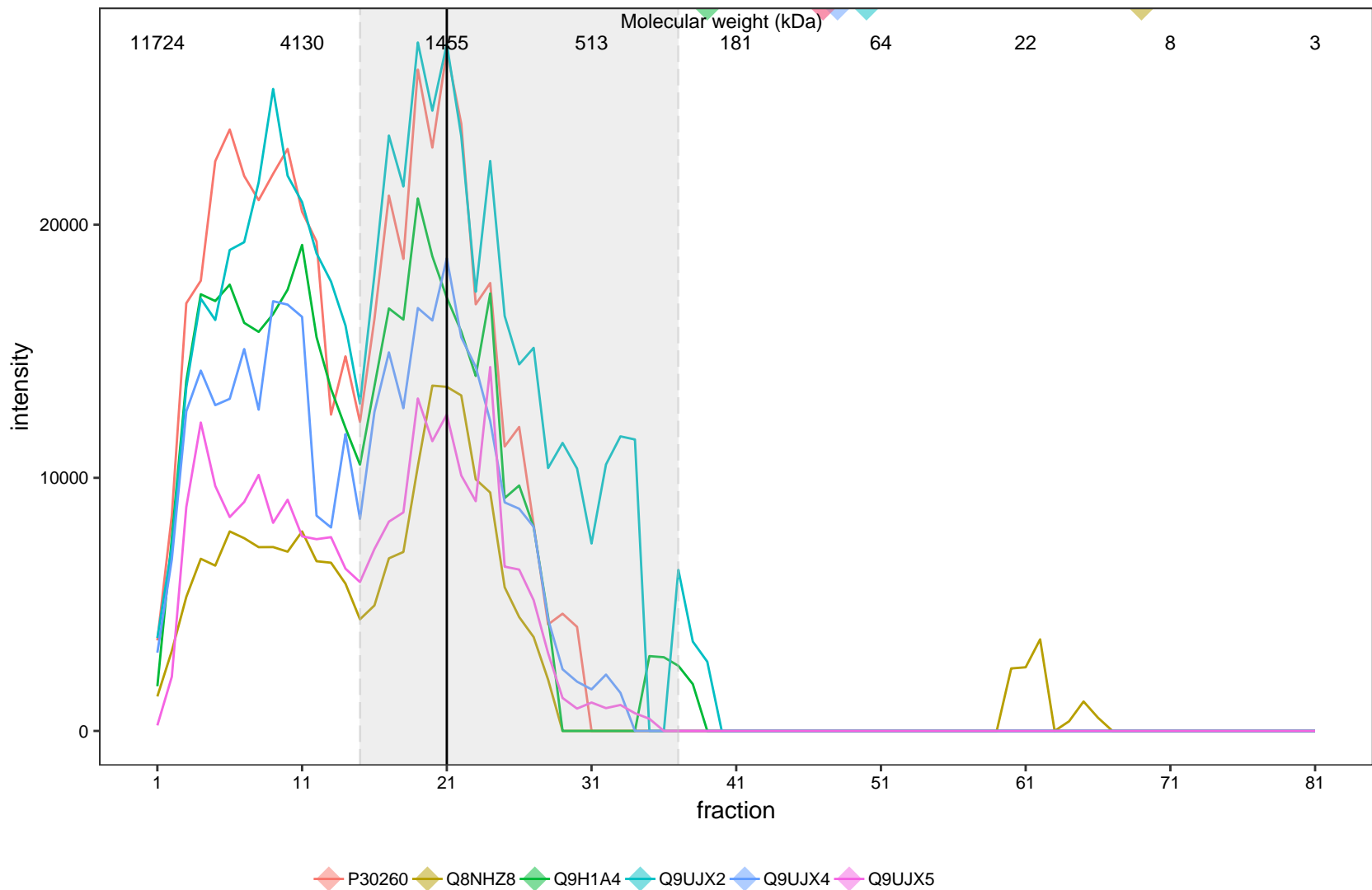

Supplement: Supplementary file 7 — Dataset EV6 [file MSB-15-e8438-s007.zip › feature_plots_bioplex/P01210.pdf]

**P01213**

**Annotated subunits: 9 Subunits with signal: 4**

**Max. coeluting subunits: 2 Max. completeness: 0.22**

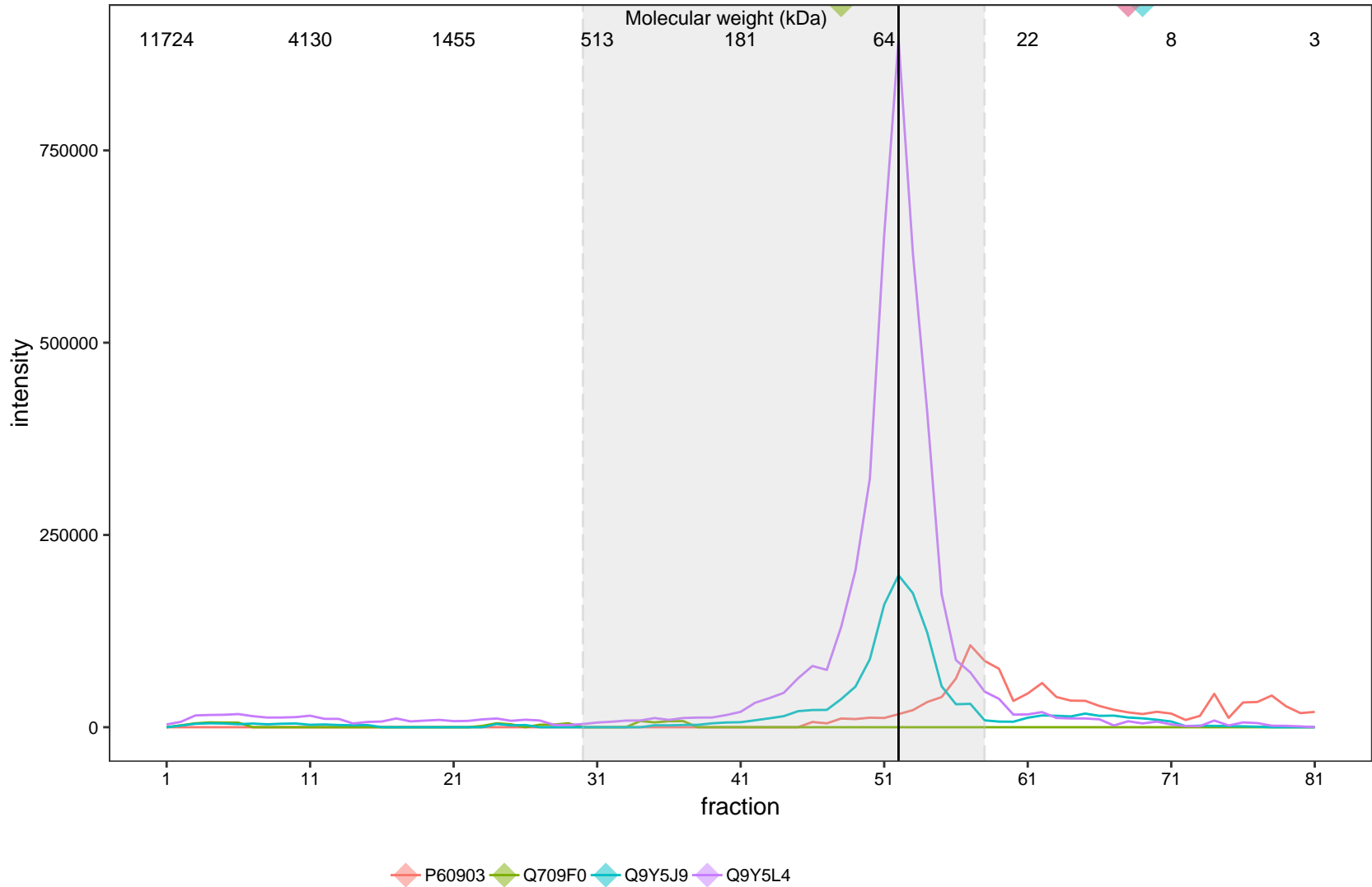

Supplement: Supplementary file 7 — Dataset EV6 [file MSB-15-e8438-s007.zip › feature_plots_bioplex/P01213.pdf]

**P03905**

**Annotated subunits: 4 Subunits with signal: 4**

**Max. coeluting subunits: 3 Max. completeness: 0.75**

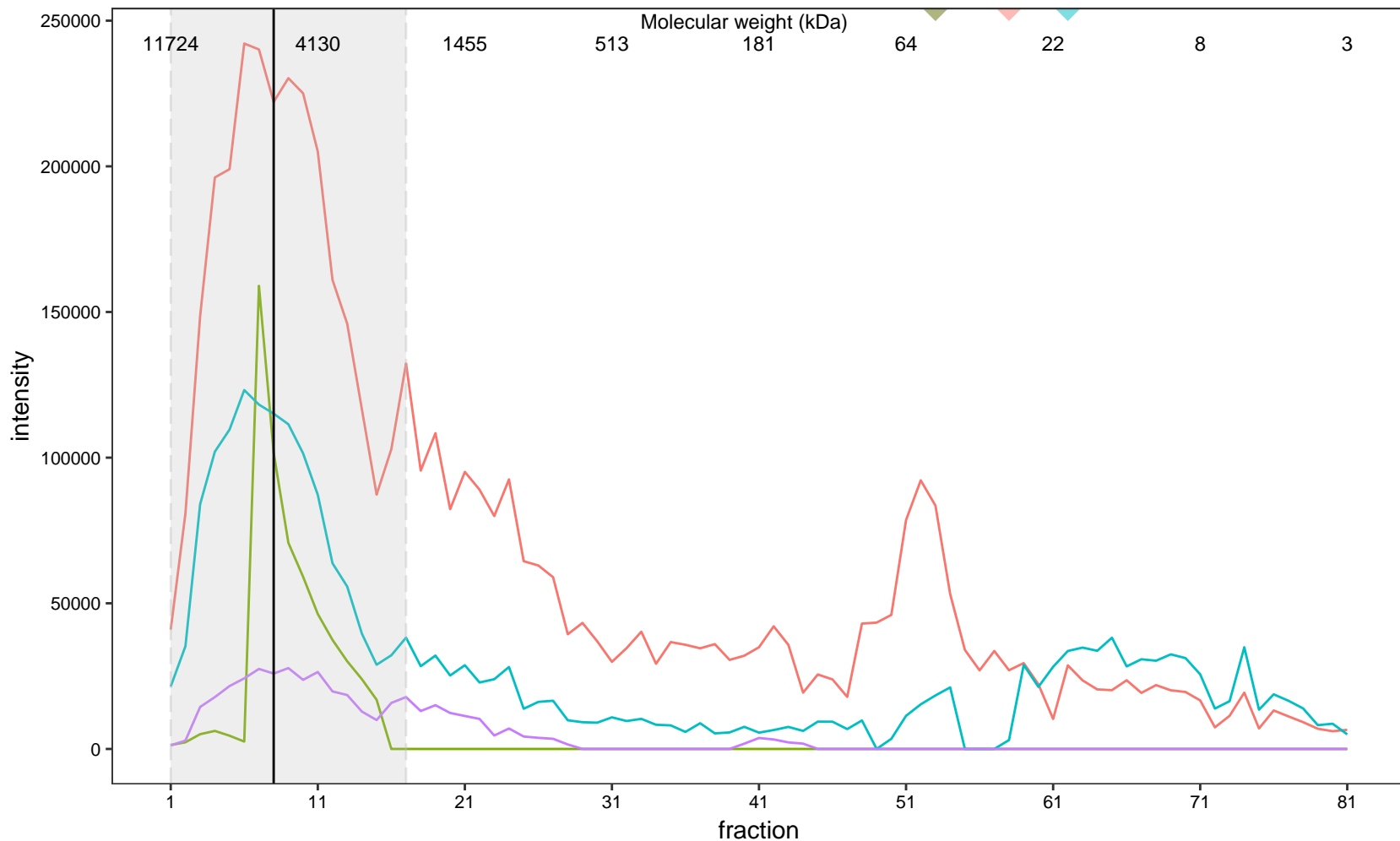

◊ O75489 ◊ P03905 ◊ P51970 ◊ Q9BQ95

Supplement: Supplementary file 7 — Dataset EV6 [file MSB-15-e8438-s007.zip › feature_plots_bioplex/P03905.pdf]

**P03915**

**Annotated subunits: 6 Subunits with signal: 4**

**Max. coeluting subunits: 4 Max. completeness: 0.67**

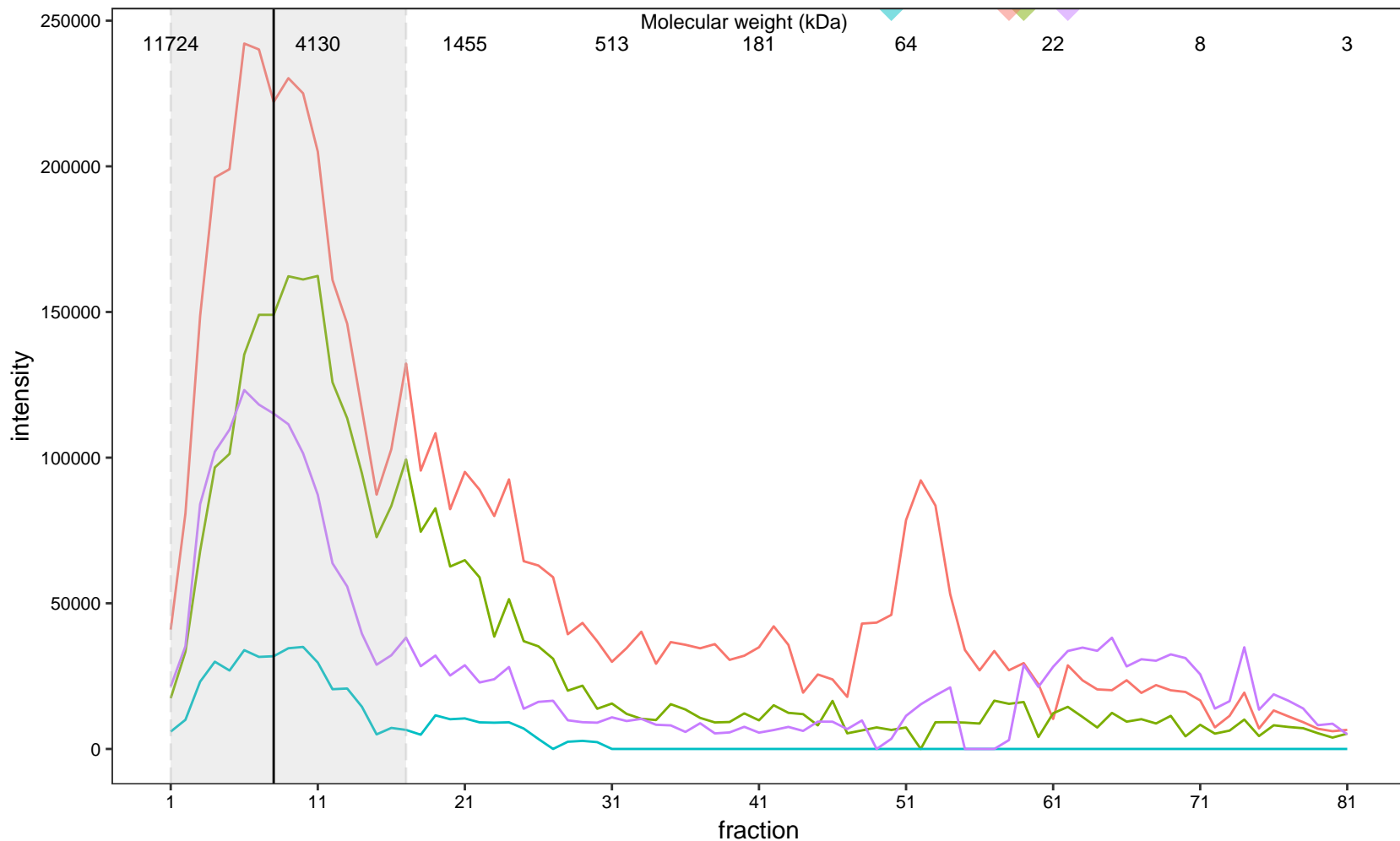

◊ O75489 ◊ O95292 ◊ P03915 ◊ P51970

Supplement: Supplementary file 7 — Dataset EV6 [file MSB-15-e8438-s007.zip › feature_plots_bioplex/P03915.pdf]

**P04035**

**Annotated subunits: 15 Subunits with signal: 7**

**Max. coeluting subunits: 5 Max. completeness: 0.33**

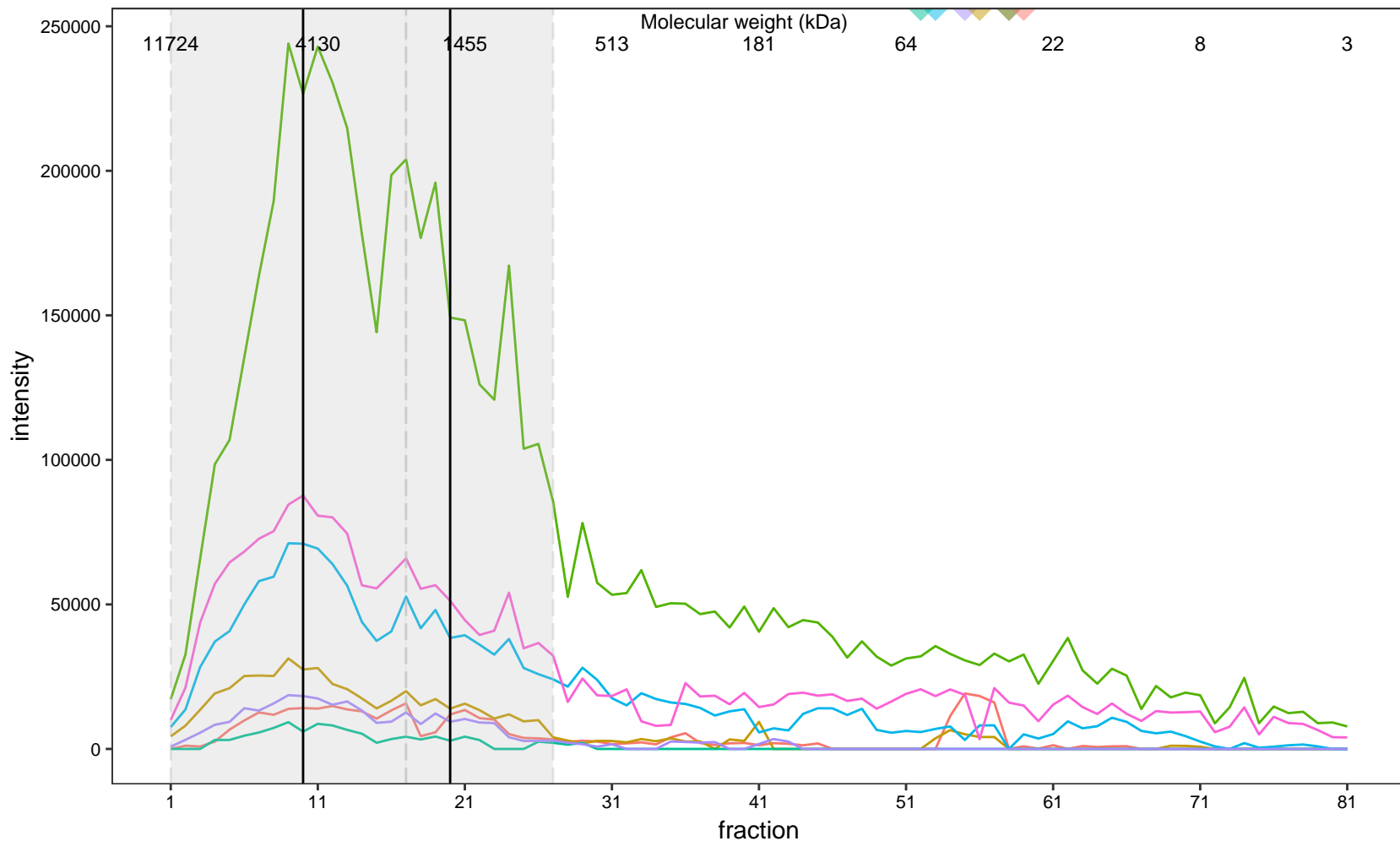

Supplement: Supplementary file 7 — Dataset EV6 [file MSB-15-e8438-s007.zip › feature_plots_bioplex/P04035.pdf]

**P04233**

**Annotated subunits: 5 Subunits with signal: 2**

**Max. coeluting subunits: 2 Max. completeness: 0.4**

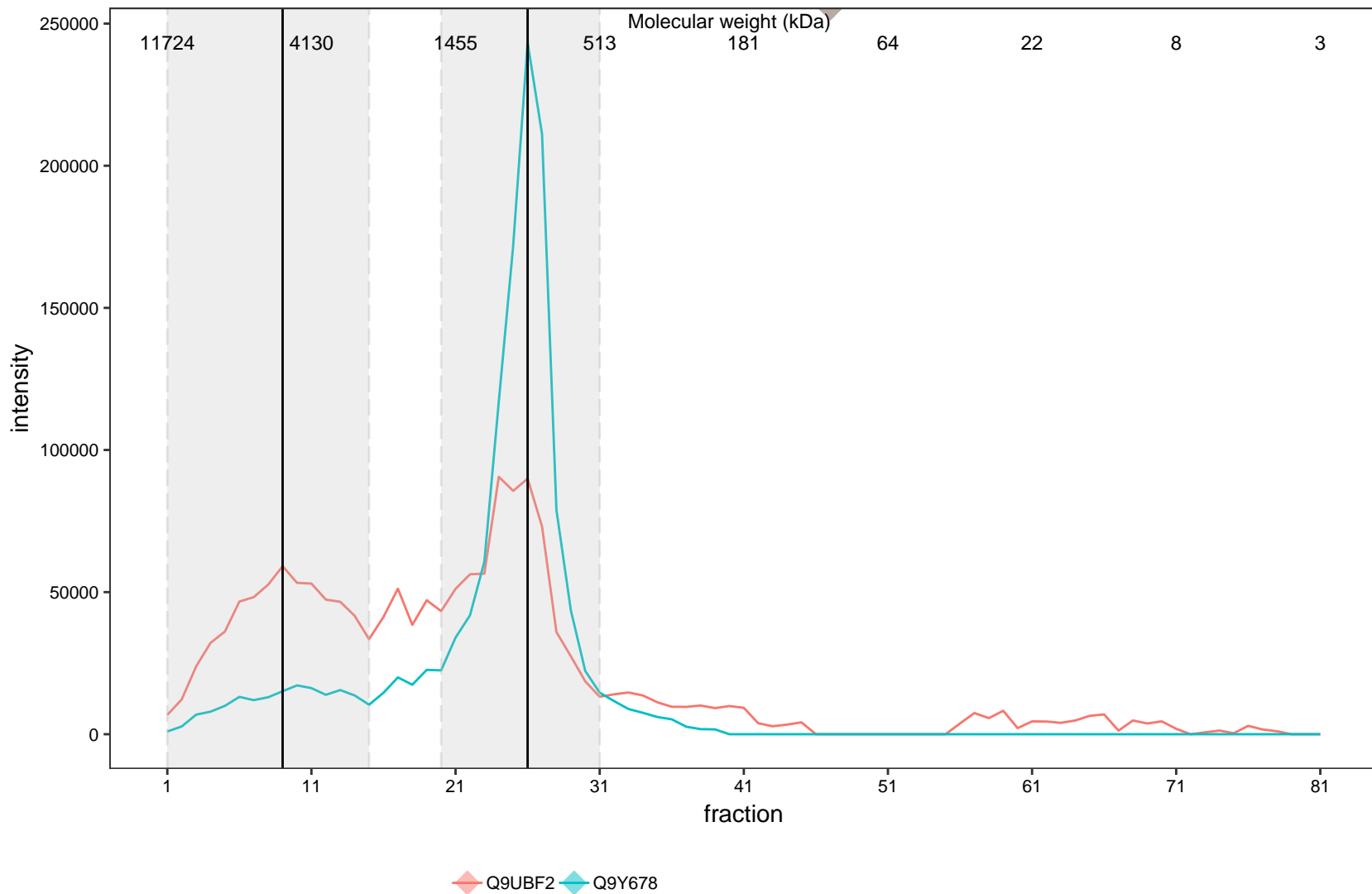

Supplement: Supplementary file 7 — Dataset EV6 [file MSB-15-e8438-s007.zip › feature_plots_bioplex/P04233.pdf]

**P05111**  
**Annotated subunits: 5   Subunits with signal: 4**  
**Max. coeluting subunits: 3   Max. completeness: 0.6**

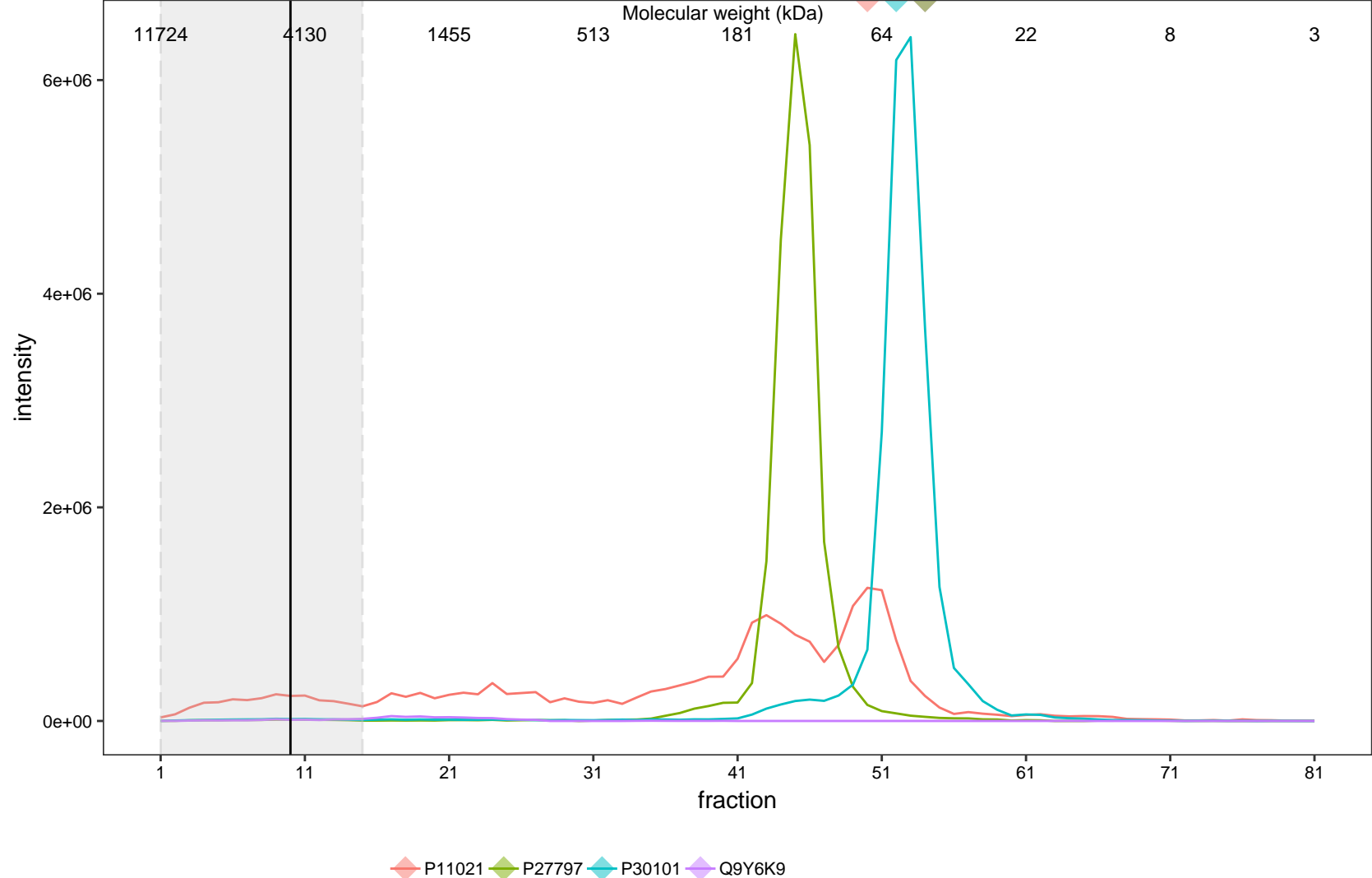

Supplement: Supplementary file 7 — Dataset EV6 [file MSB-15-e8438-s007.zip › feature_plots_bioplex/P05111.pdf]

**P05230**

**Annotated subunits: 13 Subunits with signal: 4**

**Max. coeluting subunits: 4 Max. completeness: 0.31**

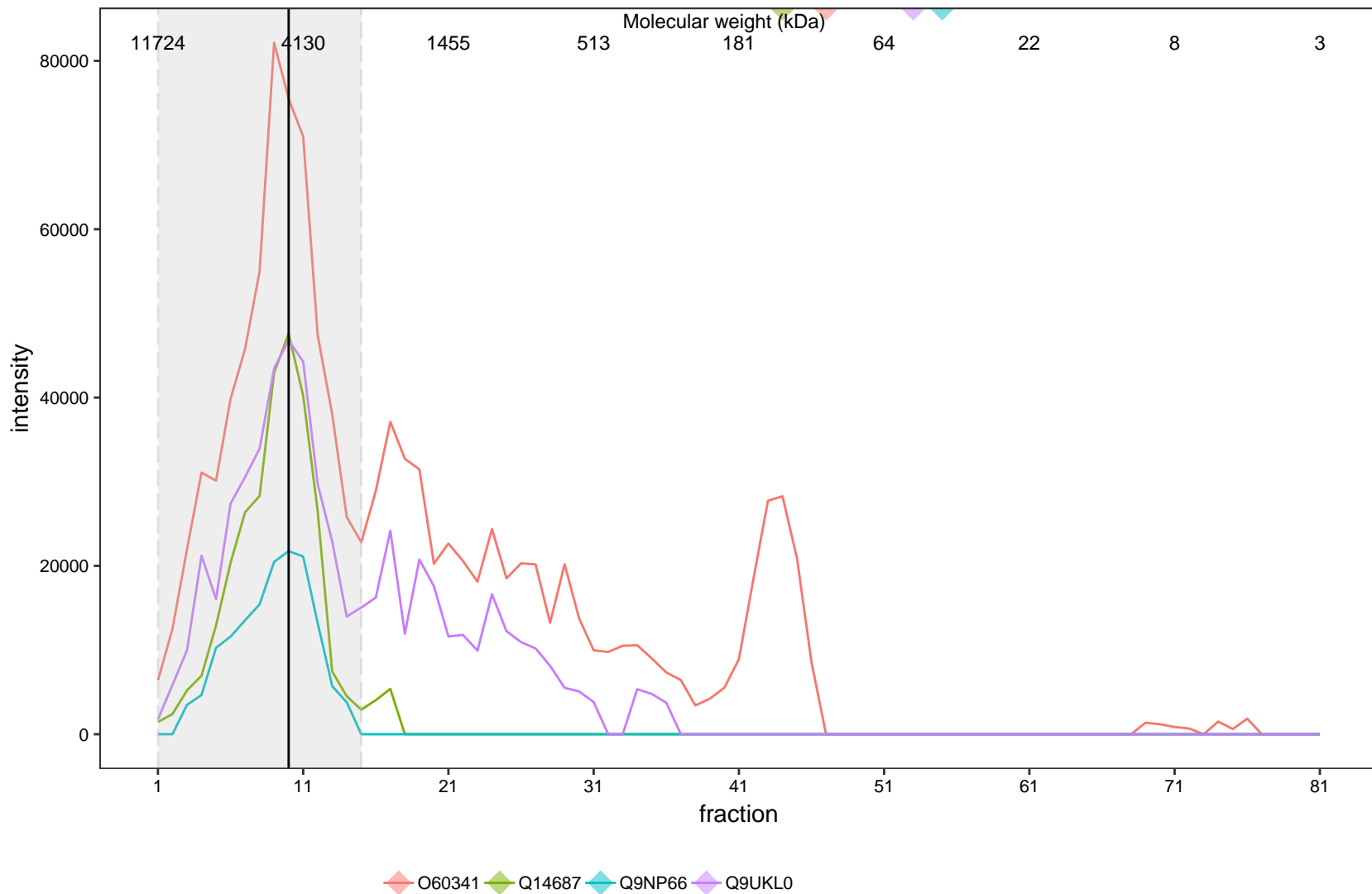

Supplement: Supplementary file 7 — Dataset EV6 [file MSB-15-e8438-s007.zip › feature_plots_bioplex/P05230.pdf]

**P05387**

**Annotated subunits: 2 Subunits with signal: 2**

**Max. coeluting subunits: 2 Max. completeness: 1**

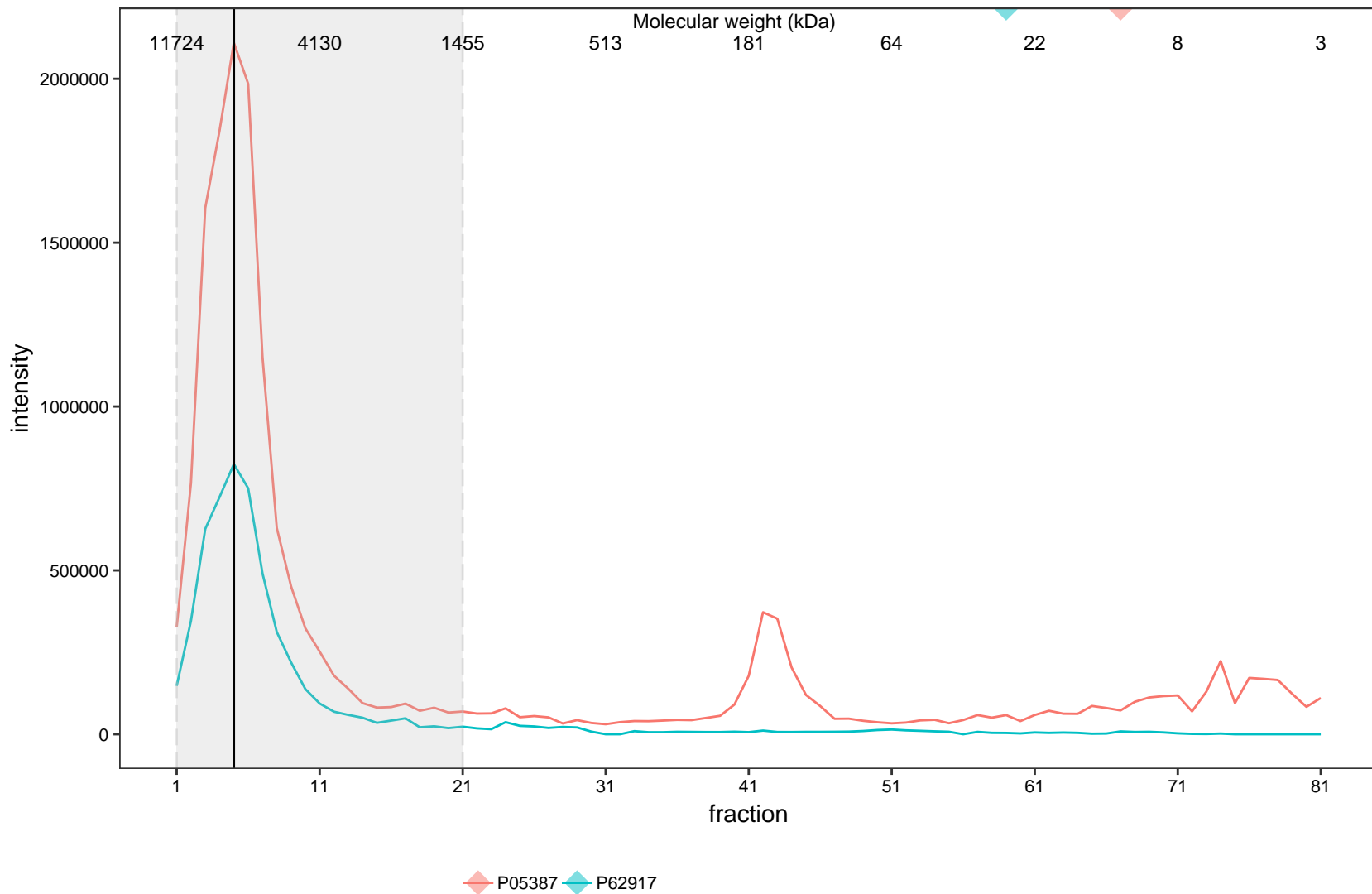

Supplement: Supplementary file 7 — Dataset EV6 [file MSB-15-e8438-s007.zip › feature_plots_bioplex/P05387.pdf]

**P05388**

**Annotated subunits: 5 Subunits with signal: 3**

**Max. coeluting subunits: 2 Max. completeness: 0.4**

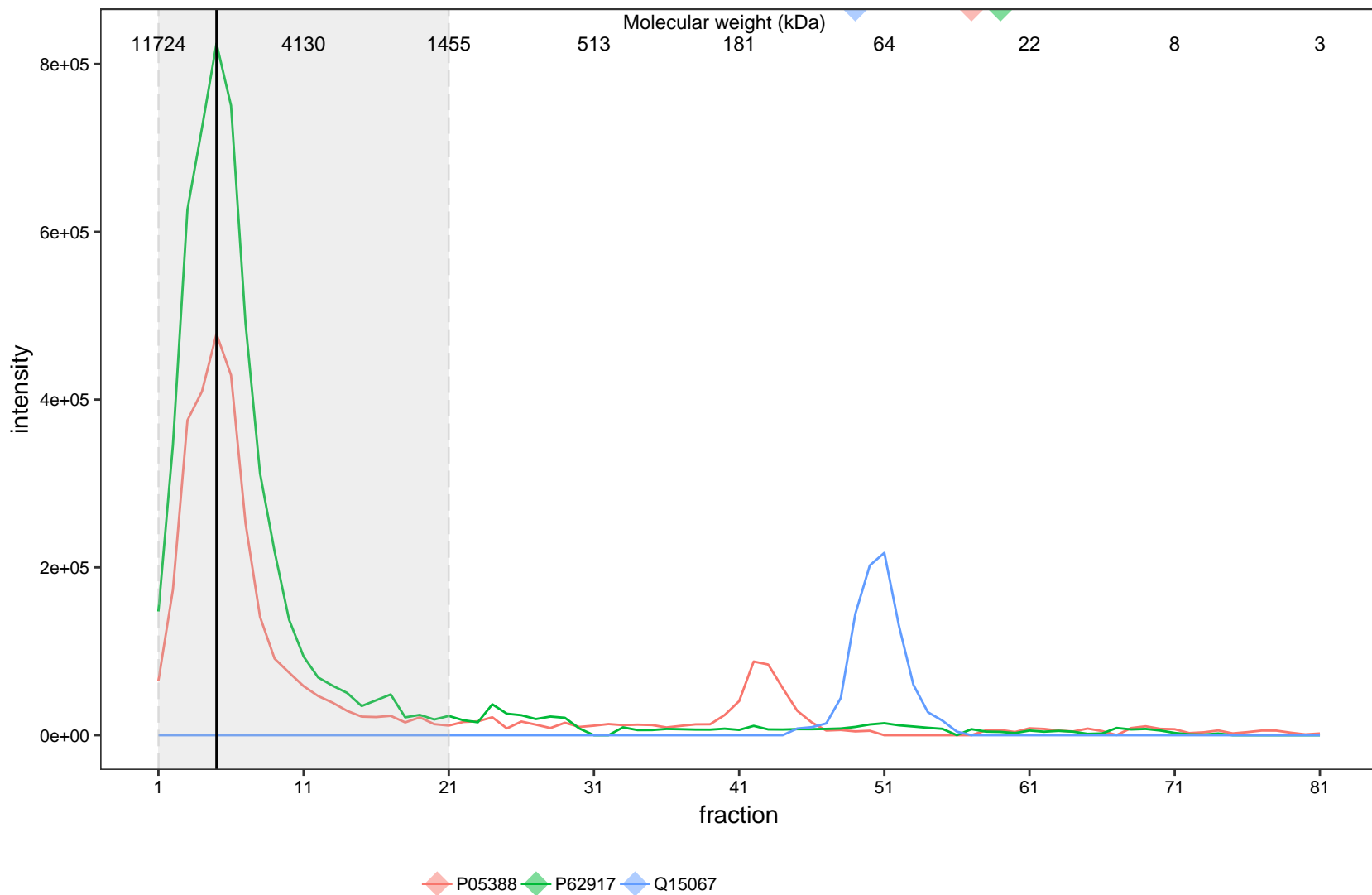

Supplement: Supplementary file 7 — Dataset EV6 [file MSB-15-e8438-s007.zip › feature_plots_bioplex/P05388.pdf]

**P06576**

**Annotated subunits: 6 Subunits with signal: 2**

**Max. coeluting subunits: 2 Max. completeness: 0.33**

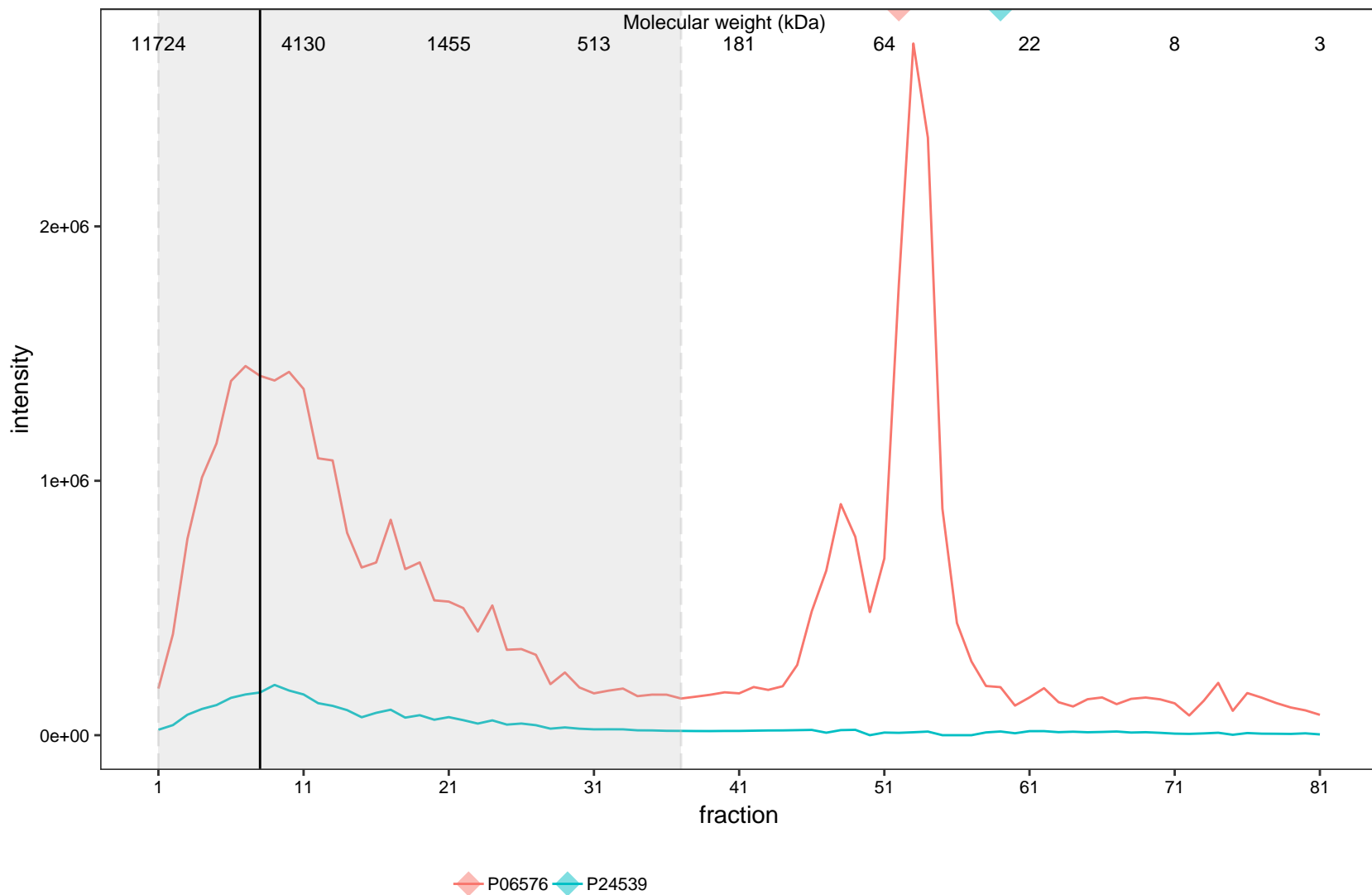

Supplement: Supplementary file 7 — Dataset EV6 [file MSB-15-e8438-s007.zip › feature_plots_bioplex/P06576.pdf]

**P07237**  
**Annotated subunits: 6   Subunits with signal: 3**  
**Max. coeluting subunits: 2   Max. completeness: 0.33**

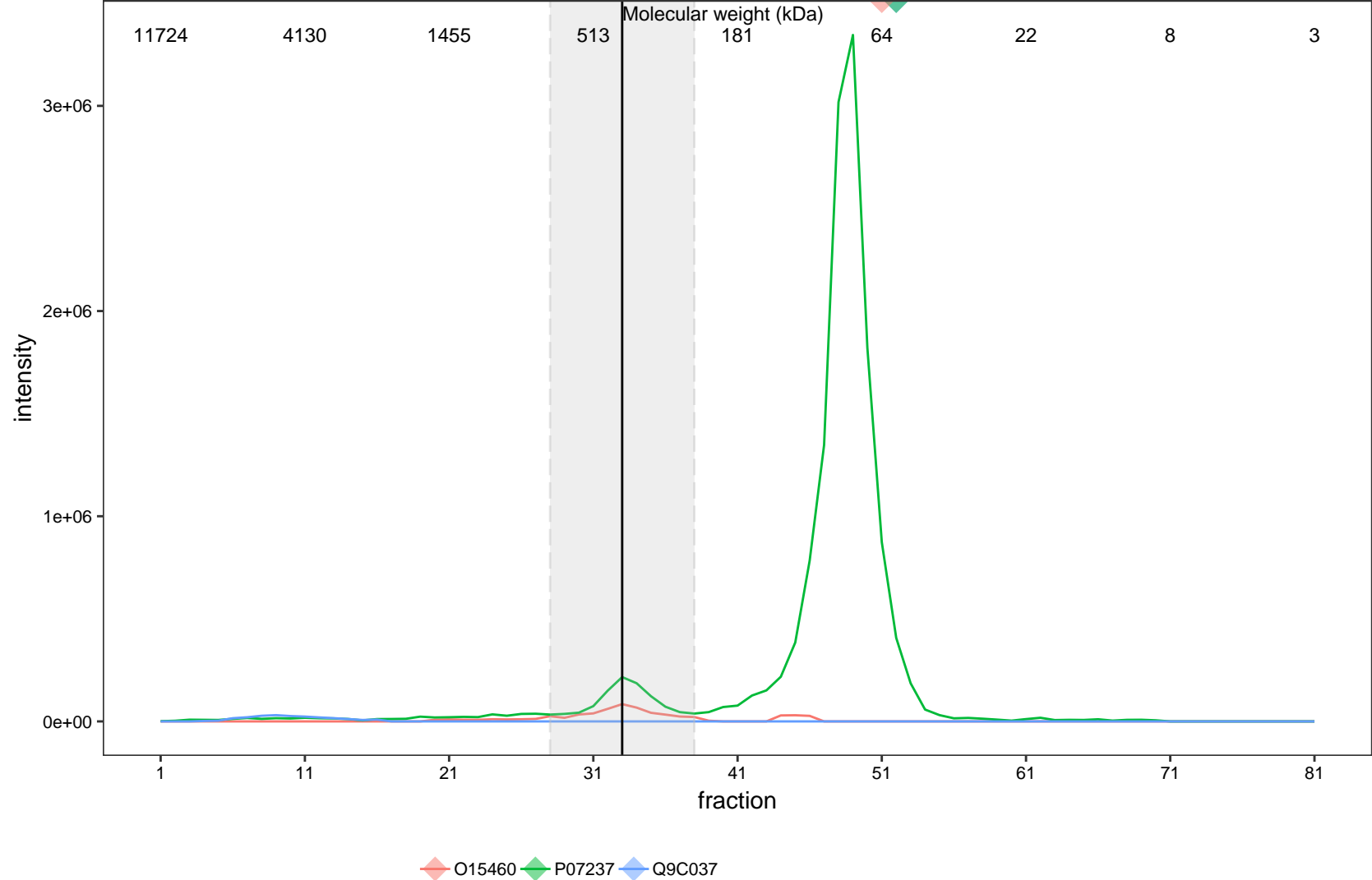

Supplement: Supplementary file 7 — Dataset EV6 [file MSB-15-e8438-s007.zip › feature_plots_bioplex/P07237.pdf]

**P07910**

**Annotated subunits: 2 Subunits with signal: 2**

**Max. coeluting subunits: 2 Max. completeness: 1**

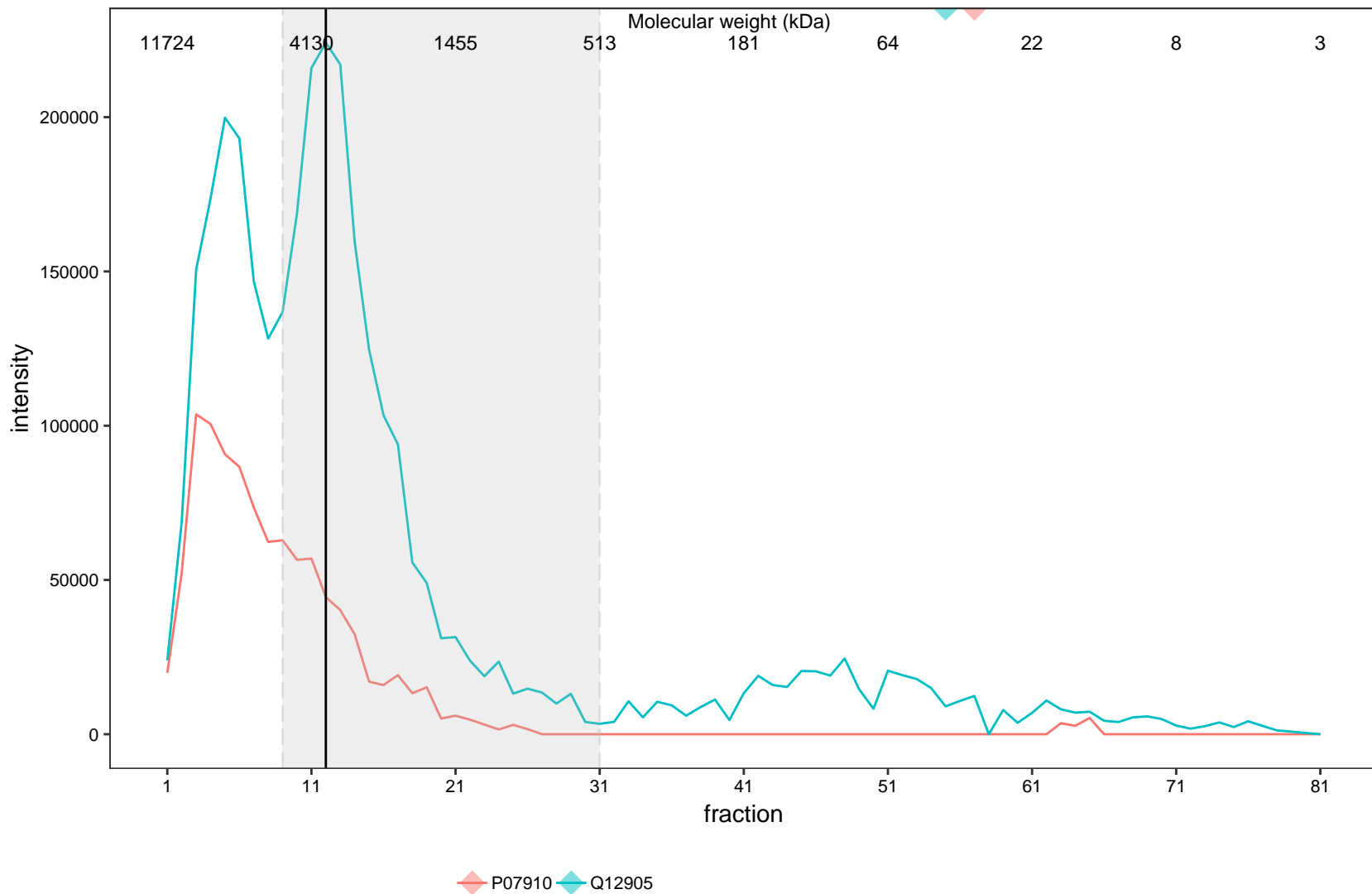

Supplement: Supplementary file 7 — Dataset EV6 [file MSB-15-e8438-s007.zip › feature_plots_bioplex/P07910.pdf]

**P07919**

**Annotated subunits: 3 Subunits with signal: 3**

**Max. coeluting subunits: 2 Max. completeness: 0.67**

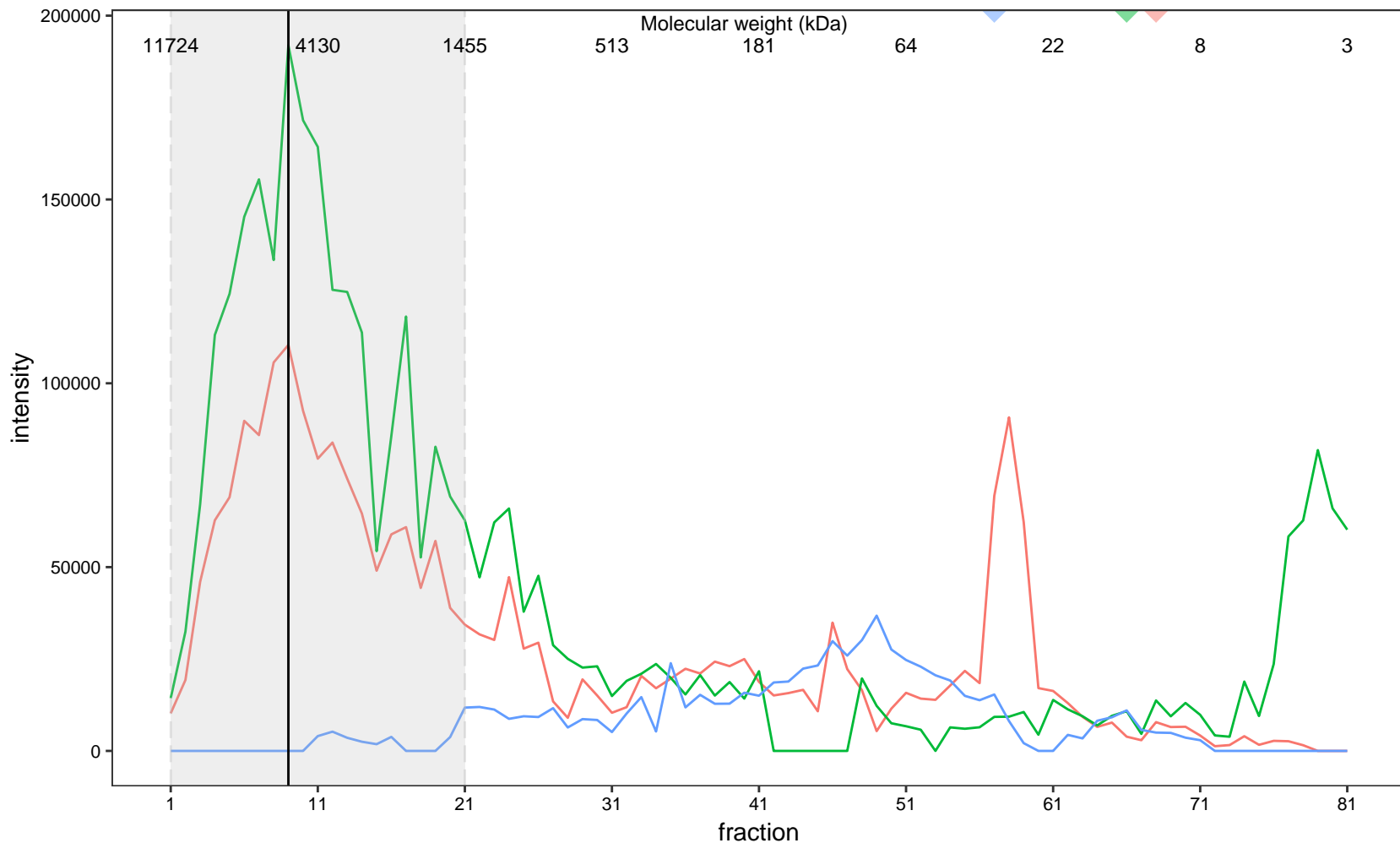

◆ P07919 ◆ P14927 ◆ P40855

Supplement: Supplementary file 7 — Dataset EV6 [file MSB-15-e8438-s007.zip › feature_plots_bioplex/P07919.pdf]

**P08240**

**Annotated subunits: 3 Subunits with signal: 3**

**Max. coeluting subunits: 3 Max. completeness: 1**

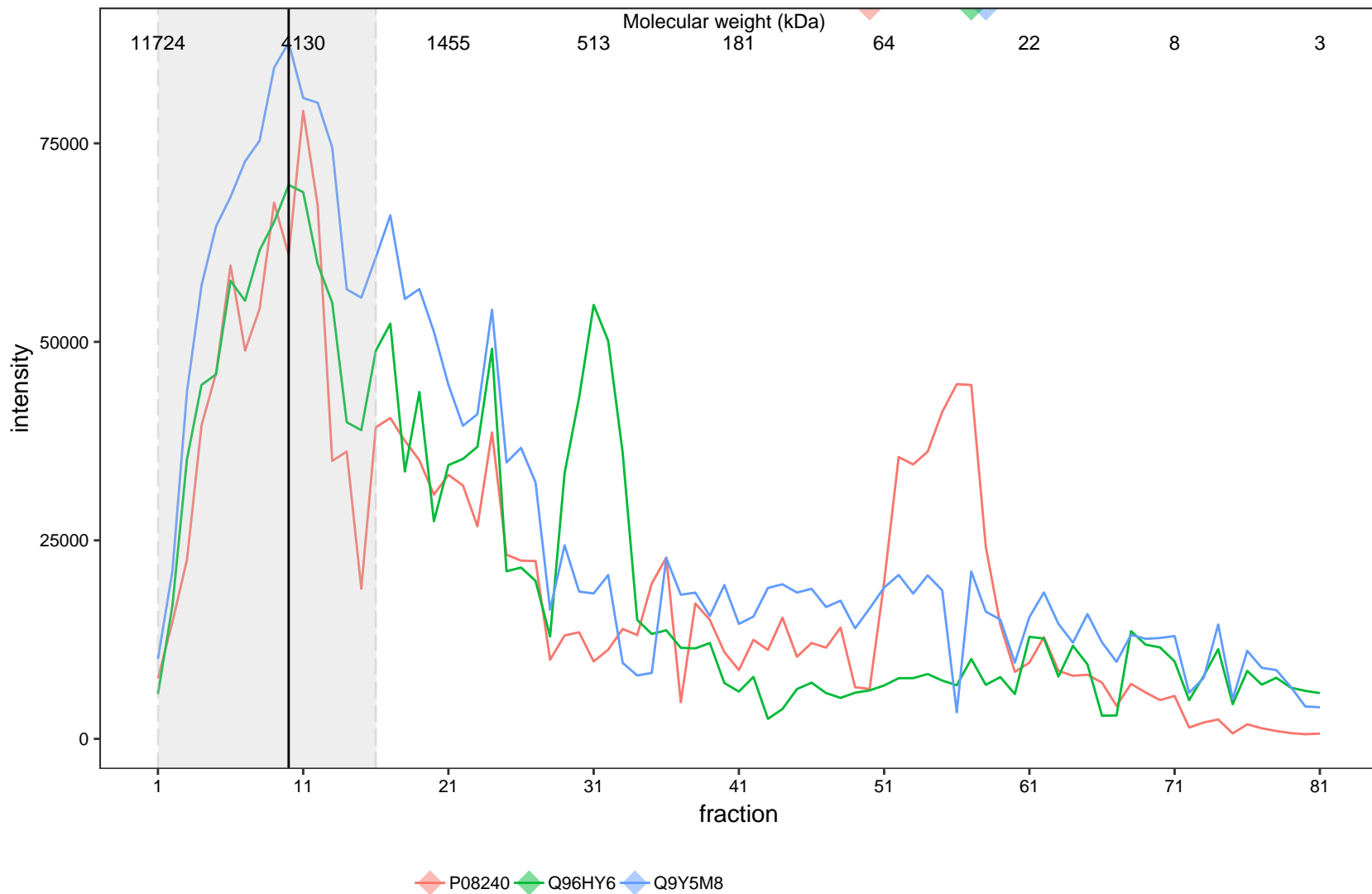

Supplement: Supplementary file 7 — Dataset EV6 [file MSB-15-e8438-s007.zip › feature_plots_bioplex/P08240.pdf]

**P08559**

**Annotated subunits: 4 Subunits with signal: 4**

**Max. coeluting subunits: 2 Max. completeness: 0.5**

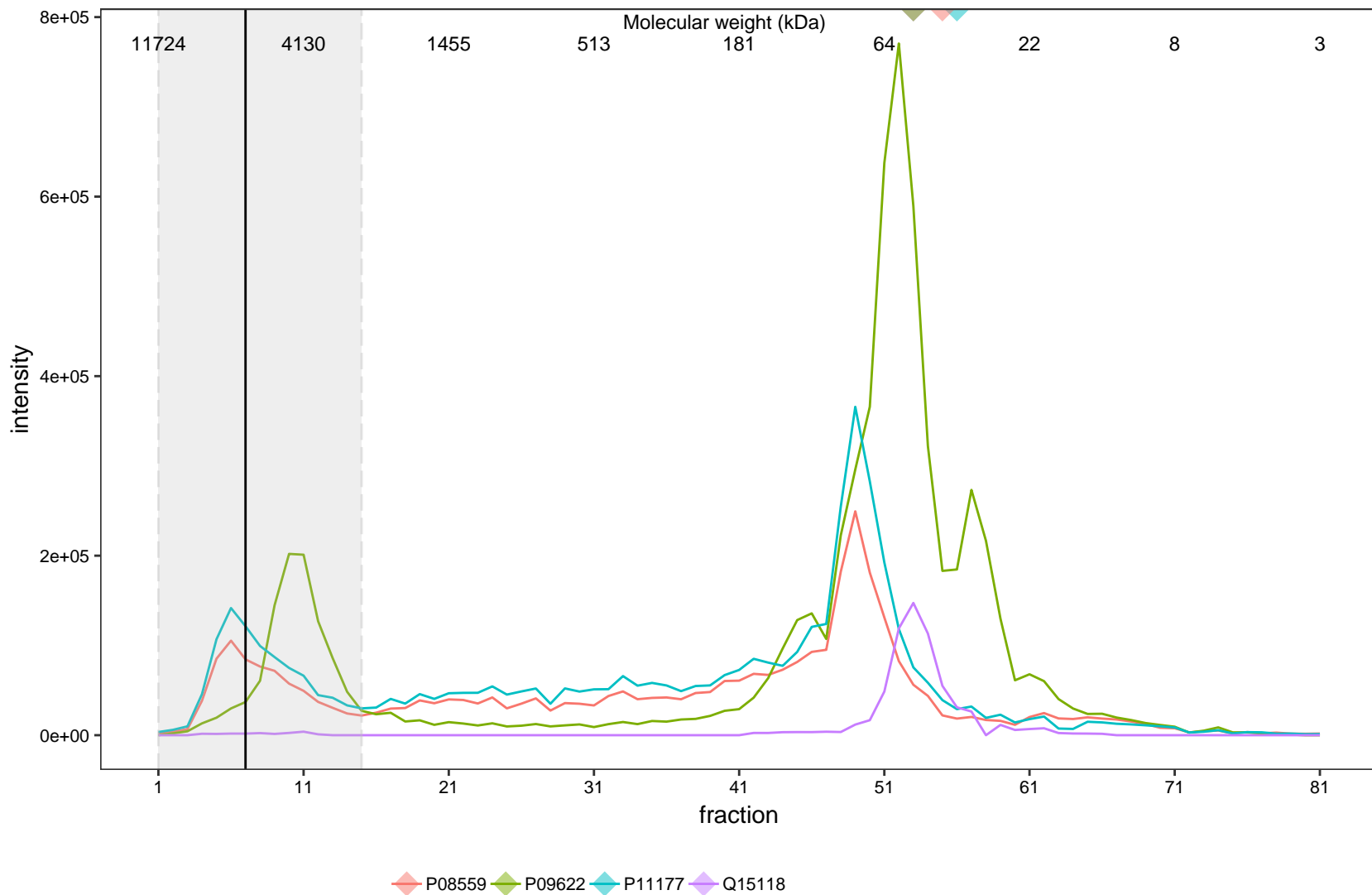

Supplement: Supplementary file 7 — Dataset EV6 [file MSB-15-e8438-s007.zip › feature_plots_bioplex/P08559.pdf]

**P08574**

**Annotated subunits: 5 Subunits with signal: 5**

**Max. coeluting subunits: 3 Max. completeness: 0.6**

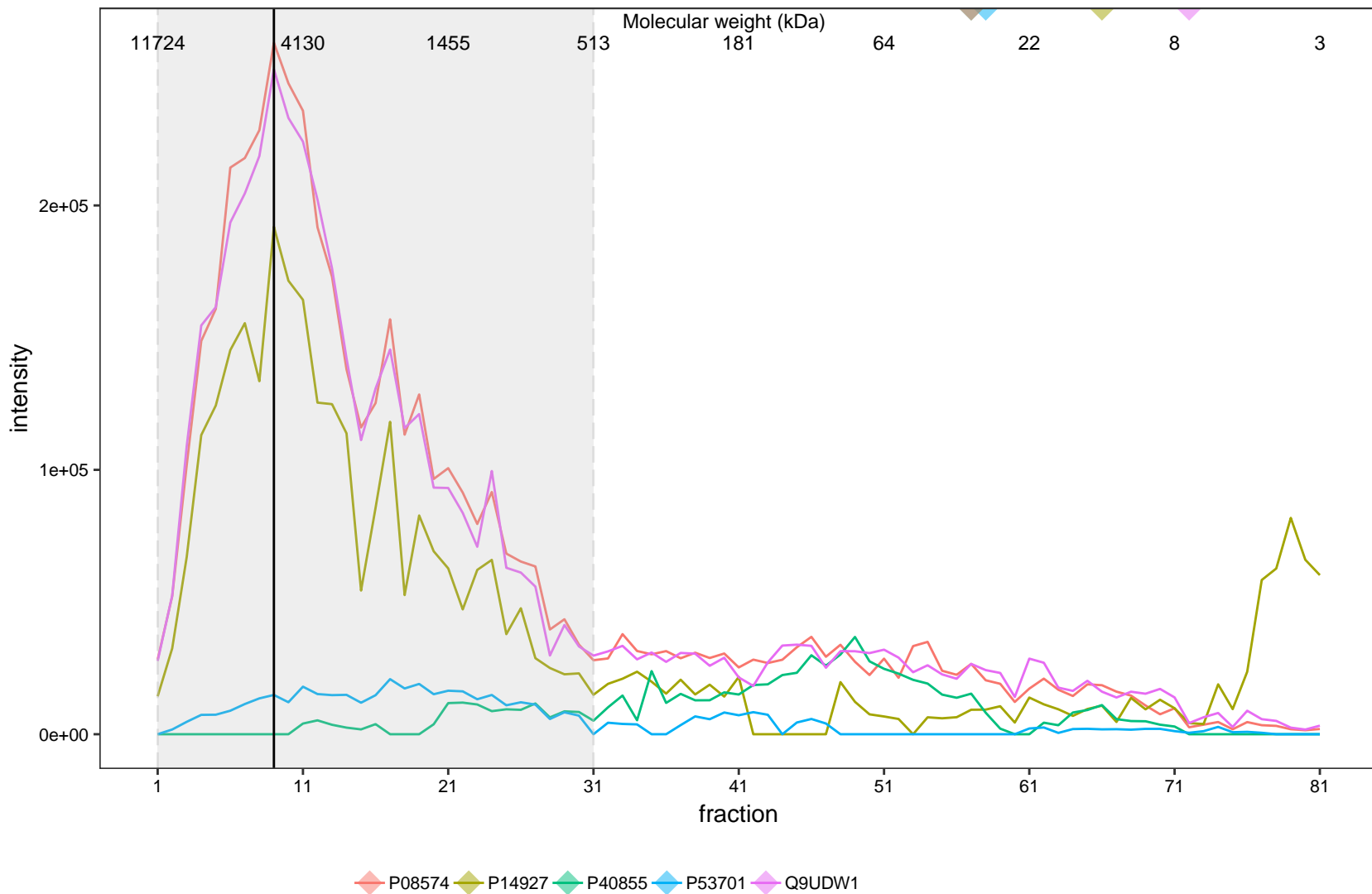

Supplement: Supplementary file 7 — Dataset EV6 [file MSB-15-e8438-s007.zip › feature_plots_bioplex/P08574.pdf]

**P08579**

**Annotated subunits: 2 Subunits with signal: 2**

**Max. coeluting subunits: 2 Max. completeness: 1**

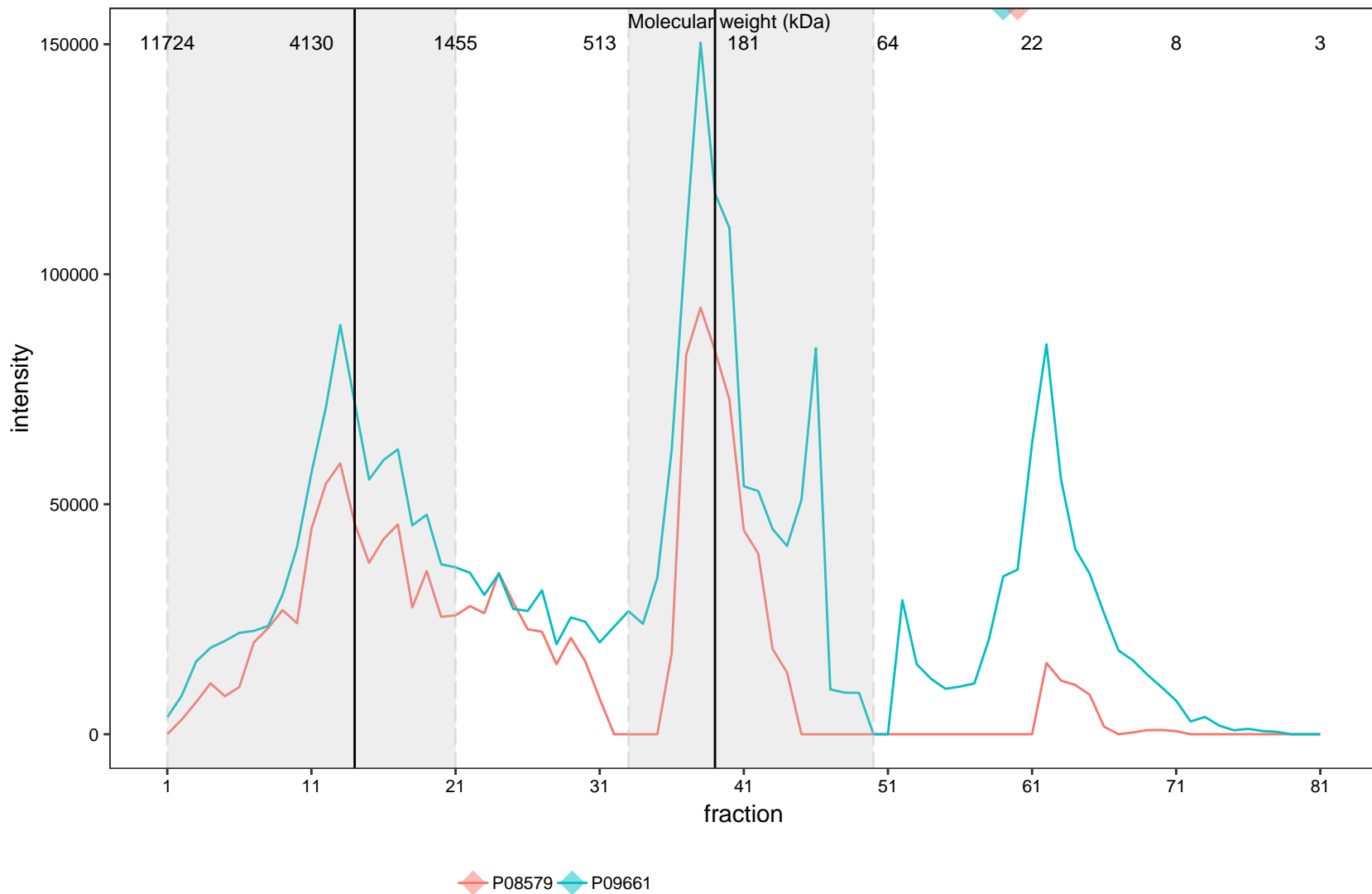

Supplement: Supplementary file 7 — Dataset EV6 [file MSB-15-e8438-s007.zip › feature_plots_bioplex/P08579.pdf]

P09237

Annotated subunits: 20 Subunits with signal: 16

Max. coeluting subunits: 7 Max. completeness: 0.35

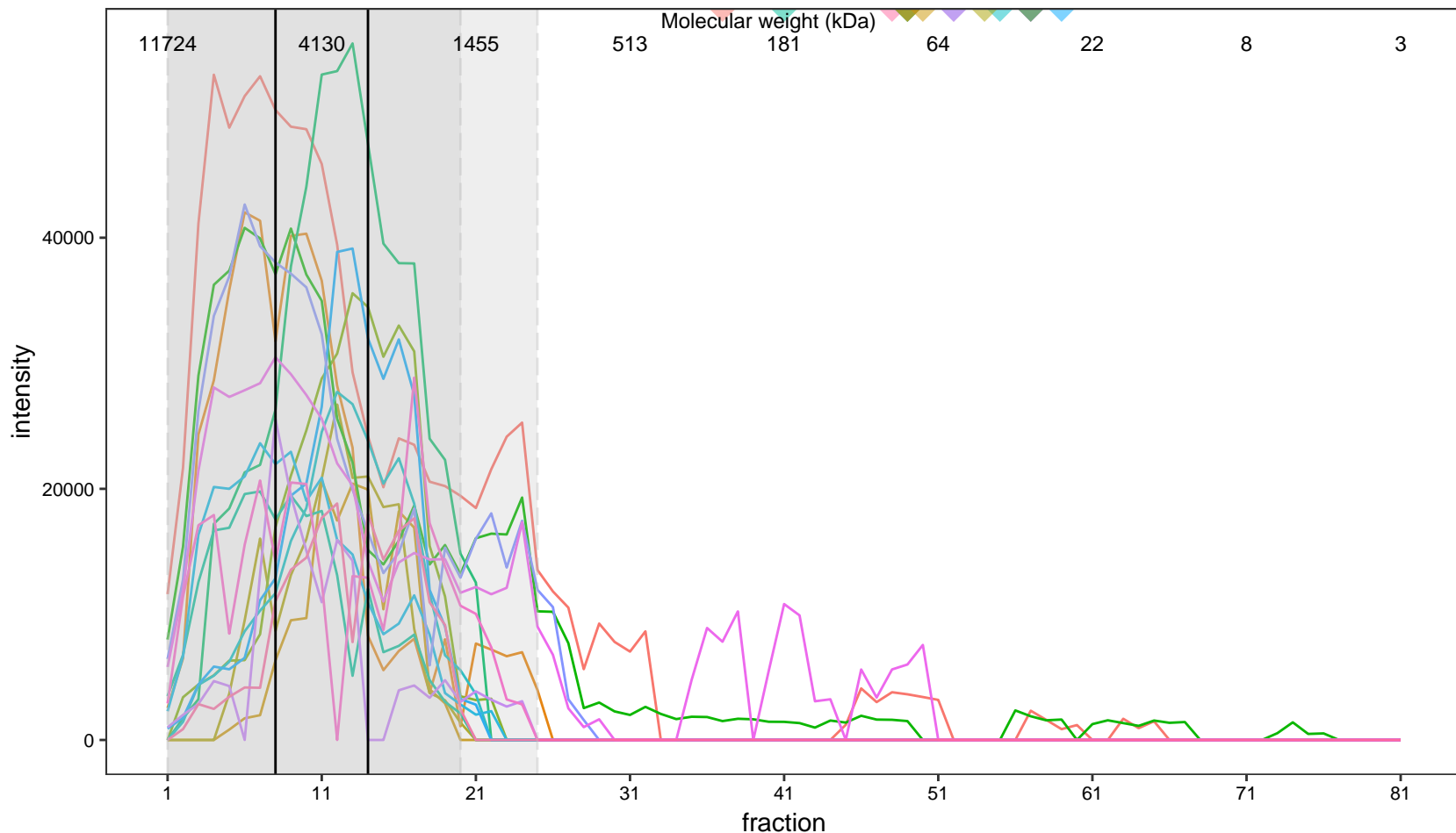

Supplement: Supplementary file 7 — Dataset EV6 [file MSB-15-e8438-s007.zip › feature_plots_bioplex/P09237.pdf]

**P09651**

**Annotated subunits: 71 Subunits with signal: 56**

**Max. coeluting subunits: 35 Max. completeness: 0.49**

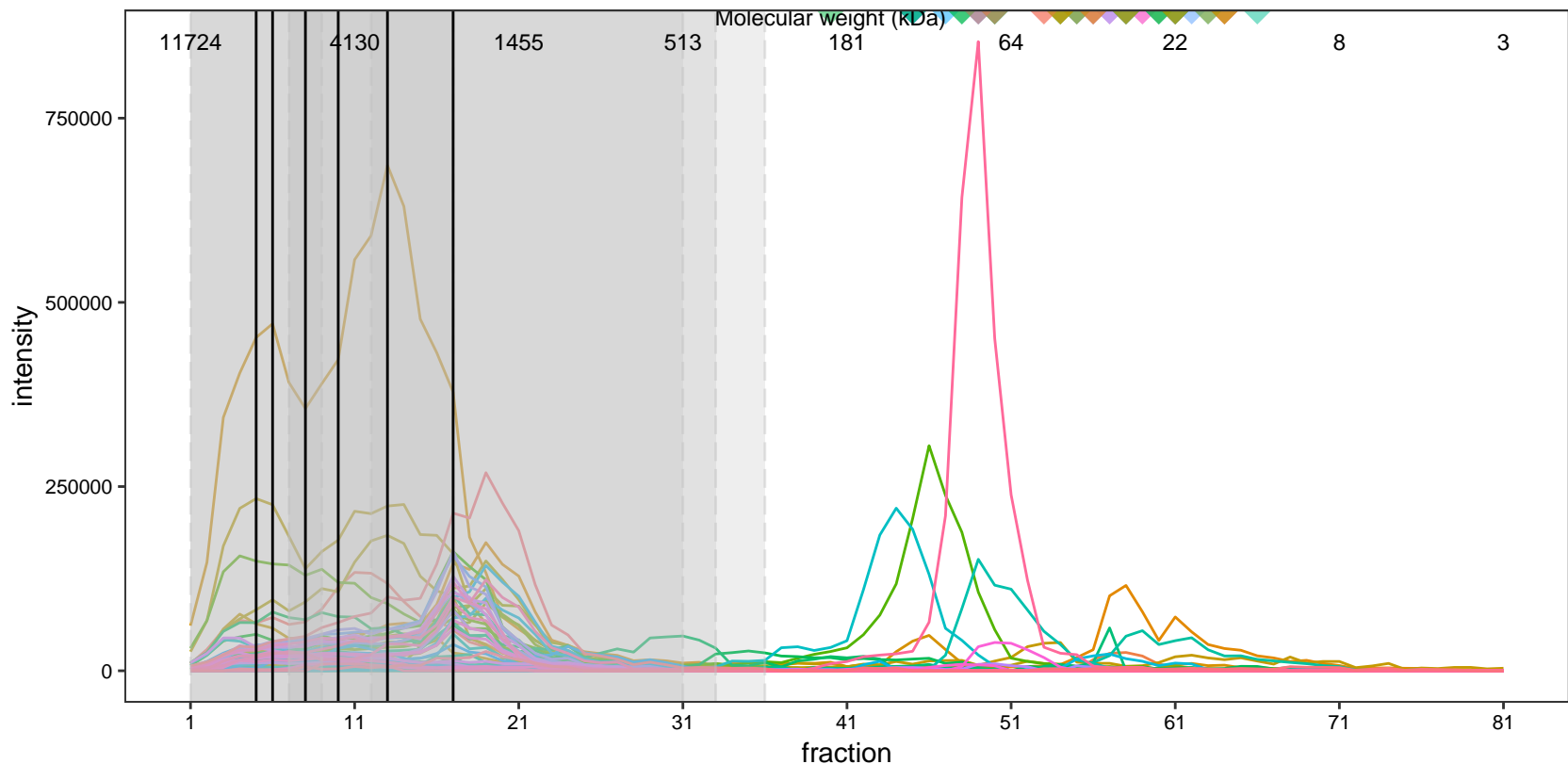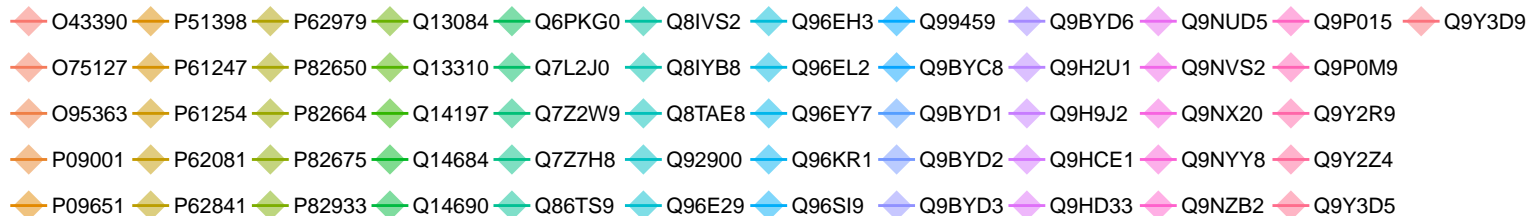

Supplement: Supplementary file 7 — Dataset EV6 [file MSB-15-e8438-s007.zip › feature_plots_bioplex/P09651.pdf]

**P0CG38**  
**Annotated subunits: 11   Subunits with signal: 5**  
**Max. coeluting subunits: 2   Max. completeness: 0.18**

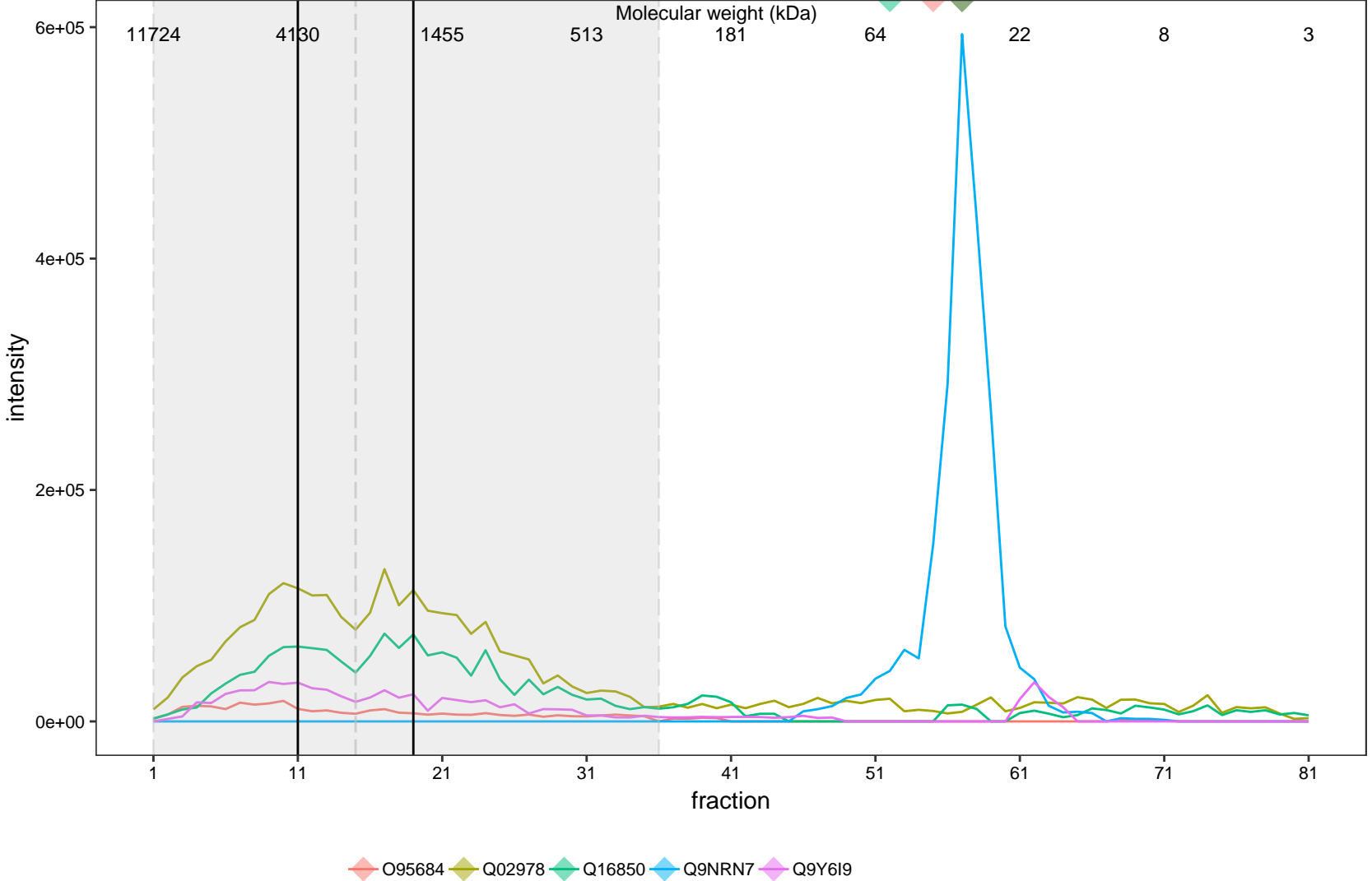

Supplement: Supplementary file 7 — Dataset EV6 [file MSB-15-e8438-s007.zip › feature_plots_bioplex/P0CG38.pdf]

**P10515**

**Annotated subunits: 3 Subunits with signal: 3**

**Max. coeluting subunits: 2 Max. completeness: 0.67**

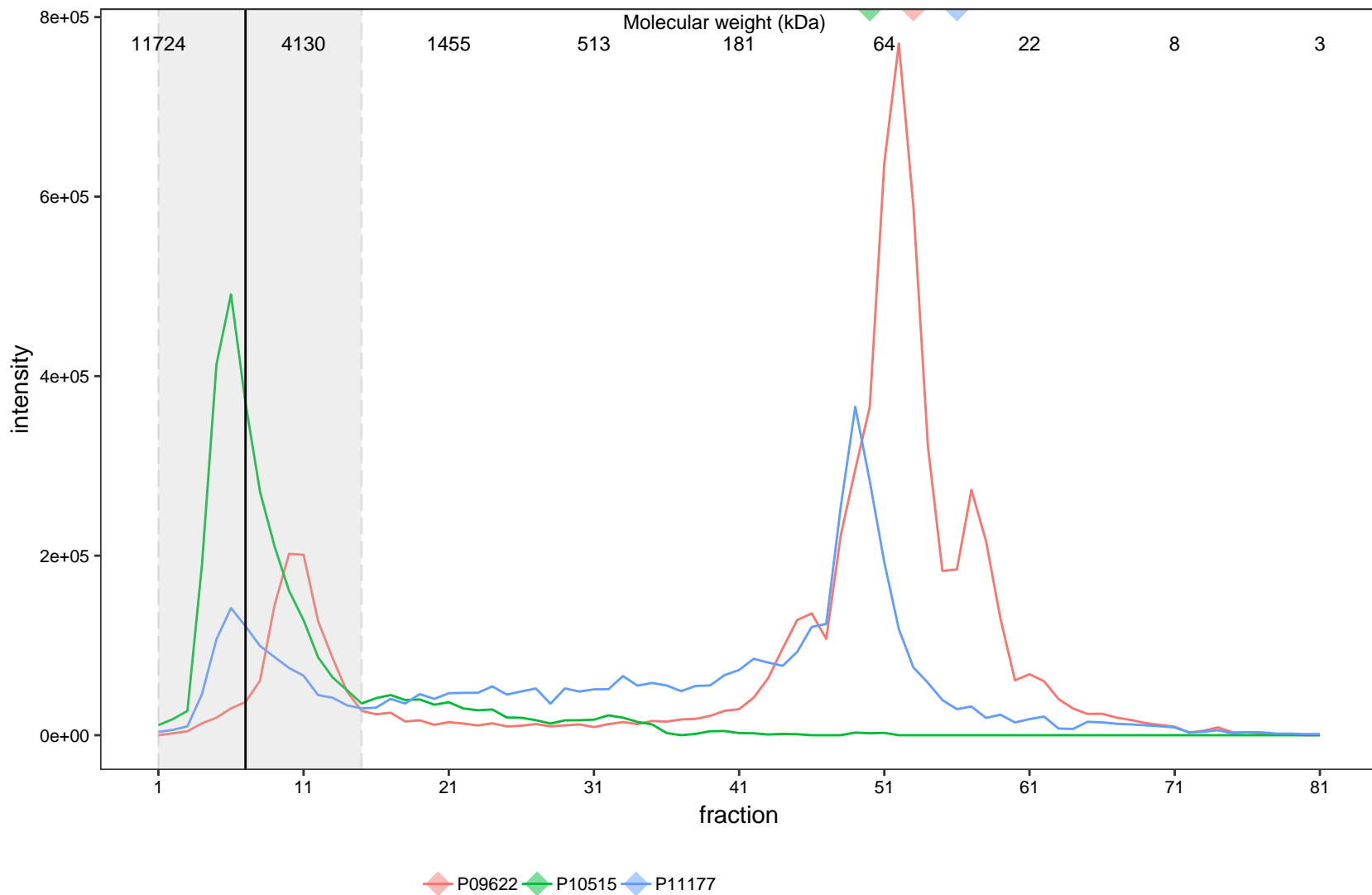

Supplement: Supplementary file 7 — Dataset EV6 [file MSB-15-e8438-s007.zip › feature_plots_bioplex/P10515.pdf]

**P11274**  
**Annotated subunits: 6   Subunits with signal: 4**  
**Max. coeluting subunits: 2   Max. completeness: 0.33**

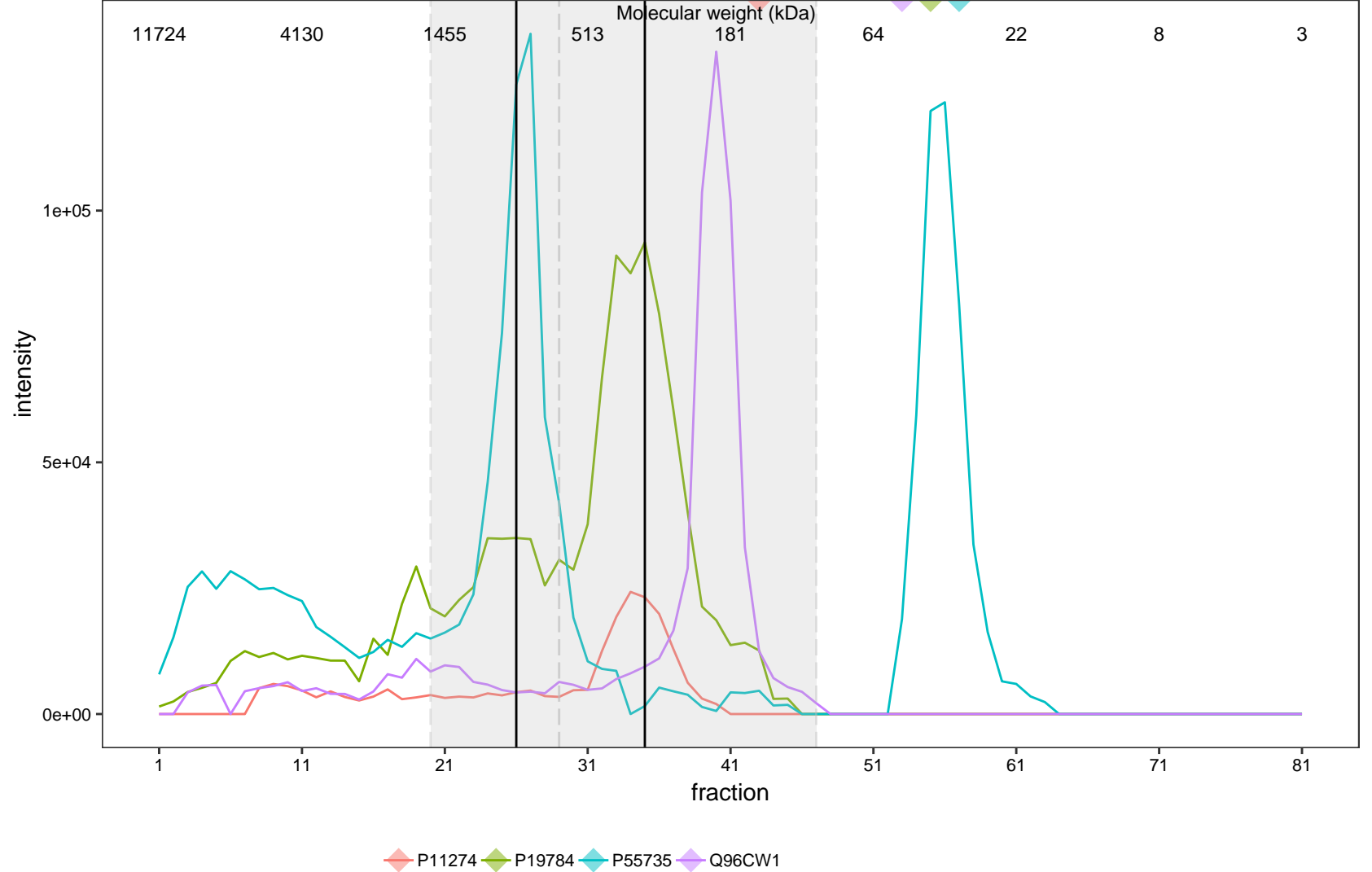

Supplement: Supplementary file 7 — Dataset EV6 [file MSB-15-e8438-s007.zip › feature_plots_bioplex/P11274.pdf]

**P11441**

**Annotated subunits: 2 Subunits with signal: 2**

**Max. coeluting subunits: 2 Max. completeness: 1**

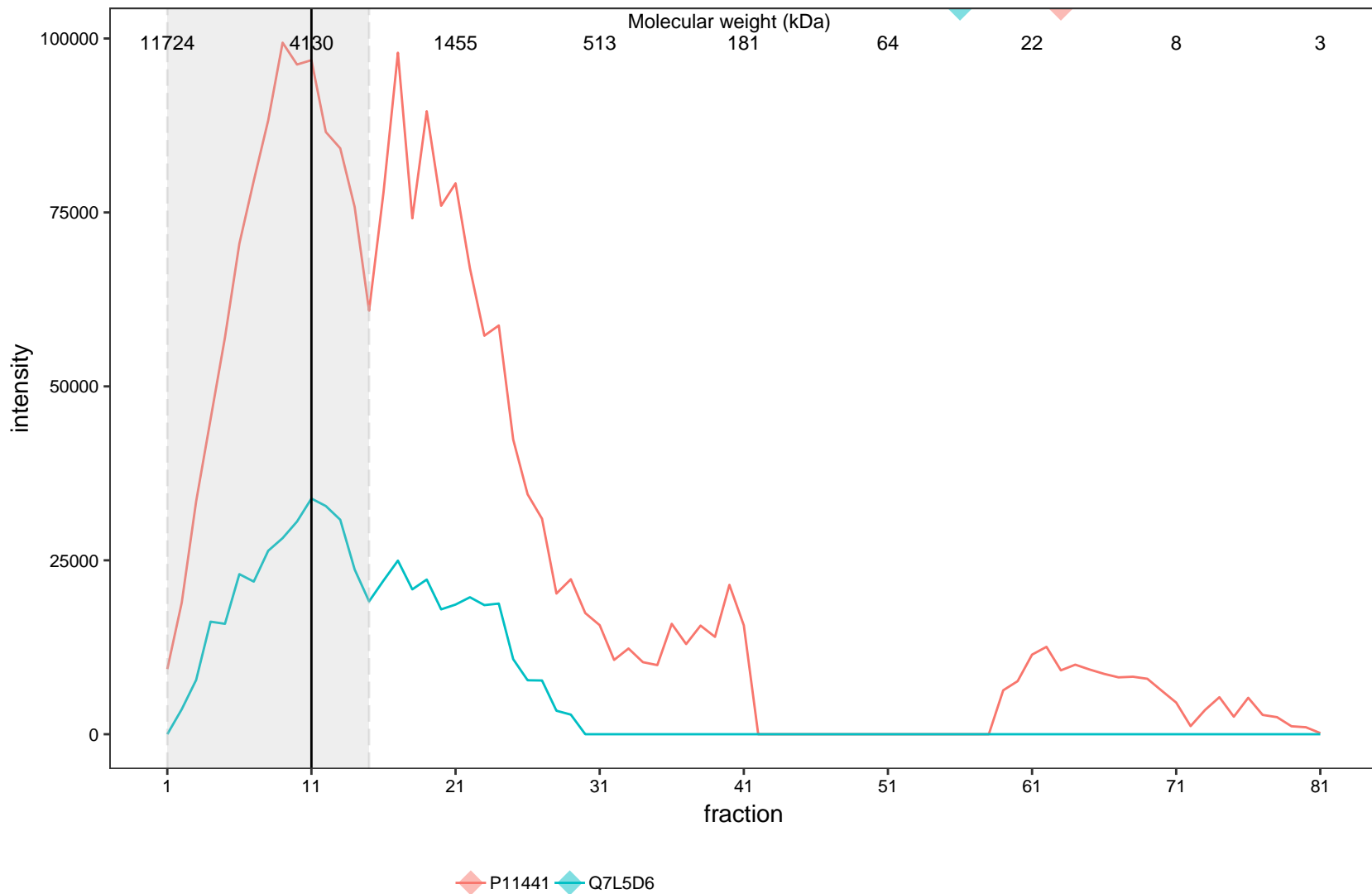

Supplement: Supplementary file 7 — Dataset EV6 [file MSB-15-e8438-s007.zip › feature_plots_bioplex/P11441.pdf]

P13385

Annotated subunits: 15 Subunits with signal: 10

Max. coeluting subunits: 8 Max. completeness: 0.53

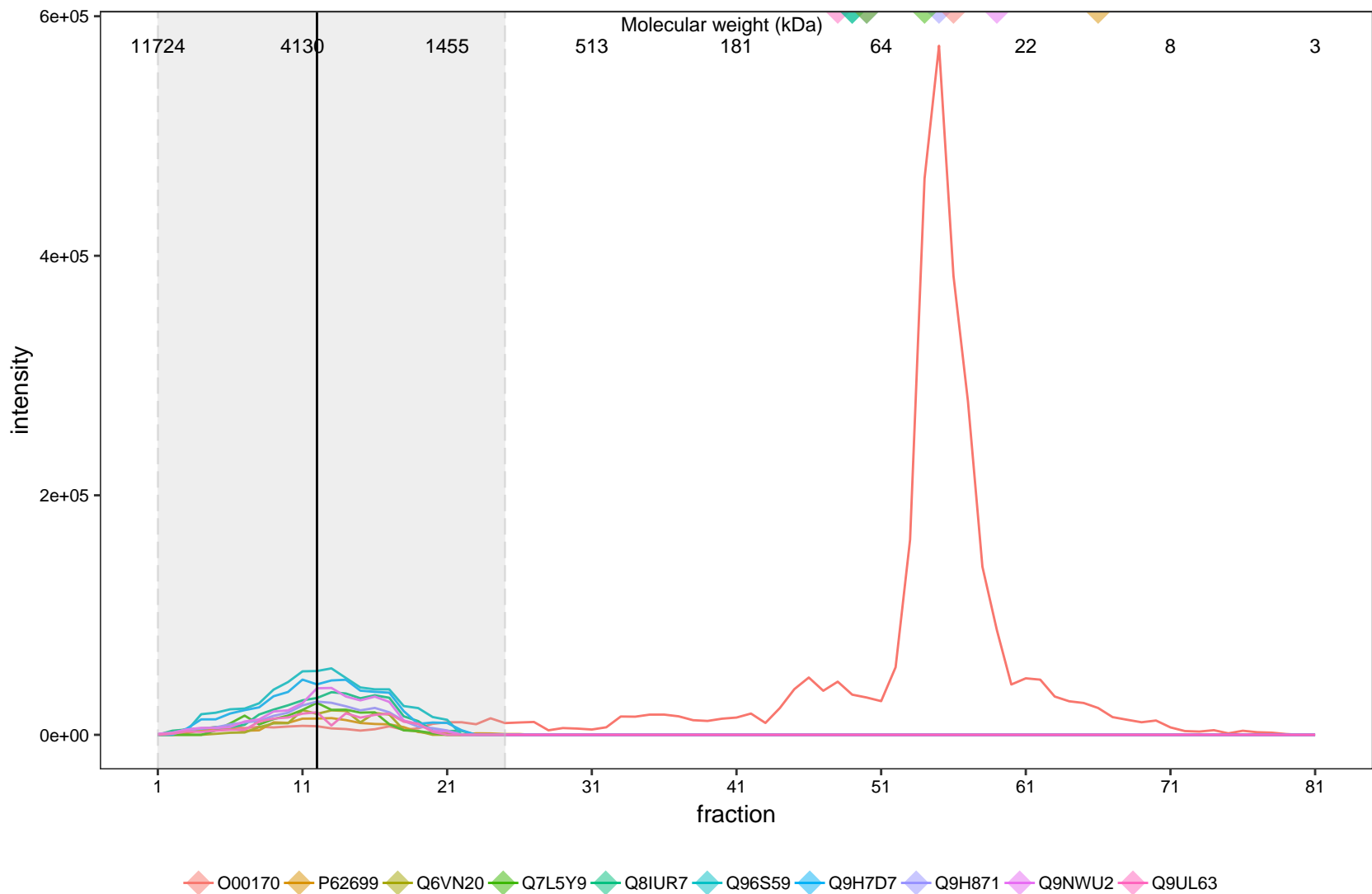

Supplement: Supplementary file 7 — Dataset EV6 [file MSB-15-e8438-s007.zip › feature_plots_bioplex/P13385.pdf]

**P13598**

**Annotated subunits: 14 Subunits with signal: 7**

**Max. coeluting subunits: 4 Max. completeness: 0.29**

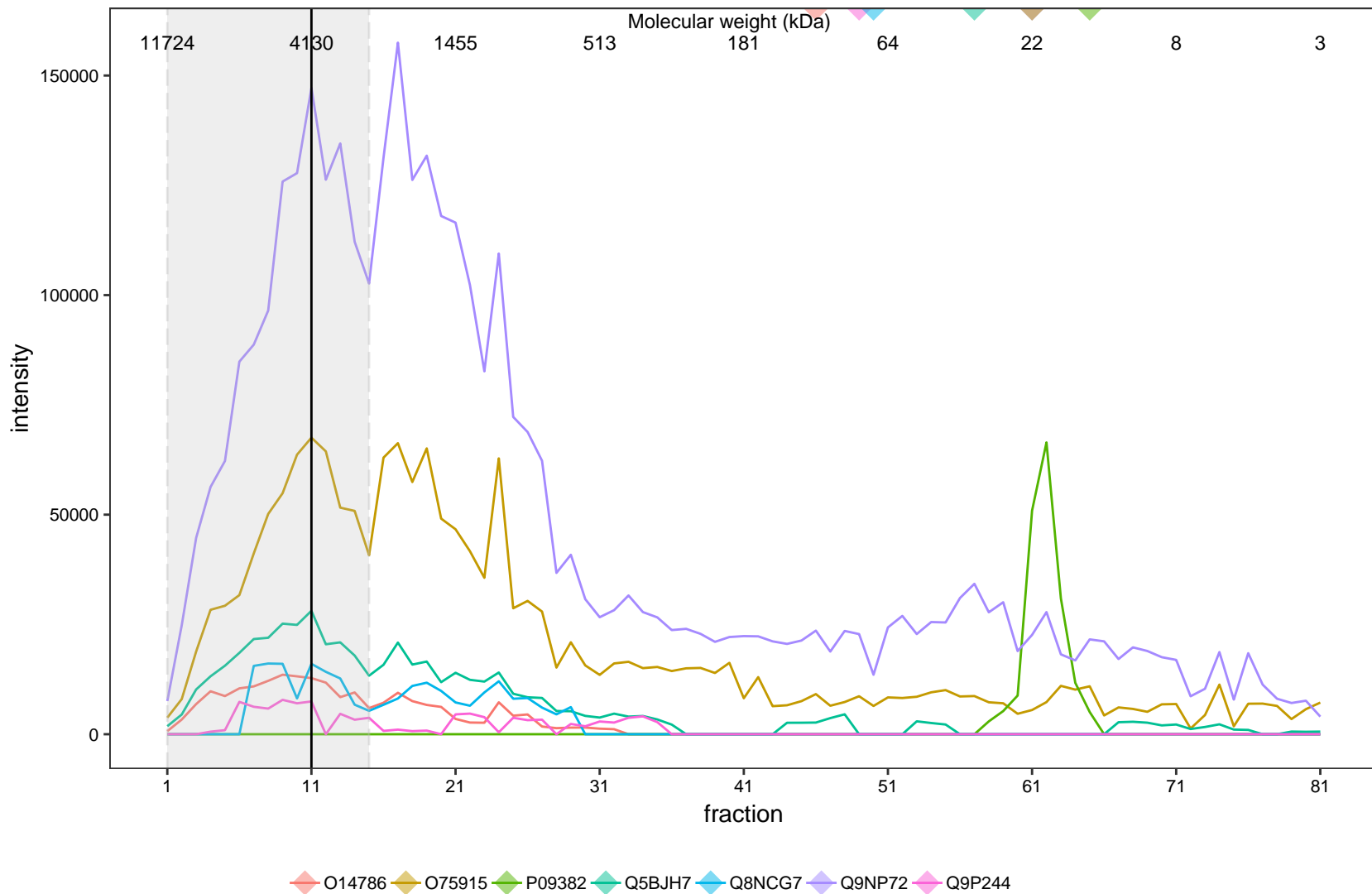

Supplement: Supplementary file 7 — Dataset EV6 [file MSB-15-e8438-s007.zip › feature_plots_bioplex/P13598.pdf]

**P13716**  
**Annotated subunits: 2   Subunits with signal: 2**  
**Max. coeluting subunits: 2   Max. completeness: 1**

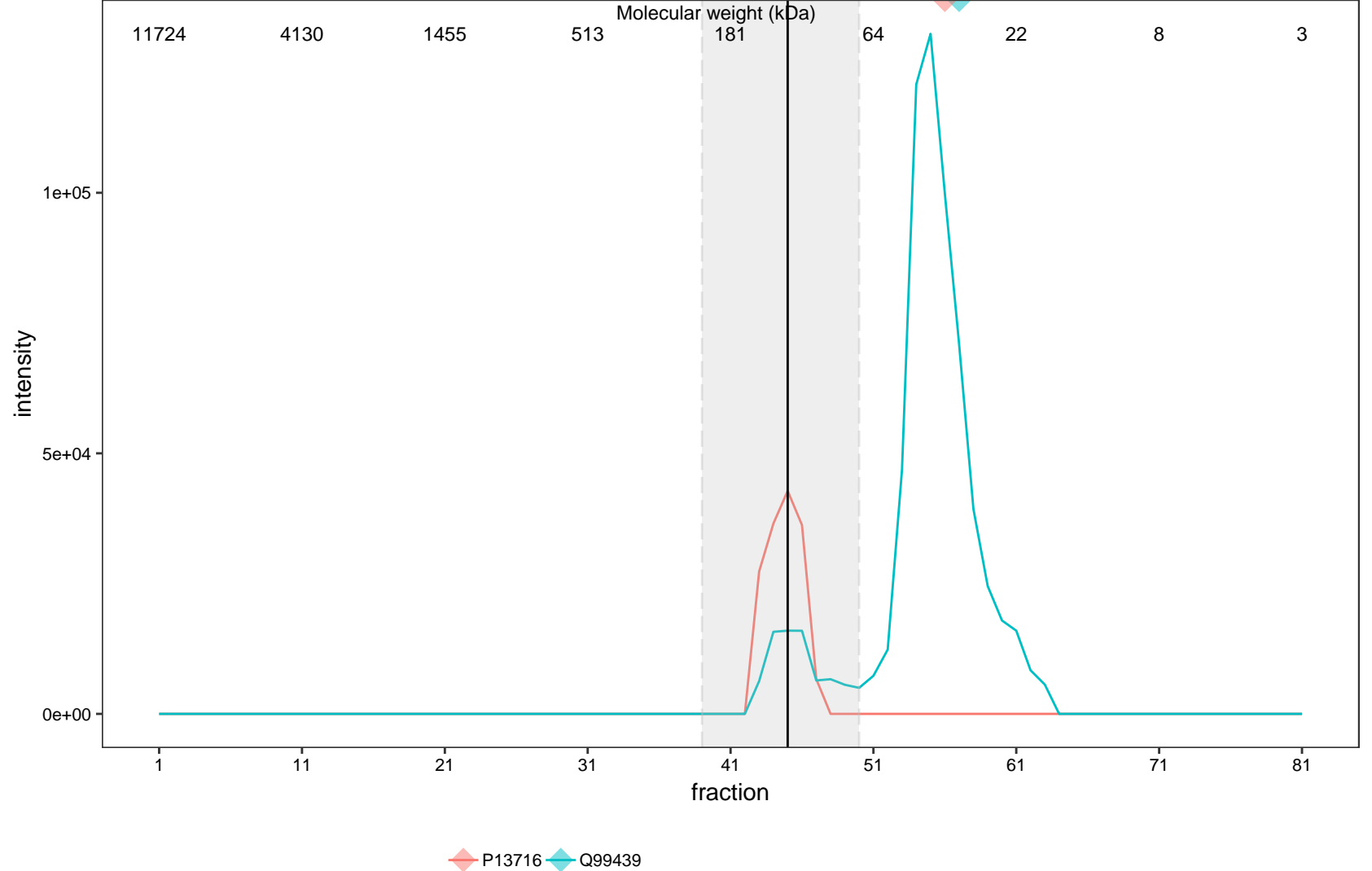

Supplement: Supplementary file 7 — Dataset EV6 [file MSB-15-e8438-s007.zip › feature_plots_bioplex/P13716.pdf]

**P13726**

**Annotated subunits: 5 Subunits with signal: 4**

**Max. coeluting subunits: 4 Max. completeness: 0.8**

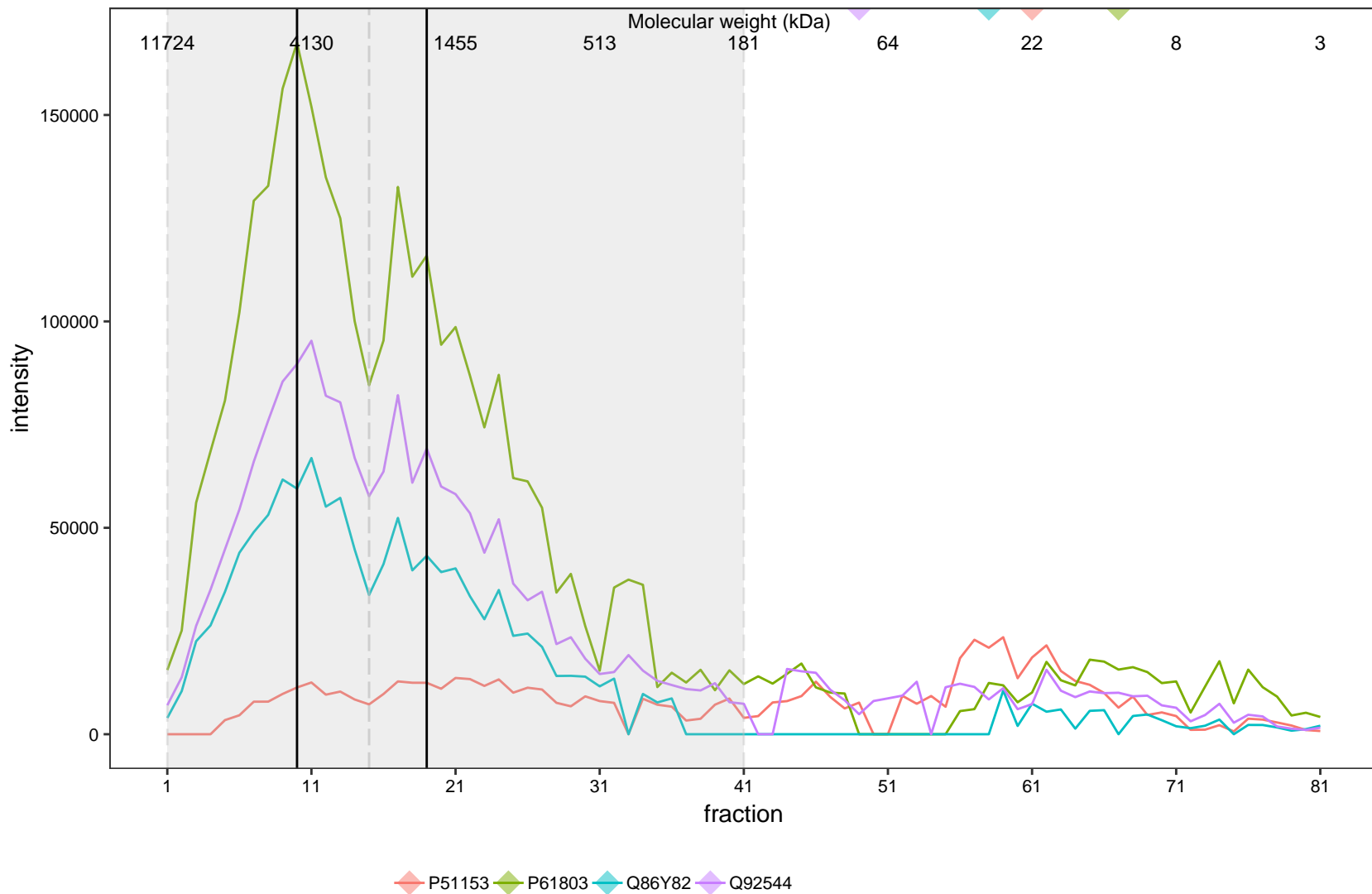

Supplement: Supplementary file 7 — Dataset EV6 [file MSB-15-e8438-s007.zip › feature_plots_bioplex/P13726.pdf]

**P13727**

**Annotated subunits: 56 Subunits with signal: 24**

**Max. coeluting subunits: 8 Max. completeness: 0.14**

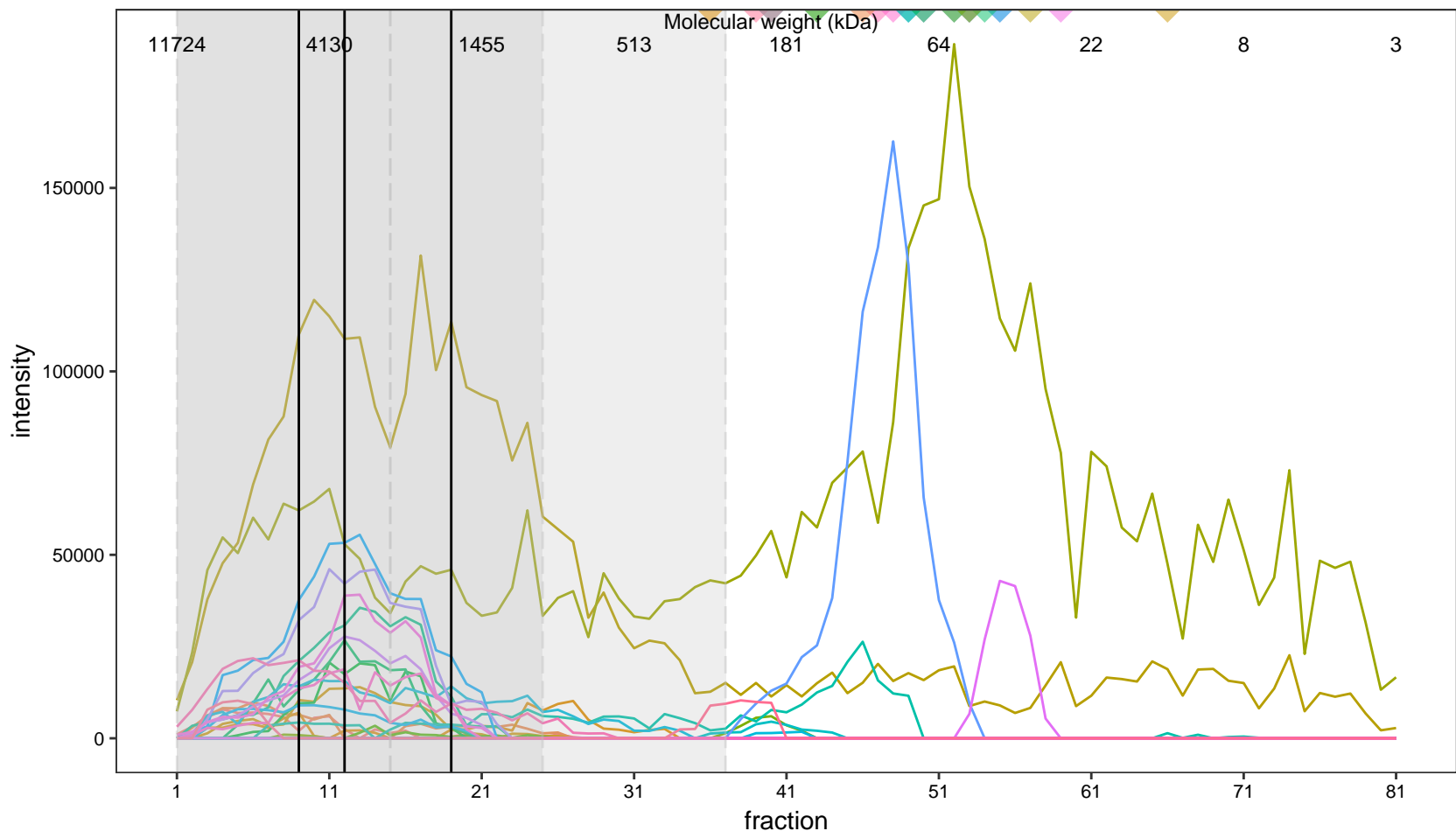

Supplement: Supplementary file 7 — Dataset EV6 [file MSB-15-e8438-s007.zip › feature_plots_bioplex/P13727.pdf]

A2A3L6  
Annotated subunits: 3   Subunits with signal: 2  
Max. coeluting subunits: 2   Max. completeness: 0.67

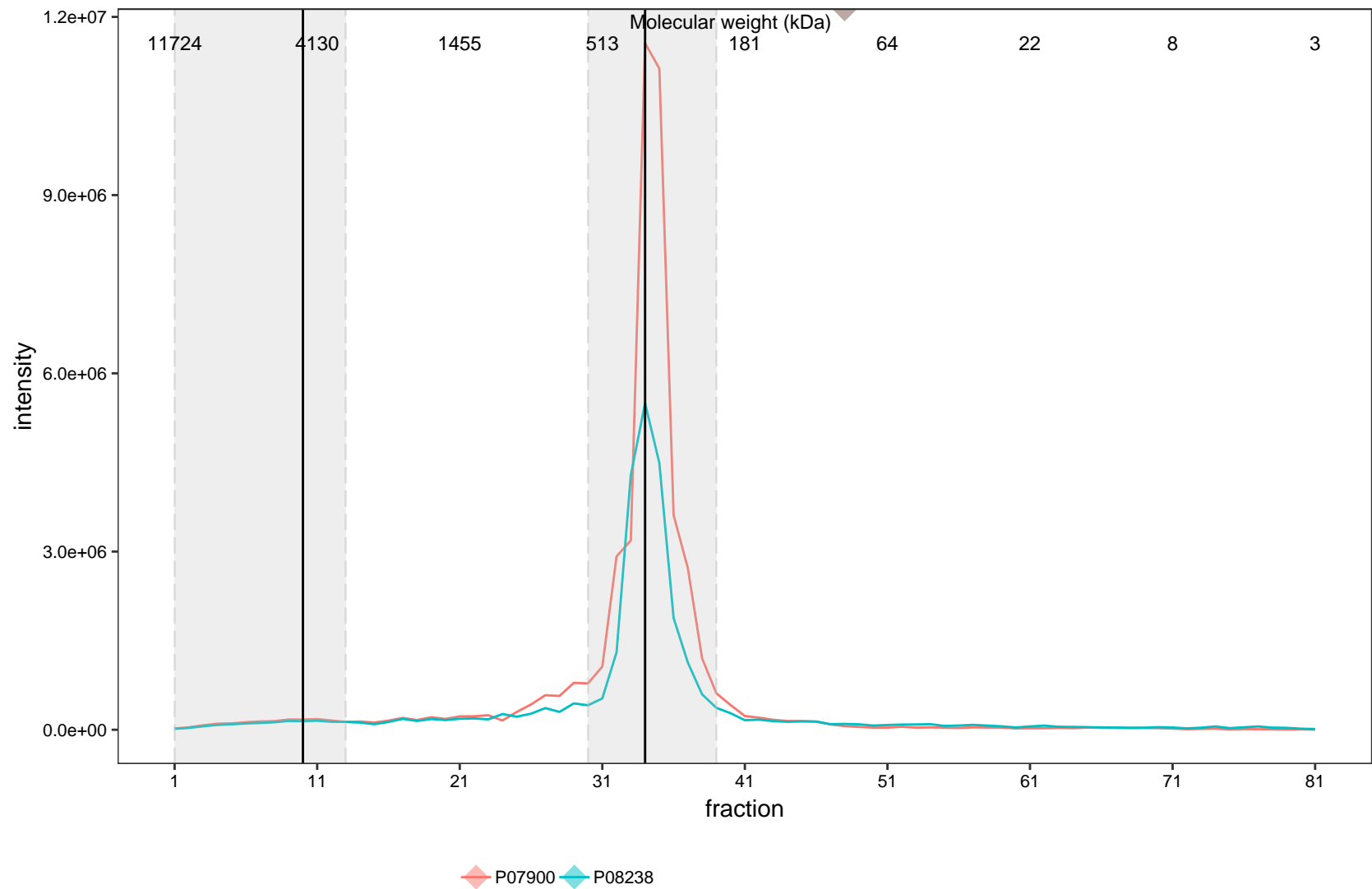

Supplement: Supplementary file 8 — Dataset EV7 [file MSB-15-e8438-s008.zip › feature_plots_string/A2A3L6.pdf]

# A2RU30

Annotated subunits: 11 Subunits with signal: 10

Max. coeluting subunits: 8 Max. completeness: 0.73

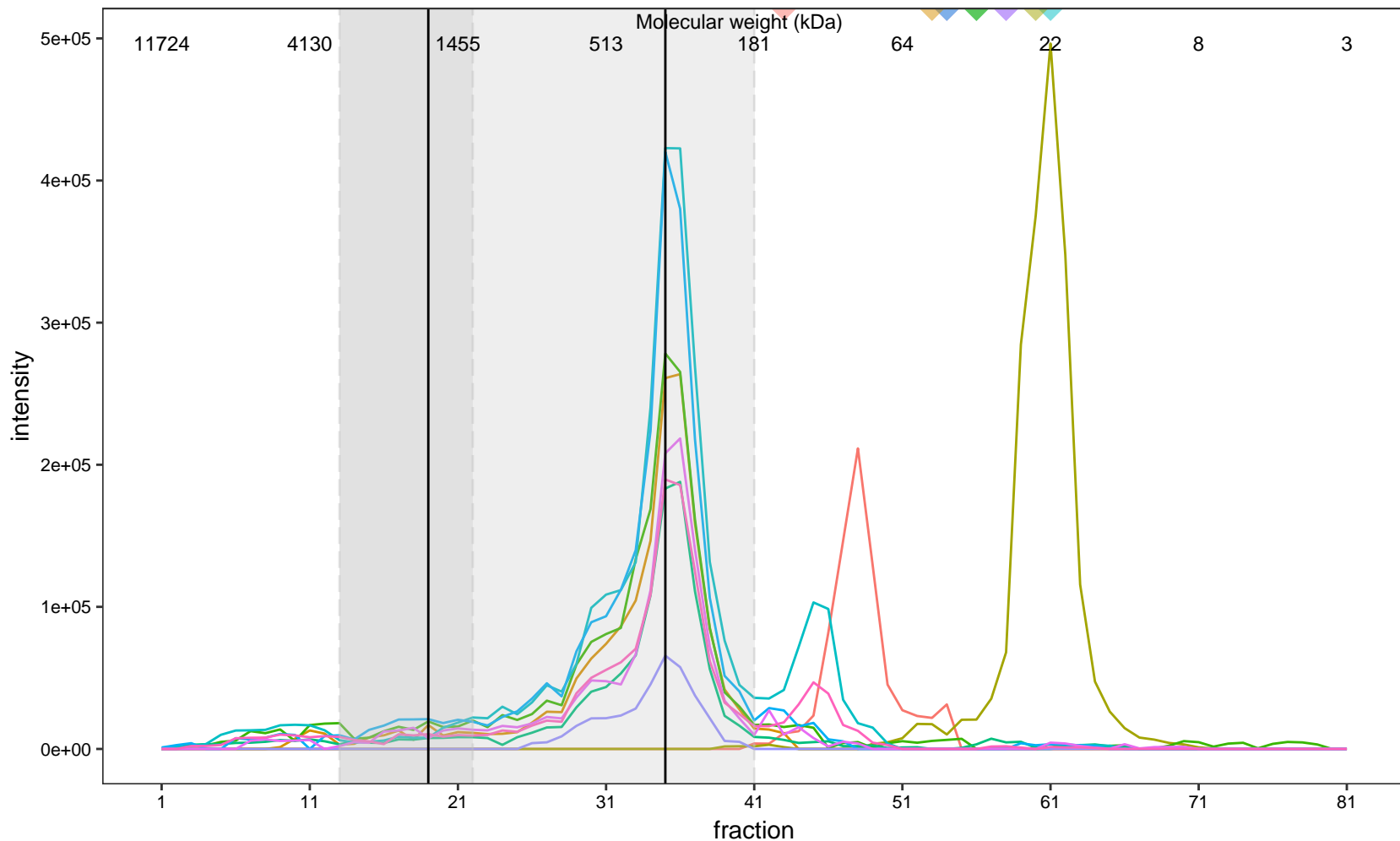

Supplement: Supplementary file 8 — Dataset EV7 [file MSB-15-e8438-s008.zip › feature_plots_string/A2RU30.pdf]

# A5YKK6

Annotated subunits: 25 Subunits with signal: 19

Max. coeluting subunits: 8 Max. completeness: 0.32

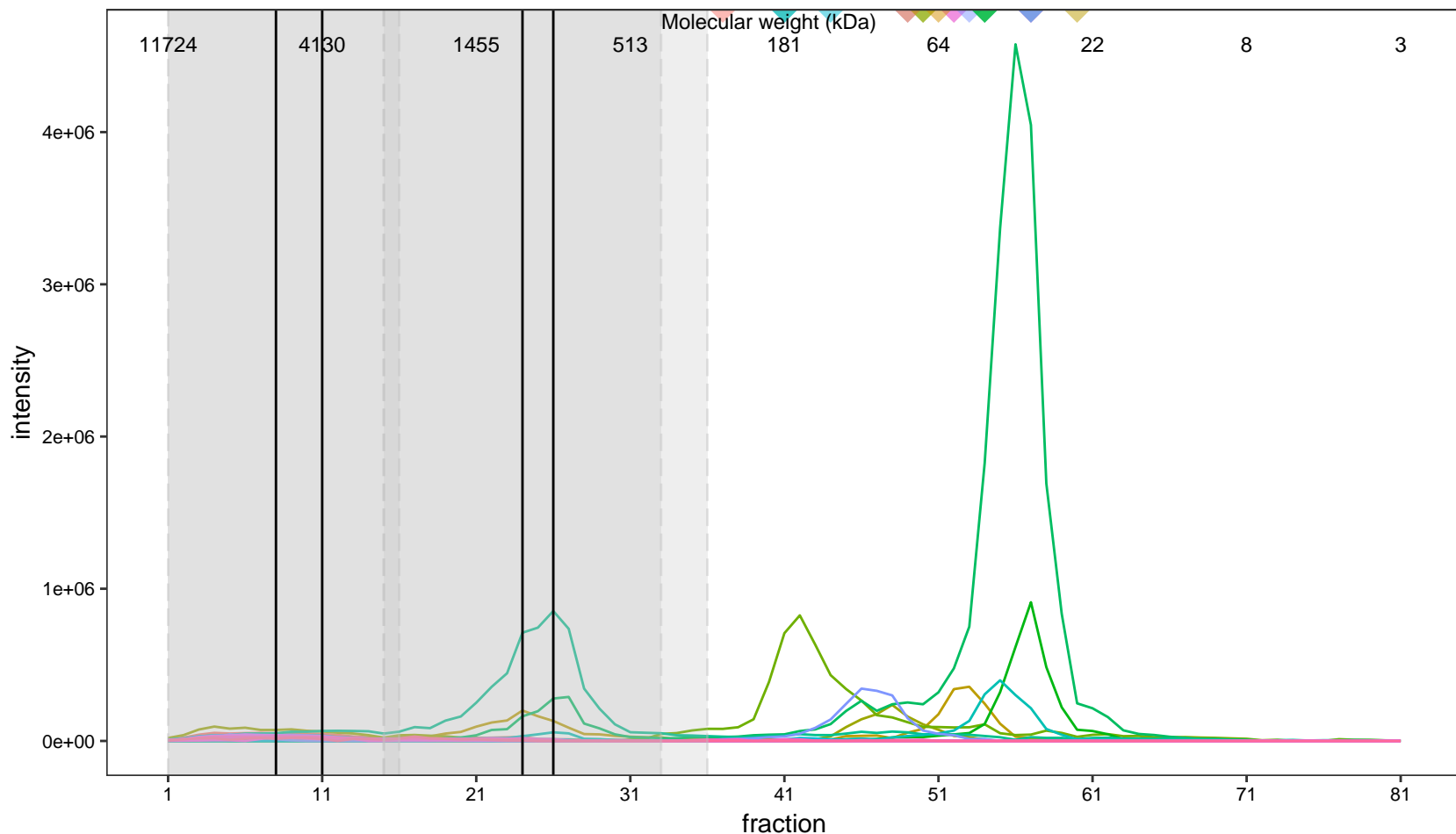

Supplement: Supplementary file 8 — Dataset EV7 [file MSB-15-e8438-s008.zip › feature_plots_string/A5YKK6.pdf]

E7EU14  
Annotated subunits: 3   Subunits with signal: 2  
Max. coeluting subunits: 2   Max. completeness: 0.67

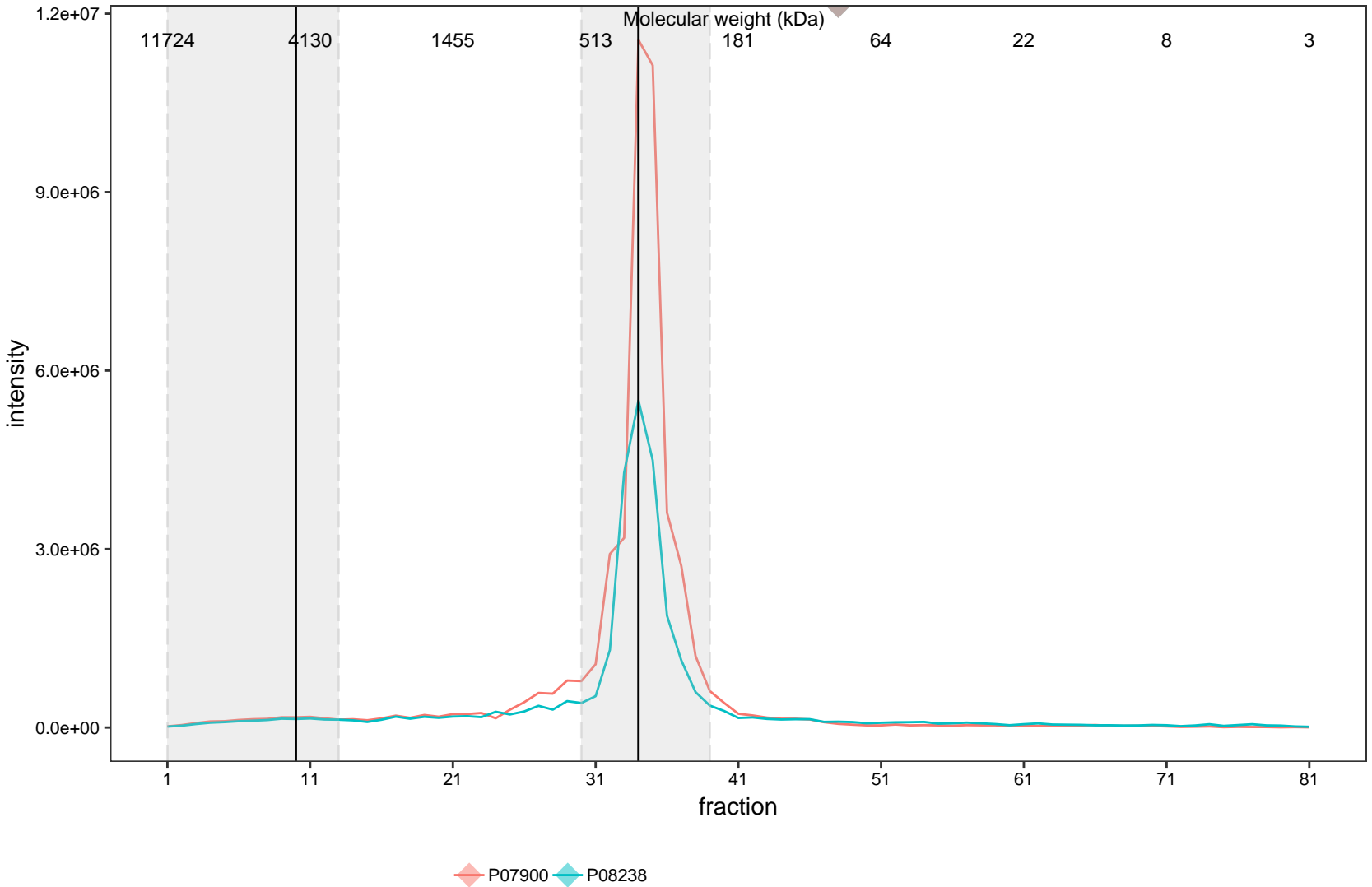

Supplement: Supplementary file 8 — Dataset EV7 [file MSB-15-e8438-s008.zip › feature_plots_string/E7EU14.pdf]

# E9PAV3

Annotated subunits: 4 Subunits with signal: 2

Max. coeluting subunits: 2 Max. completeness: 0.5

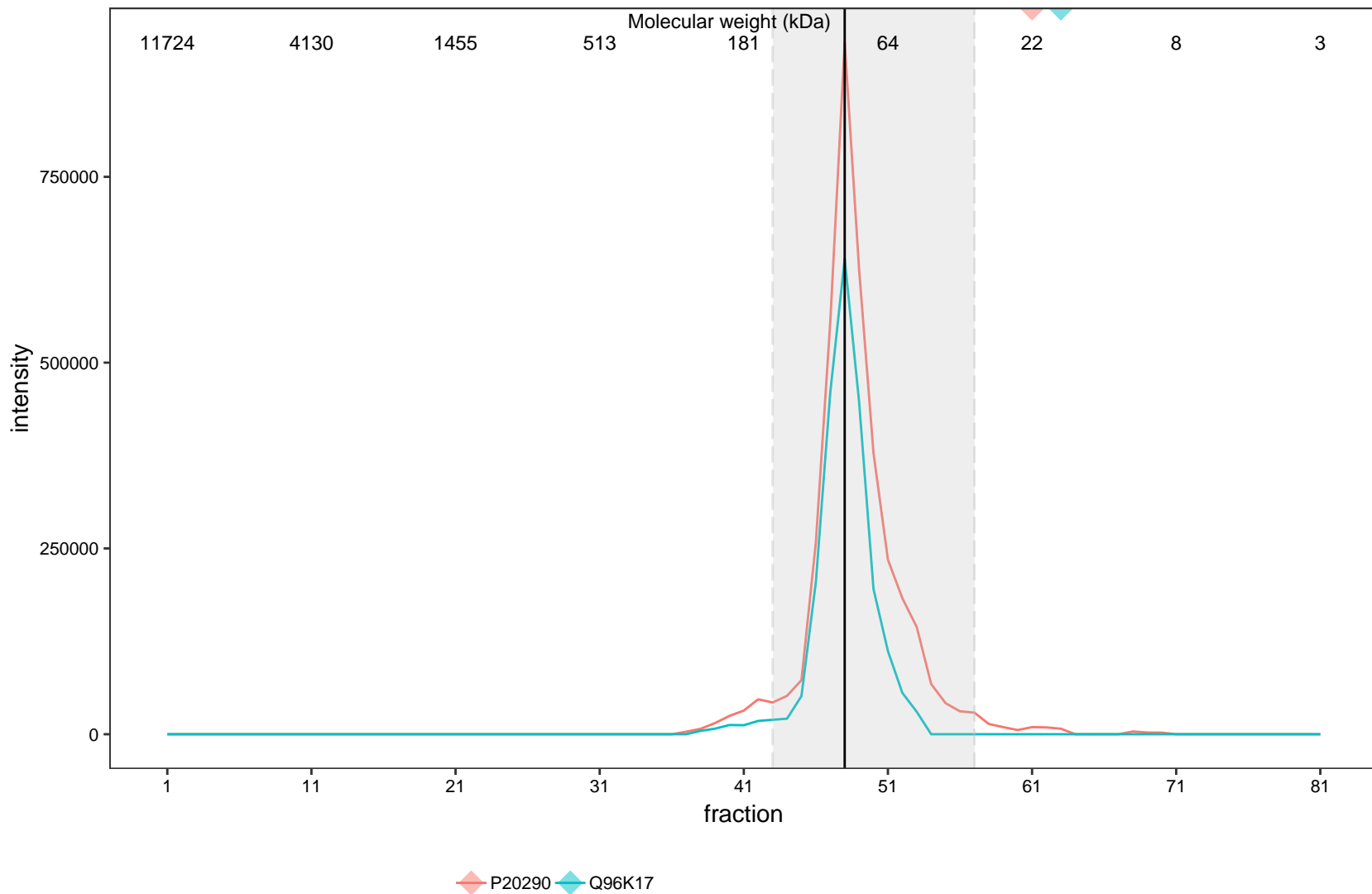

Supplement: Supplementary file 8 — Dataset EV7 [file MSB-15-e8438-s008.zip › feature_plots_string/E9PAV3.pdf]

**000116**

**Annotated subunits: 5   Subunits with signal: 4**

**Max. coeluting subunits: 3   Max. completeness: 0.6**

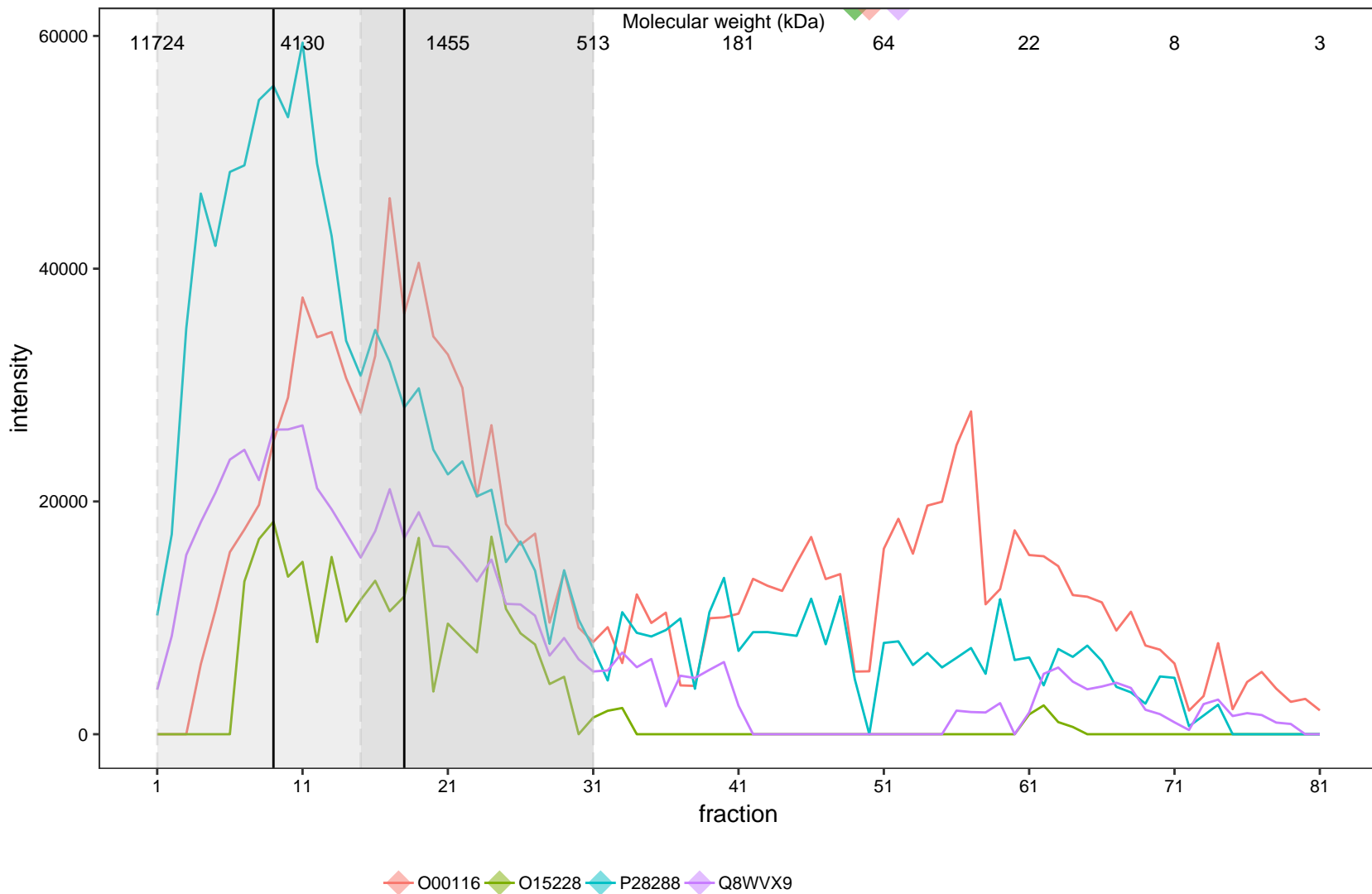

Supplement: Supplementary file 8 — Dataset EV7 [file MSB-15-e8438-s008.zip › feature_plots_string/O00116.pdf]

O00151  
Annotated subunits: 4   Subunits with signal: 3  
Max. coeluting subunits: 2   Max. completeness: 0.5

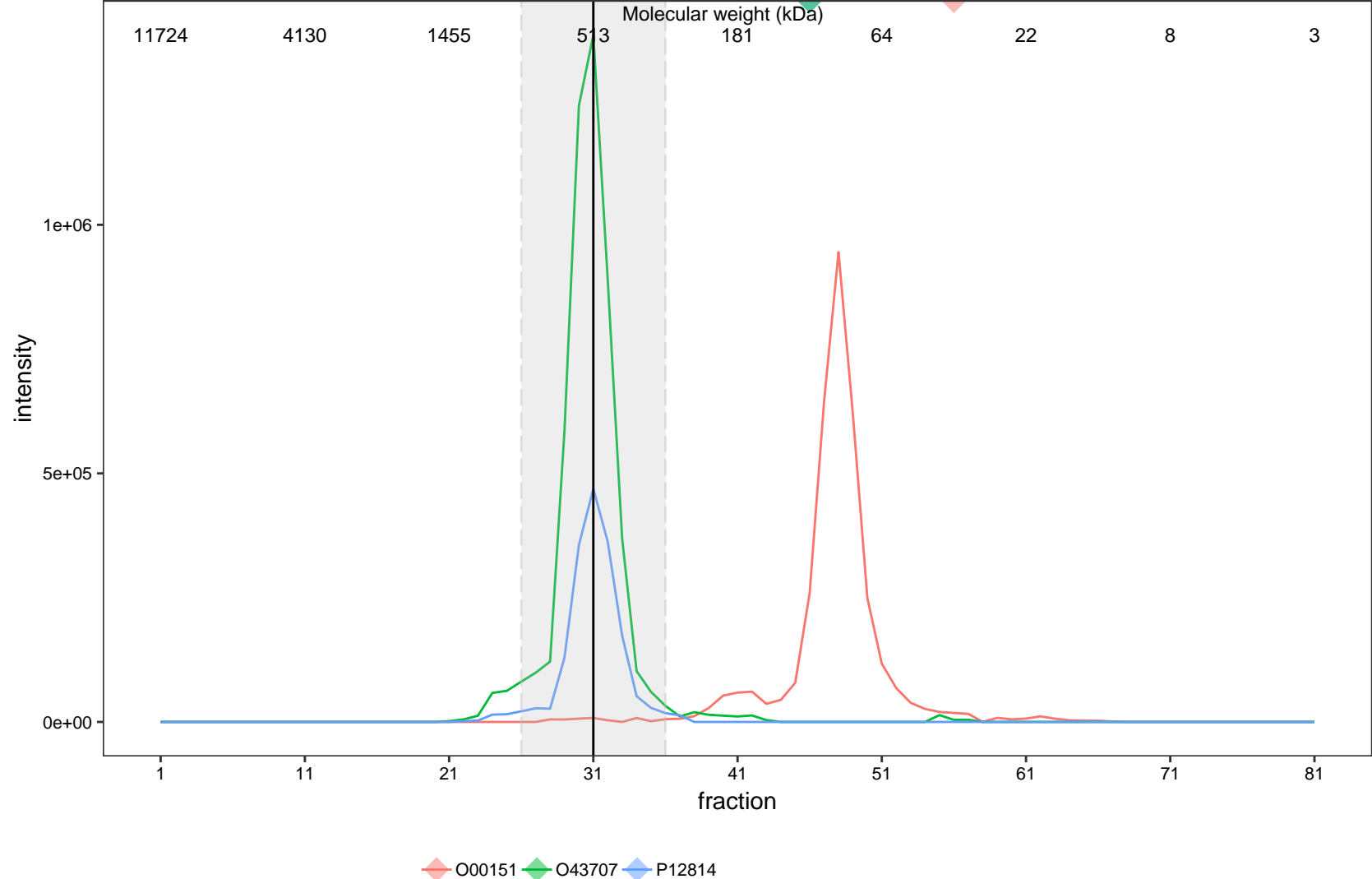

Supplement: Supplementary file 8 — Dataset EV7 [file MSB-15-e8438-s008.zip › feature_plots_string/O00151.pdf]

**O00161**

**Annotated subunits: 46 Subunits with signal: 30**

**Max. coeluting subunits: 23 Max. completeness: 0.5**

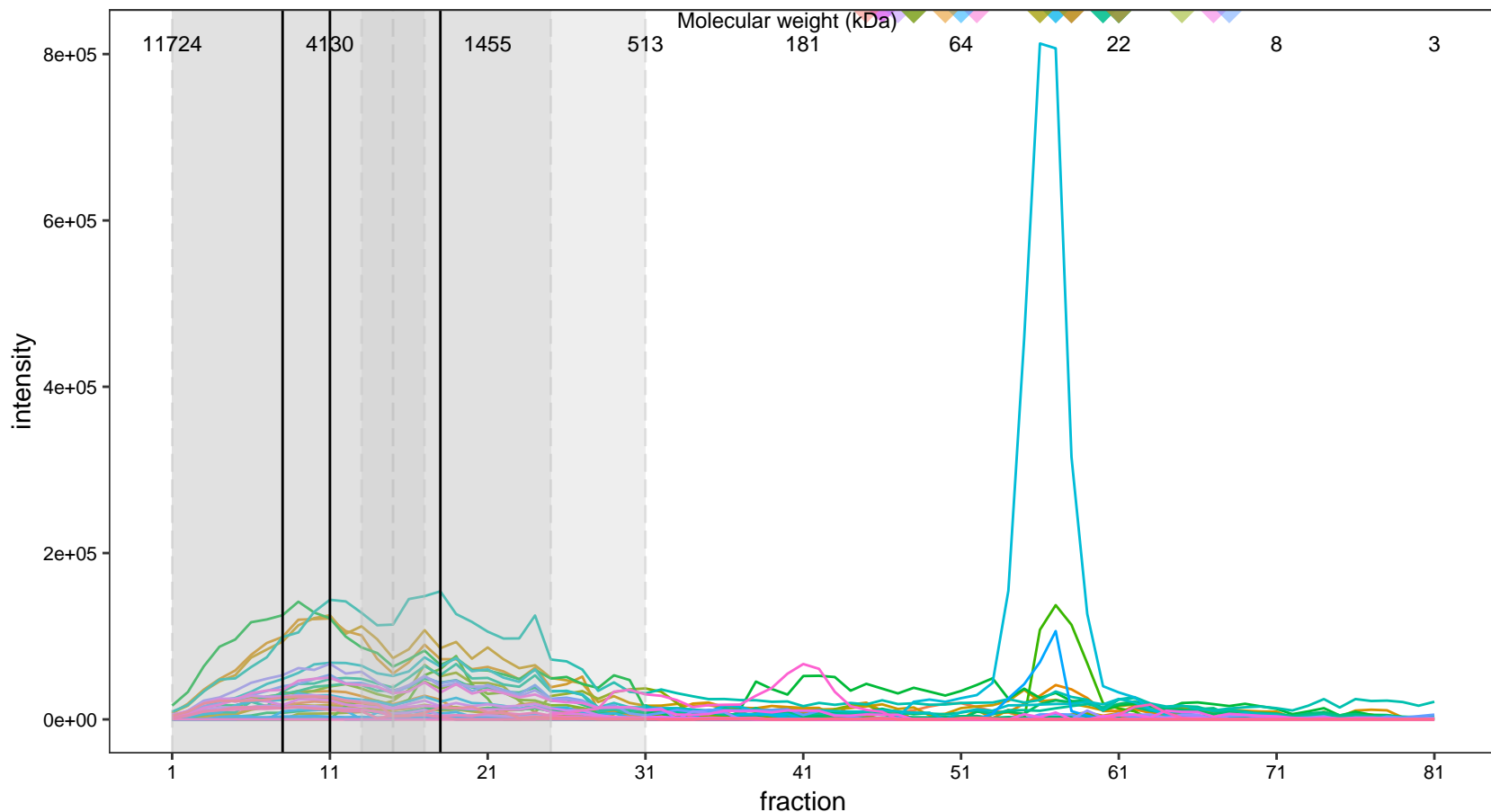

Supplement: Supplementary file 8 — Dataset EV7 [file MSB-15-e8438-s008.zip › feature_plots_string/O00161.pdf]

**O00170**  
**Annotated subunits: 6   Subunits with signal: 3**  
**Max. coeluting subunits: 2   Max. completeness: 0.33**

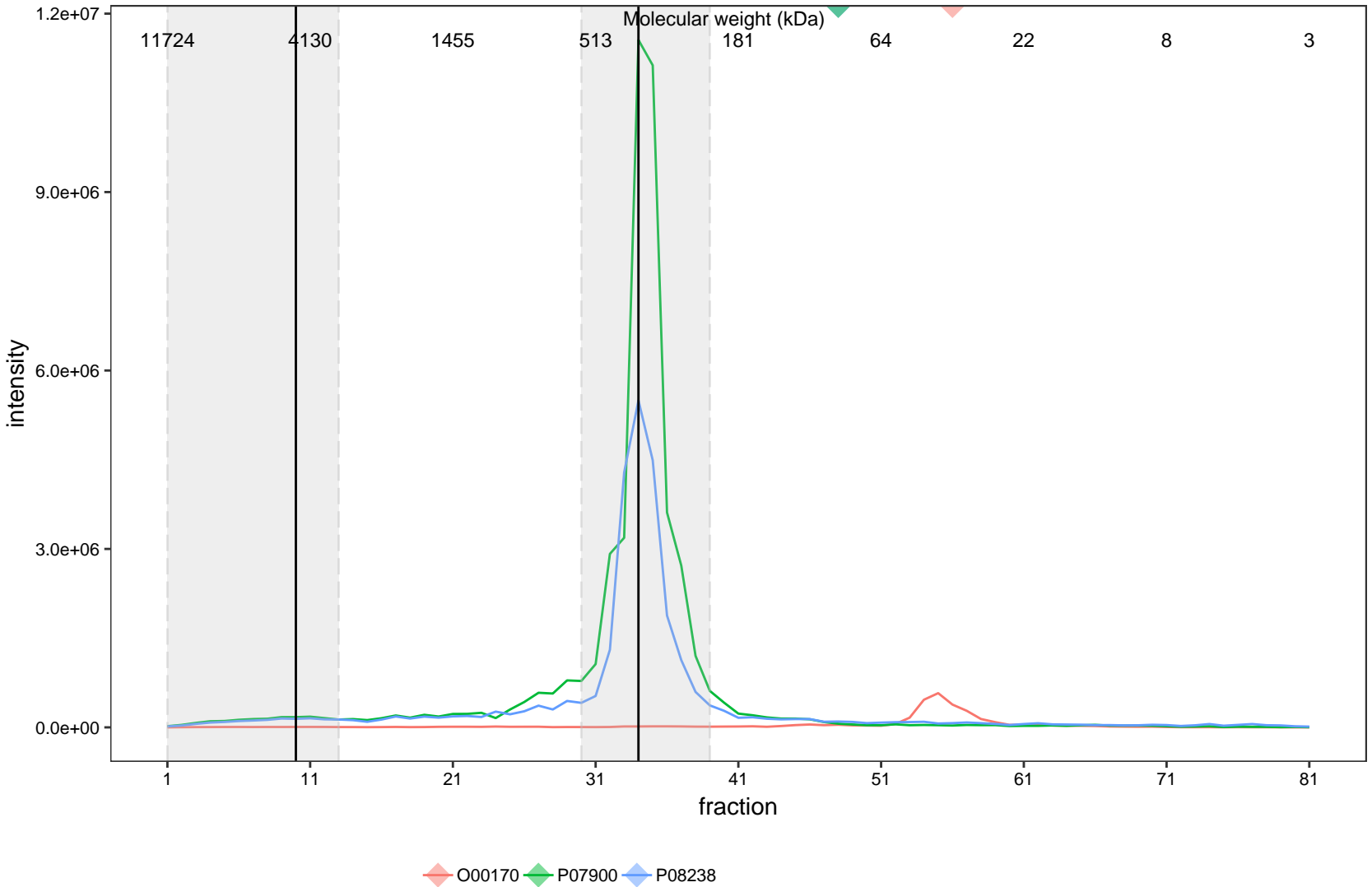

Supplement: Supplementary file 8 — Dataset EV7 [file MSB-15-e8438-s008.zip › feature_plots_string/O00170.pdf]

**000189**

**Annotated subunits: 15 Subunits with signal: 9**

**Max. coeluting subunits: 5 Max. completeness: 0.33**

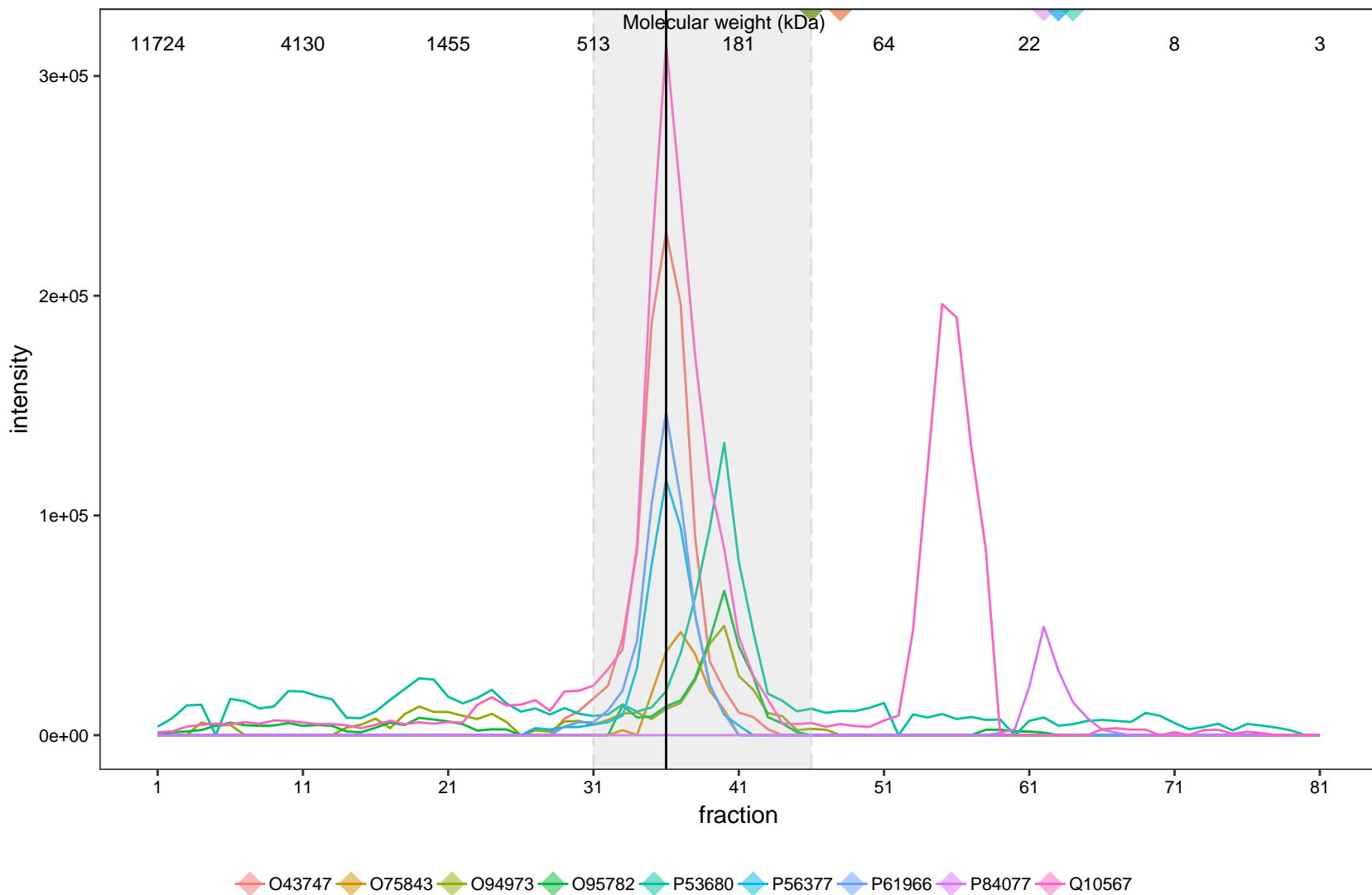

Supplement: Supplementary file 8 — Dataset EV7 [file MSB-15-e8438-s008.zip › feature_plots_string/O00189.pdf]

**O00203**

**Annotated subunits: 8 Subunits with signal: 6**

**Max. coeluting subunits: 3 Max. completeness: 0.38**

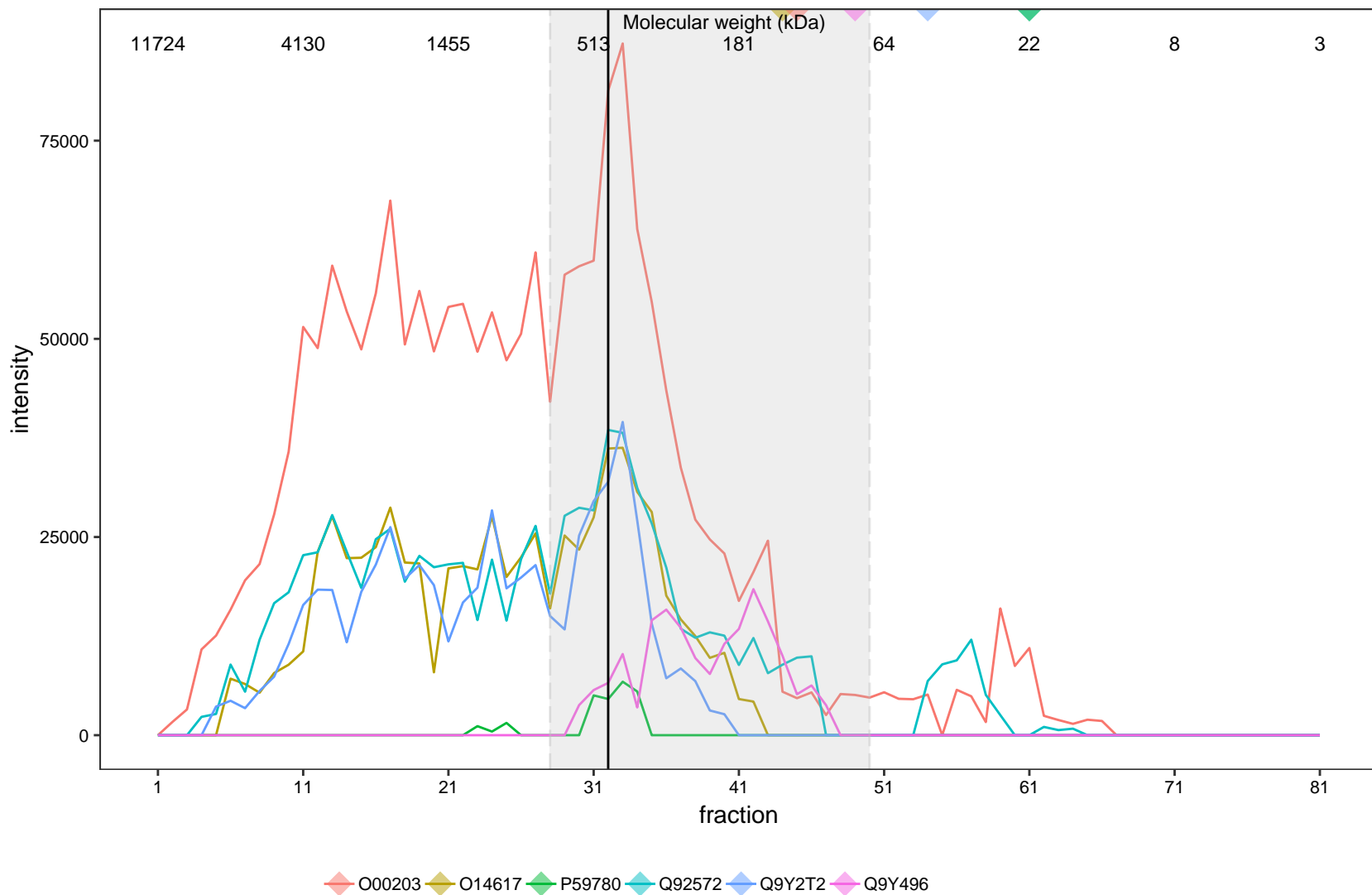

Supplement: Supplementary file 8 — Dataset EV7 [file MSB-15-e8438-s008.zip › feature_plots_string/O00203.pdf]

**O00264**  
**Annotated subunits: 5   Subunits with signal: 4**  
**Max. coeluting subunits: 2   Max. completeness: 0.4**

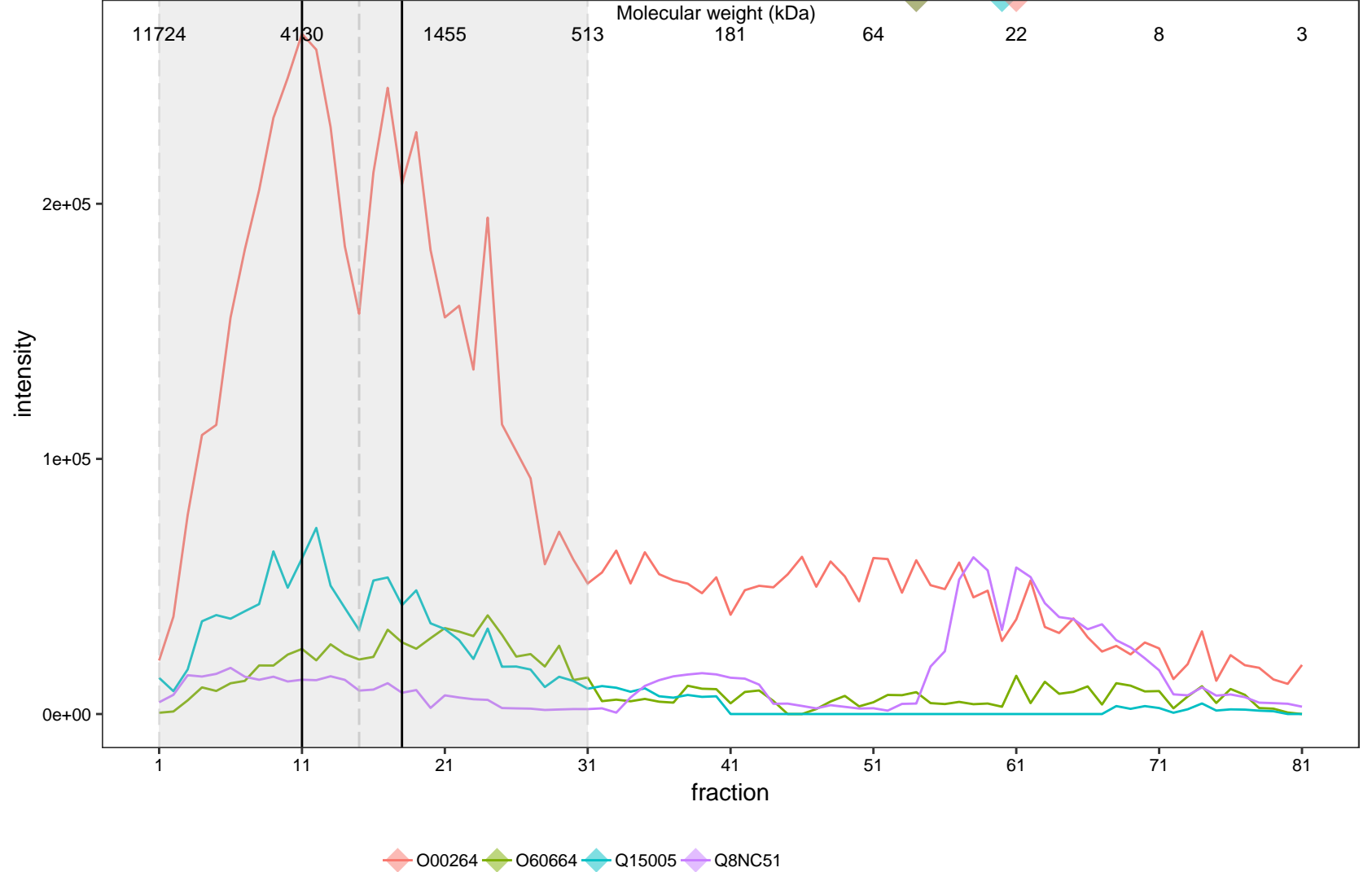

Supplement: Supplementary file 8 — Dataset EV7 [file MSB-15-e8438-s008.zip › feature_plots_string/O00264.pdf]

**O00273**  
**Annotated subunits: 7   Subunits with signal: 5**  
**Max. coeluting subunits: 3   Max. completeness: 0.43**

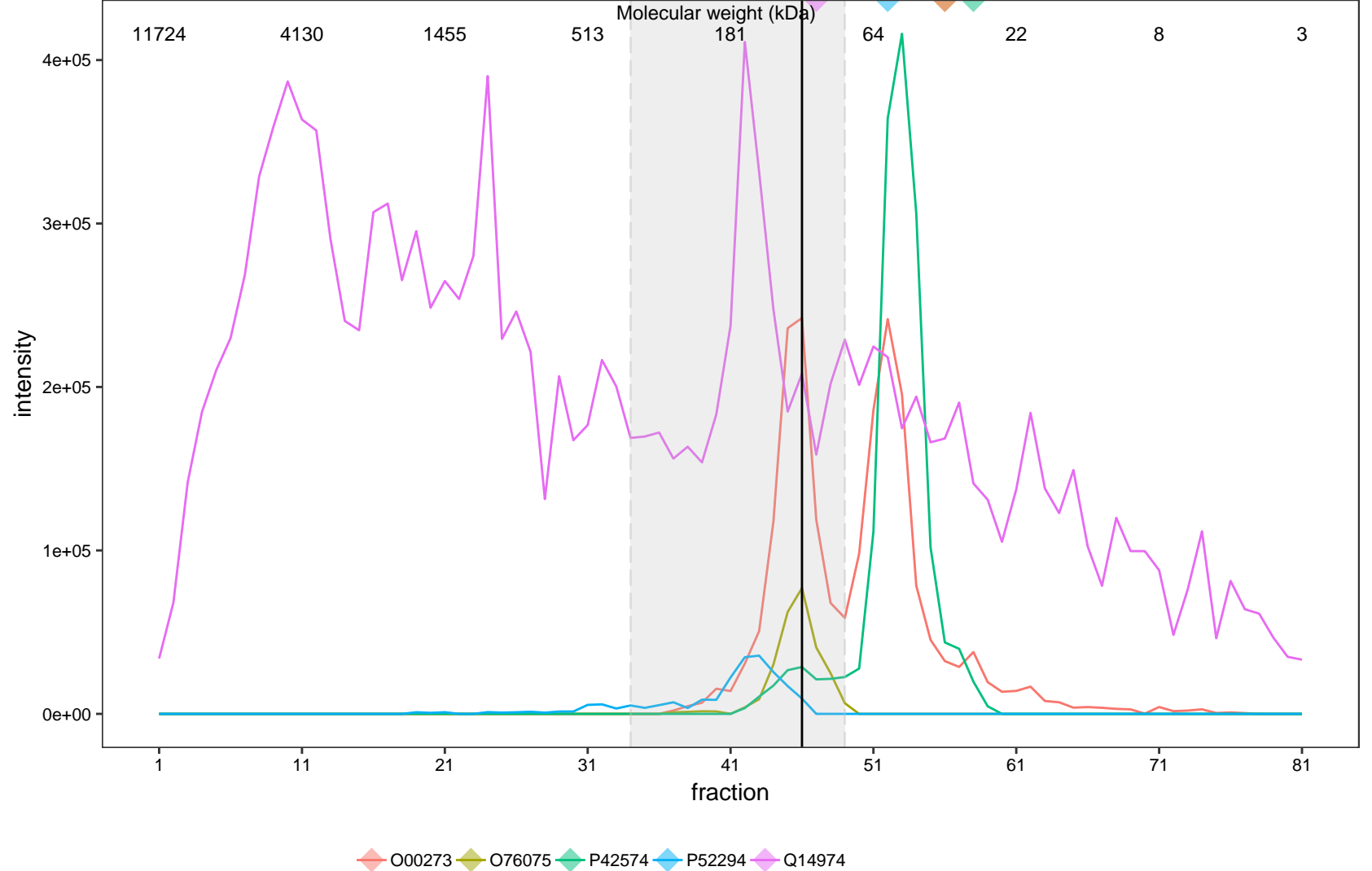

Supplement: Supplementary file 8 — Dataset EV7 [file MSB-15-e8438-s008.zip › feature_plots_string/O00273.pdf]

**O00291**

**Annotated subunits: 3 Subunits with signal: 3**

**Max. coeluting subunits: 2 Max. completeness: 0.67**

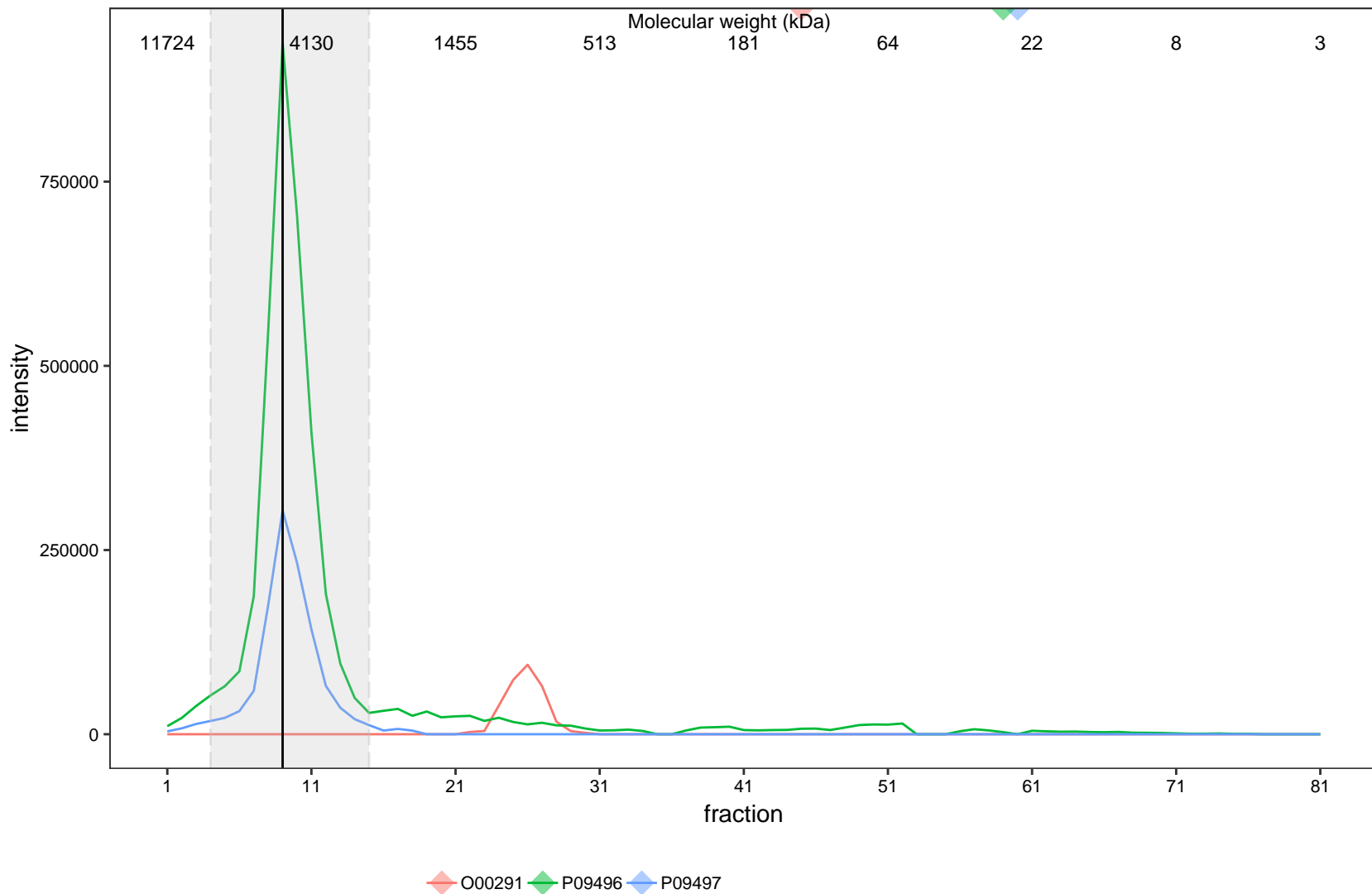

Supplement: Supplementary file 8 — Dataset EV7 [file MSB-15-e8438-s008.zip › feature_plots_string/O00291.pdf]

**O00399**

**Annotated subunits: 32 Subunits with signal: 18**

**Max. coeluting subunits: 8 Max. completeness: 0.25**

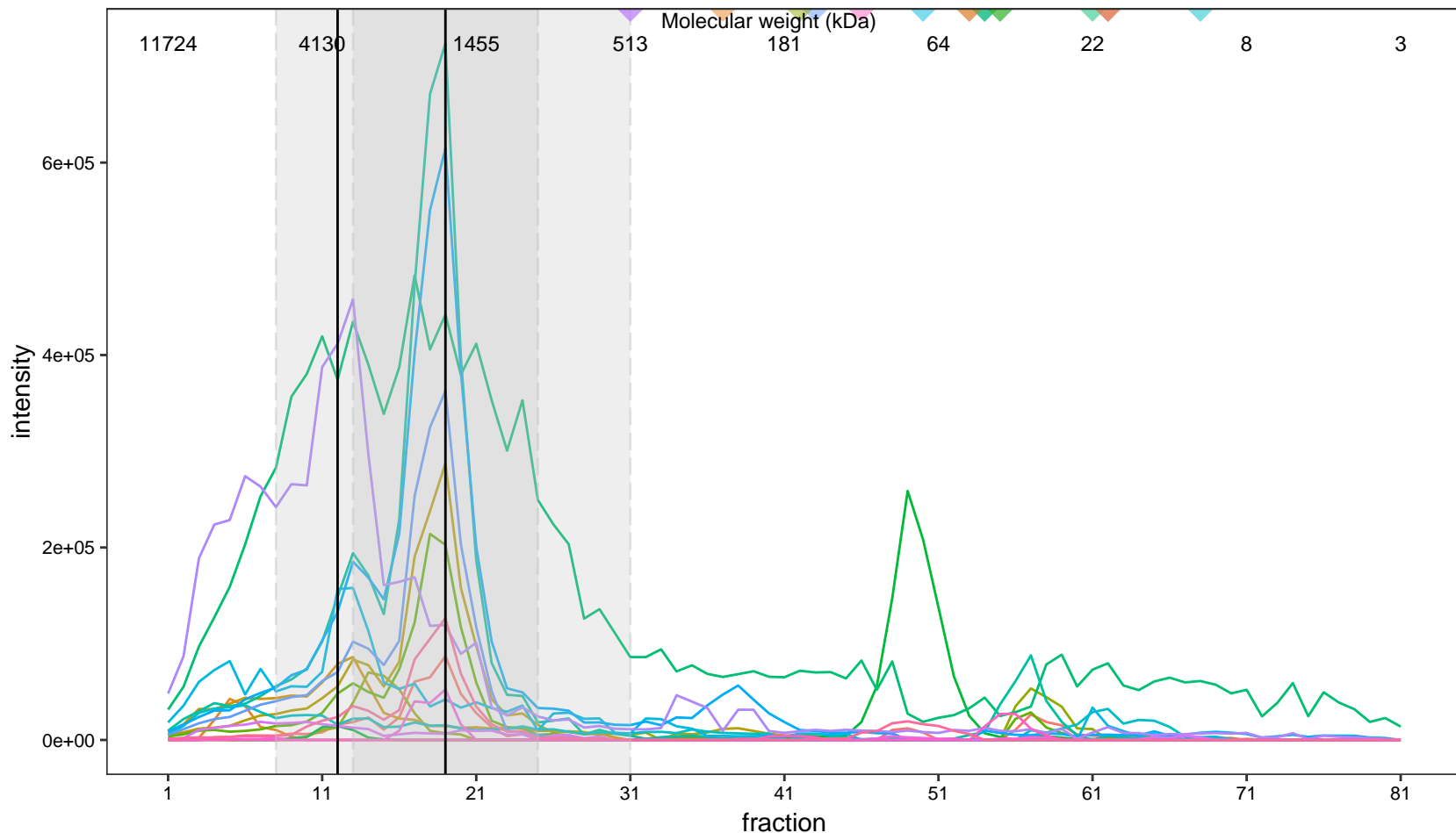

Supplement: Supplementary file 8 — Dataset EV7 [file MSB-15-e8438-s008.zip › feature_plots_string/O00399.pdf]

**O00410**

**Annotated subunits: 14 Subunits with signal: 9**

**Max. coeluting subunits: 8 Max. completeness: 0.57**

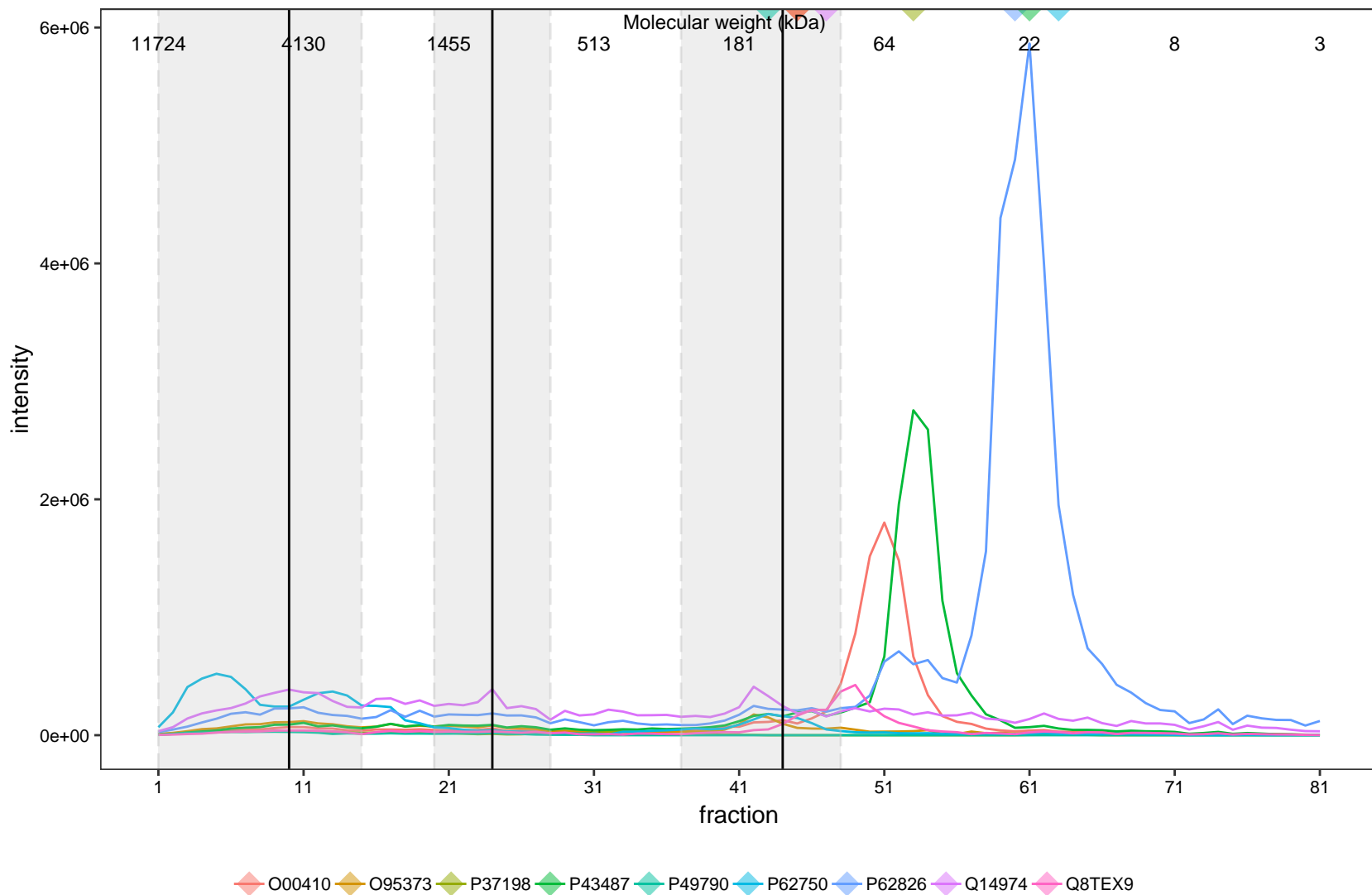

Supplement: Supplementary file 8 — Dataset EV7 [file MSB-15-e8438-s008.zip › feature_plots_string/O00410.pdf]

**Annotated subunits: 67   Subunits with signal: 57**  
**Max. coeluting subunits: 31   Max. completeness: 0.46**

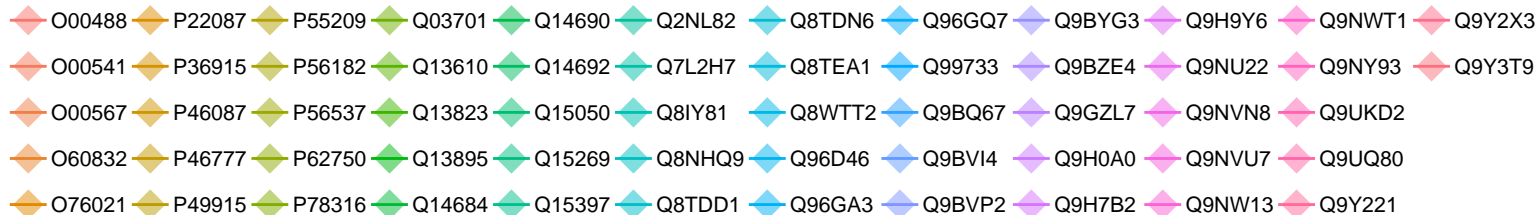

Supplement: Supplementary file 8 — Dataset EV7 [file MSB-15-e8438-s008.zip › feature_plots_string/O00541.pdf]

**O00566**

**Annotated subunits: 48 Subunits with signal: 28**

**Max. coeluting subunits: 13 Max. completeness: 0.27**

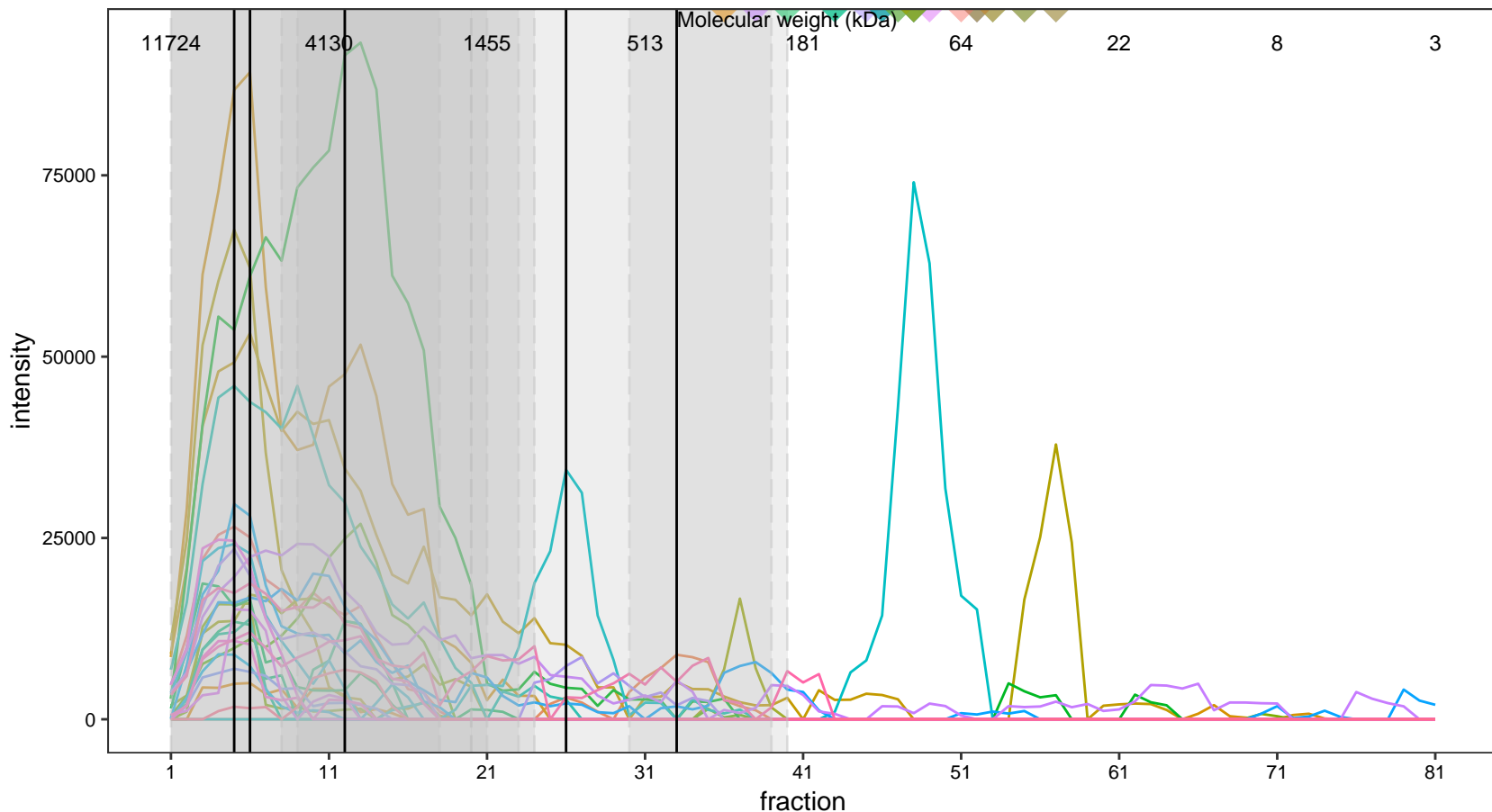

Supplement: Supplementary file 8 — Dataset EV7 [file MSB-15-e8438-s008.zip › feature_plots_string/O00566.pdf]

O00567

Annotated subunits: 77 Subunits with signal: 57

Max. coeluting subunits: 26 Max. completeness: 0.34

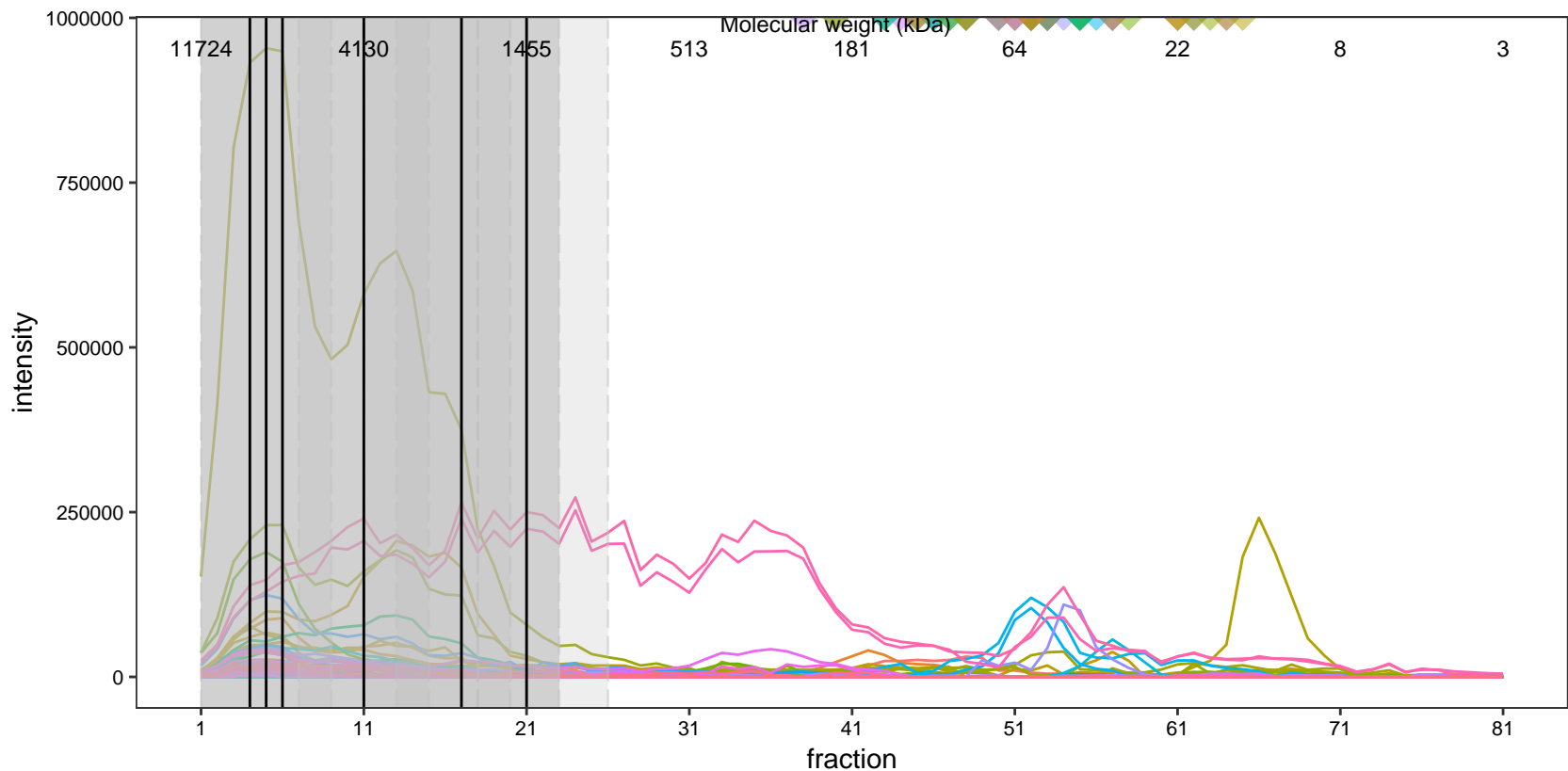

Supplement: Supplementary file 8 — Dataset EV7 [file MSB-15-e8438-s008.zip › feature_plots_string/O00567.pdf]

**O00571**

**Annotated subunits: 11 Subunits with signal: 8**

**Max. coeluting subunits: 4 Max. completeness: 0.36**

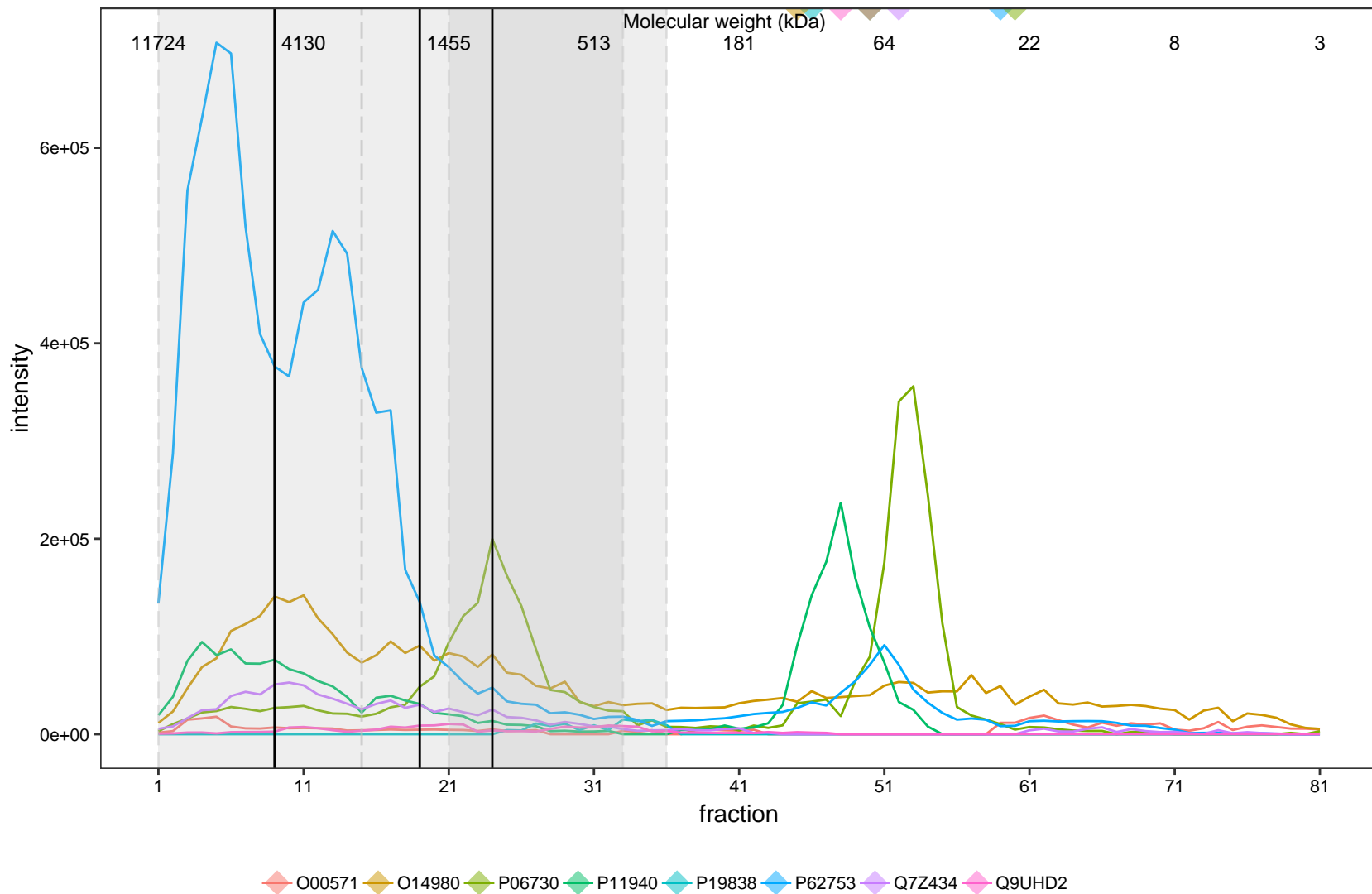

Supplement: Supplementary file 8 — Dataset EV7 [file MSB-15-e8438-s008.zip › feature_plots_string/O00571.pdf]

O00622  
Annotated subunits: 17   Subunits with signal: 5  
Max. coeluting subunits: 3   Max. completeness: 0.18

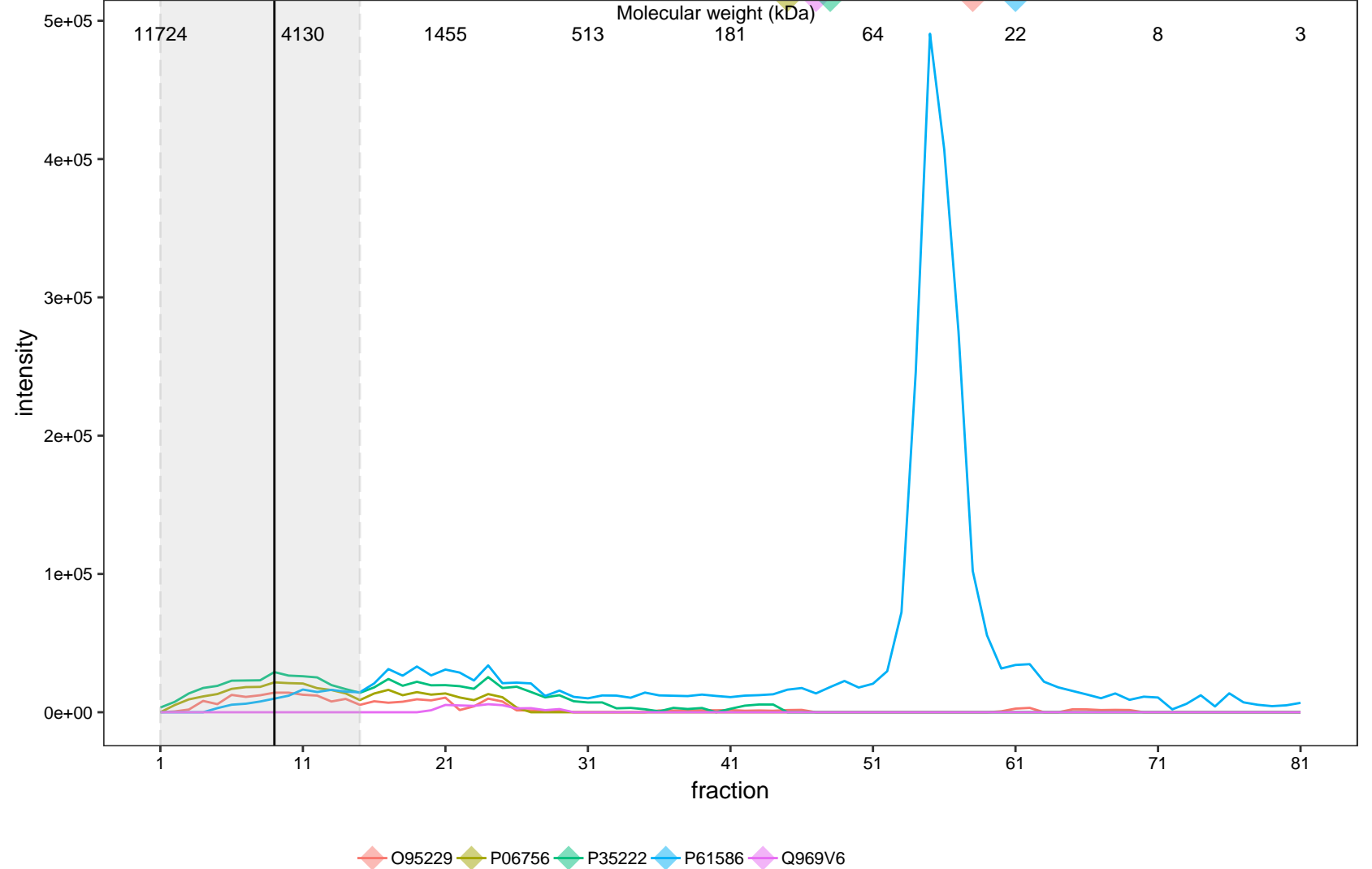

Supplement: Supplementary file 8 — Dataset EV7 [file MSB-15-e8438-s008.zip › feature_plots_string/O00622.pdf]

**O00623**

**Annotated subunits: 10   Subunits with signal: 3**

**Max. coeluting subunits: 2   Max. completeness: 0.2**

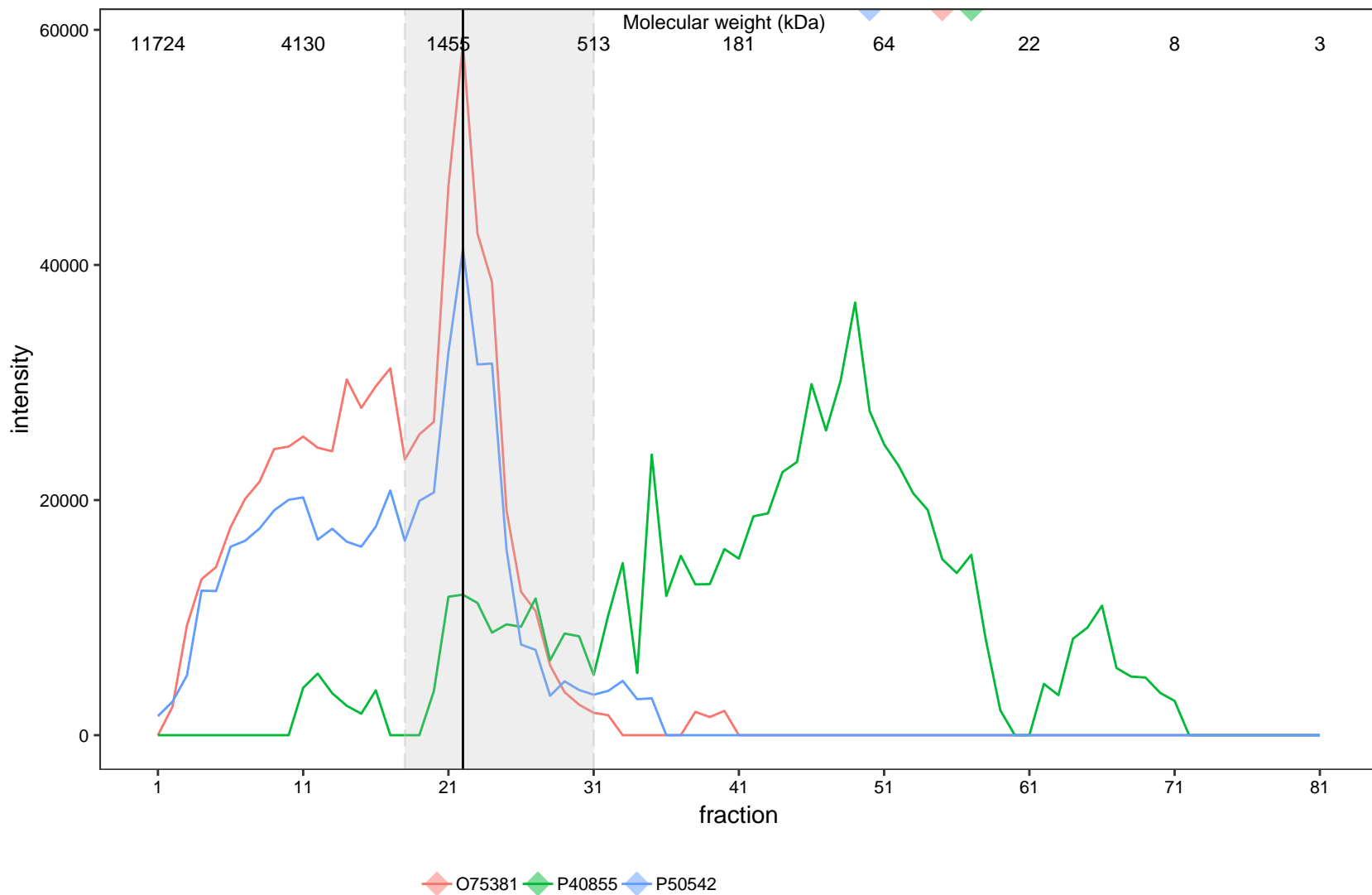

Supplement: Supplementary file 8 — Dataset EV7 [file MSB-15-e8438-s008.zip › feature_plots_string/O00623.pdf]

O00628  
Annotated subunits: 7   Subunits with signal: 5  
Max. coeluting subunits: 2   Max. completeness: 0.29

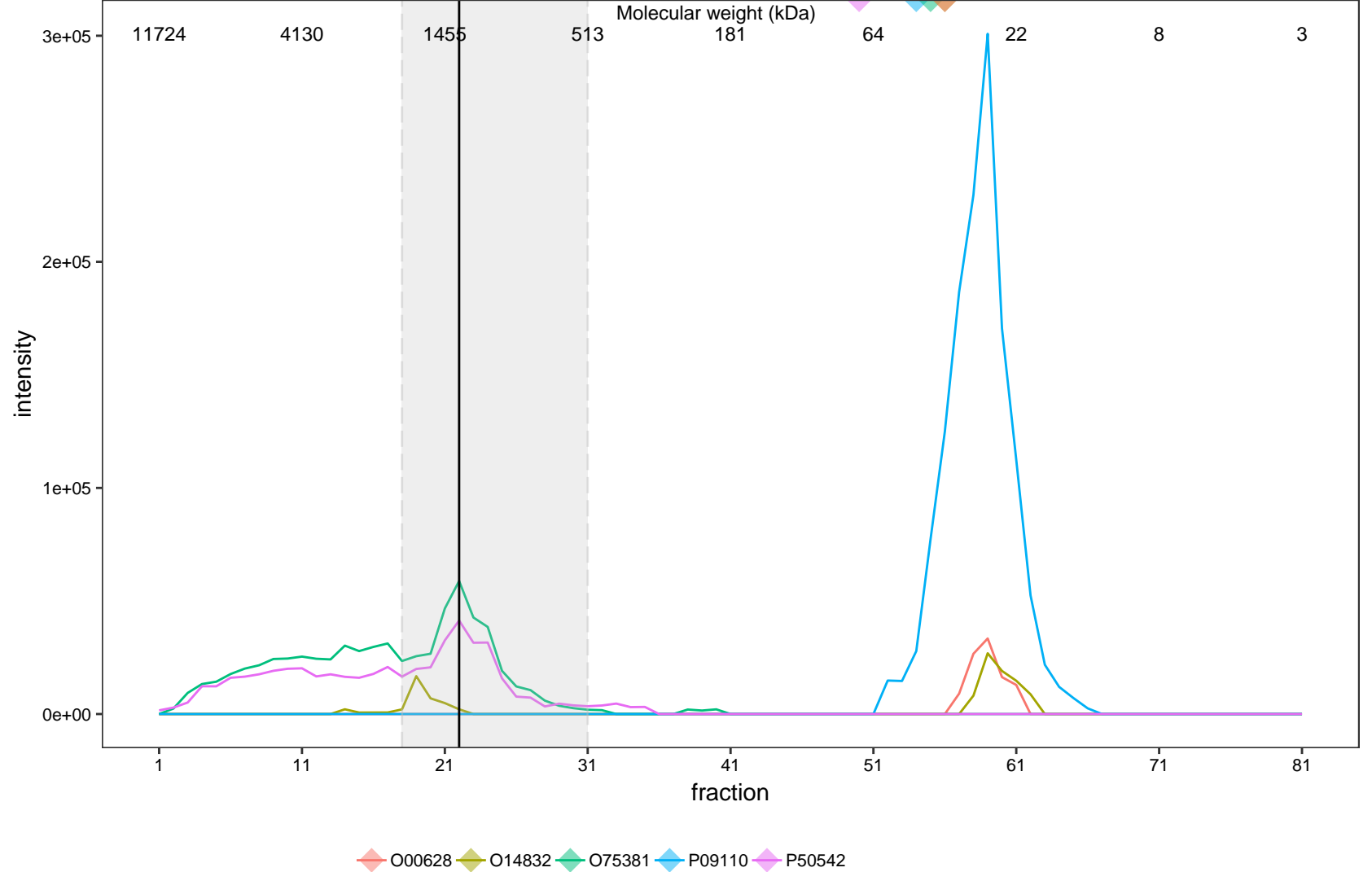

Supplement: Supplementary file 8 — Dataset EV7 [file MSB-15-e8438-s008.zip › feature_plots_string/O00628.pdf]

O00712

Annotated subunits: 17 Subunits with signal: 9

Max. coeluting subunits: 9 Max. completeness: 0.53

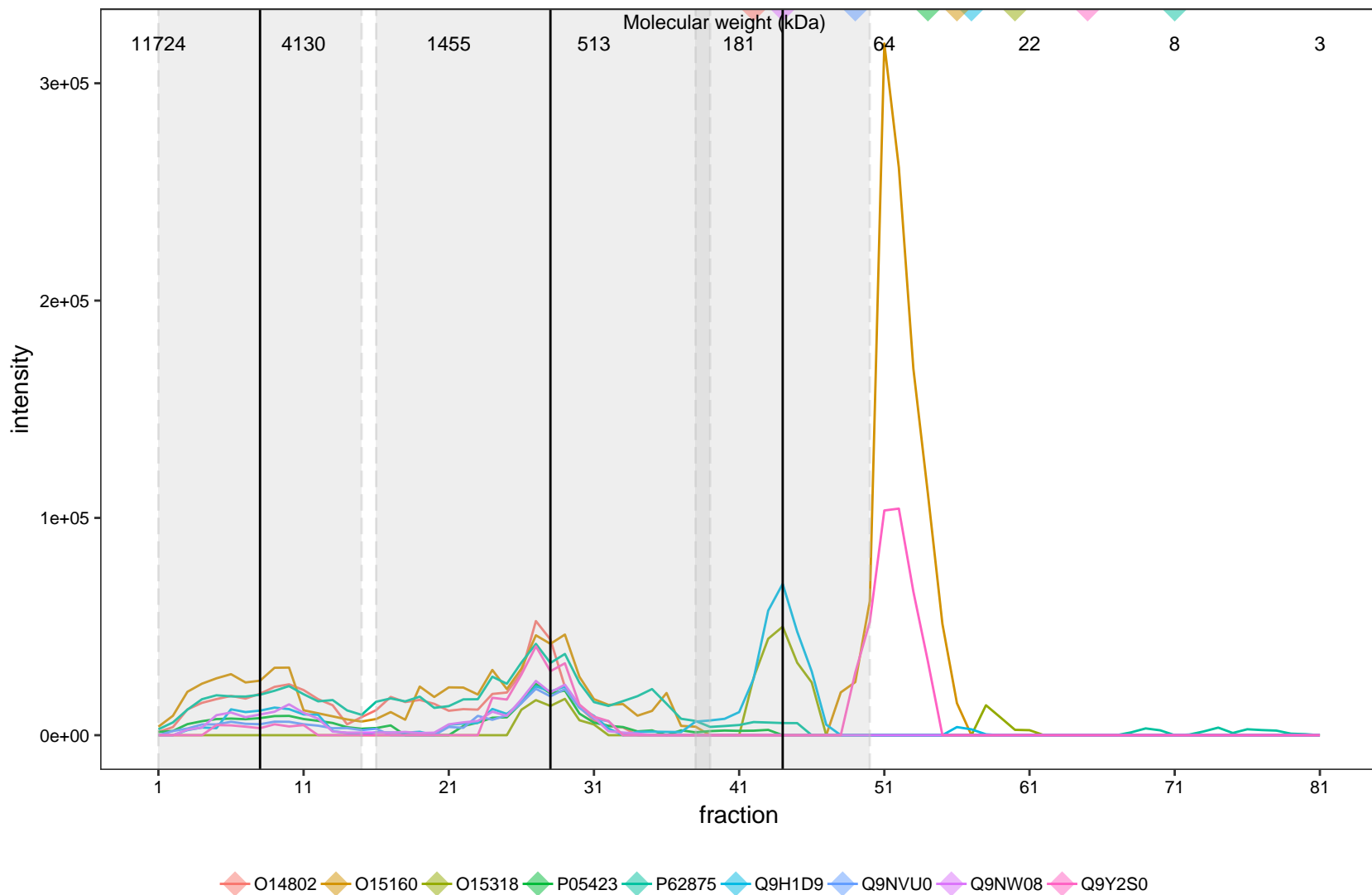

Supplement: Supplementary file 8 — Dataset EV7 [file MSB-15-e8438-s008.zip › feature_plots_string/O00712.pdf]

O00762

Annotated subunits: 115 Subunits with signal: 77

Max. coeluting subunits: 23 Max. completeness: 0.2

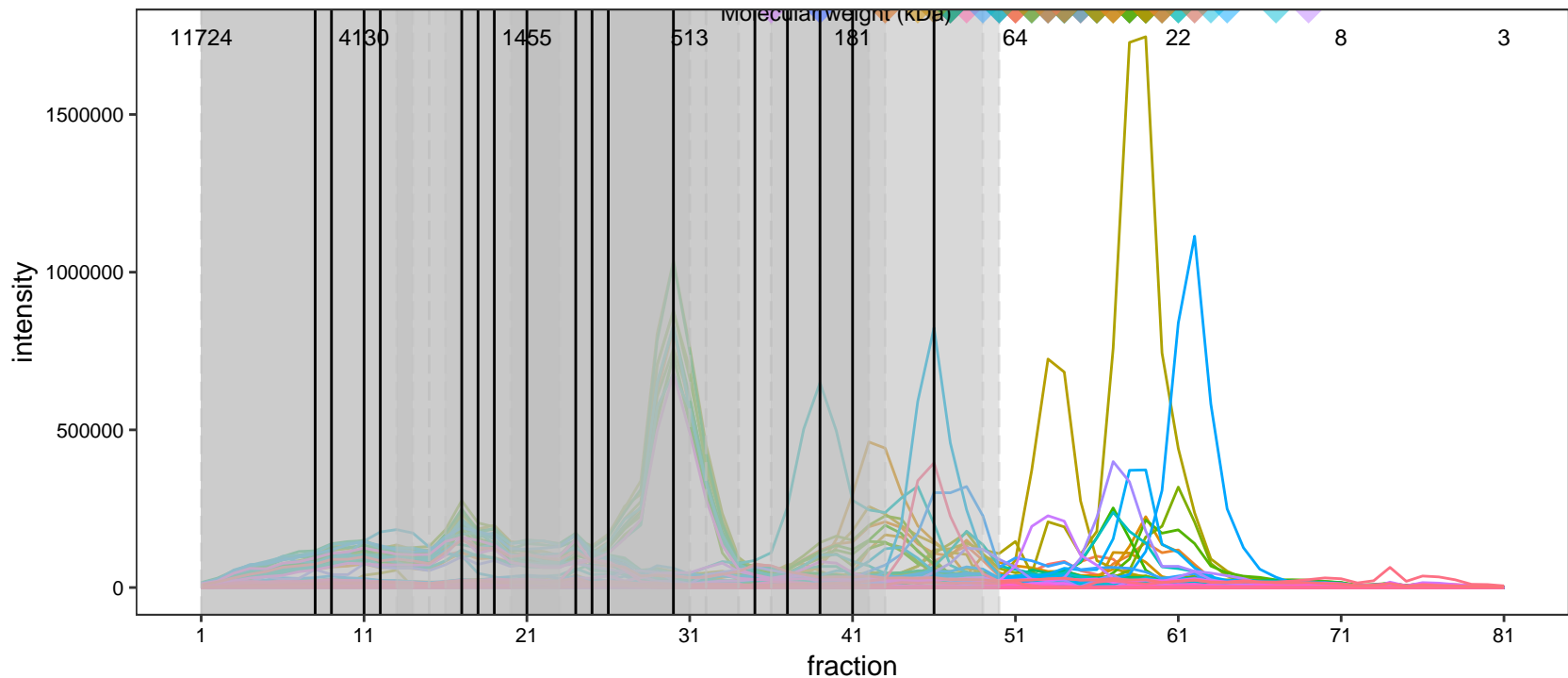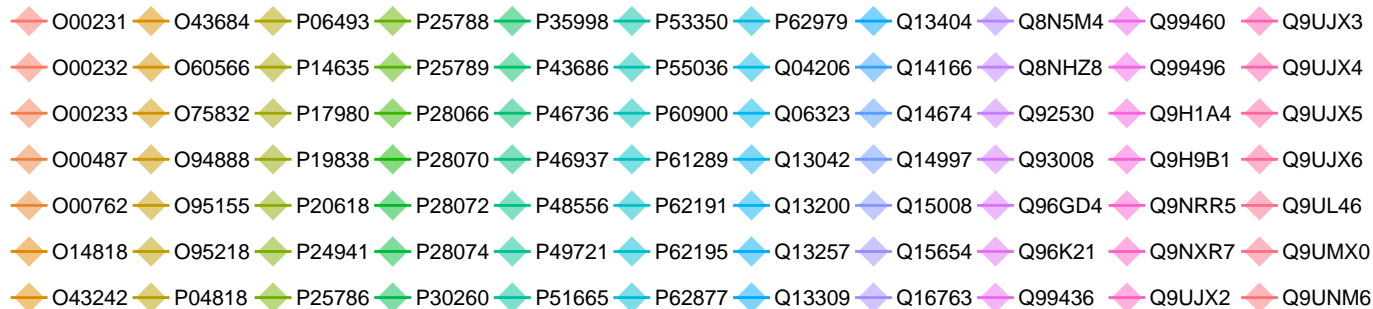

Supplement: Supplementary file 8 — Dataset EV7 [file MSB-15-e8438-s008.zip › feature_plots_string/O00762.pdf]

**O00767**  
**Annotated subunits: 11   Subunits with signal: 3**  
**Max. coeluting subunits: 3   Max. completeness: 0.27**

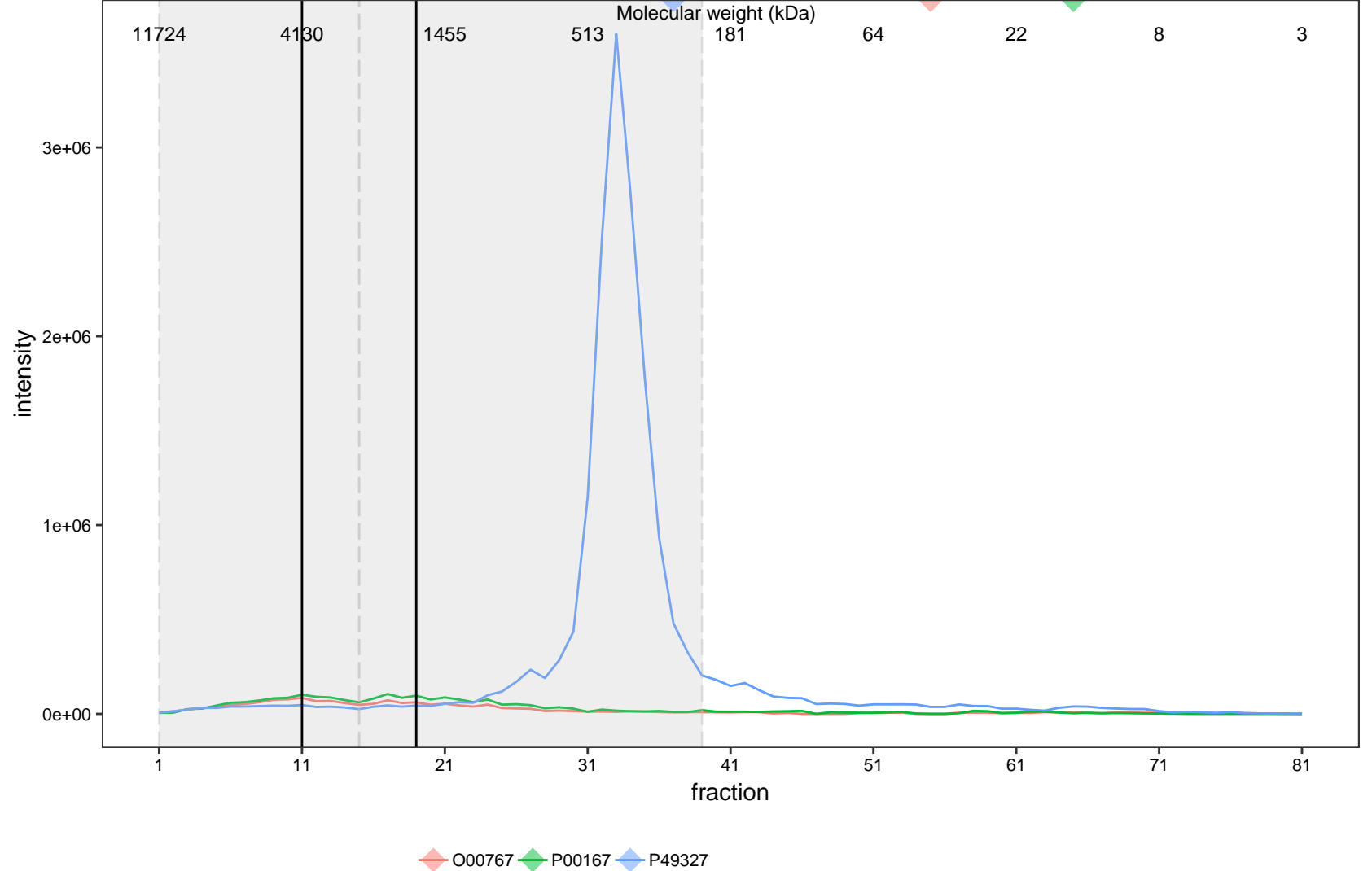

Supplement: Supplementary file 8 — Dataset EV7 [file MSB-15-e8438-s008.zip › feature_plots_string/O00767.pdf]

**O14494**

**Annotated subunits: 51 Subunits with signal: 12**

**Max. coeluting subunits: 6 Max. completeness: 0.12**

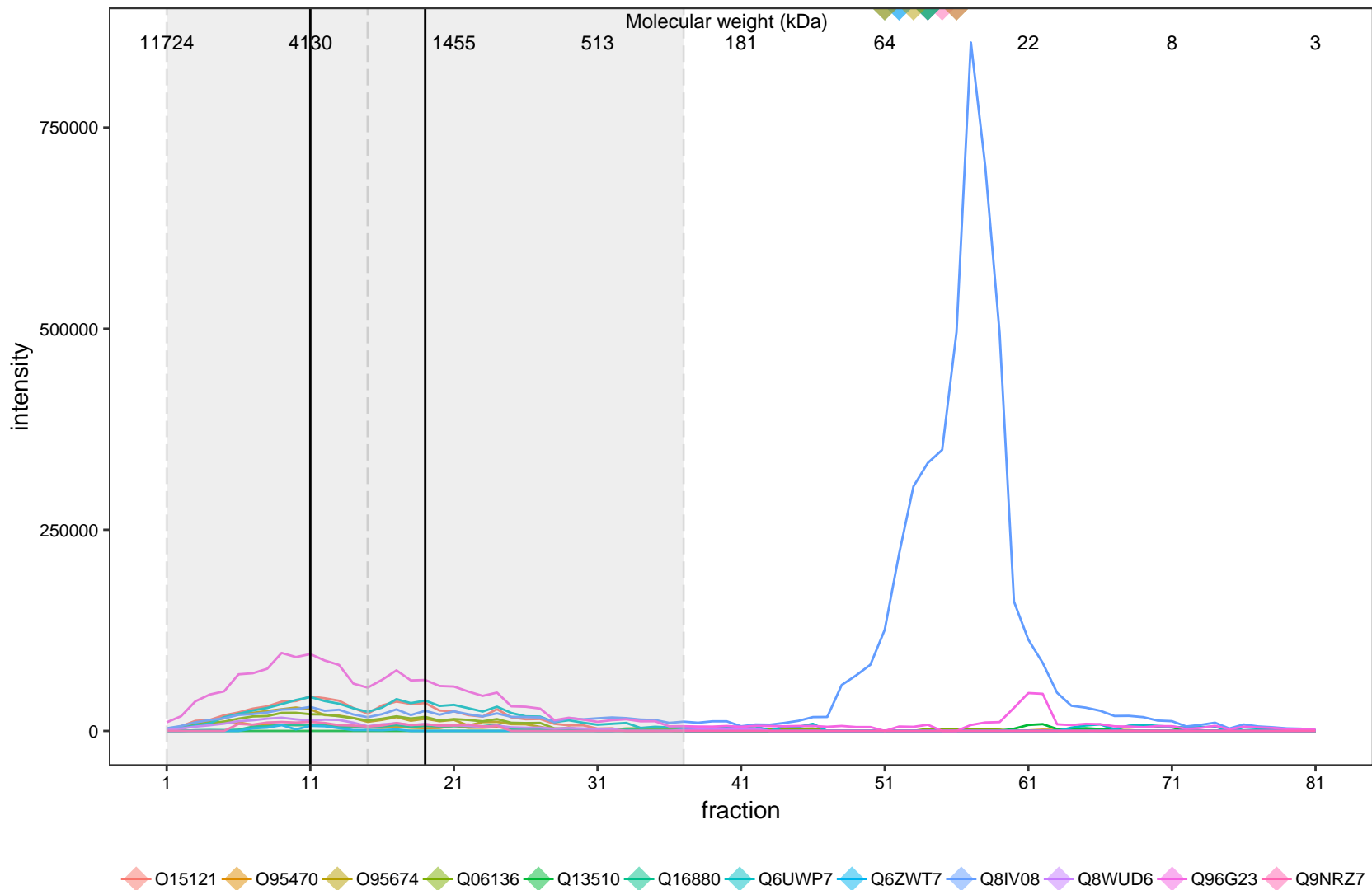

Supplement: Supplementary file 8 — Dataset EV7 [file MSB-15-e8438-s008.zip › feature_plots_string/O14494.pdf]

**O14497**

**Annotated subunits: 42 Subunits with signal: 23**

**Max. coeluting subunits: 9 Max. completeness: 0.21**

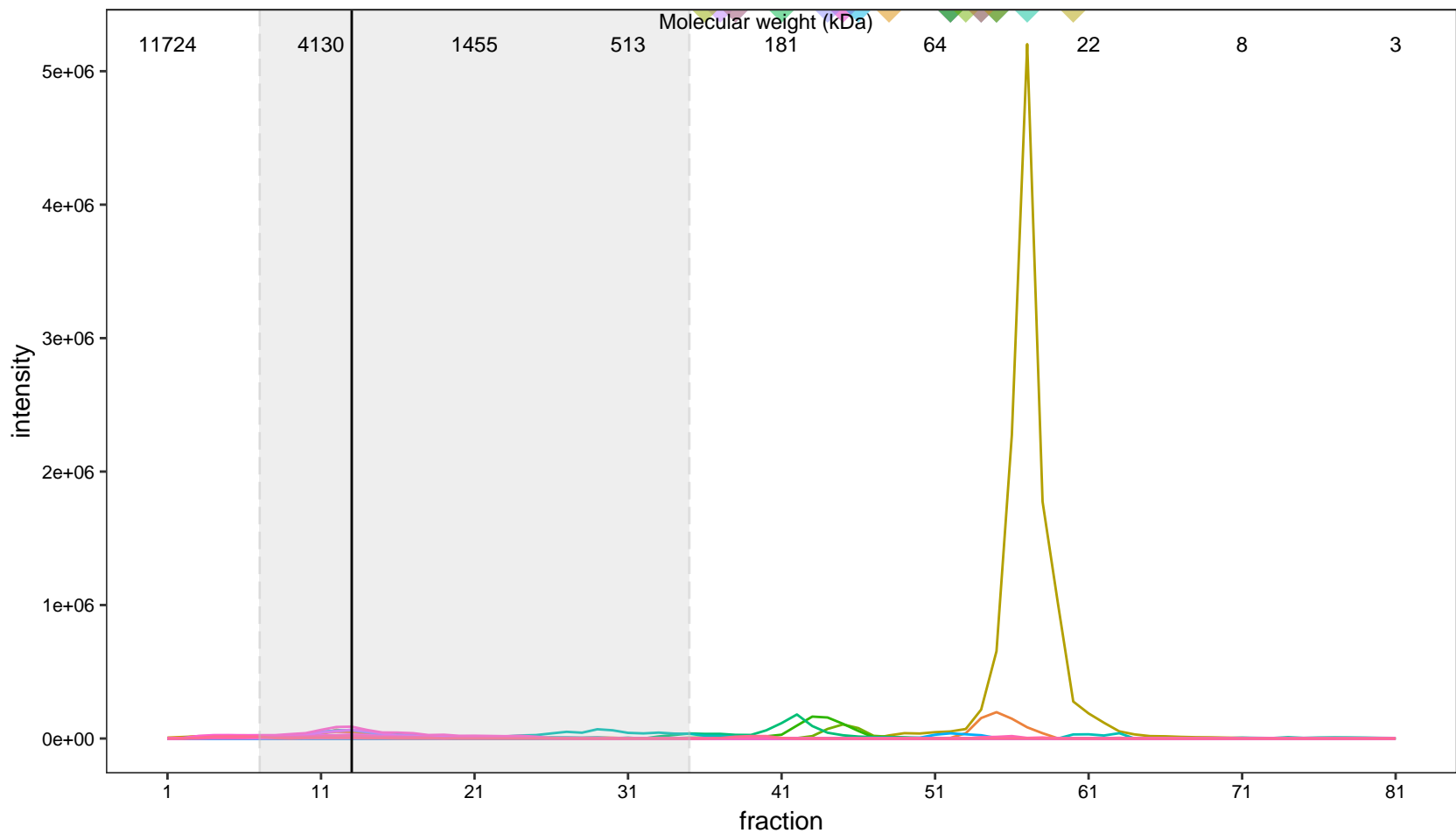

Supplement: Supplementary file 8 — Dataset EV7 [file MSB-15-e8438-s008.zip › feature_plots_string/O14497.pdf]

O14519  
Annotated subunits: 4   Subunits with signal: 4  
Max. coeluting subunits: 2   Max. completeness: 0.5

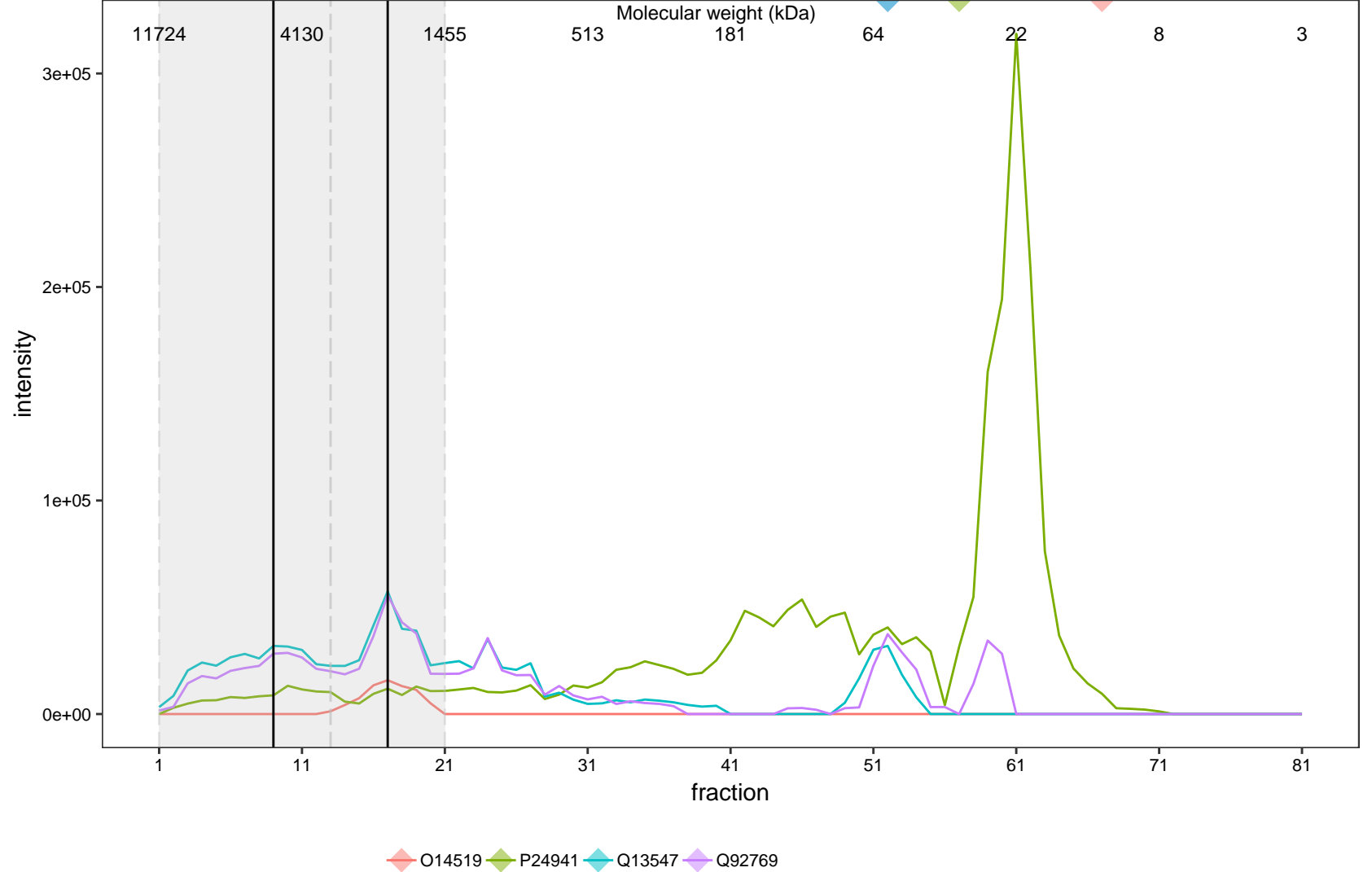

Supplement: Supplementary file 8 — Dataset EV7 [file MSB-15-e8438-s008.zip › feature_plots_string/O14519.pdf]

O14544  
Annotated subunits: 6   Subunits with signal: 3  
Max. coeluting subunits: 2   Max. completeness: 0.33

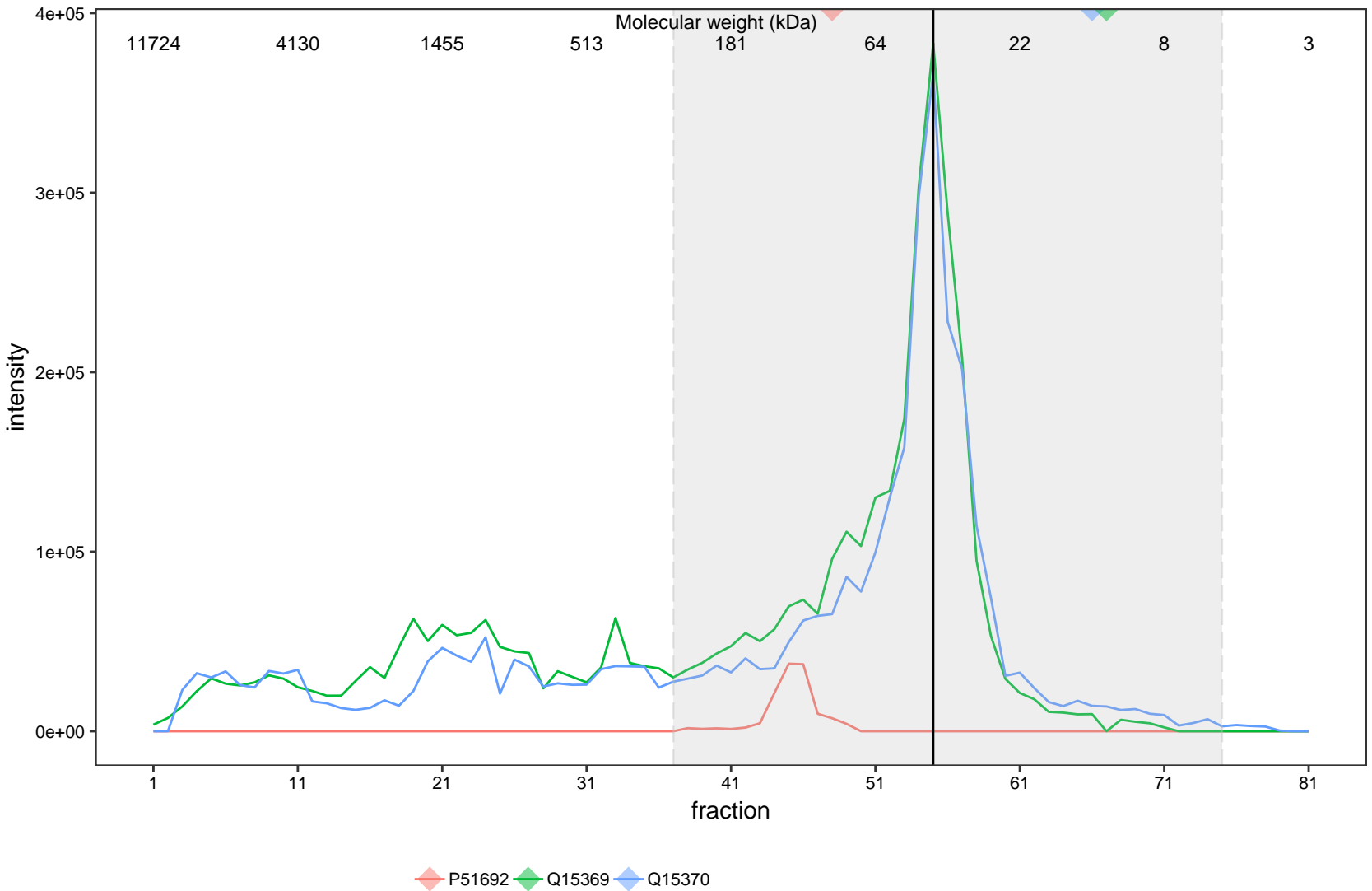

Supplement: Supplementary file 8 — Dataset EV7 [file MSB-15-e8438-s008.zip › feature_plots_string/O14544.pdf]

**O14562**

**Annotated subunits: 9 Subunits with signal: 8**

**Max. coeluting subunits: 5 Max. completeness: 0.56**

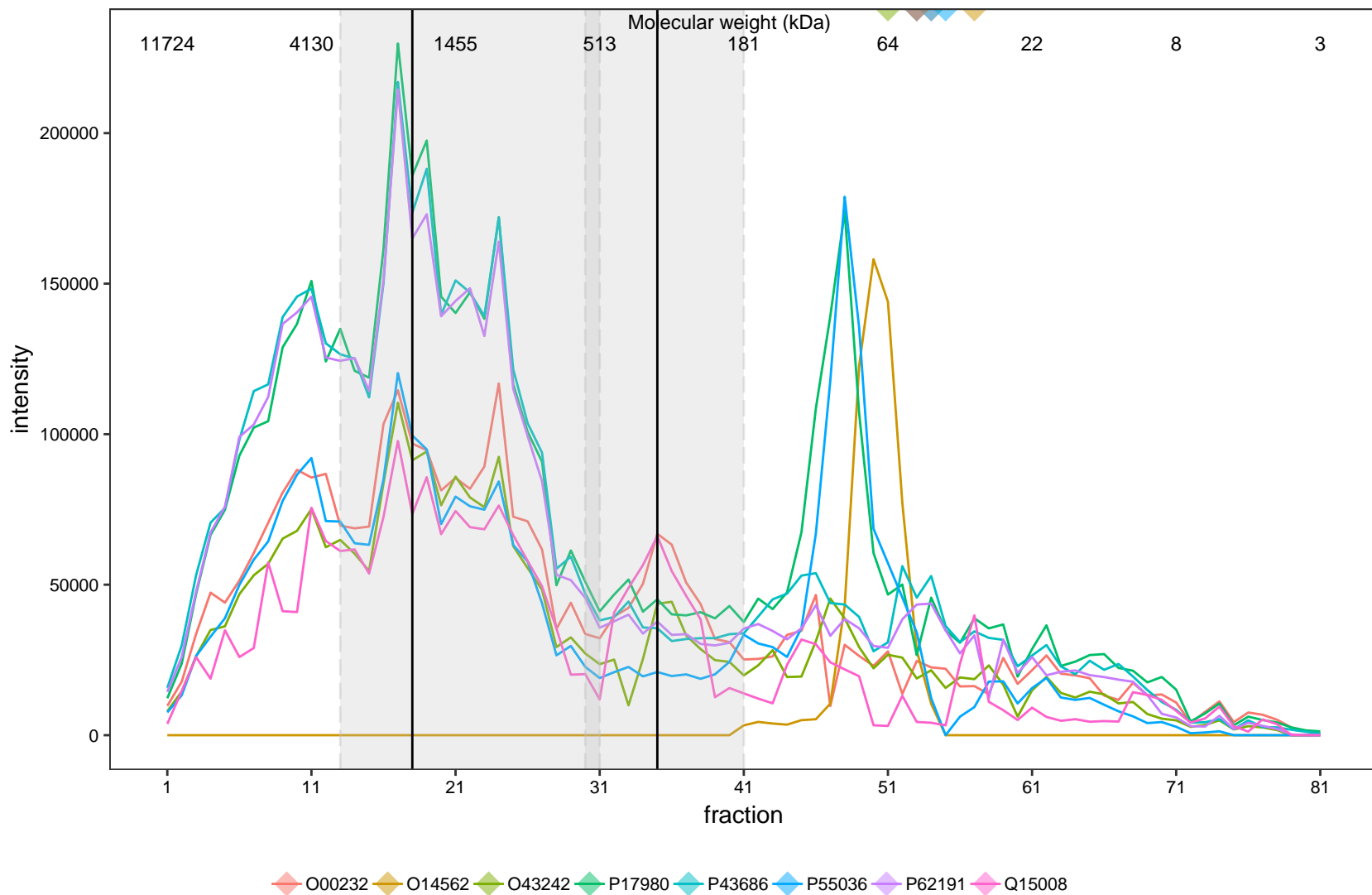

Supplement: Supplementary file 8 — Dataset EV7 [file MSB-15-e8438-s008.zip › feature_plots_string/O14562.pdf]

O14579

Annotated subunits: 13 Subunits with signal: 12

Max. coeluting subunits: 8 Max. completeness: 0.62

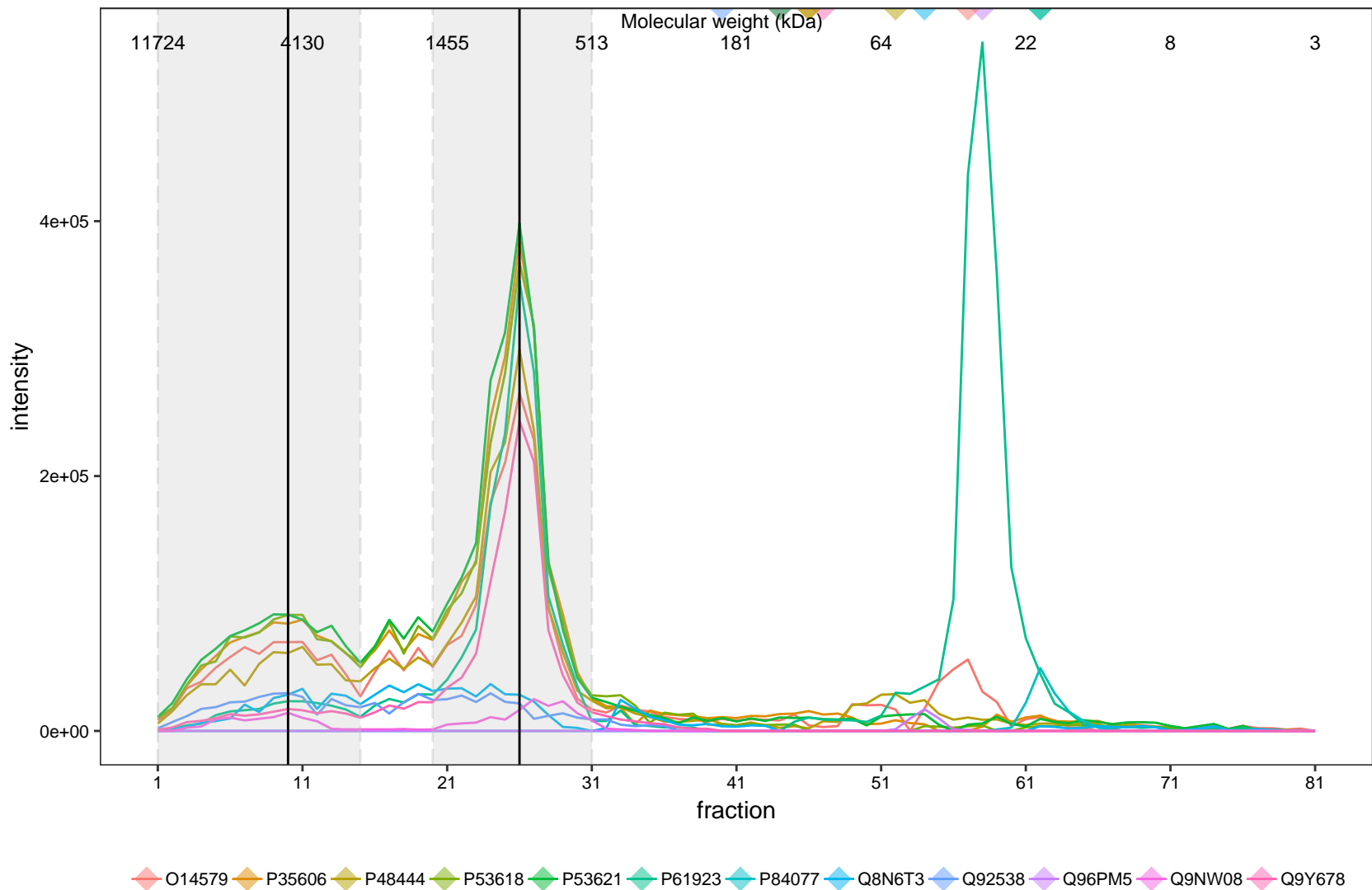

Supplement: Supplementary file 8 — Dataset EV7 [file MSB-15-e8438-s008.zip › feature_plots_string/O14579.pdf]

O14602  
Annotated subunits: 6   Subunits with signal: 5  
Max. coeluting subunits: 3   Max. completeness: 0.5

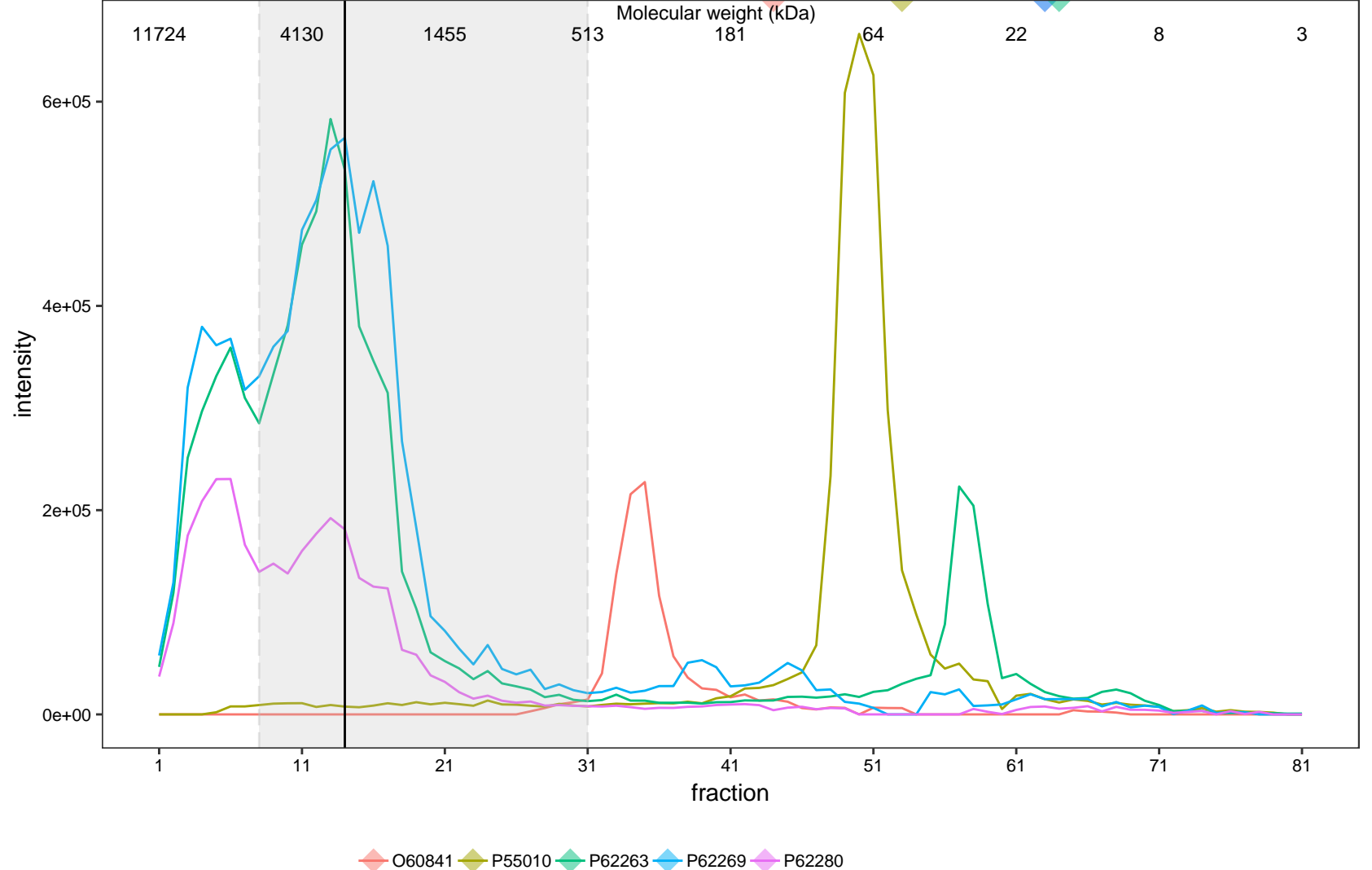

Supplement: Supplementary file 8 — Dataset EV7 [file MSB-15-e8438-s008.zip › feature_plots_string/O14602.pdf]

O14607  
Annotated subunits: 3   Subunits with signal: 2  
Max. coeluting subunits: 2   Max. completeness: 0.67

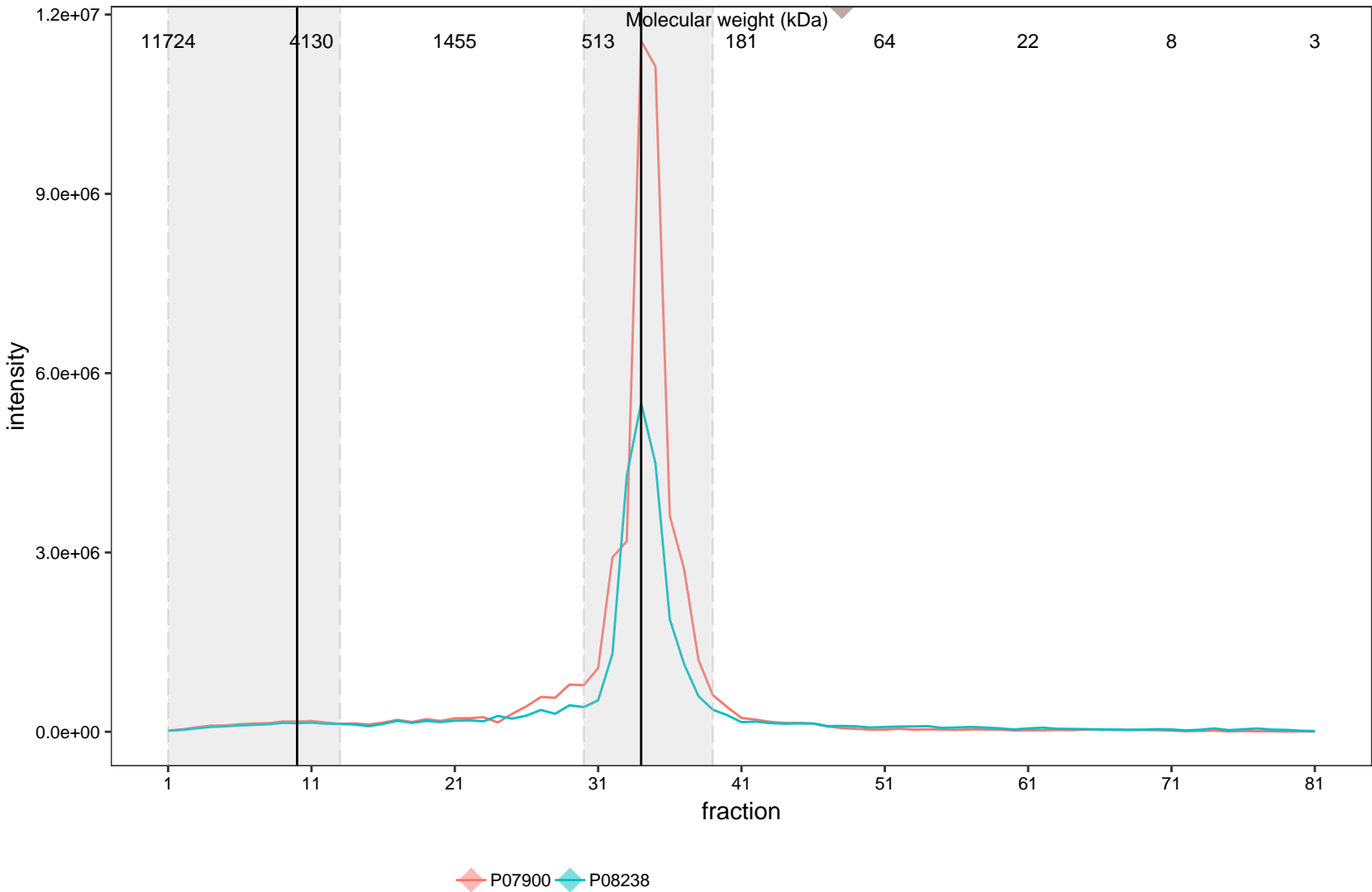

Supplement: Supplementary file 8 — Dataset EV7 [file MSB-15-e8438-s008.zip › feature_plots_string/O14607.pdf]

**O14617**

**Annotated subunits: 9 Subunits with signal: 6**

**Max. coeluting subunits: 3 Max. completeness: 0.33**

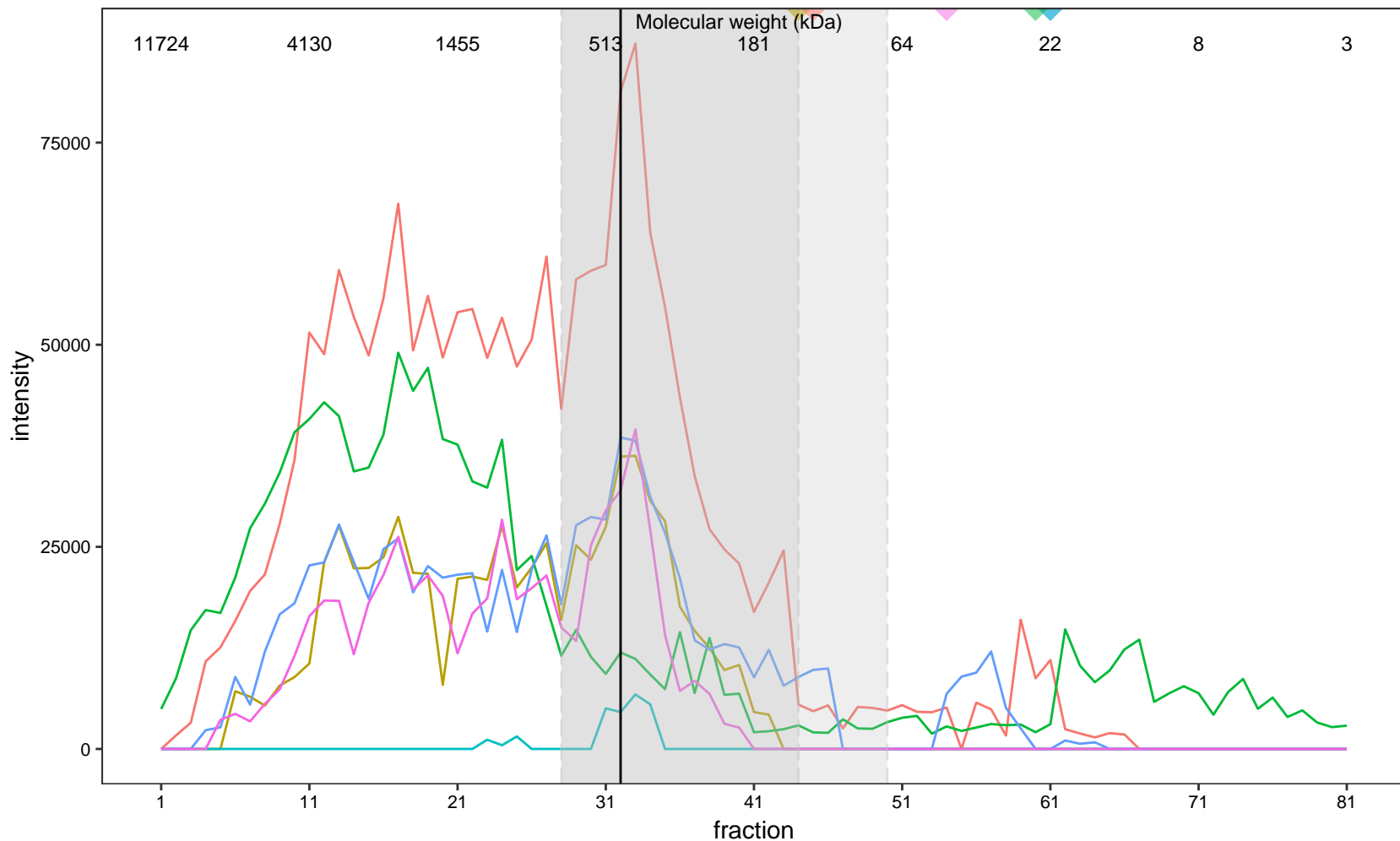

— O00203 — O14617 — P51809 — P59780 — Q92572 — Q9Y2T2

Supplement: Supplementary file 8 — Dataset EV7 [file MSB-15-e8438-s008.zip › feature_plots_string/O14617.pdf]

**O14653**

**Annotated subunits: 15 Subunits with signal: 13**

**Max. coeluting subunits: 8 Max. completeness: 0.53**

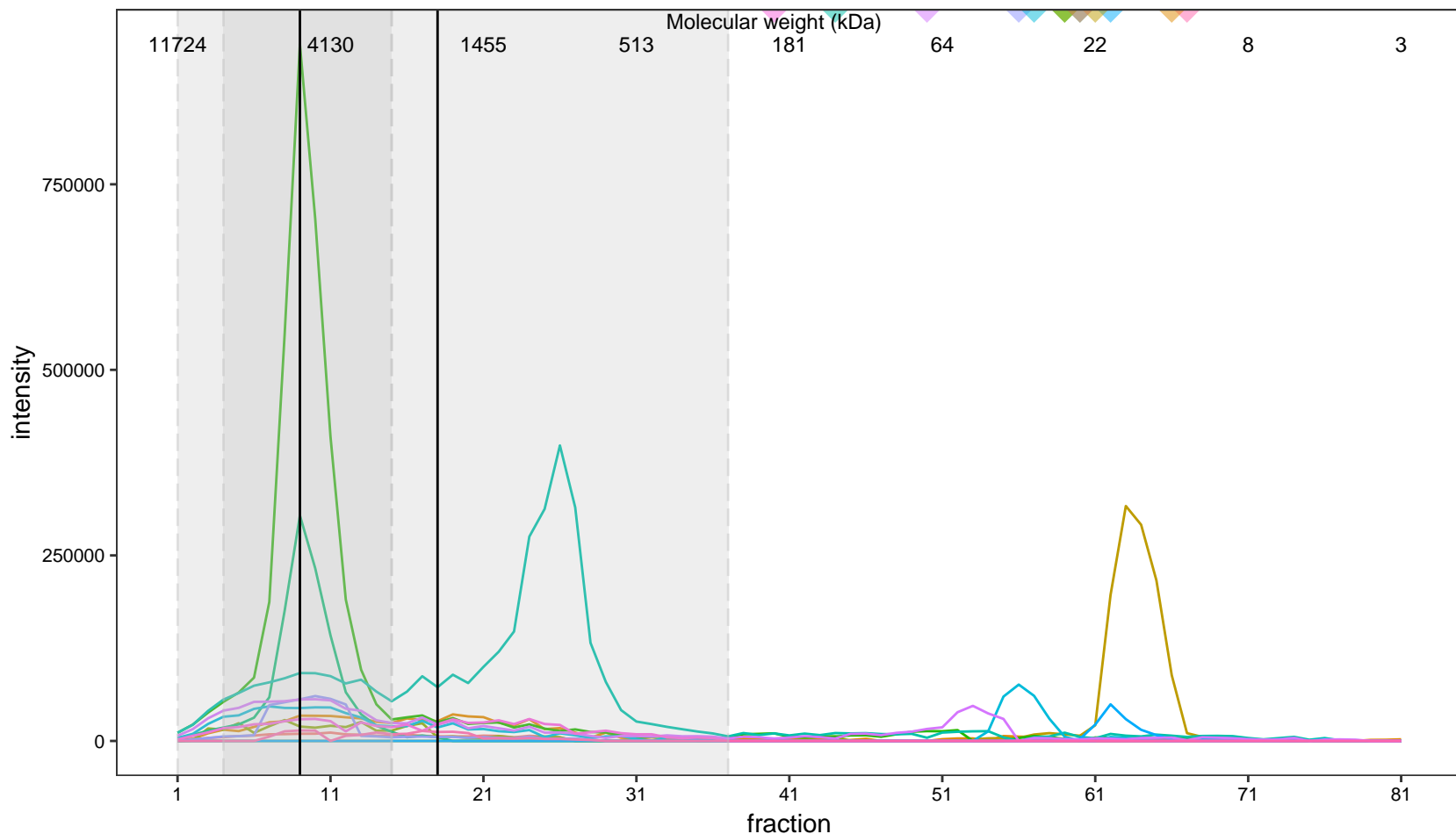

Supplement: Supplementary file 8 — Dataset EV7 [file MSB-15-e8438-s008.zip › feature_plots_string/O14653.pdf]

O14662

Annotated subunits: 25 Subunits with signal: 20

Max. coeluting subunits: 10 Max. completeness: 0.4

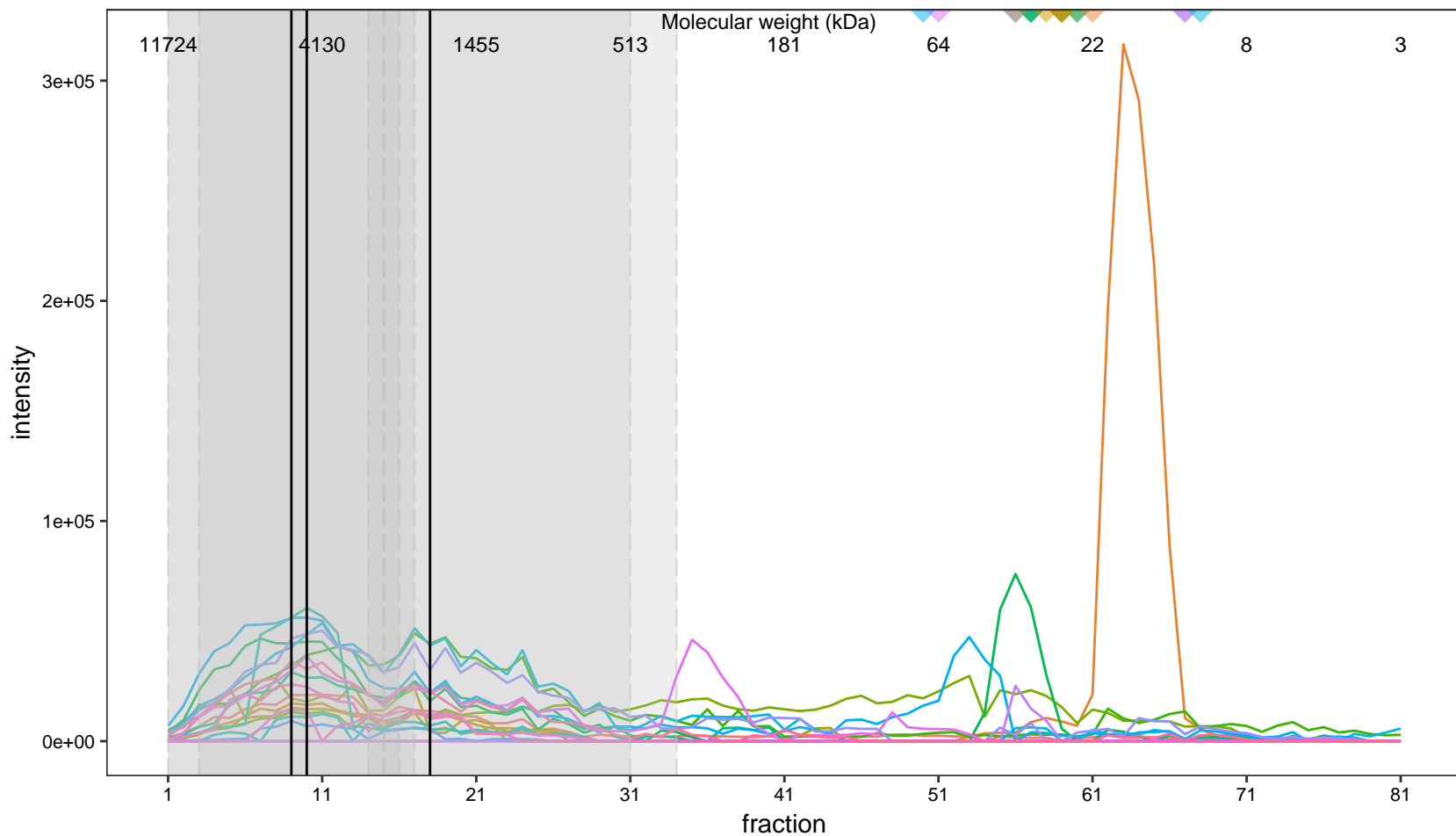

Supplement: Supplementary file 8 — Dataset EV7 [file MSB-15-e8438-s008.zip › feature_plots_string/O14662.pdf]

**Annotated subunits: 21   Subunits with signal: 10**  
**Max. coeluting subunits: 6   Max. completeness: 0.29**

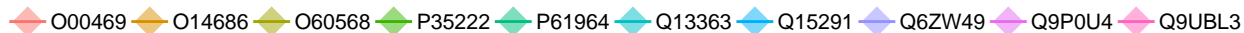

Supplement: Supplementary file 8 — Dataset EV7 [file MSB-15-e8438-s008.zip › feature_plots_string/O14686.pdf]

**Annotated subunits: 15   Subunits with signal: 10**  
**Max. coeluting subunits: 2   Max. completeness: 0.13**

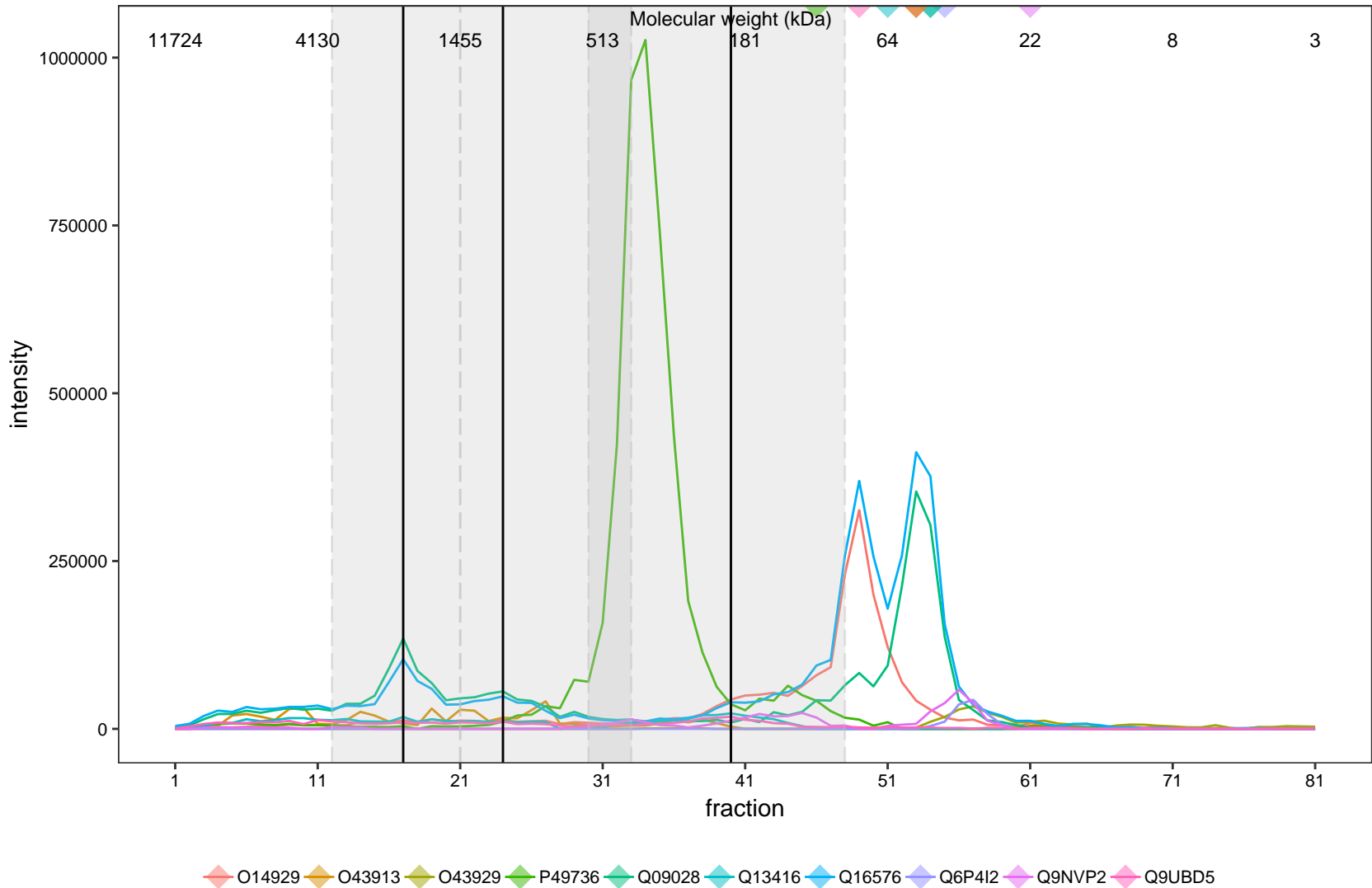

Supplement: Supplementary file 8 — Dataset EV7 [file MSB-15-e8438-s008.zip › feature_plots_string/O14929.pdf]
