# Supplementary material for: Complex‐centric proteome profiling by SEC‐SWATH‐MS
Source: Mol Syst Biol. 2019 Jan 14;15(1):e8438. doi: 10.15252/msb.20188438 (PMC6346213; doi:10.15252/msb.20188438)
Supplement: Supplementary file 8 — Dataset EV7 [file MSB-15-e8438-s008.zip › feature_plots_string/A5LHX3.pdf]

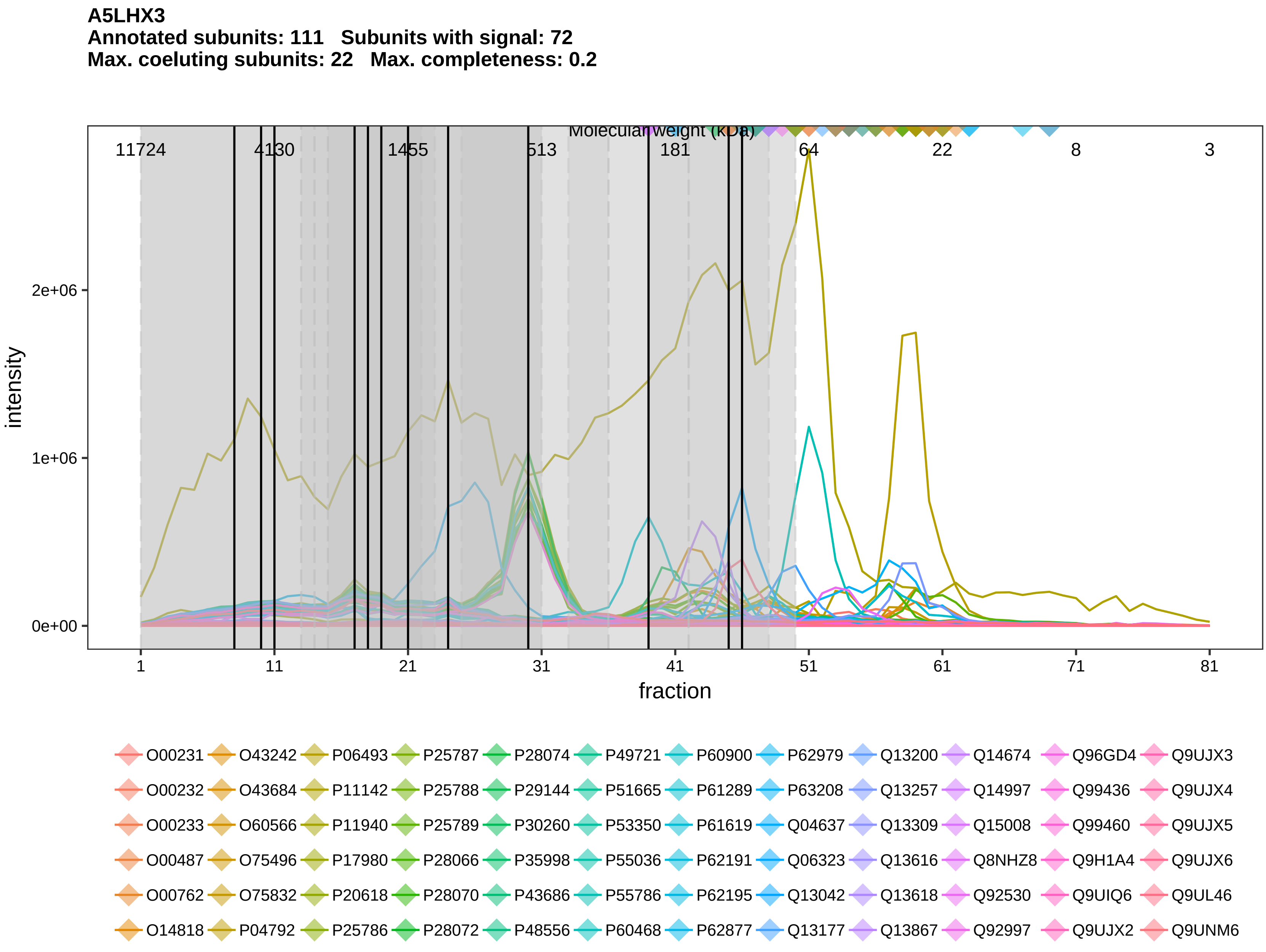
A5LHX3
Annotated subunits: 111 Subunits with signal: 72
Max. coeluting subunits: 22 Max. completeness: 0.2
Molecular weight (kDa)
11724
4130
1455
513
181
64
22
8
3
2e+06
1e+06
0e+00
intensity
1
11
21
31
41
51
61
71
81
fraction
O00231 O43242 P06493 P25787 P28074 P49721 P60900 P62979 Q13200 Q14674 Q96GD4 Q9UJX3
O00232 O43684 P11142 P25788 P29144 P51665 P61289 P63208 Q13257 Q14997 Q99436 Q9UJX4
O00233 O60566 P11940 P25789 P30260 P53350 P61619 Q04637 Q13309 Q15008 Q99460 Q9UJX5
O00487 O75496 P17980 P28066 P35998 P55036 P62191 Q06323 Q13616 Q8NHZ8 Q9H1A4 Q9UJX6
O00762 O75832 P20618 P28070 P43686 P55786 P62195 Q13042 Q13618 Q92530 Q9UIQ6 Q9UL46
O14818 P04792 P25786 P28072 P48556 P60468 P62877 Q13177 Q13867 Q92997 Q9UJX2 Q9UNM6
