# Supplementary material for: Complex‐centric proteome profiling by SEC‐SWATH‐MS
Source: Mol Syst Biol. 2019 Jan 14;15(1):e8438. doi: 10.15252/msb.20188438 (PMC6346213; doi:10.15252/msb.20188438)
Supplement: Supplementary file 8 — Dataset EV7 [file MSB-15-e8438-s008.zip › feature_plots_string/E9PQ53.pdf]

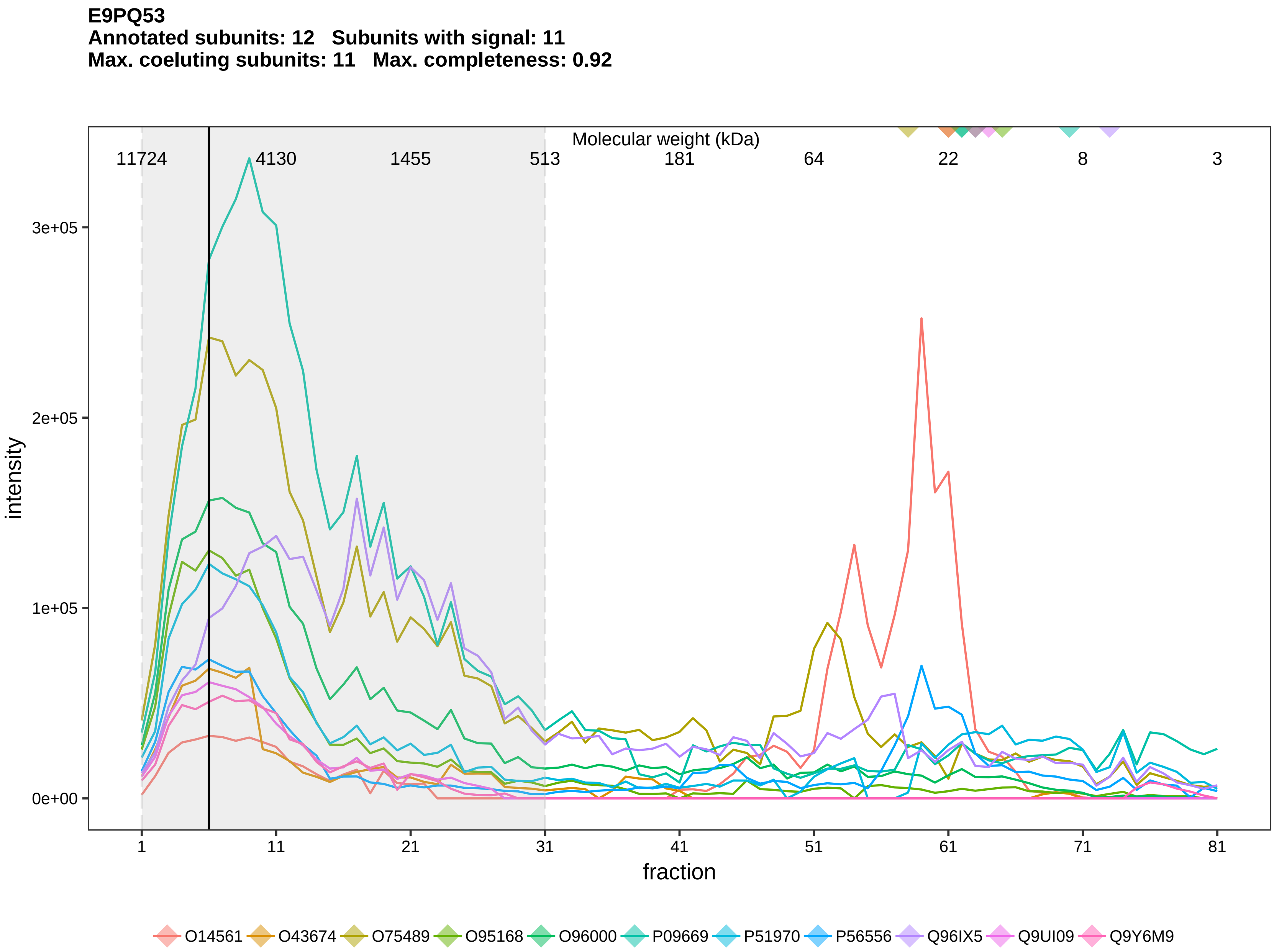
E9PQ53
Annotated subunits: 12 Subunits with signal: 11
Max. coeluting subunits: 11 Max. completeness: 0.92
Molecular weight (kDa)
11724
4130
1455
513
181
64
22
8
3
intensity
3e+05
2e+05
1e+05
0e+00
fraction
1
11
21
31
41
51
61
71
81
O14561
O43674
O75489
O95168
O96000
P09669
P51970
P56556
Q96IX5
Q9UI09
Q9Y6M9
