# Supplementary material for: Complex‐centric proteome profiling by SEC‐SWATH‐MS
Source: Mol Syst Biol. 2019 Jan 14;15(1):e8438. doi: 10.15252/msb.20188438 (PMC6346213; doi:10.15252/msb.20188438)
Supplement: Supplementary file 8 — Dataset EV7 [file MSB-15-e8438-s008.zip › feature_plots_string/O00231.pdf]

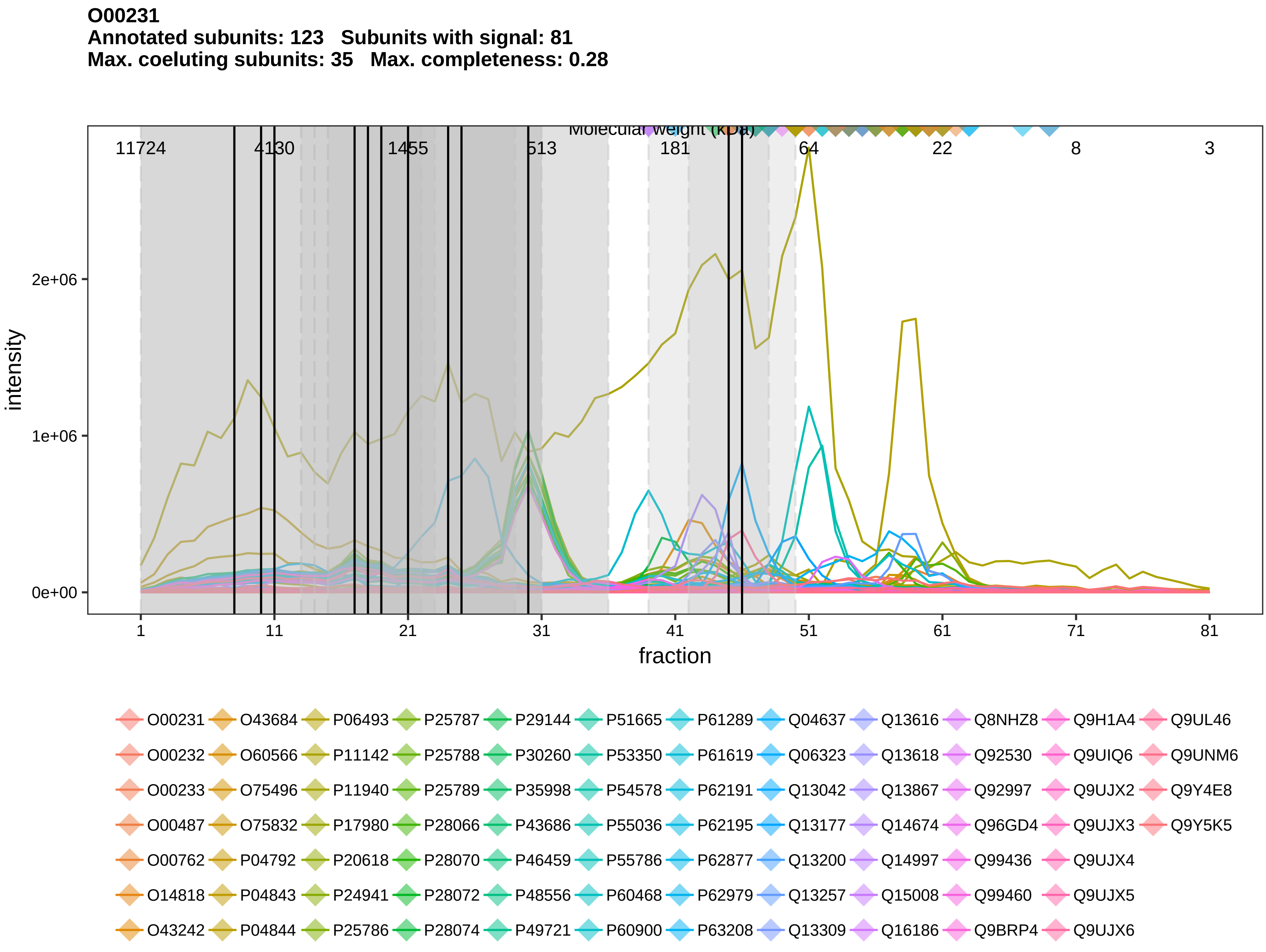
O00231
Annotated subunits: 123 Subunits with signal: 81
Max. coeluting subunits: 35 Max. completeness: 0.28
Molecular weight (kDa)
11724
4130
1455
513
181
64
22
8
3
intensity
2e+06
1e+06
0e+00
fraction
1
11
21
31
41
51
61
71
81
O00231
O00232
O00233
O00487
O00762
O14818
O43242
O43684
O60566
O75496
O75832
P04792
P04843
P04844
P06493
P11142
P11940
P17980
P20618
P24941
P25786
P25787
P25788
P25789
P28066
P28070
P28072
P28074
P29144
P30260
P35998
P43686
P46459
P48556
P49721
P51665
P53350
P54578
P55036
P55786
P60468
P60900
P61289
P61619
P62191
P62195
P62877
P62979
P63208
Q04637
Q06323
Q13042
Q13177
Q13200
Q13257
Q13309
Q13616
Q13618
Q13867
Q14674
Q14997
Q15008
Q16186
Q8NHZ8
Q92530
Q92997
Q96GD4
Q99436
Q99460
Q9BRP4
Q9H1A4
Q9UIQ6
Q9UJX2
Q9UJX3
Q9UJX4
Q9UJX5
Q9UJX6
Q9UL46
Q9UNM6
Q9Y4E8
Q9Y5K5
