# Supplementary material for: Complex‐centric proteome profiling by SEC‐SWATH‐MS
Source: Mol Syst Biol. 2019 Jan 14;15(1):e8438. doi: 10.15252/msb.20188438 (PMC6346213; doi:10.15252/msb.20188438)
Supplement: Supplementary file 8 — Dataset EV7 [file MSB-15-e8438-s008.zip › feature_plots_string/O00257.pdf]

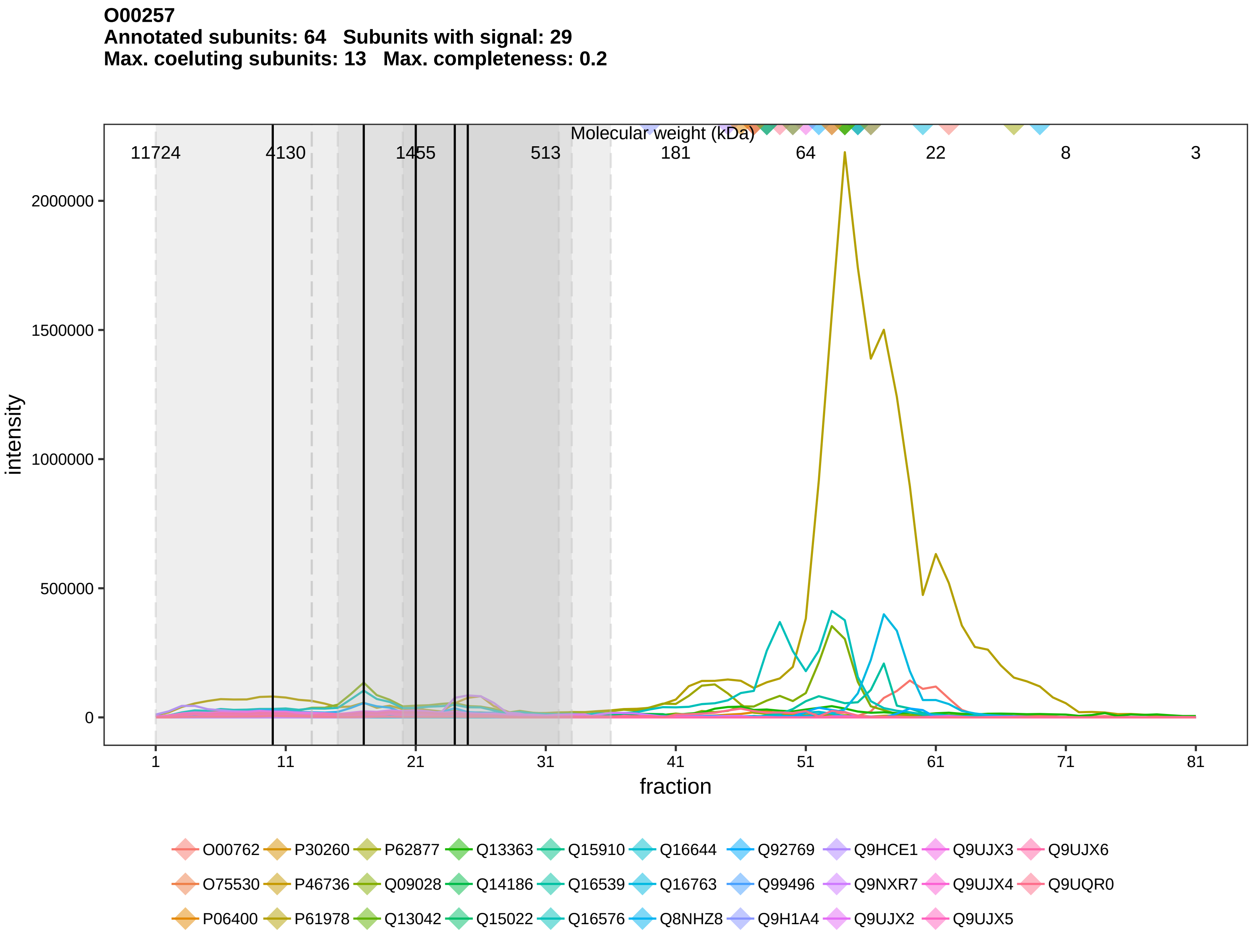
O00257
Annotated subunits: 64 Subunits with signal: 29
Max. coeluting subunits: 13 Max. completeness: 0.2
Molecular weight (kDa)
11724
4130
1455
513
181
64
22
8
3
intensity
2000000
1500000
1000000
500000
0
fraction
1
11
21
31
41
51
61
71
81
O00762
P30260
P62877
Q13363
Q15910
Q16644
Q92769
Q9HCE1
Q9UJX3
Q9UJX6
O75530
P46736
Q09028
Q14186
Q16539
Q16763
Q99496
Q9NXR7
Q9UJX4
Q9UQR0
P06400
P61978
Q13042
Q15022
Q16576
Q8NHZ8
Q9H1A4
Q9UJX2
Q9UJX5
