# Supplementary material for: Complex‐centric proteome profiling by SEC‐SWATH‐MS
Source: Mol Syst Biol. 2019 Jan 14;15(1):e8438. doi: 10.15252/msb.20188438 (PMC6346213; doi:10.15252/msb.20188438)
Supplement: Supplementary file 8 — Dataset EV7 [file MSB-15-e8438-s008.zip › feature_plots_string/O00303.pdf]

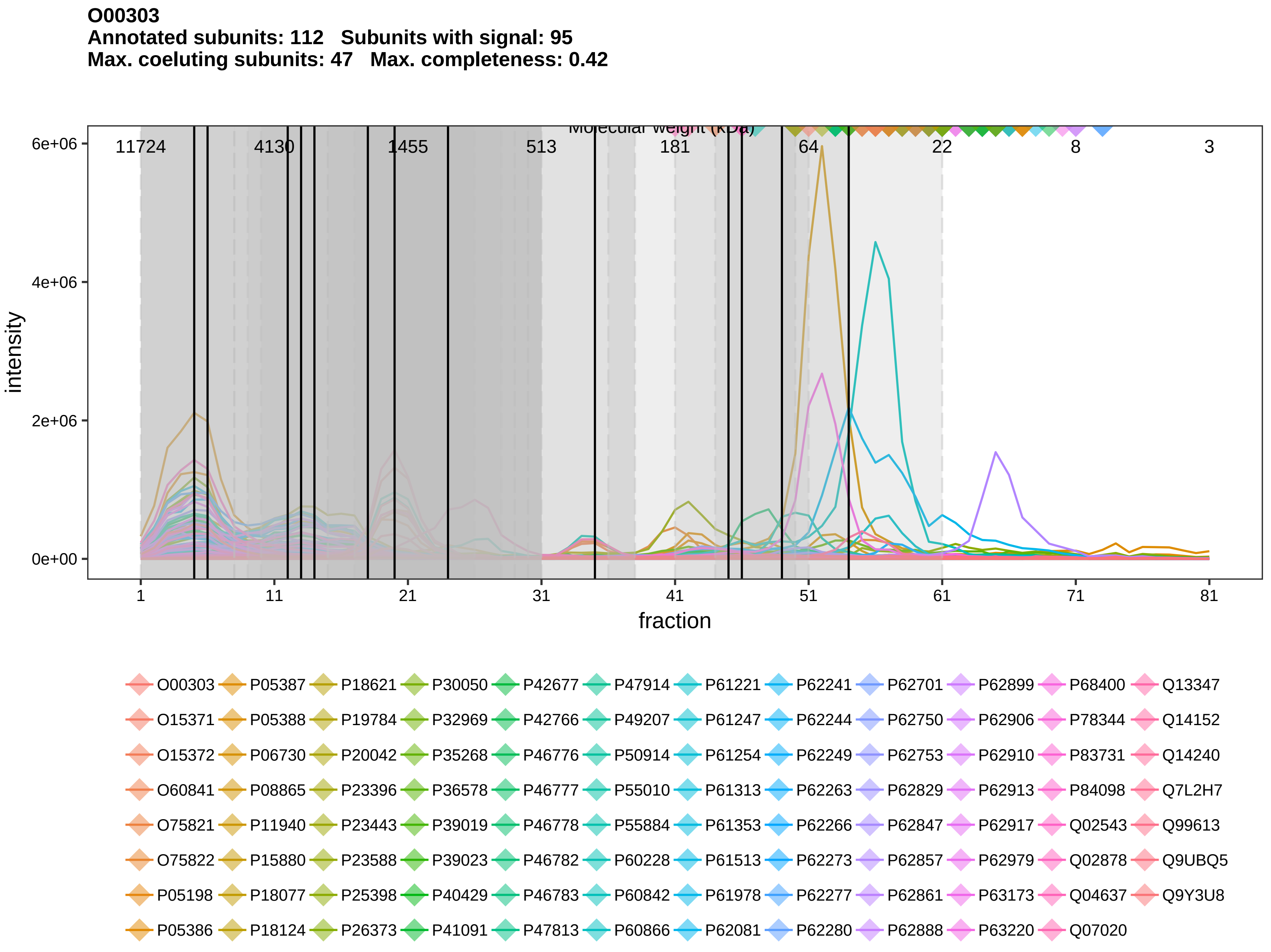
O00303
Annotated subunits: 112 Subunits with signal: 95
Max. coeluting subunits: 47 Max. completeness: 0.42
Molecular weight (kDa)
11724
4130
1455
513
181
64
22
8
3
intensity
6e+06
4e+06
2e+06
0e+00
fraction
1
11
21
31
41
51
61
71
81
O00303
P05387
P18621
P30050
P42677
P47914
P61221
P62241
P62701
P62899
P68400
Q13347
O15371
P05388
P19784
P32969
P42766
P49207
P61247
P62244
P62750
P62906
P78344
Q14152
O15372
P06730
P20042
P35268
P46776
P50914
P61254
P62249
P62753
P62910
P83731
Q14240
O60841
P08865
P23396
P36578
P46777
P55010
P61313
P62263
P62829
P62913
P84098
Q7L2H7
O75821
P11940
P23443
P39019
P46778
P55884
P61353
P62266
P62847
P62917
Q02543
Q99613
O75822
P15880
P23588
P39023
P46782
P60228
P61513
P62273
P62857
P62979
Q02878
Q9UBQ5
P05198
P18077
P25398
P40429
P46783
P60842
P61978
P62277
P62861
P63173
Q04637
Q9Y3U8
P05386
P18124
P26373
P41091
P47813
P60866
P62081
P62280
P62888
P63220
Q07020
