# Supplementary material for: Complex‐centric proteome profiling by SEC‐SWATH‐MS
Source: Mol Syst Biol. 2019 Jan 14;15(1):e8438. doi: 10.15252/msb.20188438 (PMC6346213; doi:10.15252/msb.20188438)
Supplement: Supplementary file 8 — Dataset EV7 [file MSB-15-e8438-s008.zip › feature_plots_string/O14521.pdf]

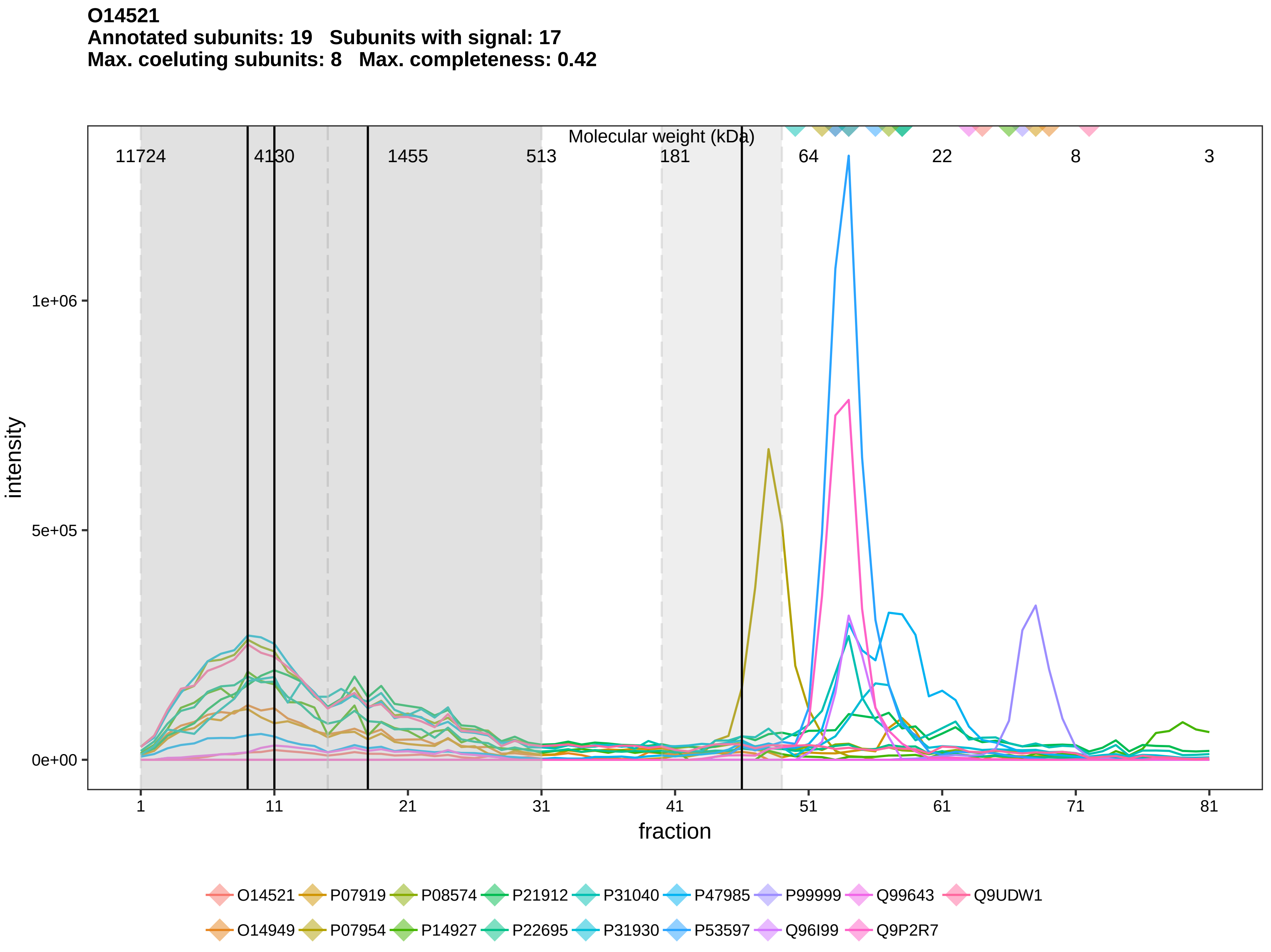
O14521
Annotated subunits: 19 Subunits with signal: 17
Max. coeluting subunits: 8 Max. completeness: 0.42
Molecular weight (kDa)
11724
4130
1455
513
181
64
22
8
3
intensity
1e+06
5e+05
0e+00
fraction
1
11
21
31
41
51
61
71
81
O14521
P07919
P08574
P21912
P31040
P47985
P99999
Q99643
Q9UDW1
O14949
P07954
P14927
P22695
P31930
P53597
Q96I99
Q9P2R7
