# Supplementary material for: Complex‐centric proteome profiling by SEC‐SWATH‐MS
Source: Mol Syst Biol. 2019 Jan 14;15(1):e8438. doi: 10.15252/msb.20188438 (PMC6346213; doi:10.15252/msb.20188438)
Supplement: Supplementary file 8 — Dataset EV7 [file MSB-15-e8438-s008.zip › feature_plots_string/O14672.pdf]

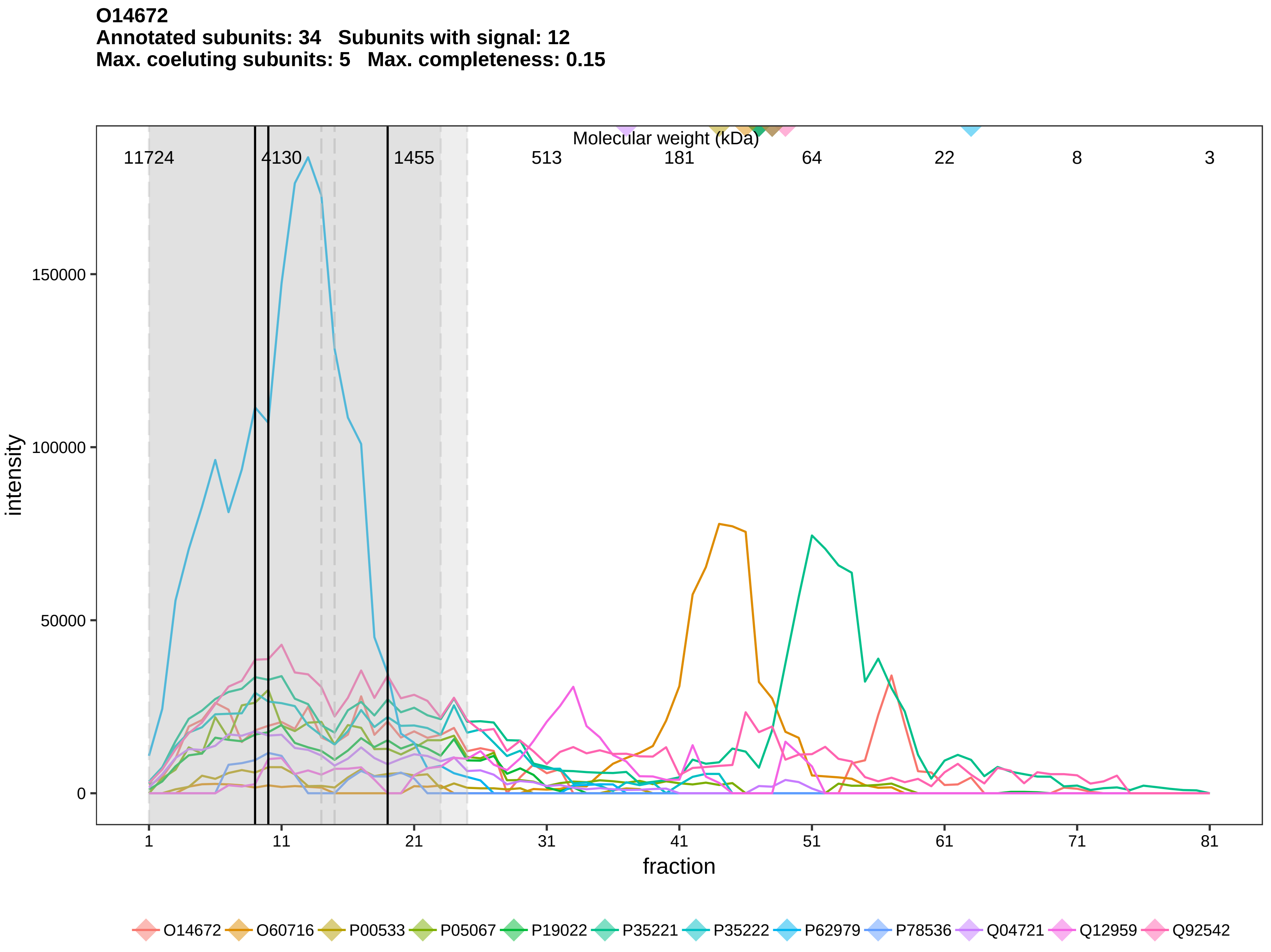
O14672
Annotated subunits: 34 Subunits with signal: 12
Max. coeluting subunits: 5 Max. completeness: 0.15
Molecular weight (kDa)
11724
4130
1455
513
181
64
22
8
3
intensity
150000
100000
50000
0
1
11
21
31
41
51
61
71
81
fraction
O14672
O60716
P00533
P05067
P19022
P35221
P35222
P62979
P78536
Q04721
Q12959
Q92542
