# Supplementary material for: Complex‐centric proteome profiling by SEC‐SWATH‐MS
Source: Mol Syst Biol. 2019 Jan 14;15(1):e8438. doi: 10.15252/msb.20188438 (PMC6346213; doi:10.15252/msb.20188438)
Supplement: Supplementary file 8 — Dataset EV7 [file MSB-15-e8438-s008.zip › feature_plots_string/O15169.pdf]

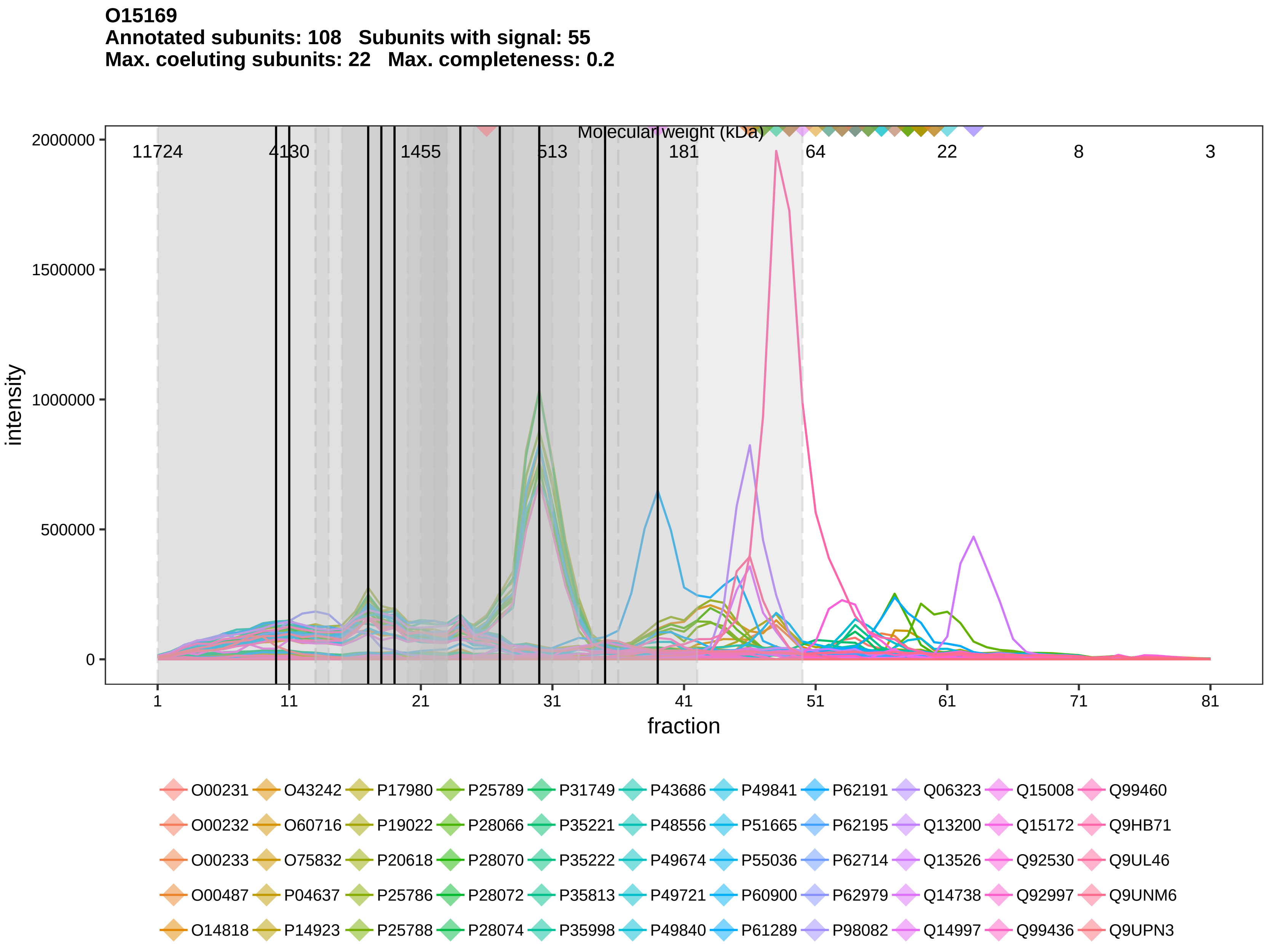
O15169
Annotated subunits: 108 Subunits with signal: 55
Max. coeluting subunits: 22 Max. completeness: 0.2
Molecular weight (kDa)
11724
4130
1455
513
181
64
22
8
3
intensity
2000000
1500000
1000000
500000
0
fraction
1
11
21
31
41
51
61
71
81
O00231
O00232
O00233
O00487
O14818
O43242
O60716
O75832
P04637
P14923
P17980
P19022
P20618
P25786
P25788
P25789
P28066
P28070
P28072
P28074
P31749
P35221
P35222
P35813
P35998
P43686
P48556
P49674
P49721
P49840
P49841
P51665
P55036
P60900
P61289
P62191
P62195
P62714
P62979
P98082
Q06323
Q13200
Q13526
Q14738
Q14997
Q15008
Q15172
Q92530
Q92997
Q99436
Q99460
Q9HB71
Q9UL46
Q9UNM6
Q9UPN3
