# Supplementary material for: Complex‐centric proteome profiling by SEC‐SWATH‐MS
Source: Mol Syst Biol. 2019 Jan 14;15(1):e8438. doi: 10.15252/msb.20188438 (PMC6346213; doi:10.15252/msb.20188438)
Supplement: Supplementary file 8 — Dataset EV7 [file MSB-15-e8438-s008.zip › feature_plots_string/O15235.pdf]

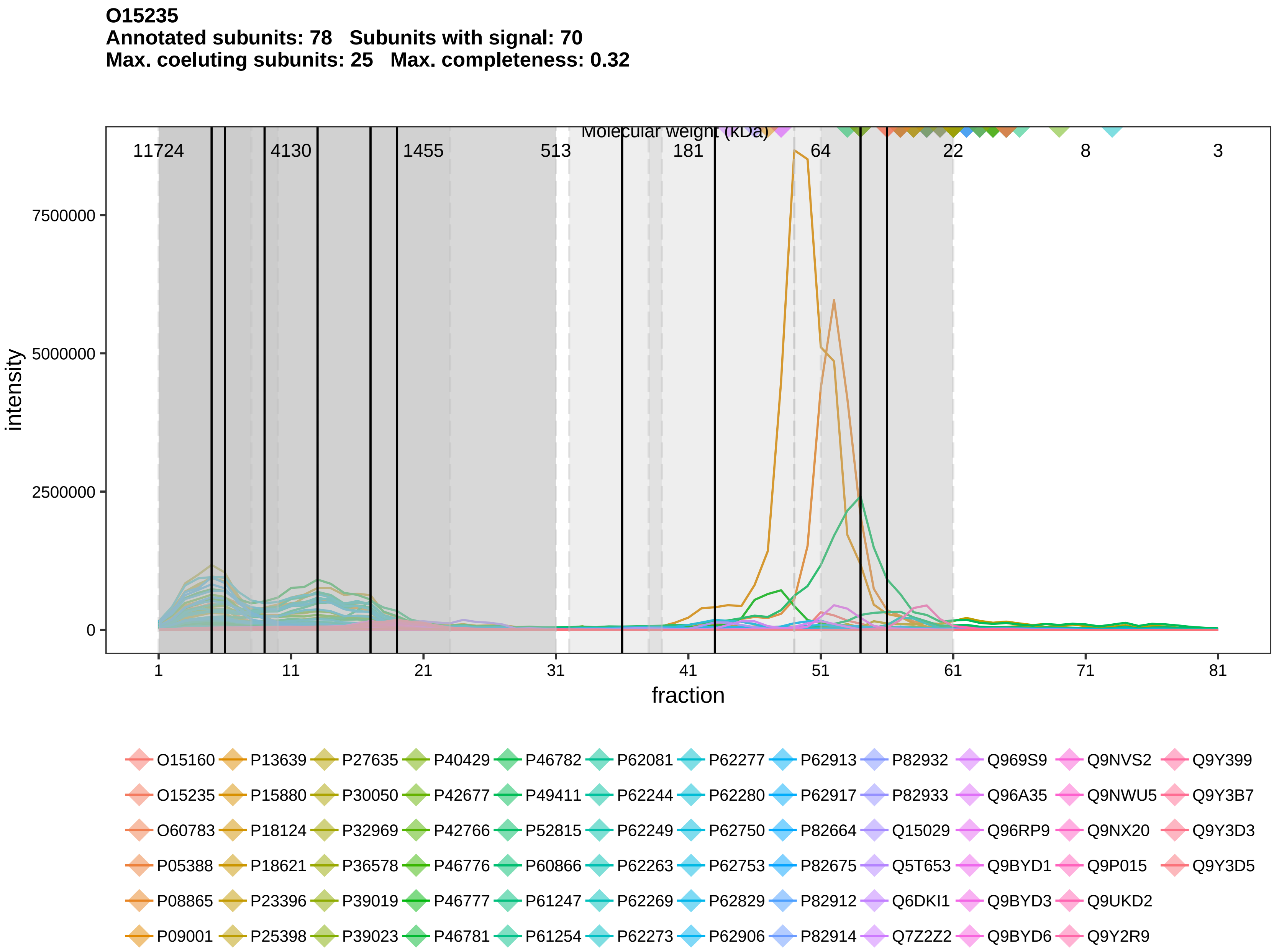

O15235
Annotated subunits: 78   Subunits with signal: 70
Max. coeluting subunits: 25   Max. completeness: 0.32
Molecular weight (kDa)
11724
4130
1455
513
181
64
22
8
3
intensity
7500000
5000000
2500000
0
1
11
21
31
41
51
61
71
81
fraction
O15160
P13639
P27635
P40429
P46782
P62081
P62277
P62913
P82932
Q969S9
Q9NVS2
Q9Y399
O15235
P15880
P30050
P42677
P49411
P62244
P62280
P62917
P82933
Q96A35
Q9NWU5
Q9Y3B7
O60783
P18124
P32969
P42766
P52815
P62249
P62750
P82664
Q15029
Q96RP9
Q9NX20
Q9Y3D3
P05388
P18621
P36578
P46776
P60866
P62263
P62753
P82675
Q5T653
Q9BYD1
Q9P015
Q9Y3D5
P08865
P23396
P39019
P46777
P61247
P62269
P62829
P82912
Q6DKI1
Q9BYD3
Q9UKD2
P09001
P25398
P39023
P46781
P61254
P62273
P62906
P82914
Q7Z2Z2
Q9BYD6
Q9Y2R9
