# Supplementary material for: Complex‐centric proteome profiling by SEC‐SWATH‐MS
Source: Mol Syst Biol. 2019 Jan 14;15(1):e8438. doi: 10.15252/msb.20188438 (PMC6346213; doi:10.15252/msb.20188438)
Supplement: Supplementary file 8 — Dataset EV7 [file MSB-15-e8438-s008.zip › feature_plots_string/O15381.pdf]

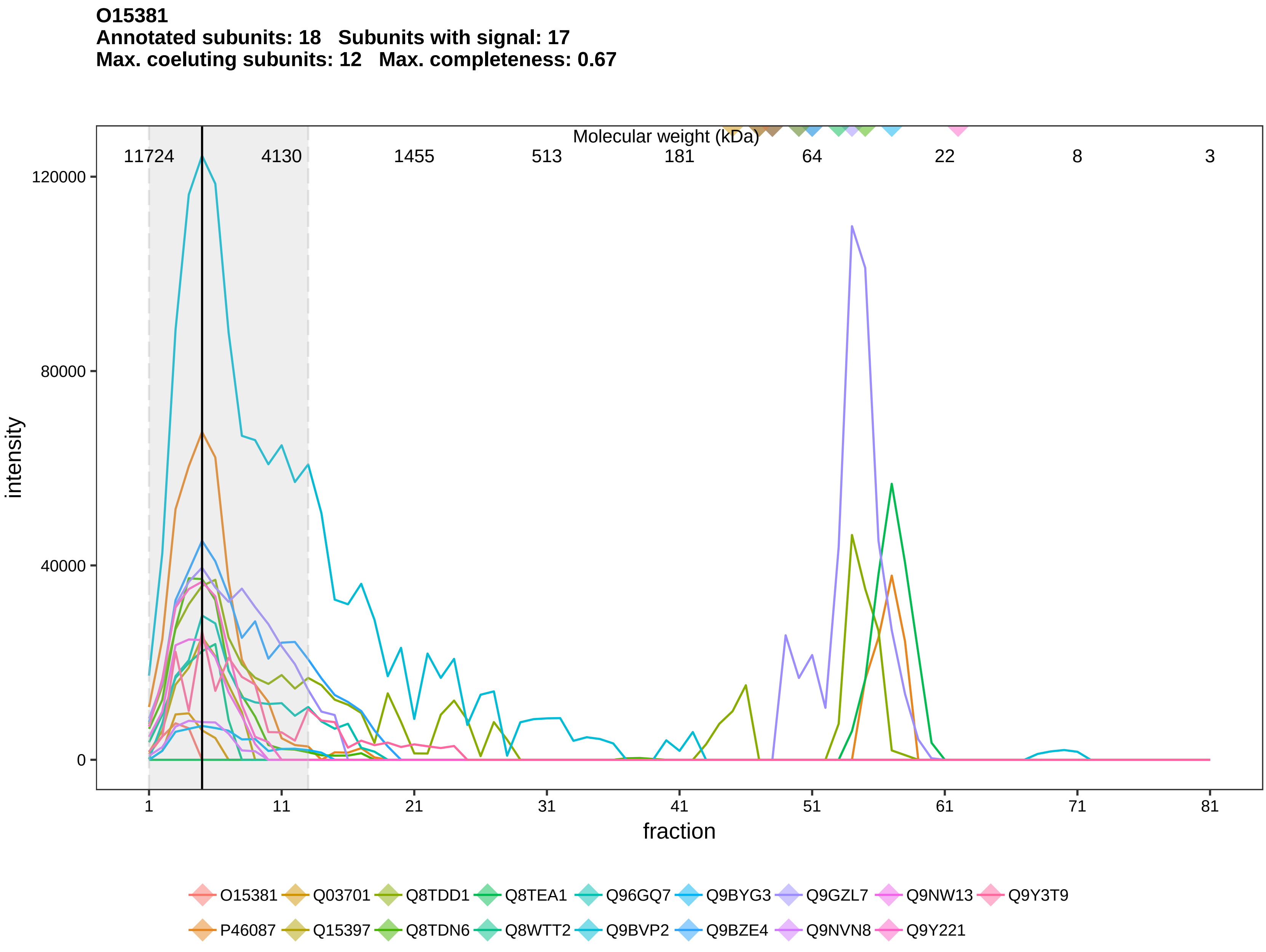
O15381
Annotated subunits: 18 Subunits with signal: 17
Max. coeluting subunits: 12 Max. completeness: 0.67
Molecular weight (kDa)
11724
4130
1455
513
181
64
22
8
3
intensity
120000
80000
40000
0
1
11
21
31
41
51
61
71
81
fraction
O15381
Q03701
Q8TDD1
Q8TEA1
Q96GQ7
Q9BYG3
Q9GZL7
Q9NW13
Q9Y3T9
P46087
Q15397
Q8TDN6
Q8WTT2
Q9BVP2
Q9BZE4
Q9NVN8
Q9Y221
