# Supplementary material for: Complex‐centric proteome profiling by SEC‐SWATH‐MS
Source: Mol Syst Biol. 2019 Jan 14;15(1):e8438. doi: 10.15252/msb.20188438 (PMC6346213; doi:10.15252/msb.20188438)
Supplement: Supplementary file 8 — Dataset EV7 [file MSB-15-e8438-s008.zip › feature_plots_string/O43172.pdf]

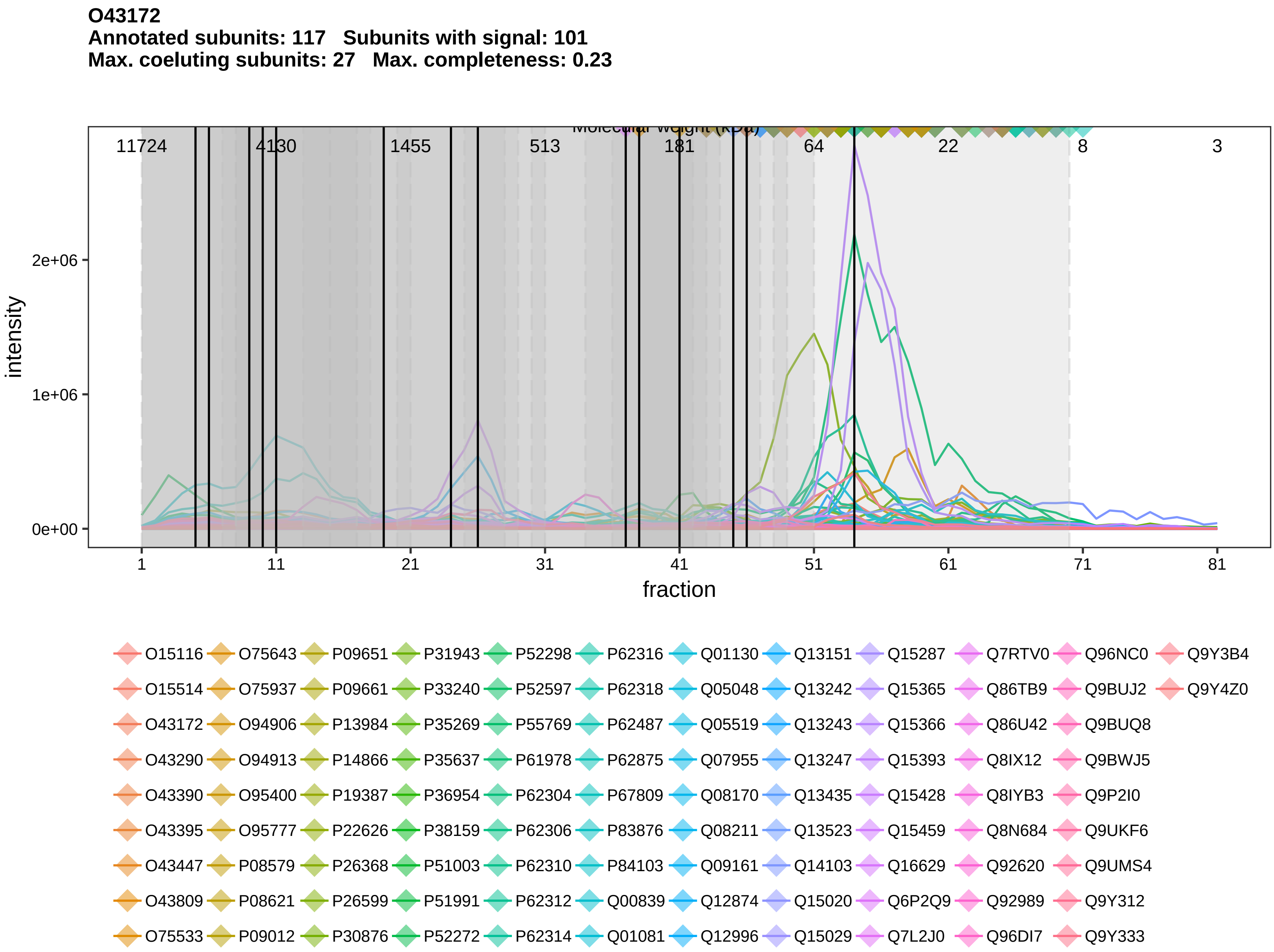
O43172
Annotated subunits: 117 Subunits with signal: 101
Max. coeluting subunits: 27 Max. completeness: 0.23
11724
4130
1455
513
181
64
22
8
3
2e+06
1e+06
0e+00
intensity
1
11
21
31
41
51
61
71
81
fraction
O15116
O15514
O43172
O43290
O43390
O43395
O43447
O43809
O75533
O75643
O75937
O94906
O94913
O95400
O95777
P08579
P08621
P09012
P09651
P09661
P13984
P14866
P19387
P22626
P26368
P26599
P30876
P31943
P33240
P35269
P35637
P36954
P38159
P51003
P51991
P52272
P52298
P52597
P55769
P61978
P62304
P62306
P62310
P62312
P62314
P62316
P62318
P62487
P62875
P67809
P83876
P84103
Q00839
Q01081
Q01130
Q05048
Q05519
Q07955
Q08170
Q08211
Q09161
Q12874
Q12996
Q13151
Q13242
Q13243
Q13247
Q13435
Q13523
Q14103
Q15020
Q15029
Q15287
Q15365
Q15366
Q15393
Q15428
Q15459
Q16629
Q6P2Q9
Q7L2J0
Q7RTV0
Q86TB9
Q86U42
Q8IX12
Q8IYB3
Q8N684
Q92620
Q92989
Q96DI7
Q96NC0
Q9BUJ2
Q9BUQ8
Q9BWJ5
Q9P2I0
Q9UKF6
Q9UMS4
Q9Y312
Q9Y333
Q9Y3B4
Q9Y4Z0
