# Supplementary material for: Complex‐centric proteome profiling by SEC‐SWATH‐MS
Source: Mol Syst Biol. 2019 Jan 14;15(1):e8438. doi: 10.15252/msb.20188438 (PMC6346213; doi:10.15252/msb.20188438)
Supplement: Supplementary file 8 — Dataset EV7 [file MSB-15-e8438-s008.zip › feature_plots_string/O43837.pdf]

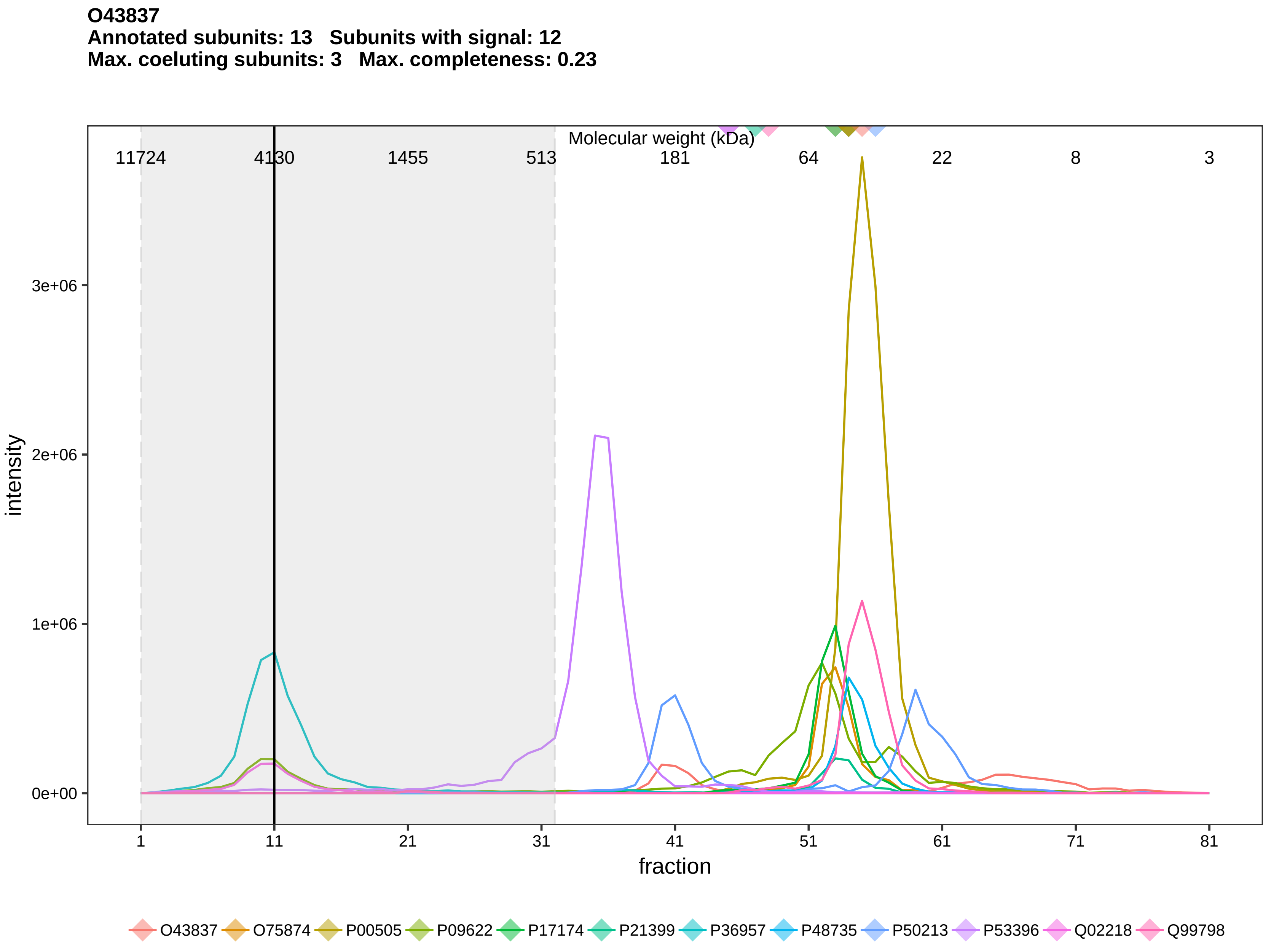

O43837
Annotated subunits: 13 Subunits with signal: 12
Max. coeluting subunits: 3 Max. completeness: 0.23
Molecular weight (kDa)
11724
4130
1455
513
181
64
22
8
3
intensity
3e+06
2e+06
1e+06
0e+00
1
11
21
31
41
51
61
71
81
fraction
O43837
O75874
P00505
P09622
P17174
P21399
P36957
P48735
P50213
P53396
Q02218
Q99798
