# Supplementary material for: Complex‐centric proteome profiling by SEC‐SWATH‐MS
Source: Mol Syst Biol. 2019 Jan 14;15(1):e8438. doi: 10.15252/msb.20188438 (PMC6346213; doi:10.15252/msb.20188438)
Supplement: Supplementary file 8 — Dataset EV7 [file MSB-15-e8438-s008.zip › feature_plots_string/O60264.pdf]

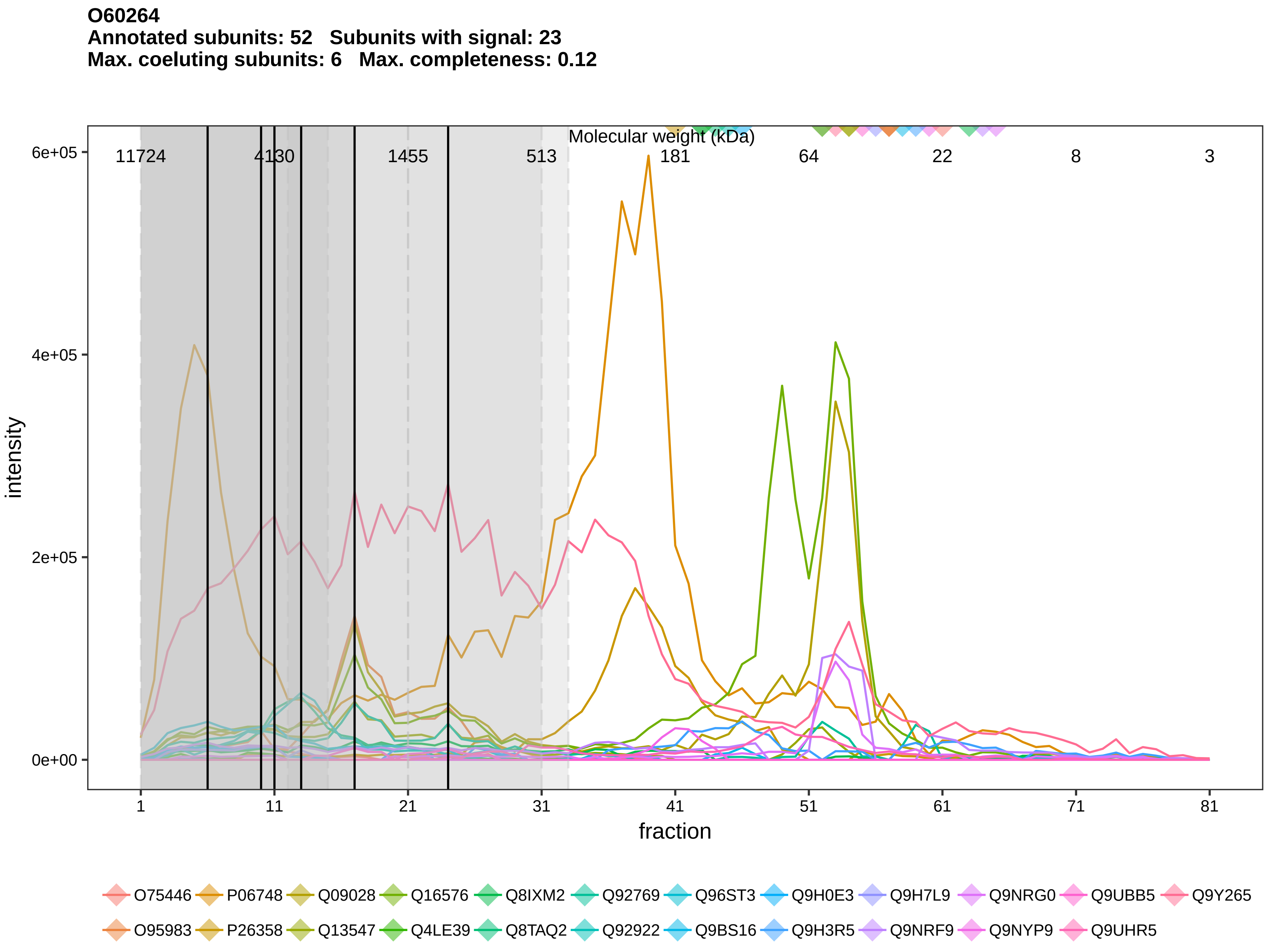
O60264
Annotated subunits: 52 Subunits with signal: 23
Max. coeluting subunits: 6 Max. completeness: 0.12
Molecular weight (kDa)
11724
4130
1455
513
181
64
22
8
3
6e+05
4e+05
2e+05
0e+00
intensity
1
11
21
31
41
51
61
71
81
fraction
O75446
P06748
Q09028
Q16576
Q8IXM2
Q92769
Q96ST3
Q9H0E3
Q9H7L9
Q9NRG0
Q9UBB5
Q9Y265
O95983
P26358
Q13547
Q4LE39
Q8TAQ2
Q92922
Q9BS16
Q9H3R5
Q9NRF9
Q9NYP9
Q9UHR5
