# Supplementary material for: Complex‐centric proteome profiling by SEC‐SWATH‐MS
Source: Mol Syst Biol. 2019 Jan 14;15(1):e8438. doi: 10.15252/msb.20188438 (PMC6346213; doi:10.15252/msb.20188438)

O75251_O43676_O95178_O43678_O95299_Q16795_O75438_Q16718_O15239_O43920_O95139_Q86Y39
Annotated subunits: 55   Subunits with signal: 45
Max. coeluting subunits: 30   Max. completeness: 0.55

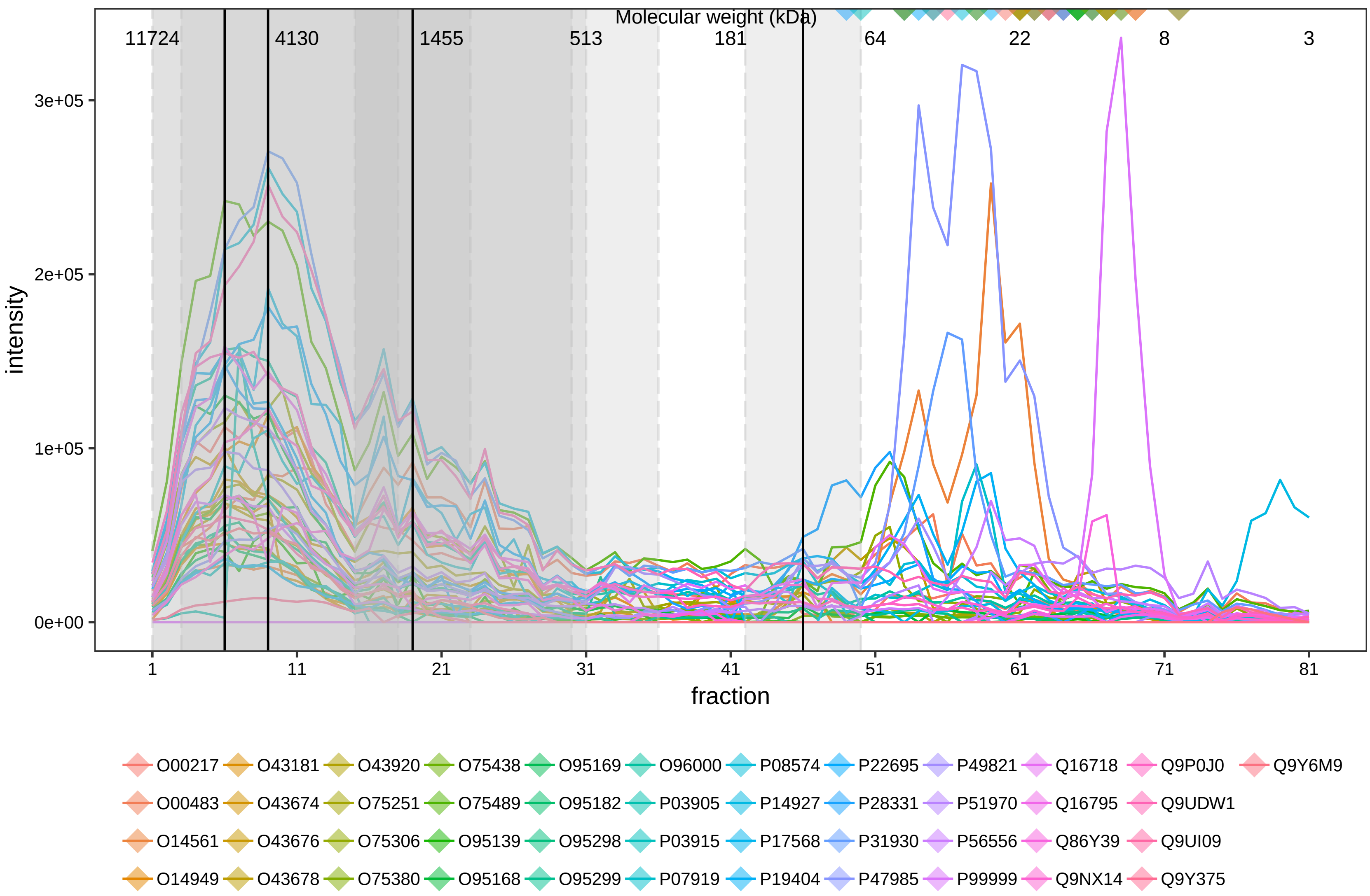

Supplement: Supplementary file 8 — Dataset EV7 [file MSB-15-e8438-s008.zip › feature_plots_string/O75251.pdf]
