# Supplementary material for: Complex‐centric proteome profiling by SEC‐SWATH‐MS
Source: Mol Syst Biol. 2019 Jan 14;15(1):e8438. doi: 10.15252/msb.20188438 (PMC6346213; doi:10.15252/msb.20188438)
Supplement: Supplementary file 8 — Dataset EV7 [file MSB-15-e8438-s008.zip › feature_plots_string/O75506.pdf]

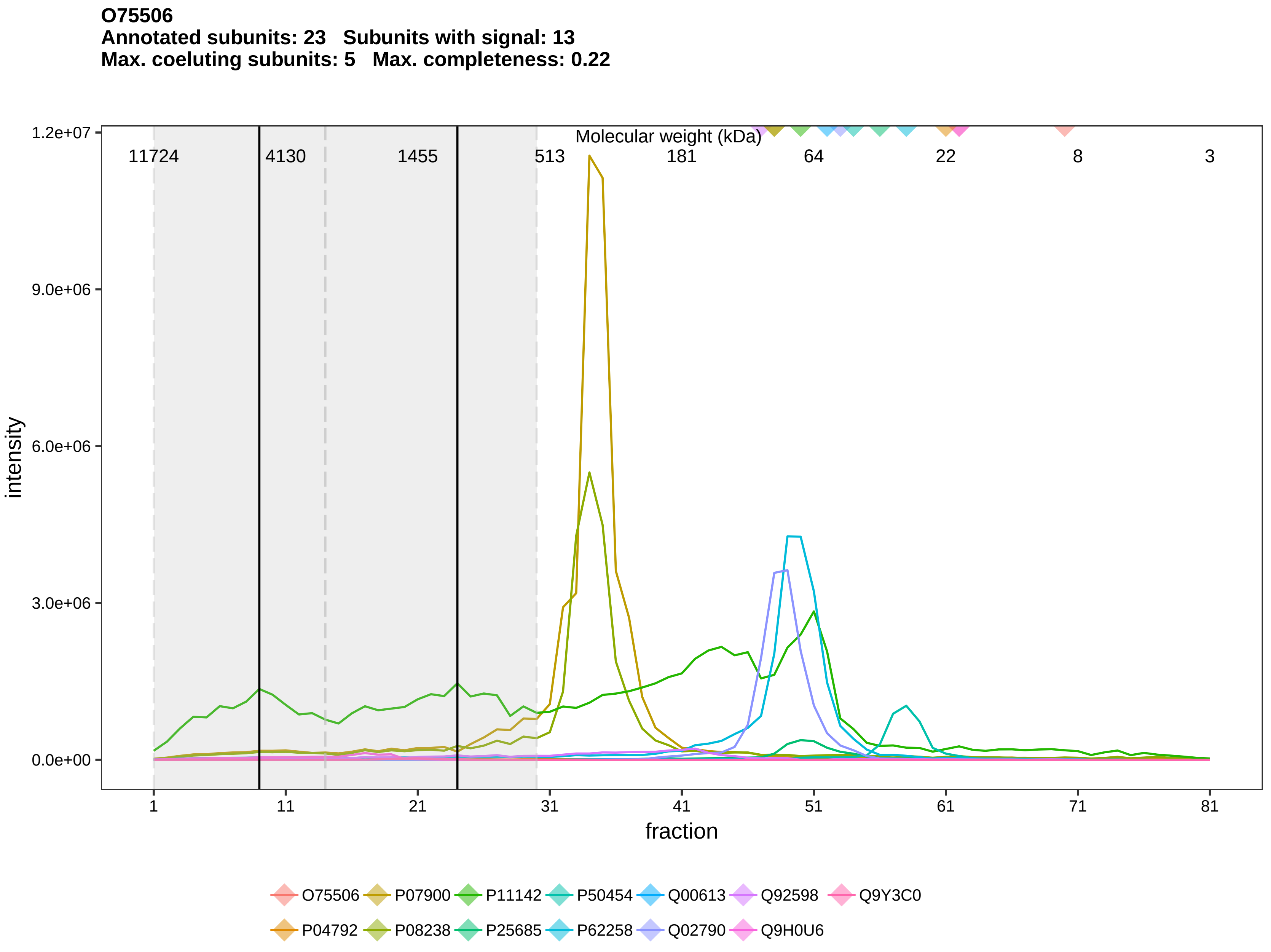

O75506
Annotated subunits: 23 Subunits with signal: 13
Max. coeluting subunits: 5 Max. completeness: 0.22
Molecular weight (kDa)
11724
4130
1455
513
181
64
22
8
3
1.2e+07
9.0e+06
6.0e+06
3.0e+06
0.0e+00
intensity
1
11
21
31
41
51
61
71
81
fraction
O75506
P07900
P11142
P50454
Q00613
Q92598
Q9Y3C0
P04792
P08238
P25685
P62258
Q02790
Q9H0U6
