# Supplementary figures and images for: Complex‐centric proteome profiling by SEC‐SWATH‐MS (part 3 of 3)
Source: Mol Syst Biol. 2019 Jan 14;15(1):e8438. doi: 10.15252/msb.20188438 (PMC6346213; doi:10.15252/msb.20188438)

O14949

Annotated subunits: 79 Subunits with signal: 62

Max. coeluting subunits: 38 Max. completeness: 0.48

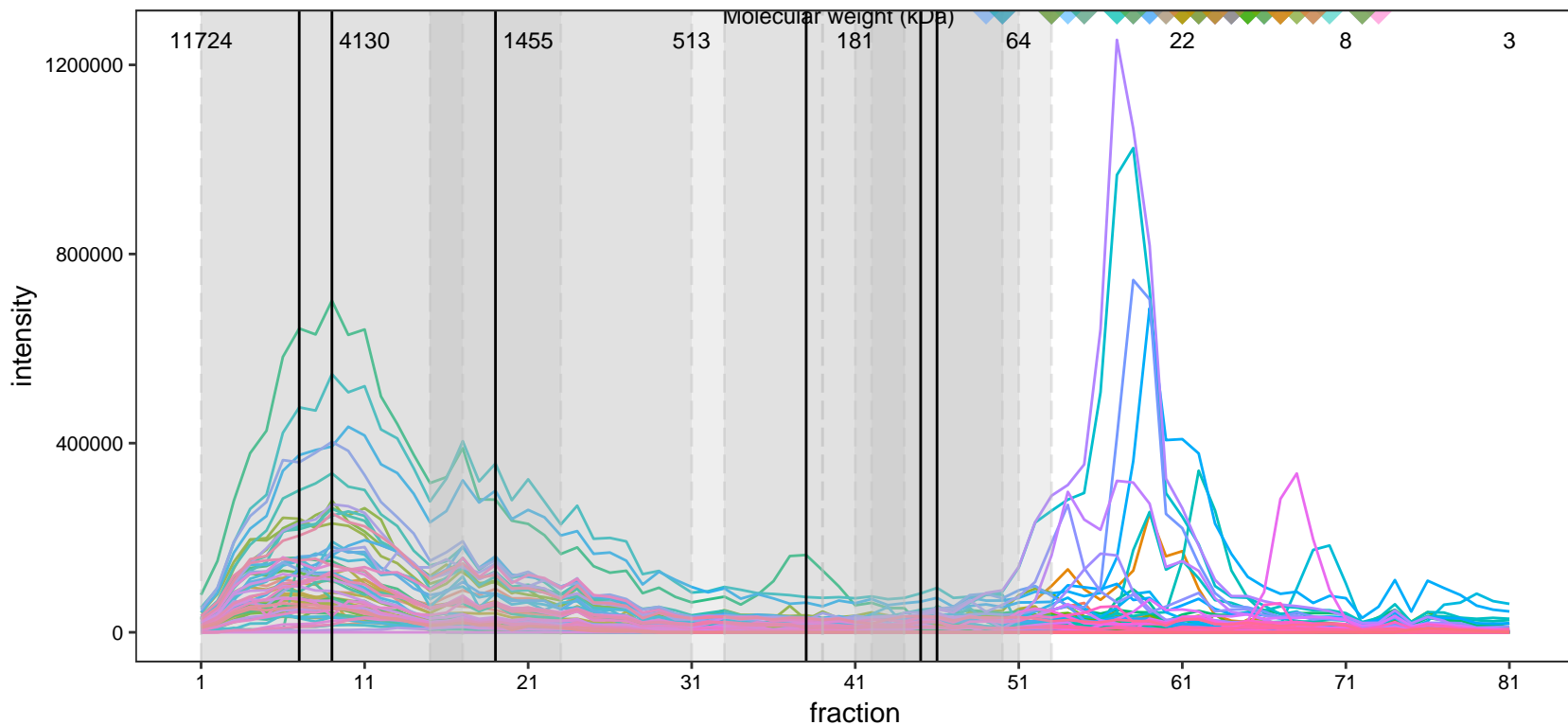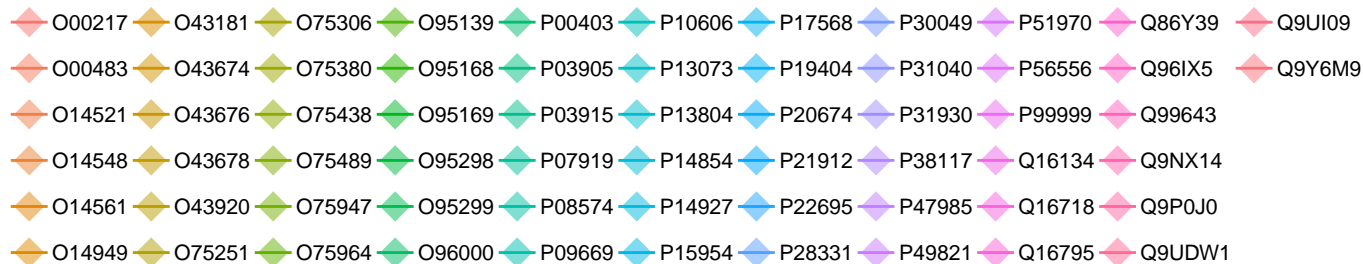

Supplement: Supplementary file 8 — Dataset EV7 [file MSB-15-e8438-s008.zip › feature_plots_string/O14949.pdf]

O14957

Annotated subunits: 6 Subunits with signal: 5

Max. coeluting subunits: 5 Max. completeness: 0.83

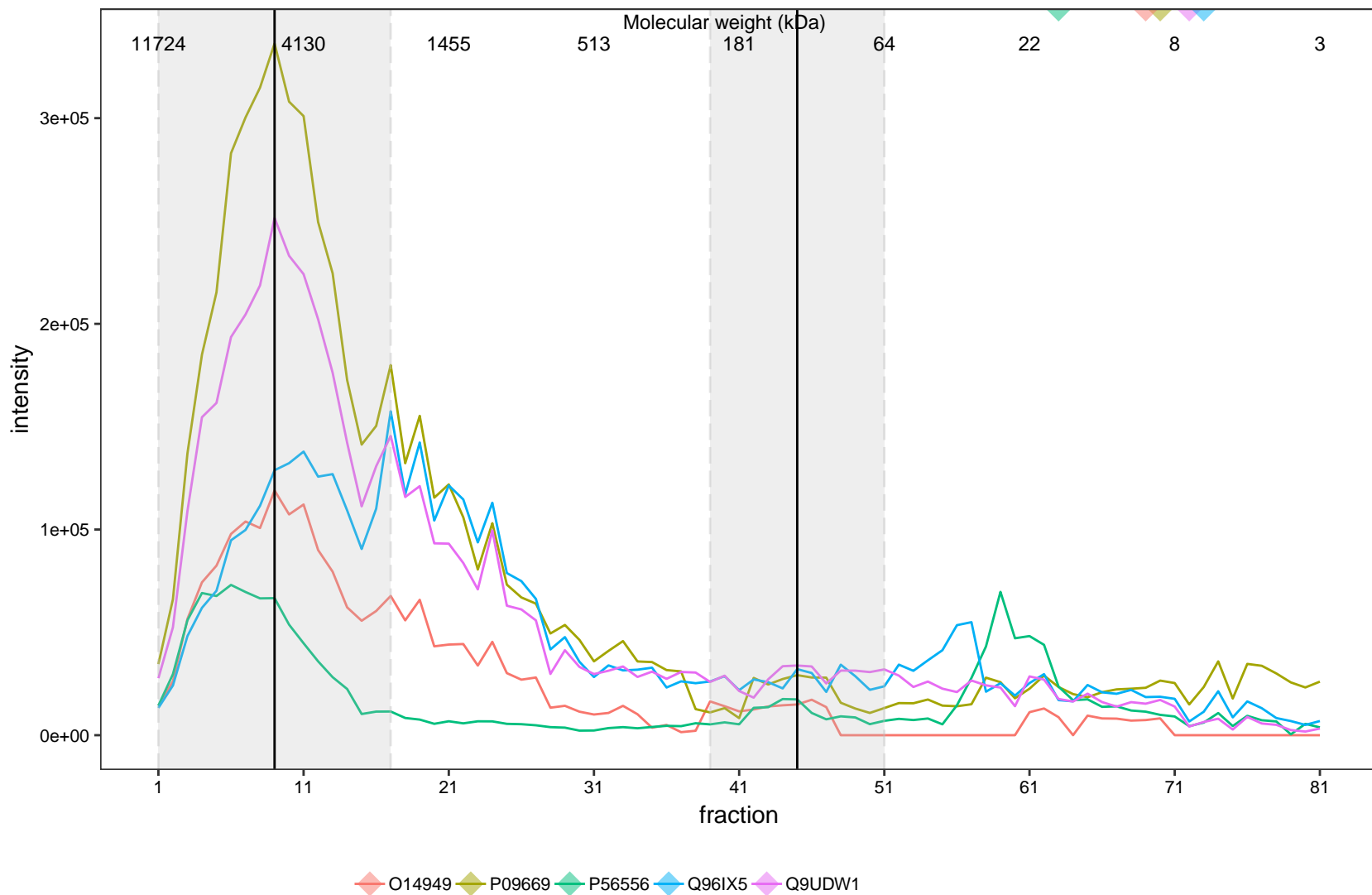

Supplement: Supplementary file 8 — Dataset EV7 [file MSB-15-e8438-s008.zip › feature_plots_string/O14957.pdf]

O14975  
Annotated subunits: 3   Subunits with signal: 3  
Max. coeluting subunits: 2   Max. completeness: 0.67

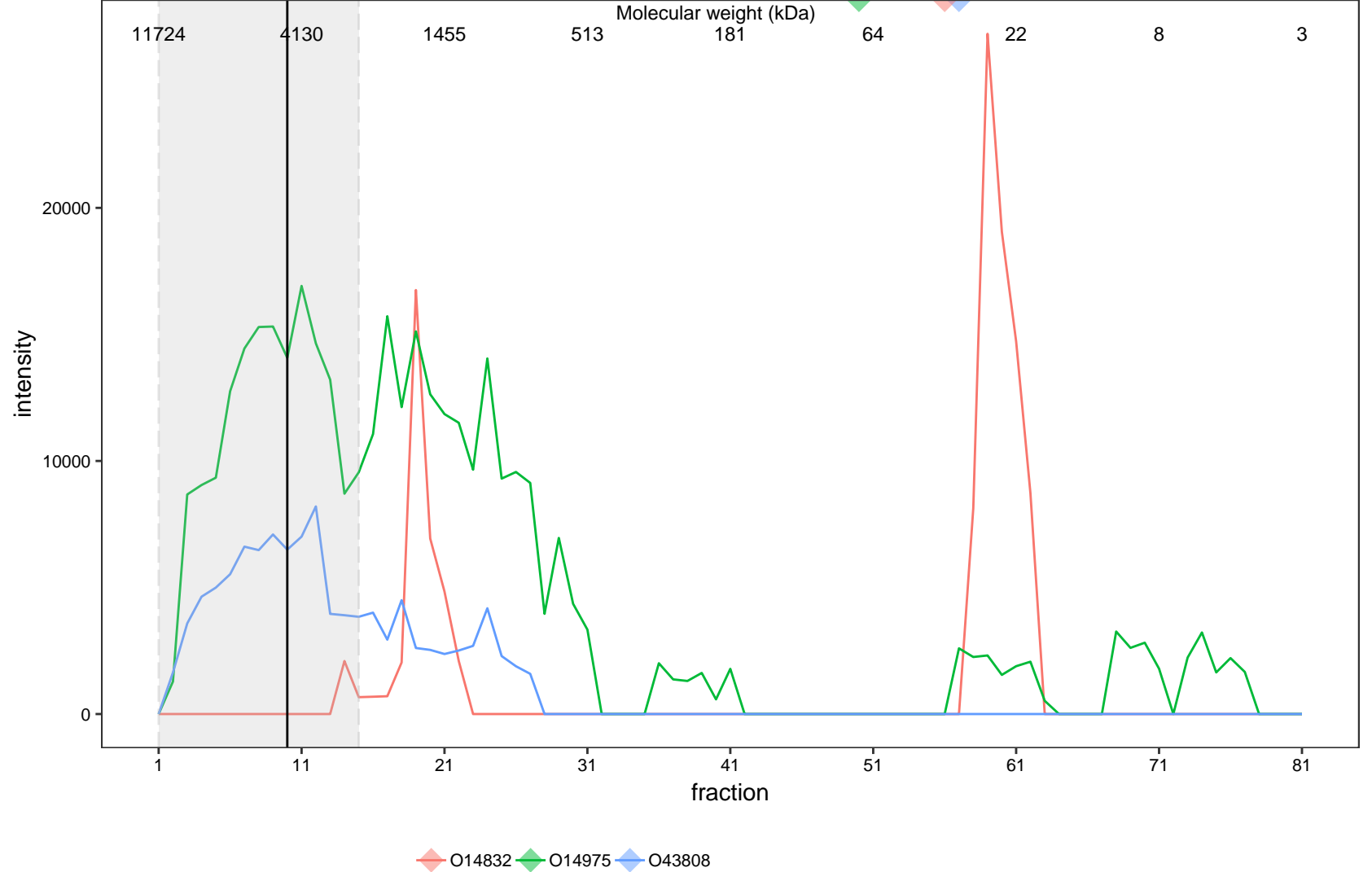

Supplement: Supplementary file 8 — Dataset EV7 [file MSB-15-e8438-s008.zip › feature_plots_string/O14975.pdf]

**O14976**

**Annotated subunits: 6 Subunits with signal: 4**

**Max. coeluting subunits: 3 Max. completeness: 0.5**

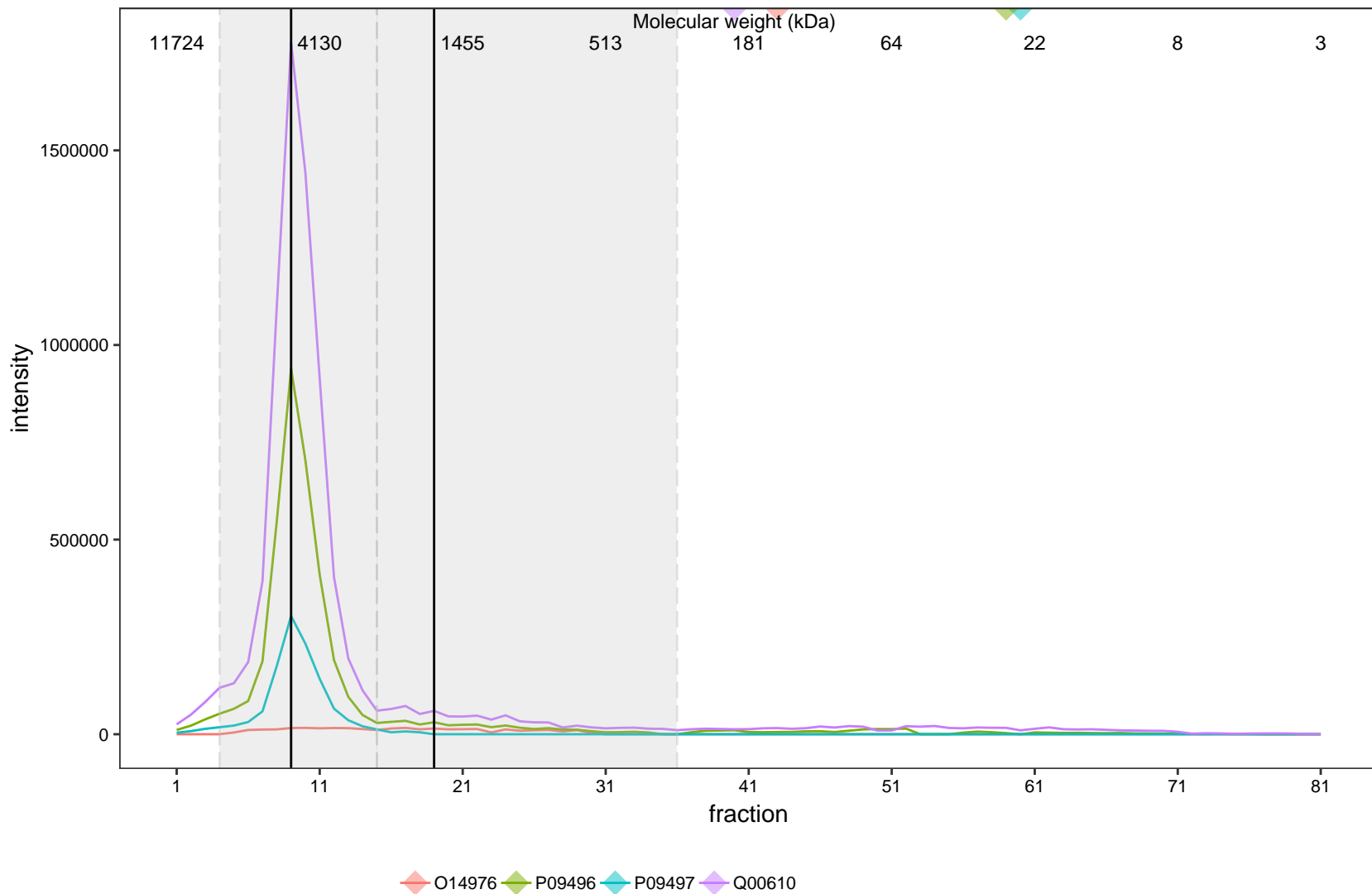

Supplement: Supplementary file 8 — Dataset EV7 [file MSB-15-e8438-s008.zip › feature_plots_string/O14976.pdf]

O15054  
Annotated subunits: 20   Subunits with signal: 5  
Max. coeluting subunits: 2   Max. completeness: 0.1

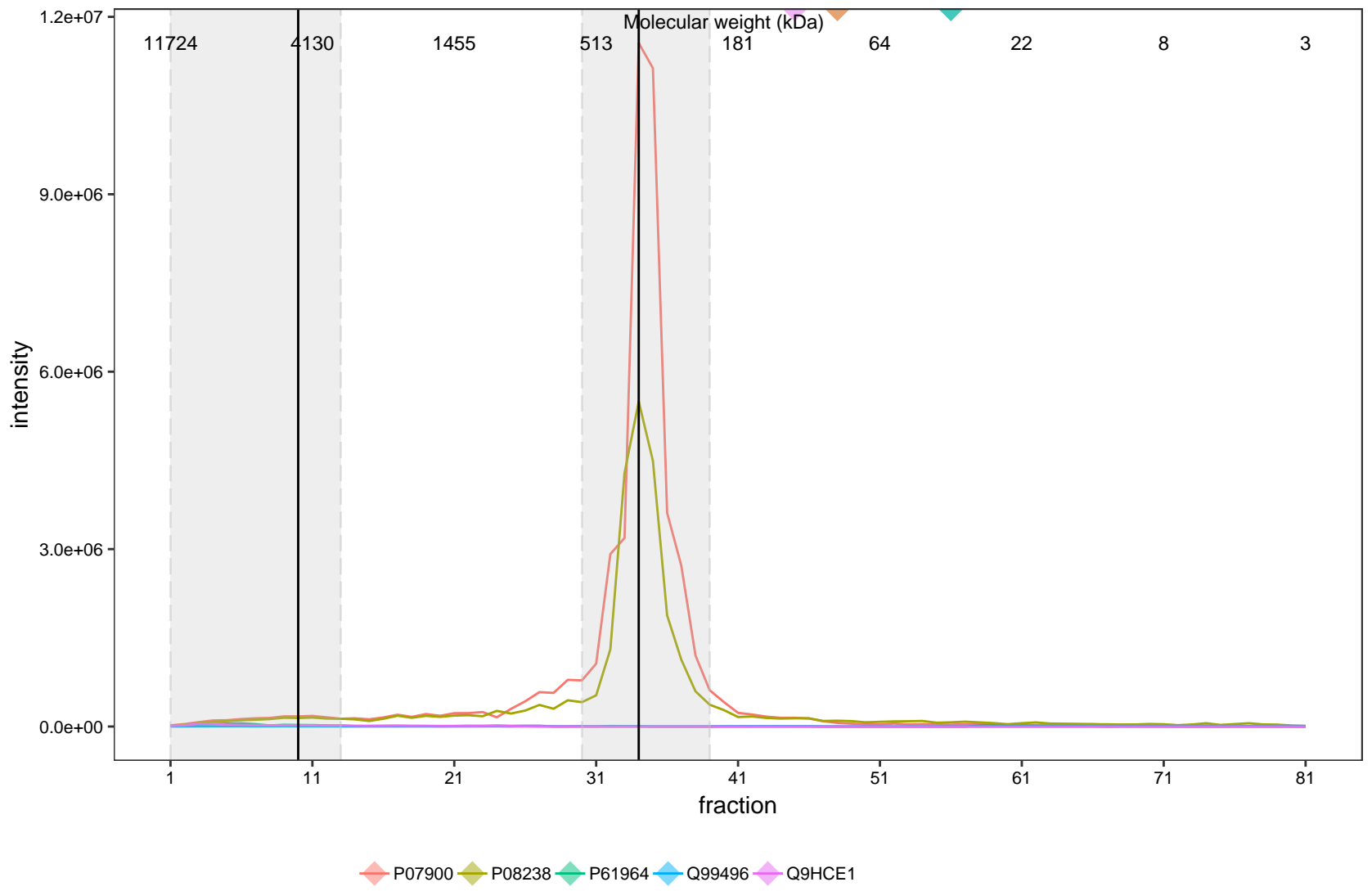

Supplement: Supplementary file 8 — Dataset EV7 [file MSB-15-e8438-s008.zip › feature_plots_string/O15054.pdf]

**O15066**

**Annotated subunits: 9   Subunits with signal: 7**

**Max. coeluting subunits: 2    Max. completeness: 0.22**

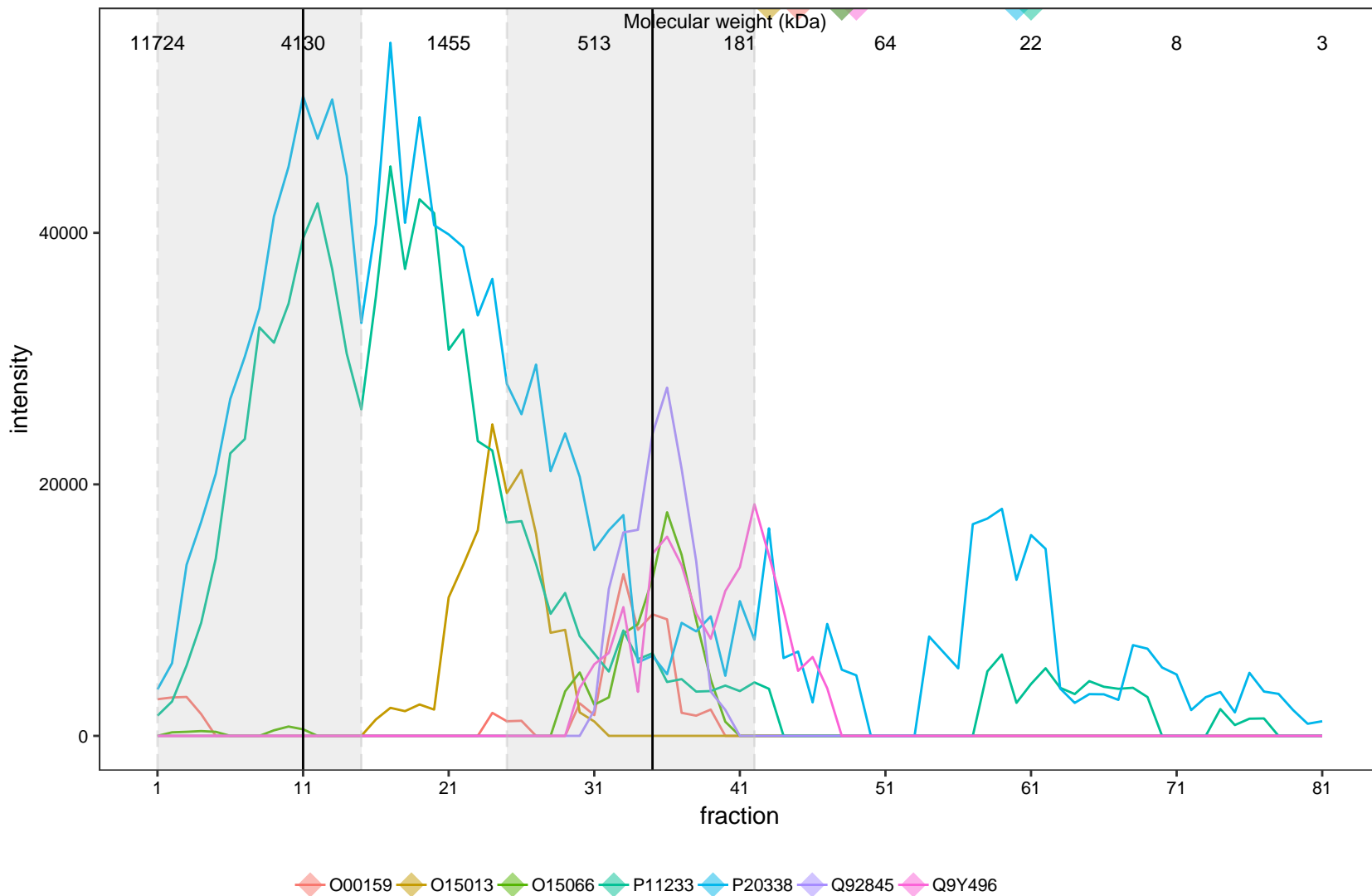

Supplement: Supplementary file 8 — Dataset EV7 [file MSB-15-e8438-s008.zip › feature_plots_string/O15066.pdf]

**O15069**

**Annotated subunits: 3 Subunits with signal: 2**

**Max. coeluting subunits: 2 Max. completeness: 0.67**

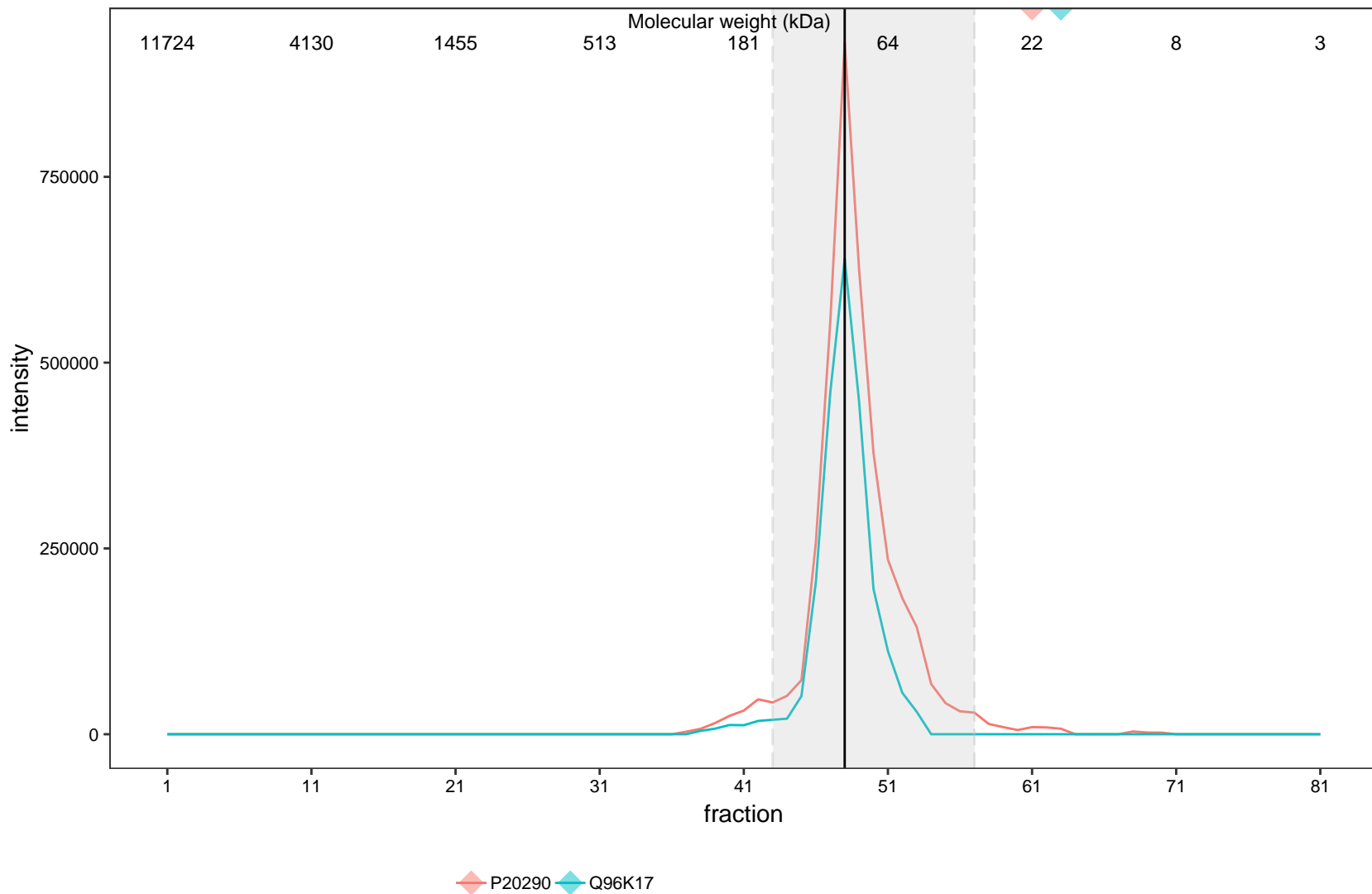

Supplement: Supplementary file 8 — Dataset EV7 [file MSB-15-e8438-s008.zip › feature_plots_string/O15069.pdf]

**O15127**

**Annotated subunits: 5 Subunits with signal: 2**

**Max. coeluting subunits: 2 Max. completeness: 0.4**

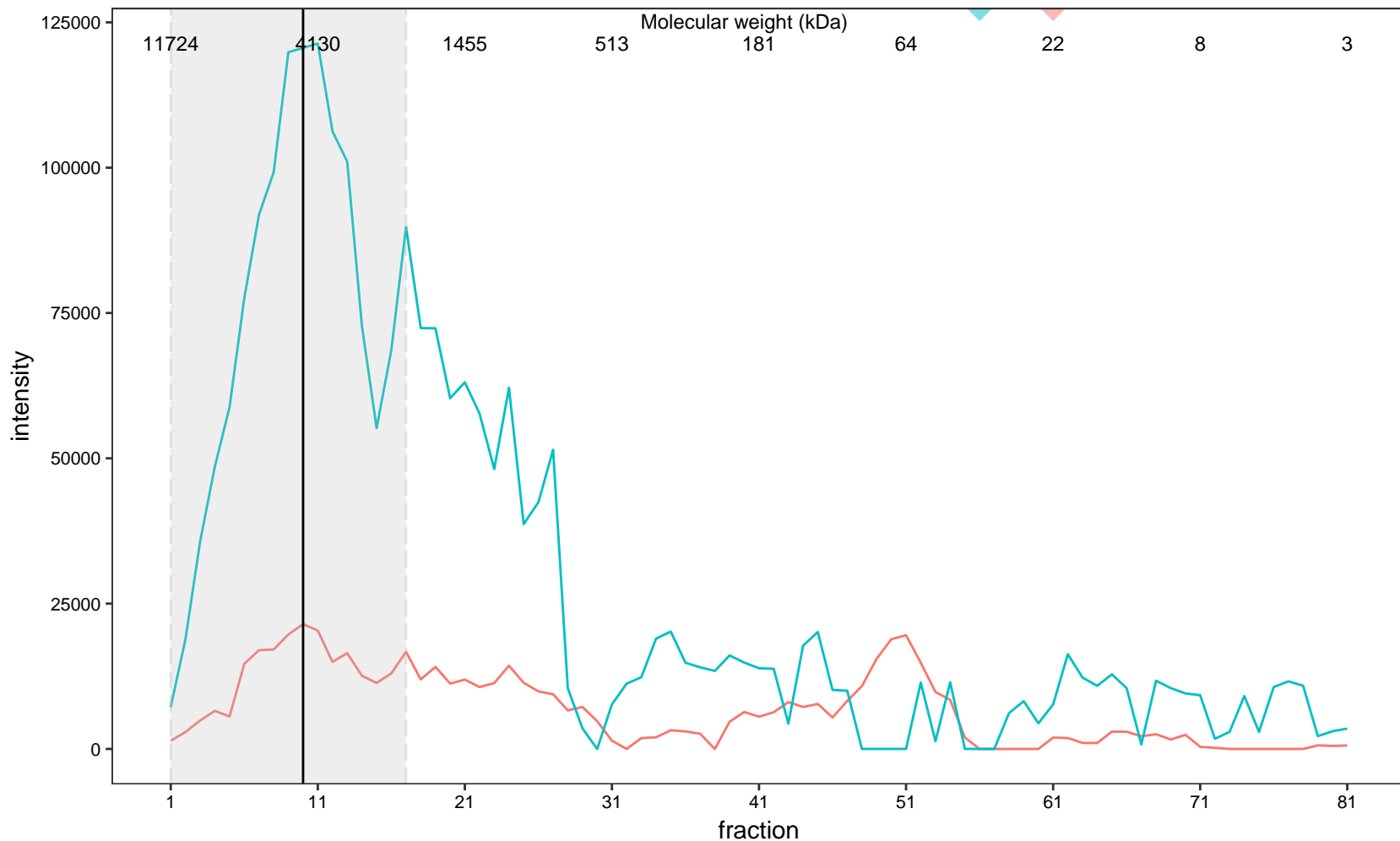

◊ O00161 ◊ O15127

Supplement: Supplementary file 8 — Dataset EV7 [file MSB-15-e8438-s008.zip › feature_plots_string/O15127.pdf]

O15145  
Annotated subunits: 38 Subunits with signal: 25  
Max. coeluting subunits: 11 Max. completeness: 0.29

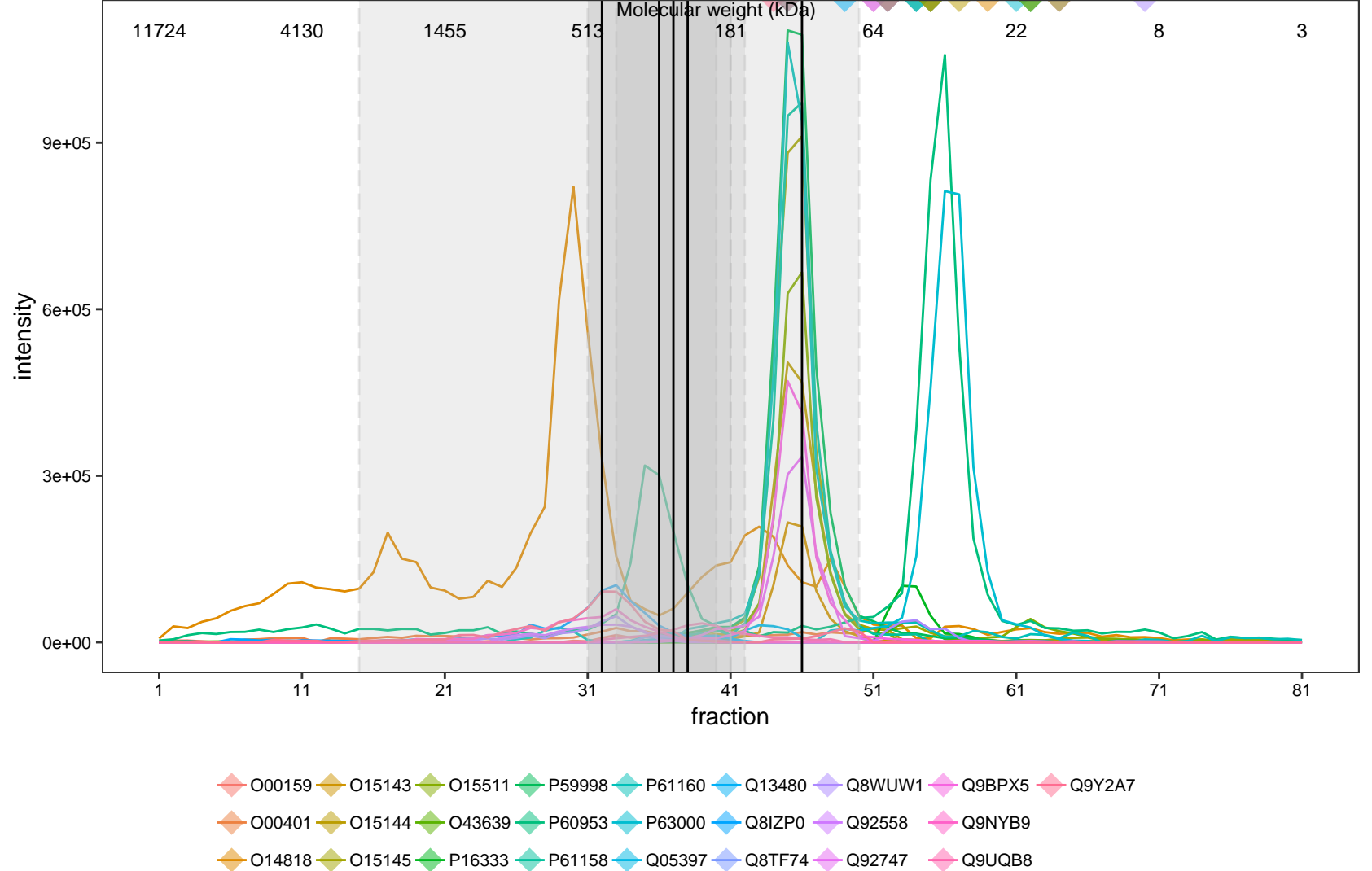

Supplement: Supplementary file 8 — Dataset EV7 [file MSB-15-e8438-s008.zip › feature_plots_string/O15145.pdf]

O15205  
Annotated subunits: 5   Subunits with signal: 3  
Max. coeluting subunits: 2   Max. completeness: 0.4

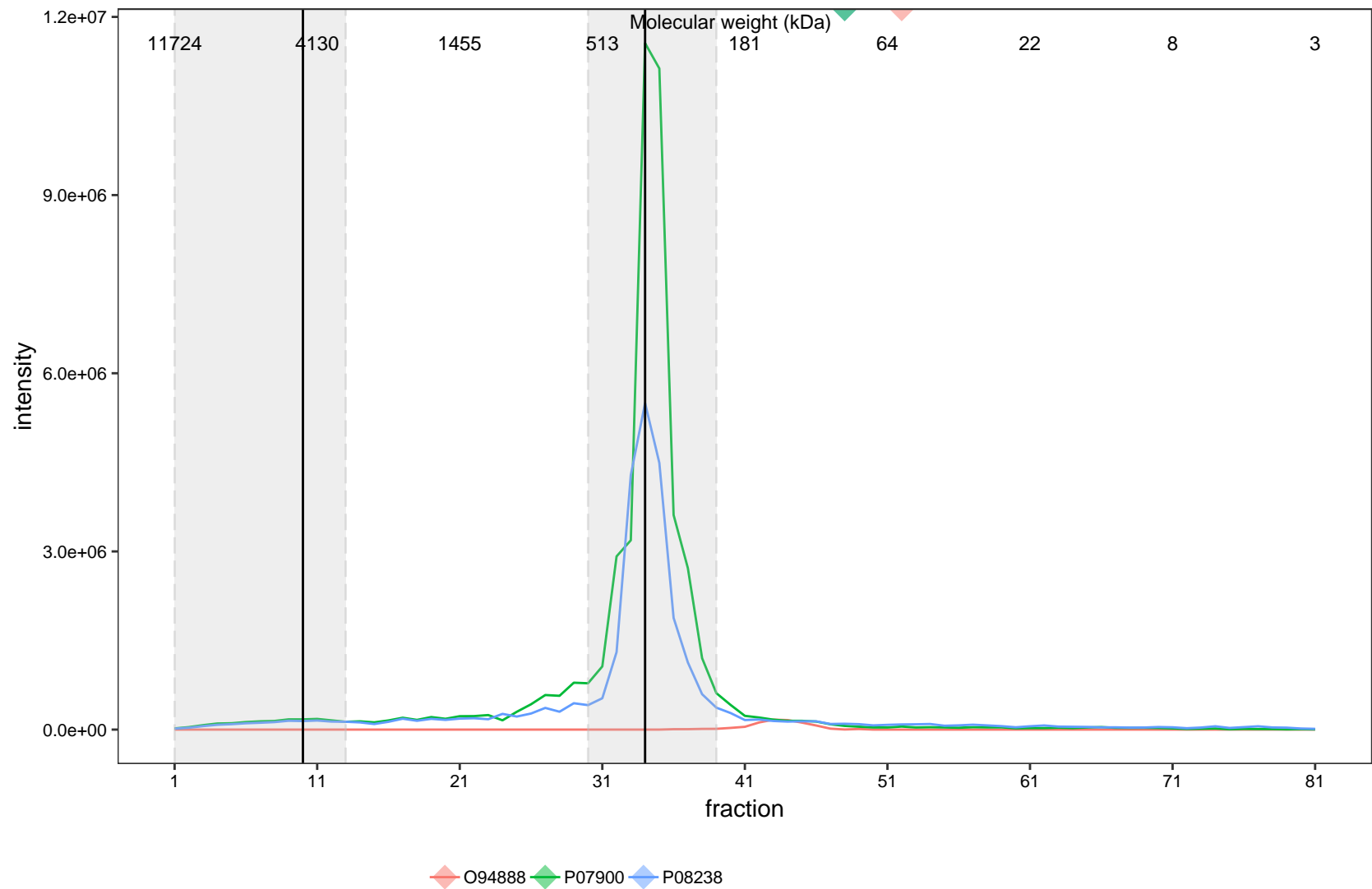

Supplement: Supplementary file 8 — Dataset EV7 [file MSB-15-e8438-s008.zip › feature_plots_string/O15205.pdf]

O15212

Annotated subunits: 4 Subunits with signal: 4

Max. coeluting subunits: 4 Max. completeness: 1

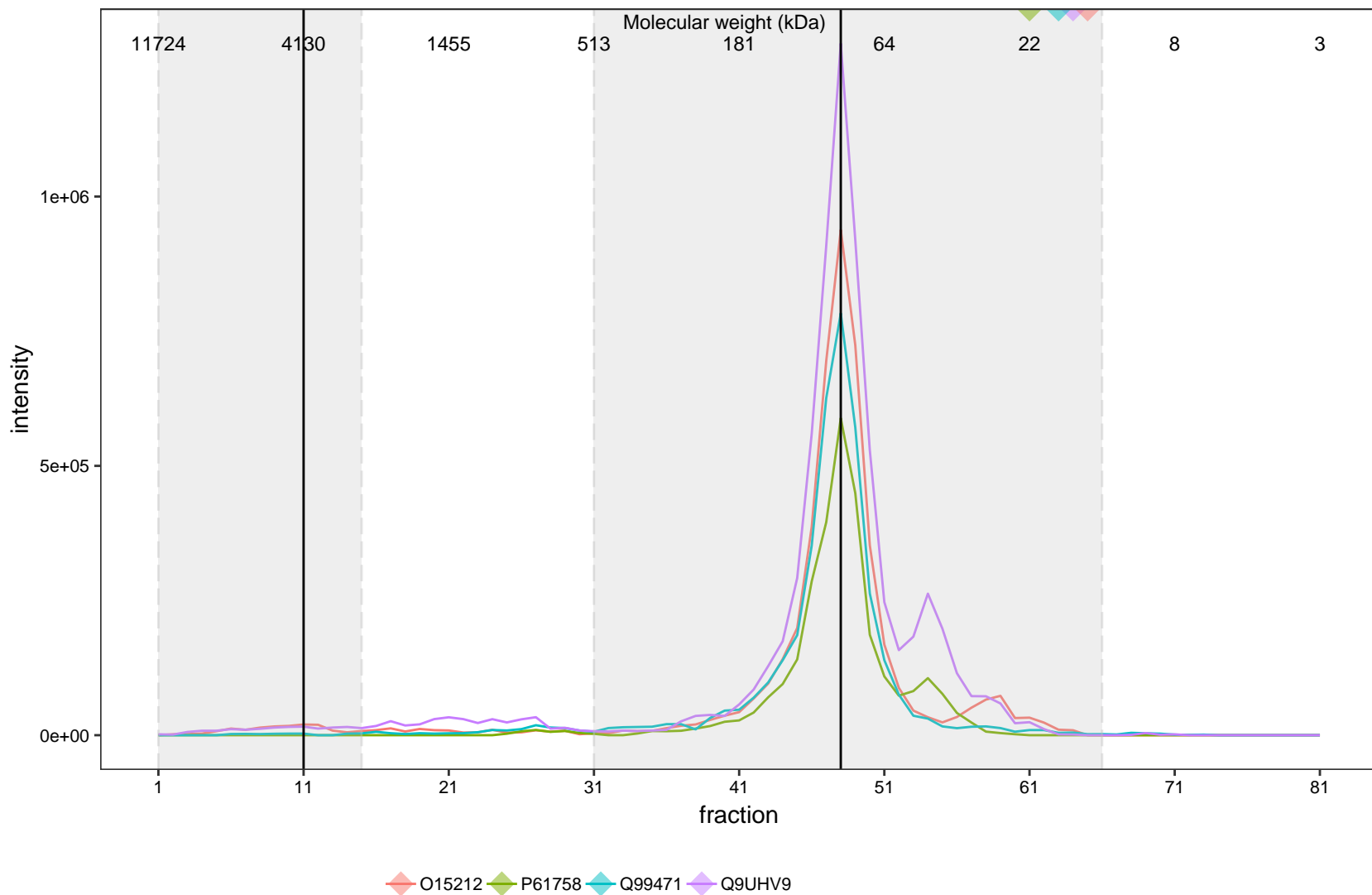

Supplement: Supplementary file 8 — Dataset EV7 [file MSB-15-e8438-s008.zip › feature_plots_string/O15212.pdf]

**O15213**

**Annotated subunits: 31 Subunits with signal: 16**

**Max. coeluting subunits: 6 Max. completeness: 0.19**

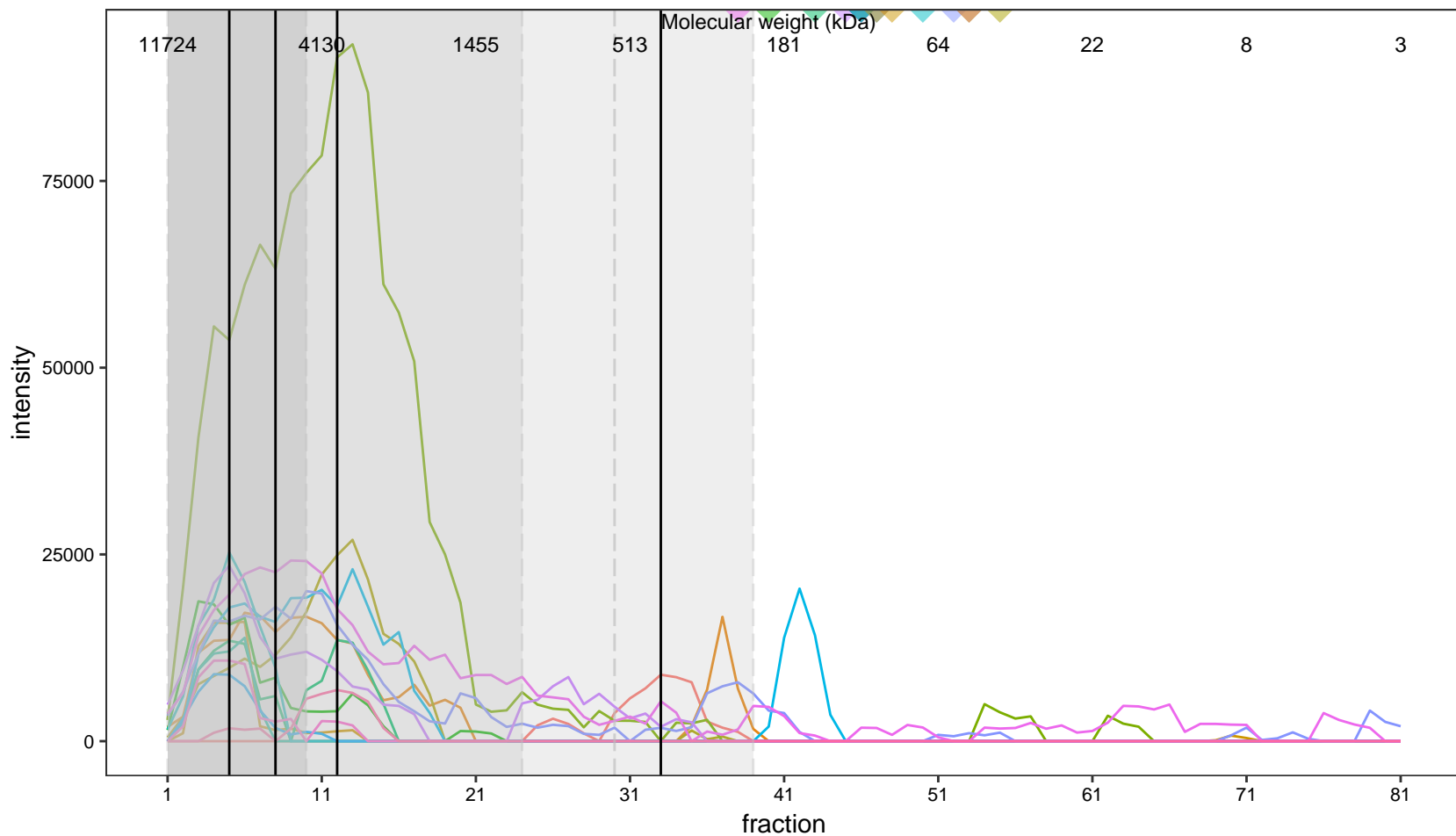

Supplement: Supplementary file 8 — Dataset EV7 [file MSB-15-e8438-s008.zip › feature_plots_string/O15213.pdf]

**O15228**

**Annotated subunits: 7 Subunits with signal: 5**

**Max. coeluting subunits: 3 Max. completeness: 0.43**

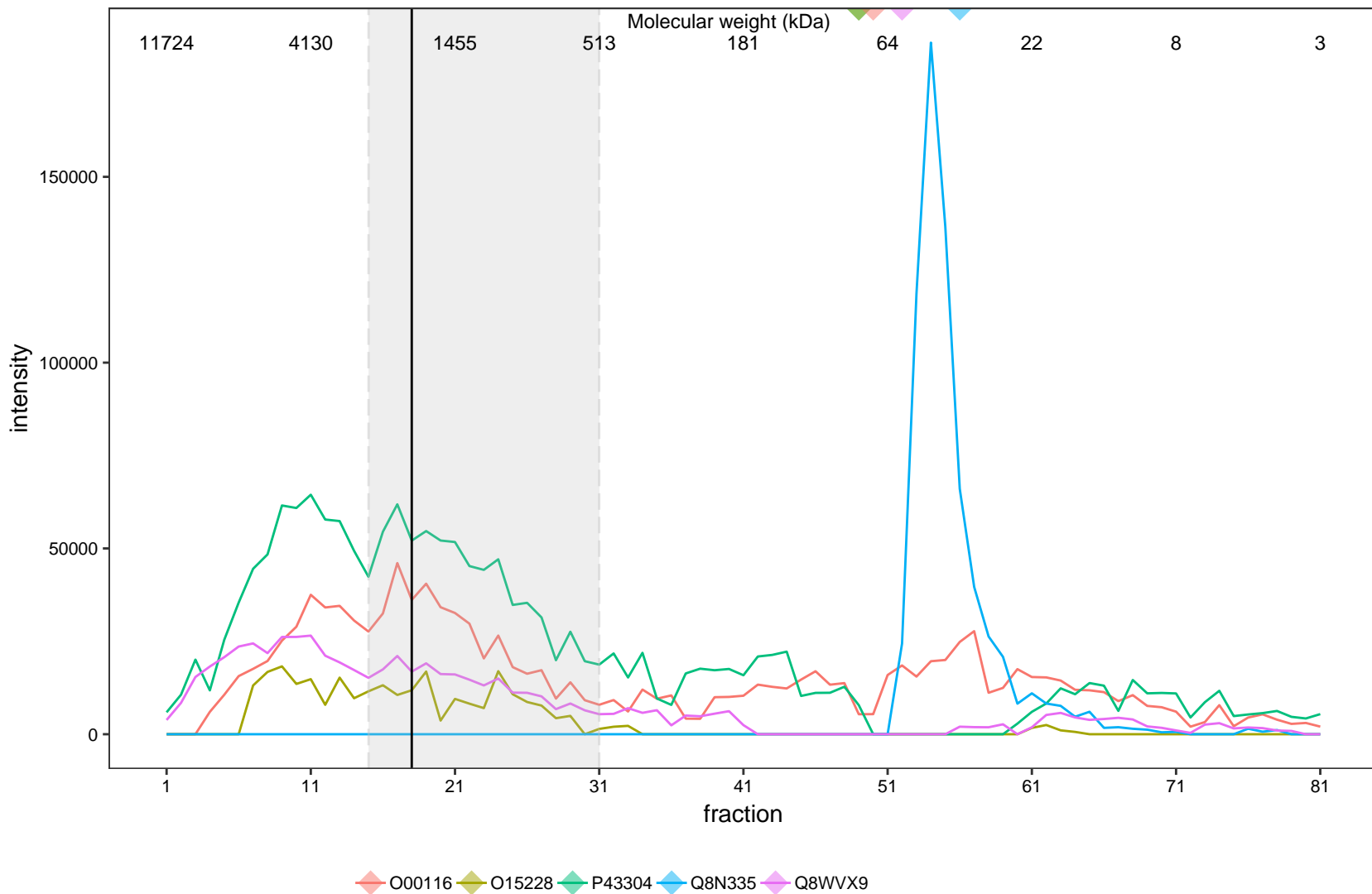

Supplement: Supplementary file 8 — Dataset EV7 [file MSB-15-e8438-s008.zip › feature_plots_string/O15228.pdf]

**O15245**

**Annotated subunits: 4 Subunits with signal: 2**

**Max. coeluting subunits: 2 Max. completeness: 0.5**

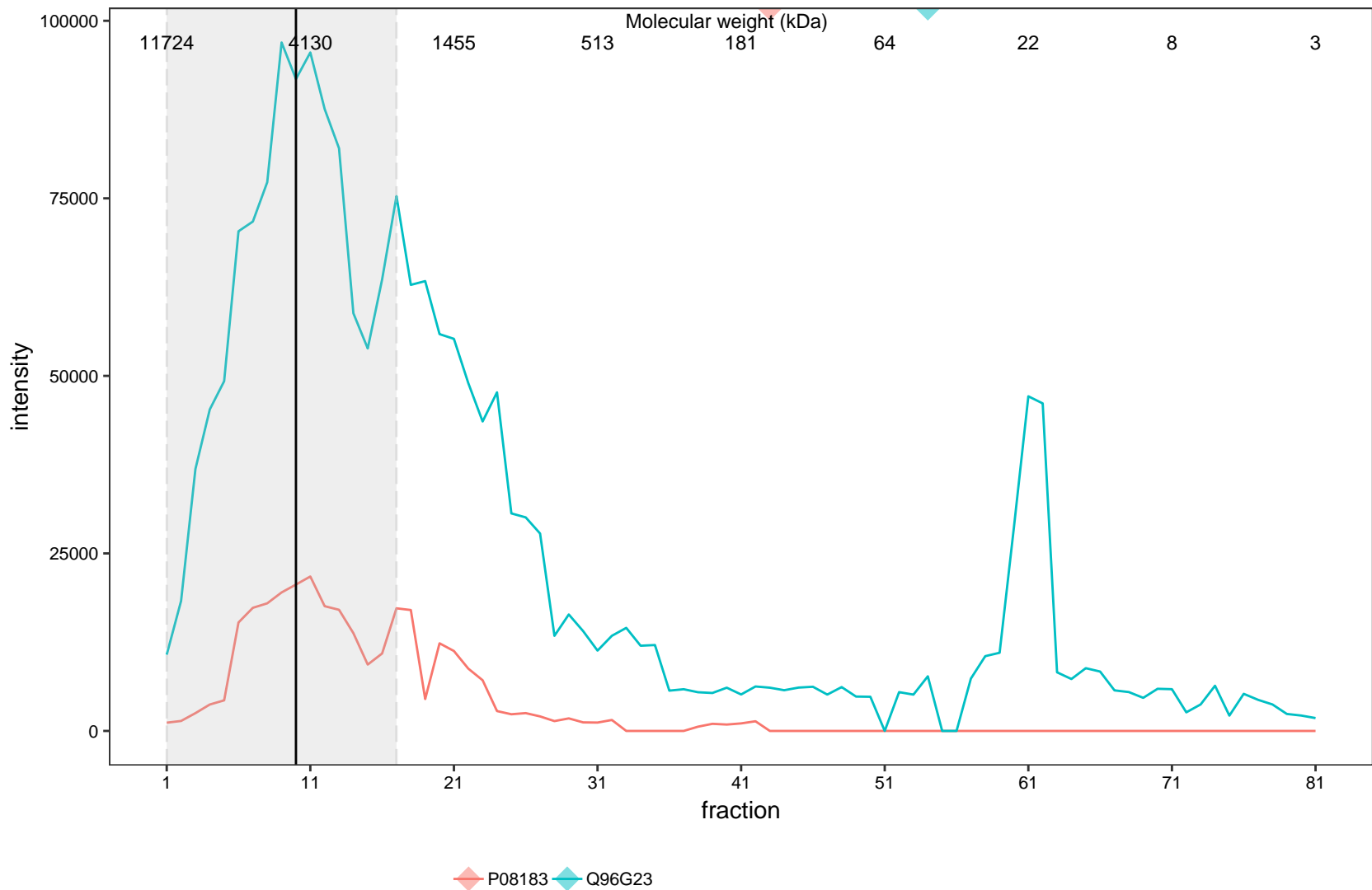

Supplement: Supplementary file 8 — Dataset EV7 [file MSB-15-e8438-s008.zip › feature_plots_string/O15245.pdf]

**O15260**

**Annotated subunits: 3 Subunits with signal: 3**

**Max. coeluting subunits: 2 Max. completeness: 0.67**

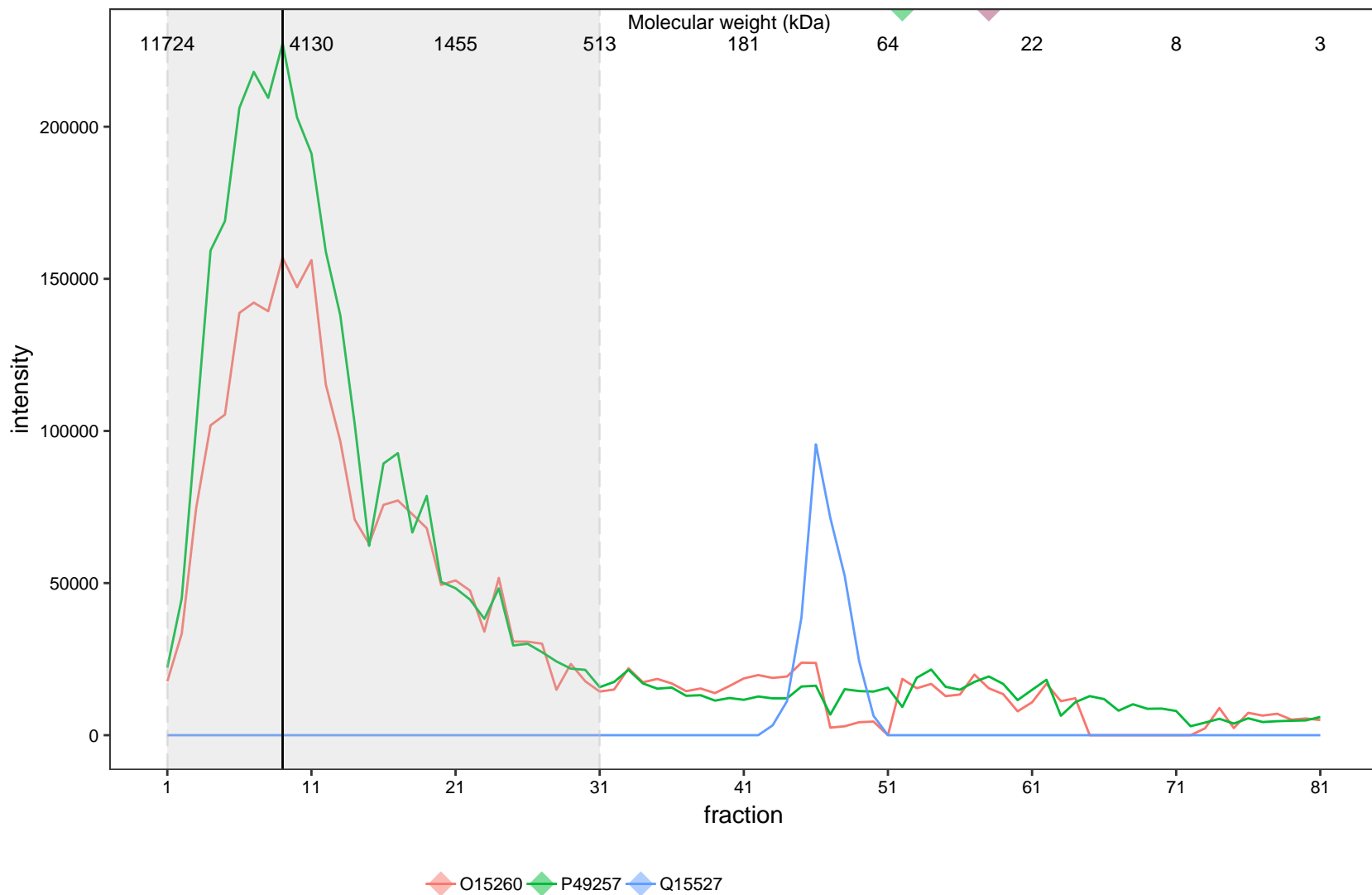

Supplement: Supplementary file 8 — Dataset EV7 [file MSB-15-e8438-s008.zip › feature_plots_string/O15260.pdf]

O15305  
Annotated subunits: 7   Subunits with signal: 5  
Max. coeluting subunits: 2   Max. completeness: 0.29

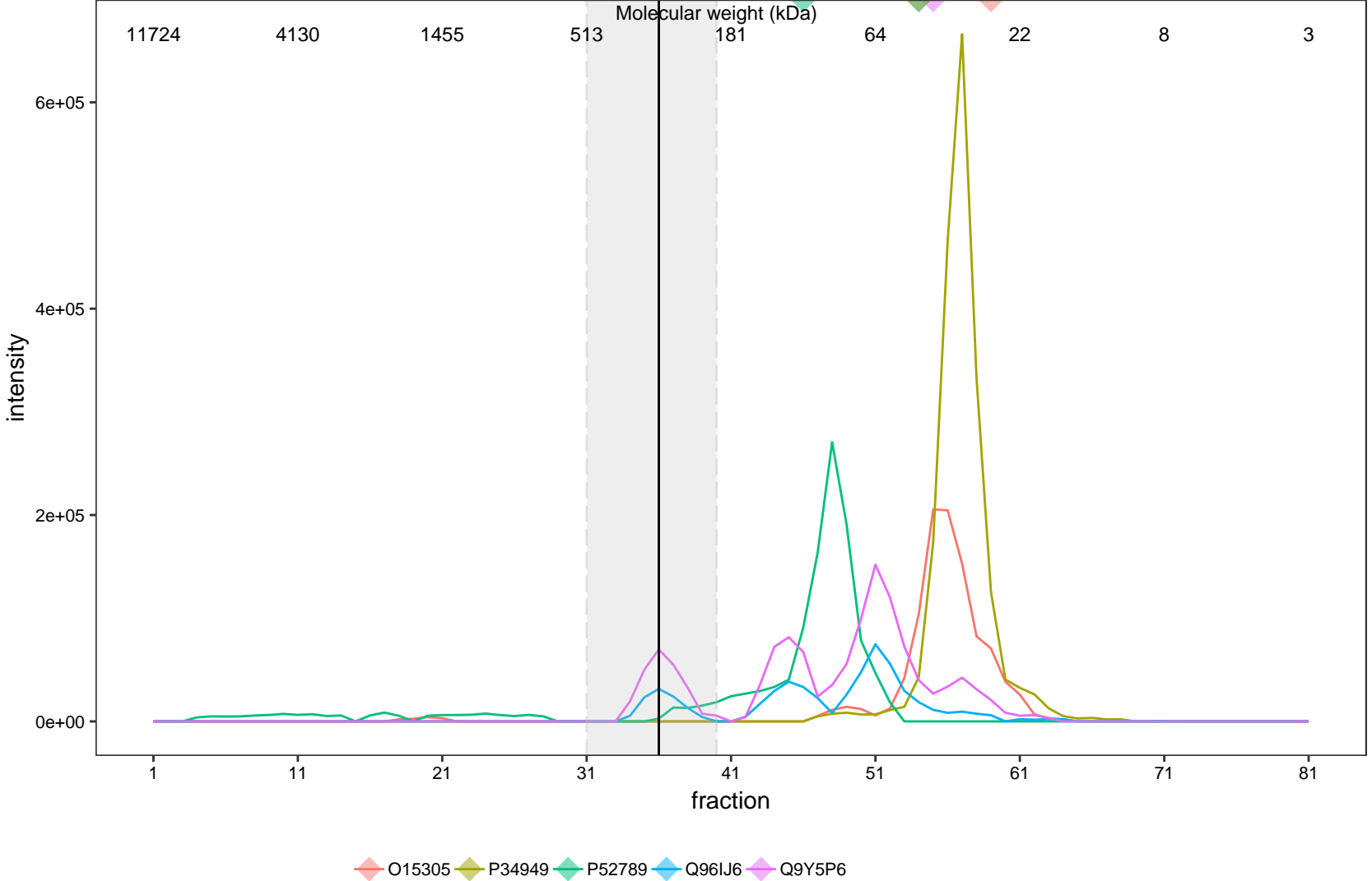

Supplement: Supplementary file 8 — Dataset EV7 [file MSB-15-e8438-s008.zip › feature_plots_string/O15305.pdf]

**O15400**

**Annotated subunits: 29 Subunits with signal: 24**

**Max. coeluting subunits: 15 Max. completeness: 0.52**

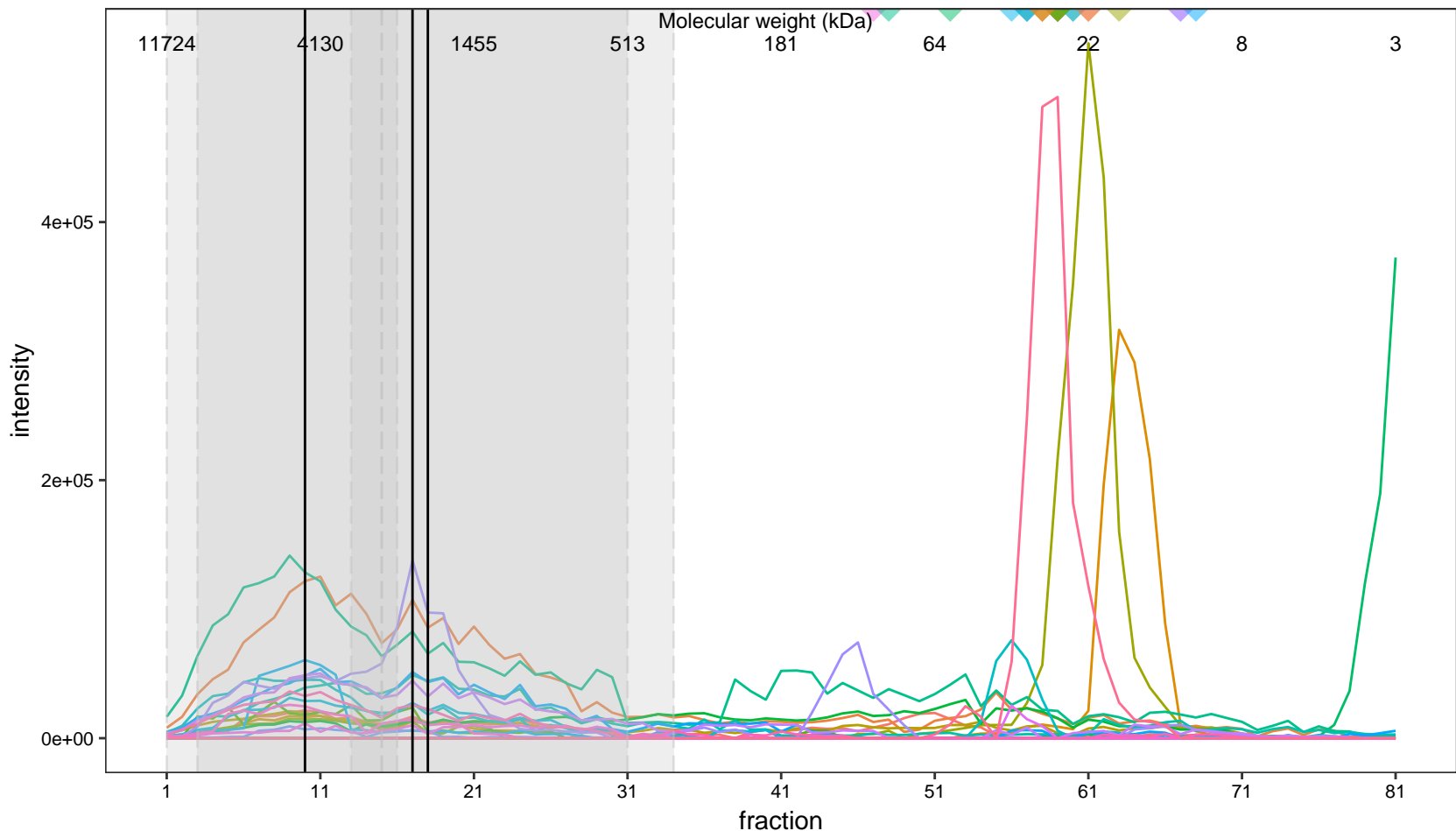

Supplement: Supplementary file 8 — Dataset EV7 [file MSB-15-e8438-s008.zip › feature_plots_string/O15400.pdf]

O15427\_O95907  
Annotated subunits: 4   Subunits with signal: 2  
Max. coeluting subunits: 2   Max. completeness: 0.5

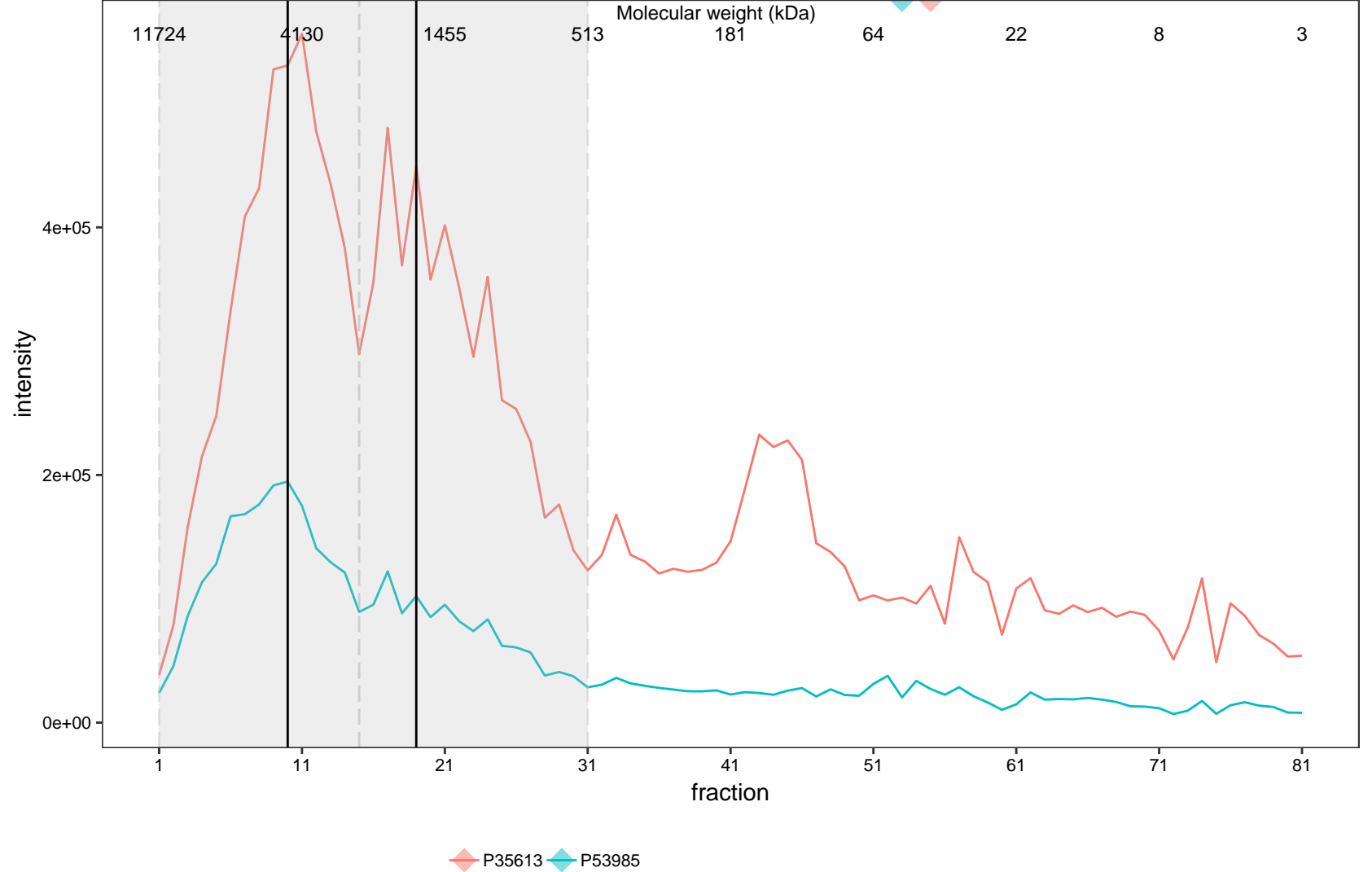

Supplement: Supplementary file 8 — Dataset EV7 [file MSB-15-e8438-s008.zip › feature_plots_string/O15427.pdf]

**O15498**

**Annotated subunits: 35 Subunits with signal: 26**

**Max. coeluting subunits: 15 Max. completeness: 0.43**

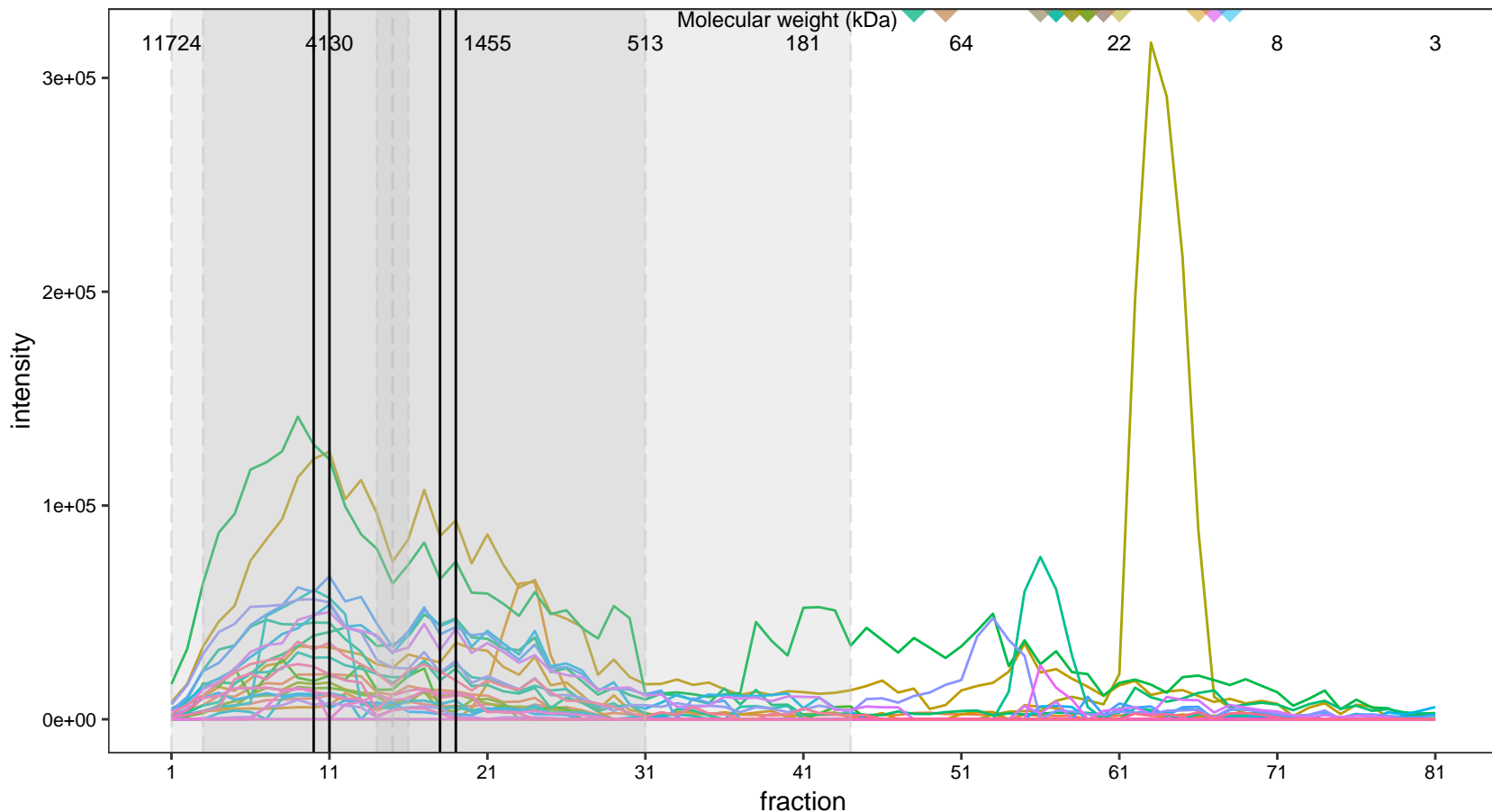

Supplement: Supplementary file 8 — Dataset EV7 [file MSB-15-e8438-s008.zip › feature_plots_string/O15498.pdf]

O15550  
Annotated subunits: 11   Subunits with signal: 7  
Max. coeluting subunits: 4   Max. completeness: 0.36

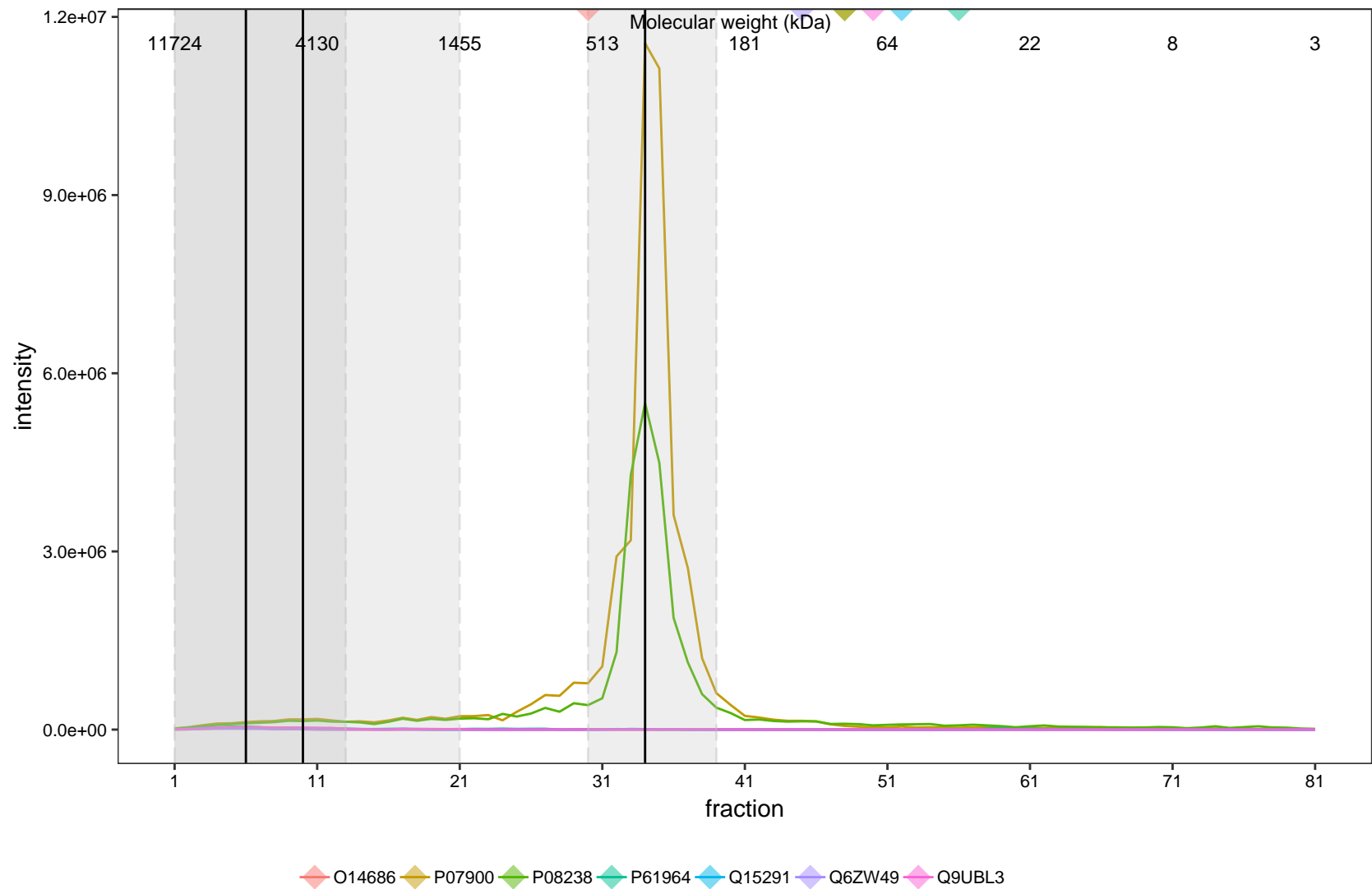

Supplement: Supplementary file 8 — Dataset EV7 [file MSB-15-e8438-s008.zip › feature_plots_string/O15550.pdf]

**O43150**

**Annotated subunits: 15 Subunits with signal: 11**

**Max. coeluting subunits: 2 Max. completeness: 0.13**

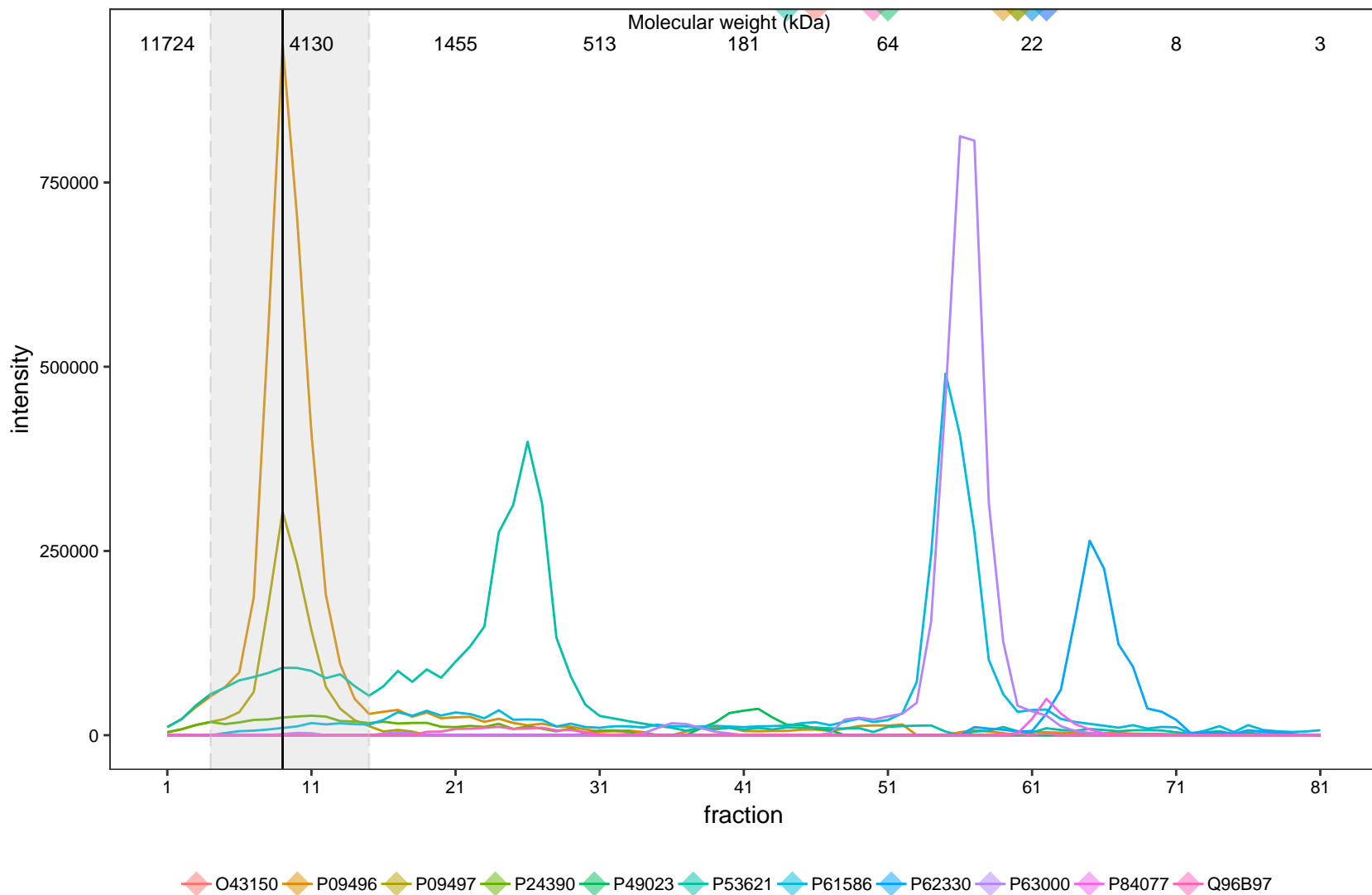

Supplement: Supplementary file 8 — Dataset EV7 [file MSB-15-e8438-s008.zip › feature_plots_string/O43150.pdf]

O43156

Annotated subunits: 18 Subunits with signal: 11

Max. coeluting subunits: 5 Max. completeness: 0.28

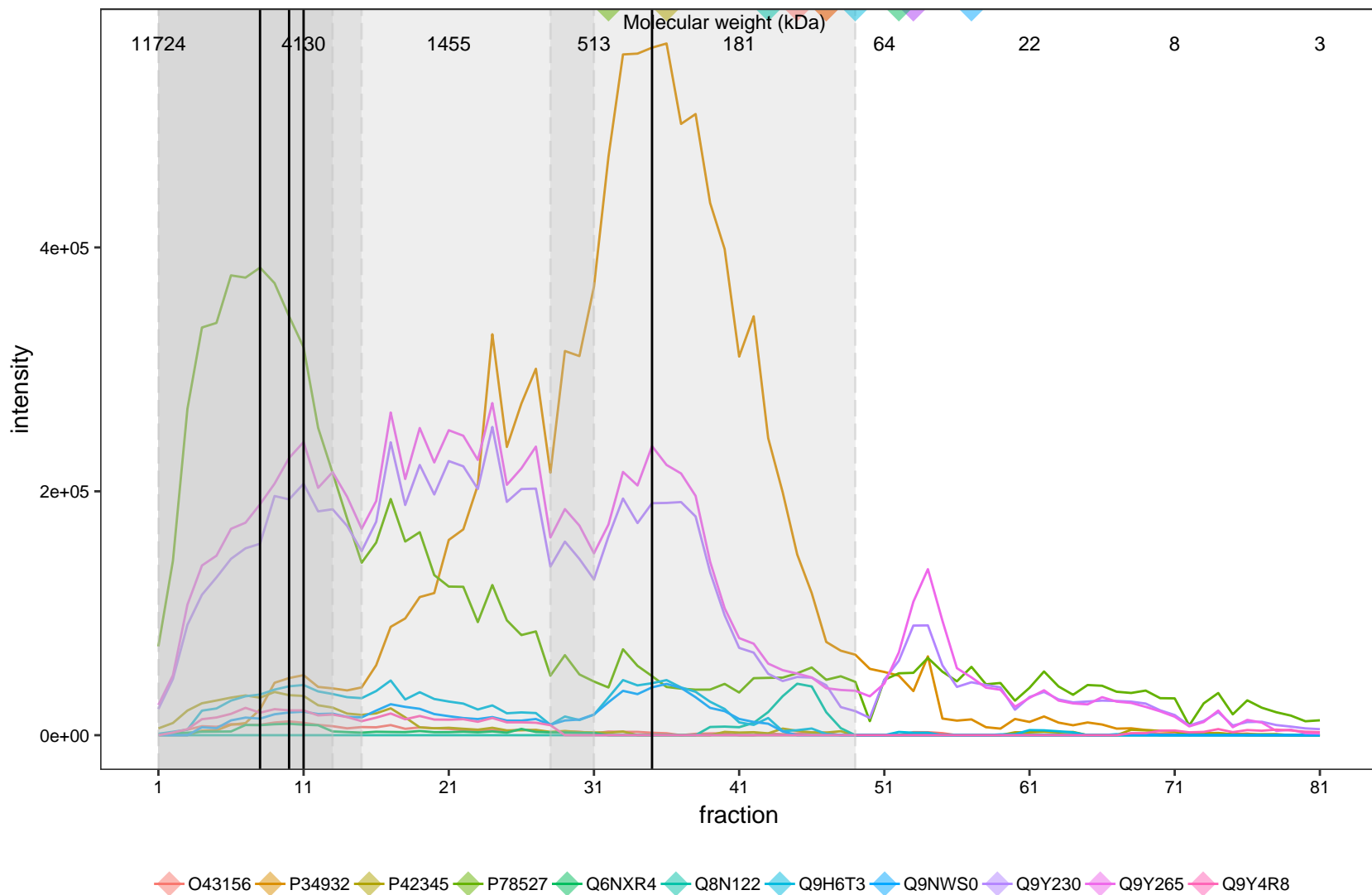

Supplement: Supplementary file 8 — Dataset EV7 [file MSB-15-e8438-s008.zip › feature_plots_string/O43156.pdf]

**O43257**

**Annotated subunits: 11 Subunits with signal: 7**

**Max. coeluting subunits: 2 Max. completeness: 0.18**

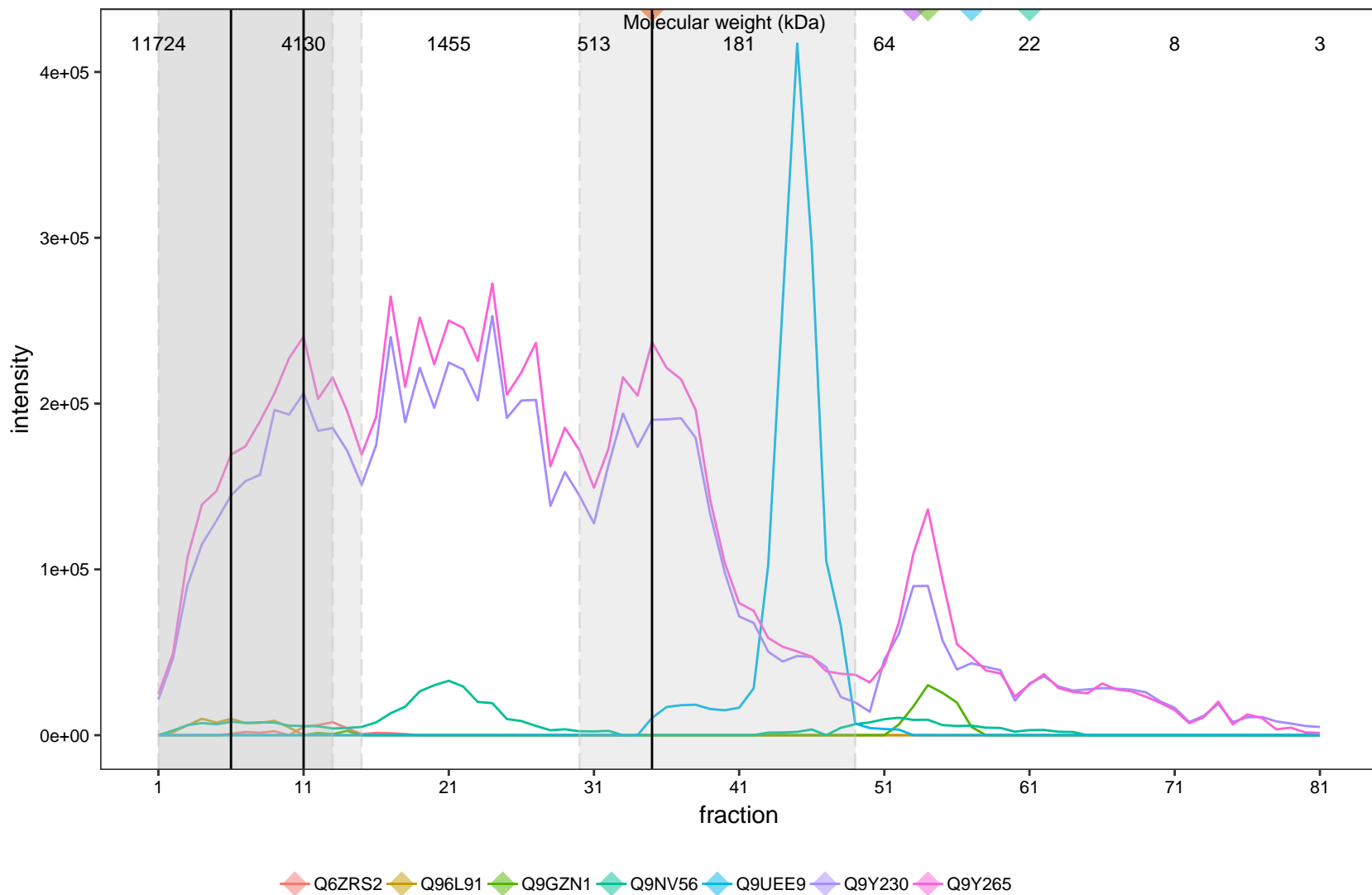

Supplement: Supplementary file 8 — Dataset EV7 [file MSB-15-e8438-s008.zip › feature_plots_string/O43257.pdf]

**O43292**

**Annotated subunits: 5 Subunits with signal: 5**

**Max. coeluting subunits: 5 Max. completeness: 1**

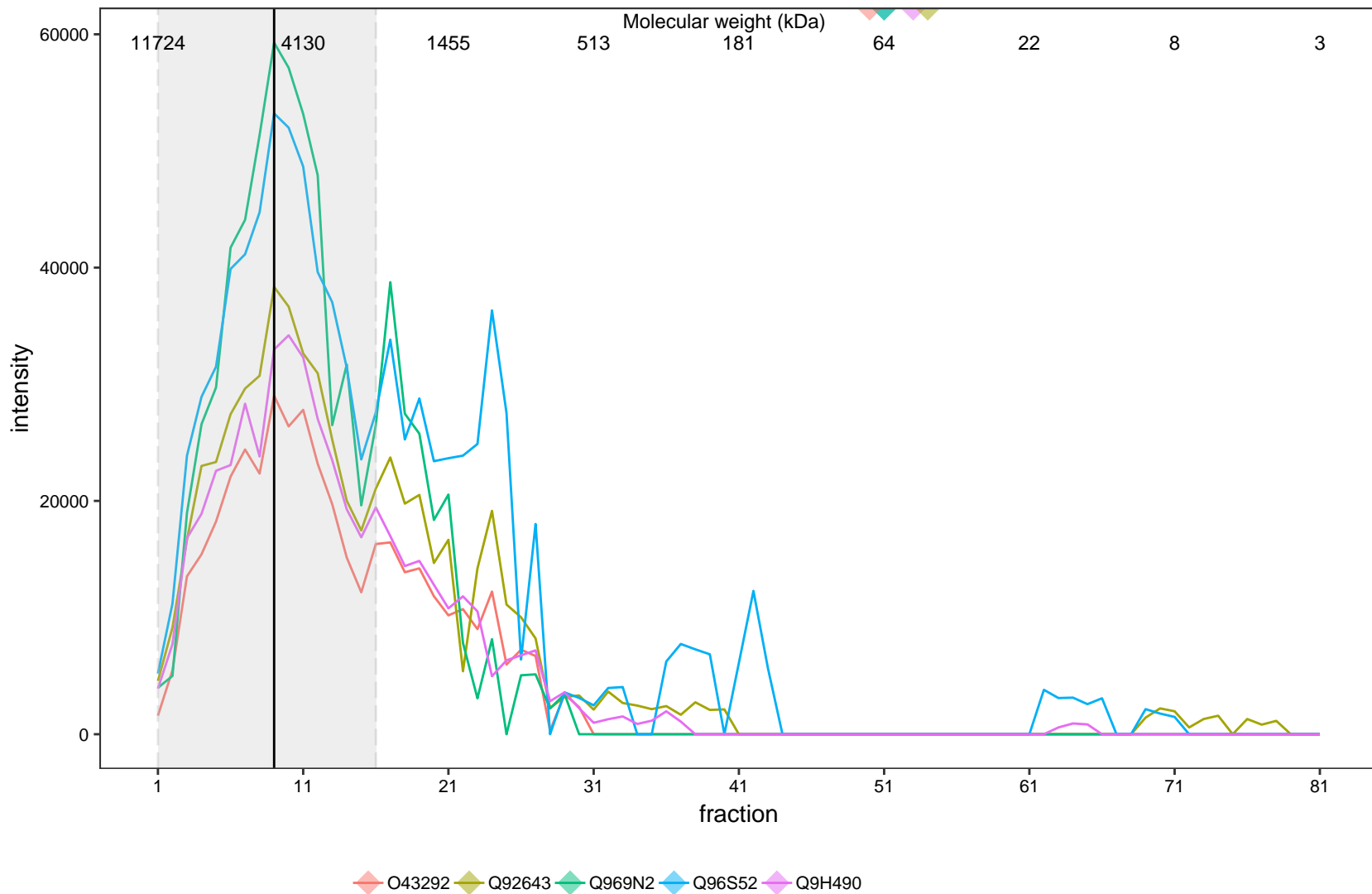

Supplement: Supplementary file 8 — Dataset EV7 [file MSB-15-e8438-s008.zip › feature_plots_string/O43292.pdf]

O43301  
Annotated subunits: 6 Subunits with signal: 6  
Max. coeluting subunits: 3 Max. completeness: 0.5

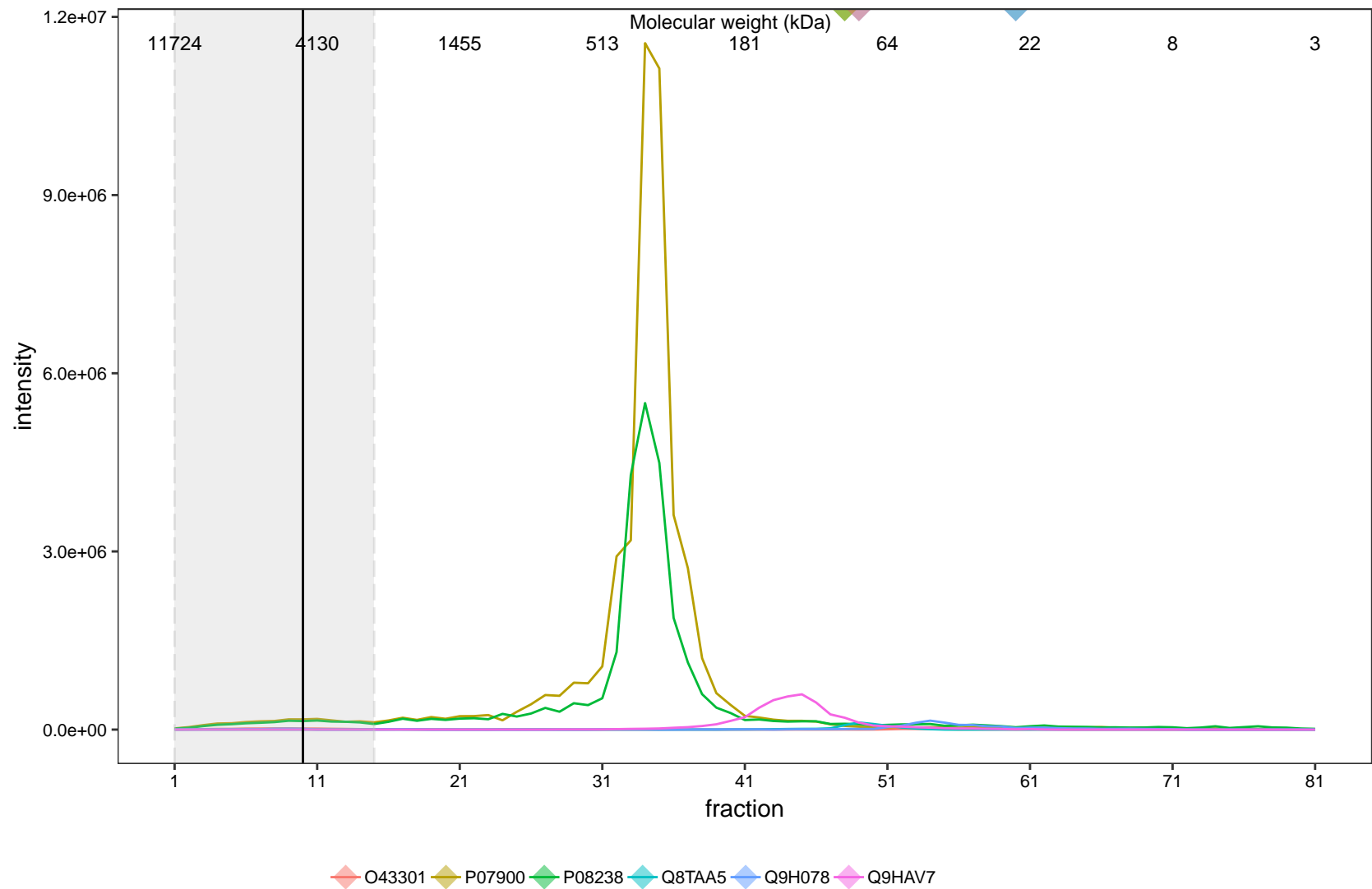

Supplement: Supplementary file 8 — Dataset EV7 [file MSB-15-e8438-s008.zip › feature_plots_string/O43301.pdf]

O43347  
Annotated subunits: 5   Subunits with signal: 3  
Max. coeluting subunits: 3   Max. completeness: 0.6

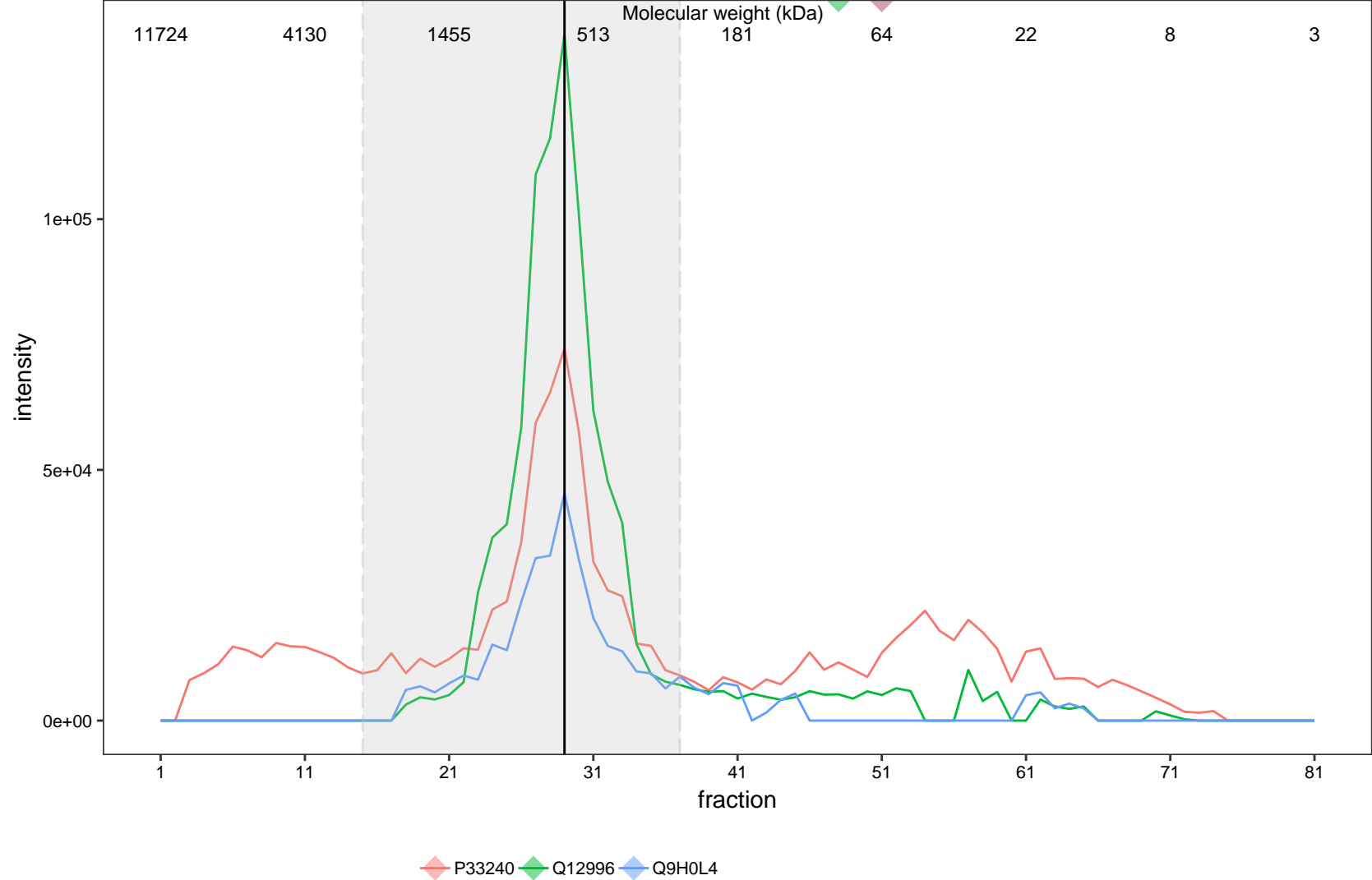

Supplement: Supplementary file 8 — Dataset EV7 [file MSB-15-e8438-s008.zip › feature_plots_string/O43347.pdf]

O43396  
Annotated subunits: 11   Subunits with signal: 10  
Max. coeluting subunits: 2   Max. completeness: 0.18

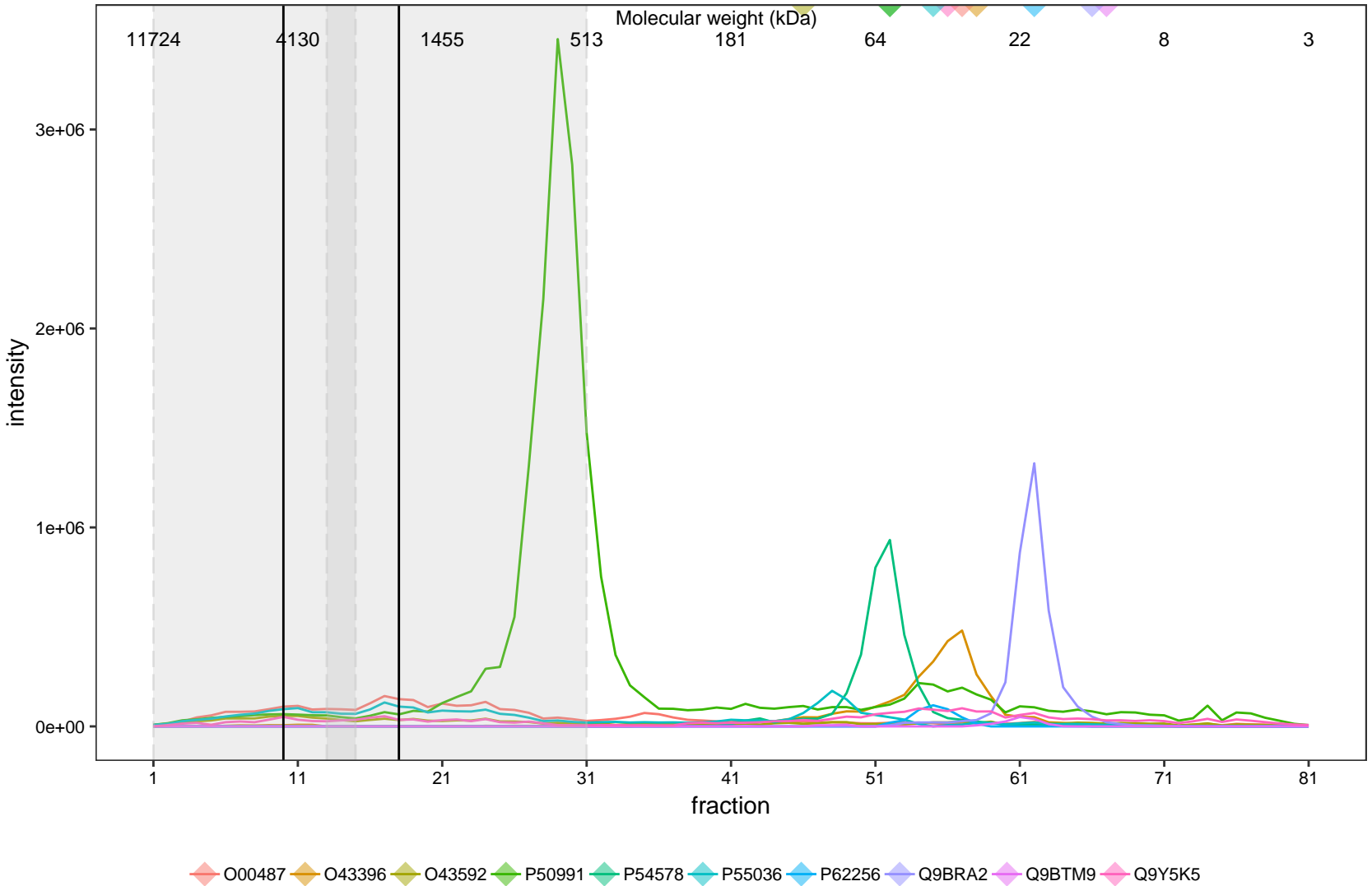

Supplement: Supplementary file 8 — Dataset EV7 [file MSB-15-e8438-s008.zip › feature_plots_string/O43396.pdf]

O43402  
Annotated subunits: 11   Subunits with signal: 11  
Max. coeluting subunits: 11   Max. completeness: 1

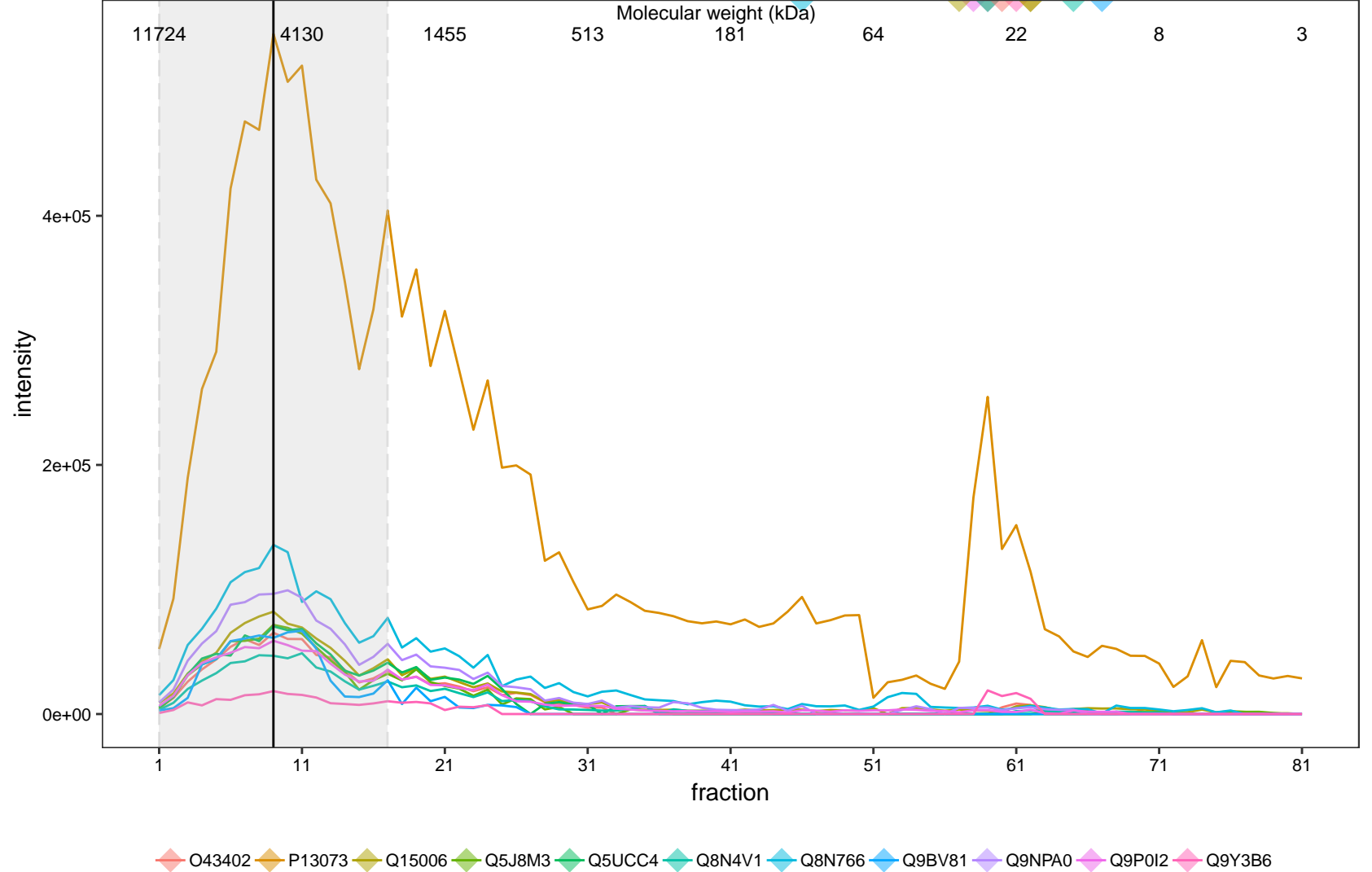

Supplement: Supplementary file 8 — Dataset EV7 [file MSB-15-e8438-s008.zip › feature_plots_string/O43402.pdf]

**O43497**

**Annotated subunits: 14 Subunits with signal: 2**

**Max. coeluting subunits: 2 Max. completeness: 0.14**

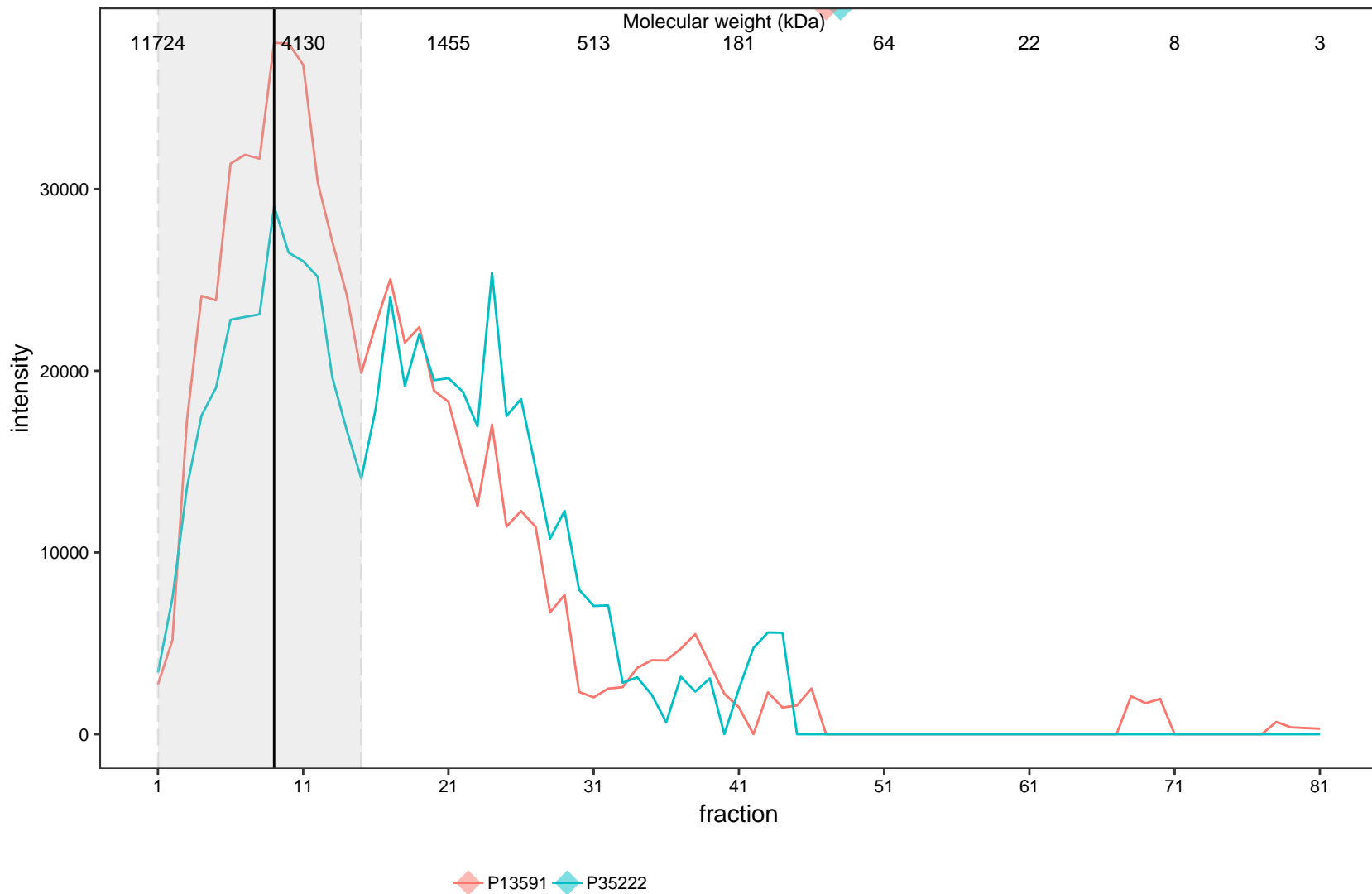

Supplement: Supplementary file 8 — Dataset EV7 [file MSB-15-e8438-s008.zip › feature_plots_string/O43497.pdf]

**O43525**

**Annotated subunits: 52 Subunits with signal: 6**

**Max. coeluting subunits: 4 Max. completeness: 0.08**

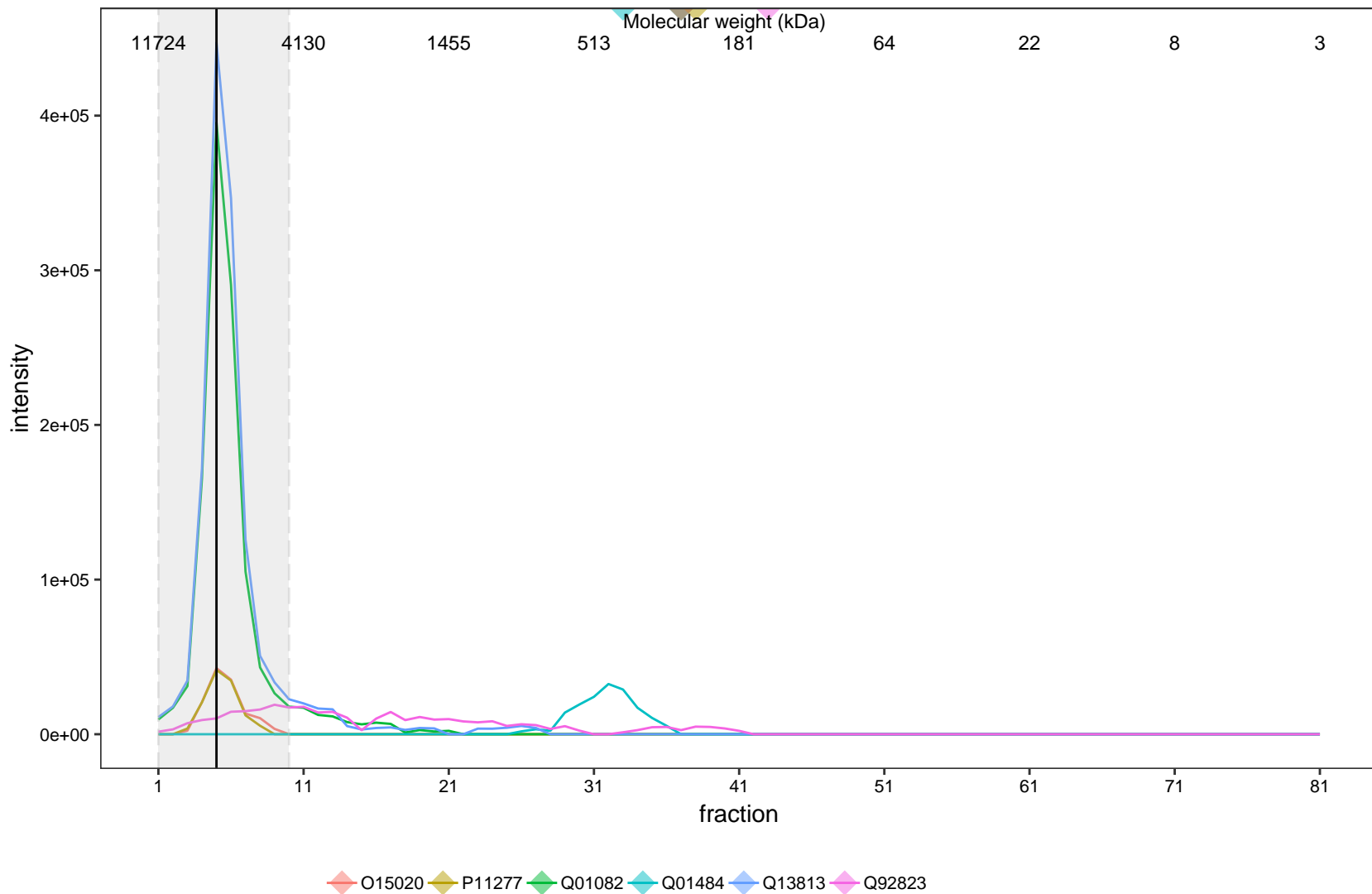

Supplement: Supplementary file 8 — Dataset EV7 [file MSB-15-e8438-s008.zip › feature_plots_string/O43525.pdf]

O43526  
Annotated subunits: 56   Subunits with signal: 8  
Max. coeluting subunits: 4   Max. completeness: 0.07

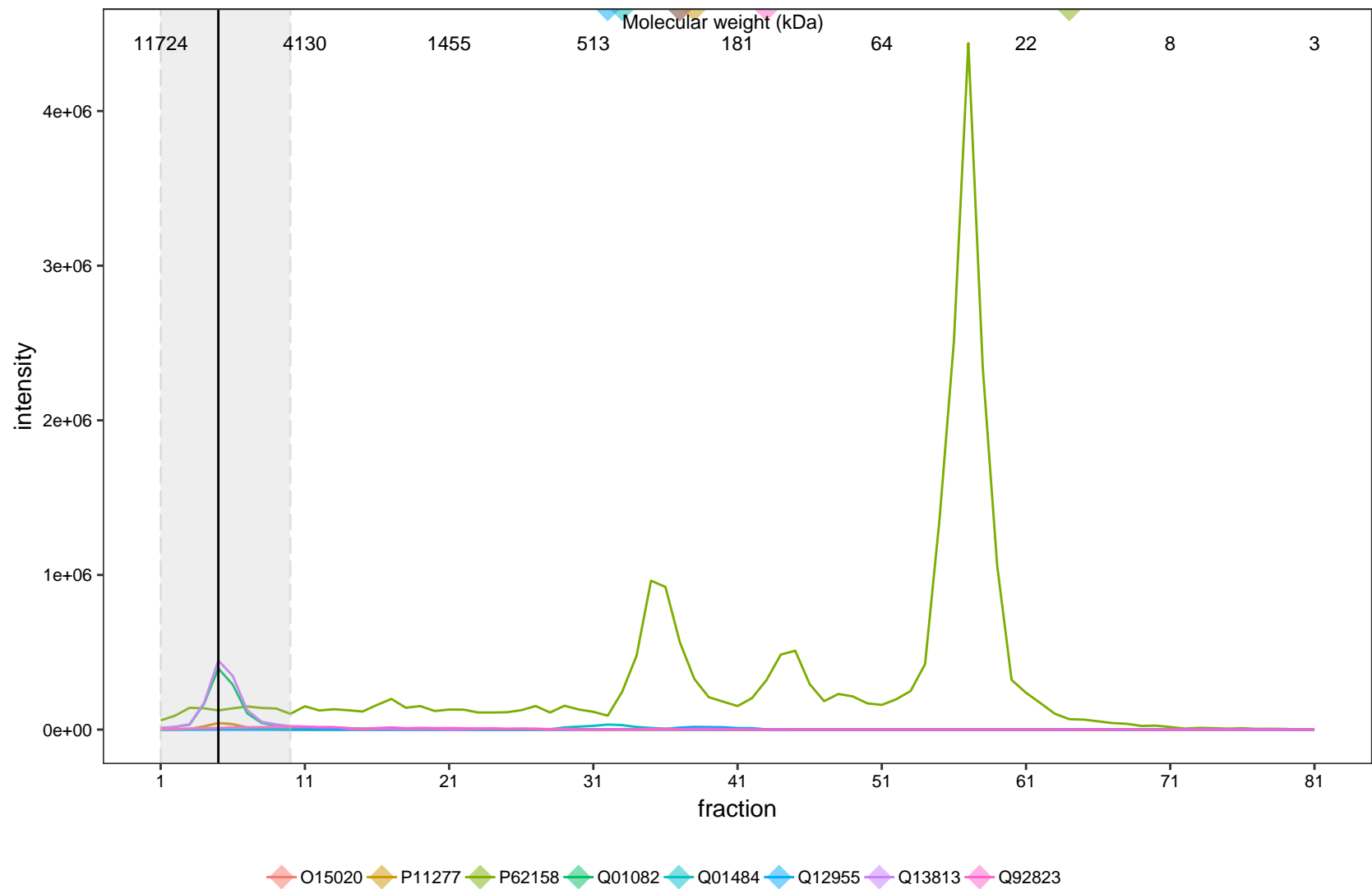

Supplement: Supplementary file 8 — Dataset EV7 [file MSB-15-e8438-s008.zip › feature_plots_string/O43526.pdf]

O43572

Annotated subunits: 10 Subunits with signal: 5

Max. coeluting subunits: 2 Max. completeness: 0.2

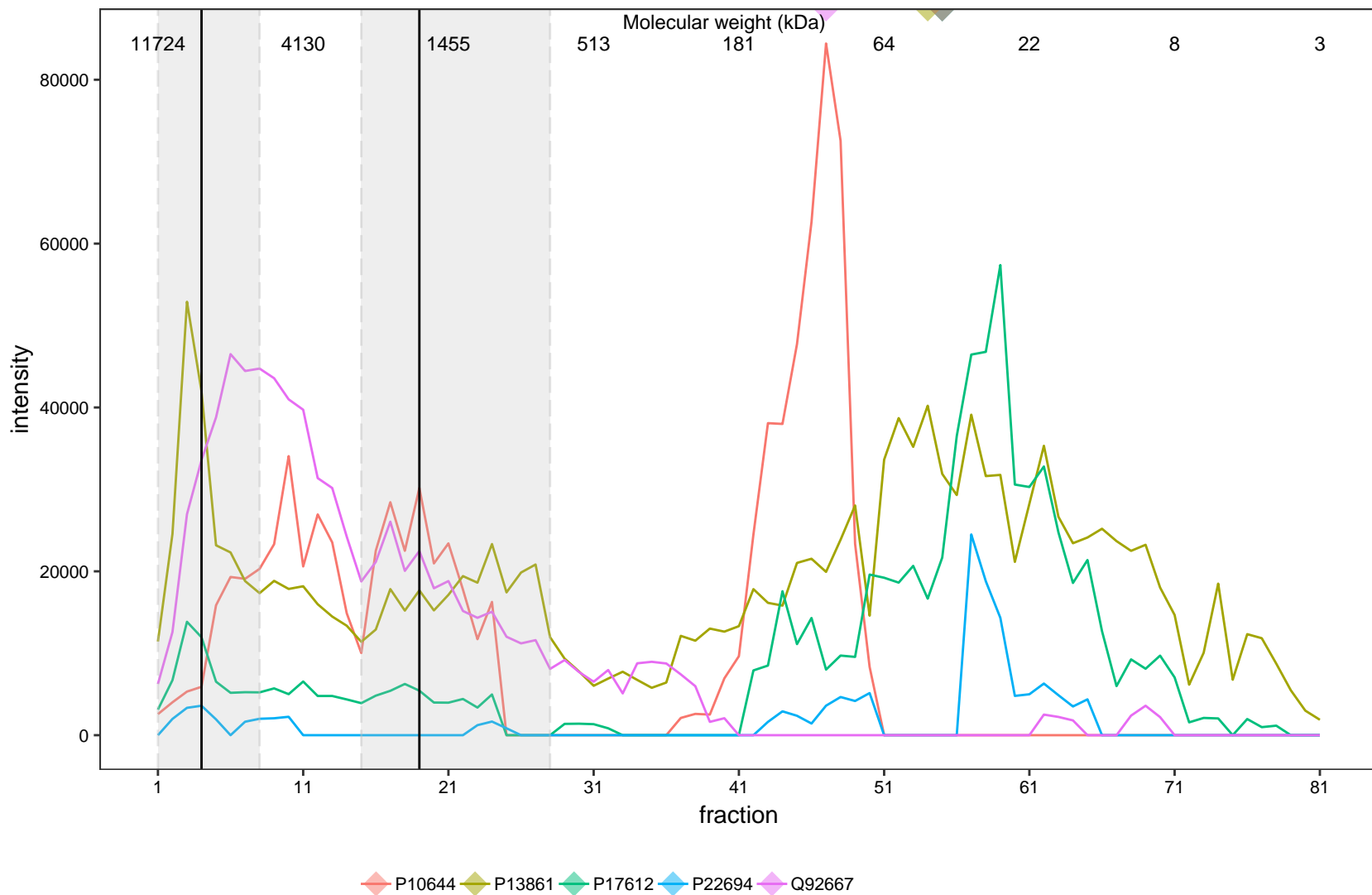

Supplement: Supplementary file 8 — Dataset EV7 [file MSB-15-e8438-s008.zip › feature_plots_string/O43572.pdf]

O43583  
Annotated subunits: 3   Subunits with signal: 2  
Max. coeluting subunits: 2   Max. completeness: 0.67

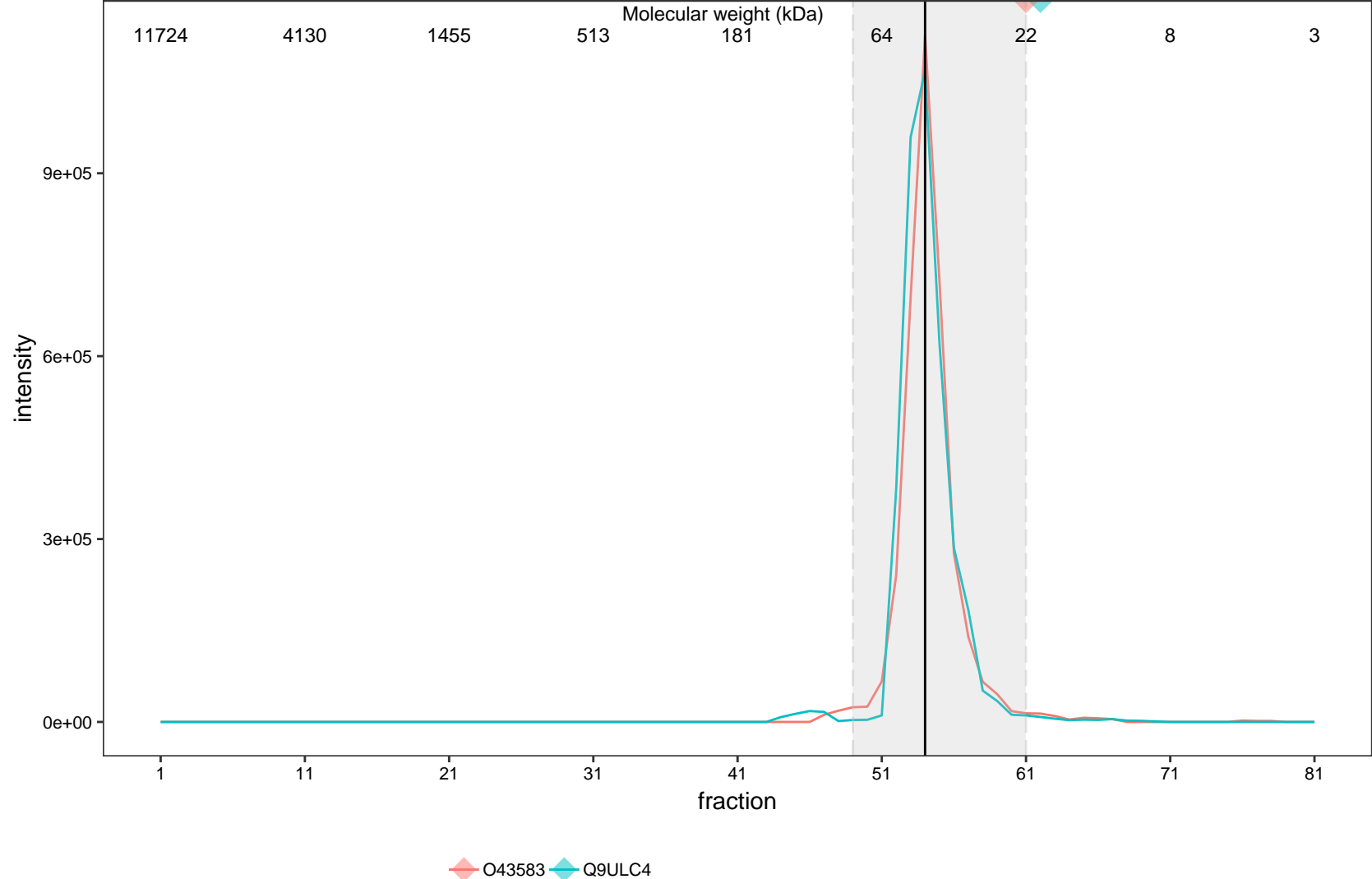

Supplement: Supplementary file 8 — Dataset EV7 [file MSB-15-e8438-s008.zip › feature_plots_string/O43583.pdf]

O43592  
Annotated subunits: 7 Subunits with signal: 7  
Max. coeluting subunits: 2 Max. completeness: 0.29

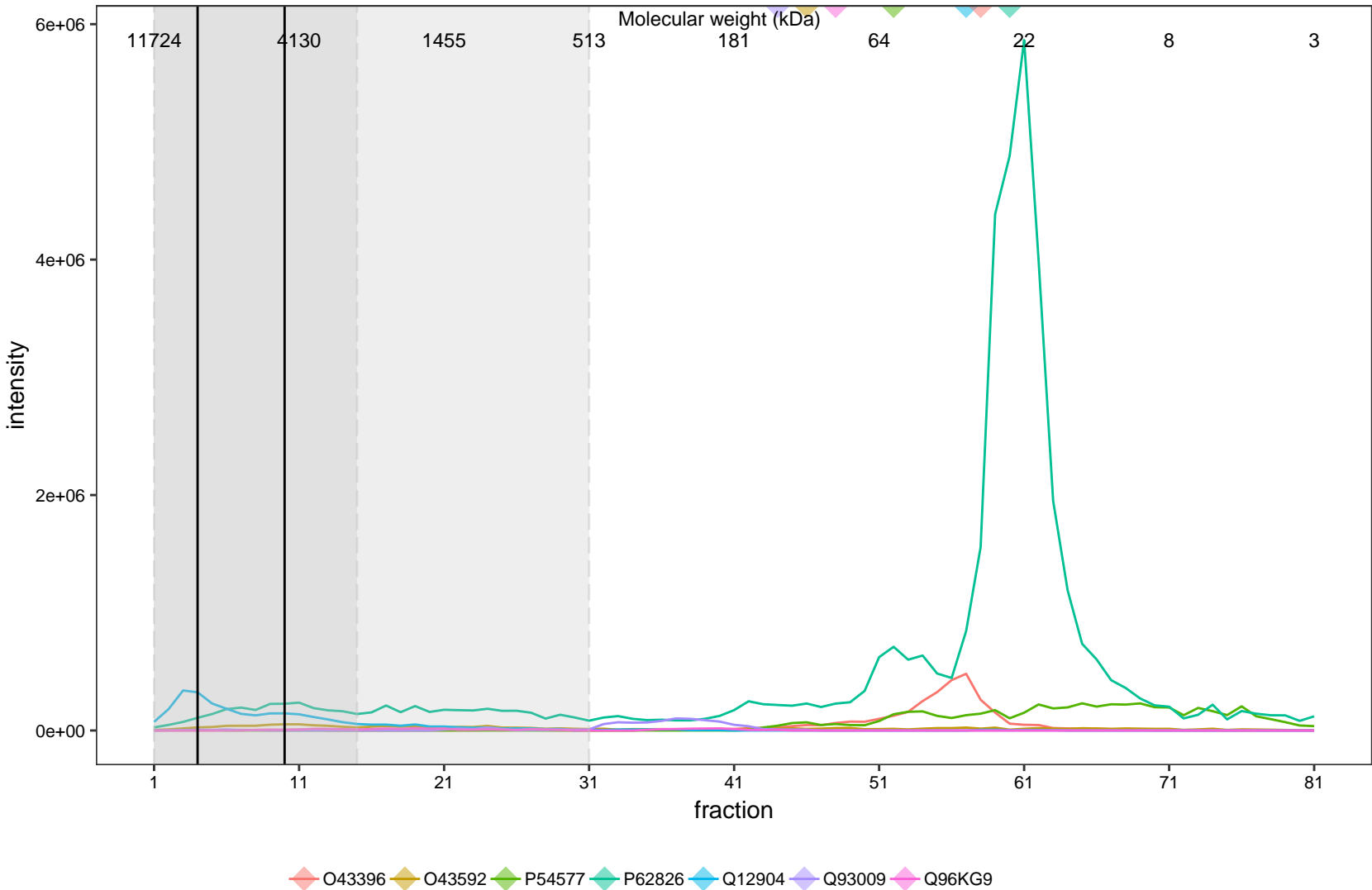

Supplement: Supplementary file 8 — Dataset EV7 [file MSB-15-e8438-s008.zip › feature_plots_string/O43592.pdf]

**O43617**

**Annotated subunits: 13 Subunits with signal: 8**

**Max. coeluting subunits: 7 Max. completeness: 0.54**

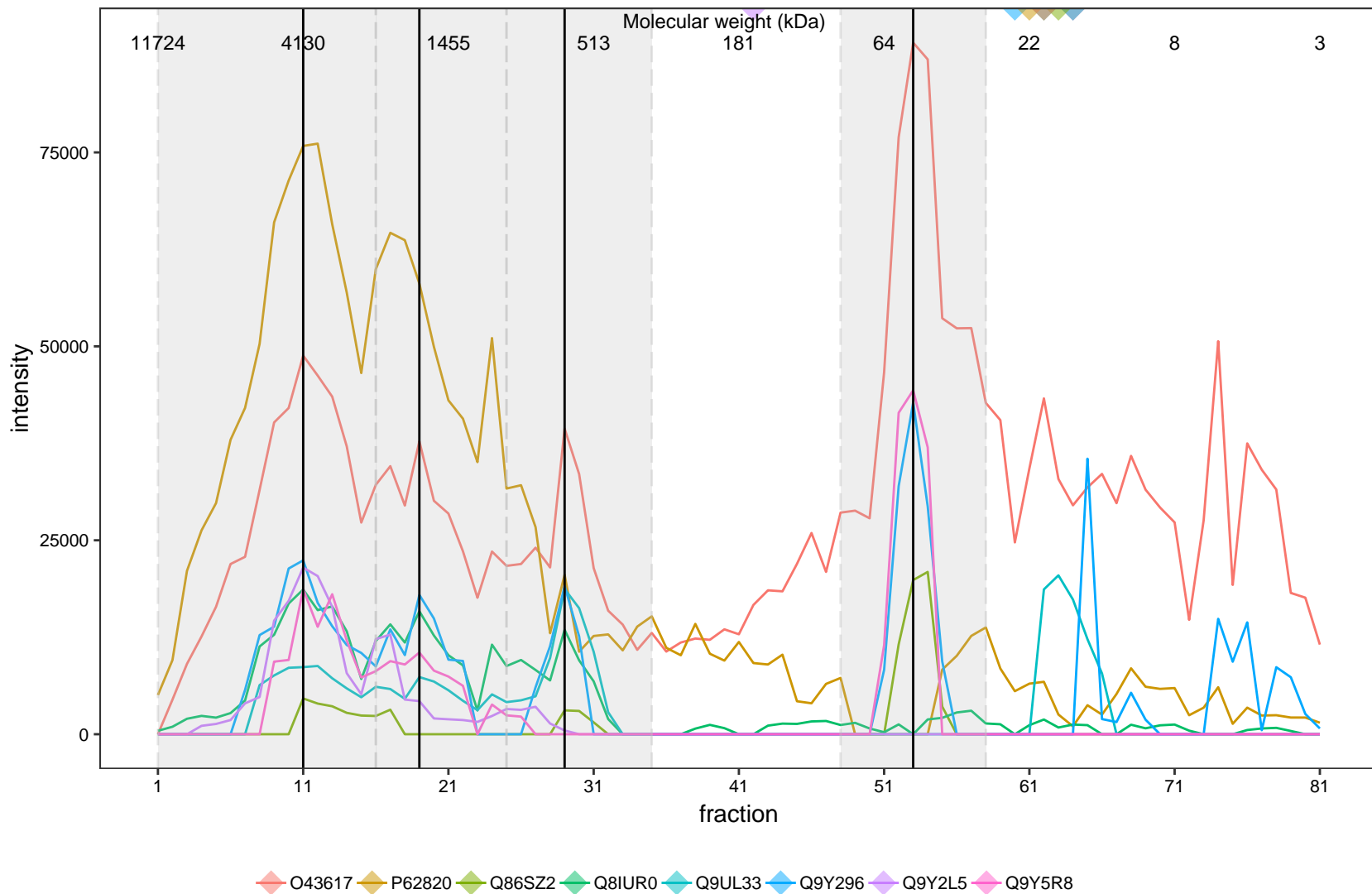

Supplement: Supplementary file 8 — Dataset EV7 [file MSB-15-e8438-s008.zip › feature_plots_string/O43617.pdf]

O43670  
Annotated subunits: 3 Subunits with signal: 3  
Max. coeluting subunits: 2 Max. completeness: 0.67

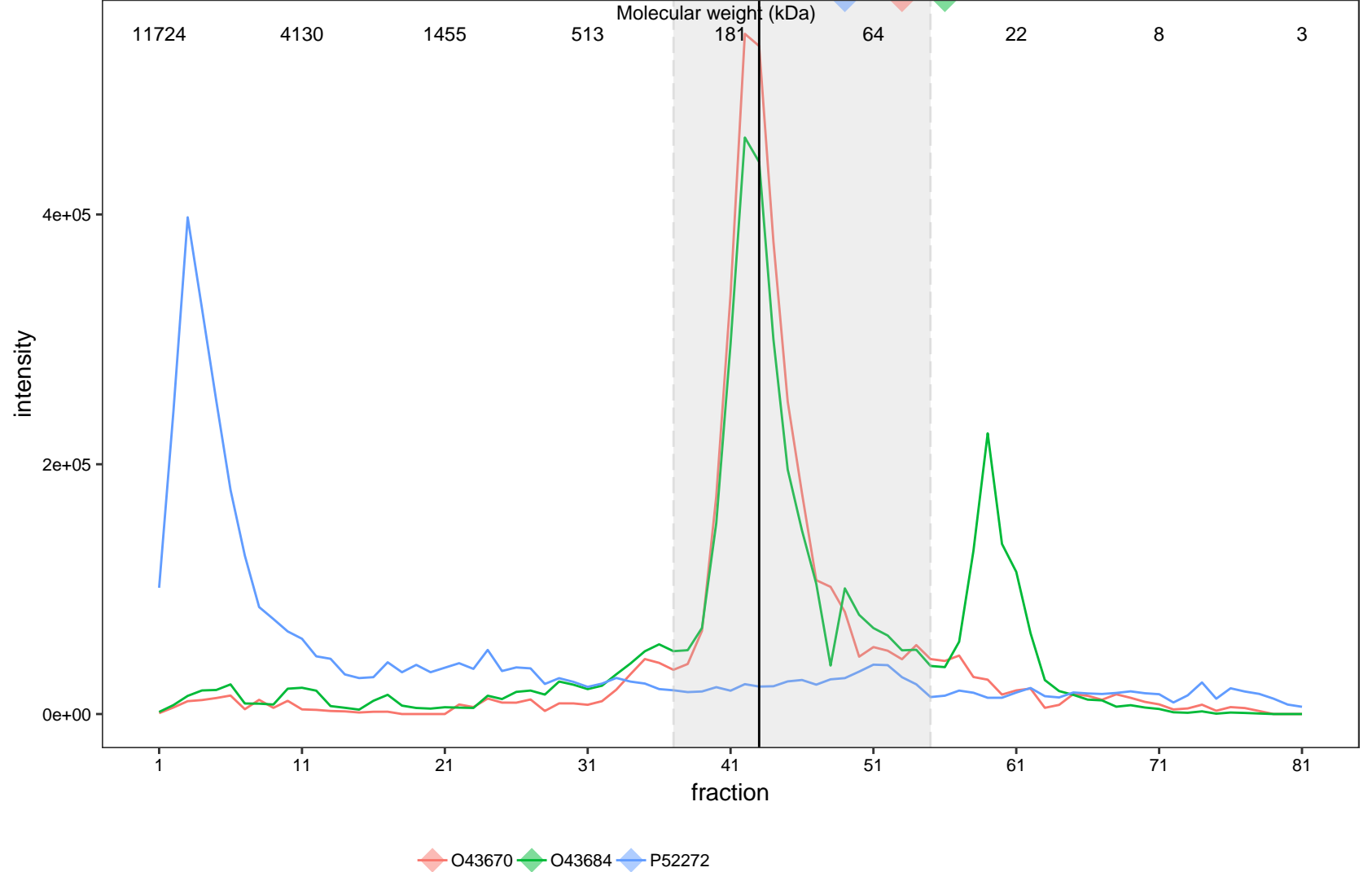

Supplement: Supplementary file 8 — Dataset EV7 [file MSB-15-e8438-s008.zip › feature_plots_string/O43670.pdf]

**O43681**

**Annotated subunits: 8 Subunits with signal: 7**

**Max. coeluting subunits: 6 Max. completeness: 0.75**

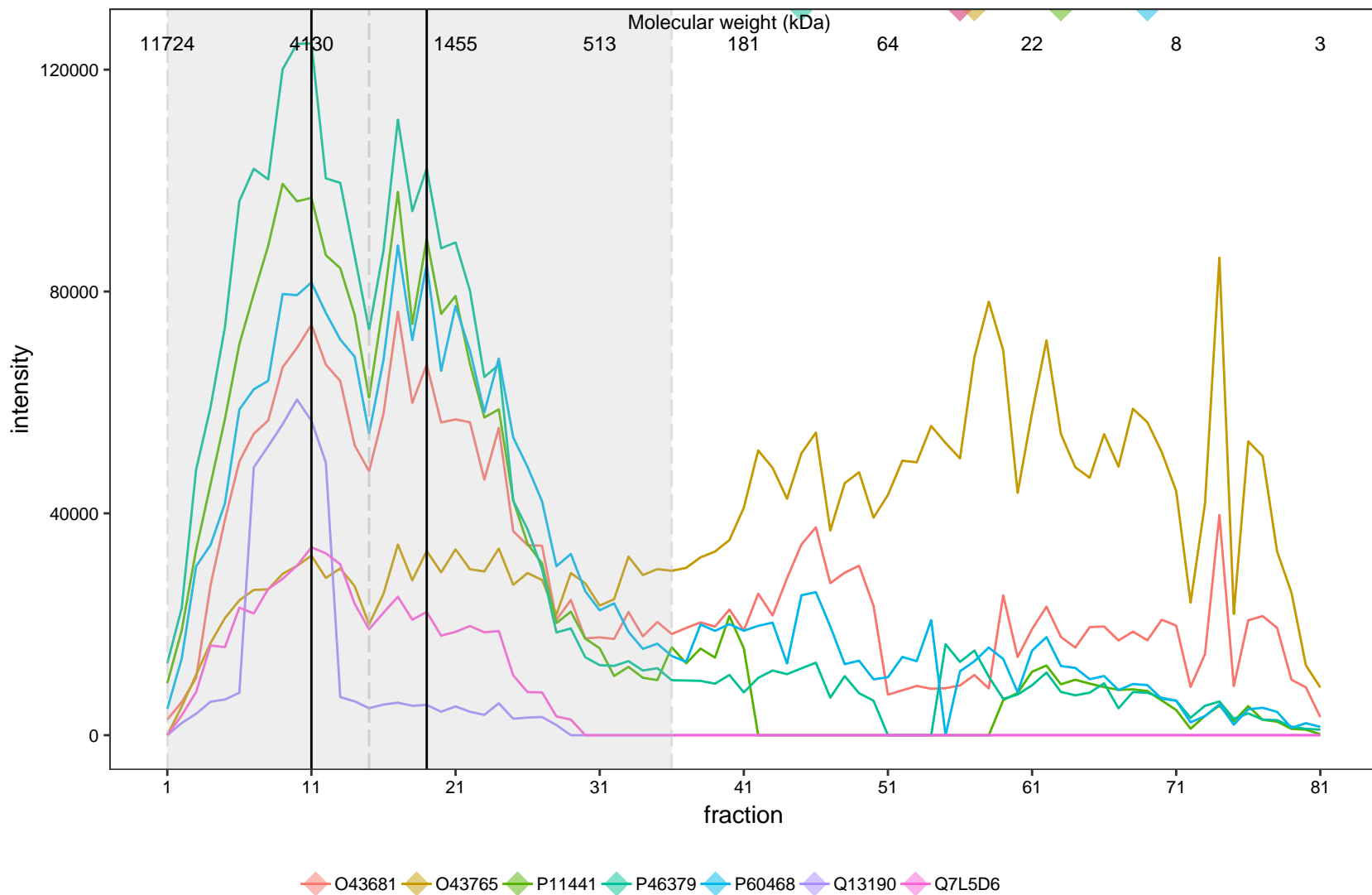

Supplement: Supplementary file 8 — Dataset EV7 [file MSB-15-e8438-s008.zip › feature_plots_string/O43681.pdf]

O43765  
Annotated subunits: 11   Subunits with signal: 9  
Max. coeluting subunits: 7   Max. completeness: 0.64

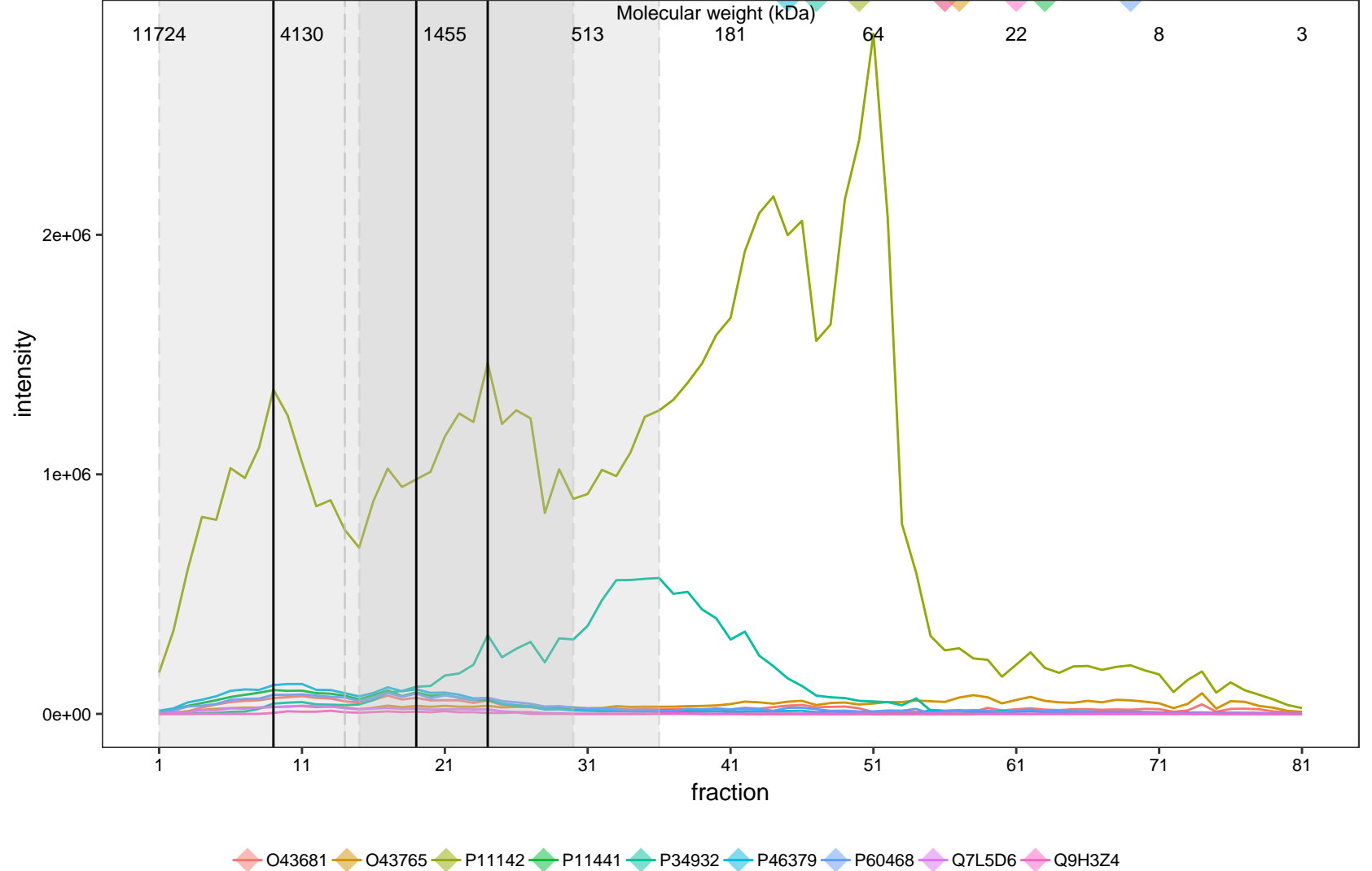

Supplement: Supplementary file 8 — Dataset EV7 [file MSB-15-e8438-s008.zip › feature_plots_string/O43765.pdf]

**O43776**

**Annotated subunits: 15 Subunits with signal: 13**

**Max. coeluting subunits: 6 Max. completeness: 0.4**

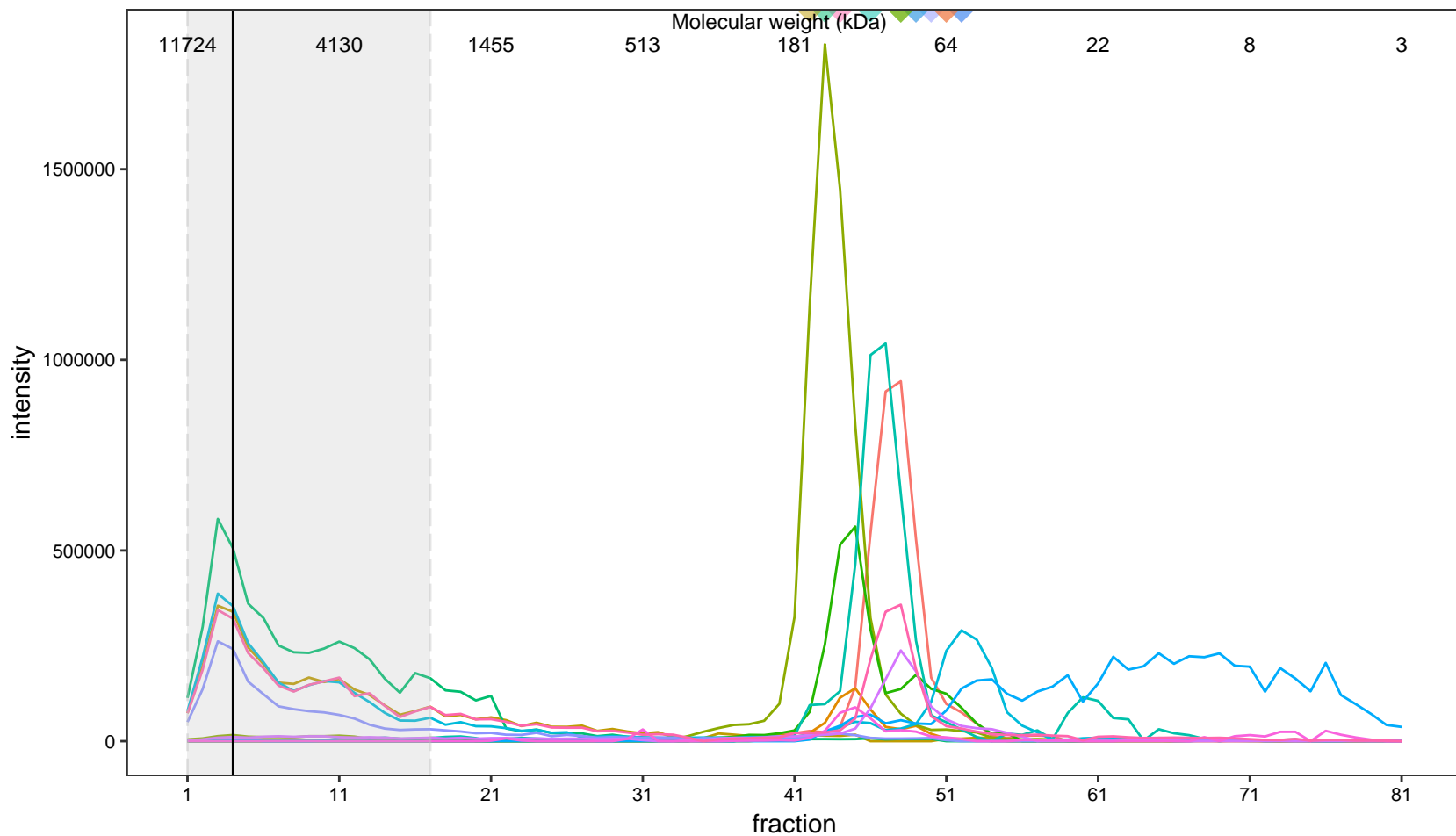

Supplement: Supplementary file 8 — Dataset EV7 [file MSB-15-e8438-s008.zip › feature_plots_string/O43776.pdf]

**O43808**

**Annotated subunits: 5 Subunits with signal: 4**

**Max. coeluting subunits: 3 Max. completeness: 0.6**

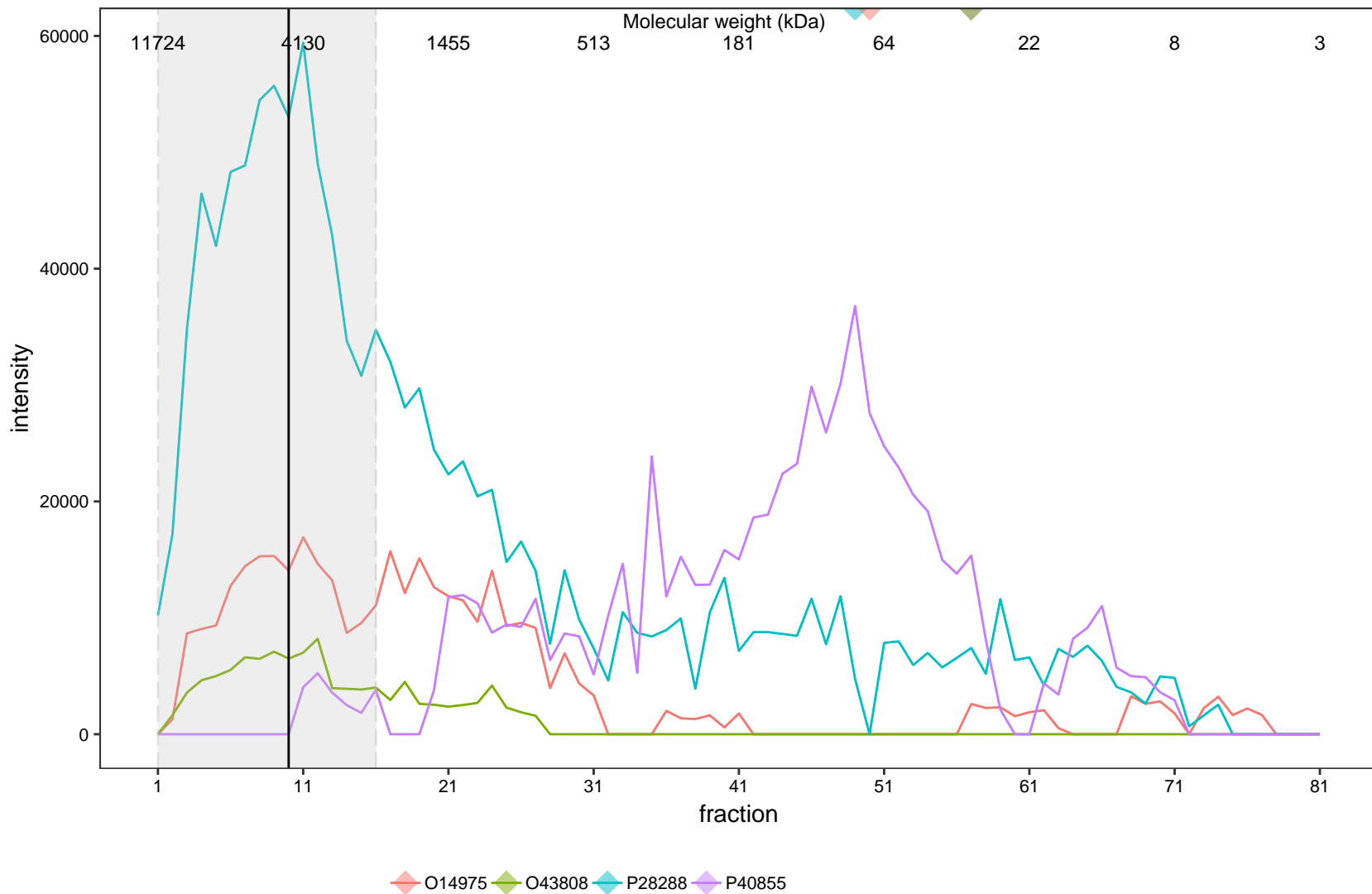

Supplement: Supplementary file 8 — Dataset EV7 [file MSB-15-e8438-s008.zip › feature_plots_string/O43808.pdf]

**O60220**

**Annotated subunits: 6 Subunits with signal: 5**

**Max. coeluting subunits: 3 Max. completeness: 0.5**

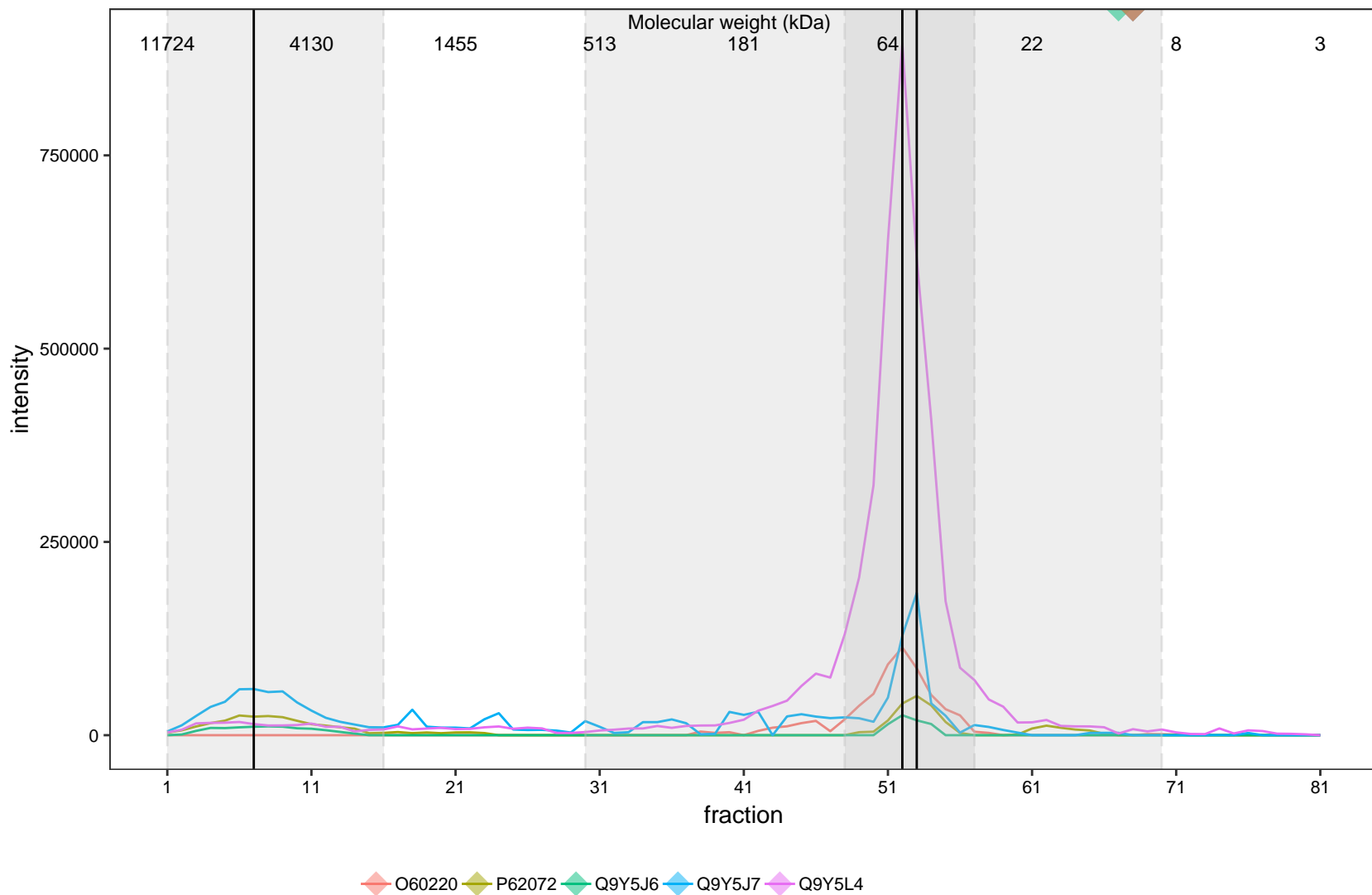

Supplement: Supplementary file 8 — Dataset EV7 [file MSB-15-e8438-s008.zip › feature_plots_string/O60220.pdf]

O60271

Annotated subunits: 21 Subunits with signal: 12

Max. coeluting subunits: 4 Max. completeness: 0.19

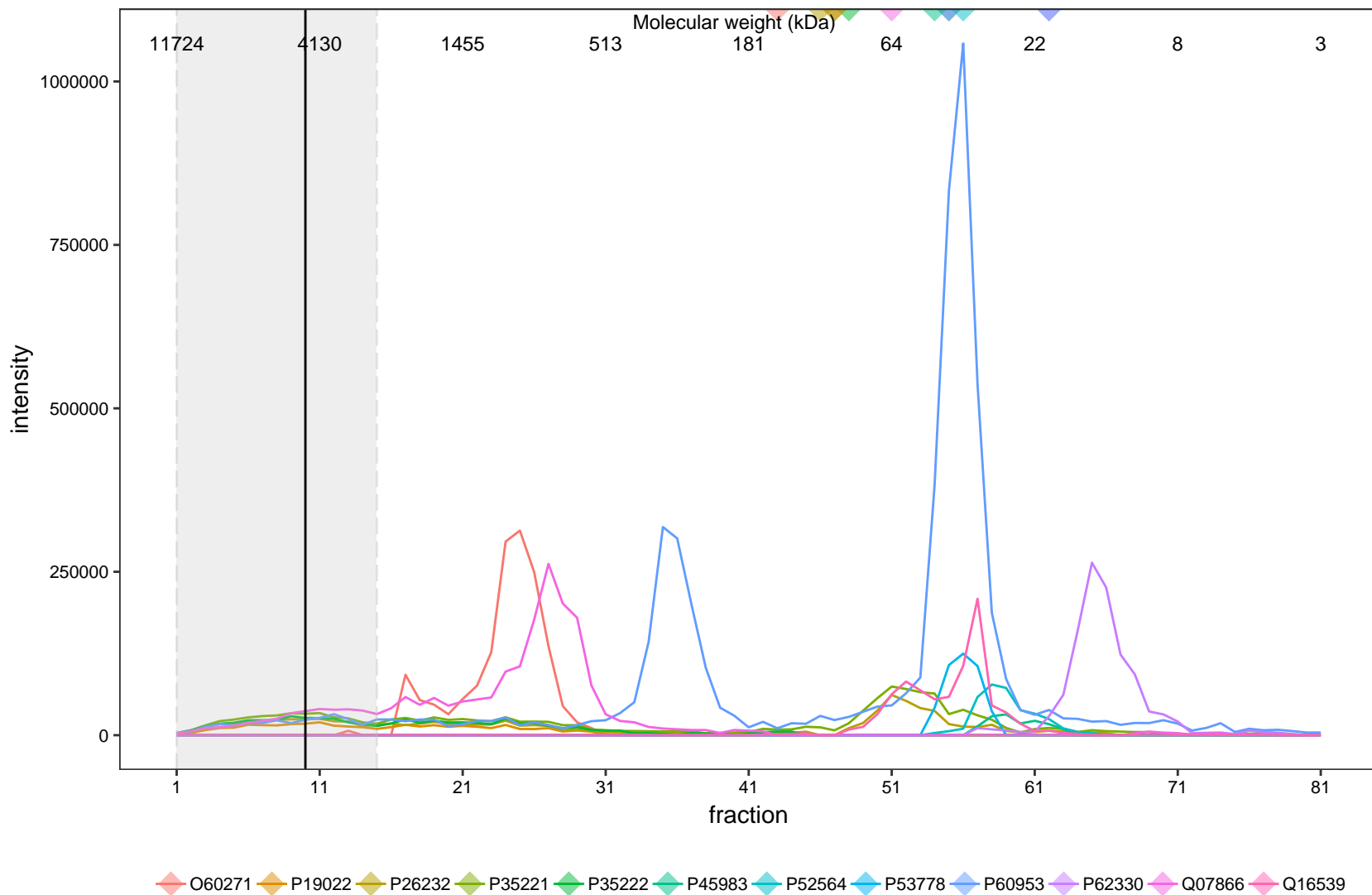

Supplement: Supplementary file 8 — Dataset EV7 [file MSB-15-e8438-s008.zip › feature_plots_string/O60271.pdf]

**O60282**  
Annotated subunits: 5   Subunits with signal: 3  
Max. coeluting subunits: 3   Max. completeness: 0.6

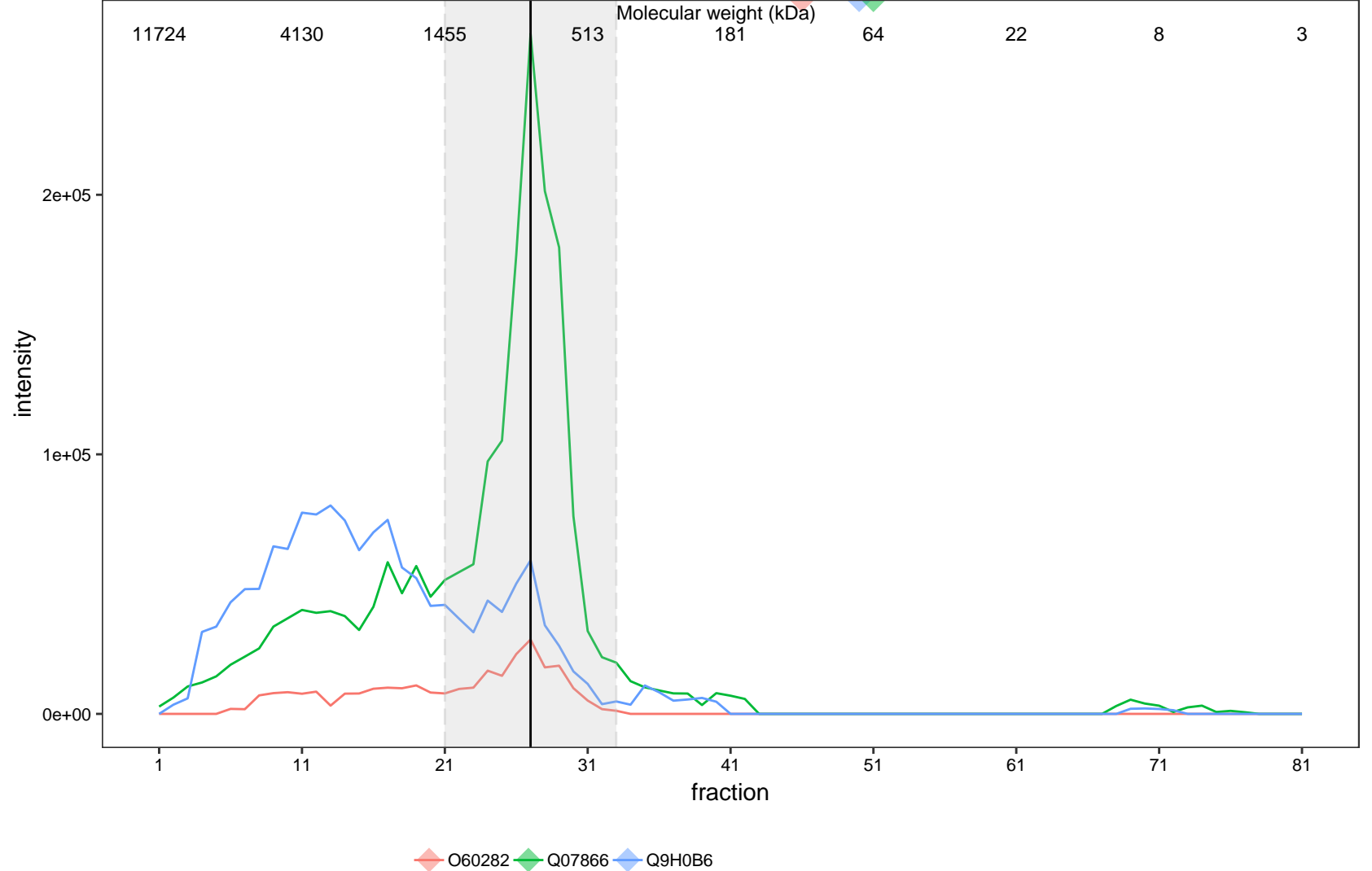

Supplement: Supplementary file 8 — Dataset EV7 [file MSB-15-e8438-s008.zip › feature_plots_string/O60282.pdf]

**O60285**

**Annotated subunits: 4 Subunits with signal: 3**

**Max. coeluting subunits: 2 Max. completeness: 0.5**

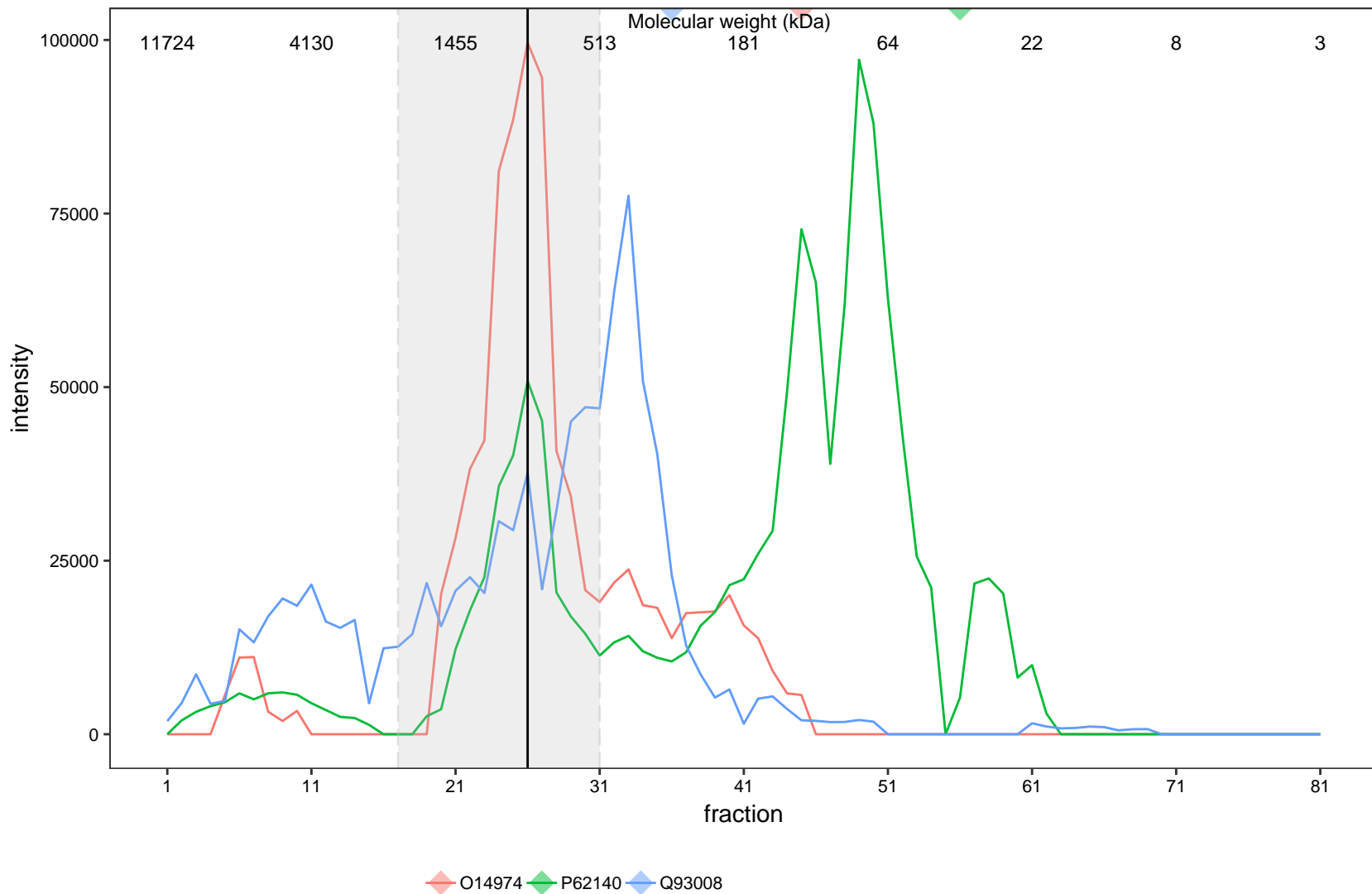

Supplement: Supplementary file 8 — Dataset EV7 [file MSB-15-e8438-s008.zip › feature_plots_string/O60285.pdf]

O60306  
Annotated subunits: 6   Subunits with signal: 4  
Max. coeluting subunits: 2   Max. completeness: 0.33

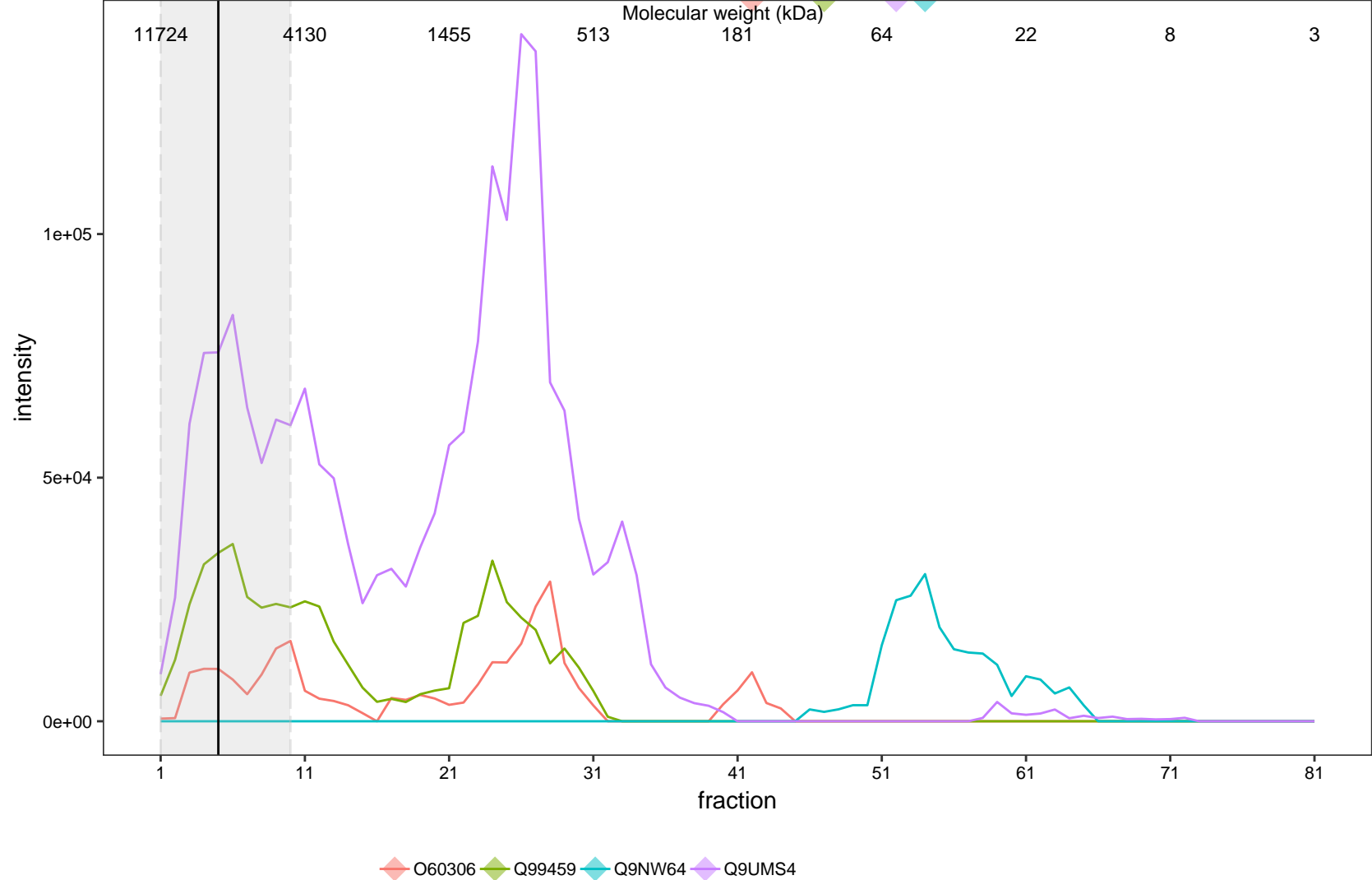

Supplement: Supplementary file 8 — Dataset EV7 [file MSB-15-e8438-s008.zip › feature_plots_string/O60306.pdf]

O60337

Annotated subunits: 9 Subunits with signal: 6

Max. coeluting subunits: 4 Max. completeness: 0.44

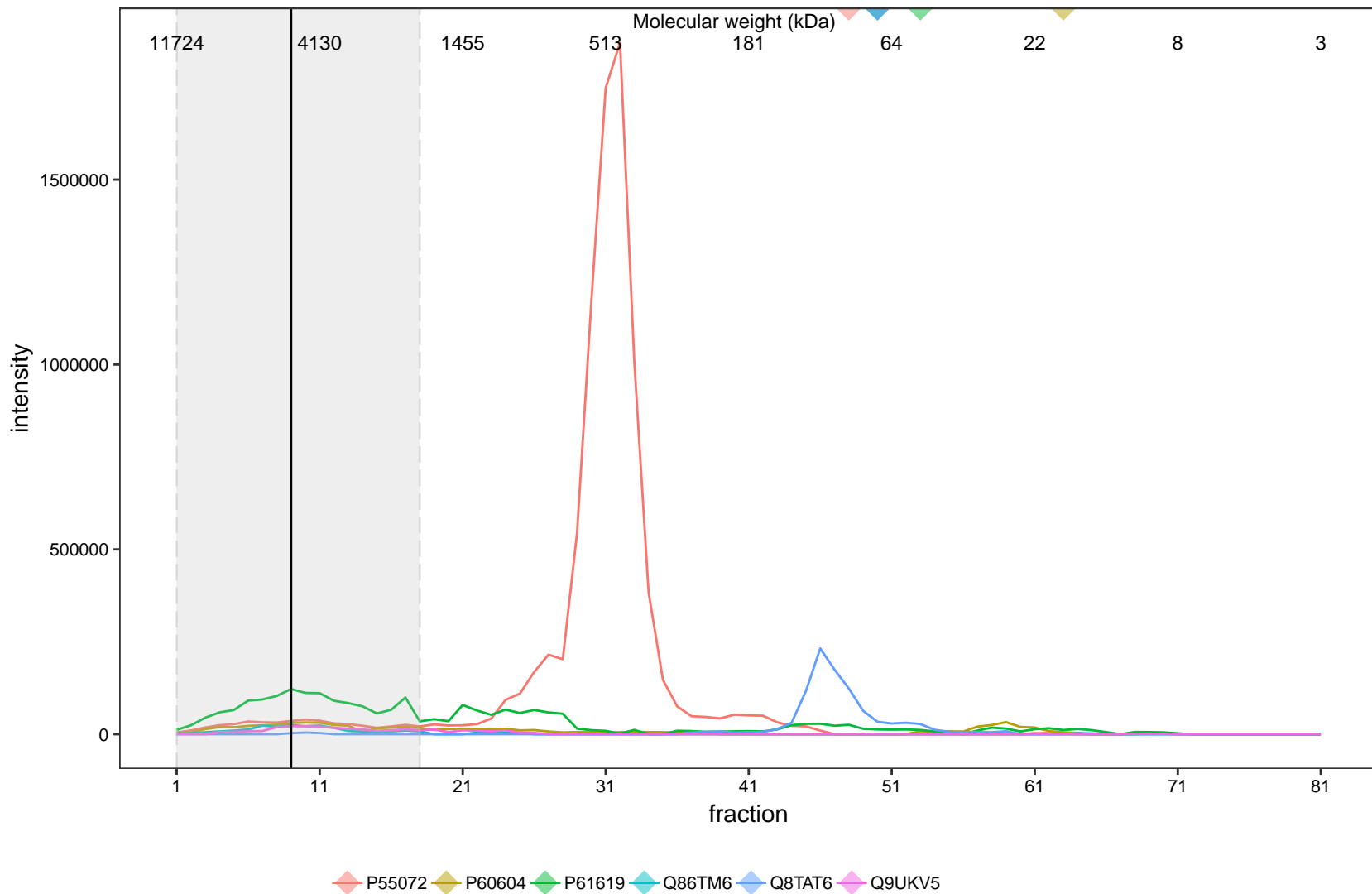

Supplement: Supplementary file 8 — Dataset EV7 [file MSB-15-e8438-s008.zip › feature_plots_string/O60337.pdf]

**O60502**

**Annotated subunits: 4 Subunits with signal: 3**

**Max. coeluting subunits: 2 Max. completeness: 0.5**

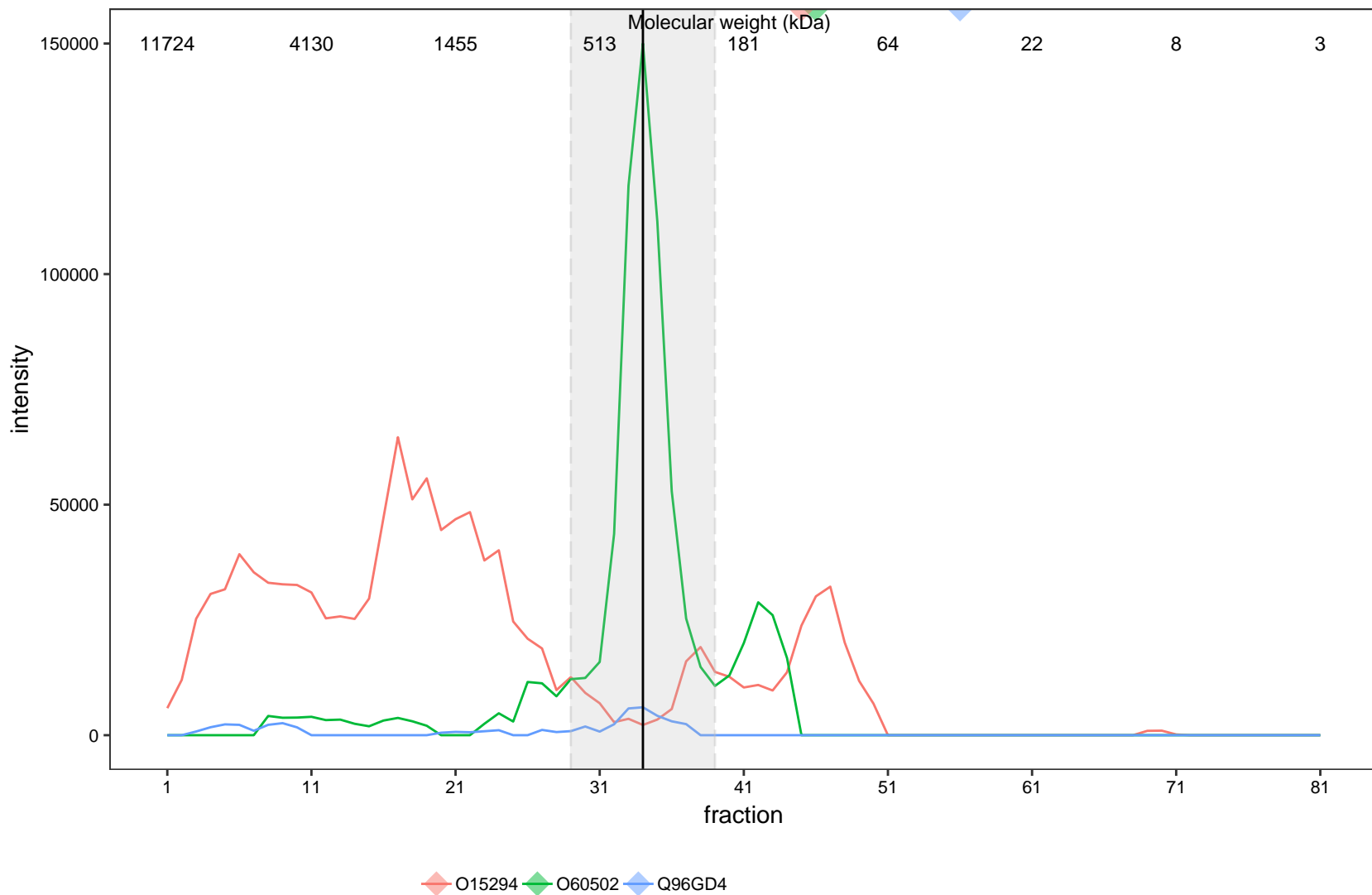

Supplement: Supplementary file 8 — Dataset EV7 [file MSB-15-e8438-s008.zip › feature_plots_string/O60502.pdf]

**O60508**

**Annotated subunits: 138 Subunits with signal: 119**

**Max. coeluting subunits: 30 Max. completeness: 0.22**

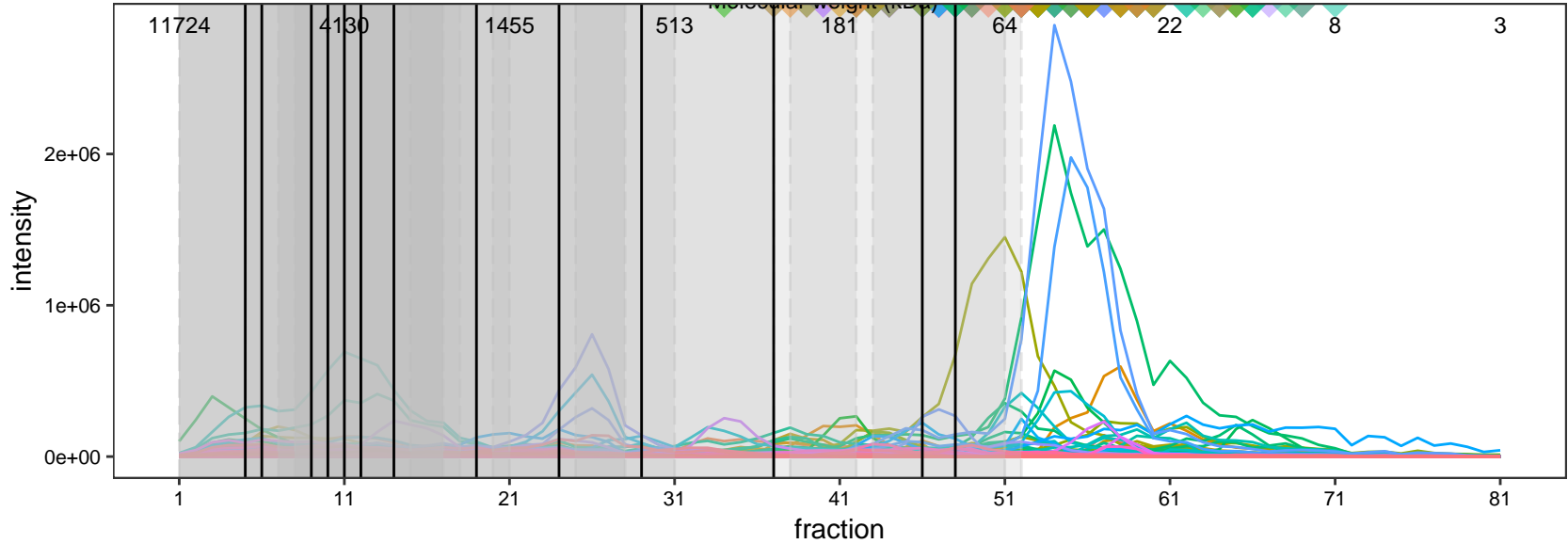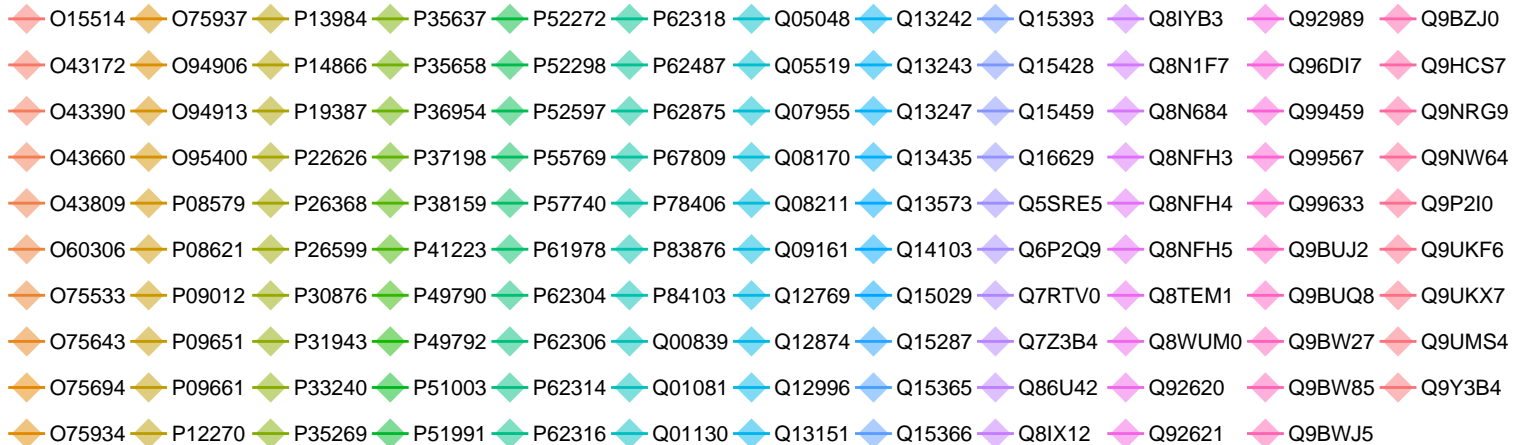

Supplement: Supplementary file 8 — Dataset EV7 [file MSB-15-e8438-s008.zip › feature_plots_string/O60508.pdf]

O60516  
Annotated subunits: 9   Subunits with signal: 6  
Max. coeluting subunits: 3   Max. completeness: 0.33

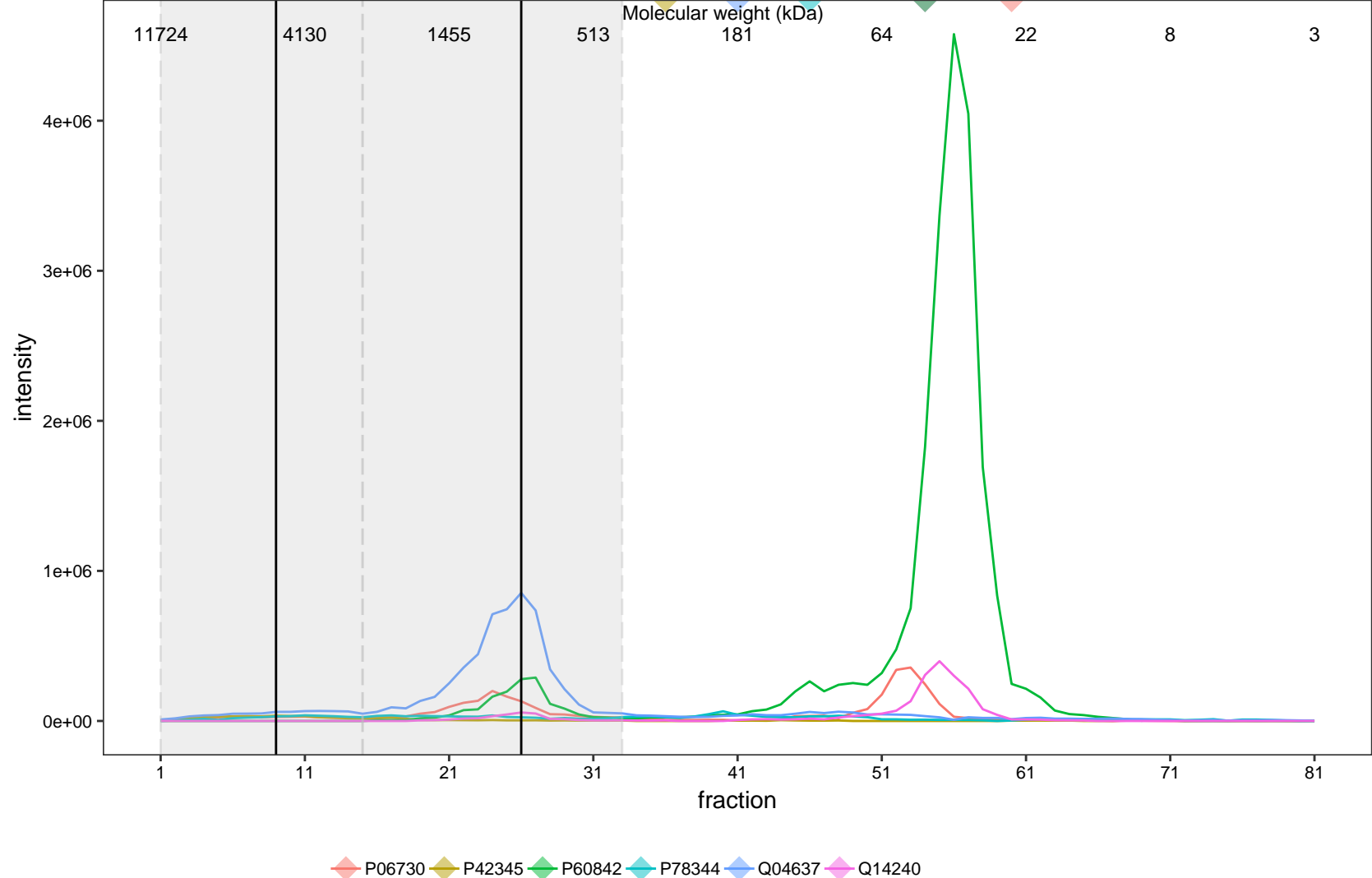

Supplement: Supplementary file 8 — Dataset EV7 [file MSB-15-e8438-s008.zip › feature_plots_string/O60516.pdf]

**O60547**

**Annotated subunits: 4 Subunits with signal: 4**

**Max. coeluting subunits: 2 Max. completeness: 0.5**

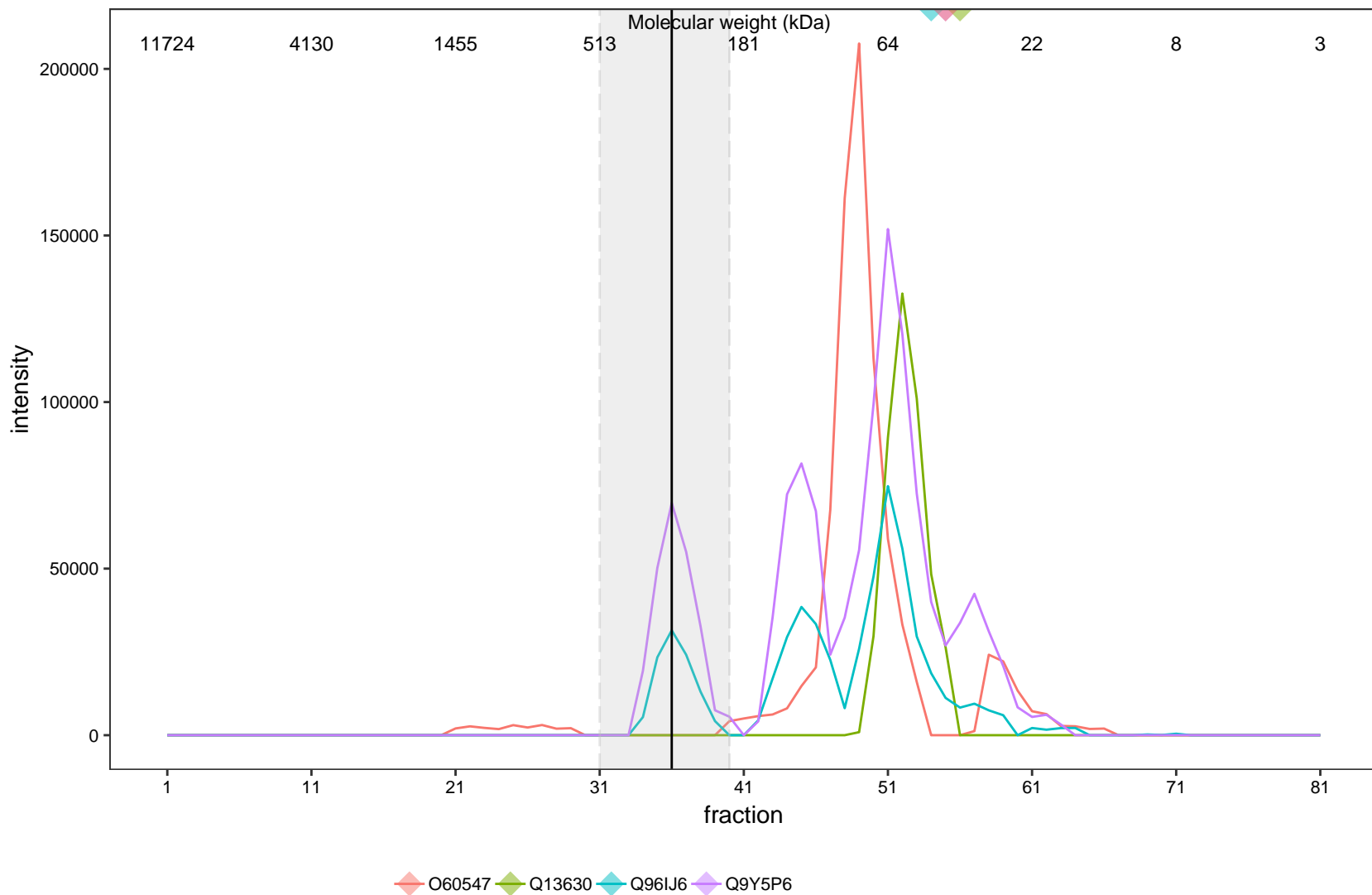

Supplement: Supplementary file 8 — Dataset EV7 [file MSB-15-e8438-s008.zip › feature_plots_string/O60547.pdf]

**O60573**

**Annotated subunits: 19 Subunits with signal: 14**

**Max. coeluting subunits: 3 Max. completeness: 0.16**

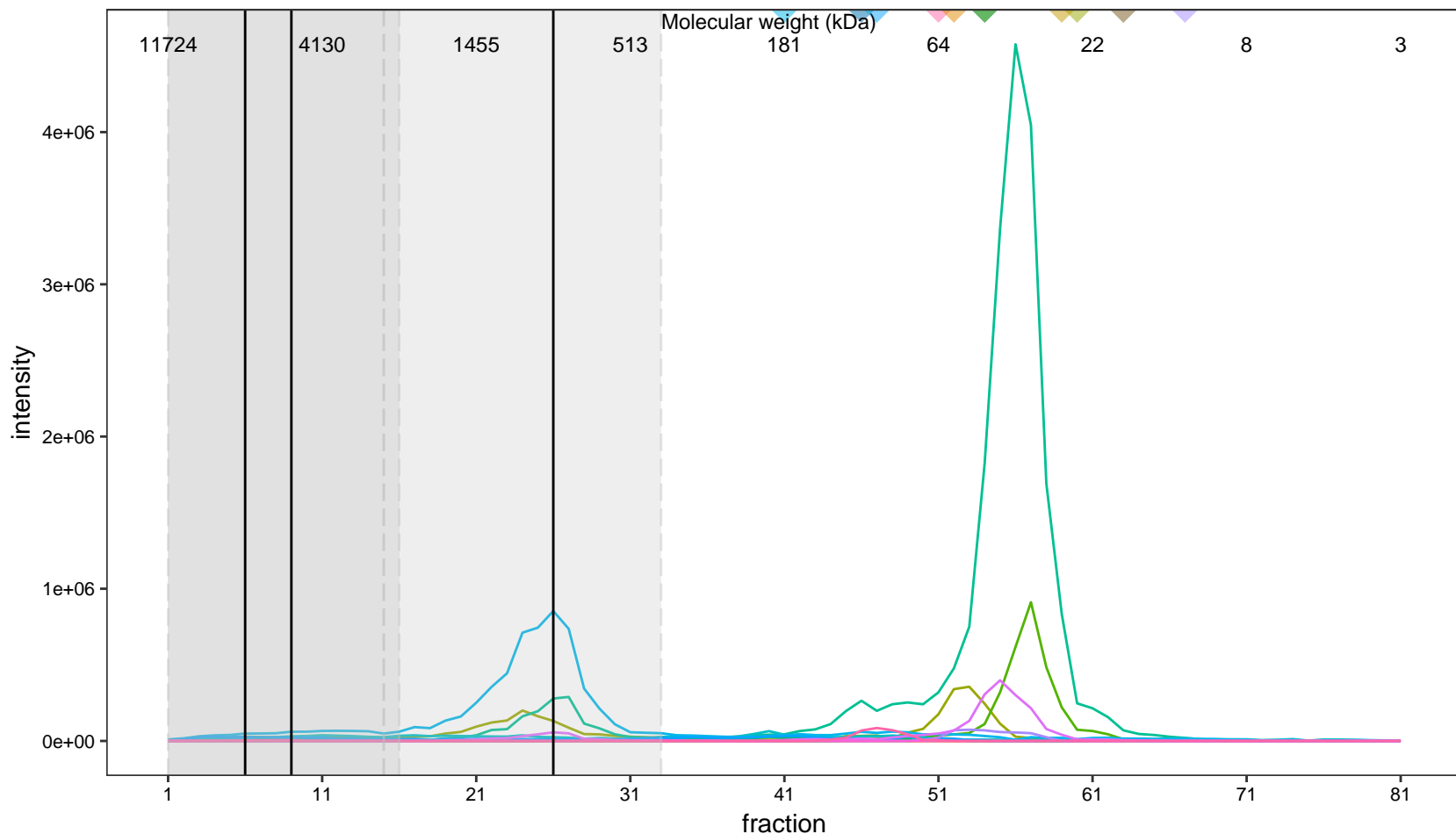

Supplement: Supplementary file 8 — Dataset EV7 [file MSB-15-e8438-s008.zip › feature_plots_string/O60573.pdf]

**O60637**

**Annotated subunits: 2 Subunits with signal: 2**

**Max. coeluting subunits: 2 Max. completeness: 1**

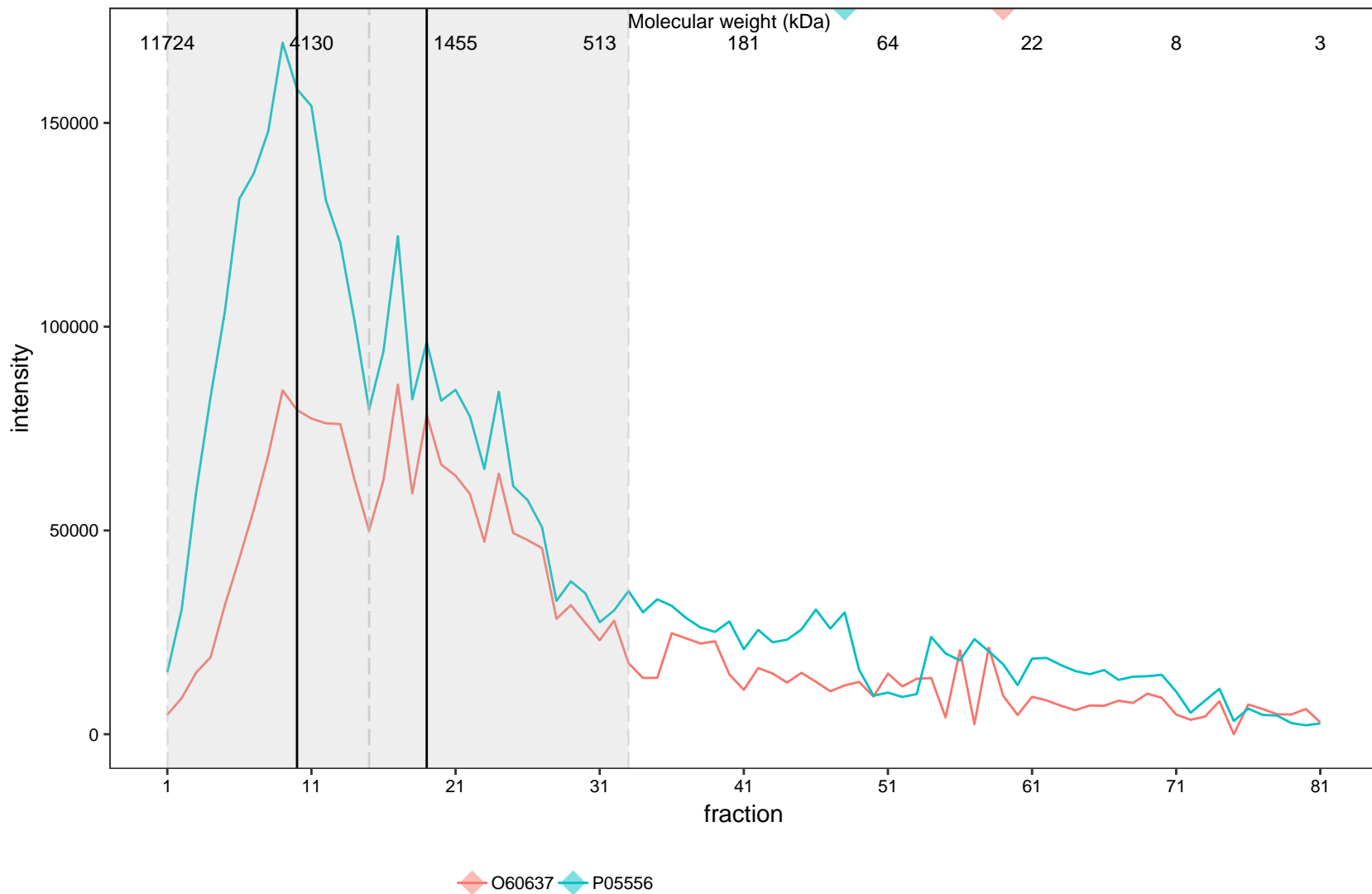

Supplement: Supplementary file 8 — Dataset EV7 [file MSB-15-e8438-s008.zip › feature_plots_string/O60637.pdf]

O60664  
Annotated subunits: 8 Subunits with signal: 6  
Max. coeluting subunits: 3 Max. completeness: 0.38

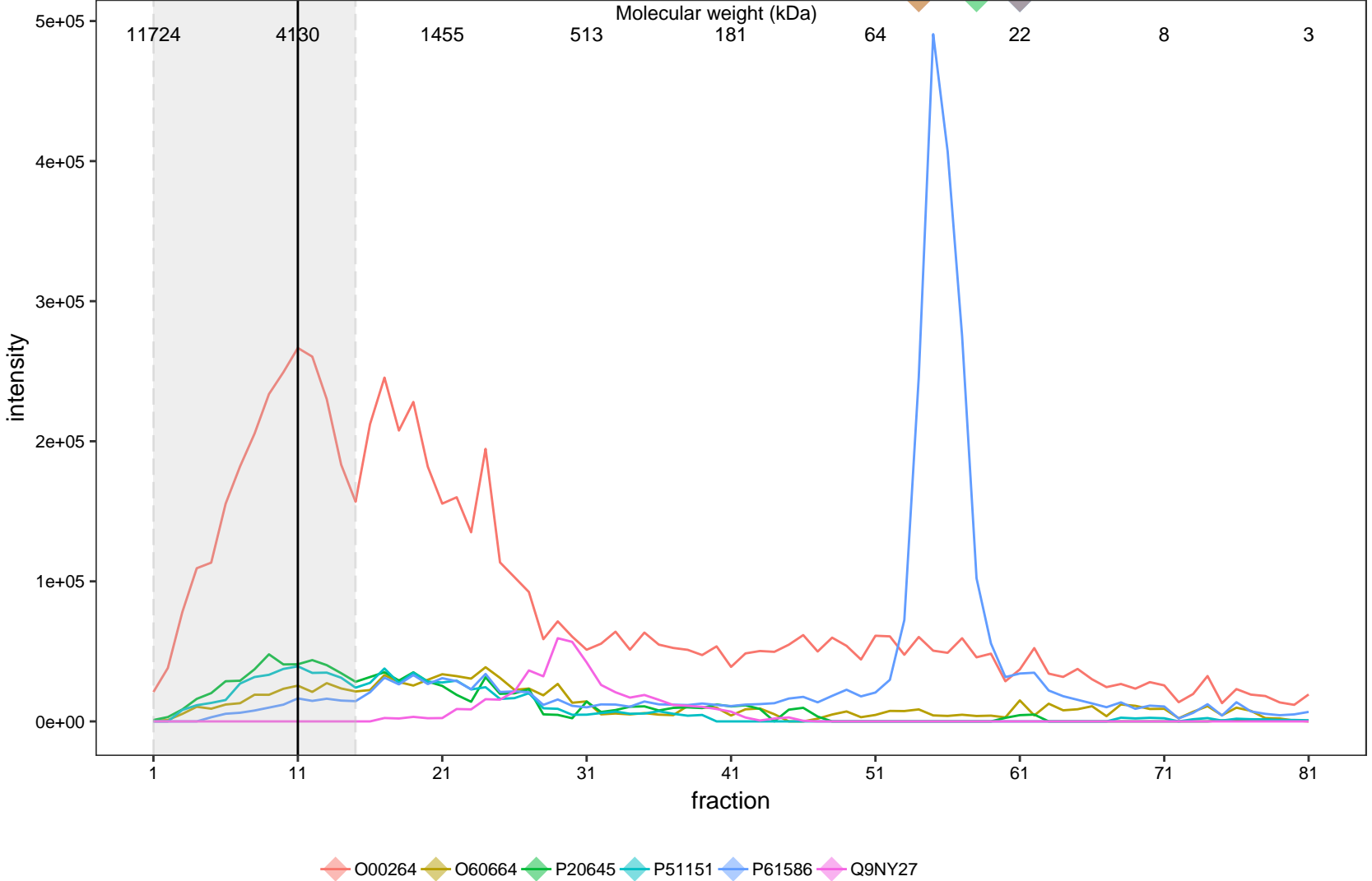

Supplement: Supplementary file 8 — Dataset EV7 [file MSB-15-e8438-s008.zip › feature_plots_string/O60664.pdf]

O60678  
Annotated subunits: 4   Subunits with signal: 4  
Max. coeluting subunits: 2   Max. completeness: 0.5

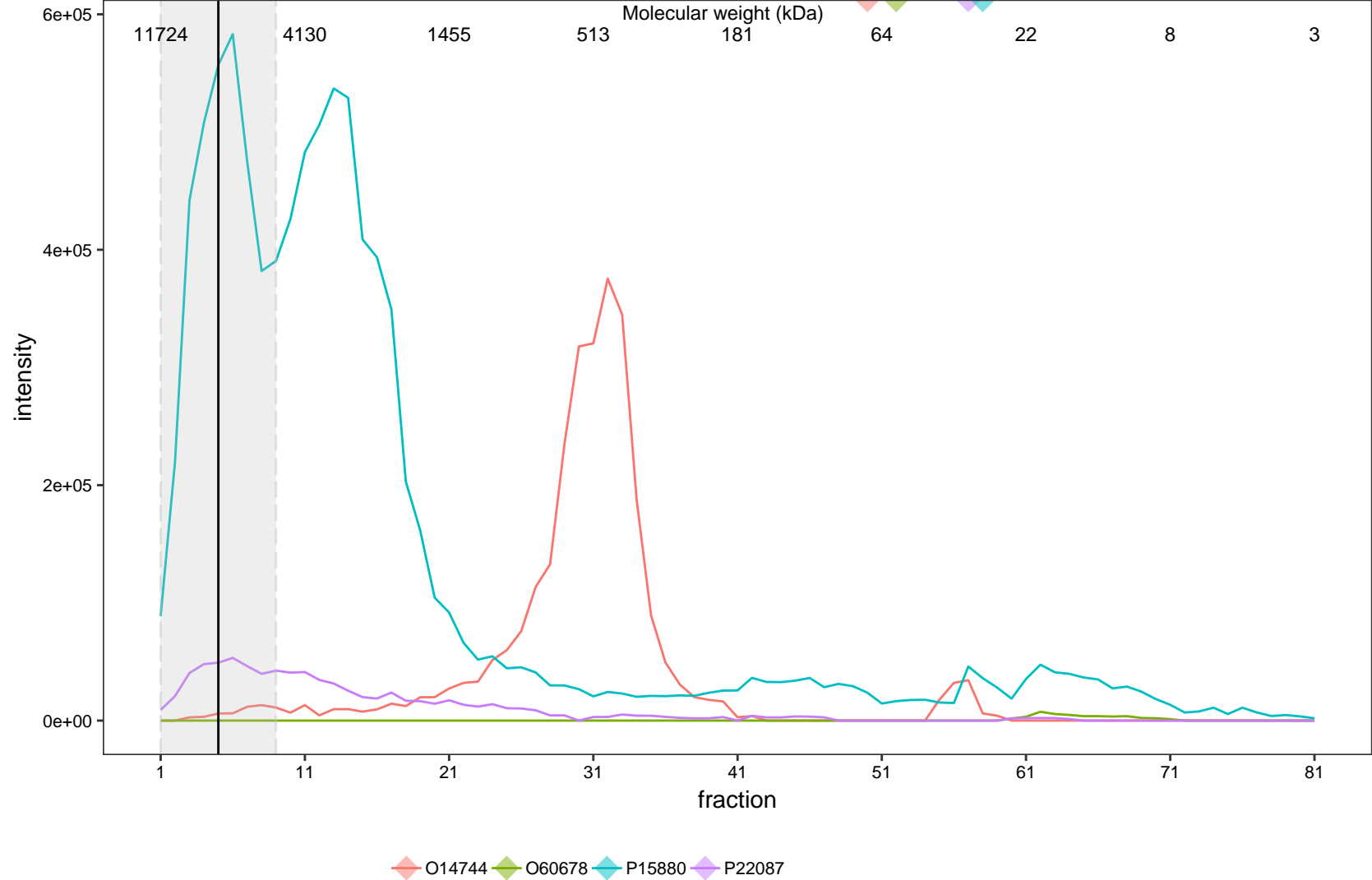

Supplement: Supplementary file 8 — Dataset EV7 [file MSB-15-e8438-s008.zip › feature_plots_string/O60678.pdf]

O60683

Annotated subunits: 10 Subunits with signal: 4

Max. coeluting subunits: 2 Max. completeness: 0.2

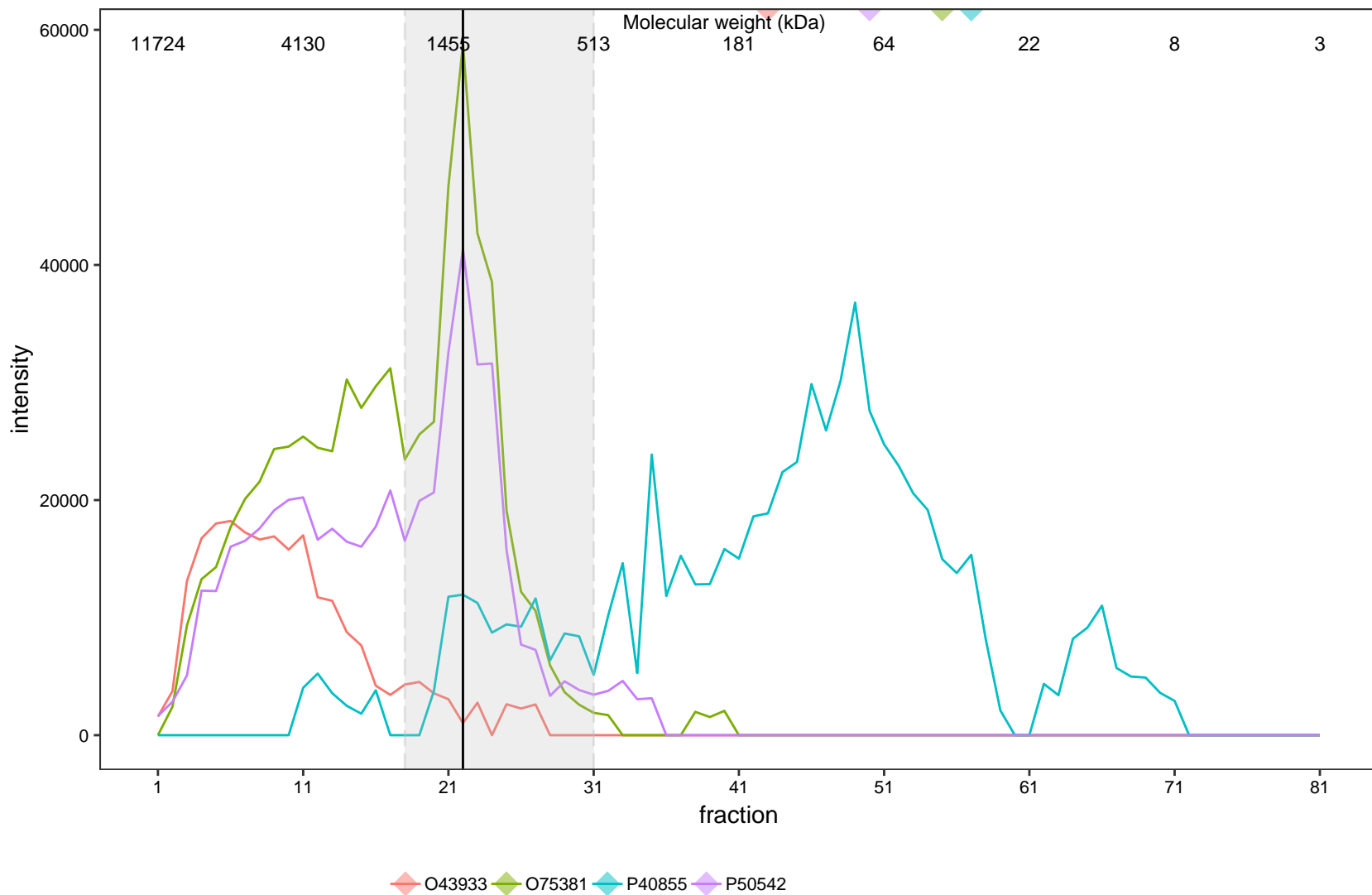

Supplement: Supplementary file 8 — Dataset EV7 [file MSB-15-e8438-s008.zip › feature_plots_string/O60683.pdf]

O60739

Annotated subunits: 9 Subunits with signal: 8

Max. coeluting subunits: 5 Max. completeness: 0.56

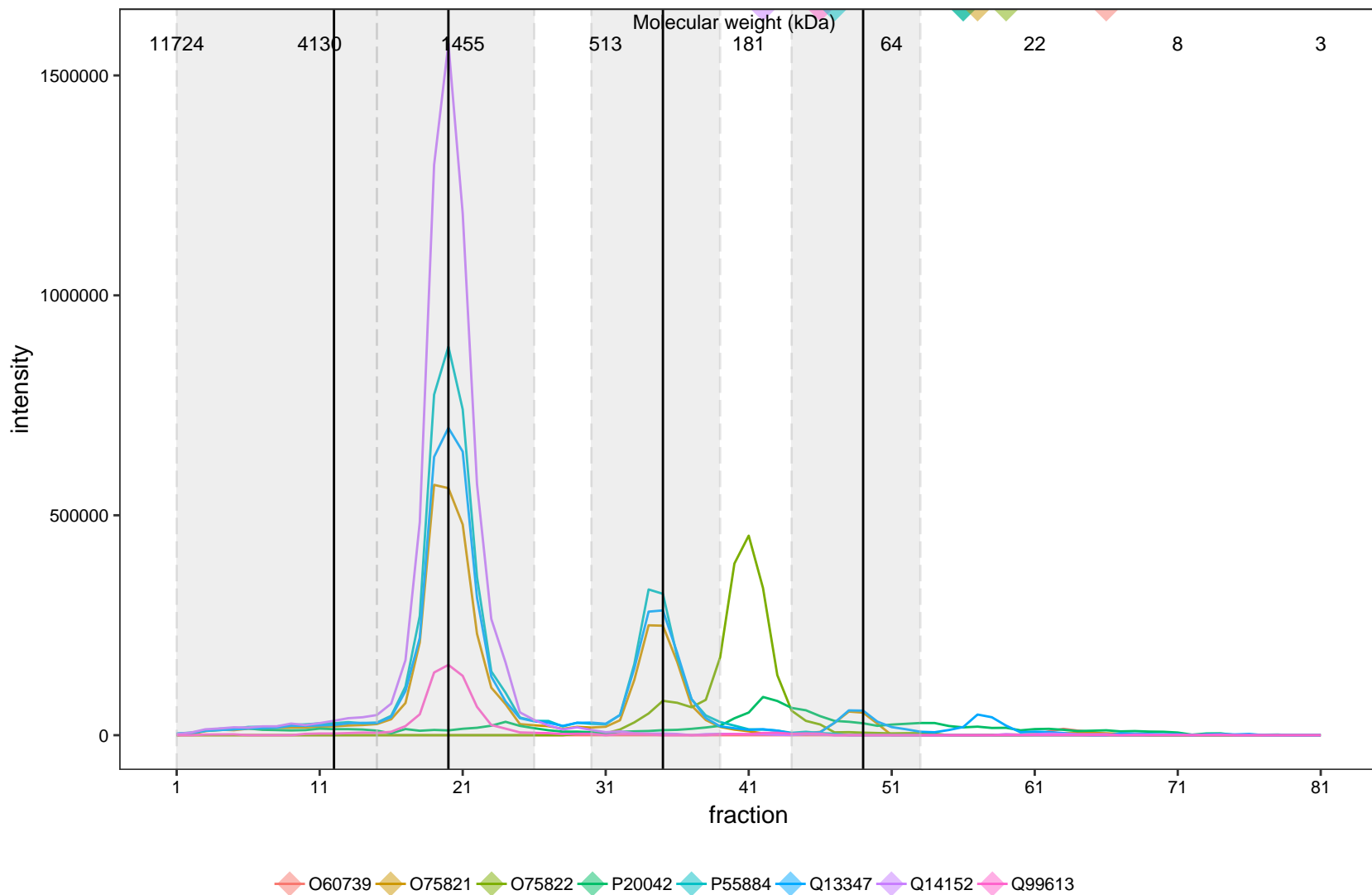

Supplement: Supplementary file 8 — Dataset EV7 [file MSB-15-e8438-s008.zip › feature_plots_string/O60739.pdf]

**O60749**

**Annotated subunits: 10 Subunits with signal: 6**

**Max. coeluting subunits: 2 Max. completeness: 0.2**

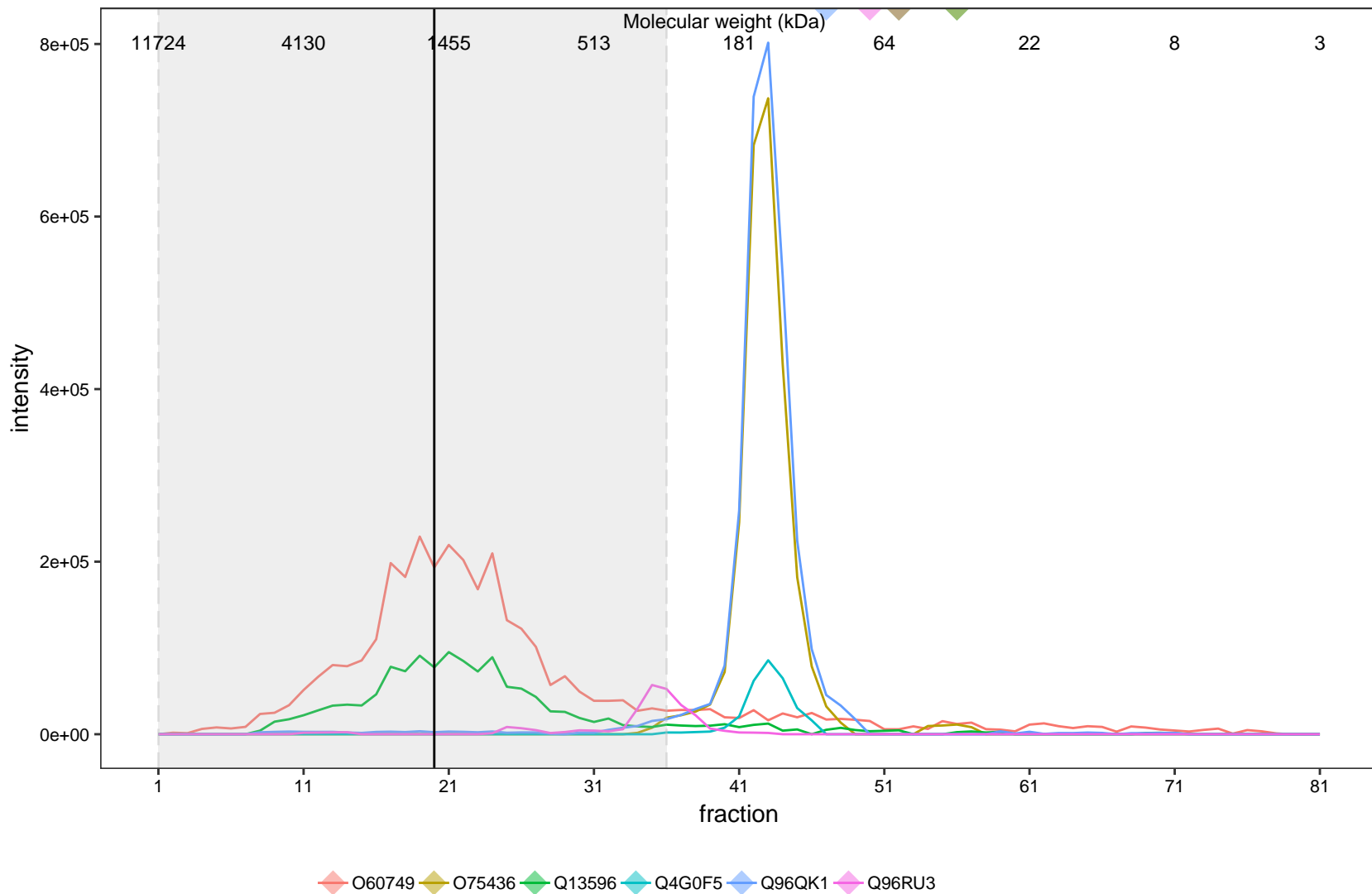

Supplement: Supplementary file 8 — Dataset EV7 [file MSB-15-e8438-s008.zip › feature_plots_string/O60749.pdf]

**O60825**

**Annotated subunits: 14 Subunits with signal: 9**

**Max. coeluting subunits: 3 Max. completeness: 0.21**

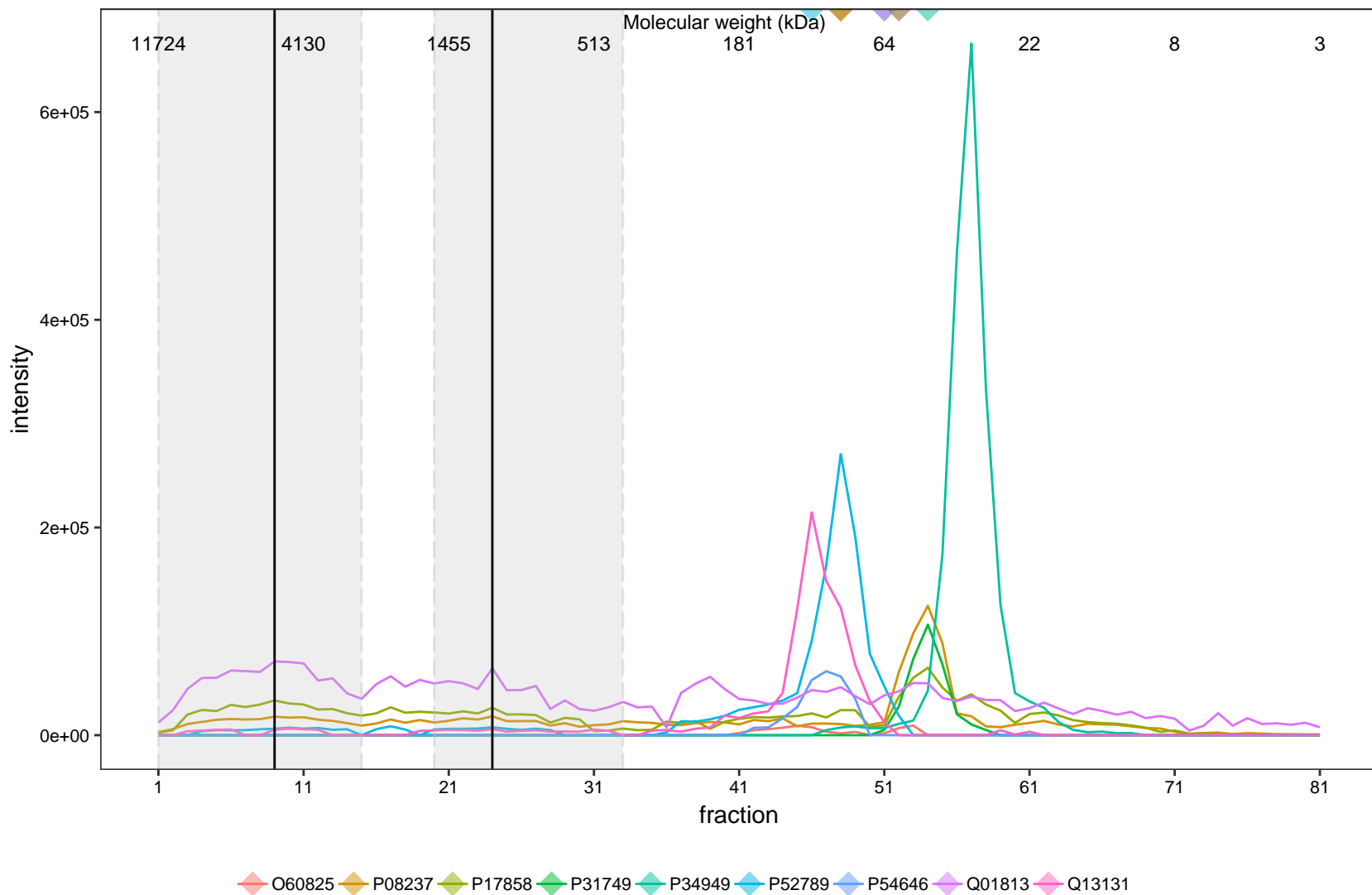

Supplement: Supplementary file 8 — Dataset EV7 [file MSB-15-e8438-s008.zip › feature_plots_string/O60825.pdf]

**O60830**

**Annotated subunits: 9 Subunits with signal: 8**

**Max. coeluting subunits: 8 Max. completeness: 0.89**

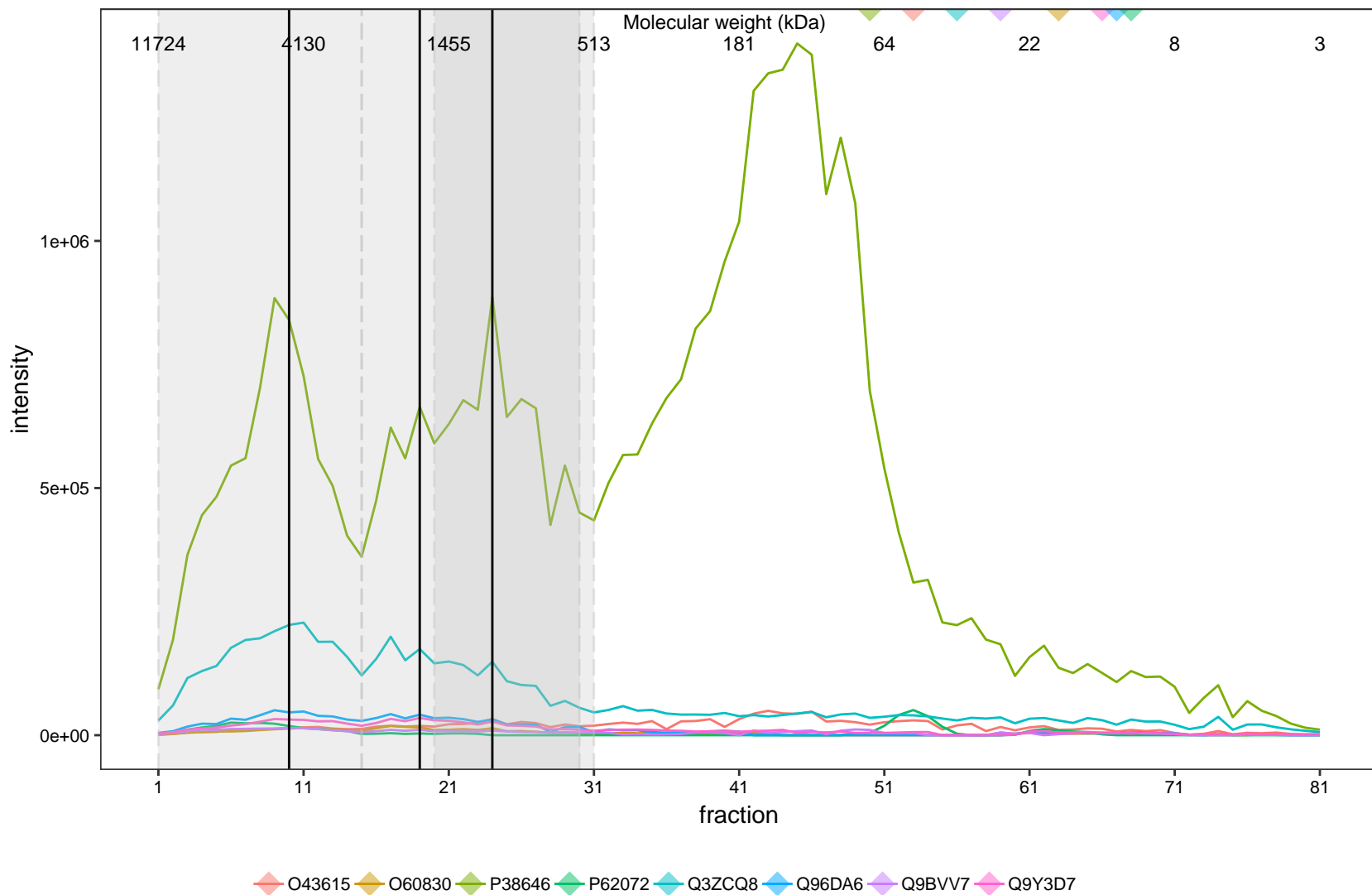

Supplement: Supplementary file 8 — Dataset EV7 [file MSB-15-e8438-s008.zip › feature_plots_string/O60830.pdf]

**O60885**

**Annotated subunits: 6 Subunits with signal: 4**

**Max. coeluting subunits: 4 Max. completeness: 0.67**

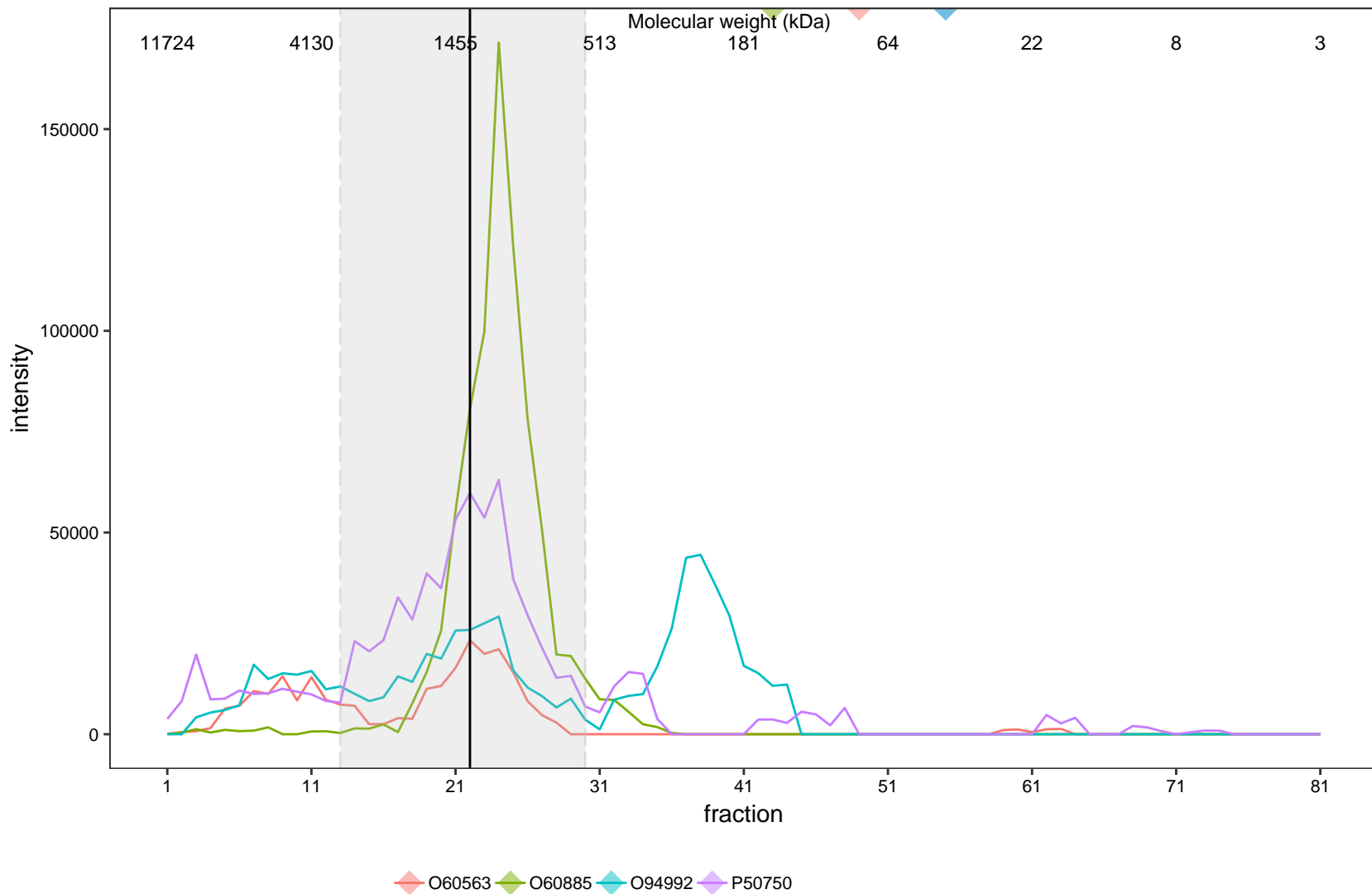

Supplement: Supplementary file 8 — Dataset EV7 [file MSB-15-e8438-s008.zip › feature_plots_string/O60885.pdf]

**O60925**

**Annotated subunits: 22 Subunits with signal: 14**

**Max. coeluting subunits: 8 Max. completeness: 0.36**

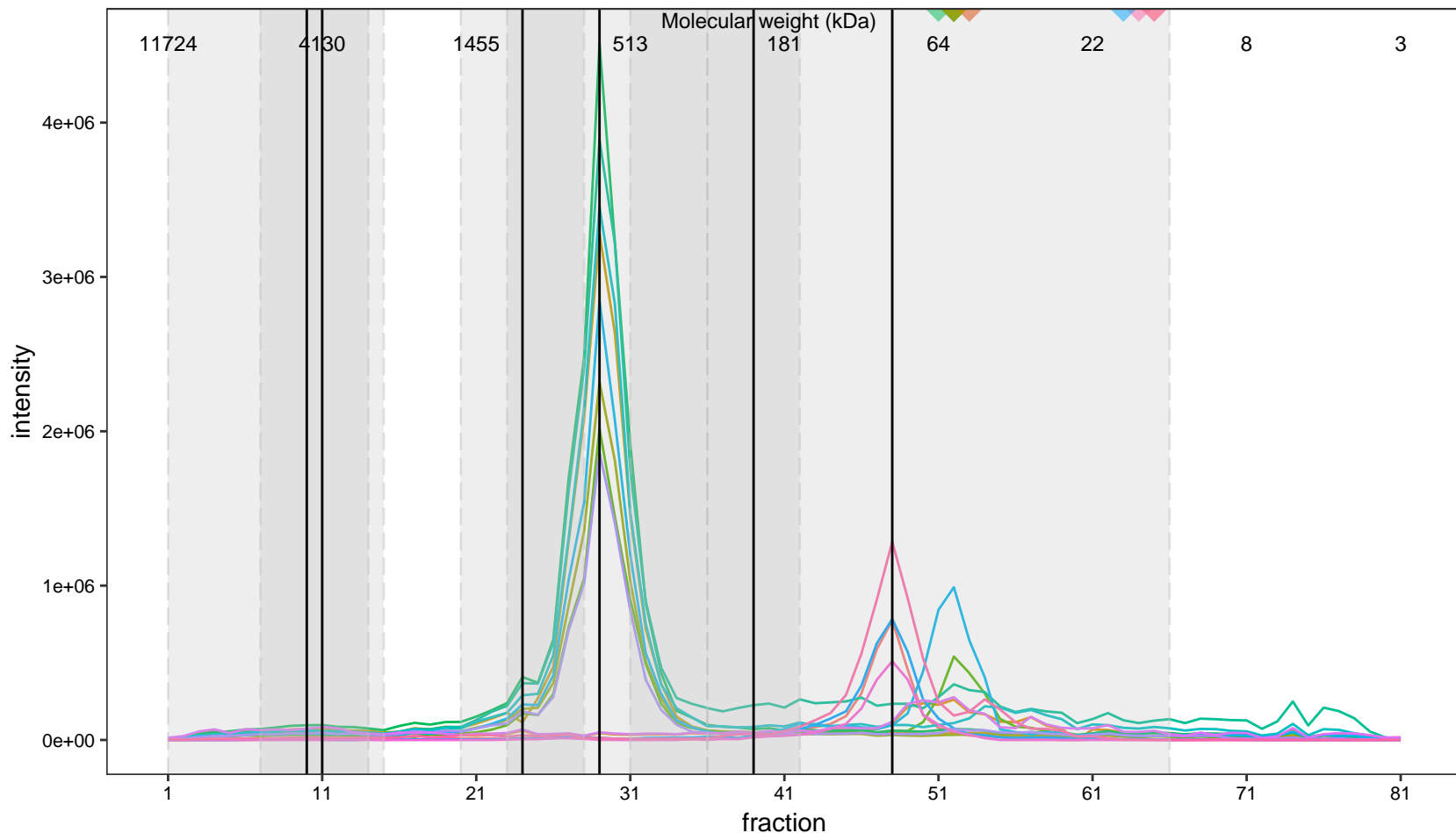

Supplement: Supplementary file 8 — Dataset EV7 [file MSB-15-e8438-s008.zip › feature_plots_string/O60925.pdf]

O60930  
Annotated subunits: 4   Subunits with signal: 3  
Max. coeluting subunits: 2   Max. completeness: 0.5

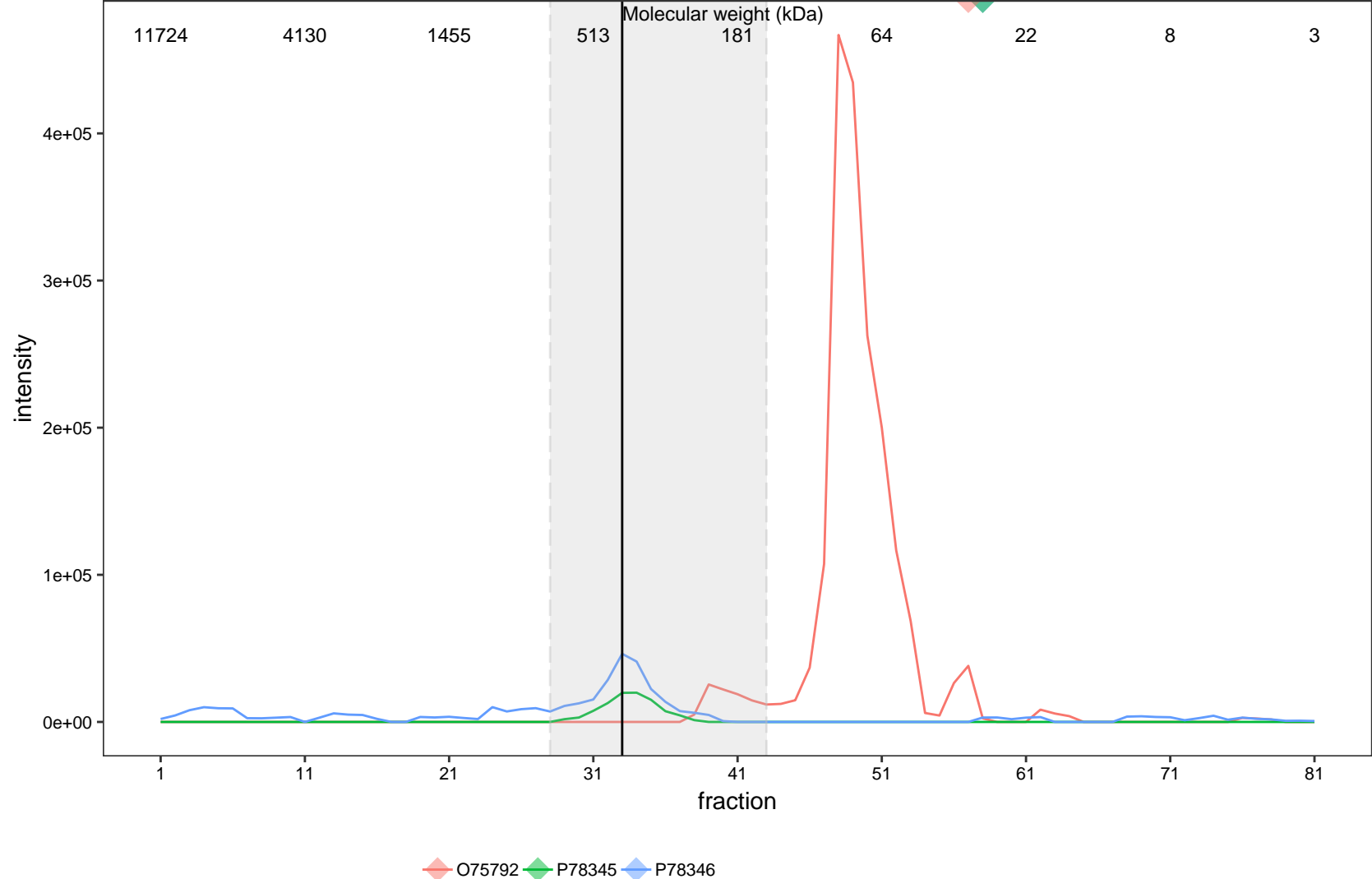

Supplement: Supplementary file 8 — Dataset EV7 [file MSB-15-e8438-s008.zip › feature_plots_string/O60930.pdf]

O60942

Annotated subunits: 23 Subunits with signal: 16

Max. coeluting subunits: 7 Max. completeness: 0.3

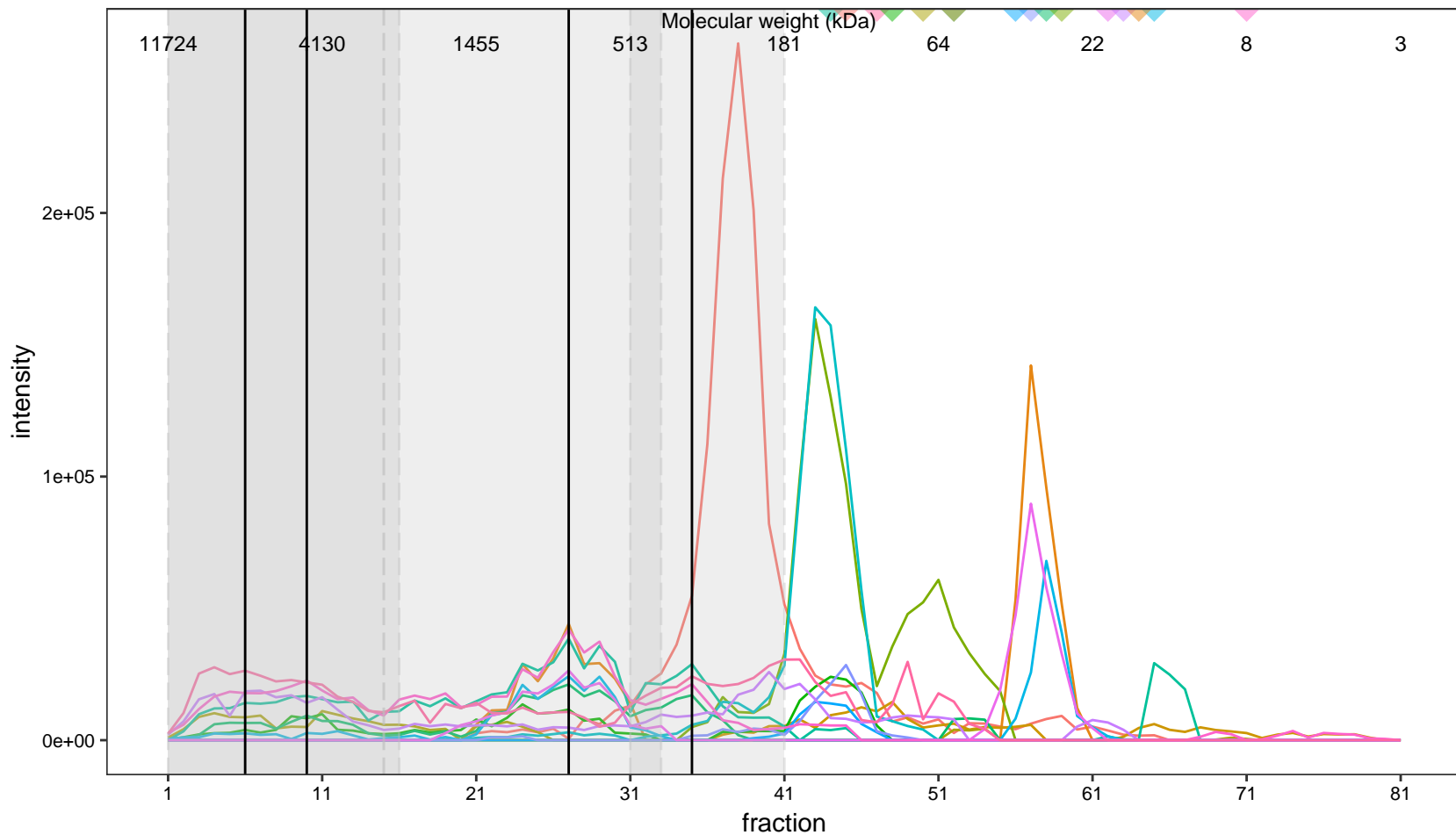

Supplement: Supplementary file 8 — Dataset EV7 [file MSB-15-e8438-s008.zip › feature_plots_string/O60942.pdf]

**O75027**  
Annotated subunits: 14   Subunits with signal: 11  
Max. coeluting subunits: 3   Max. completeness: 0.21

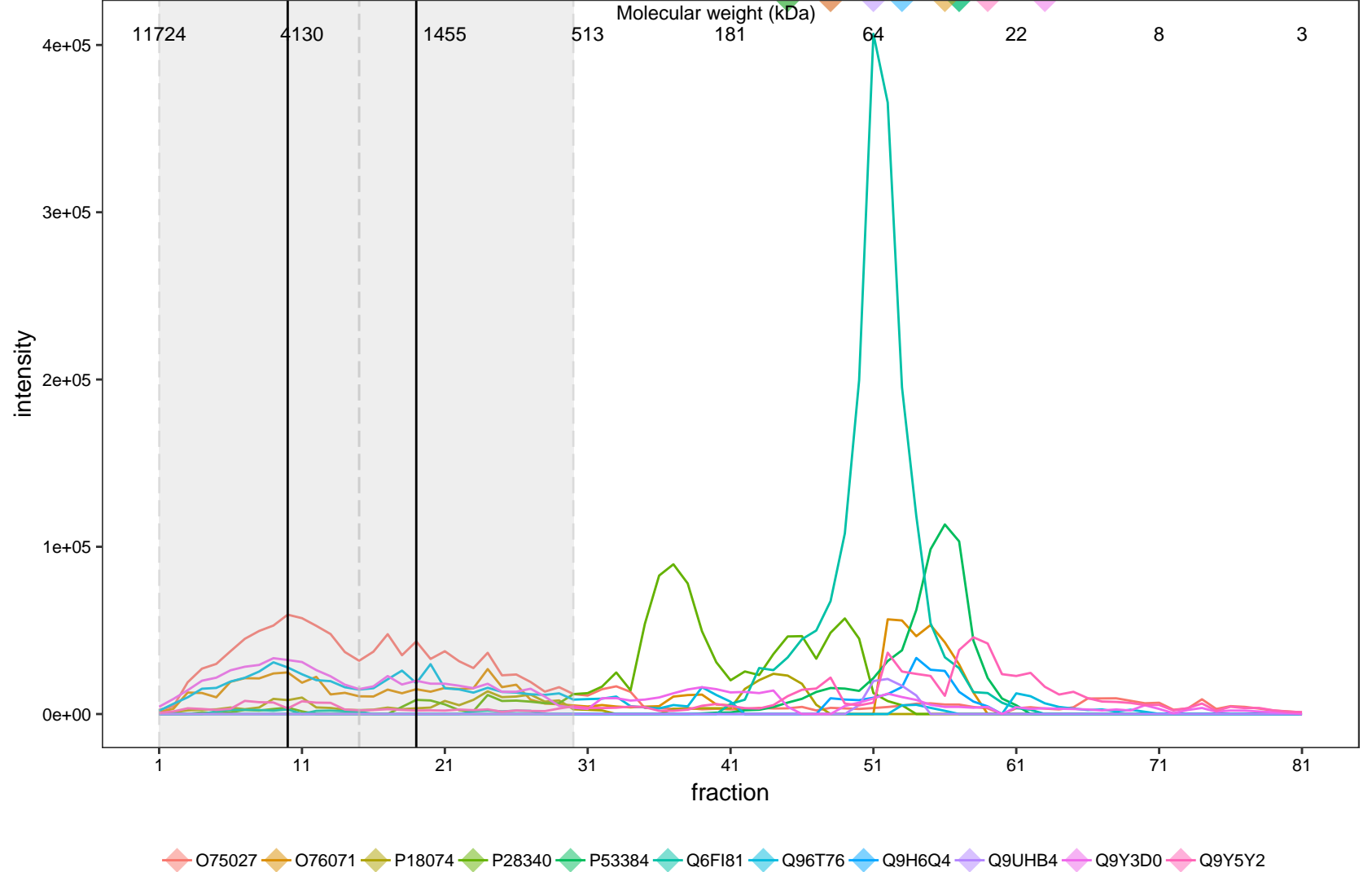

Supplement: Supplementary file 8 — Dataset EV7 [file MSB-15-e8438-s008.zip › feature_plots_string/O75027.pdf]

O75051

Annotated subunits: 26 Subunits with signal: 15

Max. coeluting subunits: 4 Max. completeness: 0.15

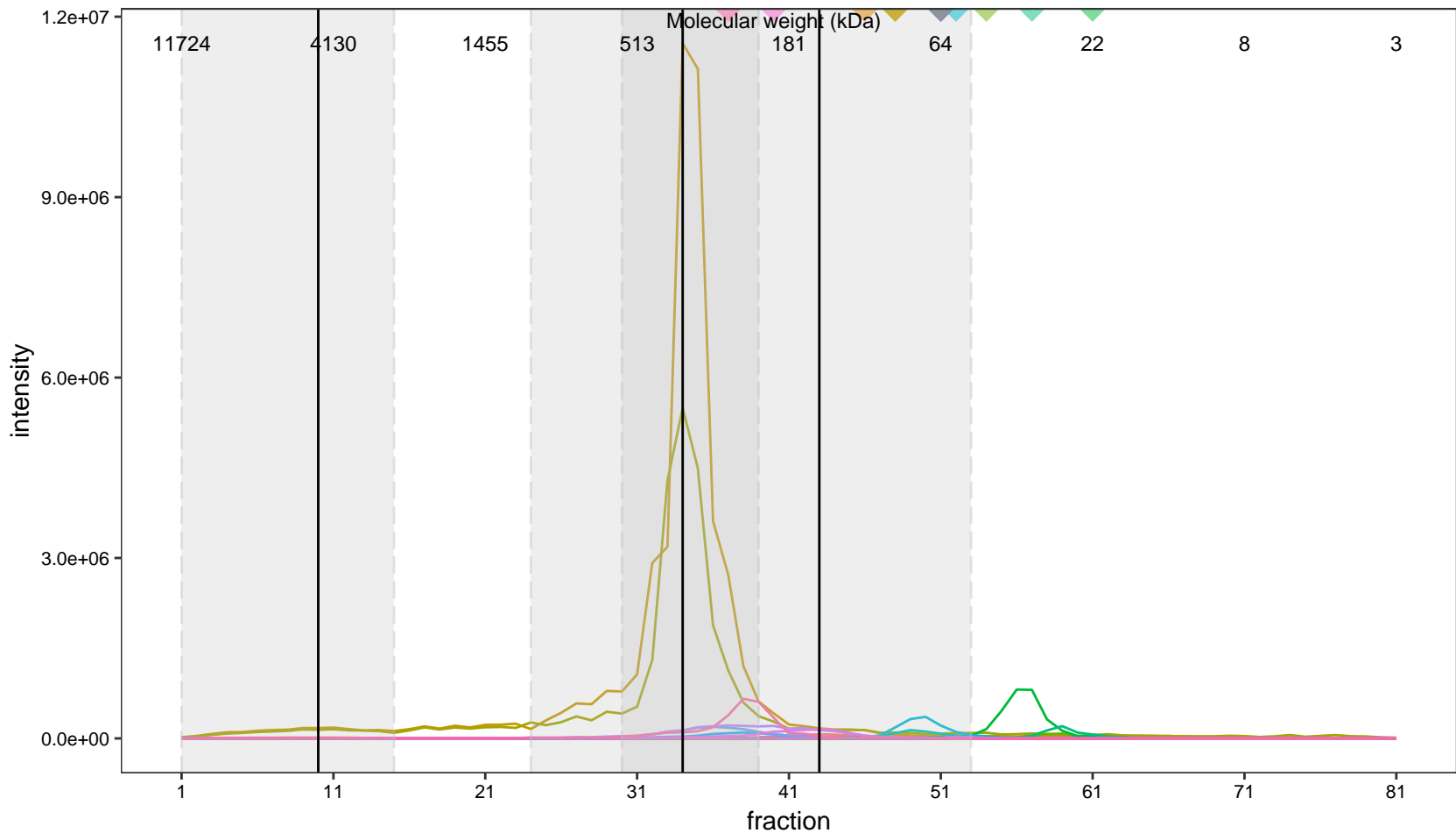

Supplement: Supplementary file 8 — Dataset EV7 [file MSB-15-e8438-s008.zip › feature_plots_string/O75051.pdf]

**O75081**

**Annotated subunits: 8 Subunits with signal: 3**

**Max. coeluting subunits: 2 Max. completeness: 0.25**

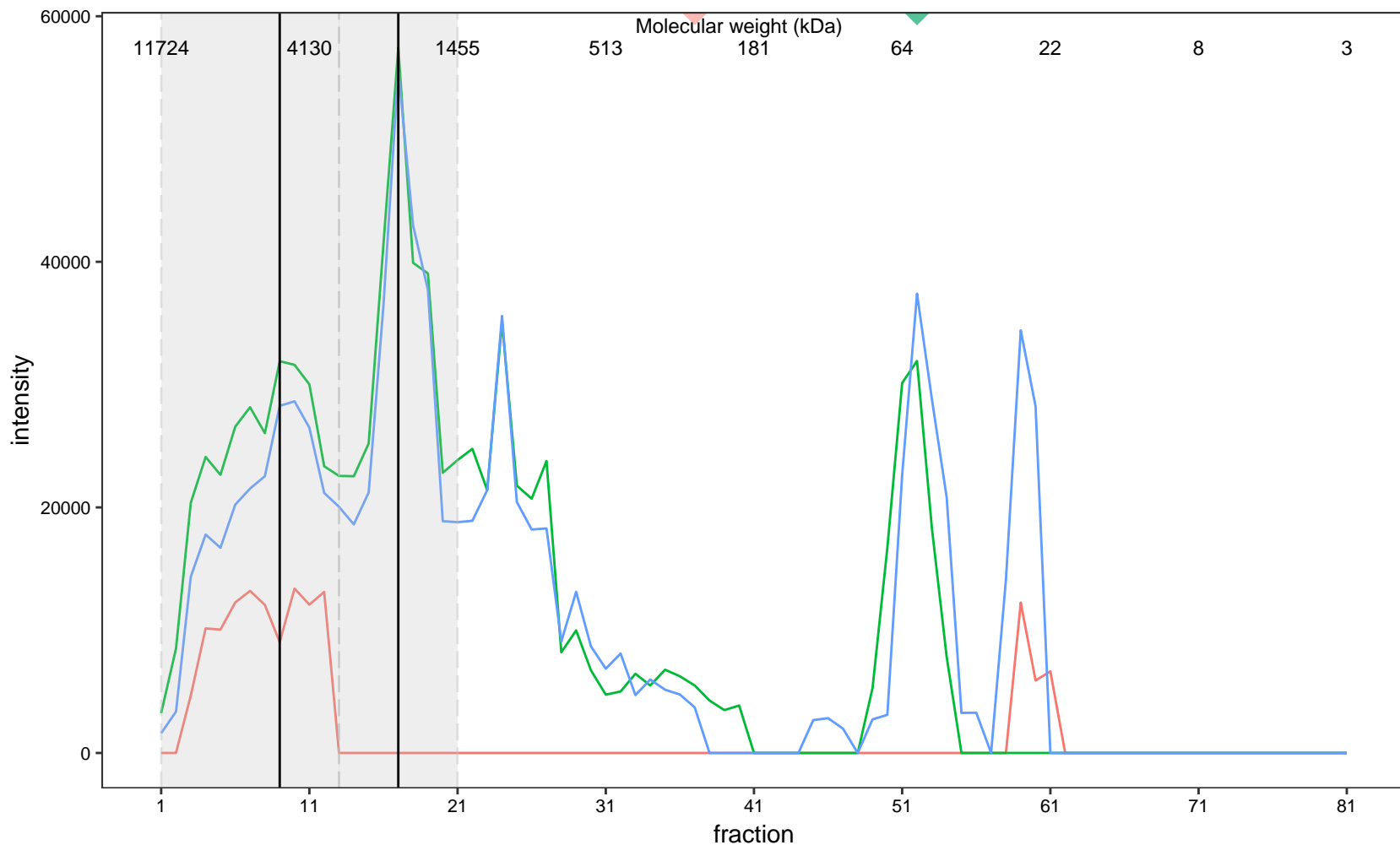

◀ O75376 ◀ Q13547 ◀ Q92769

Supplement: Supplementary file 8 — Dataset EV7 [file MSB-15-e8438-s008.zip › feature_plots_string/O75081.pdf]

**O75093**

**Annotated subunits: 15 Subunits with signal: 4**

**Max. coeluting subunits: 3 Max. completeness: 0.2**

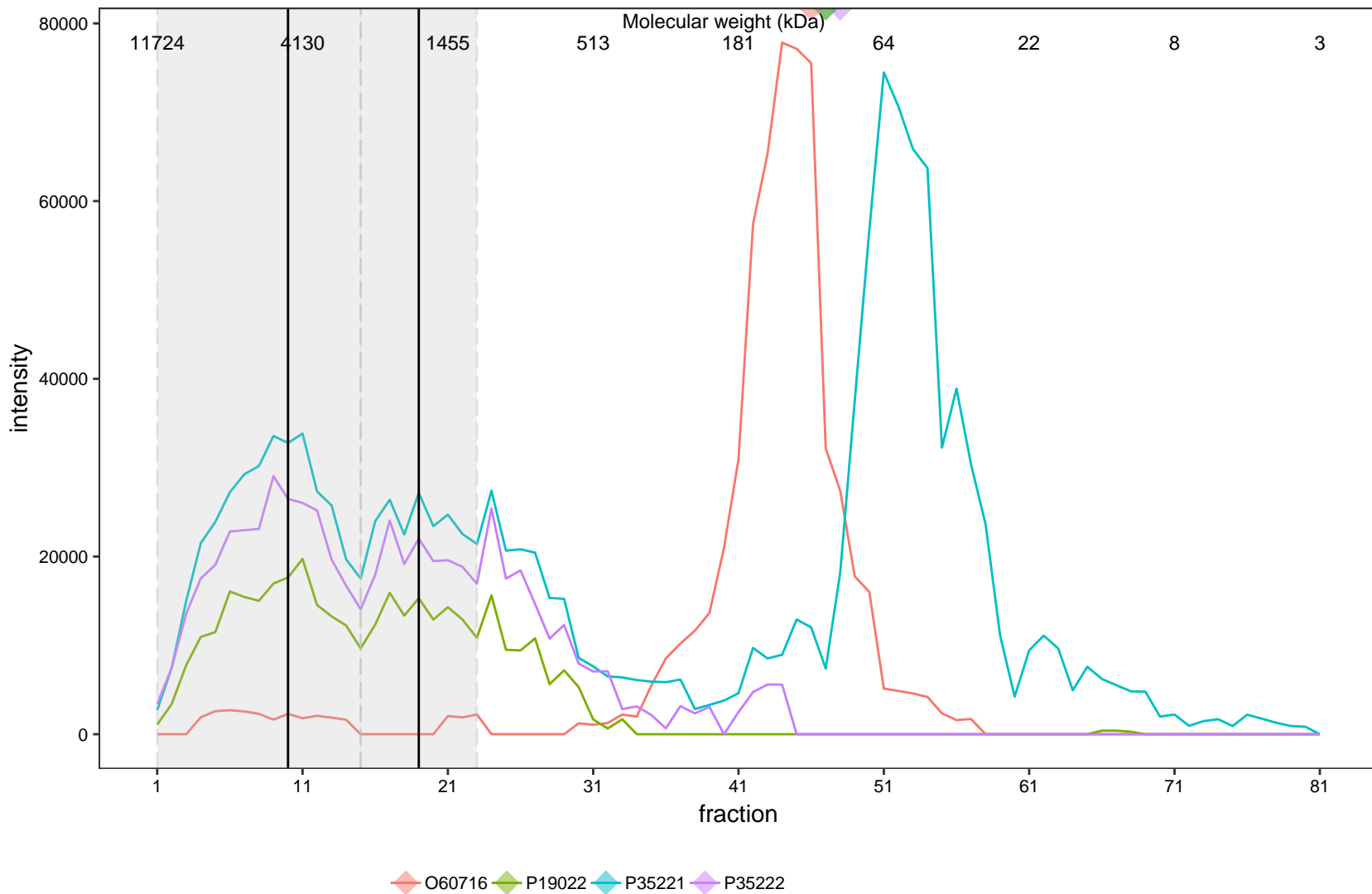

Supplement: Supplementary file 8 — Dataset EV7 [file MSB-15-e8438-s008.zip › feature_plots_string/O75093.pdf]

**O75146**

**Annotated subunits: 8 Subunits with signal: 5**

**Max. coeluting subunits: 2 Max. completeness: 0.25**

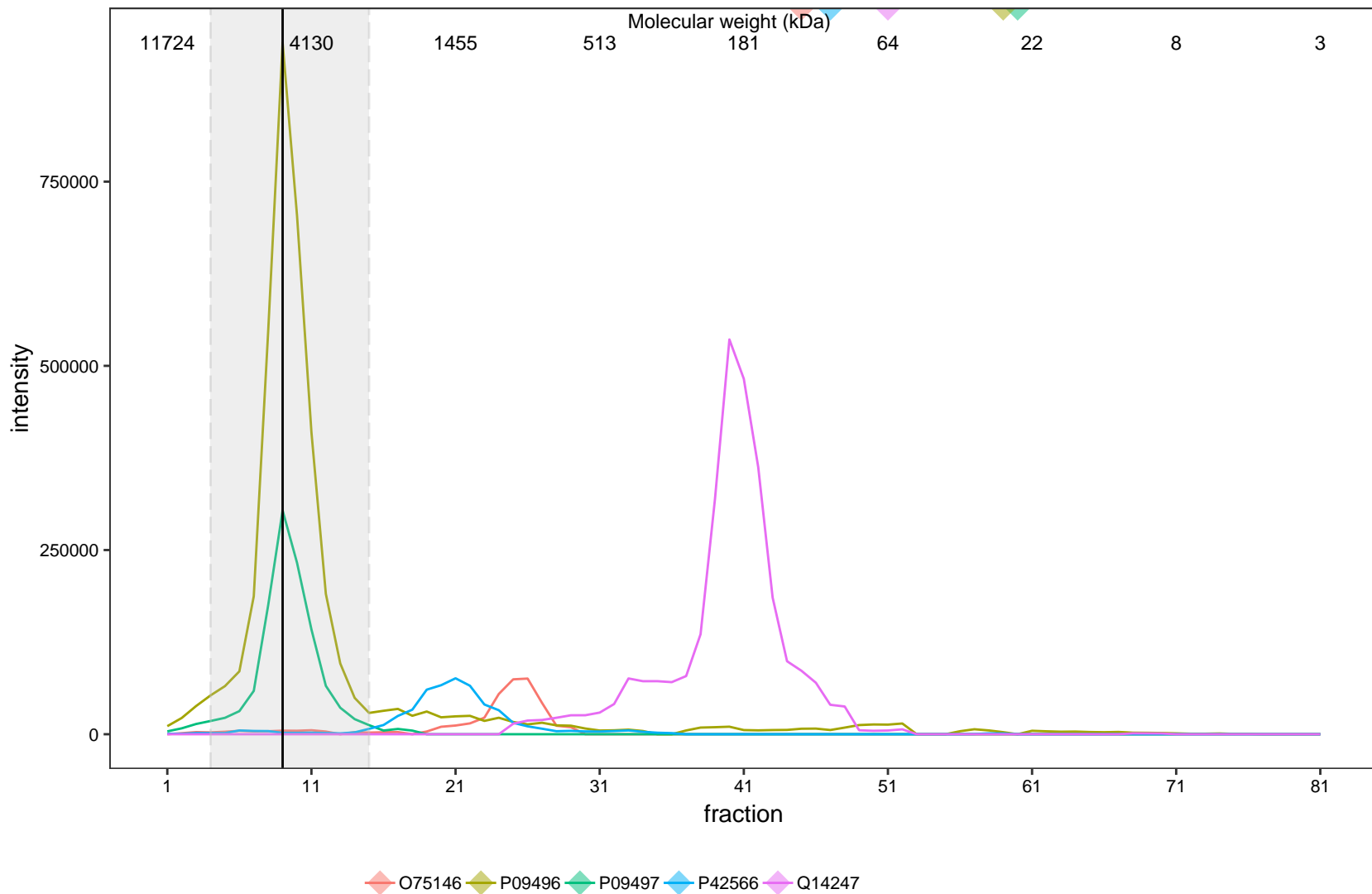

Supplement: Supplementary file 8 — Dataset EV7 [file MSB-15-e8438-s008.zip › feature_plots_string/O75146.pdf]

**O75150**

**Annotated subunits: 9 Subunits with signal: 7**

**Max. coeluting subunits: 2 Max. completeness: 0.22**

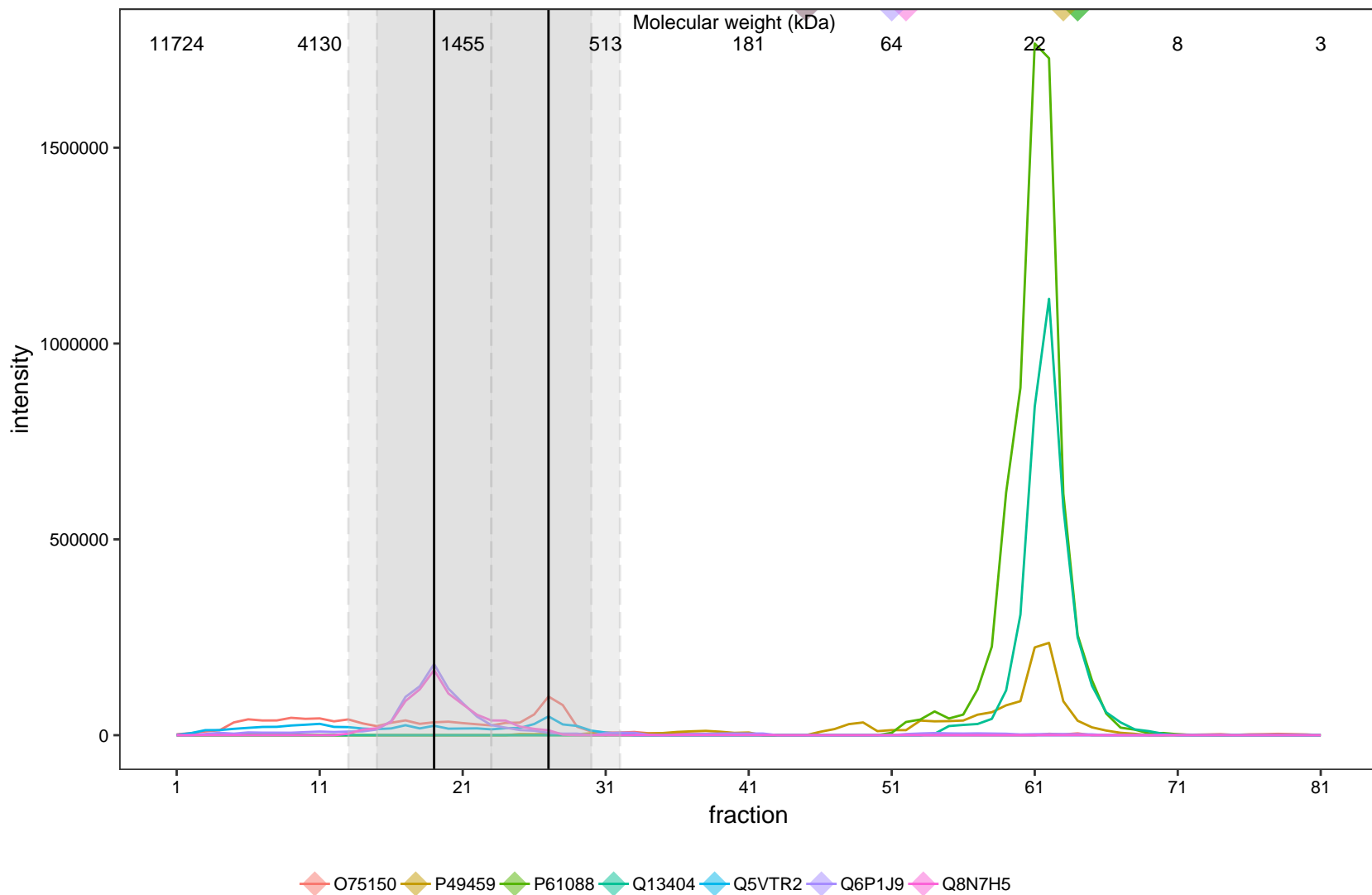

Supplement: Supplementary file 8 — Dataset EV7 [file MSB-15-e8438-s008.zip › feature_plots_string/O75150.pdf]

**O75170**  
Annotated subunits: 5   Subunits with signal: 4  
Max. coeluting subunits: 2   Max. completeness: 0.4

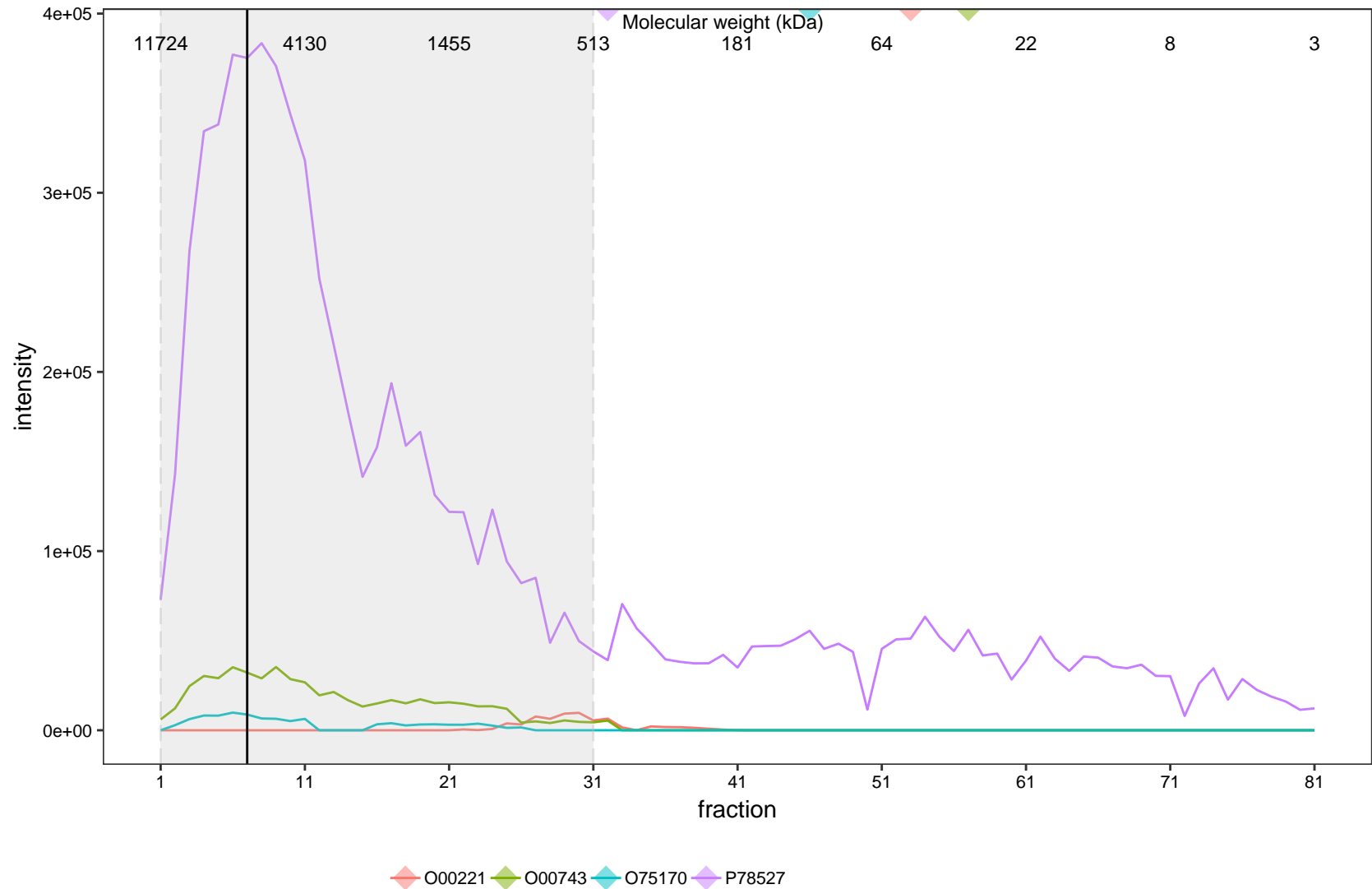

Supplement: Supplementary file 8 — Dataset EV7 [file MSB-15-e8438-s008.zip › feature_plots_string/O75170.pdf]

**O75175**

**Annotated subunits: 3 Subunits with signal: 3**

**Max. coeluting subunits: 3 Max. completeness: 1**

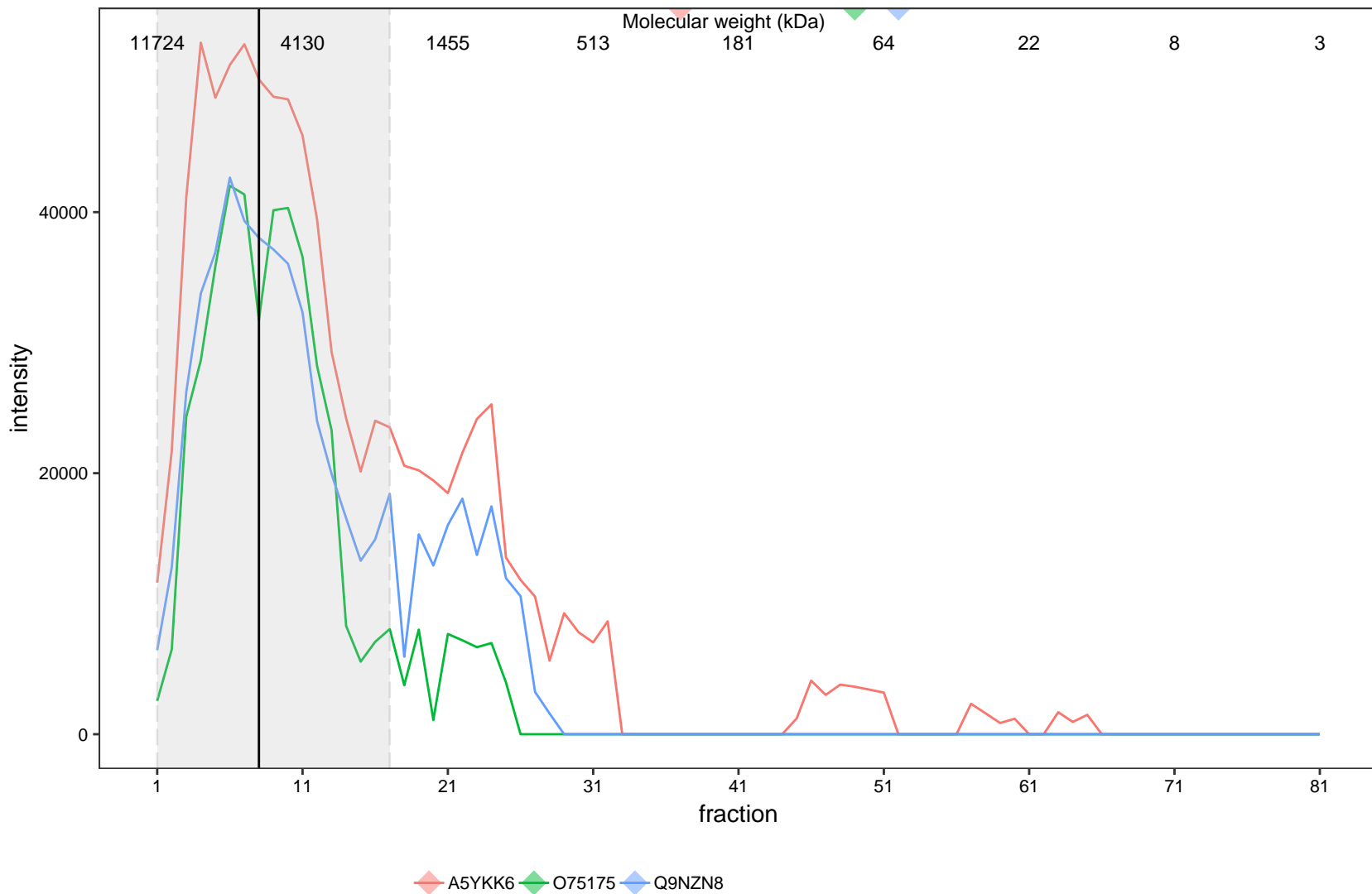

Supplement: Supplementary file 8 — Dataset EV7 [file MSB-15-e8438-s008.zip › feature_plots_string/O75175.pdf]

**O75197**  
**Annotated subunits: 35   Subunits with signal: 7**  
**Max. coeluting subunits: 3   Max. completeness: 0.09**

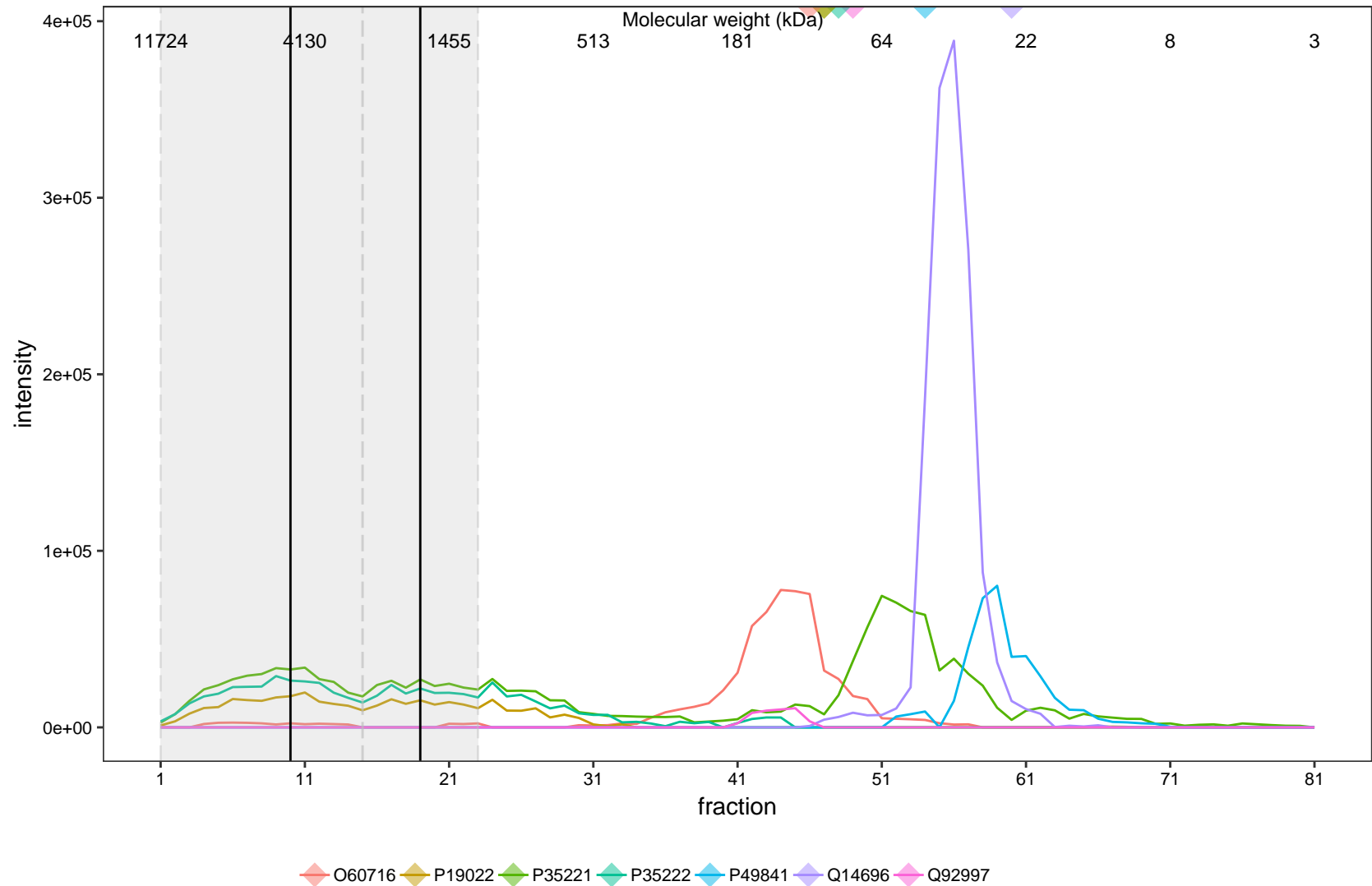

Supplement: Supplementary file 8 — Dataset EV7 [file MSB-15-e8438-s008.zip › feature_plots_string/O75197.pdf]

**O75208**

**Annotated subunits: 9 Subunits with signal: 6**

**Max. coeluting subunits: 3 Max. completeness: 0.33**

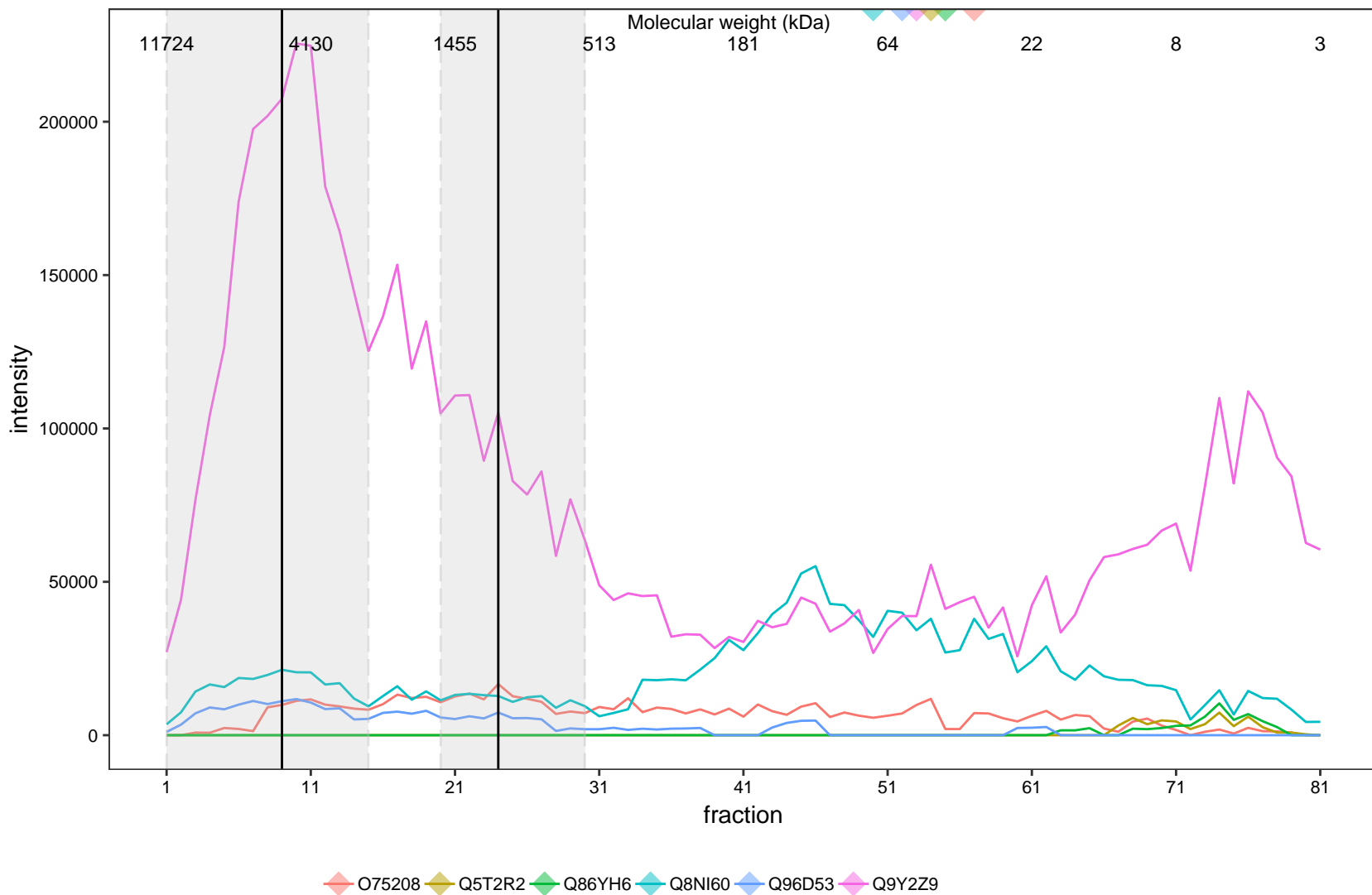

Supplement: Supplementary file 8 — Dataset EV7 [file MSB-15-e8438-s008.zip › feature_plots_string/O75208.pdf]

**O75306**

**Annotated subunits: 57 Subunits with signal: 47**

**Max. coeluting subunits: 30 Max. completeness: 0.53**

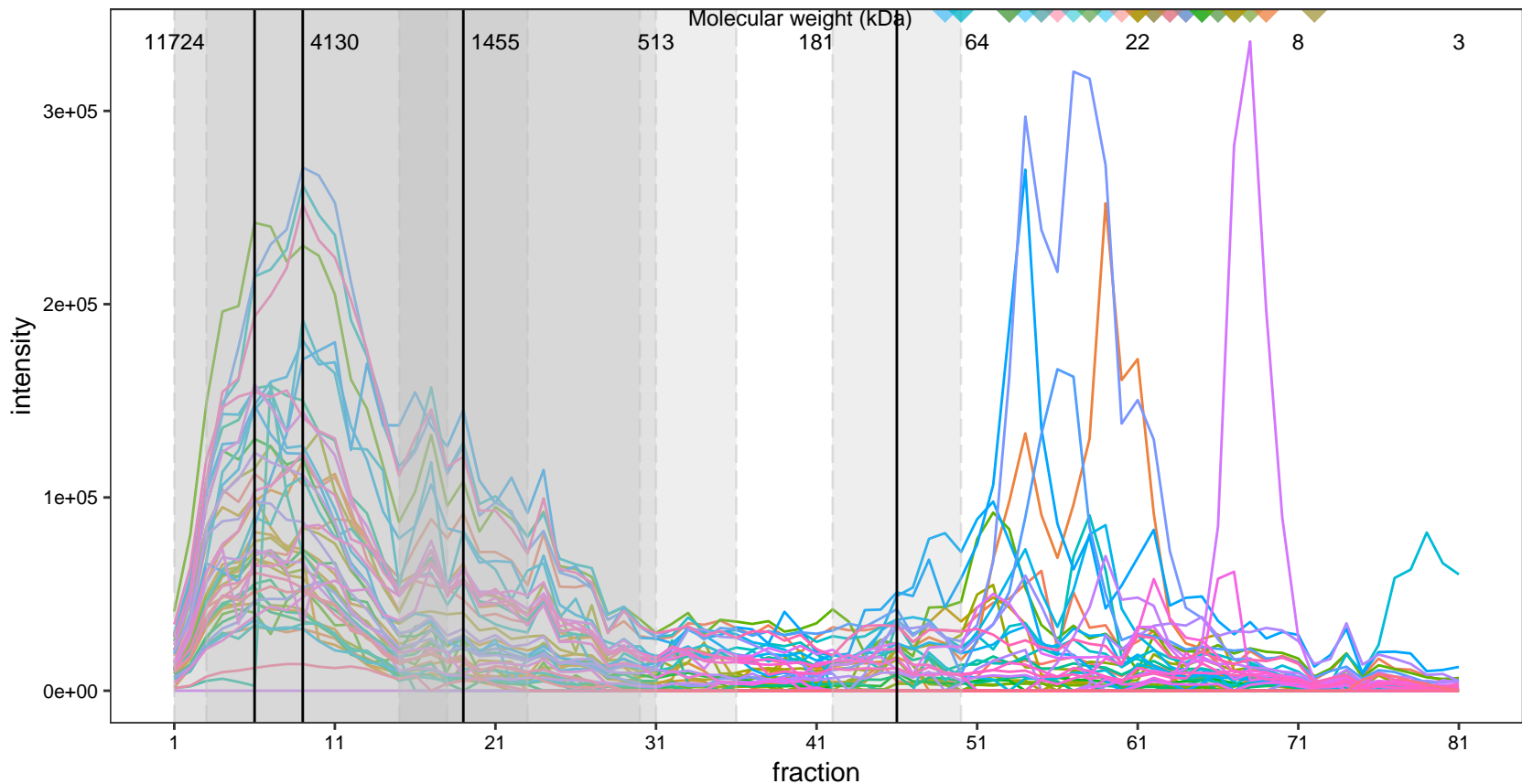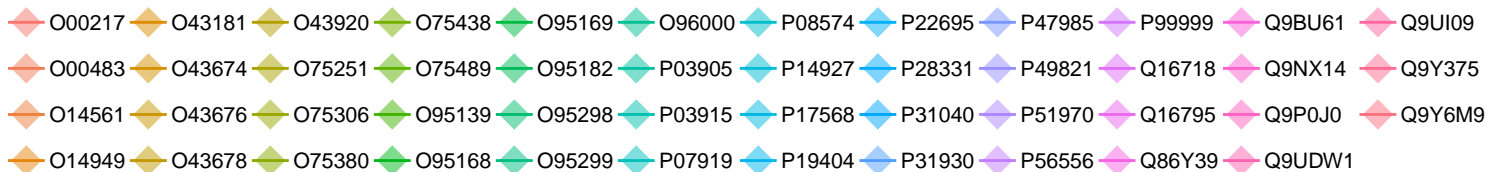

Supplement: Supplementary file 8 — Dataset EV7 [file MSB-15-e8438-s008.zip › feature_plots_string/O75306.pdf]

**O75312**

**Annotated subunits: 21 Subunits with signal: 18**

**Max. coeluting subunits: 8 Max. completeness: 0.38**

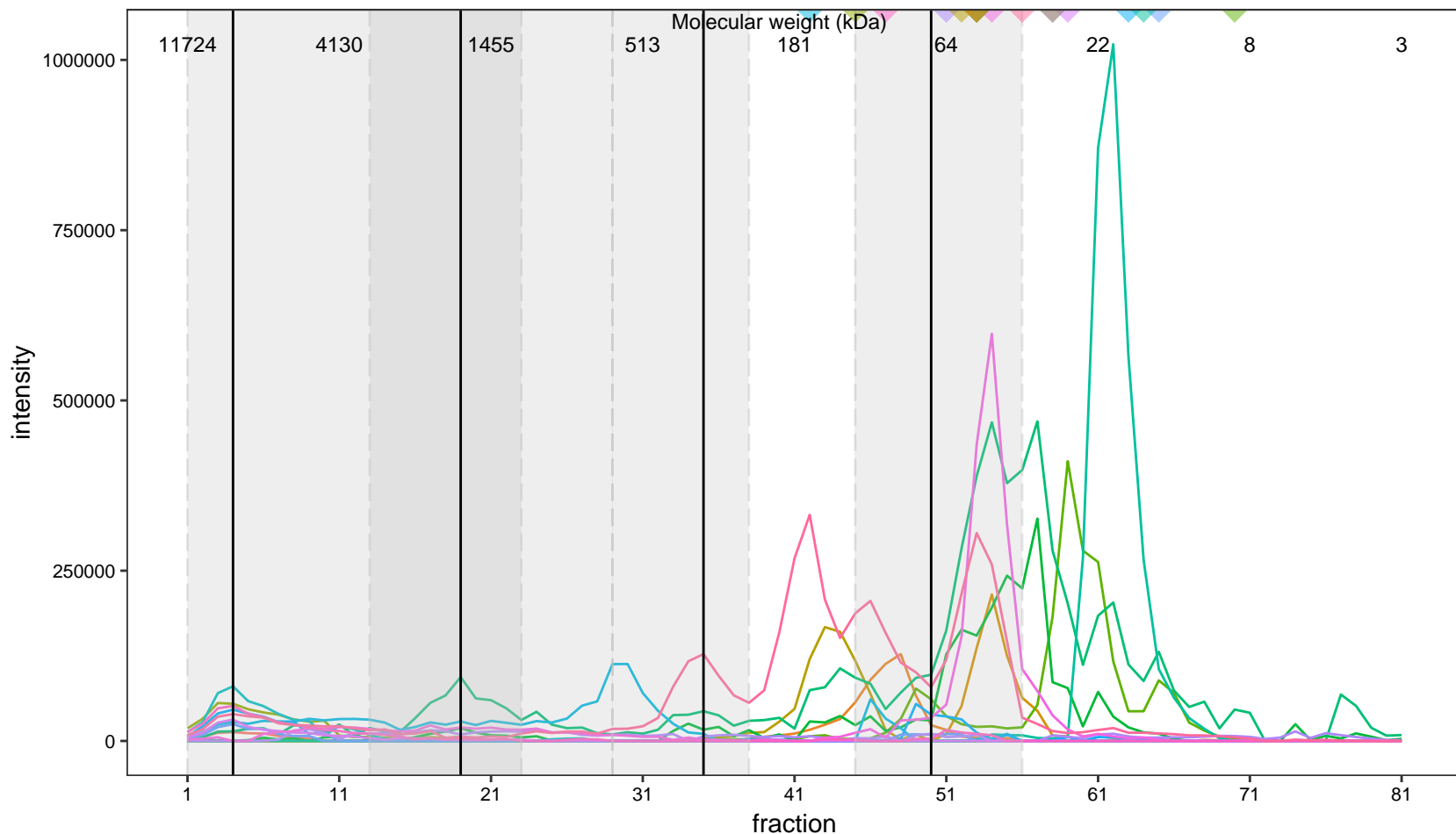

Supplement: Supplementary file 8 — Dataset EV7 [file MSB-15-e8438-s008.zip › feature_plots_string/O75312.pdf]

**O75323**

**Annotated subunits: 4 Subunits with signal: 4**

**Max. coeluting subunits: 2 Max. completeness: 0.5**

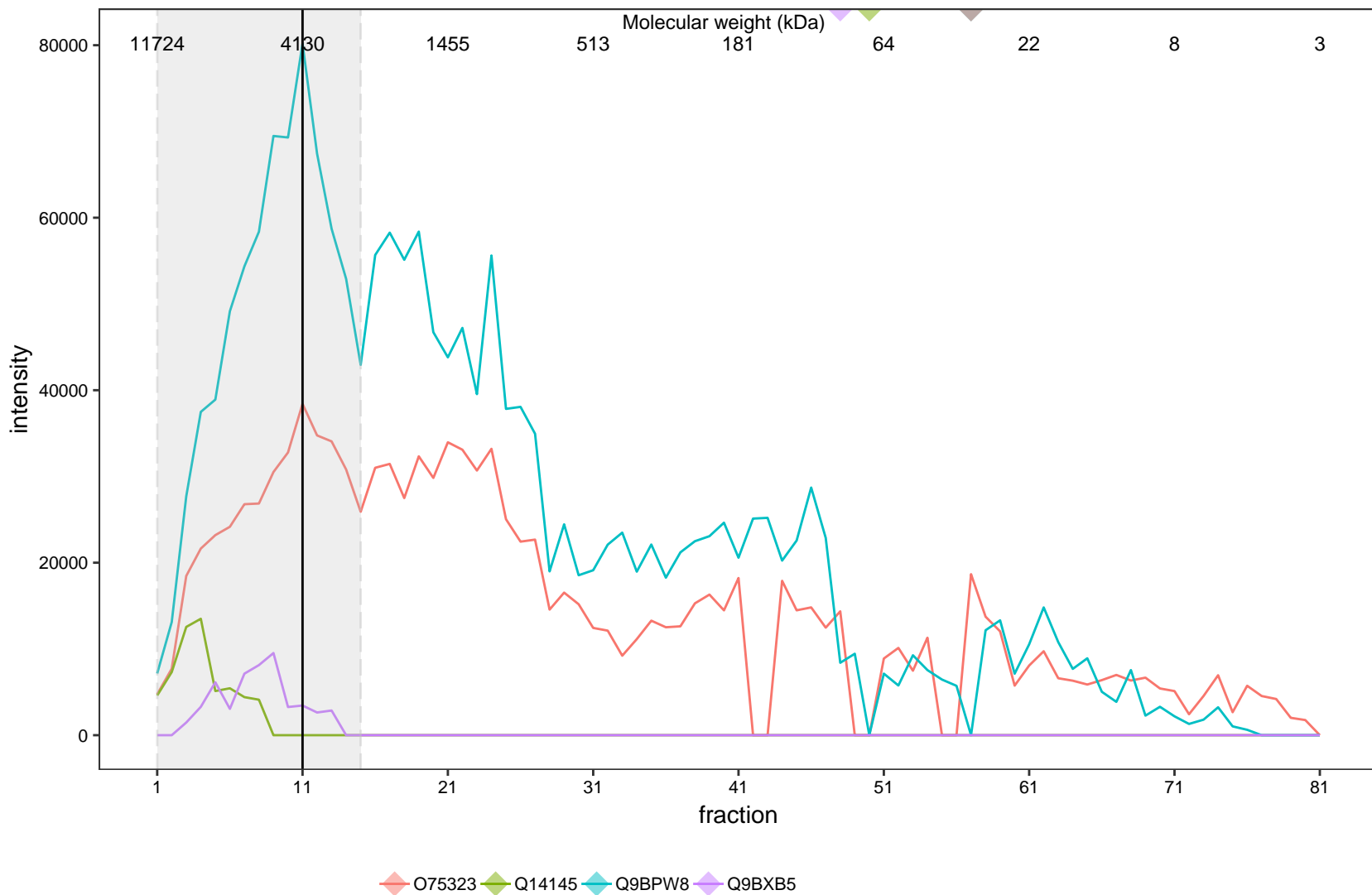

Supplement: Supplementary file 8 — Dataset EV7 [file MSB-15-e8438-s008.zip › feature_plots_string/O75323.pdf]

**O75326**

**Annotated subunits: 4 Subunits with signal: 2**

**Max. coeluting subunits: 2 Max. completeness: 0.5**

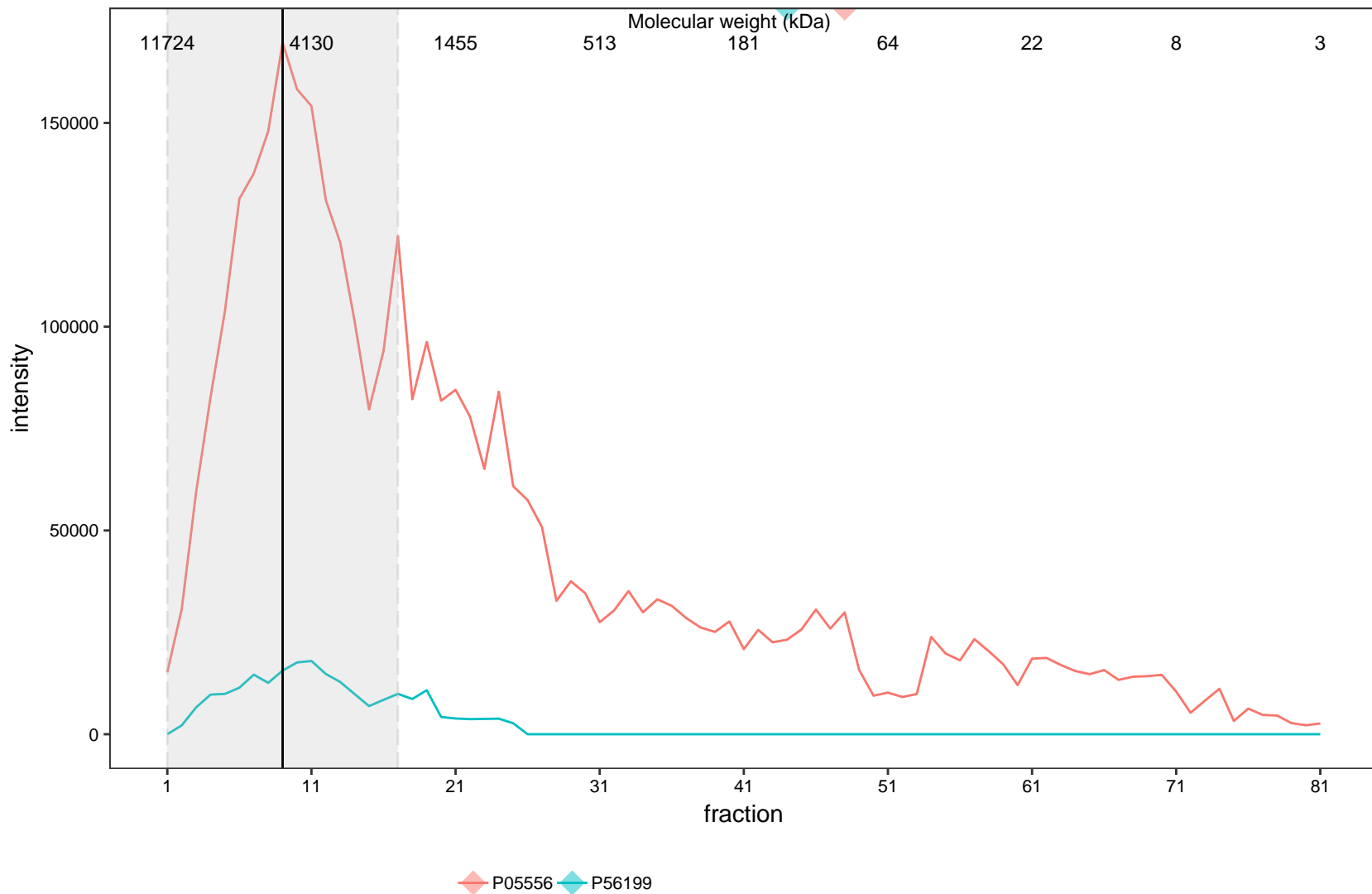

Supplement: Supplementary file 8 — Dataset EV7 [file MSB-15-e8438-s008.zip › feature_plots_string/O75326.pdf]

O75344  
Annotated subunits: 3   Subunits with signal: 2  
Max. coeluting subunits: 2   Max. completeness: 0.67

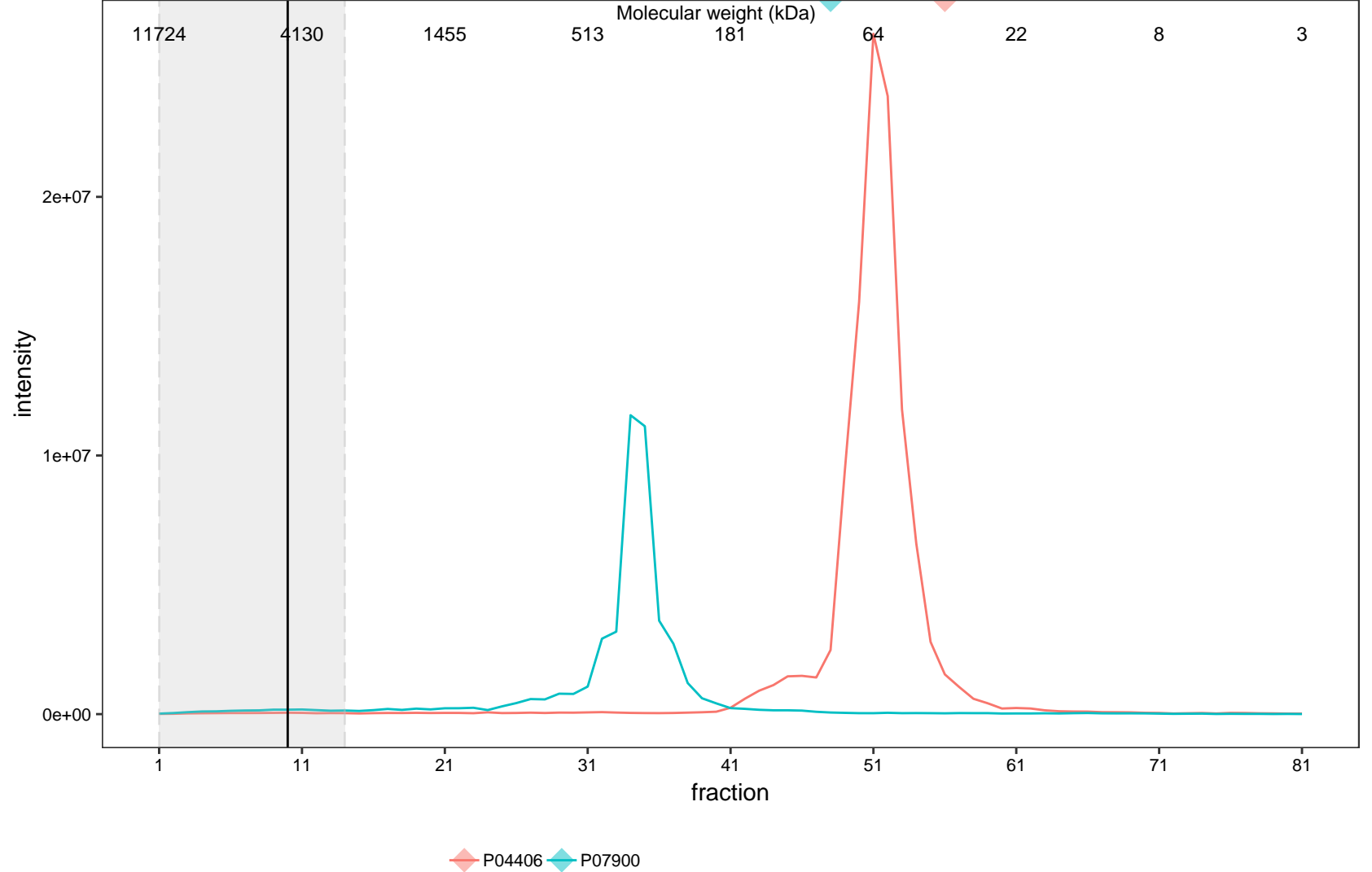

Supplement: Supplementary file 8 — Dataset EV7 [file MSB-15-e8438-s008.zip › feature_plots_string/O75344.pdf]

**O75381**

**Annotated subunits: 16 Subunits with signal: 6**

**Max. coeluting subunits: 2 Max. completeness: 0.12**

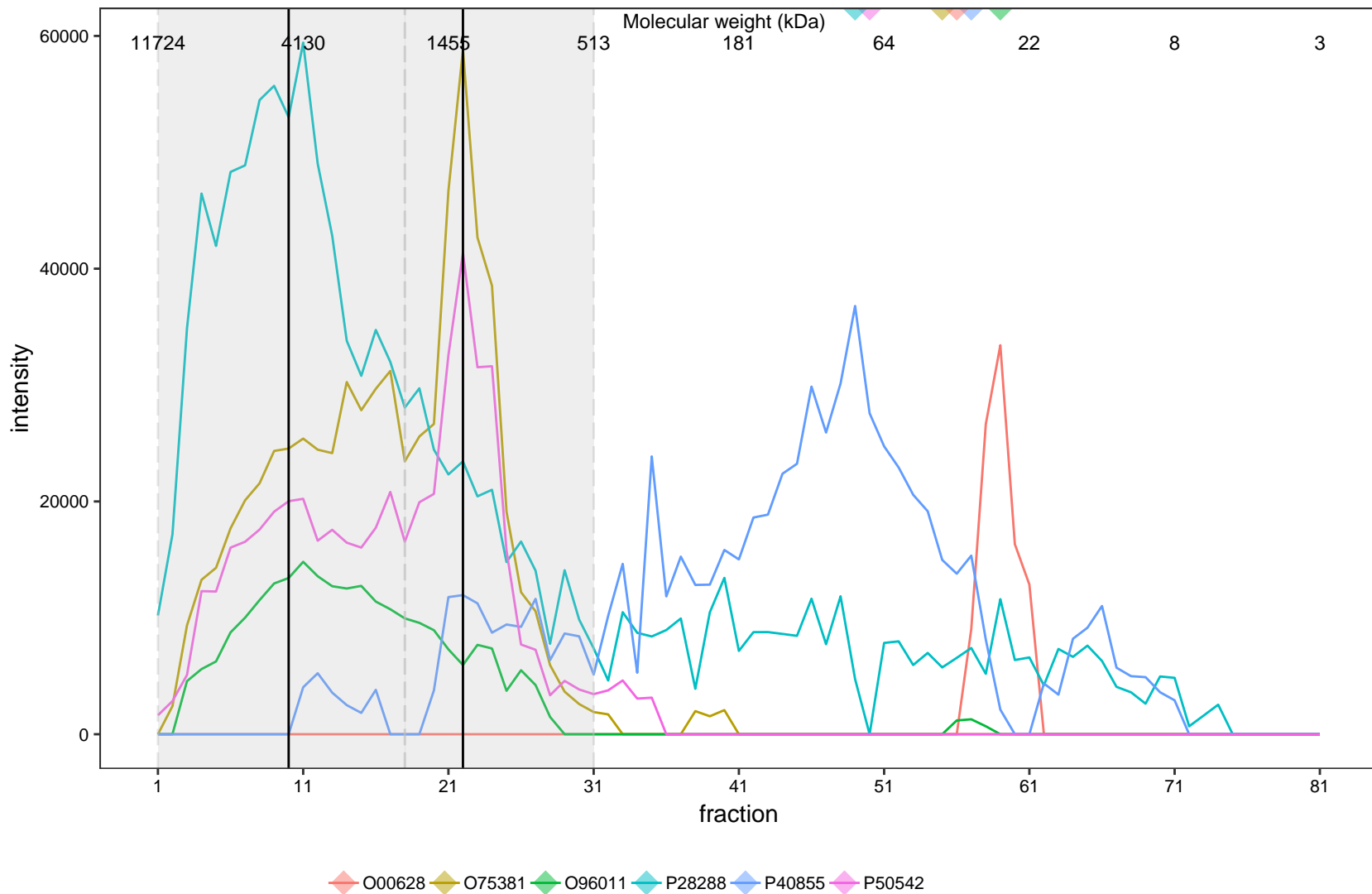

Supplement: Supplementary file 8 — Dataset EV7 [file MSB-15-e8438-s008.zip › feature_plots_string/O75381.pdf]

**O75400**

**Annotated subunits: 13 Subunits with signal: 10**

**Max. coeluting subunits: 3 Max. completeness: 0.23**

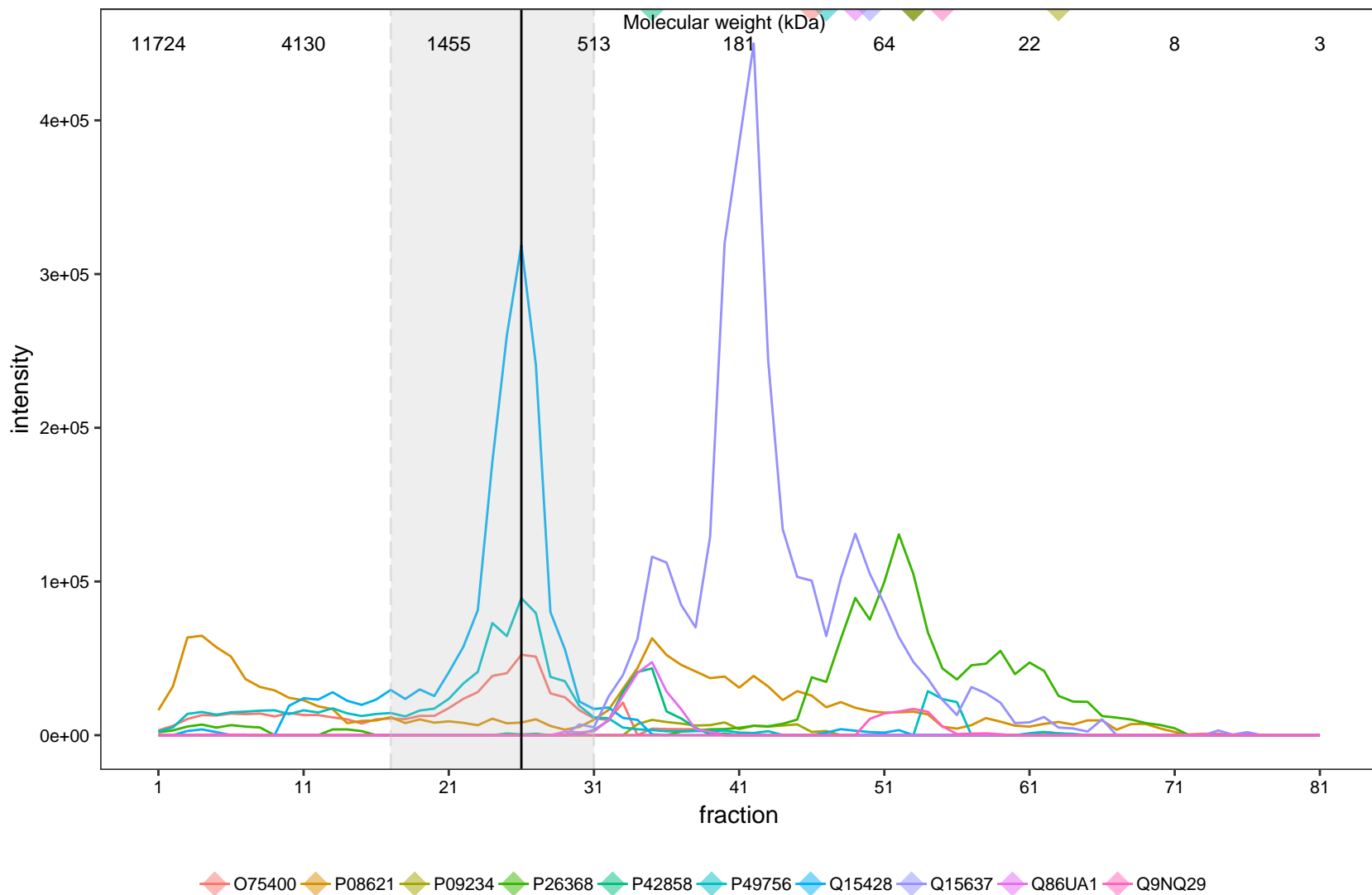

Supplement: Supplementary file 8 — Dataset EV7 [file MSB-15-e8438-s008.zip › feature_plots_string/O75400.pdf]

O75460  
Annotated subunits: 13   Subunits with signal: 6  
Max. coeluting subunits: 3   Max. completeness: 0.23

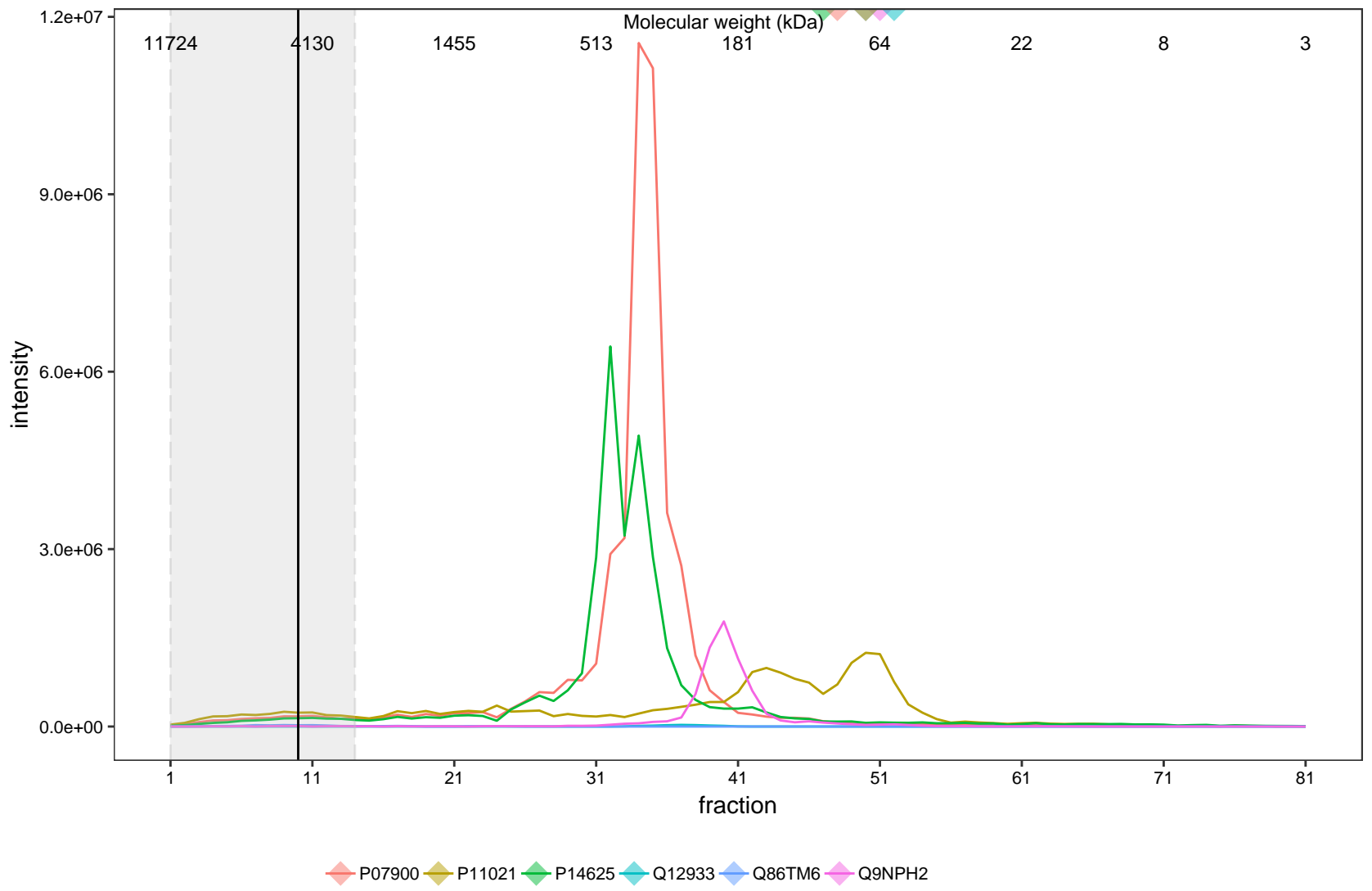

Supplement: Supplementary file 8 — Dataset EV7 [file MSB-15-e8438-s008.zip › feature_plots_string/O75460.pdf]

**O75477**

**Annotated subunits: 2 Subunits with signal: 2**

**Max. coeluting subunits: 2 Max. completeness: 1**

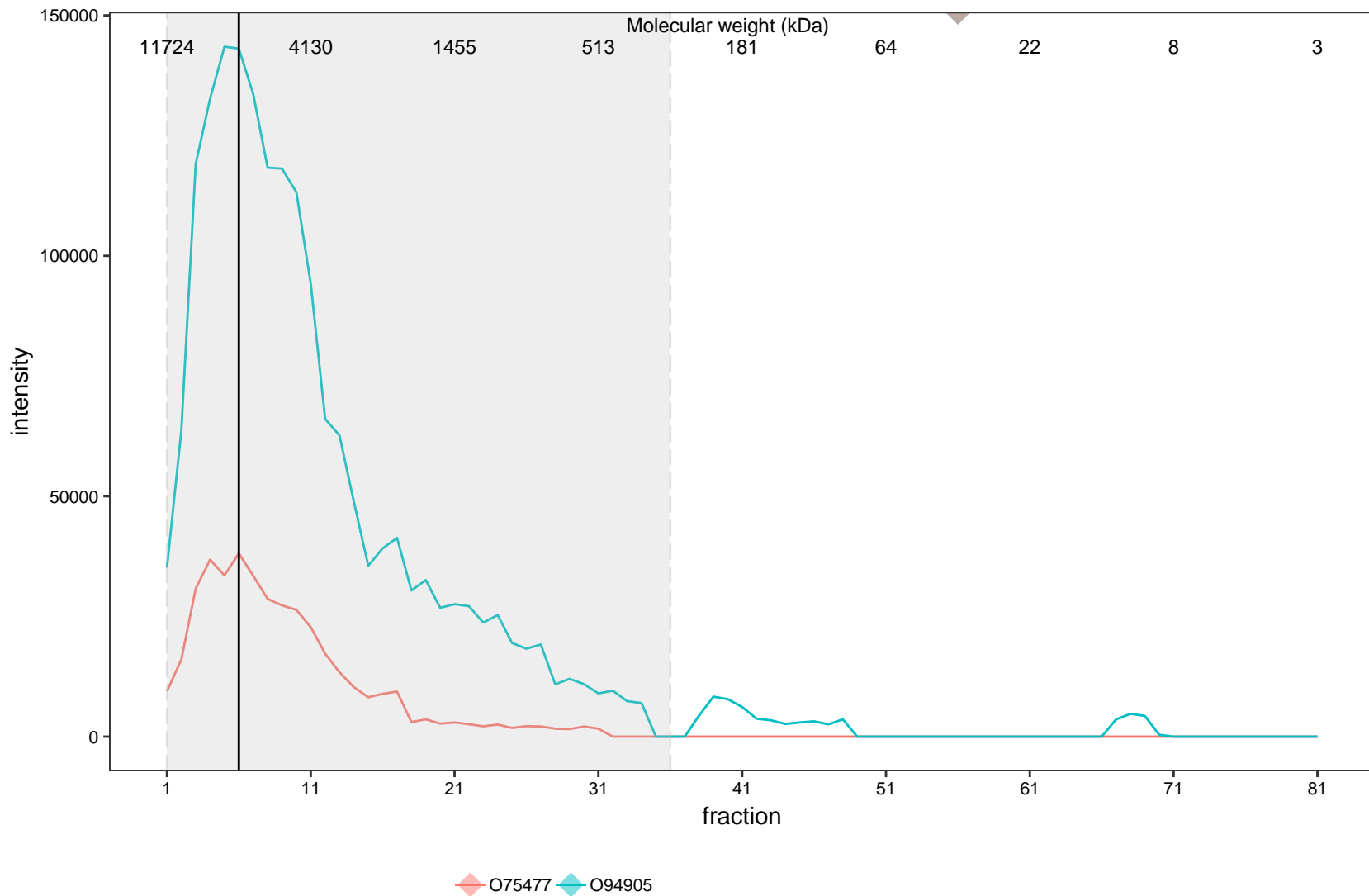

Supplement: Supplementary file 8 — Dataset EV7 [file MSB-15-e8438-s008.zip › feature_plots_string/O75477.pdf]

**O75496**

**Annotated subunits: 62 Subunits with signal: 47**

**Max. coeluting subunits: 22 Max. completeness: 0.35**

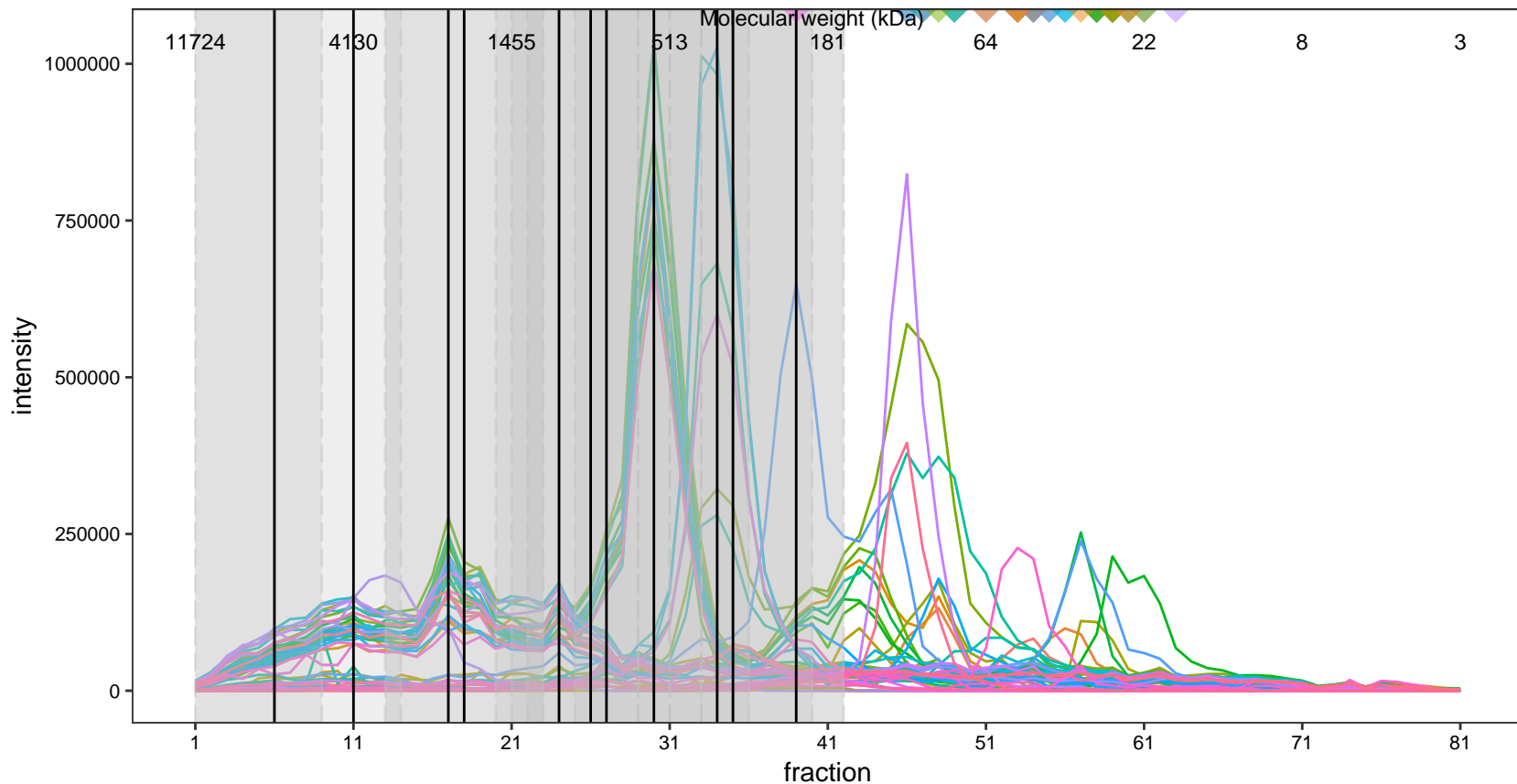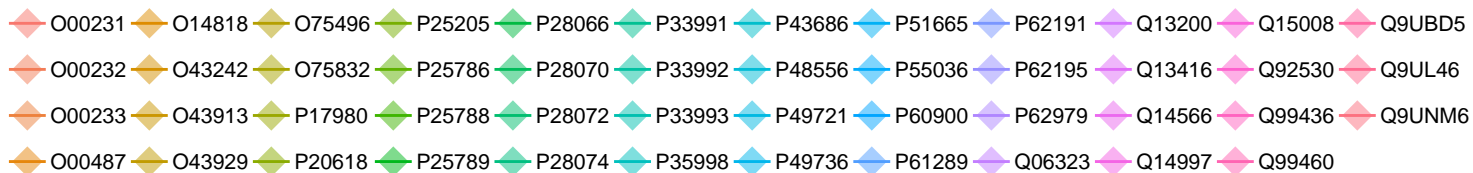

Supplement: Supplementary file 8 — Dataset EV7 [file MSB-15-e8438-s008.zip › feature_plots_string/O75496.pdf]

**O75529**

**Annotated subunits: 30 Subunits with signal: 9**

**Max. coeluting subunits: 6 Max. completeness: 0.2**

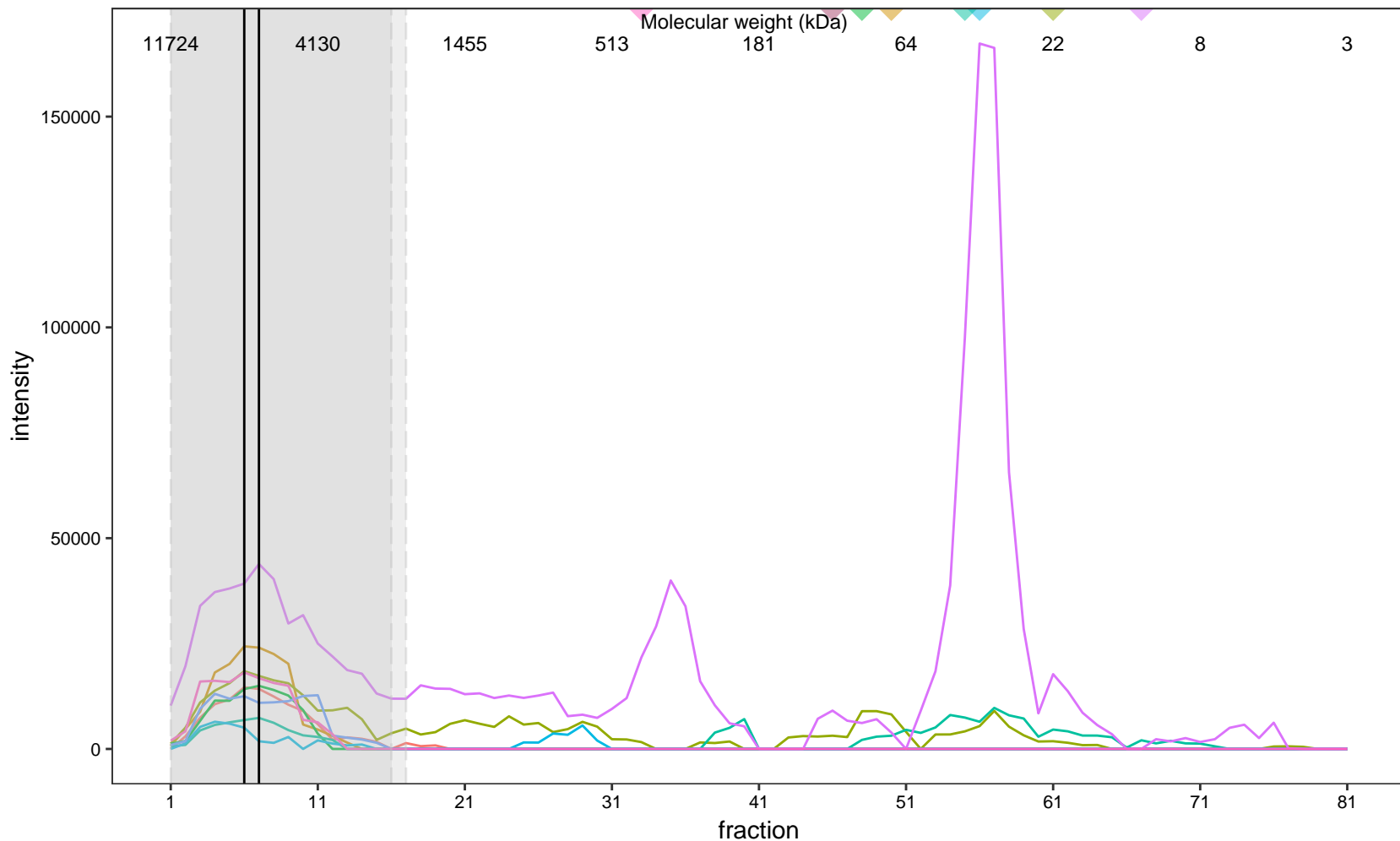

Supplement: Supplementary file 8 — Dataset EV7 [file MSB-15-e8438-s008.zip › feature_plots_string/O75529.pdf]

**O75531**

**Annotated subunits: 11 Subunits with signal: 8**

**Max. coeluting subunits: 4 Max. completeness: 0.36**

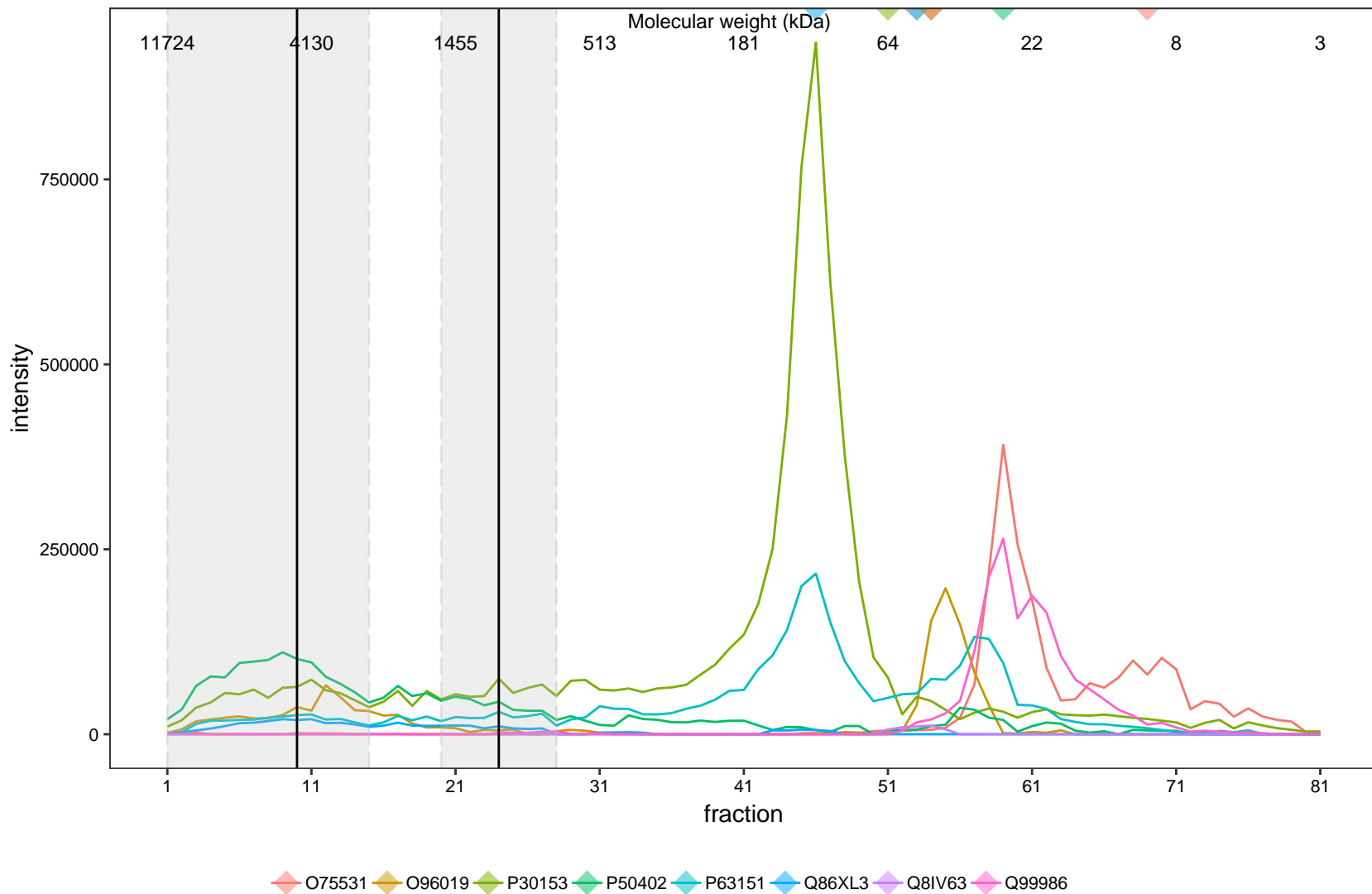

Supplement: Supplementary file 8 — Dataset EV7 [file MSB-15-e8438-s008.zip › feature_plots_string/O75531.pdf]

**O75534**

**Annotated subunits: 12 Subunits with signal: 8**

**Max. coeluting subunits: 5 Max. completeness: 0.42**

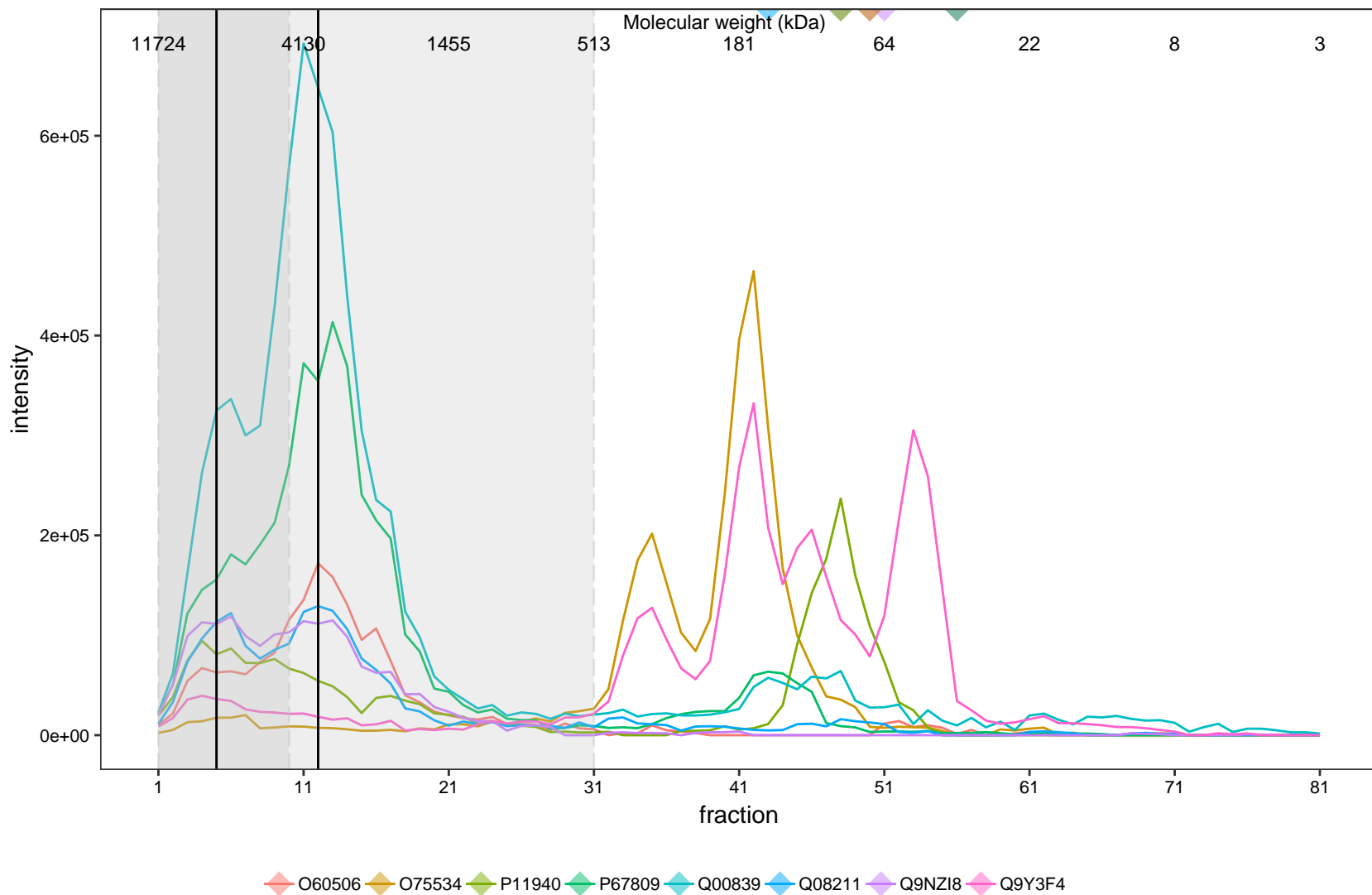

Supplement: Supplementary file 8 — Dataset EV7 [file MSB-15-e8438-s008.zip › feature_plots_string/O75534.pdf]

O75558  
Annotated subunits: 4   Subunits with signal: 2  
Max. coeluting subunits: 2   Max. completeness: 0.5

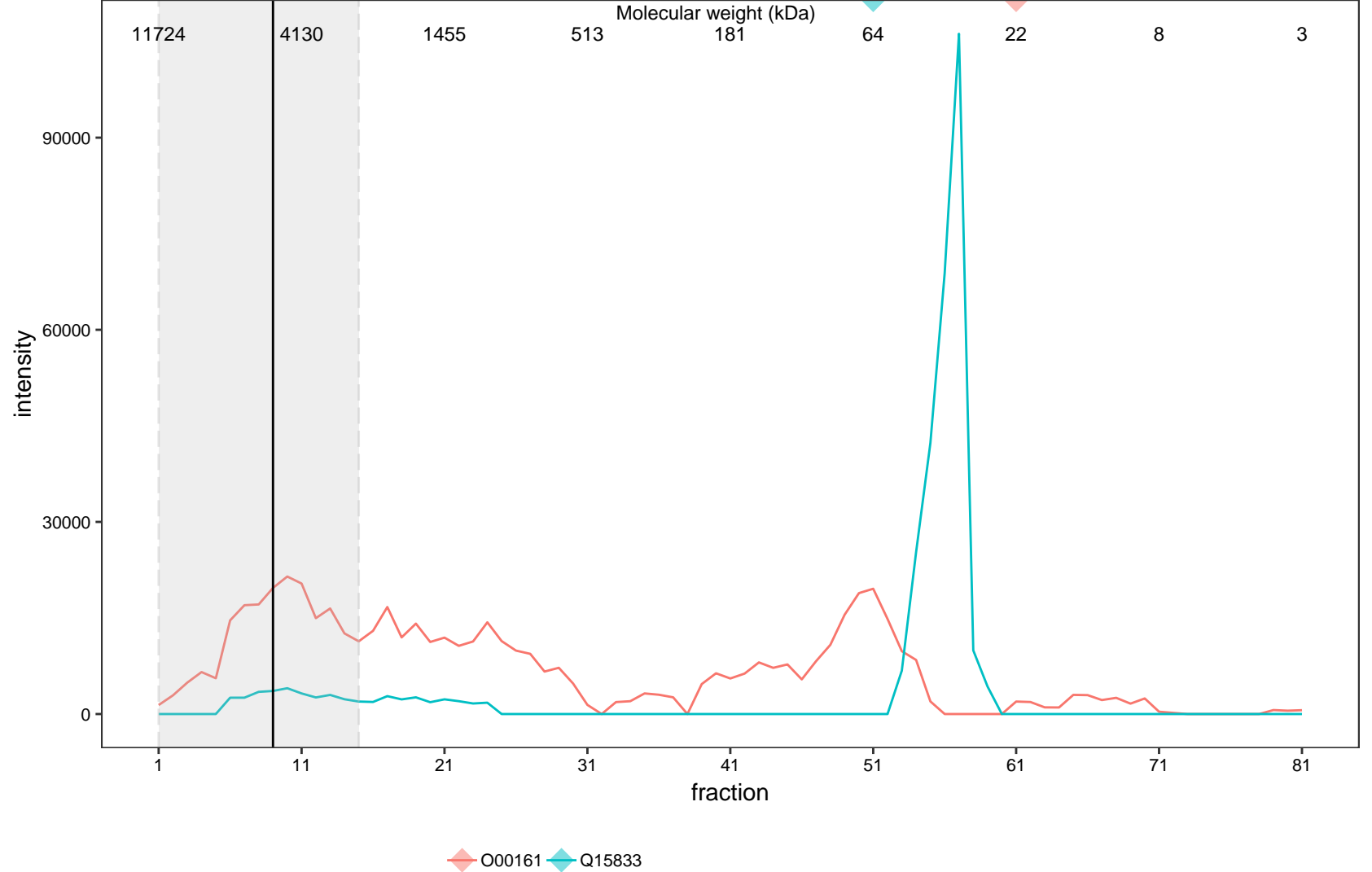

Supplement: Supplementary file 8 — Dataset EV7 [file MSB-15-e8438-s008.zip › feature_plots_string/O75558.pdf]

**O75575**

**Annotated subunits: 16 Subunits with signal: 9**

**Max. coeluting subunits: 5 Max. completeness: 0.31**

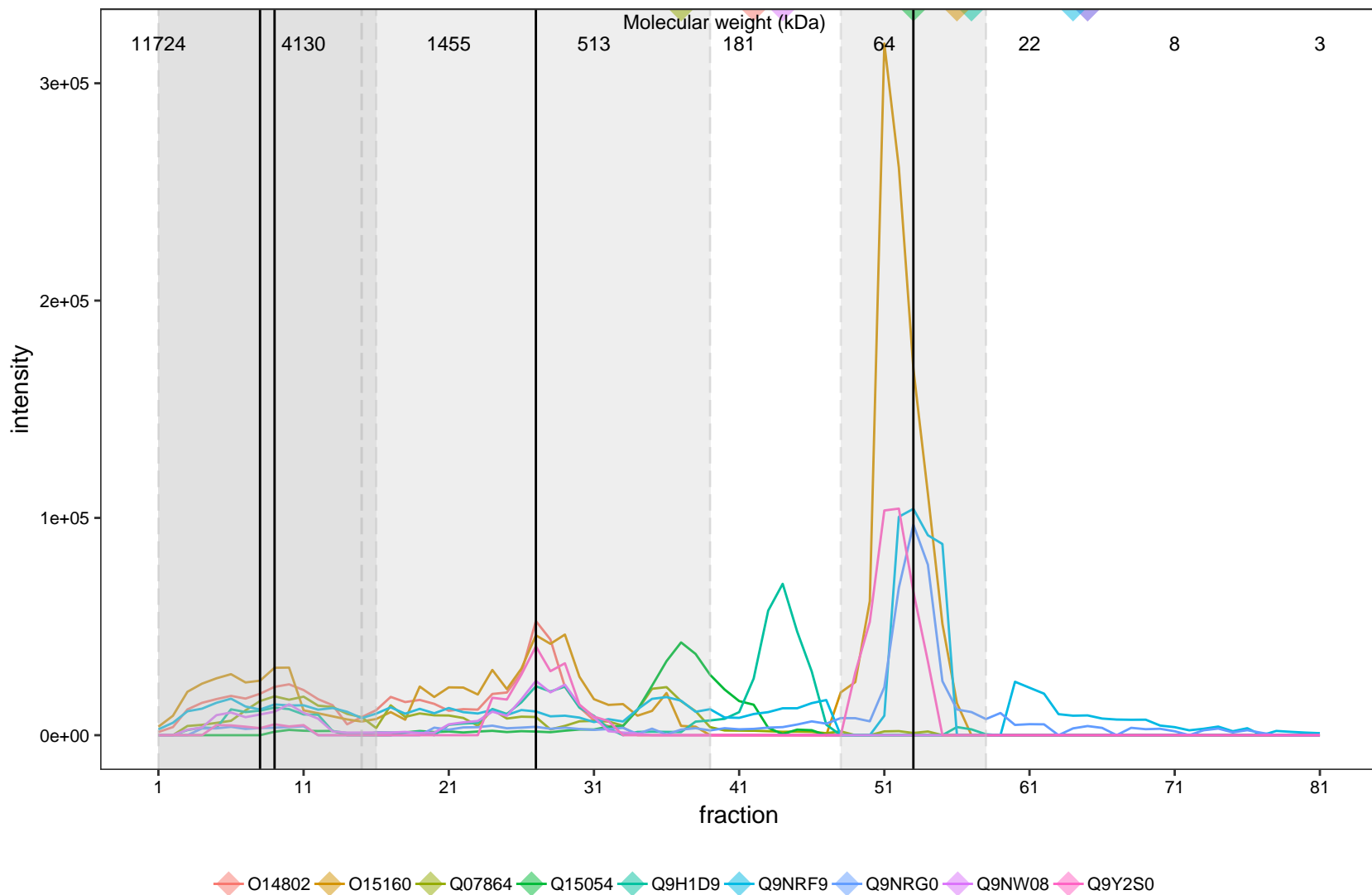

Supplement: Supplementary file 8 — Dataset EV7 [file MSB-15-e8438-s008.zip › feature_plots_string/O75575.pdf]

**O75663**

**Annotated subunits: 7 Subunits with signal: 6**

**Max. coeluting subunits: 2 Max. completeness: 0.29**

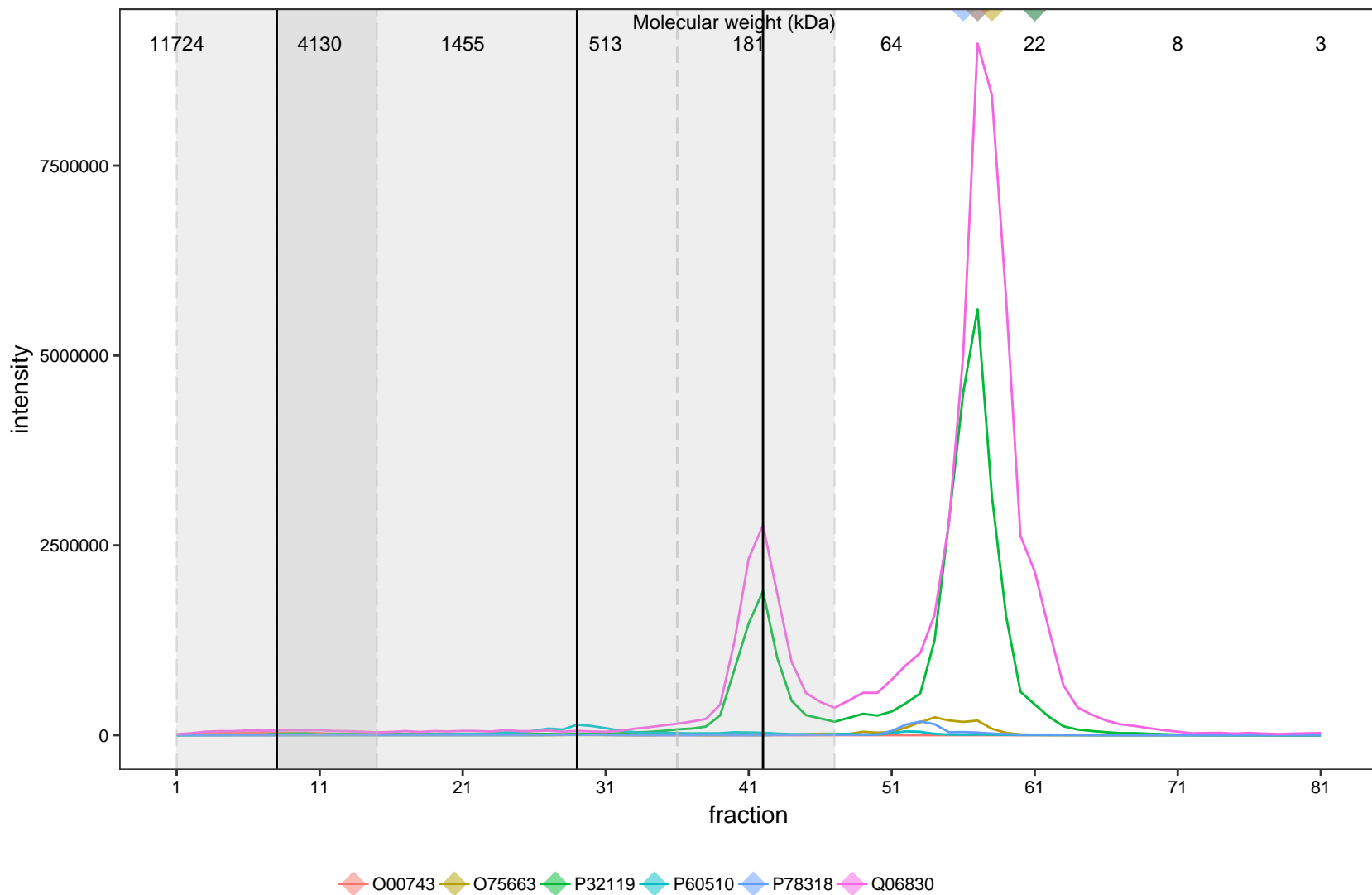

Supplement: Supplementary file 8 — Dataset EV7 [file MSB-15-e8438-s008.zip › feature_plots_string/O75663.pdf]
