# Supplementary material for: Complex‐centric proteome profiling by SEC‐SWATH‐MS
Source: Mol Syst Biol. 2019 Jan 14;15(1):e8438. doi: 10.15252/msb.20188438 (PMC6346213; doi:10.15252/msb.20188438)

Feature ID 1

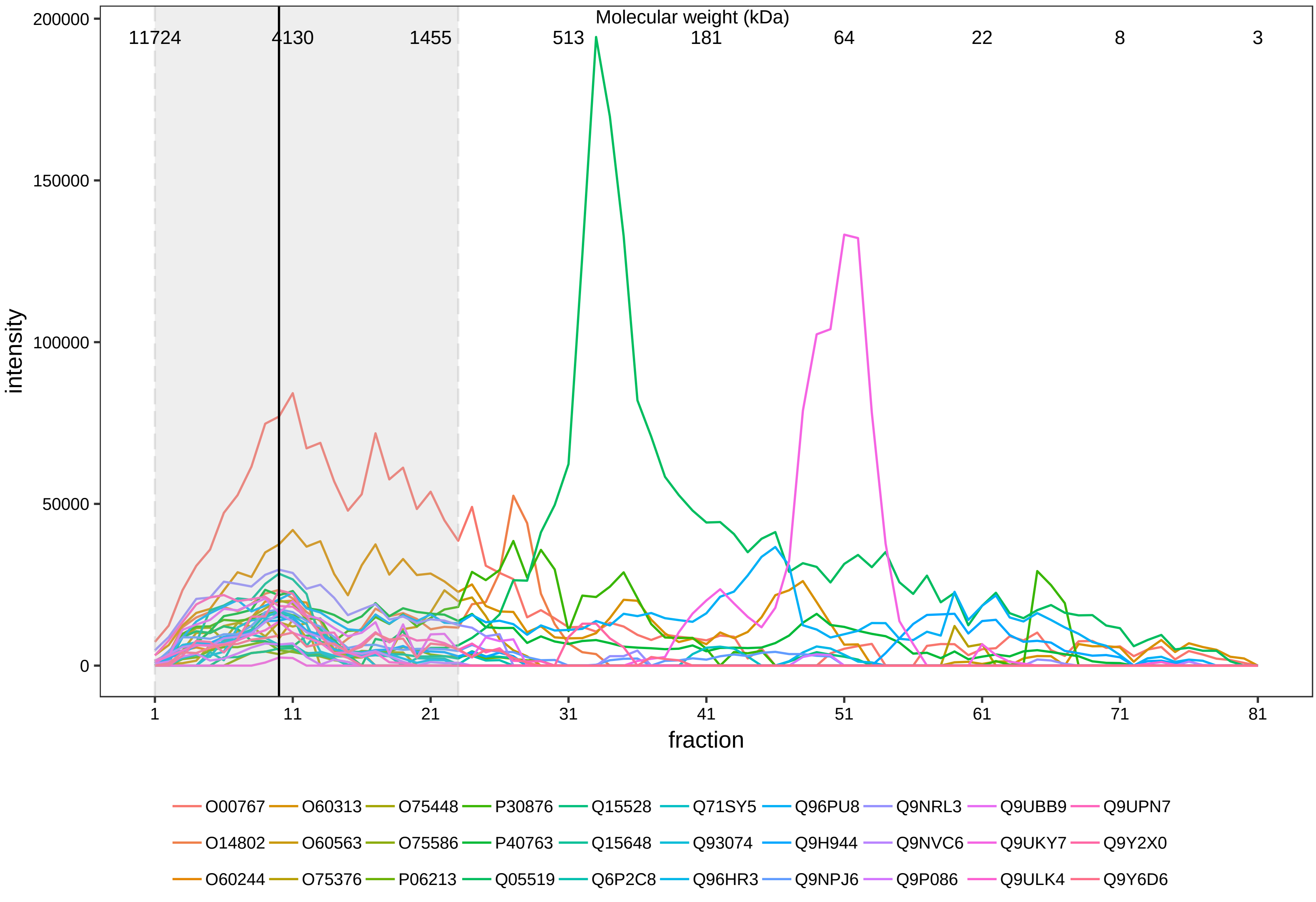

Molecular weight (kDa)
11724
4130
1455
513
181
64
22
8
3
200000
150000
100000
50000
0
intensity
1
11
21
31
41
51
61
71
81
fraction
O00767
O60313
O75448
P30876
Q15528
Q71SY5
Q96PU8
Q9NRL3
Q9UBB9
Q9UPN7
O14802
O60563
O75586
P40763
Q15648
Q93074
Q9H944
Q9NVC6
Q9UKY7
Q9Y2X0
O60244
O75376
P06213
Q05519
Q6P2C8
Q96HR3
Q9NPJ6
Q9P086
Q9ULK4
Q9Y6D6

Feature ID 2

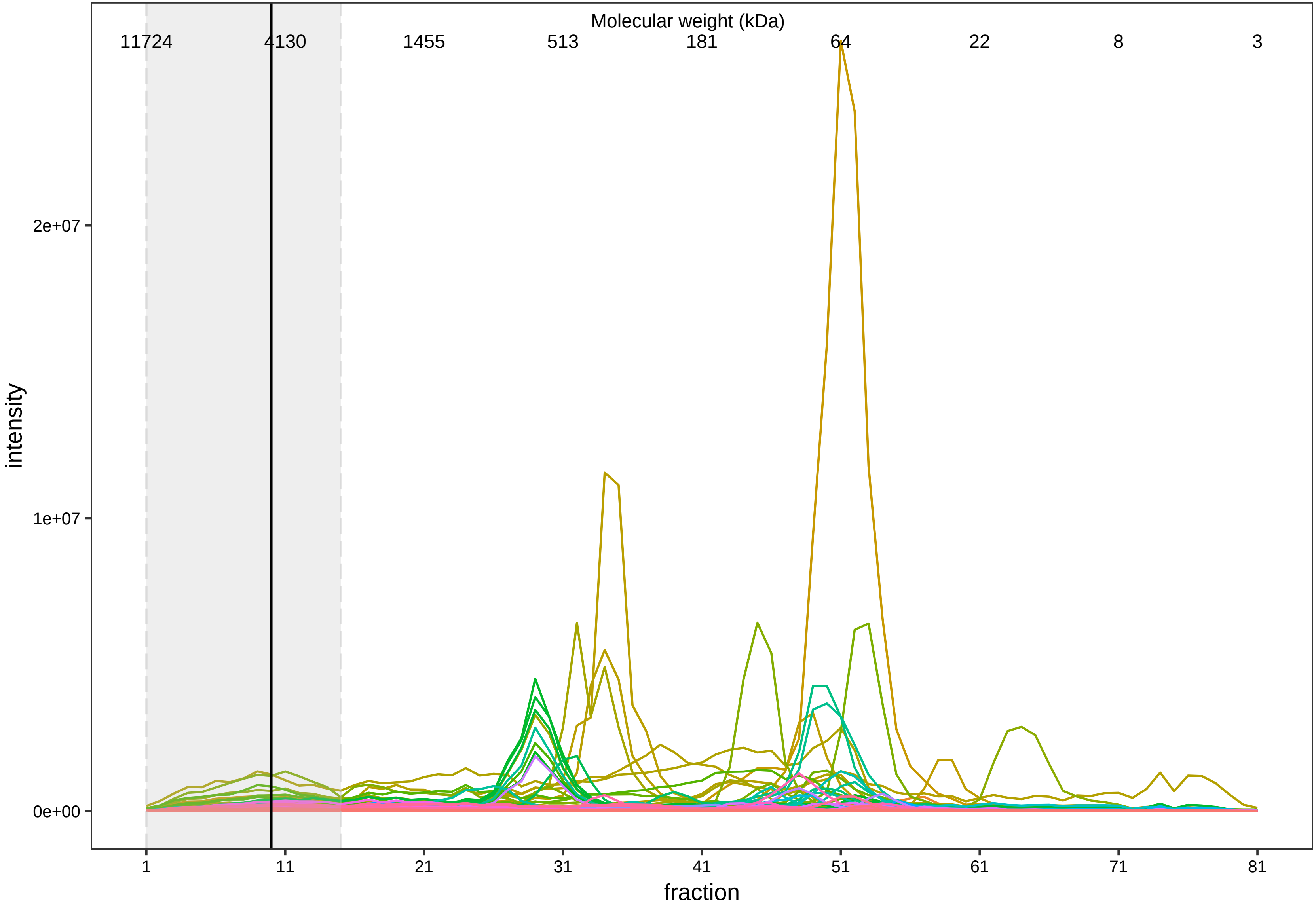

Molecular weight (kDa)
11724
4130
1455
513
181
64
22
8
3
2e+07
1e+07
0e+00
intensity
1
11
21
31
41
51
61
71
81
fraction

Feature ID 3

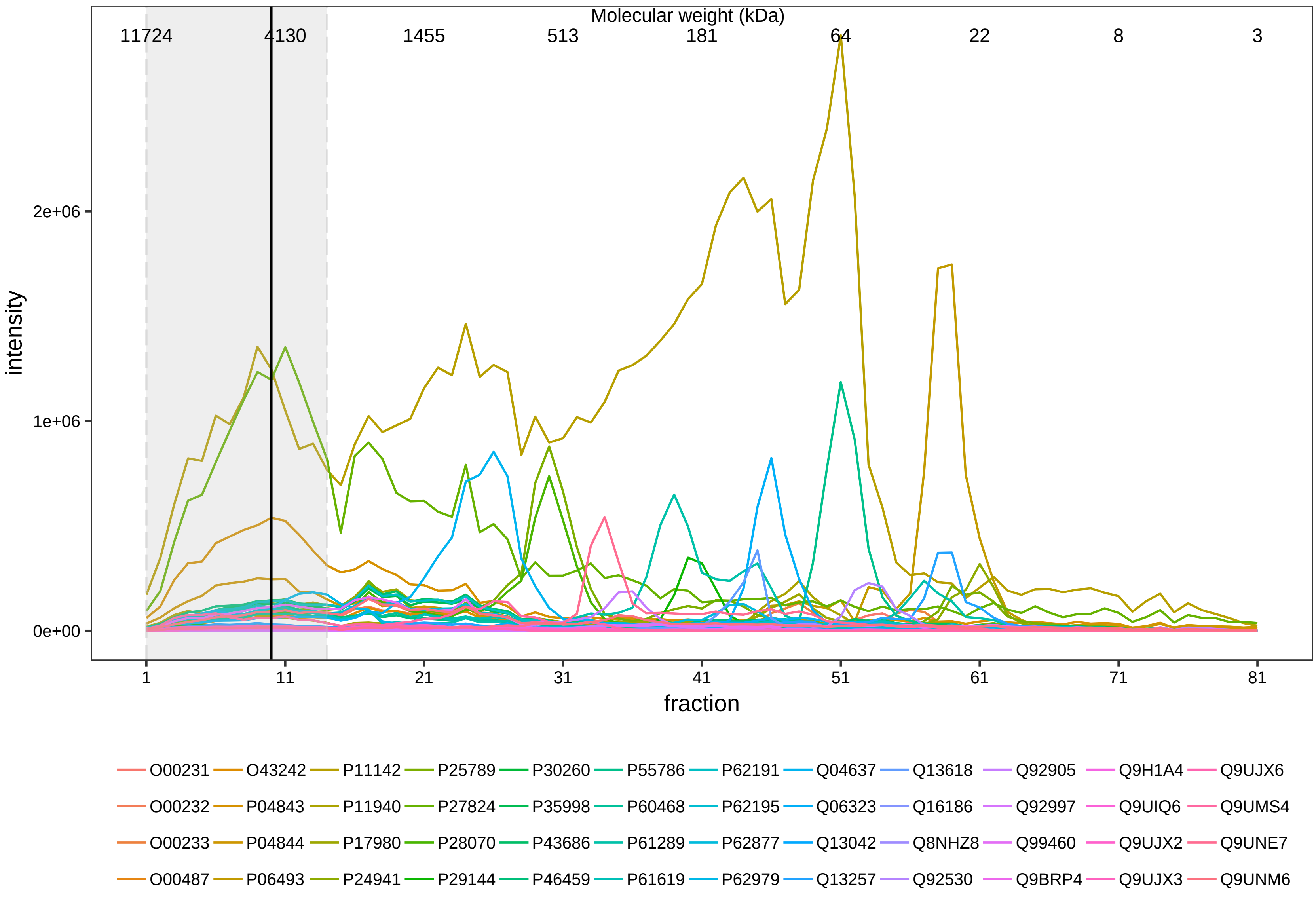
Molecular weight (kDa)
11724
4130
1455
513
181
64
22
8
3
intensity
2e+06
1e+06
0e+00
fraction
1
11
21
31
41
51
61
71
81
O00231 O43242 P11142 P25789 P30260 P55786 P62191 Q04637 Q13618 Q92905 Q9H1A4 Q9UJX6
O00232 P04843 P11940 P27824 P35998 P60468 P62195 Q06323 Q16186 Q92997 Q9UIQ6 Q9UMS4
O00233 P04844 P17980 P28070 P43686 P61289 P62877 Q13042 Q8NHZ8 Q99460 Q9UJX2 Q9UNE7
O00487 P06493 P24941 P29144 P46459 P61619 P62979 Q13257 Q92530 Q9BRP4 Q9UJX3 Q9UNM6

Feature ID 4

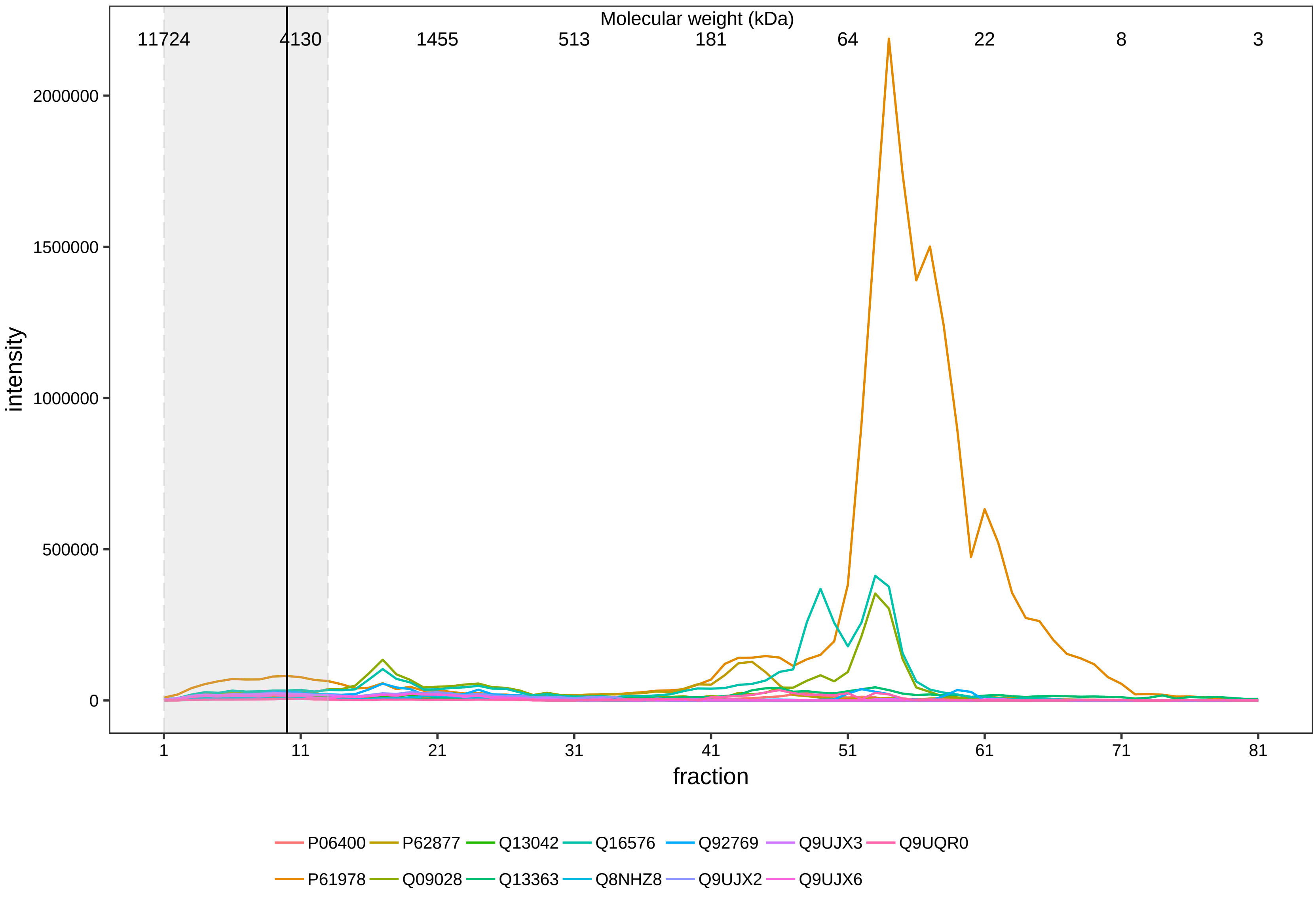

Molecular weight (kDa)
11724
4130
1455
513
181
64
22
8
3
2000000
1500000
1000000
500000
0
intensity
1
11
21
31
41
51
61
71
81
fraction
P06400
P62877
Q13042
Q16576
Q92769
Q9UJX3
Q9UQR0
P61978
Q09028
Q13363
Q8NHZ8
Q9UJX2
Q9UJX6

Feature ID 5

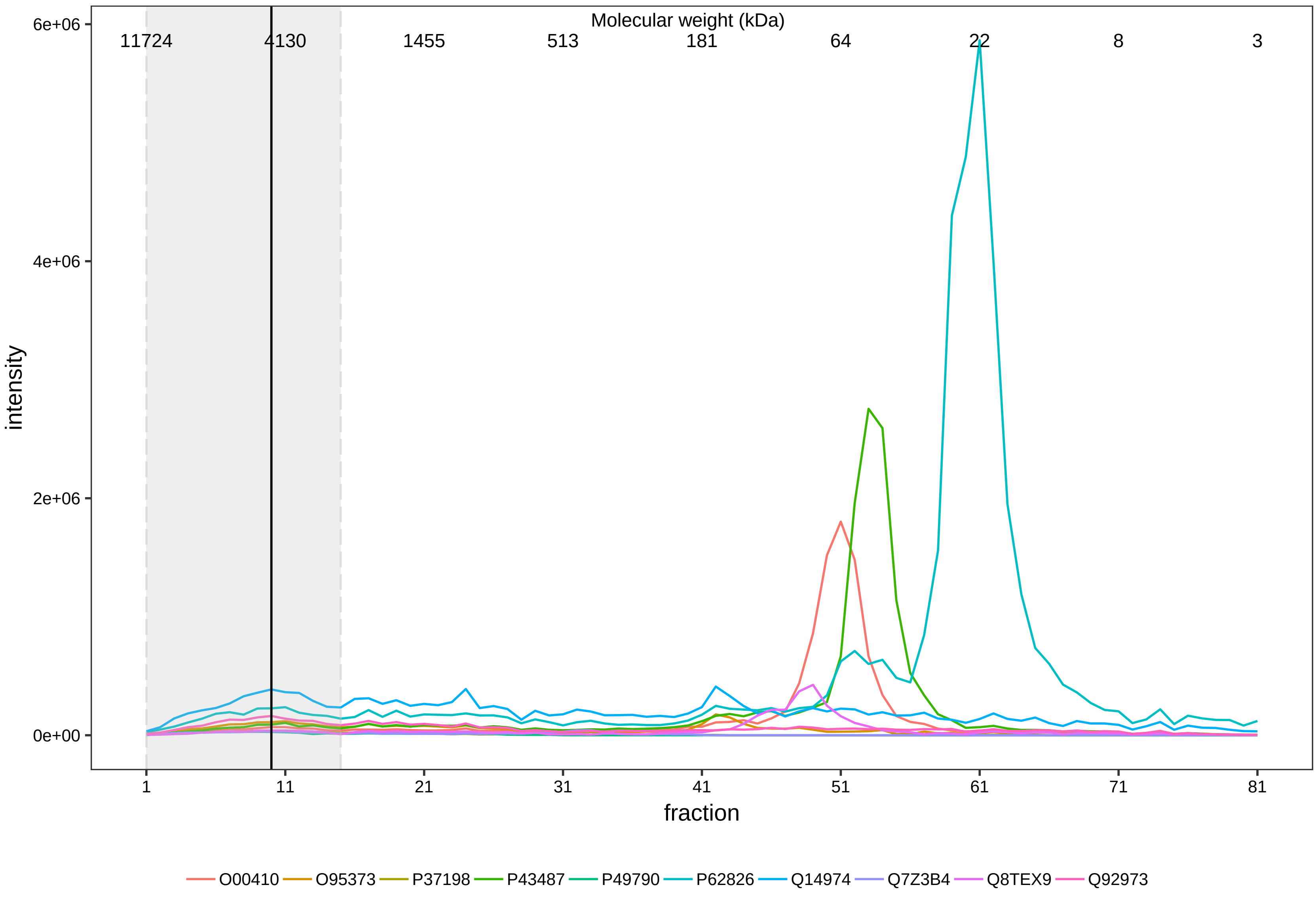
Molecular weight (kDa)
11724
4130
1455
513
181
64
22
8
3
6e+06
4e+06
2e+06
0e+00
intensity
1
11
21
31
41
51
61
71
81
fraction
O00410
O95373
P37198
P43487
P49790
P62826
Q14974
Q7Z3B4
Q8TEX9
Q92973

Feature ID 6

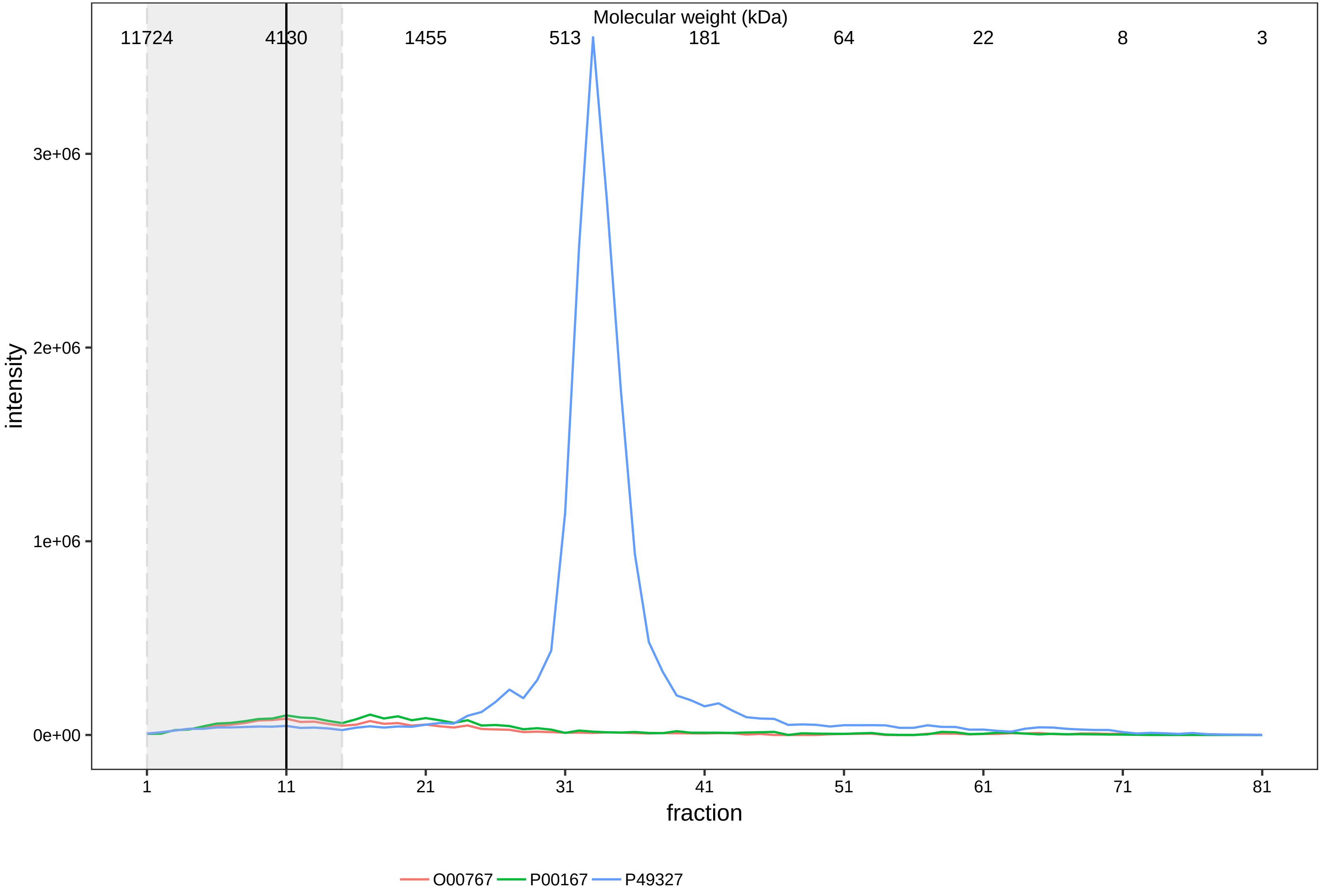

Molecular weight (kDa)
11724
4130
1455
513
181
64
22
8
3
3e+06
2e+06
1e+06
0e+00
intensity
1
11
21
31
41
51
61
71
81
fraction
O00767
P00167
P49327

Feature ID 7

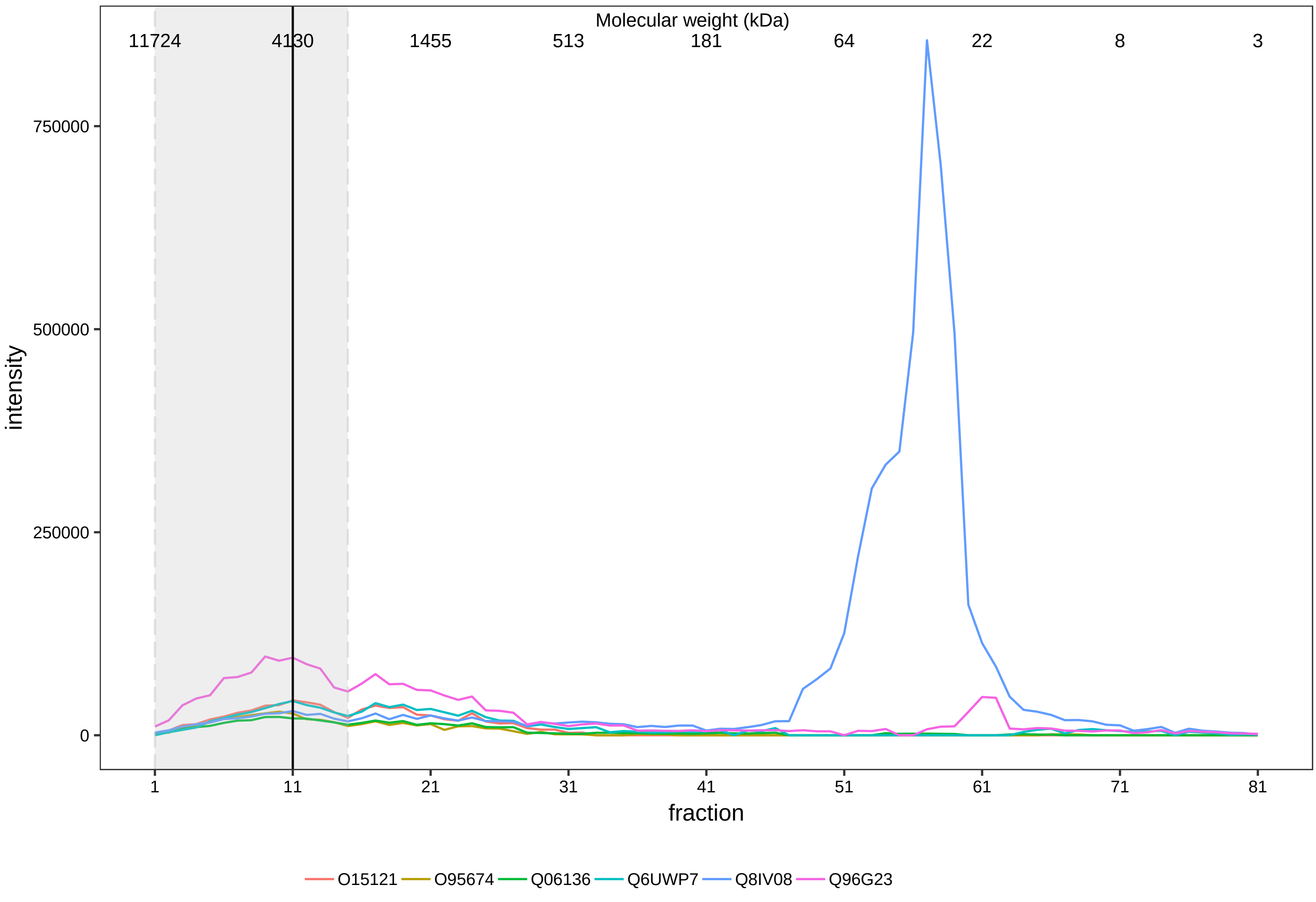

Molecular weight (kDa)
11724
4130
1455
513
181
64
22
8
3
intensity
750000
500000
250000
0
fraction
1
11
21
31
41
51
61
71
81
O15121
O95674
Q06136
Q6UWP7
Q8IV08
Q96G23

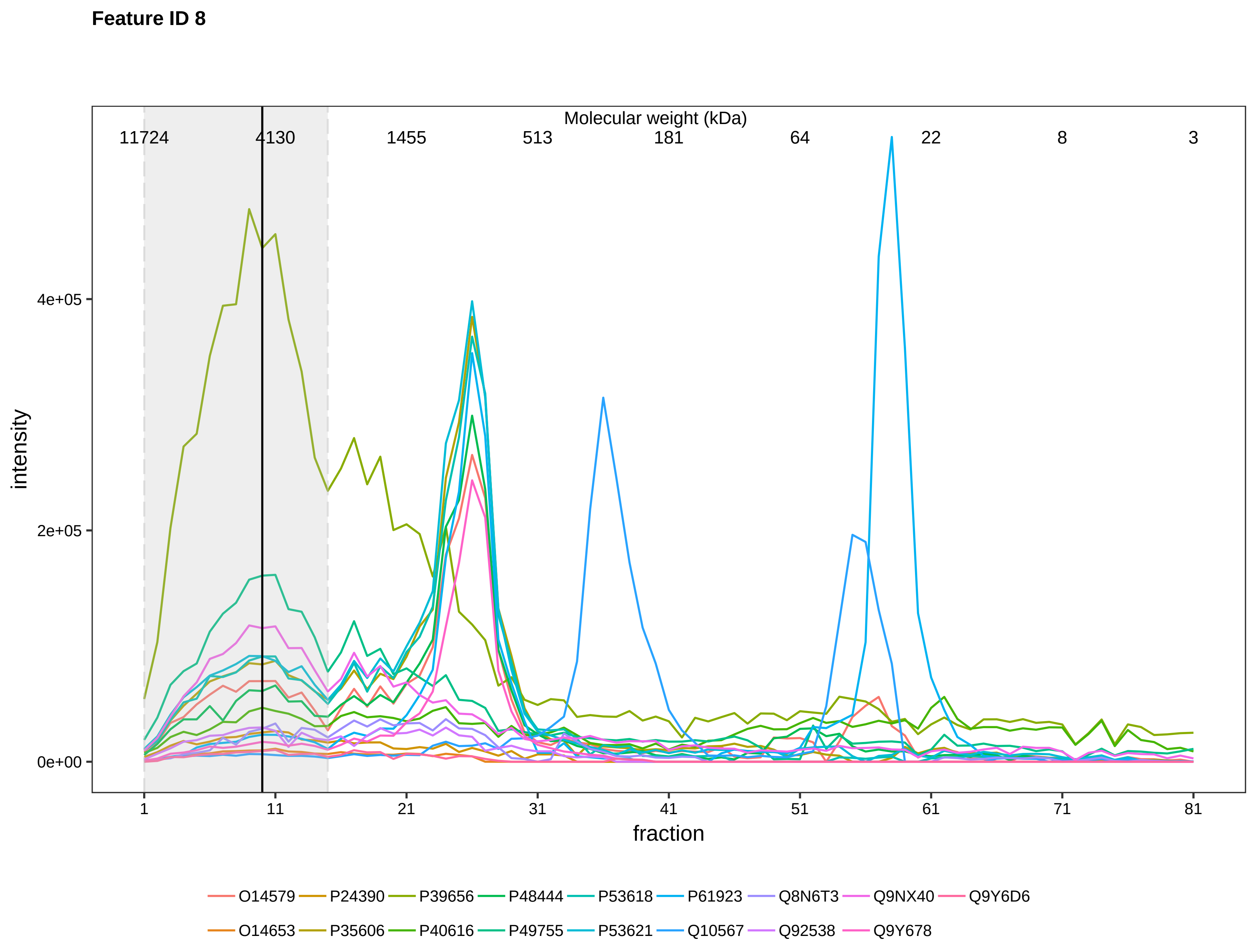
Feature ID 8
Molecular weight (kDa)
11724
4130
1455
513
181
64
22
8
3
intensity
4e+05
2e+05
0e+00
1
11
21
31
41
51
61
71
81
fraction
O14579
P24390
P39656
P48444
P53618
P61923
Q8N6T3
Q9NX40
Q9Y6D6
O14653
P35606
P40616
P49755
P53621
Q10567
Q92538
Q9Y678

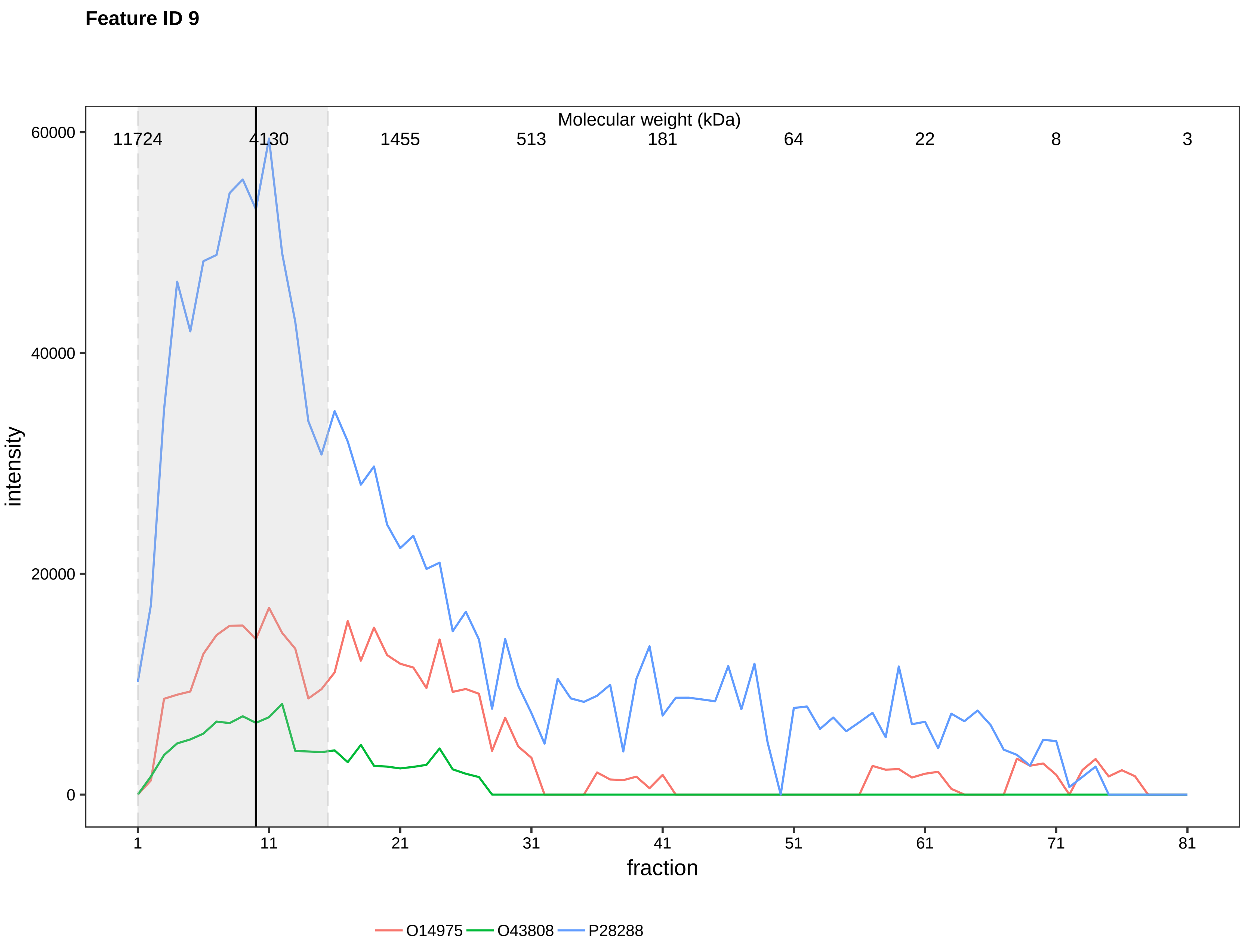

Feature ID 9
Molecular weight (kDa)
11724
4130
1455
513
181
64
22
8
3
60000
40000
20000
0
intensity
1
11
21
31
41
51
61
71
81
fraction
O14975
O43808
P28288

Feature ID 10

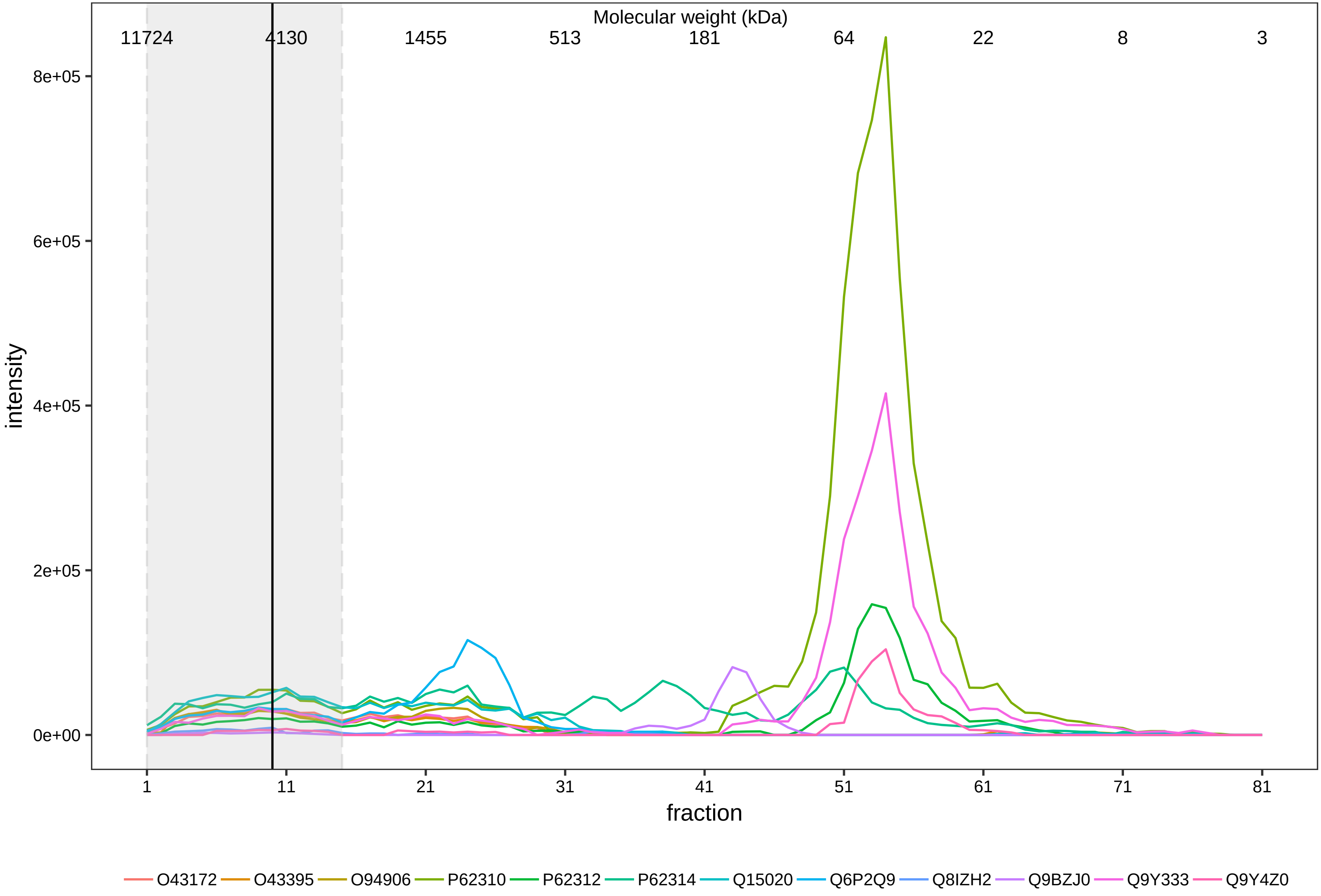

Feature ID 11

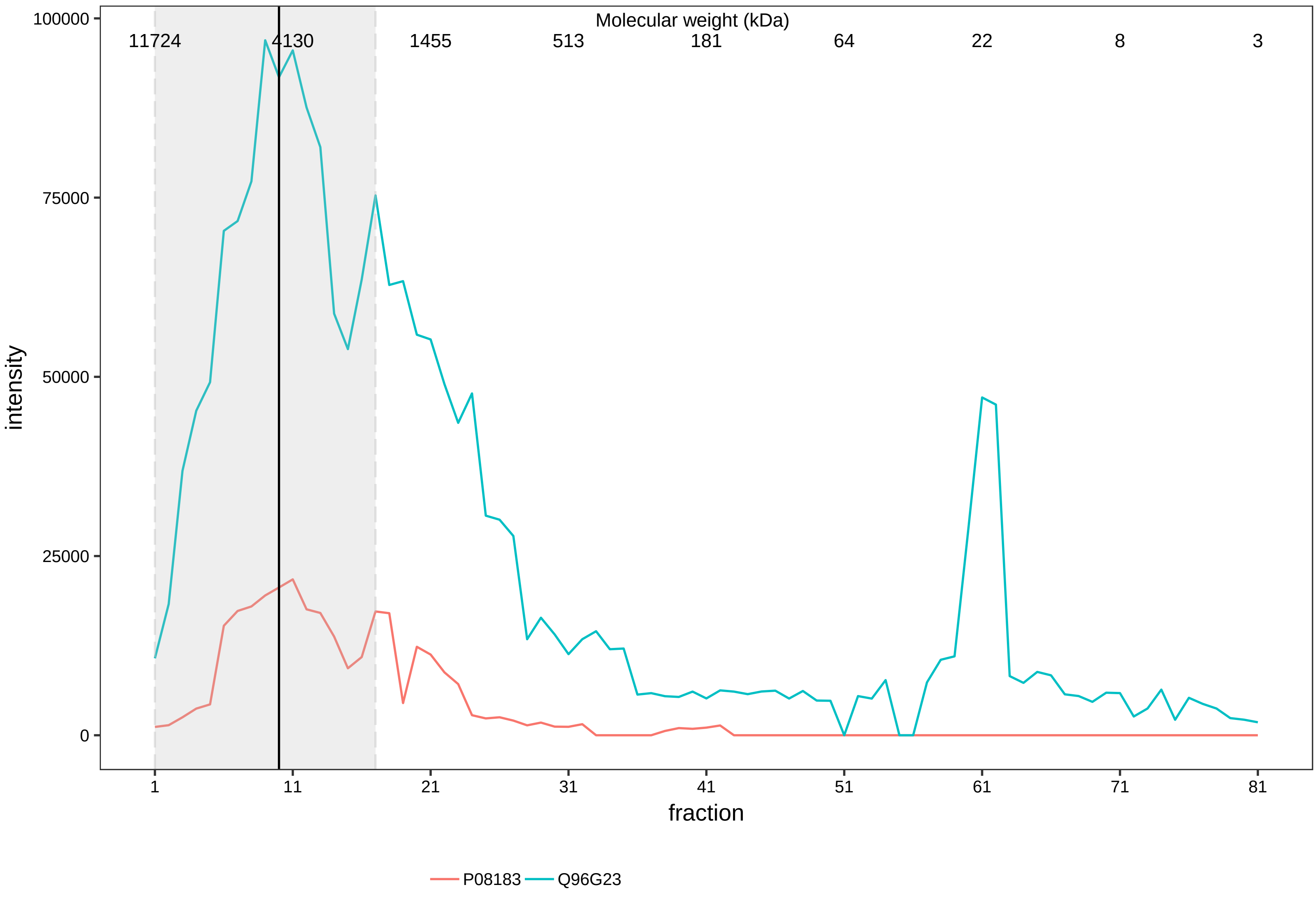

Feature ID 12

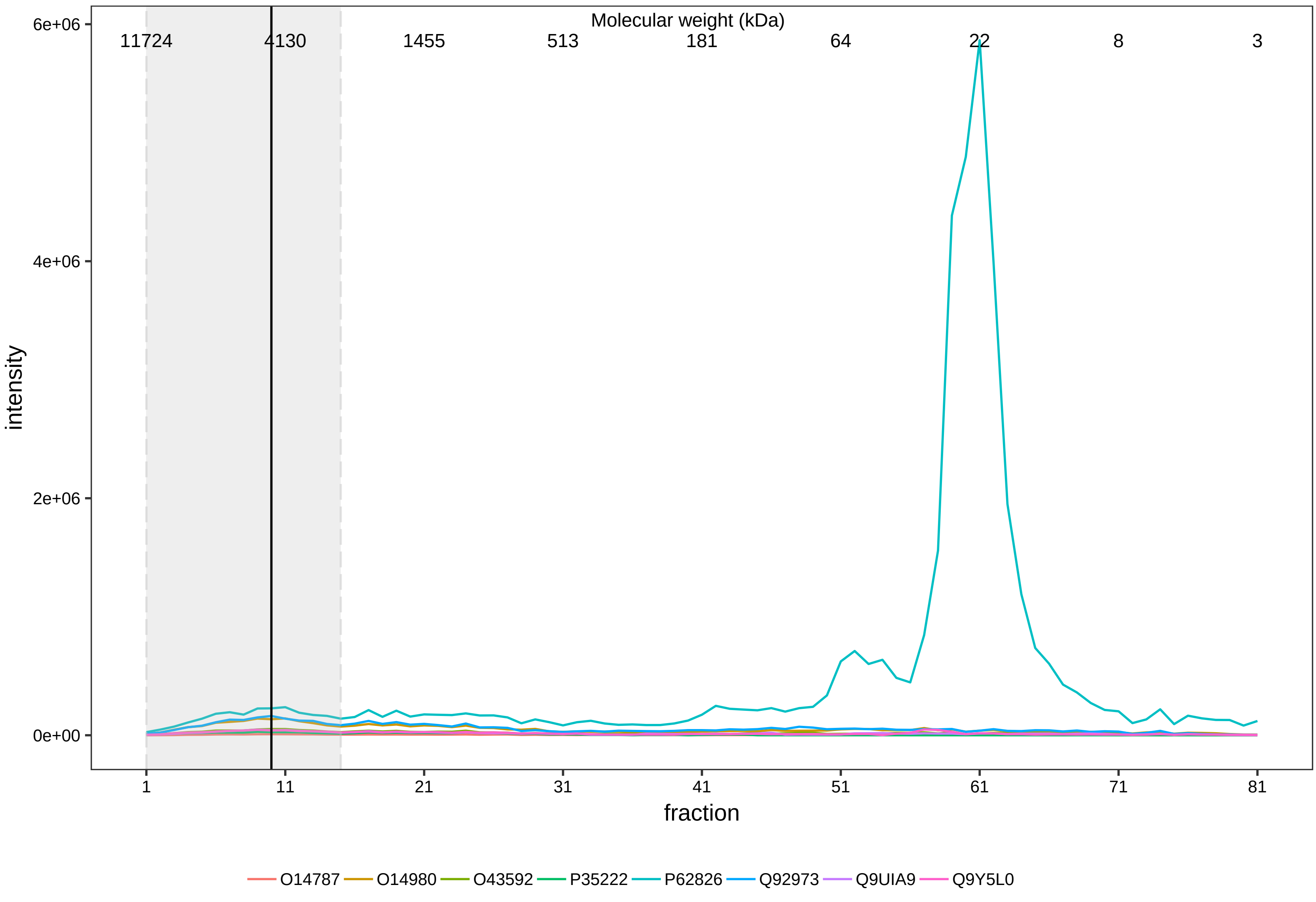

Feature ID 13

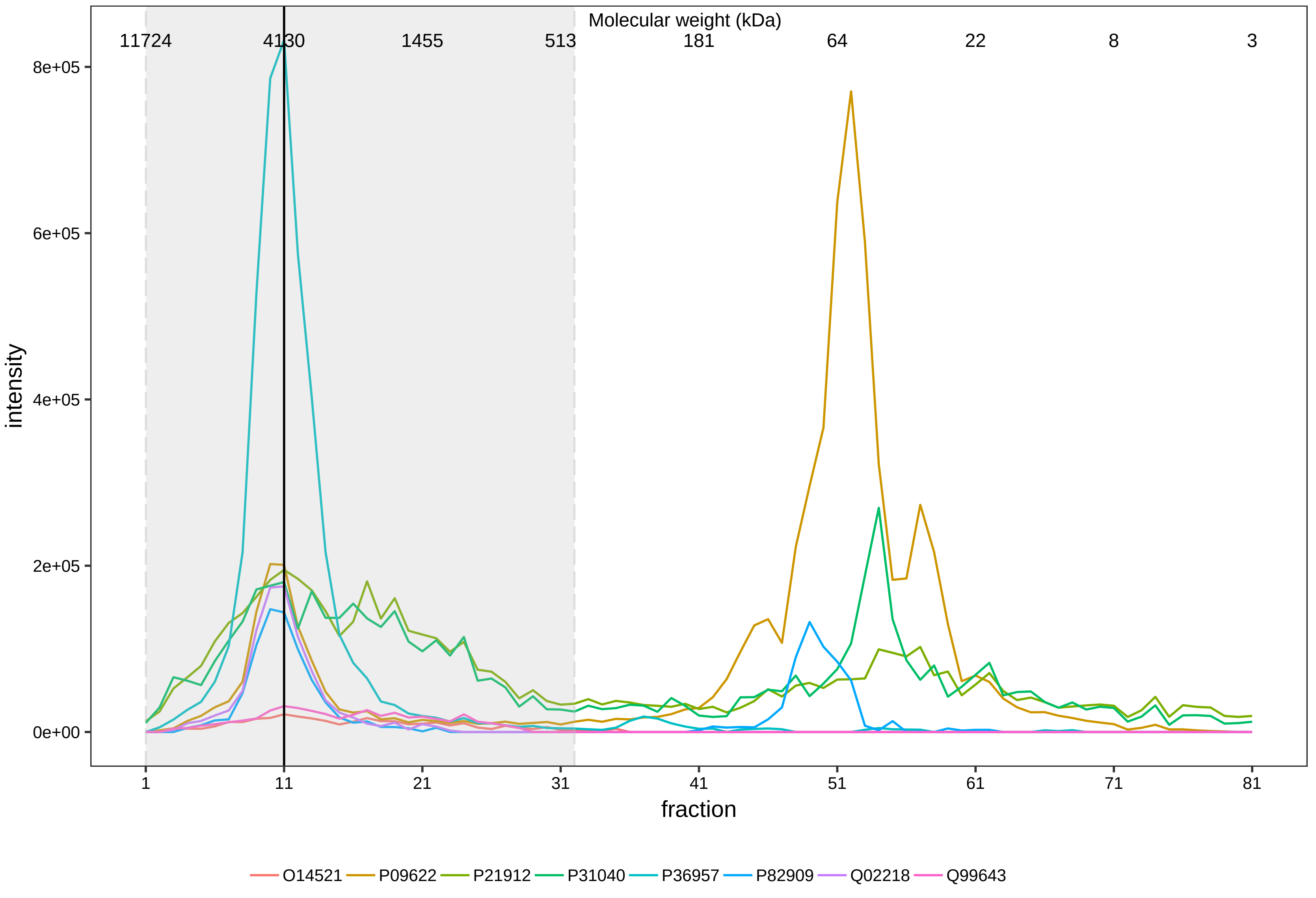

Feature ID 14

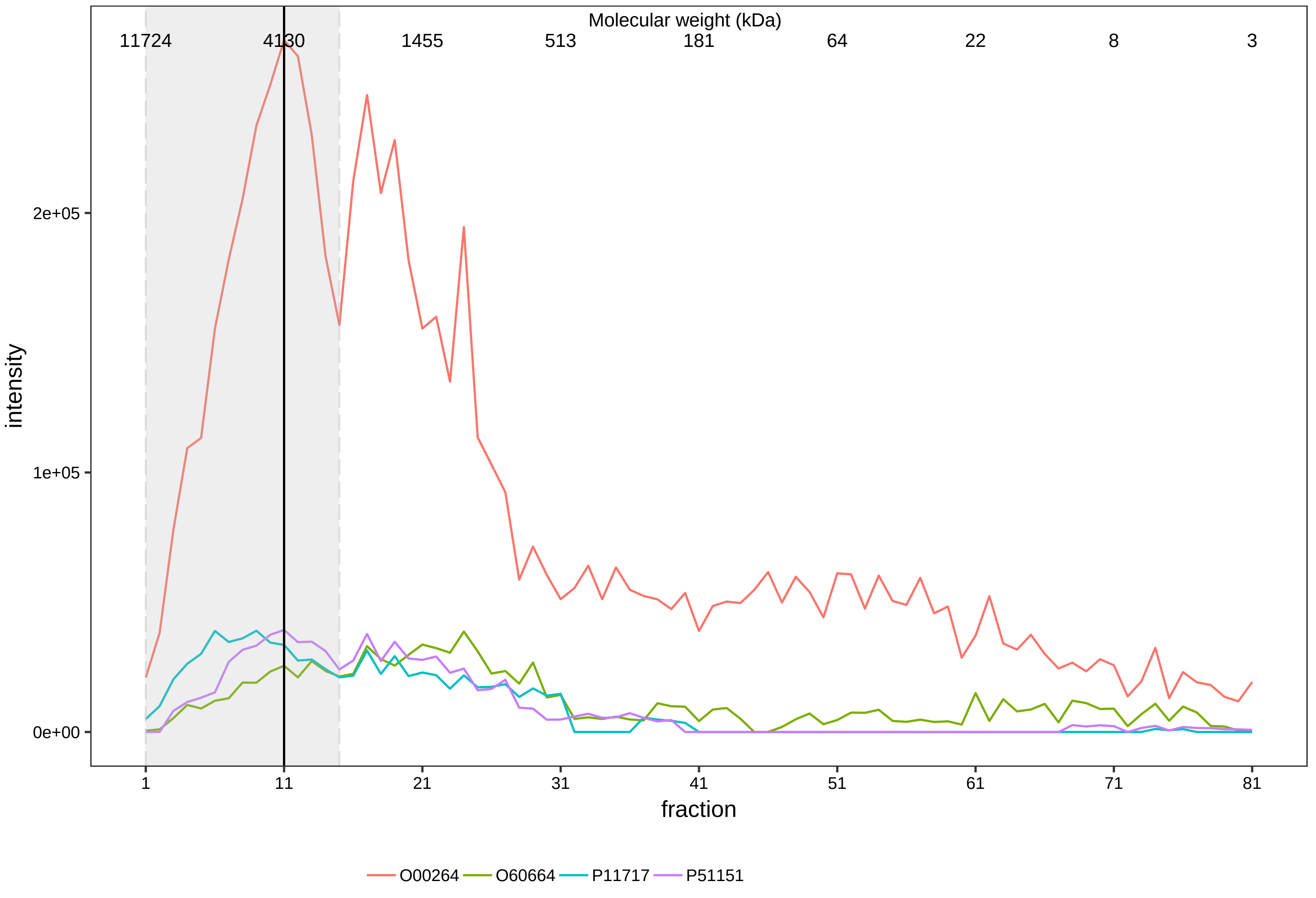

Feature ID 15

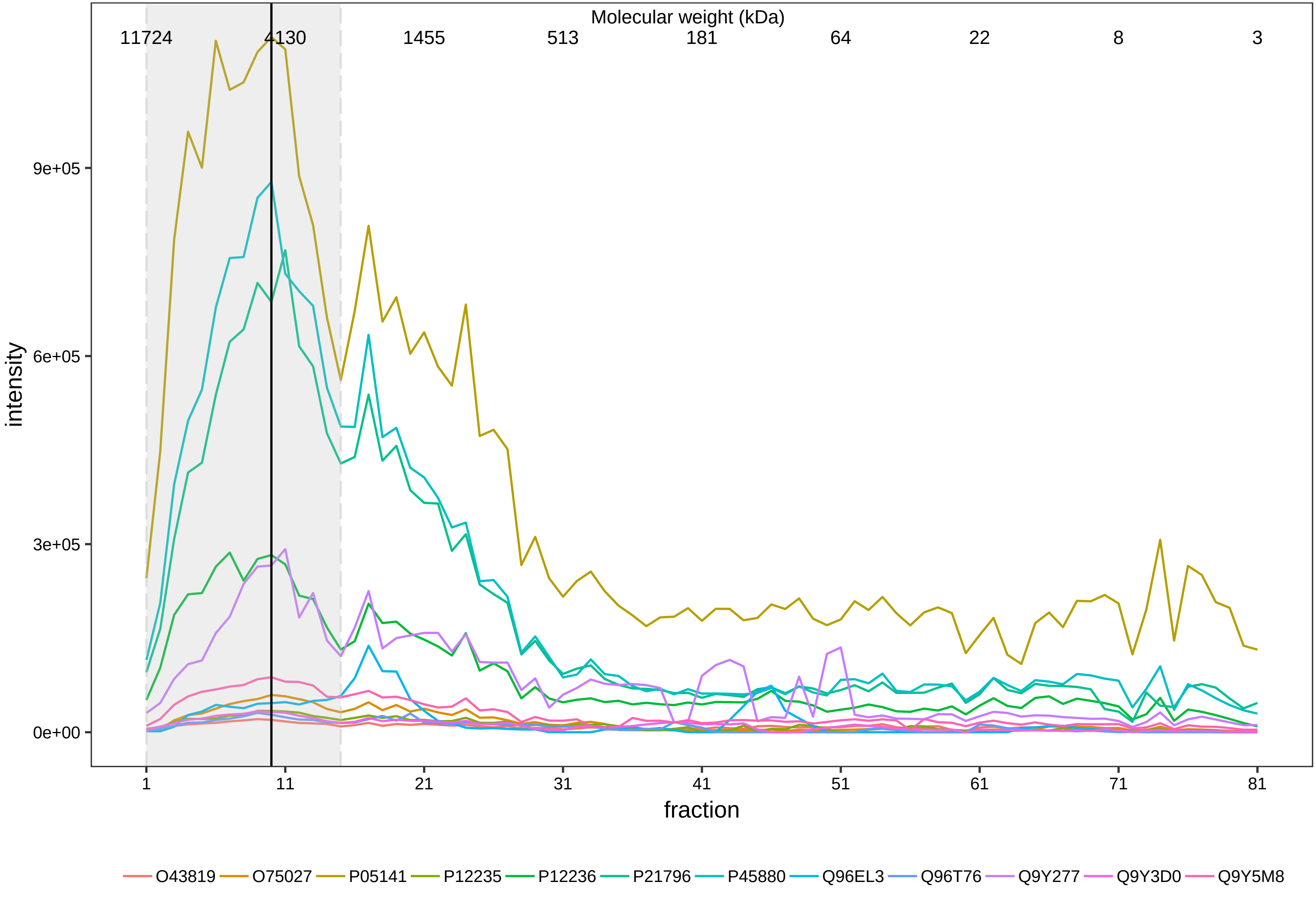

Feature ID 16

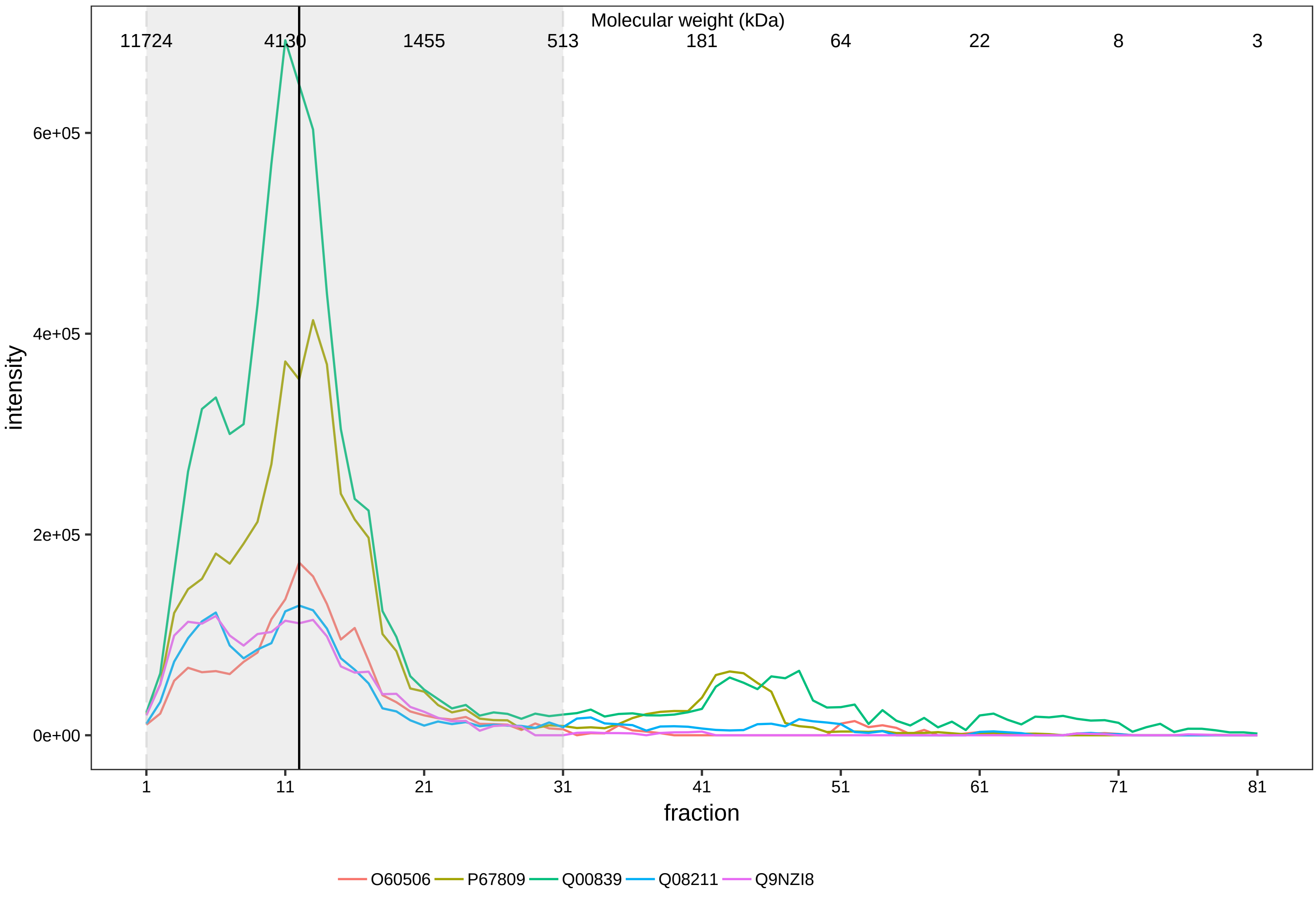

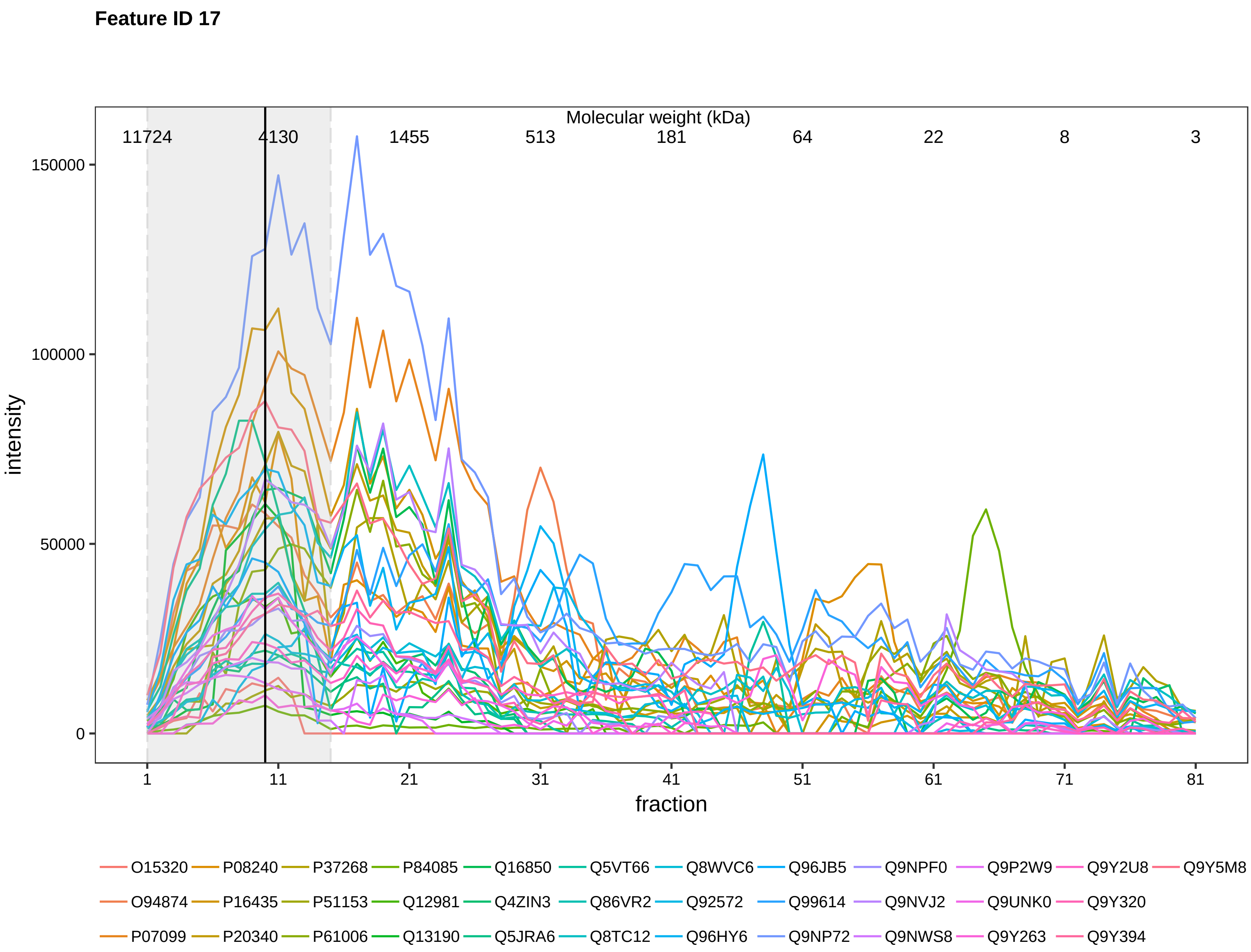
Feature ID 17
Molecular weight (kDa)
11724
4130
1455
513
181
64
22
8
3
150000
100000
50000
0
intensity
1
11
21
31
41
51
61
71
81
fraction
O15320
P08240
P37268
P84085
Q16850
Q5VT66
Q8WVC6
Q96JB5
Q9NPF0
Q9P2W9
Q9Y2U8
Q9Y5M8
O94874
P16435
P51153
Q12981
Q4ZIN3
Q86VR2
Q92572
Q99614
Q9NVJ2
Q9UNK0
Q9Y320
P07099
P20340
P61006
Q13190
Q5JRA6
Q8TC12
Q96HY6
Q9NP72
Q9NWS8
Q9Y263
Q9Y394

Feature ID 18

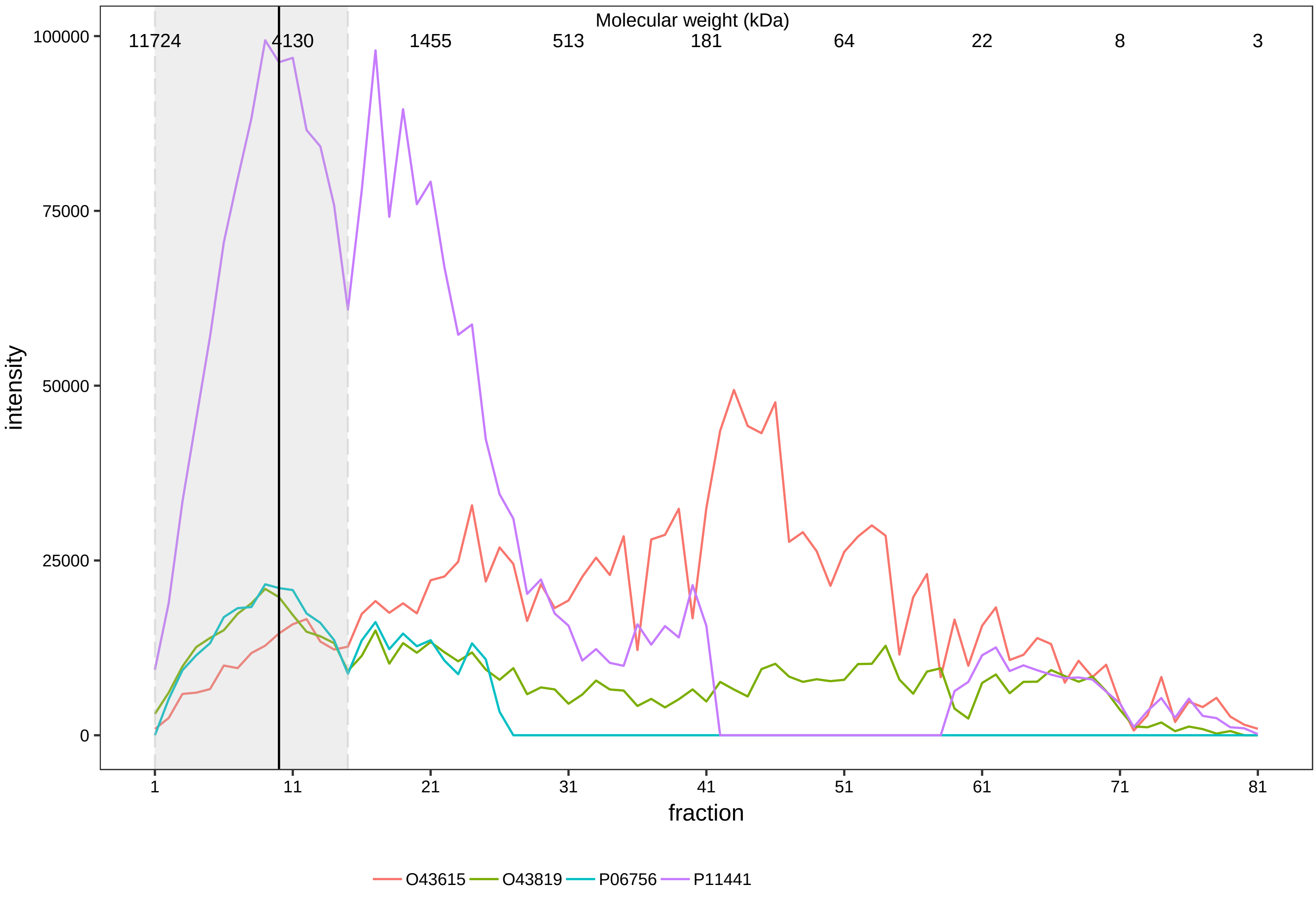

Feature ID 19

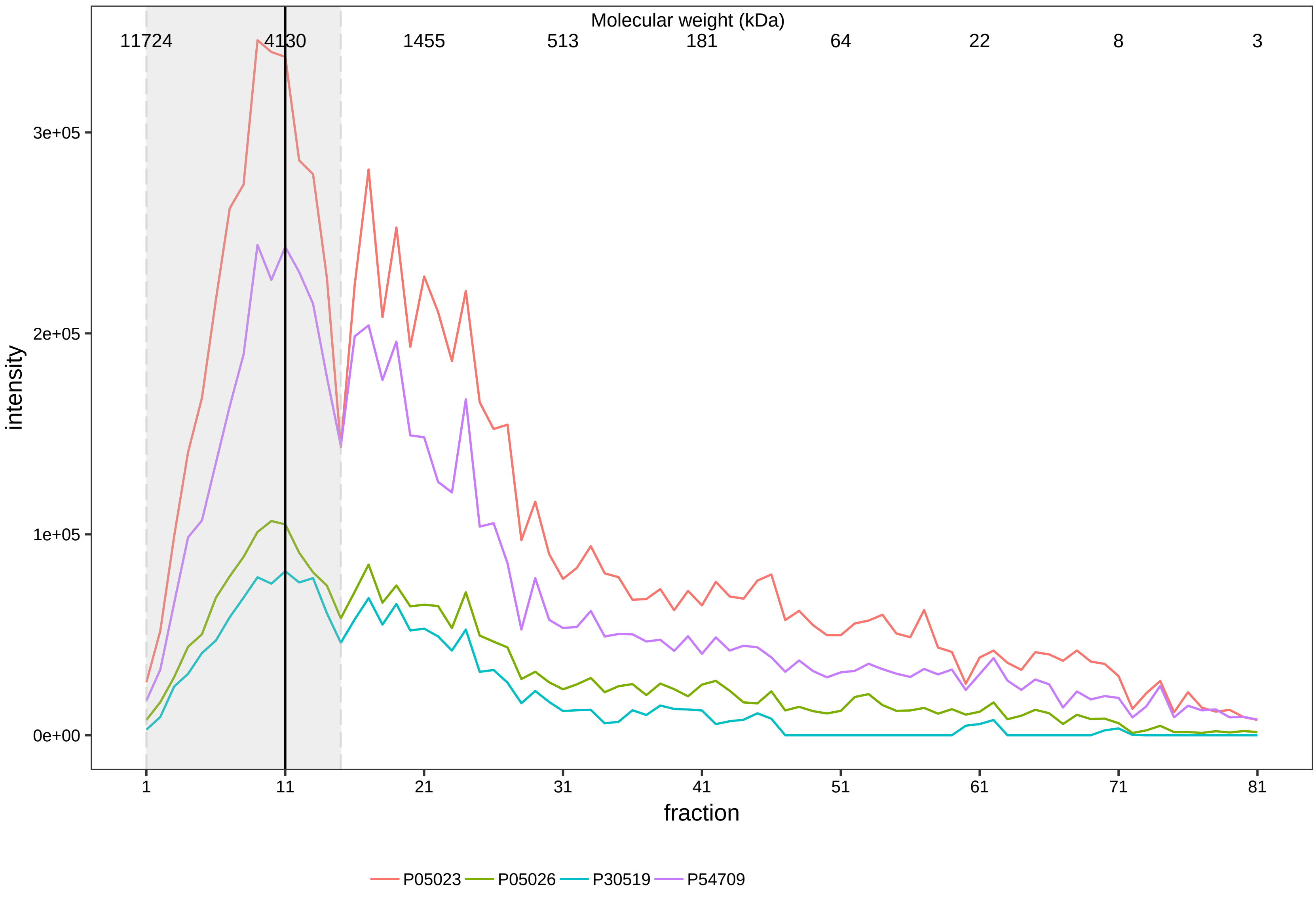

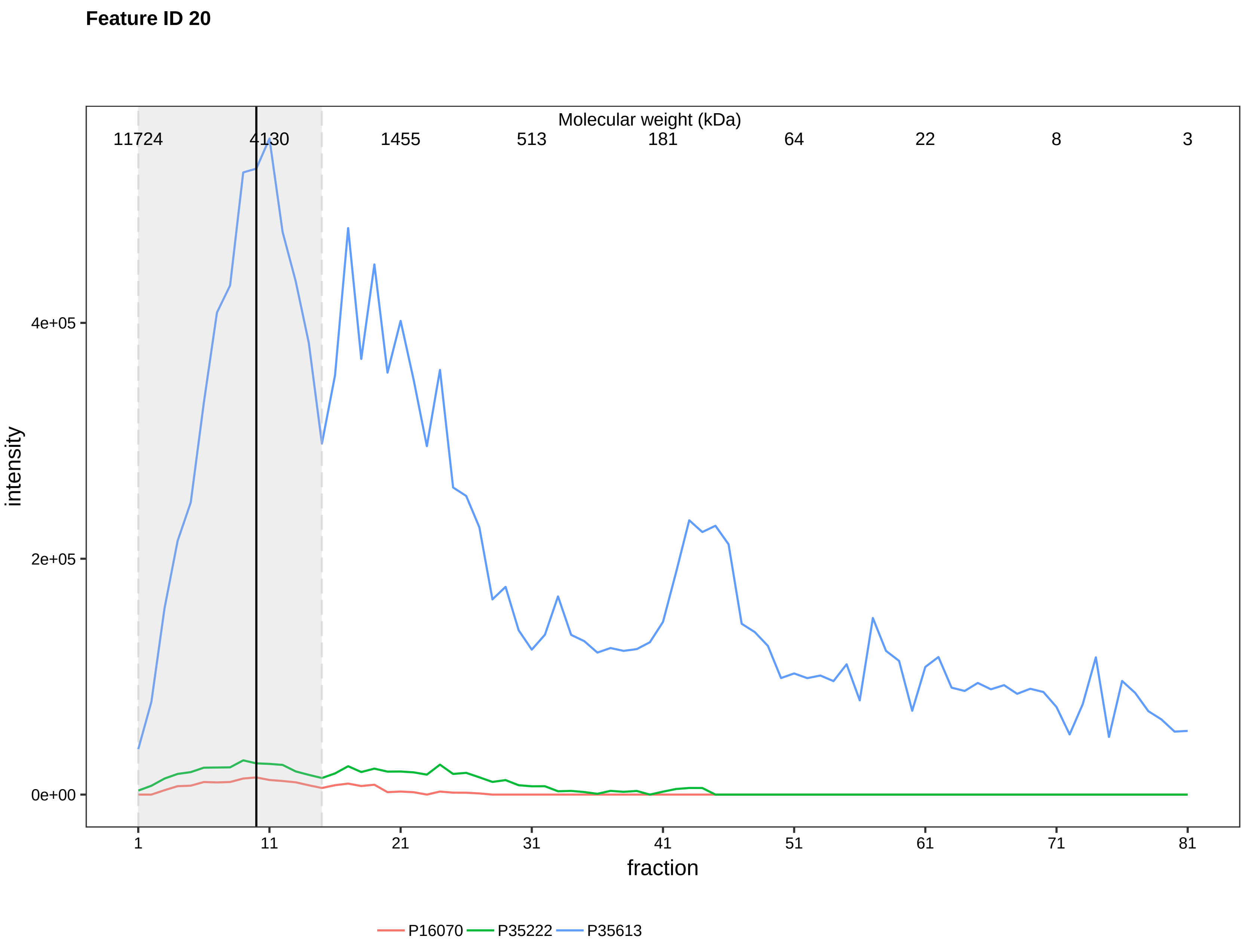
Feature ID 20
Molecular weight (kDa)
11724
4130
1455
513
181
64
22
8
3
intensity
4e+05
2e+05
0e+00
fraction
1
11
21
31
41
51
61
71
81
P16070
P35222
P35613

Feature ID 21

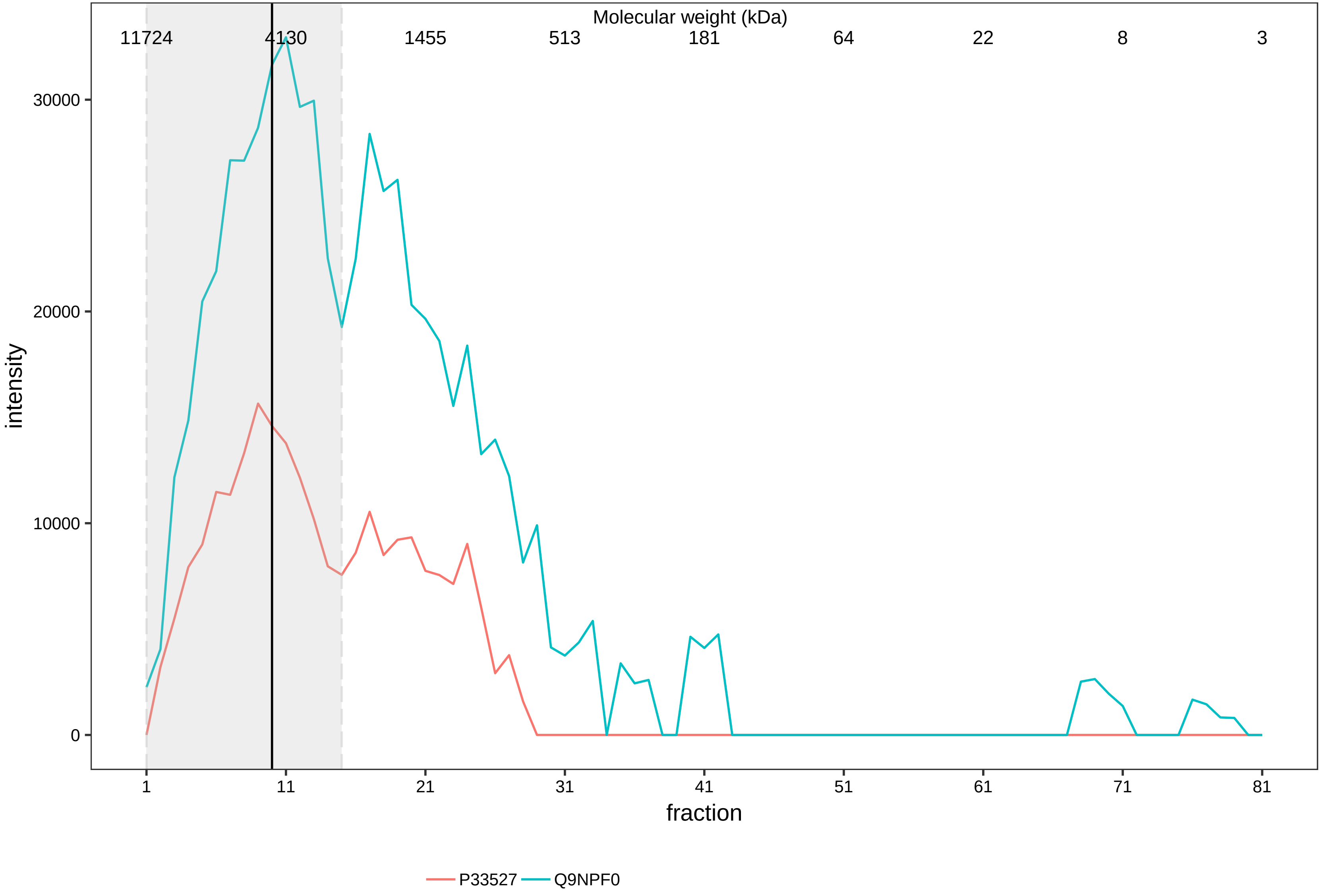

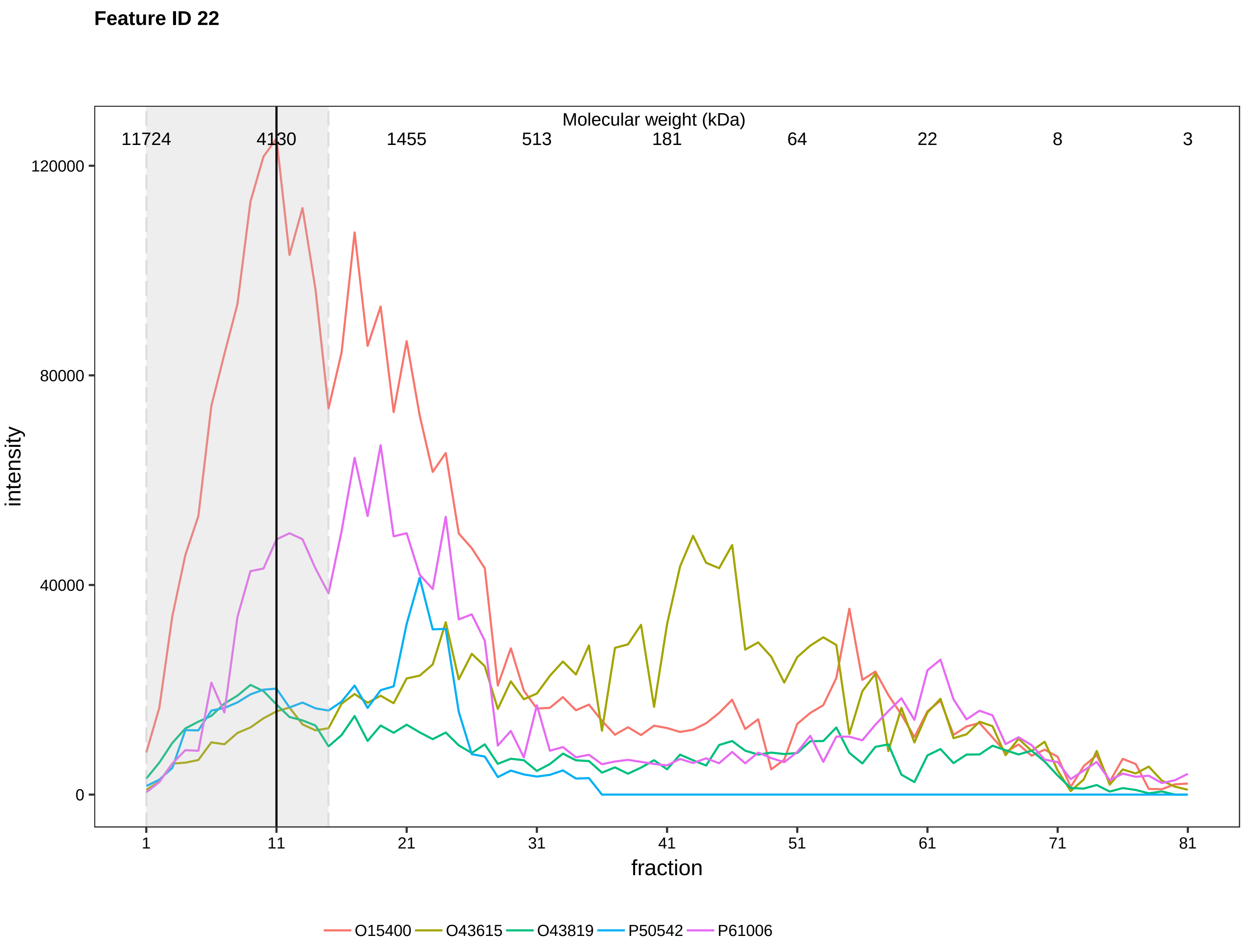

Feature ID 22
Molecular weight (kDa)
11724
4130
1455
513
181
64
22
8
3
120000
80000
40000
0
intensity
1
11
21
31
41
51
61
71
81
fraction
O15400
O43615
O43819
P50542
P61006

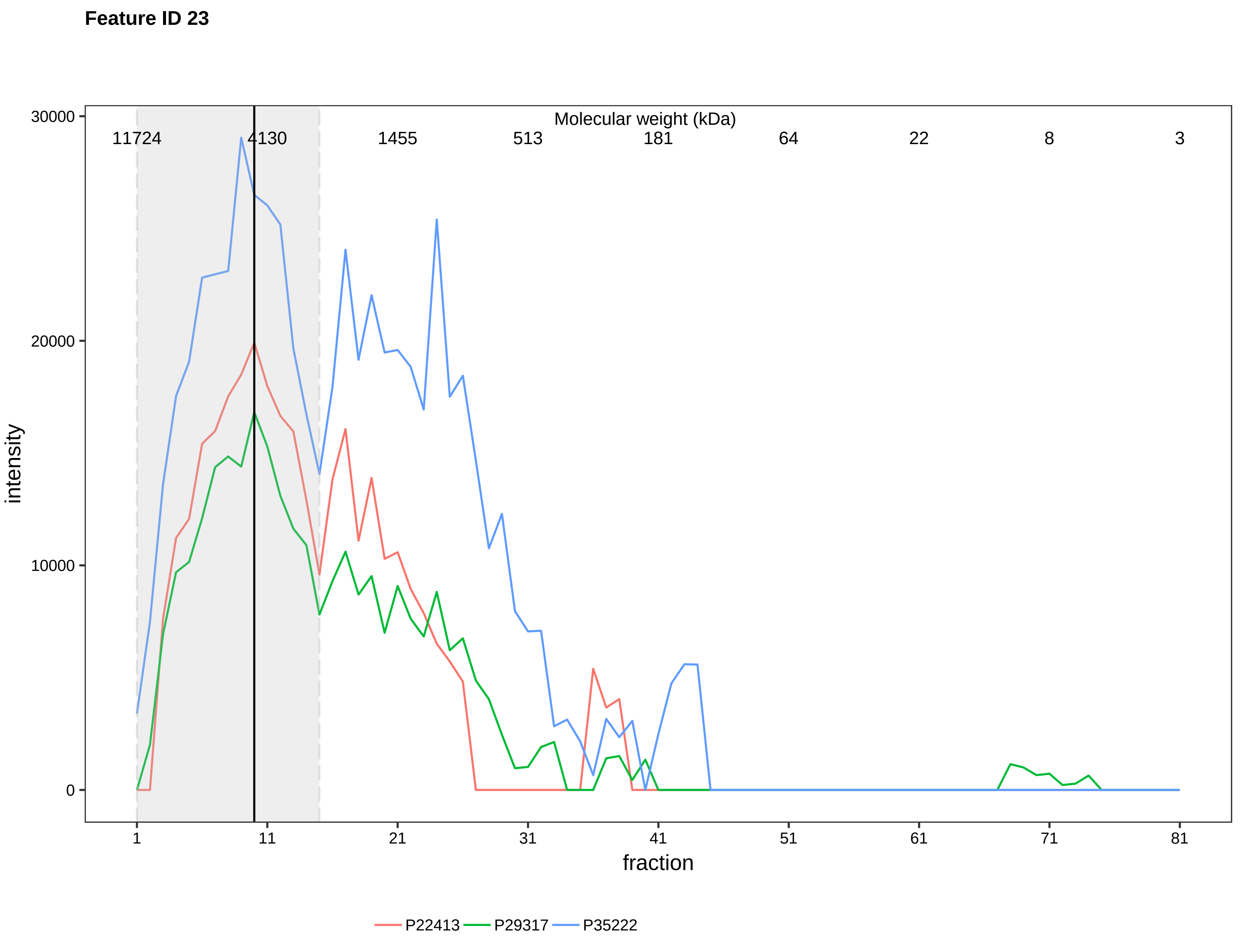

Feature ID 23
Molecular weight (kDa)
11724
4130
1455
513
181
64
22
8
3
30000
20000
10000
0
intensity
1
11
21
31
41
51
61
71
81
fraction
P22413
P29317
P35222

Feature ID 24

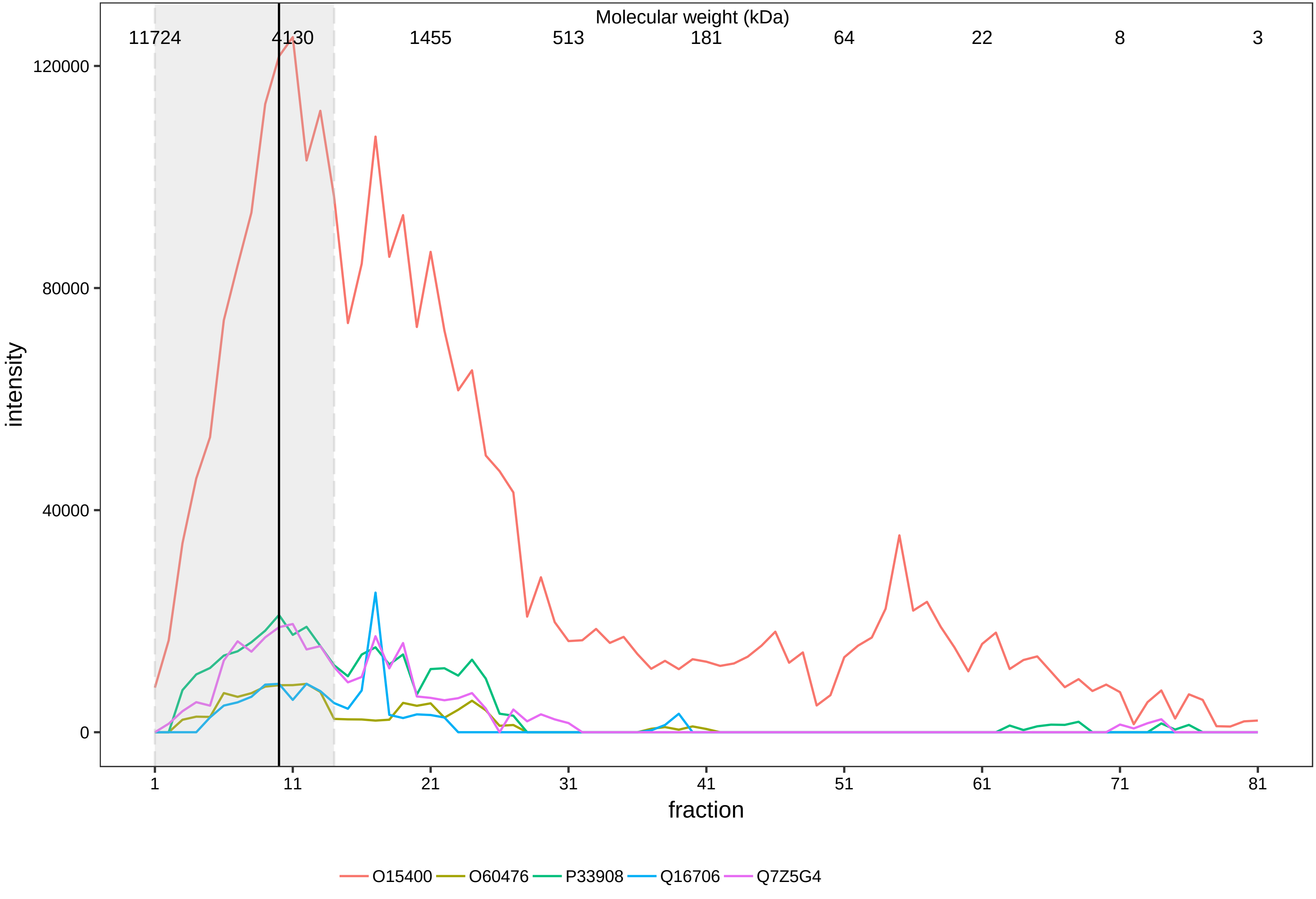

Feature ID 25

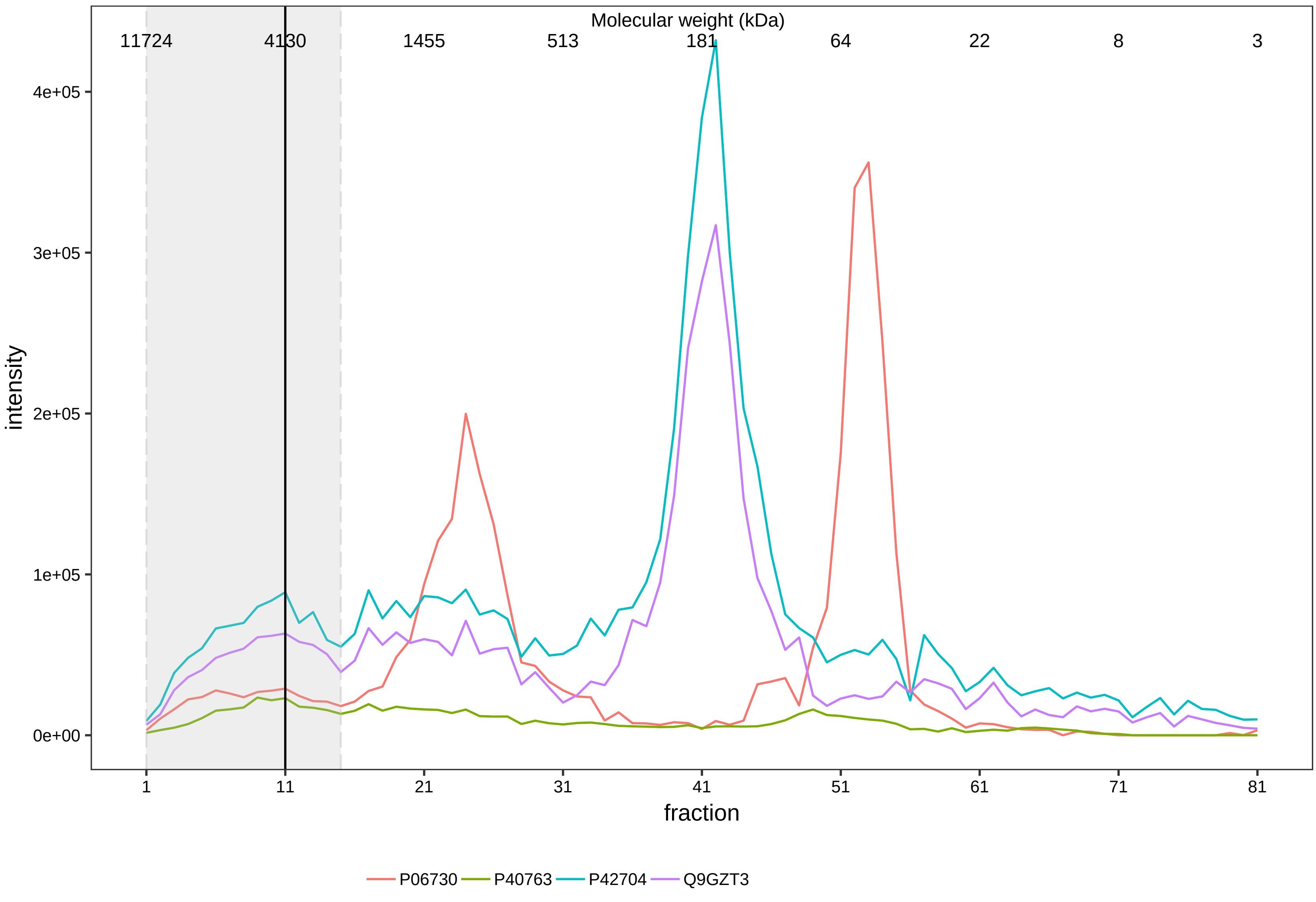

Feature ID 26

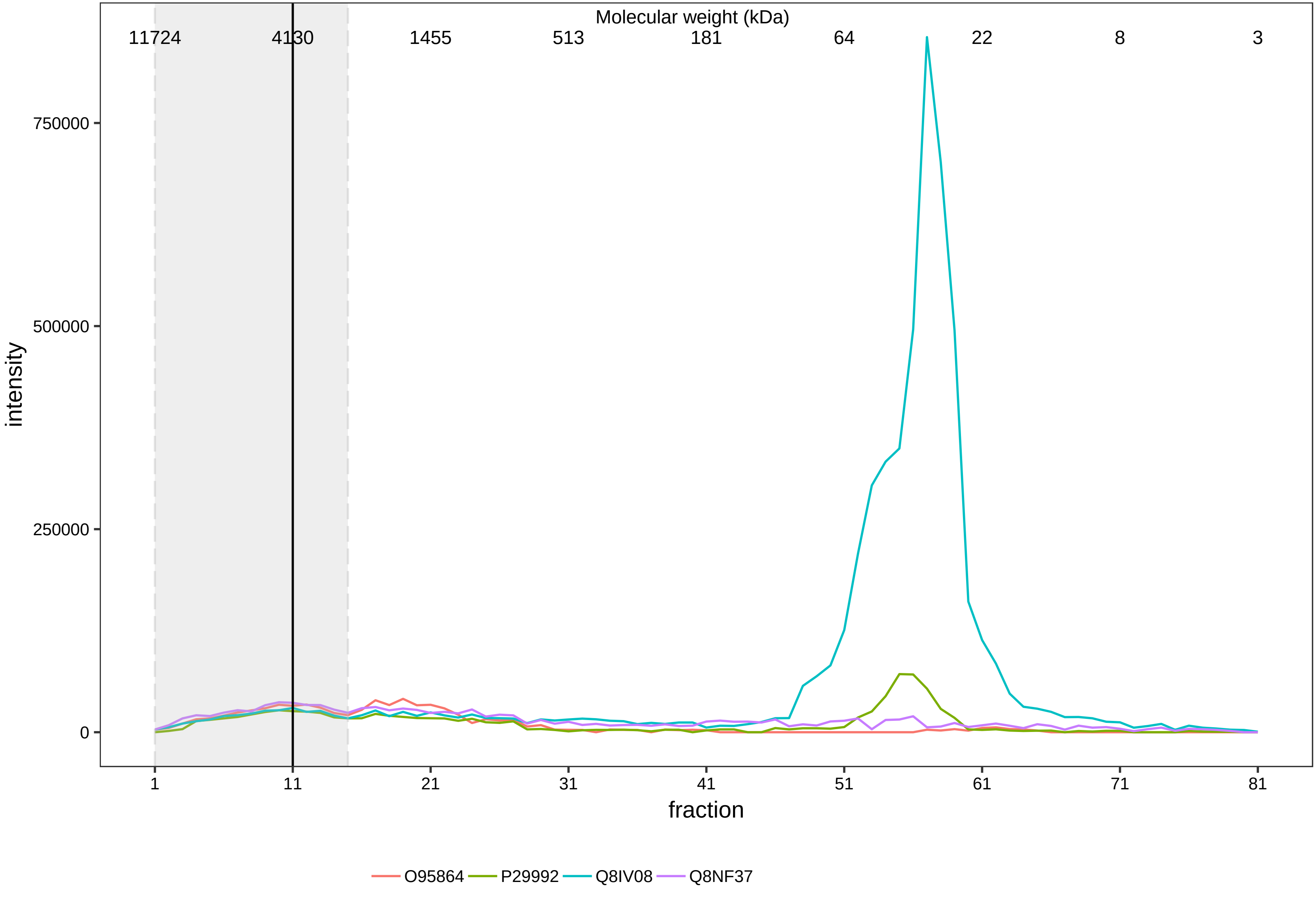

Feature ID 27

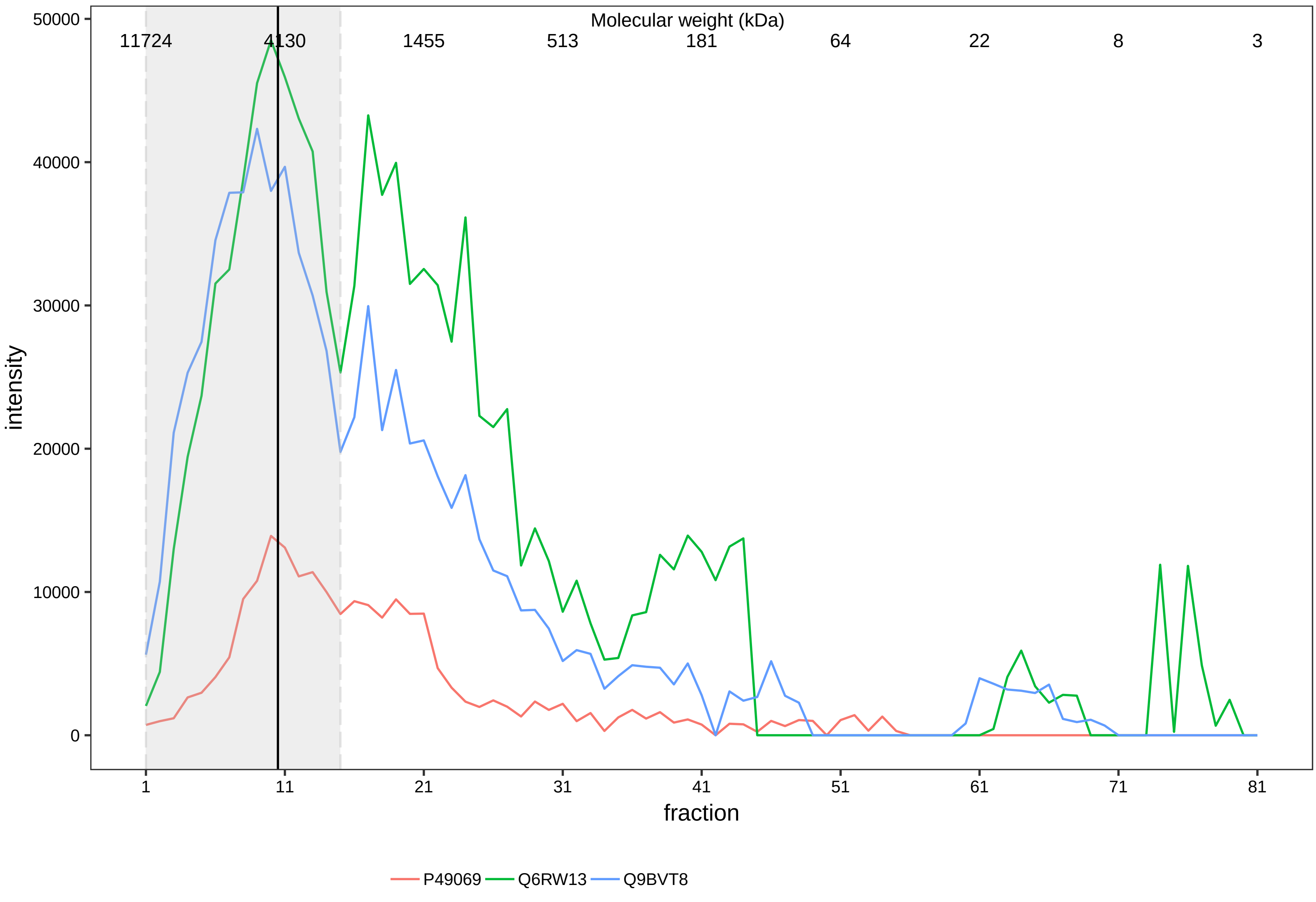

Feature ID 28

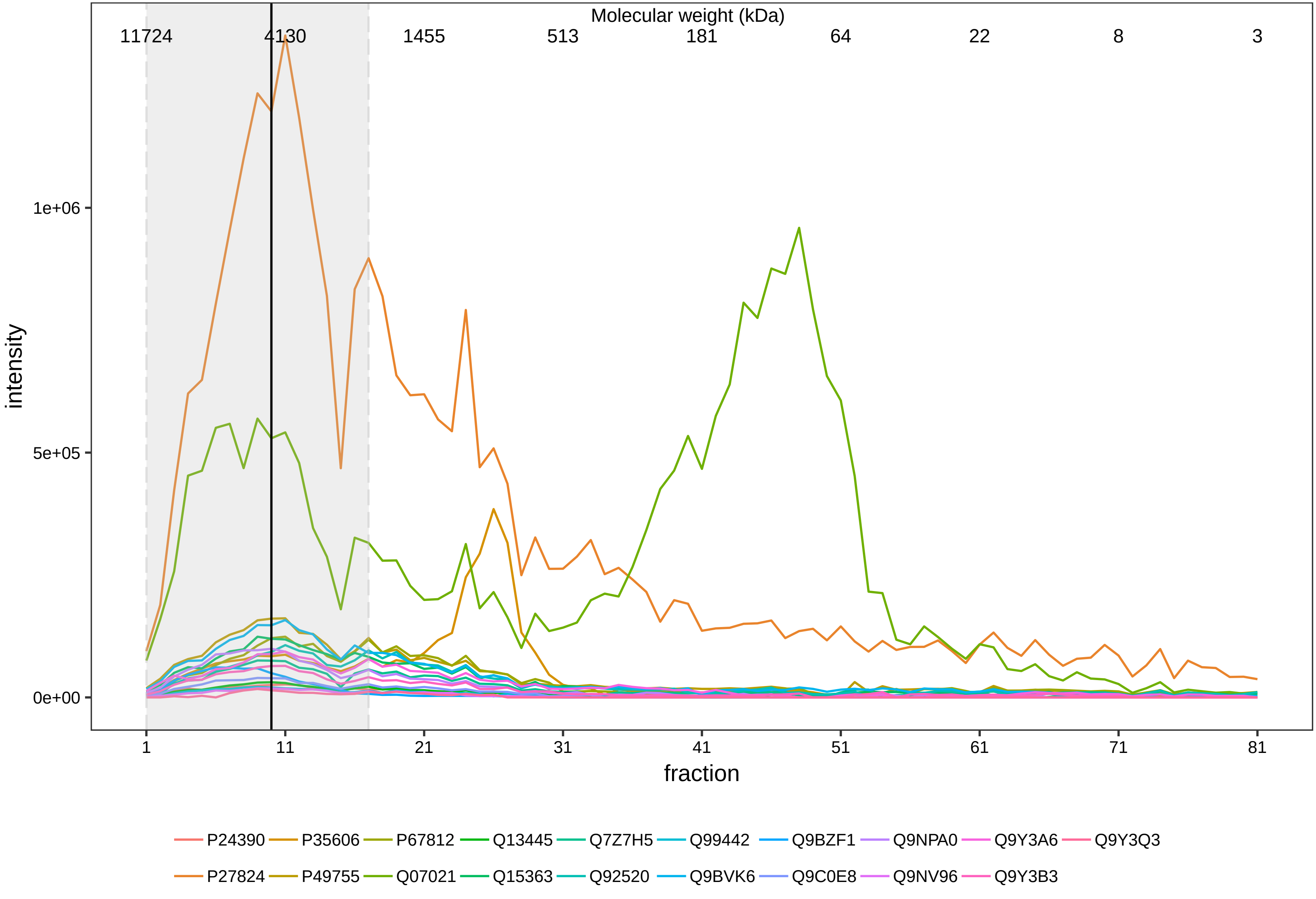

Feature ID 29

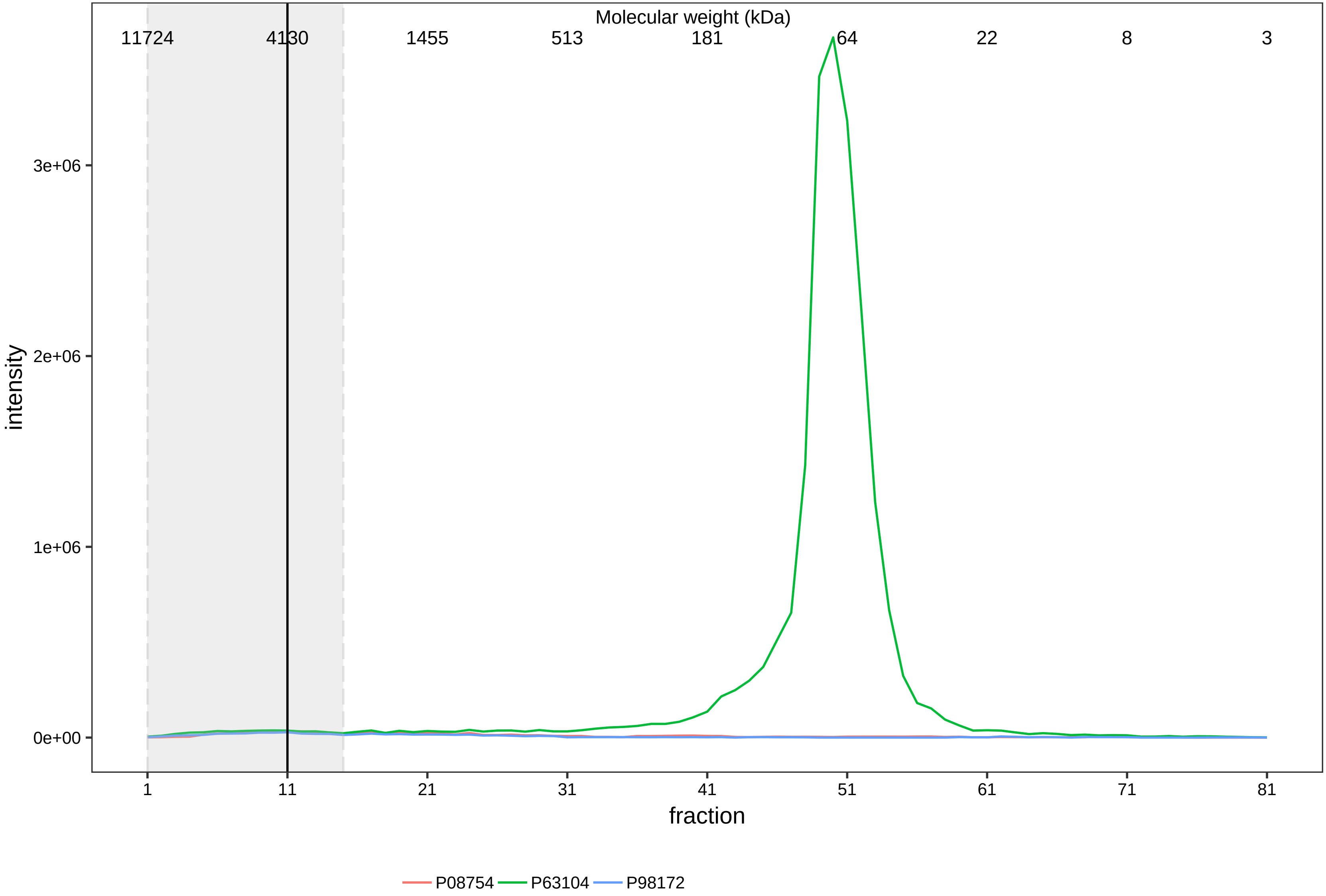

Feature ID 30

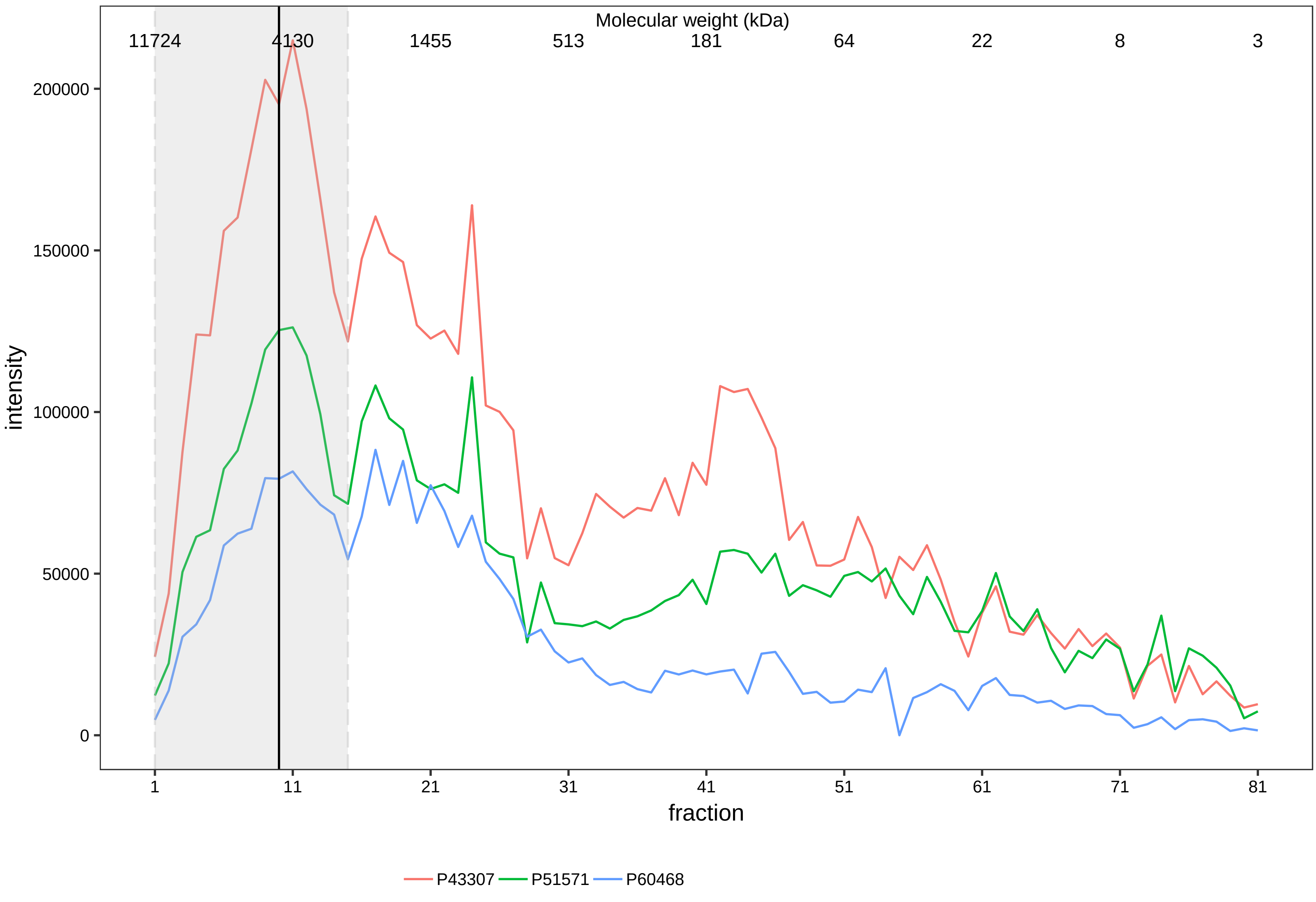

Feature ID 31

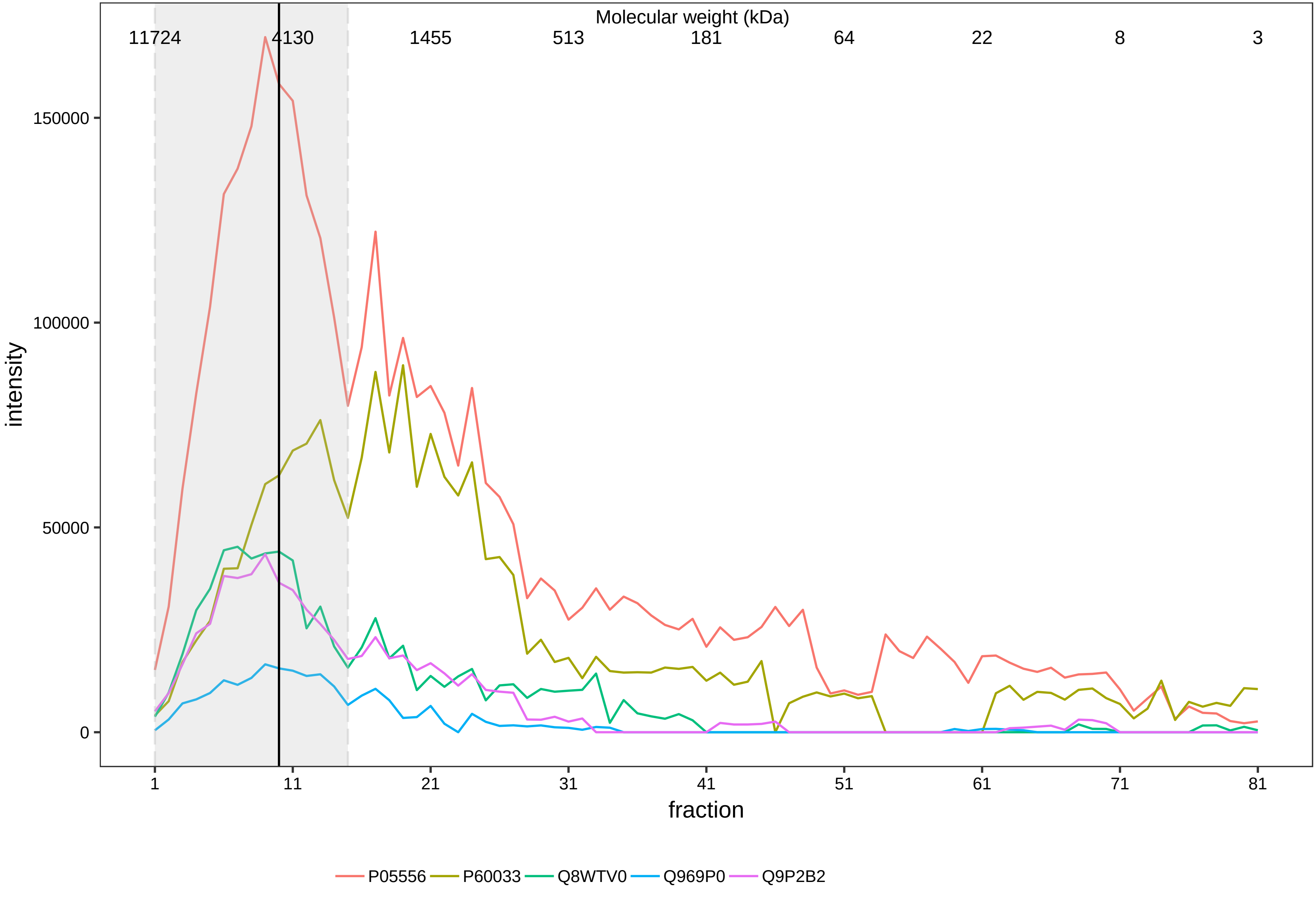

Feature ID 32

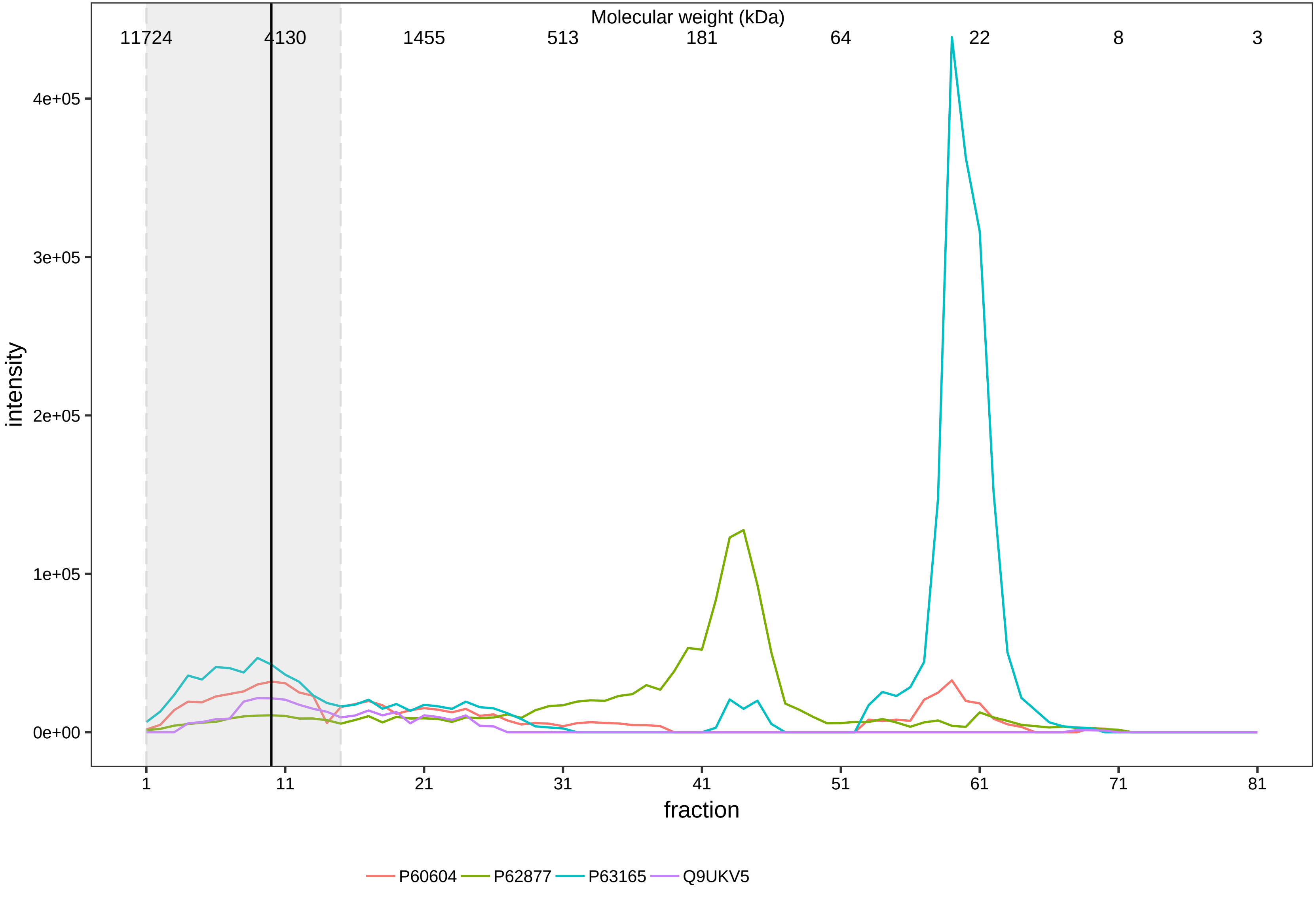

Feature ID 33

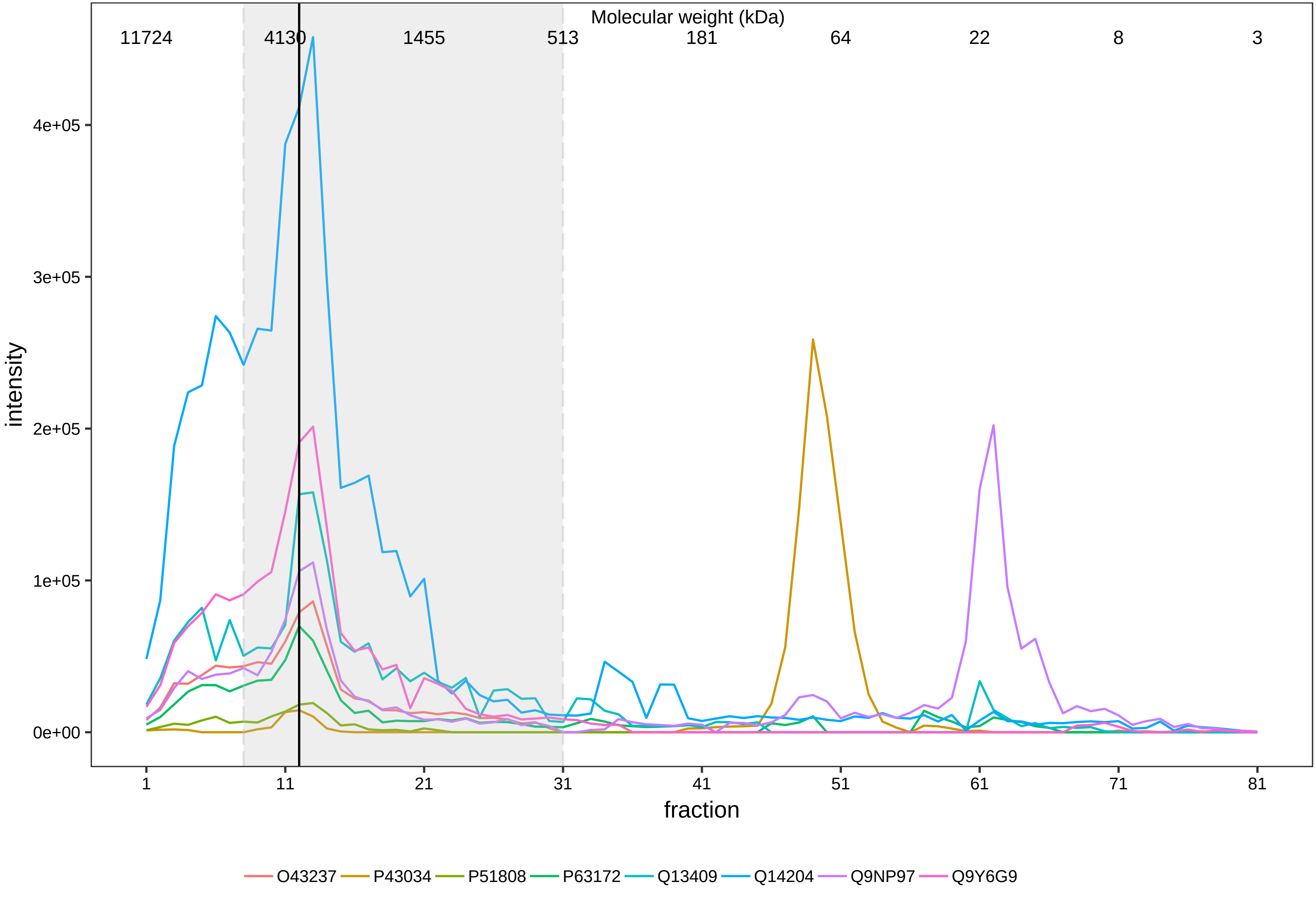

Feature ID 34

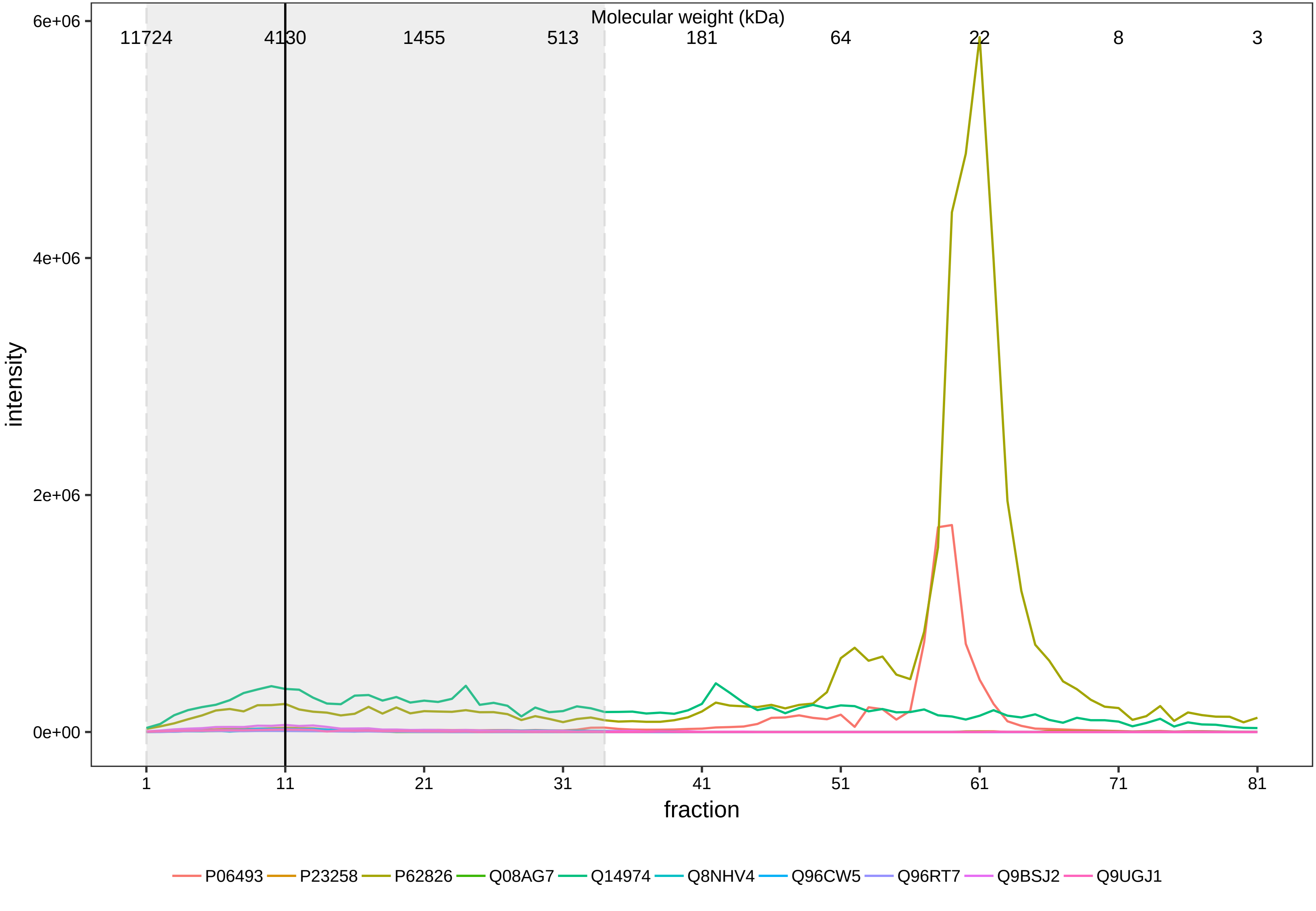

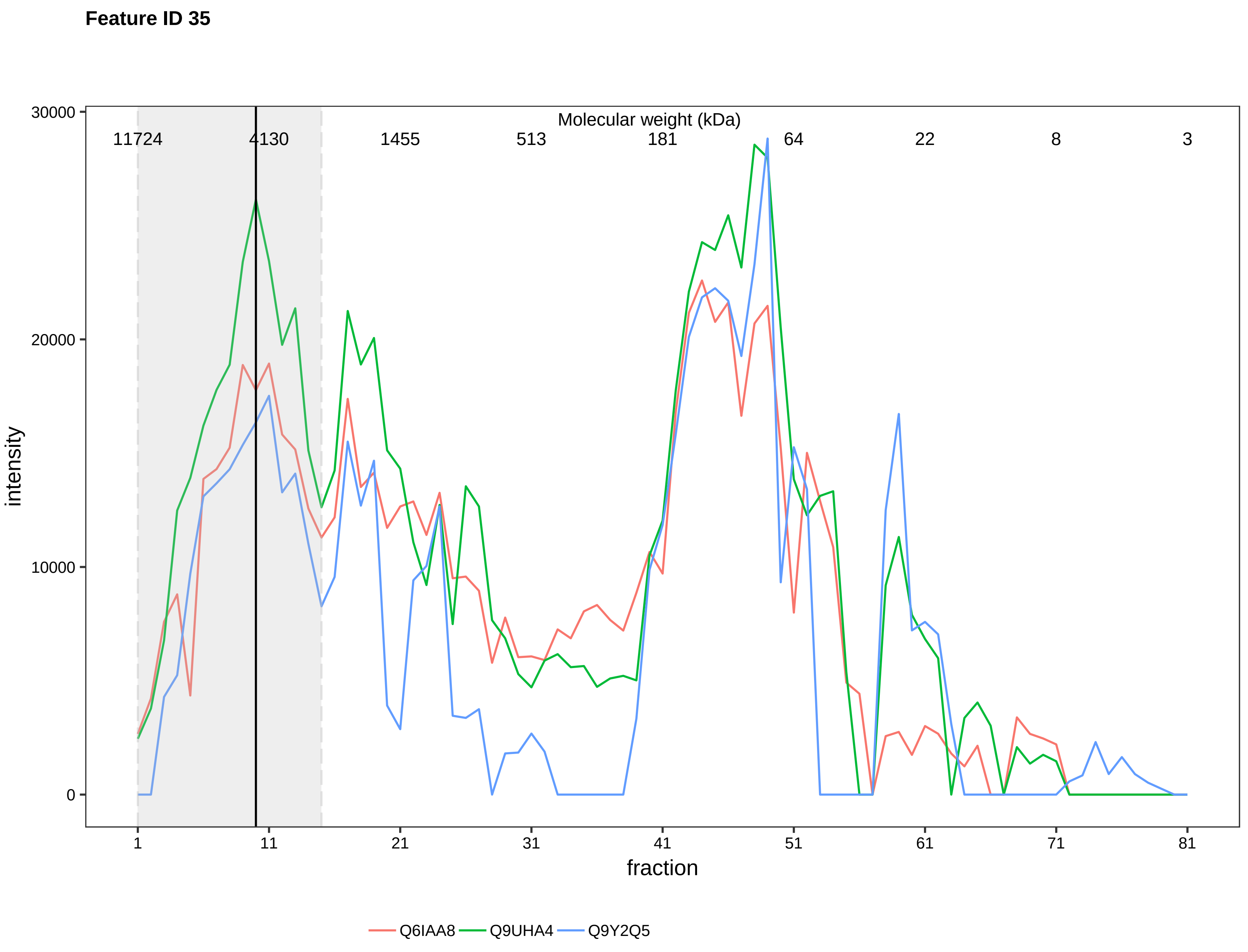

Feature ID 35
Molecular weight (kDa)
11724
4130
1455
513
181
64
22
8
3
30000
20000
10000
0
intensity
1
11
21
31
41
51
61
71
81
fraction
Q6IAA8
Q9UHA4
Q9Y2Q5

Feature ID 36

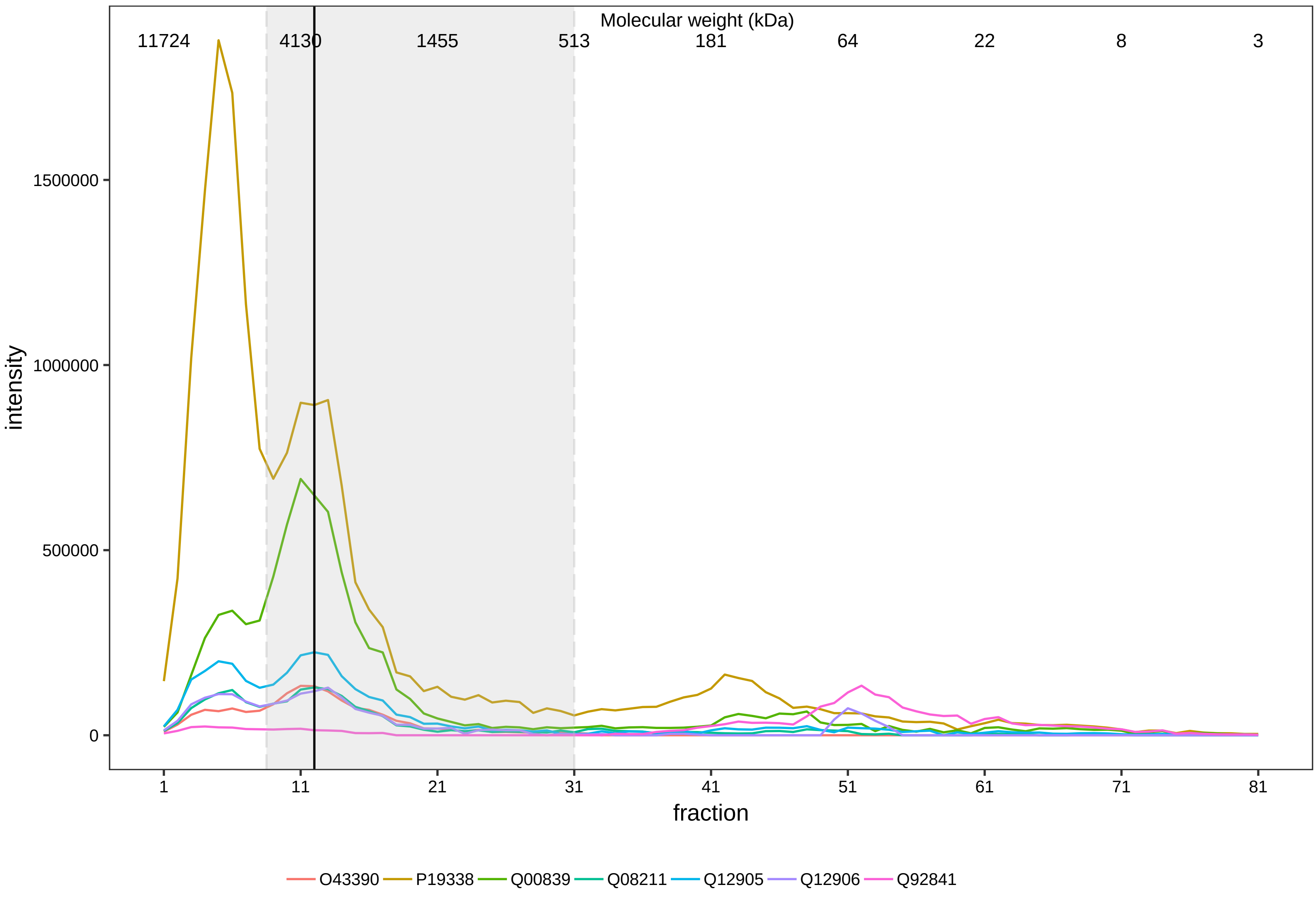

Feature ID 37

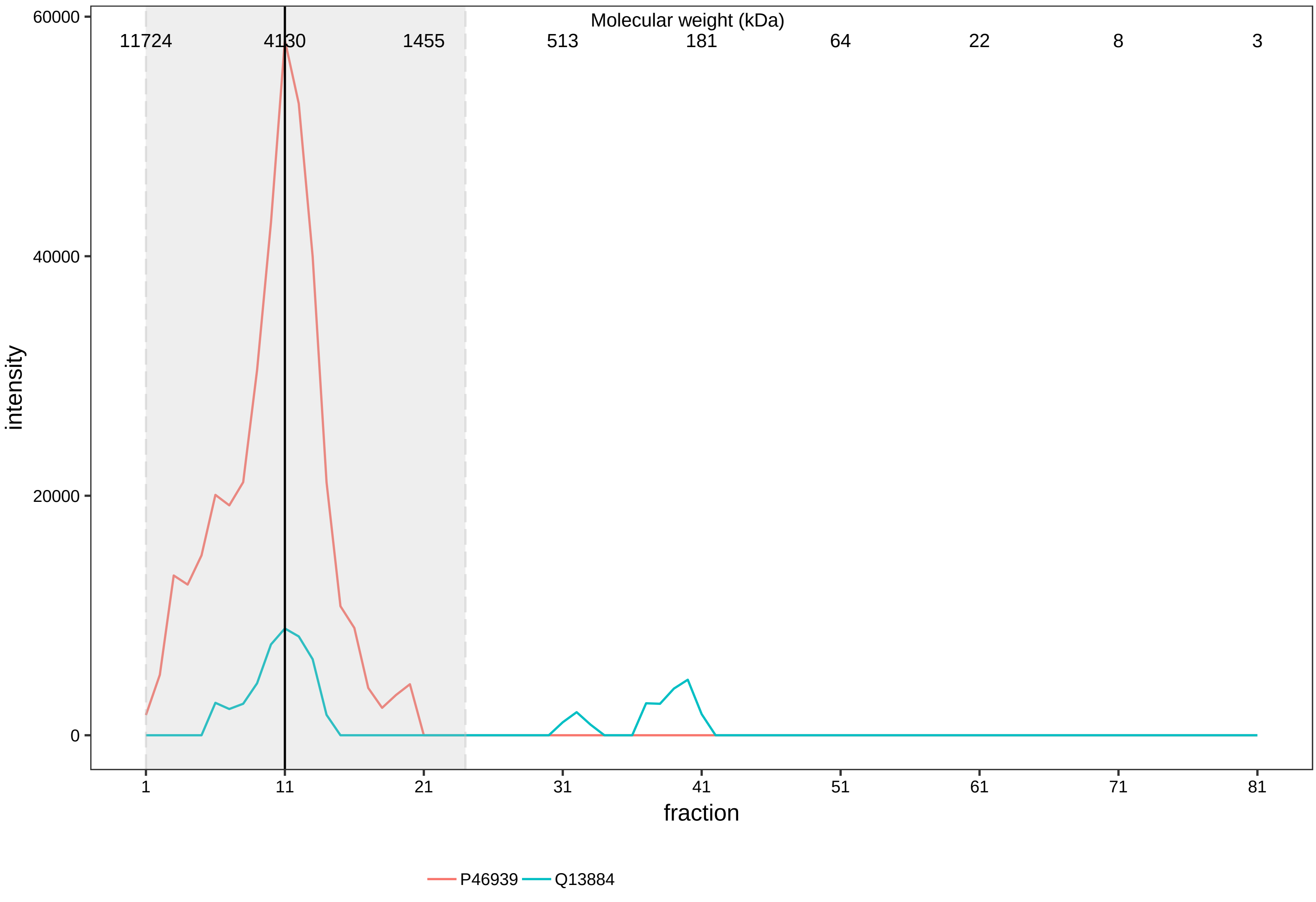

Feature ID 38

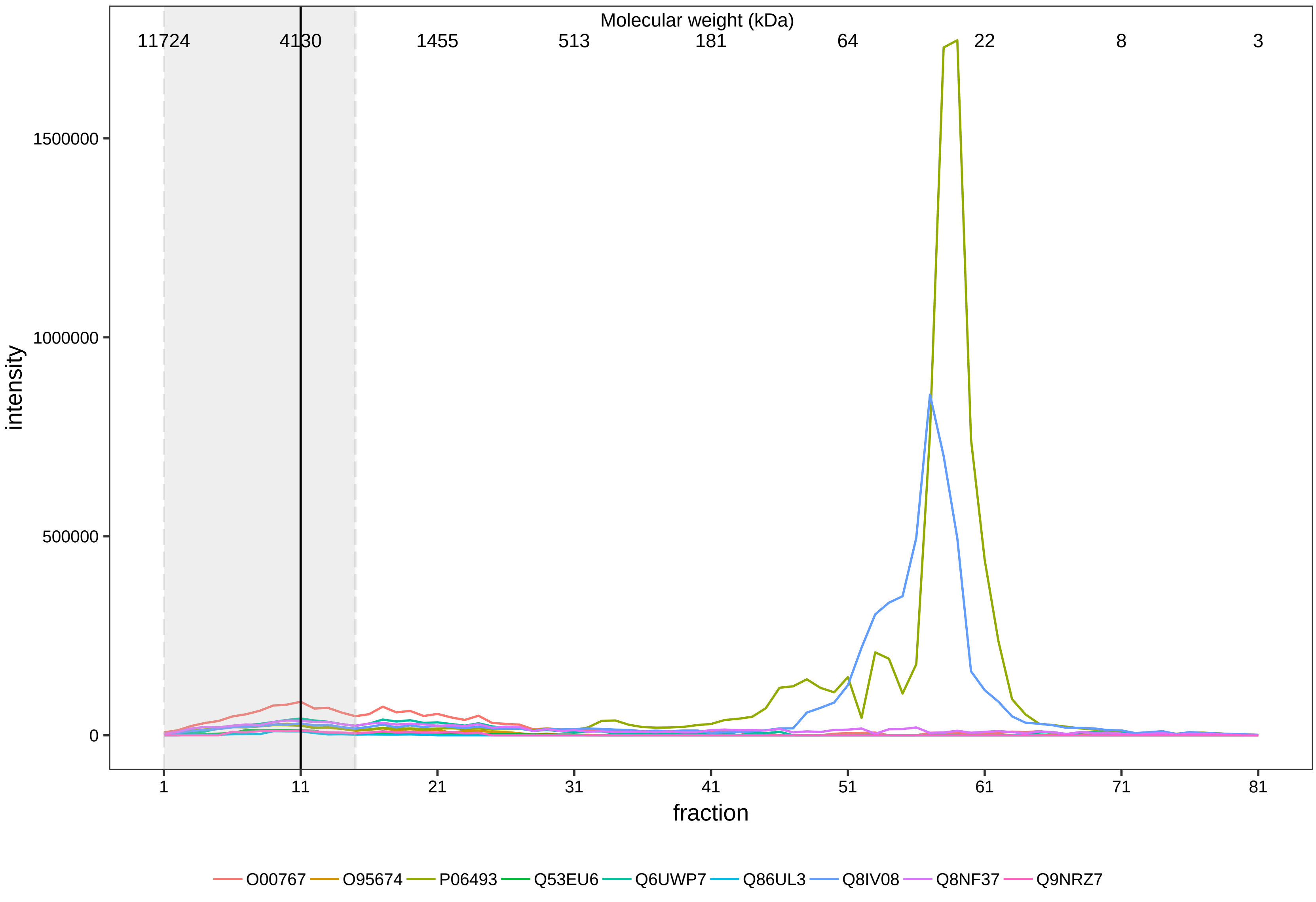

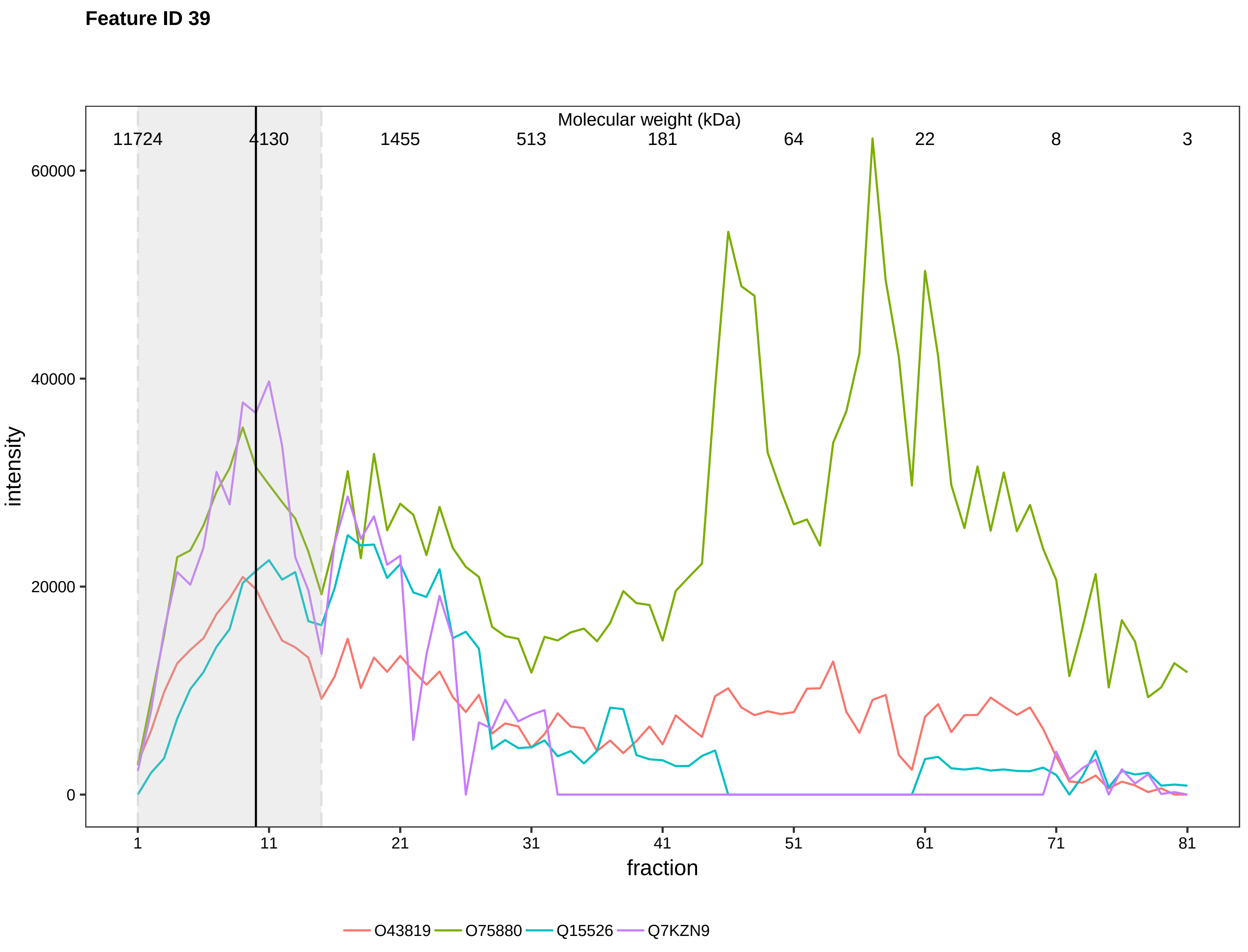

Feature ID 39
Molecular weight (kDa)
11724
4130
1455
513
181
64
22
8
3
60000
40000
20000
0
intensity
1
11
21
31
41
51
61
71
81
fraction
O43819
O75880
Q15526
Q7KZN9

Feature ID 40

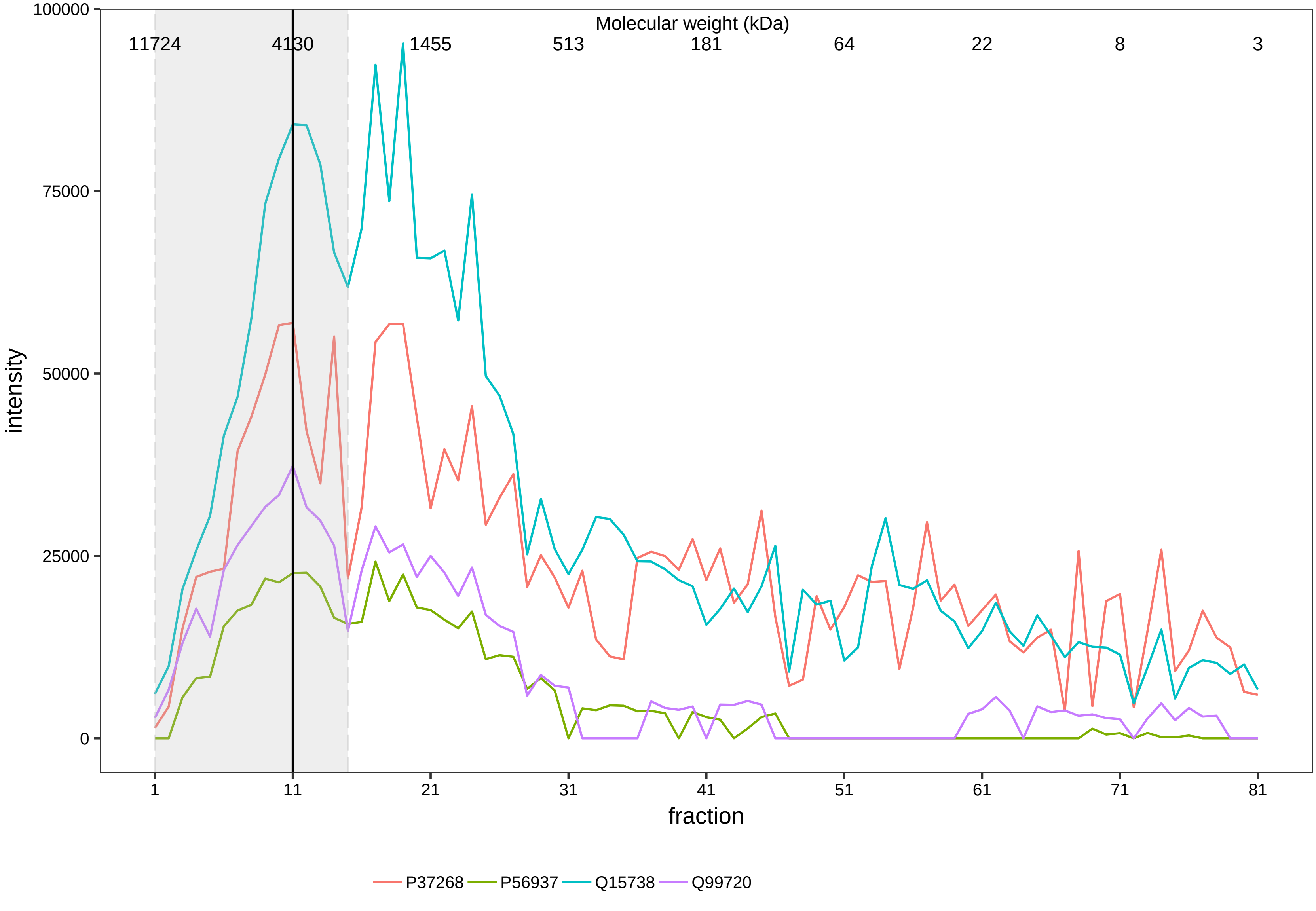

Feature ID 41

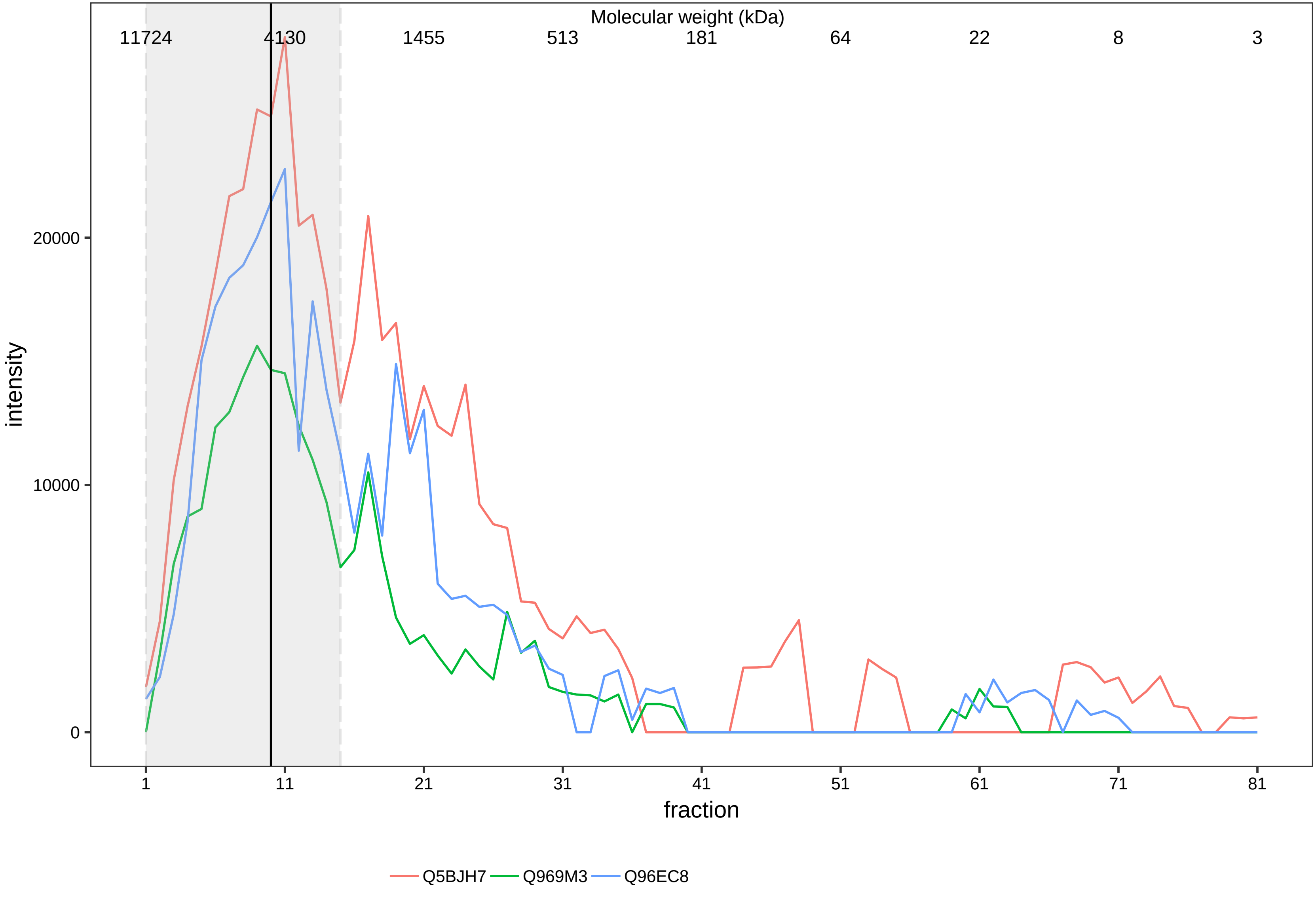

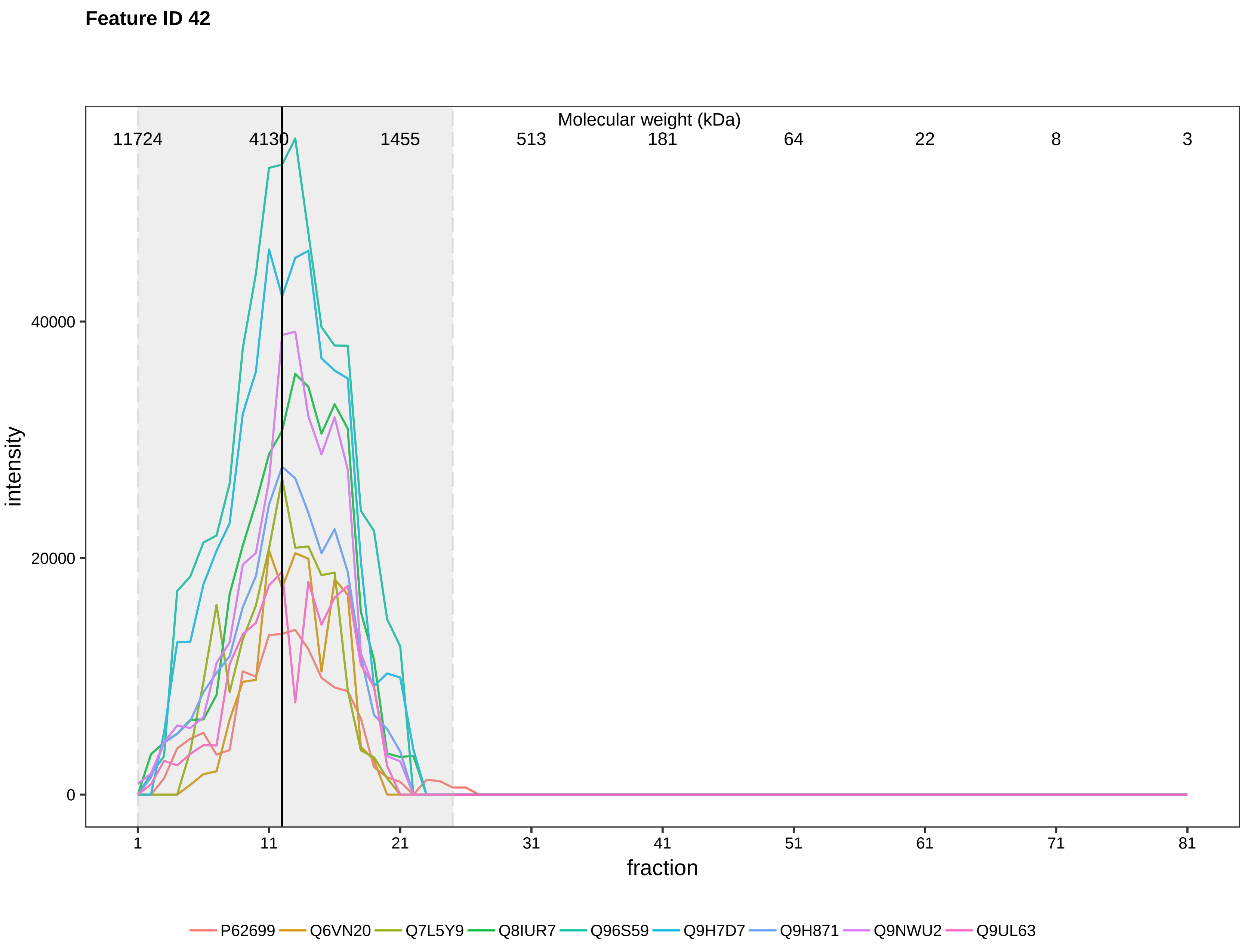

Feature ID 42
Molecular weight (kDa)
11724
4130
1455
513
181
64
22
8
3
intensity
40000
20000
0
1
11
21
31
41
51
61
71
81
fraction
P62699
Q6VN20
Q7L5Y9
Q8IUR7
Q96S59
Q9H7D7
Q9H871
Q9NWU2
Q9UL63

Feature ID 43

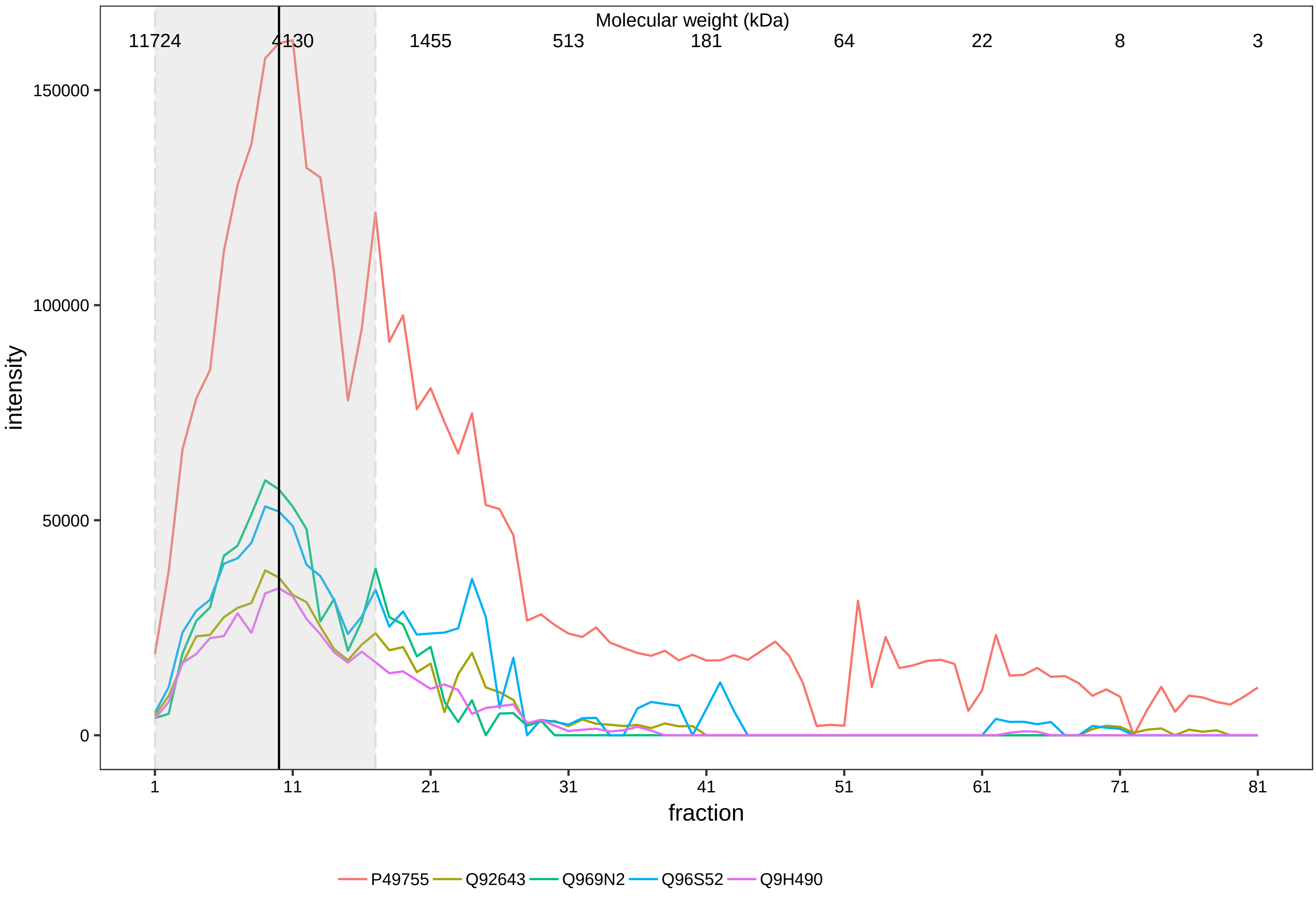

Feature ID 44

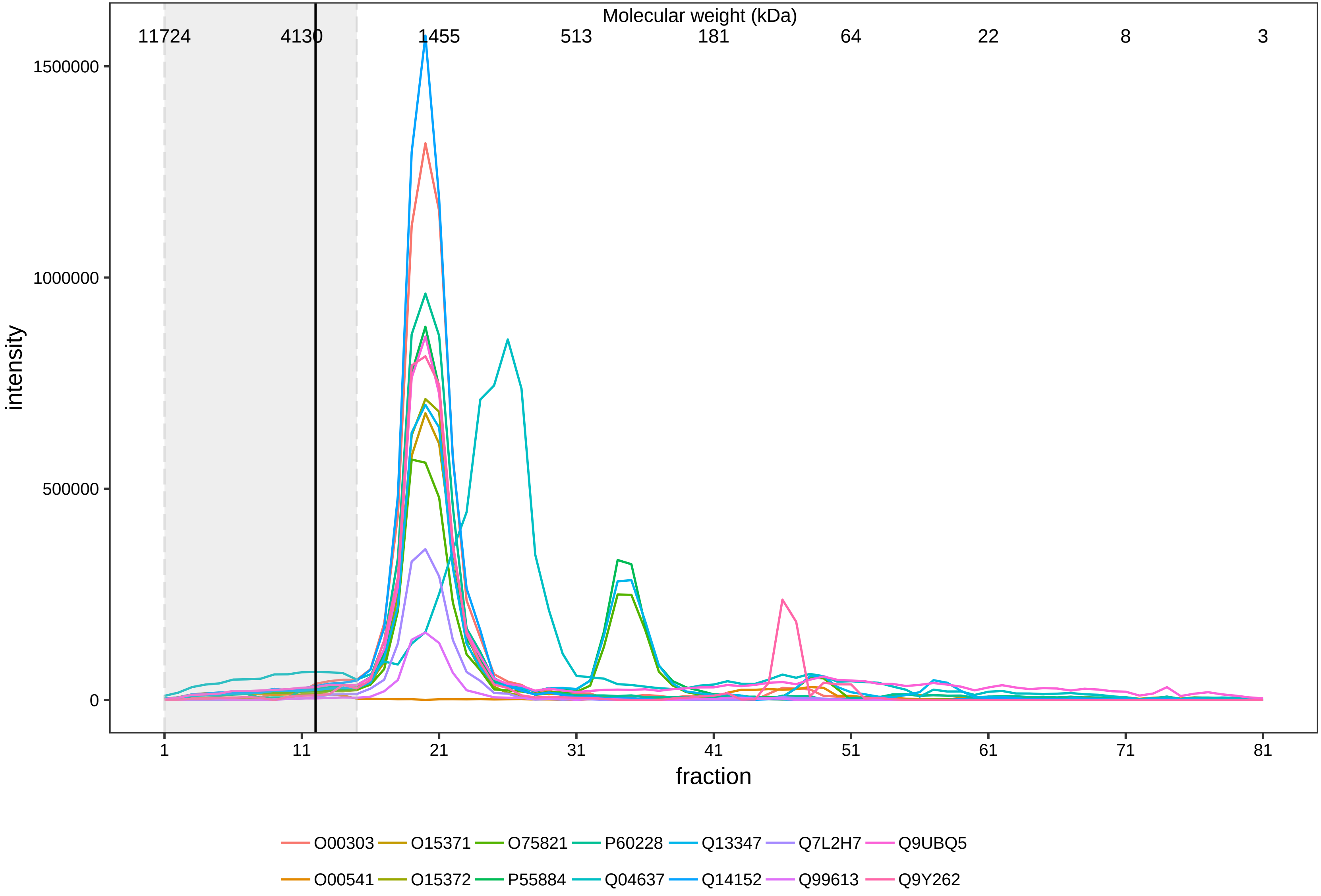

Feature ID 45

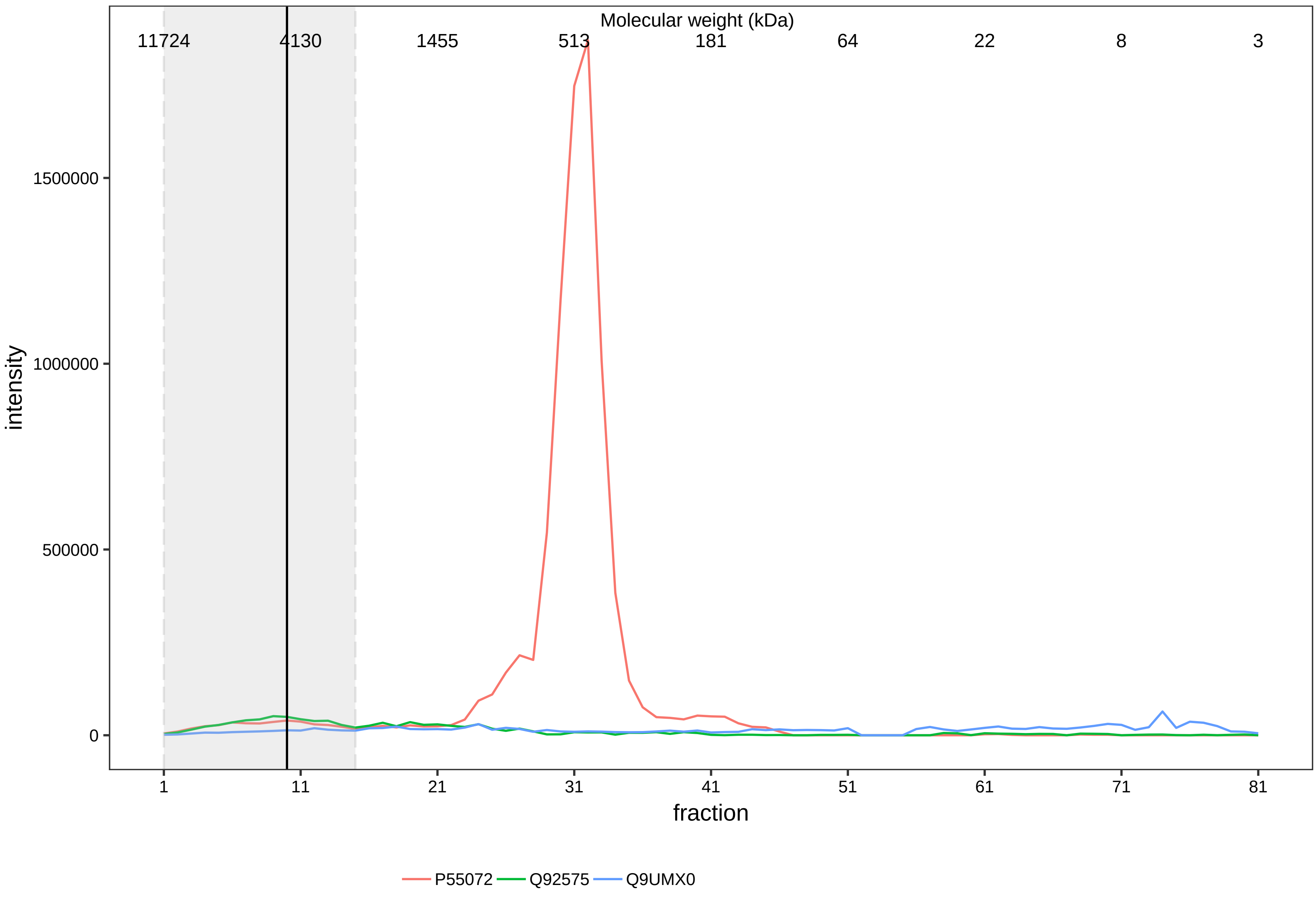

Feature ID 46

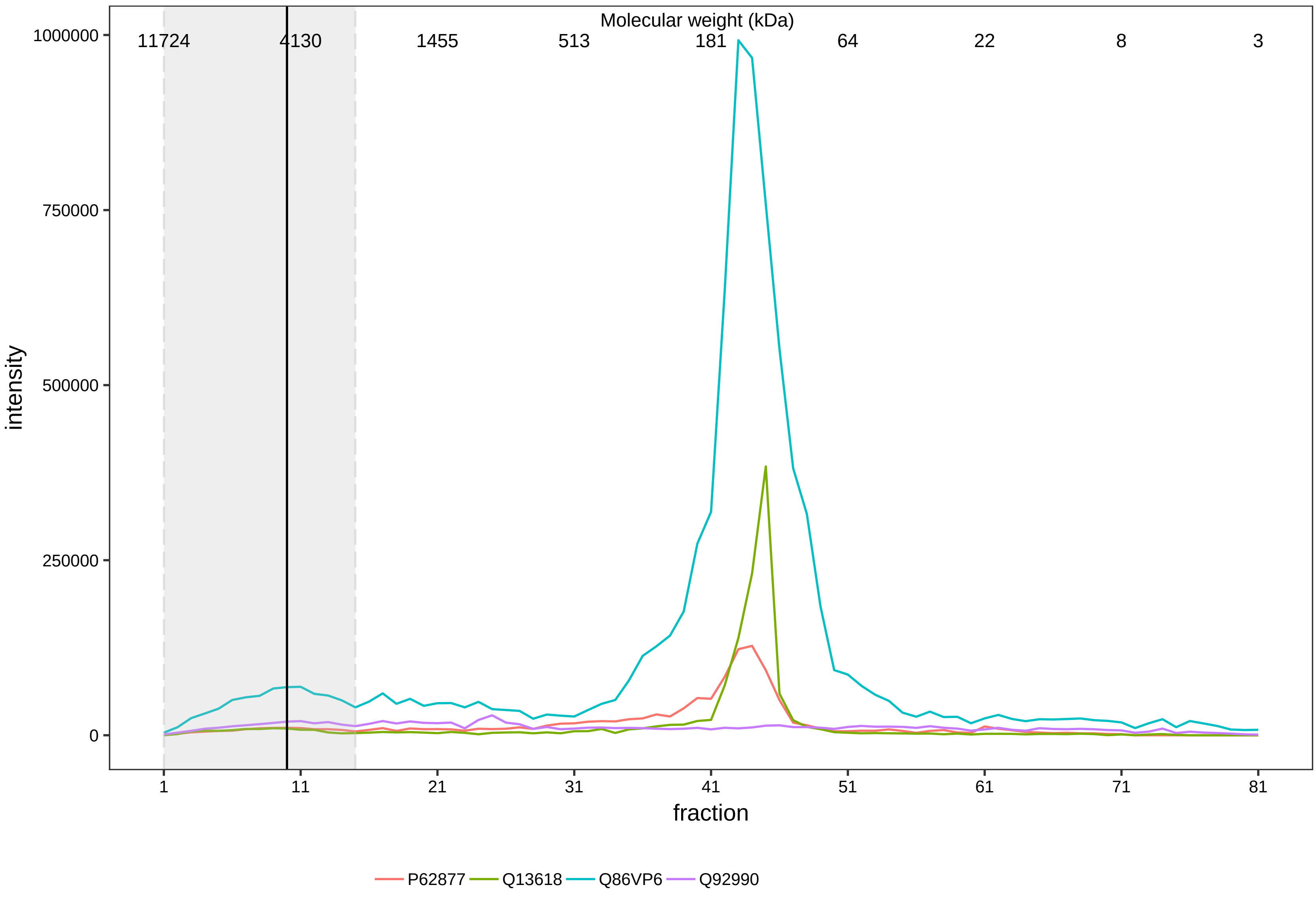

Feature ID 47

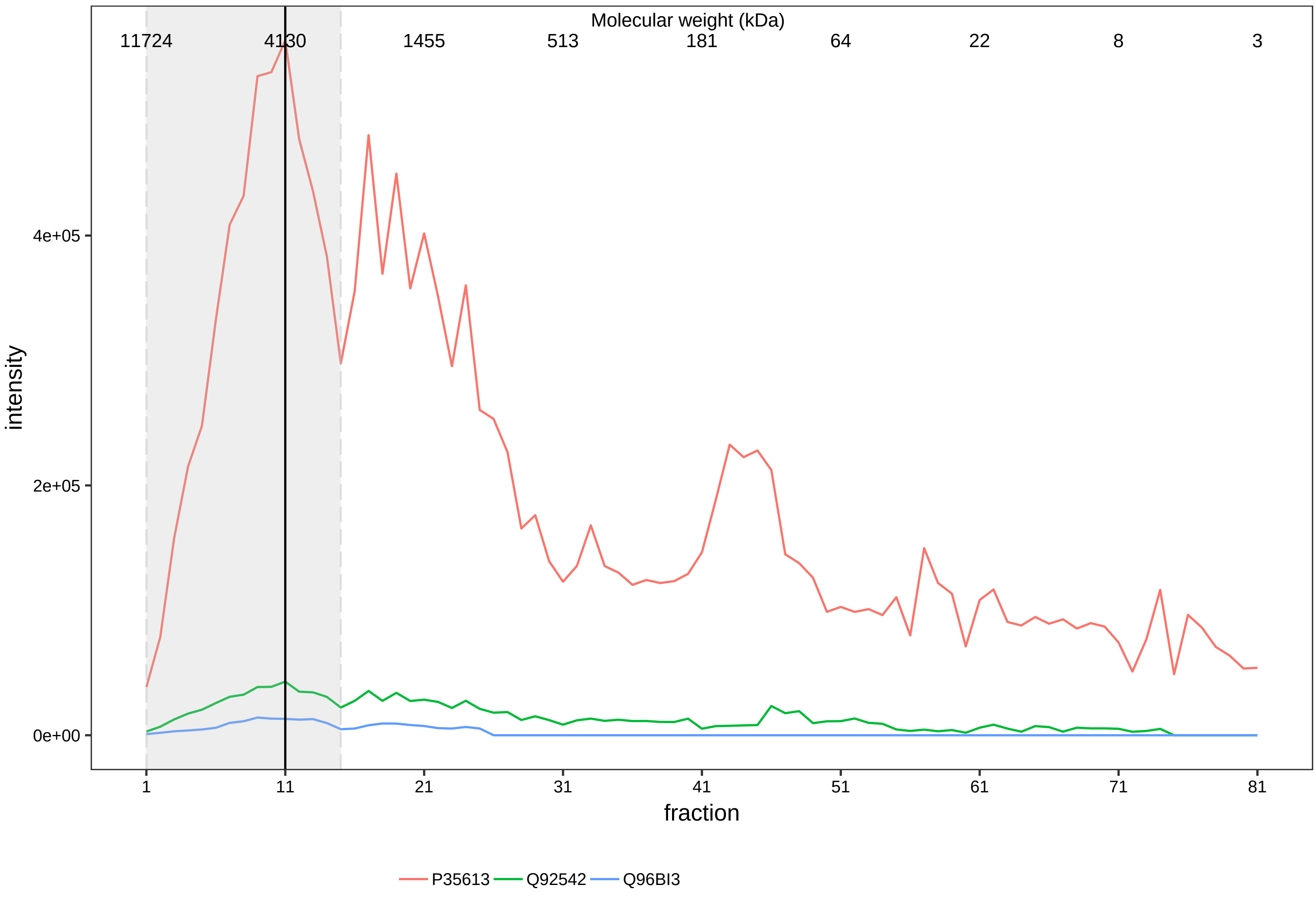

Feature ID 48

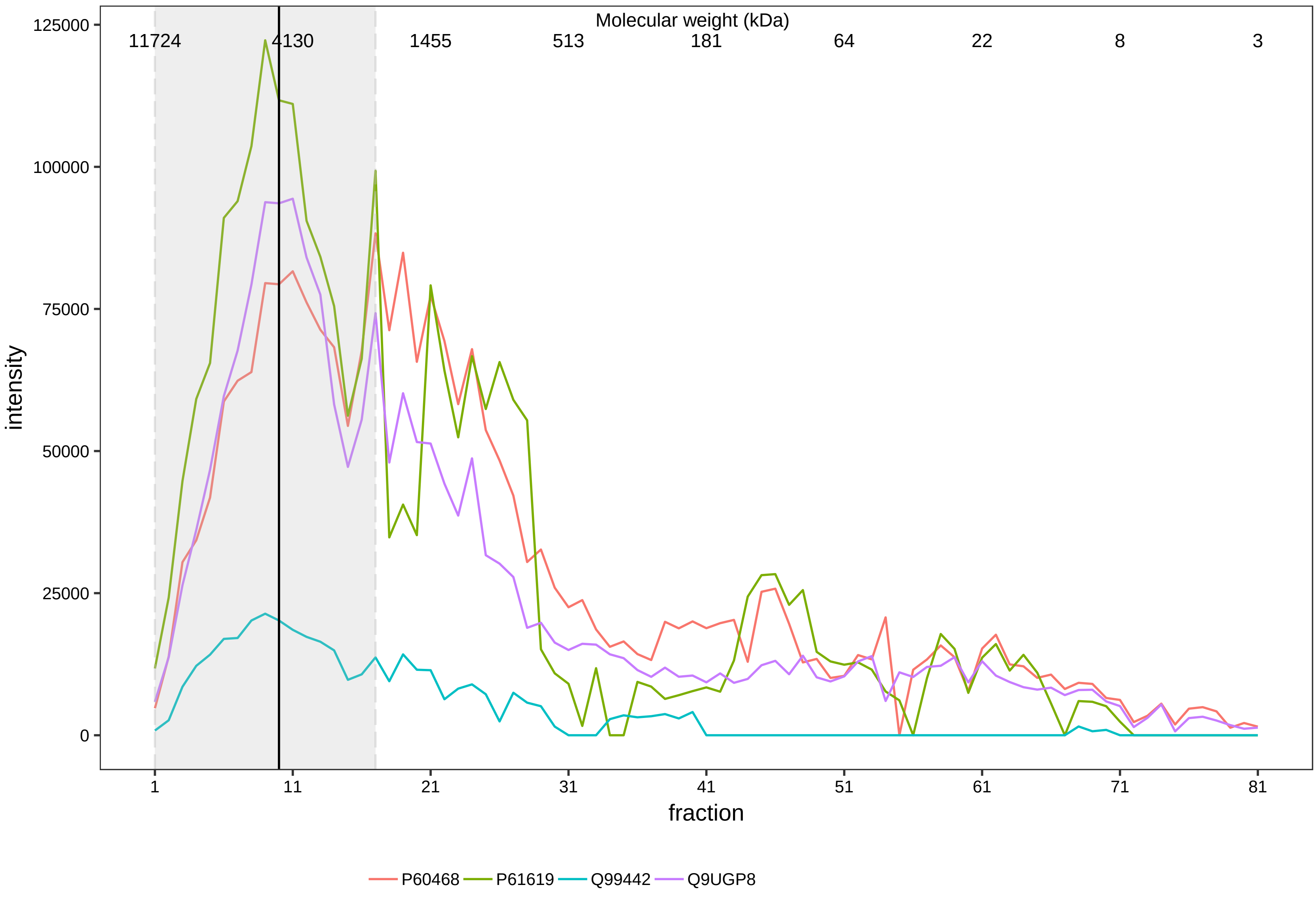

Feature ID 49

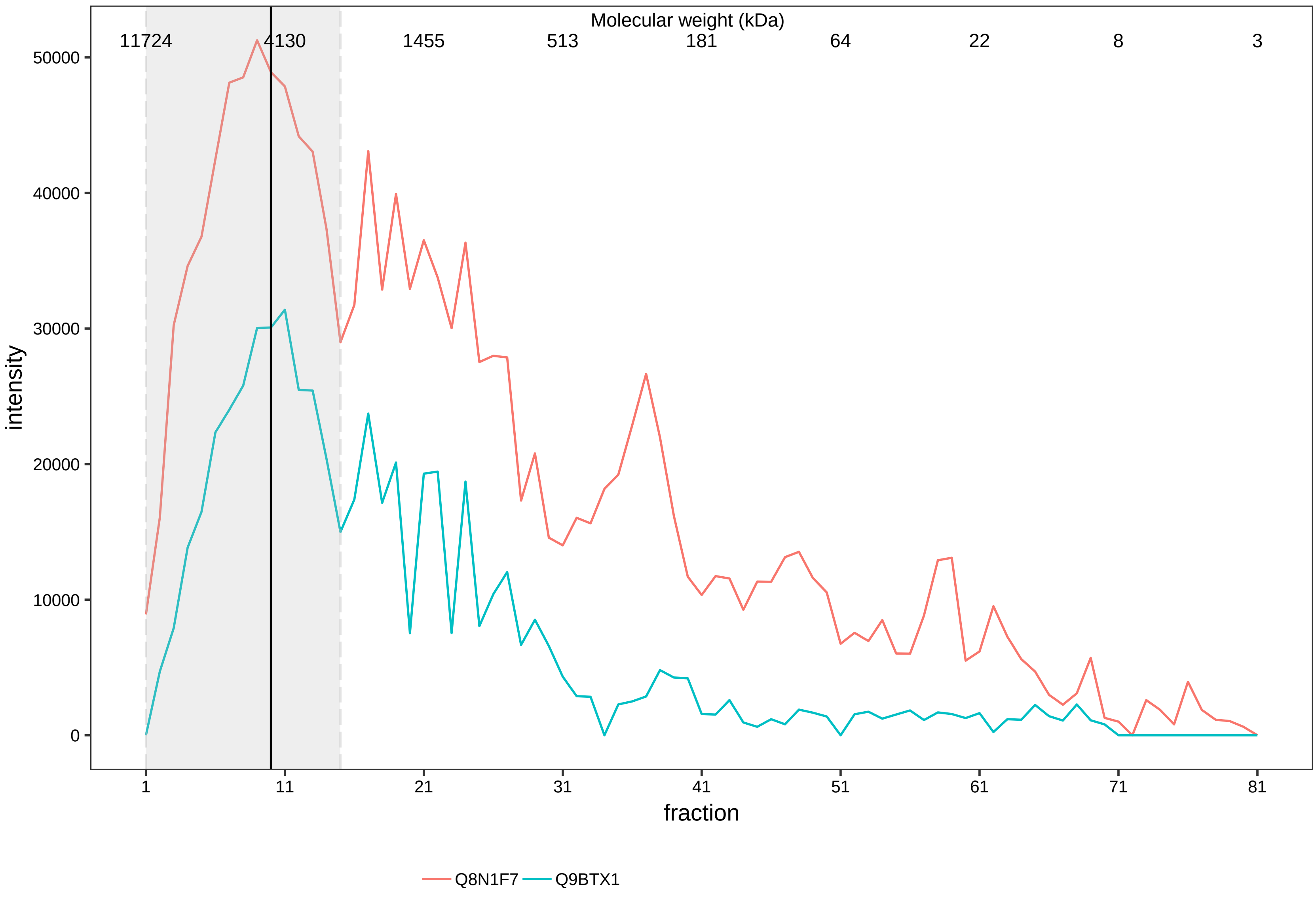

Feature ID 50

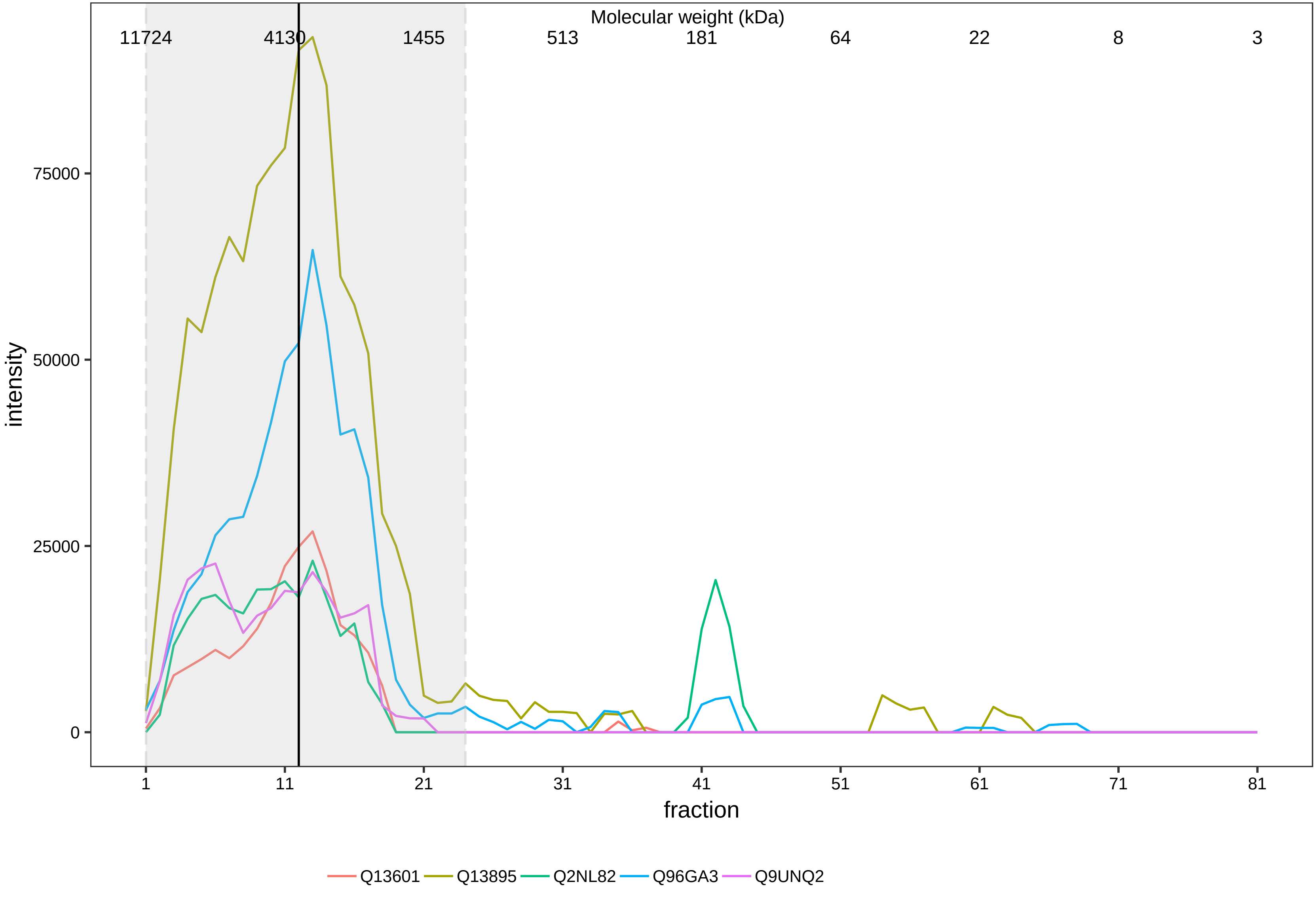

Feature ID 51

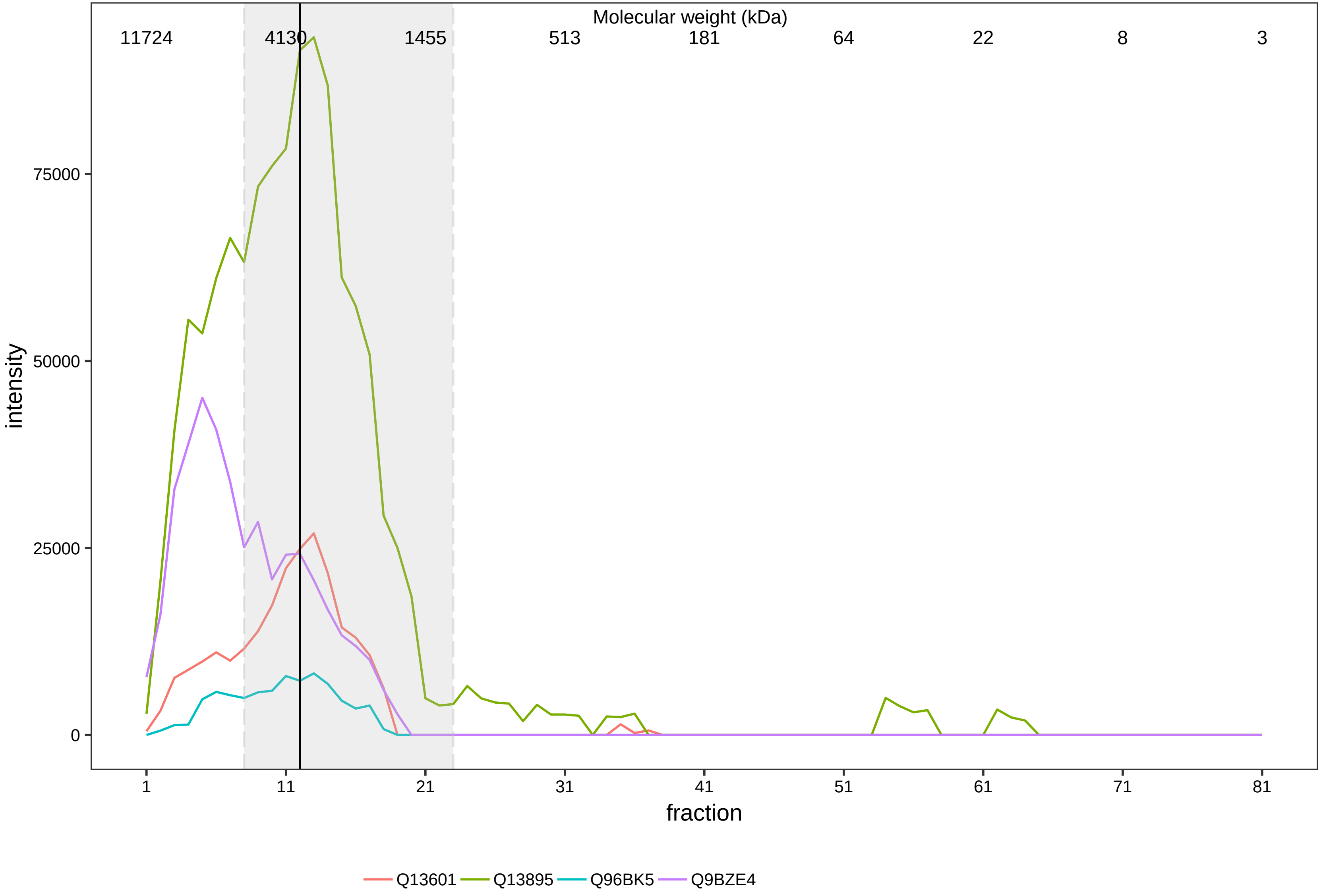

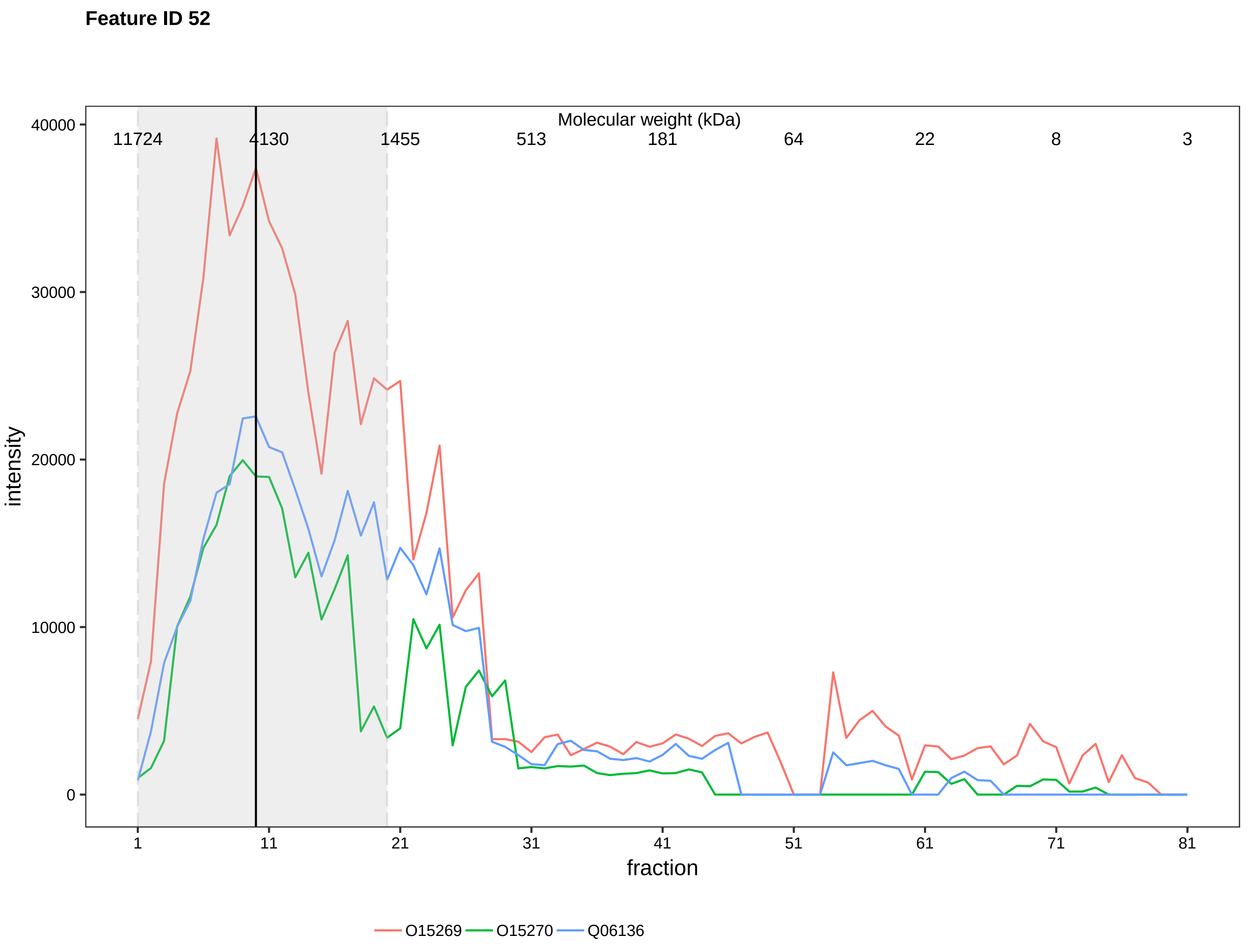
Feature ID 52
Molecular weight (kDa)
11724
4130
1455
513
181
64
22
8
3
40000
30000
20000
10000
0
intensity
1
11
21
31
41
51
61
71
81
fraction
O15269
O15270
Q06136

Feature ID 53

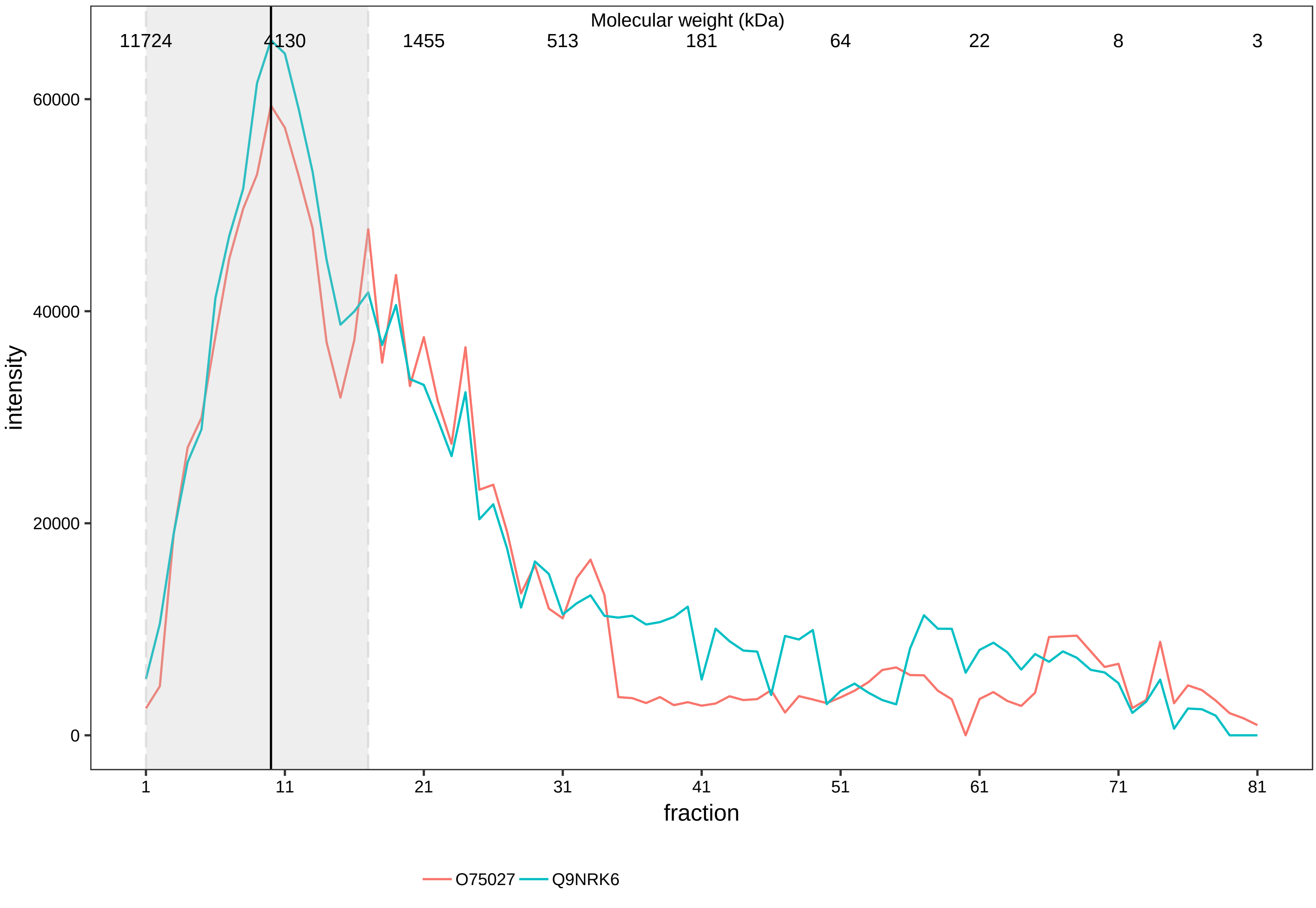

Feature ID 54

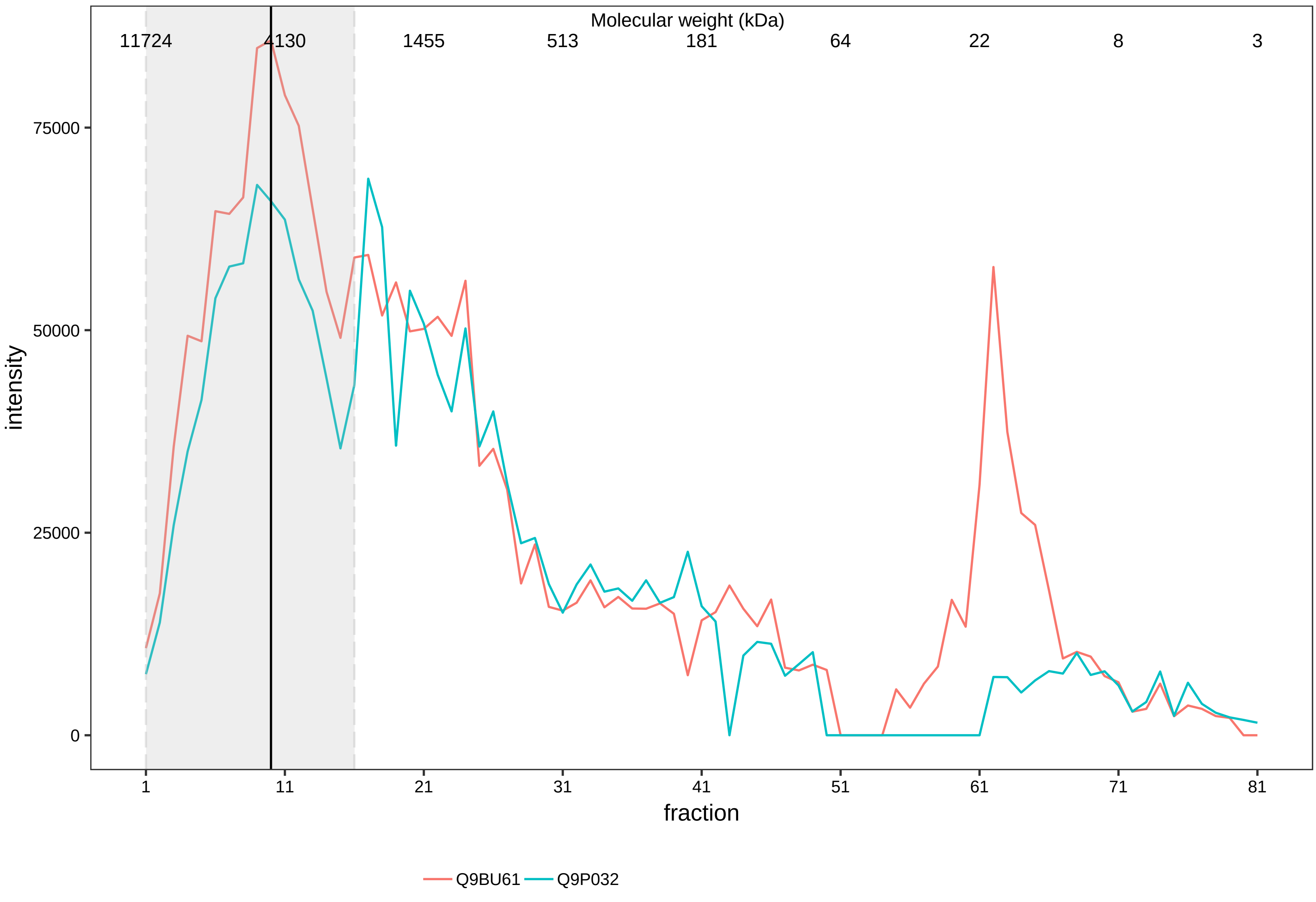

Feature ID 55

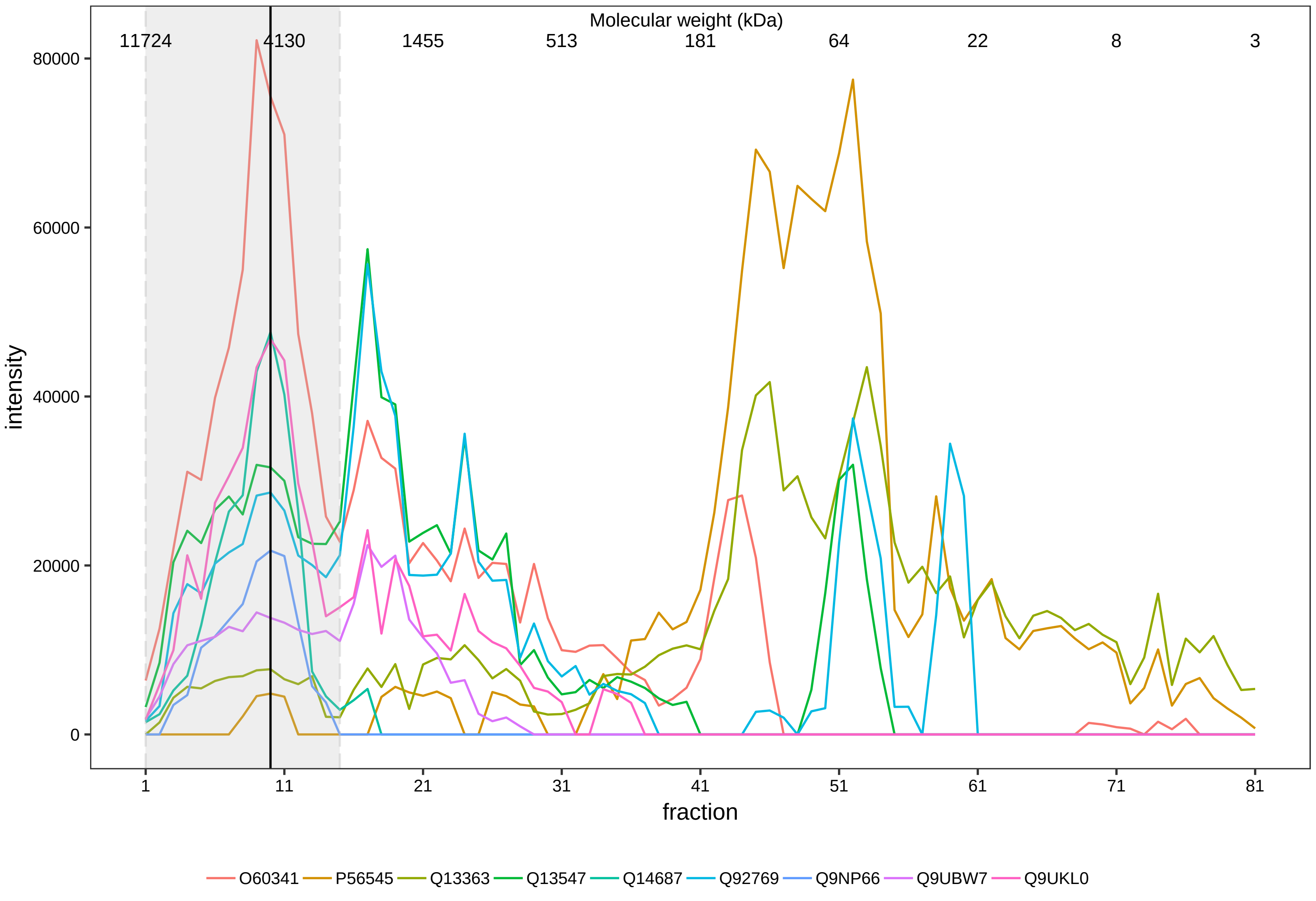

Feature ID 56

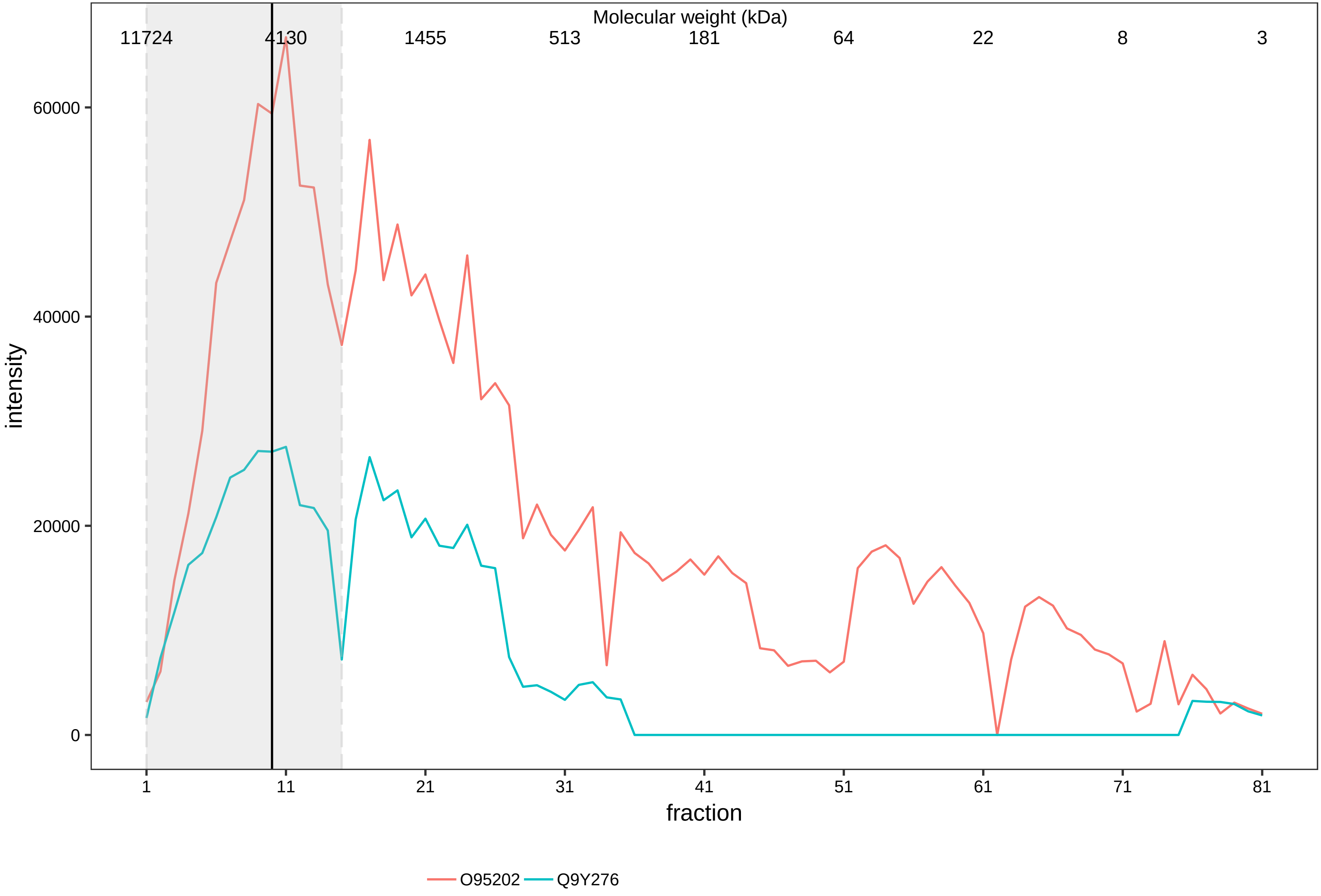

Feature ID 57

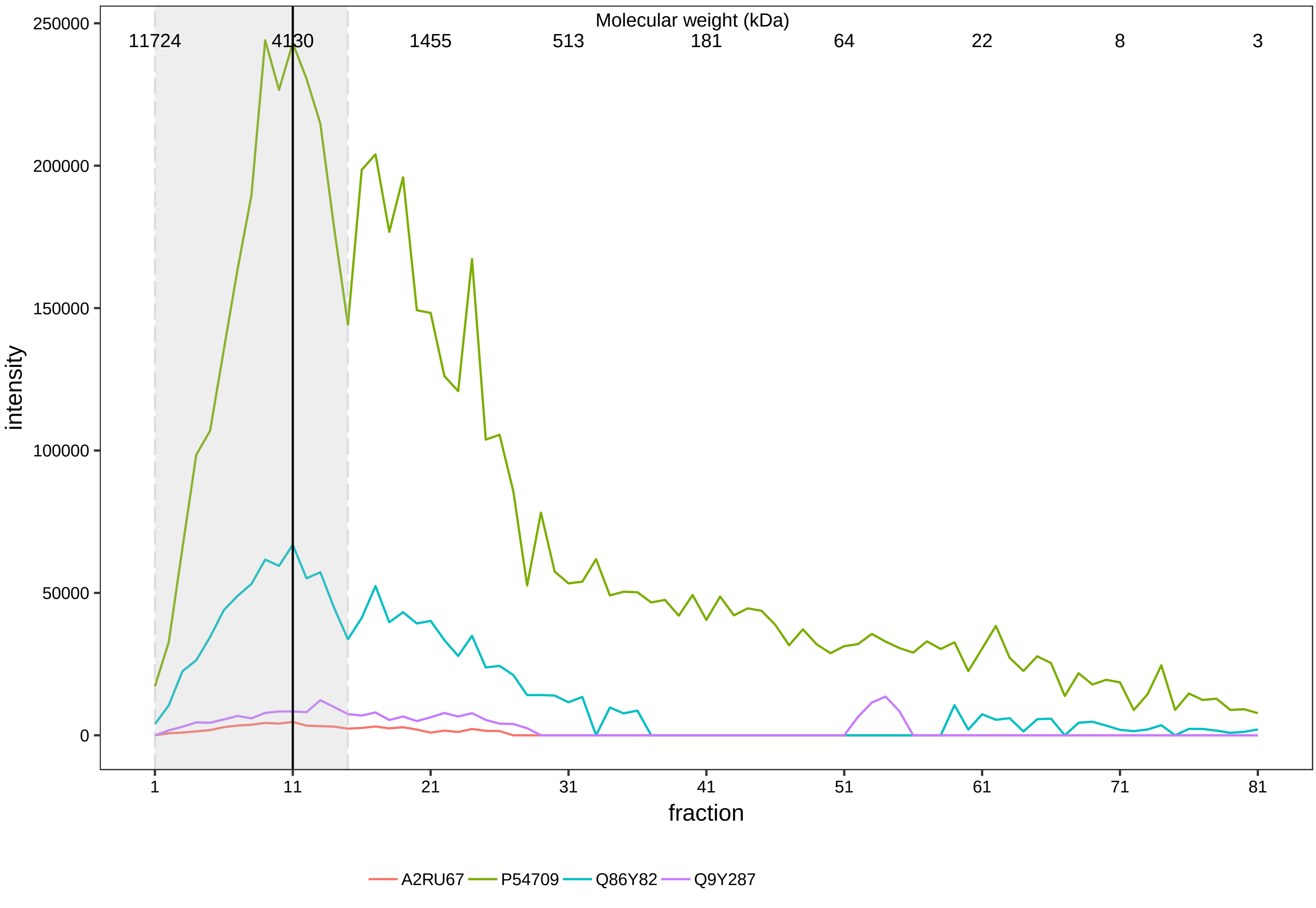

Feature ID 58

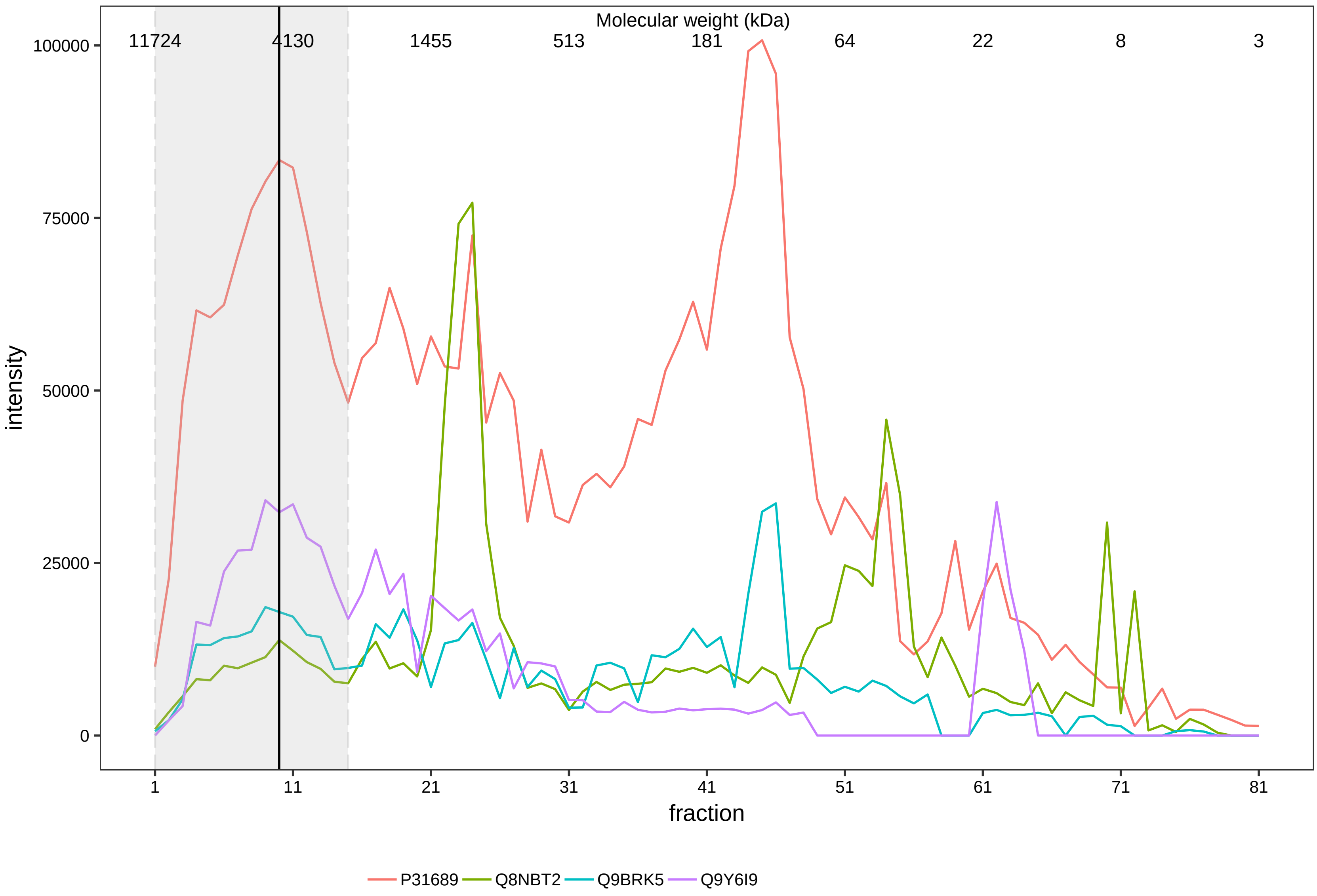

Feature ID 59

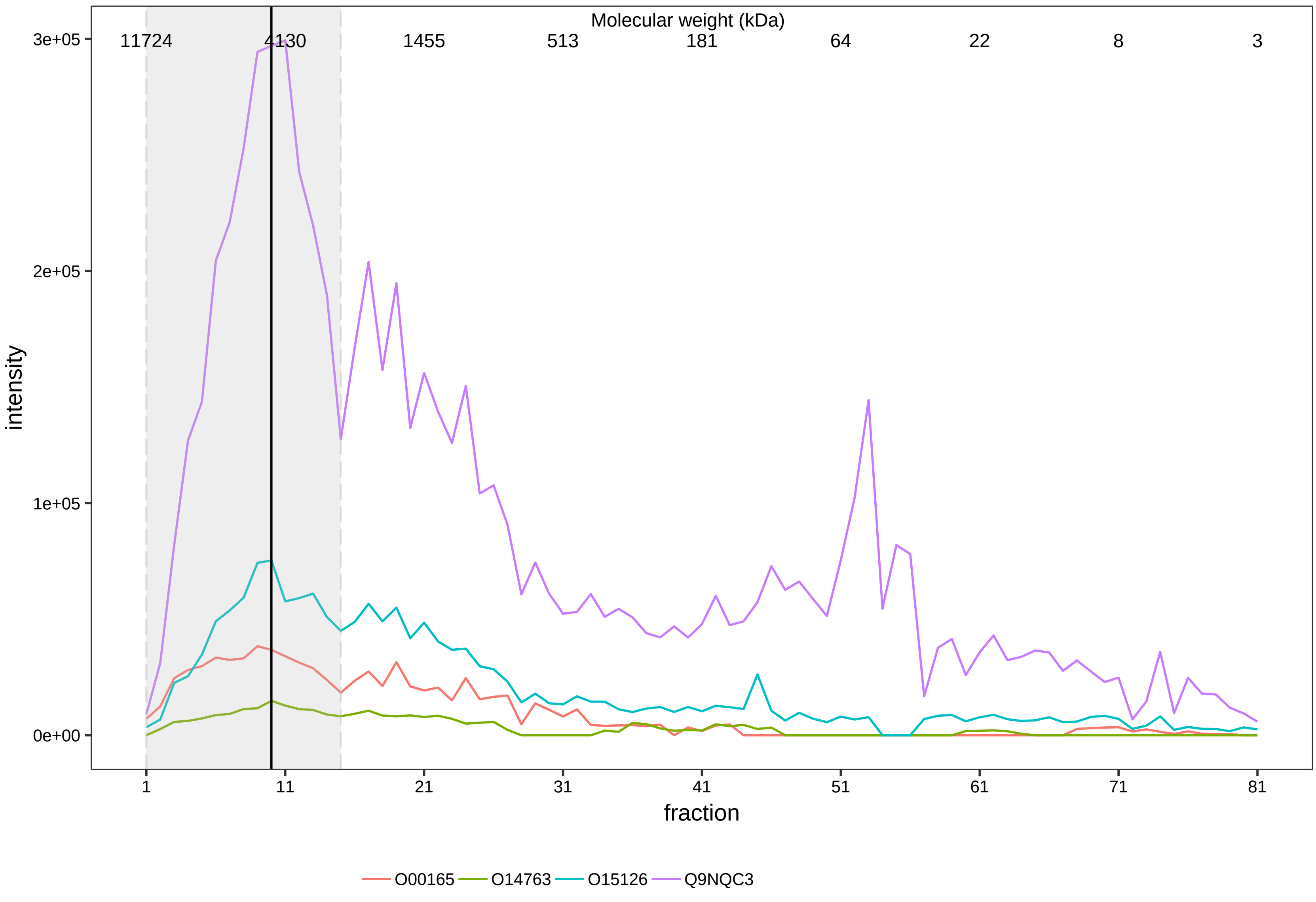

Feature ID 60

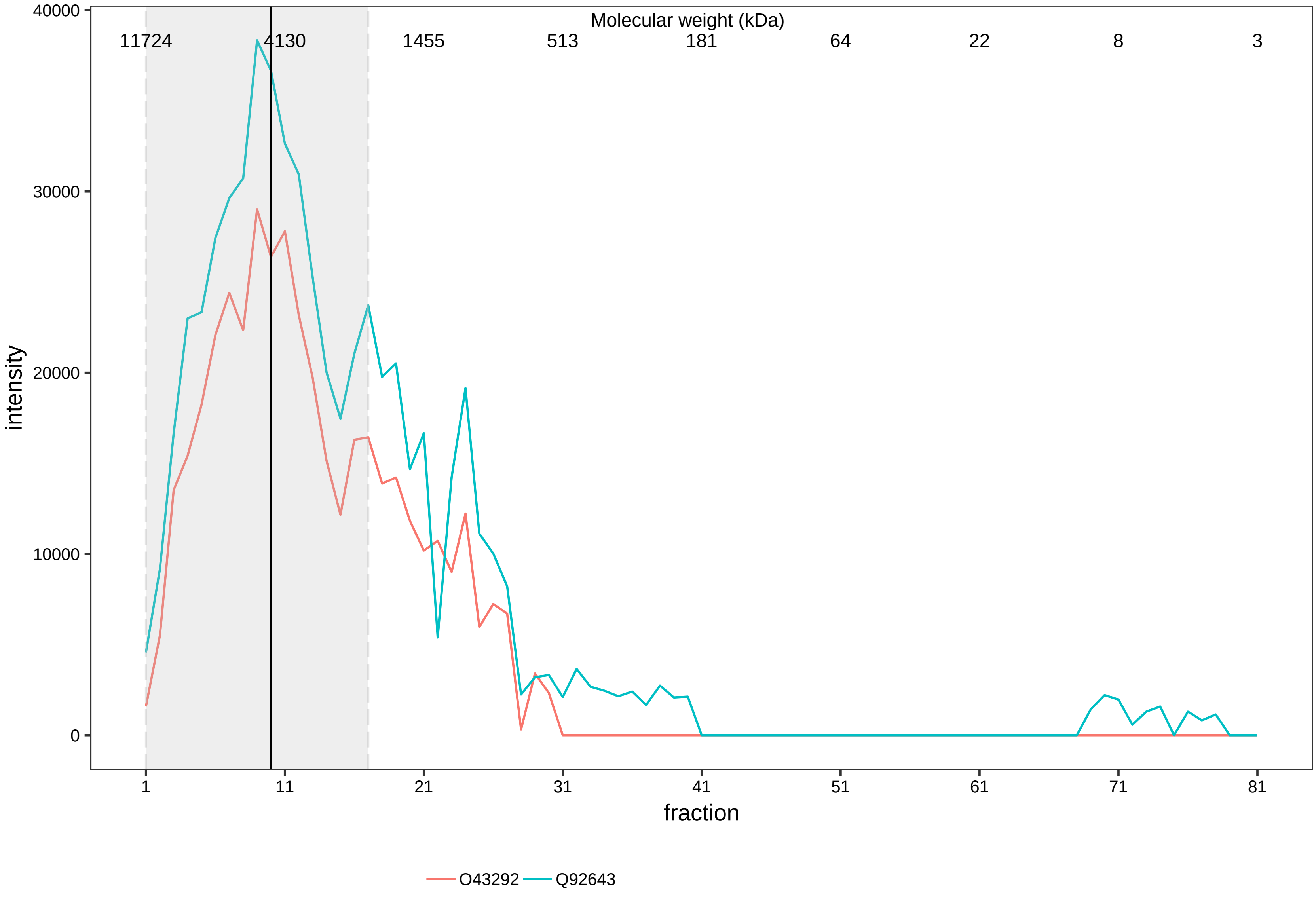

Feature ID 61

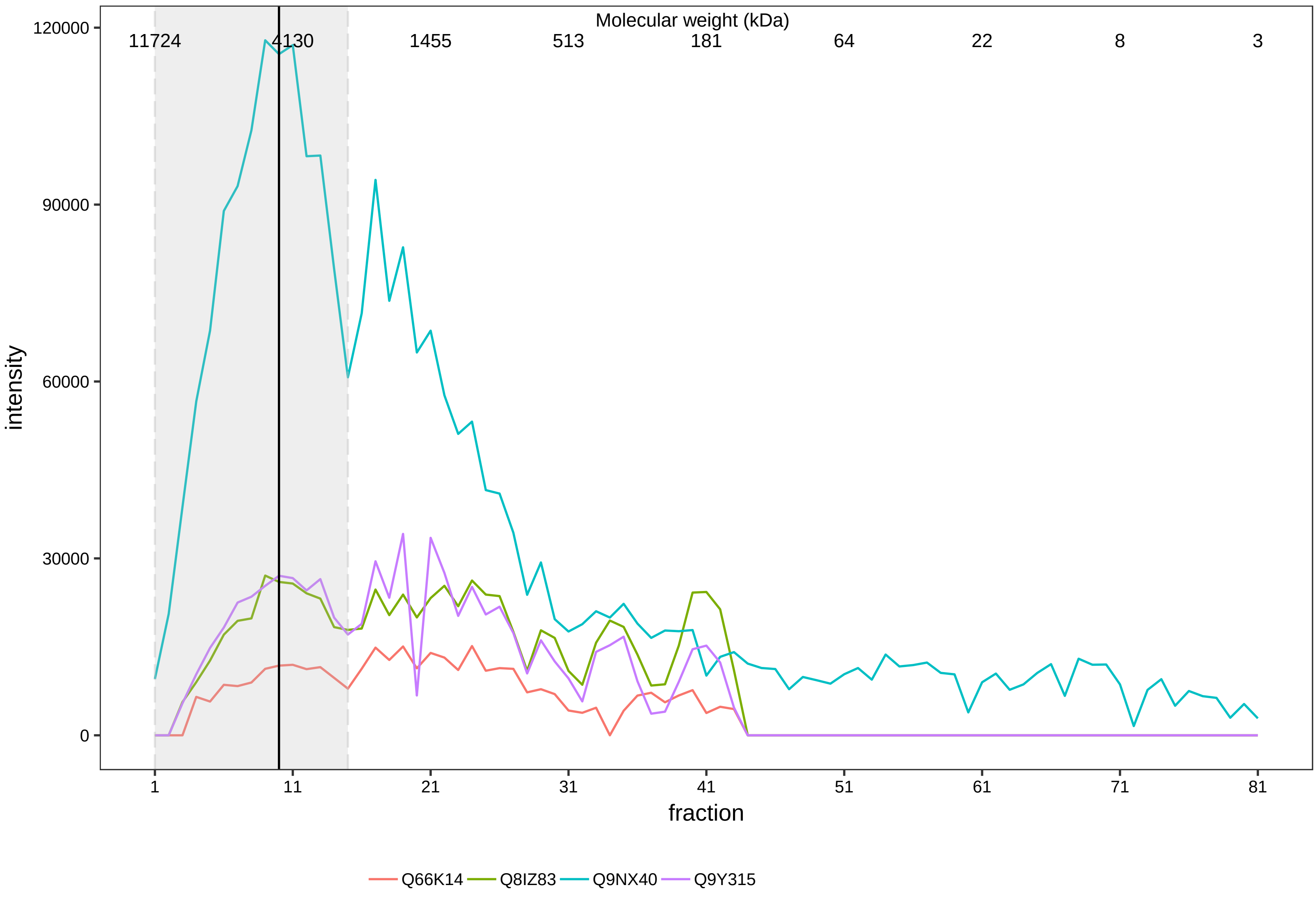

Feature ID 62

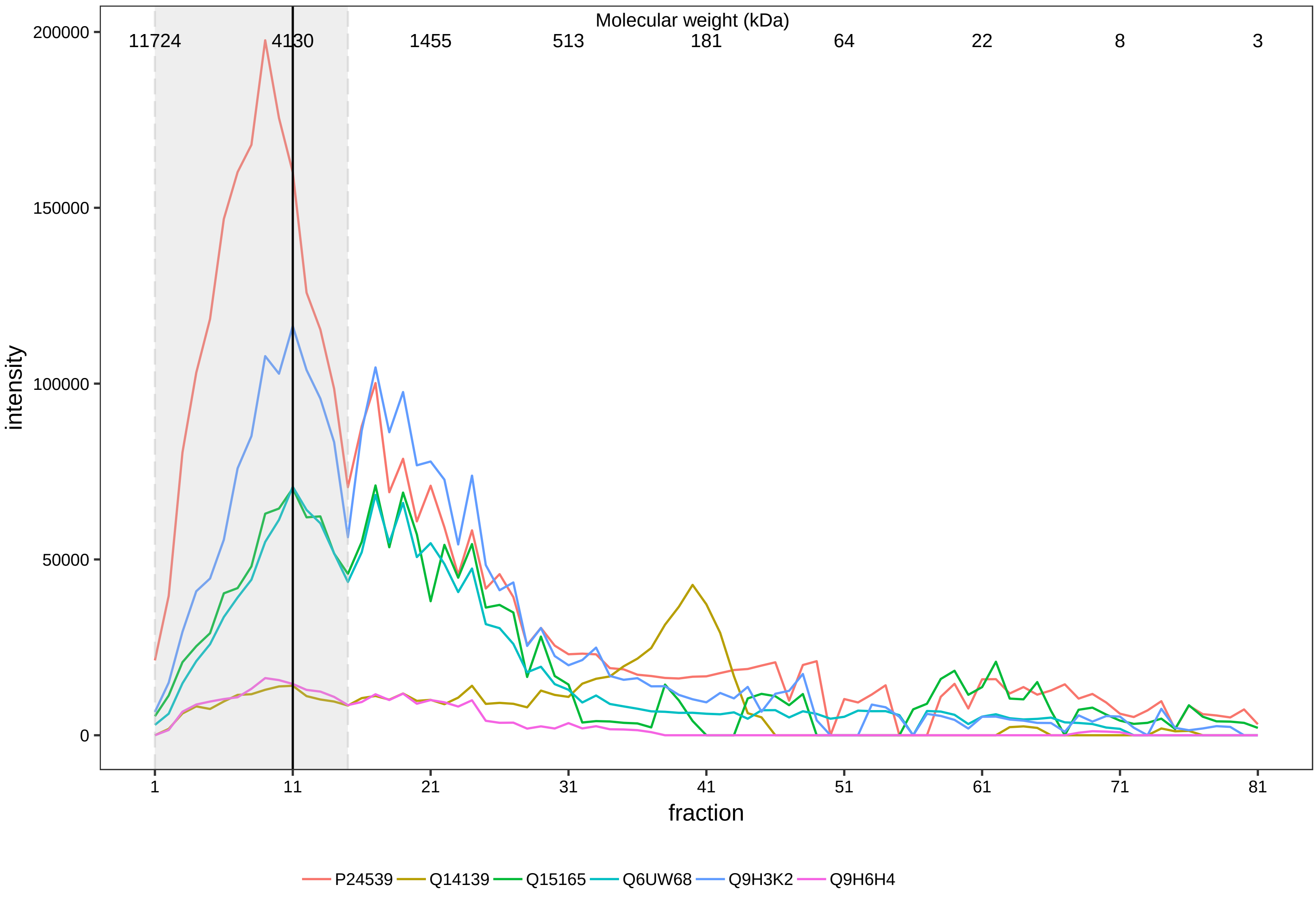

Feature ID 63

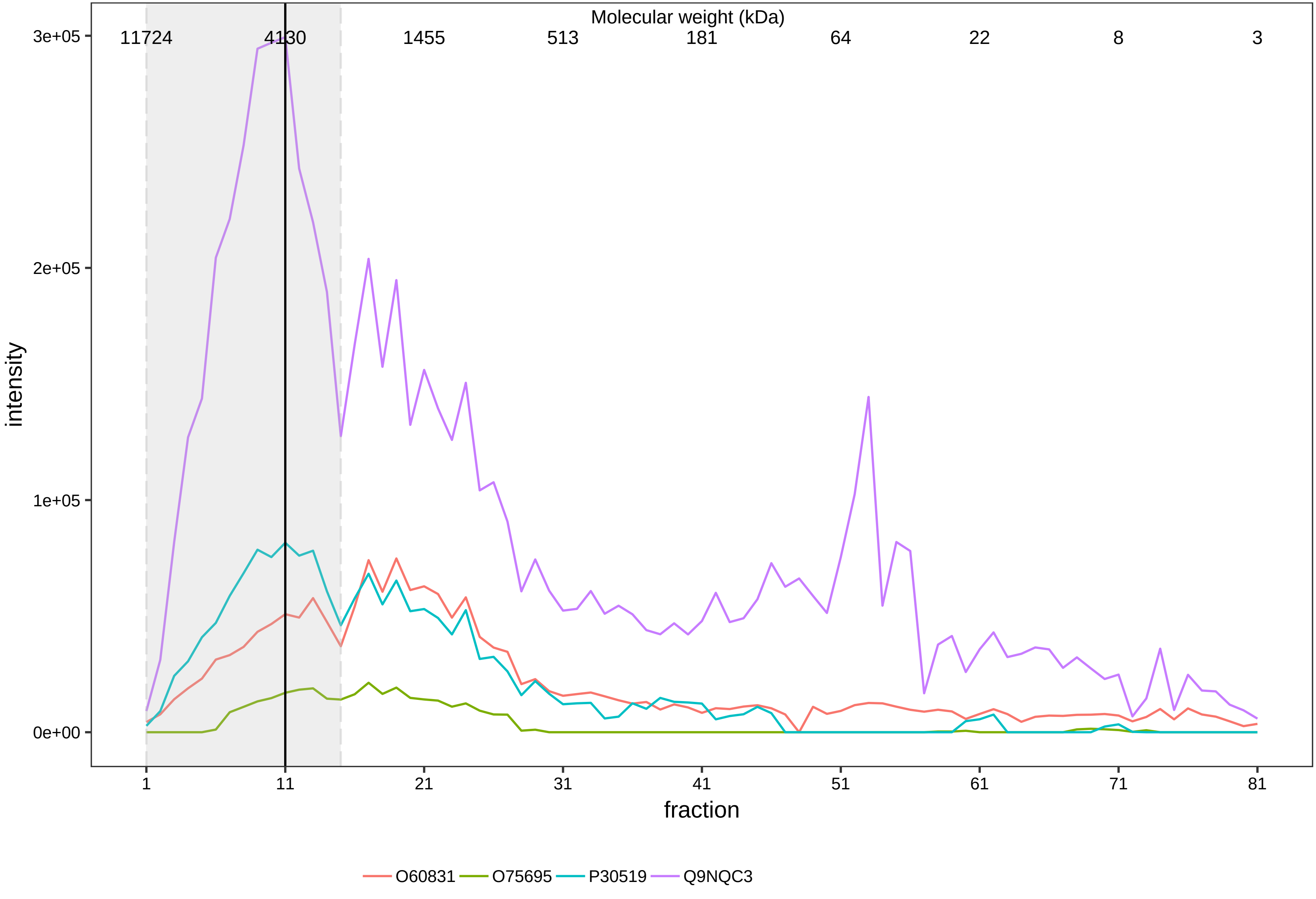

Feature ID 64

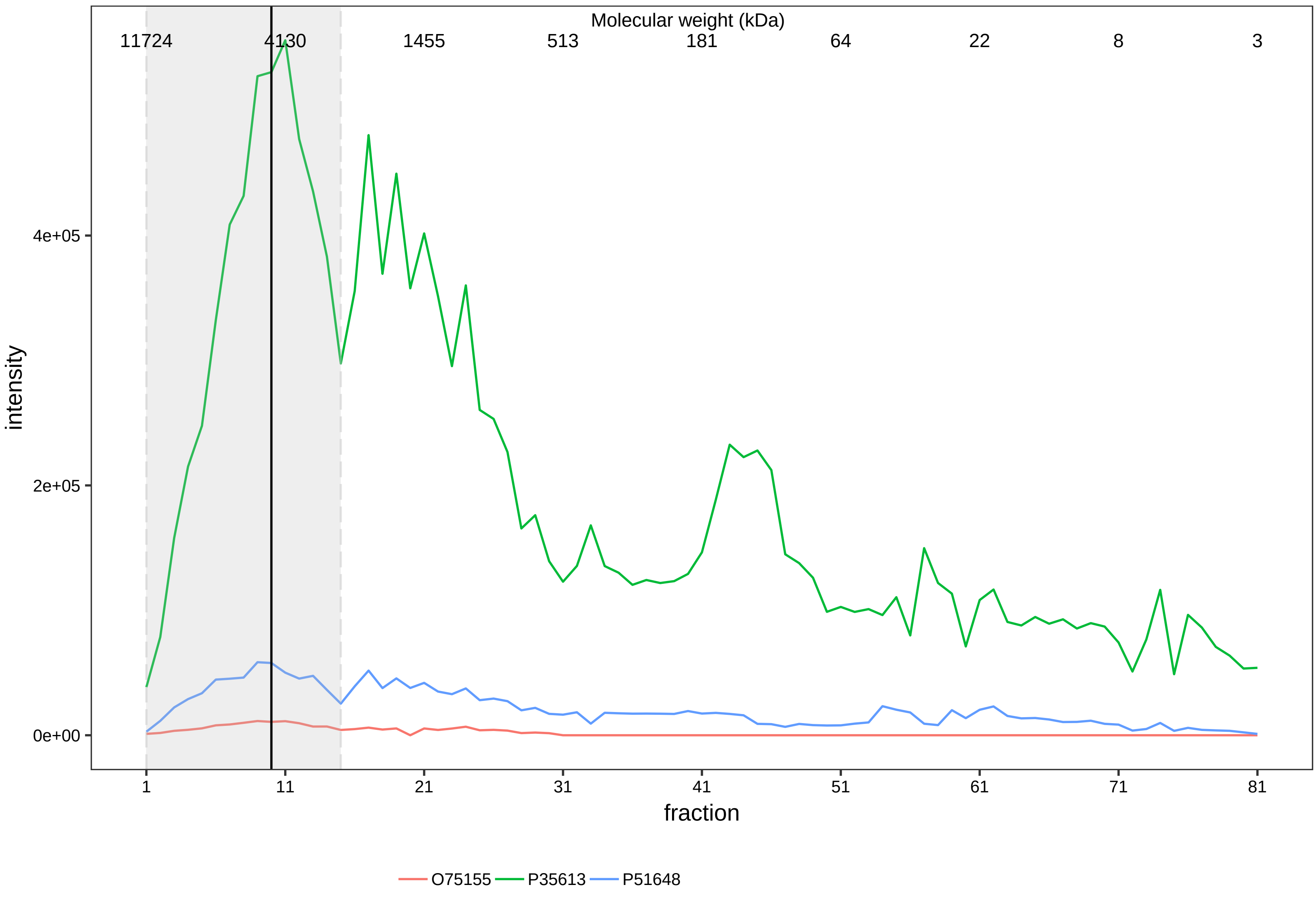

Feature ID 65

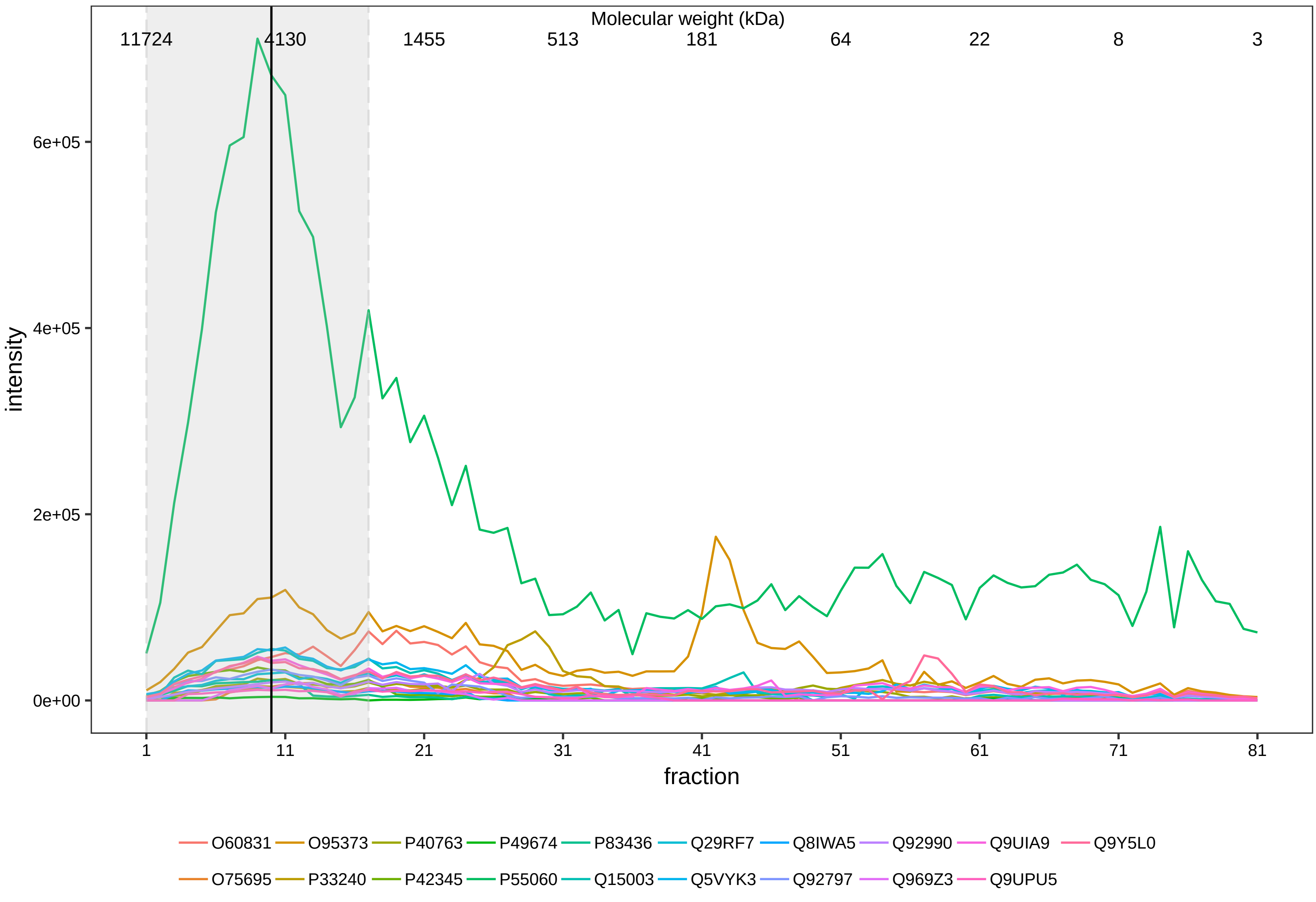

Feature ID 66

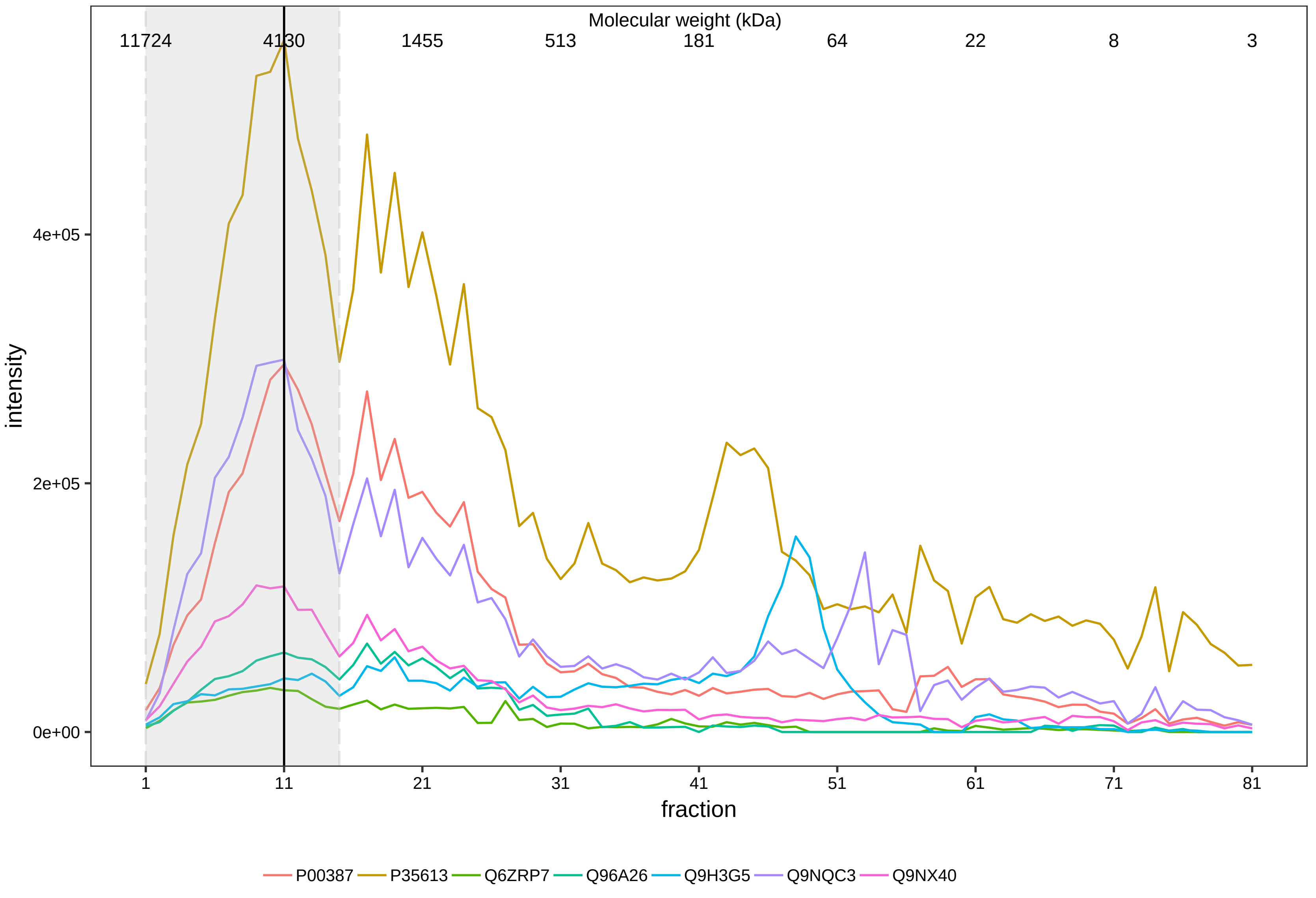

Feature ID 67

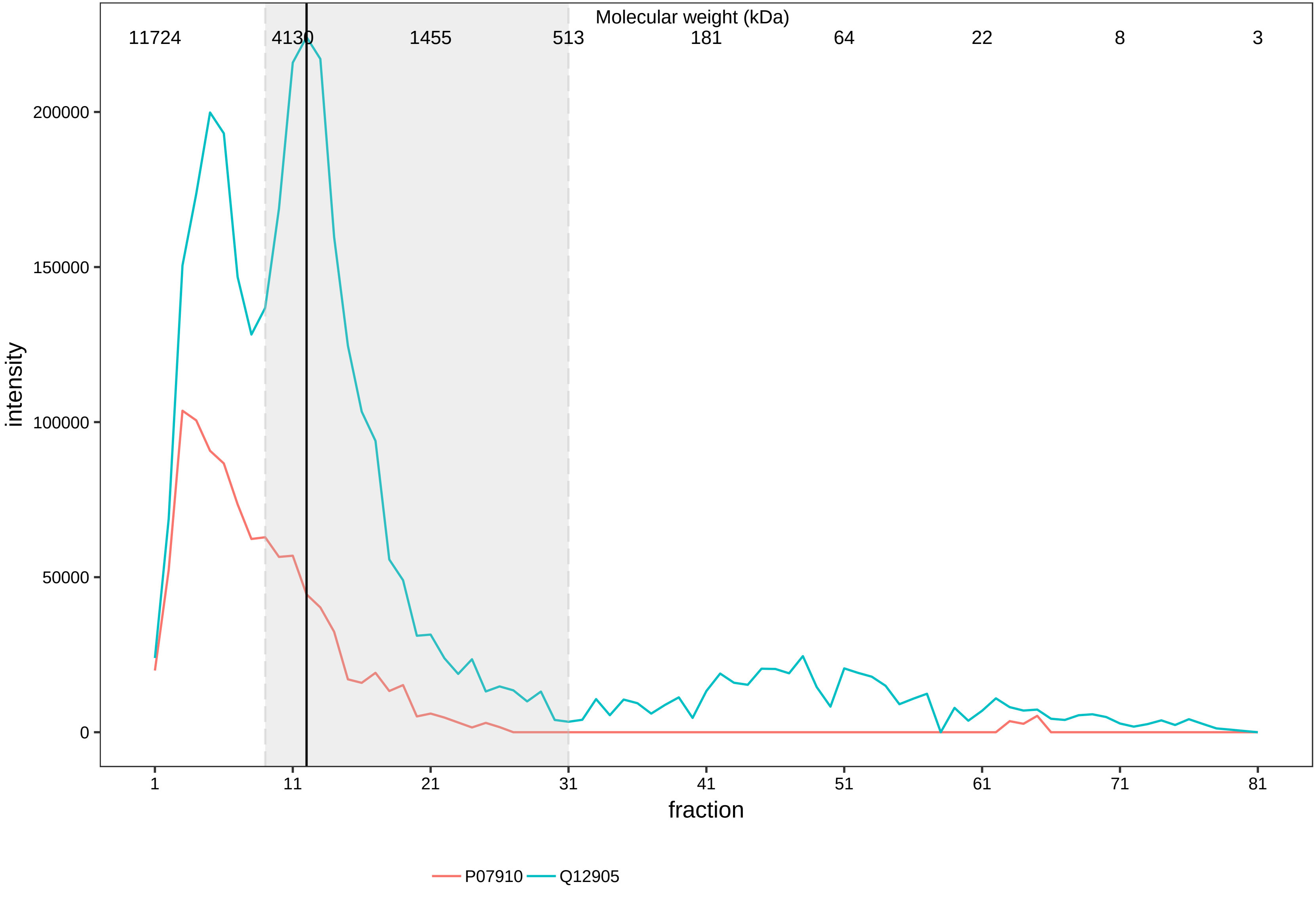

Feature ID 68

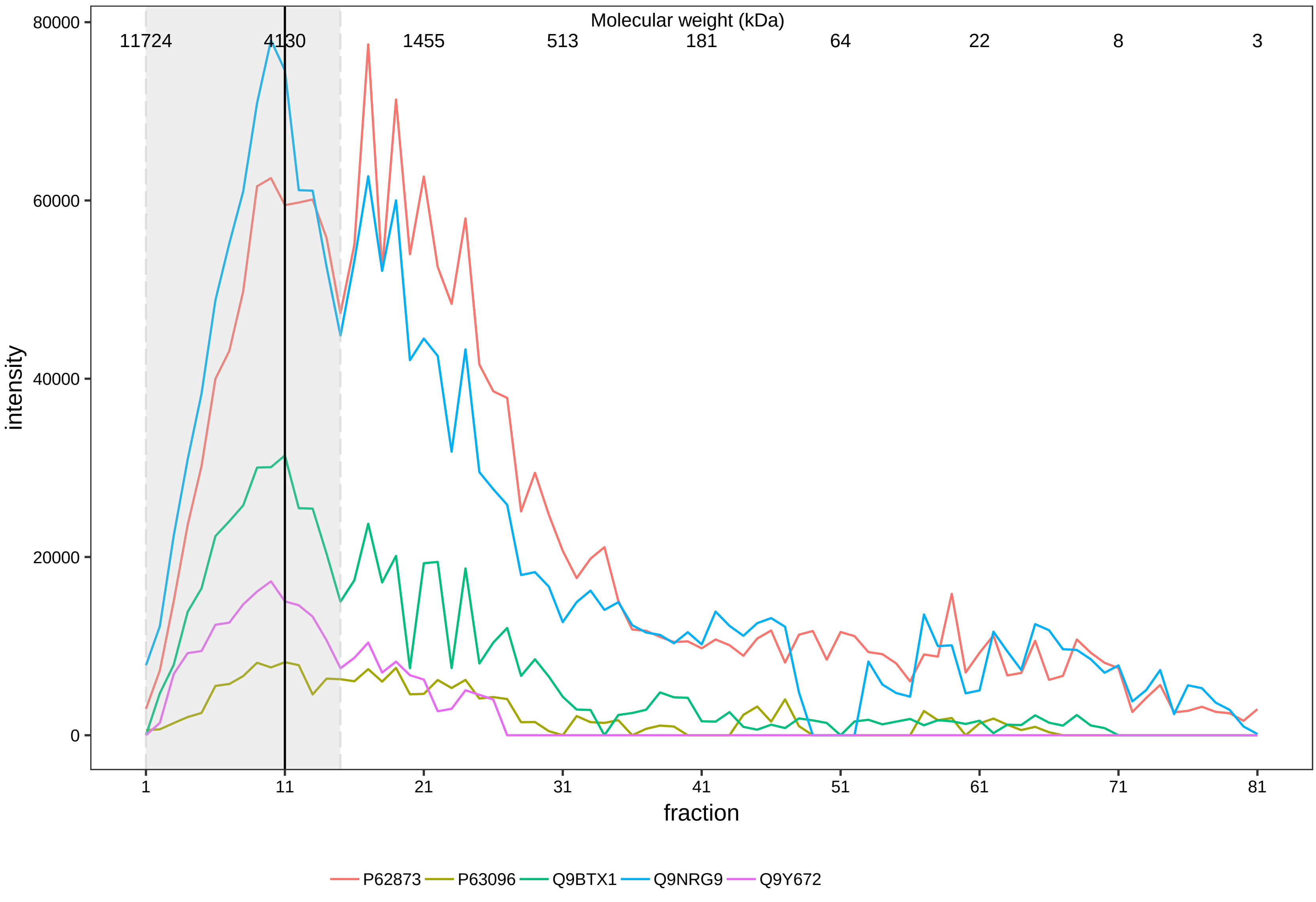

Feature ID 69

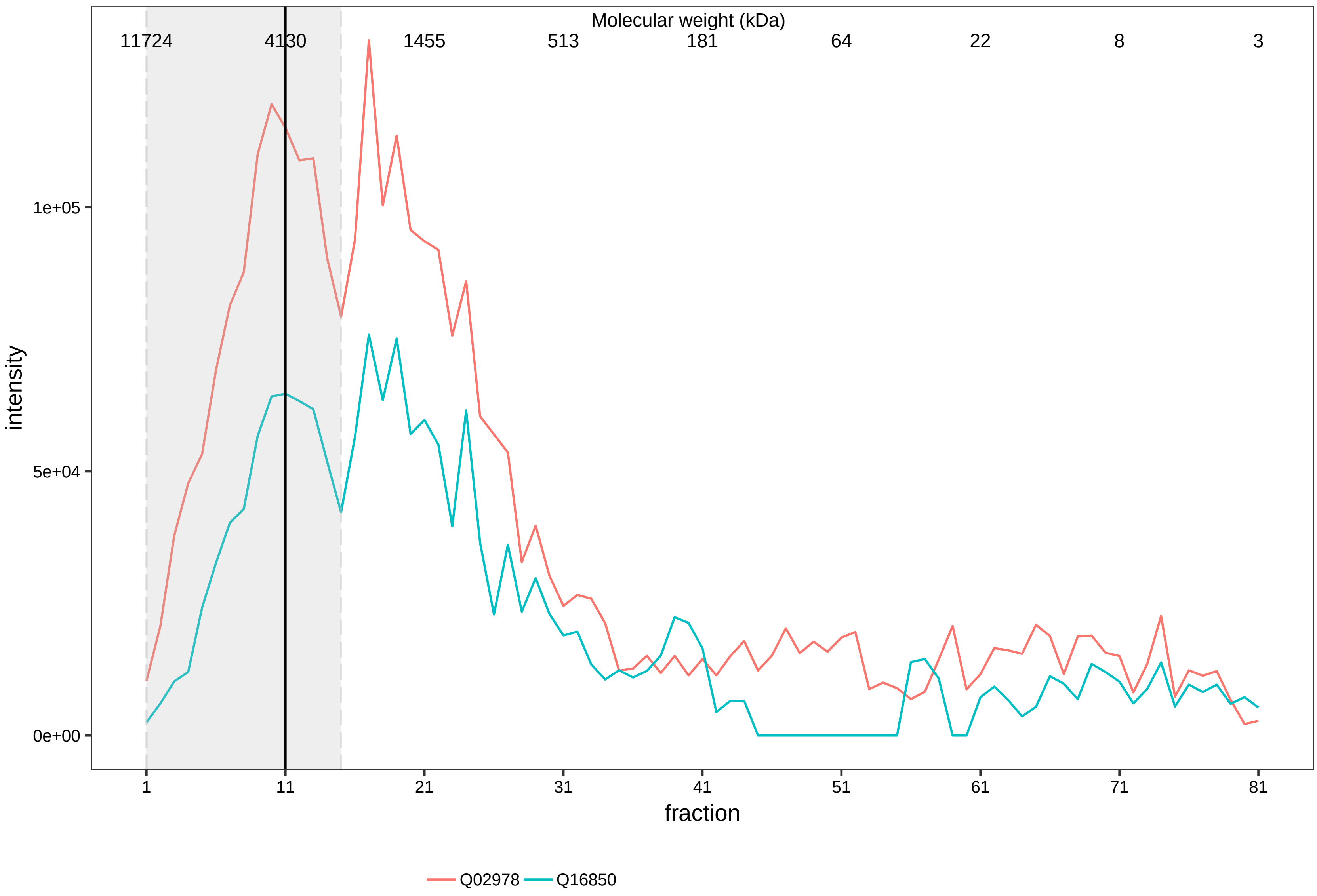

Feature ID 70

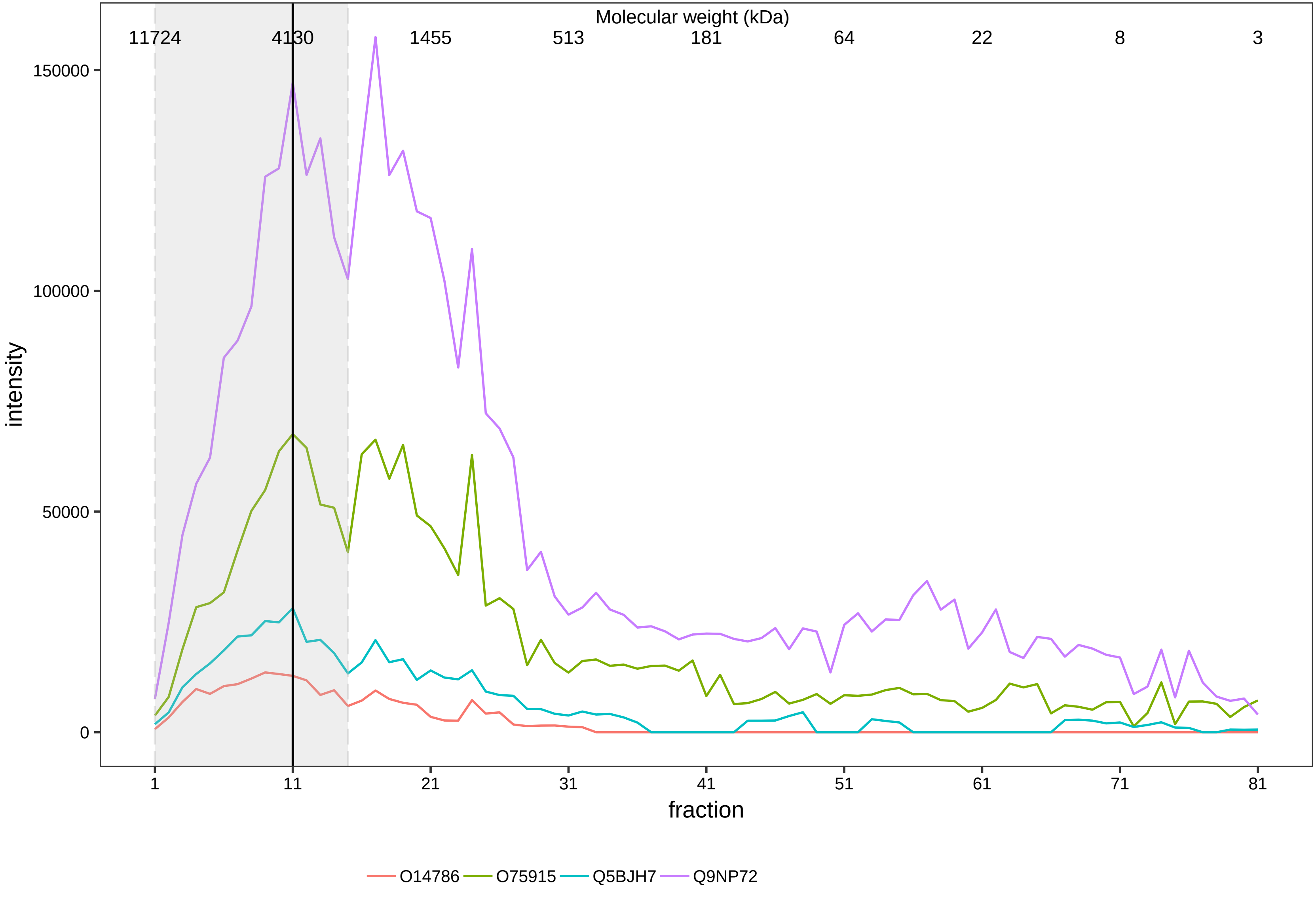

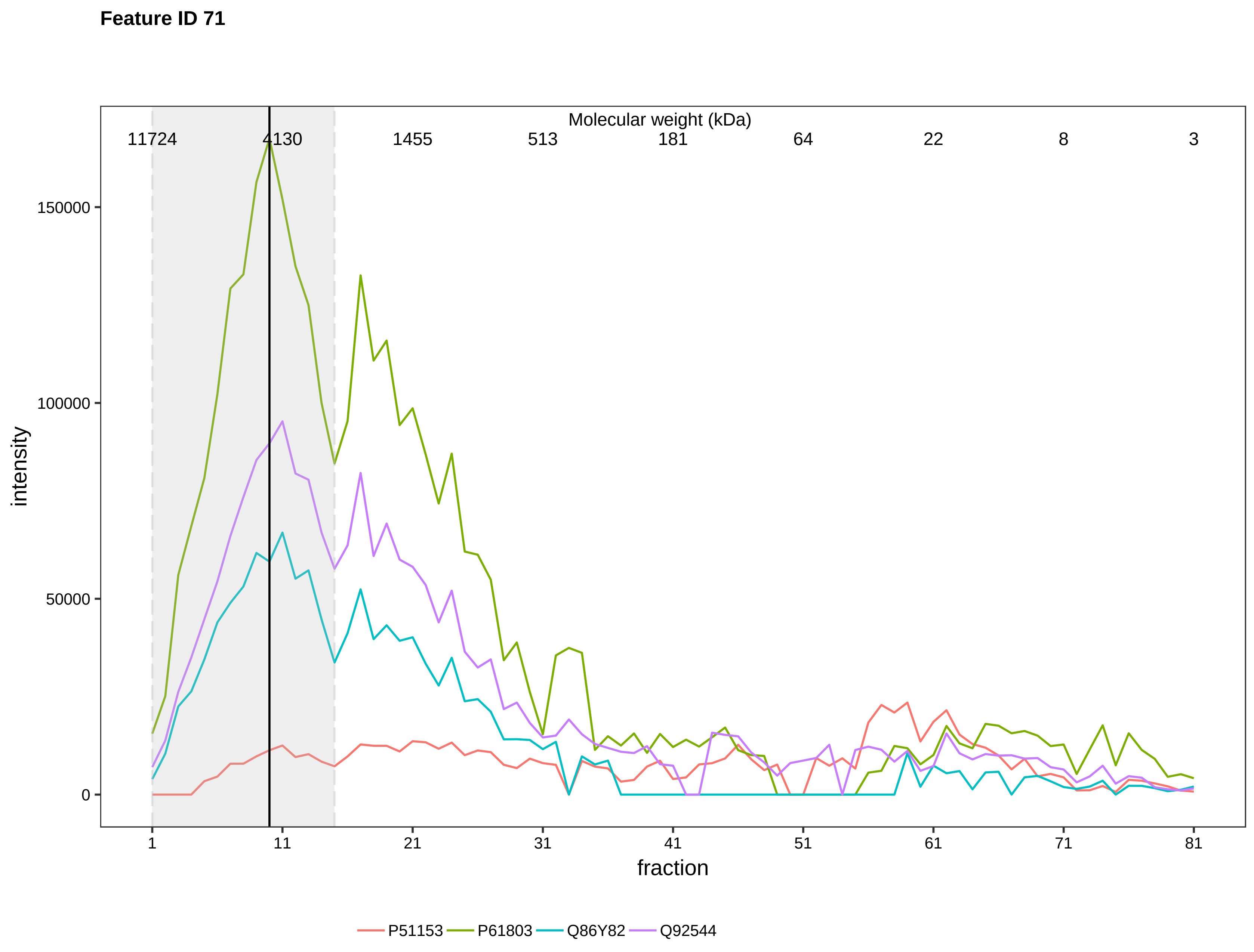
Feature ID 71
Molecular weight (kDa)
11724
4130
1455
513
181
64
22
8
3
150000
100000
50000
0
intensity
1
11
21
31
41
51
61
71
81
fraction
P51153
P61803
Q86Y82
Q92544

Feature ID 72

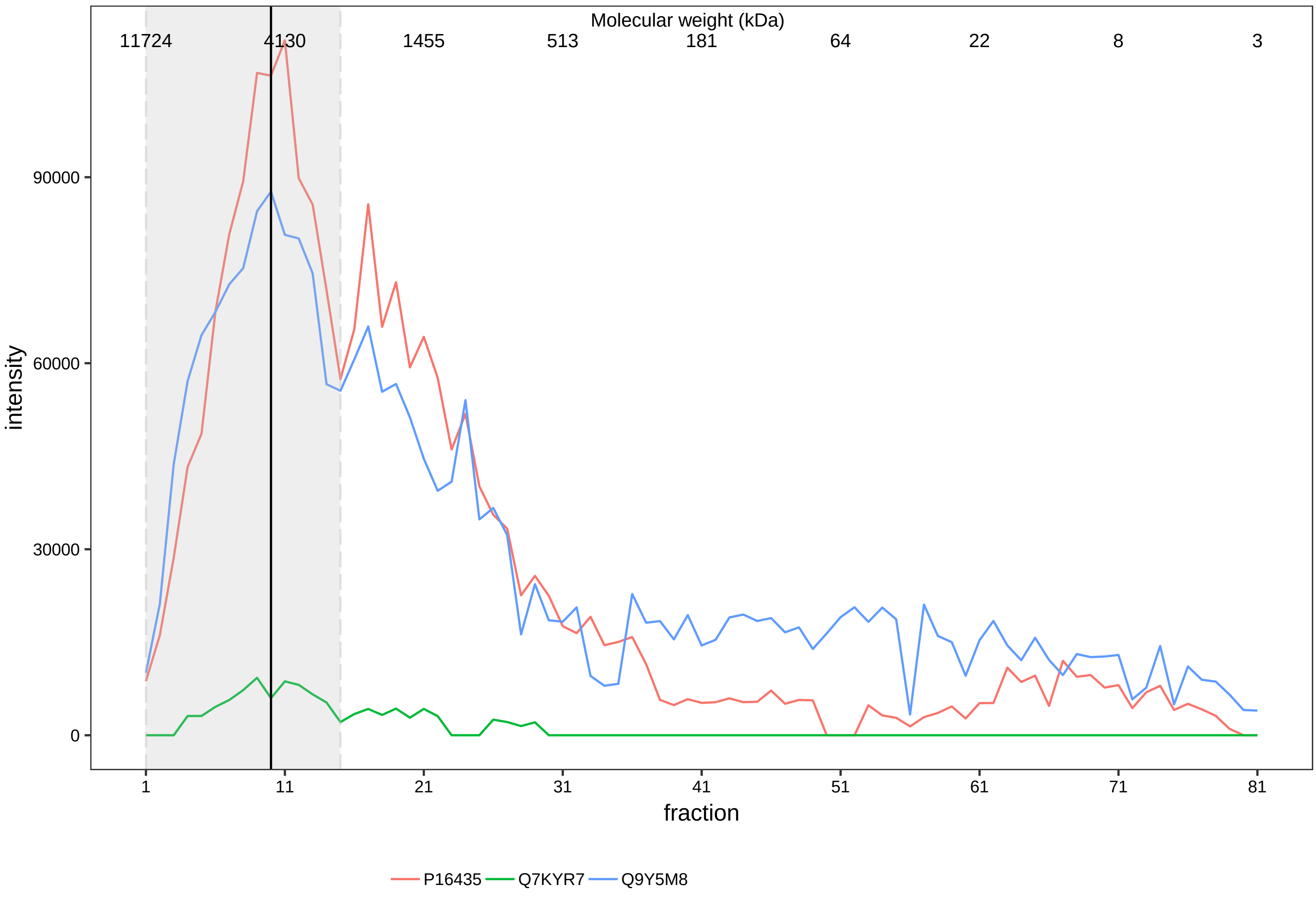

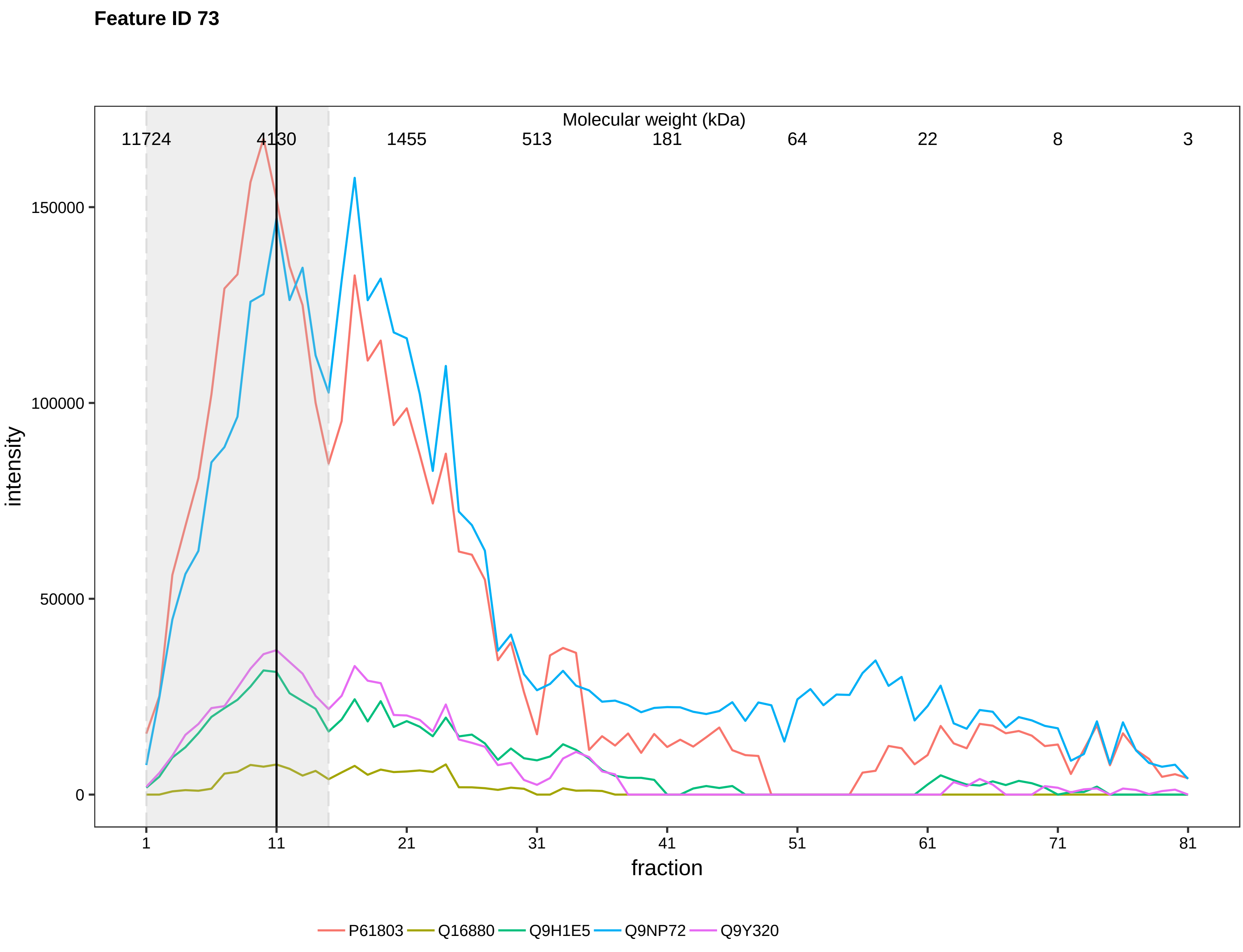
Feature ID 73
Molecular weight (kDa)
11724
4130
1455
513
181
64
22
8
3
150000
100000
50000
0
intensity
1
11
21
31
41
51
61
71
81
fraction
P61803
Q16880
Q9H1E5
Q9NP72
Q9Y320

Feature ID 74

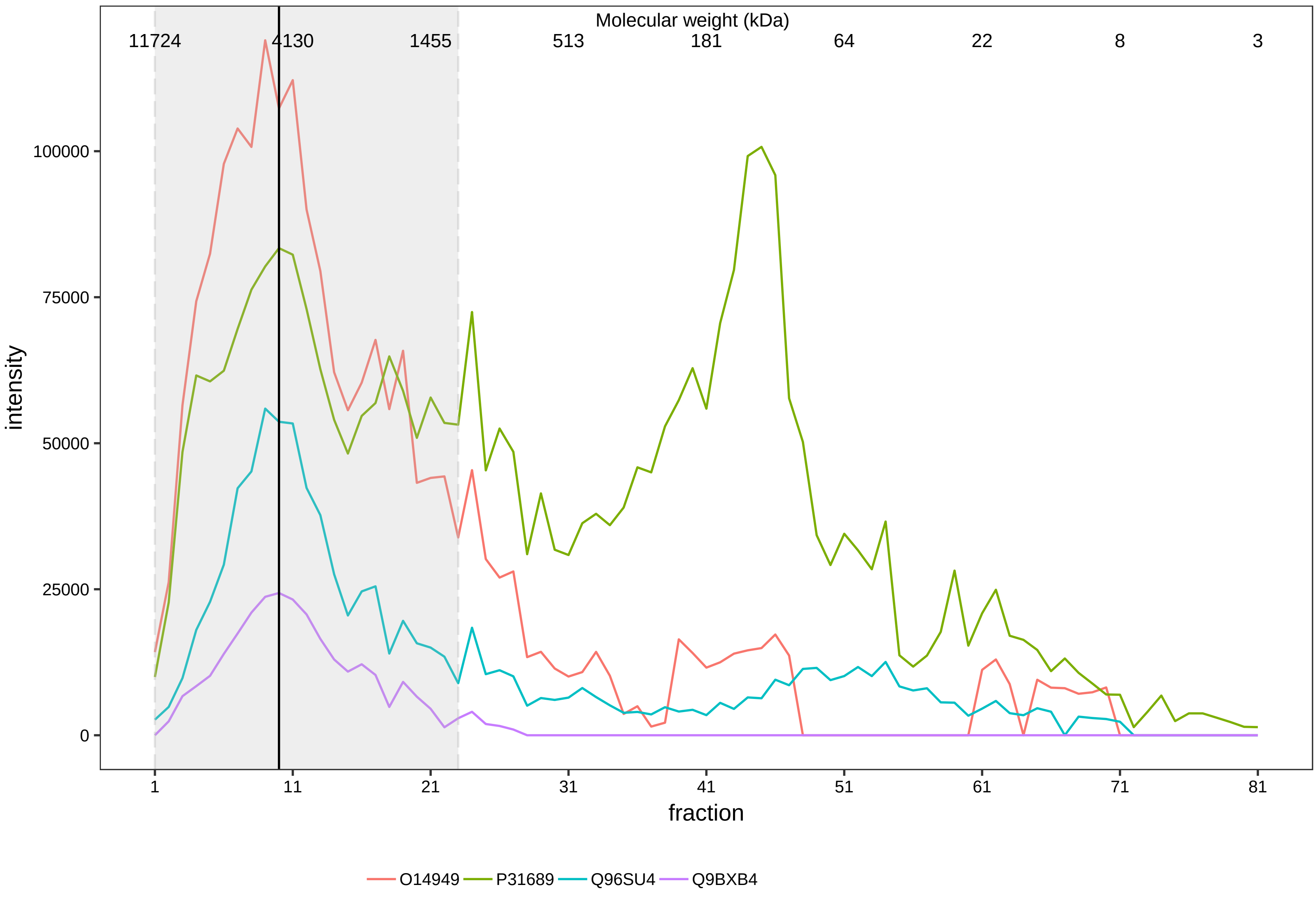

Feature ID 75

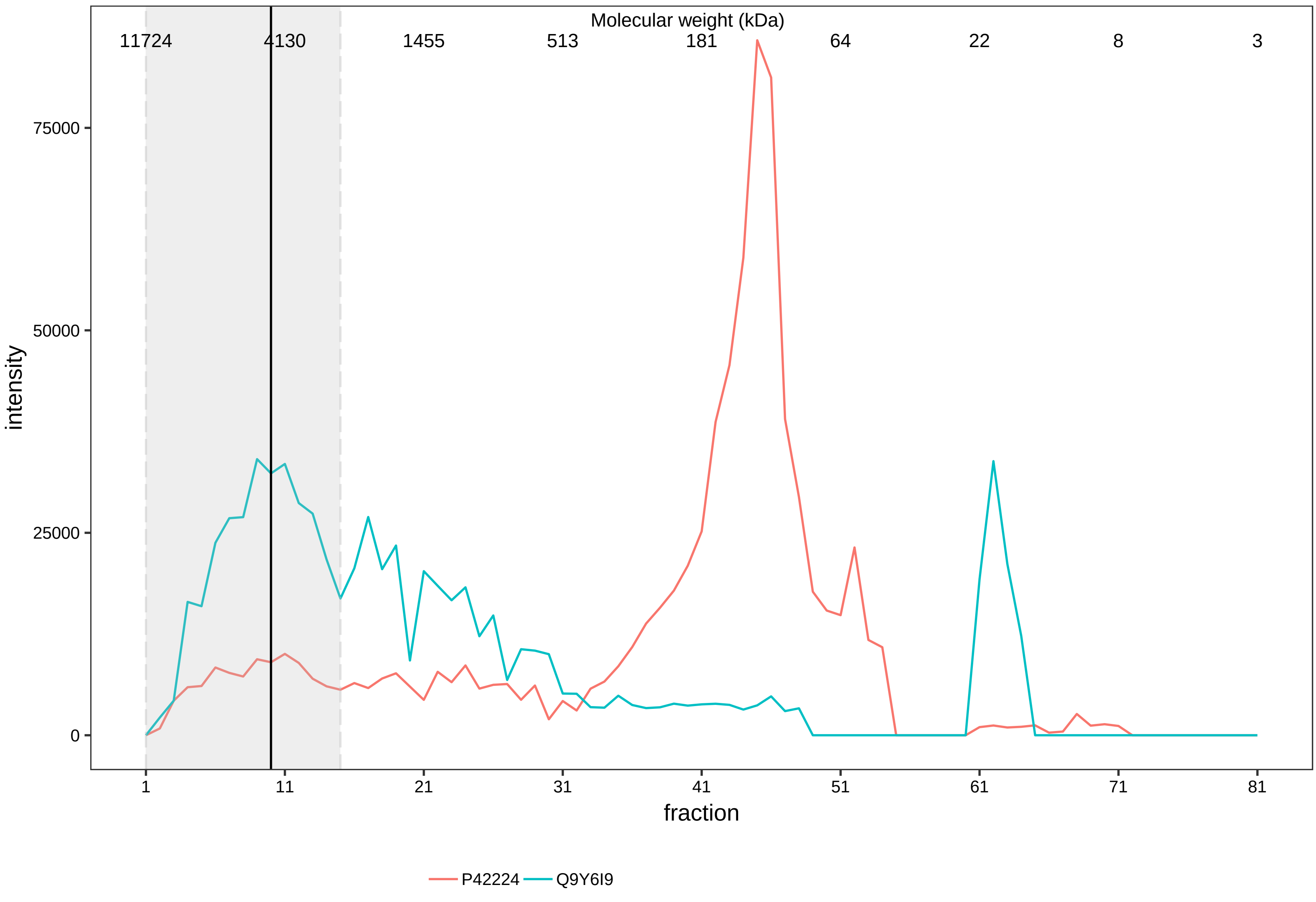

Feature ID 76

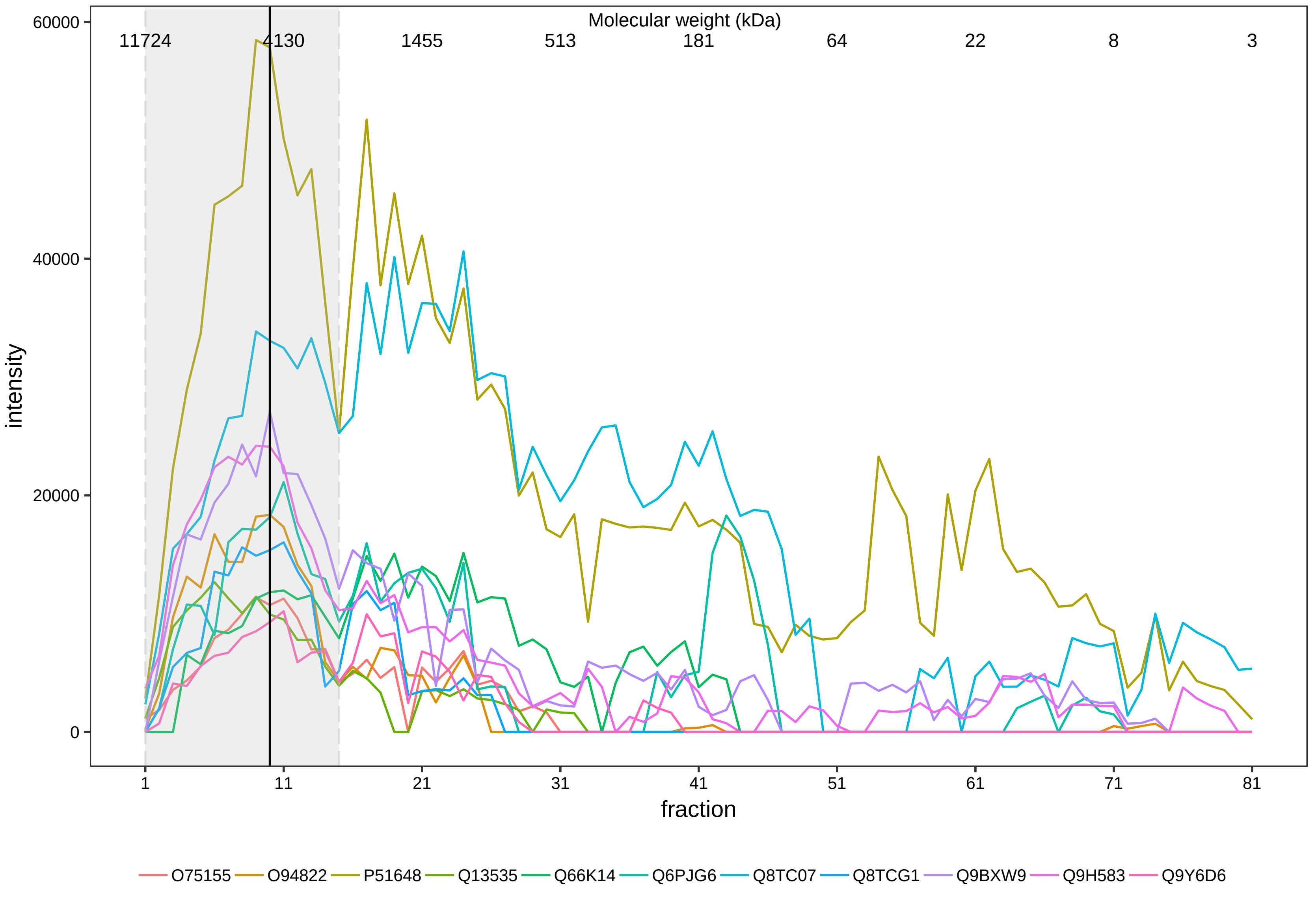

Feature ID 77

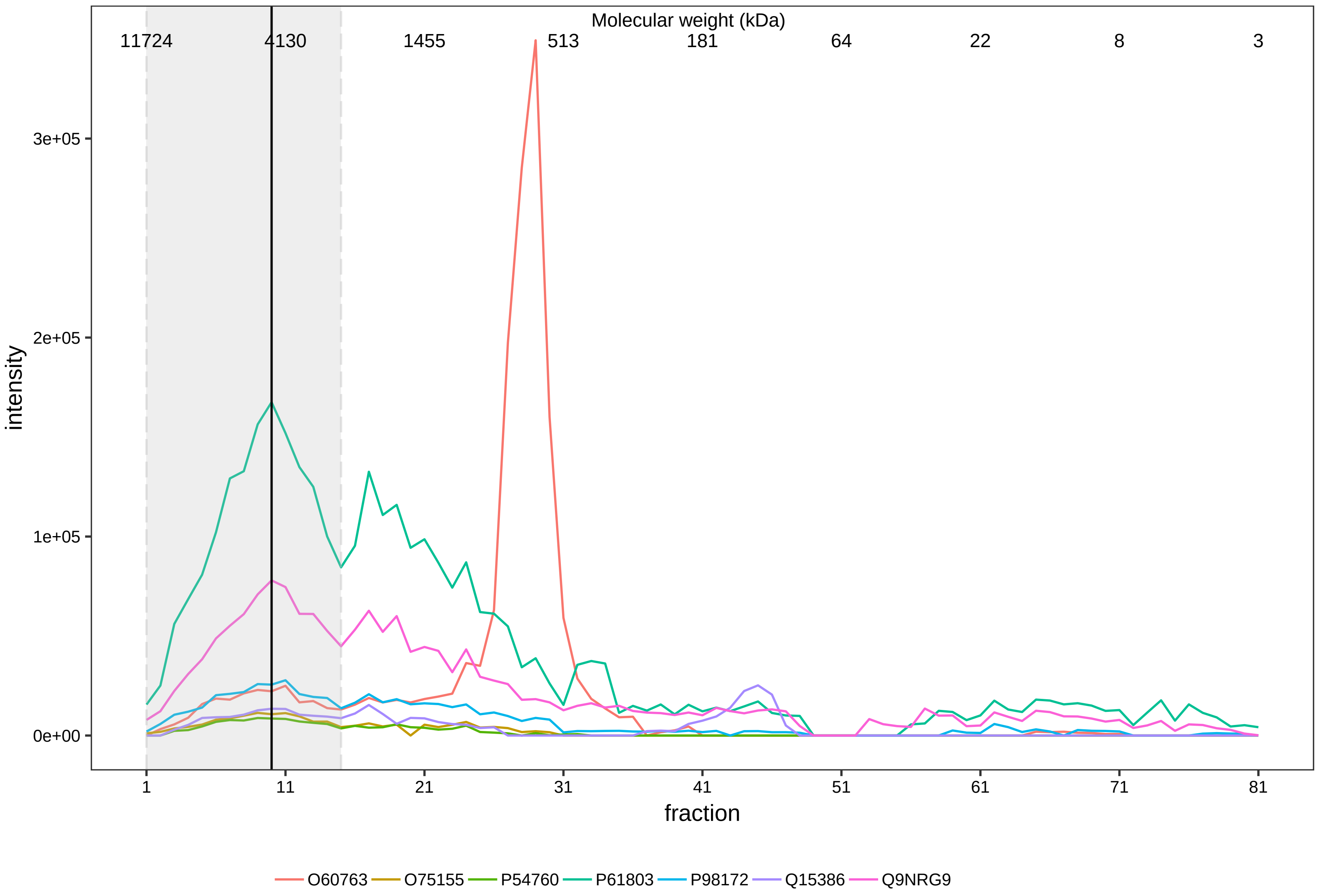

Feature ID 78

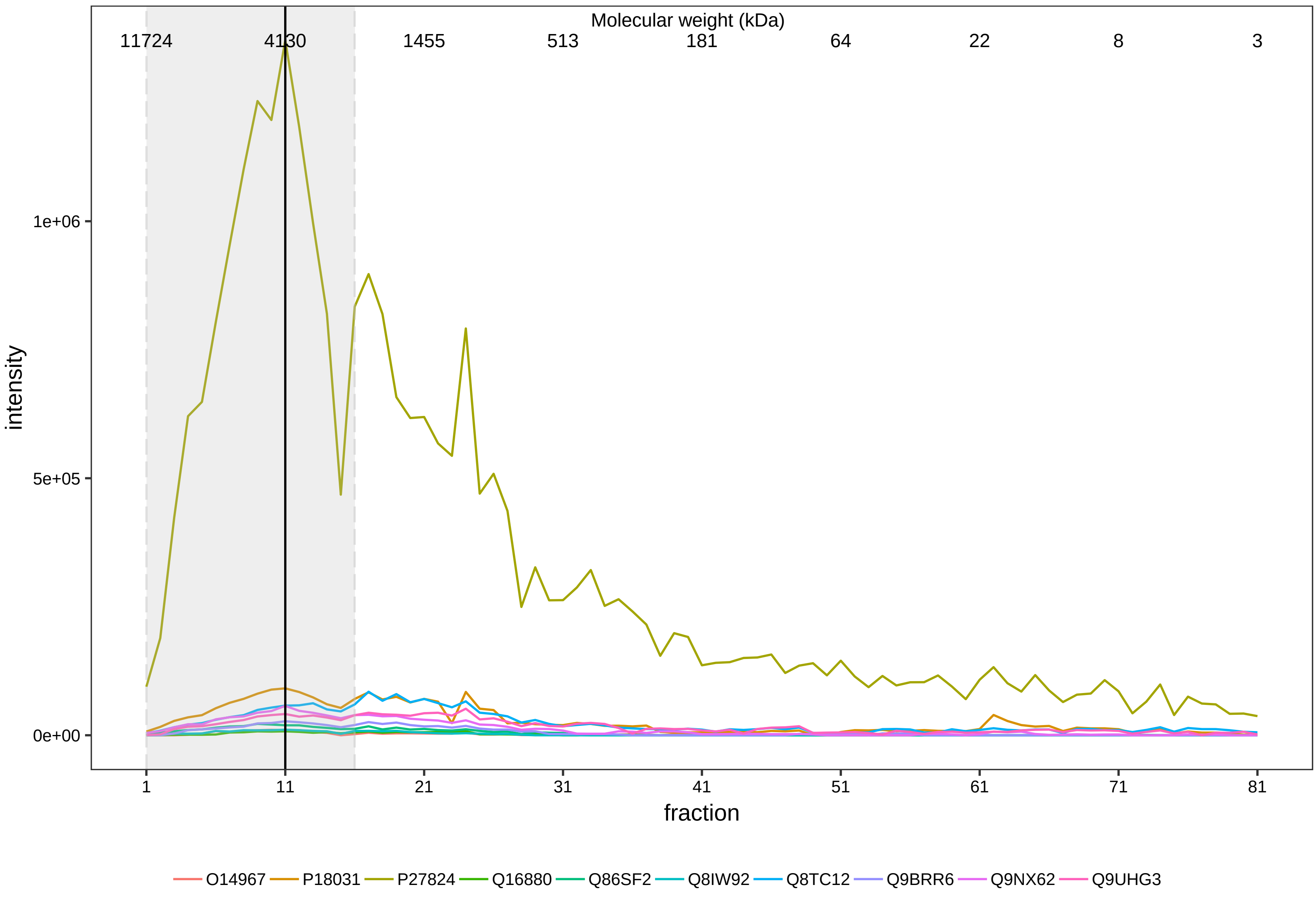

Feature ID 79

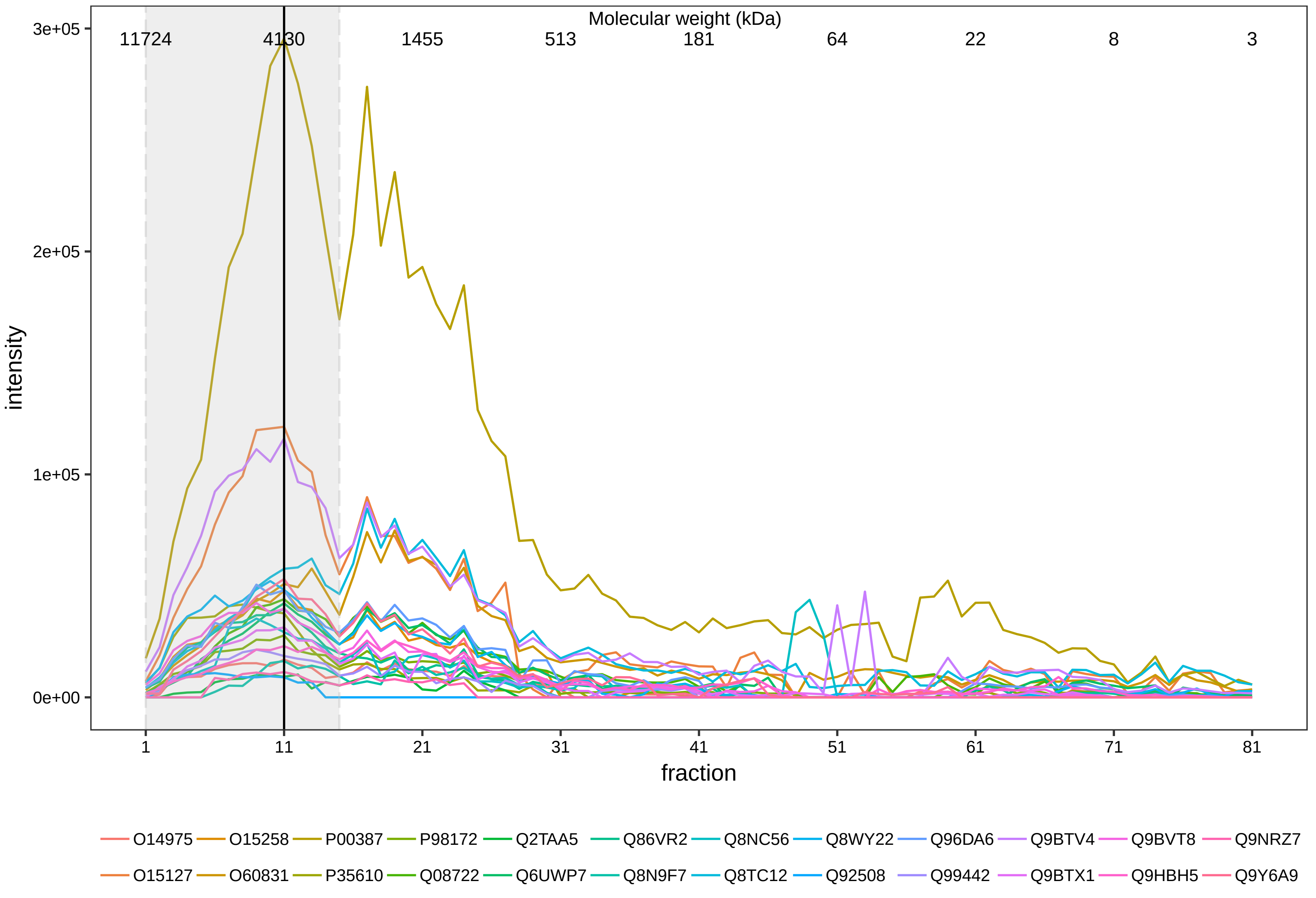

Feature ID 80

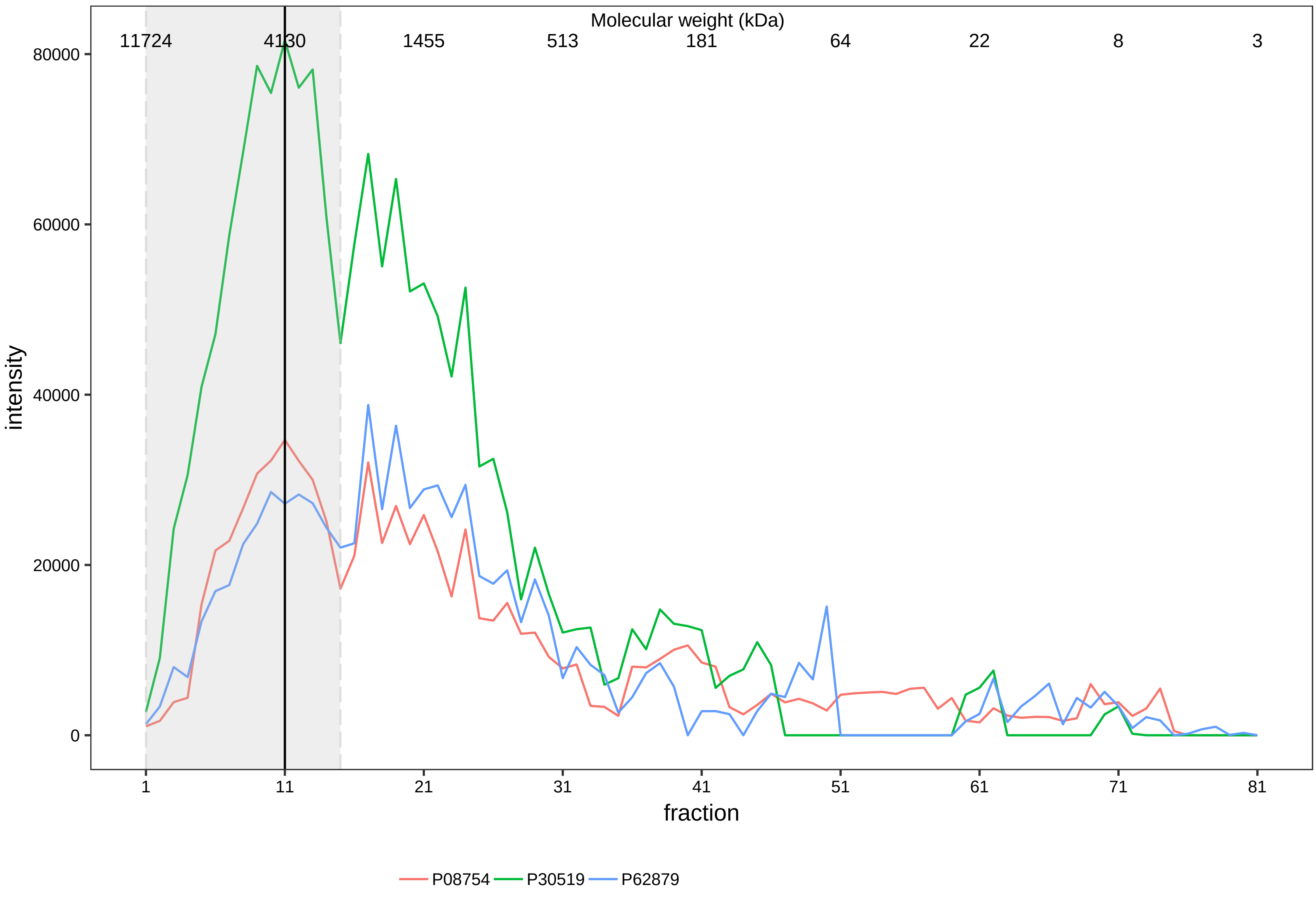

Feature ID 81

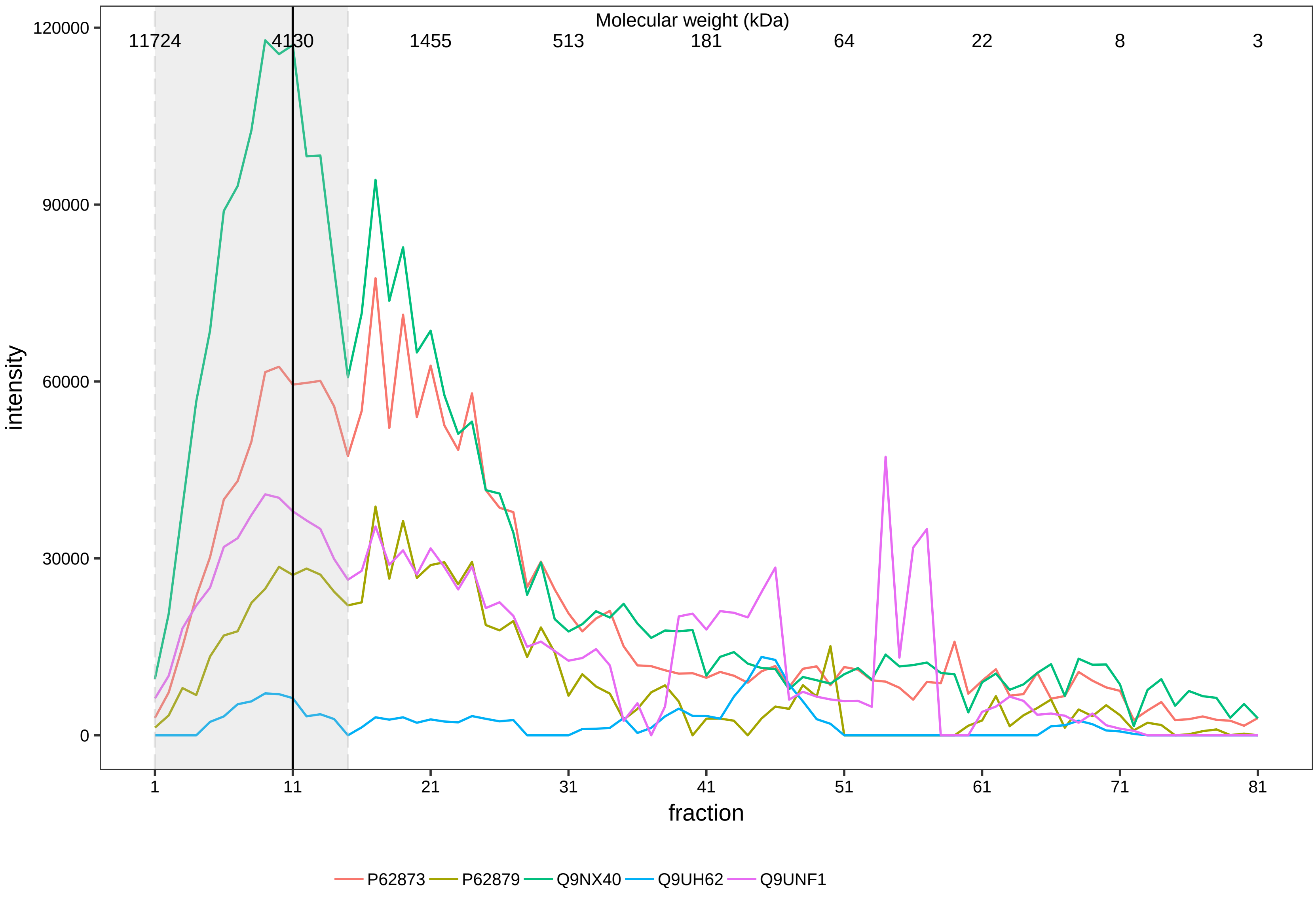

Feature ID 82

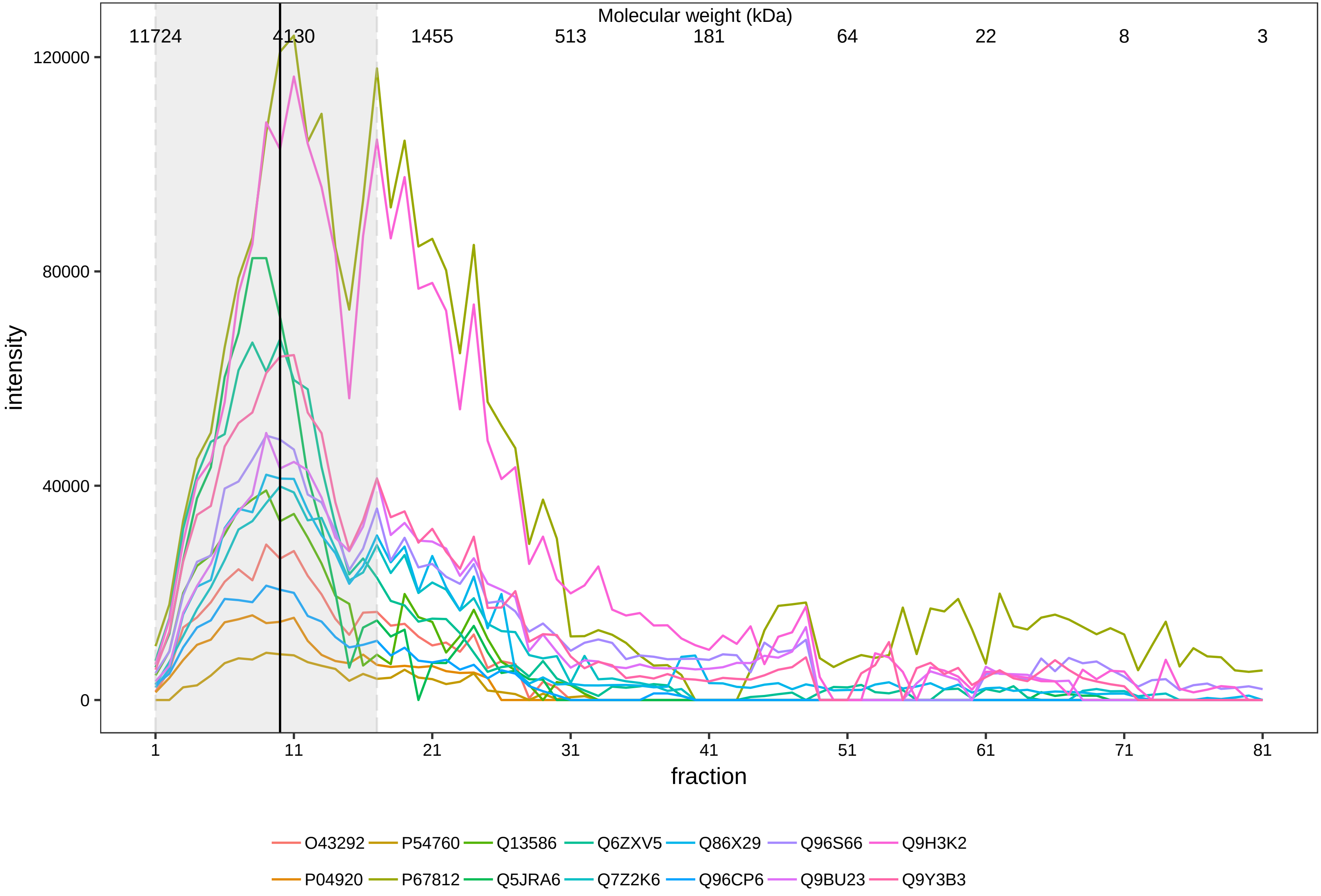

Feature ID 83

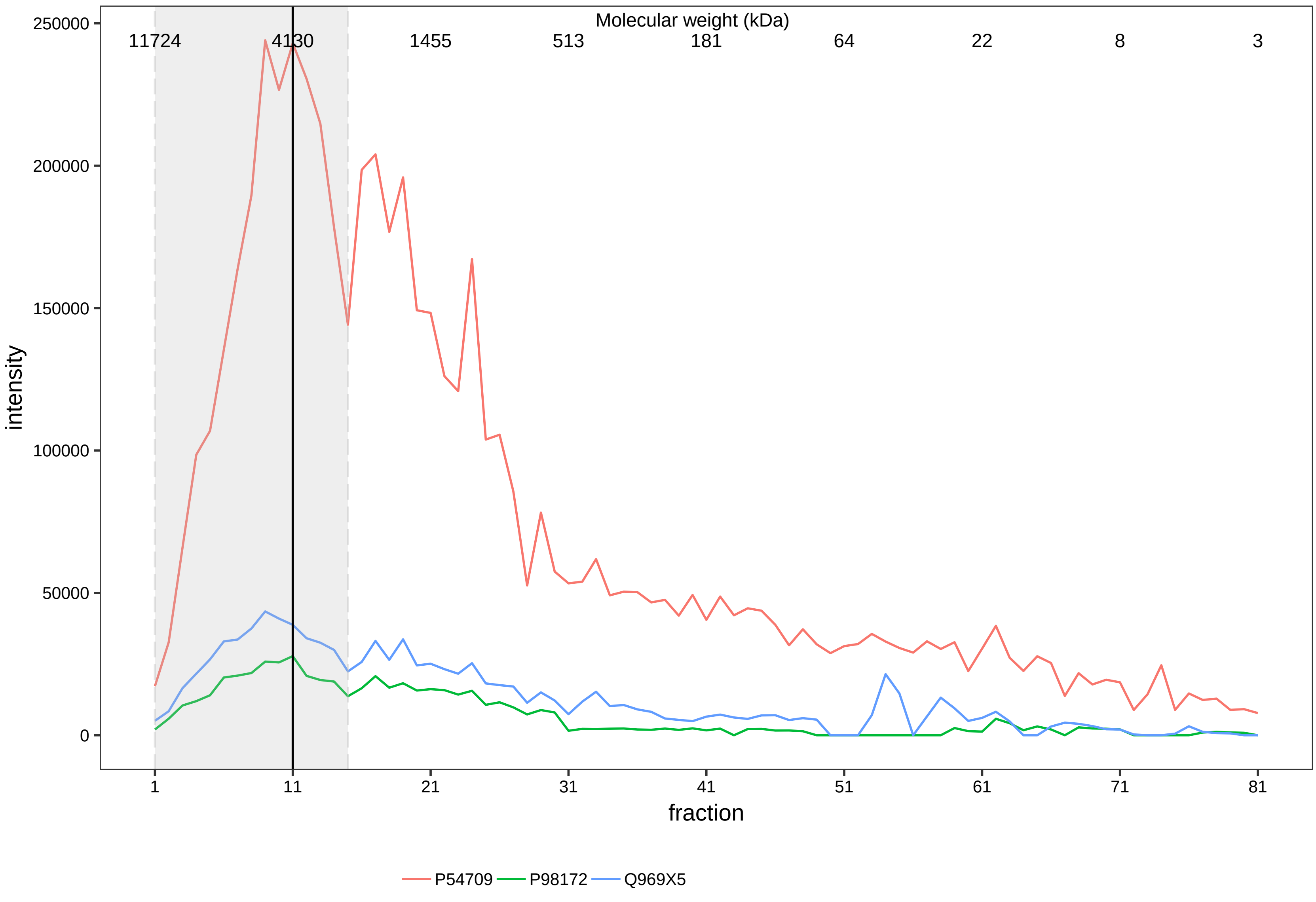

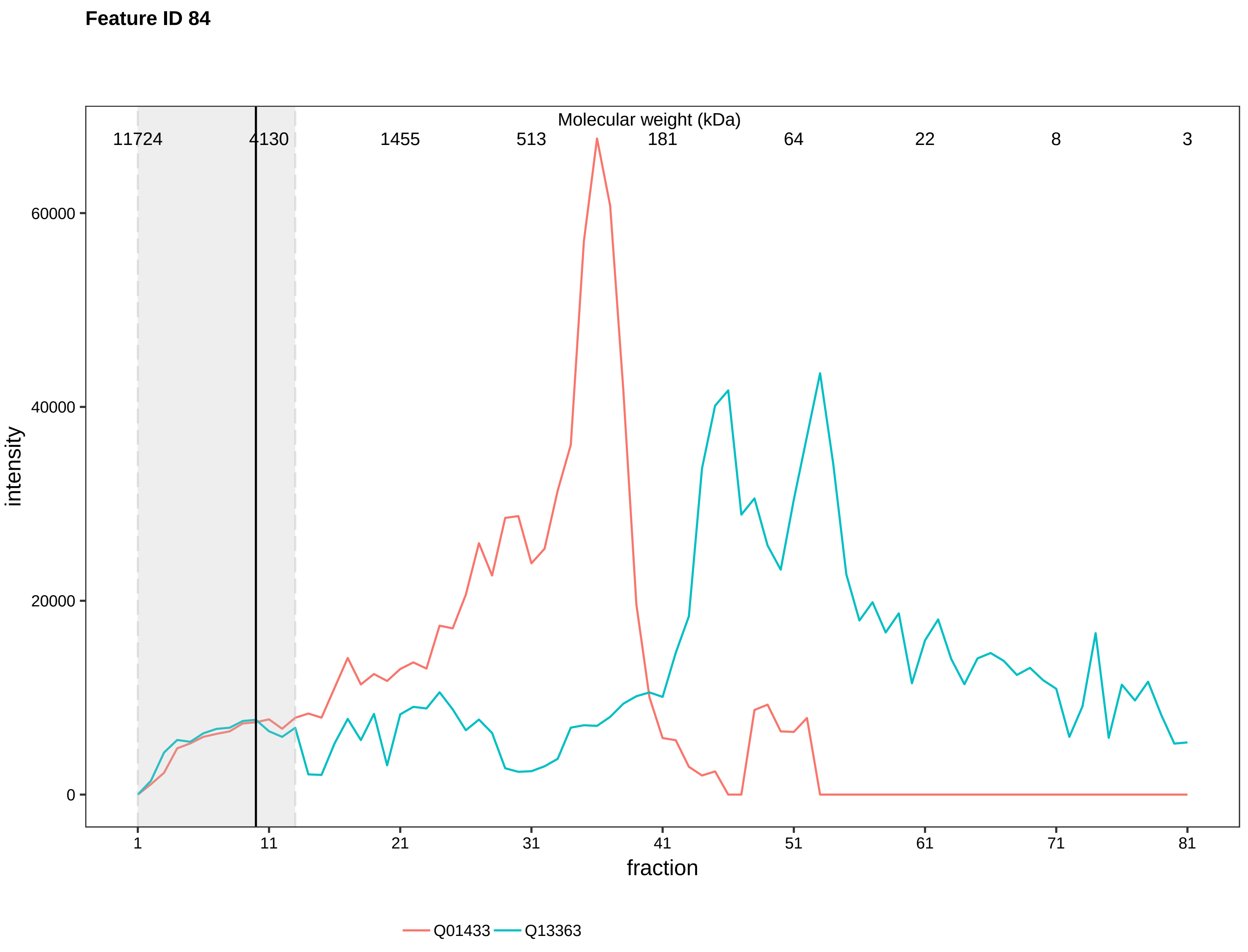
Feature ID 84
Molecular weight (kDa)
11724
4130
1455
513
181
64
22
8
3
60000
40000
20000
0
intensity
1
11
21
31
41
51
61
71
81
fraction
Q01433
Q13363

Feature ID 85

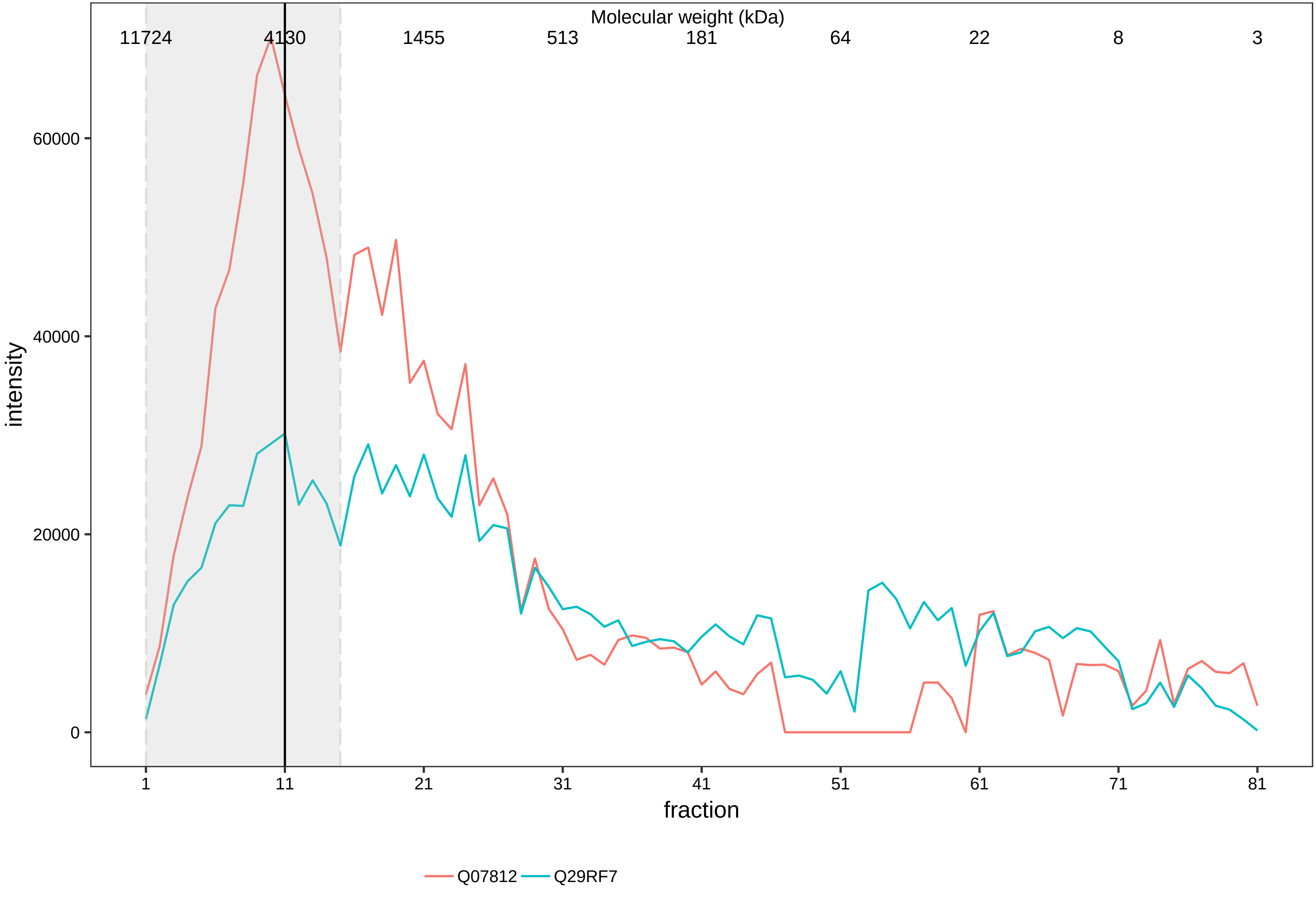

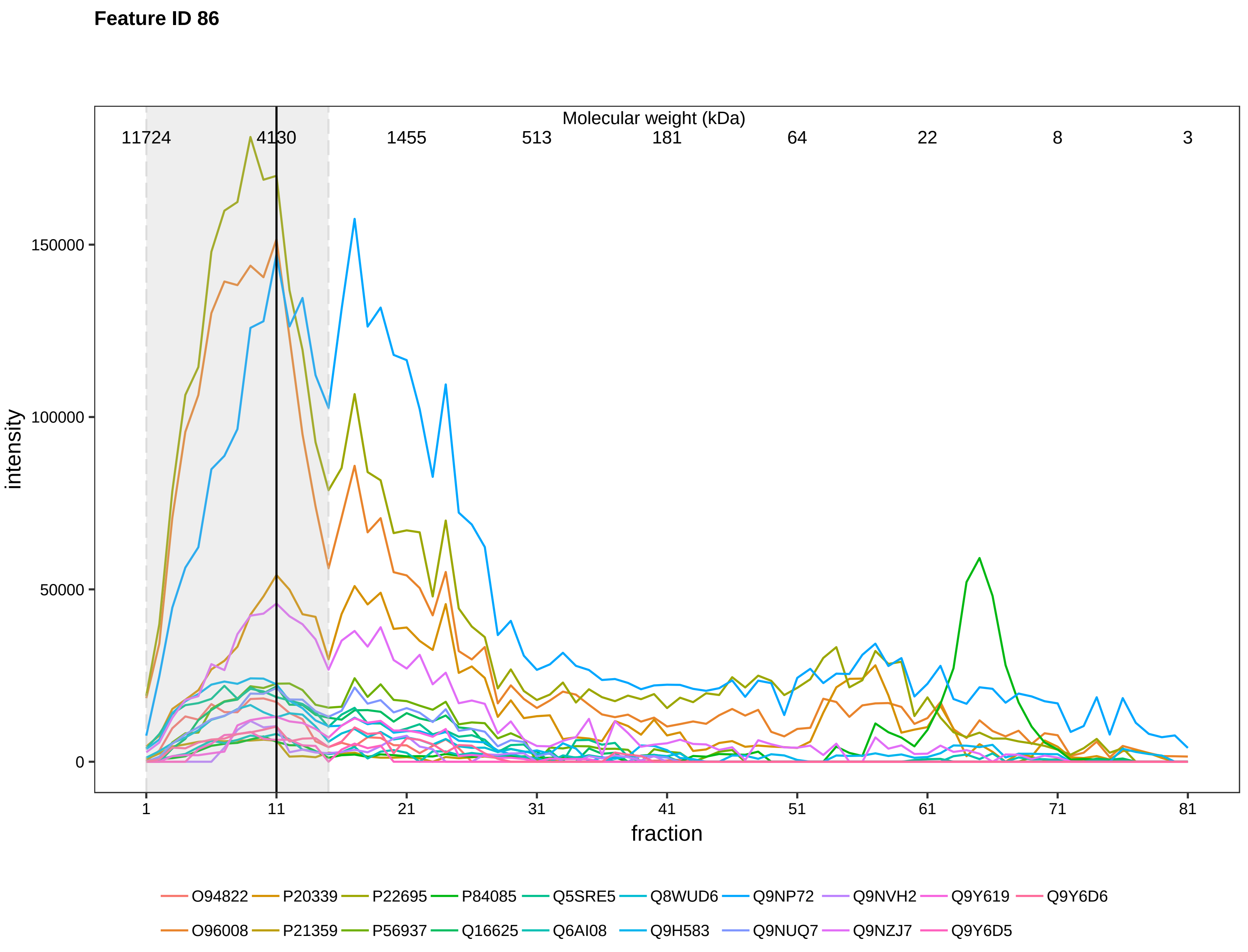
Feature ID 86
Molecular weight (kDa)
11724
4130
1455
513
181
64
22
8
3
intensity
150000
100000
50000
0
1
11
21
31
41
51
61
71
81
fraction
O94822
P20339
P22695
P84085
Q5SRE5
Q8WUD6
Q9NP72
Q9NVH2
Q9Y619
Q9Y6D6
O96008
P21359
P56937
Q16625
Q6AI08
Q9H583
Q9NUQ7
Q9NZJ7
Q9Y6D5

Feature ID 87

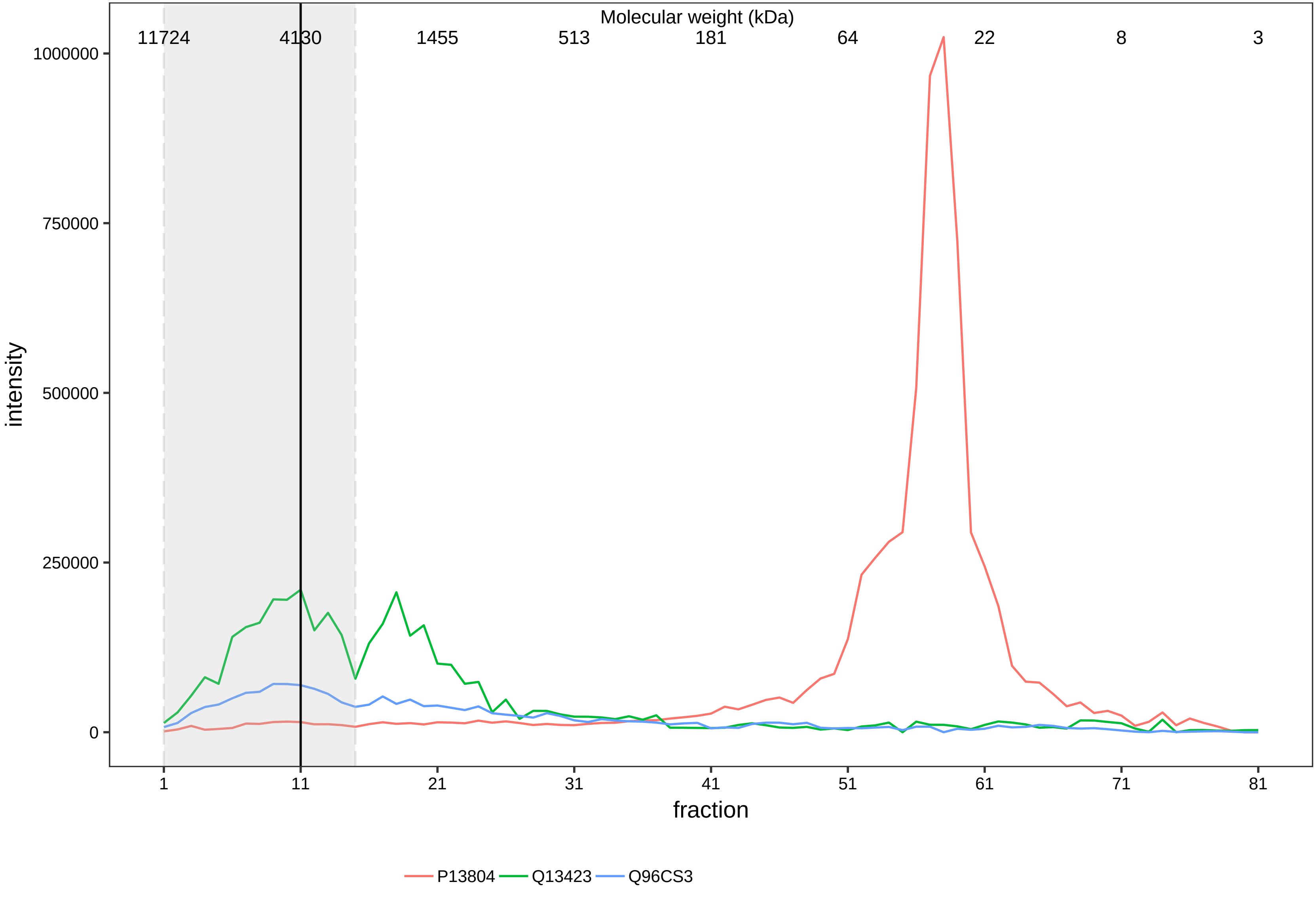

Feature ID 88

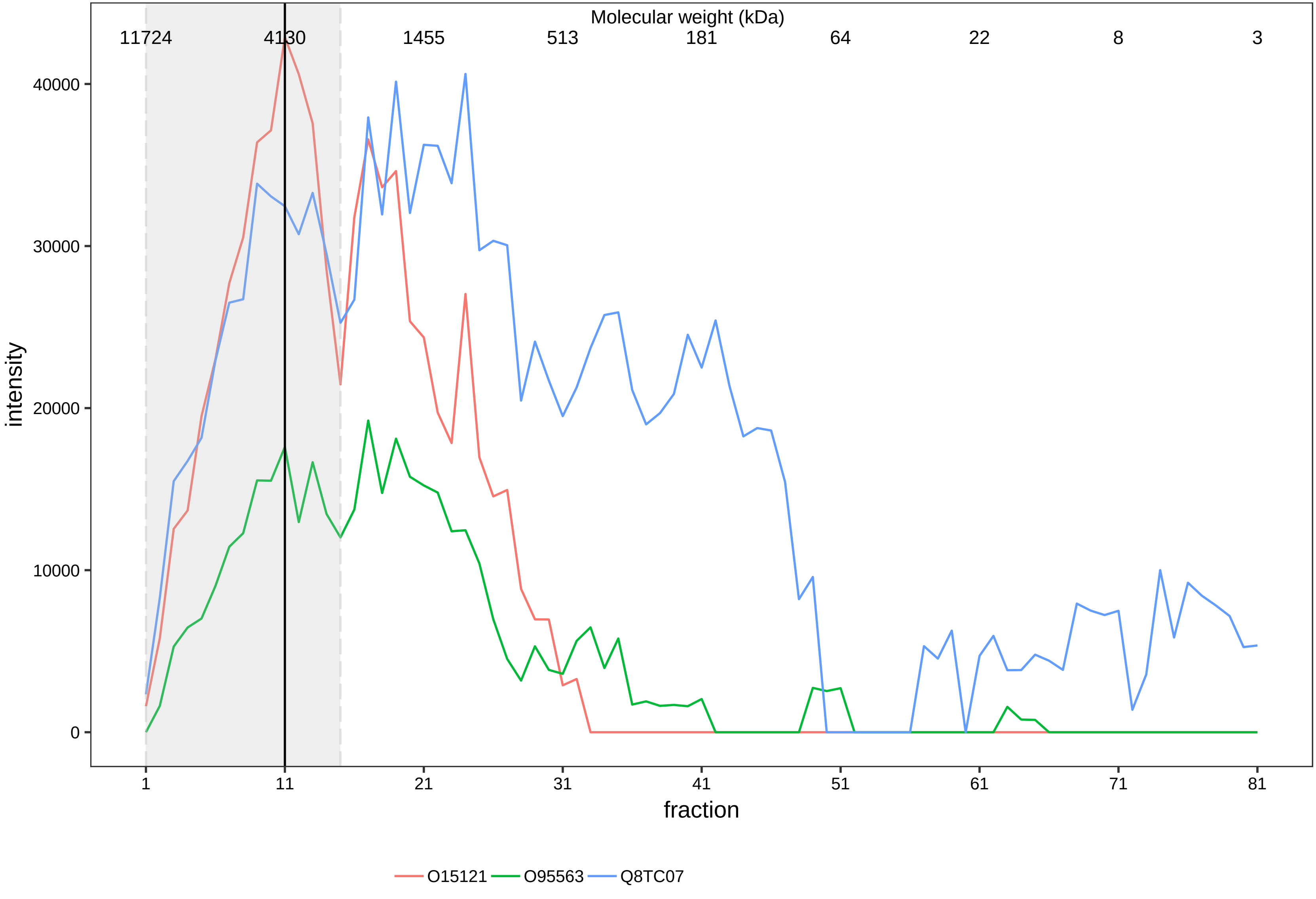

Feature ID 89

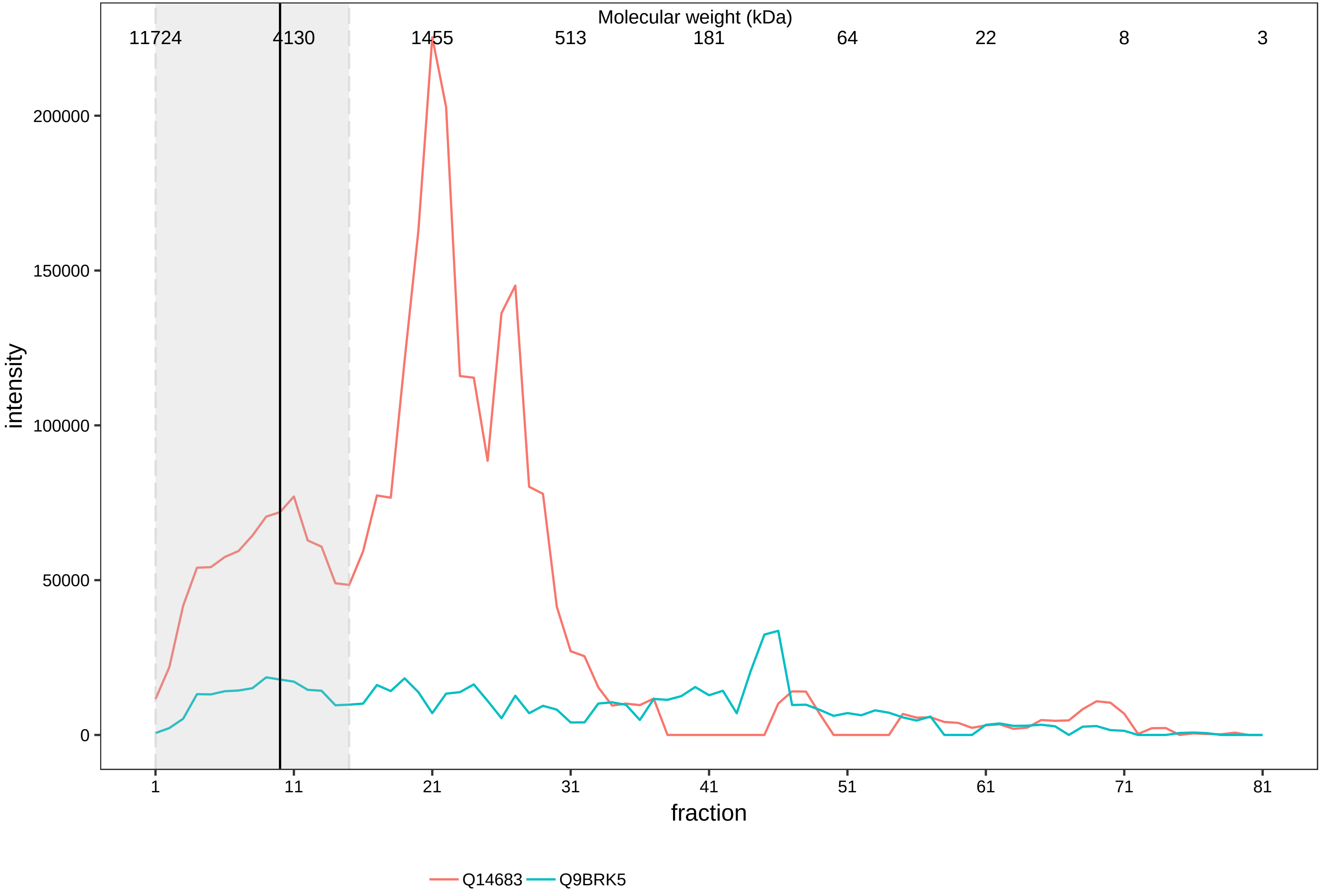

Feature ID 90

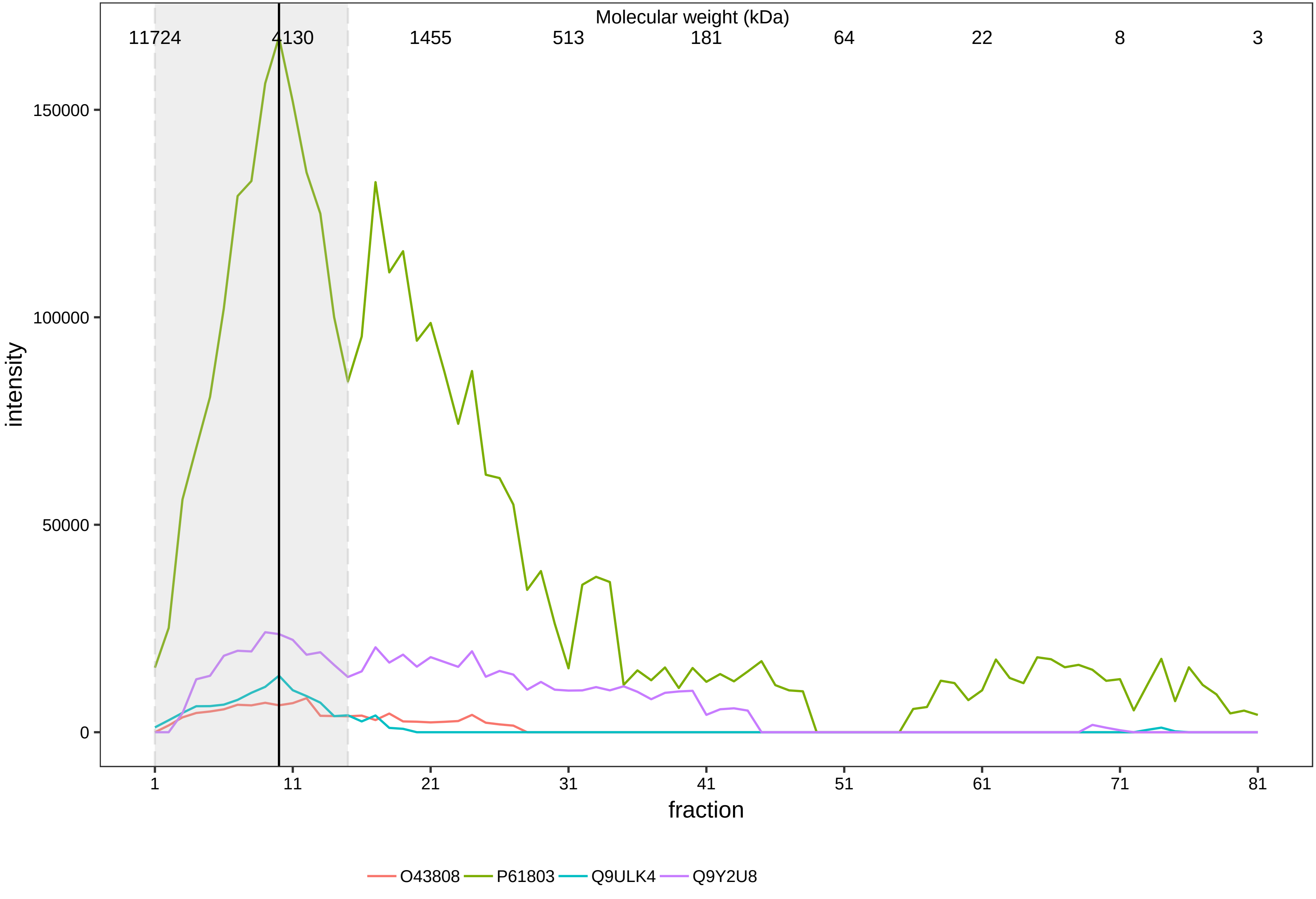

Feature ID 91

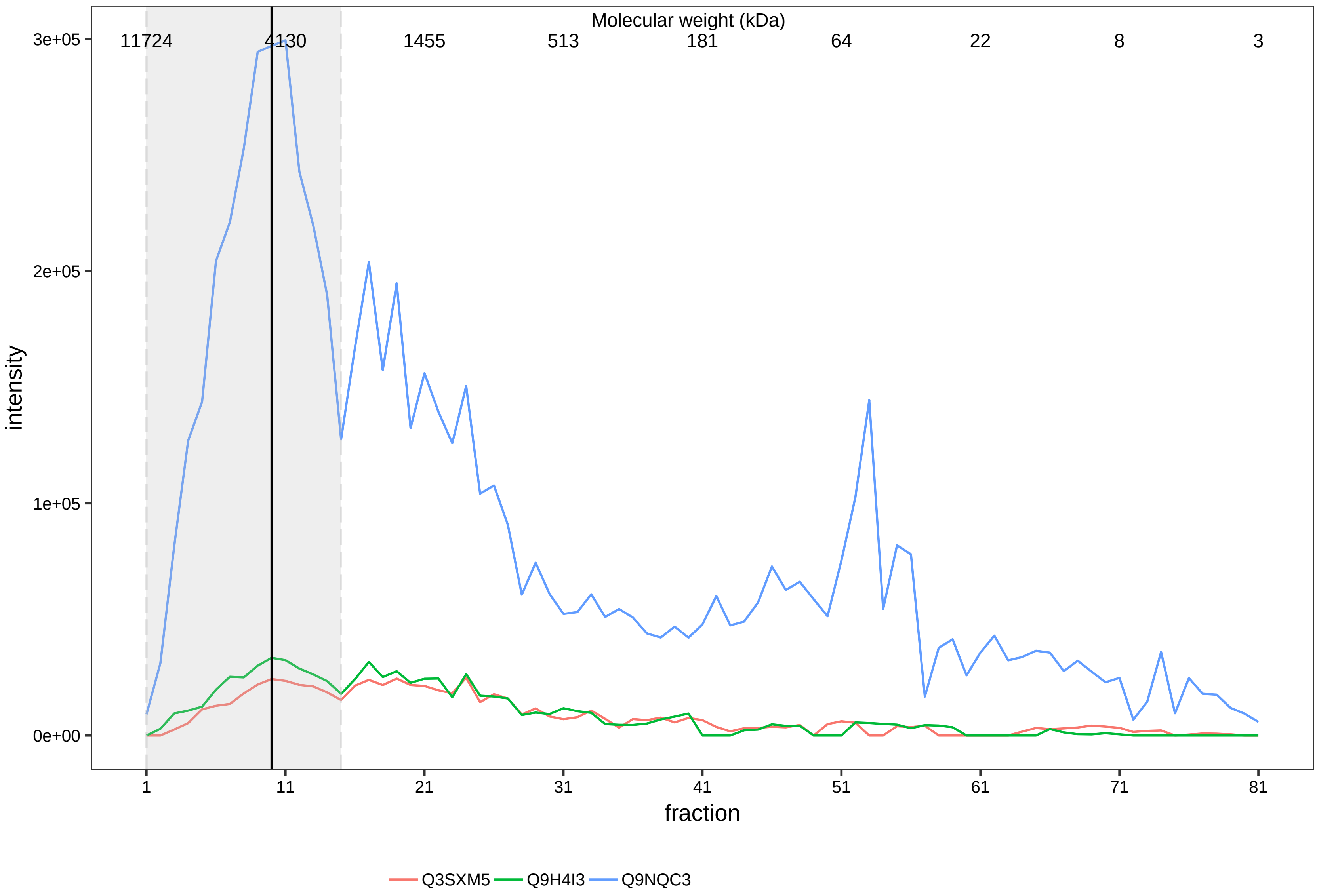

Feature ID 92

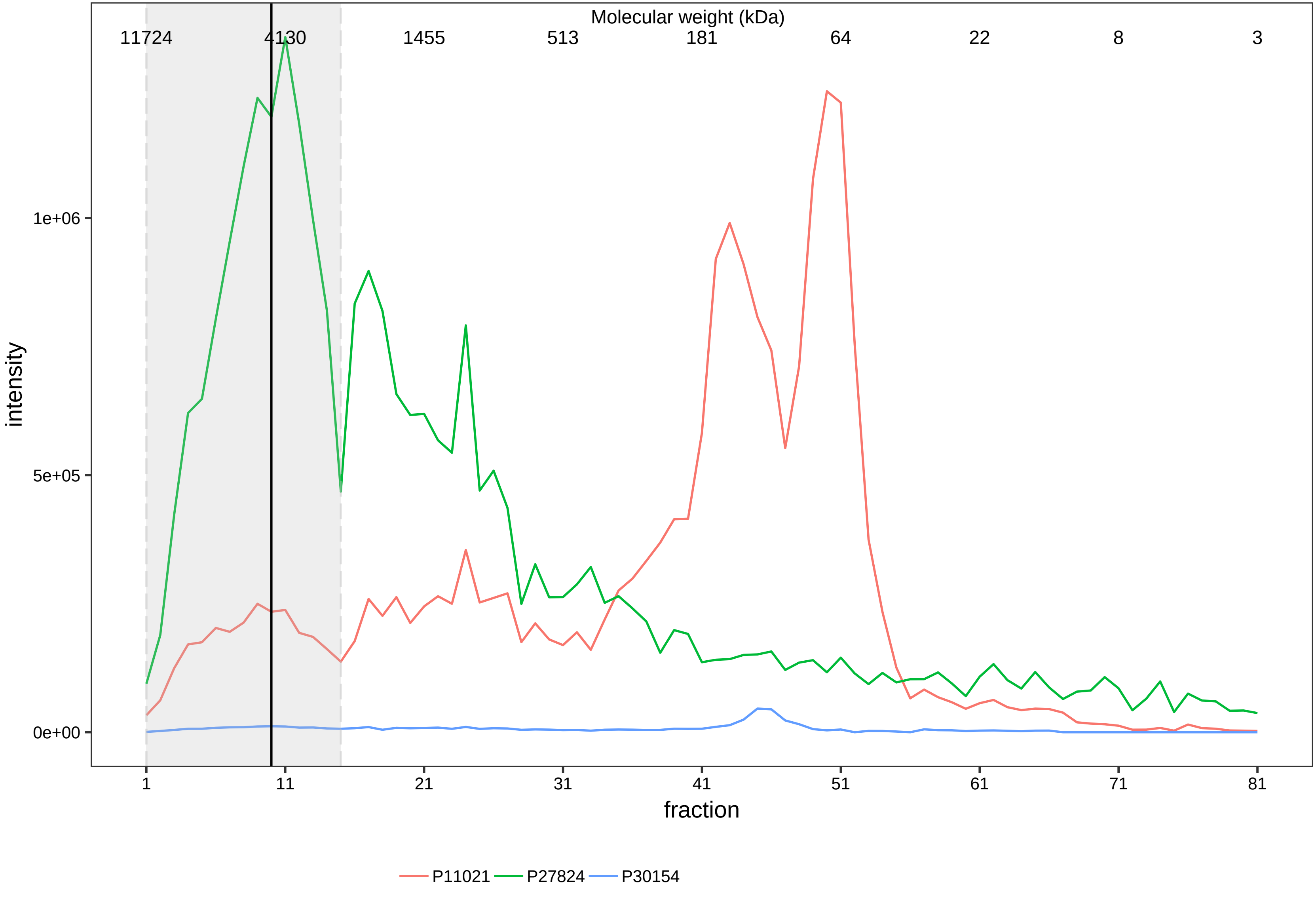

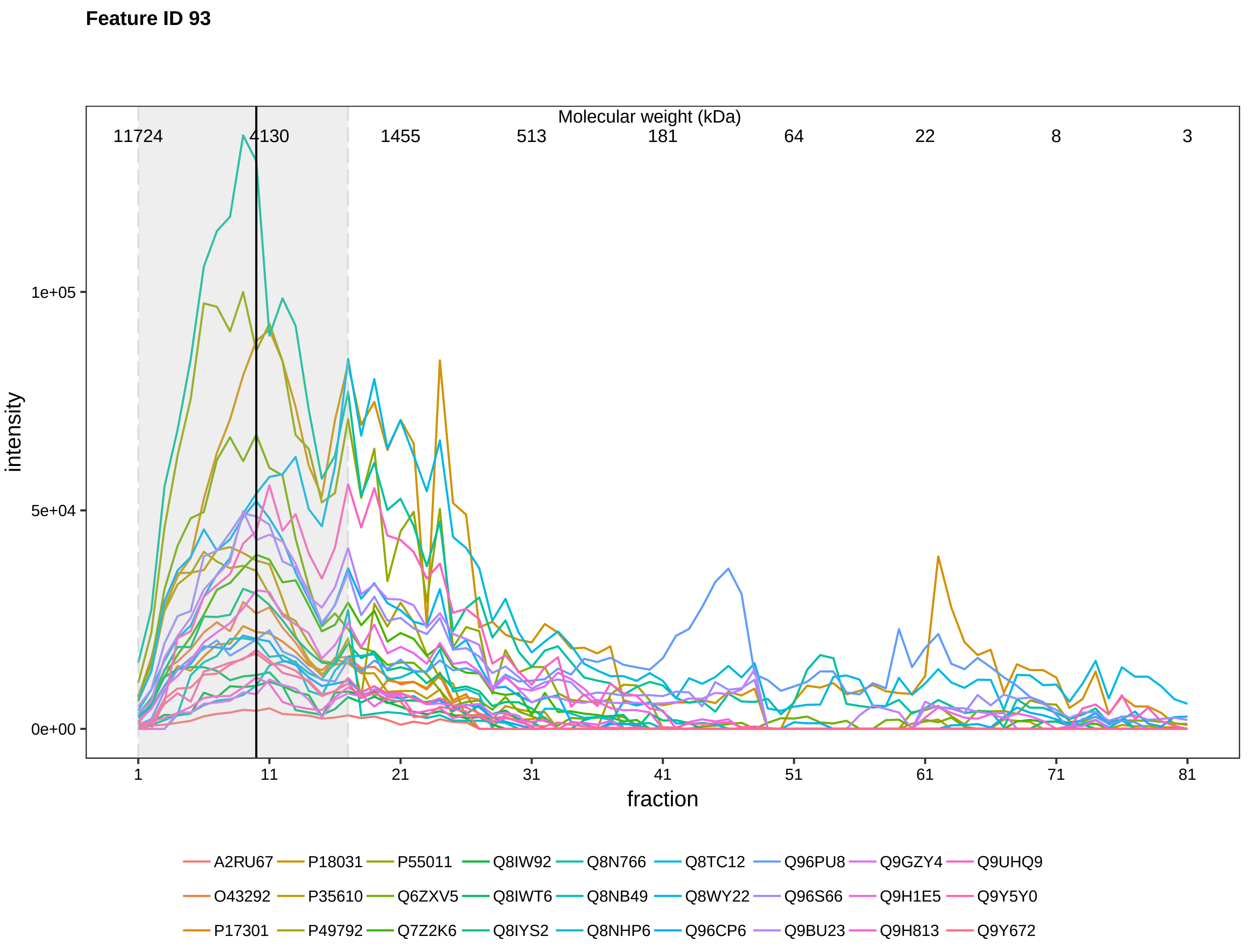

Feature ID 93
Molecular weight (kDa)
11724
4130
1455
513
181
64
22
8
3
intensity
1e+05
5e+04
0e+00
1
11
21
31
41
51
61
71
81
fraction
A2RU67
P18031
P55011
Q8IW92
Q8N766
Q8TC12
Q96PU8
Q9GZY4
Q9UHQ9
O43292
P35610
Q6ZXV5
Q8IWT6
Q8NB49
Q8WY22
Q96S66
Q9H1E5
Q9Y5Y0
P17301
P49792
Q7Z2K6
Q8IYS2
Q8NHP6
Q96CP6
Q9BU23
Q9H813
Q9Y672

Feature ID 94

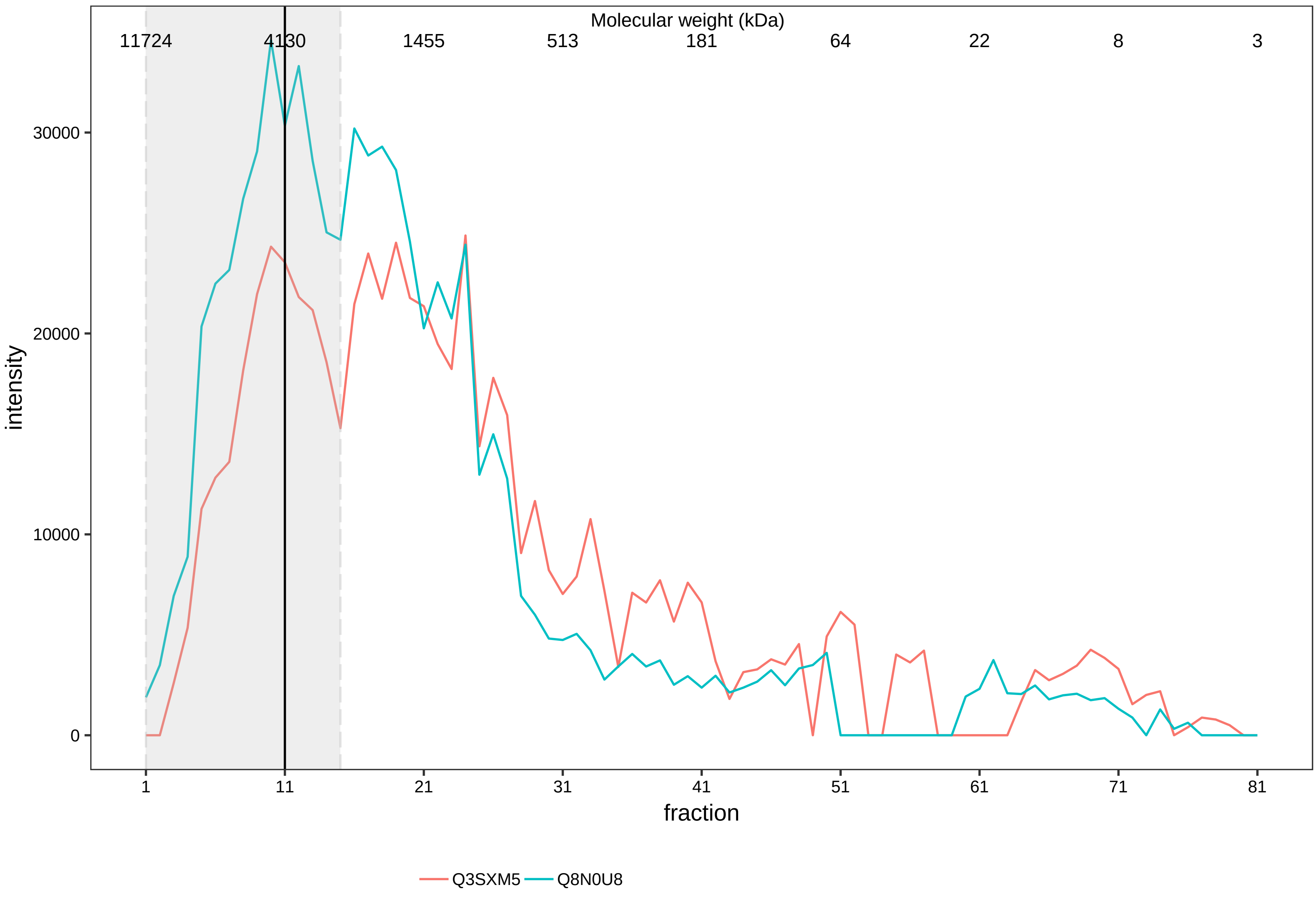

Feature ID 95

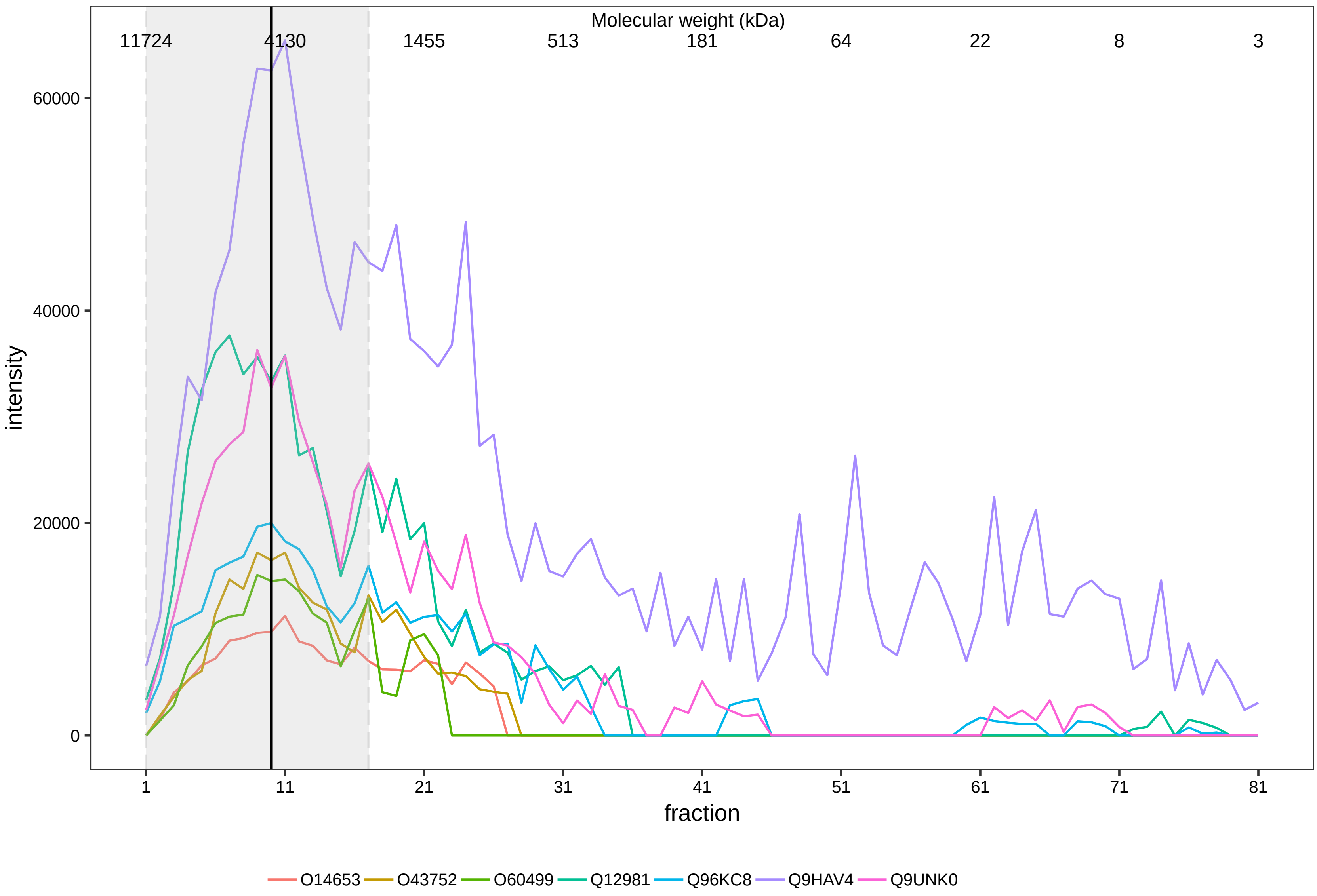

Feature ID 96

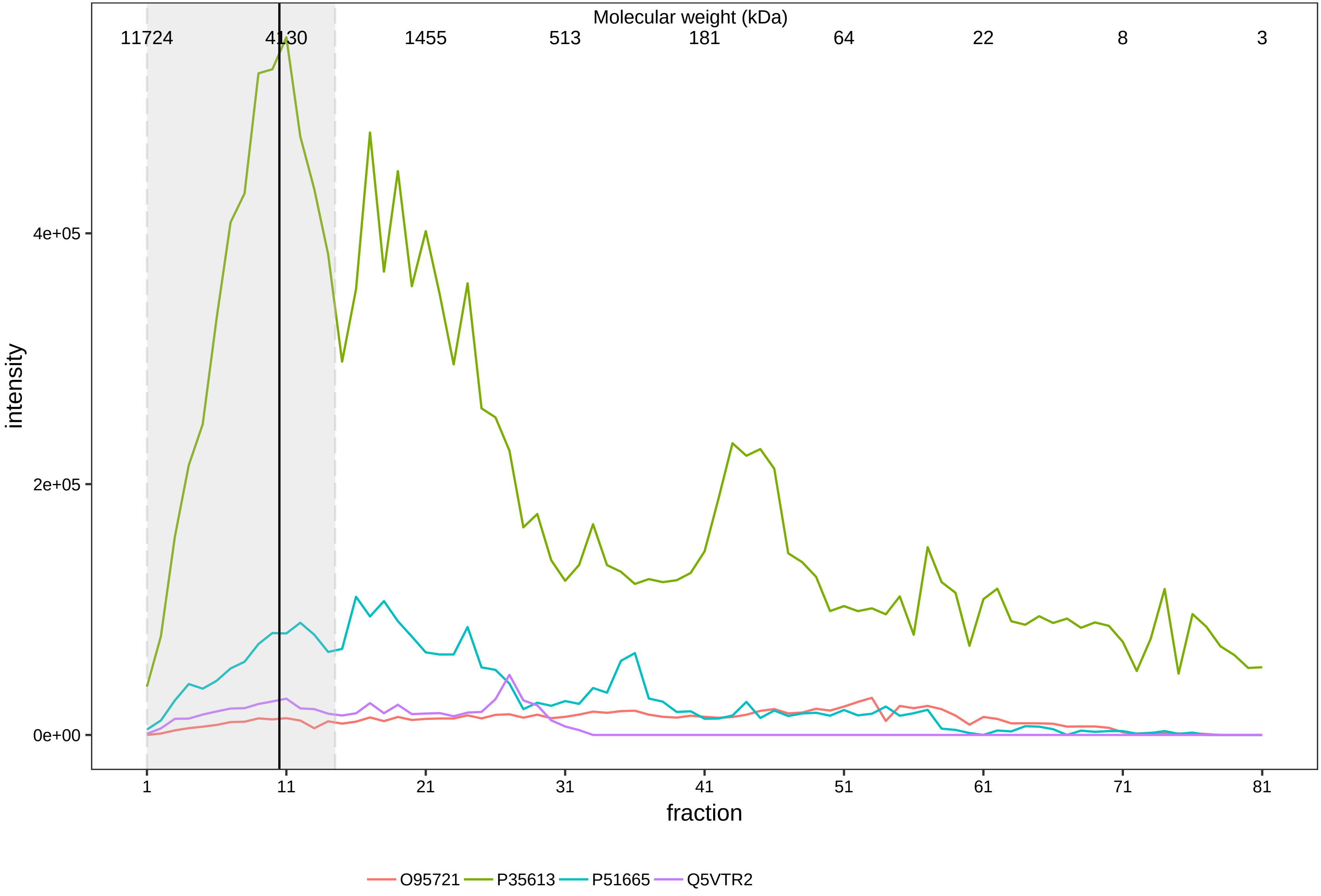

Feature ID 97

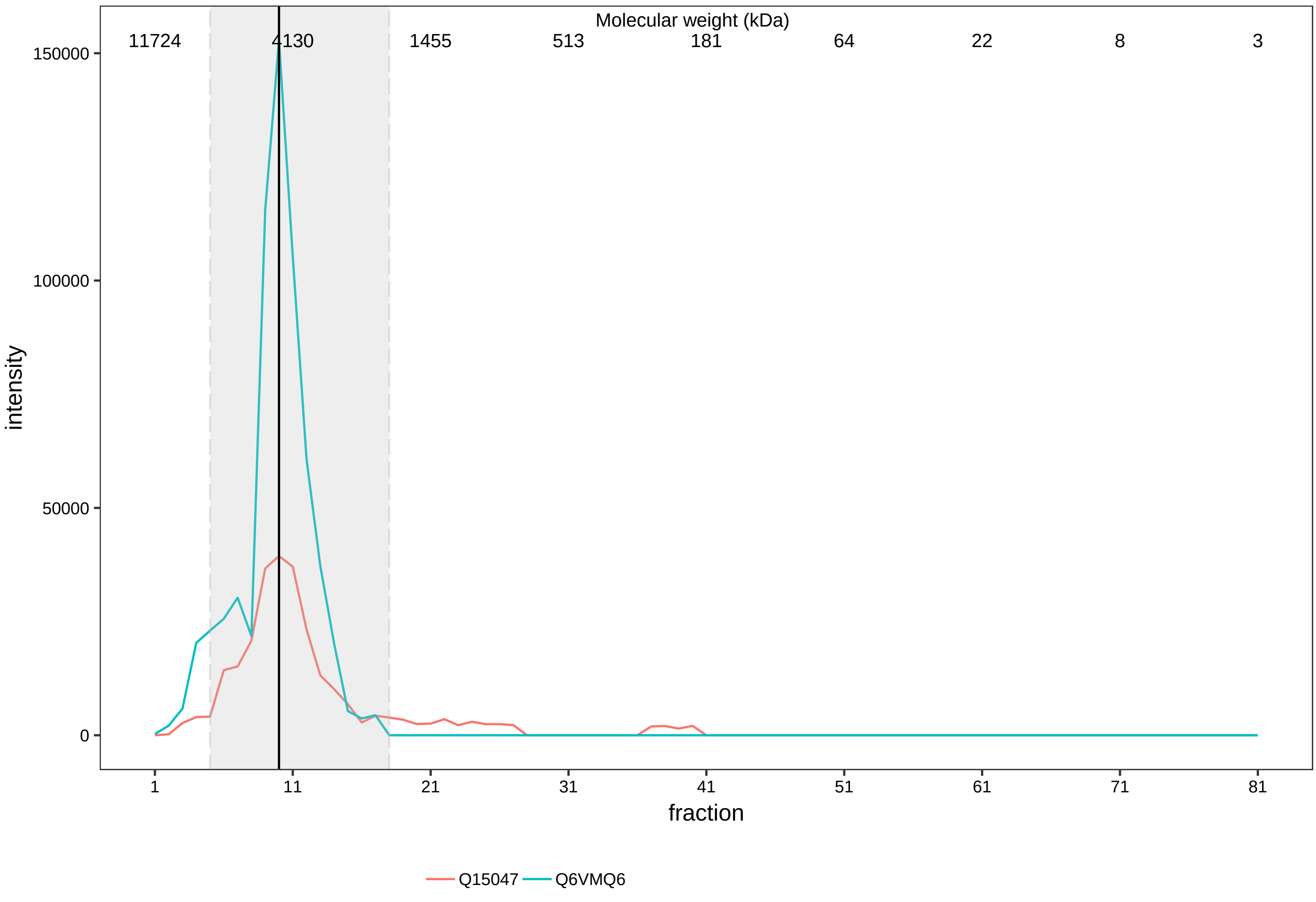

Feature ID 98

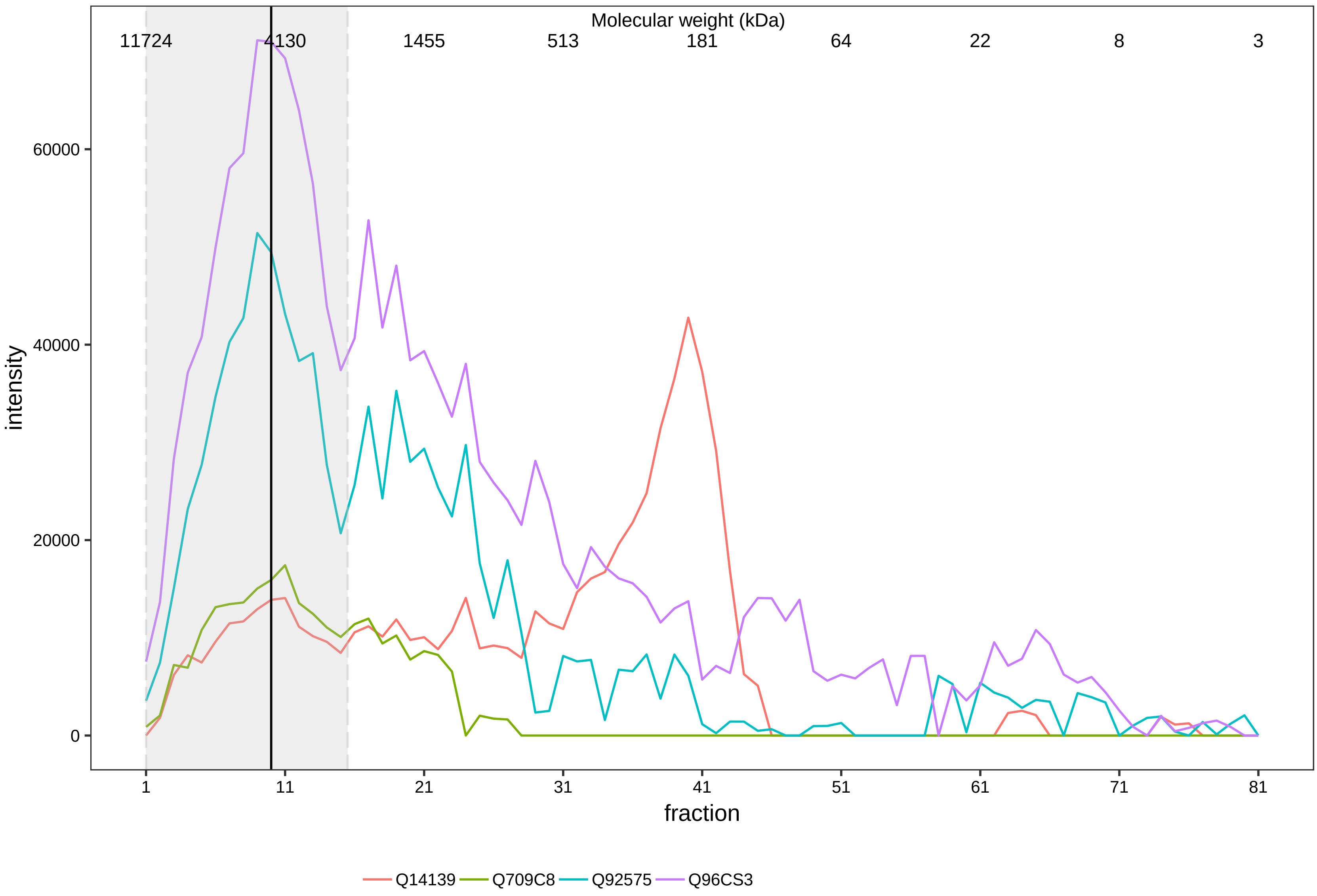

Feature ID 99

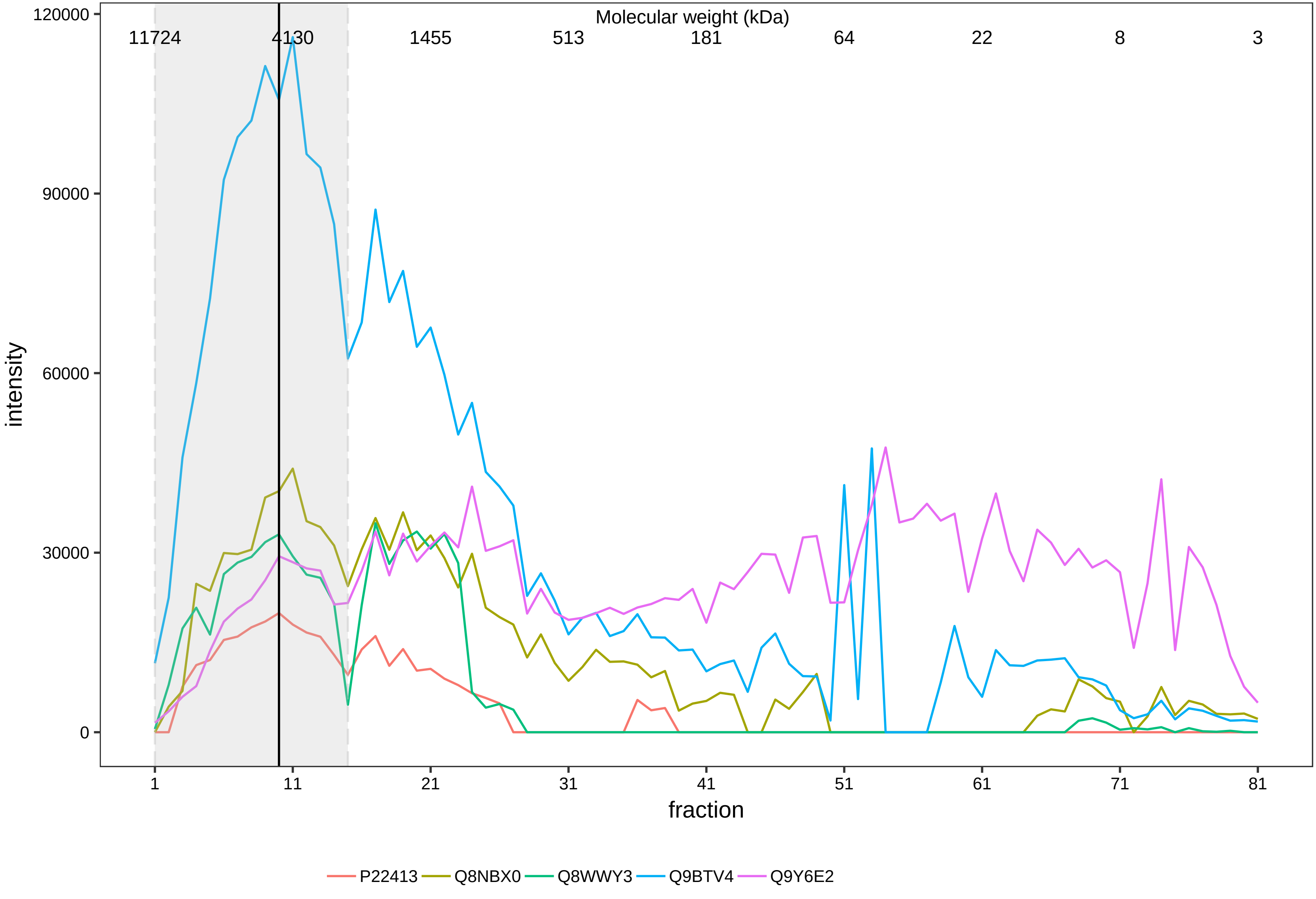

Feature ID 100

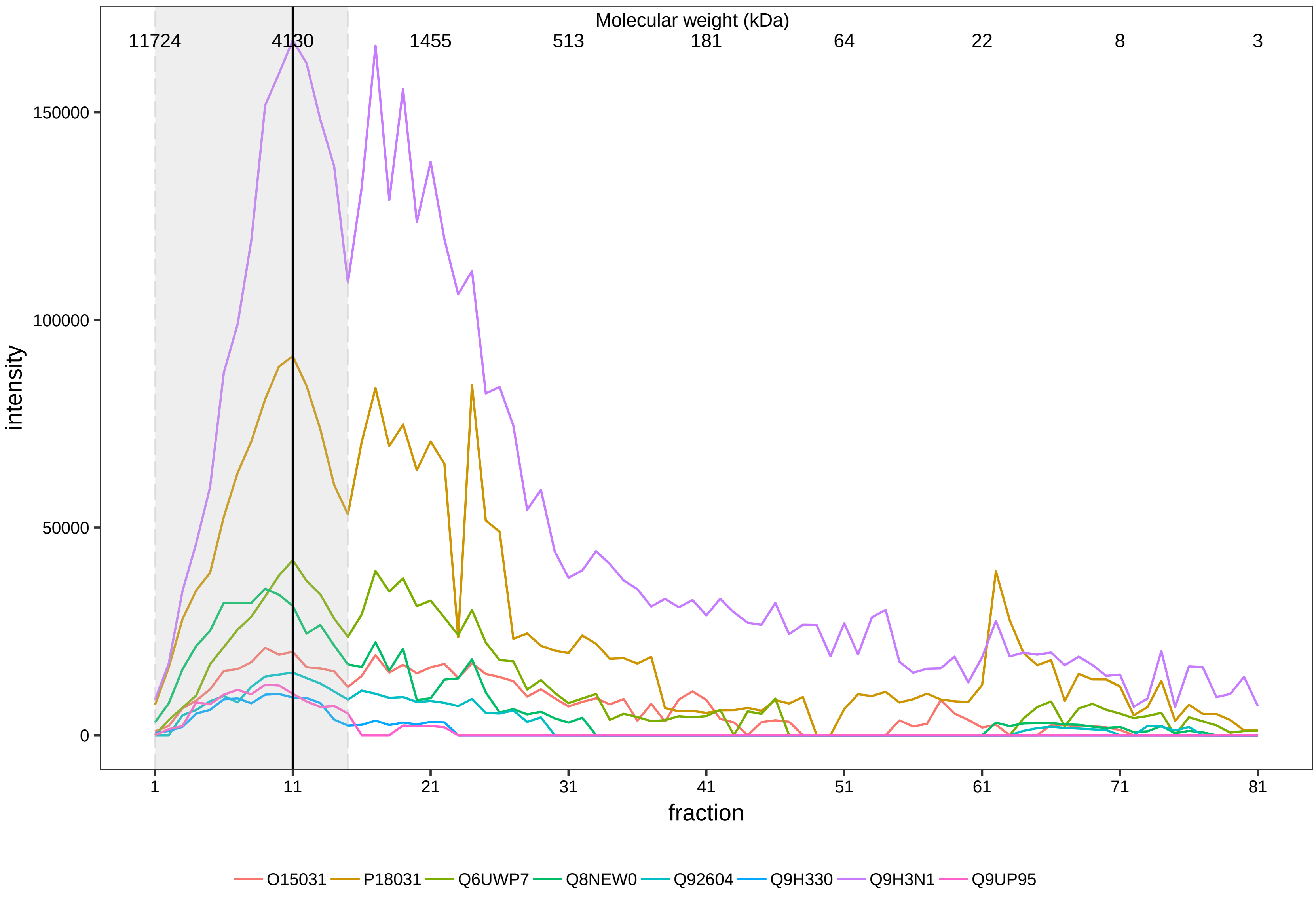

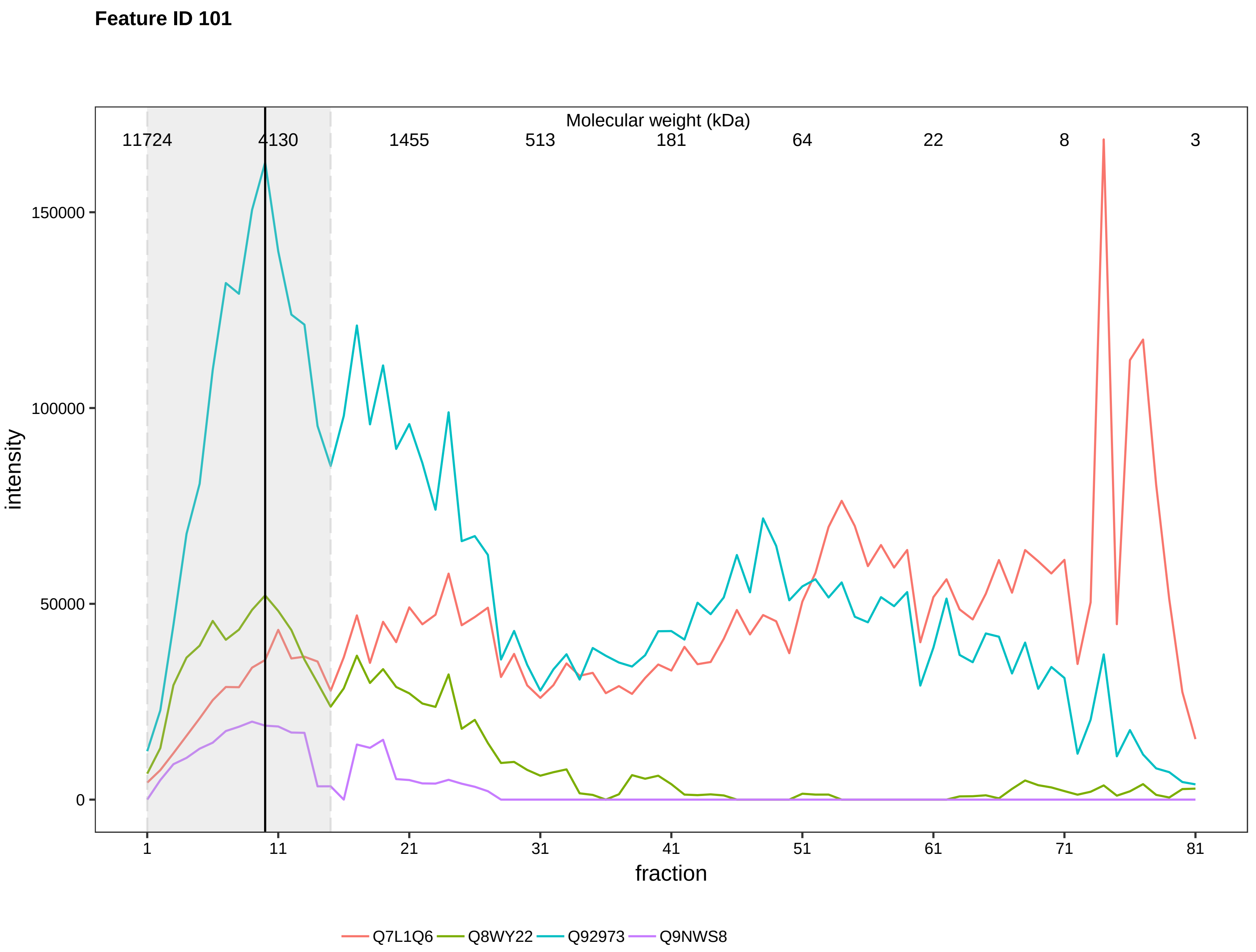

Feature ID 101
Molecular weight (kDa)
11724
4130
1455
513
181
64
22
8
3
150000
100000
50000
0
intensity
1
11
21
31
41
51
61
71
81
fraction
Q7L1Q6
Q8WY22
Q92973
Q9NWS8

Feature ID 102

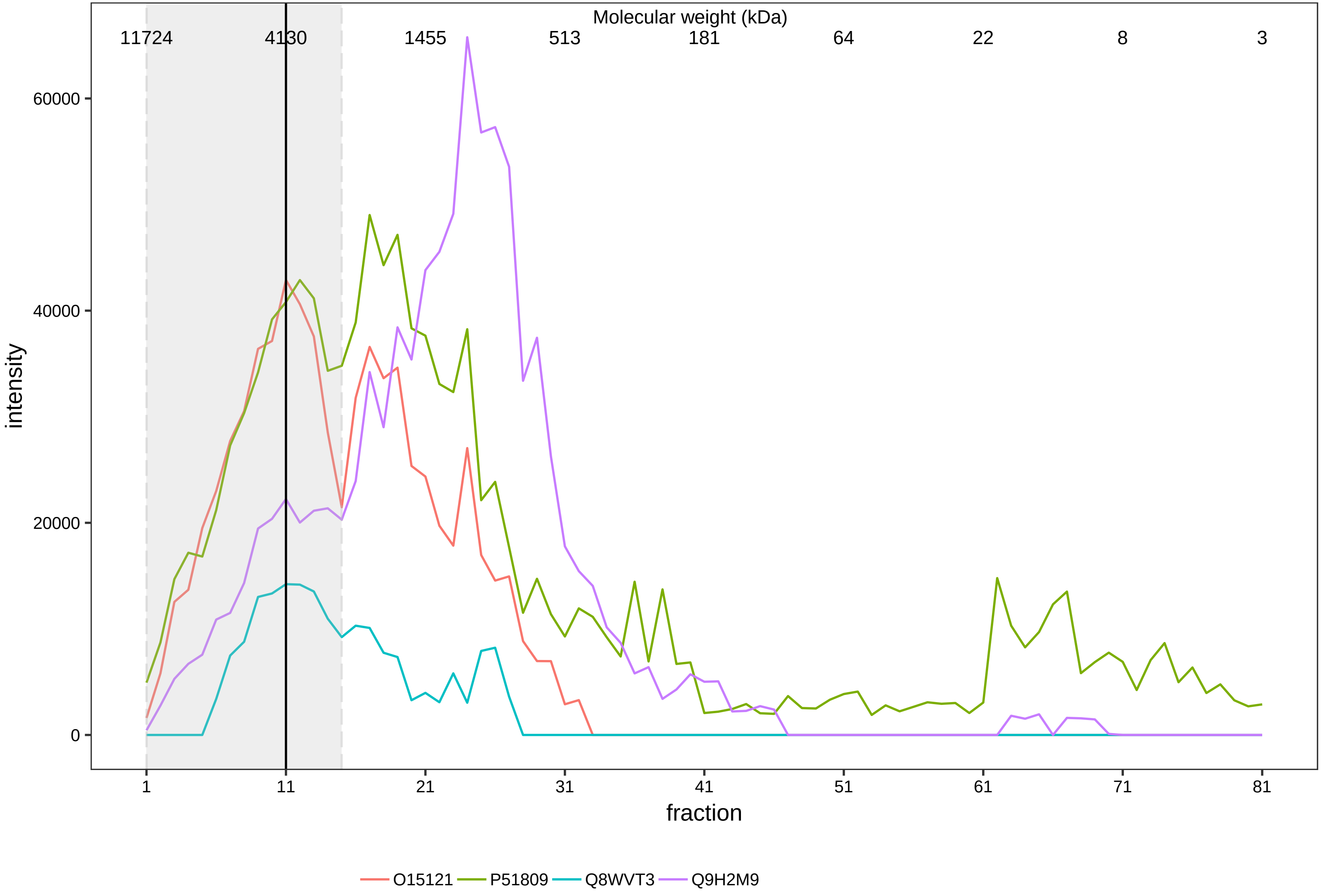

Feature ID 103

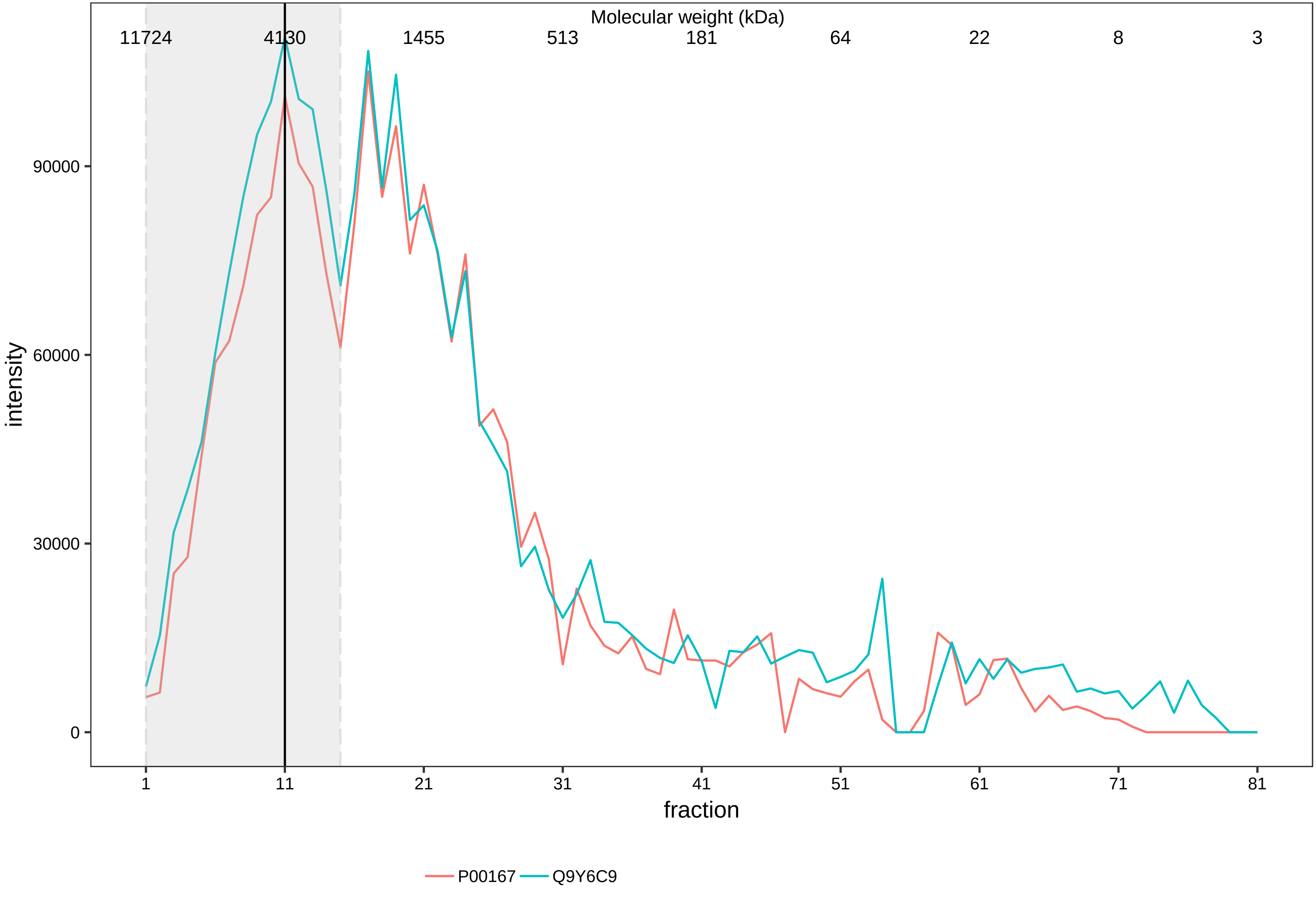

Feature ID 104

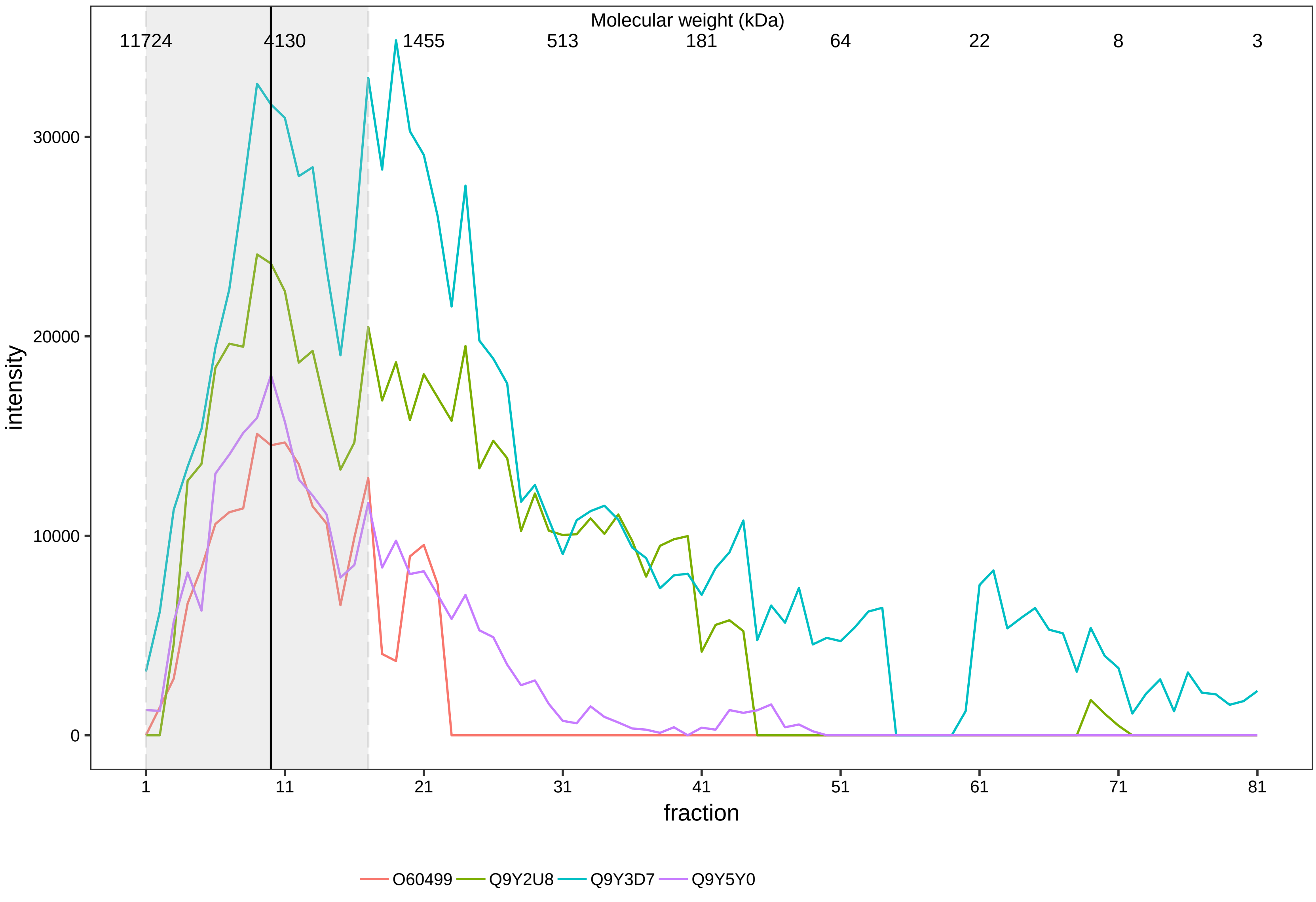

Feature ID 105

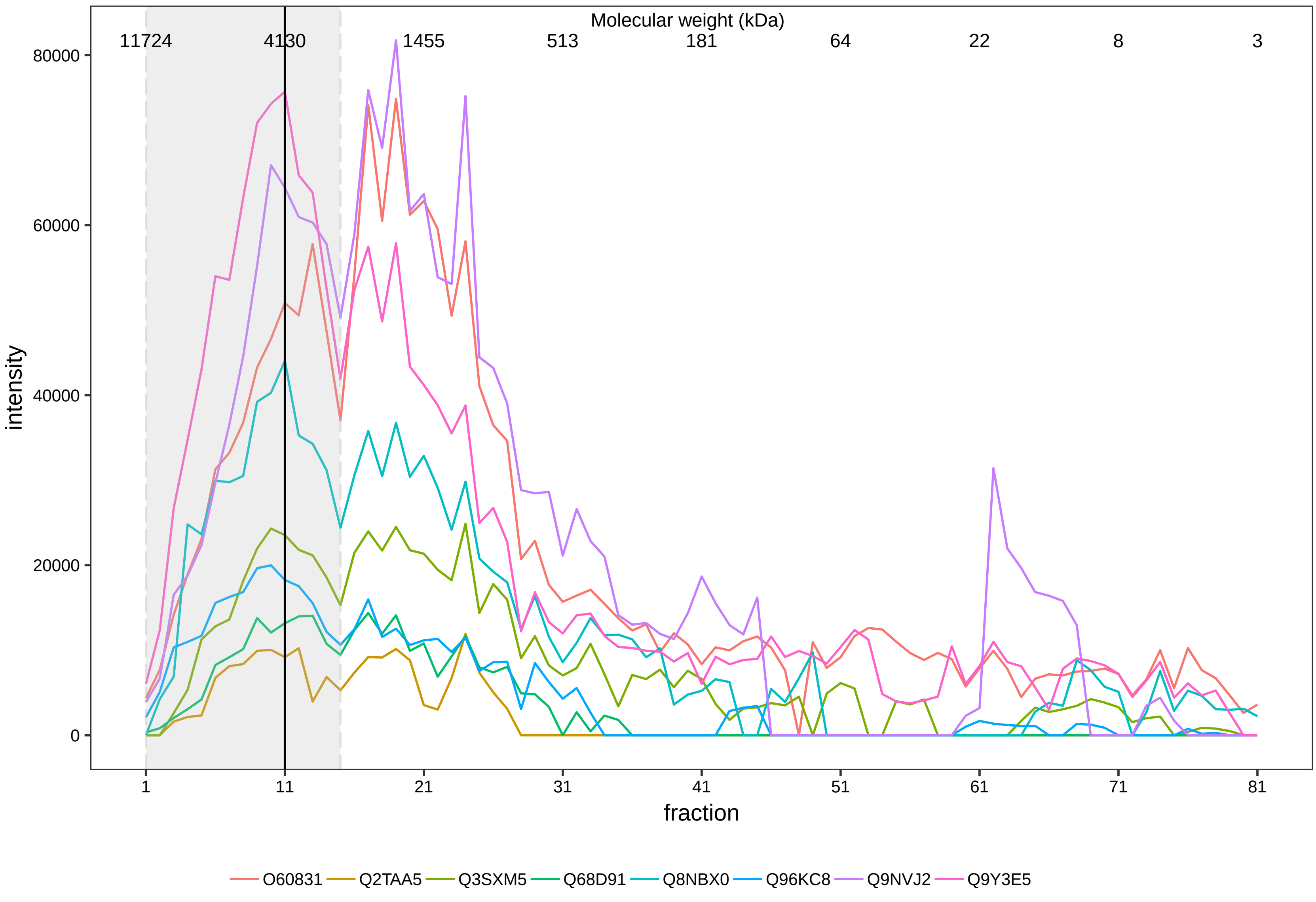

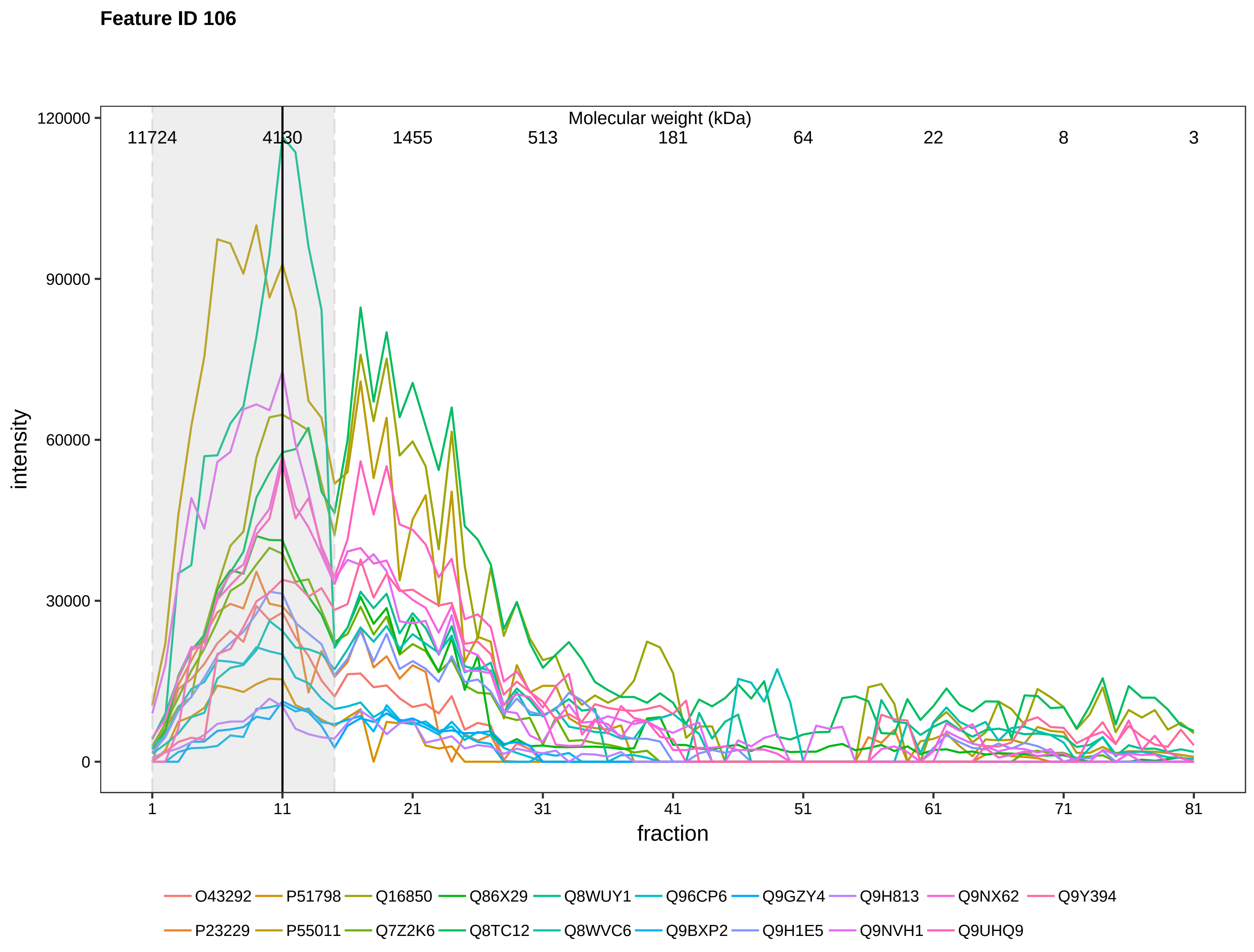

Feature ID 106
Molecular weight (kDa)
11724
4130
1455
513
181
64
22
8
3
120000
90000
60000
30000
0
intensity
1
11
21
31
41
51
61
71
81
fraction
O43292
P51798
Q16850
Q86X29
Q8WUY1
Q96CP6
Q9GZY4
Q9H813
Q9NX62
Q9Y394
P23229
P55011
Q7Z2K6
Q8TC12
Q8WVC6
Q9BXP2
Q9H1E5
Q9NVH1
Q9UHQ9

Feature ID 107

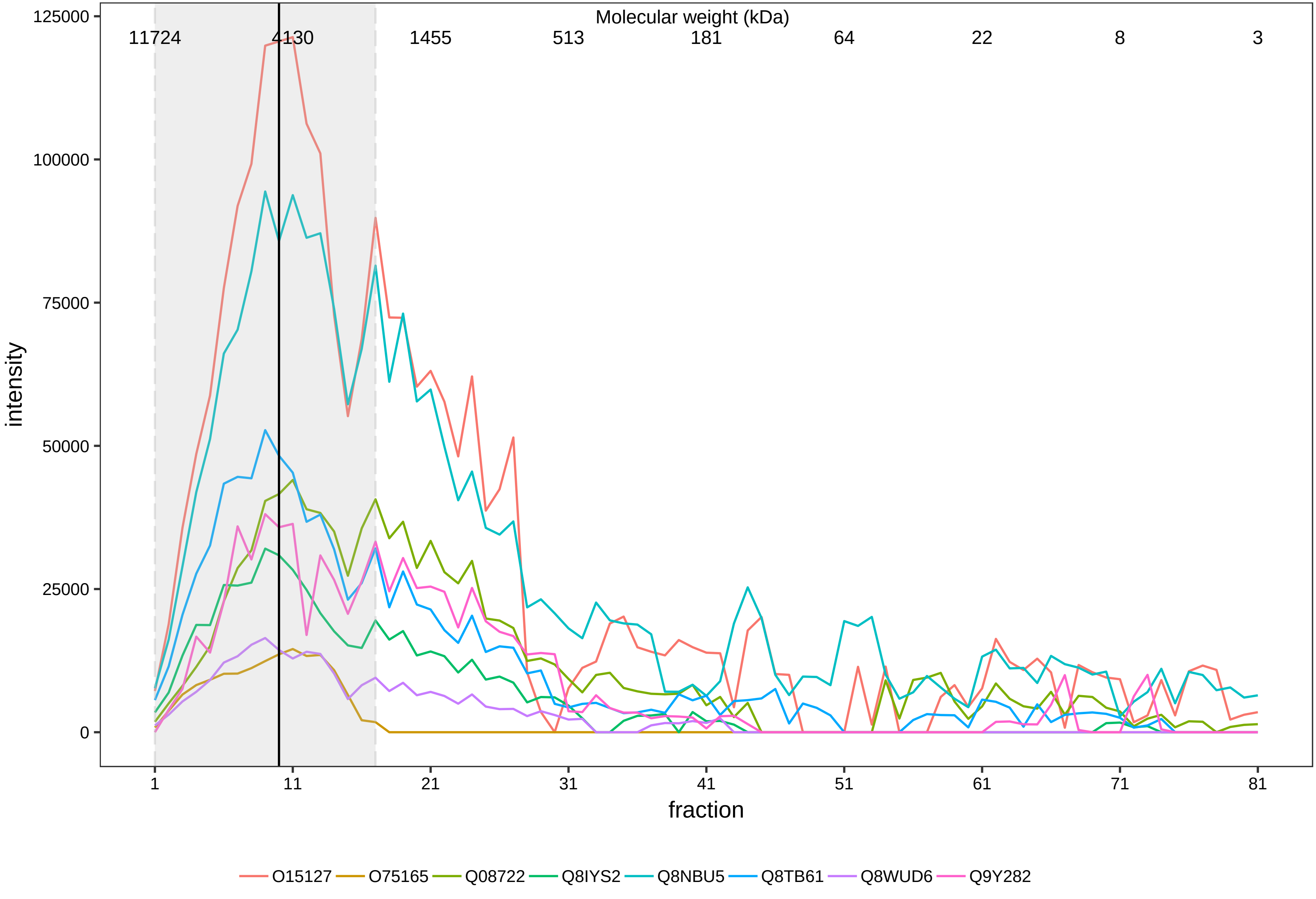

Feature ID 108

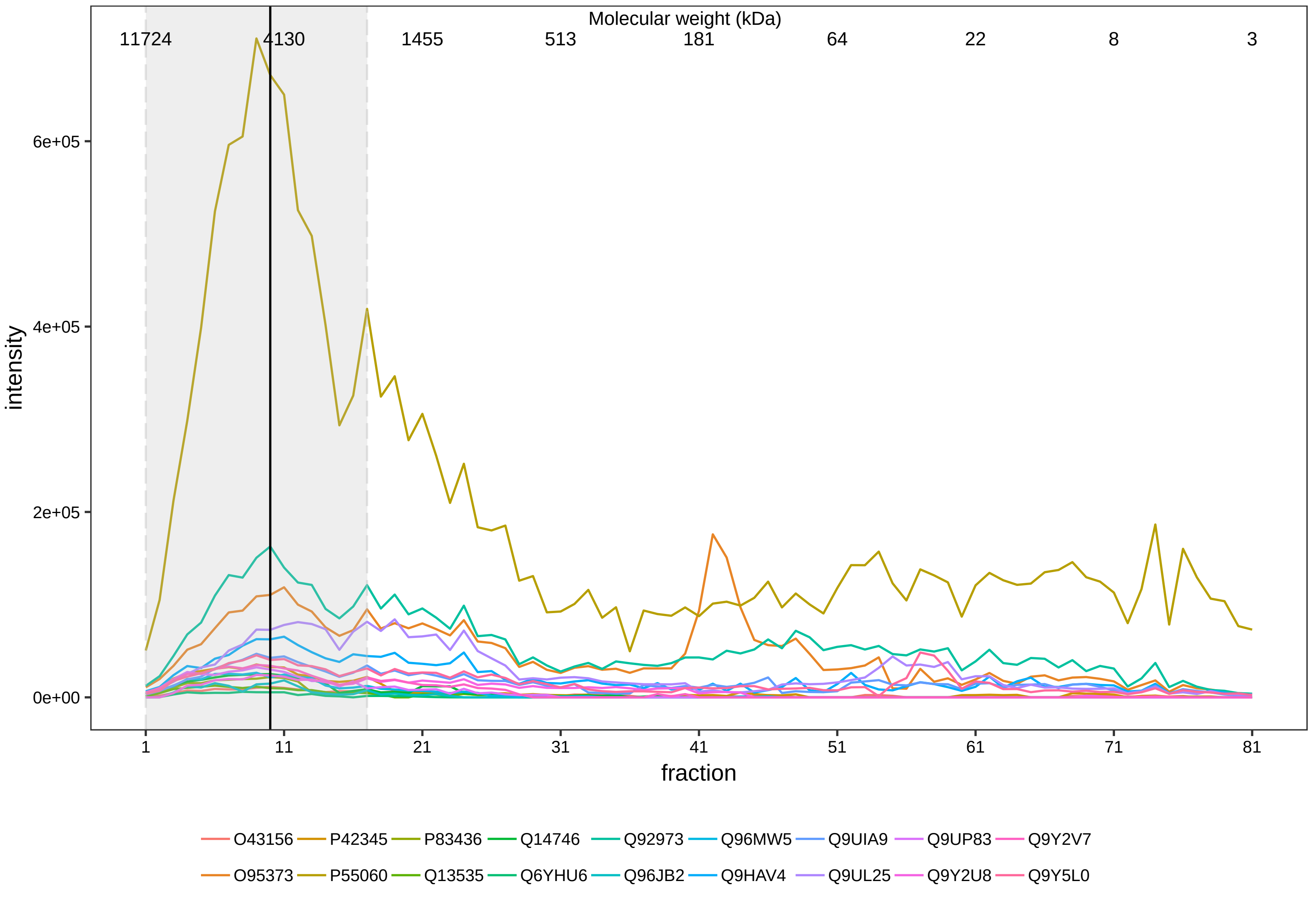

Feature ID 109

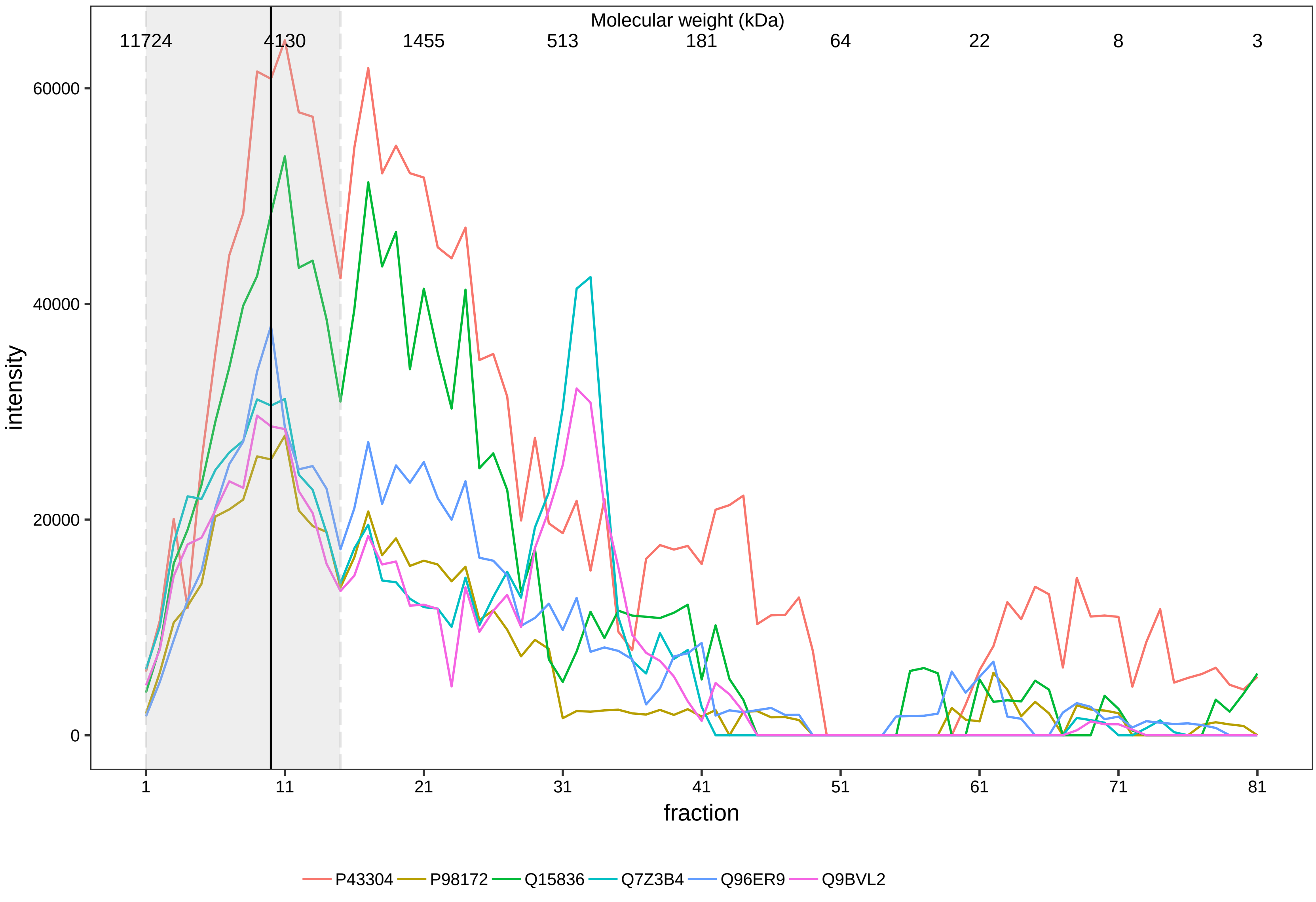

Feature ID 110

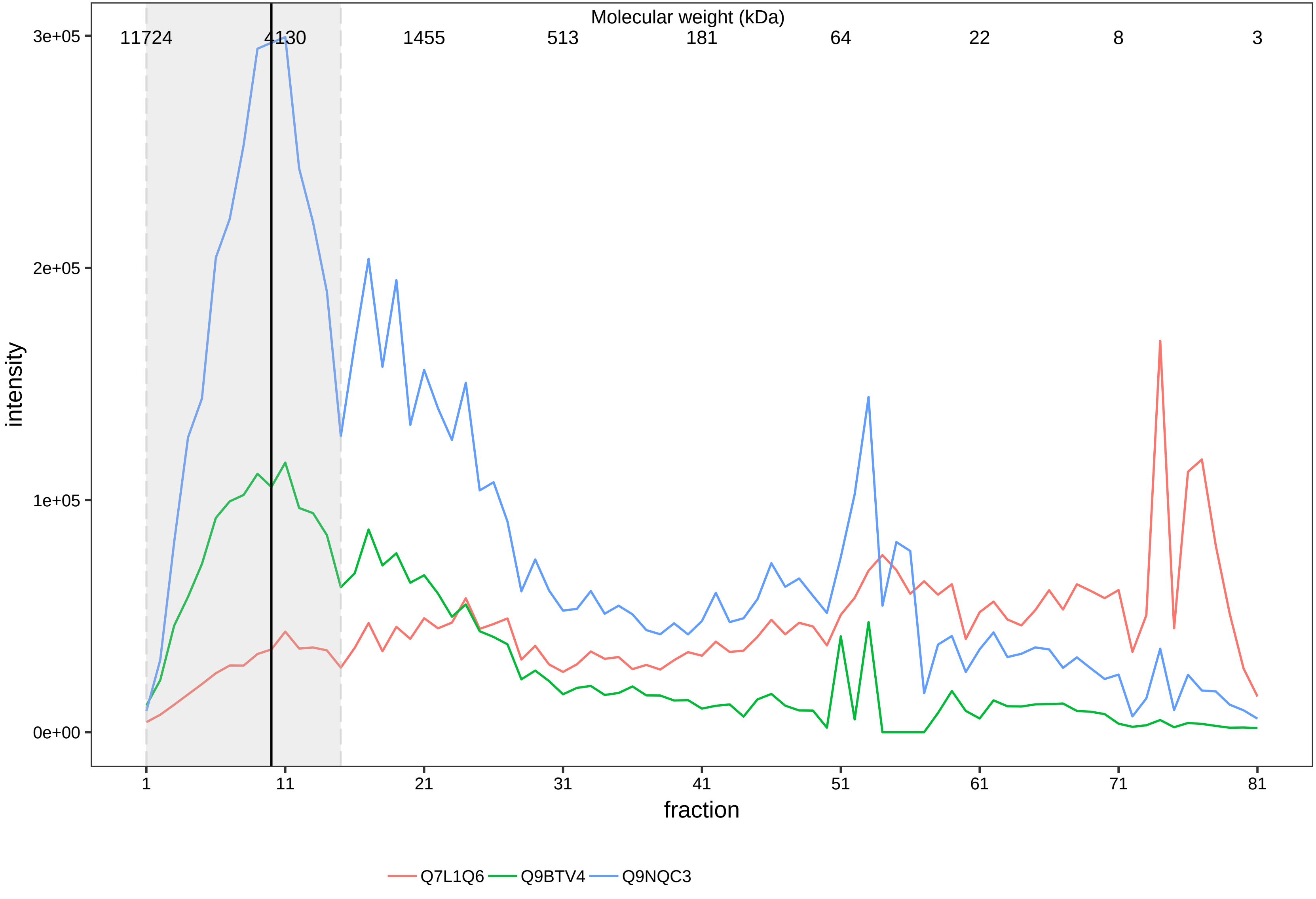

Feature ID 111

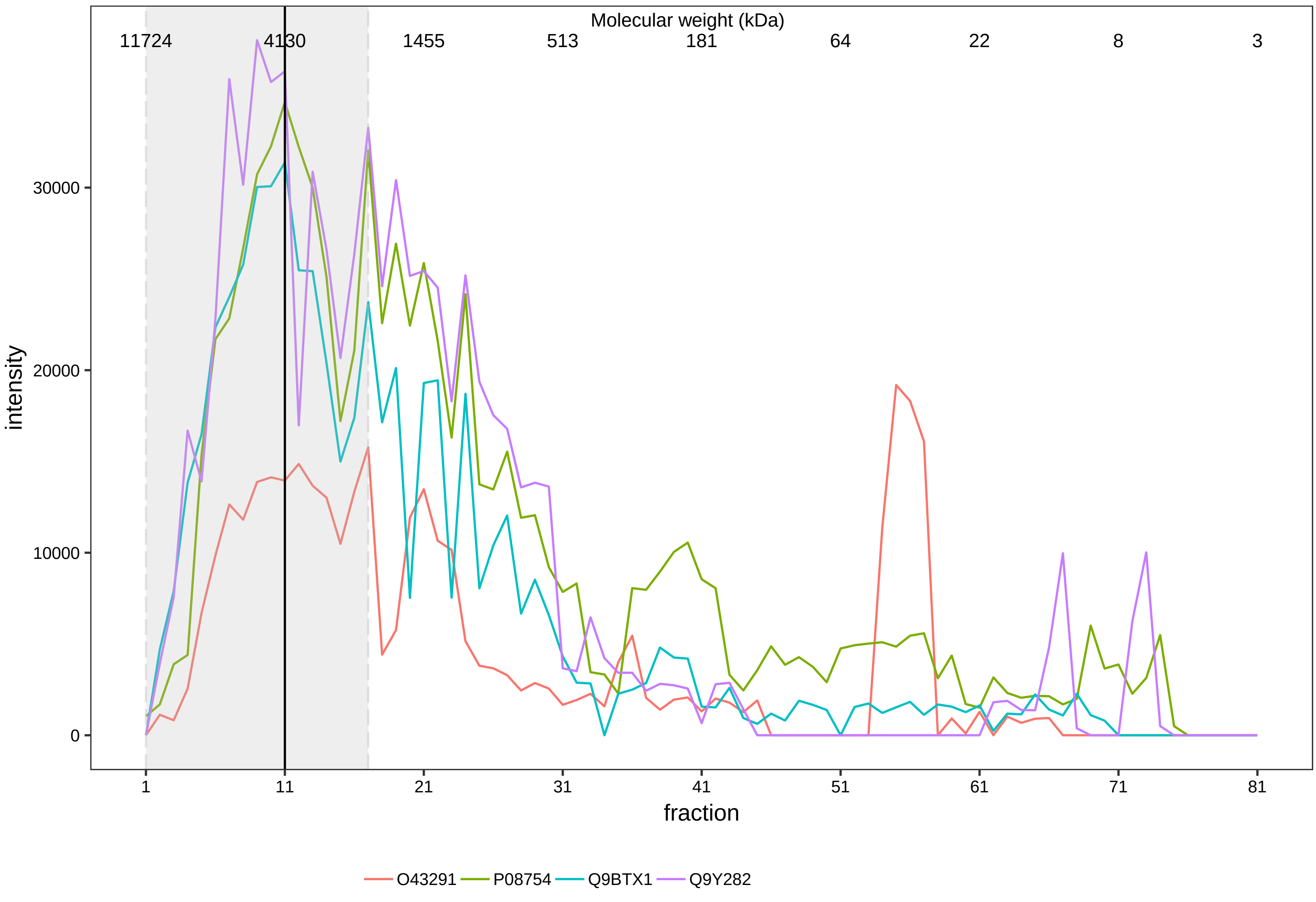

Feature ID 112

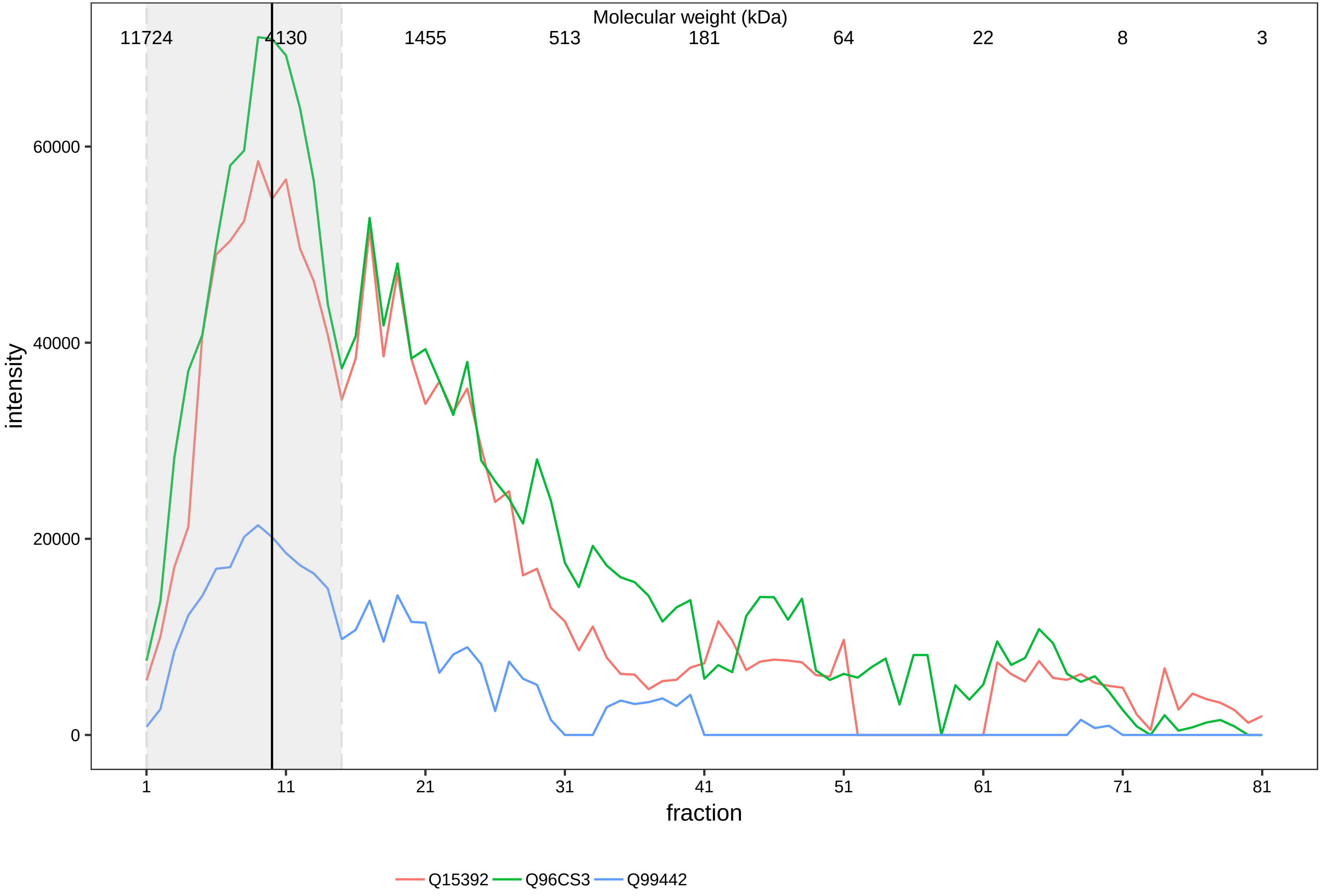

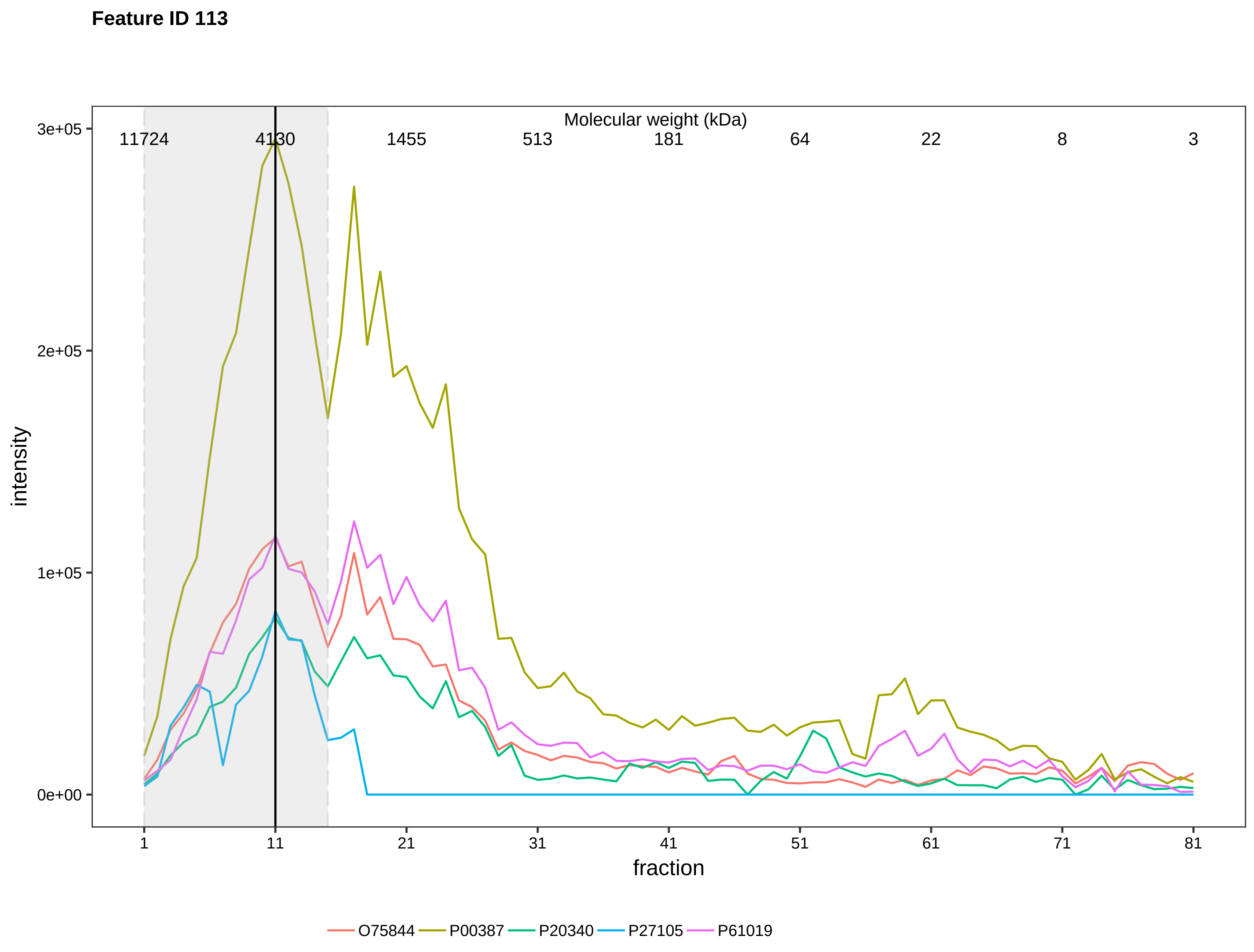
Feature ID 113
Molecular weight (kDa)
11724
4130
1455
513
181
64
22
8
3
3e+05
2e+05
1e+05
0e+00
intensity
1
11
21
31
41
51
61
71
81
fraction
O75844
P00387
P20340
P27105
P61019

Feature ID 114

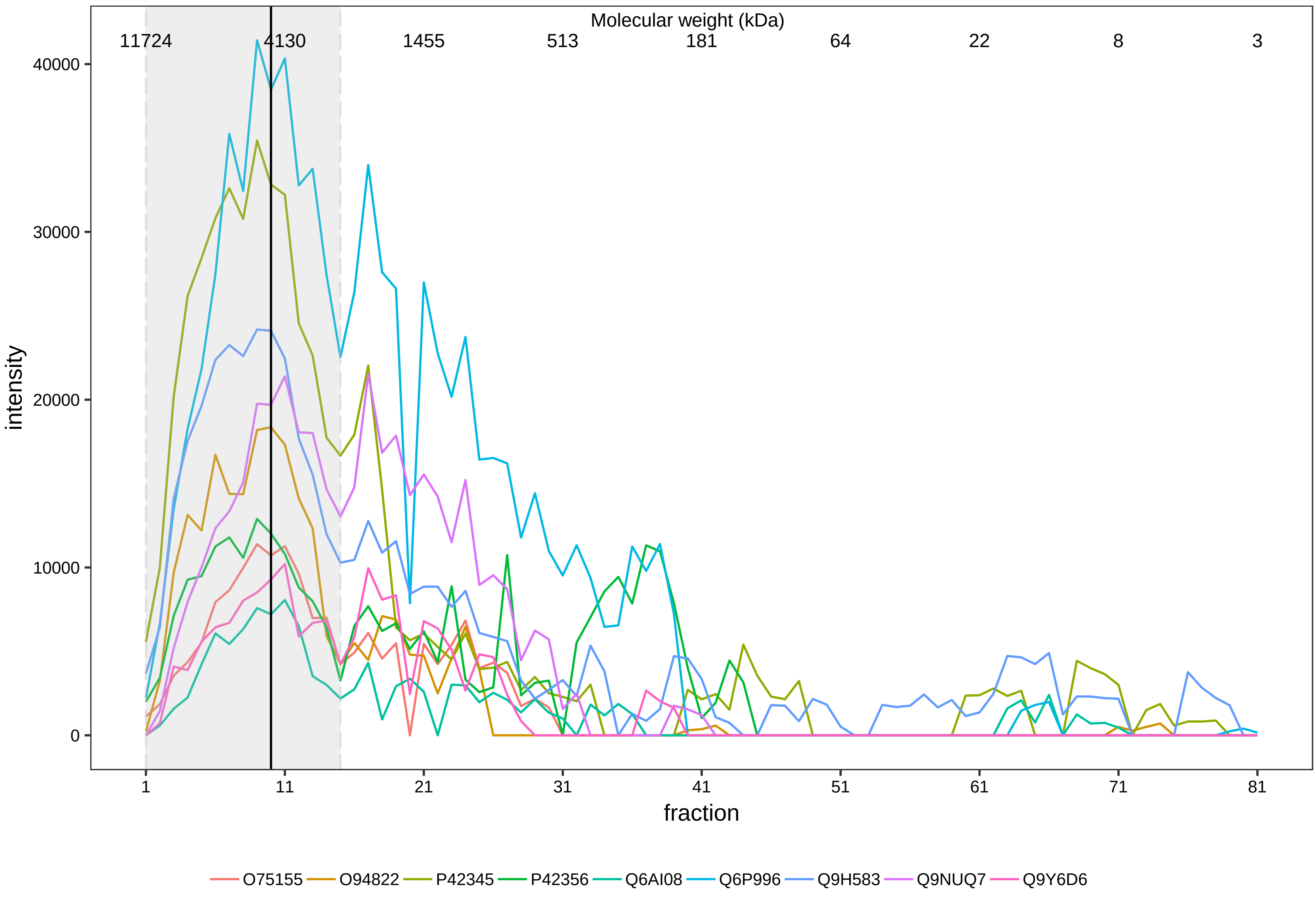

Feature ID 115

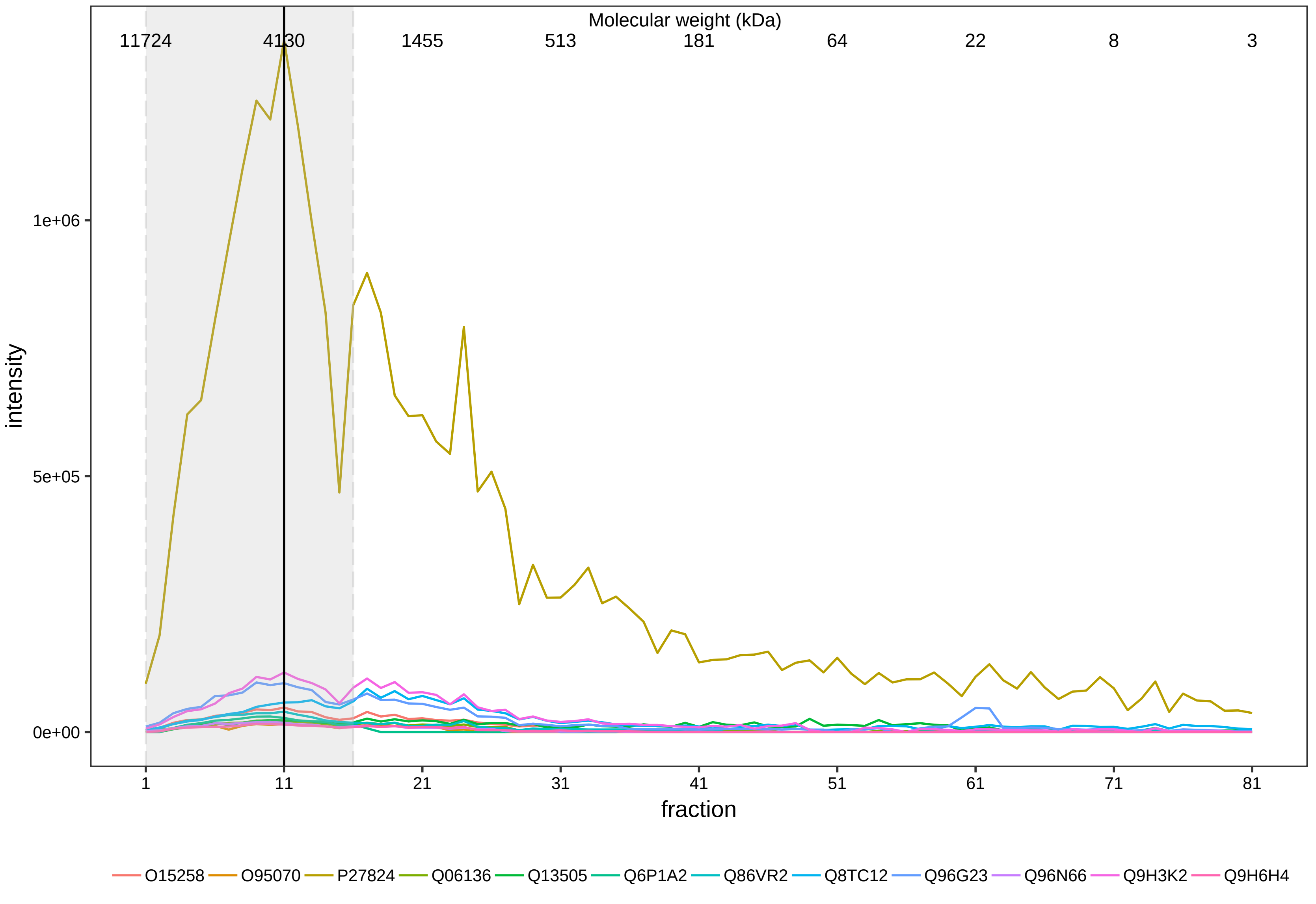

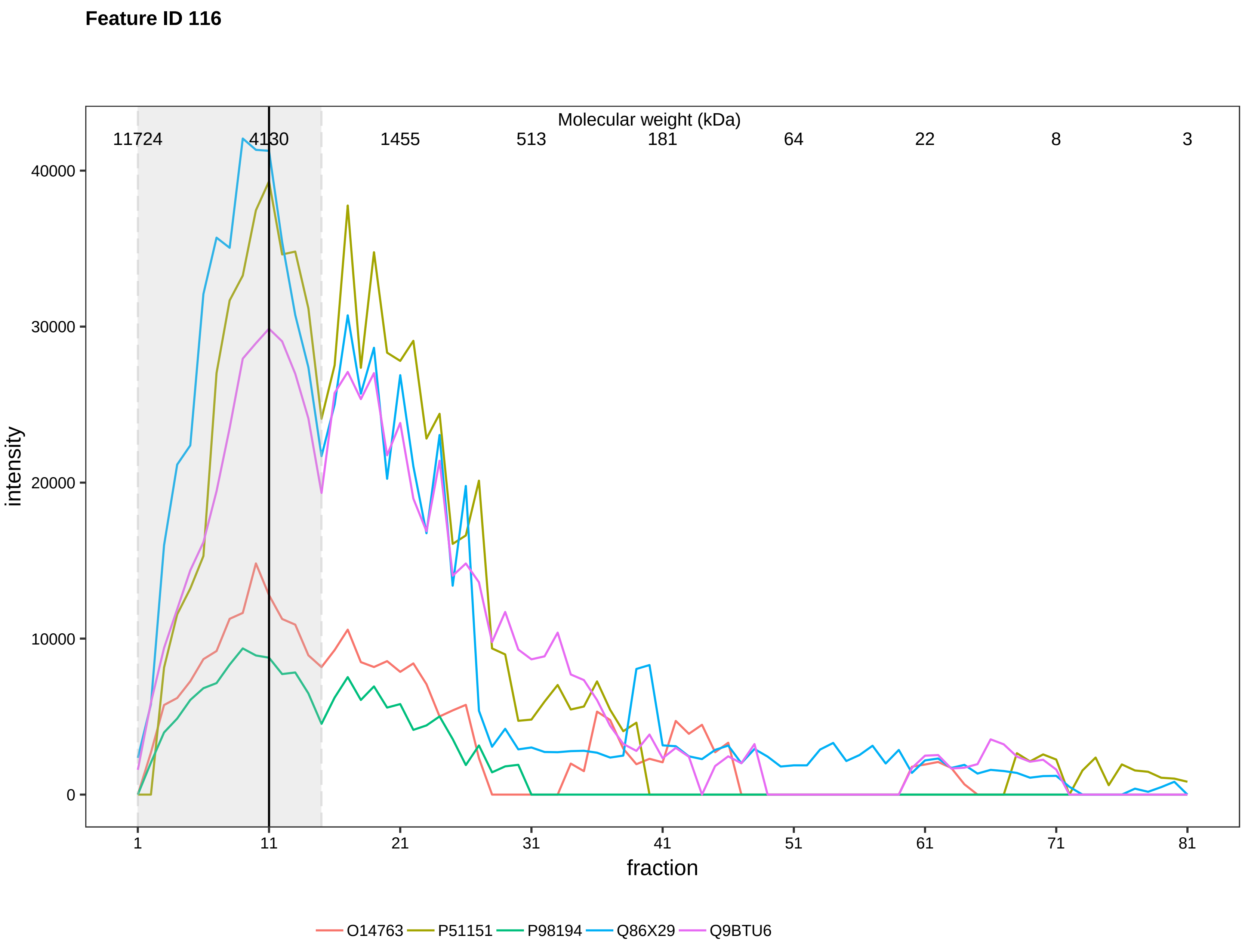
Feature ID 116
Molecular weight (kDa)
11724
4130
1455
513
181
64
22
8
3
40000
30000
20000
10000
0
intensity
1
11
21
31
41
51
61
71
81
fraction
O14763
P51151
P98194
Q86X29
Q9BTU6

Feature ID 117

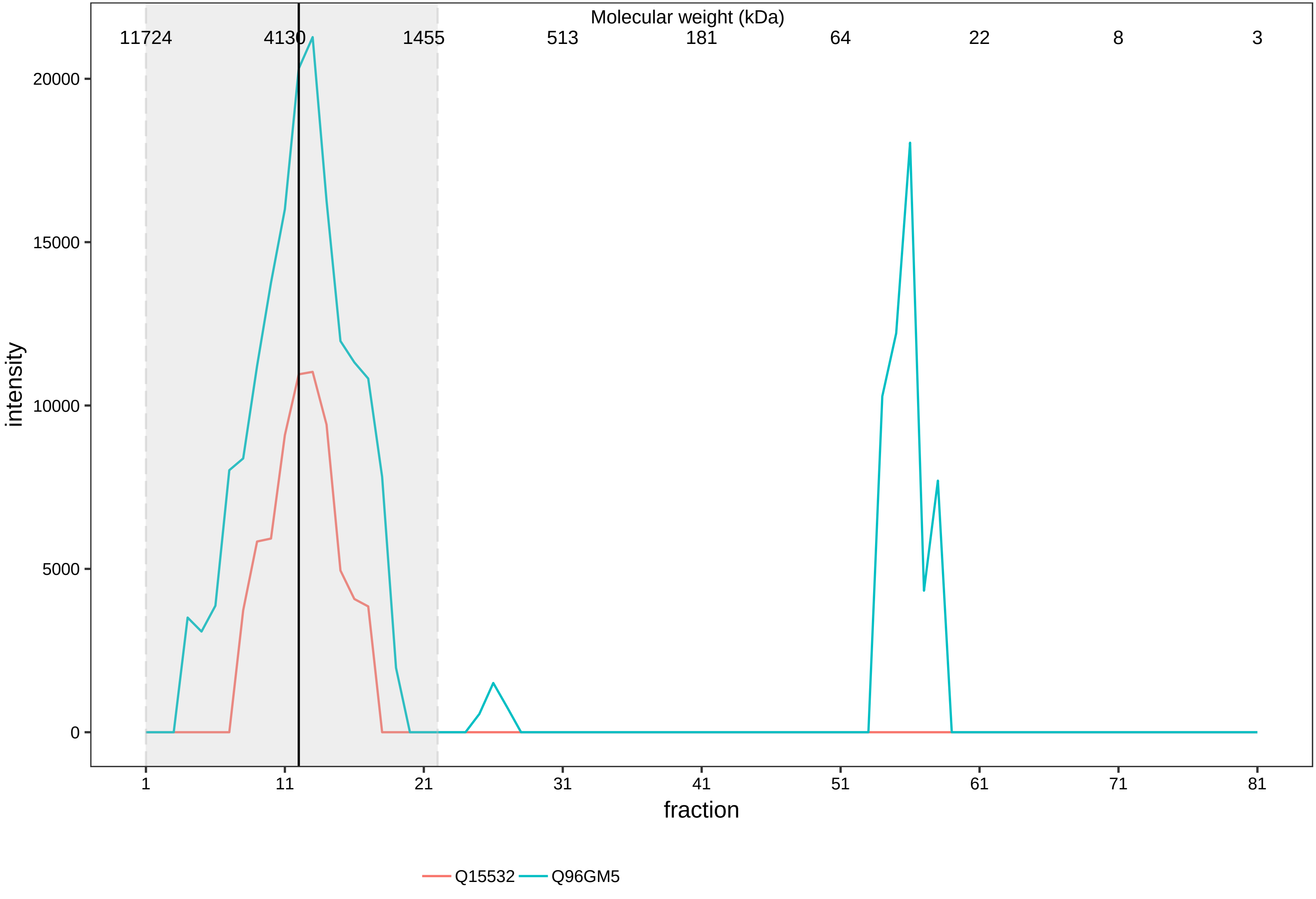

Feature ID 118

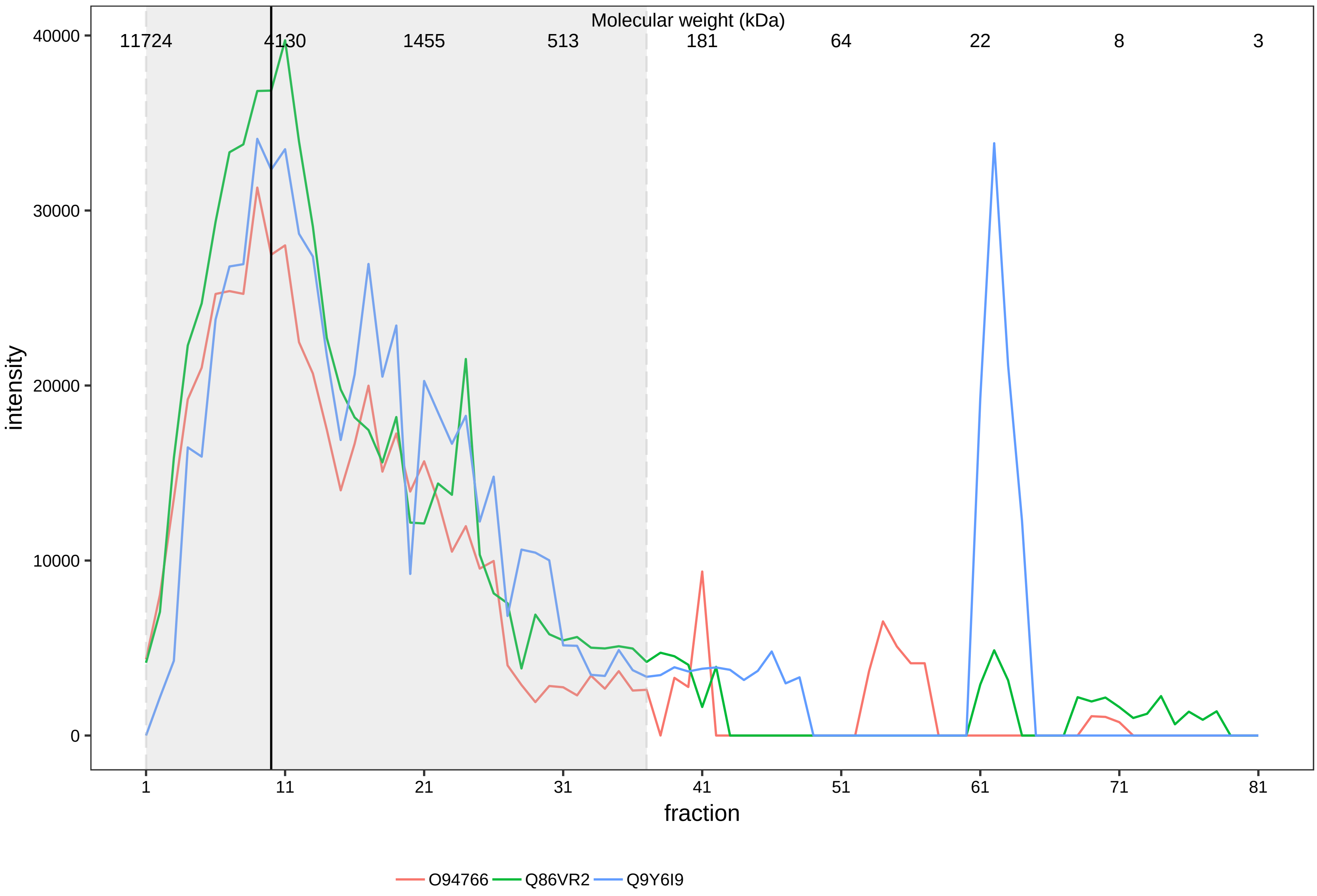

Feature ID 119

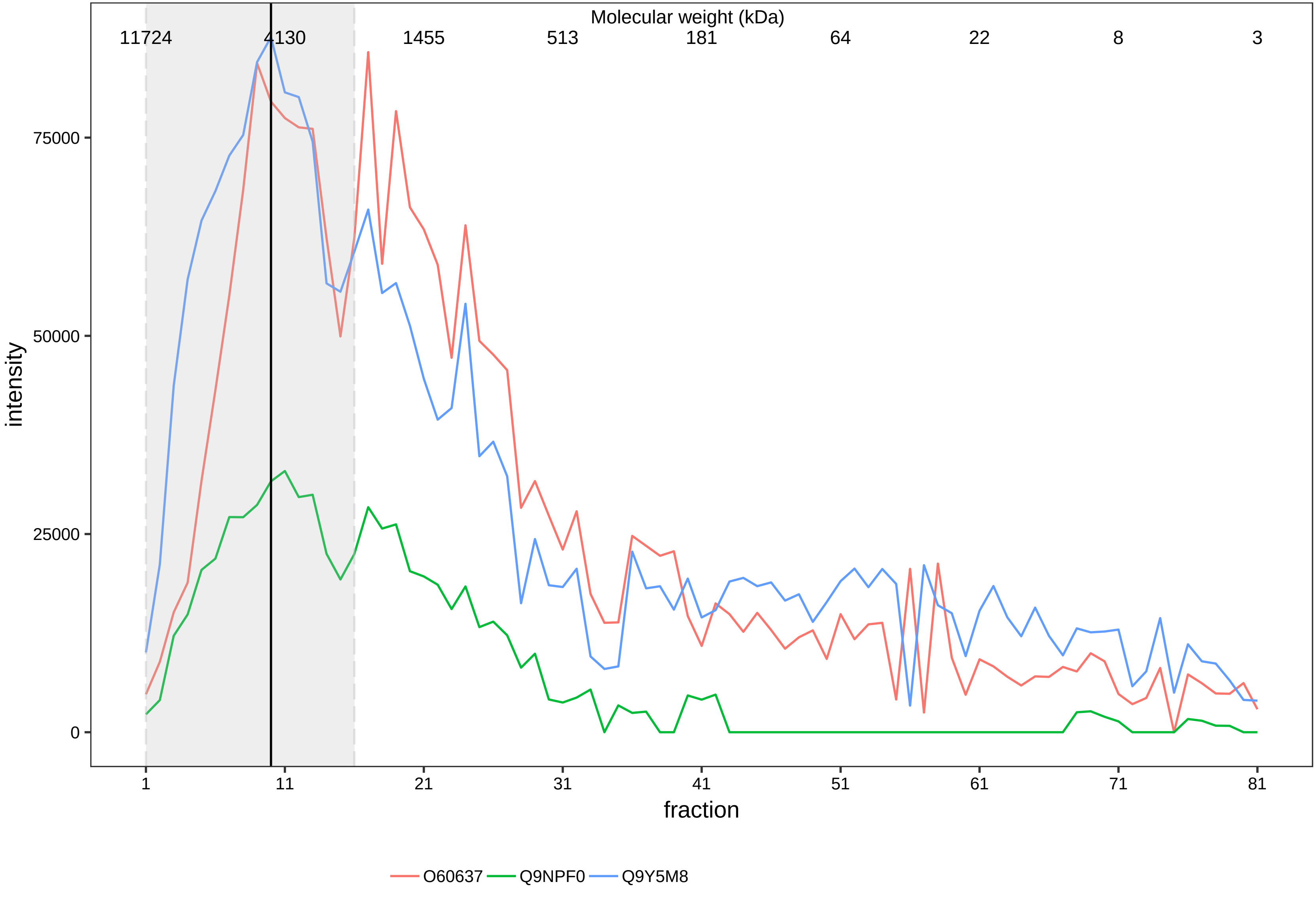

Feature ID 120

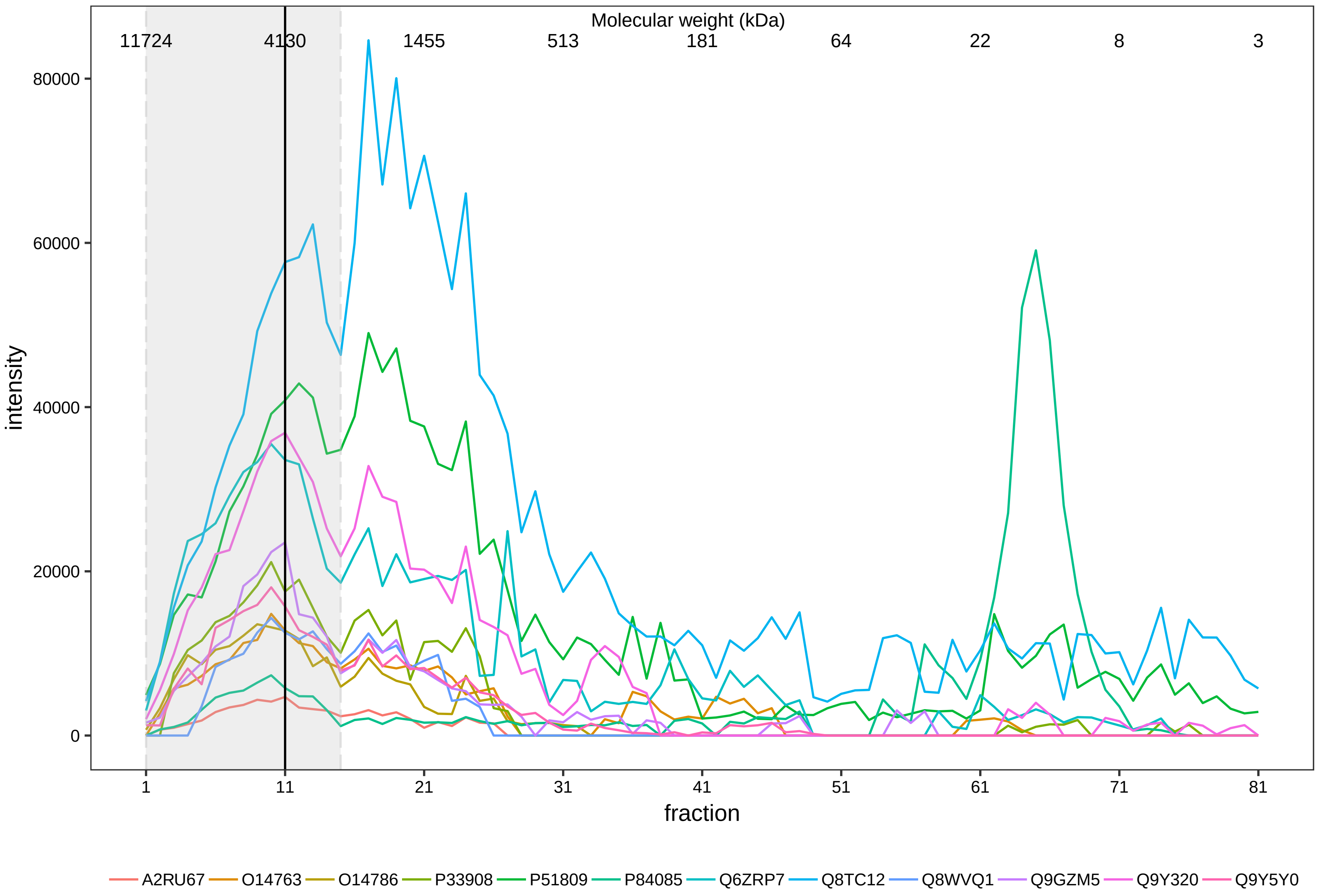

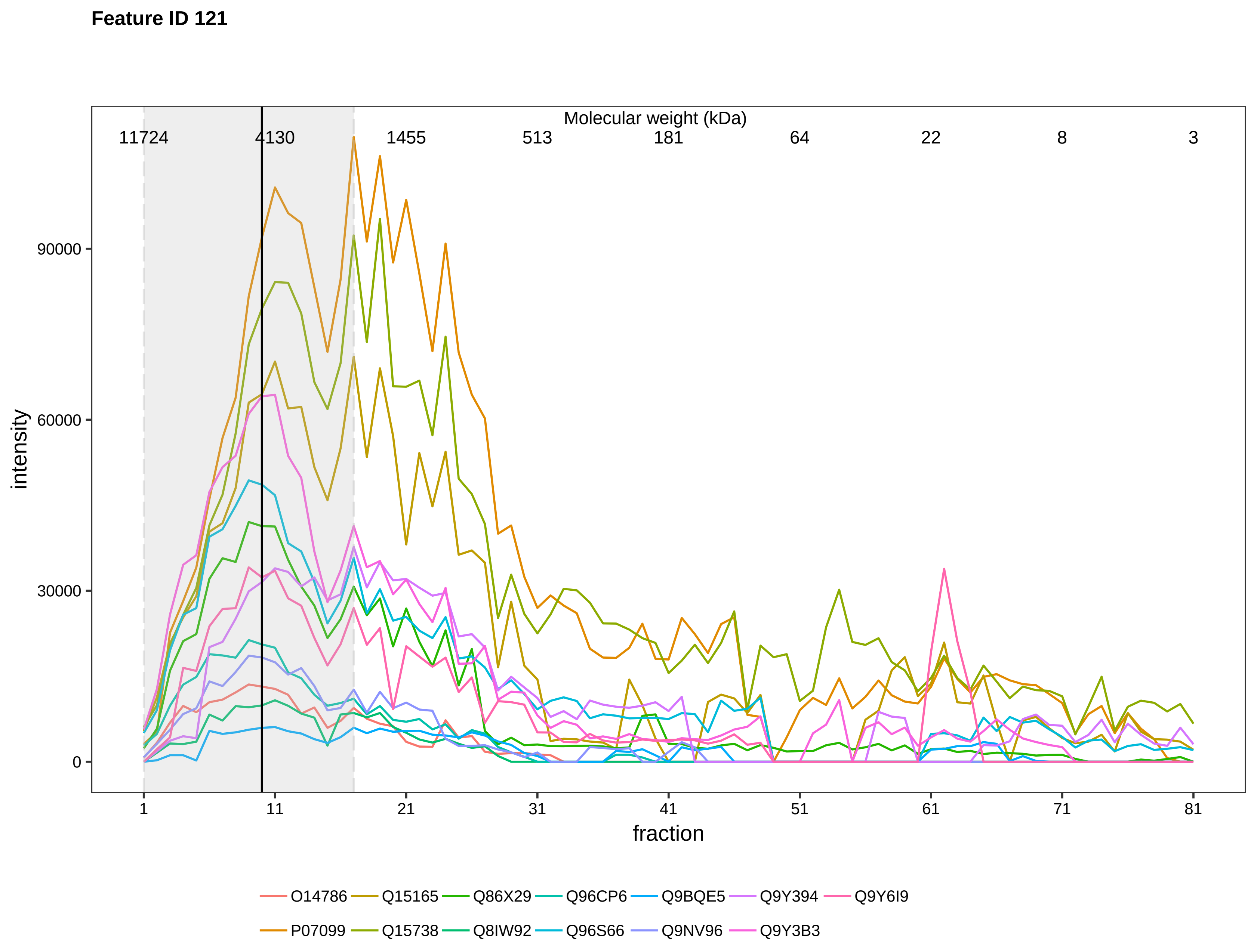

Feature ID 121
Molecular weight (kDa)
11724
4130
1455
513
181
64
22
8
3
intensity
90000
60000
30000
0
1
11
21
31
41
51
61
71
81
fraction
O14786
Q15165
Q86X29
Q96CP6
Q9BQE5
Q9Y394
Q9Y6I9
P07099
Q15738
Q8IW92
Q96S66
Q9NV96
Q9Y3B3

Feature ID 122

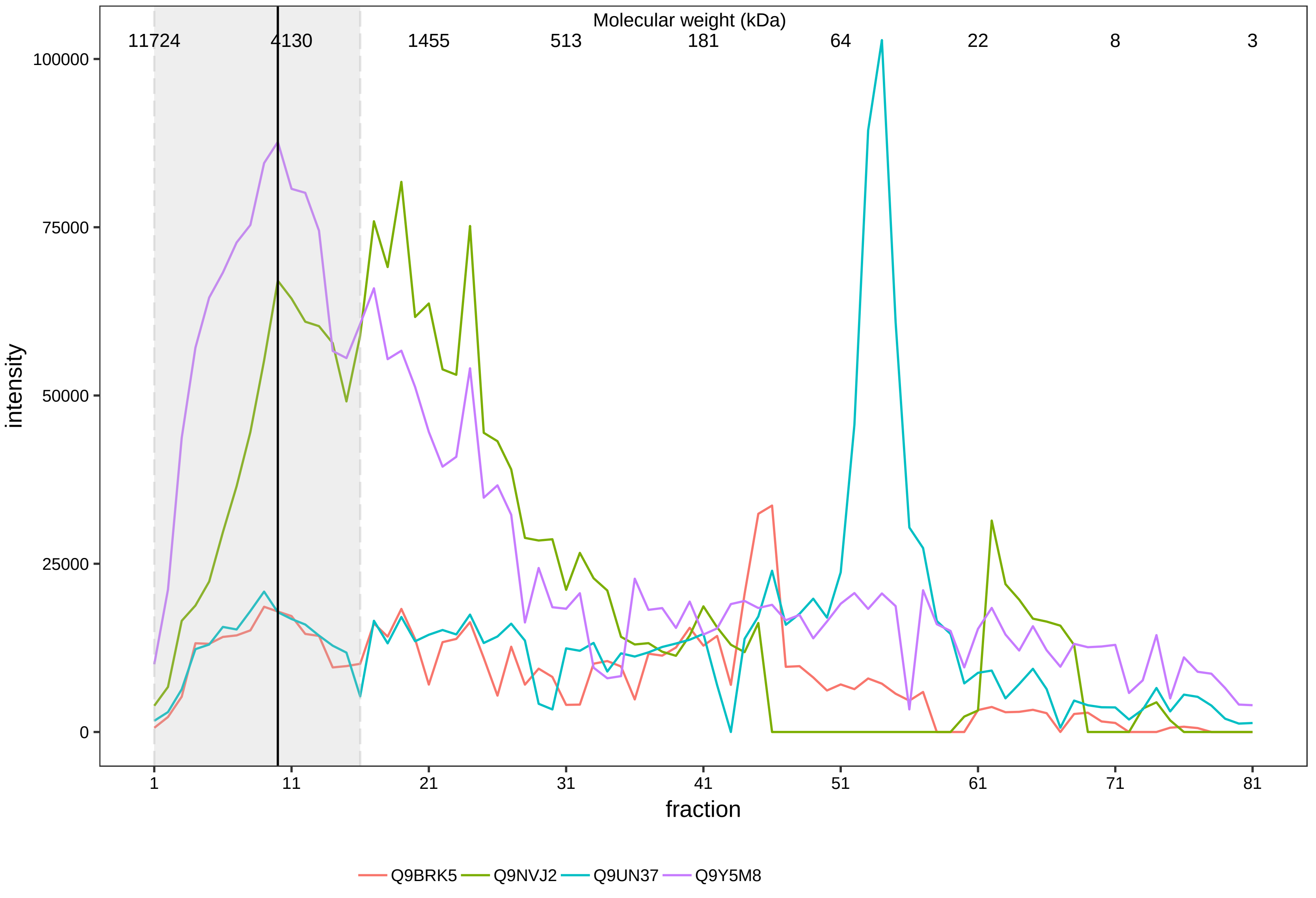

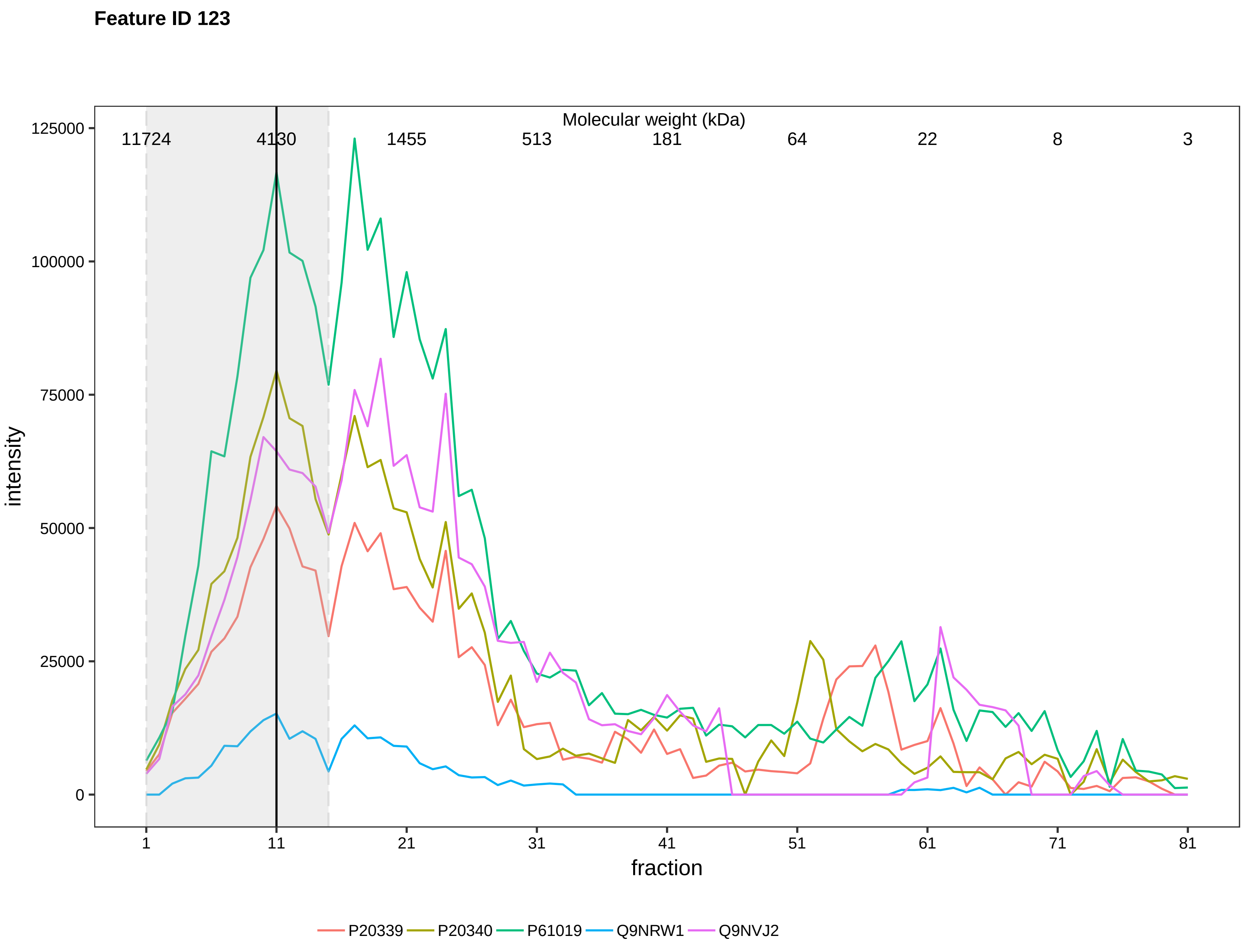

Feature ID 123
Molecular weight (kDa)
11724
4130
1455
513
181
64
22
8
3
125000
100000
75000
50000
25000
0
intensity
1
11
21
31
41
51
61
71
81
fraction
P20339
P20340
P61019
Q9NRW1
Q9NVJ2

Feature ID 124

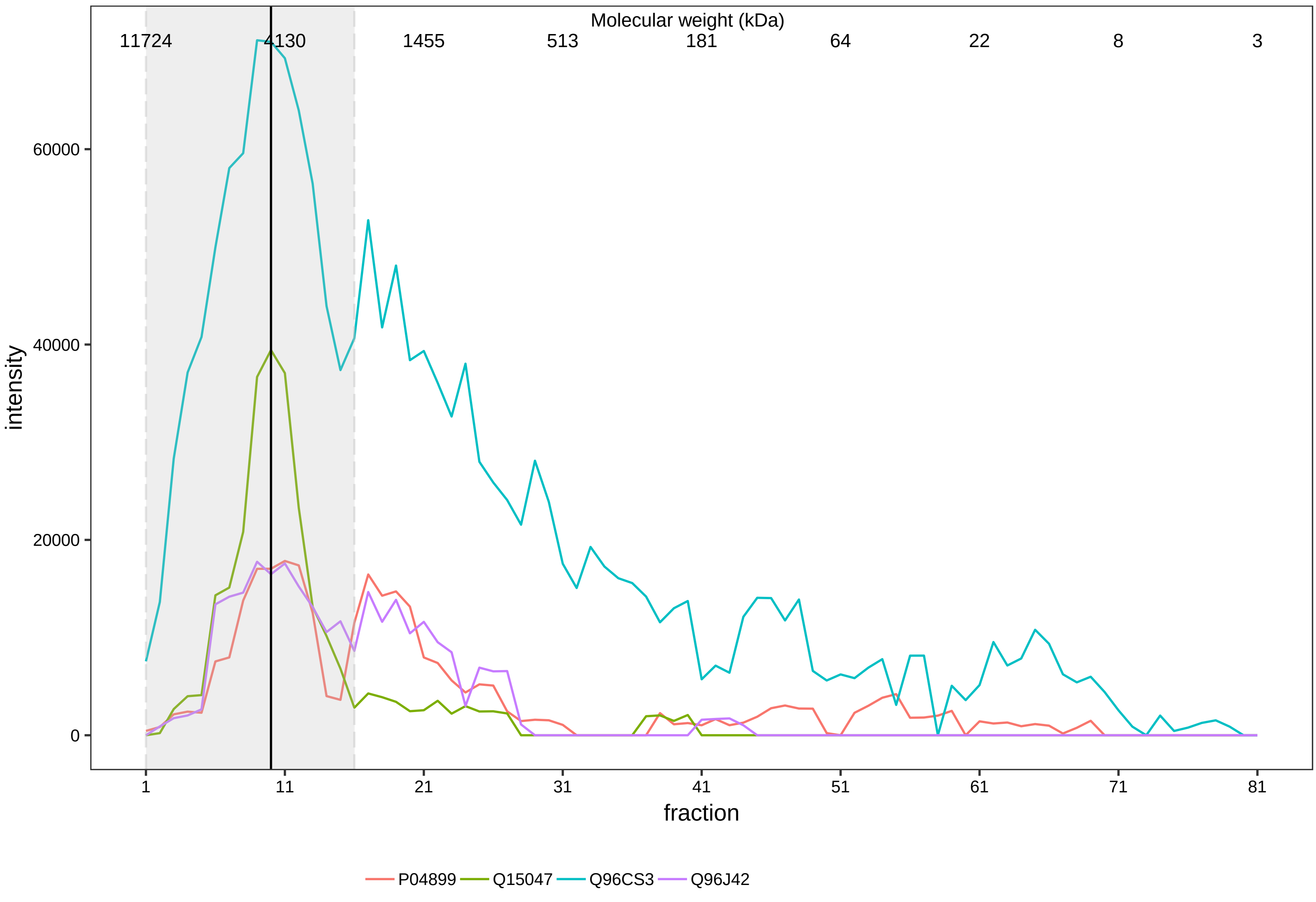

Feature ID 125

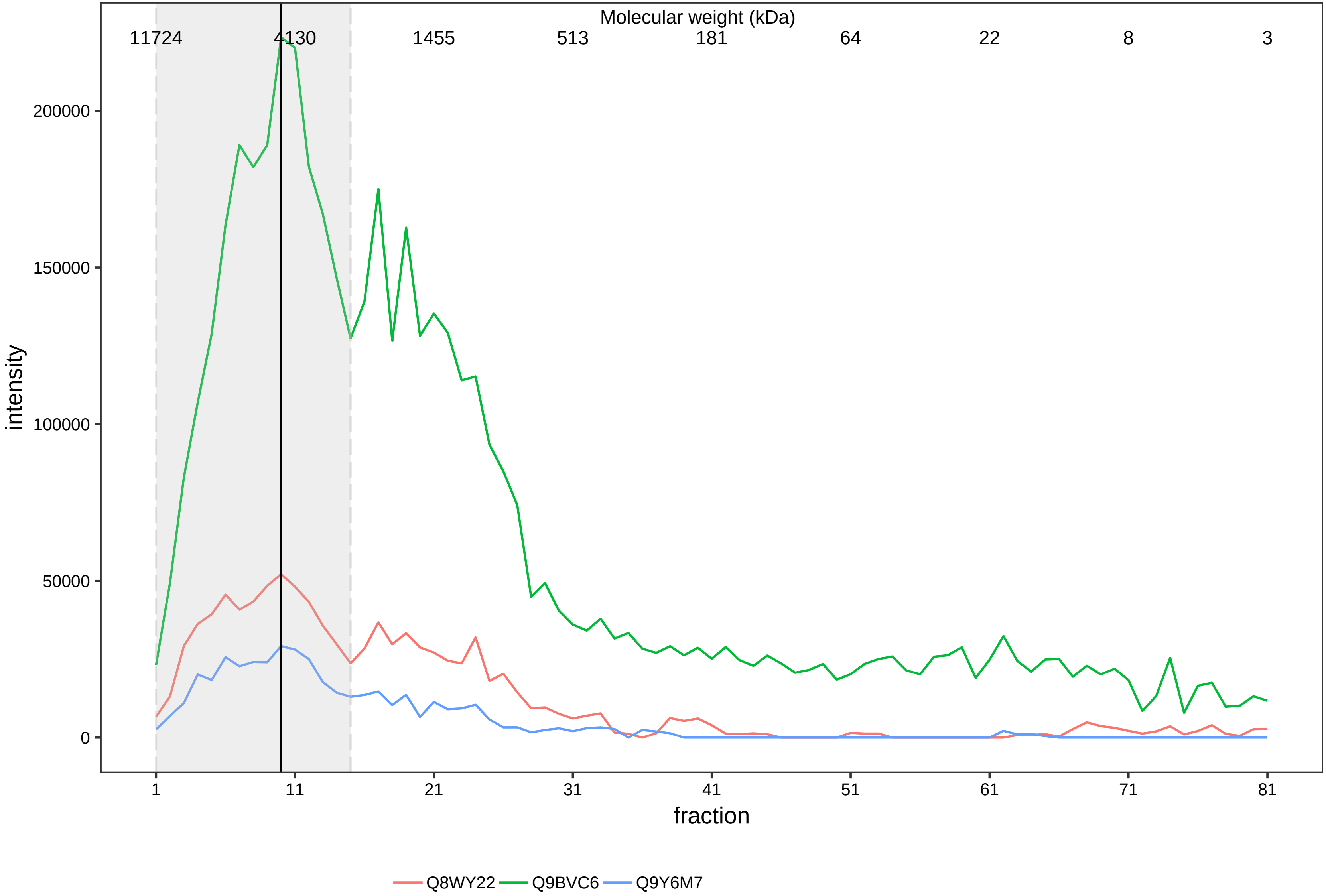

Feature ID 126

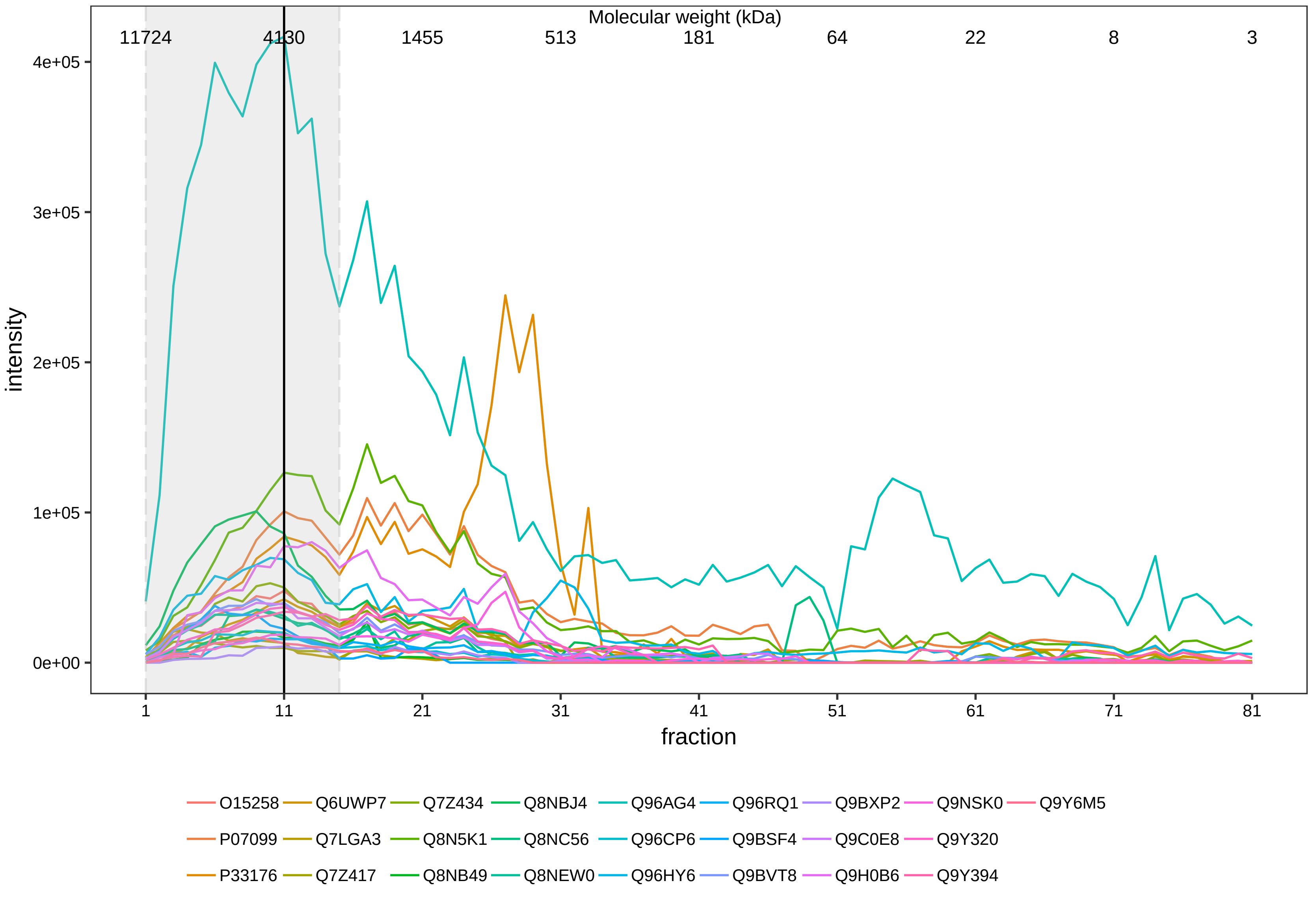

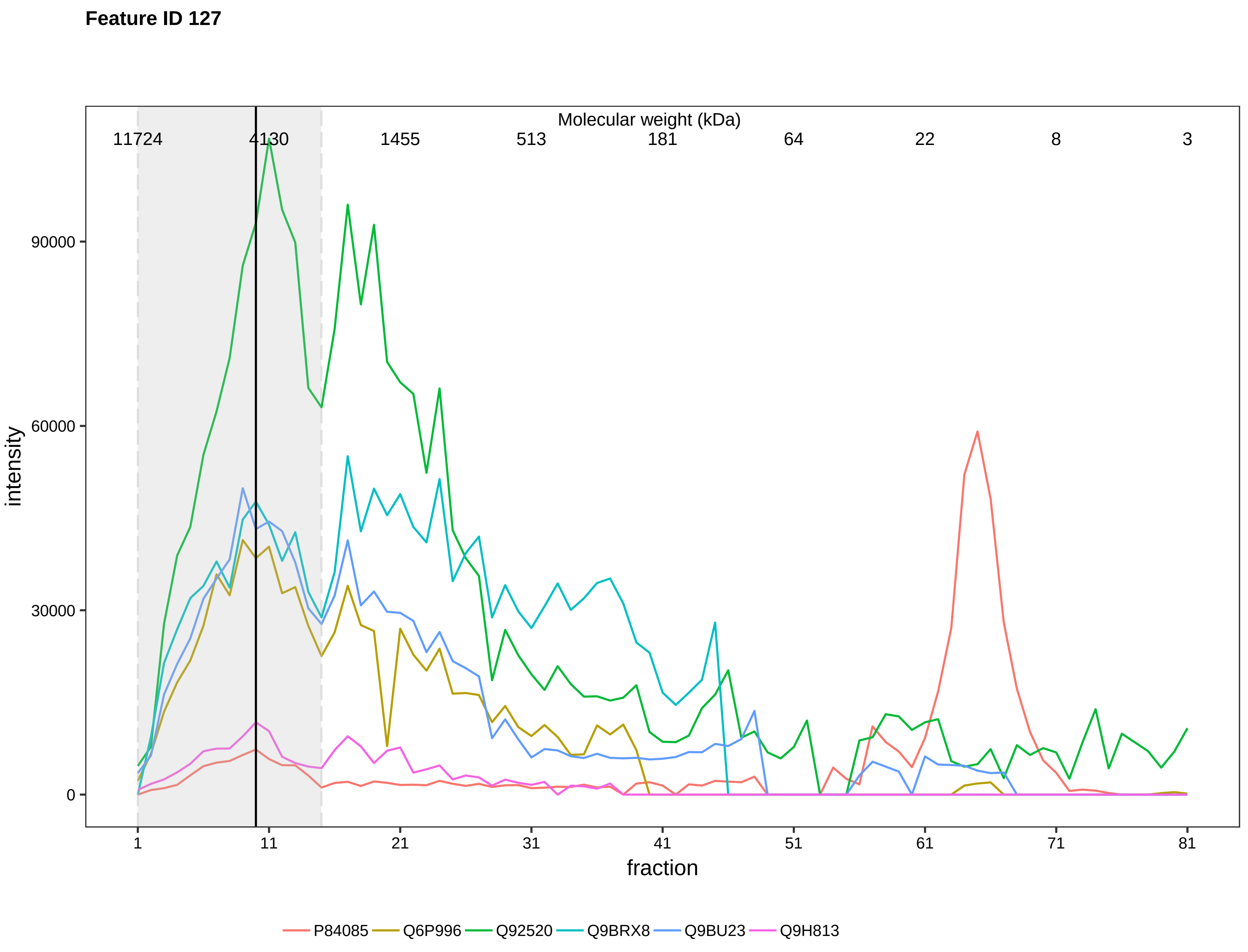

Feature ID 127
Molecular weight (kDa)
11724
4130
1455
513
181
64
22
8
3
90000
60000
30000
0
intensity
1
11
21
31
41
51
61
71
81
fraction
P84085
Q6P996
Q92520
Q9BRX8
Q9BU23
Q9H813

Feature ID 128

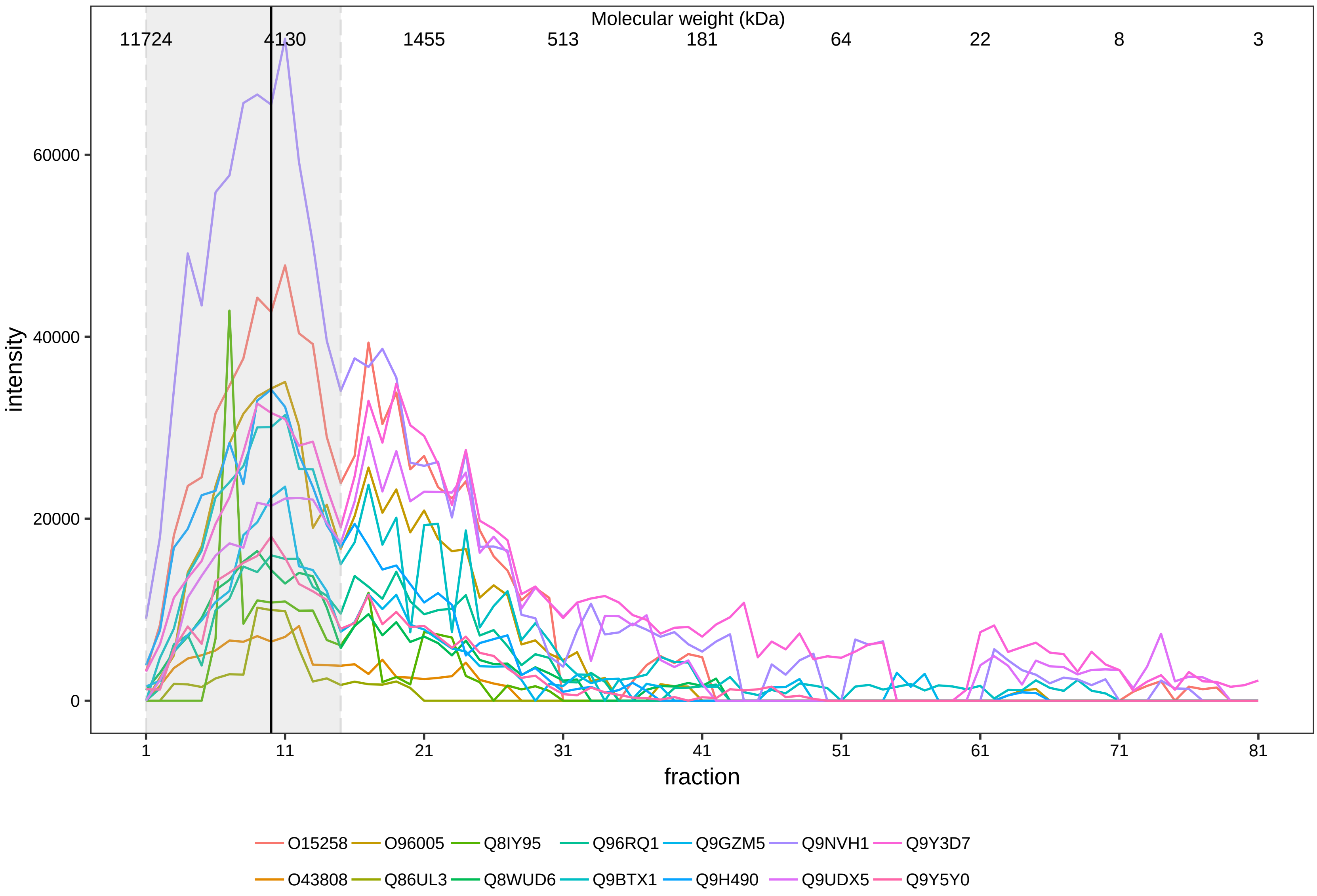

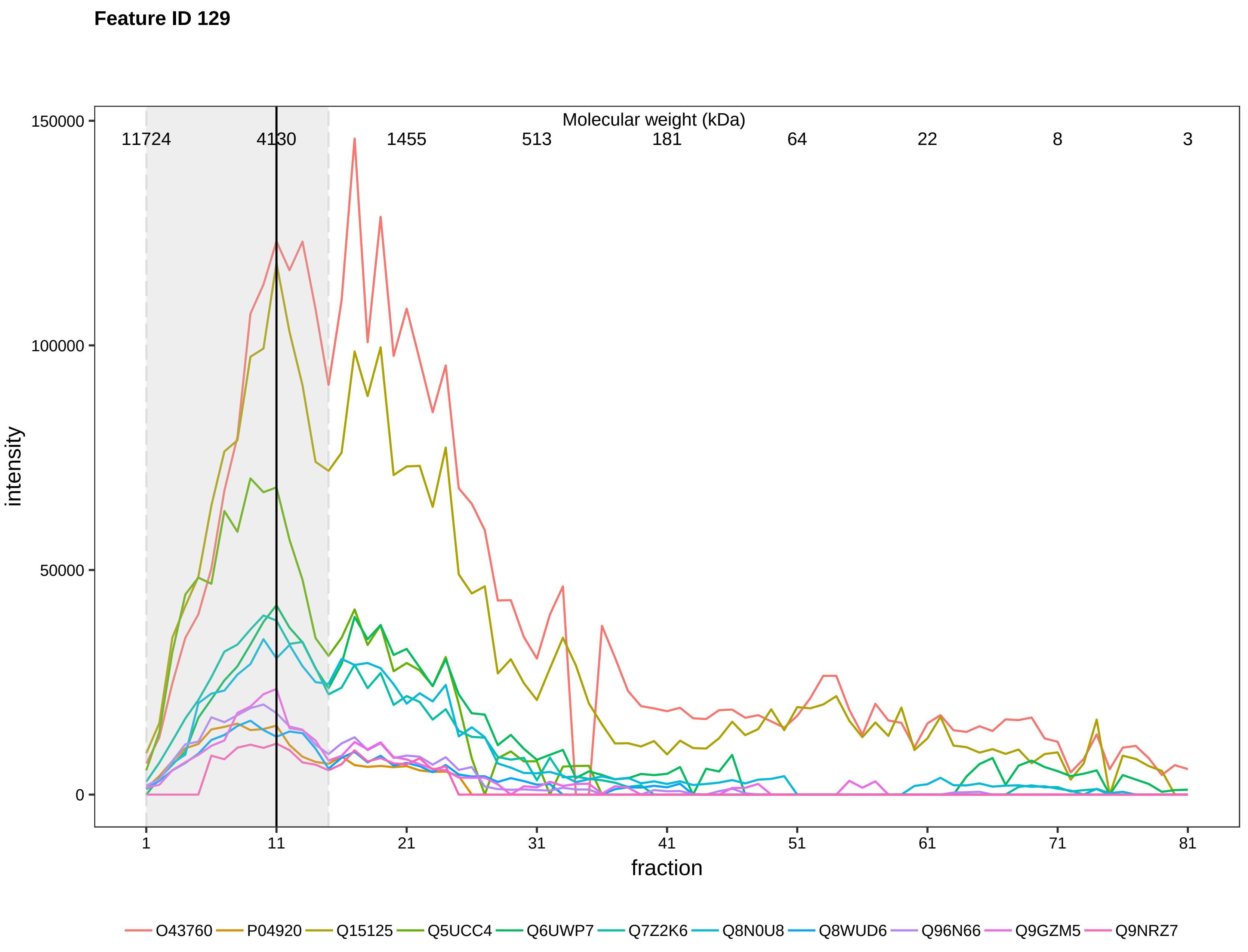
Feature ID 129
Molecular weight (kDa)
11724
4130
1455
513
181
64
22
8
3
150000
100000
50000
0
intensity
1
11
21
31
41
51
61
71
81
fraction
O43760
P04920
Q15125
Q5UCC4
Q6UWP7
Q7Z2K6
Q8N0U8
Q8WUD6
Q96N66
Q9GZM5
Q9NRZ7

Feature ID 130

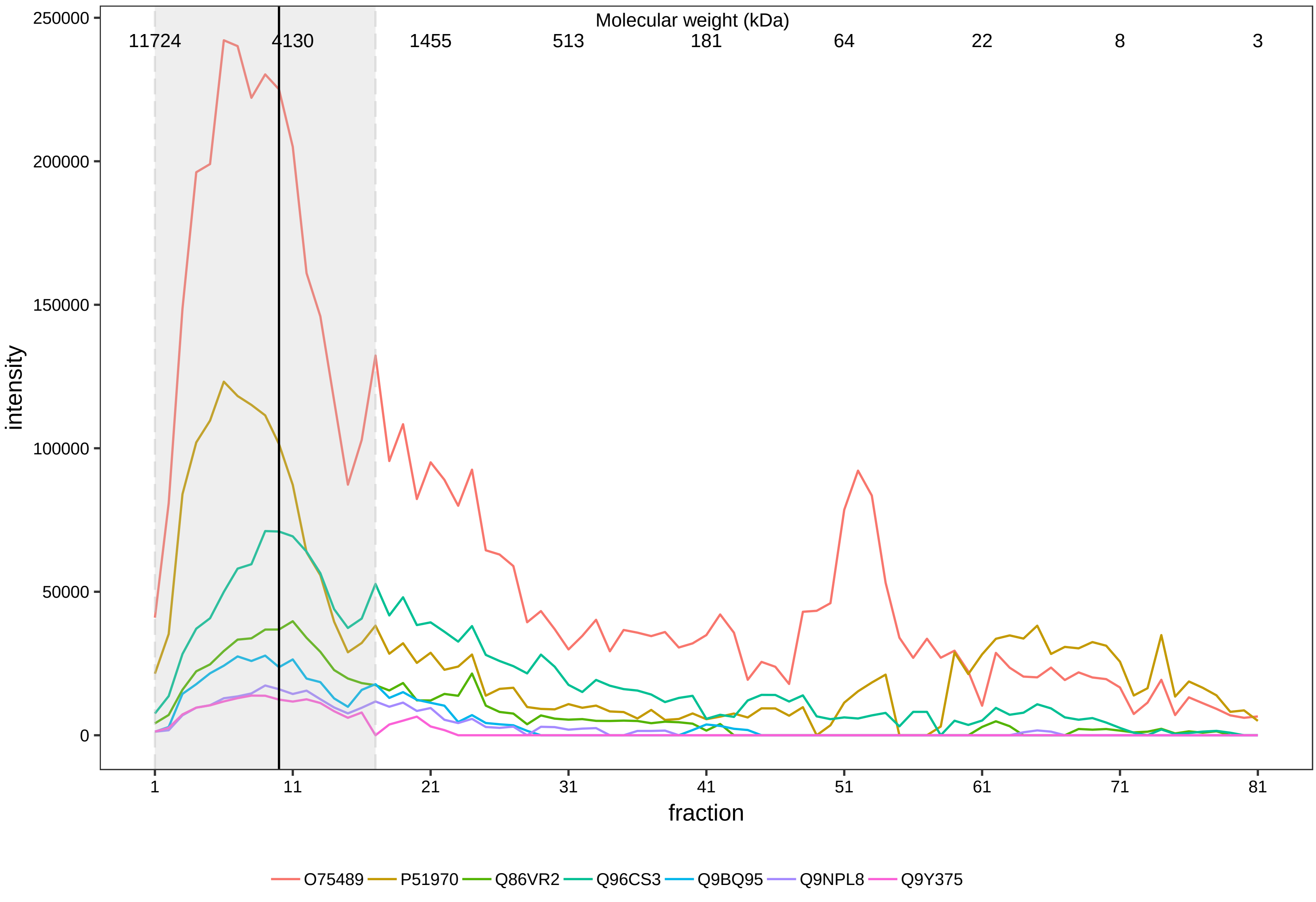

Feature ID 131

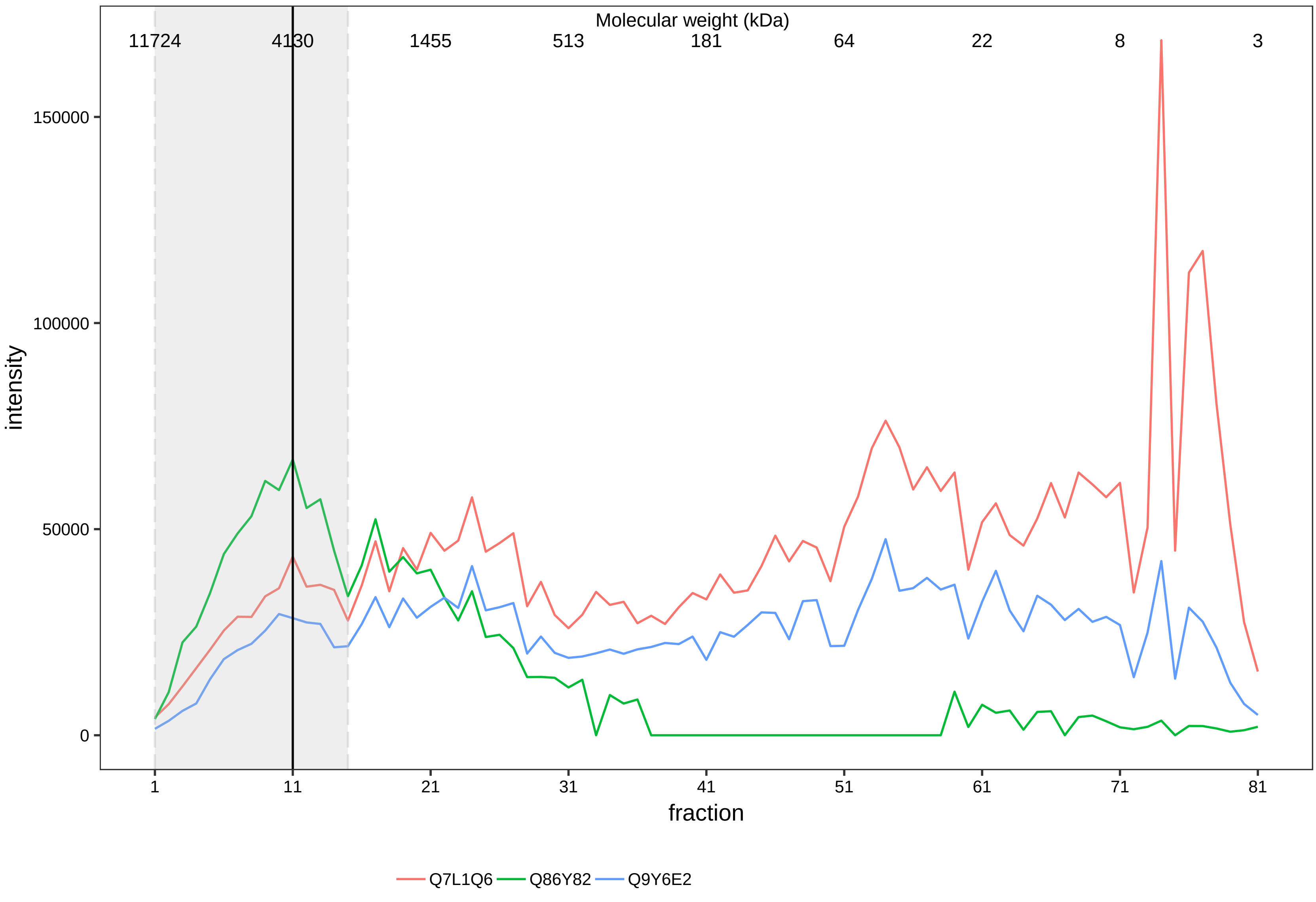

Feature ID 132

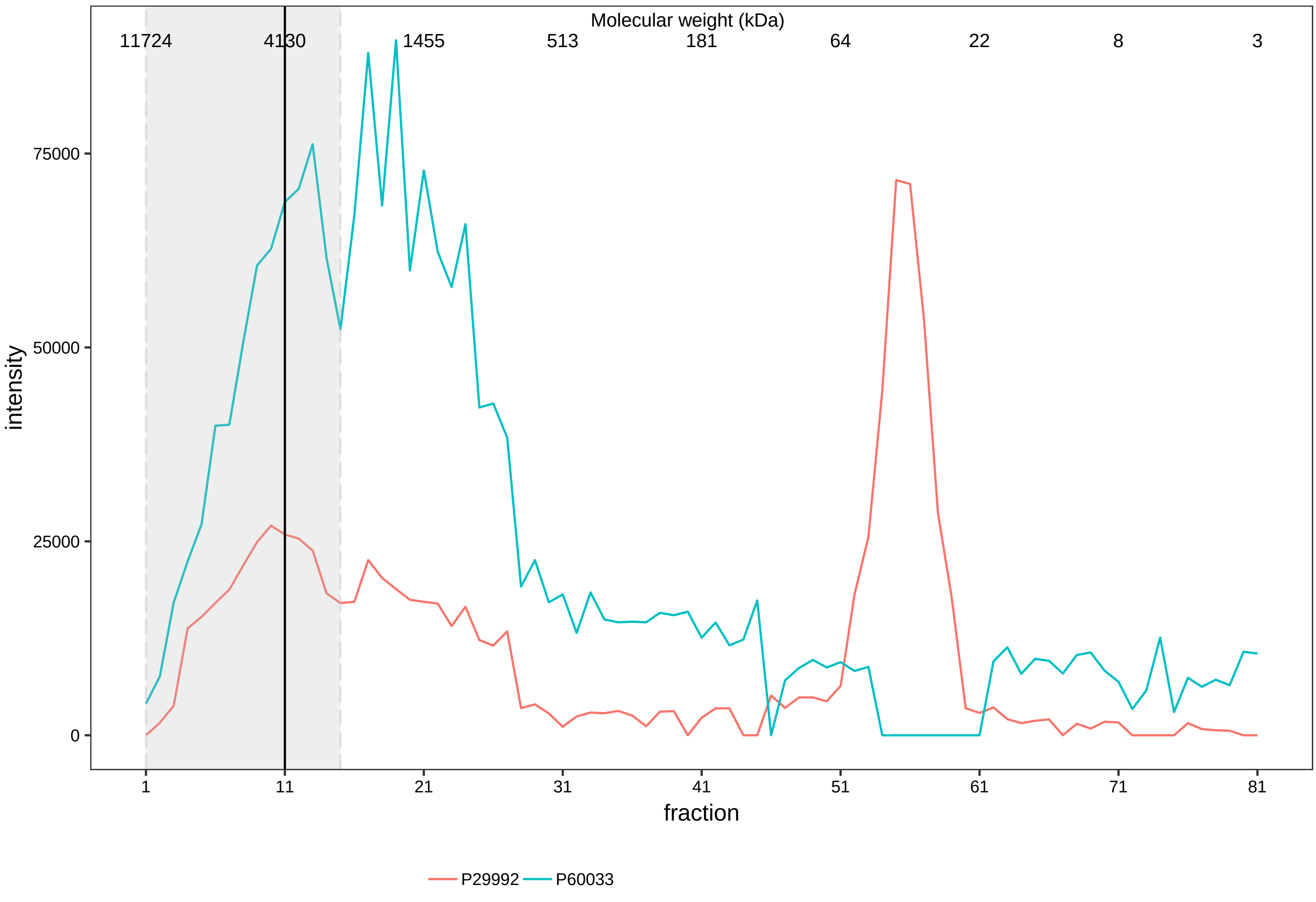

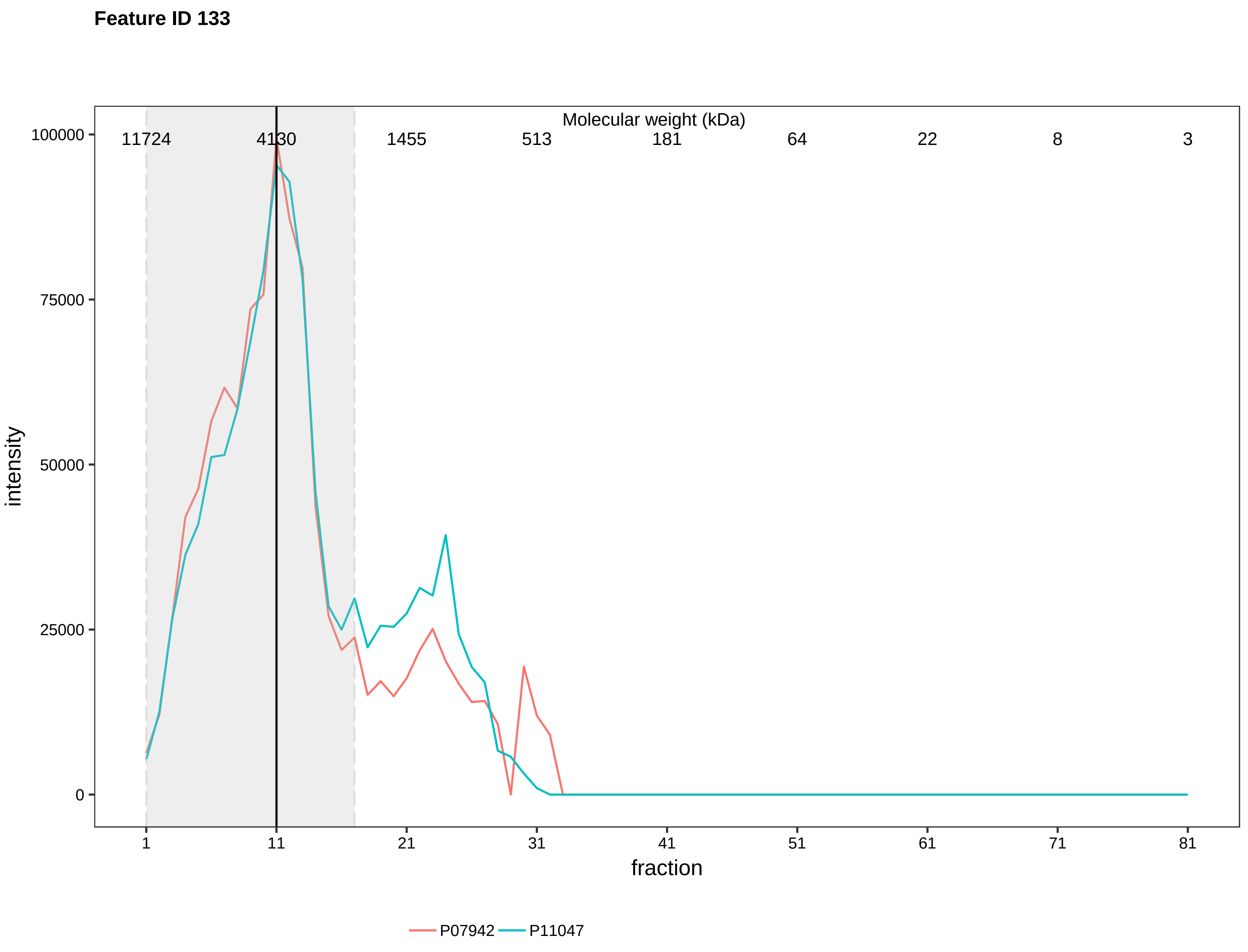

Feature ID 133
Molecular weight (kDa)
11724
4130
1455
513
181
64
22
8
3
100000
75000
50000
25000
0
intensity
1
11
21
31
41
51
61
71
81
fraction
P07942
P11047

Feature ID 134

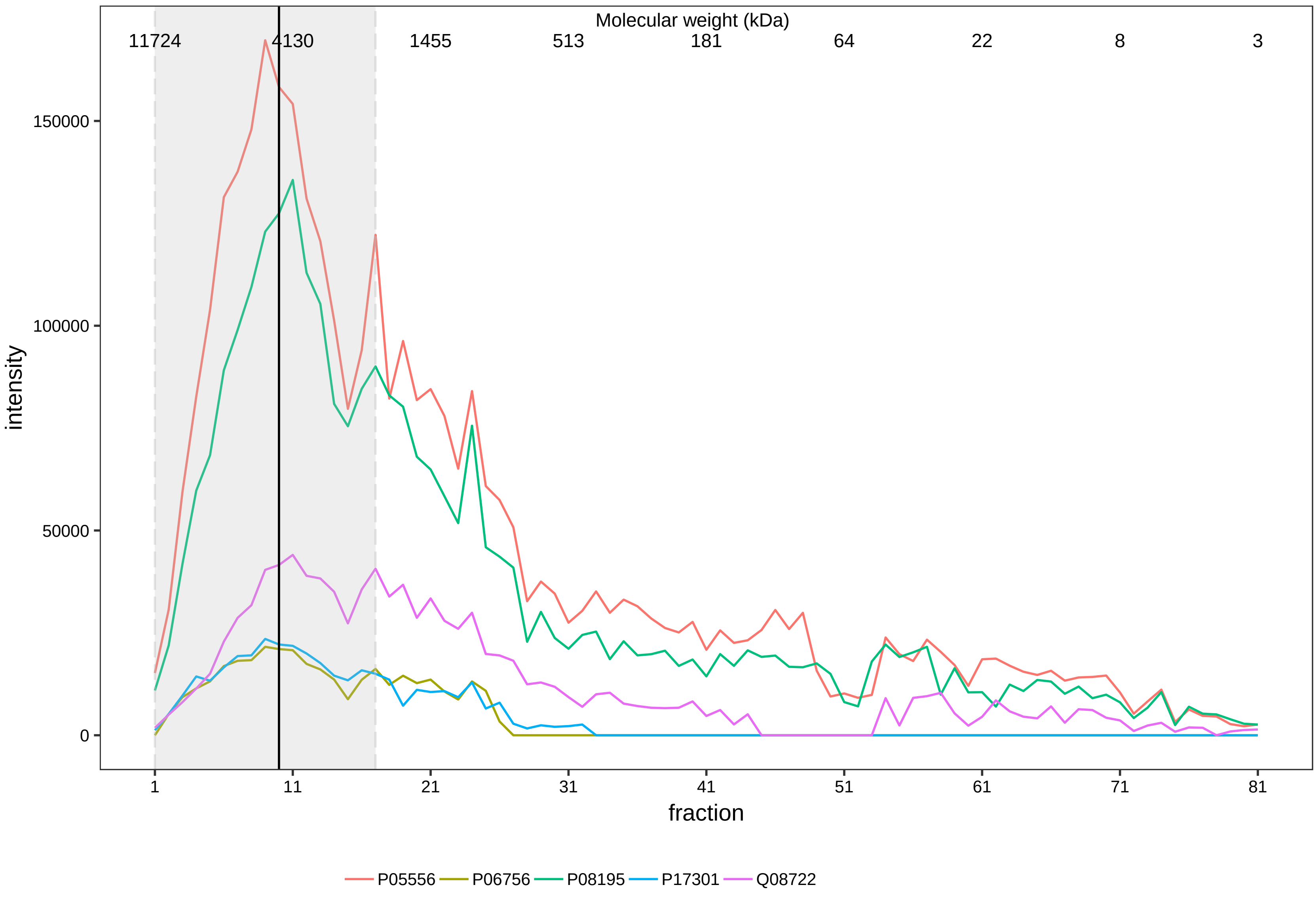

Feature ID 135

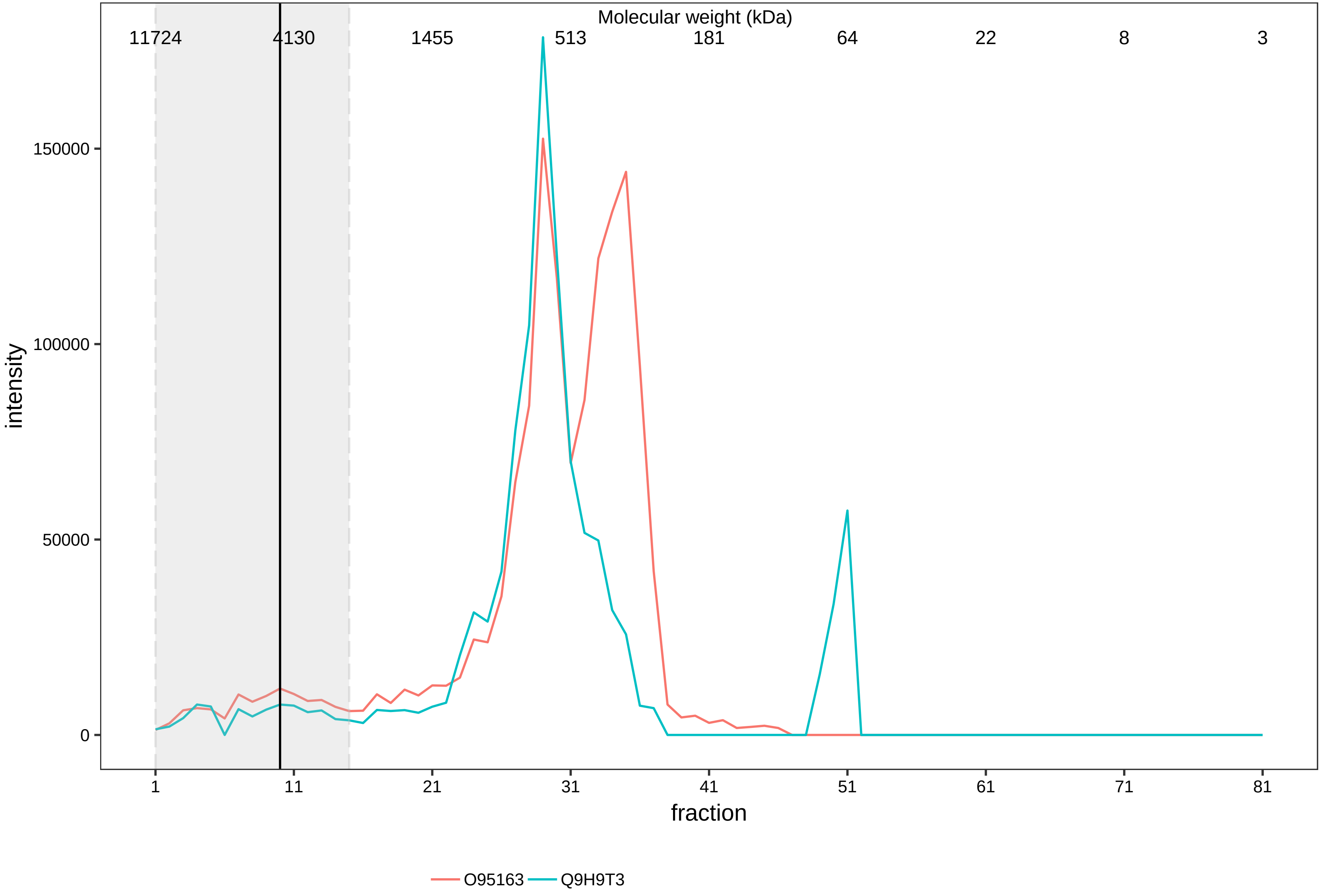

Feature ID 136

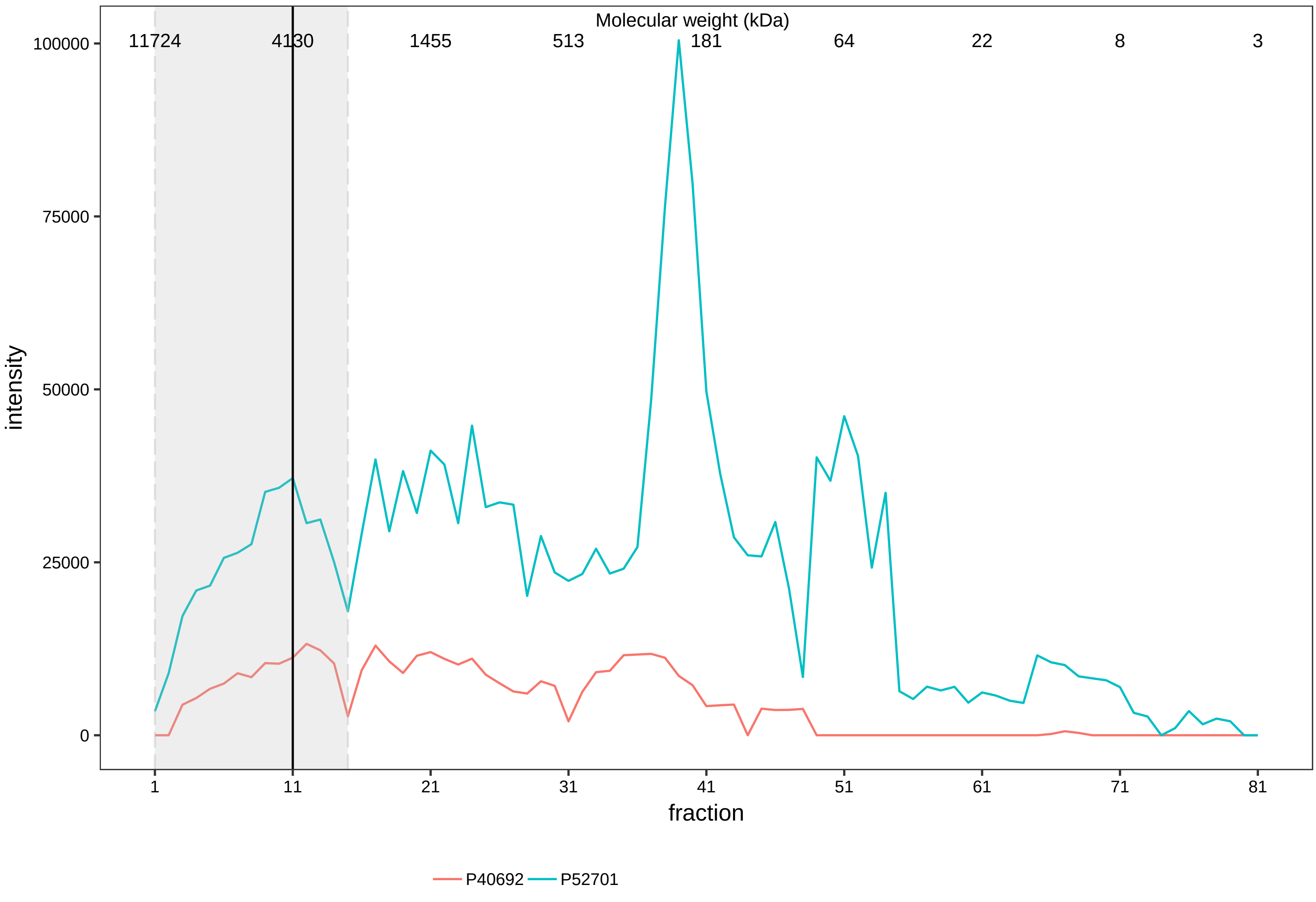

Feature ID 137

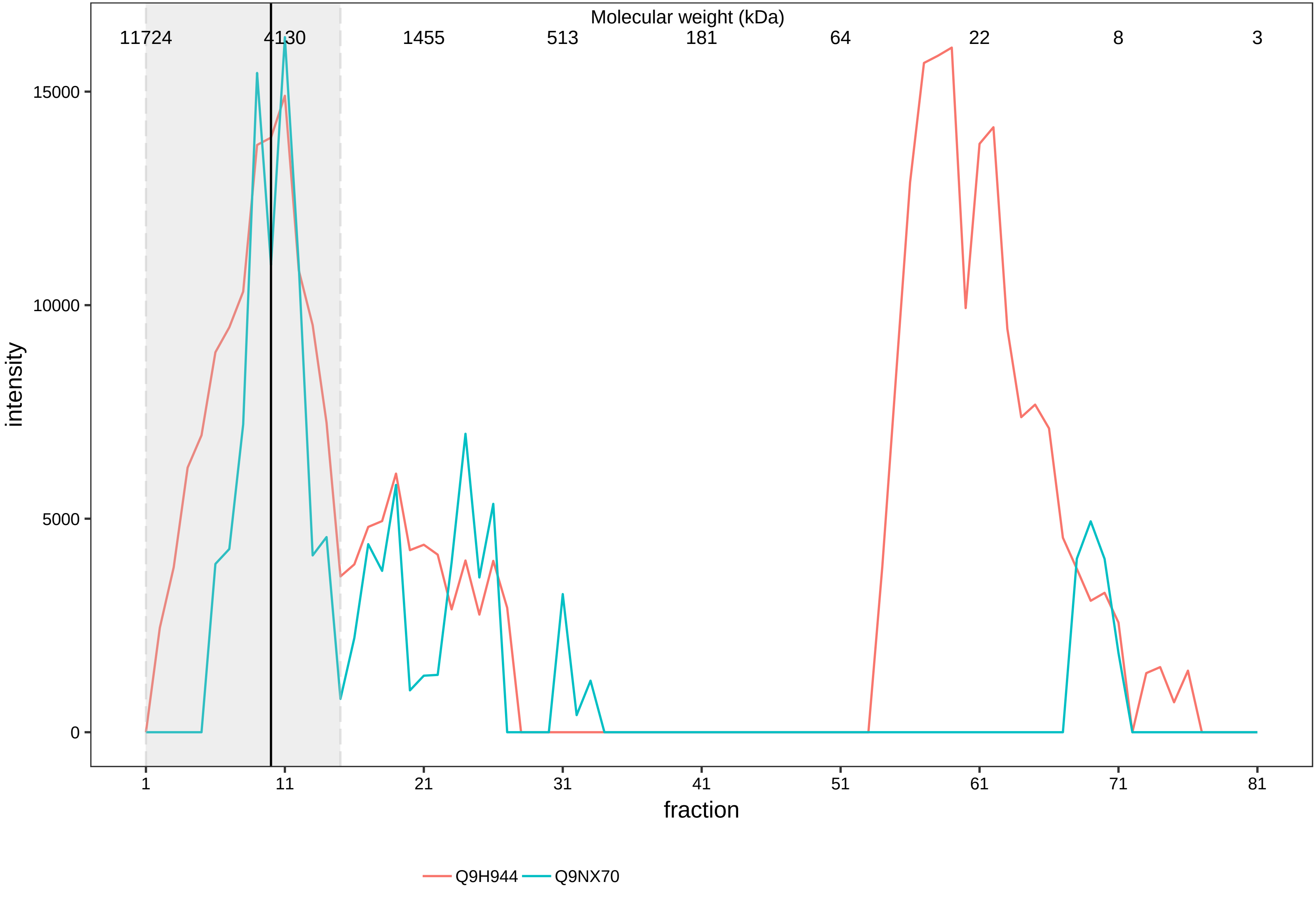

Feature ID 138

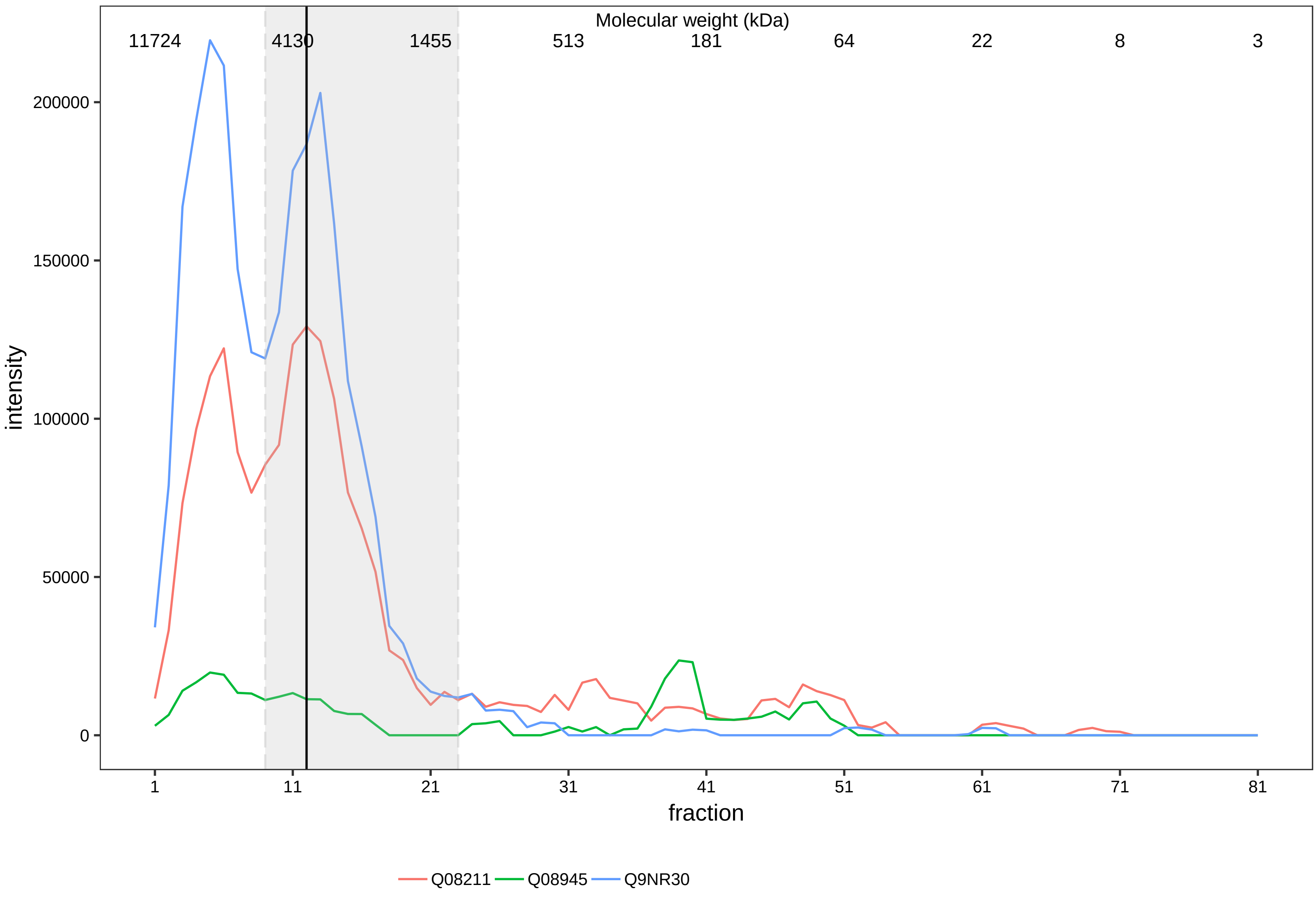

Feature ID 139

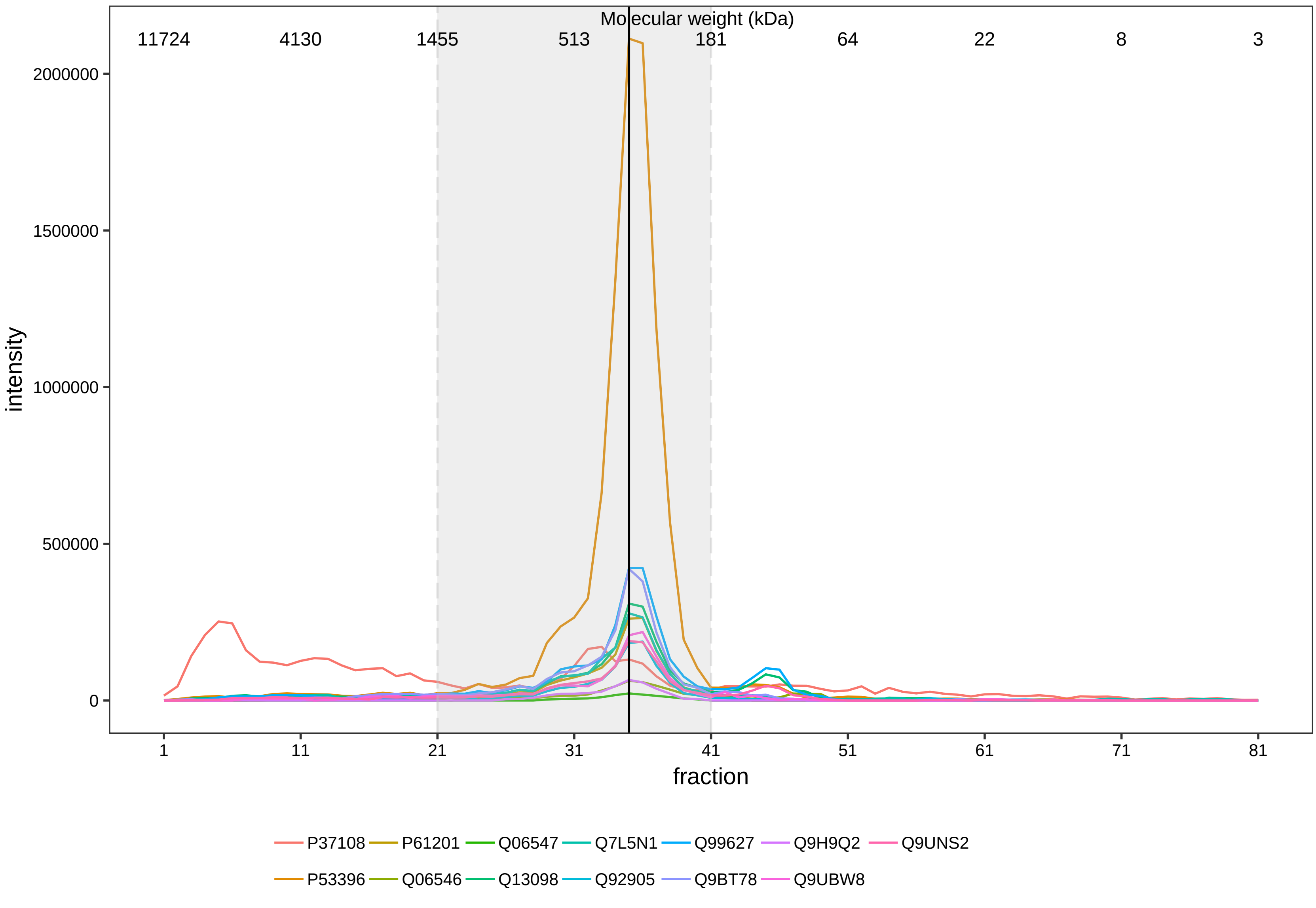

Feature ID 140

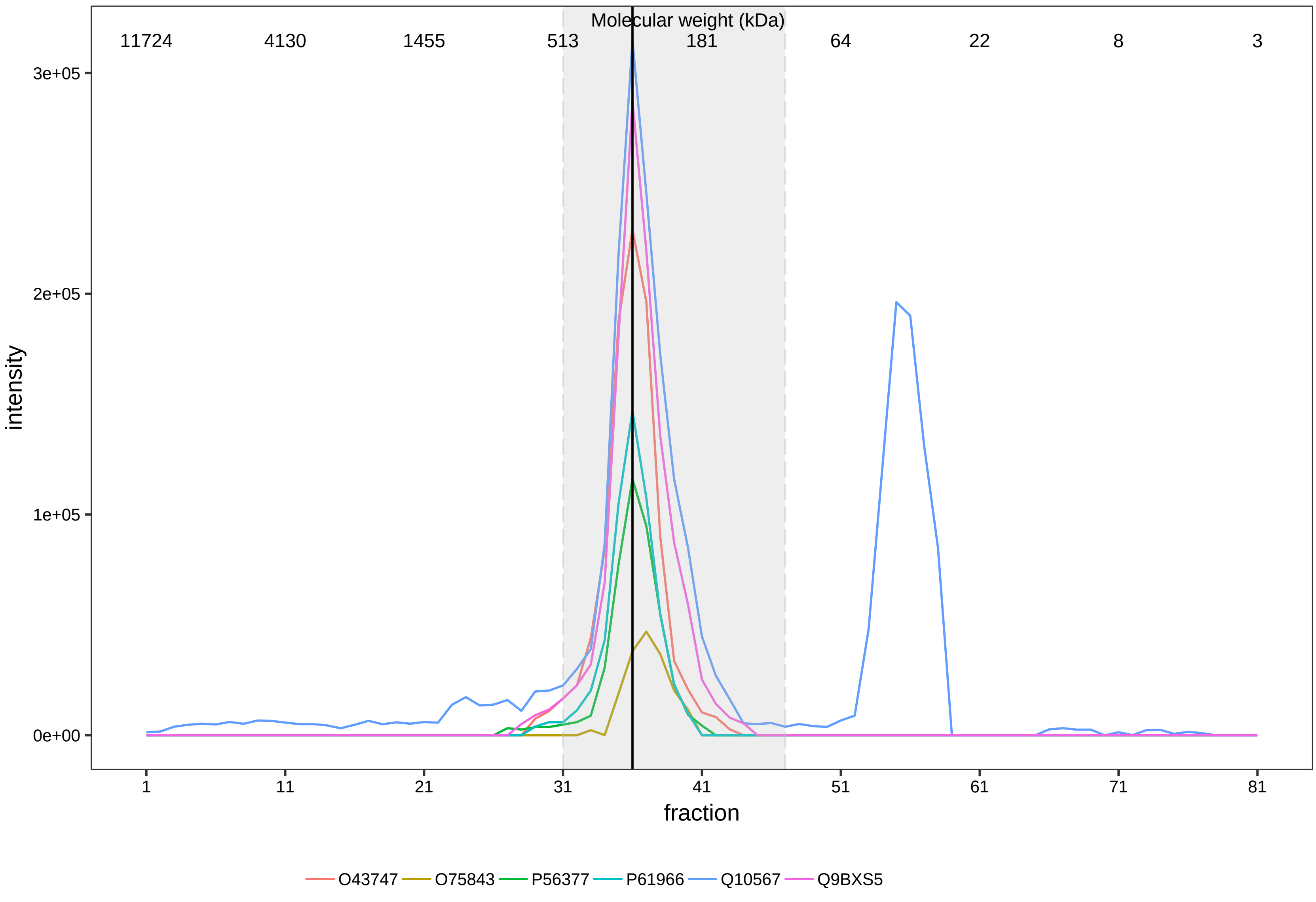

Feature ID 141

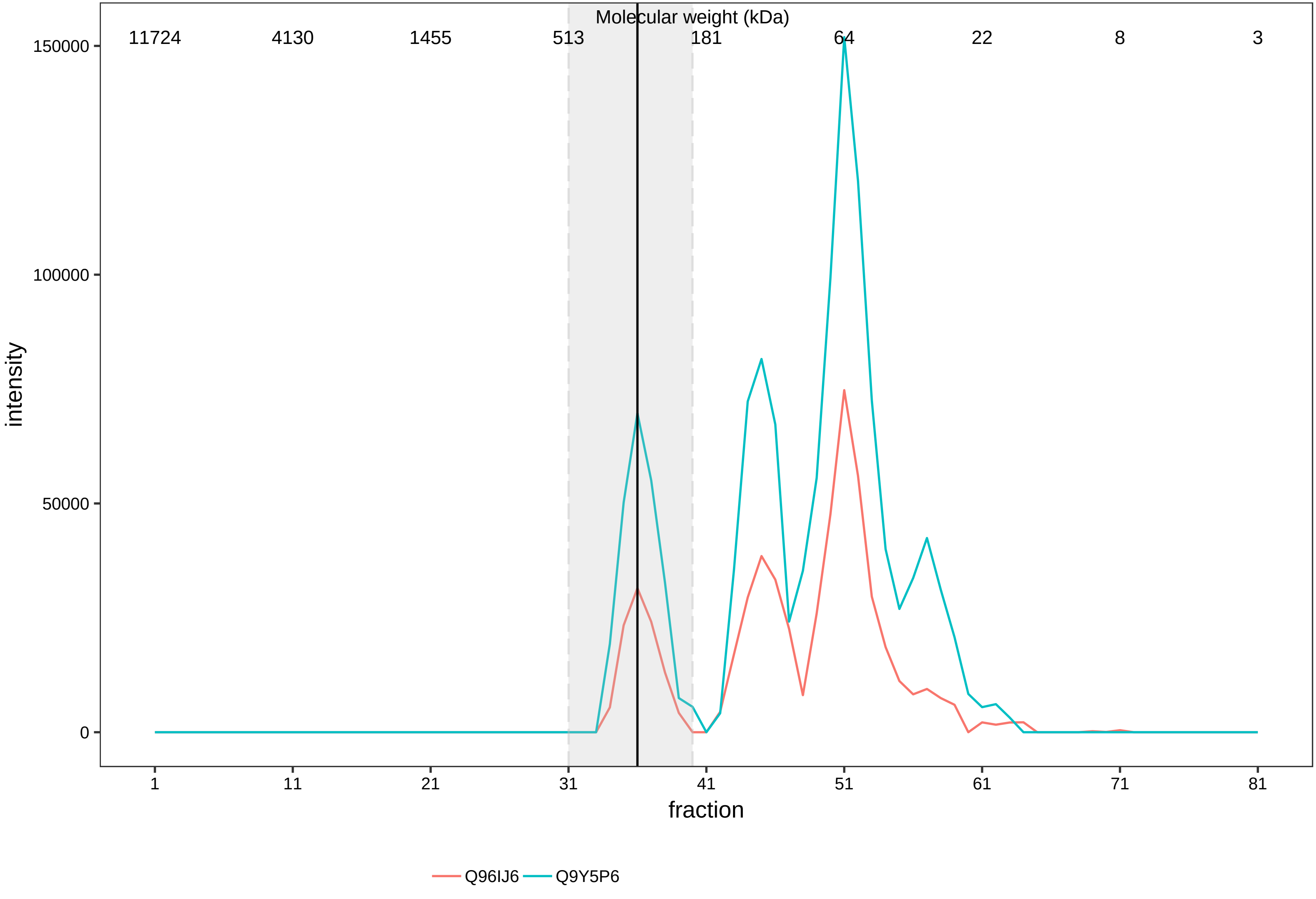

Feature ID 142

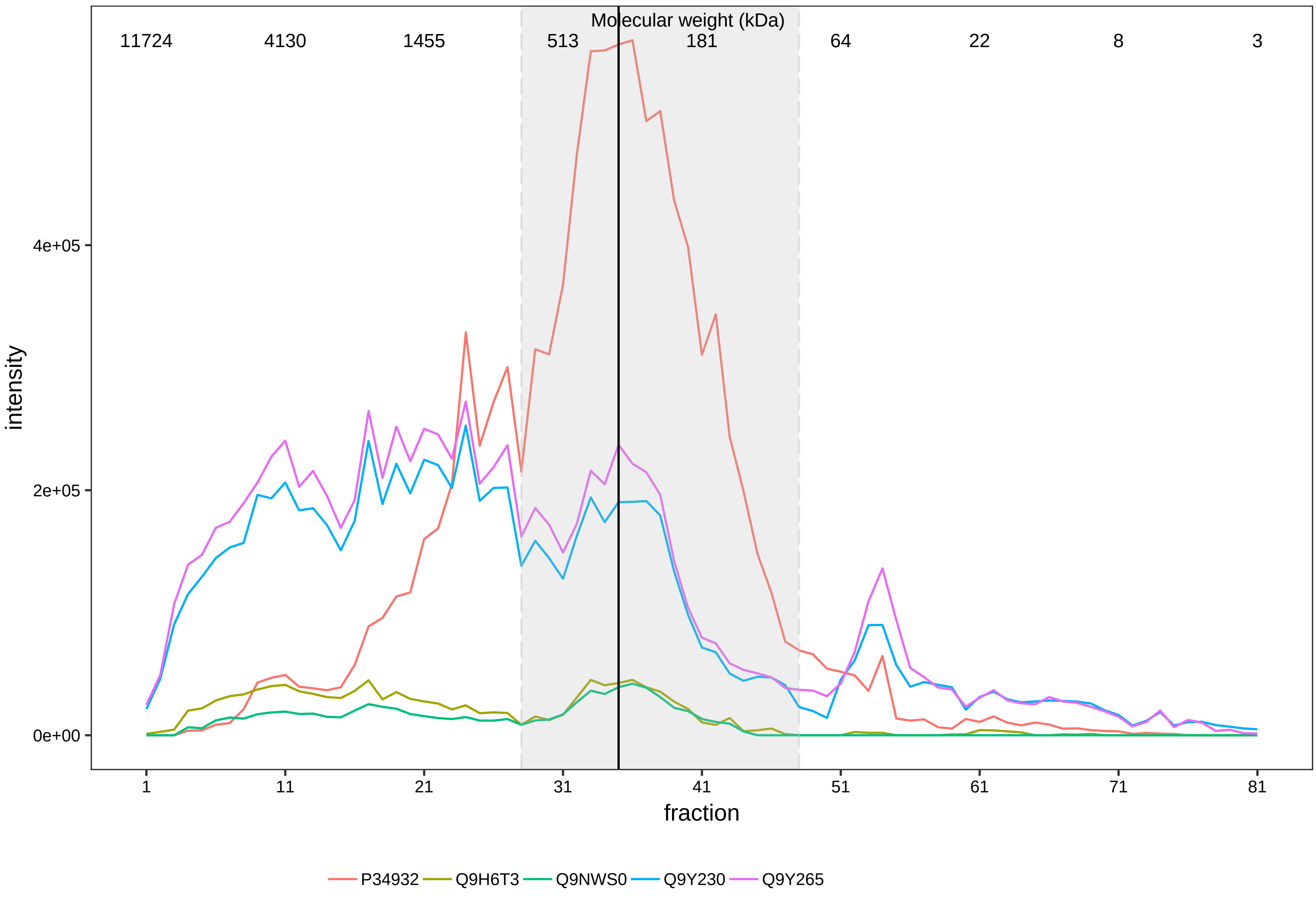

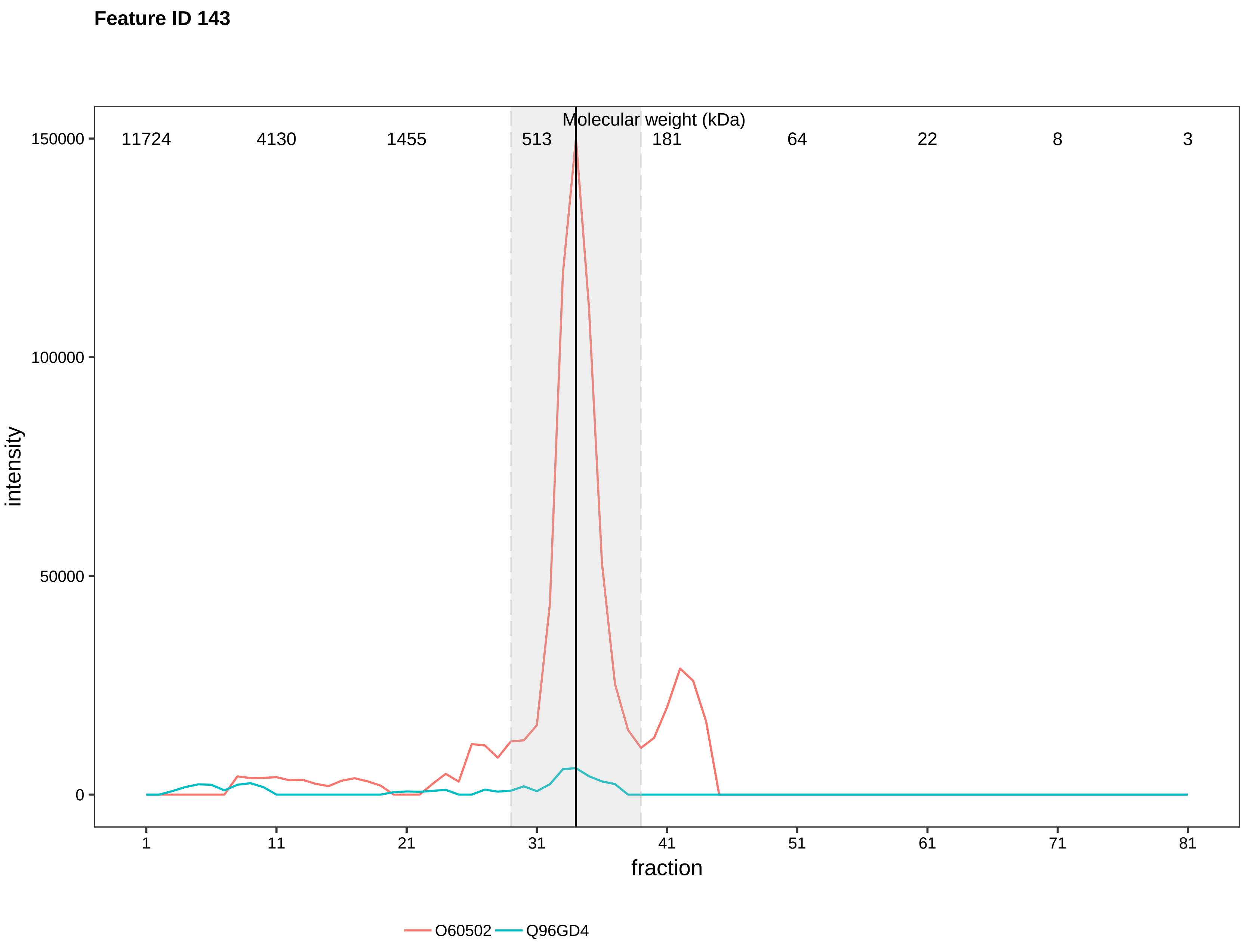
Feature ID 143
Molecular weight (kDa)
11724
4130
1455
513
181
64
22
8
3
150000
100000
50000
0
intensity
1
11
21
31
41
51
61
71
81
fraction
O60502
Q96GD4

Feature ID 144

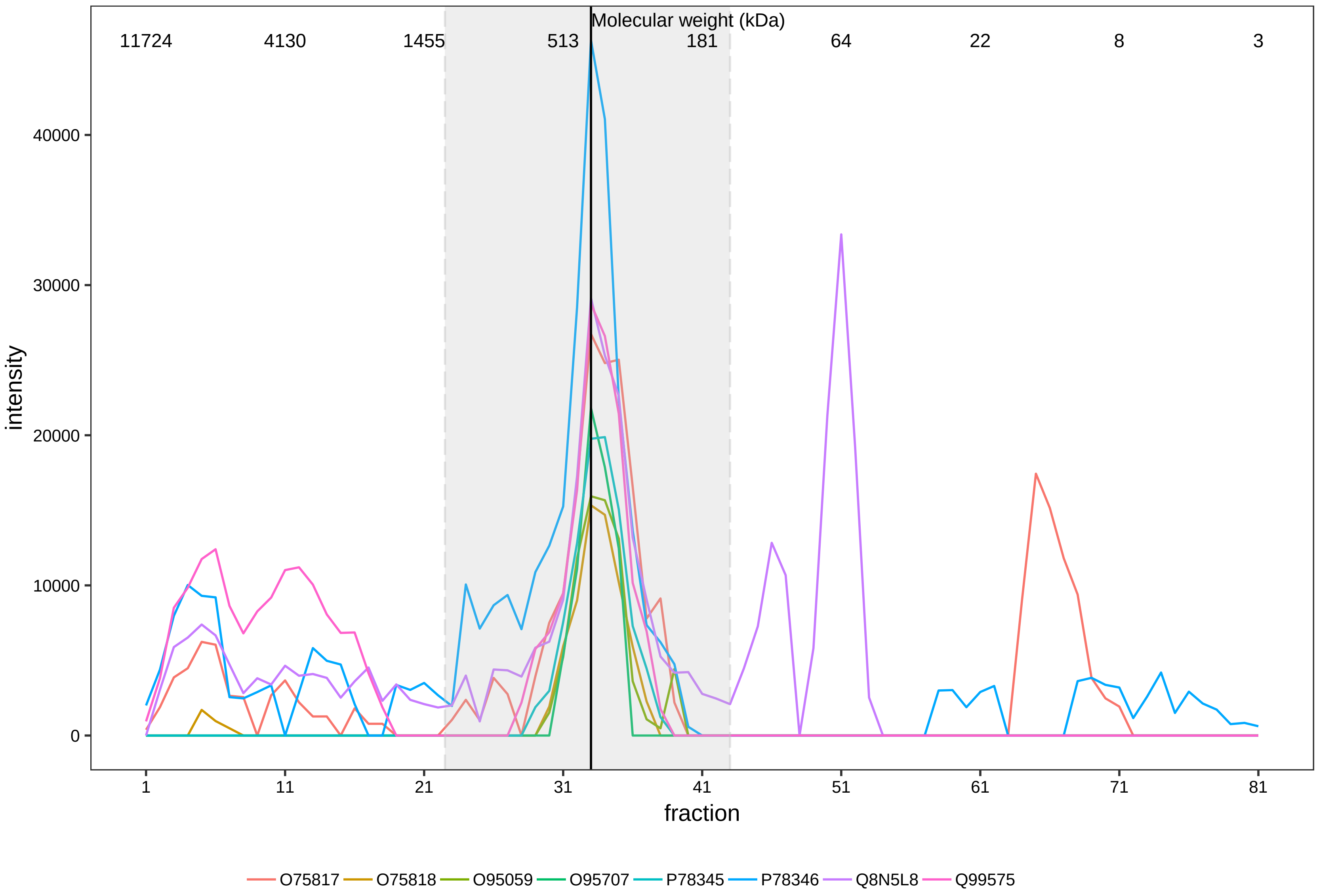

Feature ID 145

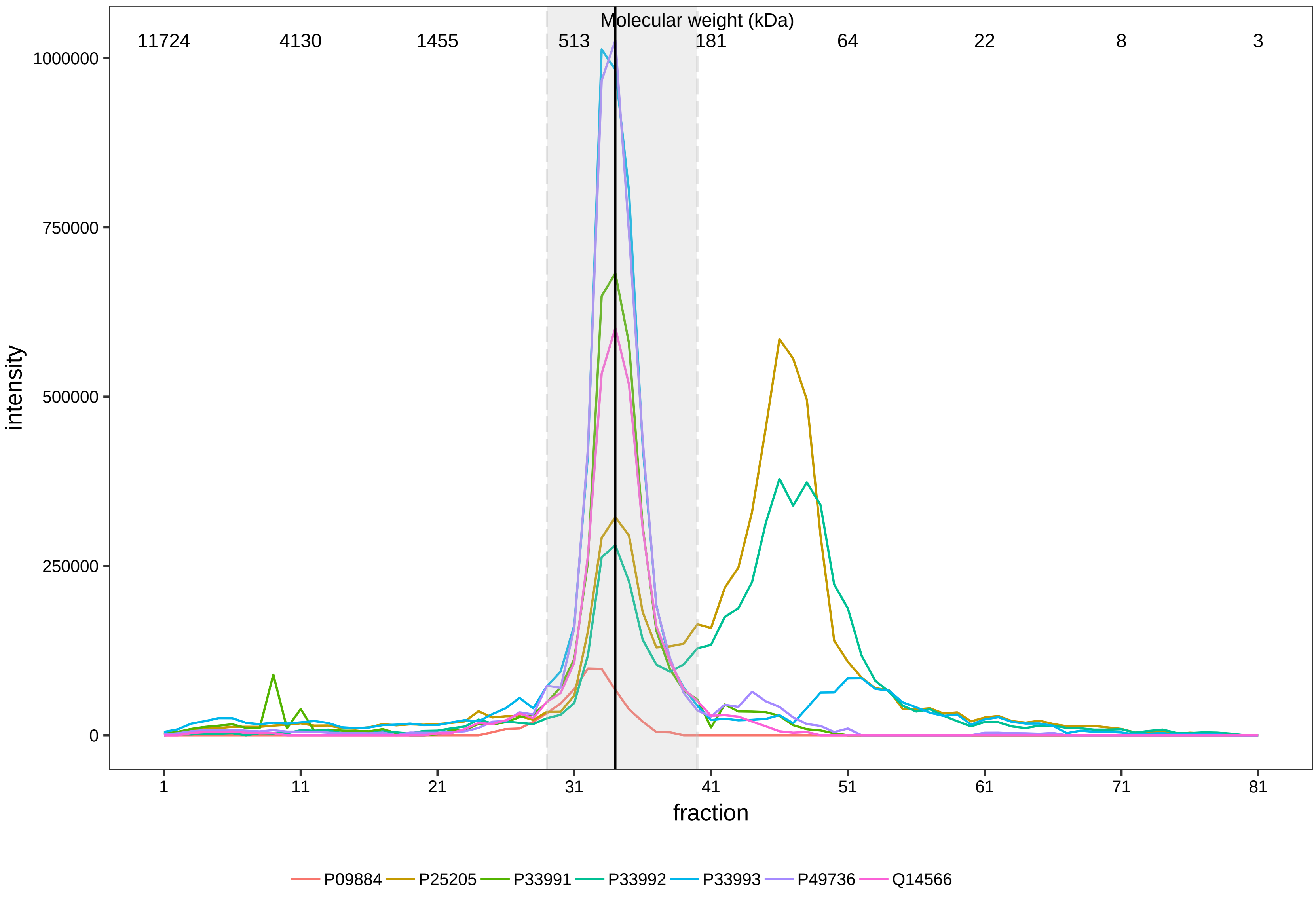

Feature ID 146

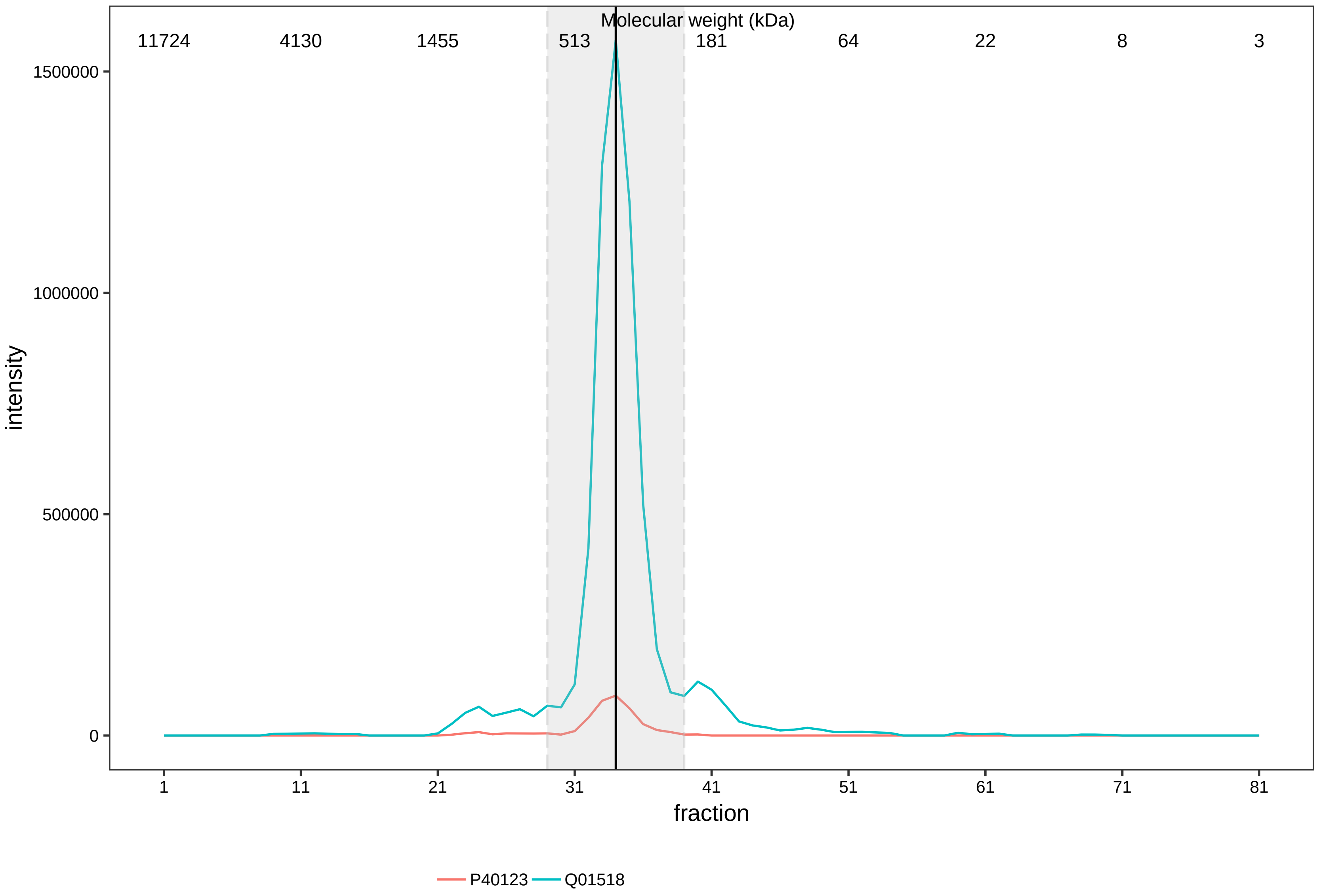

Feature ID 147

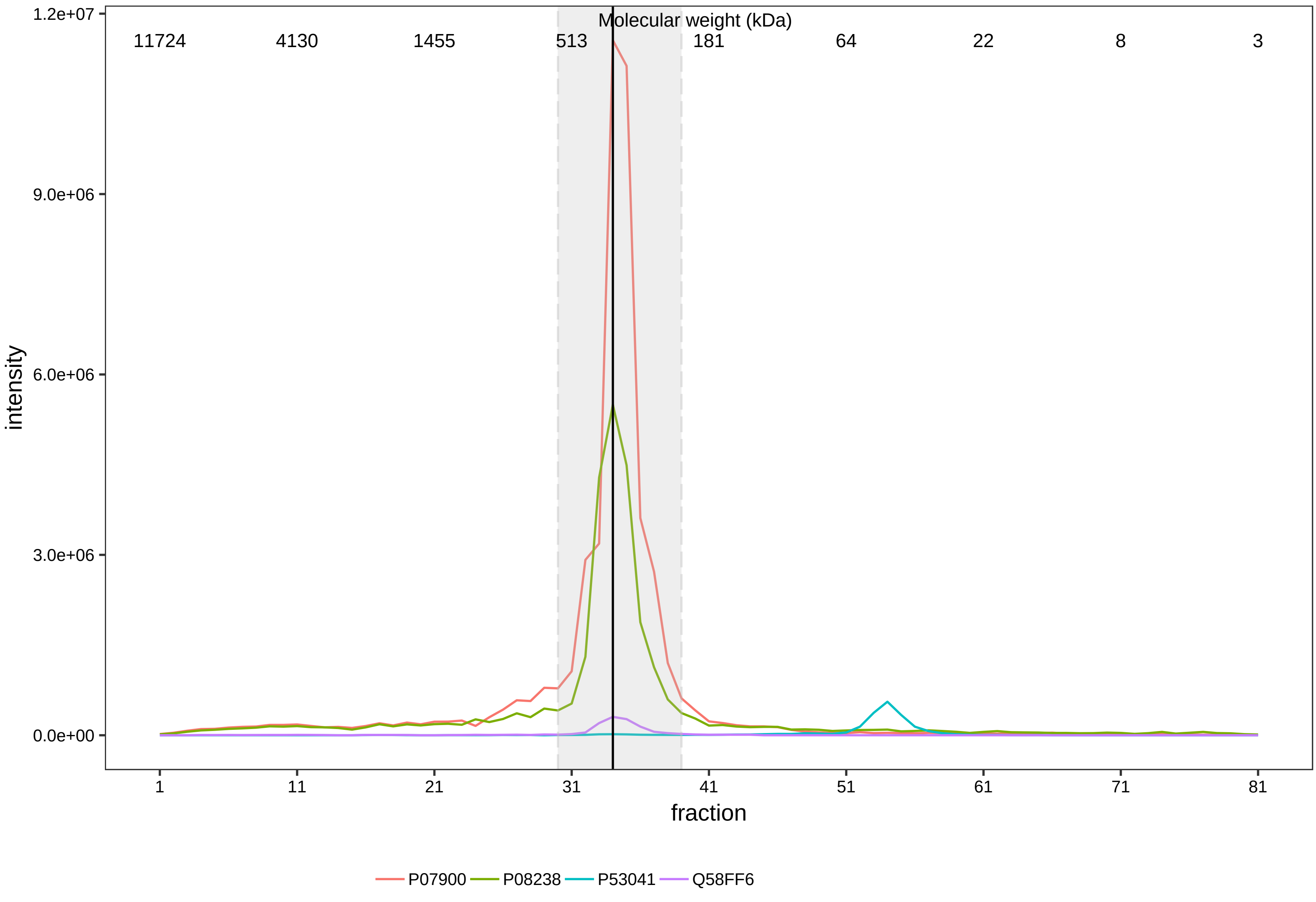

Feature ID 148

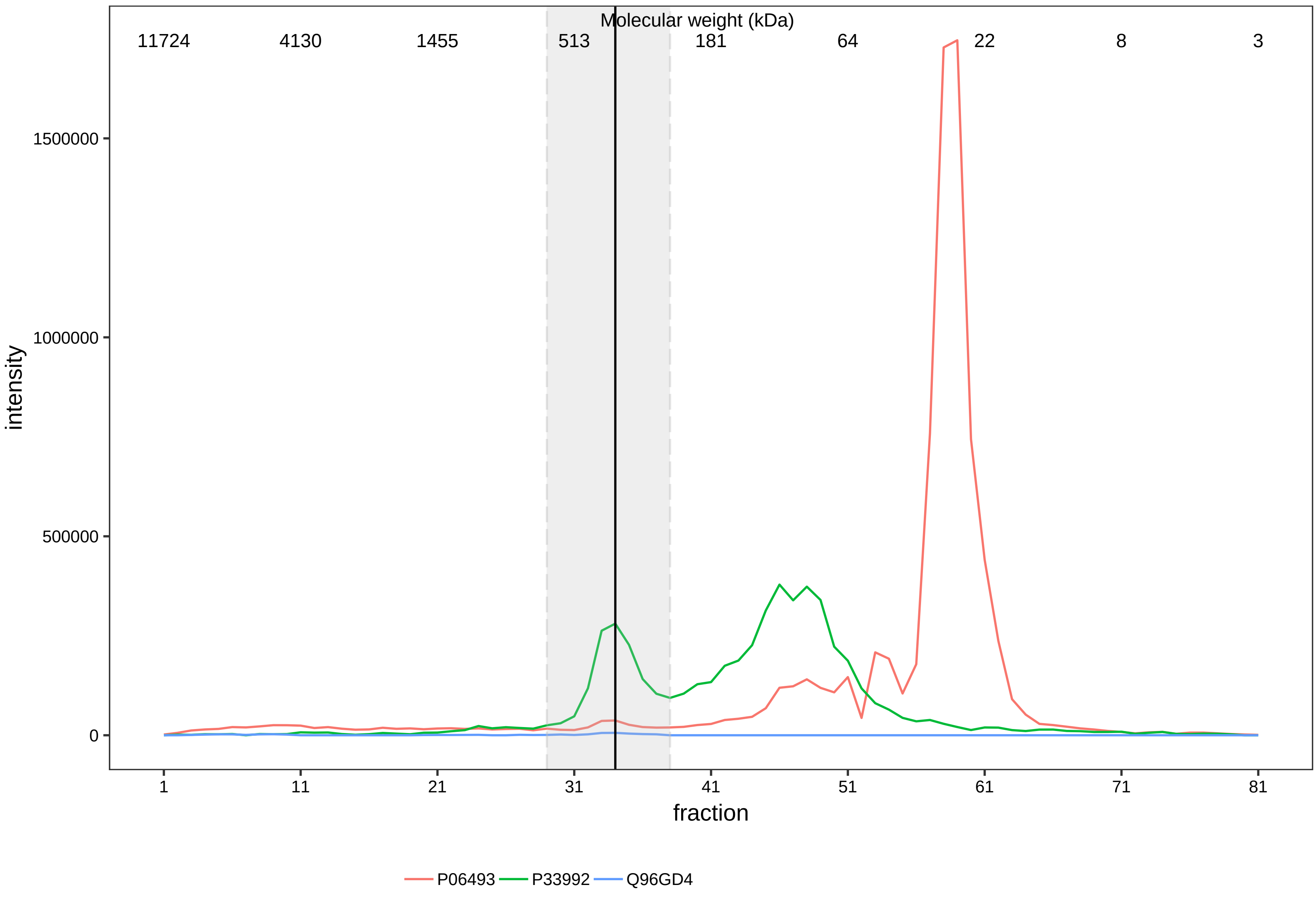

Feature ID 149

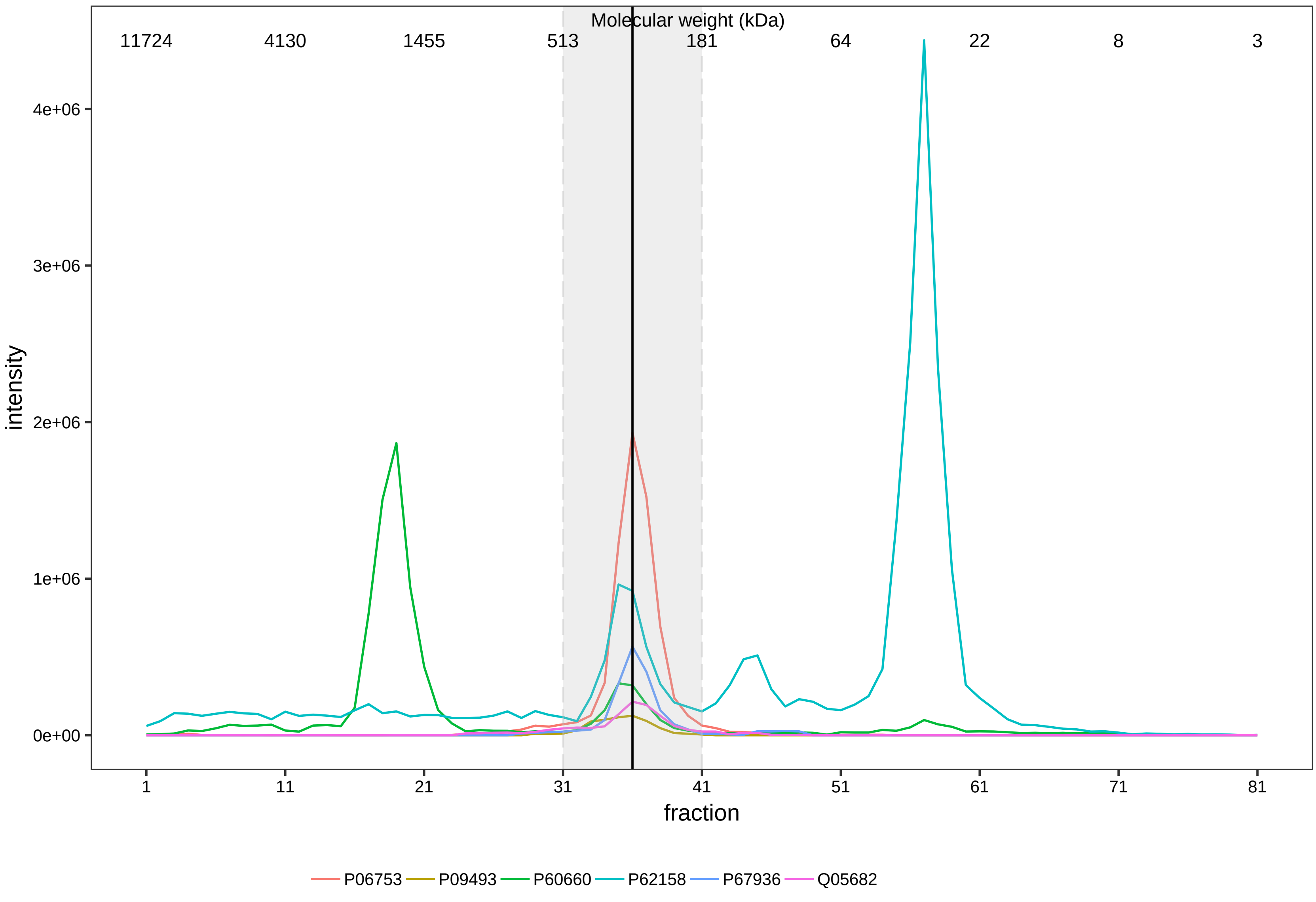

Feature ID 150

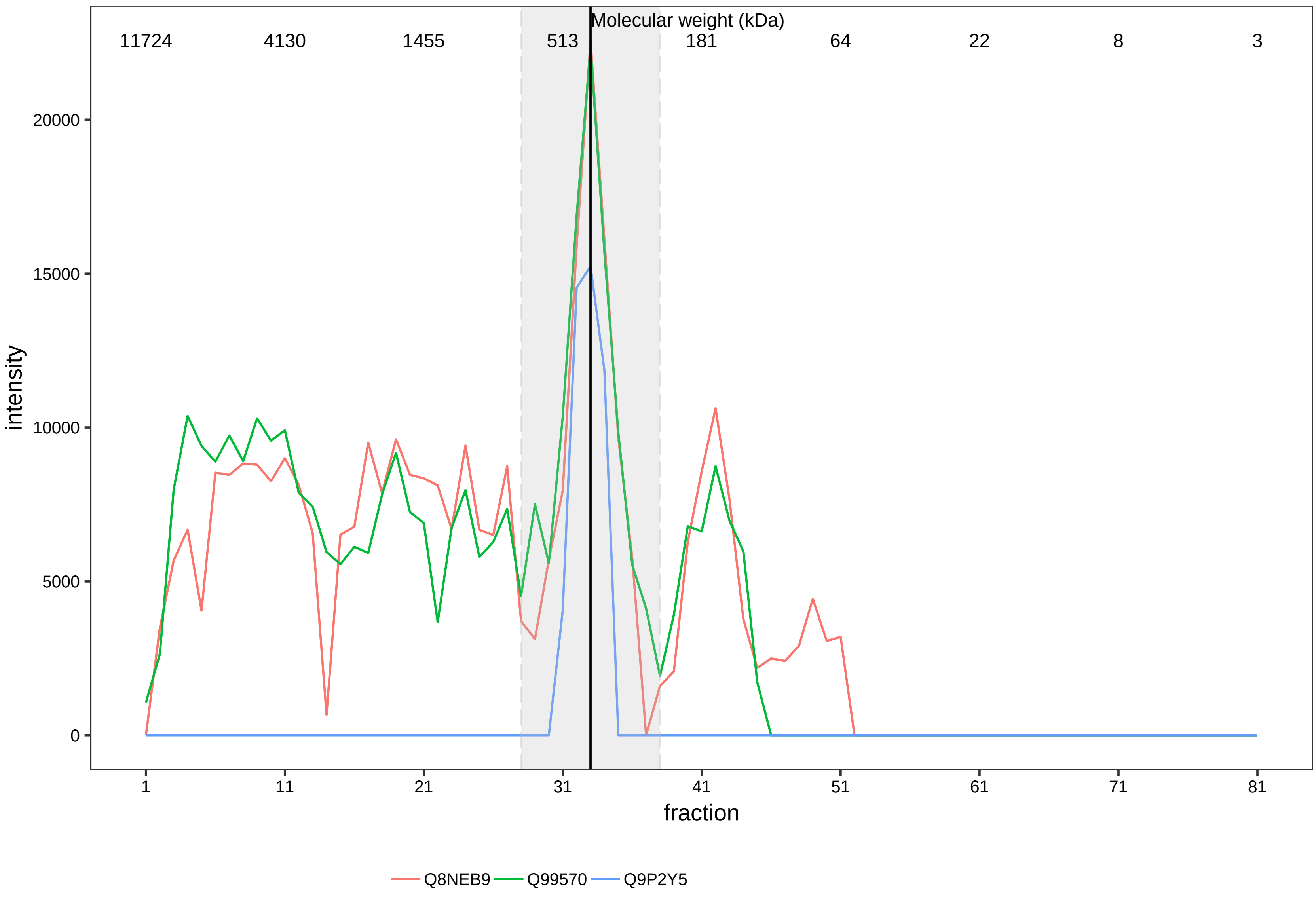

Feature ID 151

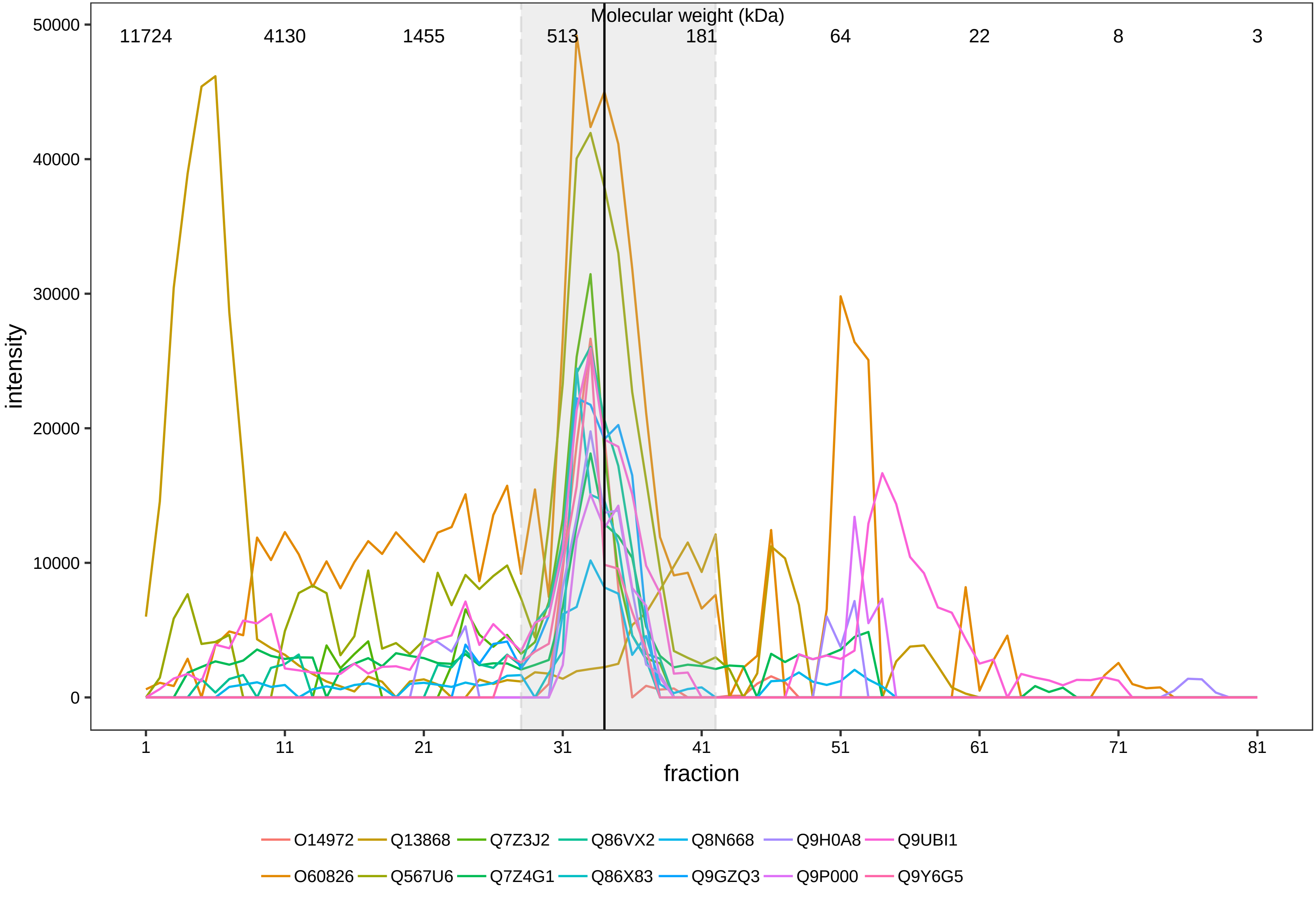

Feature ID 152

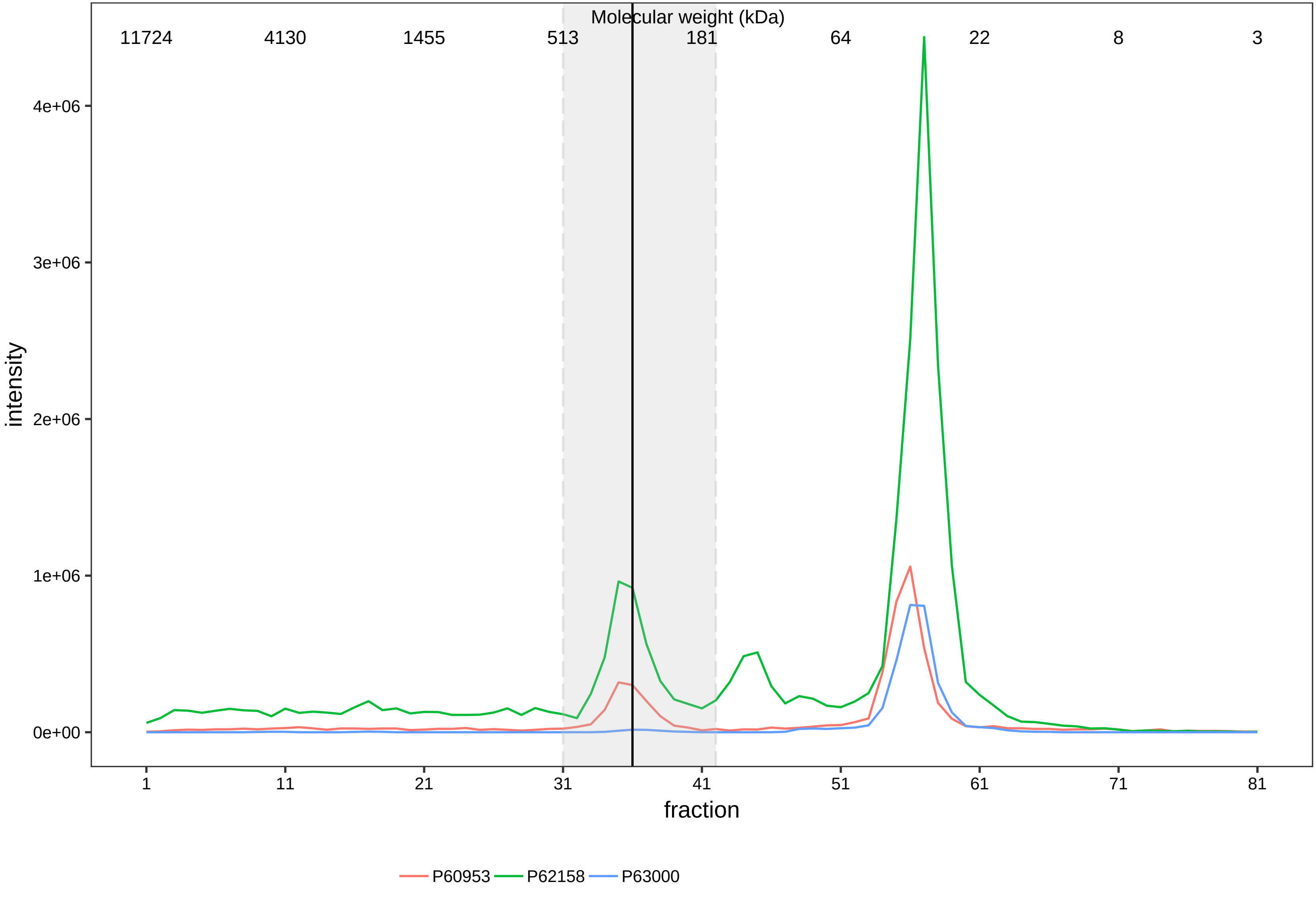

Feature ID 153

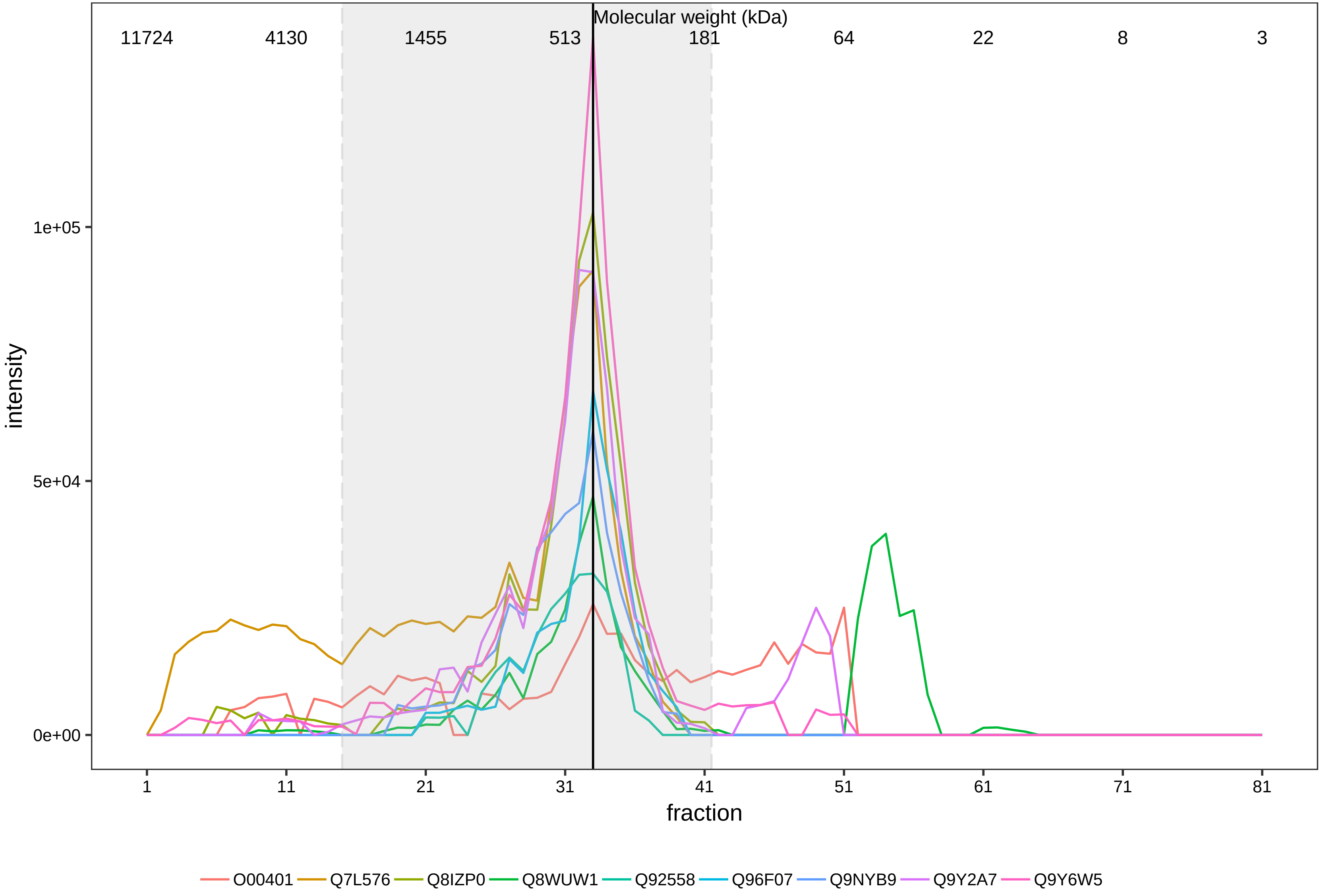

Feature ID 154

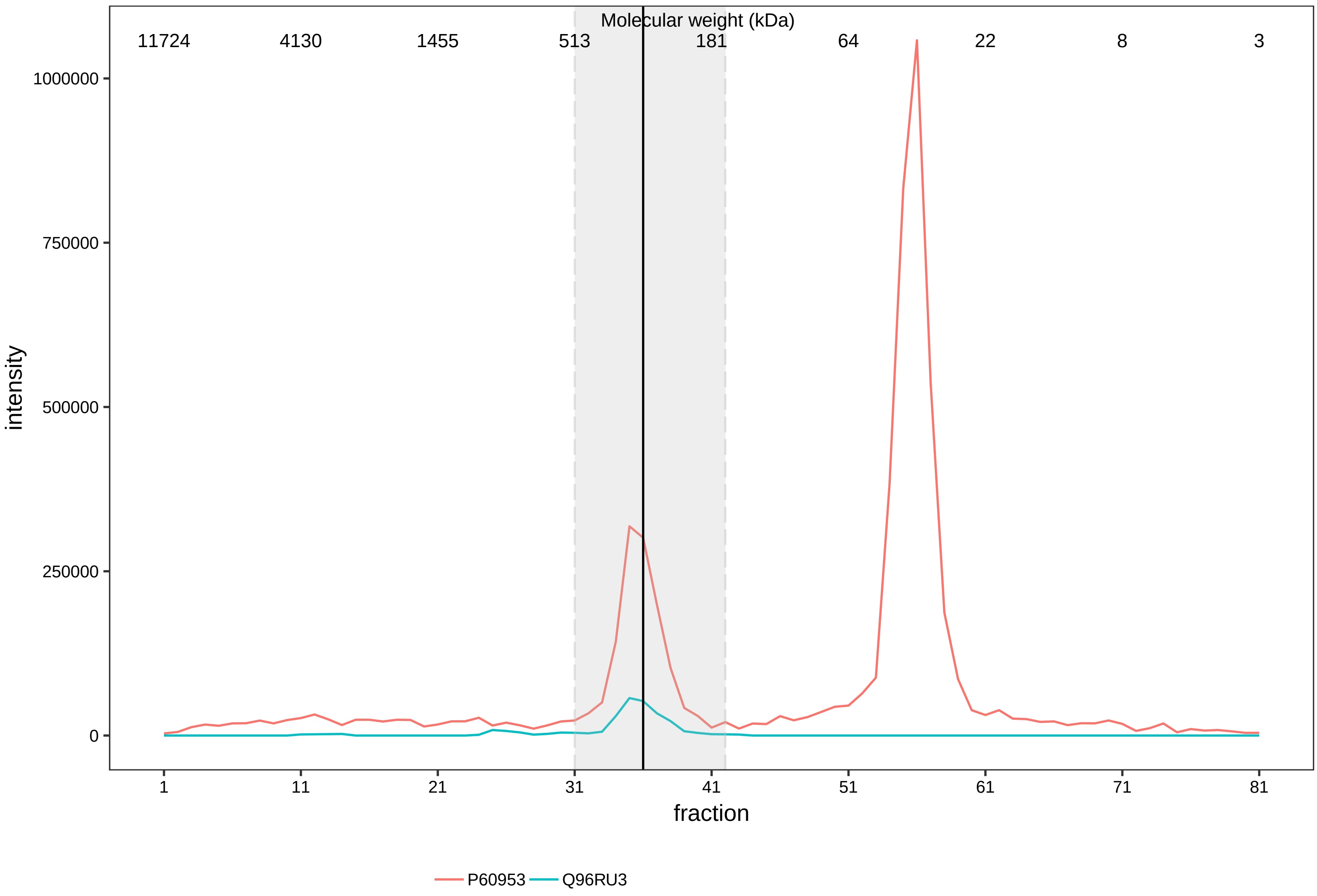

Feature ID 155

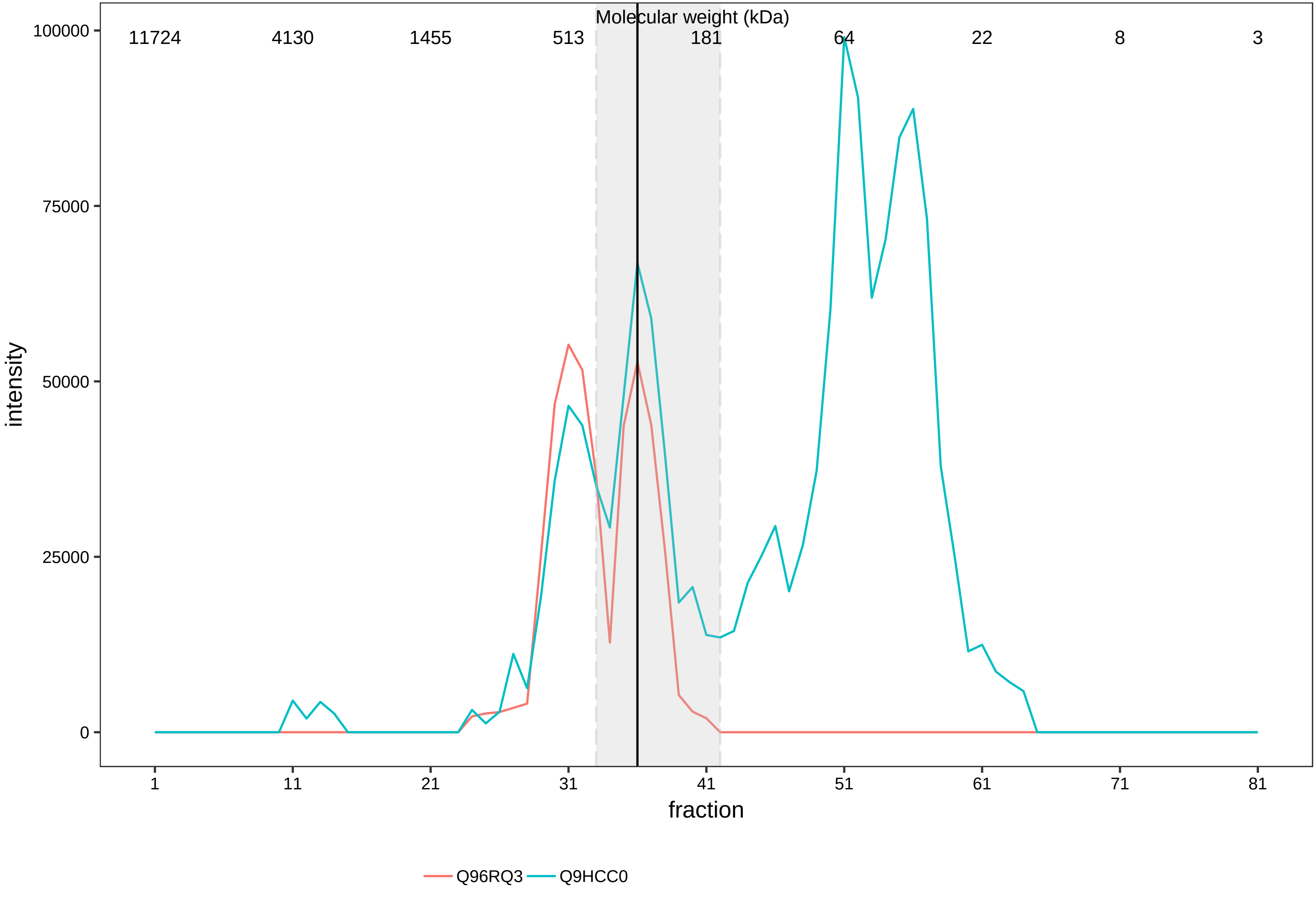

Feature ID 156

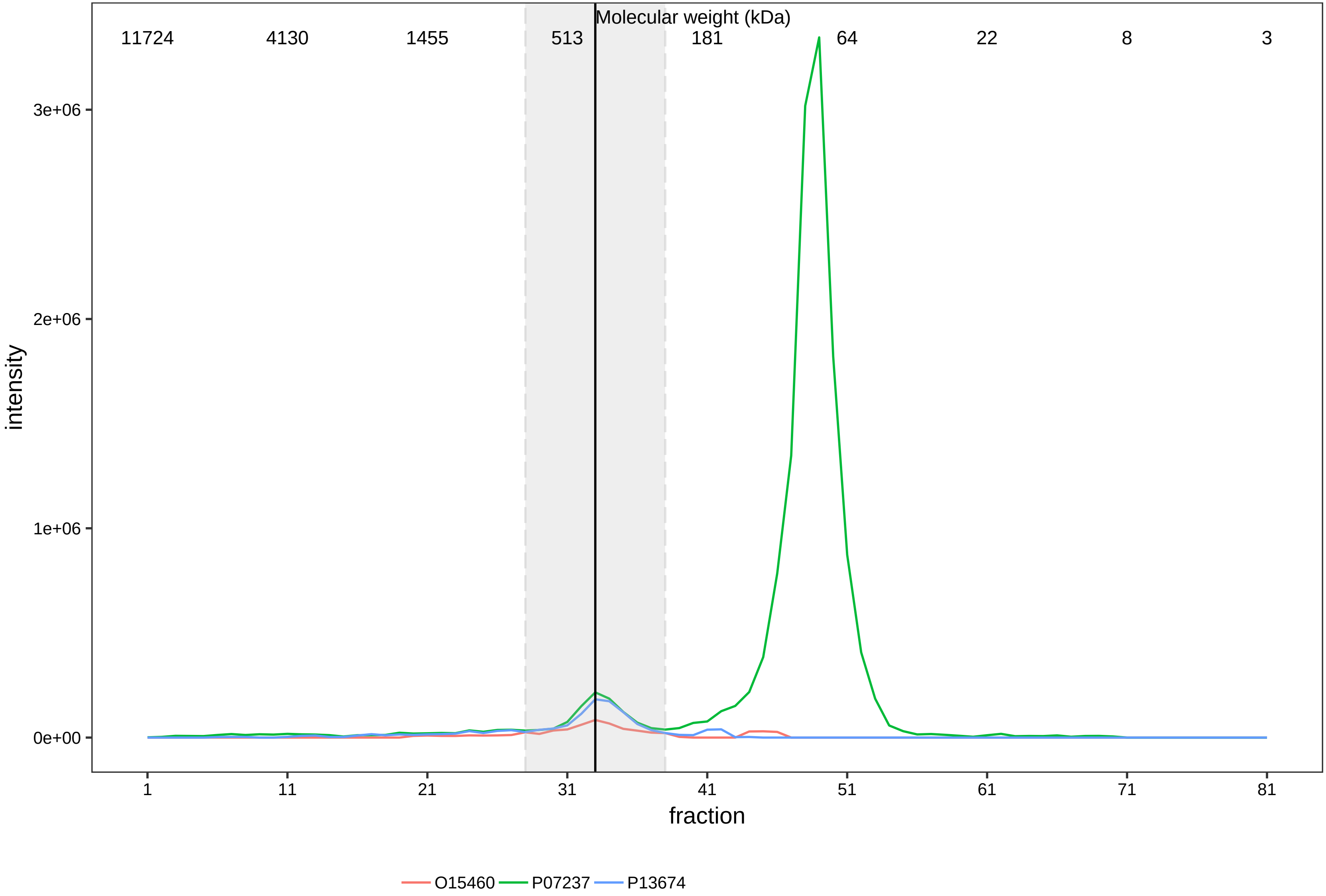

Feature ID 157

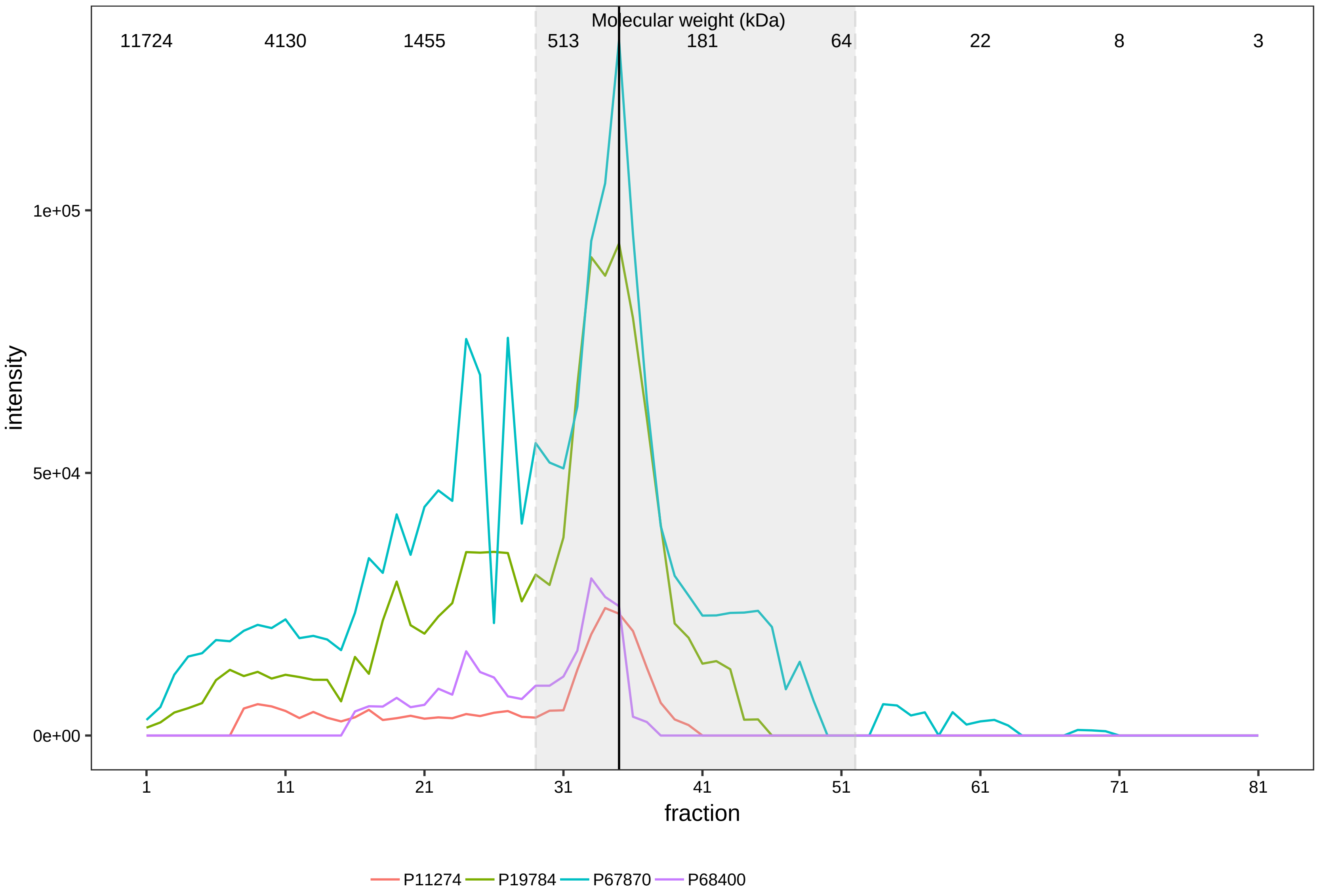

Feature ID 158

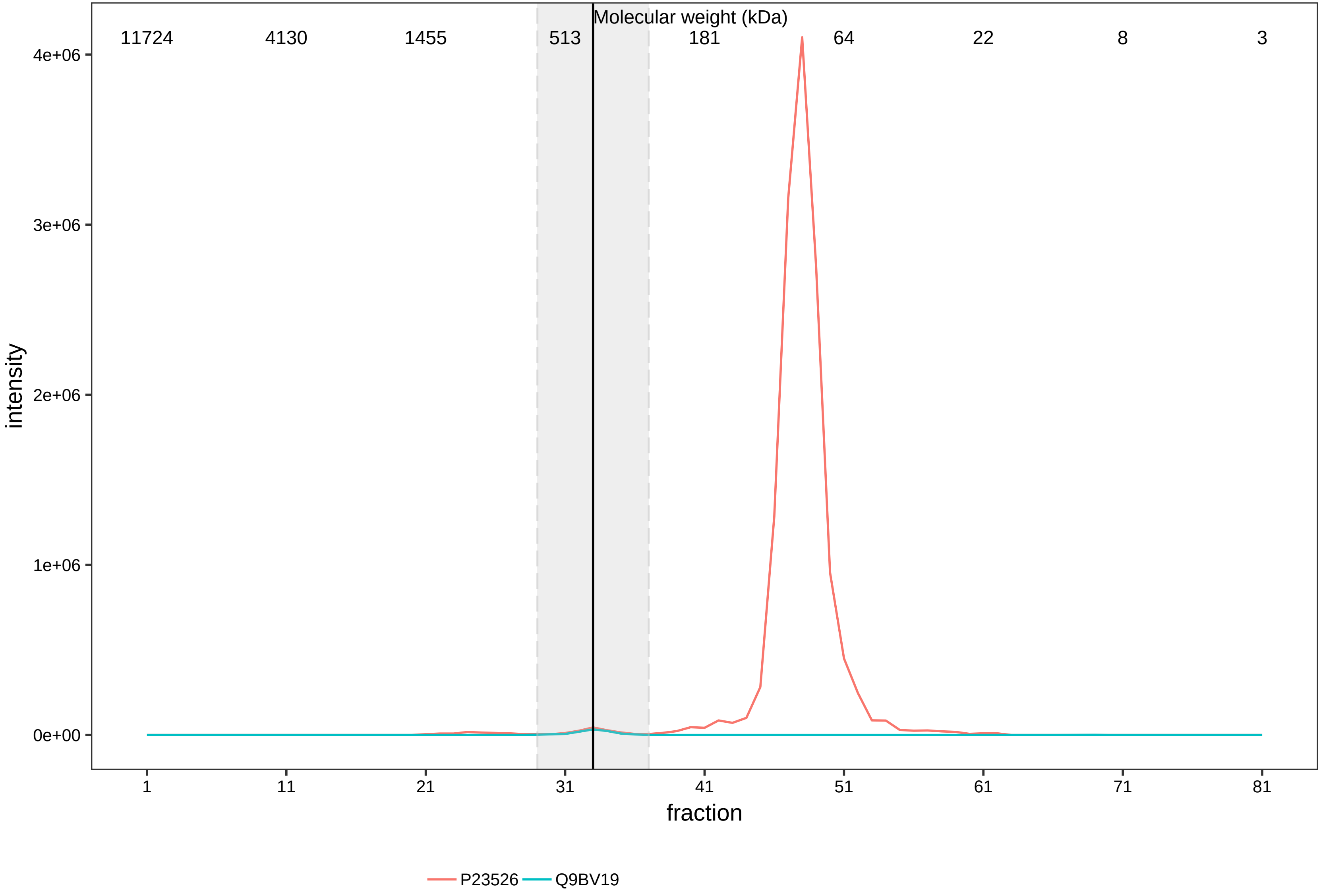

Feature ID 159

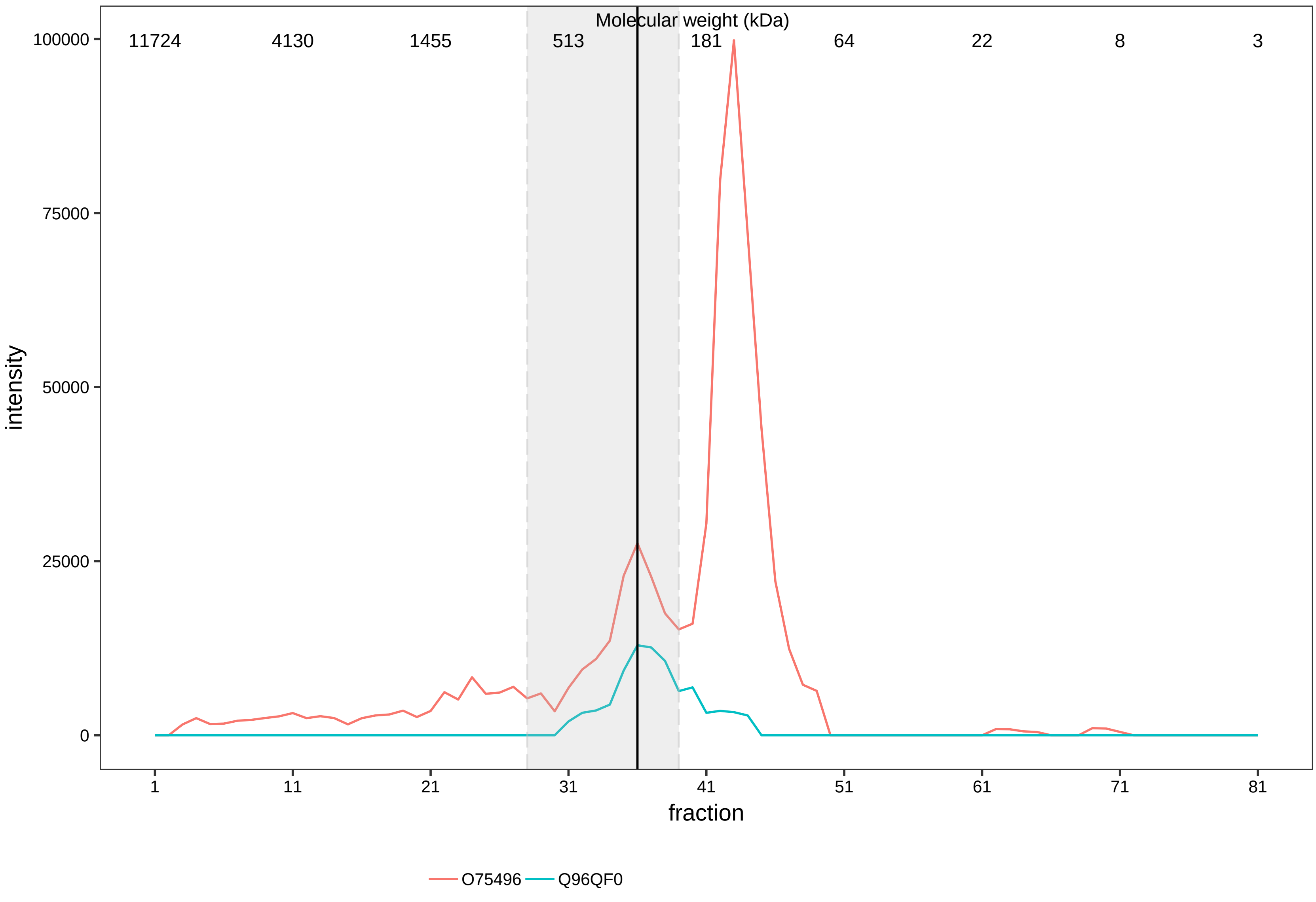

Feature ID 160

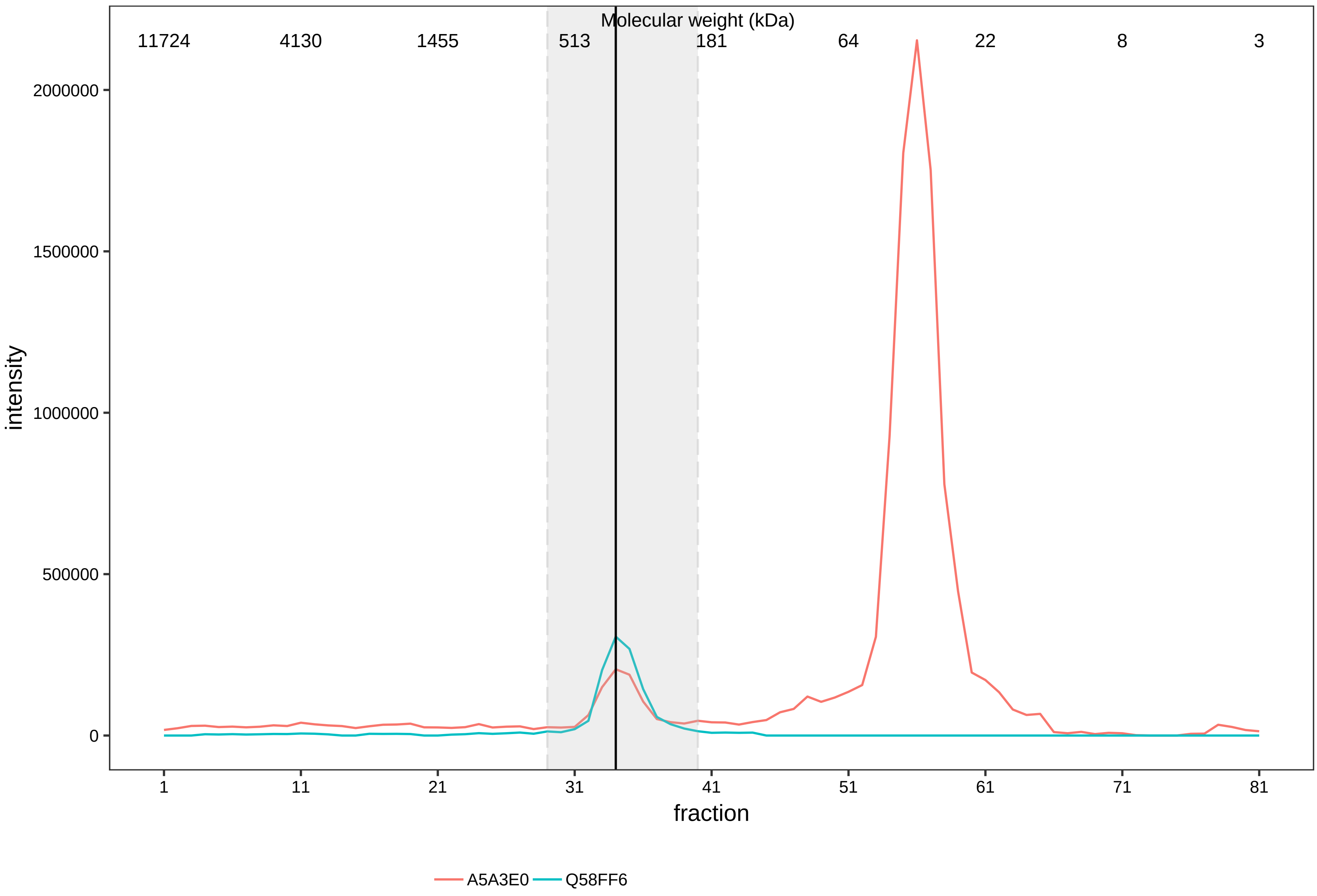

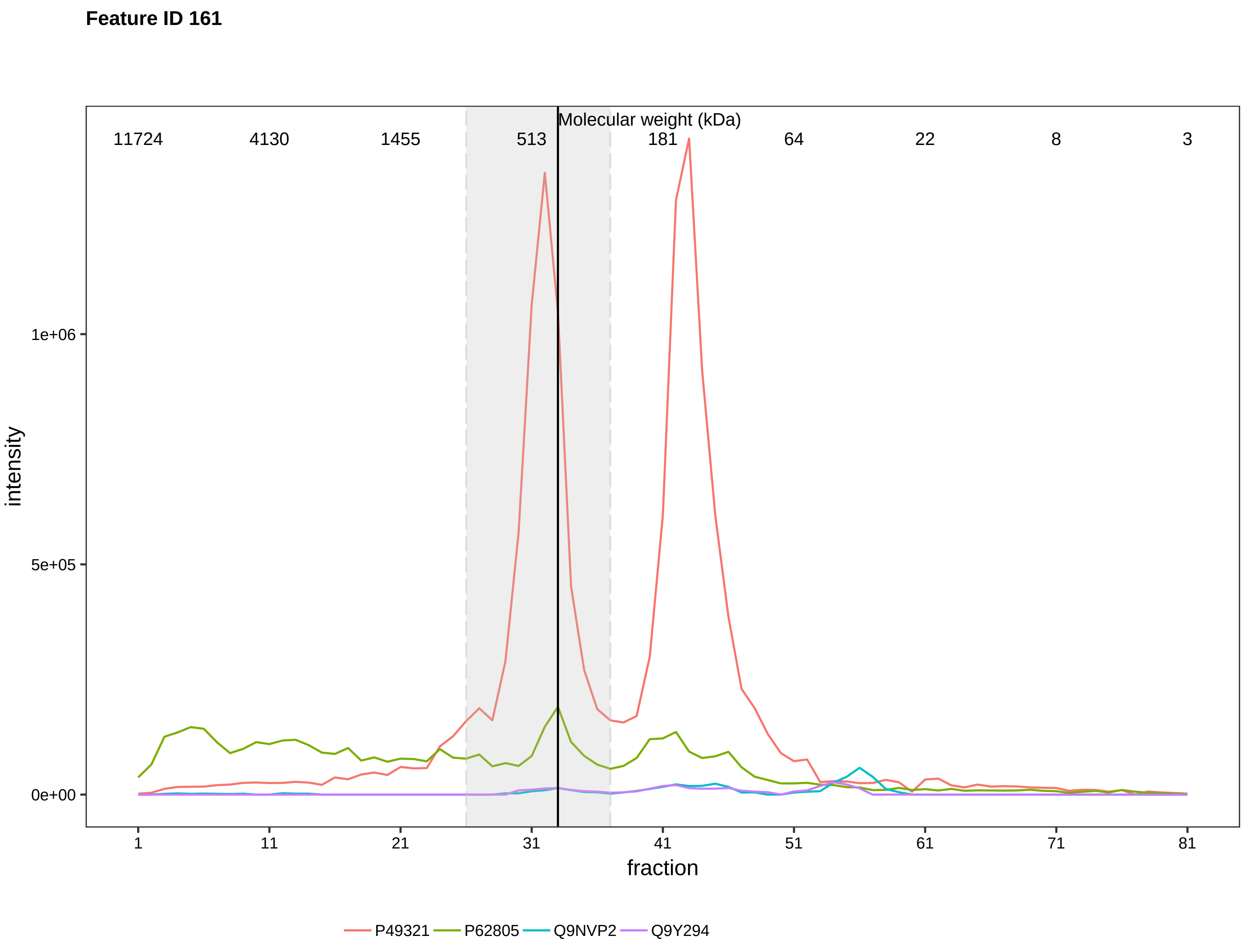
Feature ID 161
Molecular weight (kDa)
11724
4130
1455
513
181
64
22
8
3
1e+06
5e+05
0e+00
intensity
1
11
21
31
41
51
61
71
81
fraction
P49321
P62805
Q9NVP2
Q9Y294

Feature ID 162

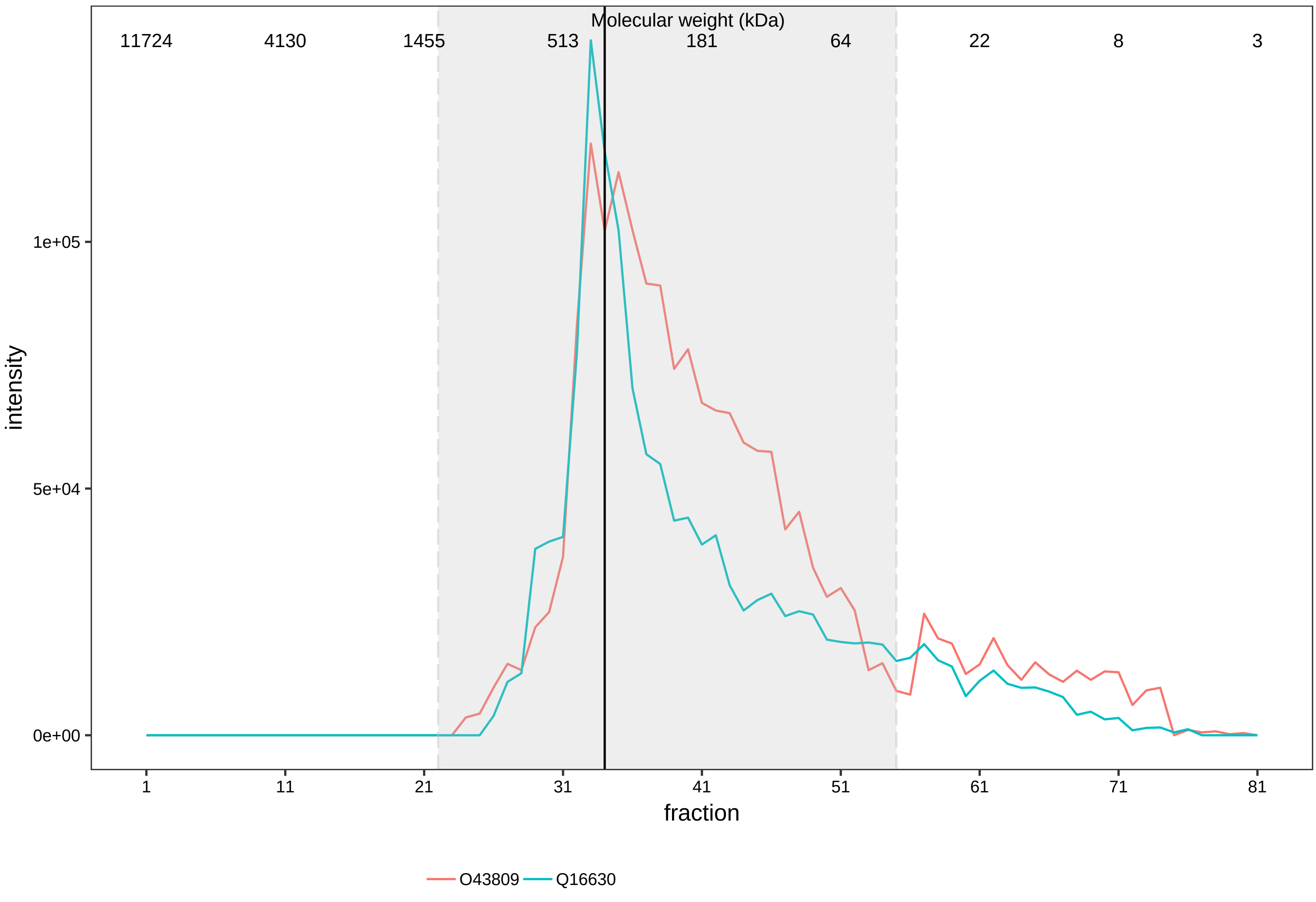

Feature ID 163

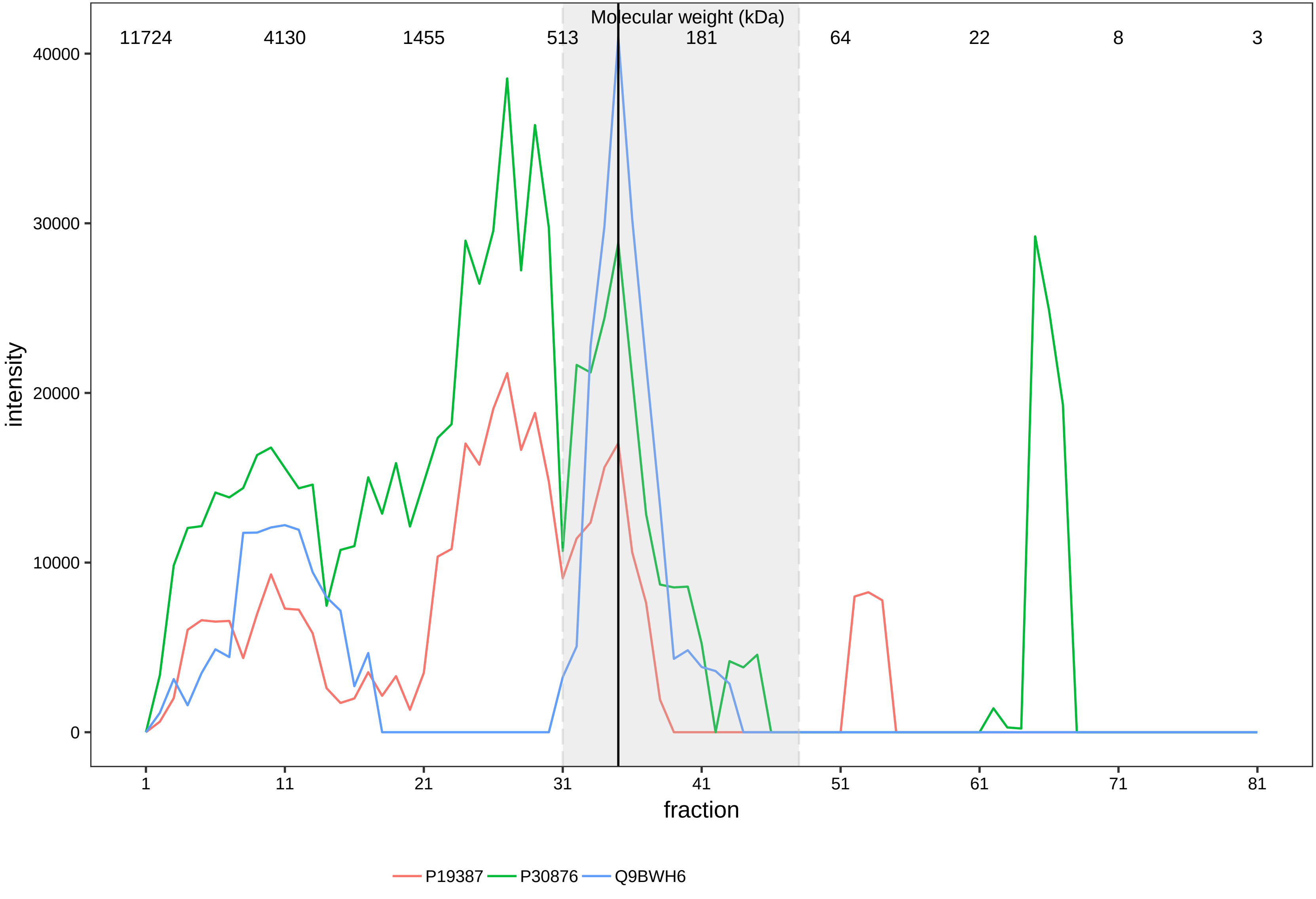

Feature ID 164

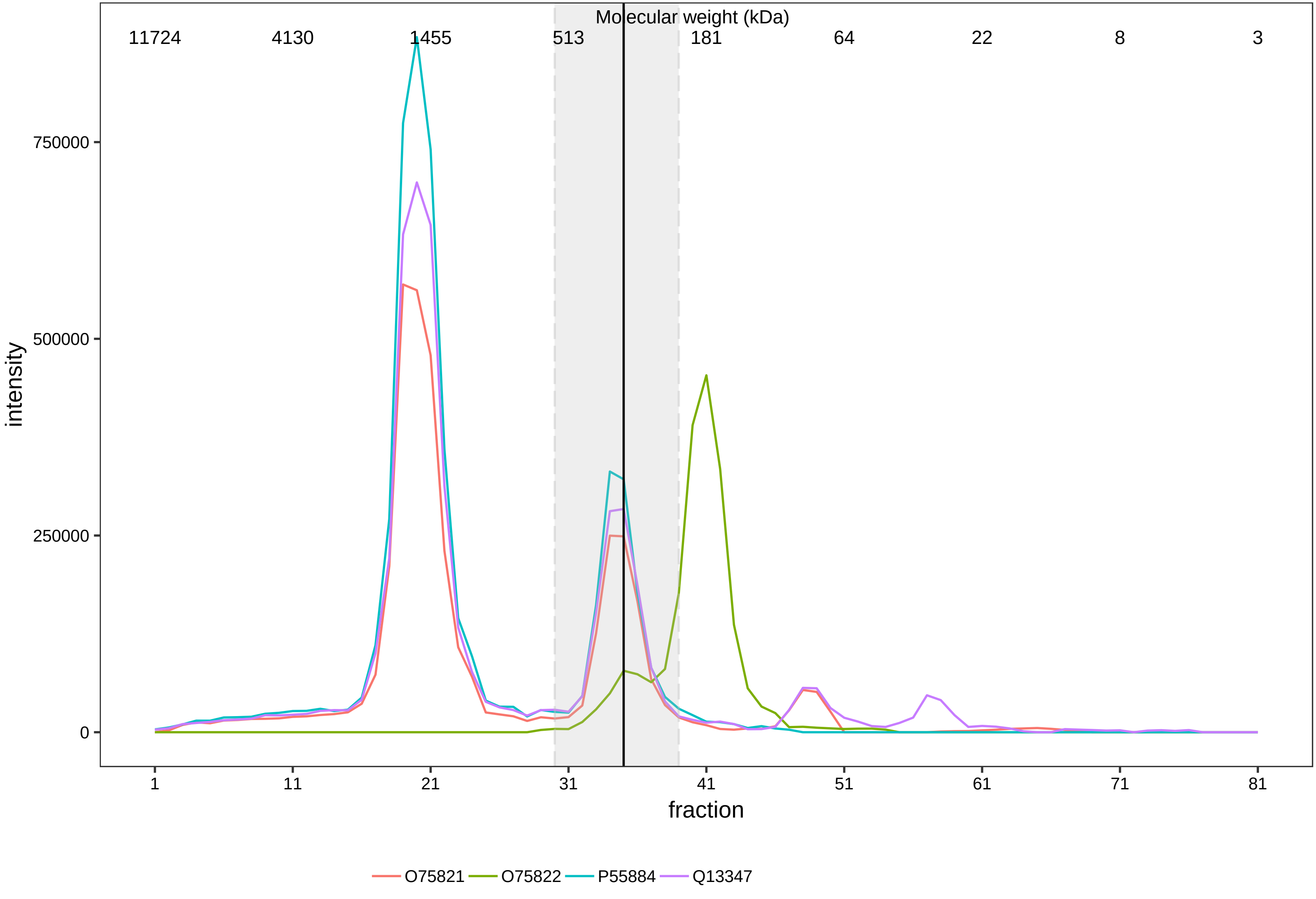

Feature ID 165

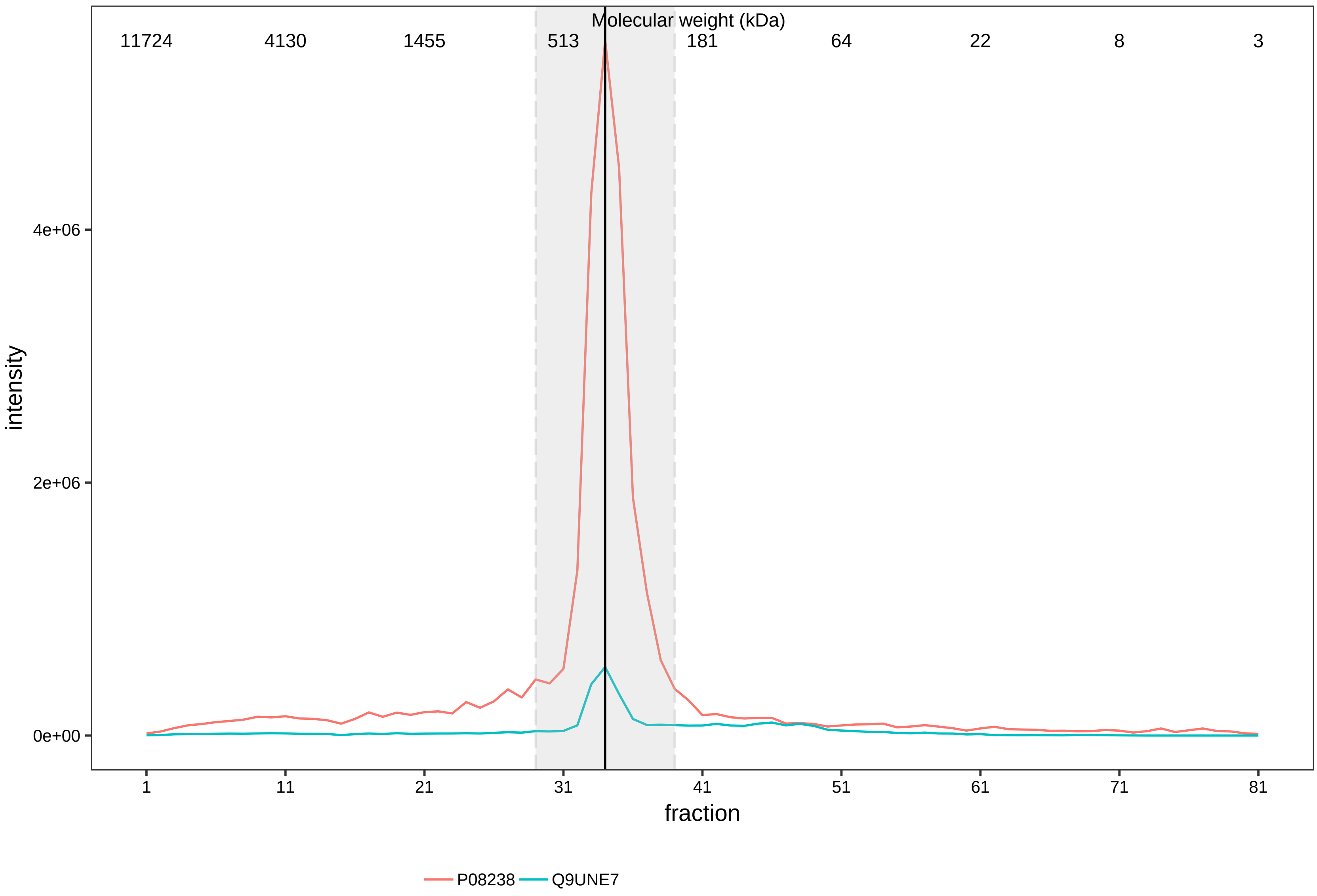

Feature ID 166

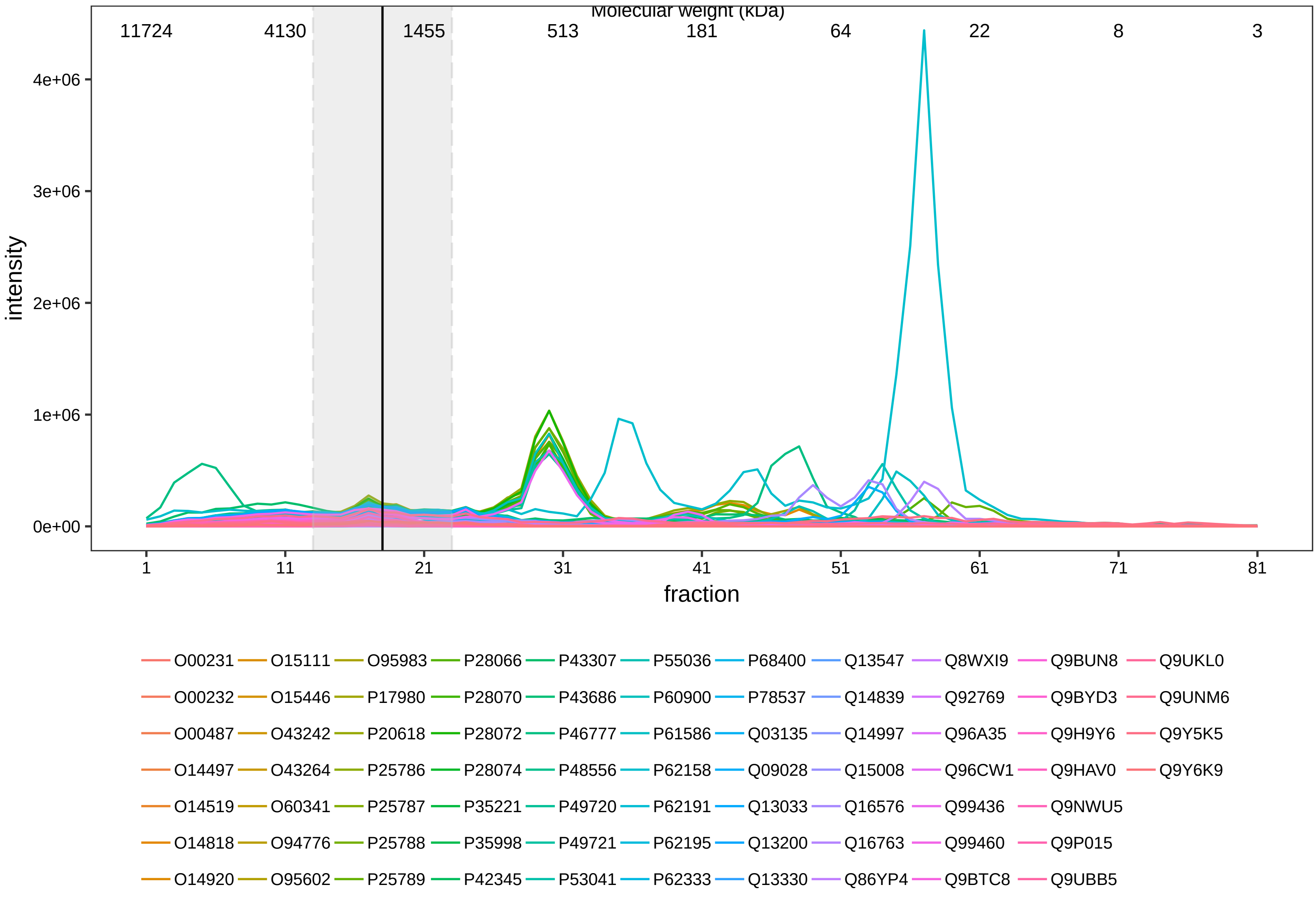

Feature ID 167

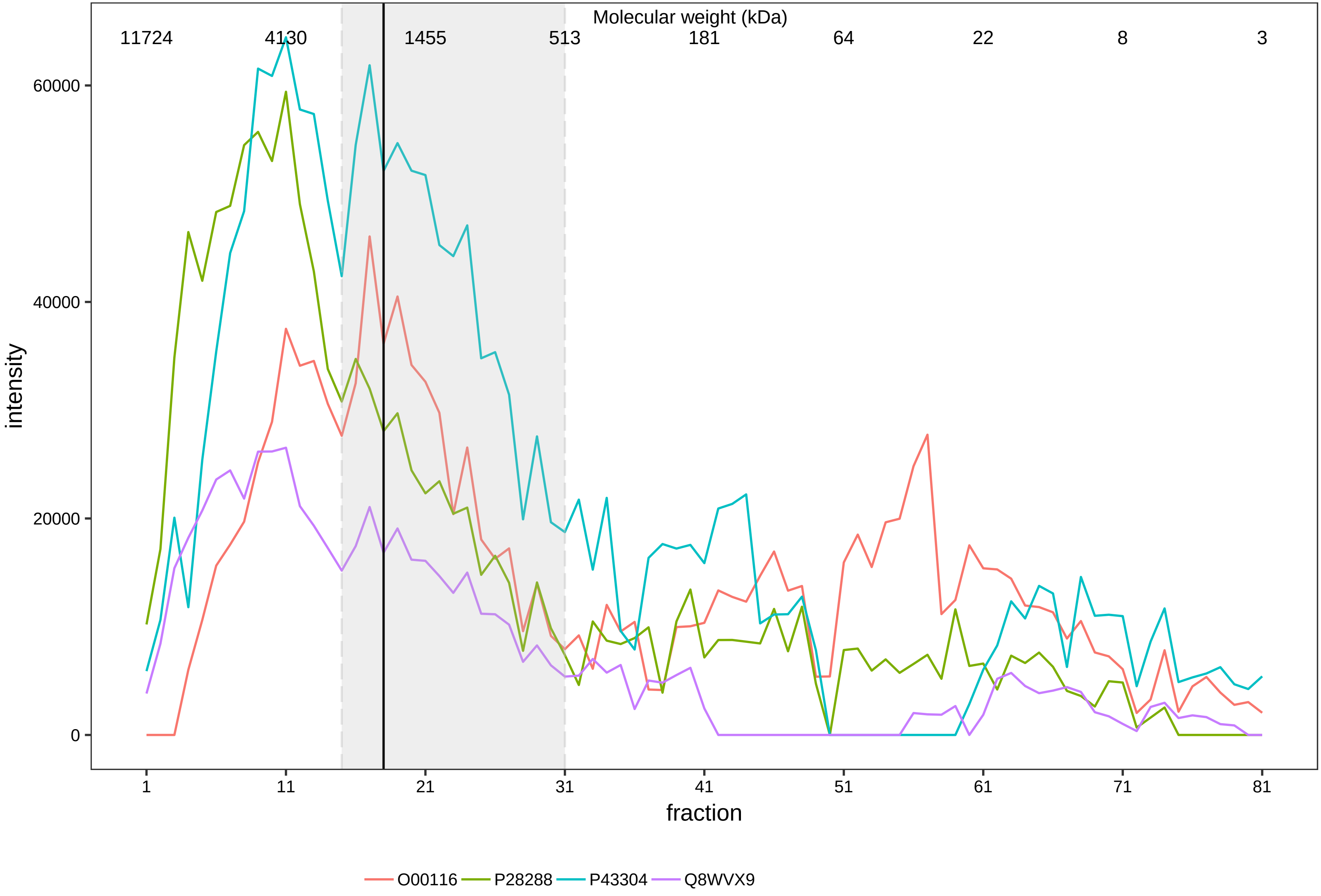

Feature ID 168

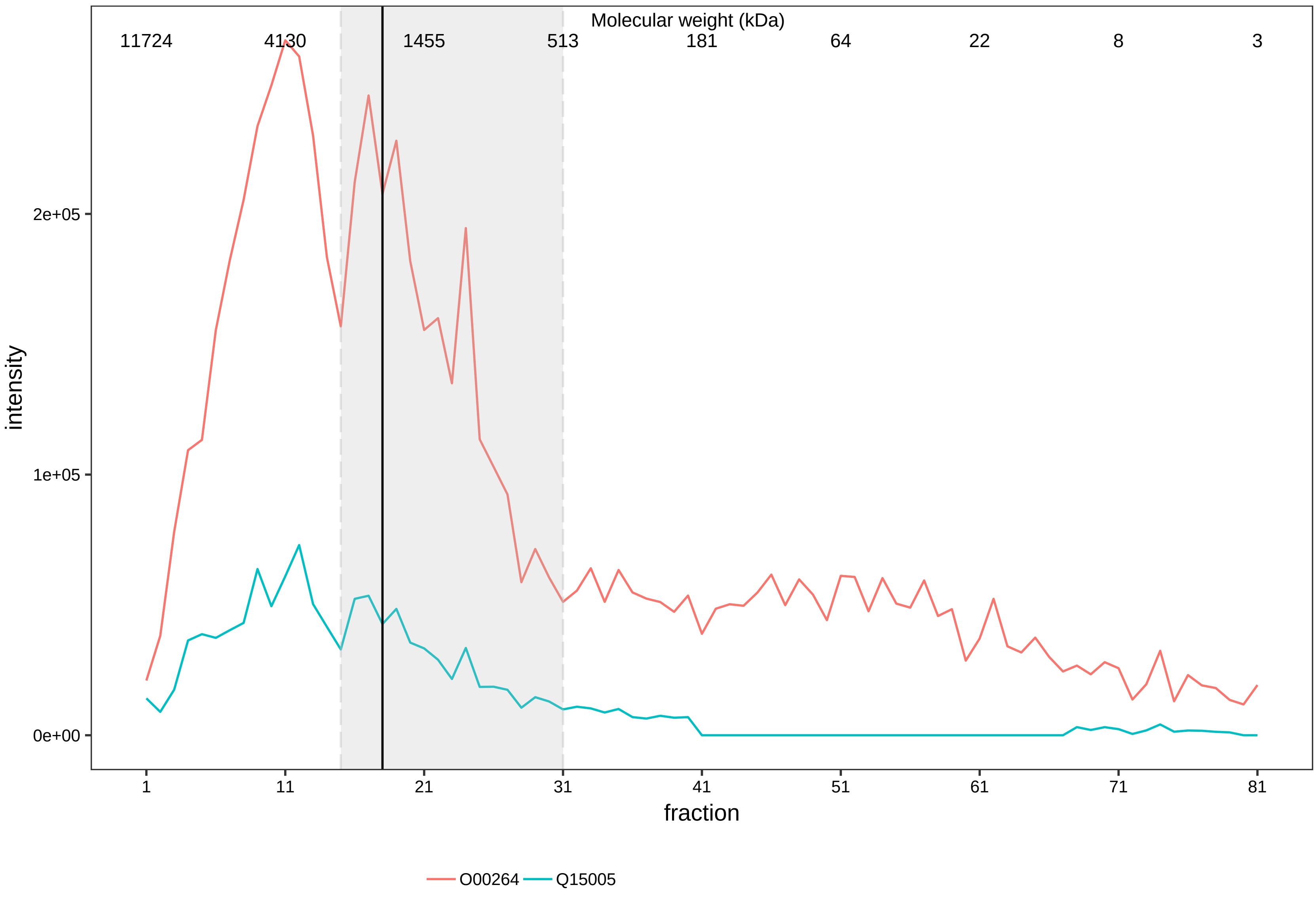

Feature ID 169

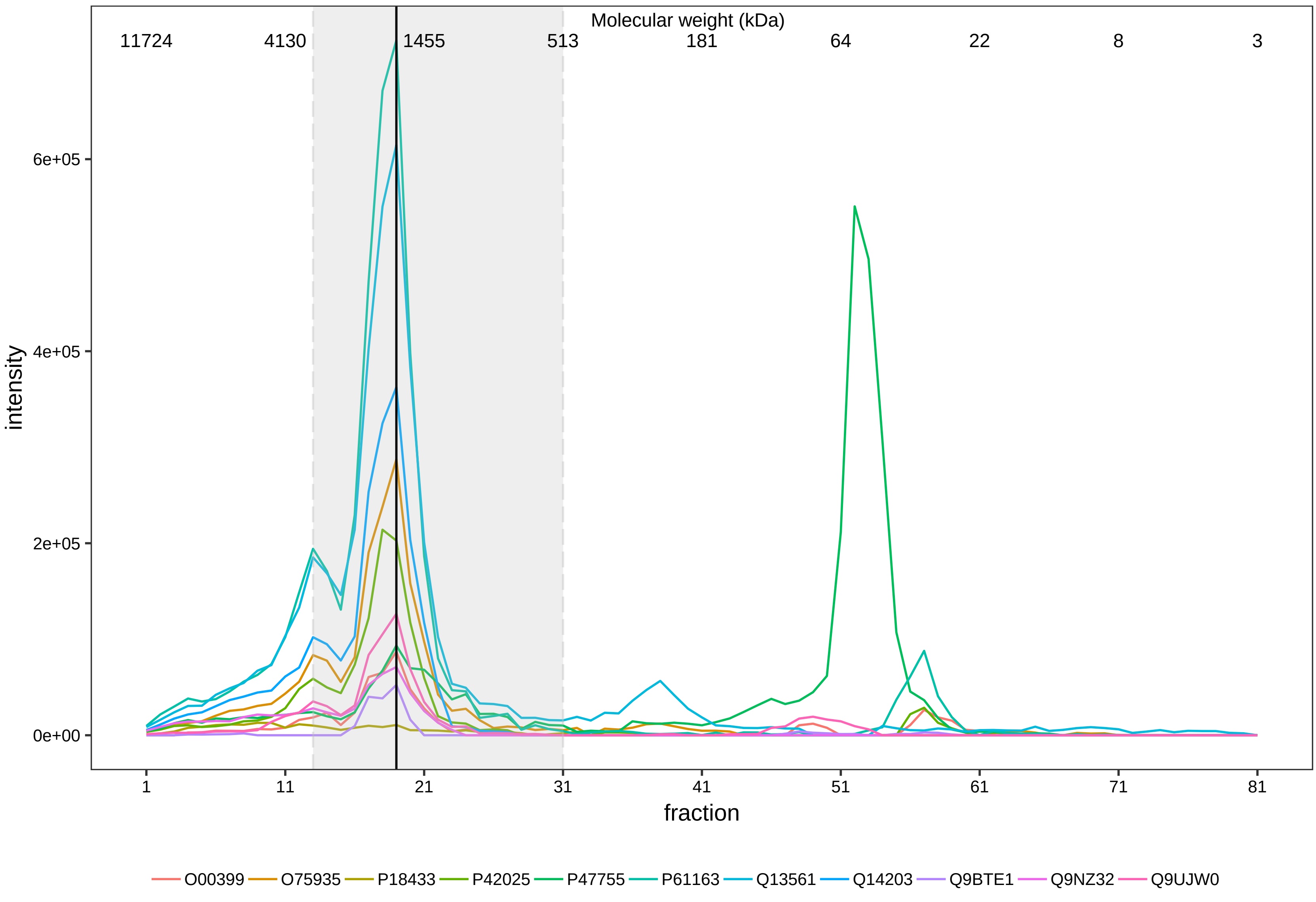

Feature ID 170

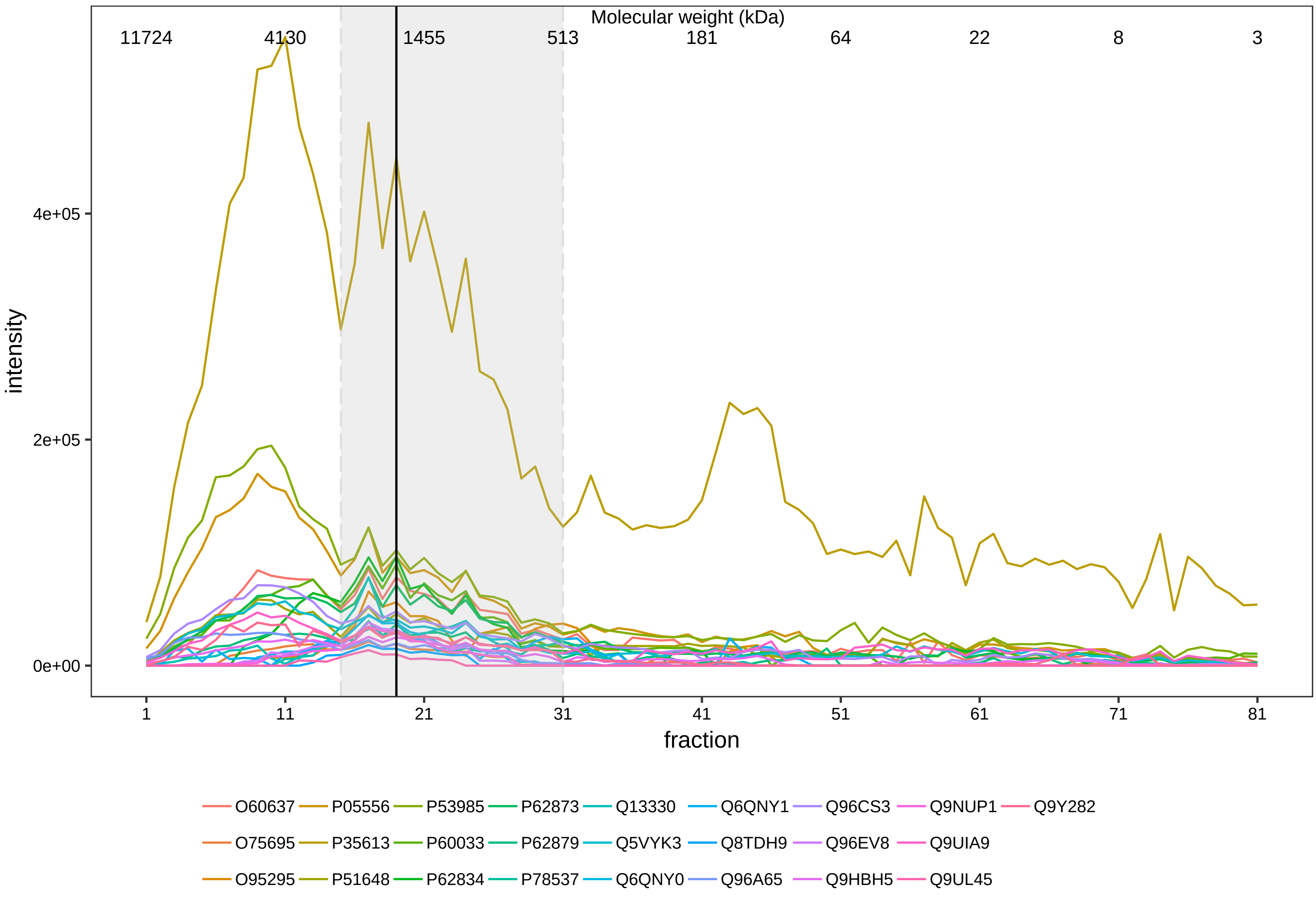

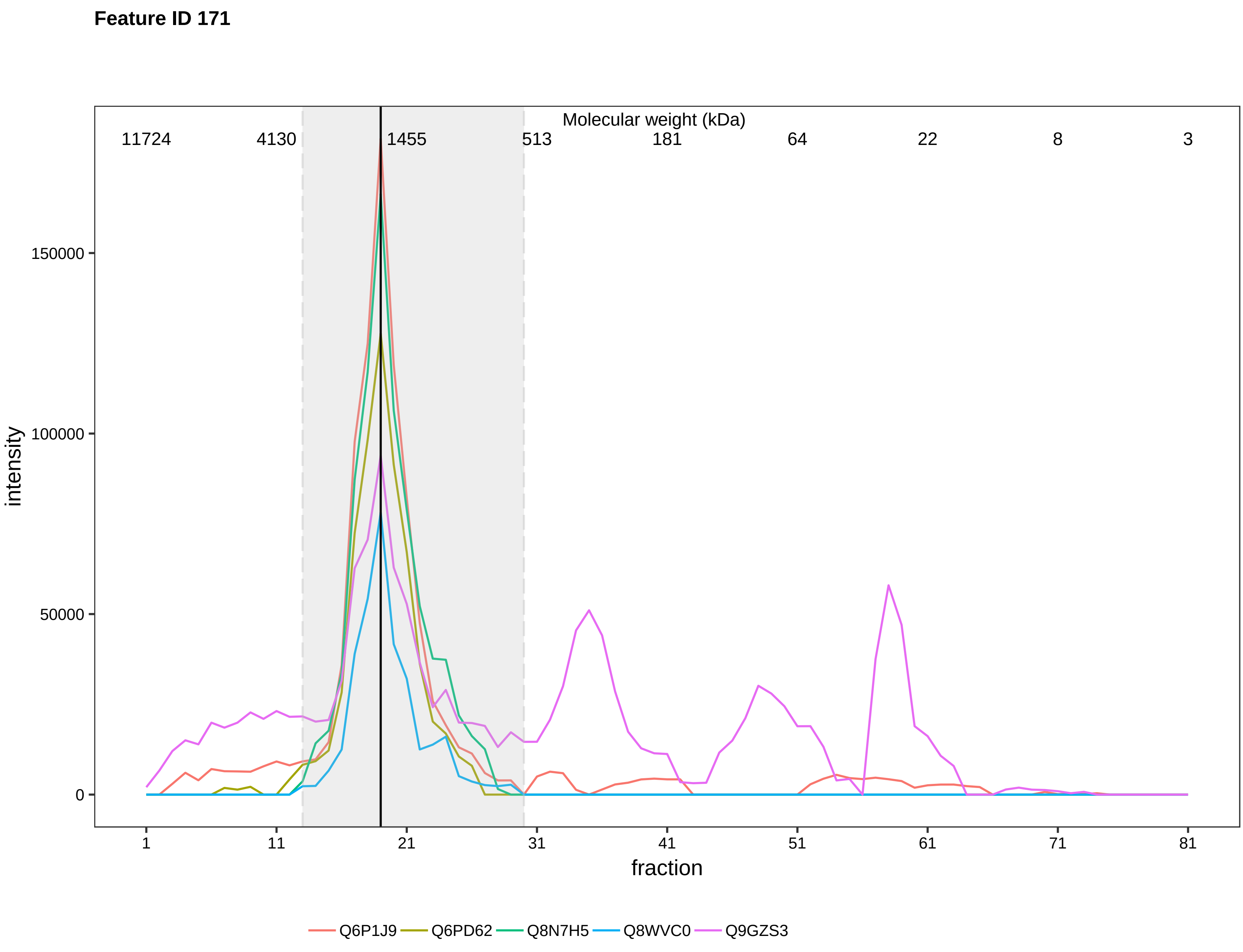
Feature ID 171
Molecular weight (kDa)
11724
4130
1455
513
181
64
22
8
3
150000
100000
50000
0
intensity
1
11
21
31
41
51
61
71
81
fraction
Q6P1J9
Q6PD62
Q8N7H5
Q8WVC0
Q9GZS3

Feature ID 172

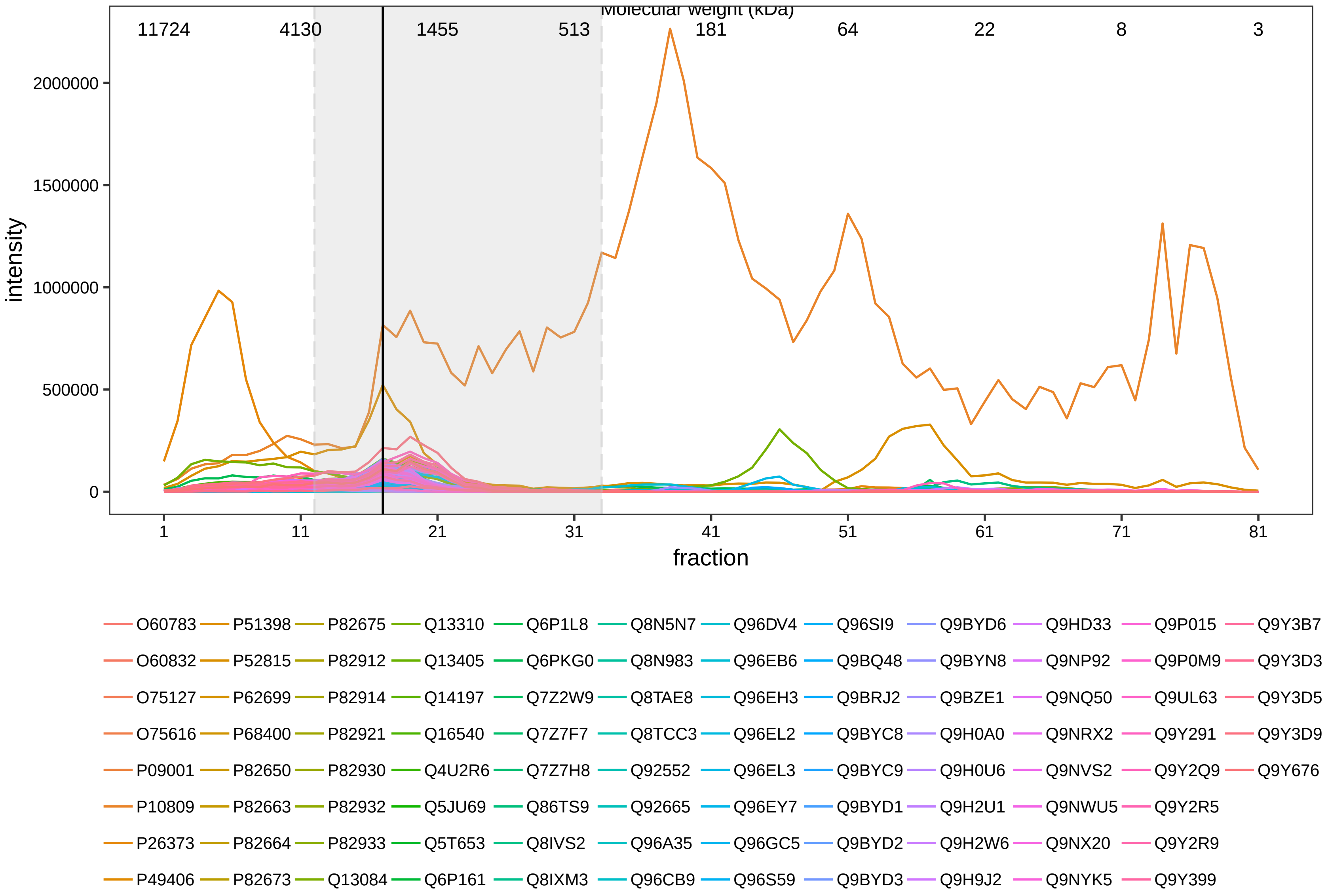

Feature ID 173

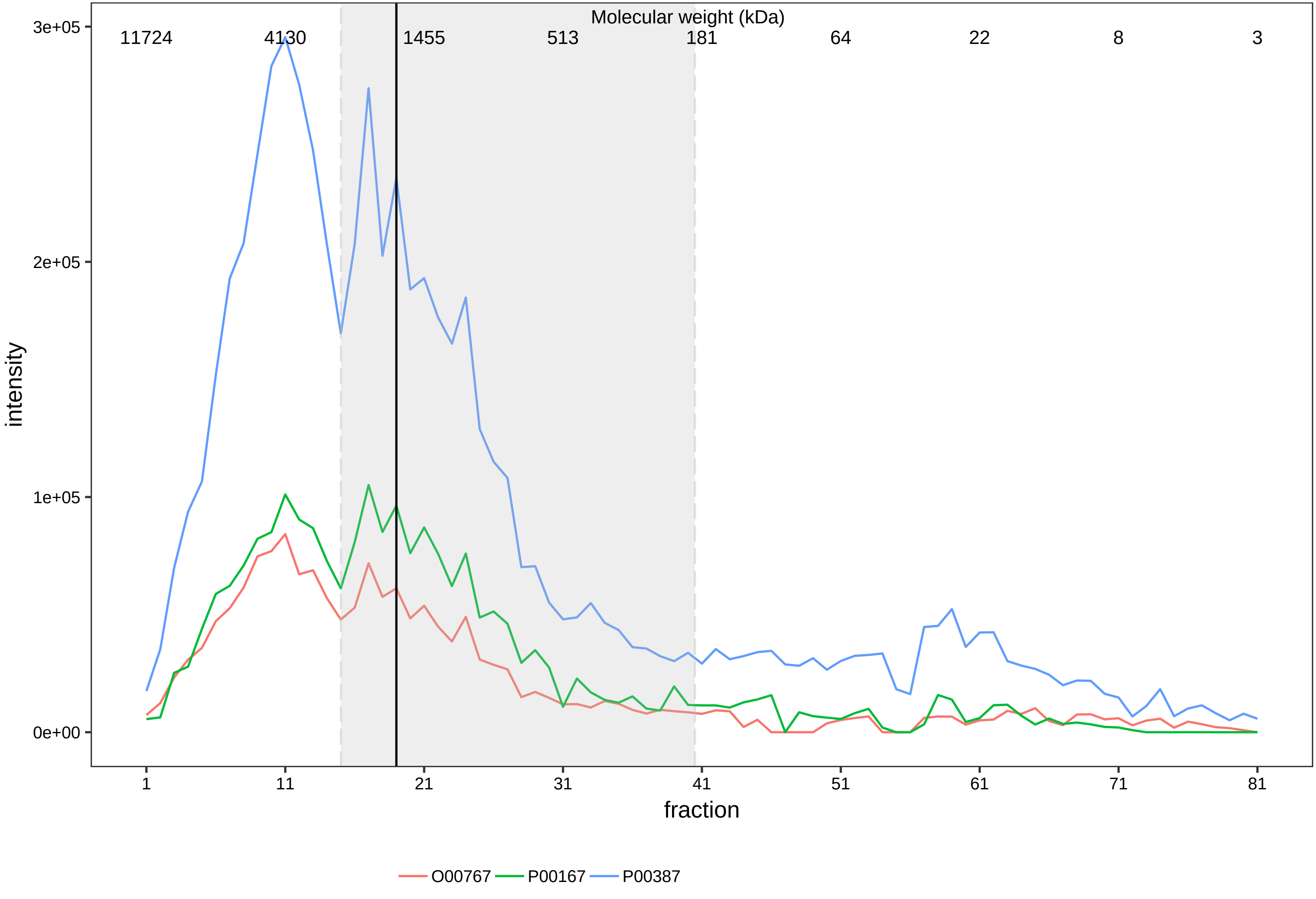

Feature ID 174

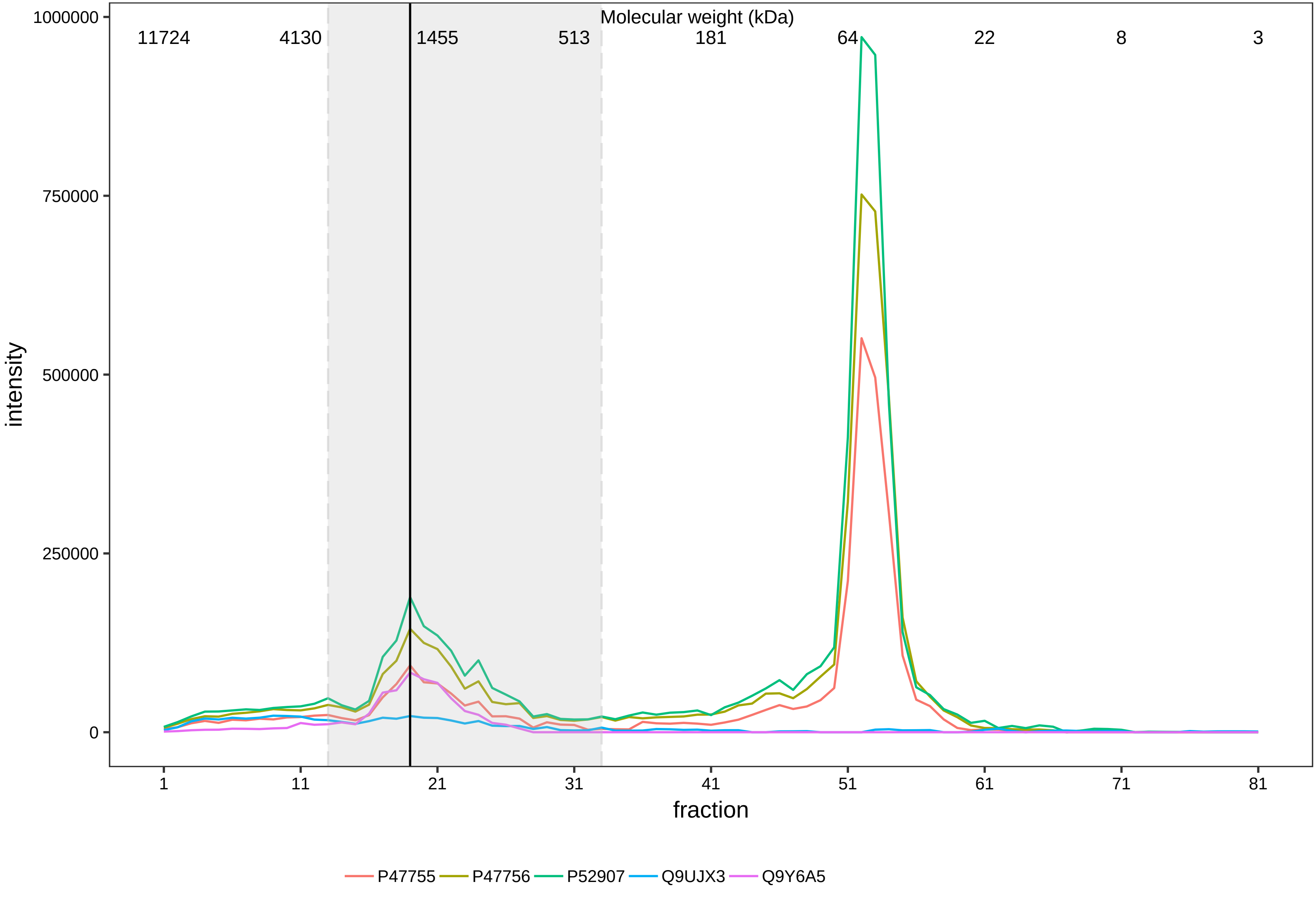

Feature ID 175

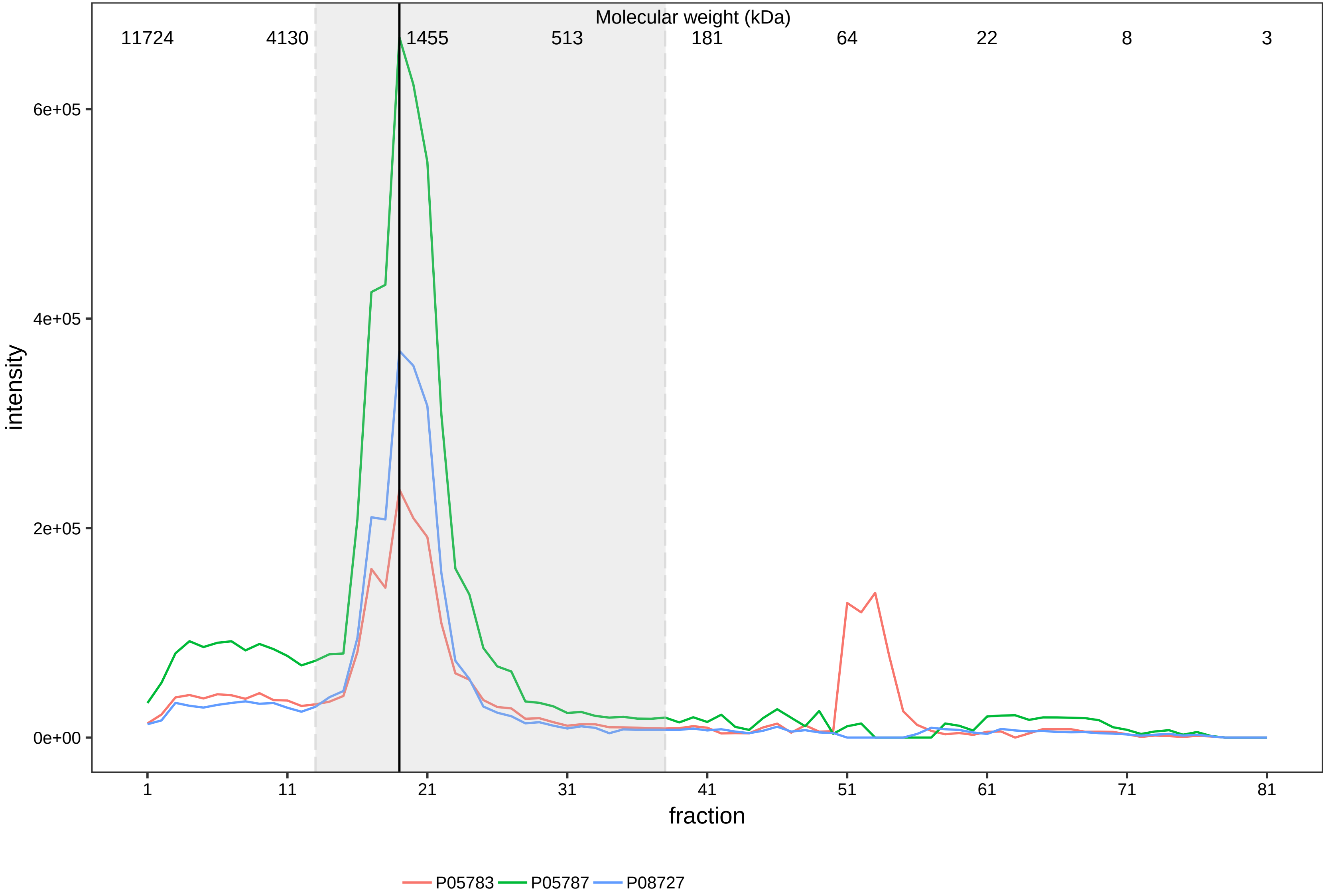

Feature ID 176

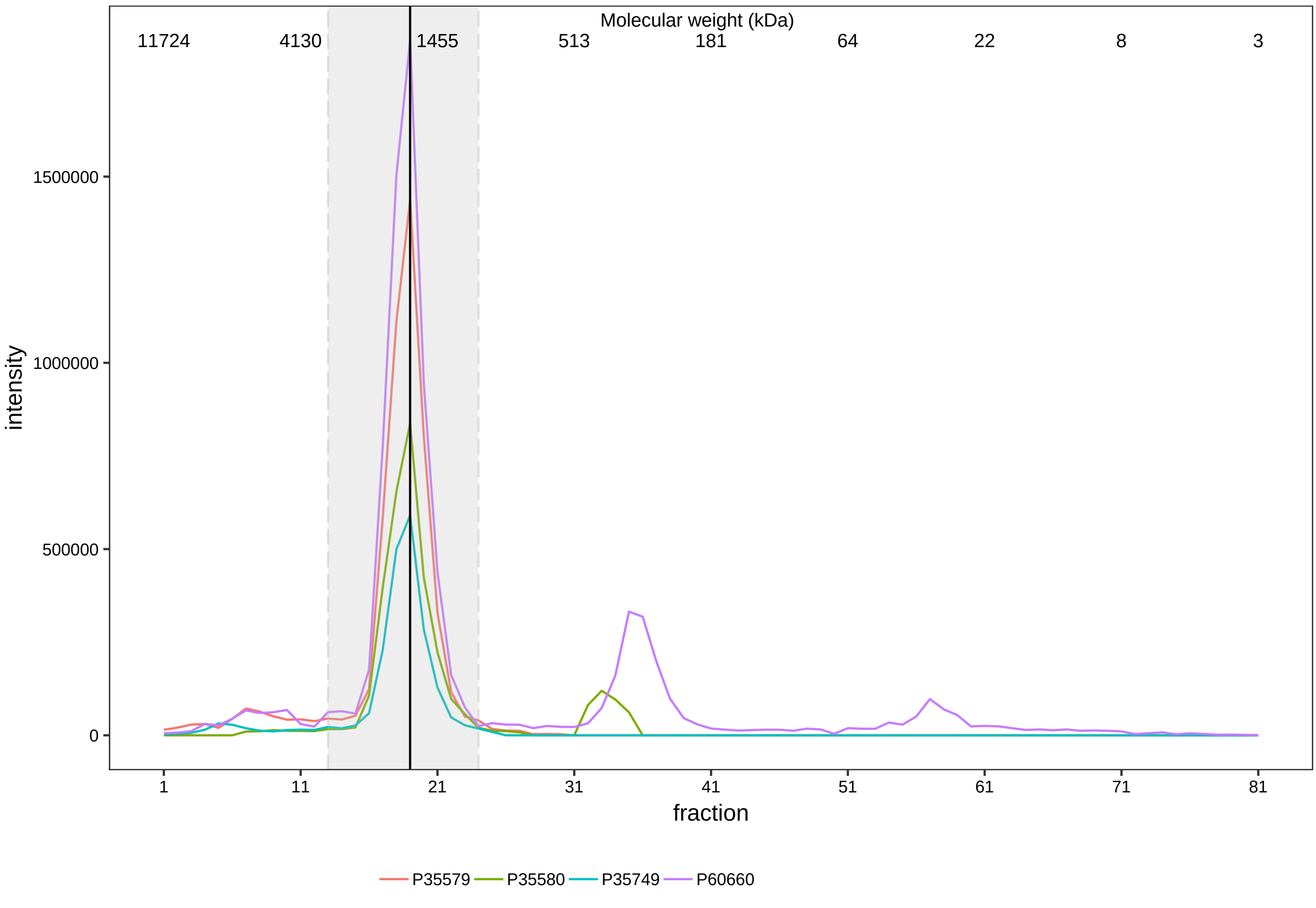

Feature ID 177

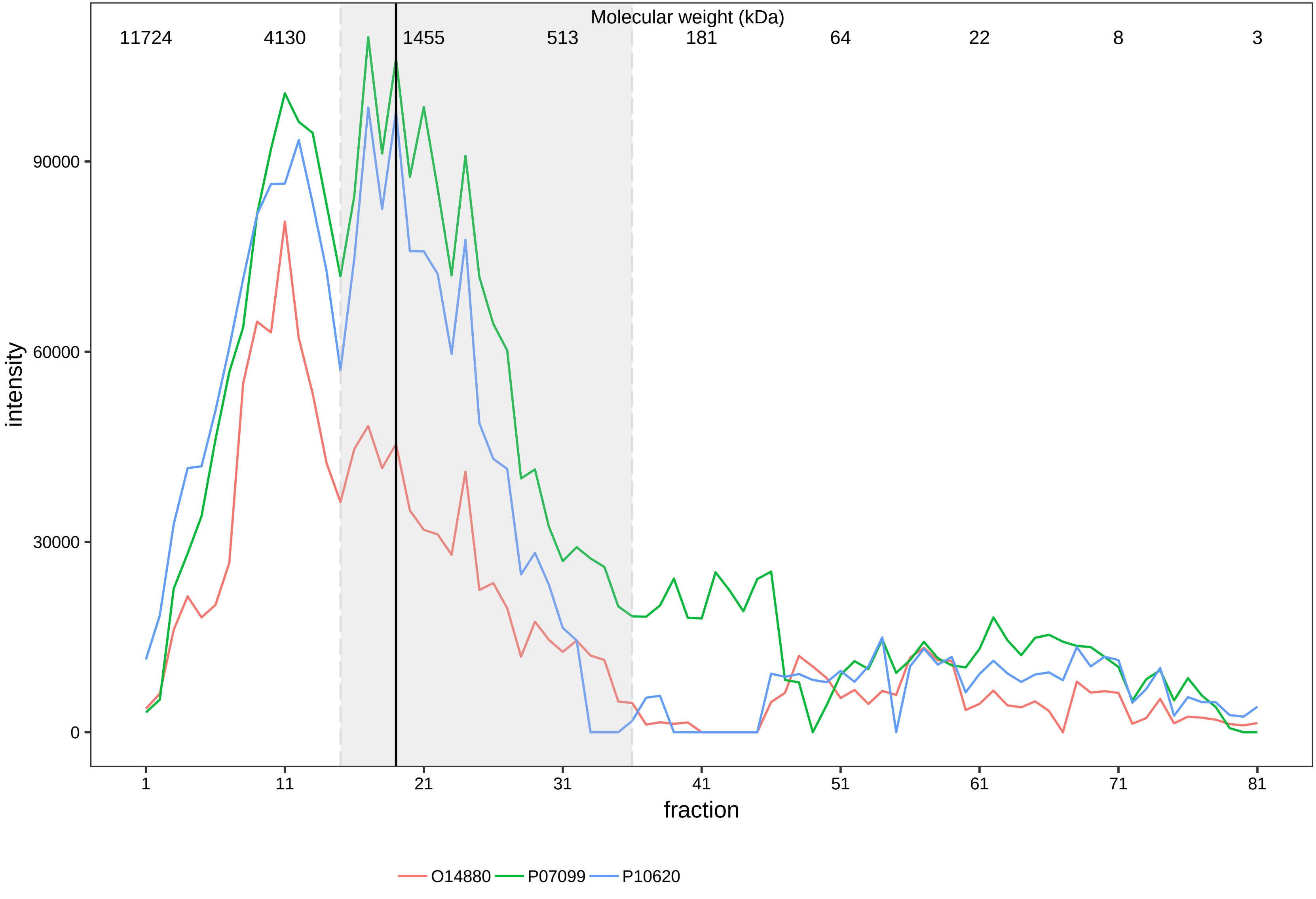

Feature ID 178

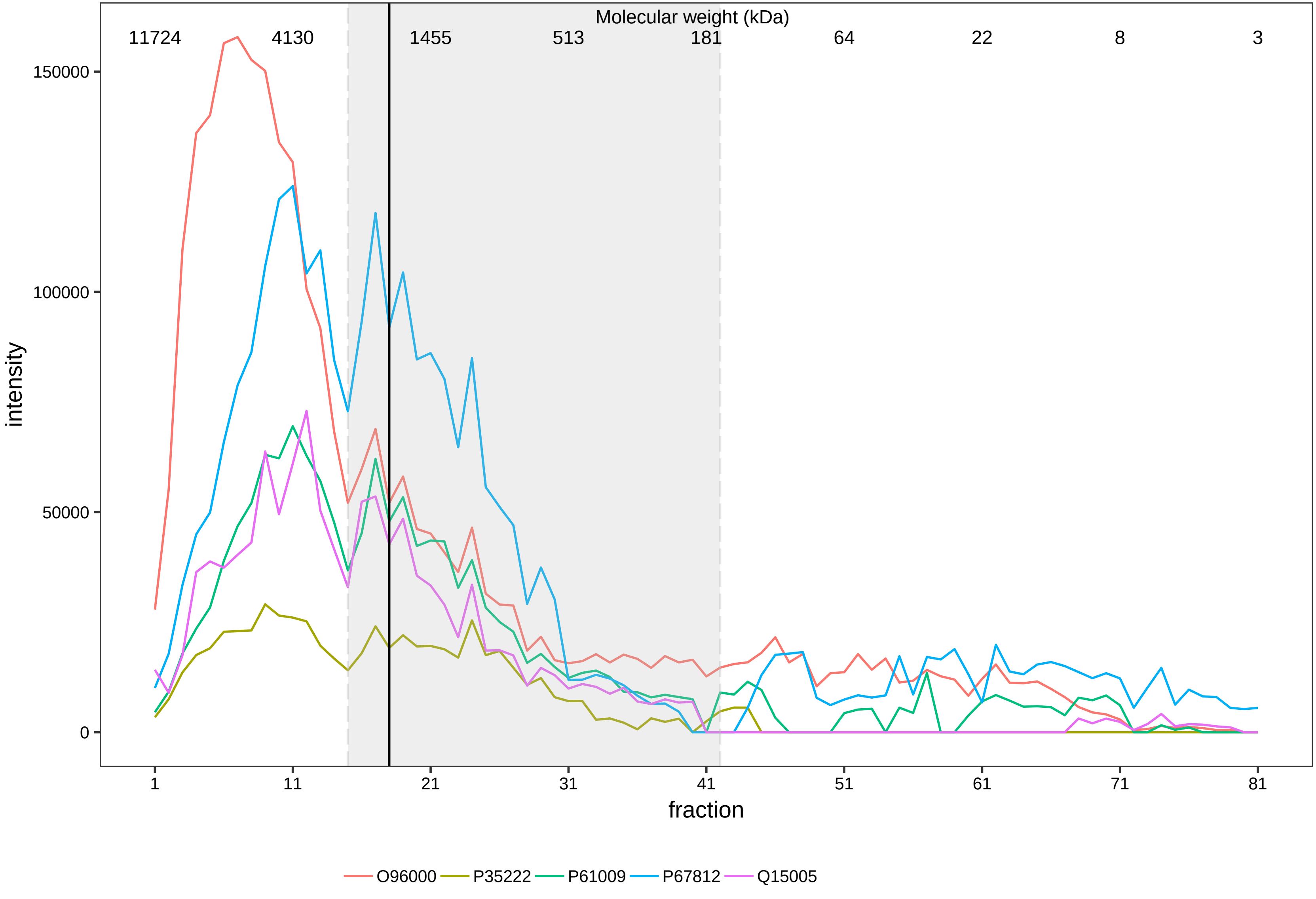

Feature ID 179

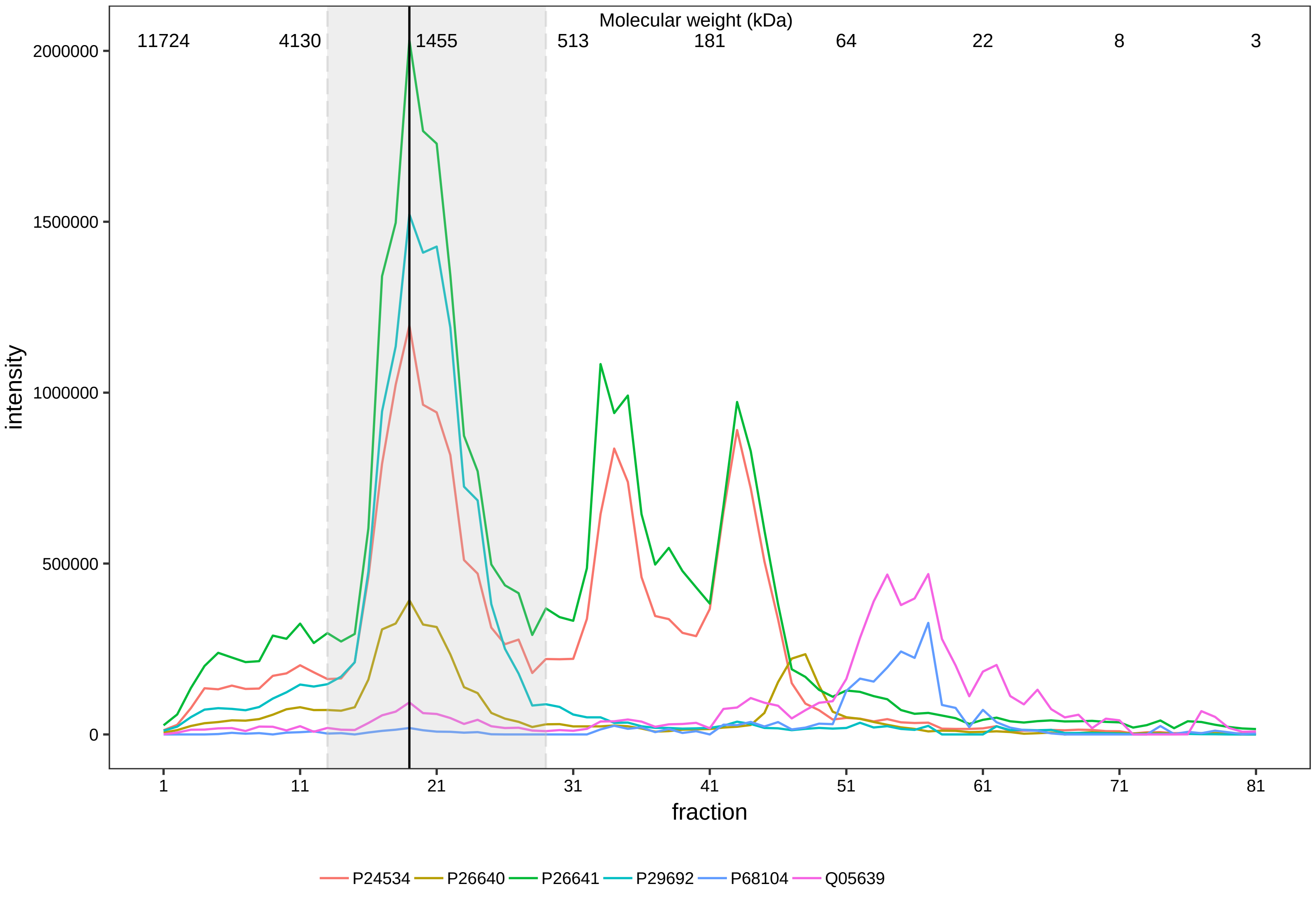

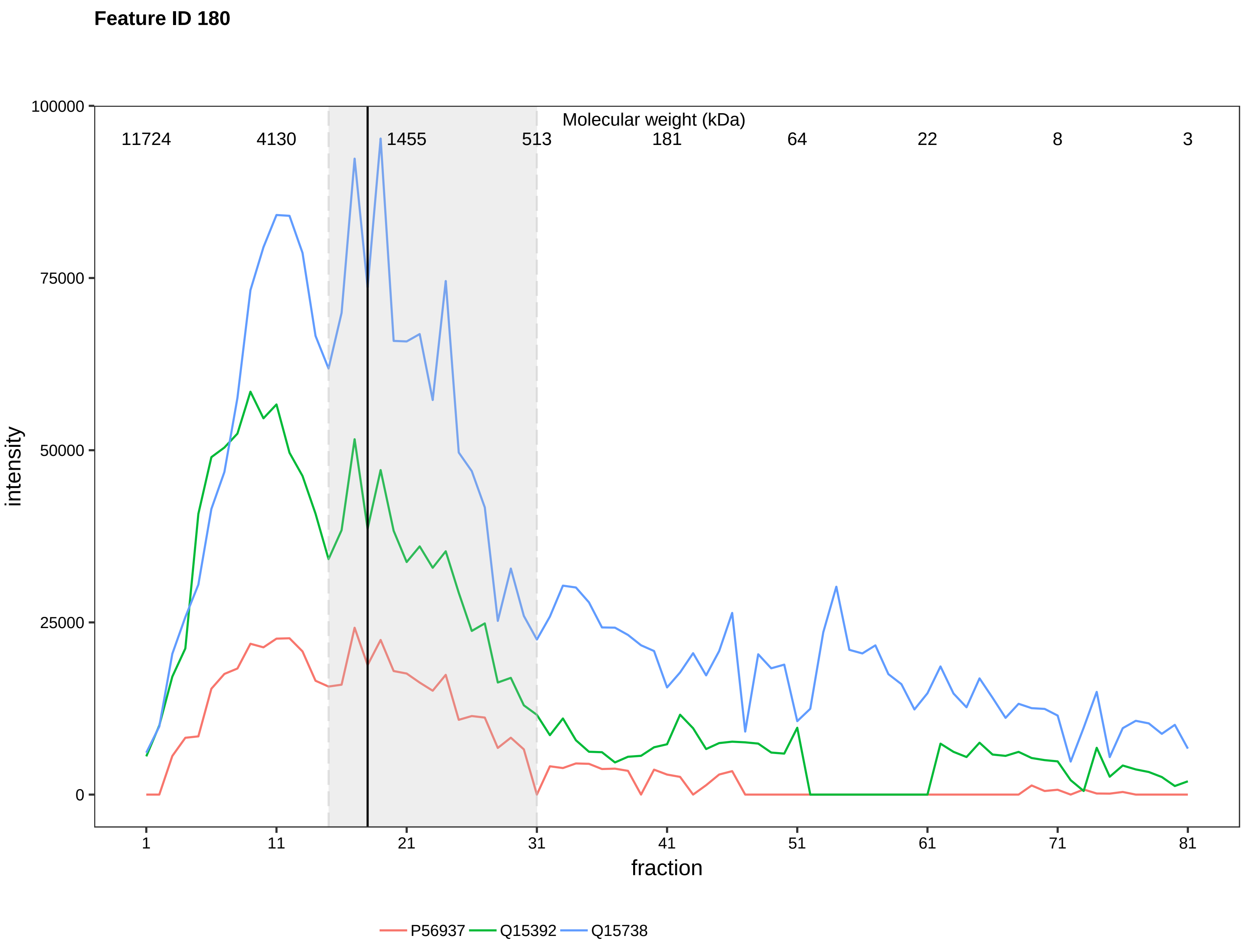
Feature ID 180
Molecular weight (kDa)
11724
4130
1455
513
181
64
22
8
3
100000
75000
50000
25000
0
intensity
1
11
21
31
41
51
61
71
81
fraction
P56937
Q15392
Q15738

Feature ID 181

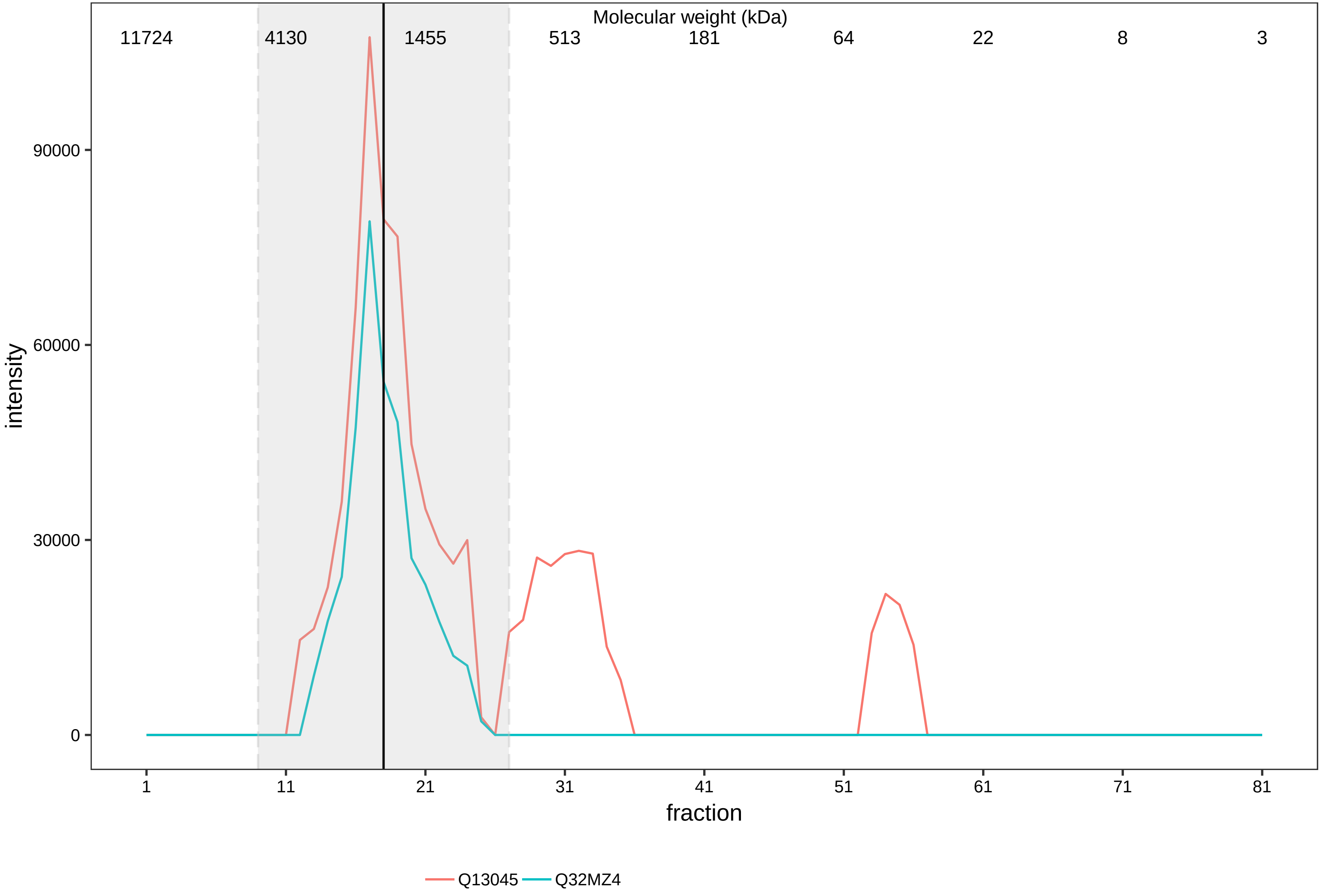

Feature ID 182

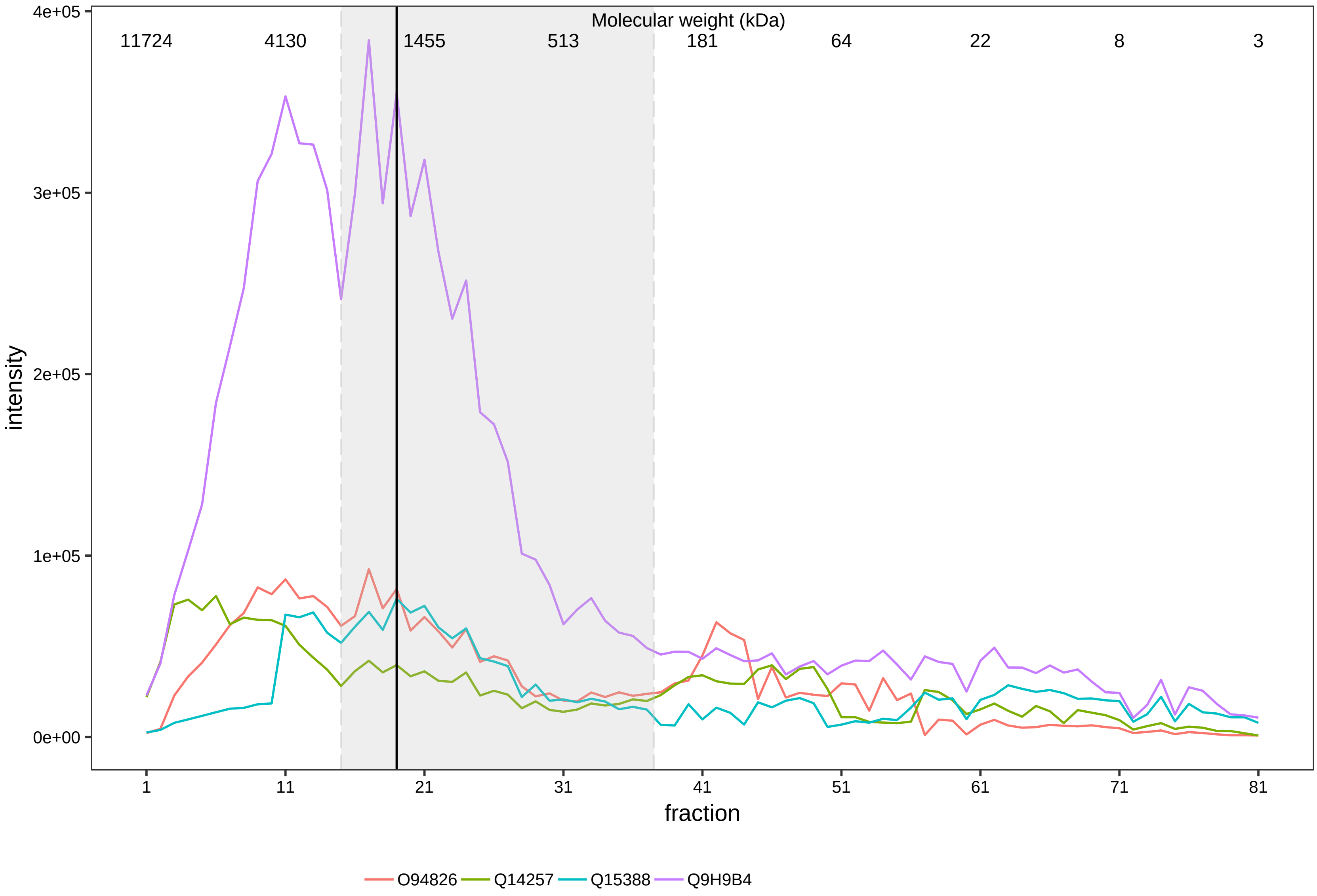

Feature ID 183

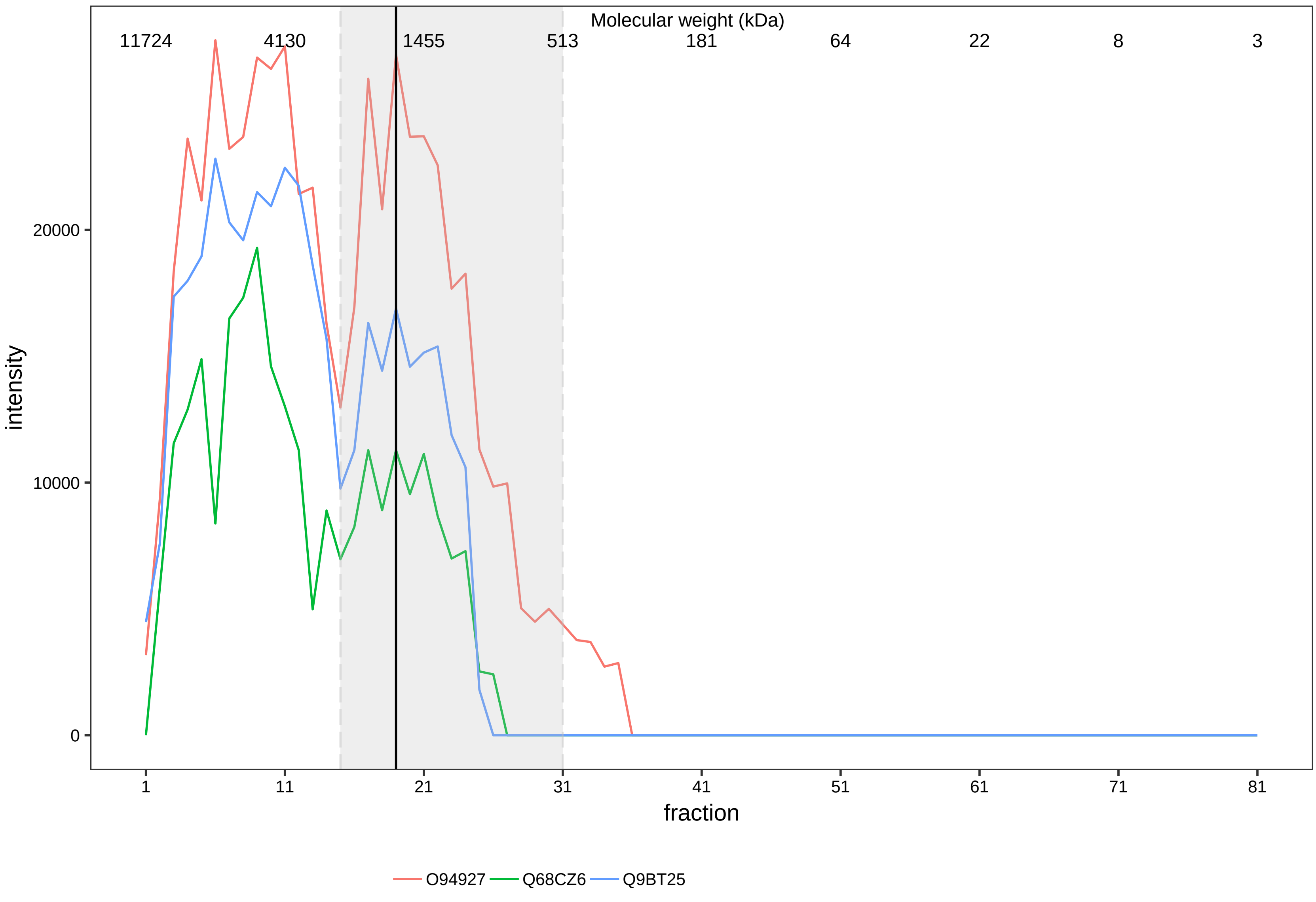

Feature ID 184

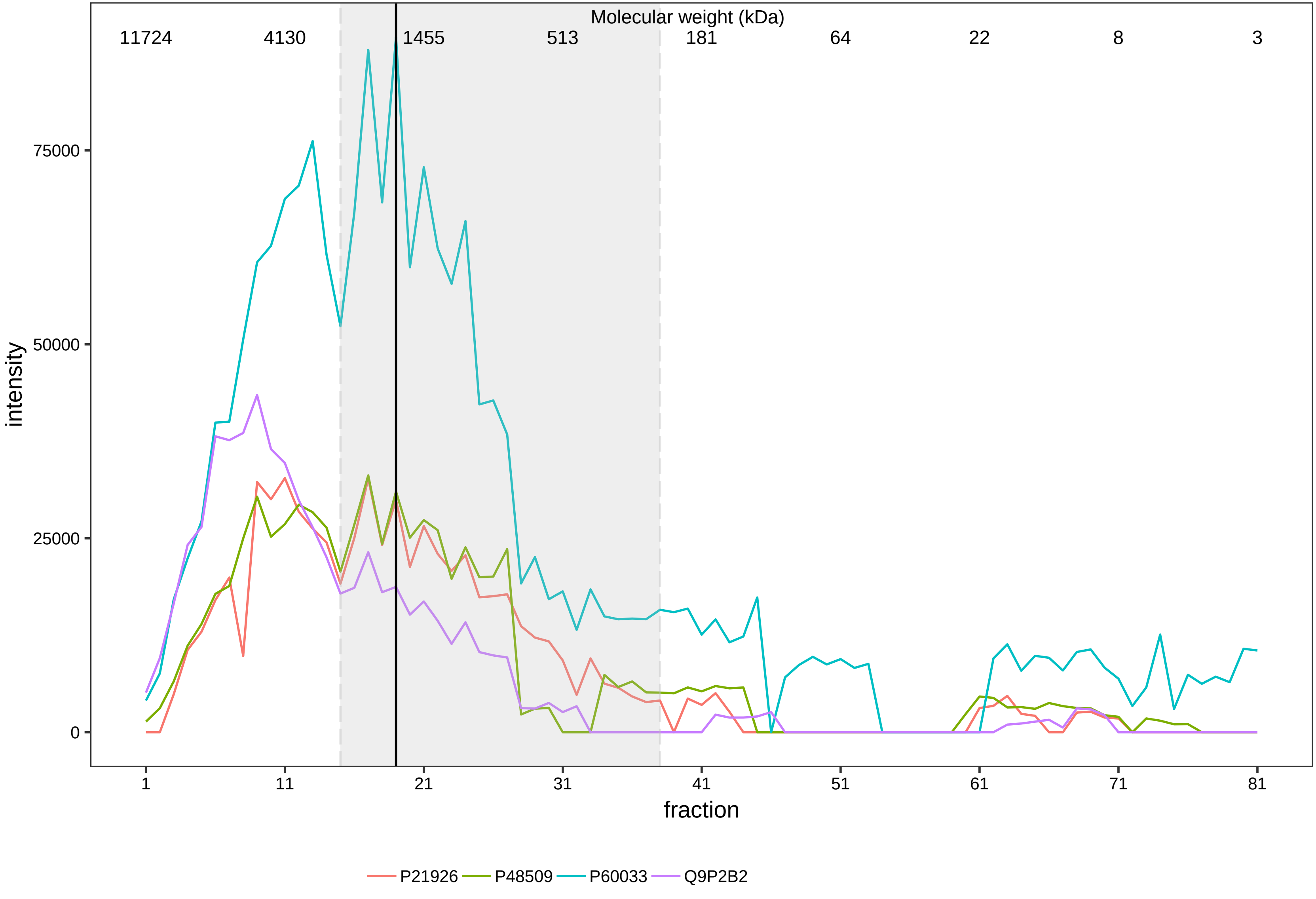

Feature ID 185

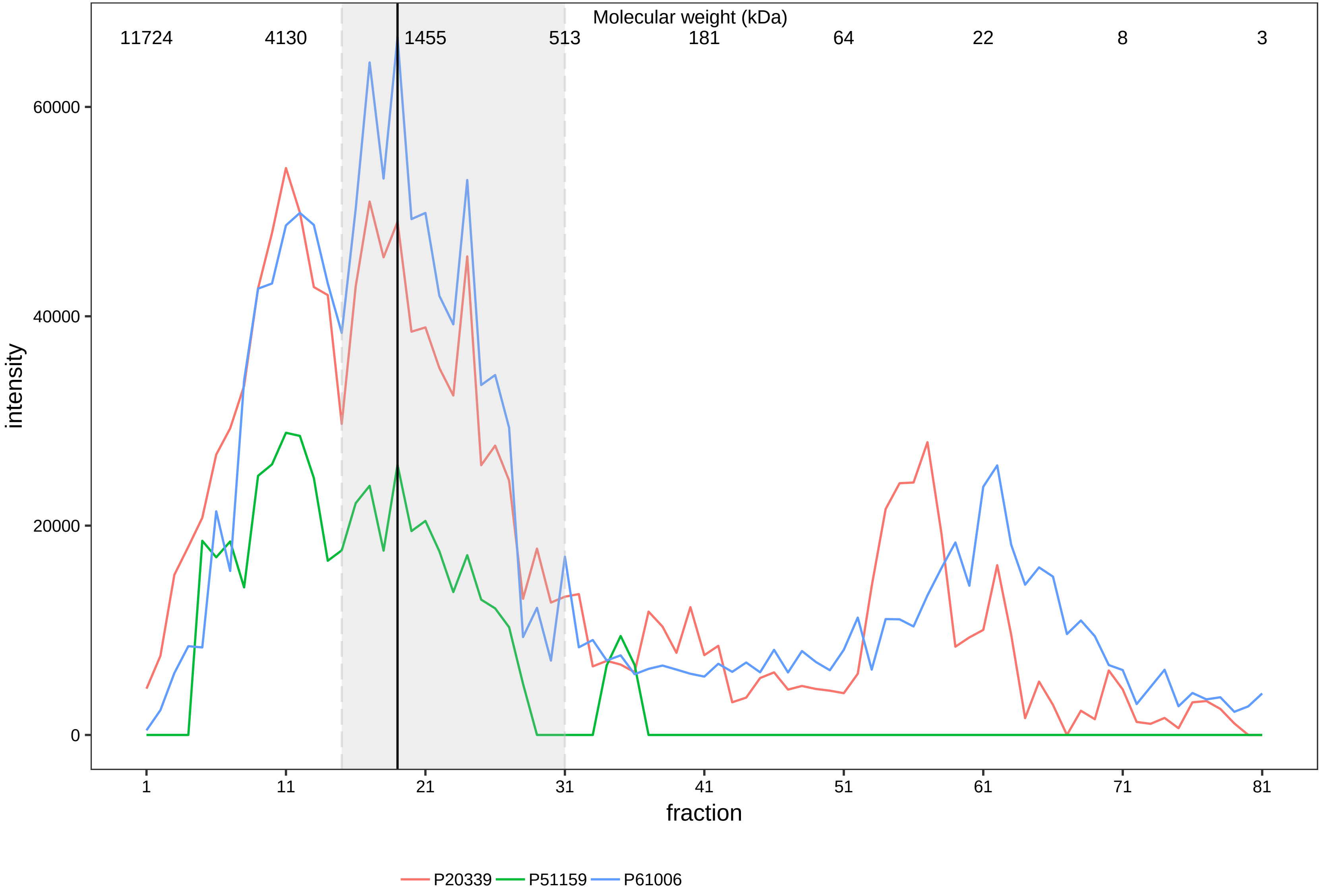

Feature ID 186

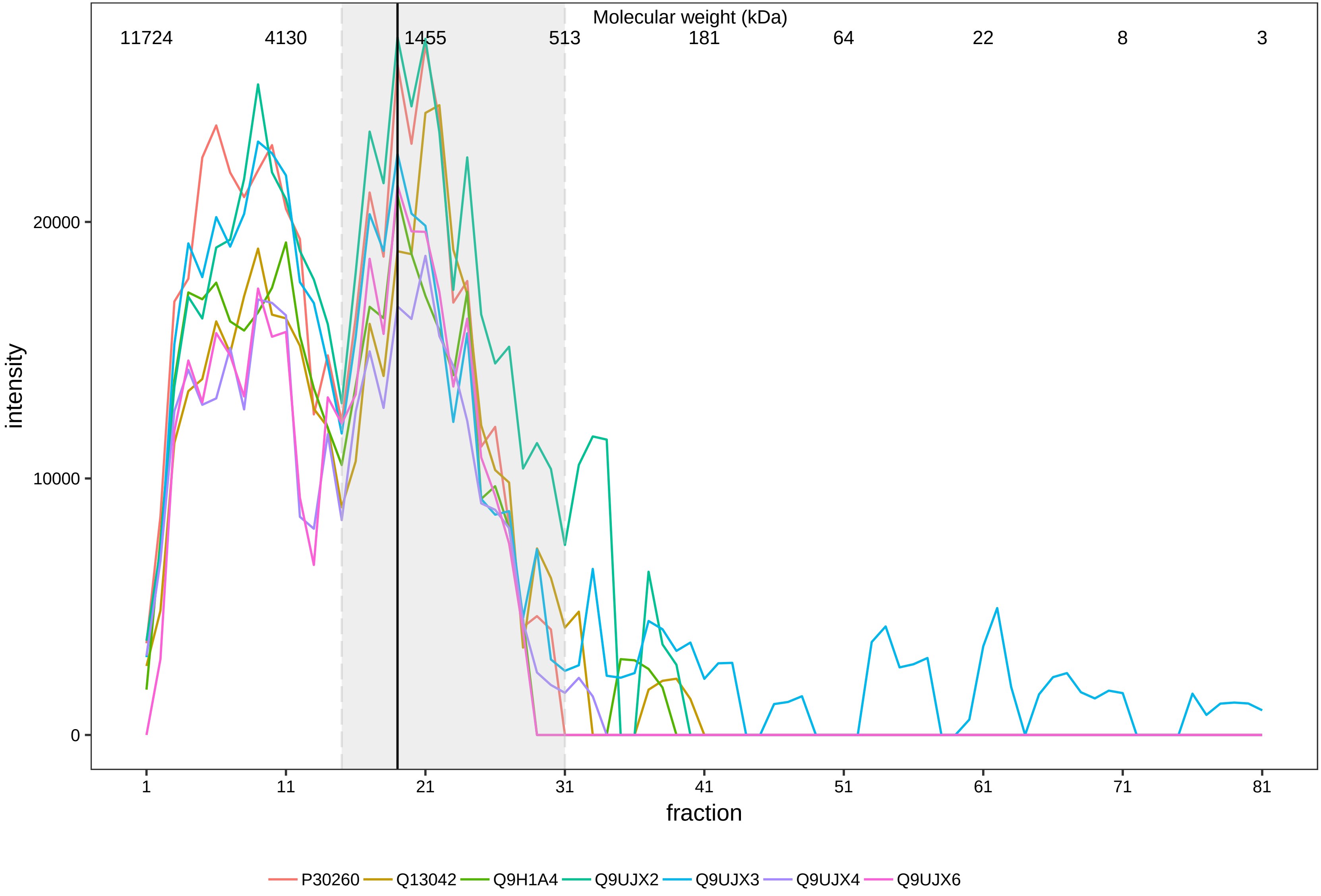

Feature ID 187

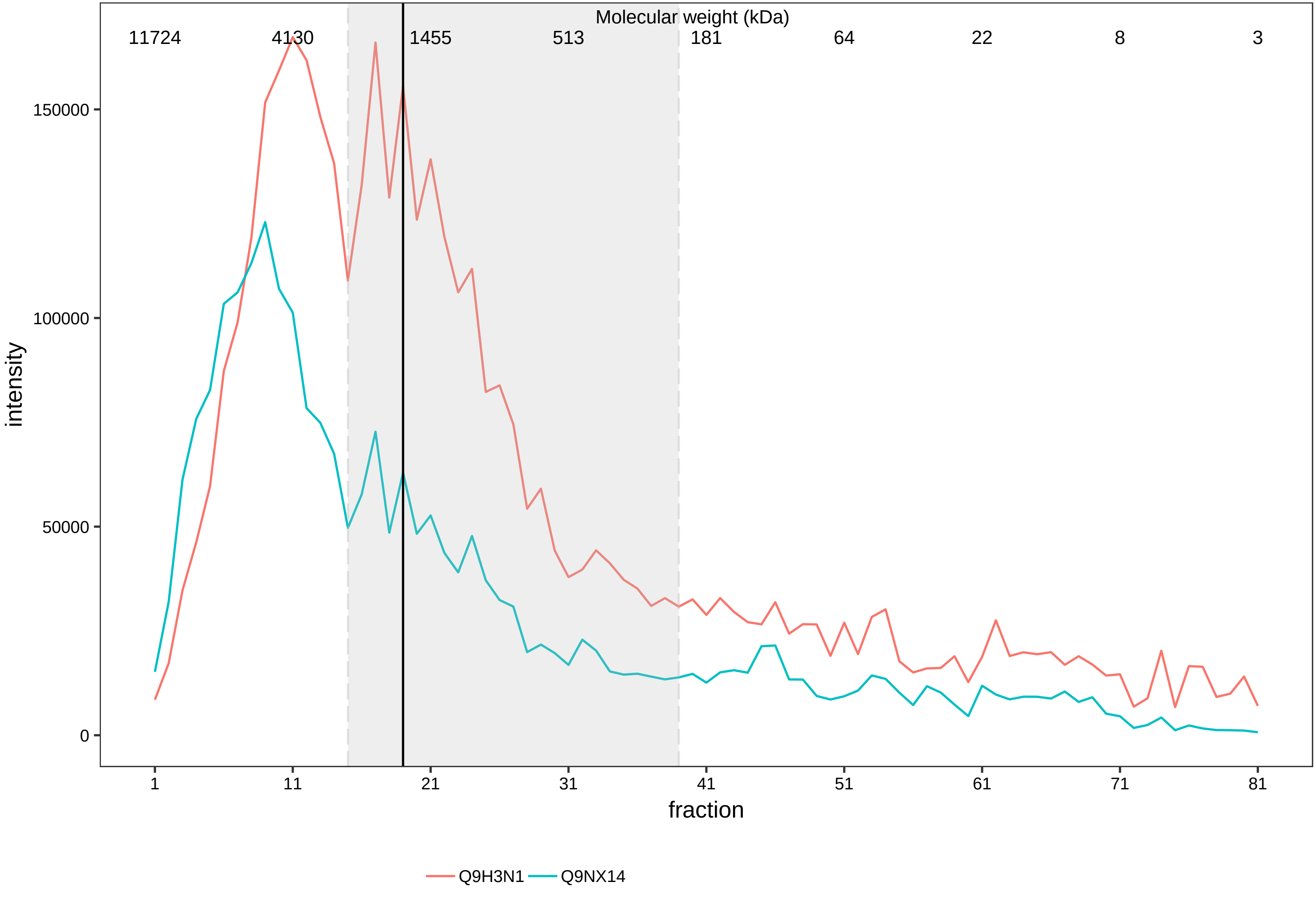

Feature ID 188

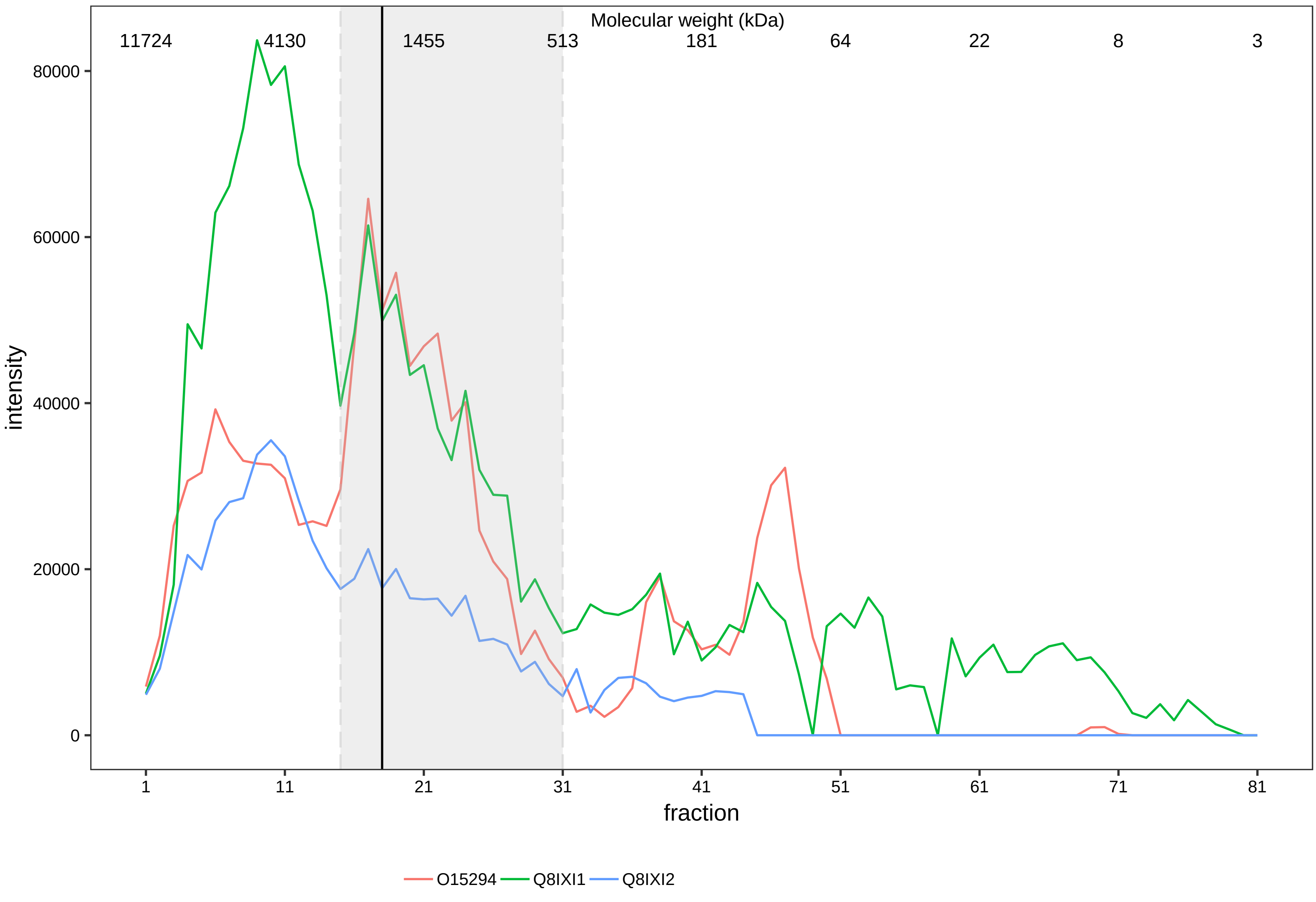

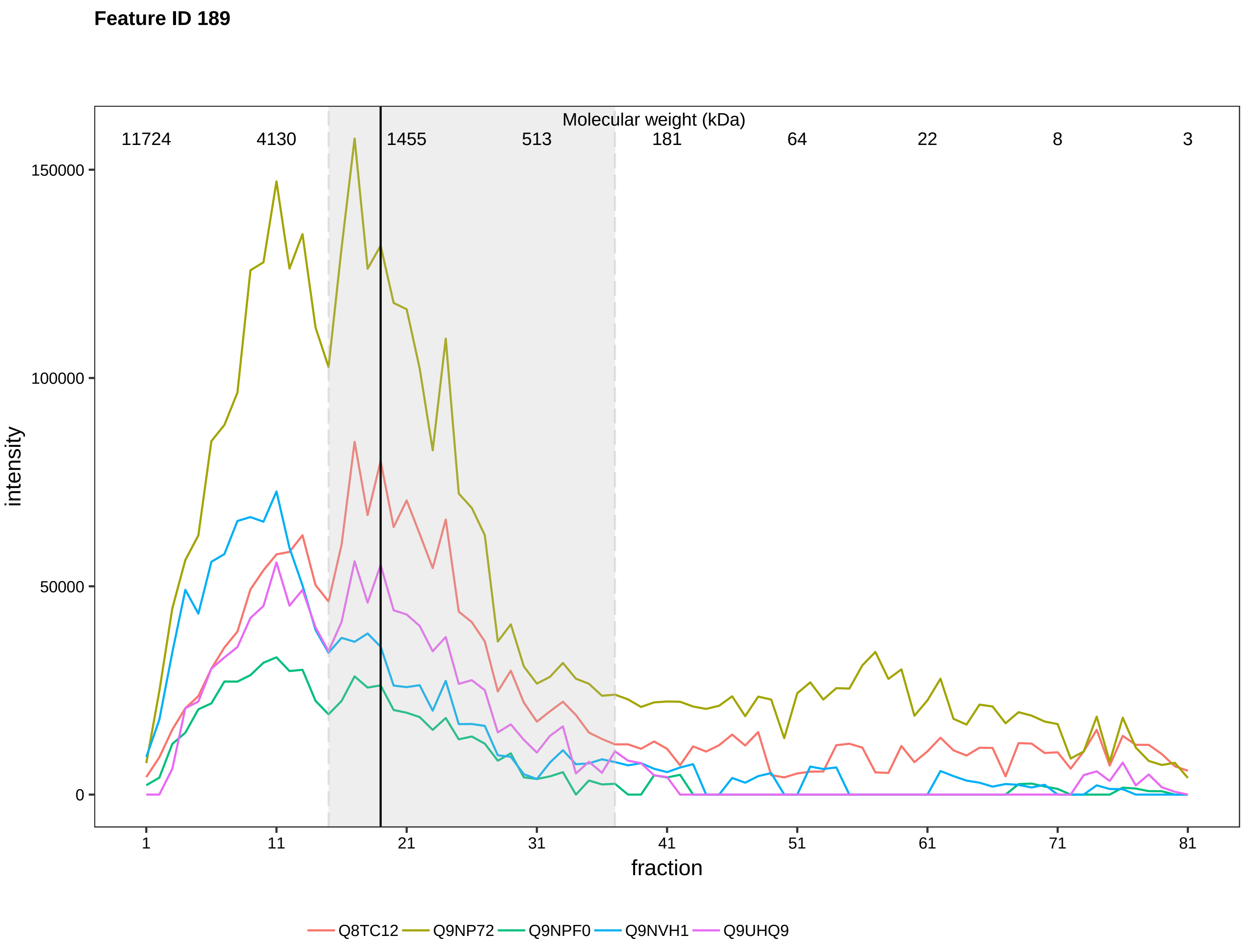
Feature ID 189
Molecular weight (kDa)
11724
4130
1455
513
181
64
22
8
3
150000
100000
50000
0
intensity
1
11
21
31
41
51
61
71
81
fraction
Q8TC12
Q9NP72
Q9NPF0
Q9NVH1
Q9UHQ9

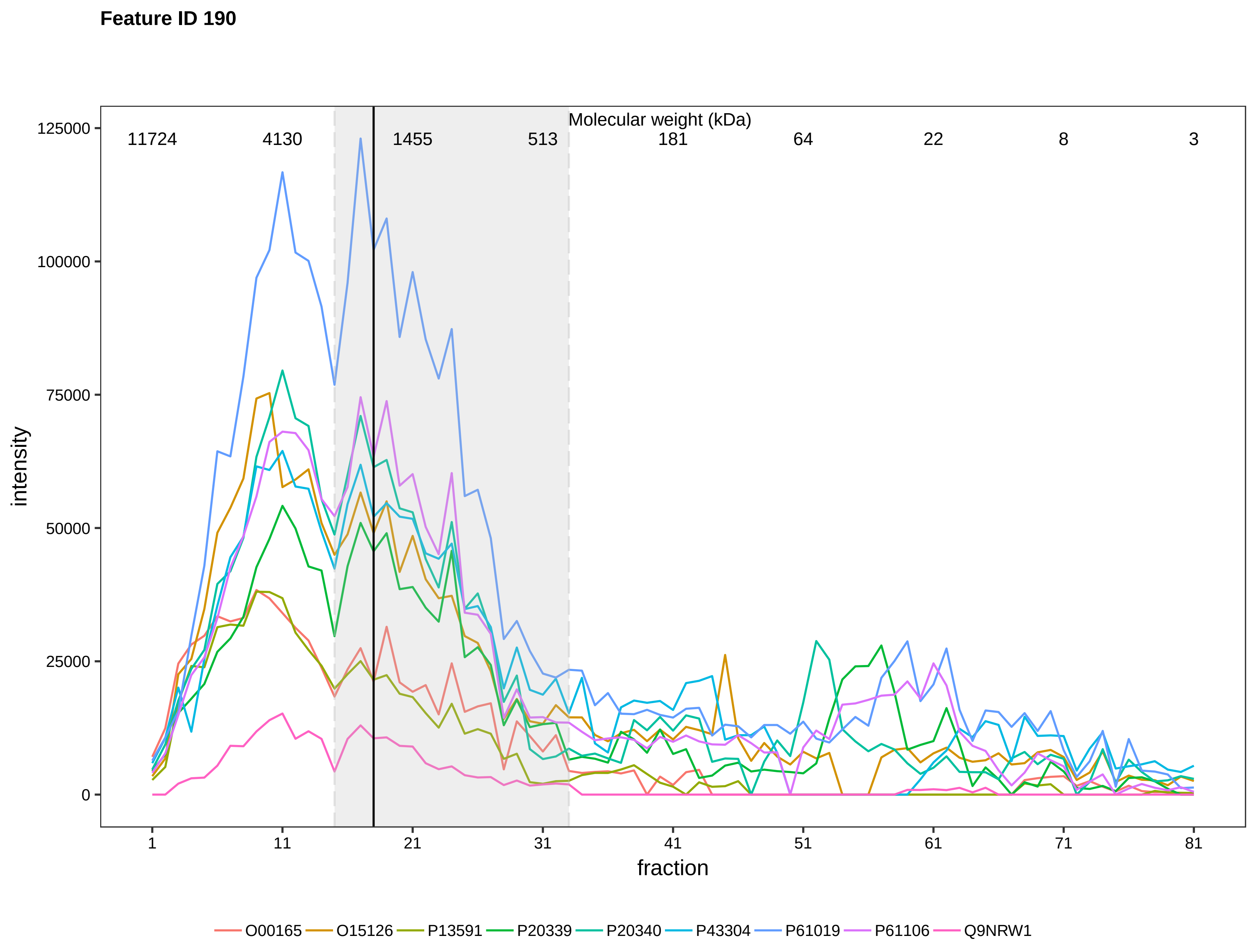

Feature ID 190
Molecular weight (kDa)
11724
4130
1455
513
181
64
22
8
3
intensity
125000
100000
75000
50000
25000
0
fraction
1
11
21
31
41
51
61
71
81
O00165
O15126
P13591
P20339
P20340
P43304
P61019
P61106
Q9NRW1

Feature ID 191

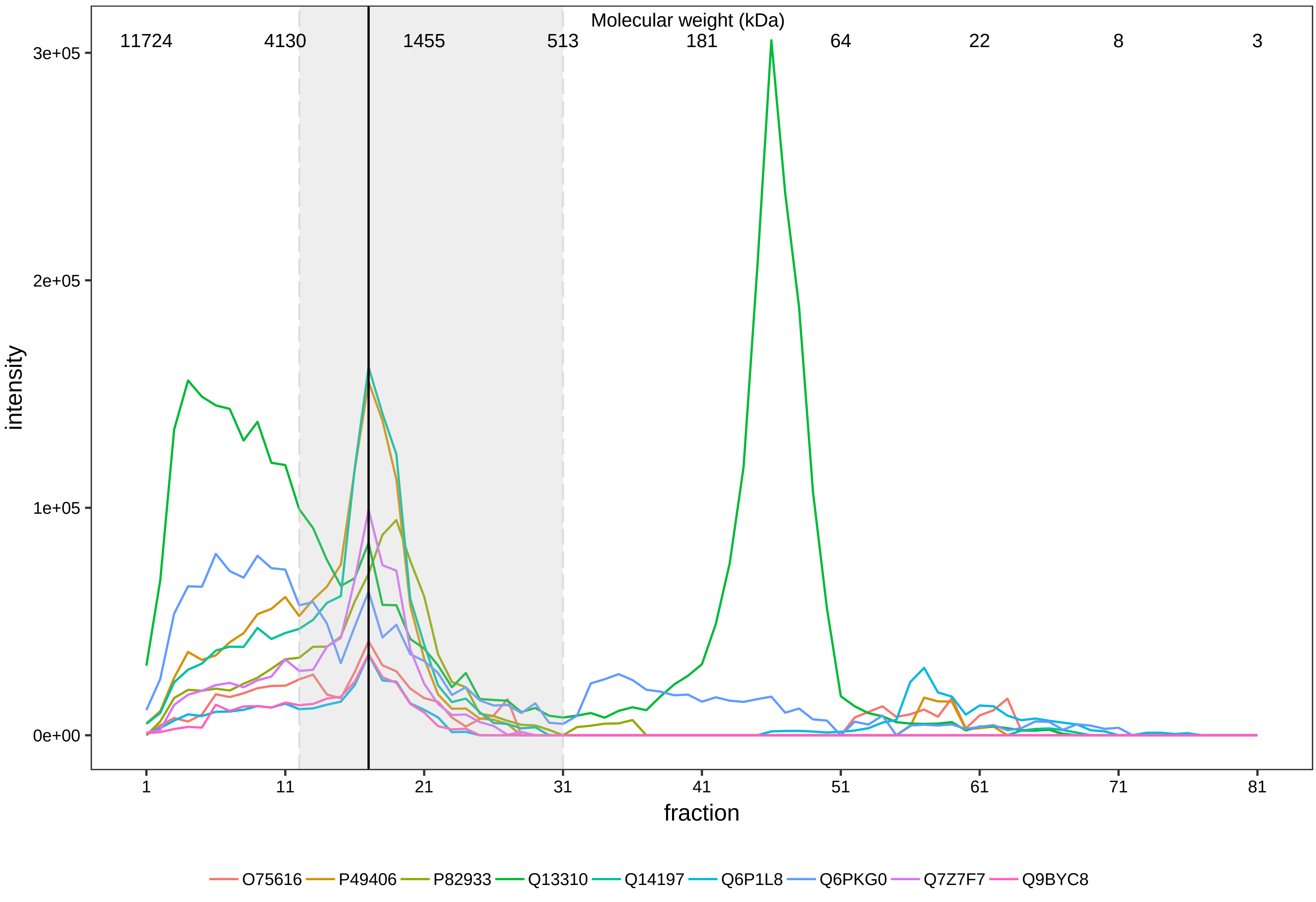

Feature ID 192

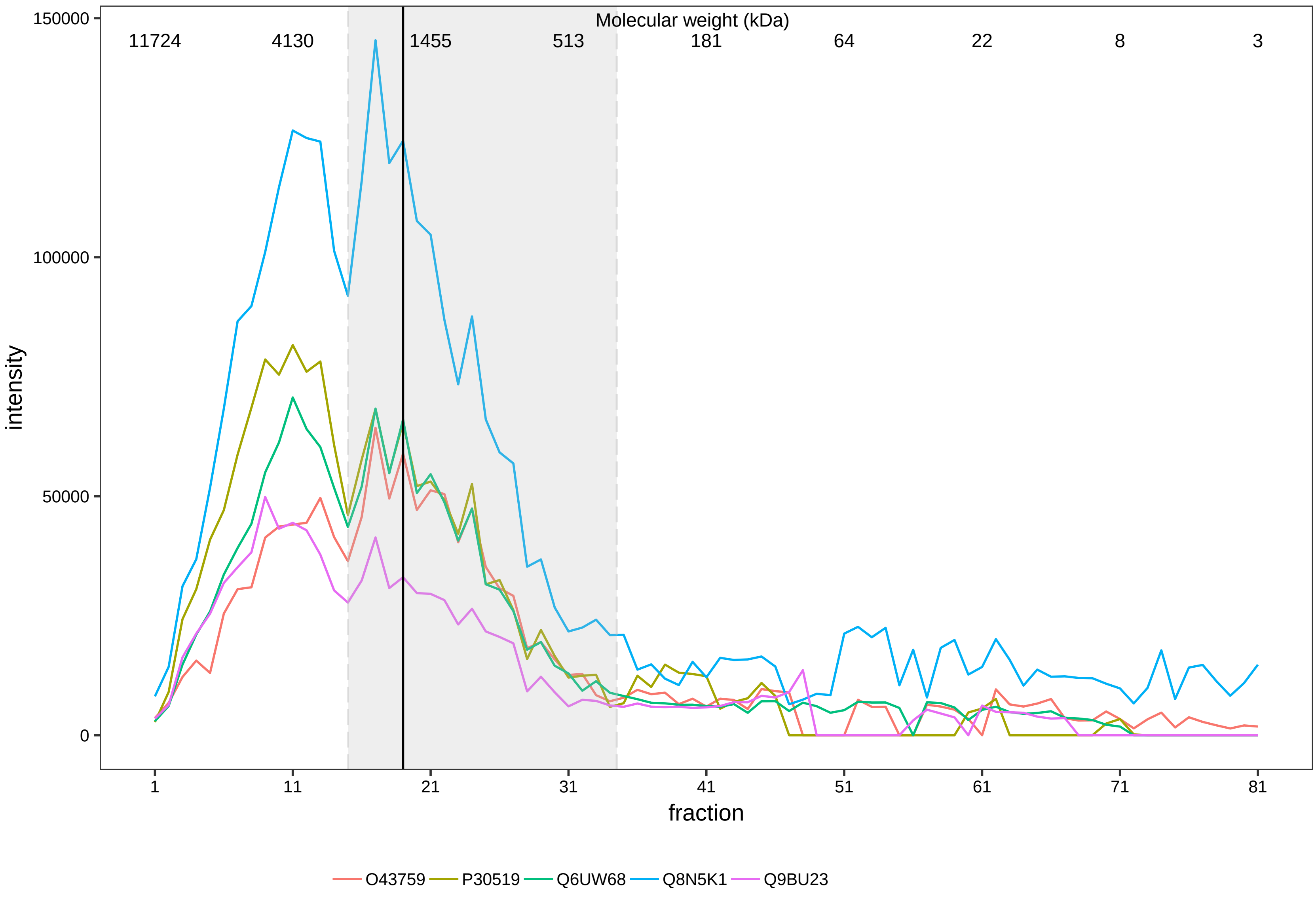

Feature ID 193

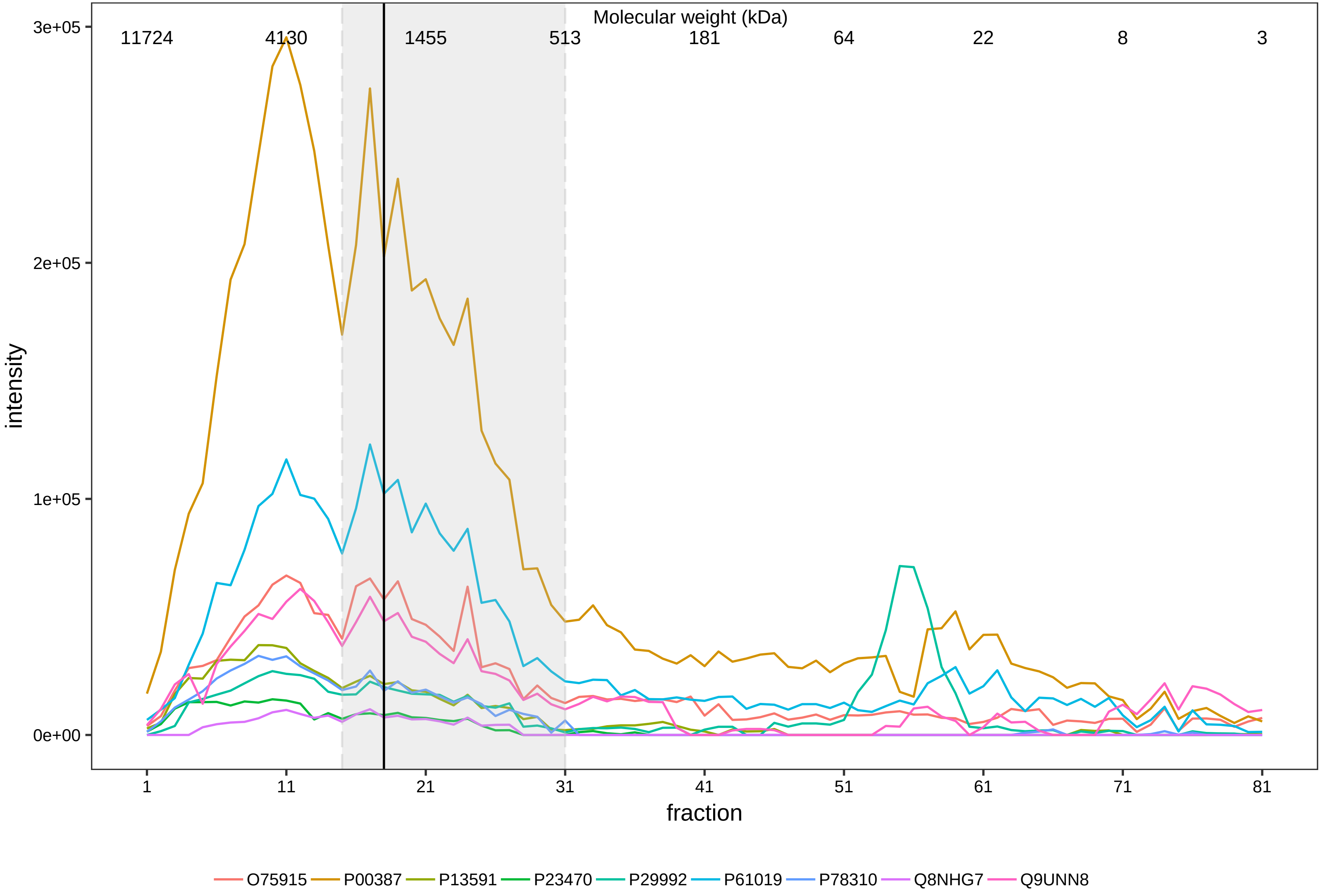

Feature ID 194

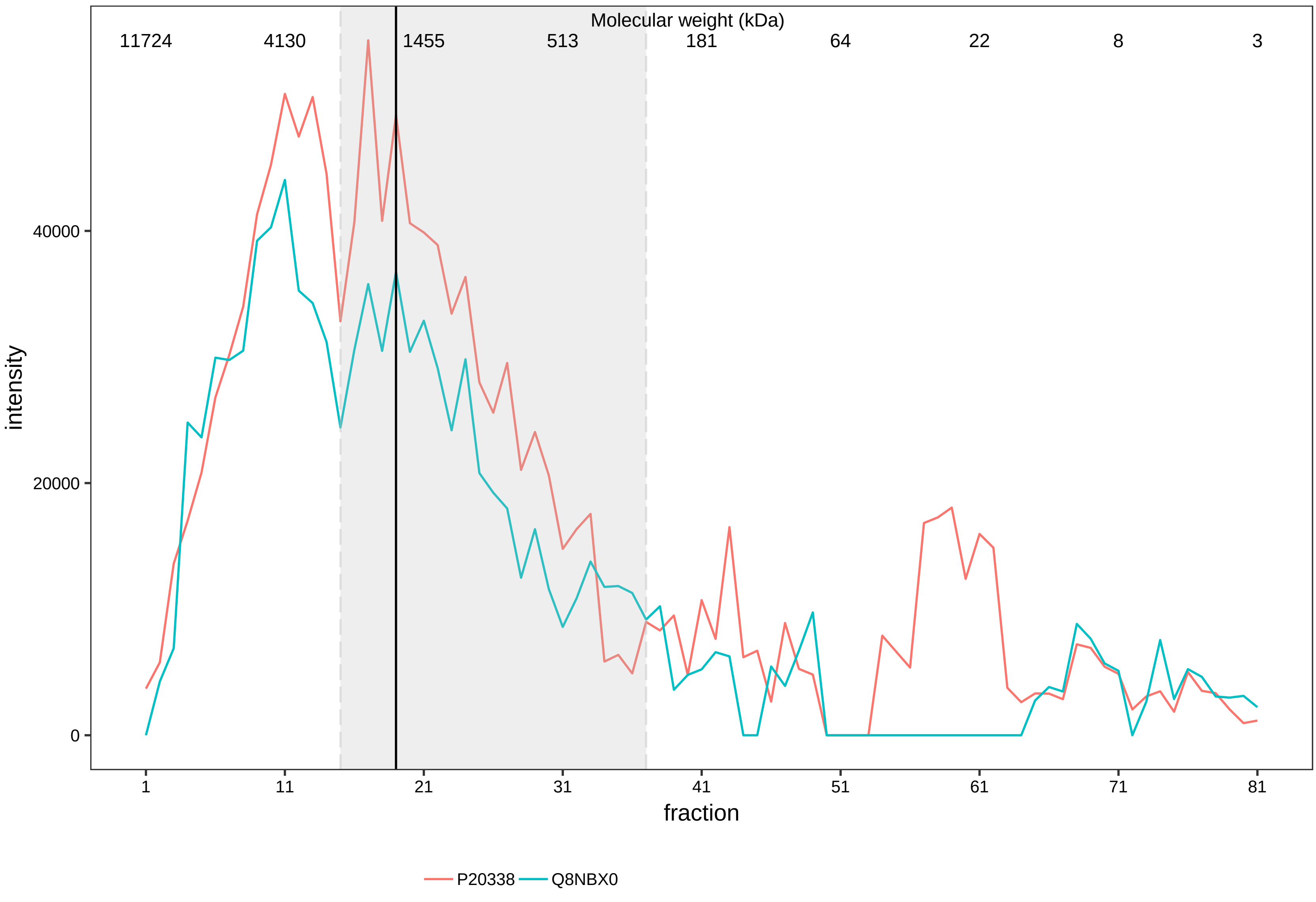

Feature ID 195

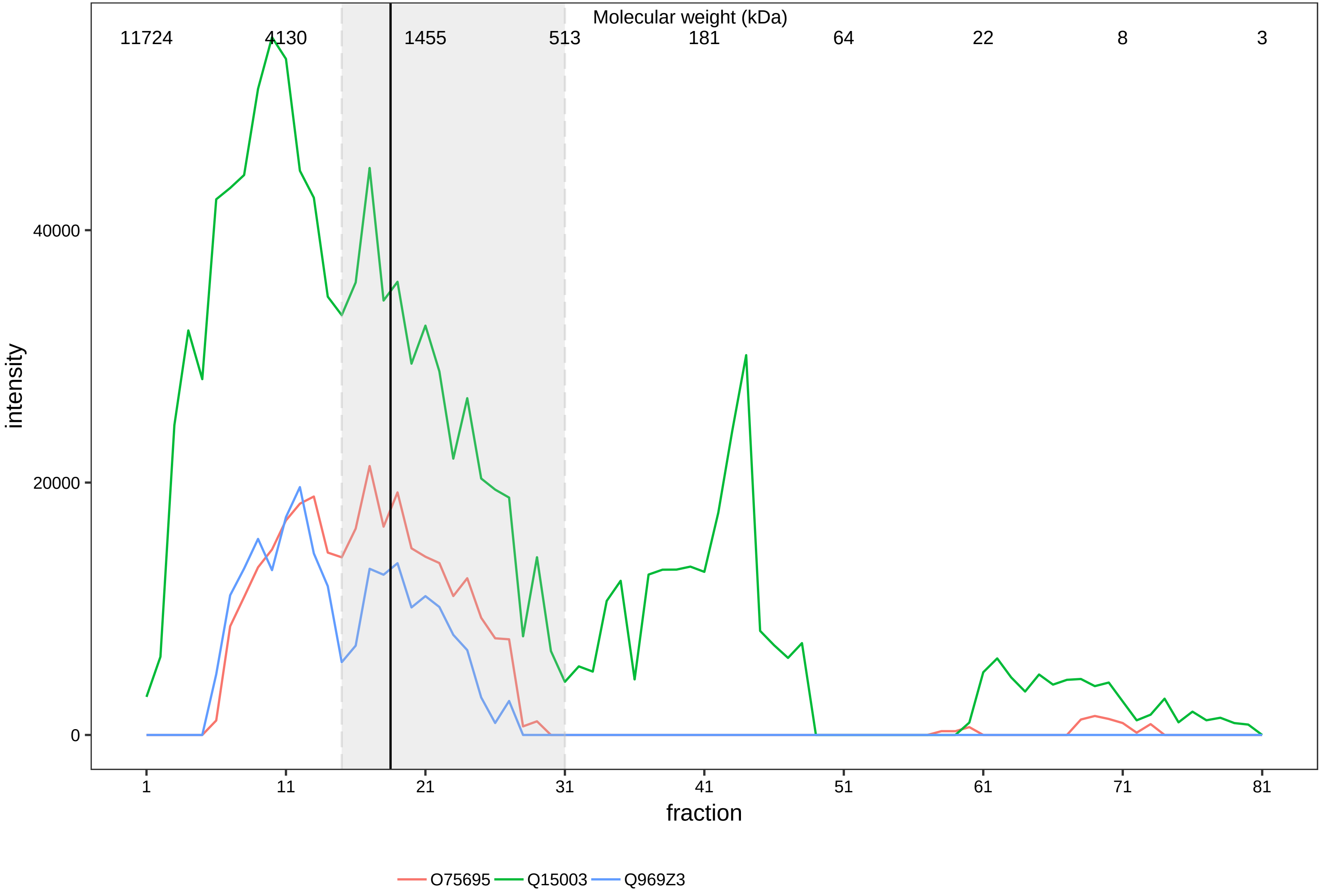

Feature ID 196

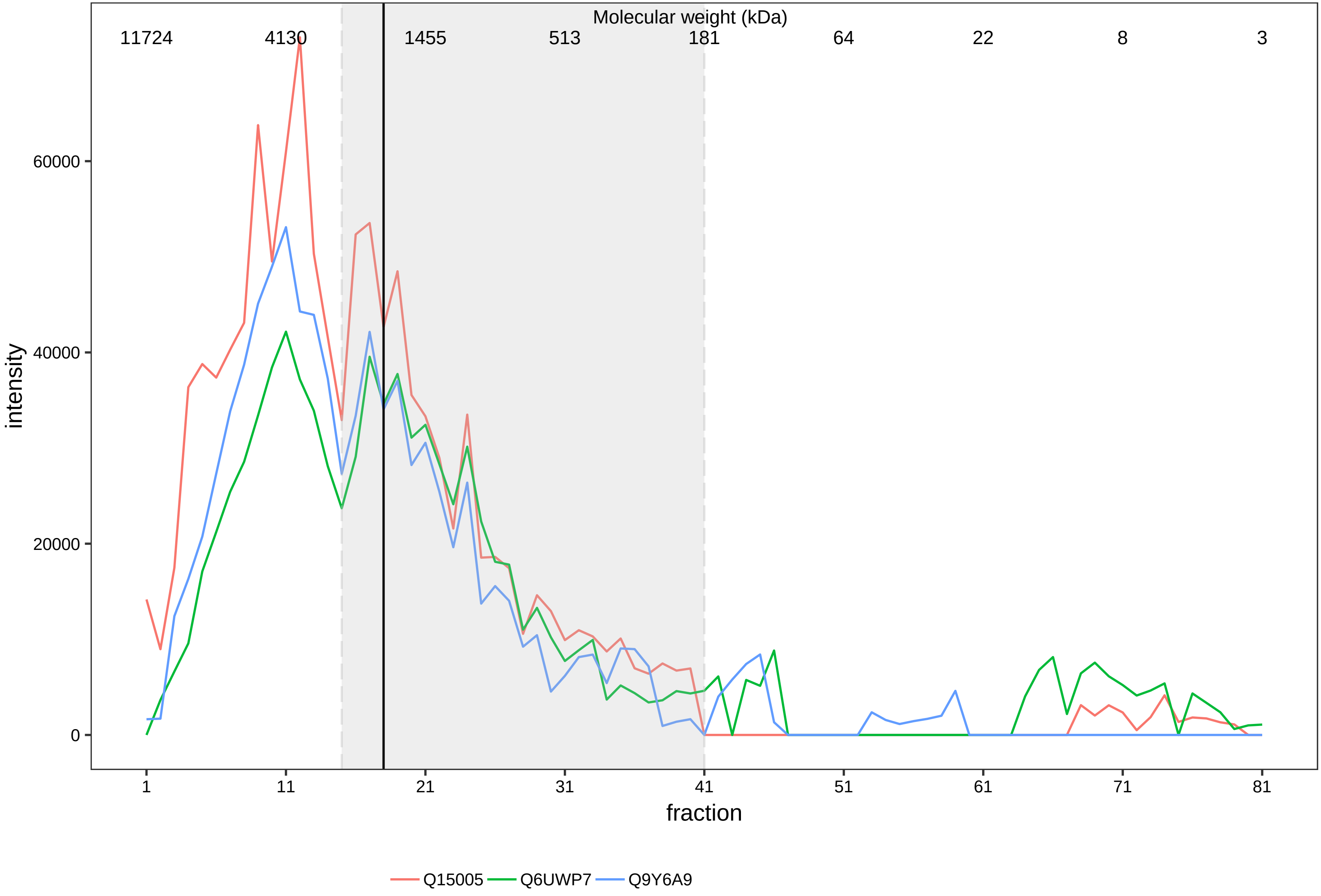

Feature ID 197

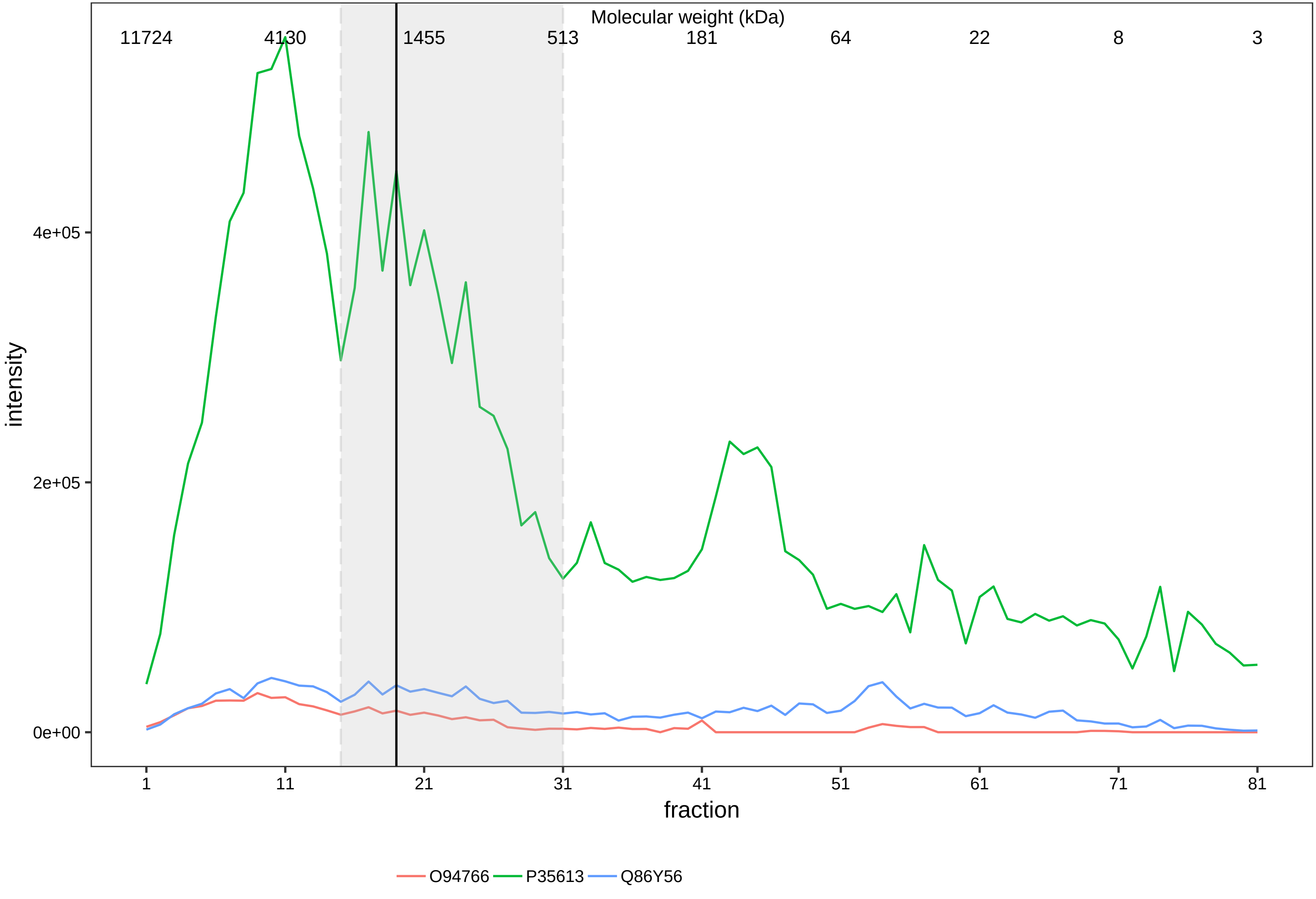

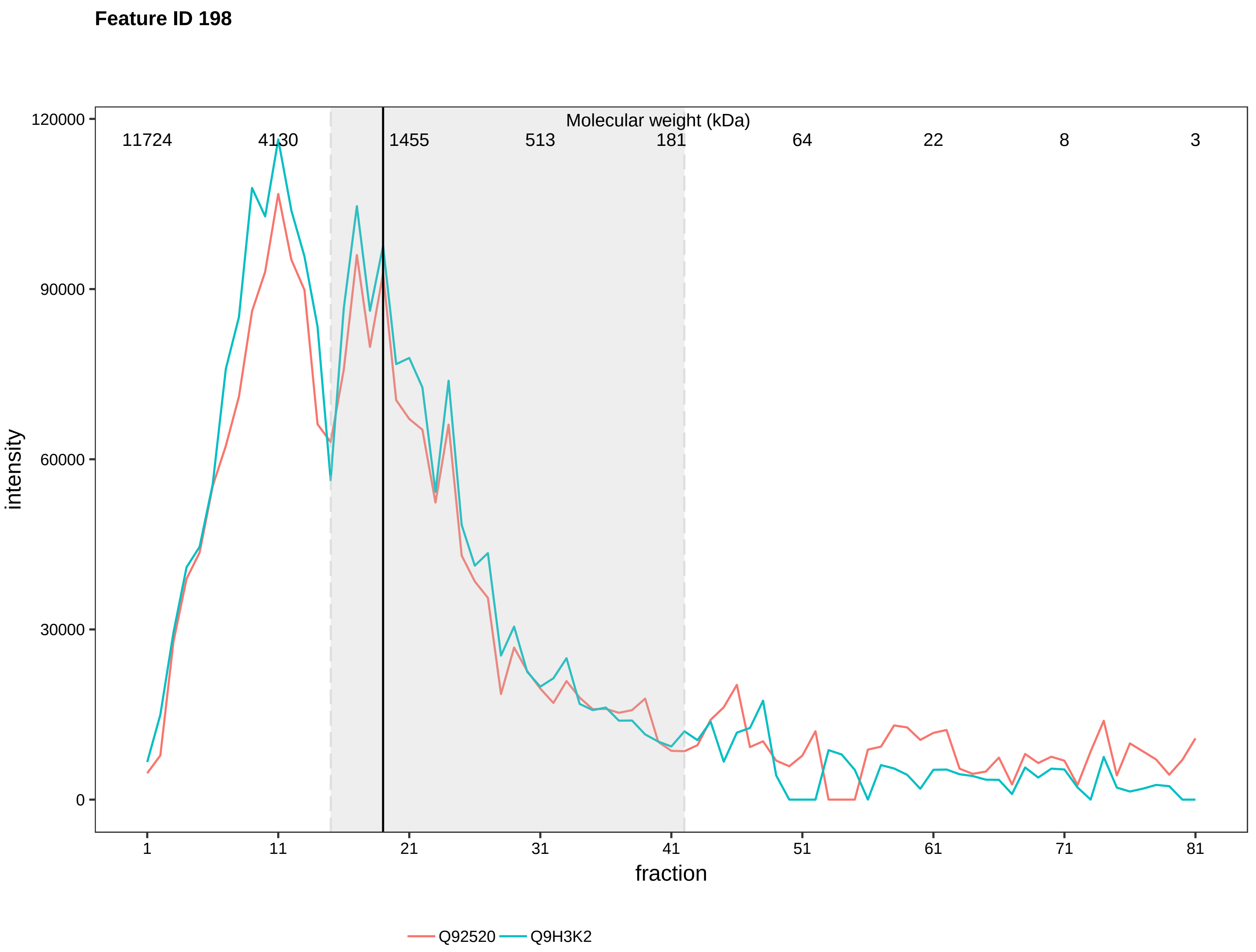
Feature ID 198
Molecular weight (kDa)
11724
4130
1455
513
181
64
22
8
3
120000
90000
60000
30000
0
intensity
1
11
21
31
41
51
61
71
81
fraction
Q92520
Q9H3K2

Feature ID 199

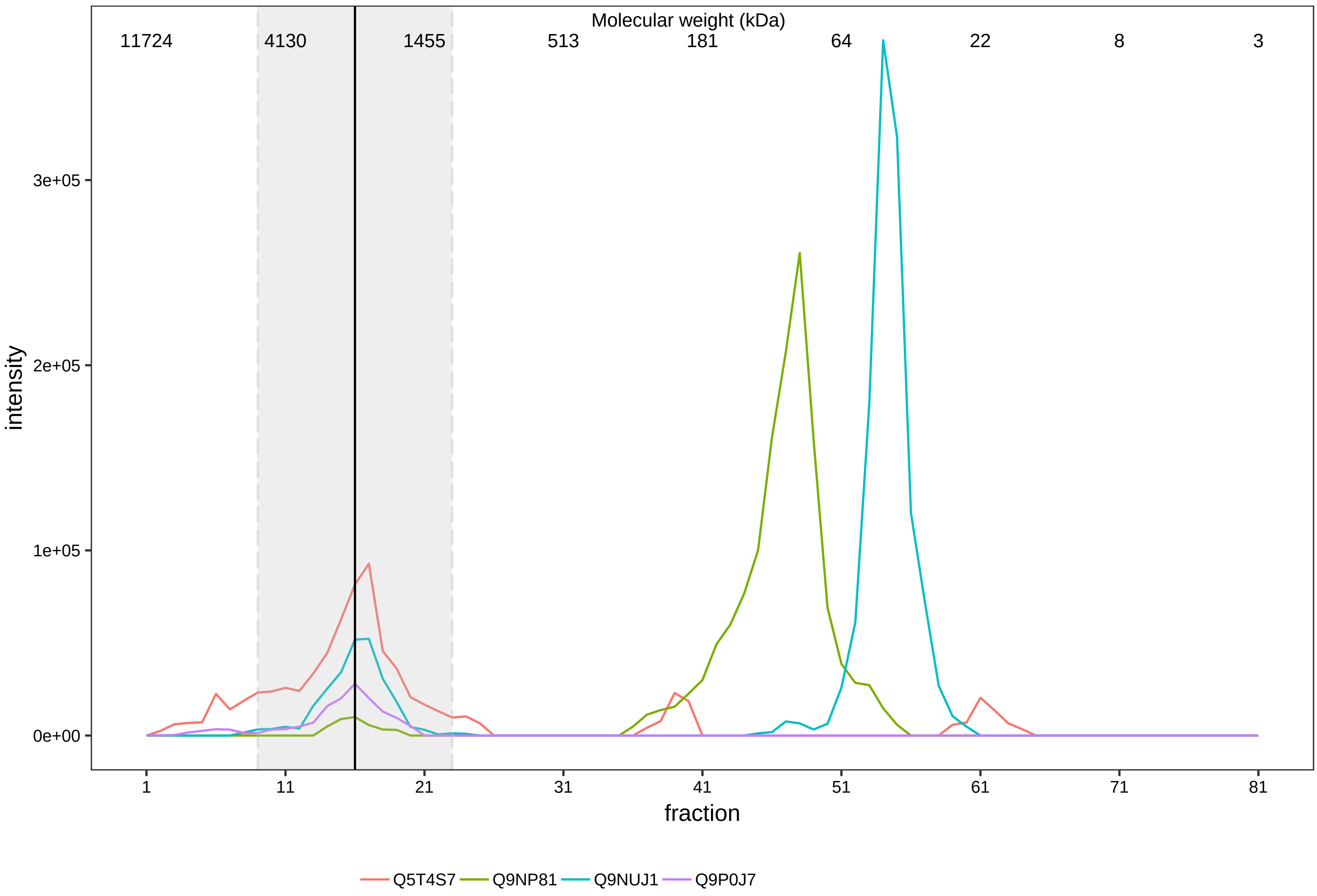

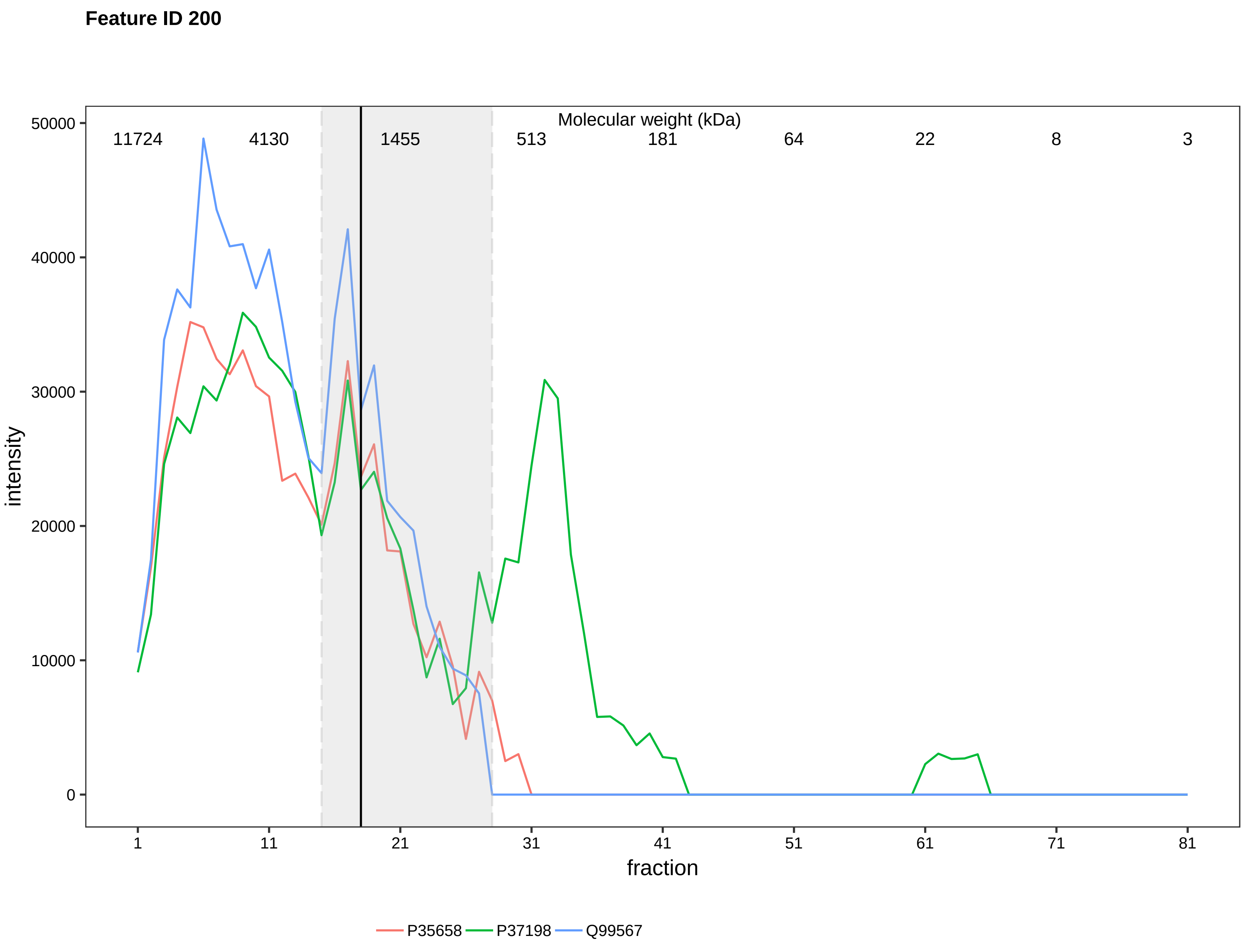

Feature ID 200
Molecular weight (kDa)
11724
4130
1455
513
181
64
22
8
3
intensity
50000
40000
30000
20000
10000
0
1
11
21
31
41
51
61
71
81
fraction
P35658
P37198
Q99567

Feature ID 201

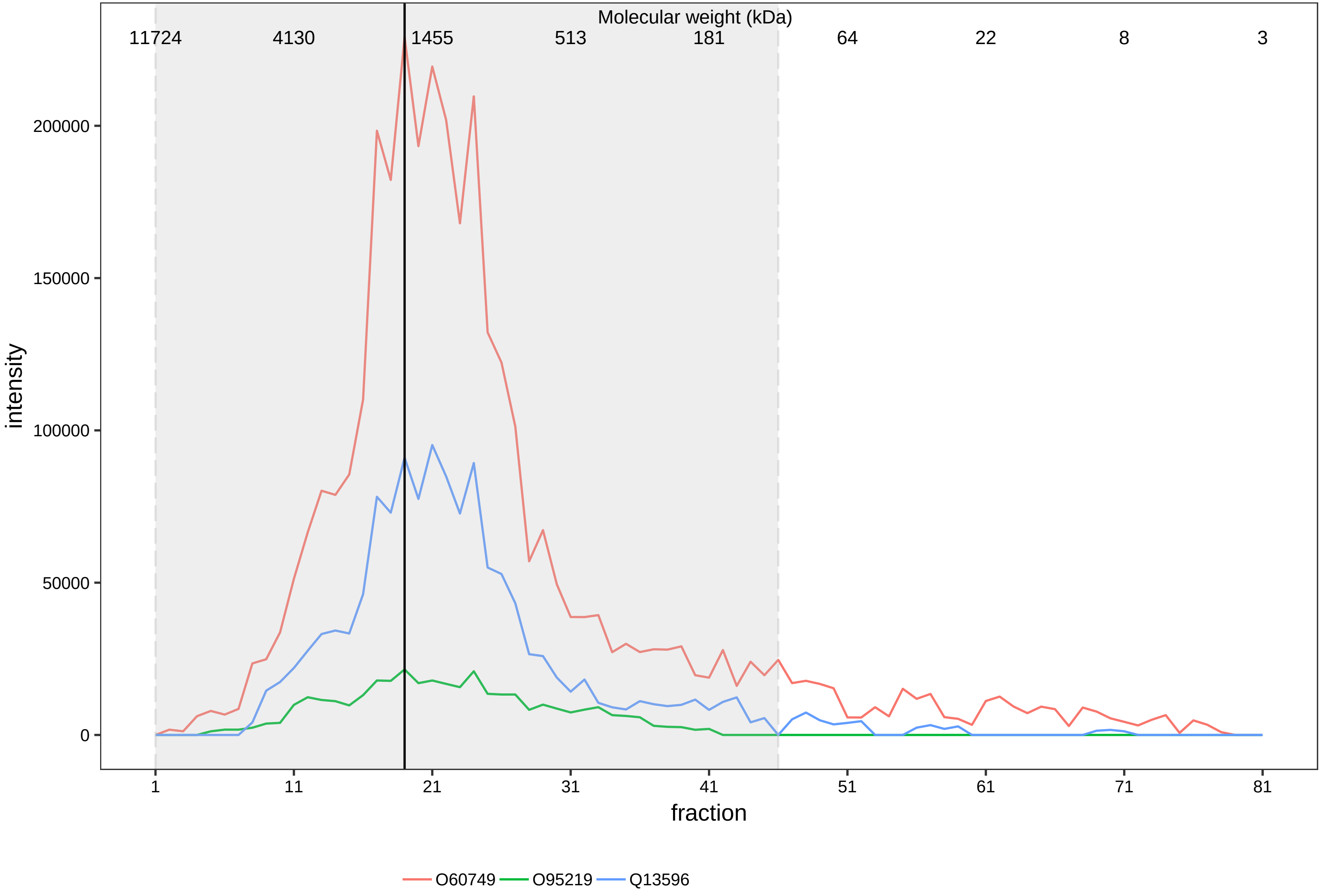

Feature ID 202

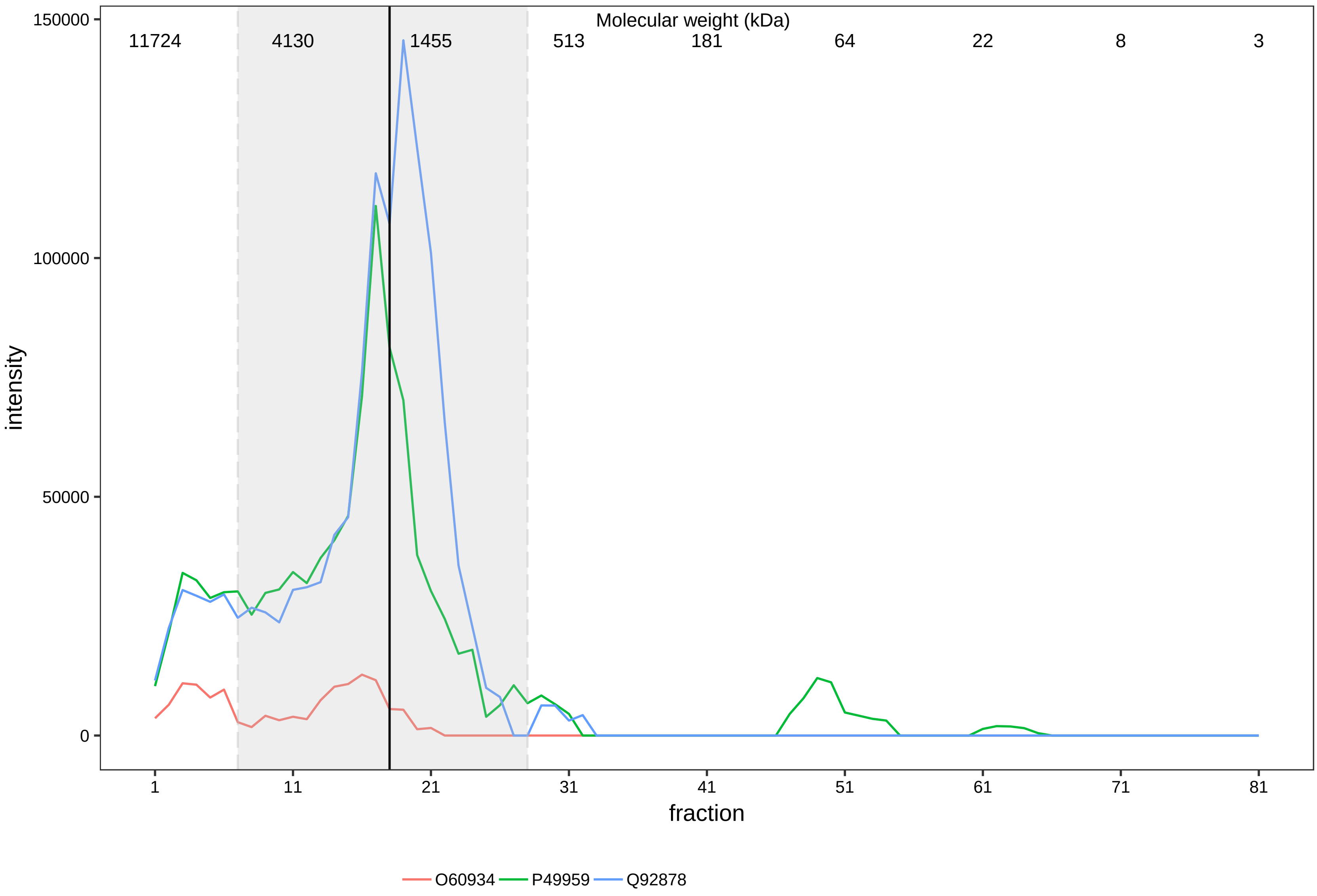

Feature ID 203

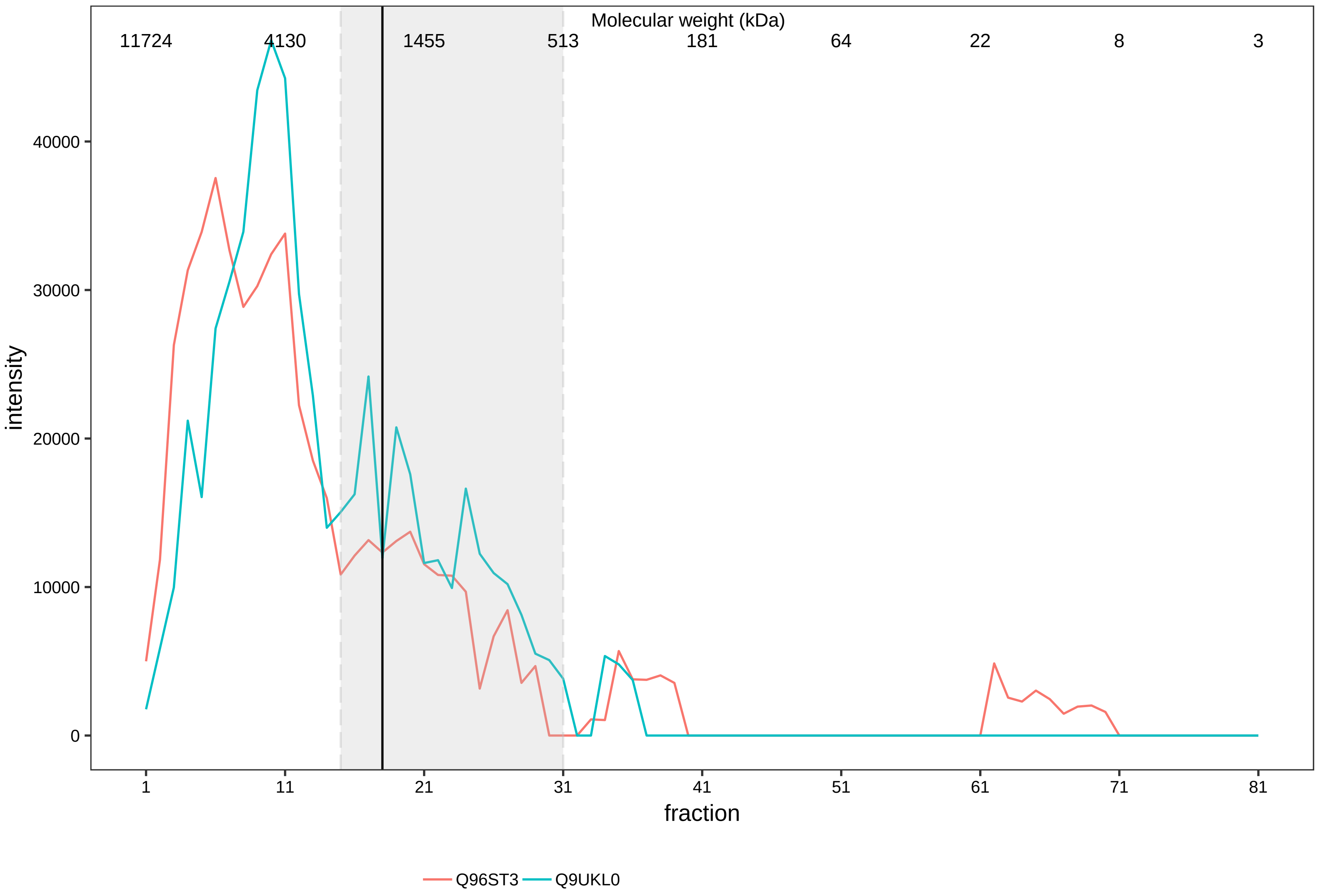

Feature ID 204

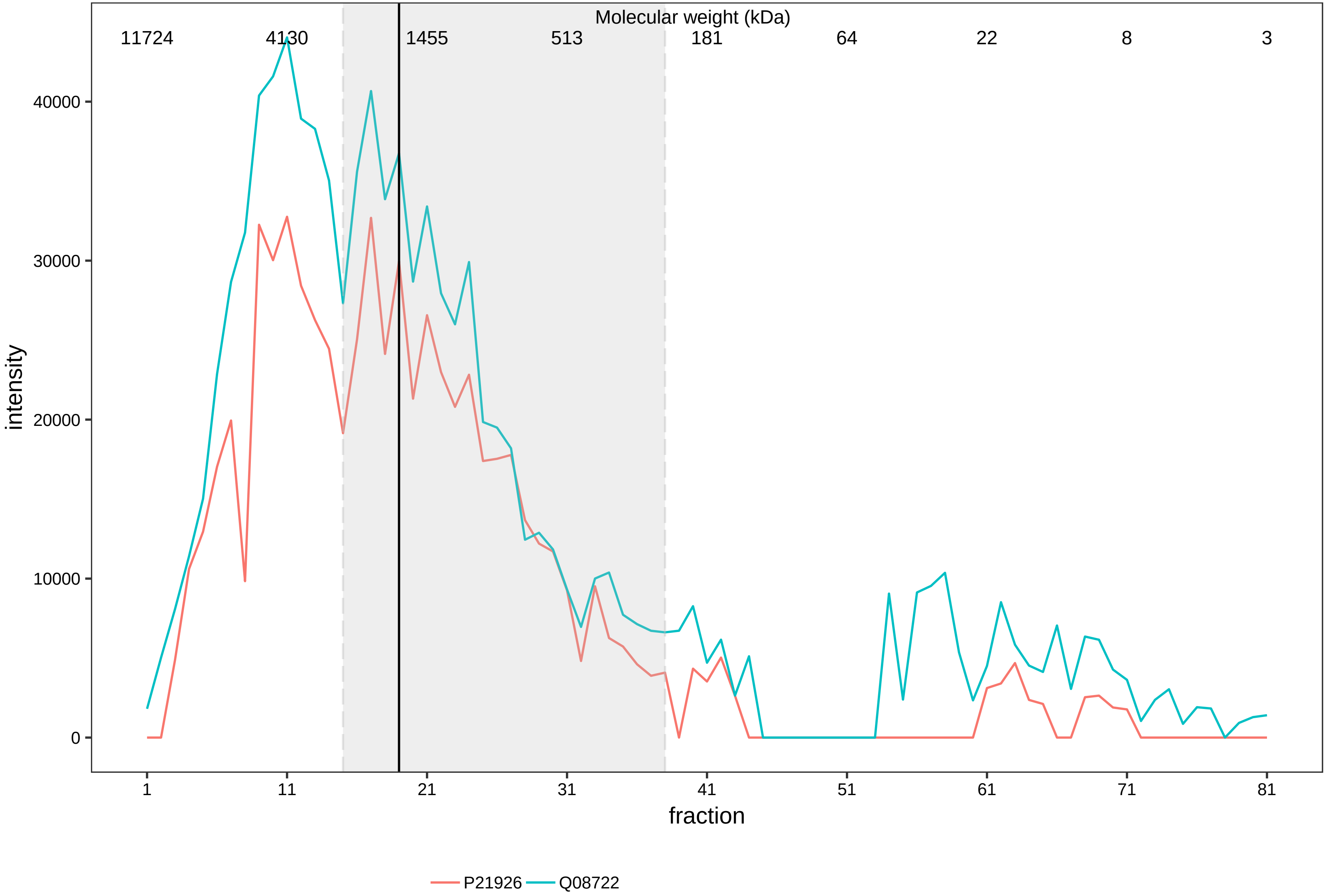

Feature ID 205

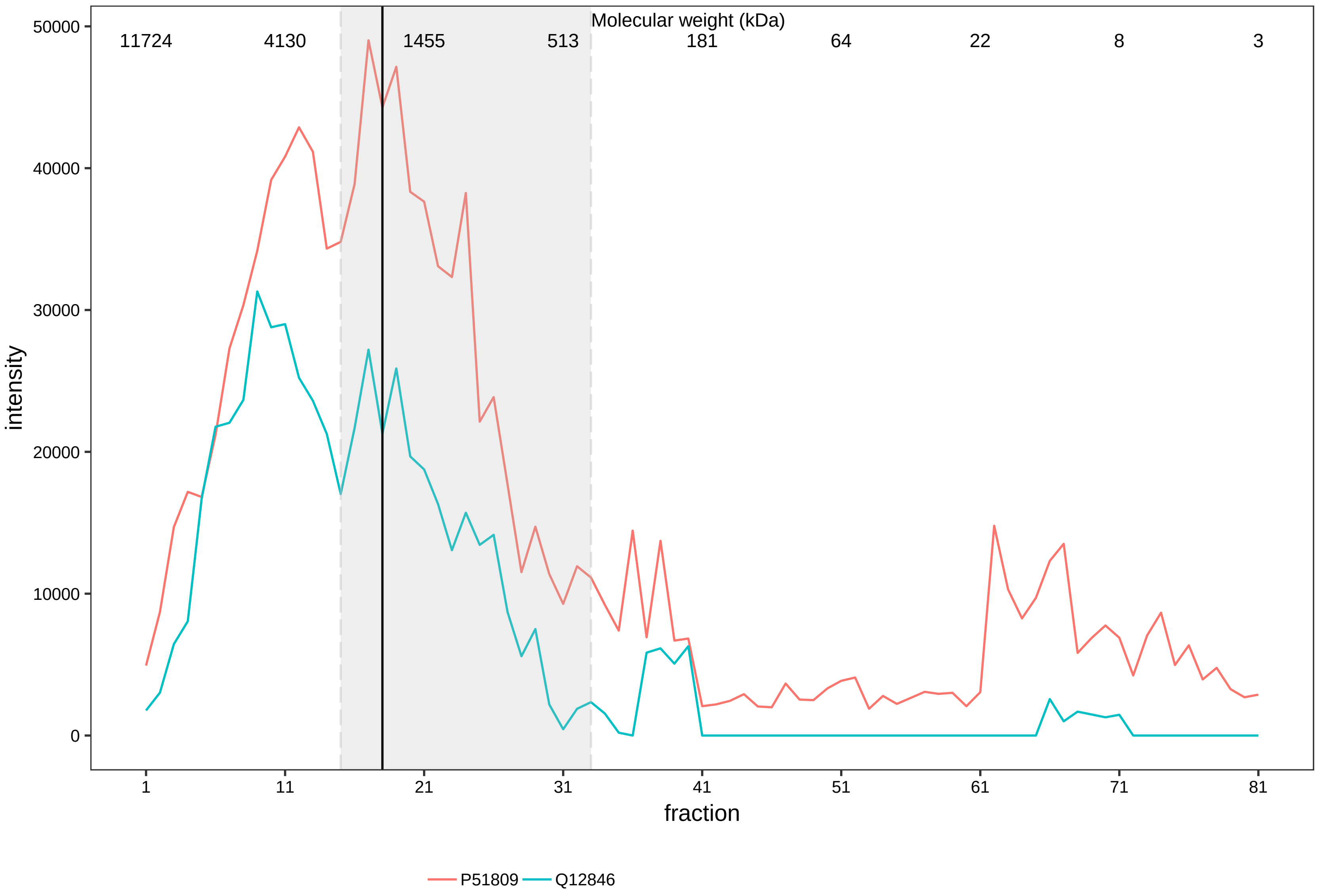

Feature ID 206

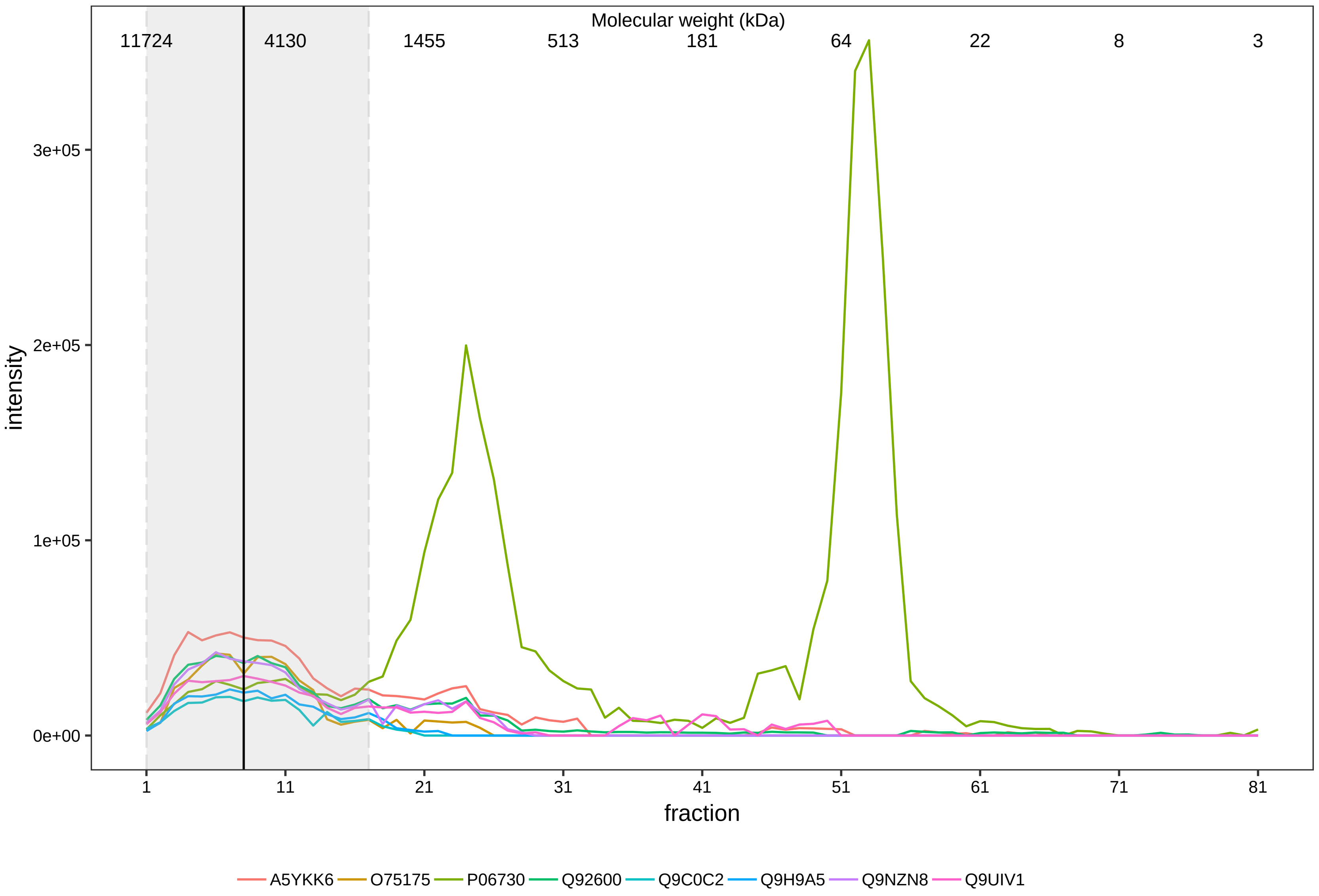

Feature ID 207

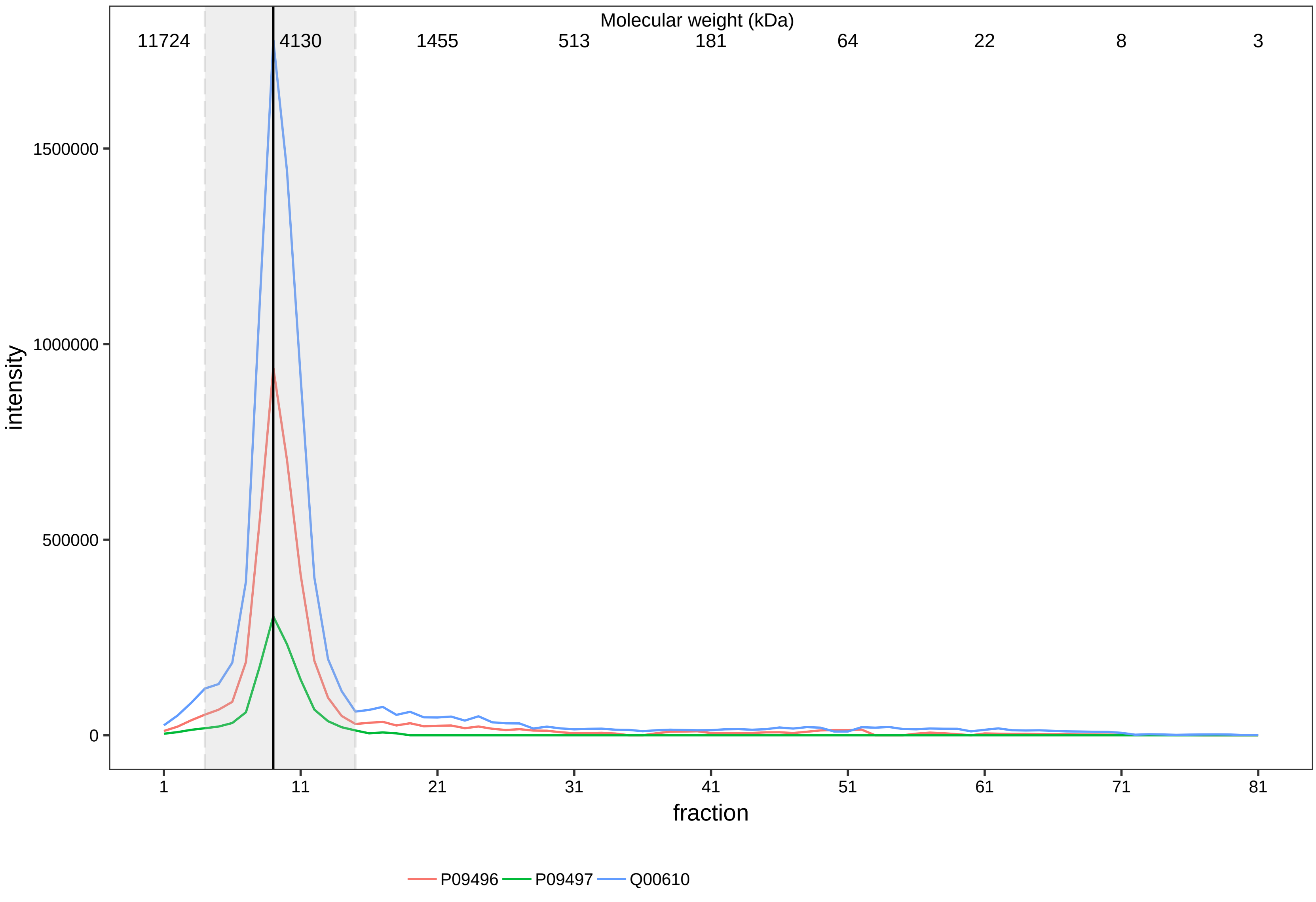

Feature ID 208

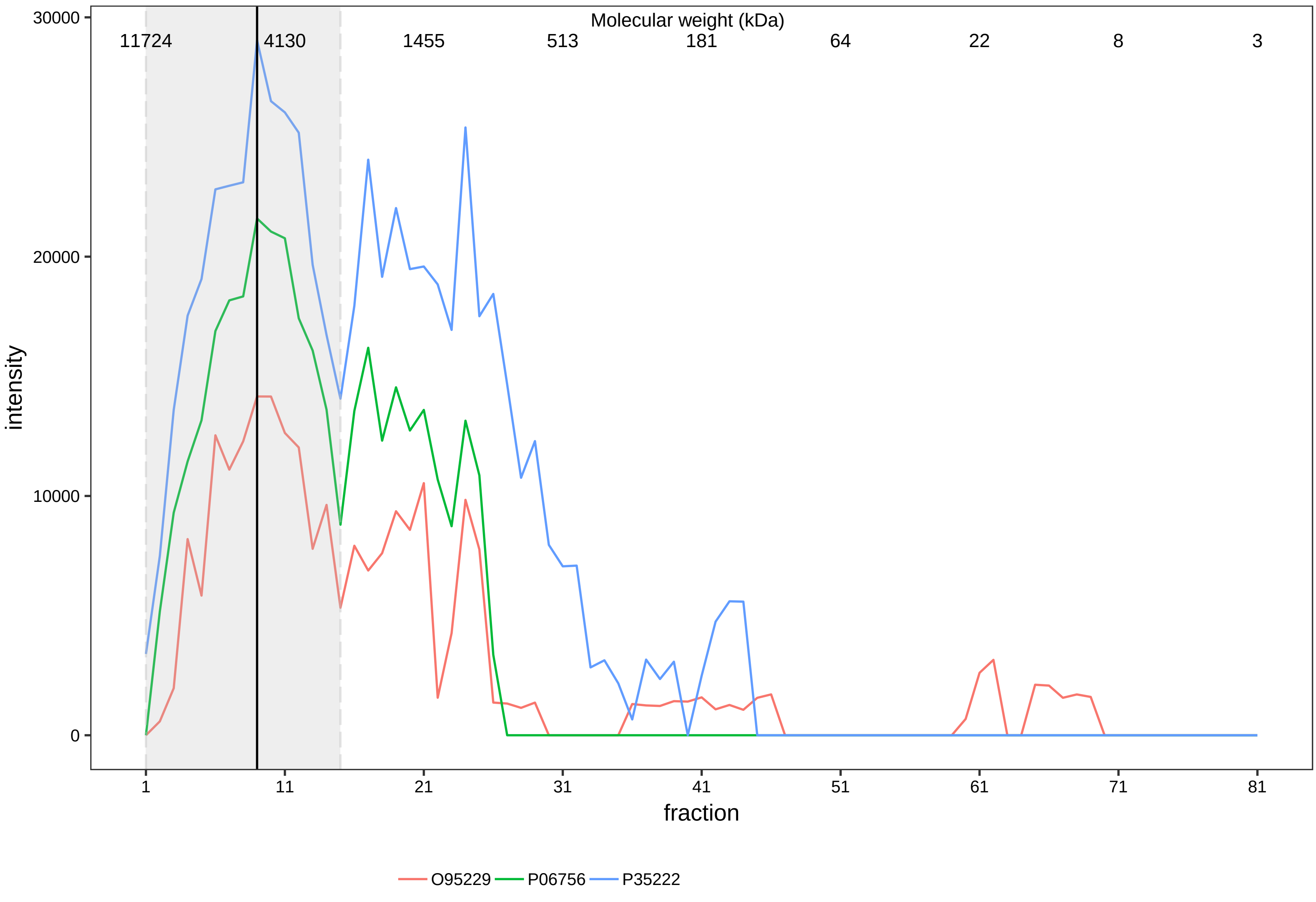

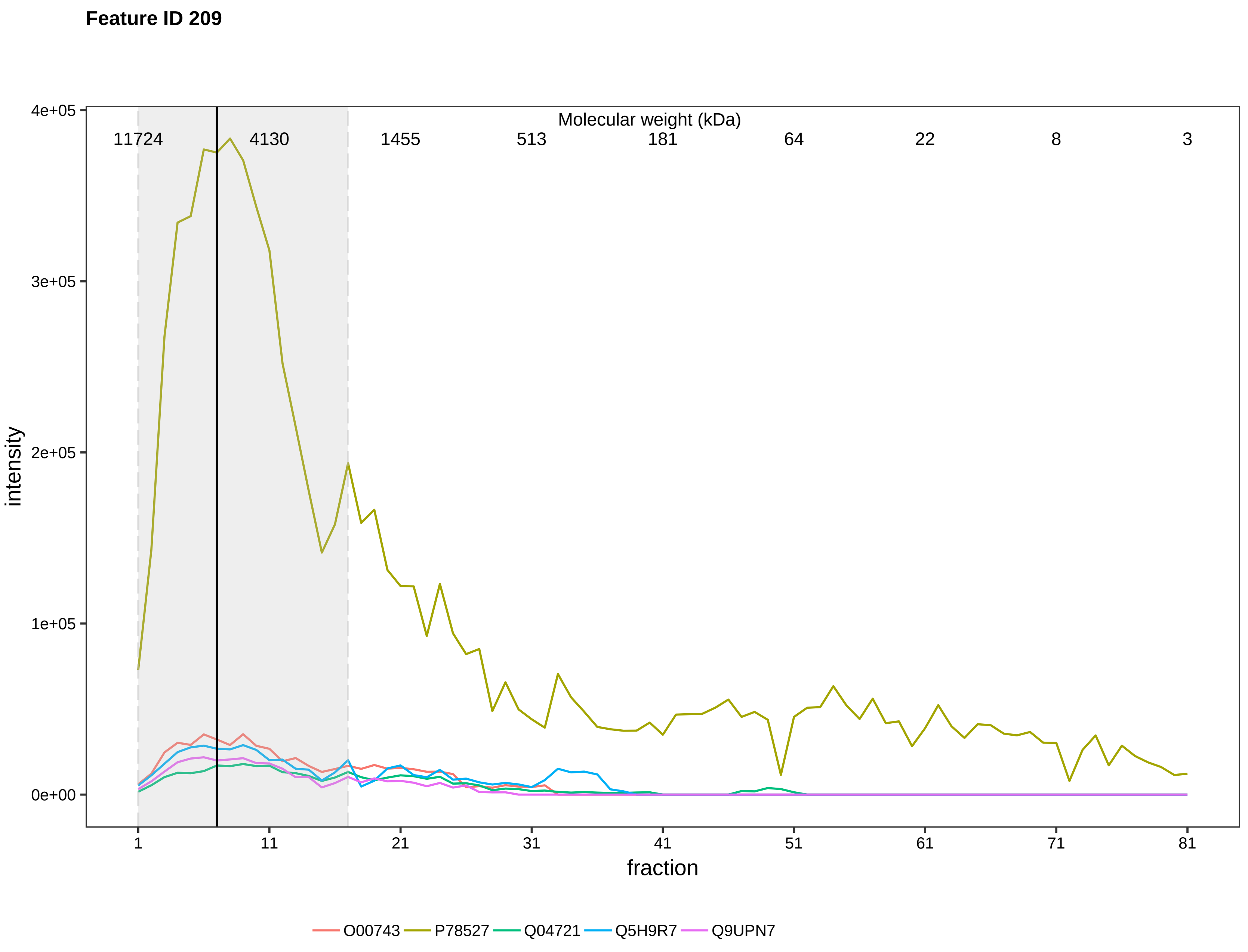

Feature ID 209
Molecular weight (kDa)
11724
4130
1455
513
181
64
22
8
3
4e+05
3e+05
2e+05
1e+05
0e+00
intensity
1
11
21
31
41
51
61
71
81
fraction
O00743
P78527
Q04721
Q5H9R7
Q9UPN7

Feature ID 210

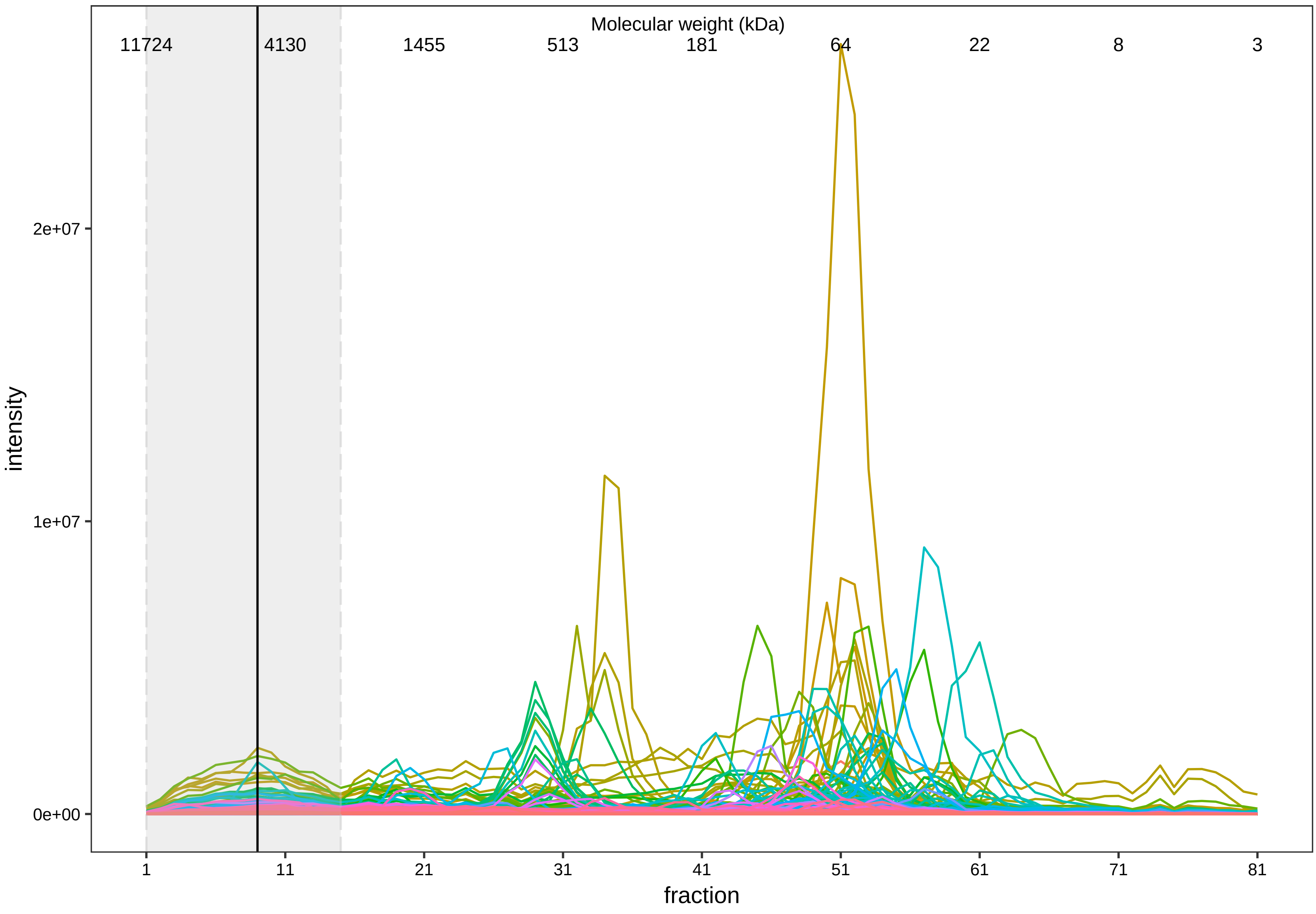

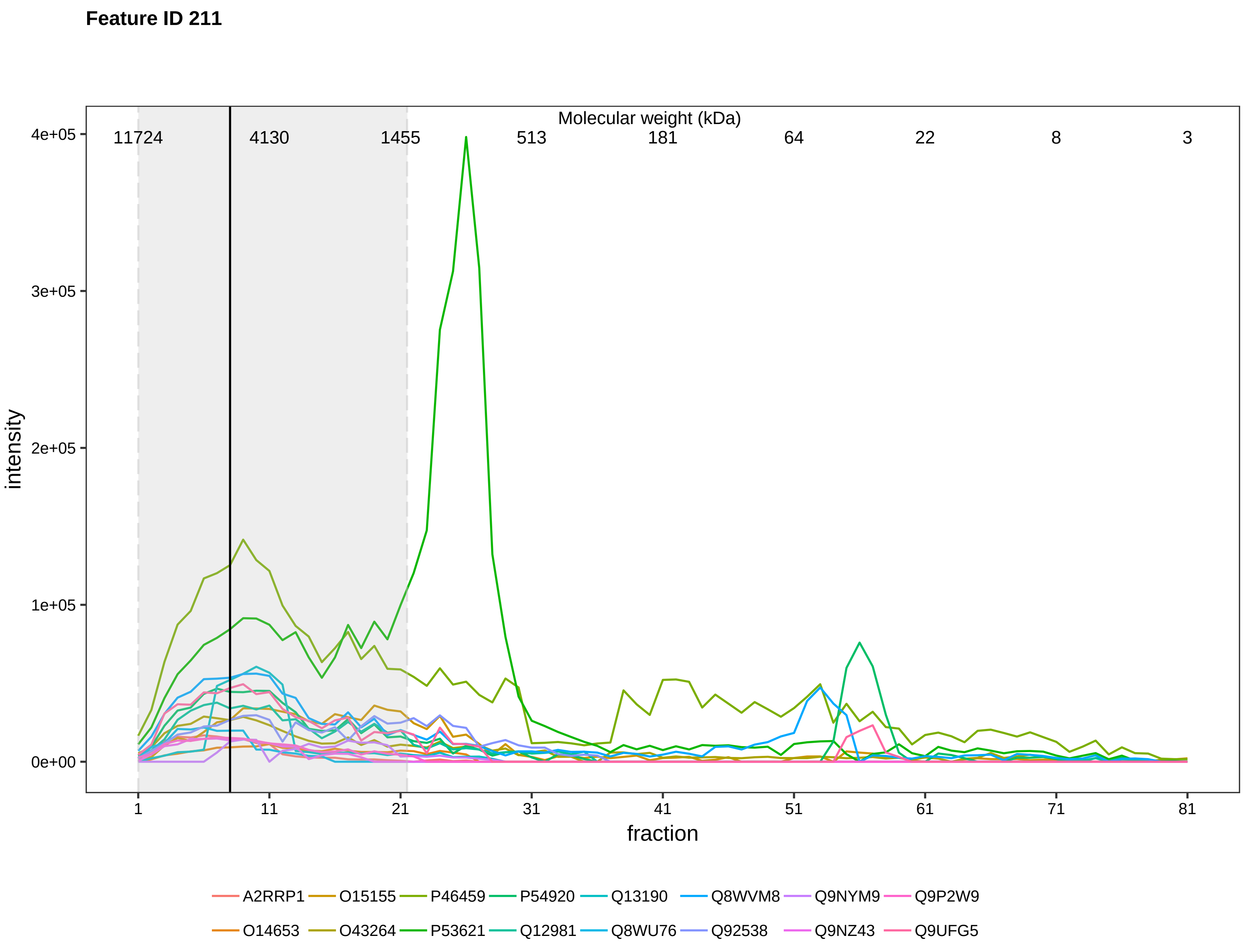

Feature ID 211
Molecular weight (kDa)
11724
4130
1455
513
181
64
22
8
3
intensity
4e+05
3e+05
2e+05
1e+05
0e+00
fraction
1
11
21
31
41
51
61
71
81
A2RRP1
O15155
P46459
P54920
Q13190
Q8WVM8
Q9NYM9
Q9P2W9
O14653
O43264
P53621
Q12981
Q8WU76
Q92538
Q9NZ43
Q9UFG5

Feature ID 212

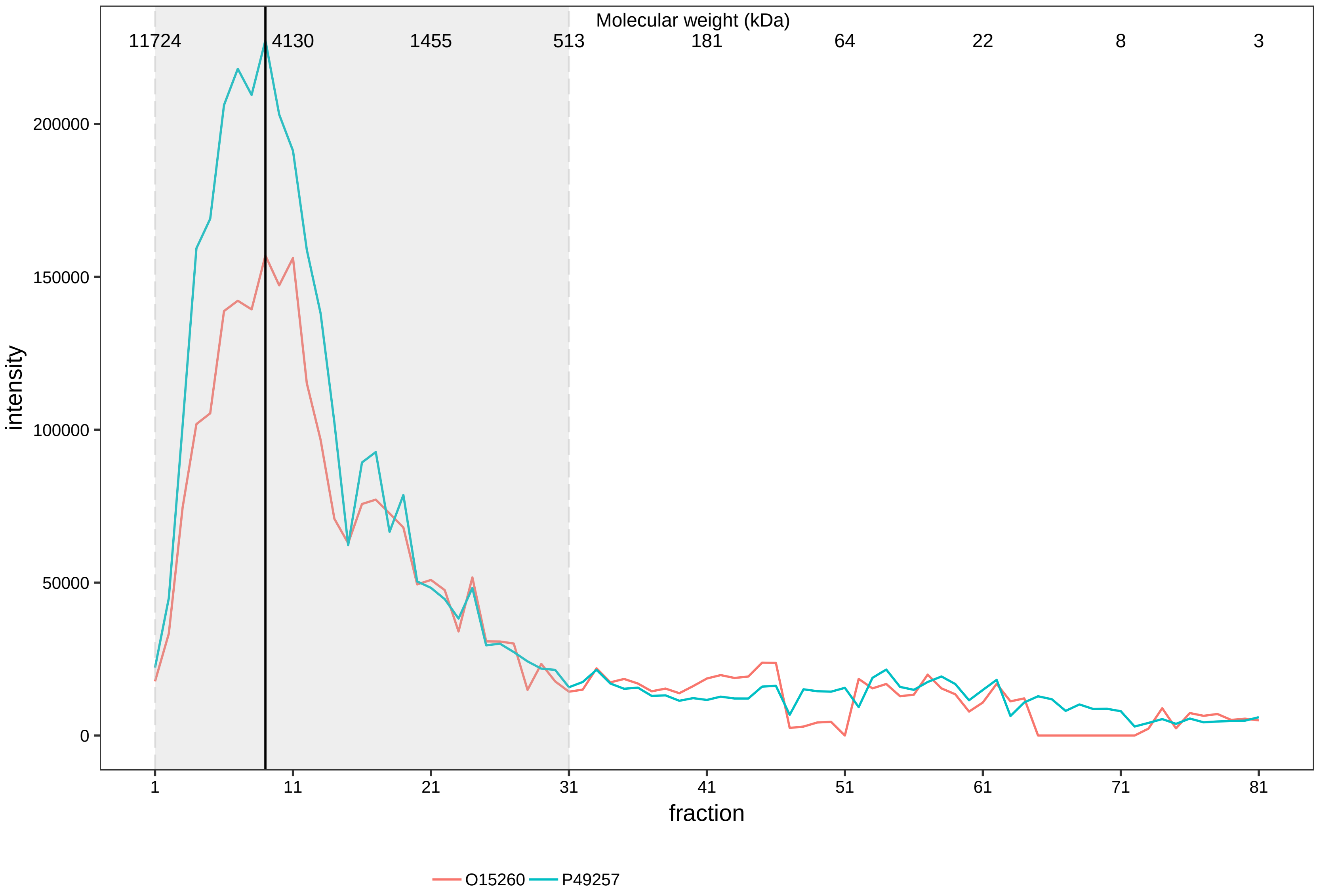

Feature ID 213

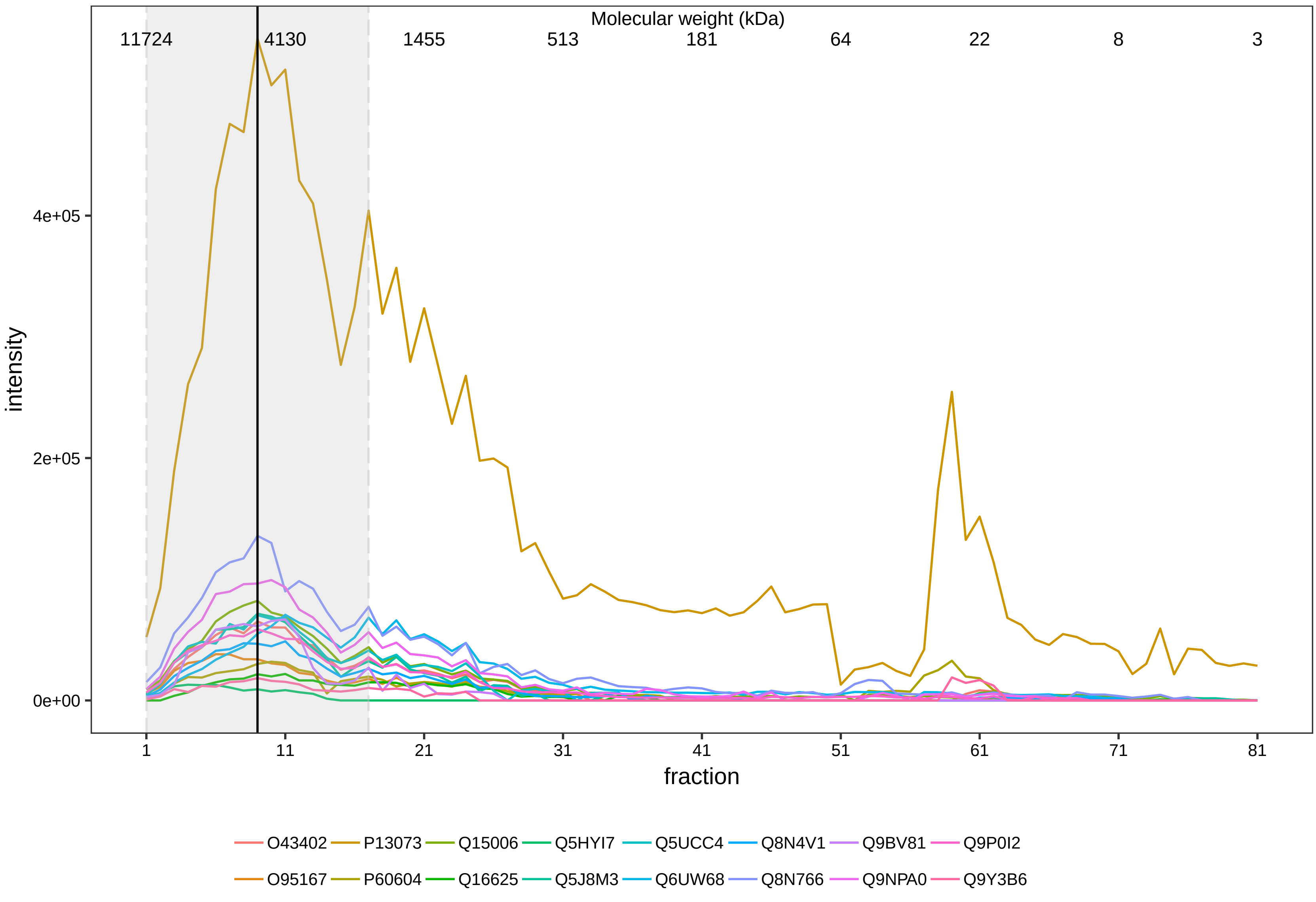

Feature ID 214

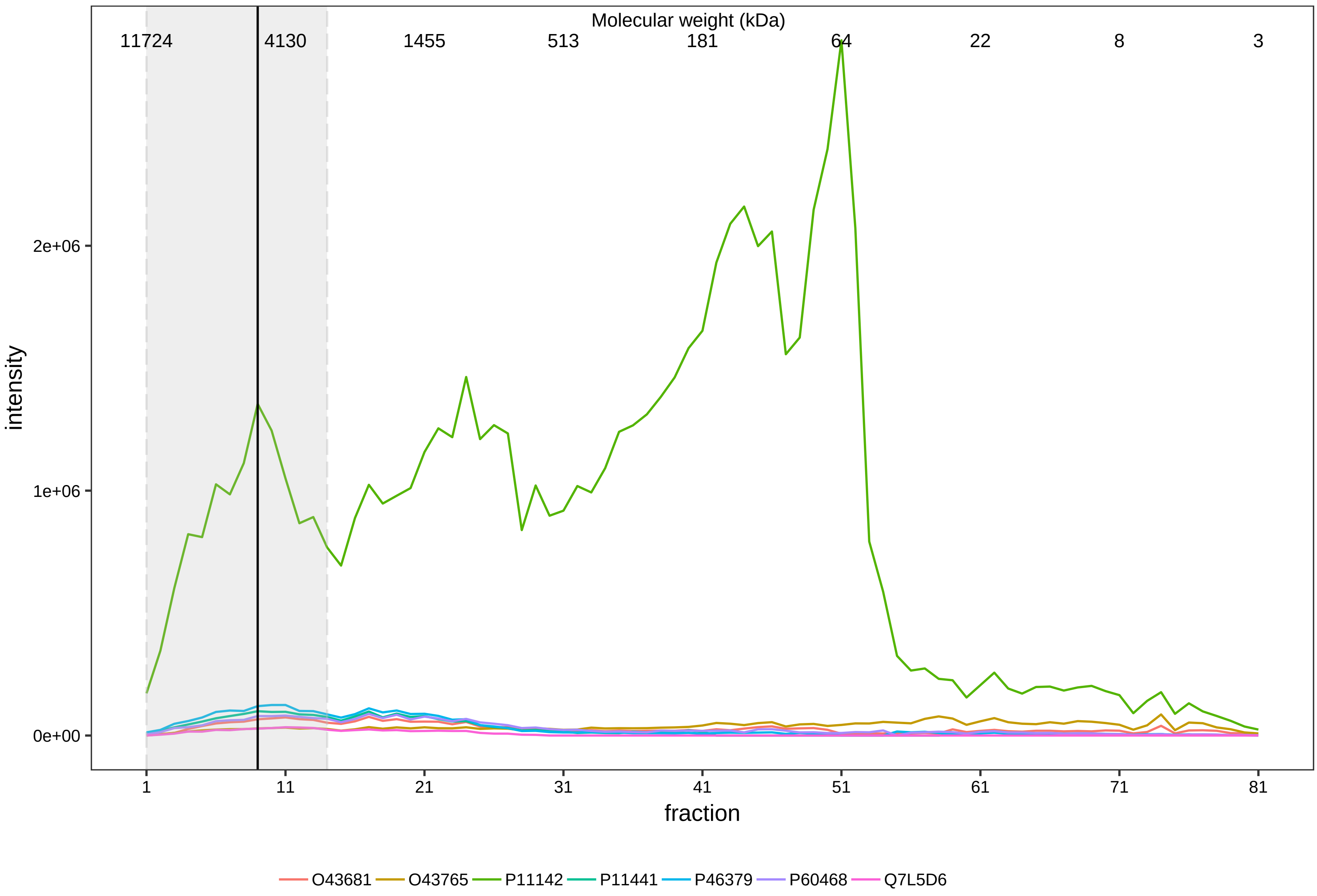

Feature ID 215

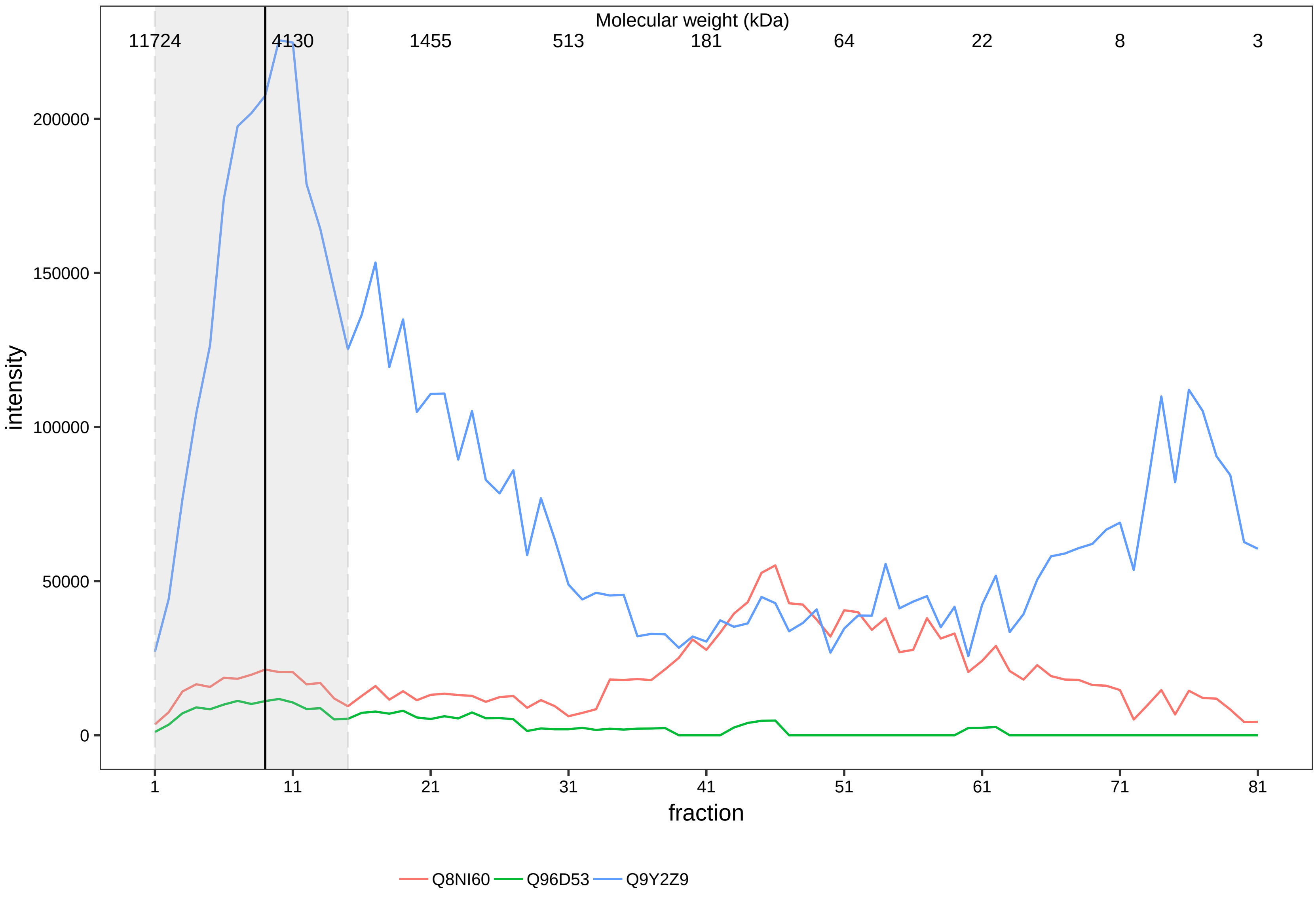

Feature ID 216

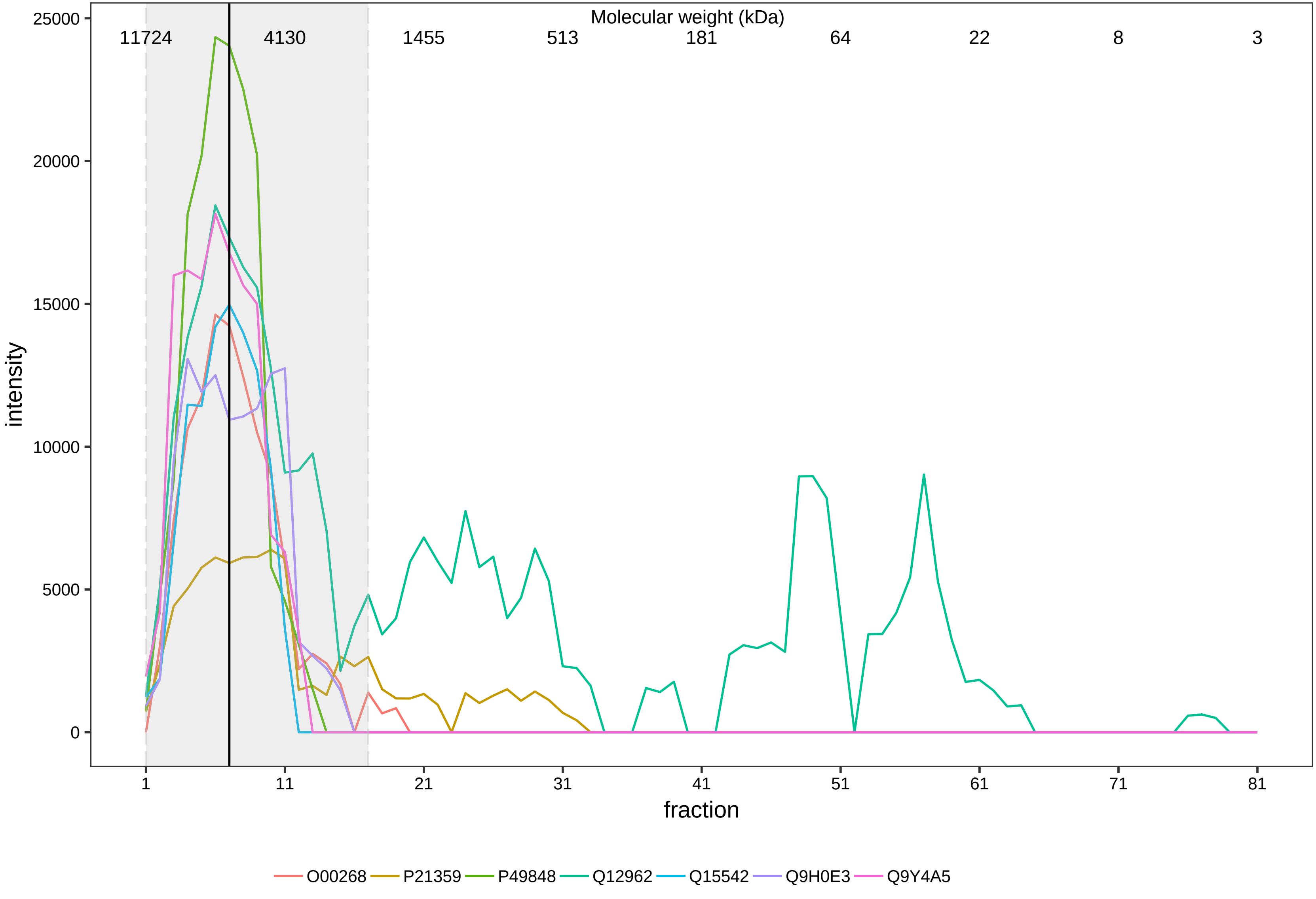

Feature ID 217

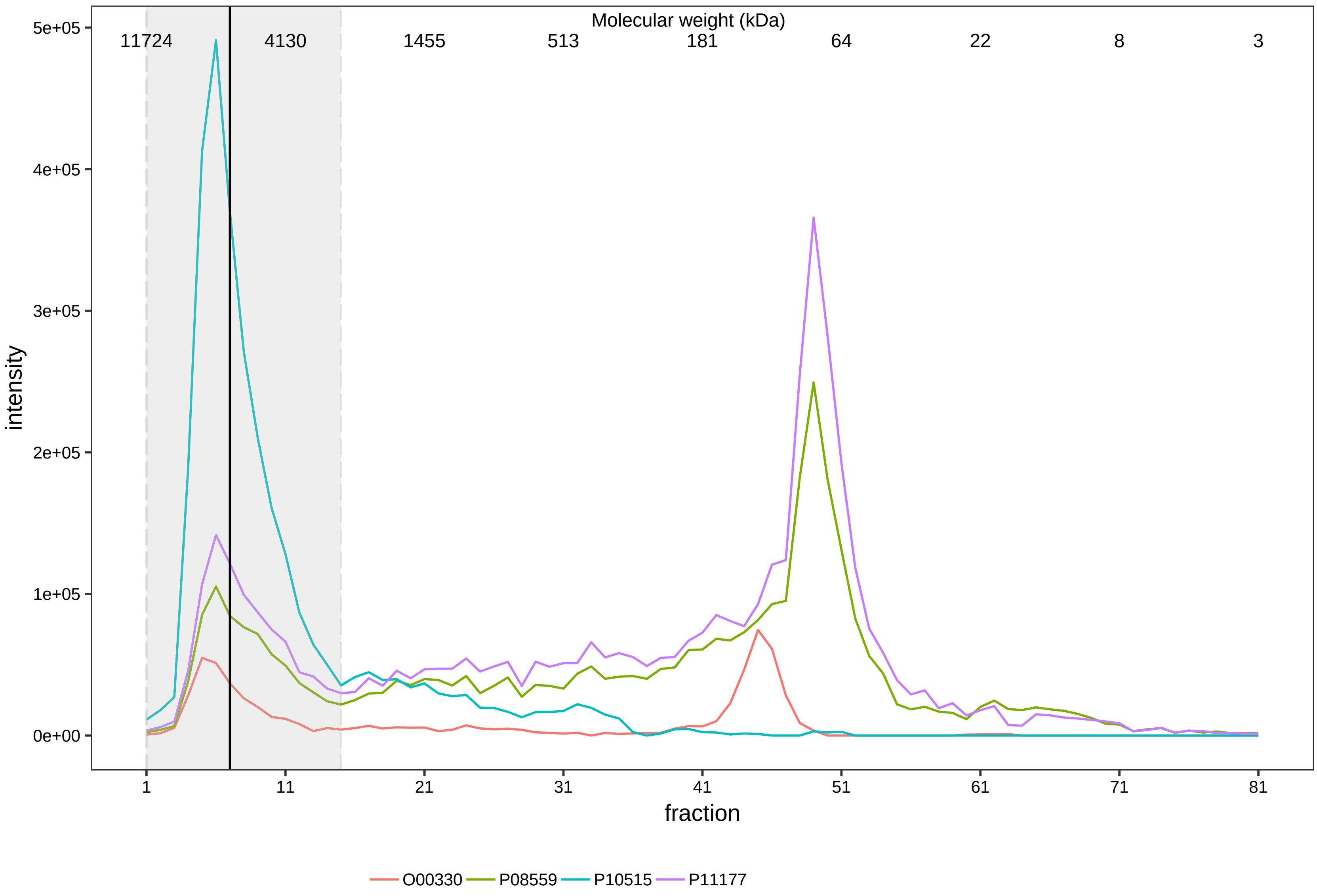

Feature ID 218

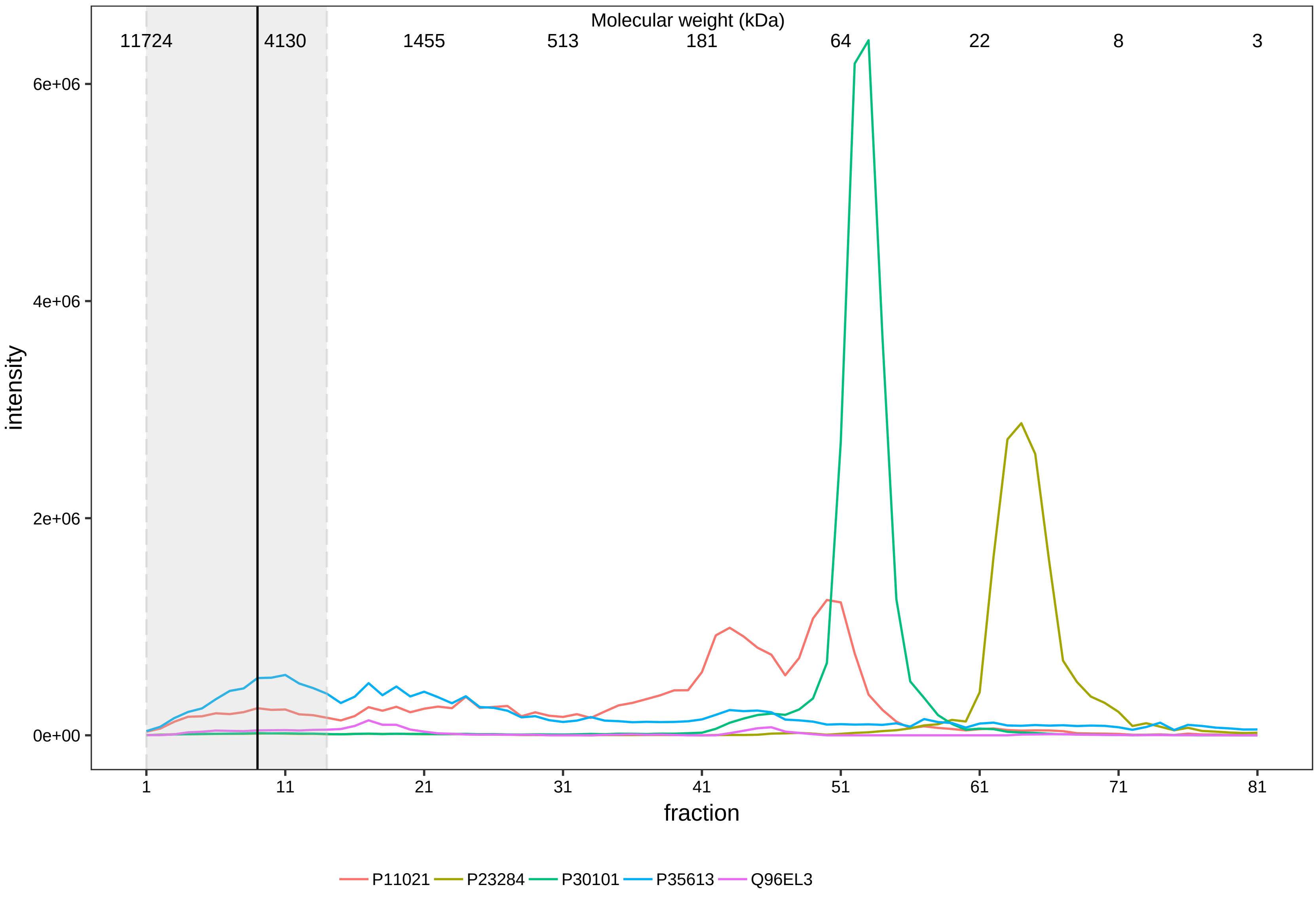

Feature ID 219

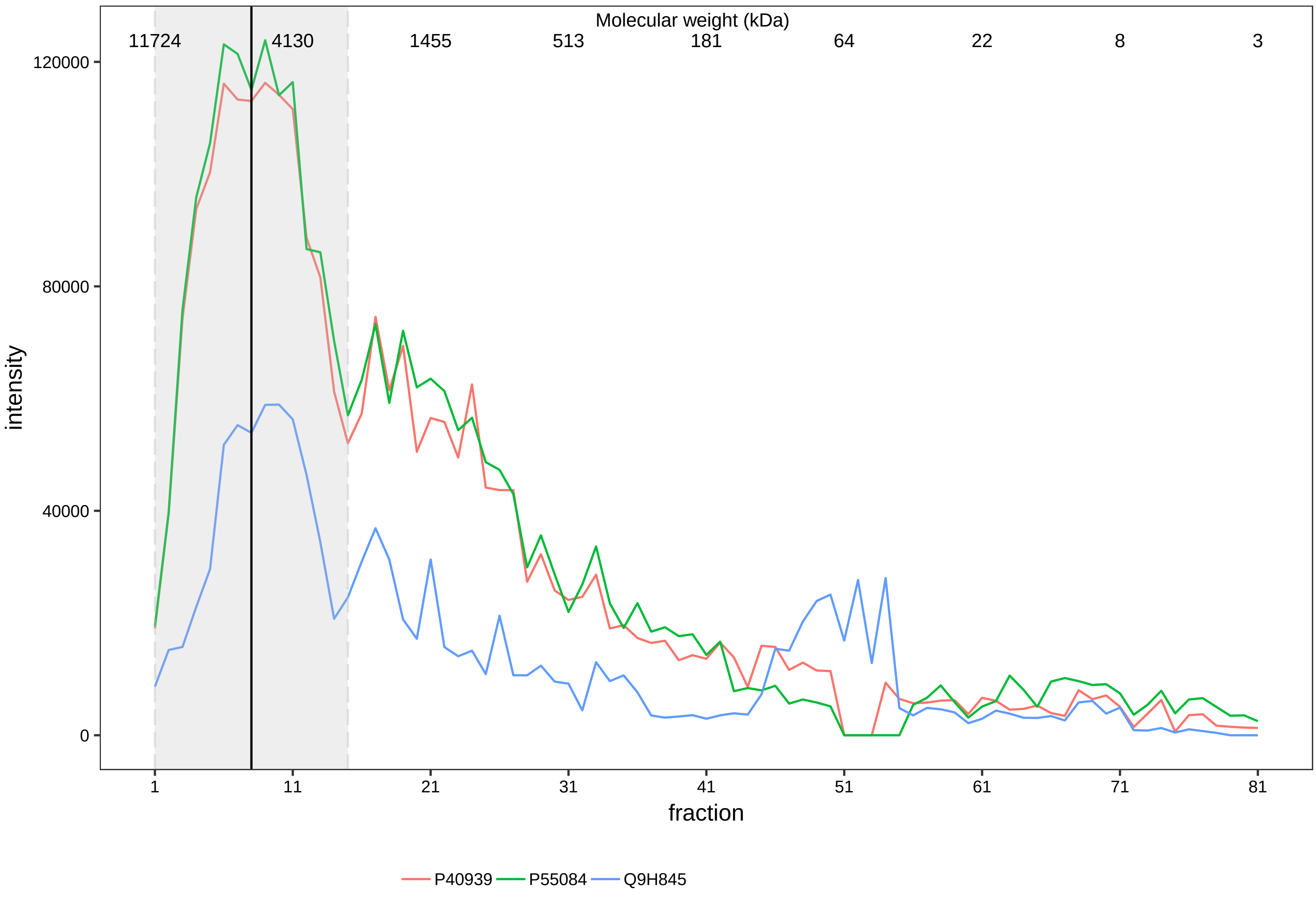

Feature ID 220

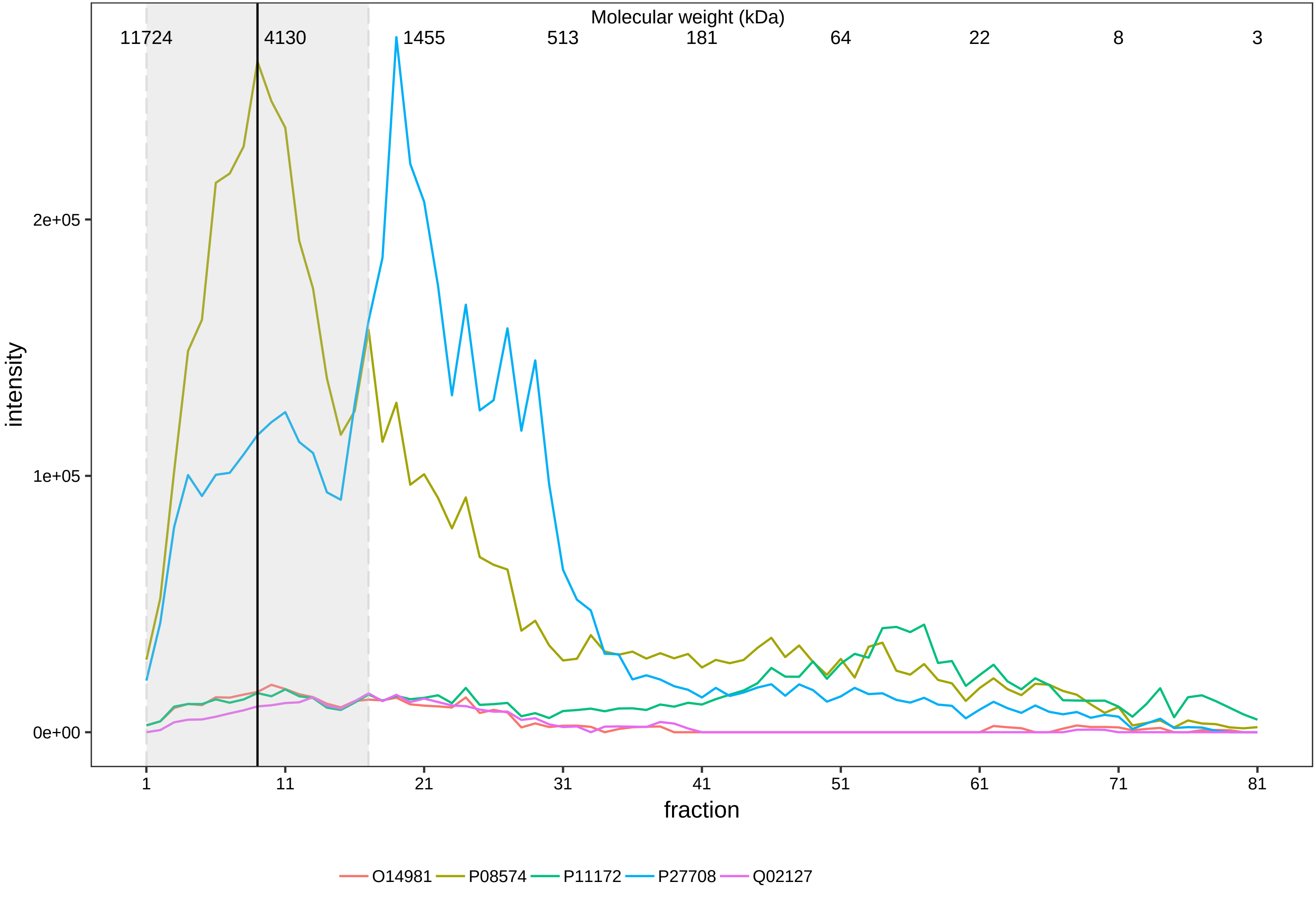

Feature ID 221

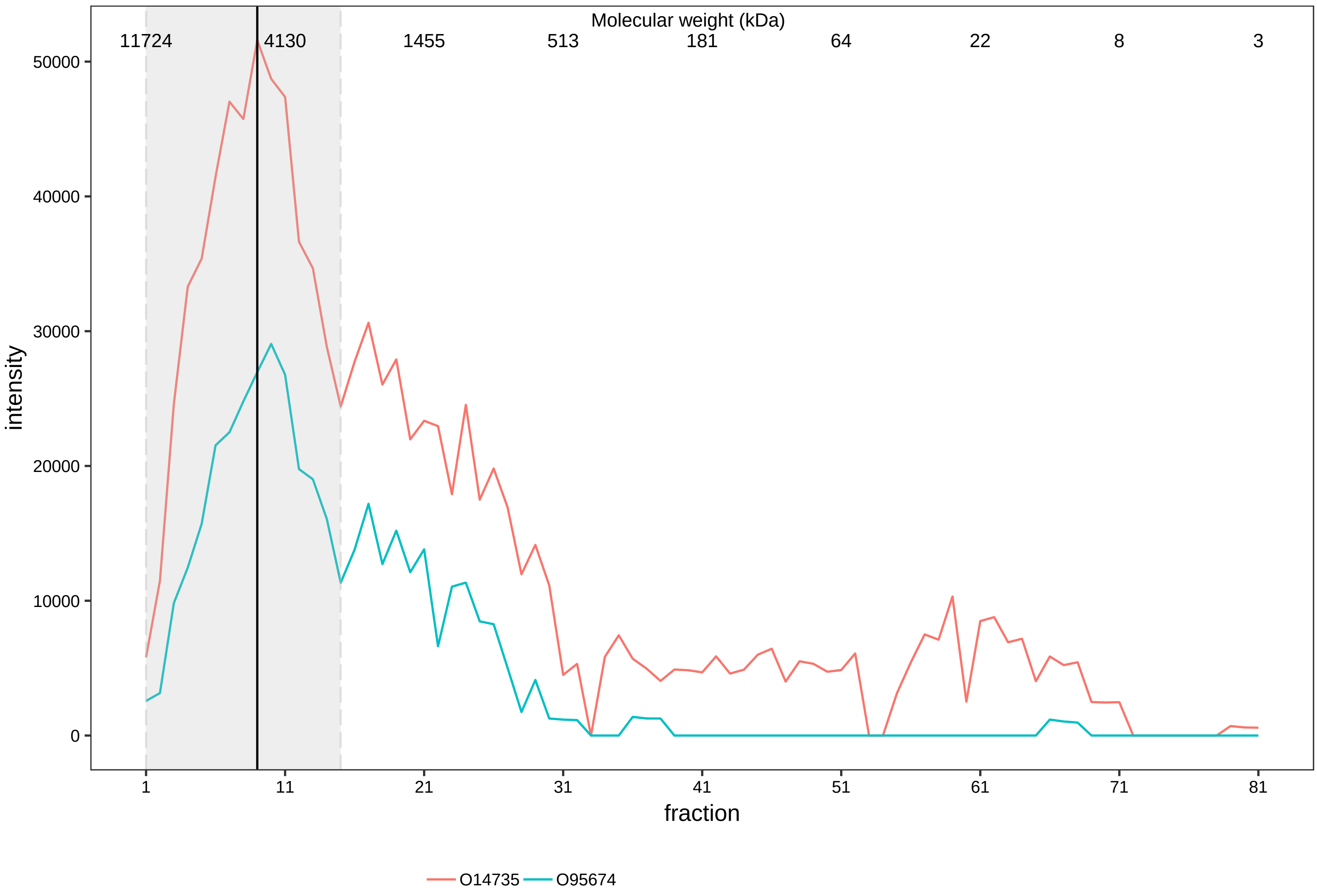

Feature ID 222

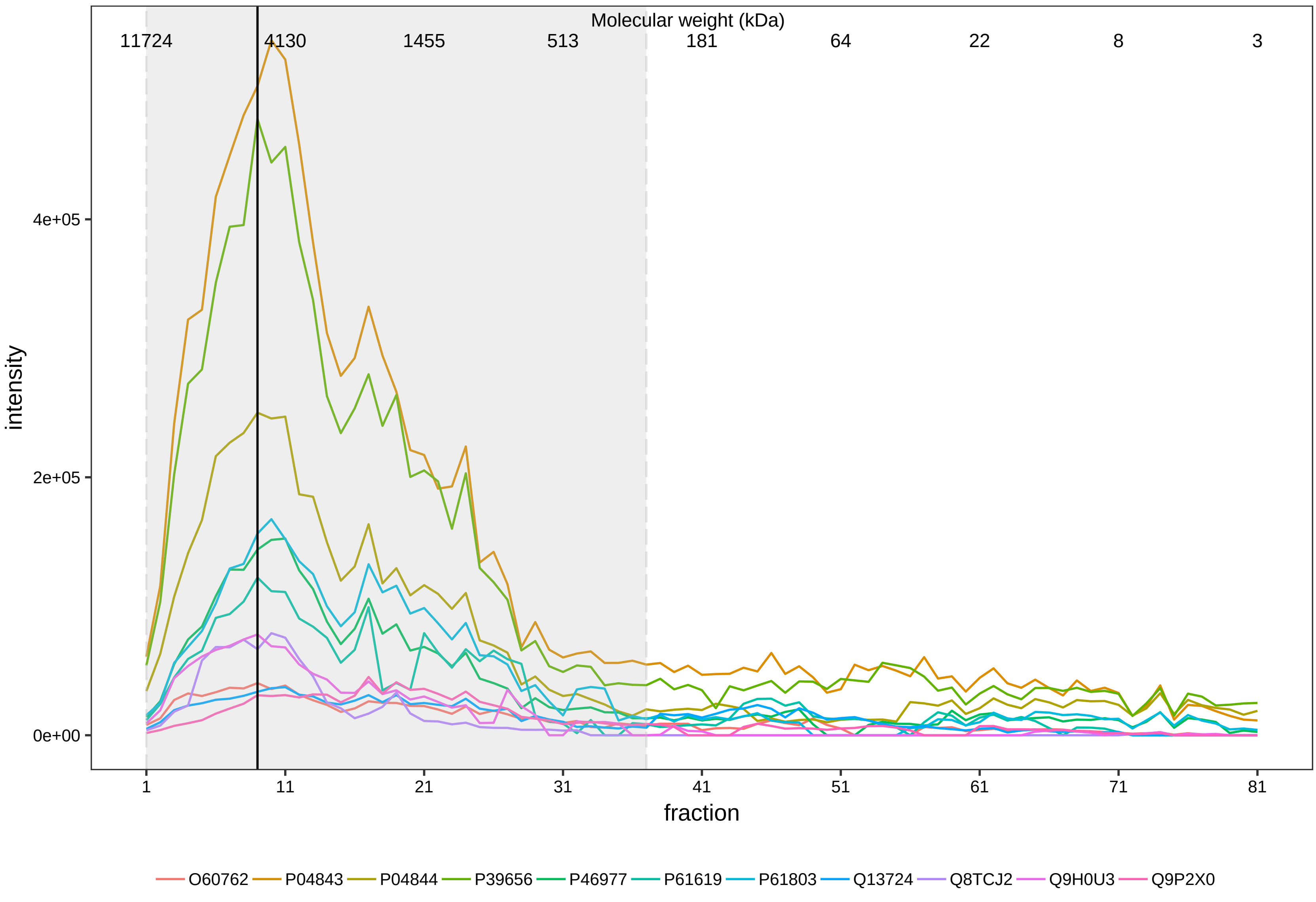

Feature ID 223

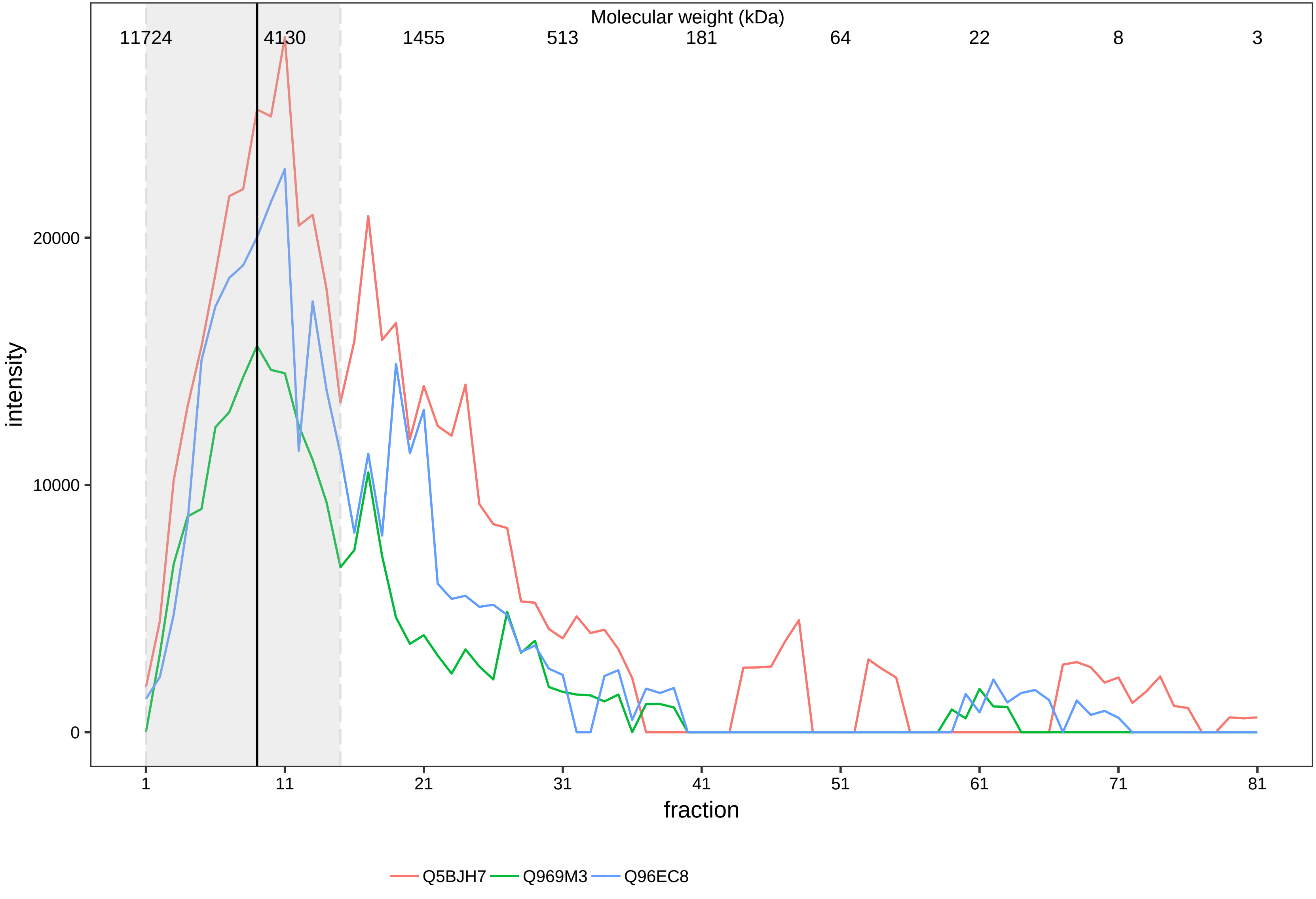

Feature ID 224

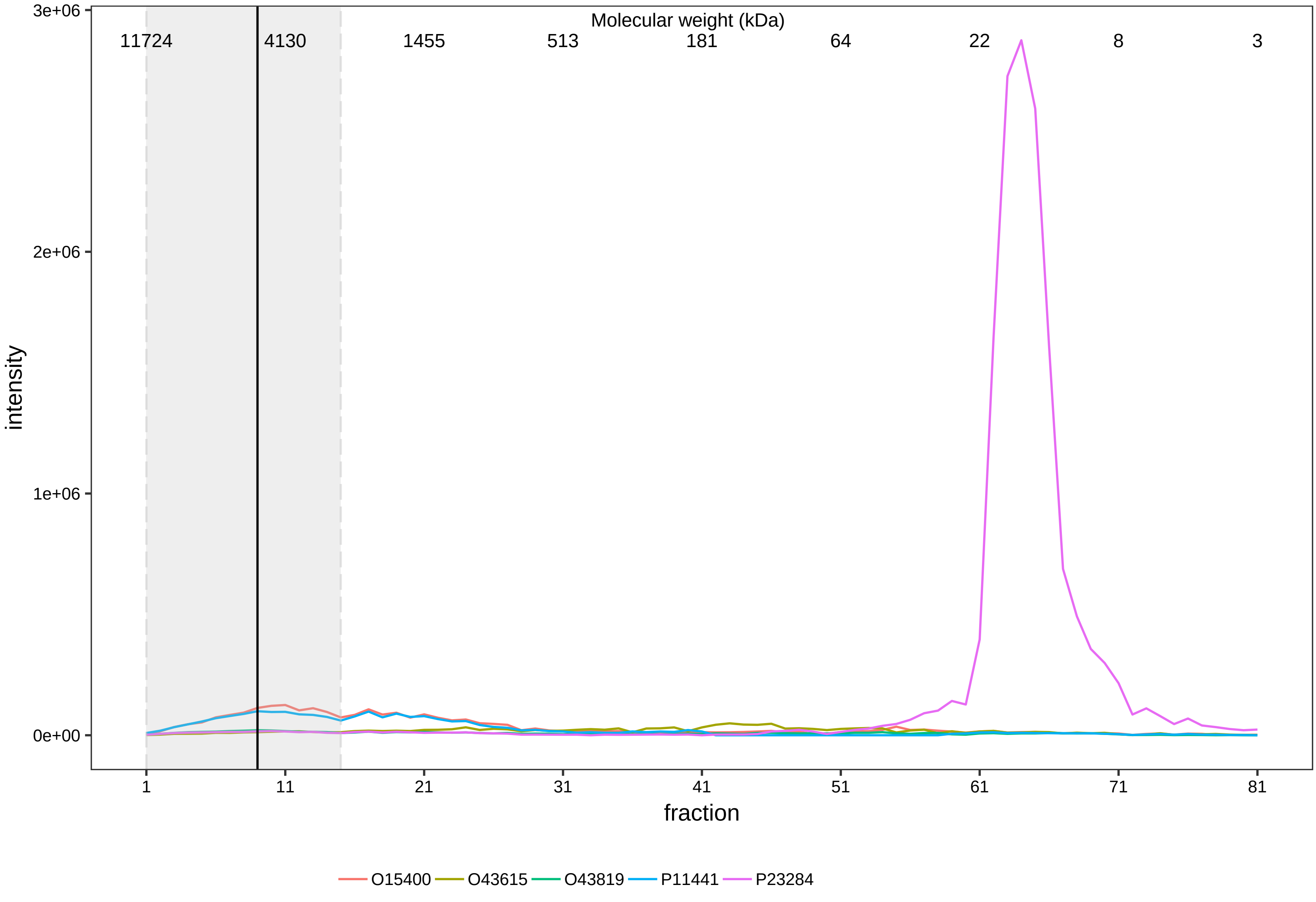

Feature ID 225

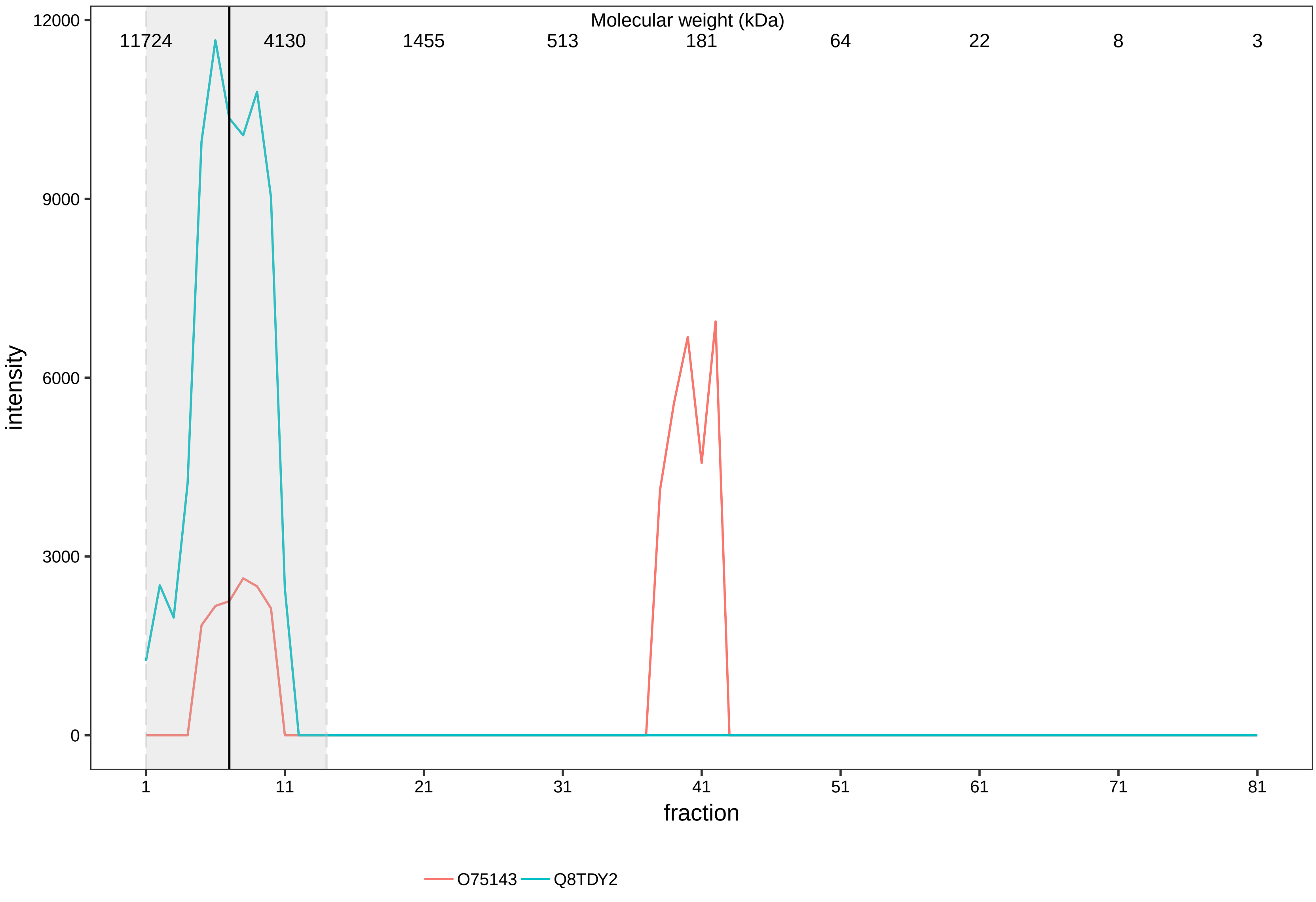

Feature ID 226

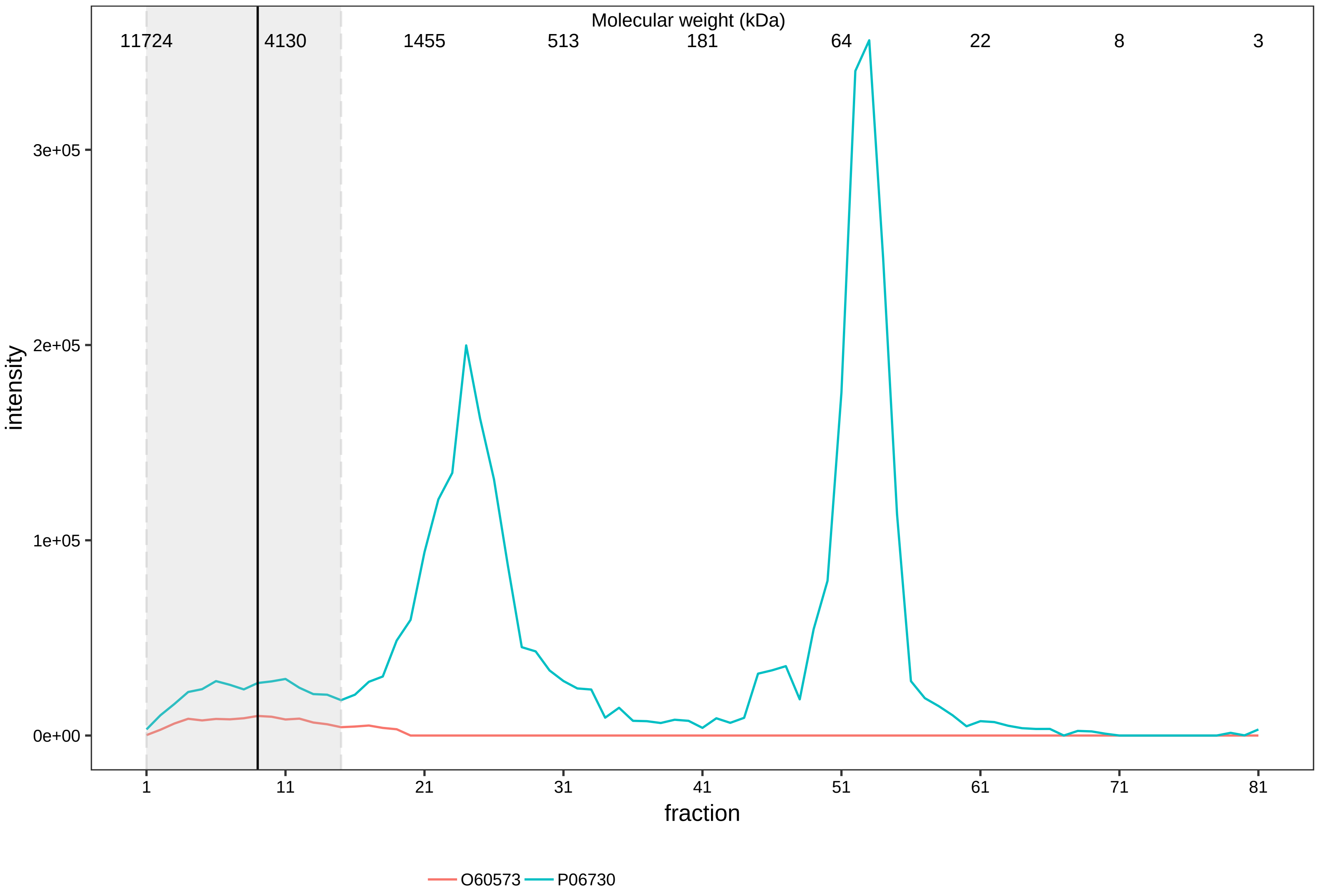

Feature ID 227

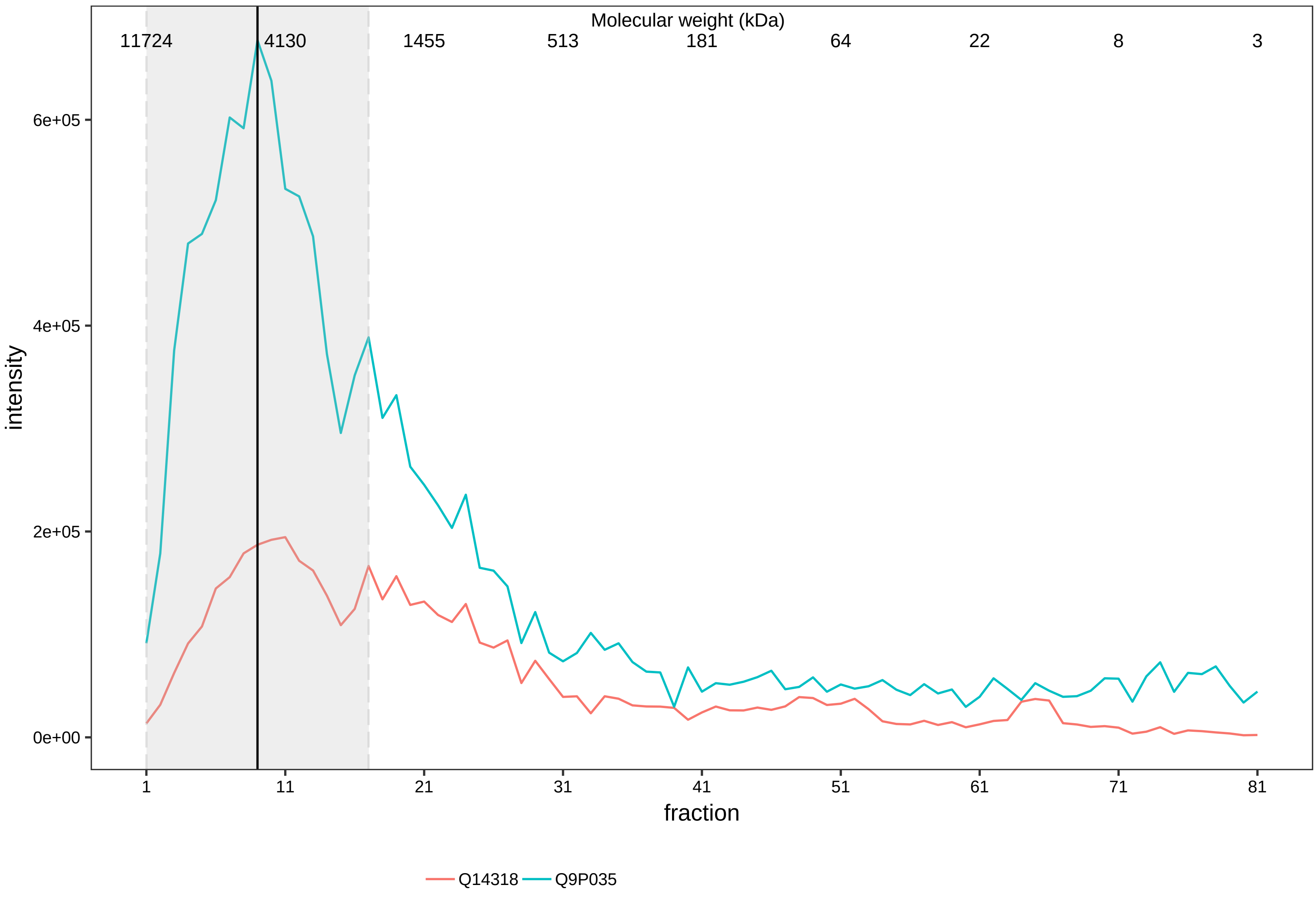

Feature ID 228

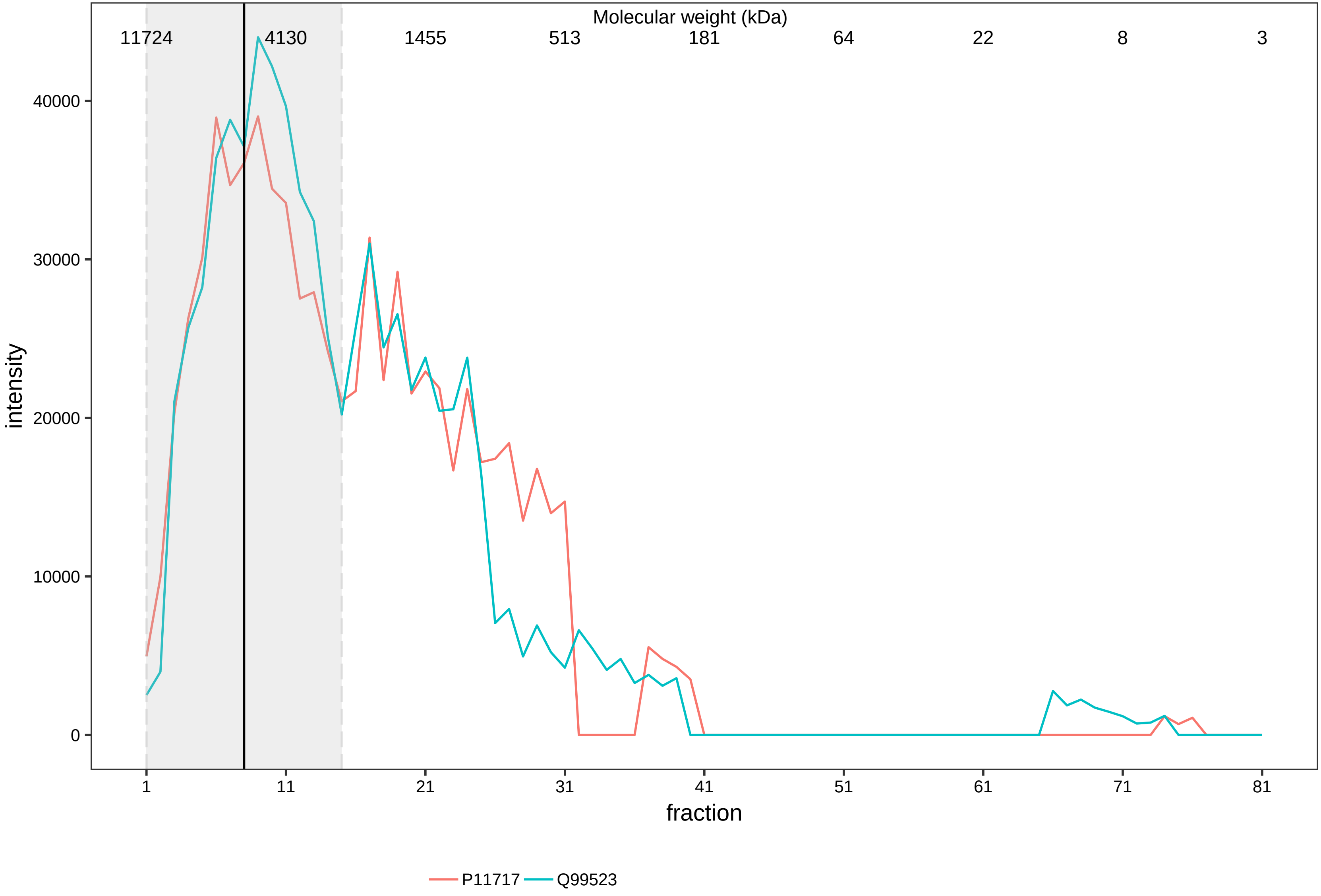

Feature ID 229

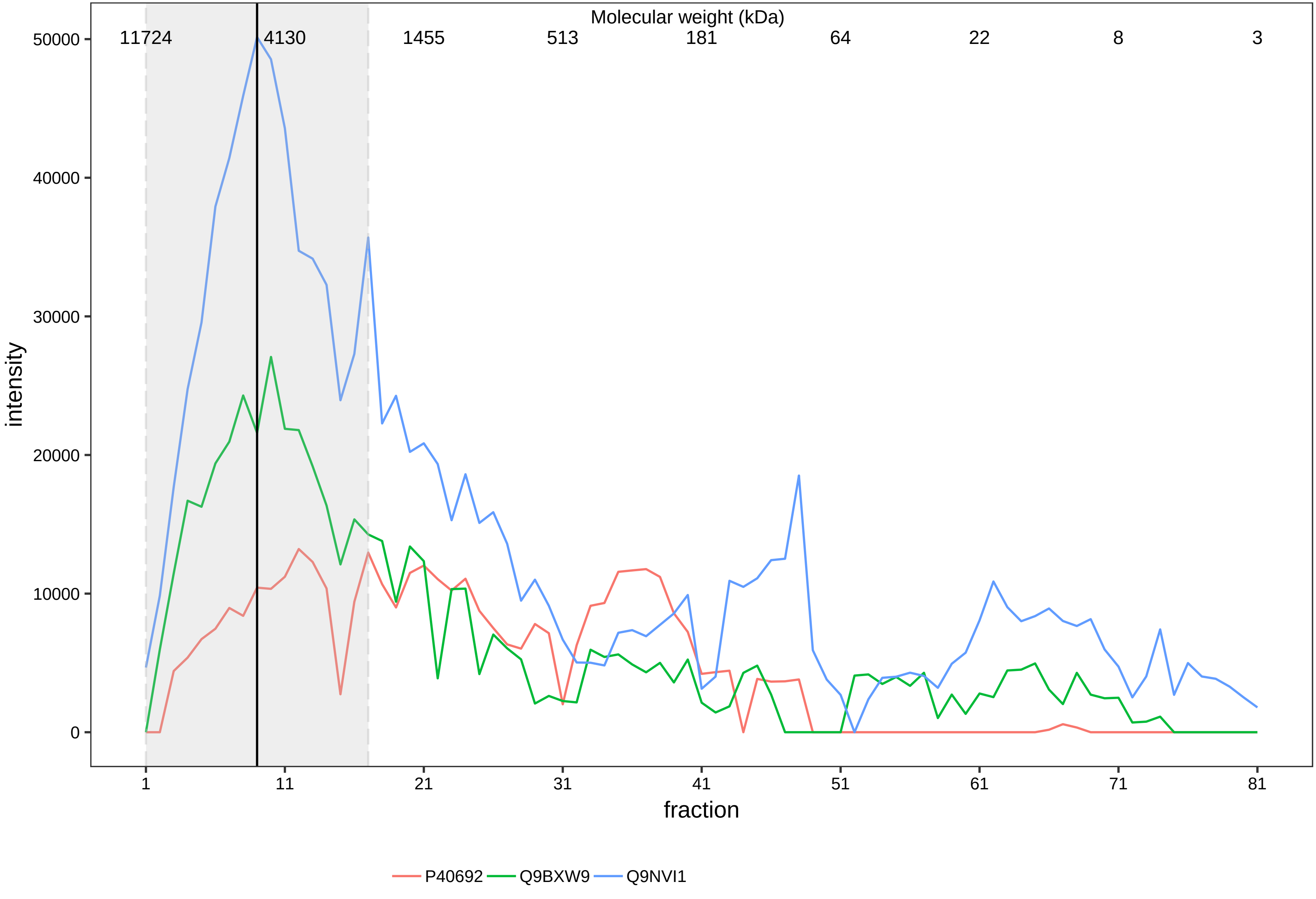

Feature ID 230

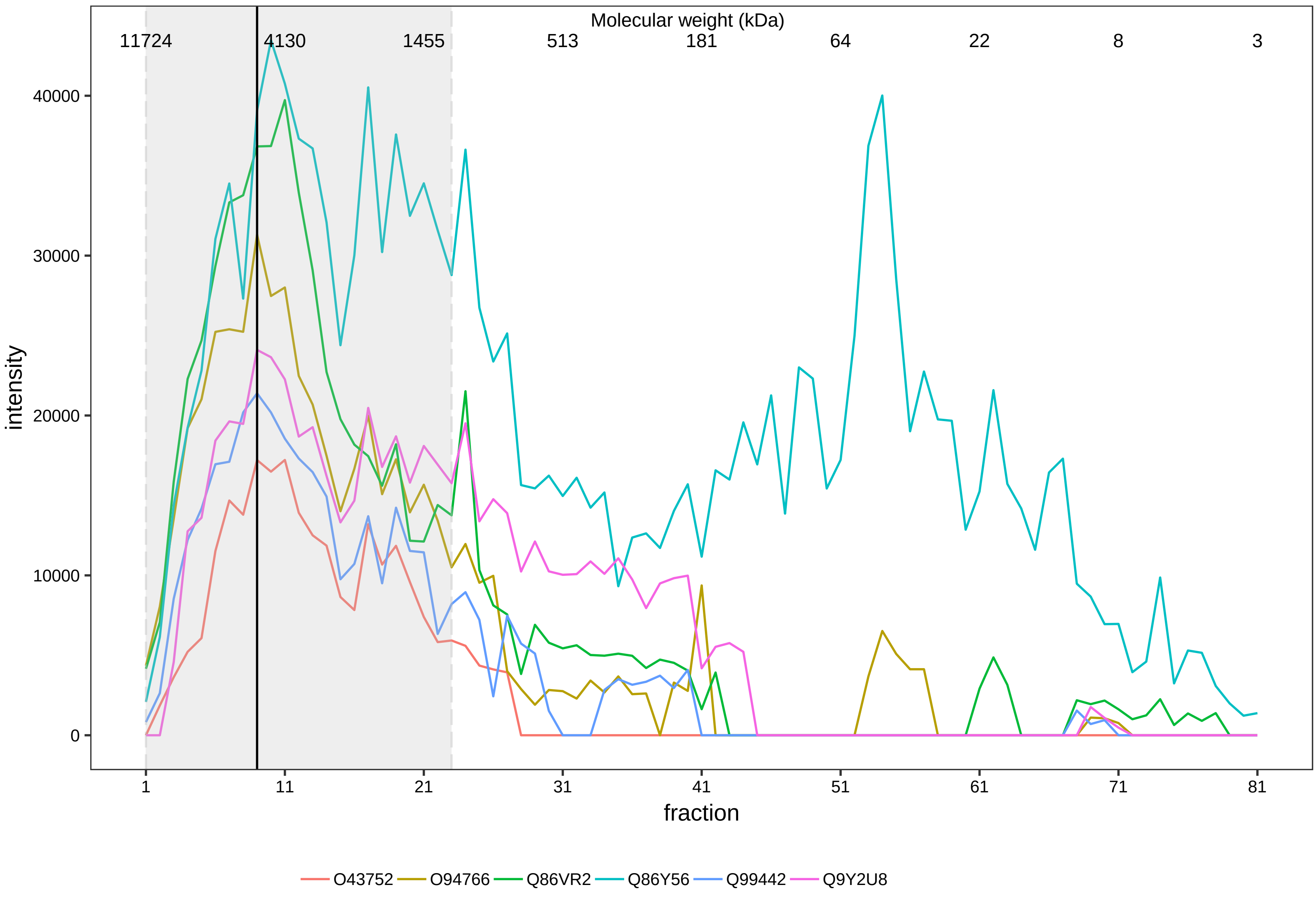

Feature ID 231

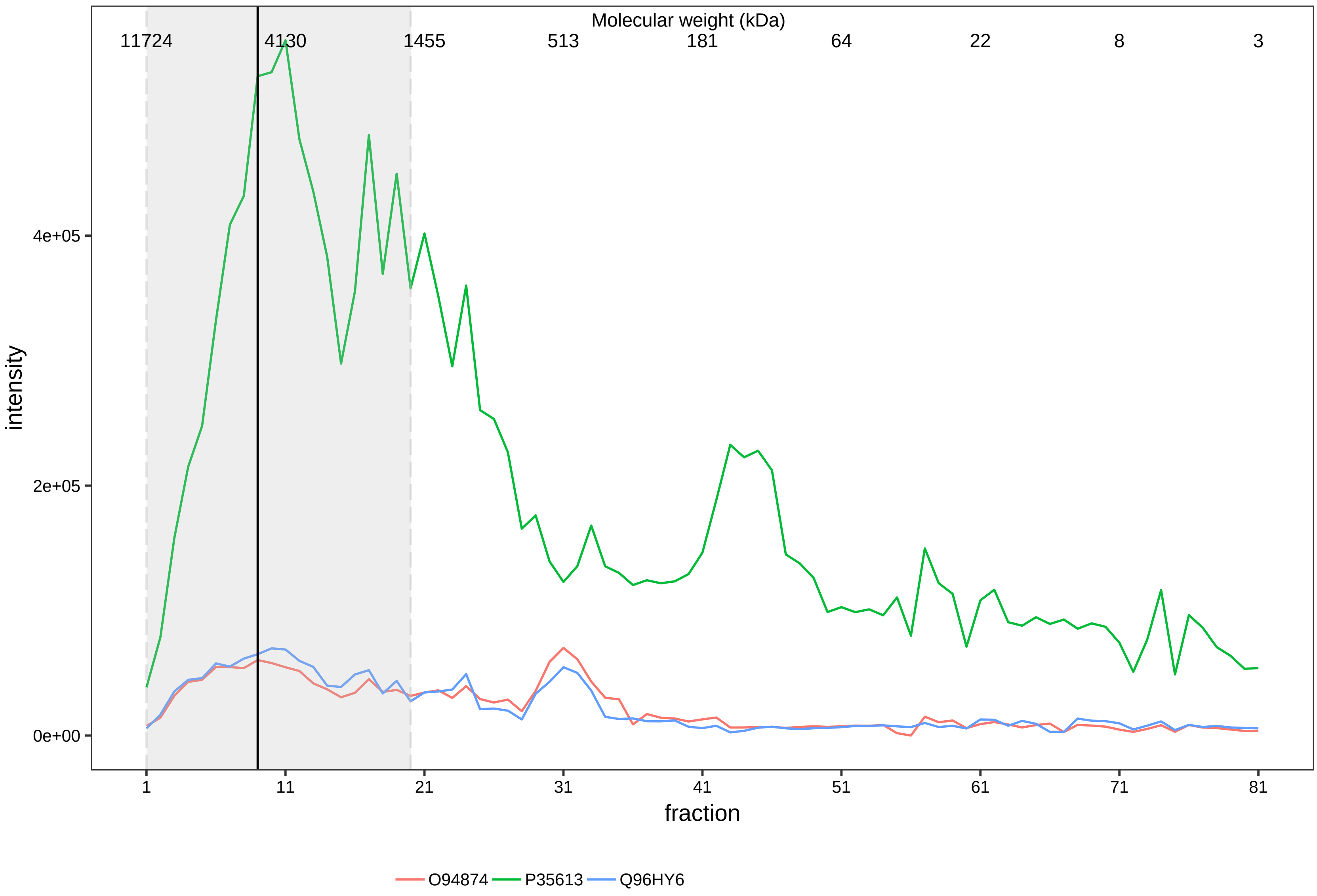

Feature ID 232

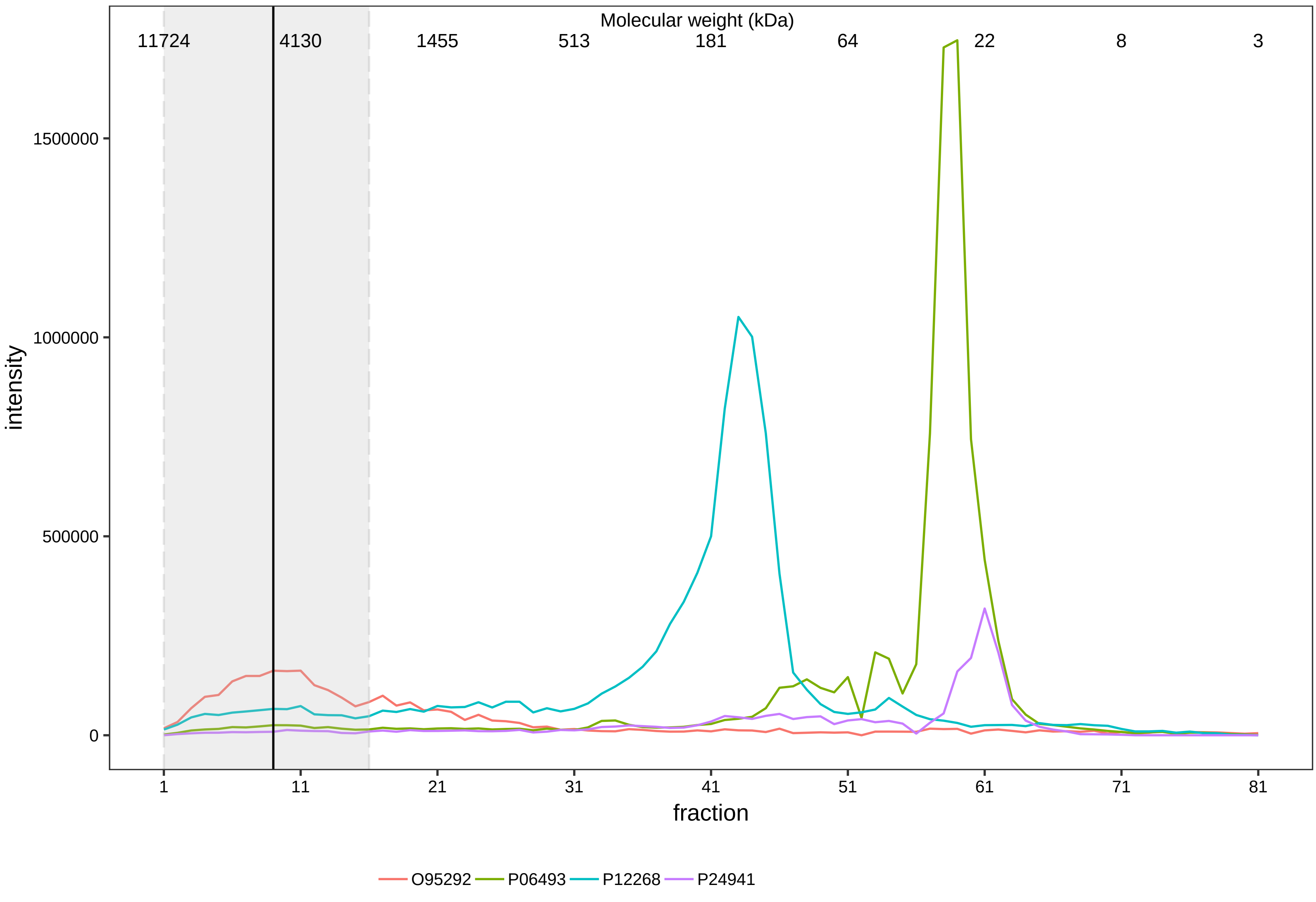

Feature ID 233

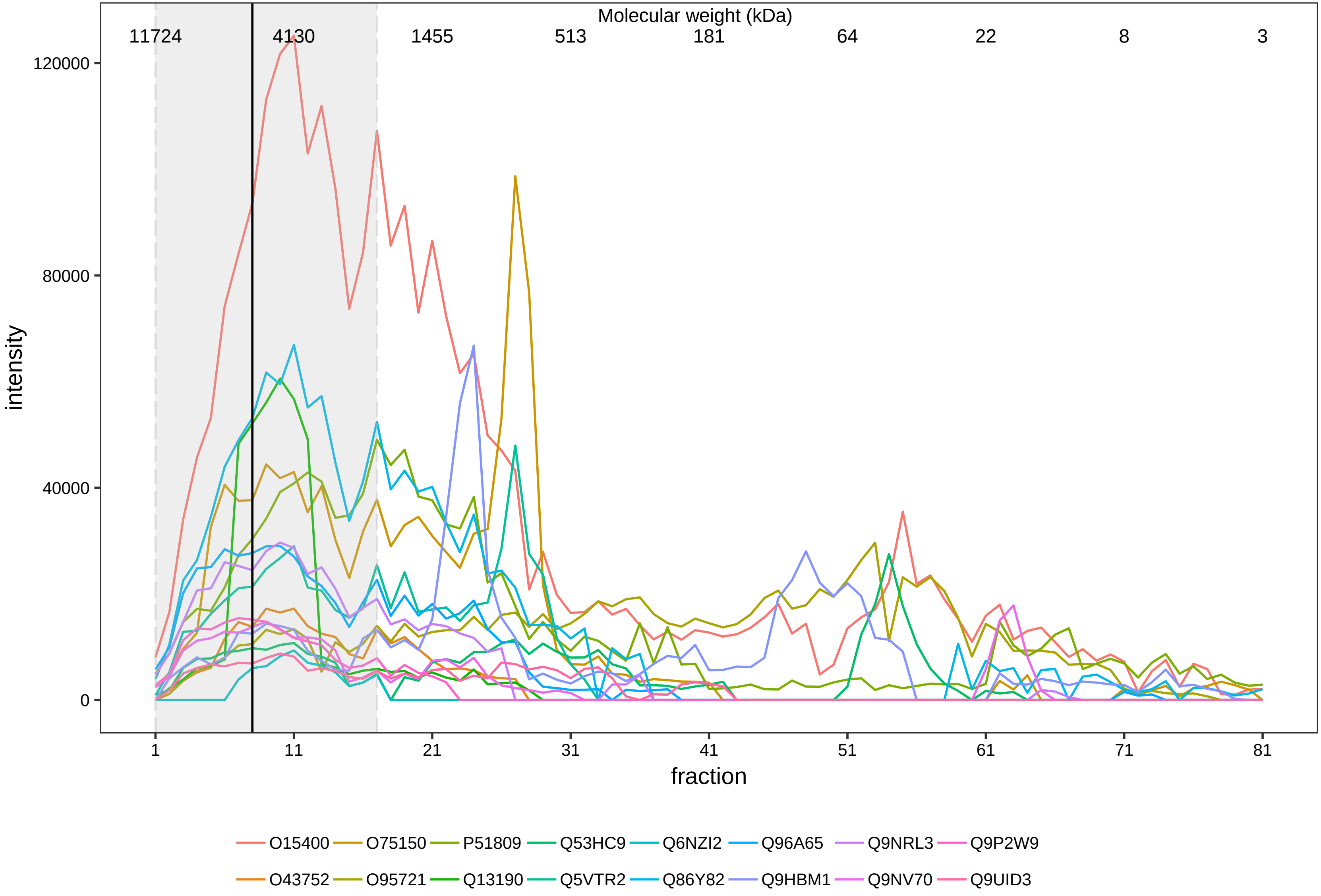

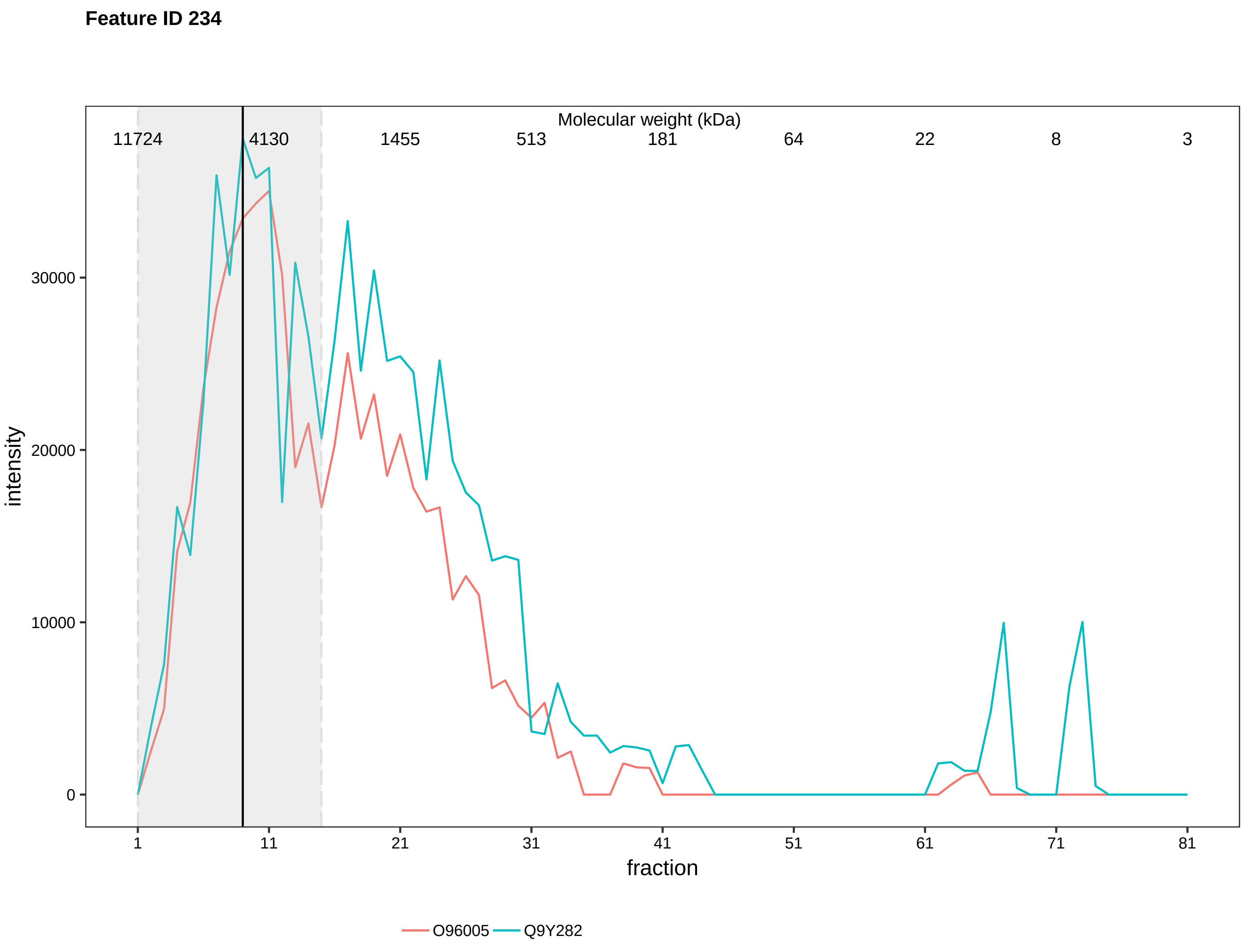

Feature ID 234
Molecular weight (kDa)
11724
4130
1455
513
181
64
22
8
3
intensity
30000
20000
10000
0
1
11
21
31
41
51
61
71
81
fraction
O96005
Q9Y282

Feature ID 235

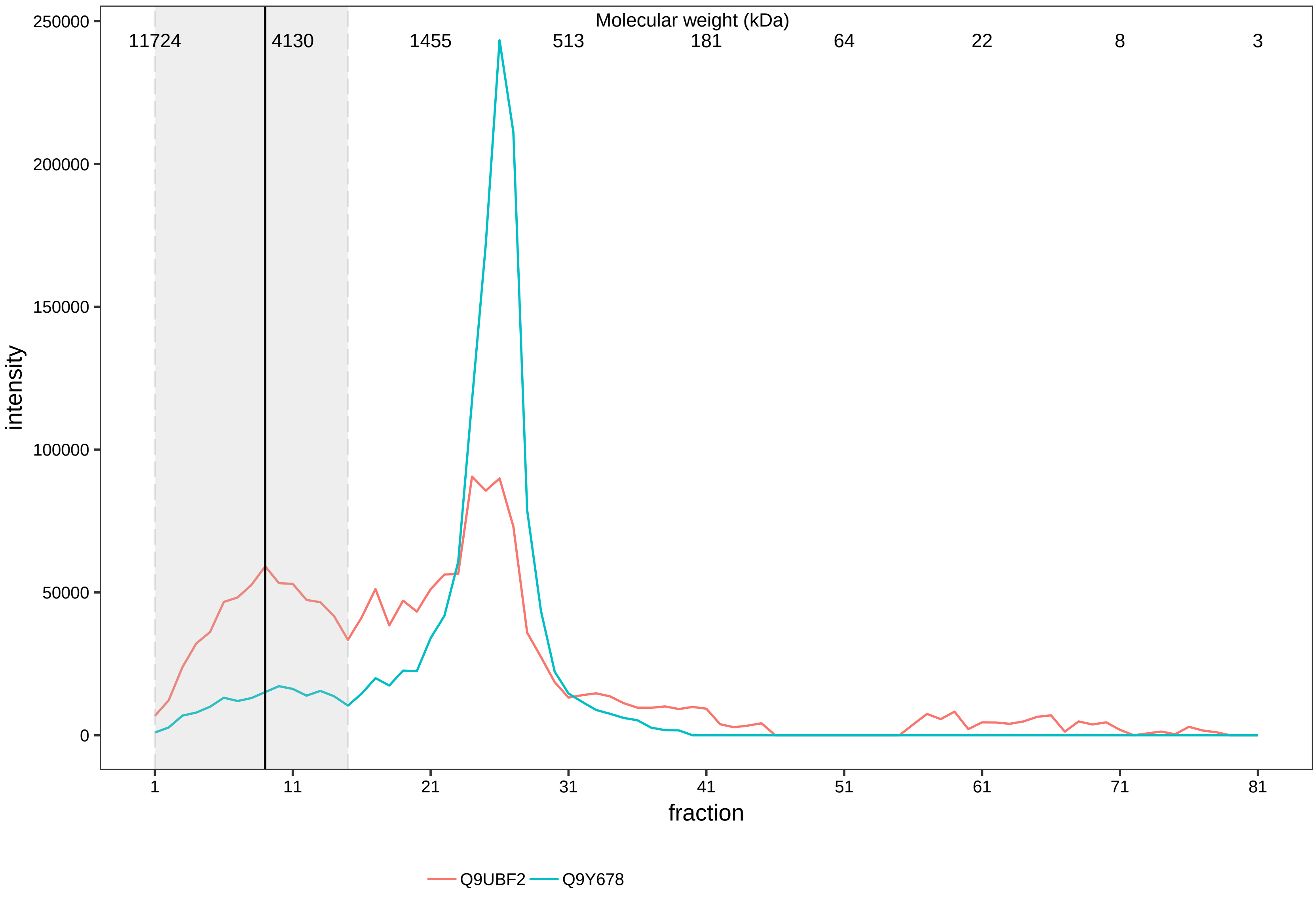

Feature ID 236

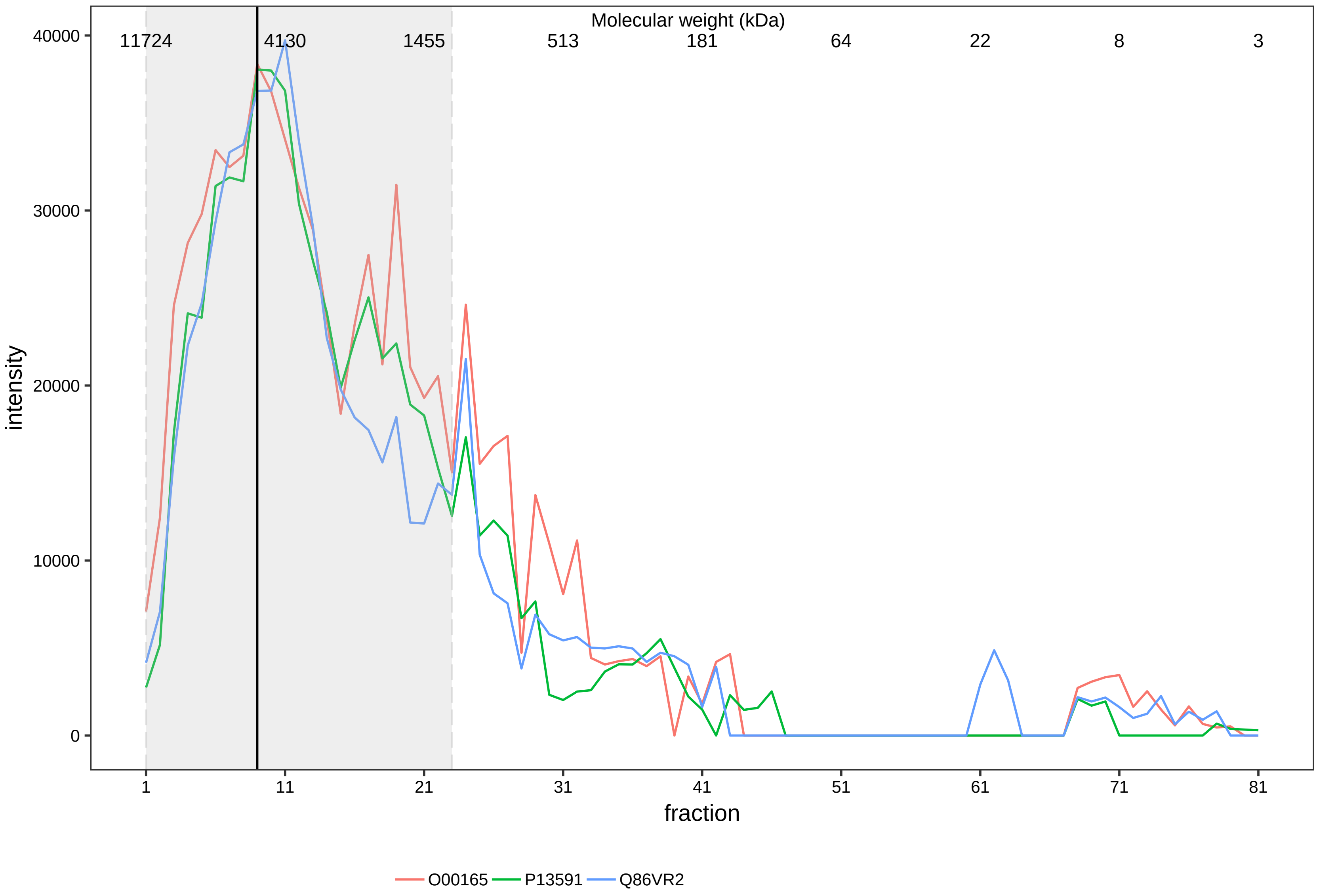

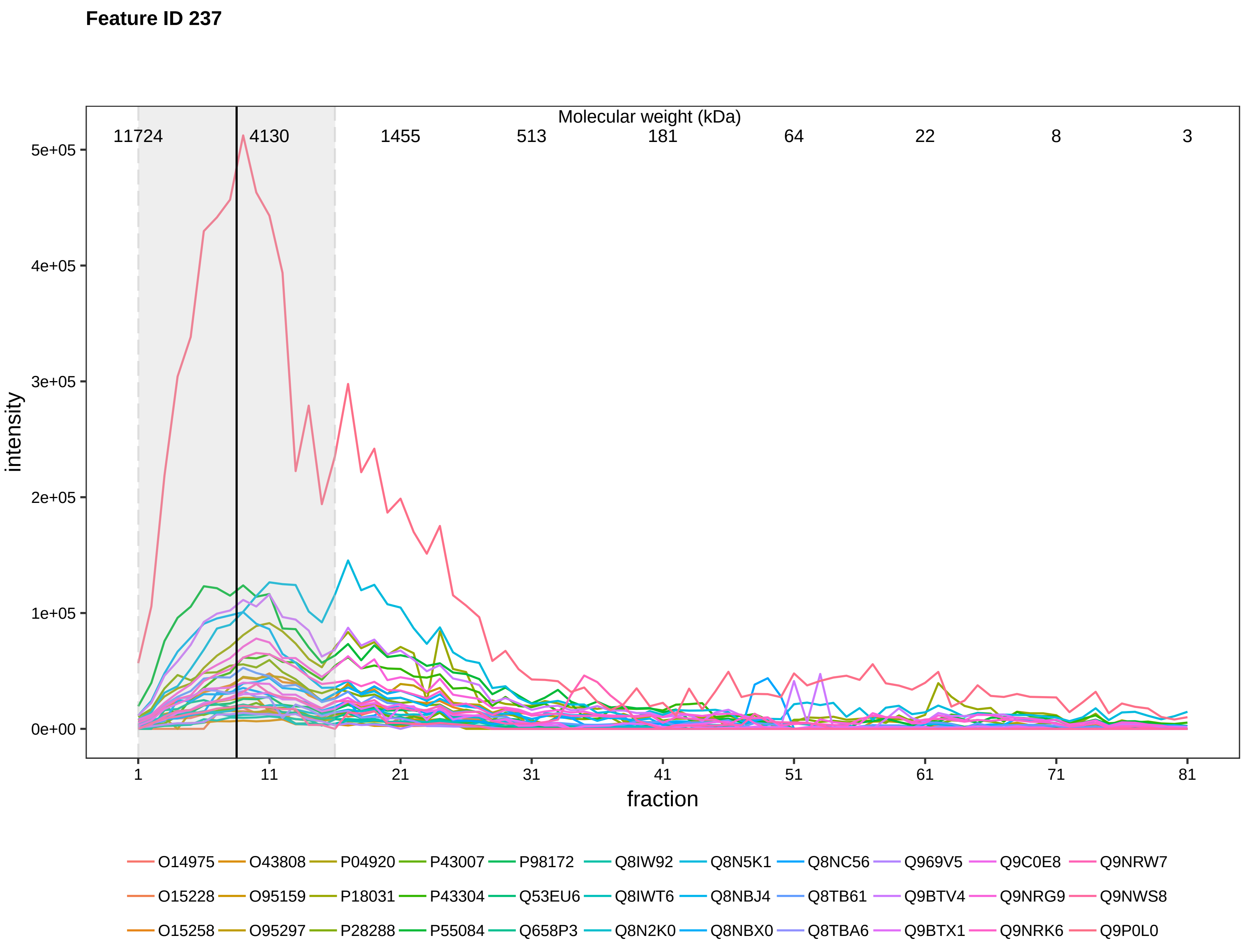

Feature ID 237
Molecular weight (kDa)
11724
4130
1455
513
181
64
22
8
3
5e+05
4e+05
3e+05
2e+05
1e+05
0e+00
intensity
1
11
21
31
41
51
61
71
81
fraction
O14975
O43808
P04920
P43007
P98172
Q8IW92
Q8N5K1
Q8NC56
Q969V5
Q9C0E8
Q9NRW7
O15228
O95159
P18031
P43304
Q53EU6
Q8IWT6
Q8NBJ4
Q8TB61
Q9BTV4
Q9NRG9
Q9NWS8
O15258
O95297
P28288
P55084
Q658P3
Q8N2K0
Q8NBX0
Q8TBA6
Q9BTX1
Q9NRK6
Q9P0L0

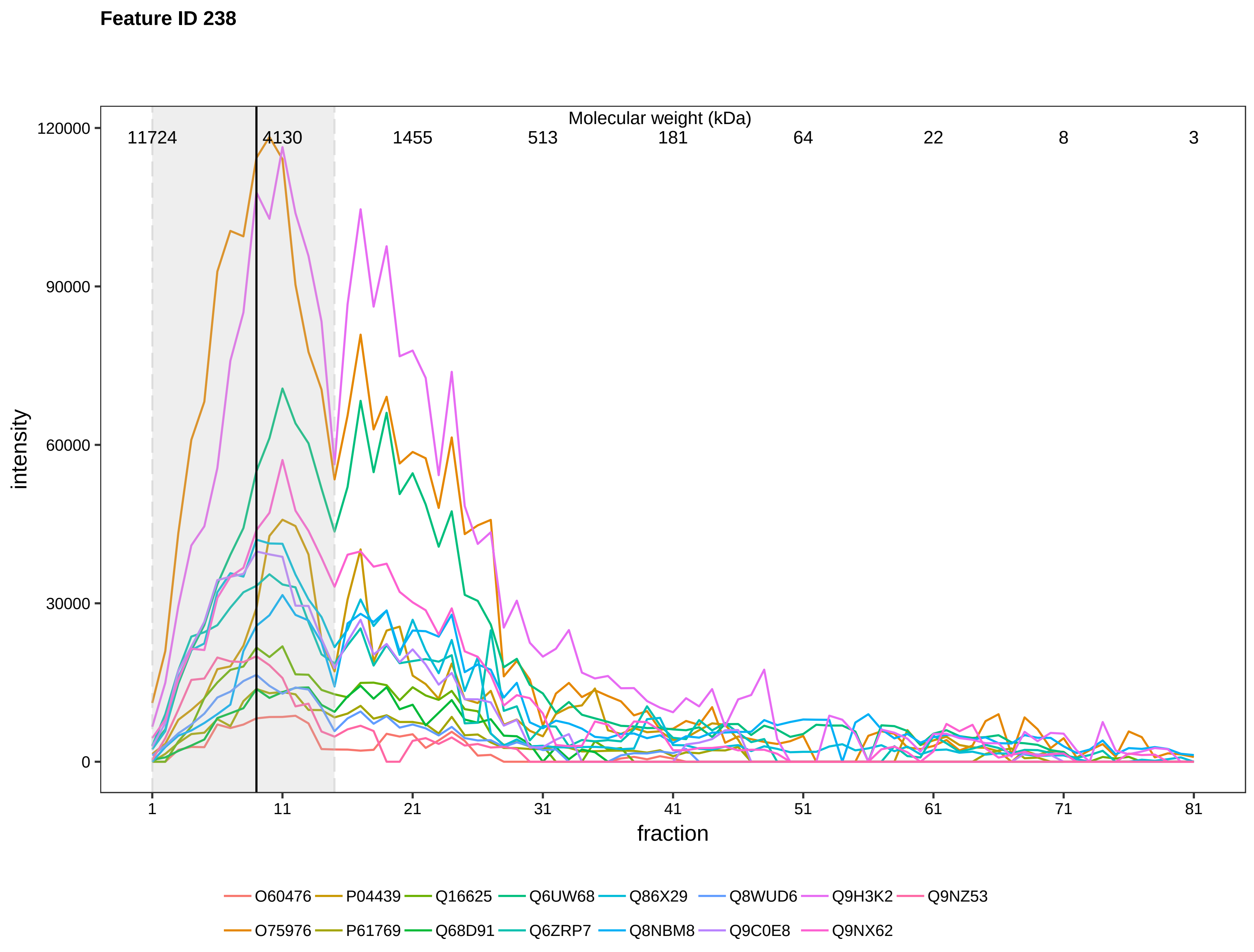
Feature ID 238
Molecular weight (kDa)
11724
4130
1455
513
181
64
22
8
3
120000
90000
60000
30000
0
intensity
1
11
21
31
41
51
61
71
81
fraction
O60476
P04439
Q16625
Q6UW68
Q86X29
Q8WUD6
Q9H3K2
Q9NZ53
O75976
P61769
Q68D91
Q6ZRP7
Q8NBM8
Q9C0E8
Q9NX62

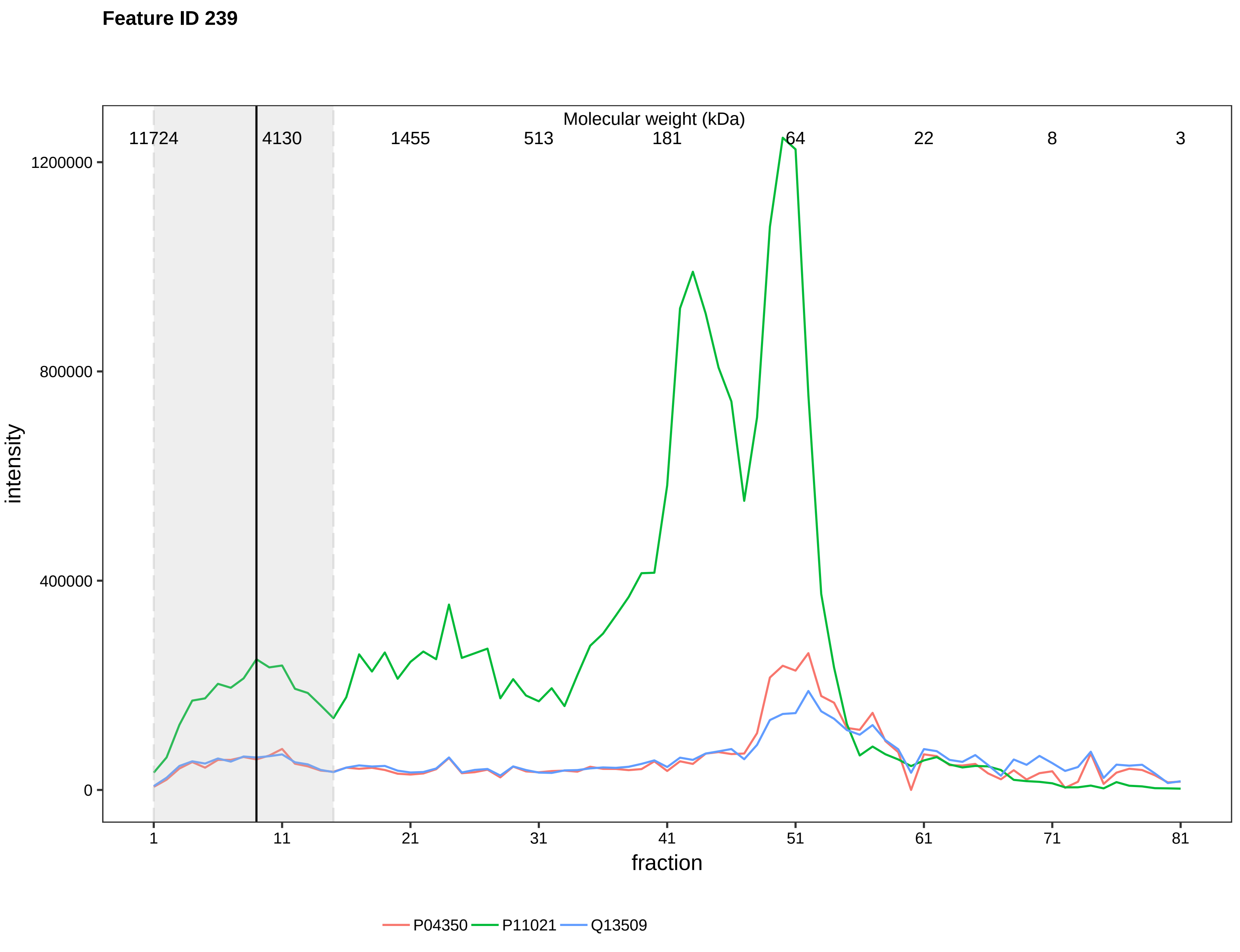
Feature ID 239
Molecular weight (kDa)
11724
4130
1455
513
181
64
22
8
3
1200000
800000
400000
0
intensity
1
11
21
31
41
51
61
71
81
fraction
P04350
P11021
Q13509

Feature ID 240

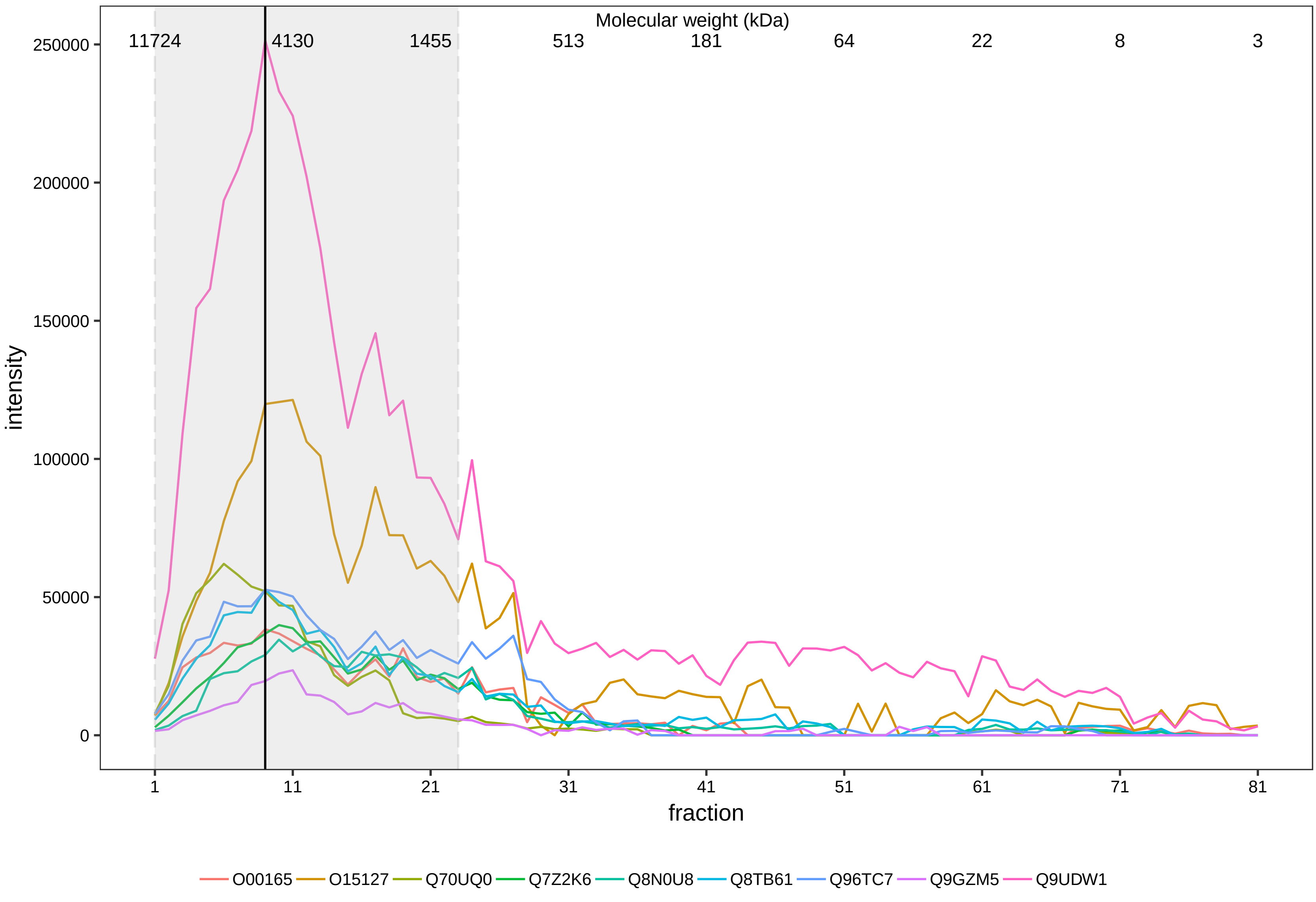

Feature ID 241

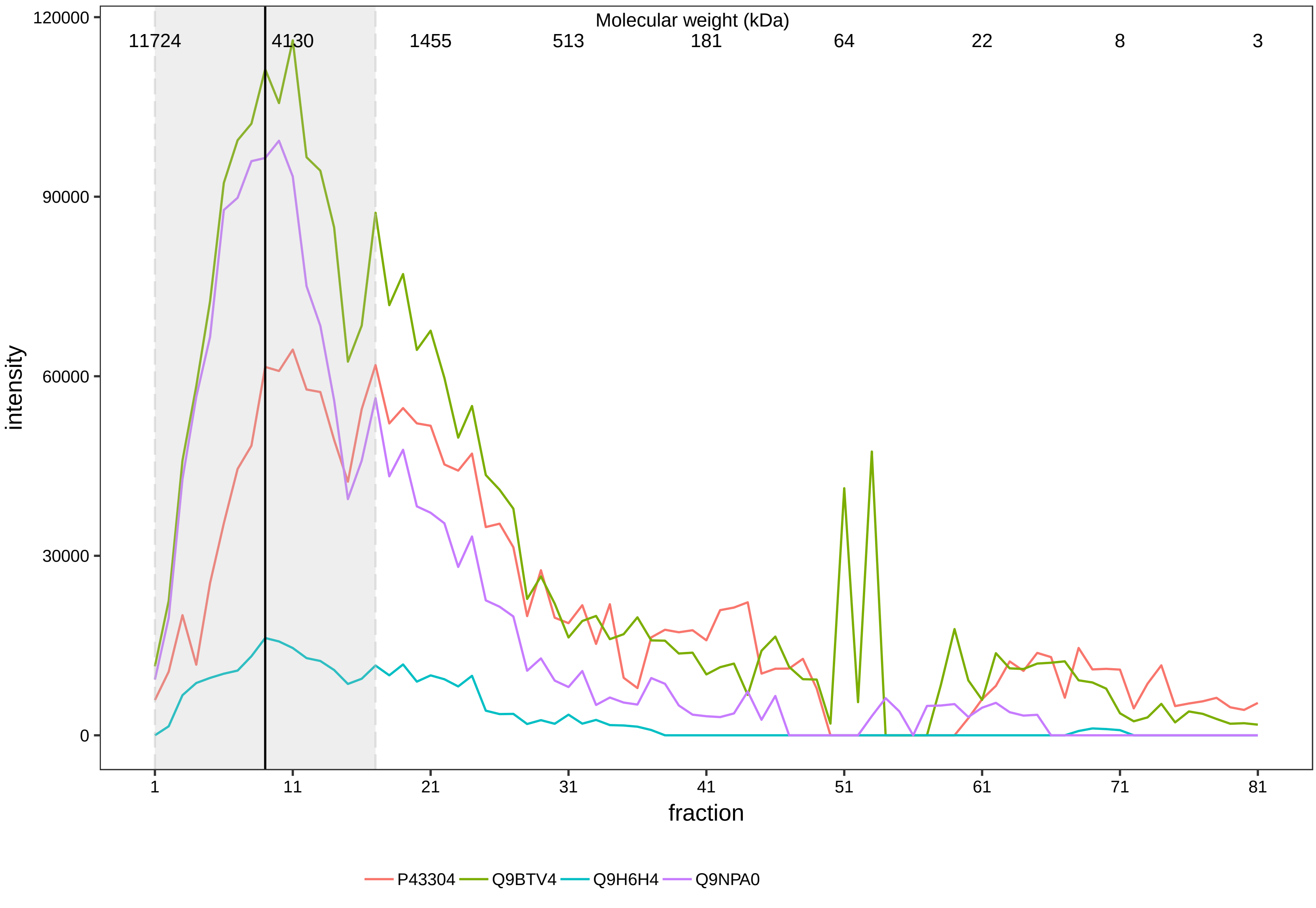

Feature ID 242

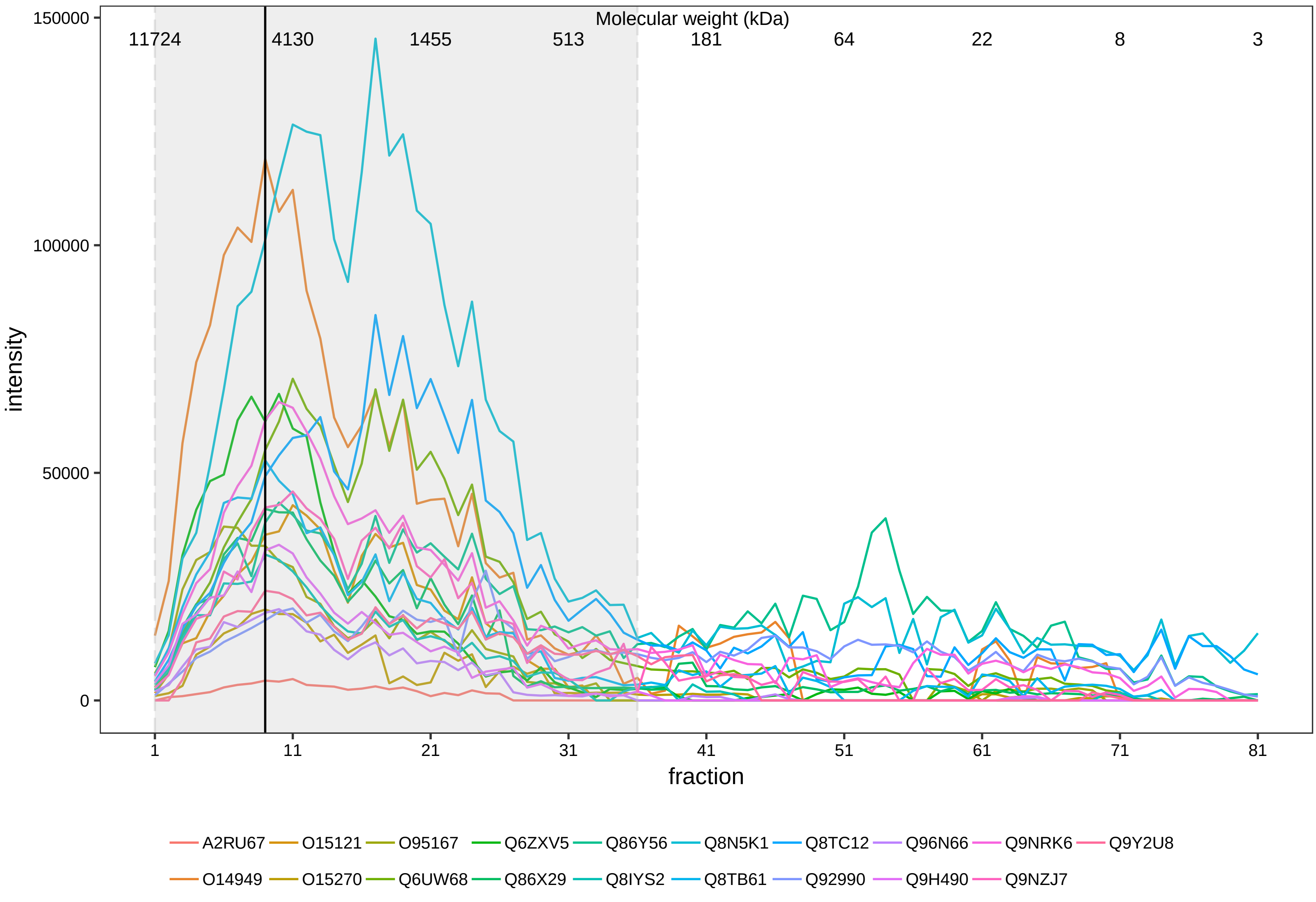

Feature ID 243

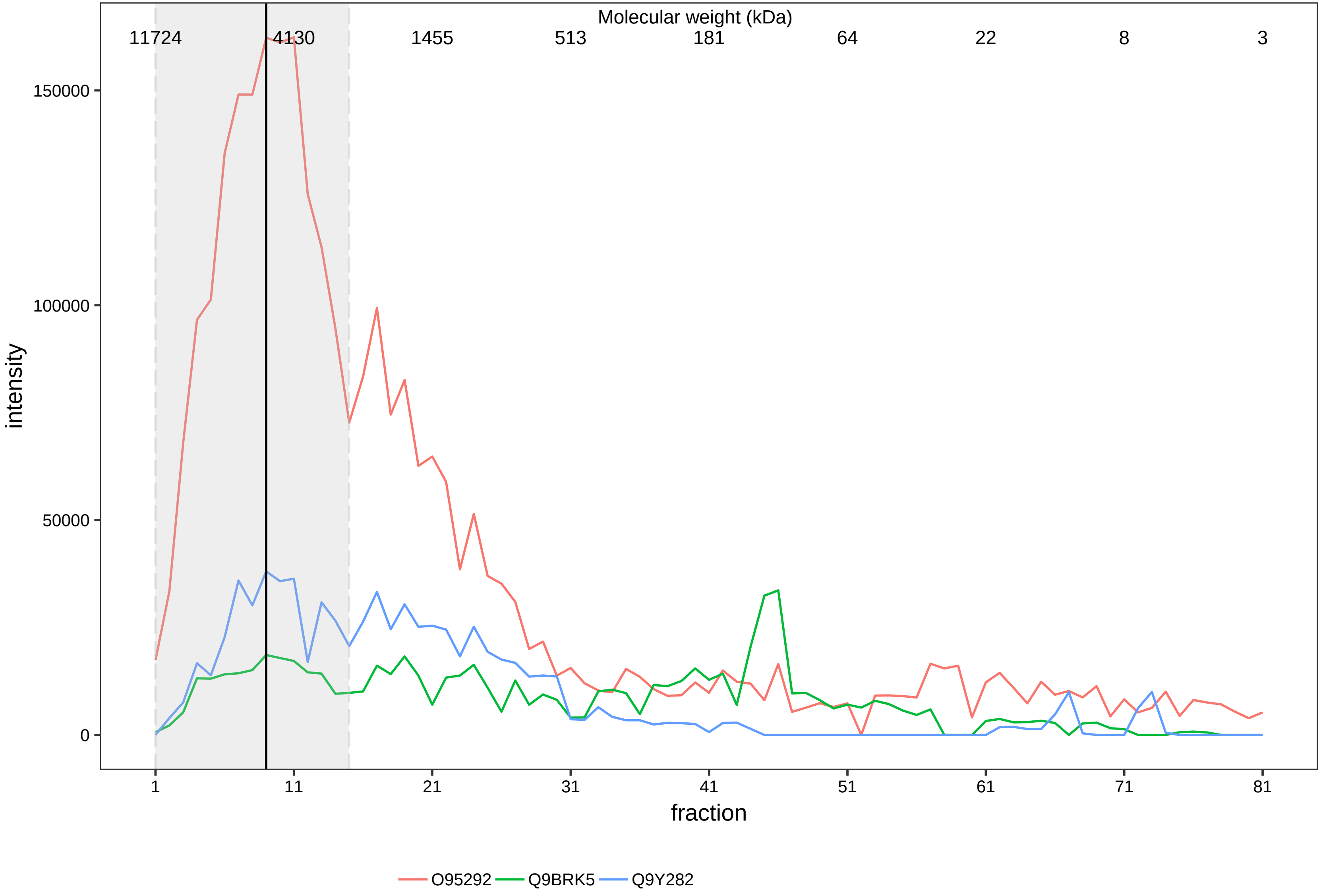

Feature ID 244

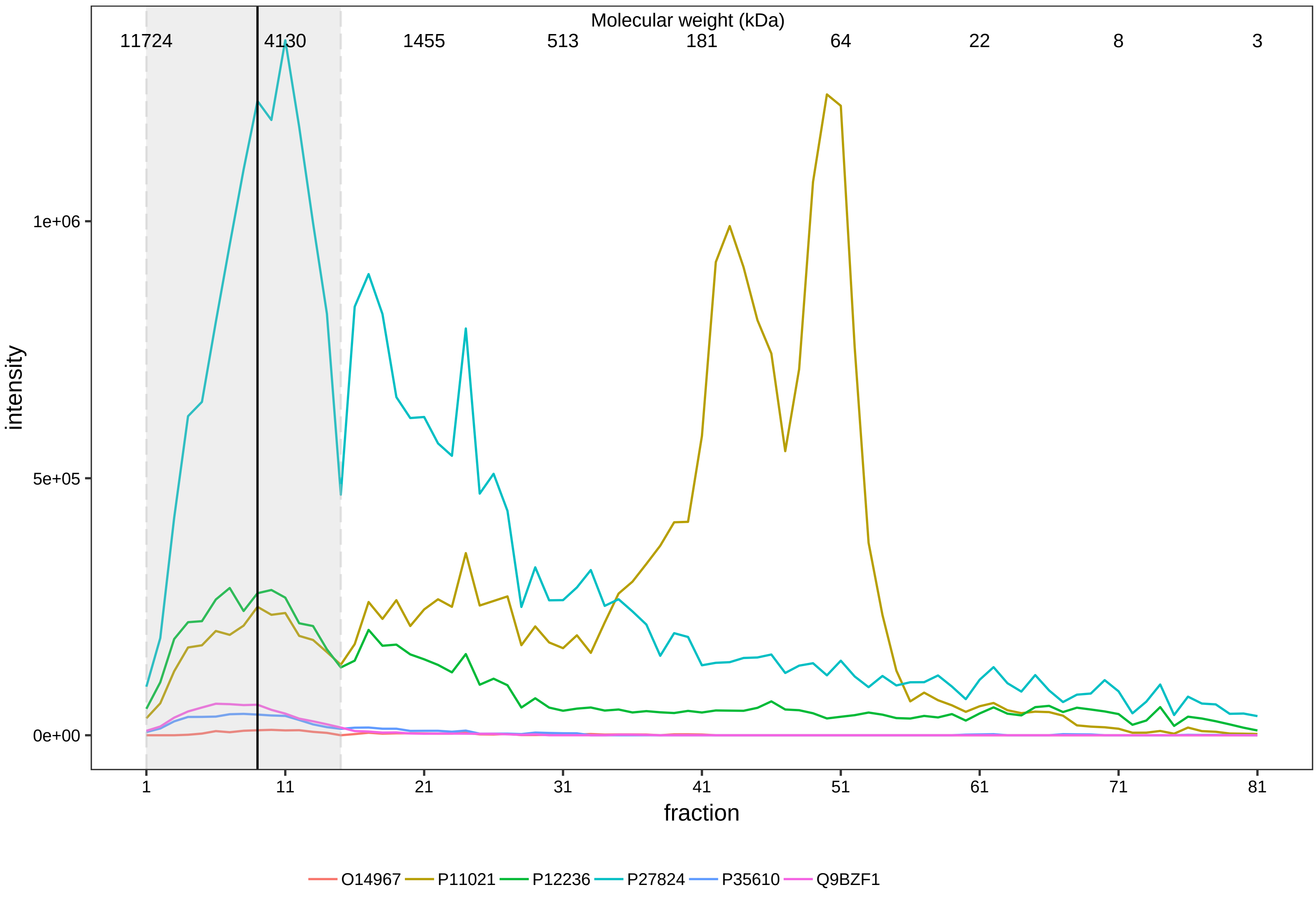

Feature ID 245

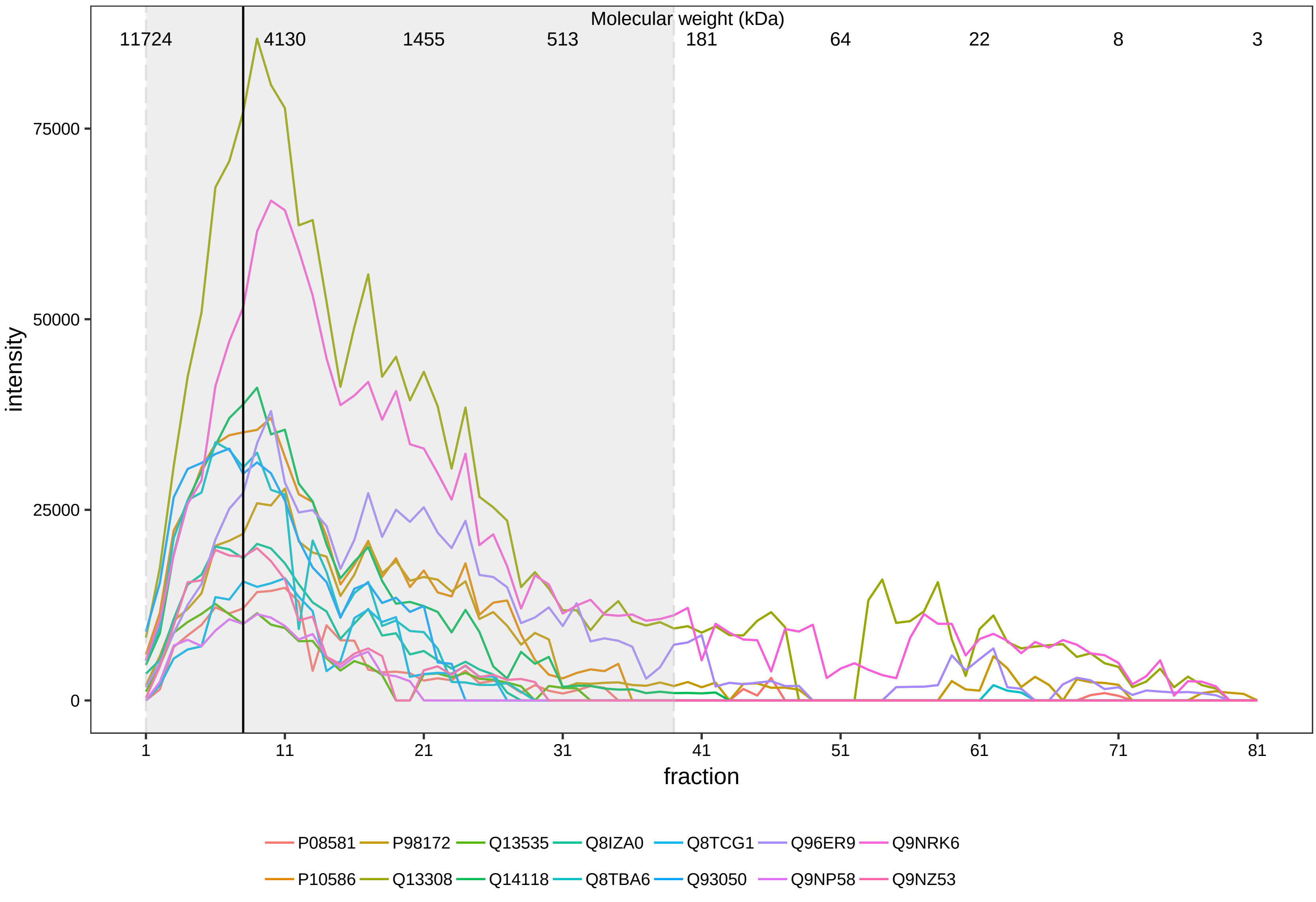

Feature ID 246

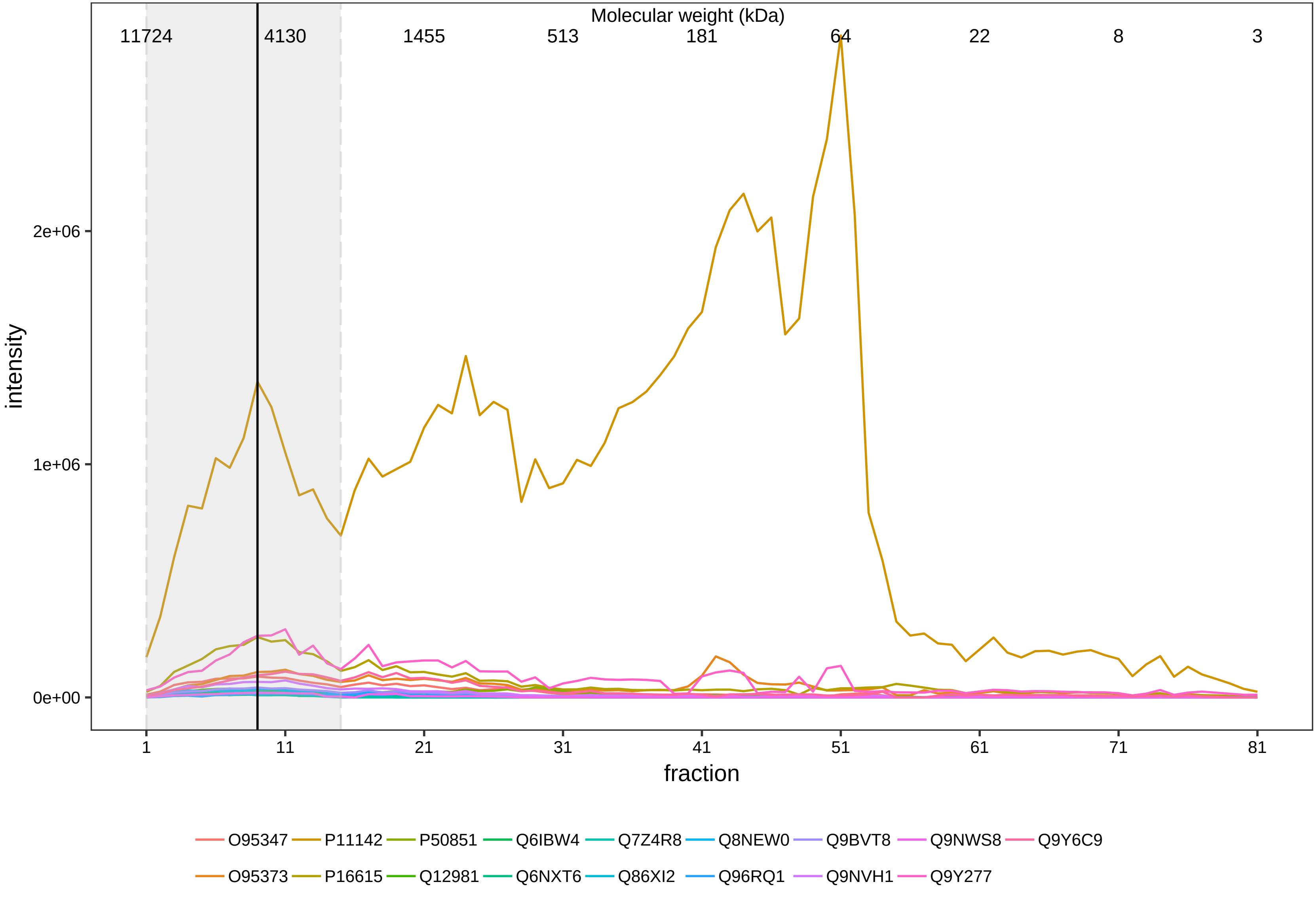

Feature ID 247

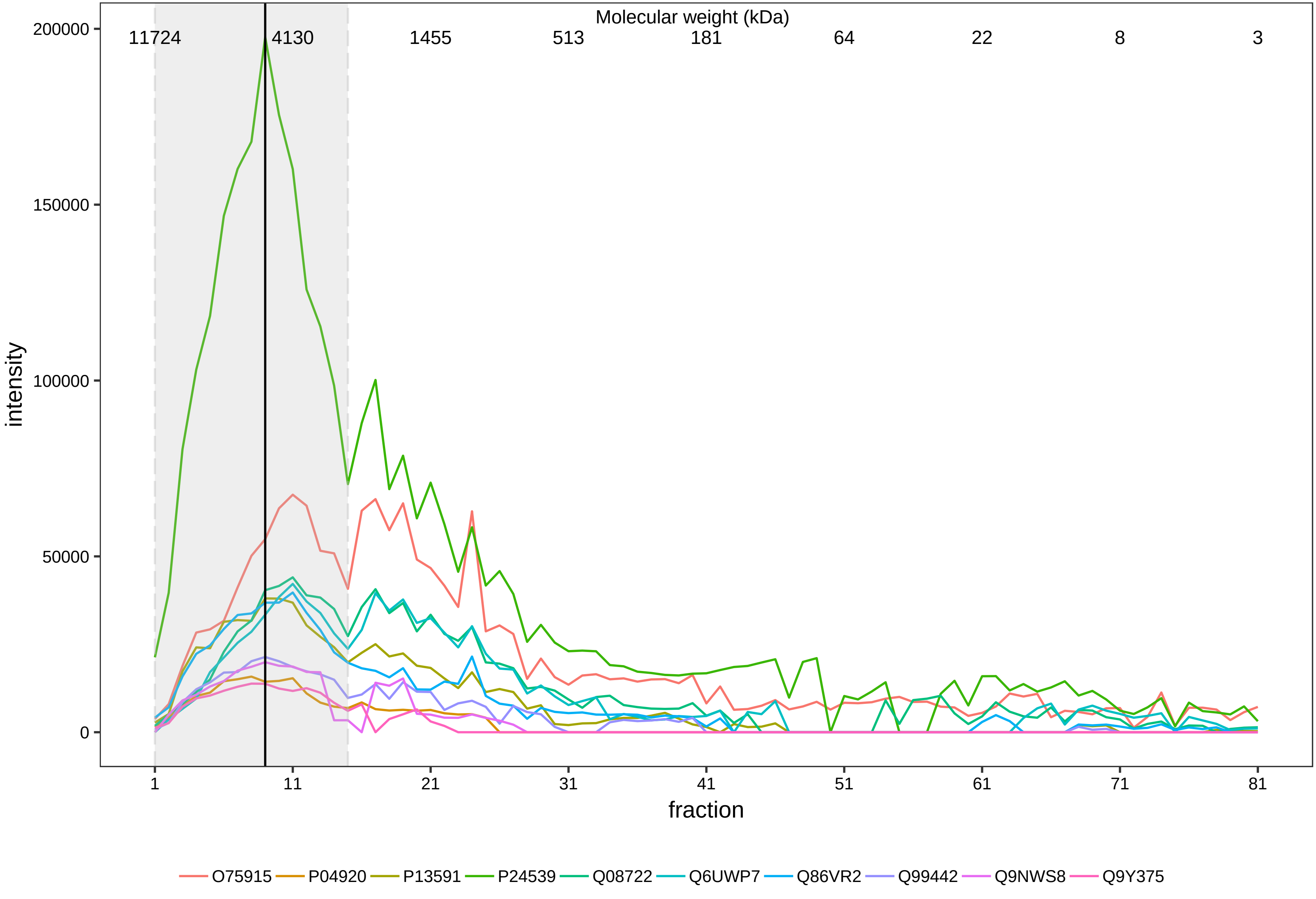

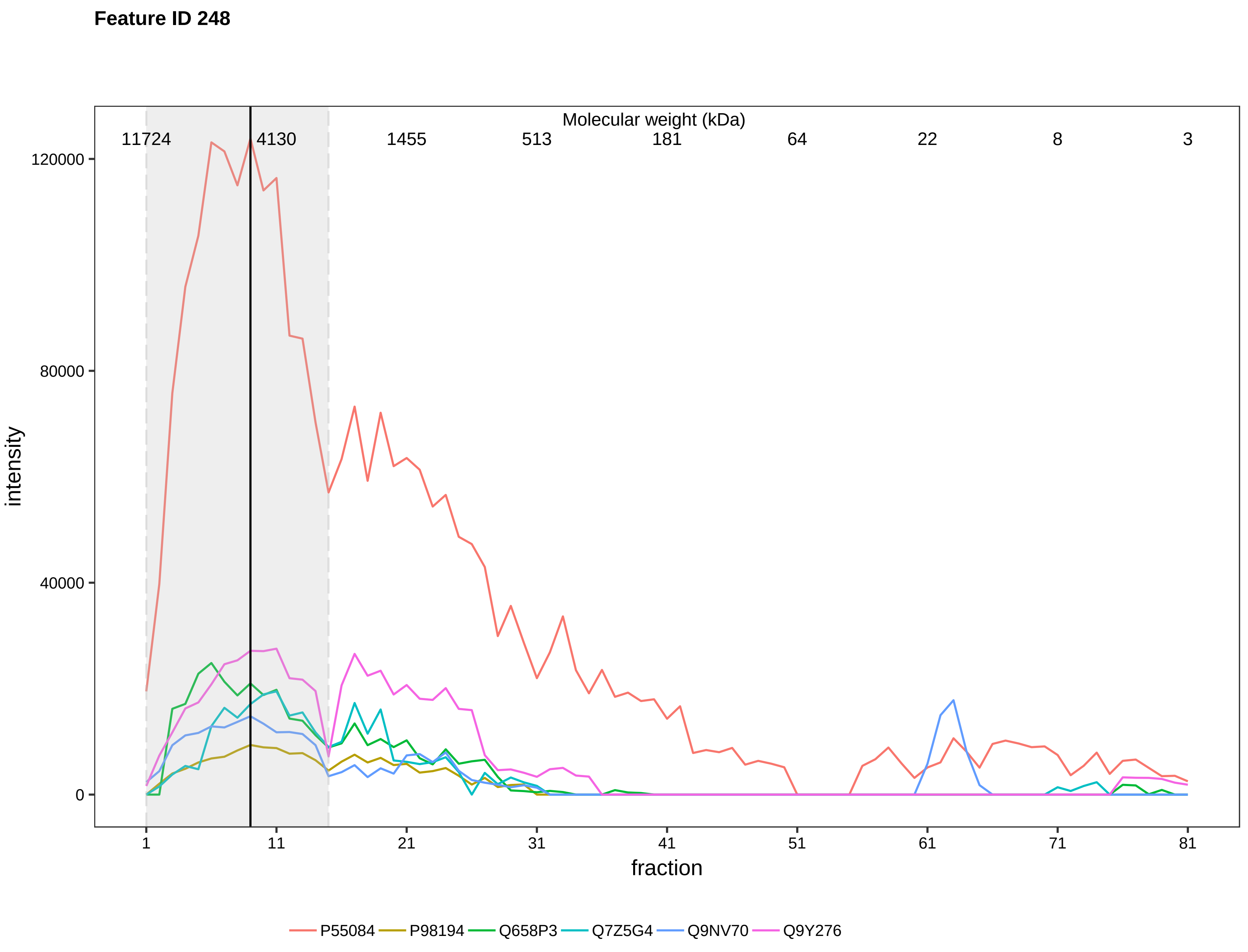
Feature ID 248
Molecular weight (kDa)
11724
4130
1455
513
181
64
22
8
3
120000
80000
40000
0
intensity
1
11
21
31
41
51
61
71
81
fraction
P55084
P98194
Q658P3
Q7Z5G4
Q9NV70
Q9Y276

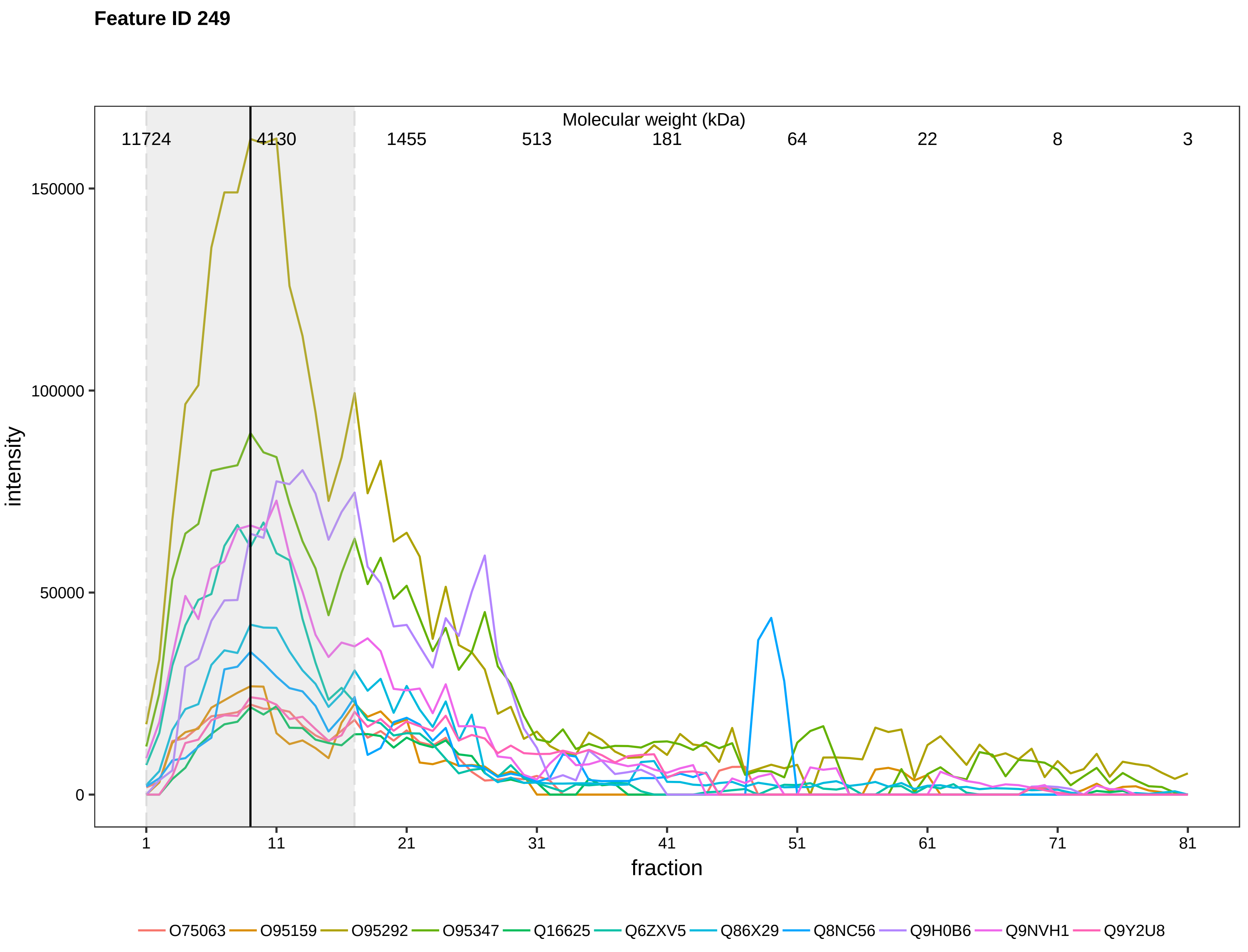
Feature ID 249
Molecular weight (kDa)
11724
4130
1455
513
181
64
22
8
3
intensity
150000
100000
50000
0
1
11
21
31
41
51
61
71
81
fraction
O75063
O95159
O95292
O95347
Q16625
Q6ZXV5
Q86X29
Q8NC56
Q9H0B6
Q9NVH1
Q9Y2U8

Feature ID 250

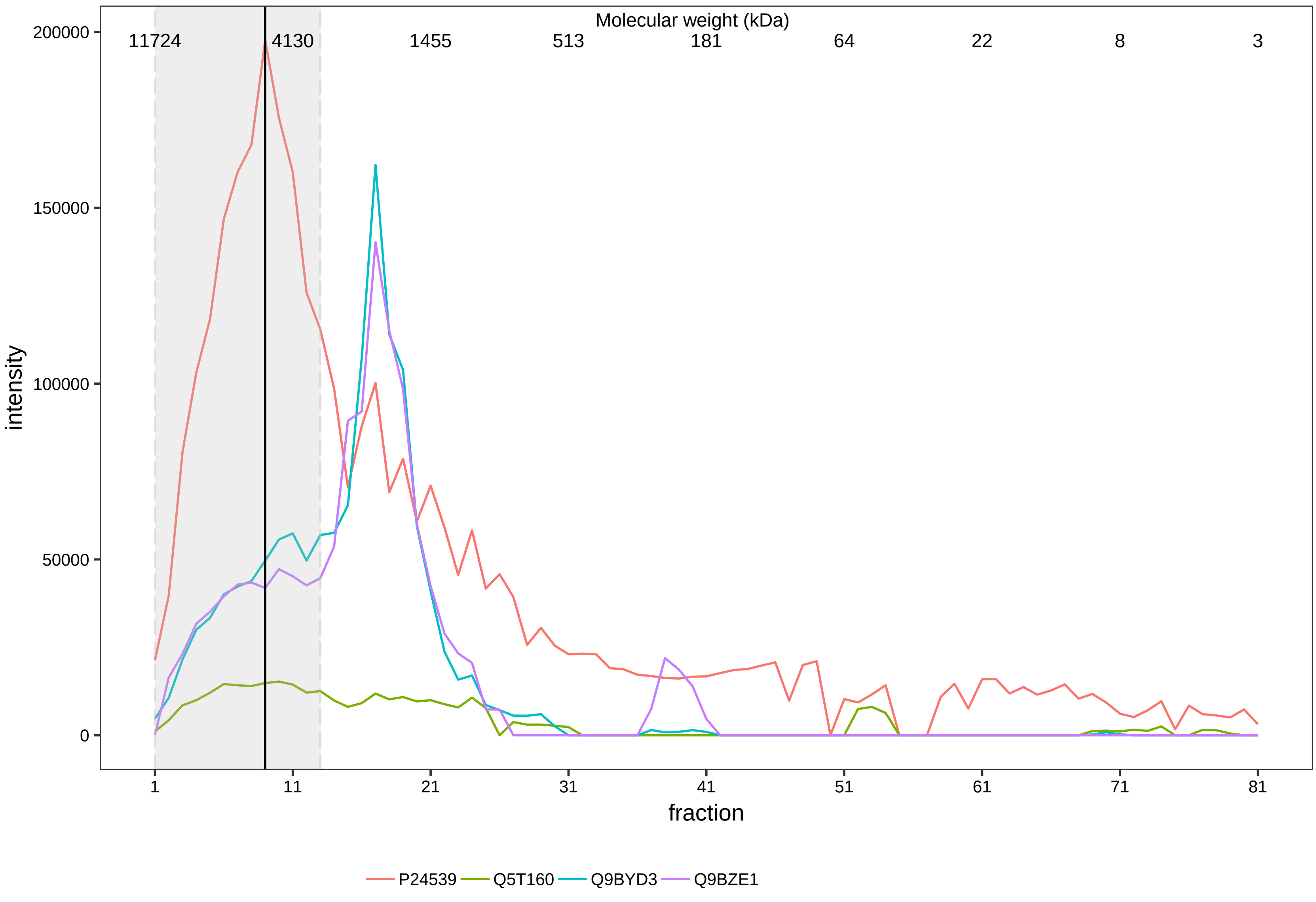

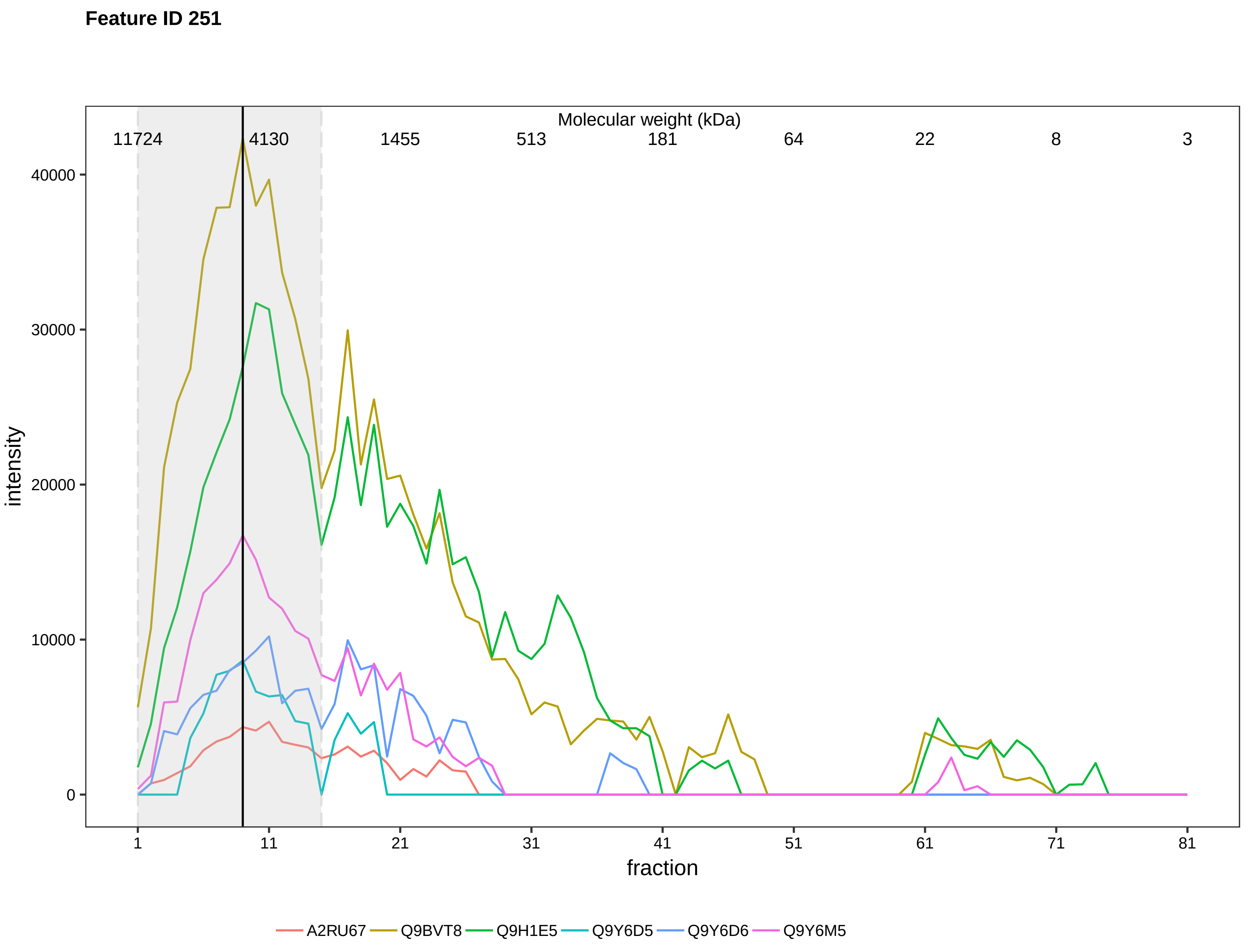

Feature ID 251
Molecular weight (kDa)
11724
4130
1455
513
181
64
22
8
3
40000
30000
20000
10000
0
intensity
1
11
21
31
41
51
61
71
81
fraction
A2RU67
Q9BVT8
Q9H1E5
Q9Y6D5
Q9Y6D6
Q9Y6M5

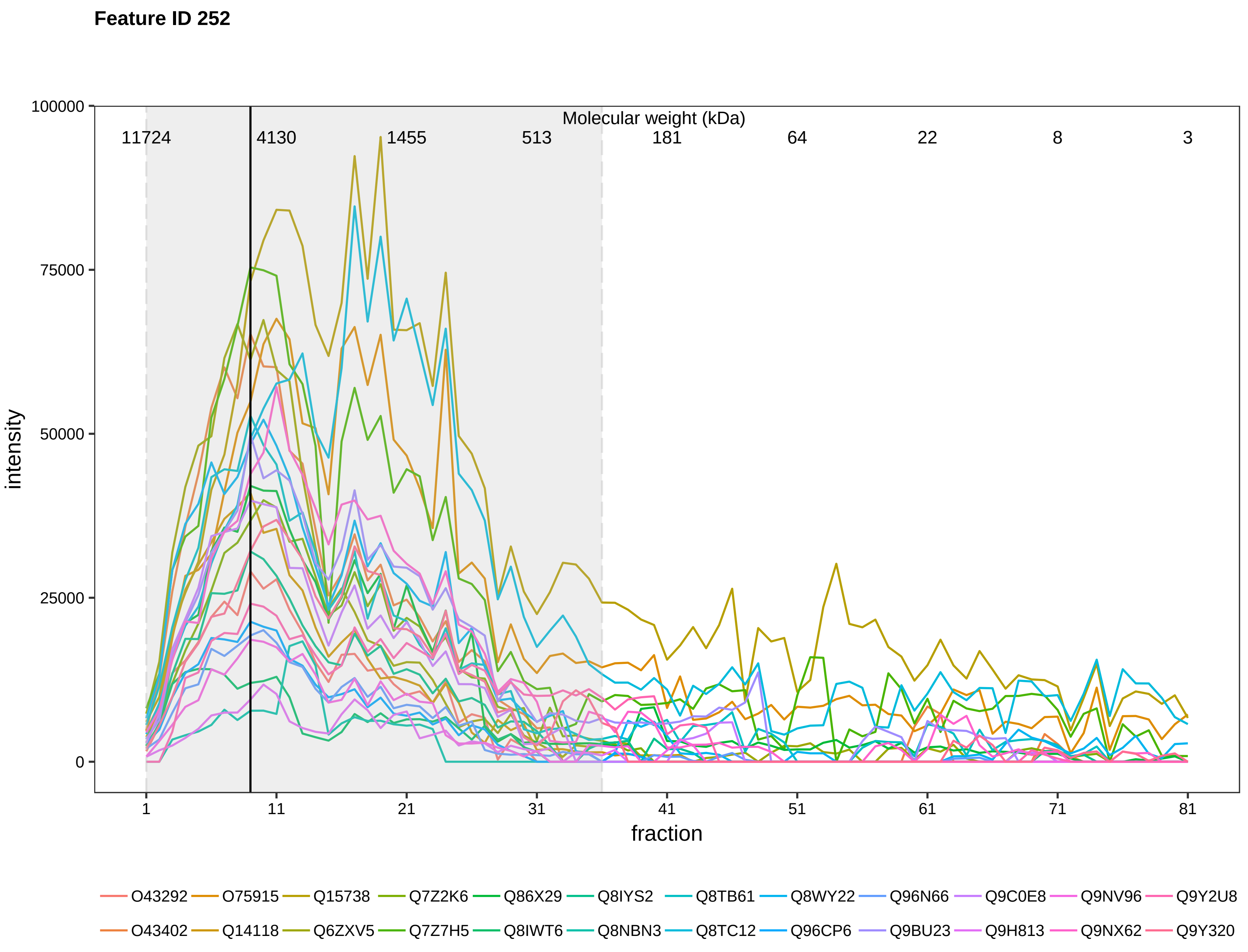

Feature ID 252
Molecular weight (kDa)
11724
4130
1455
513
181
64
22
8
3
100000
75000
50000
25000
0
intensity
1
11
21
31
41
51
61
71
81
fraction
O43292
O75915
Q15738
Q7Z2K6
Q86X29
Q8IYS2
Q8TB61
Q8WY22
Q96N66
Q9C0E8
Q9NV96
Q9Y2U8
O43402
Q14118
Q6ZXV5
Q7Z7H5
Q8IWT6
Q8NBN3
Q8TC12
Q96CP6
Q9BU23
Q9H813
Q9NX62
Q9Y320

Feature ID 253

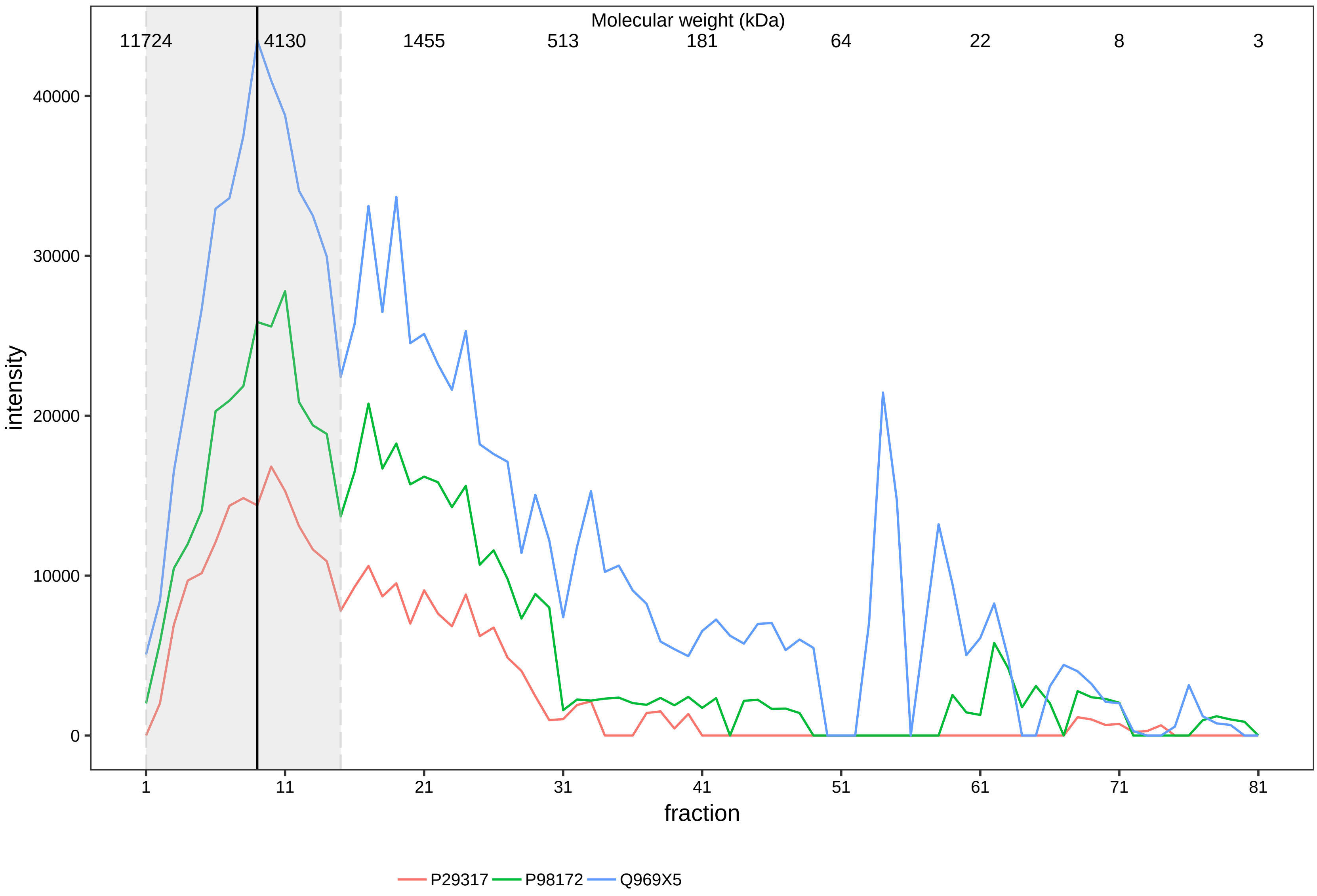

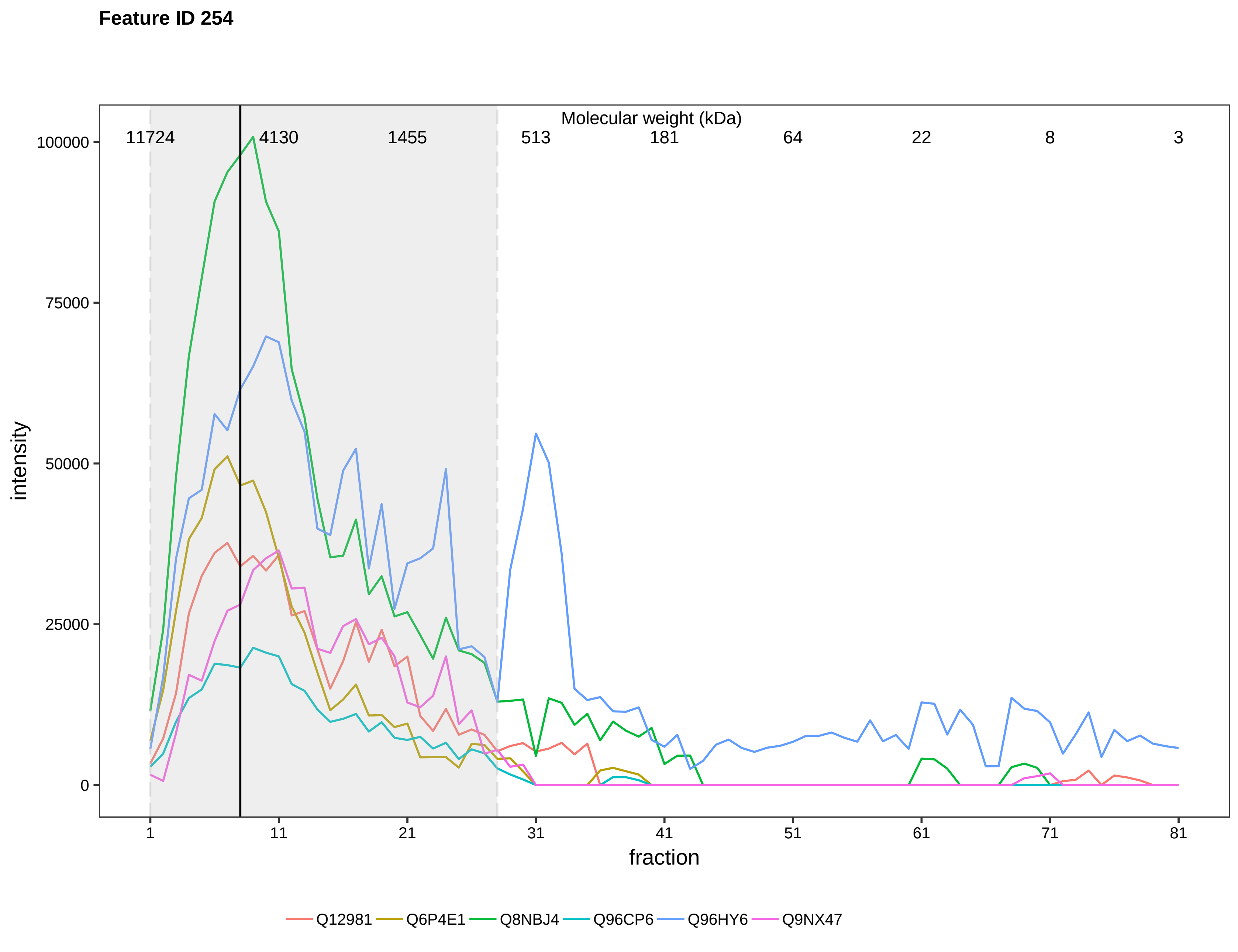
Feature ID 254
Molecular weight (kDa)
11724
4130
1455
513
181
64
22
8
3
100000
75000
50000
25000
0
intensity
1
11
21
31
41
51
61
71
81
fraction
Q12981
Q6P4E1
Q8NBJ4
Q96CP6
Q96HY6
Q9NX47

Feature ID 255

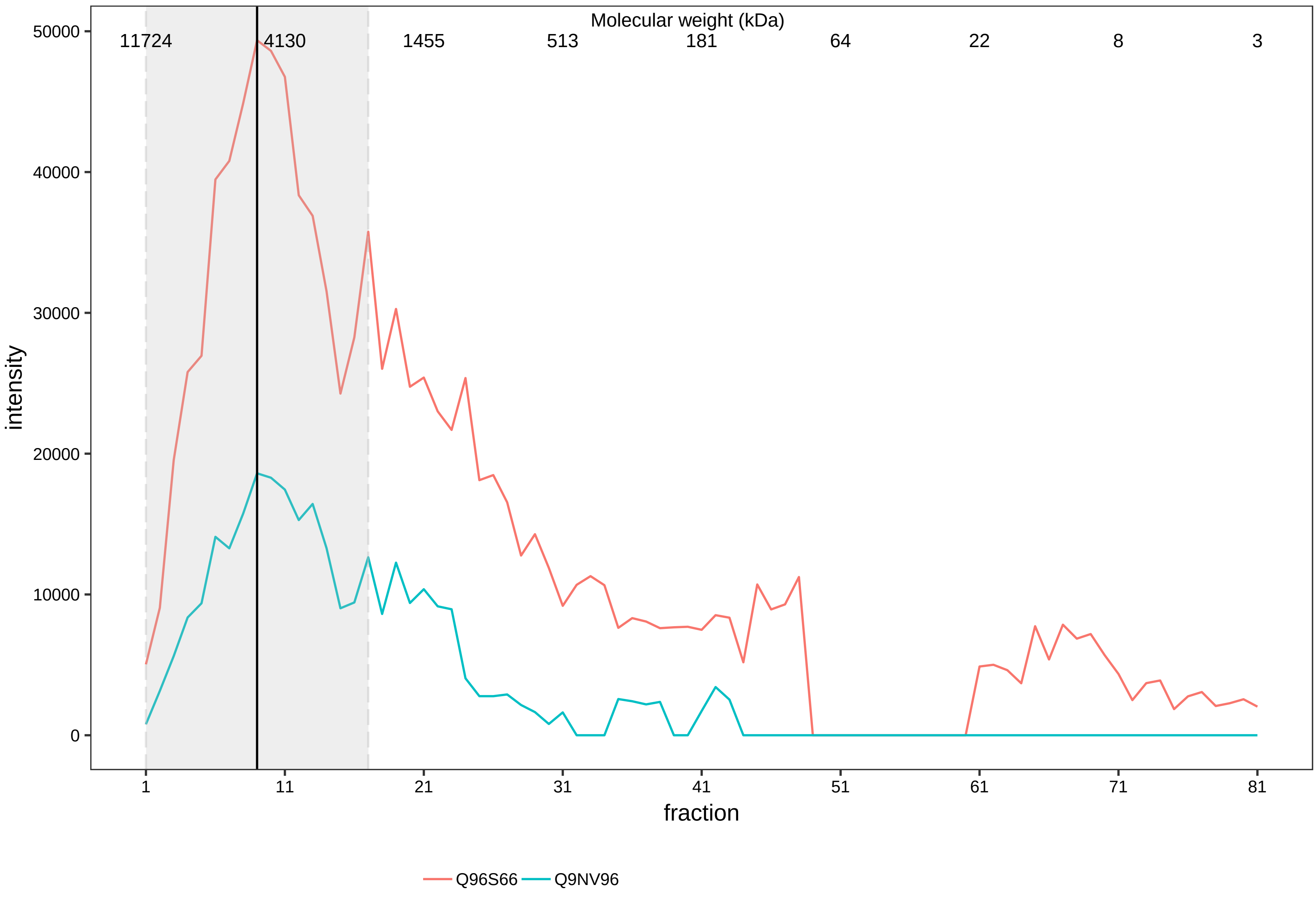

Feature ID 256

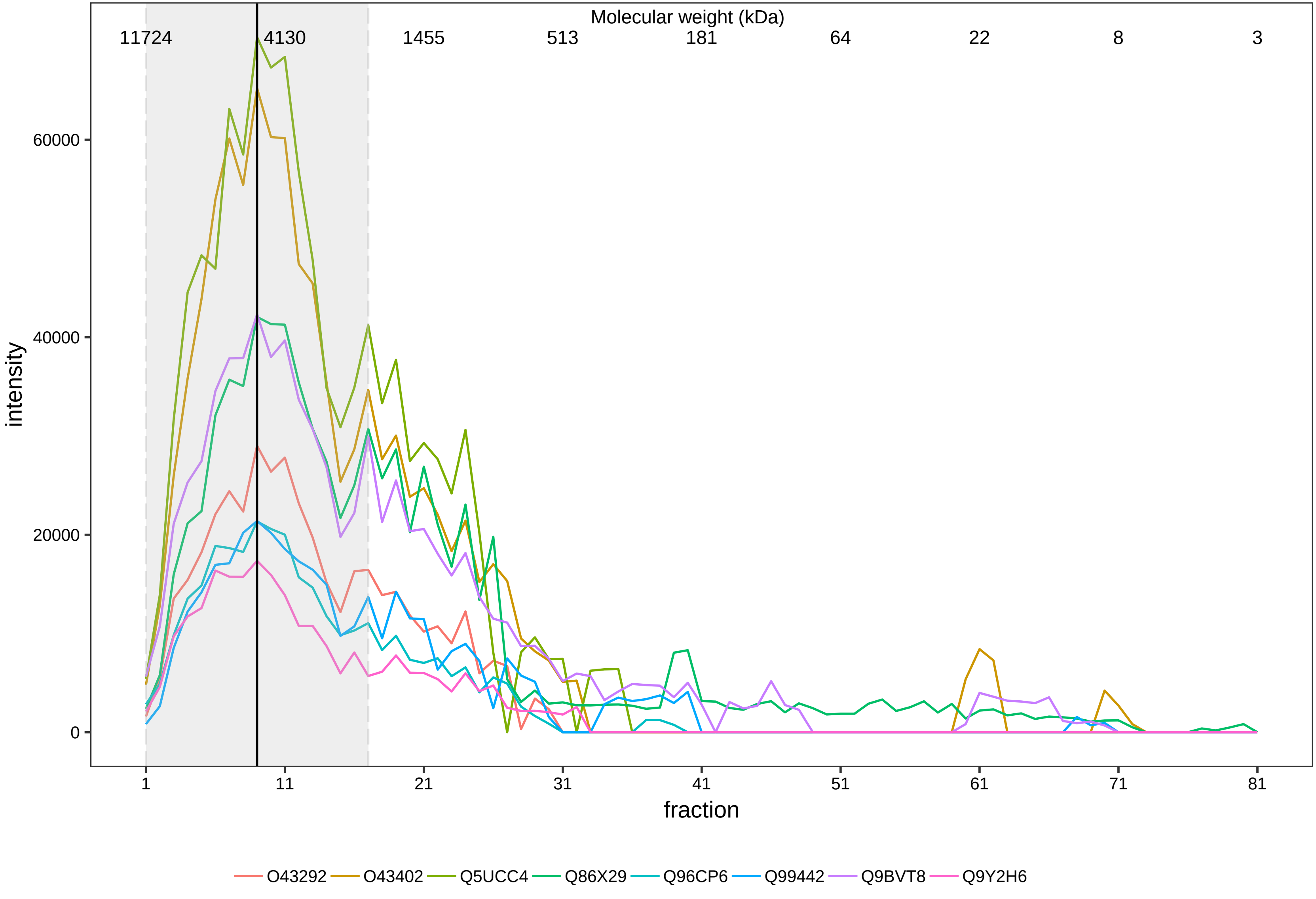

Feature ID 257

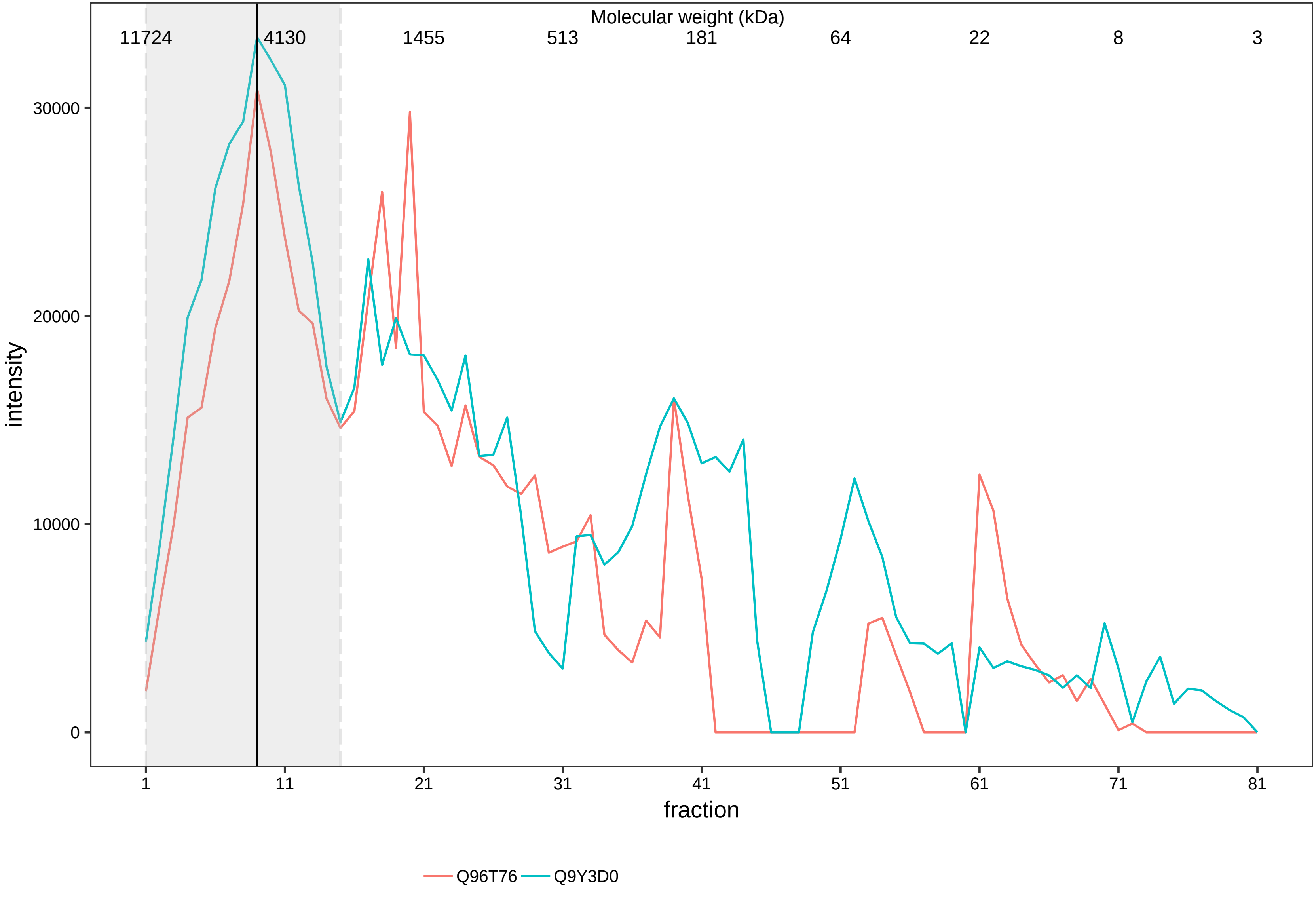

Feature ID 258

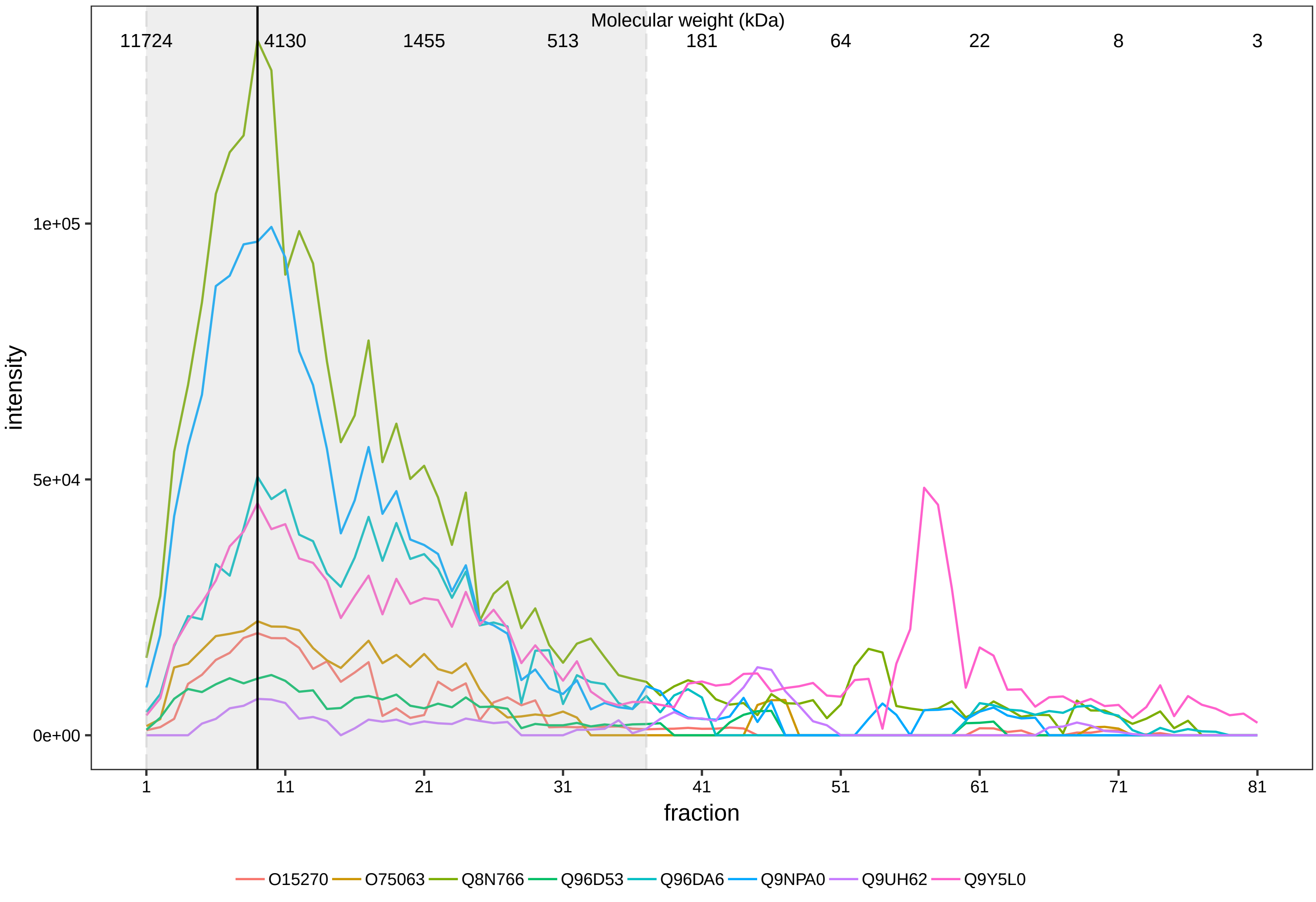

Feature ID 259

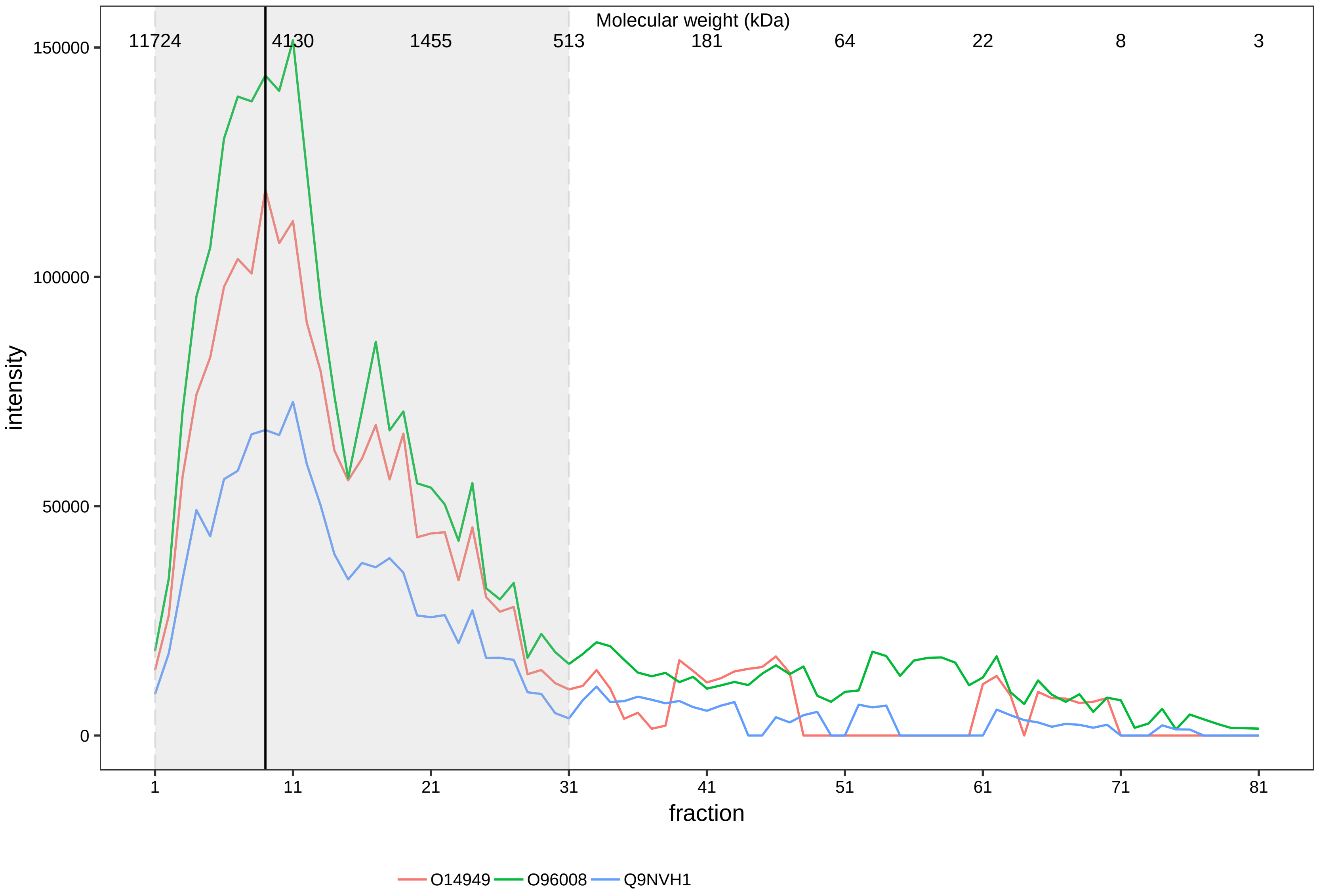

Feature ID 260

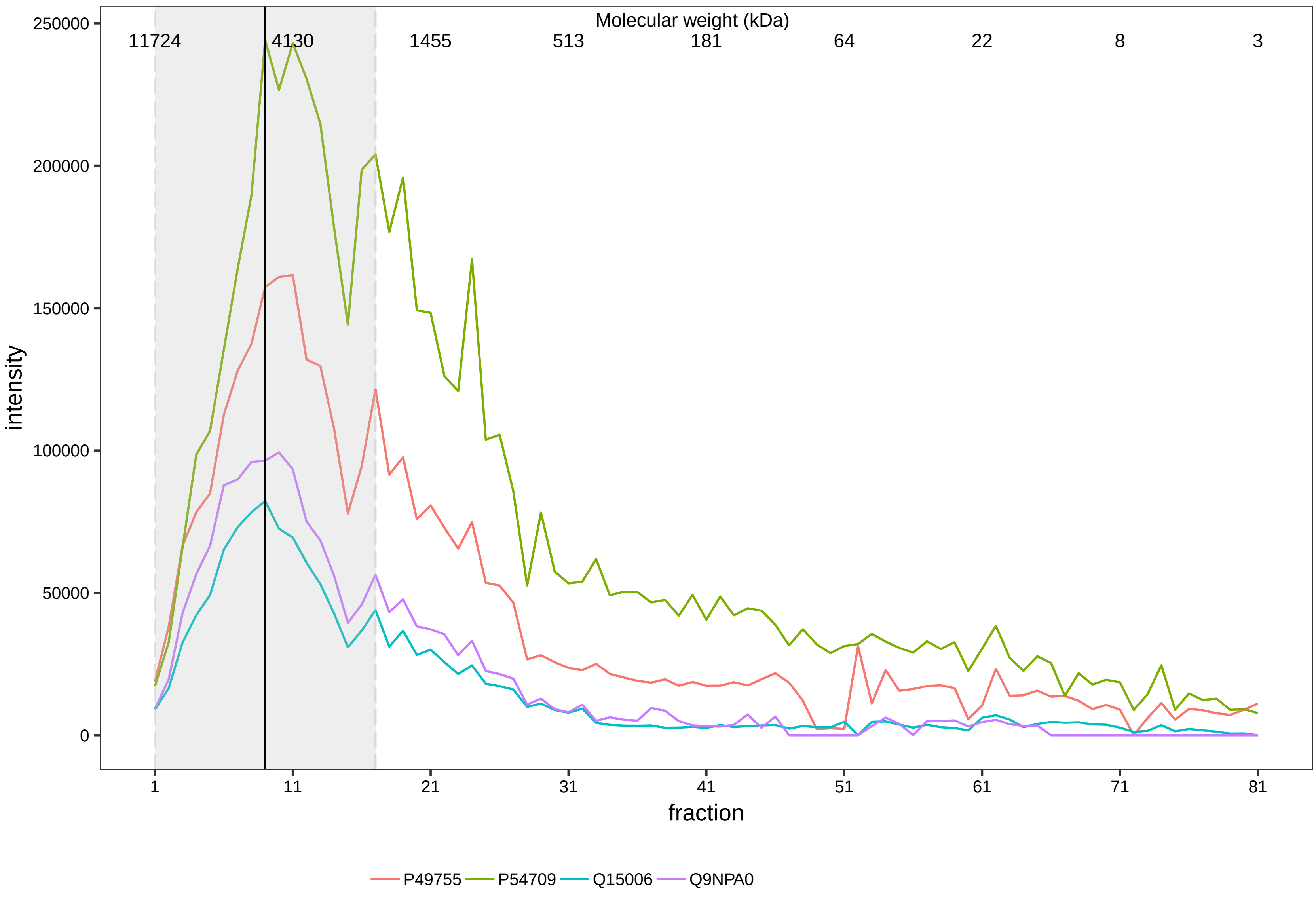

Feature ID 261

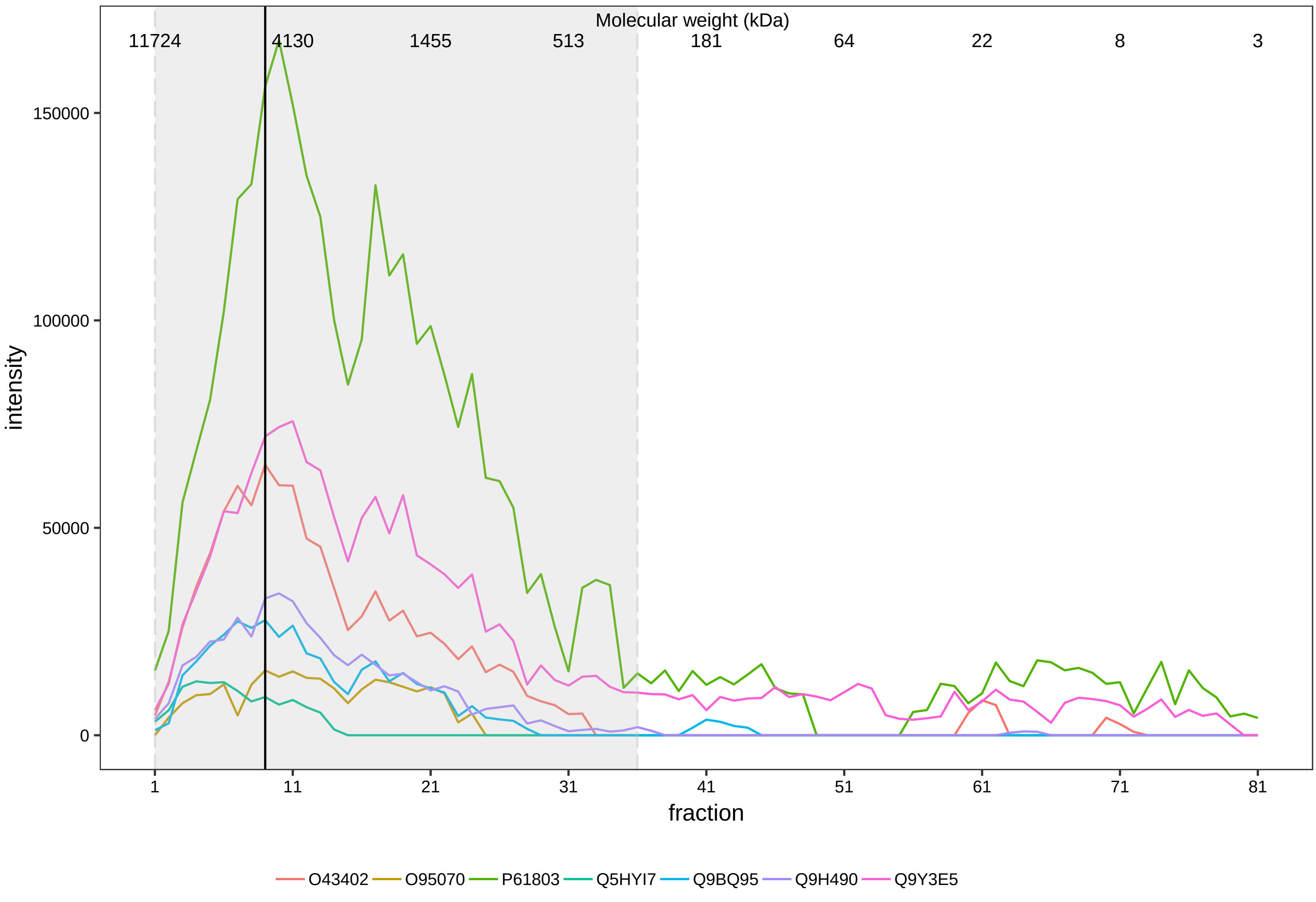

Feature ID 262

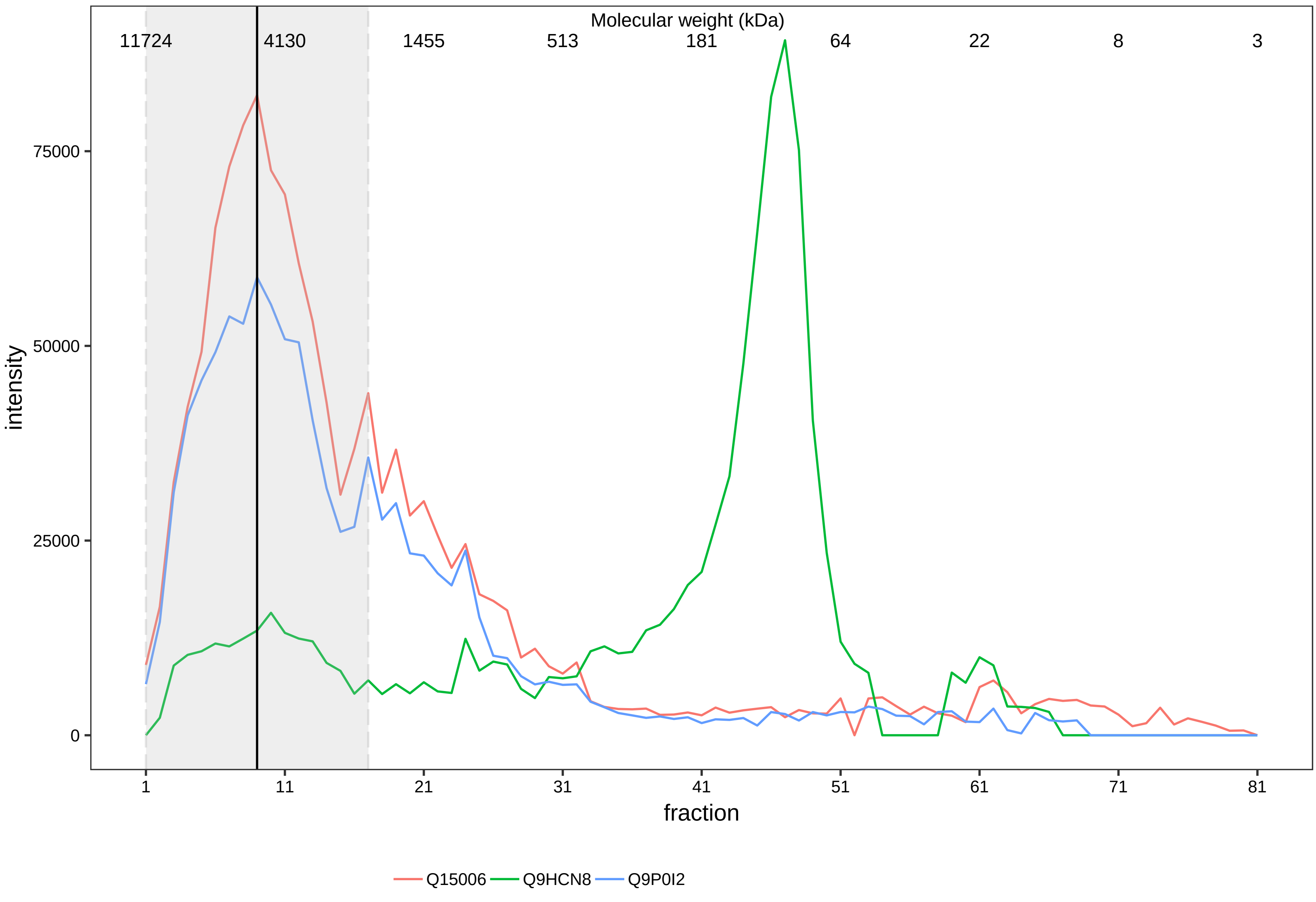

Feature ID 263

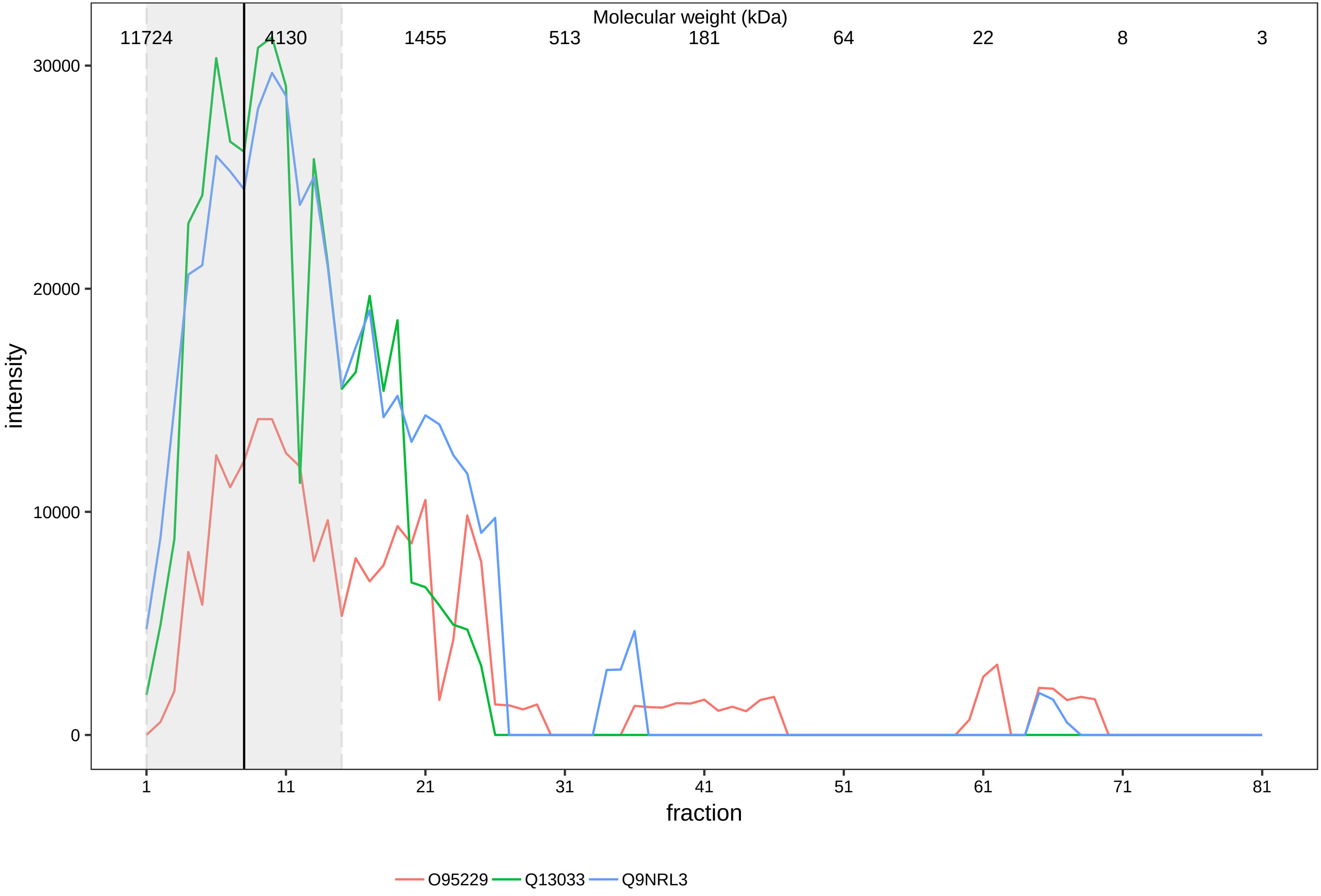

Feature ID 264

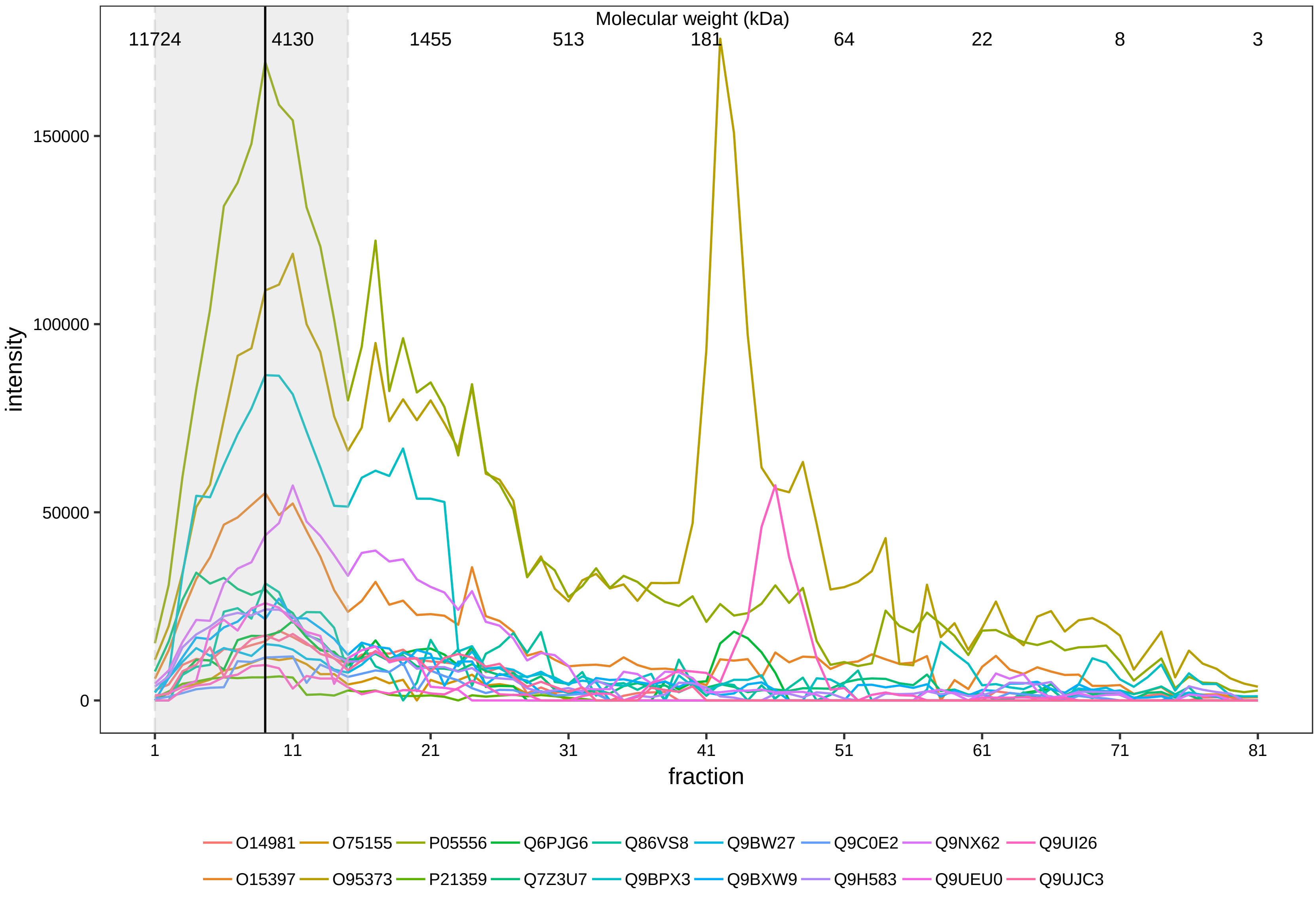

Feature ID 265

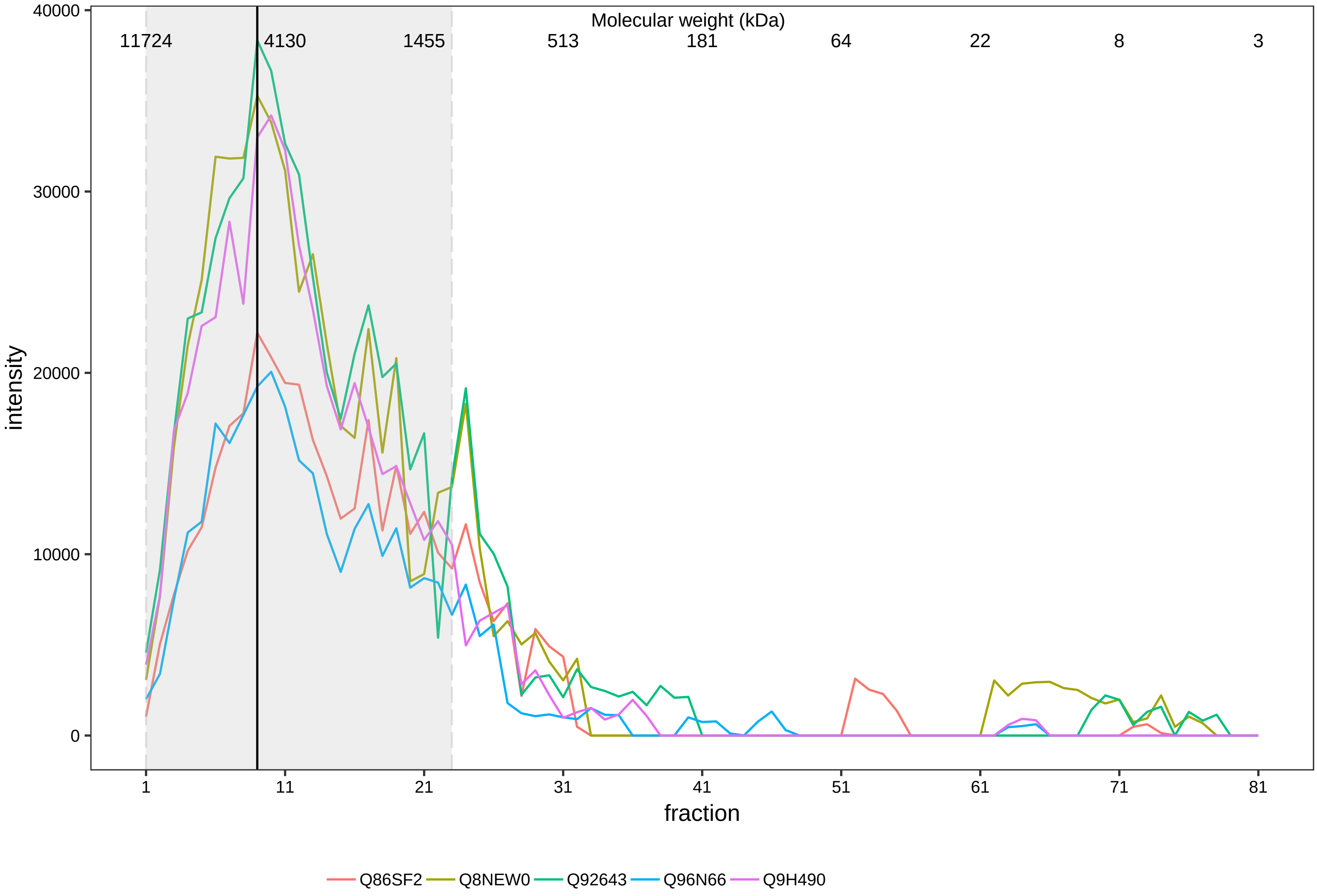

Feature ID 266

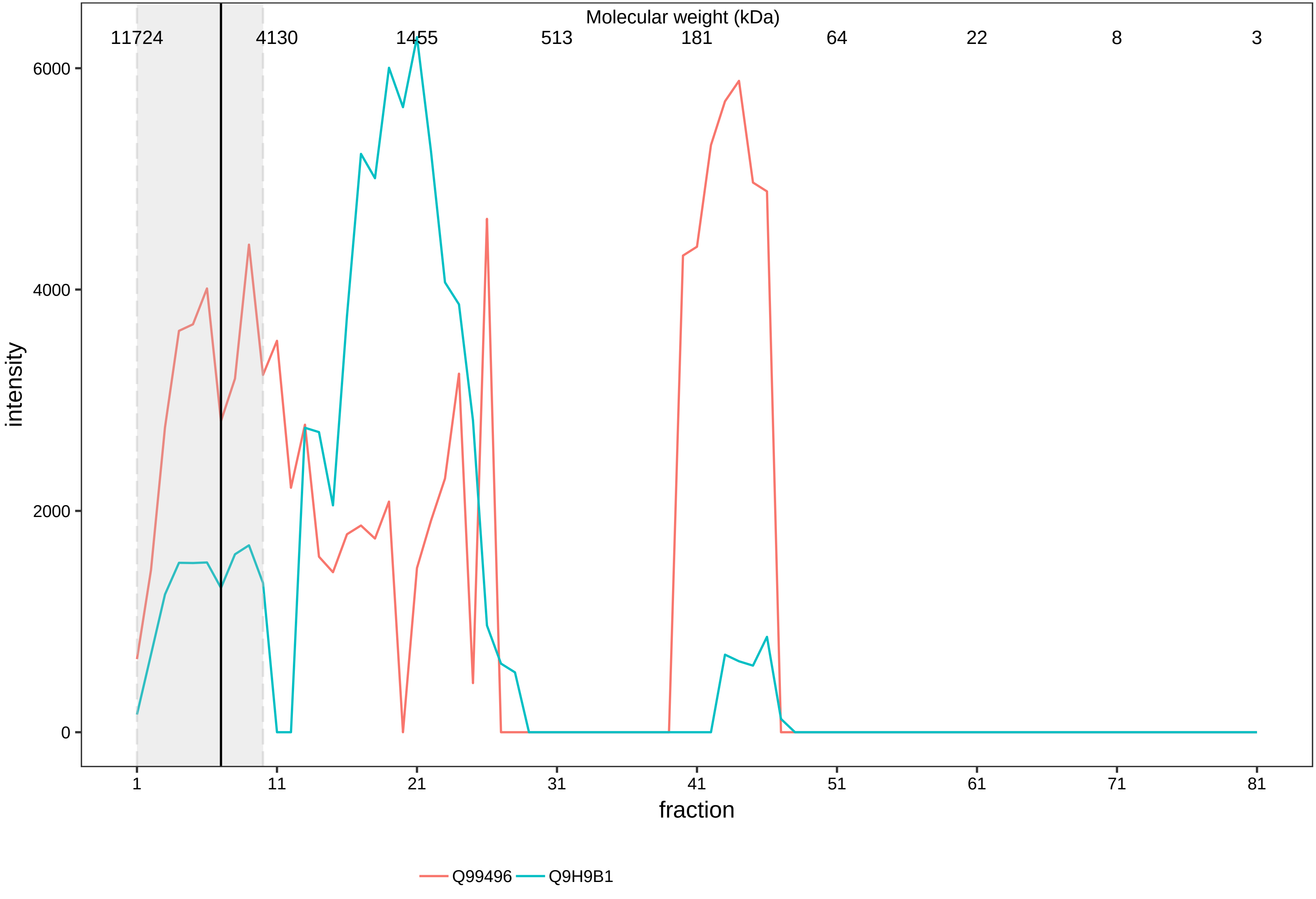

Feature ID 267

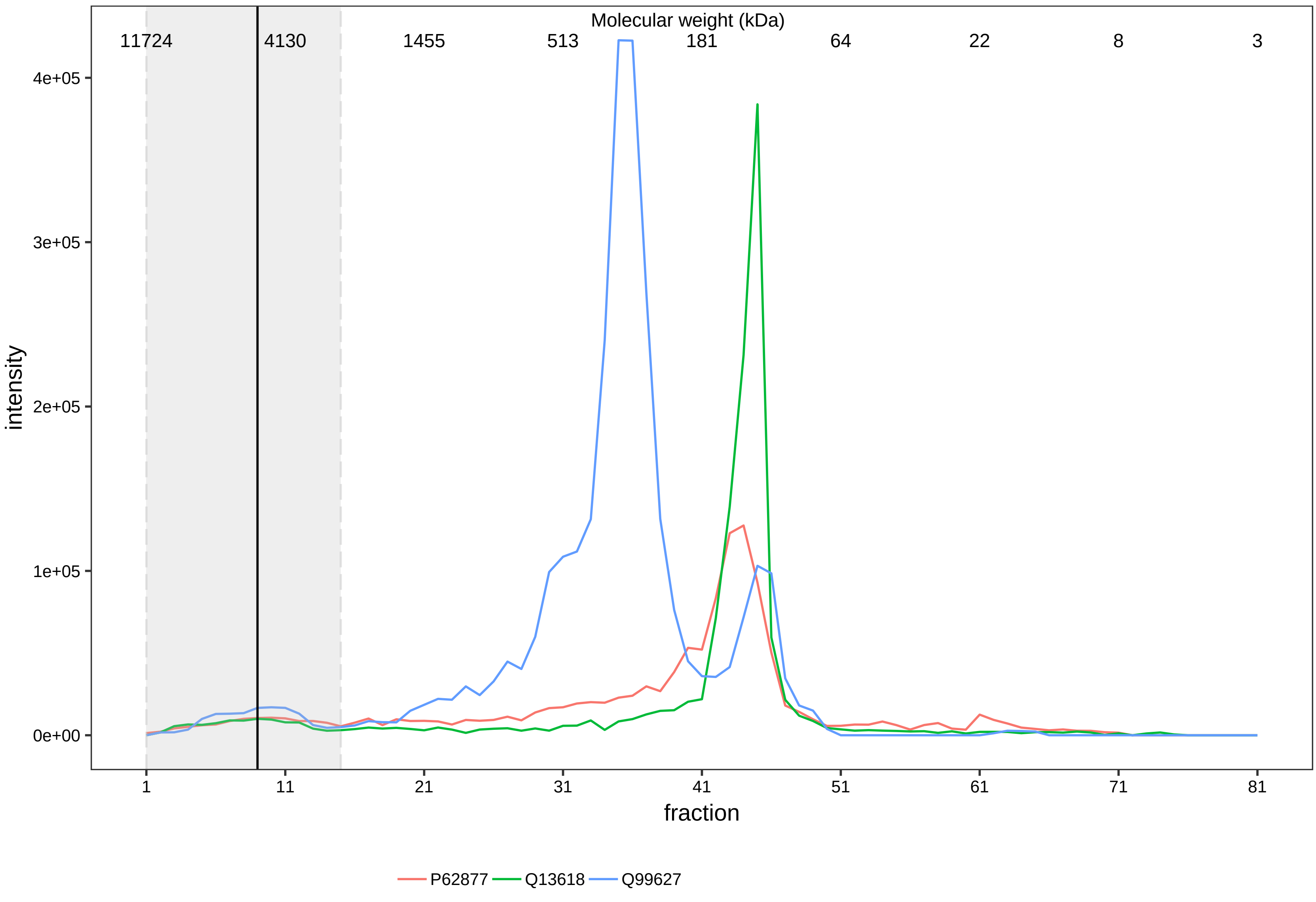

Feature ID 268

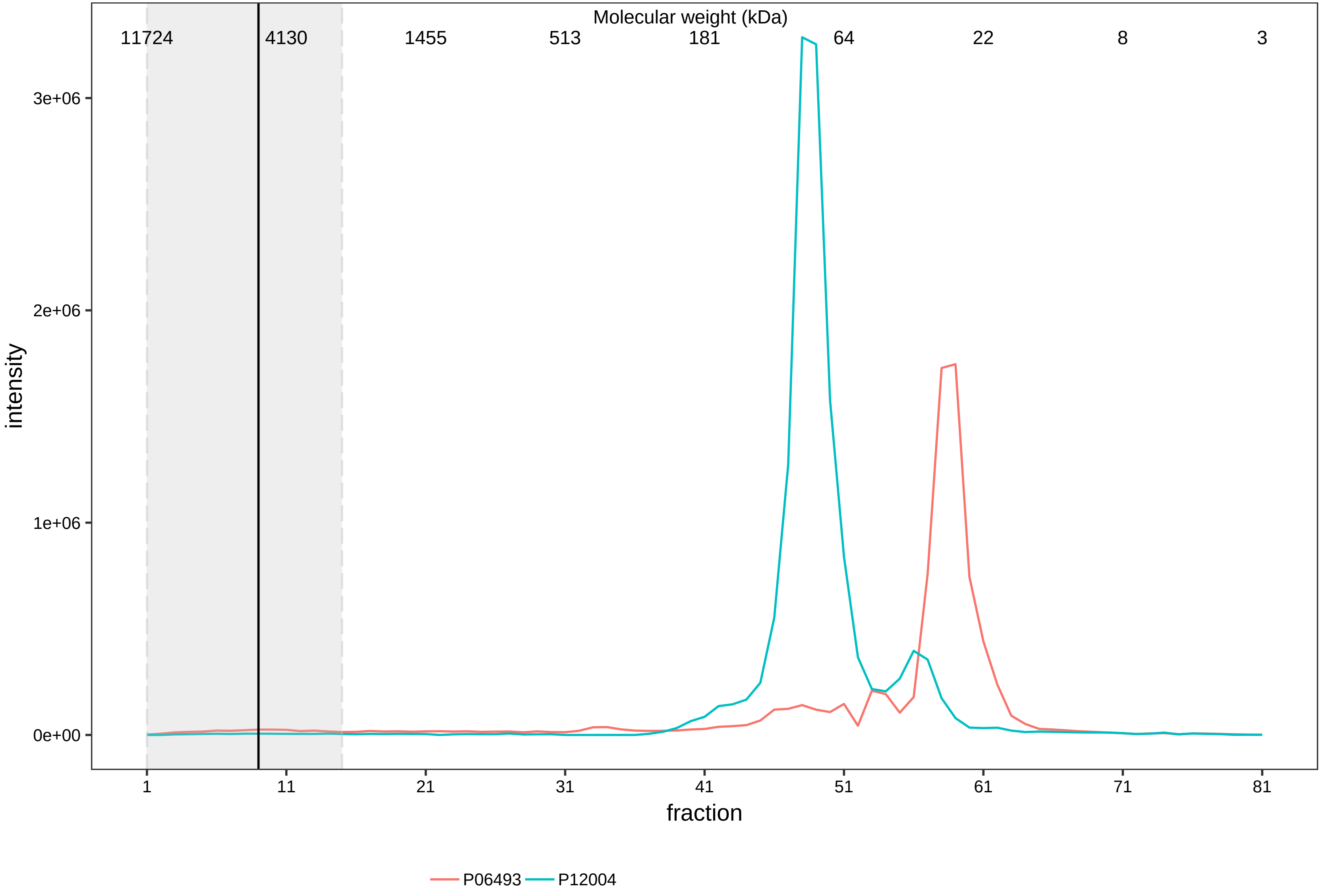

Feature ID 269

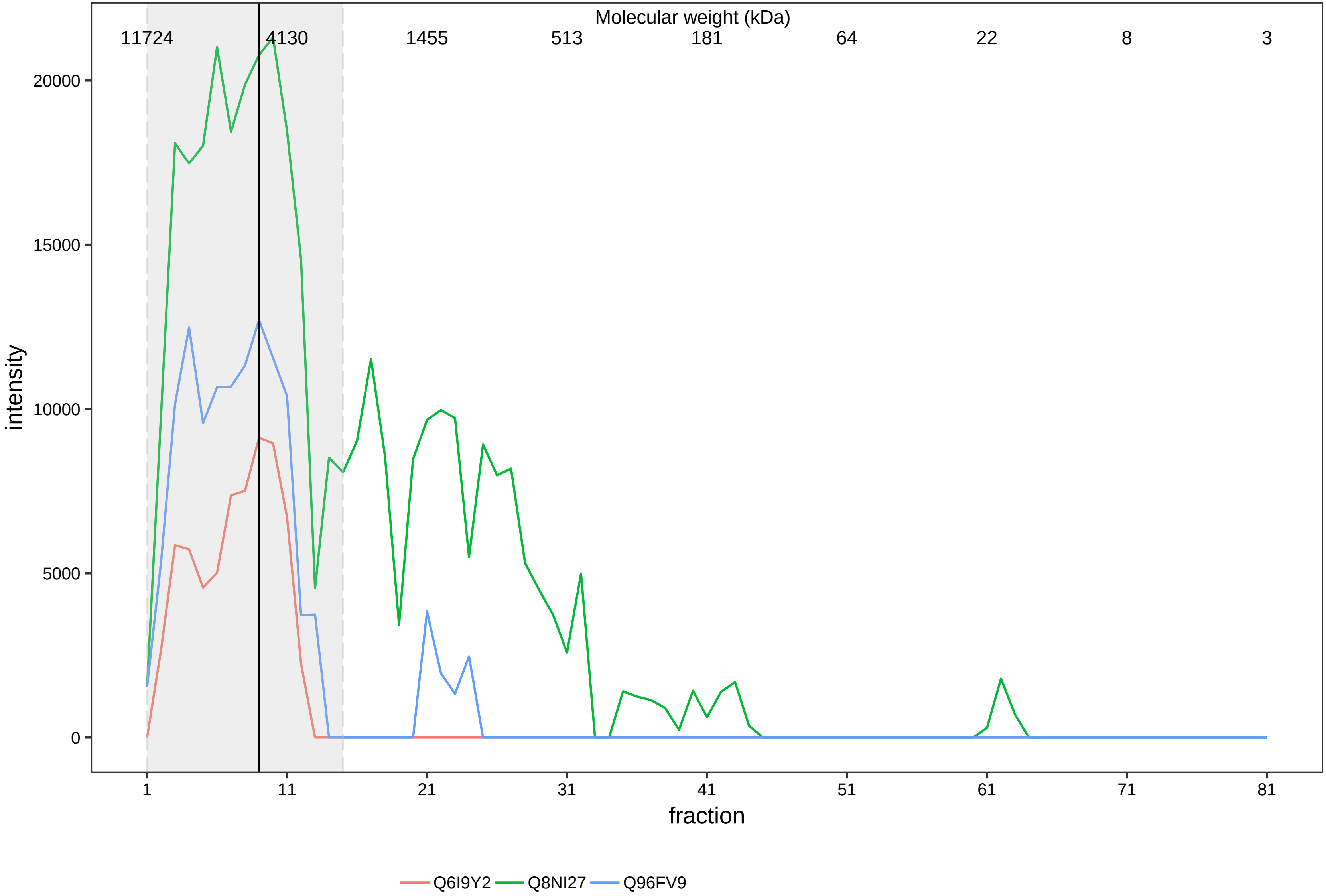

Feature ID 270

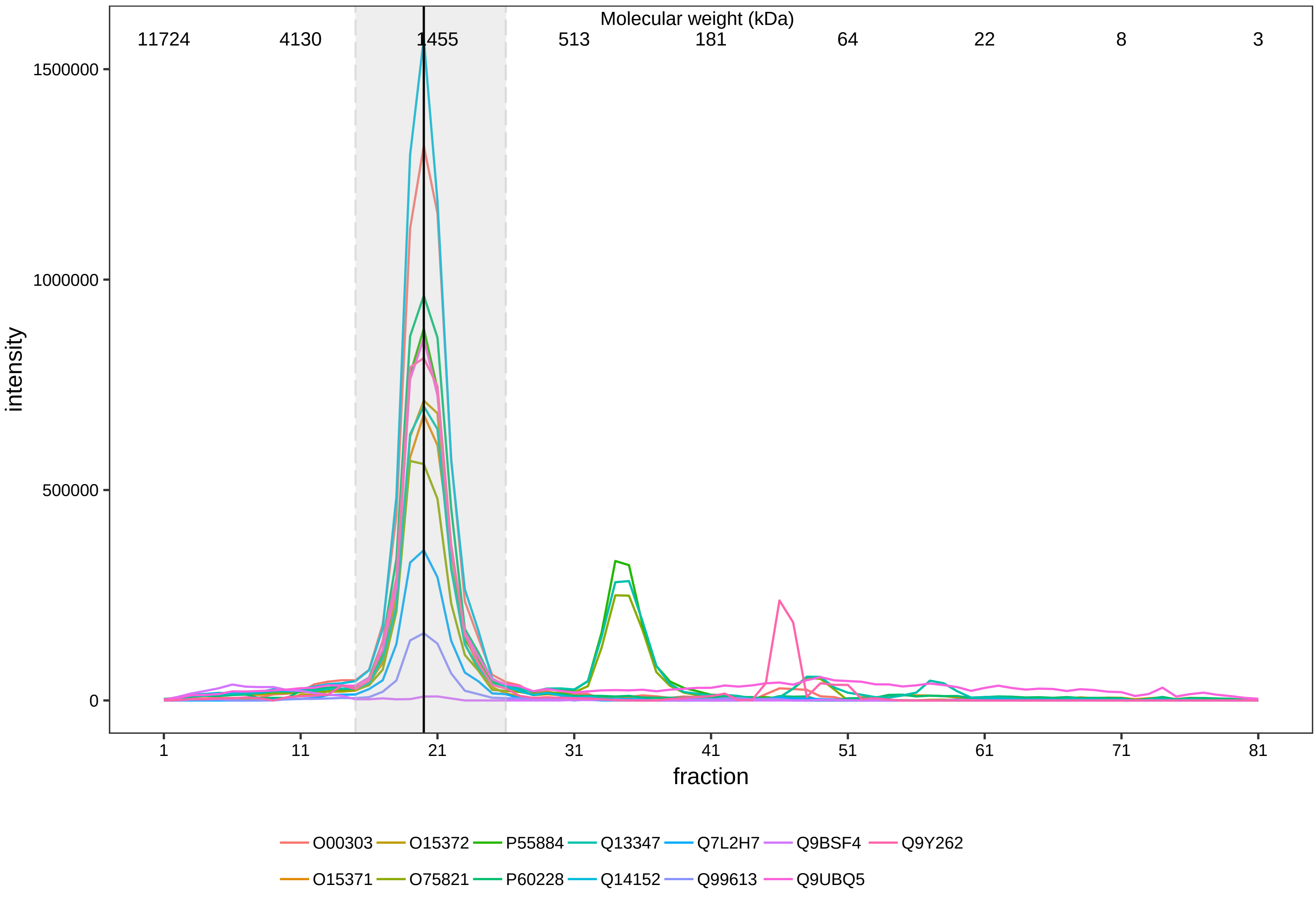

Feature ID 271

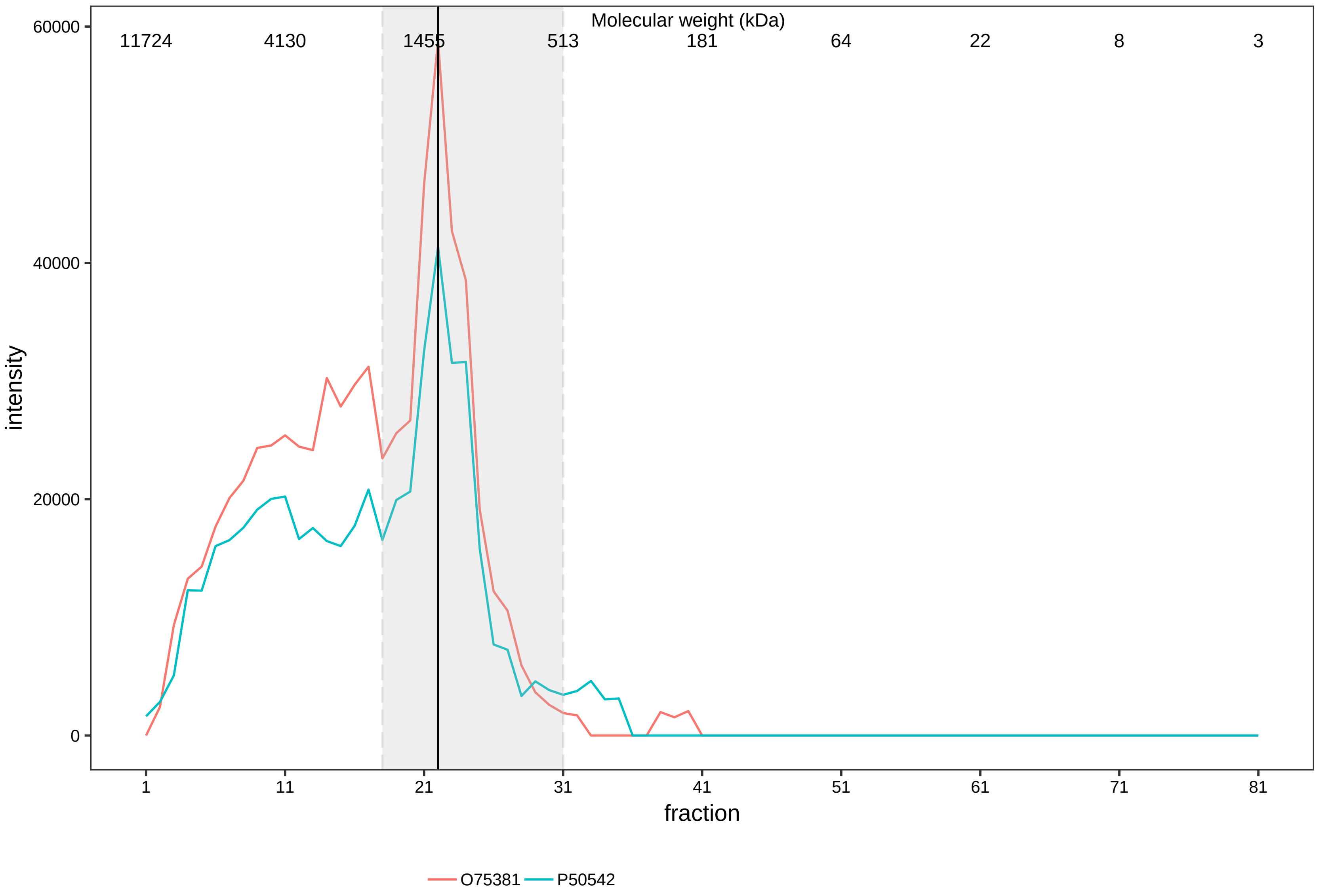

Feature ID 272

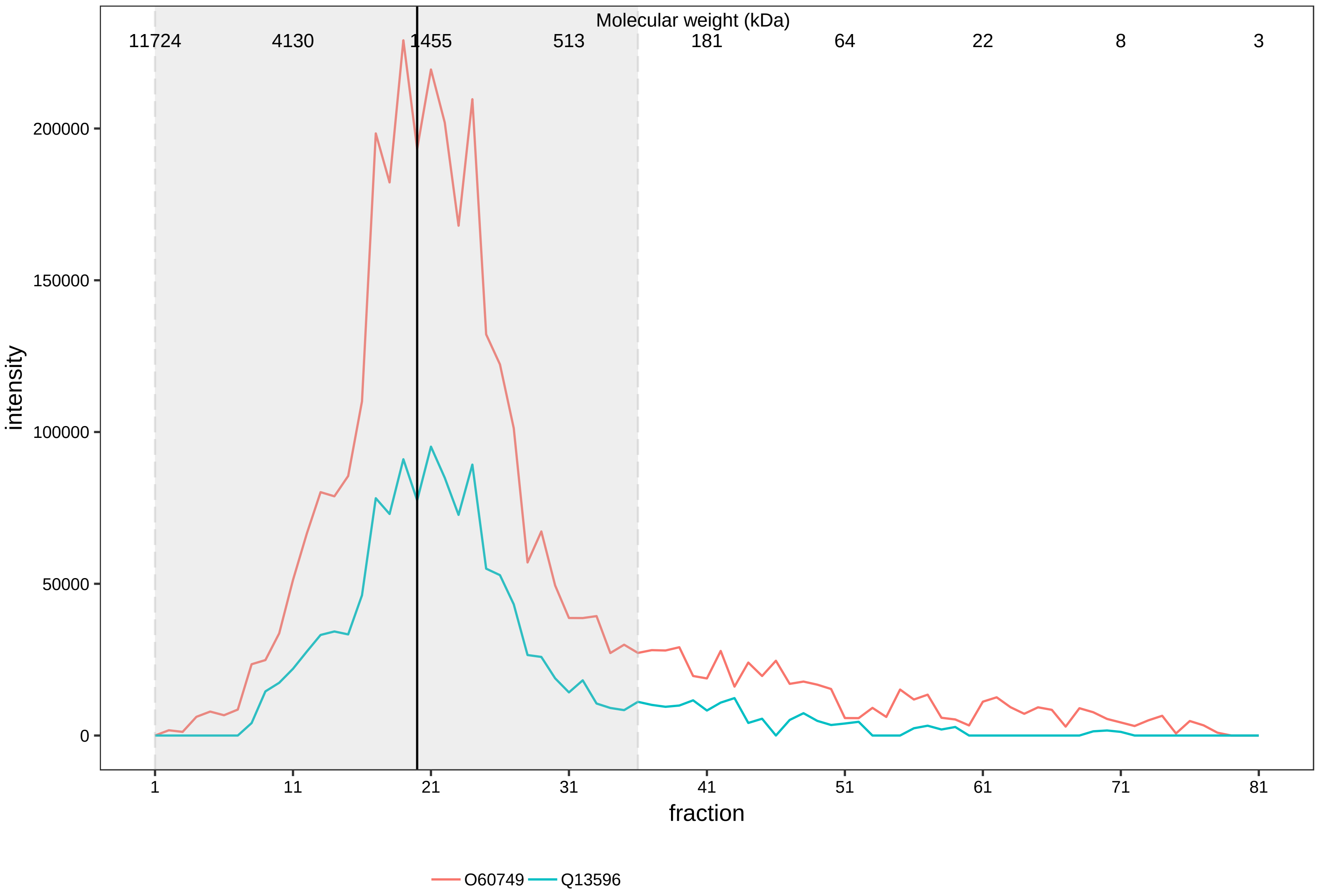

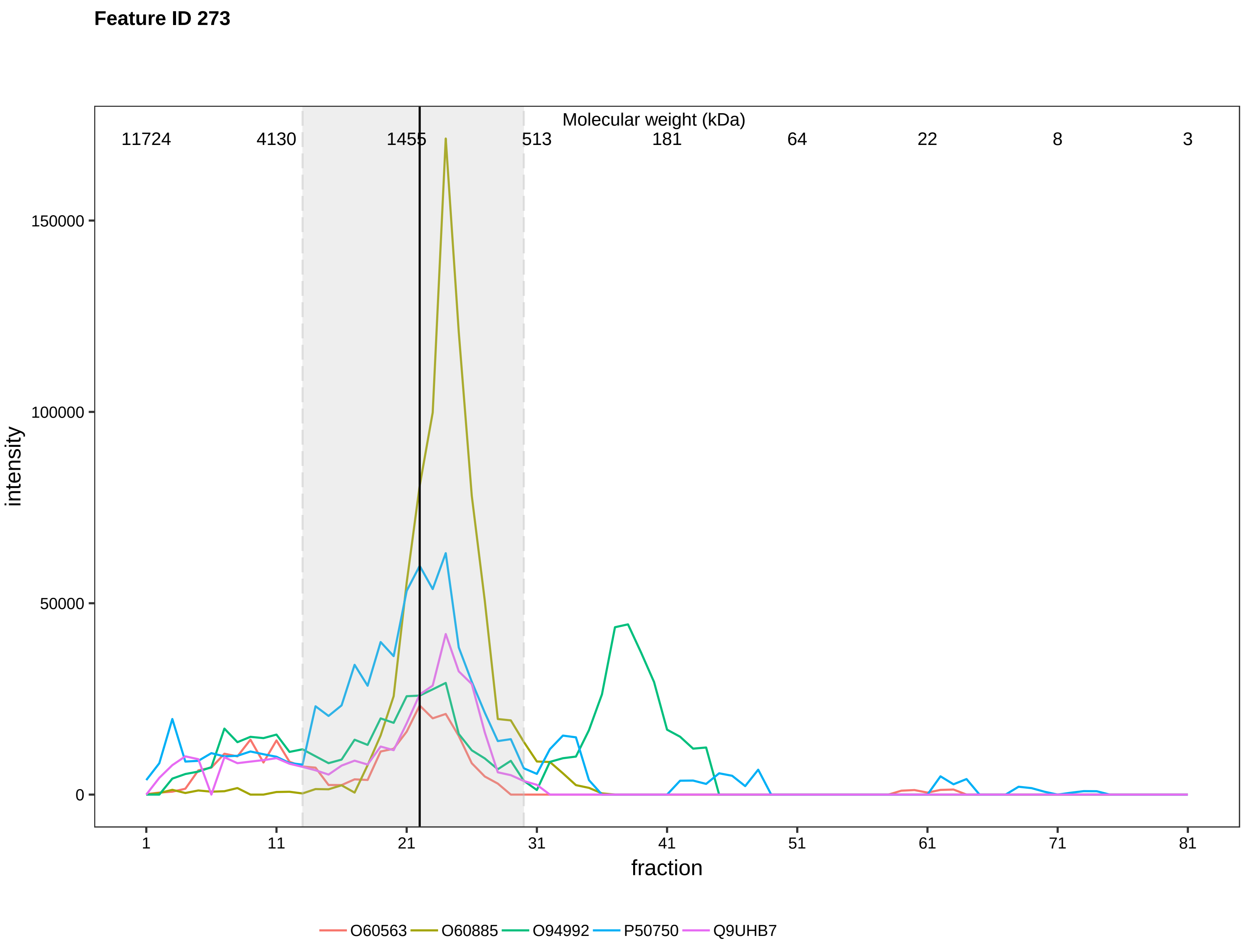
Feature ID 273
Molecular weight (kDa)
11724
4130
1455
513
181
64
22
8
3
150000
100000
50000
0
intensity
1
11
21
31
41
51
61
71
81
fraction
O60563
O60885
O94992
P50750
Q9UHB7

Feature ID 274

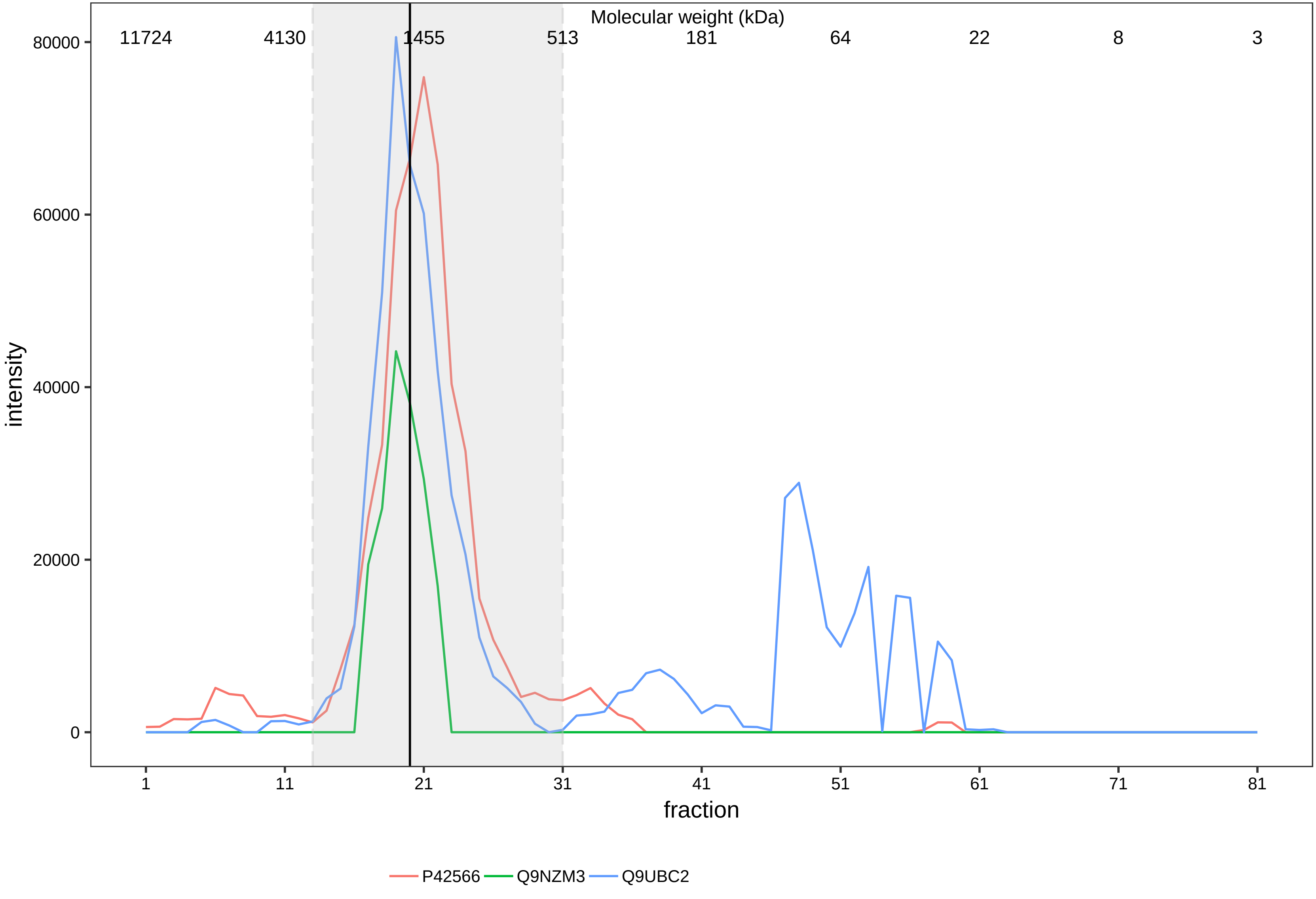

Feature ID 275

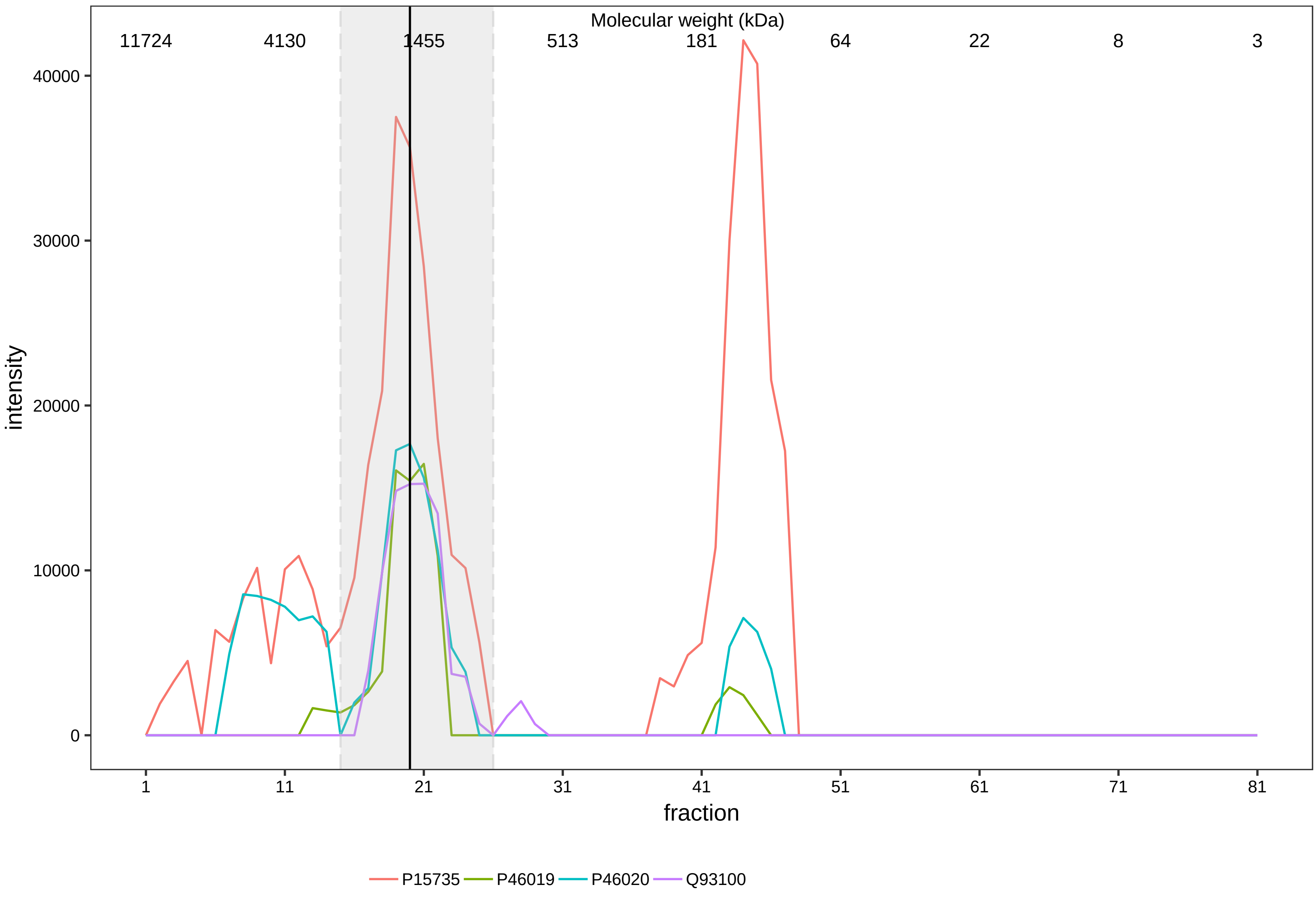

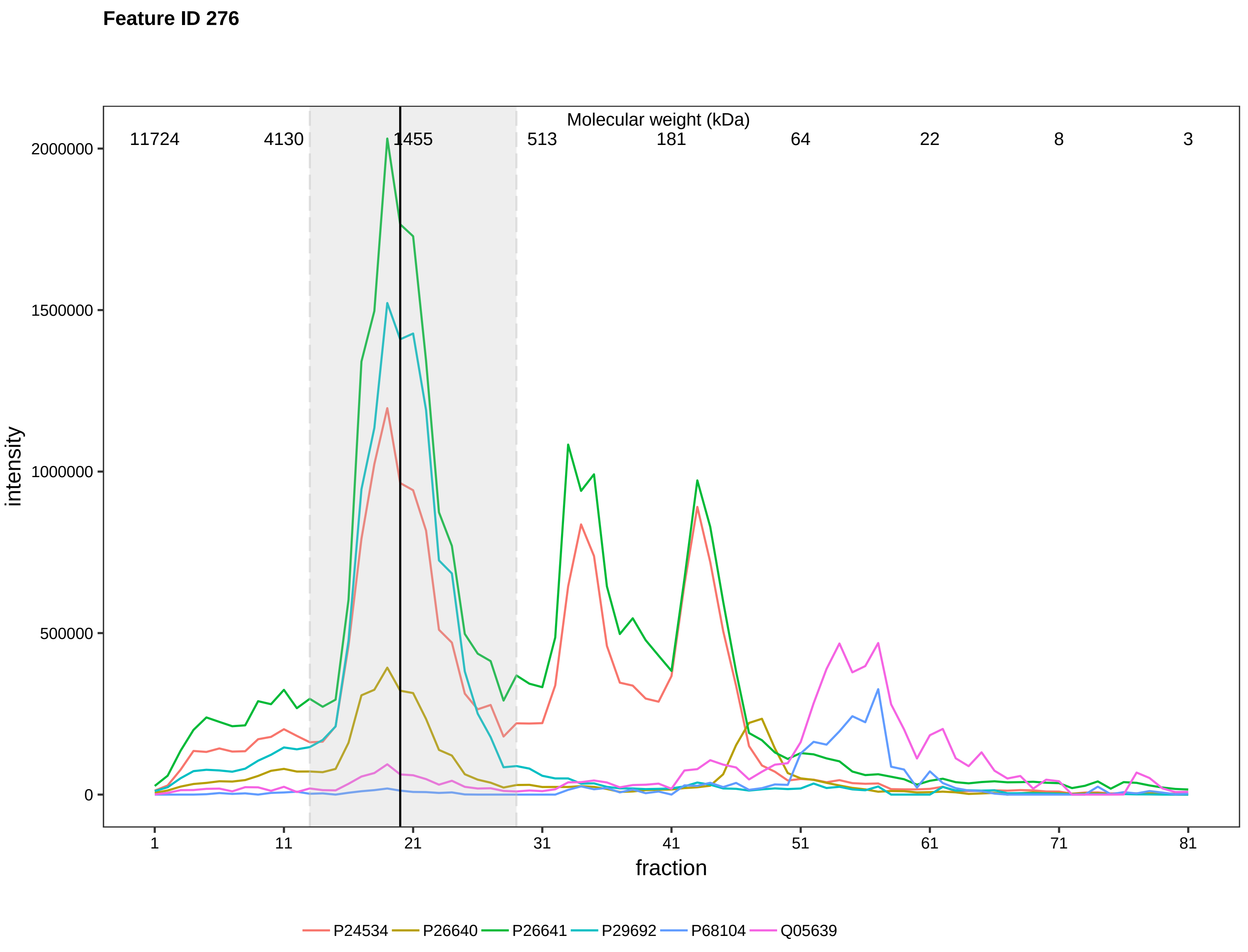
Feature ID 276
Molecular weight (kDa)
11724
4130
1455
513
181
64
22
8
3
2000000
1500000
1000000
500000
0
intensity
1
11
21
31
41
51
61
71
81
fraction
P24534
P26640
P26641
P29692
P68104
Q05639

Feature ID 277

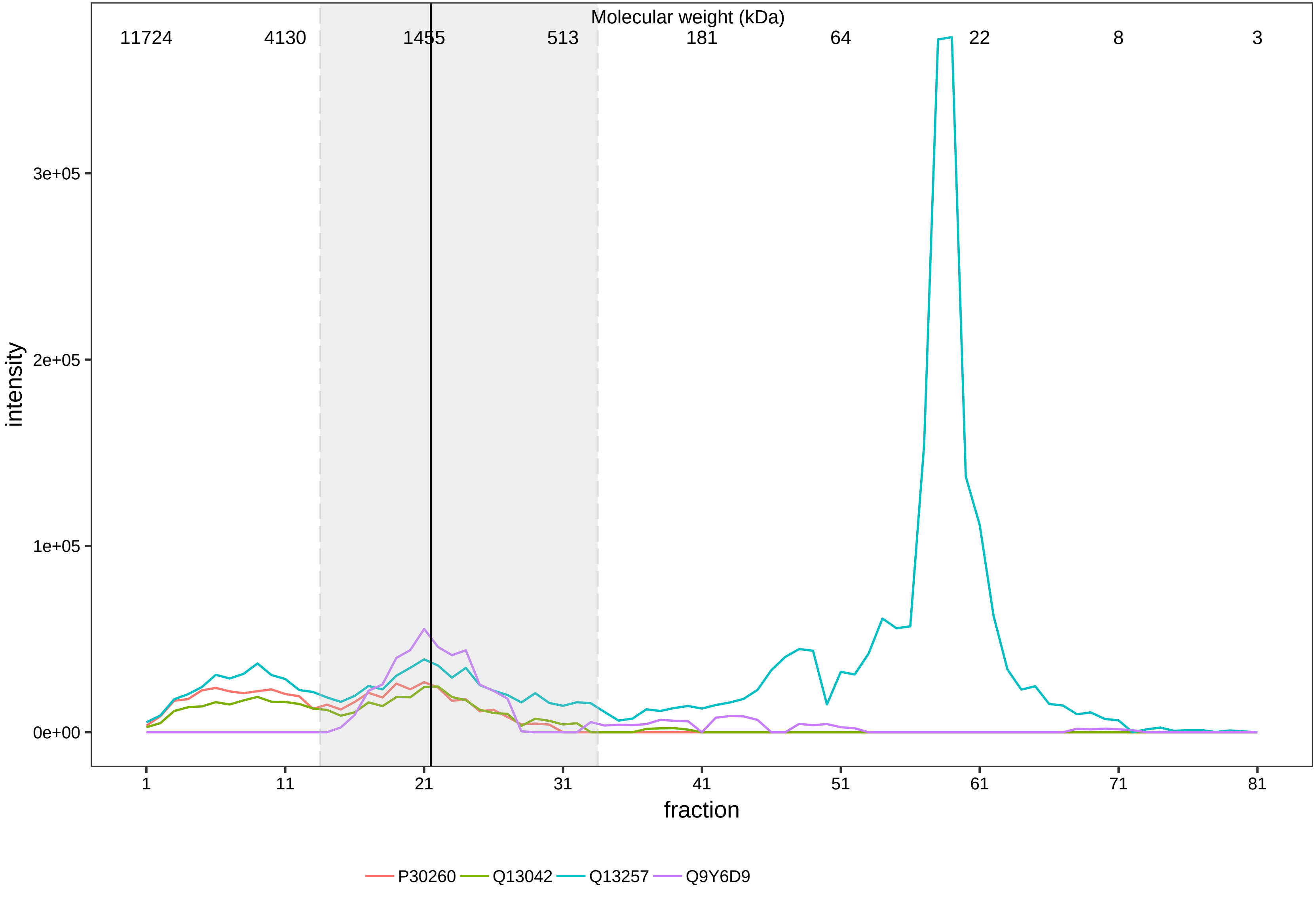

Feature ID 278

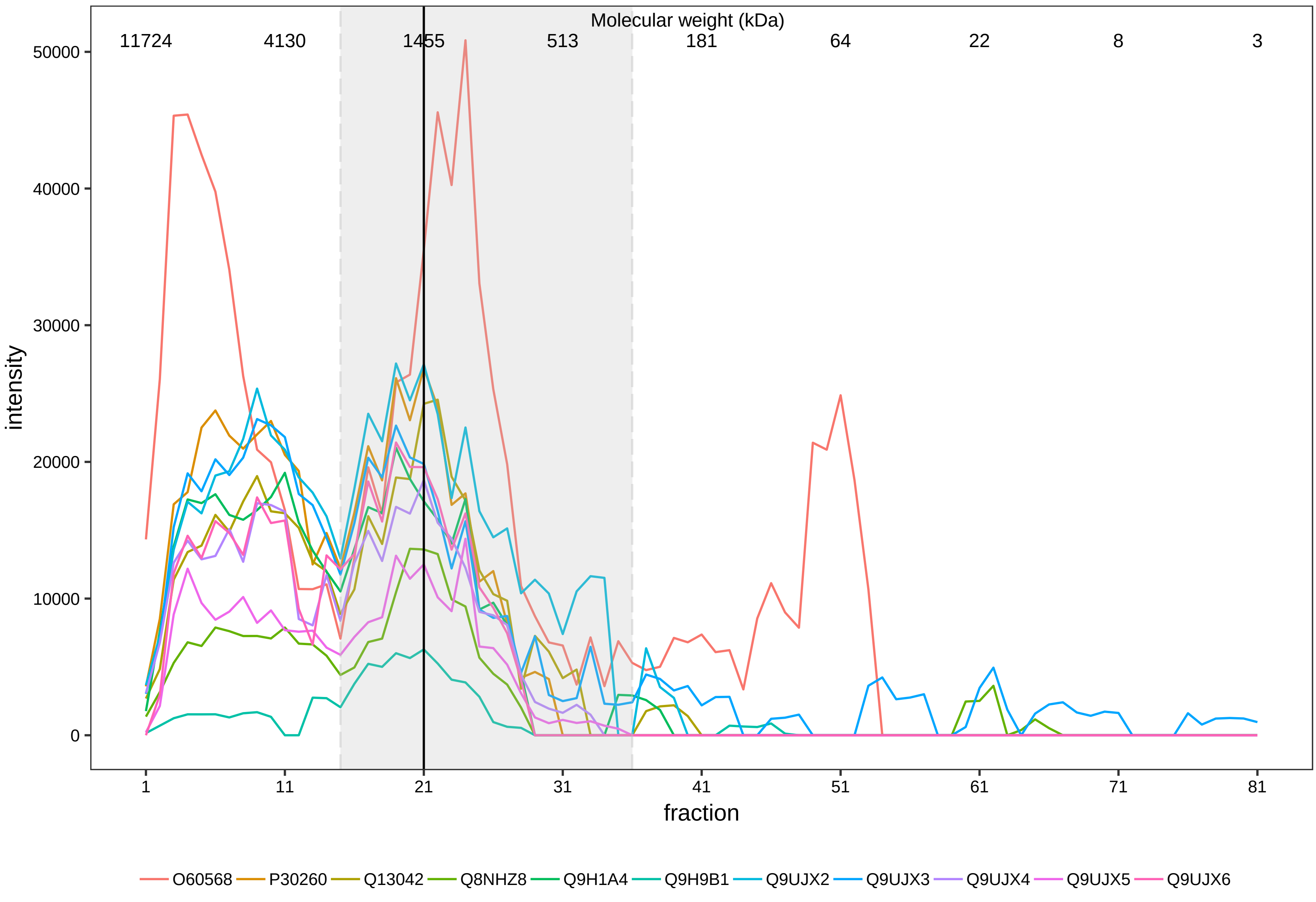

Feature ID 279

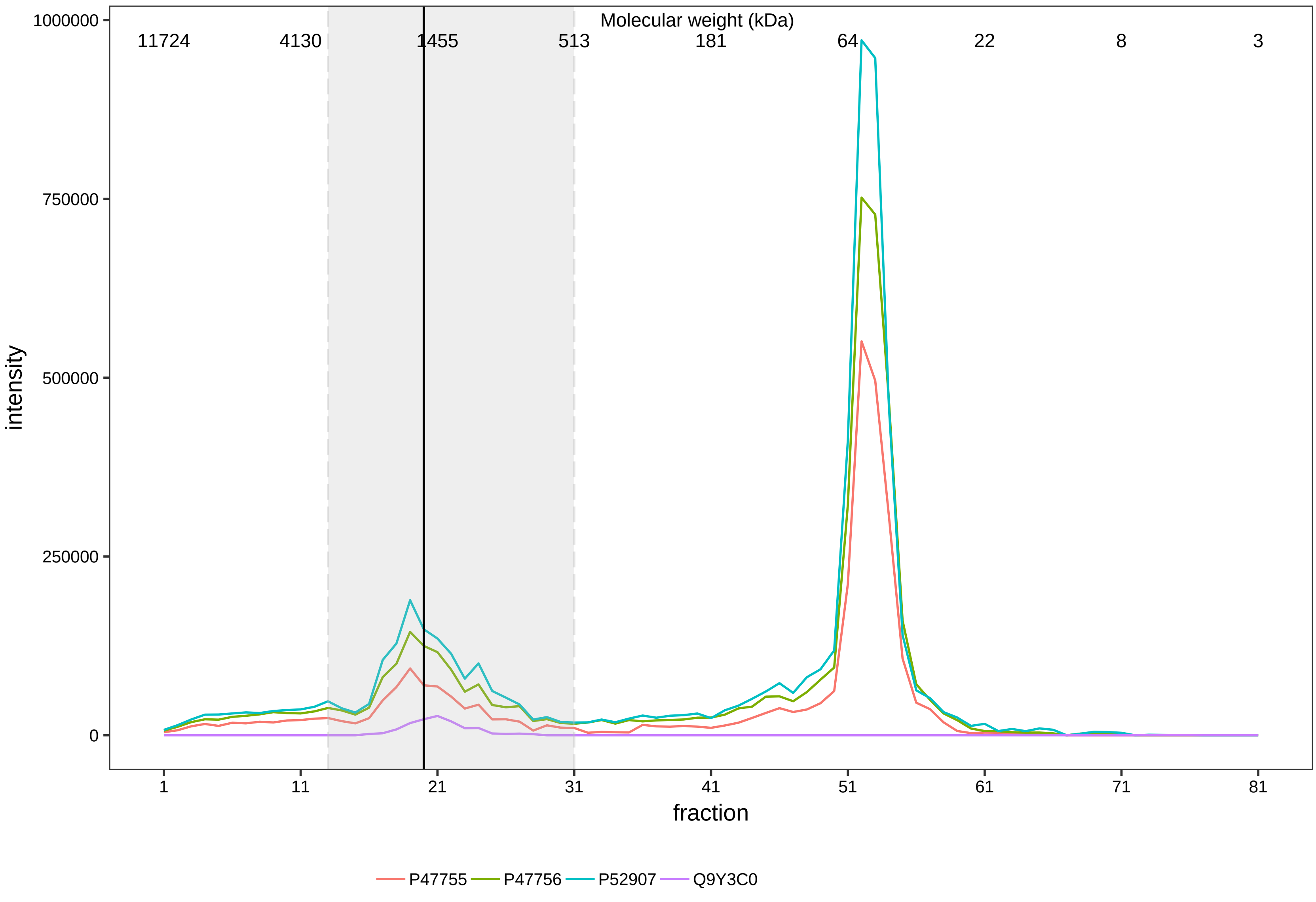

Feature ID 280

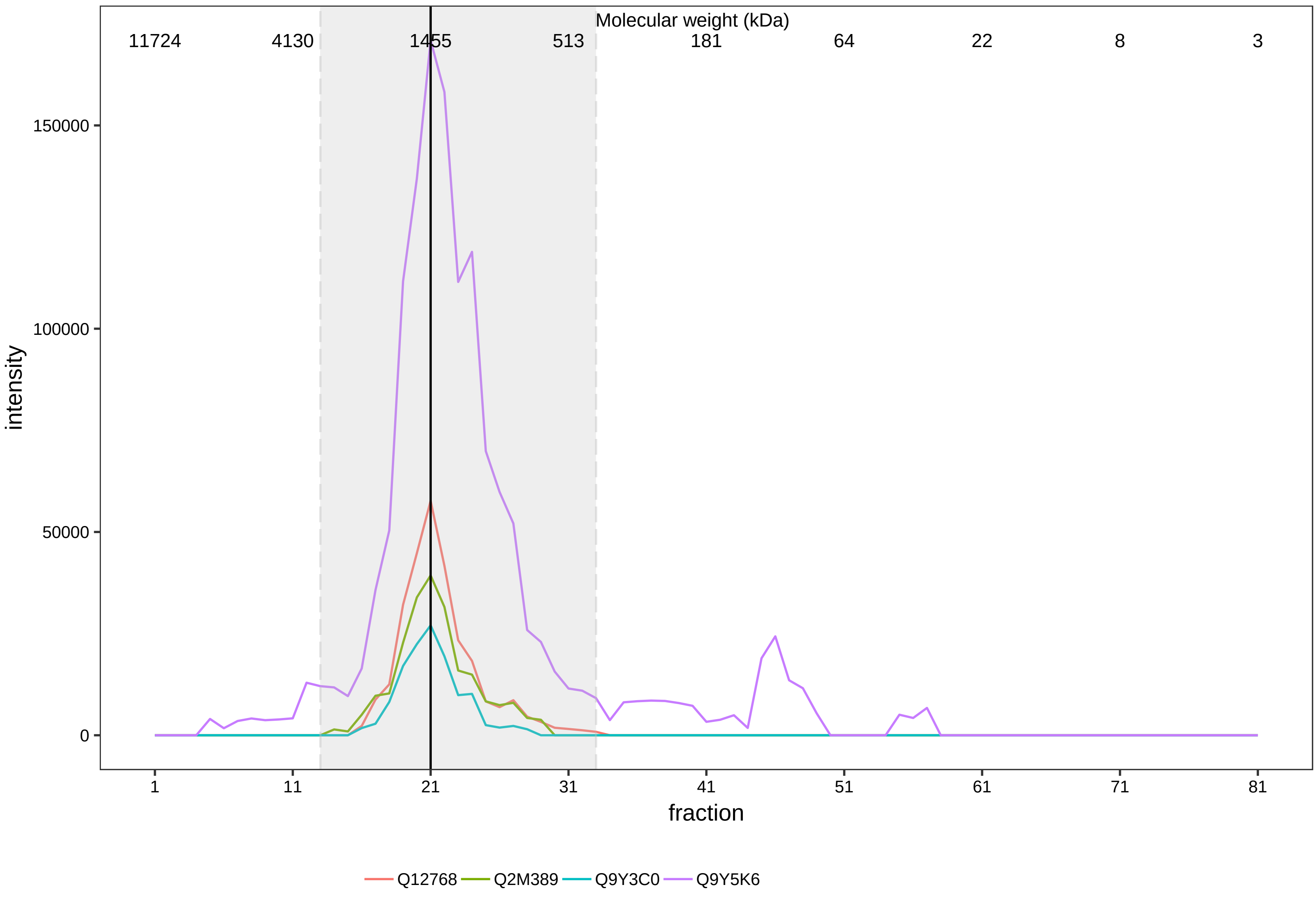

Feature ID 281

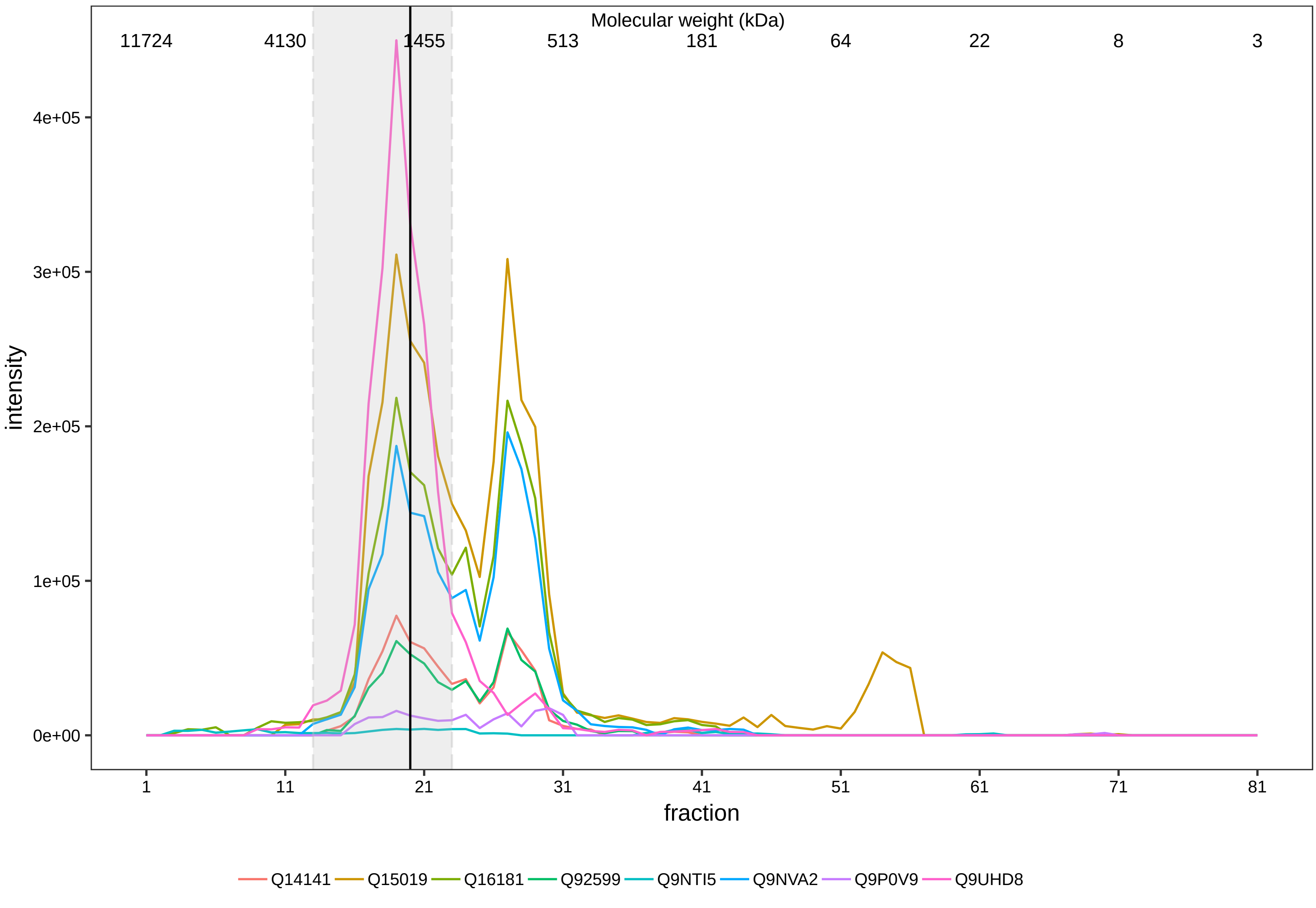

Feature ID 282

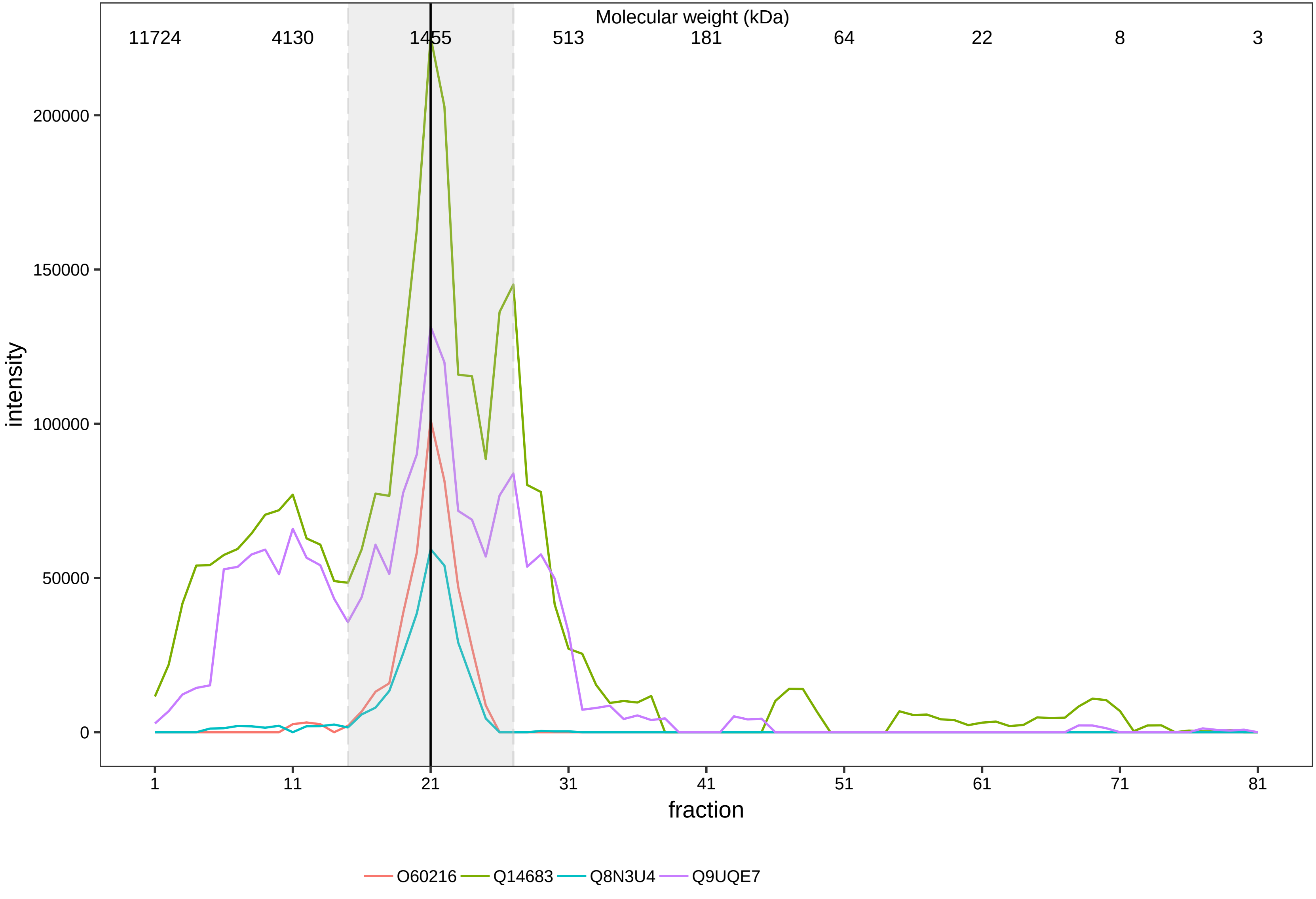

Feature ID 283

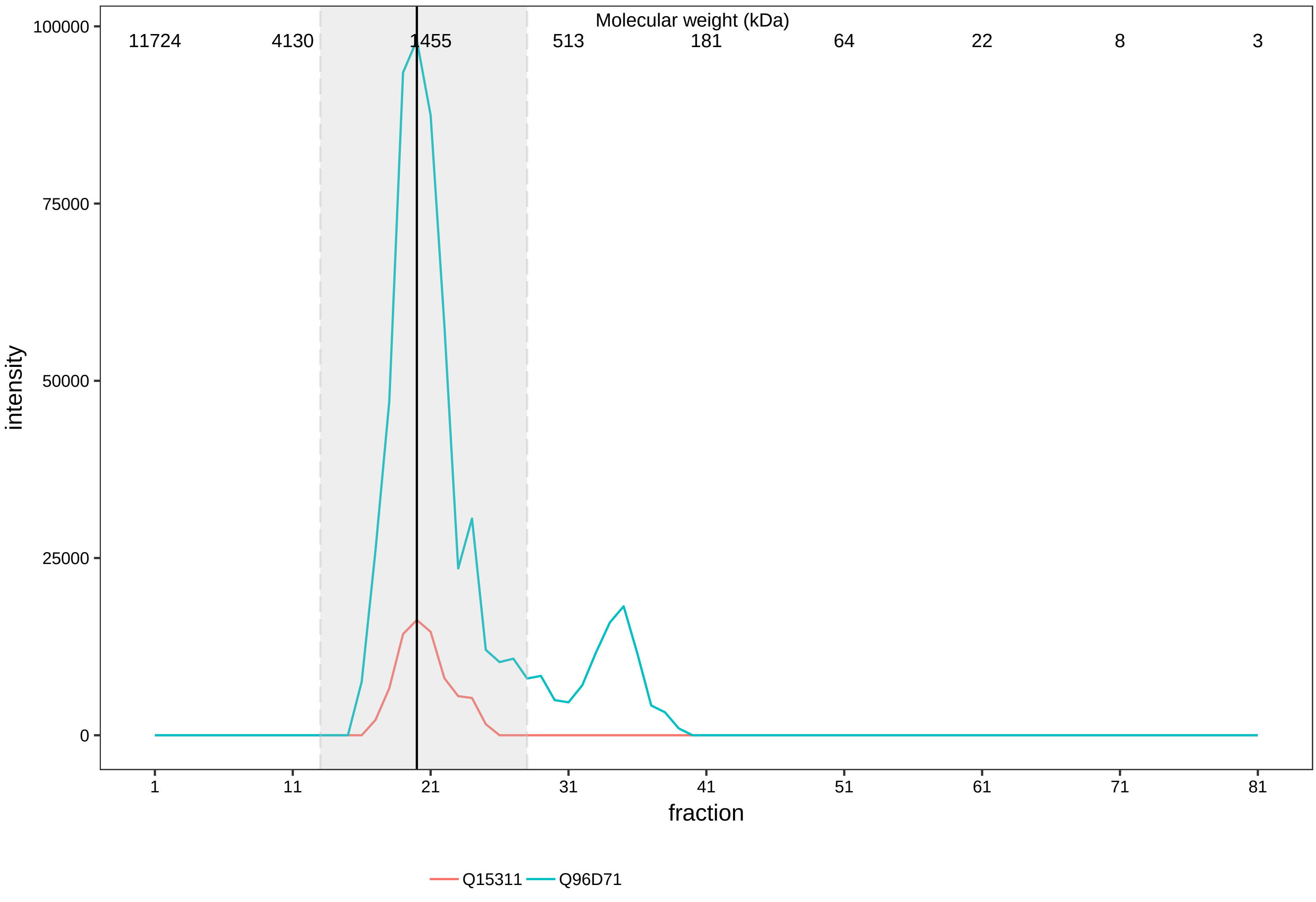

Feature ID 284

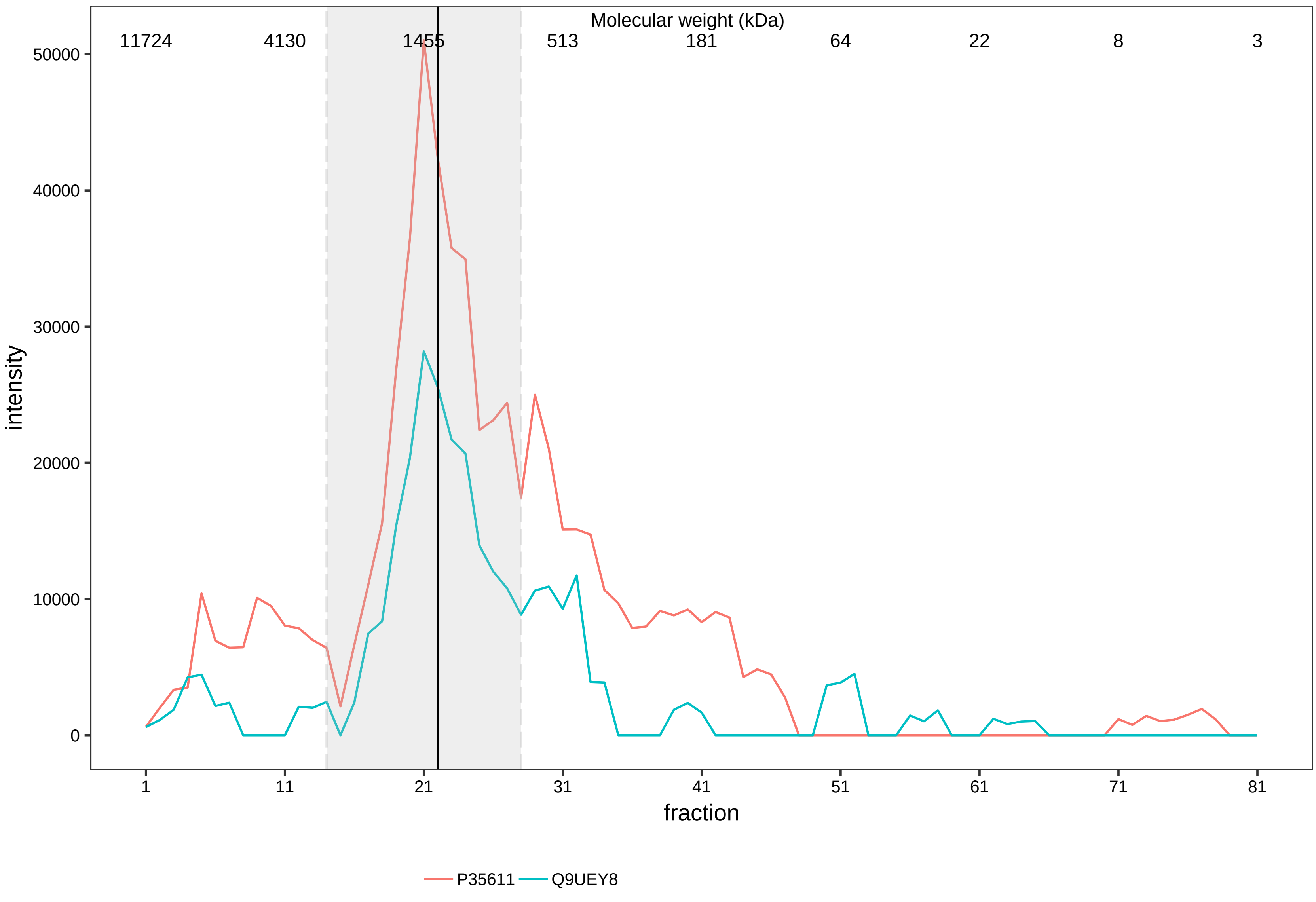

Feature ID 285

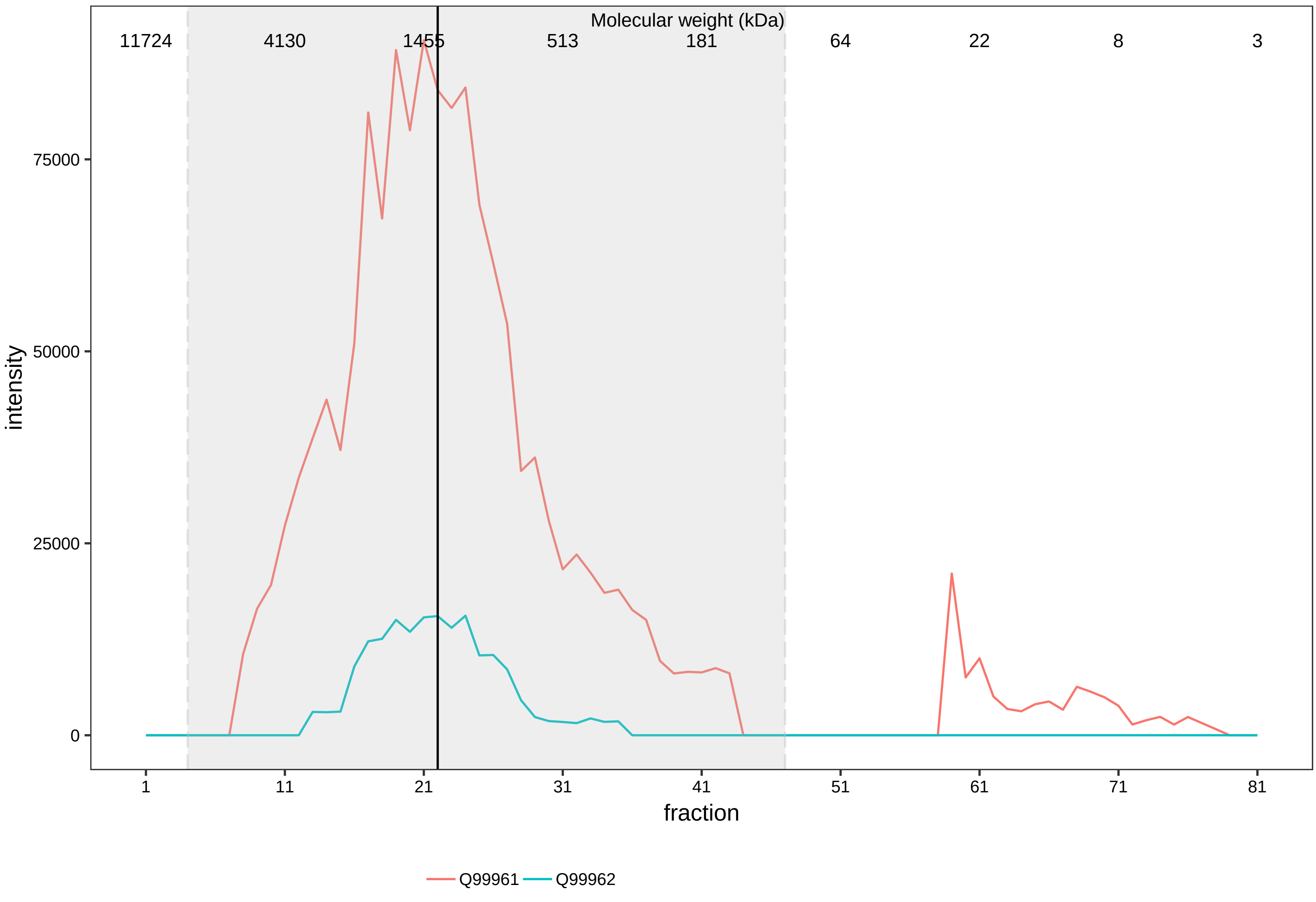

Feature ID 286

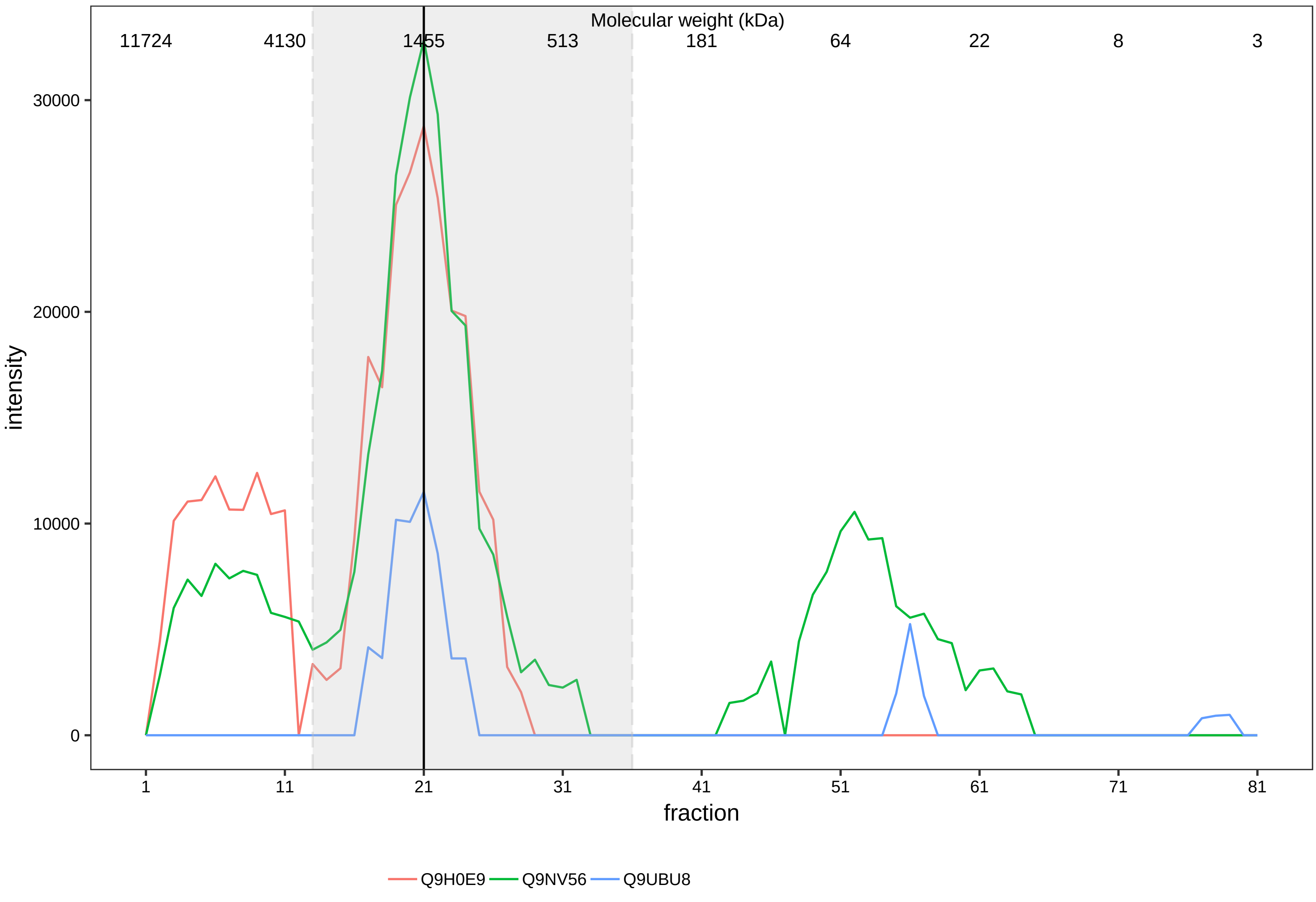

Feature ID 287

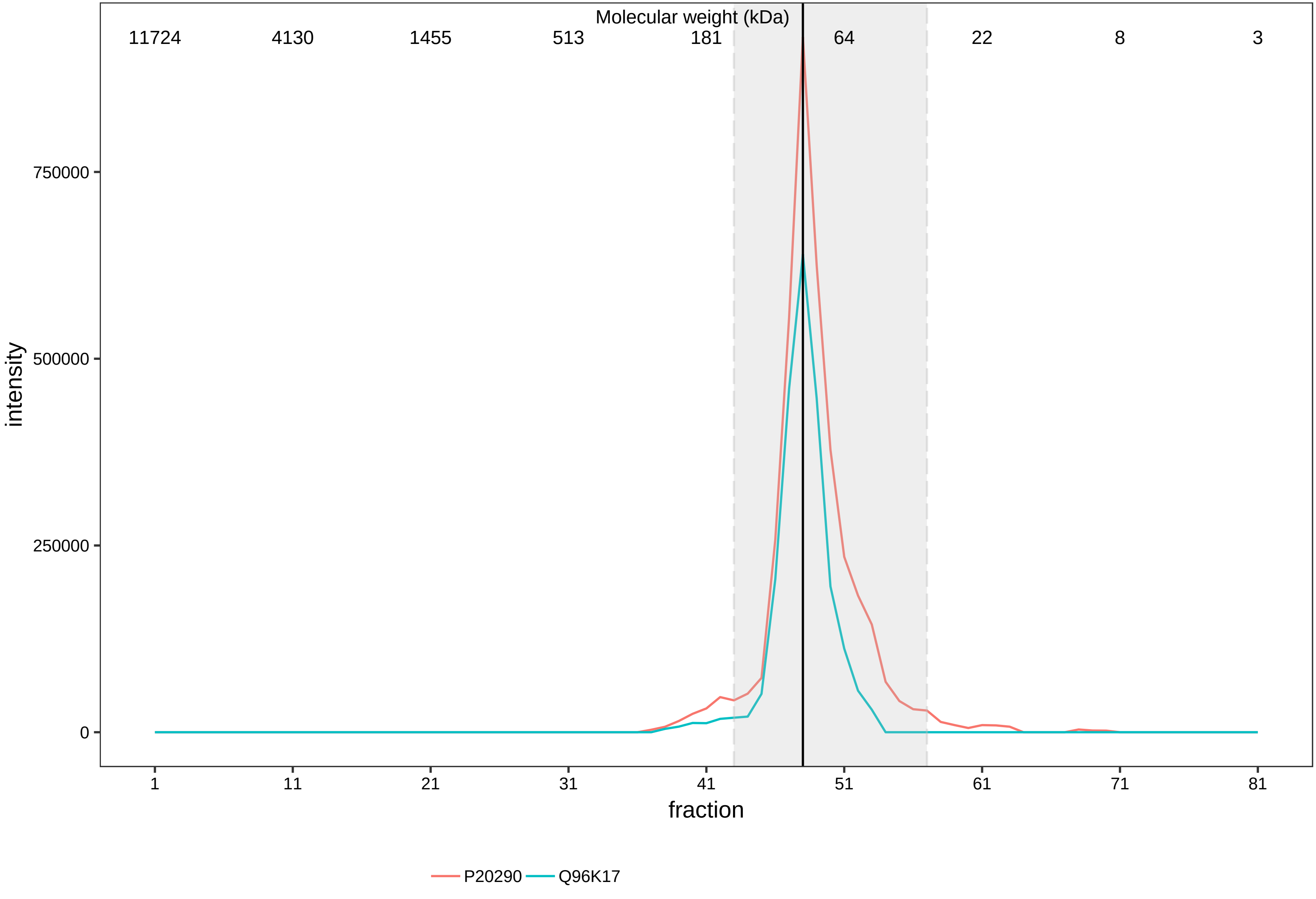

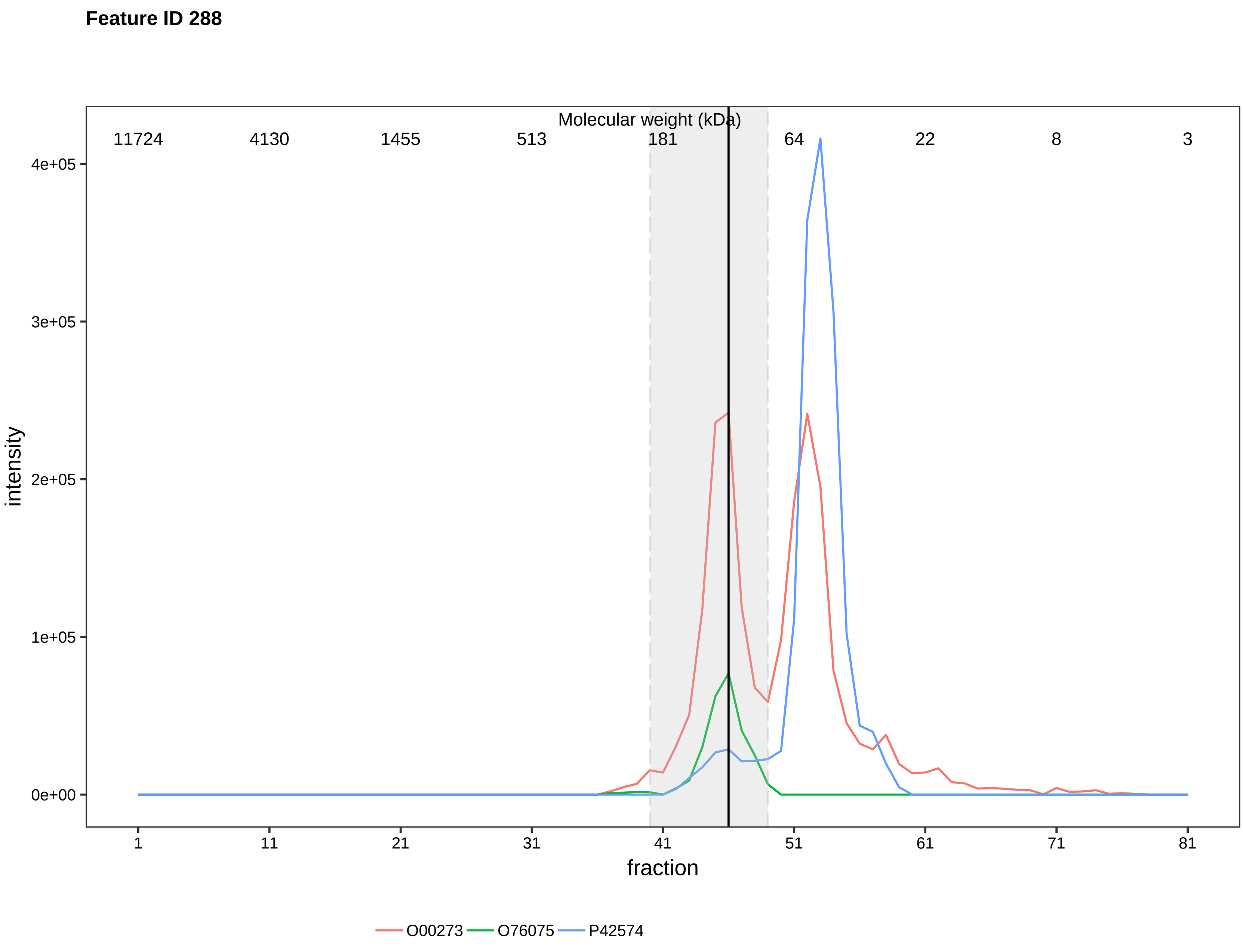

Feature ID 288
Molecular weight (kDa)
11724
4130
1455
513
181
64
22
8
3
4e+05
3e+05
2e+05
1e+05
0e+00
intensity
1
11
21
31
41
51
61
71
81
fraction
O00273
O76075
P42574

Feature ID 289

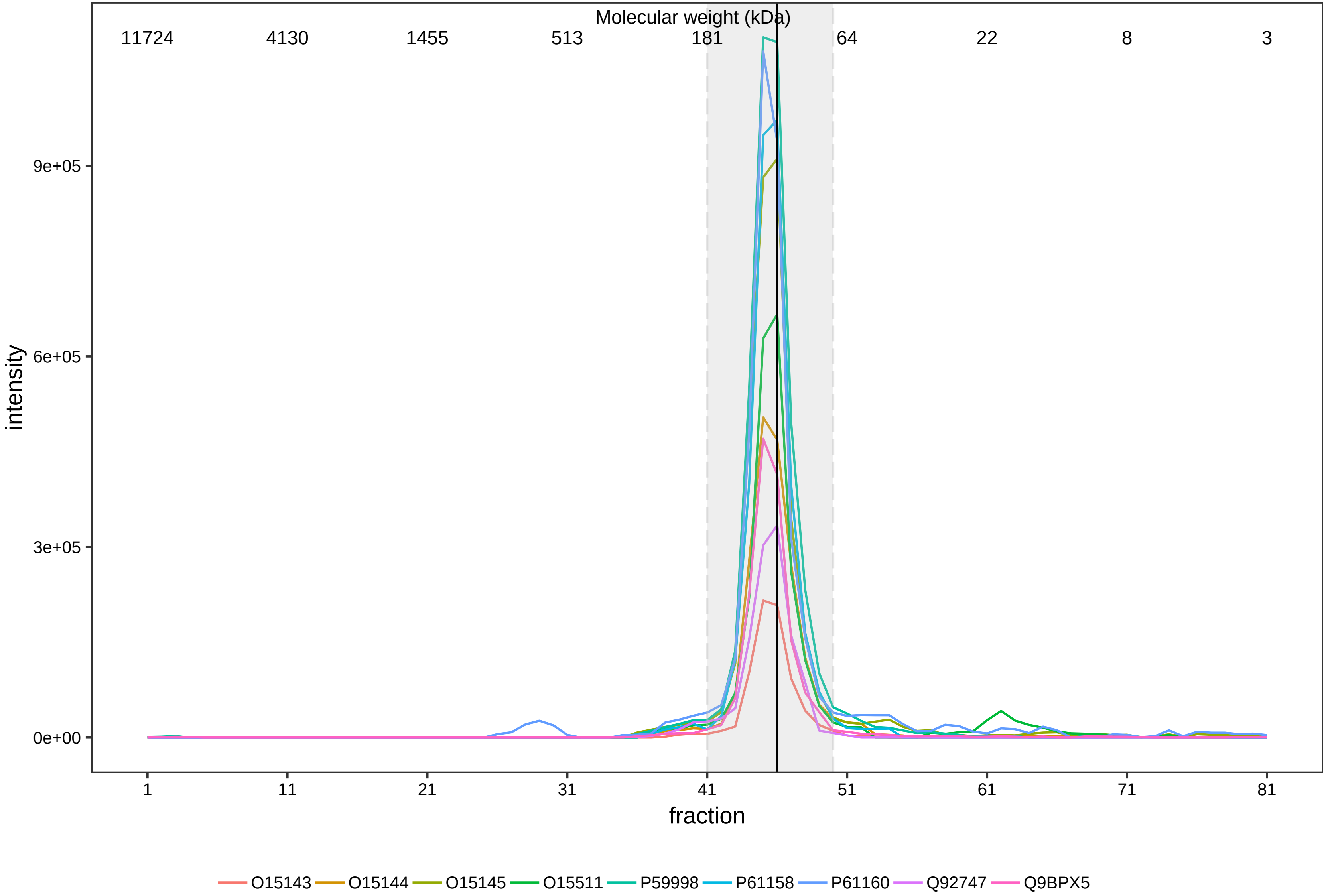

Feature ID 290

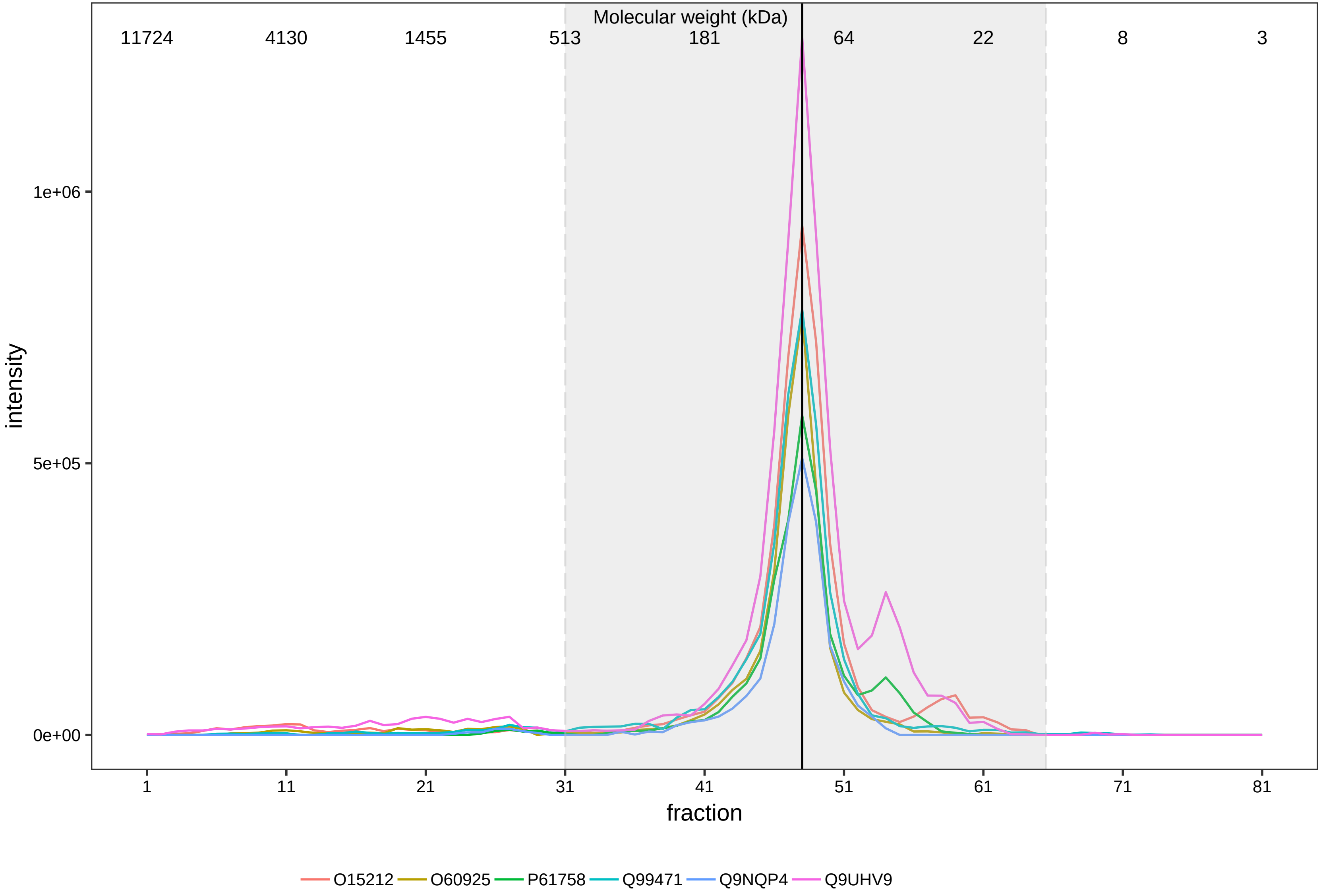

Feature ID 291

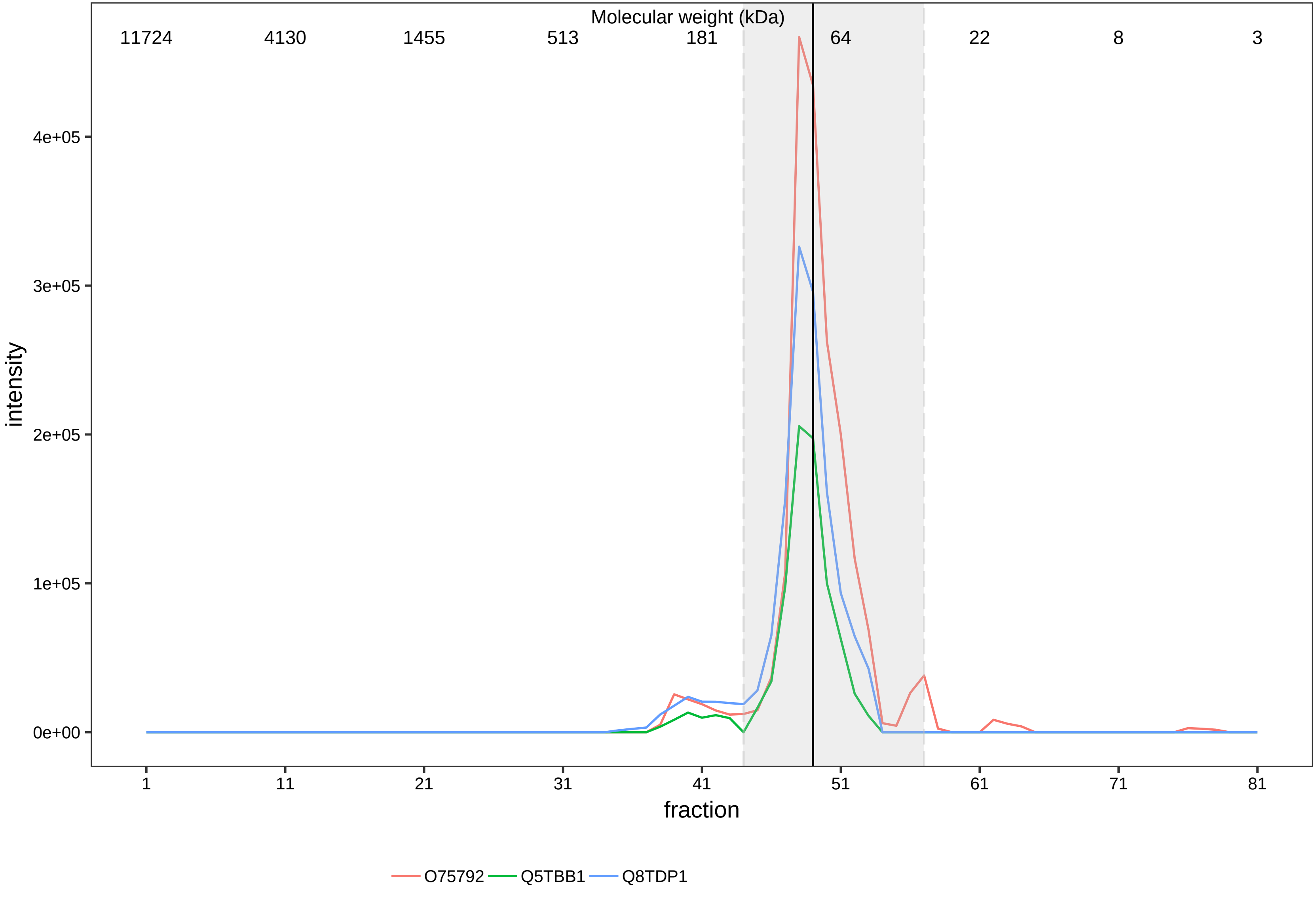

Feature ID 292

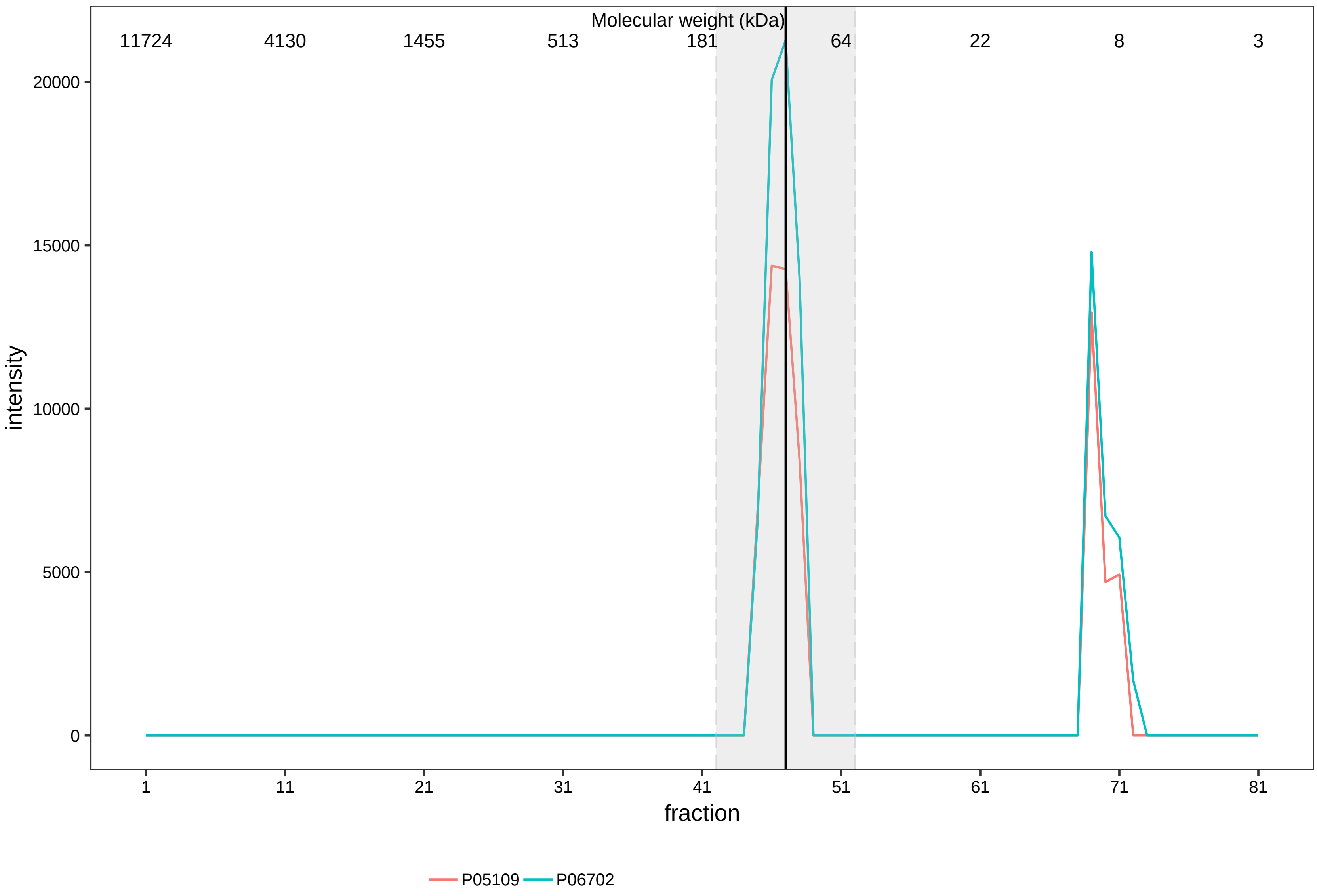

Feature ID 293

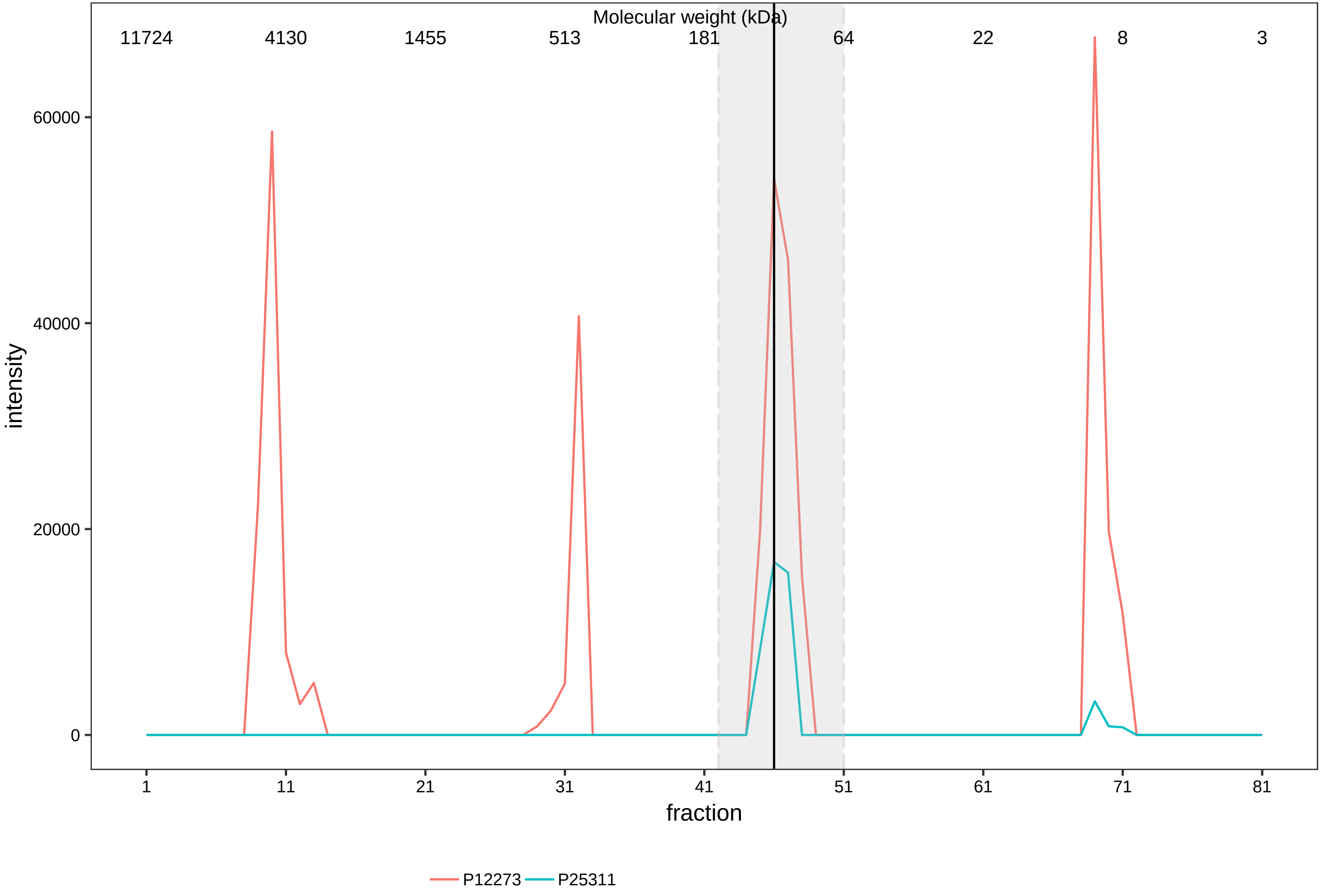

Feature ID 294

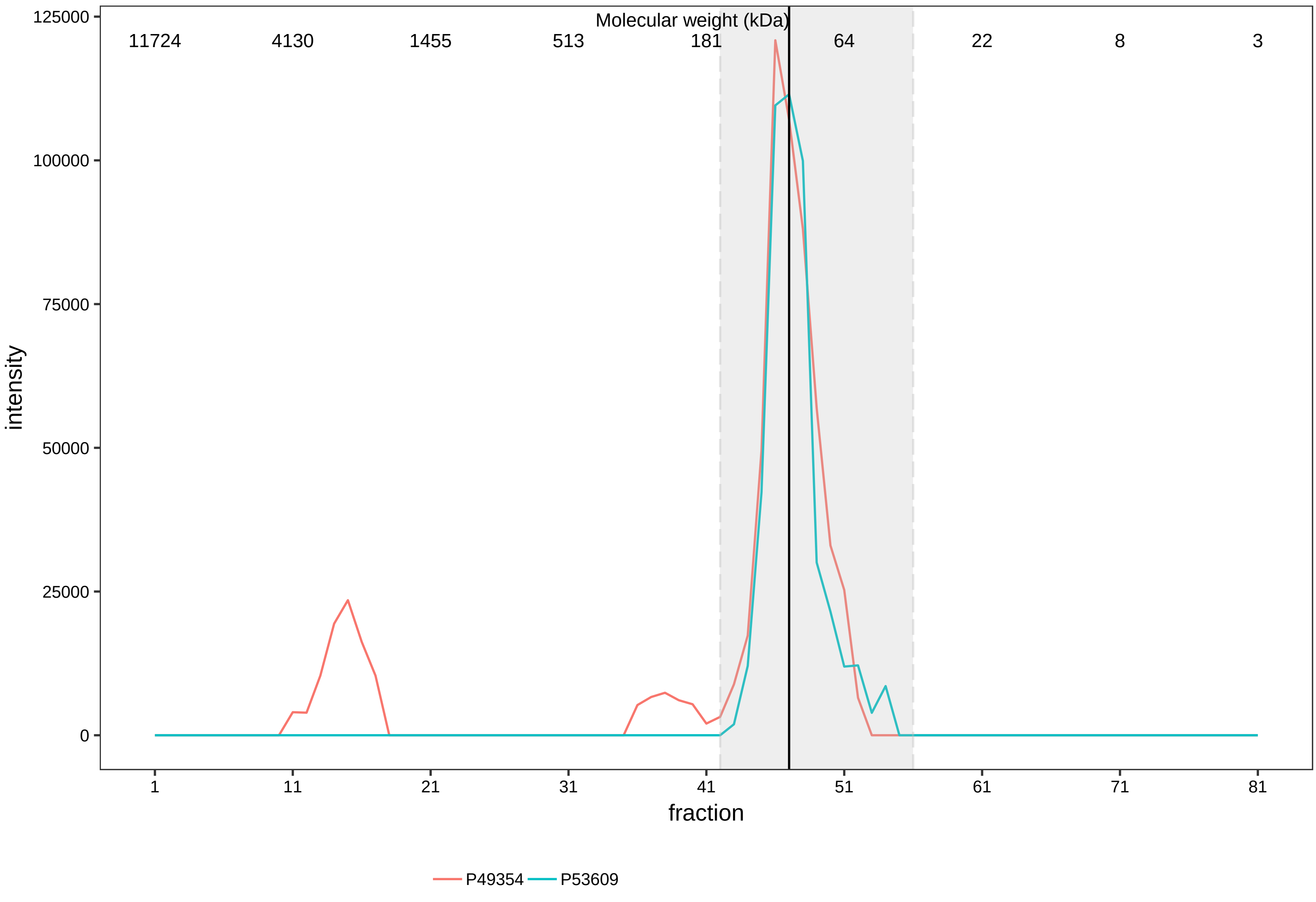

Feature ID 295

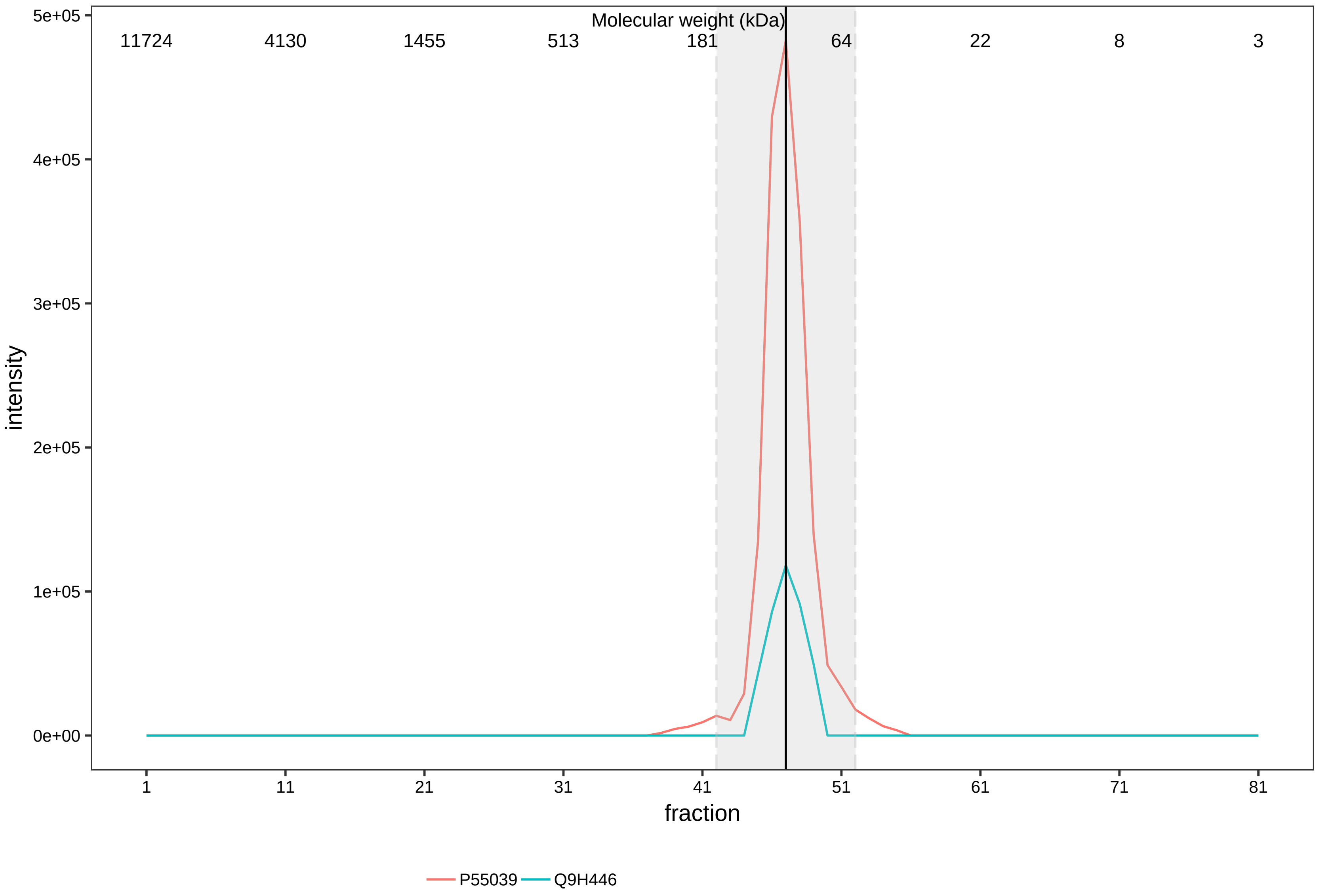

Feature ID 296

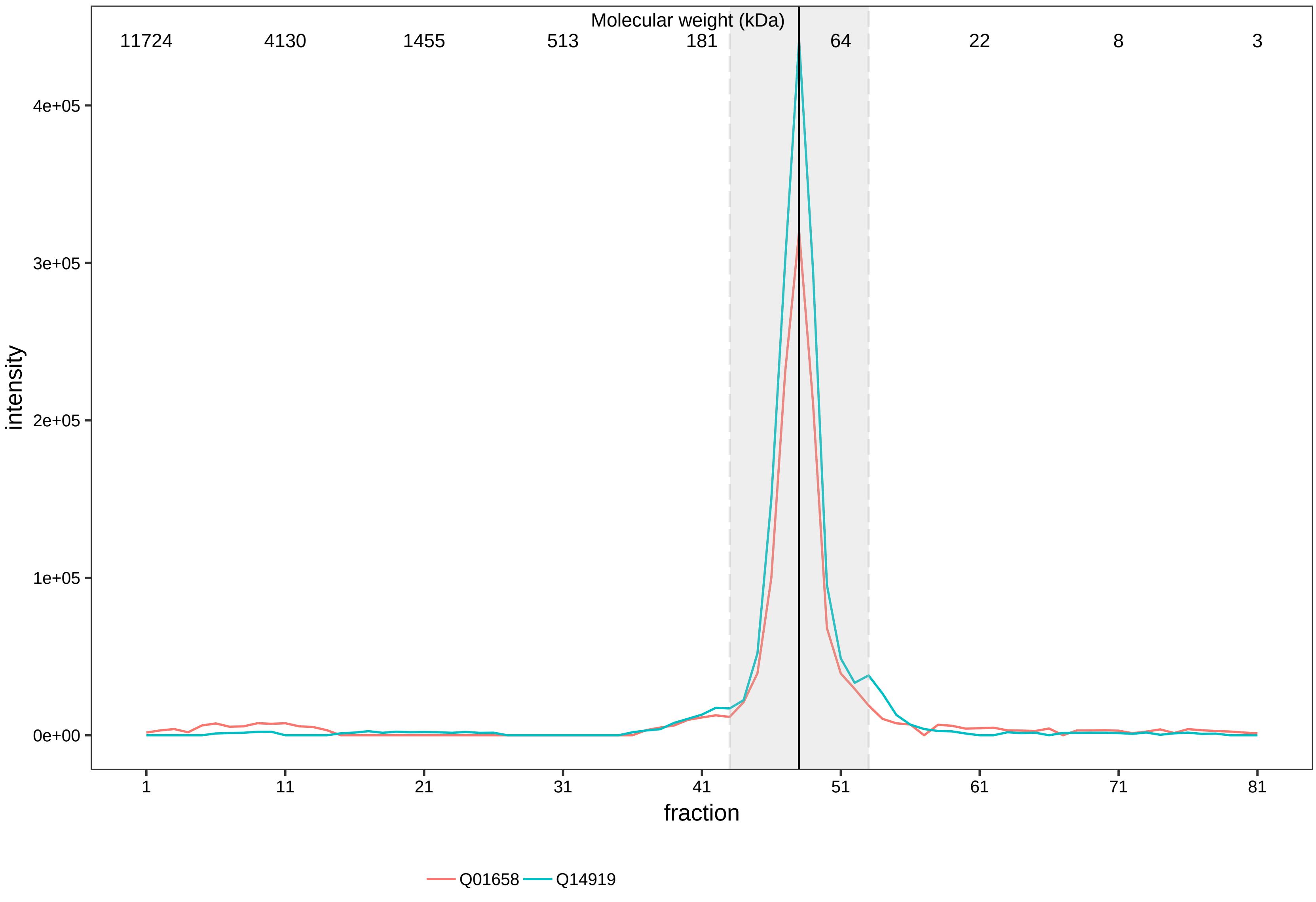

Feature ID 297

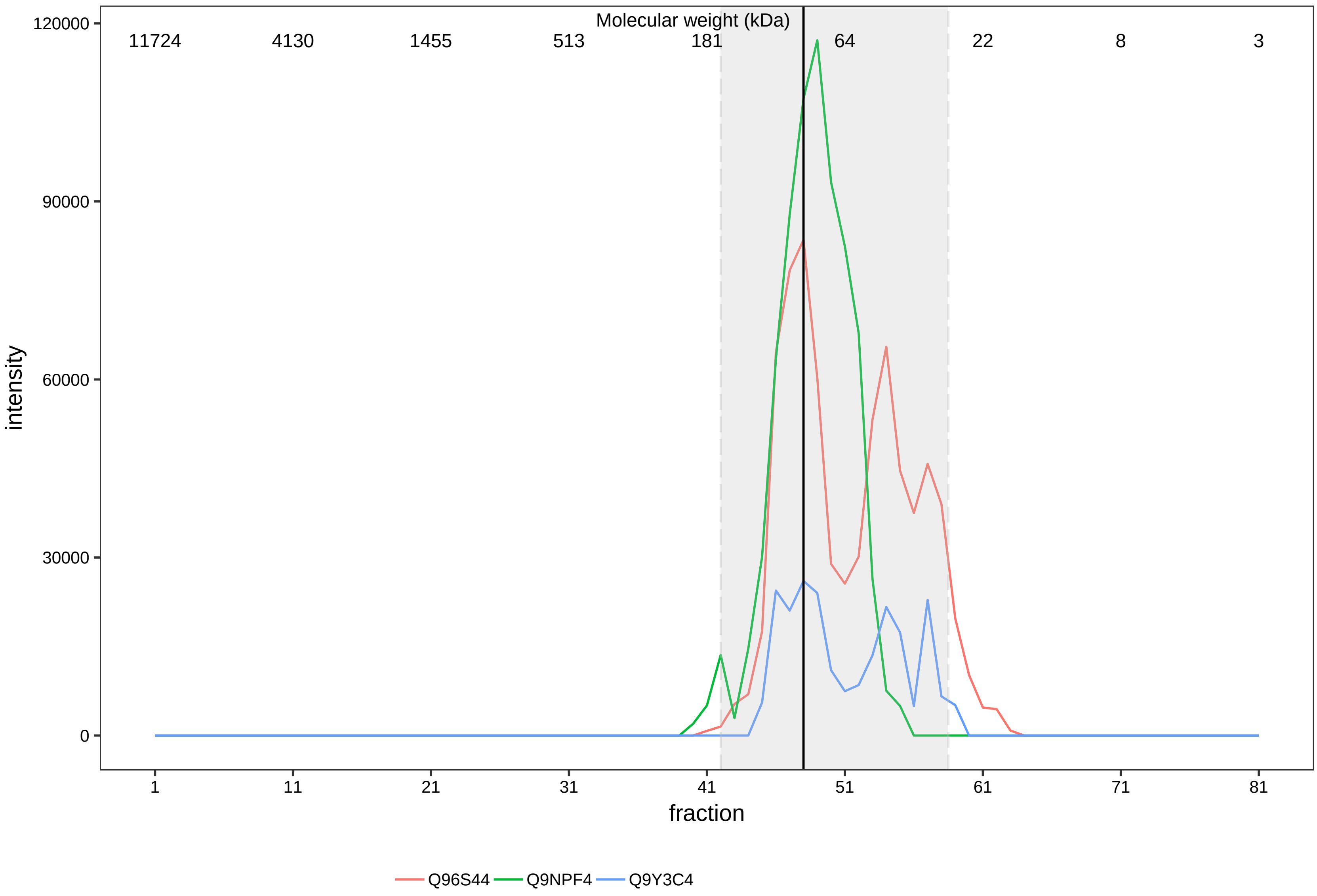

Feature ID 298

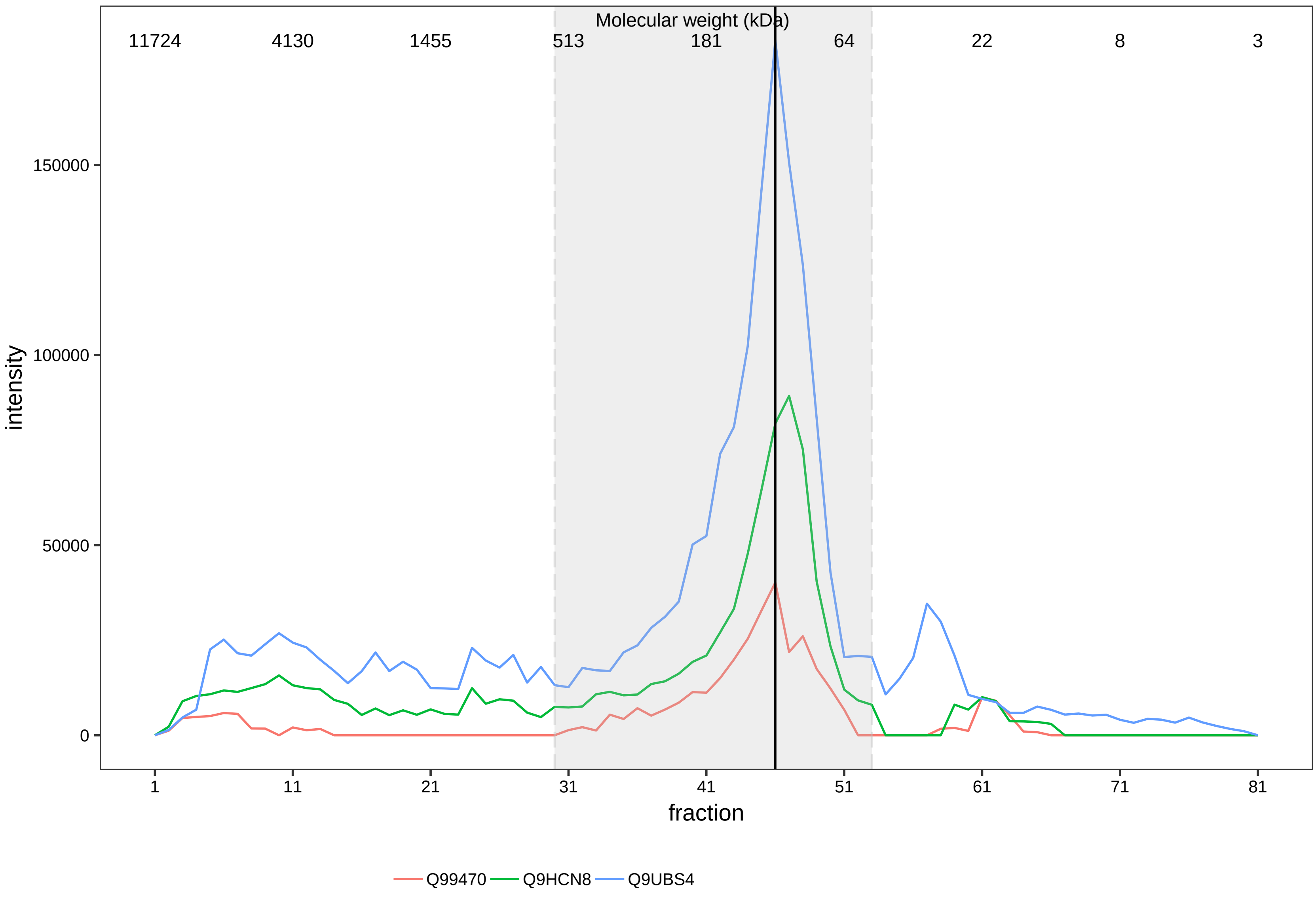

Feature ID 299

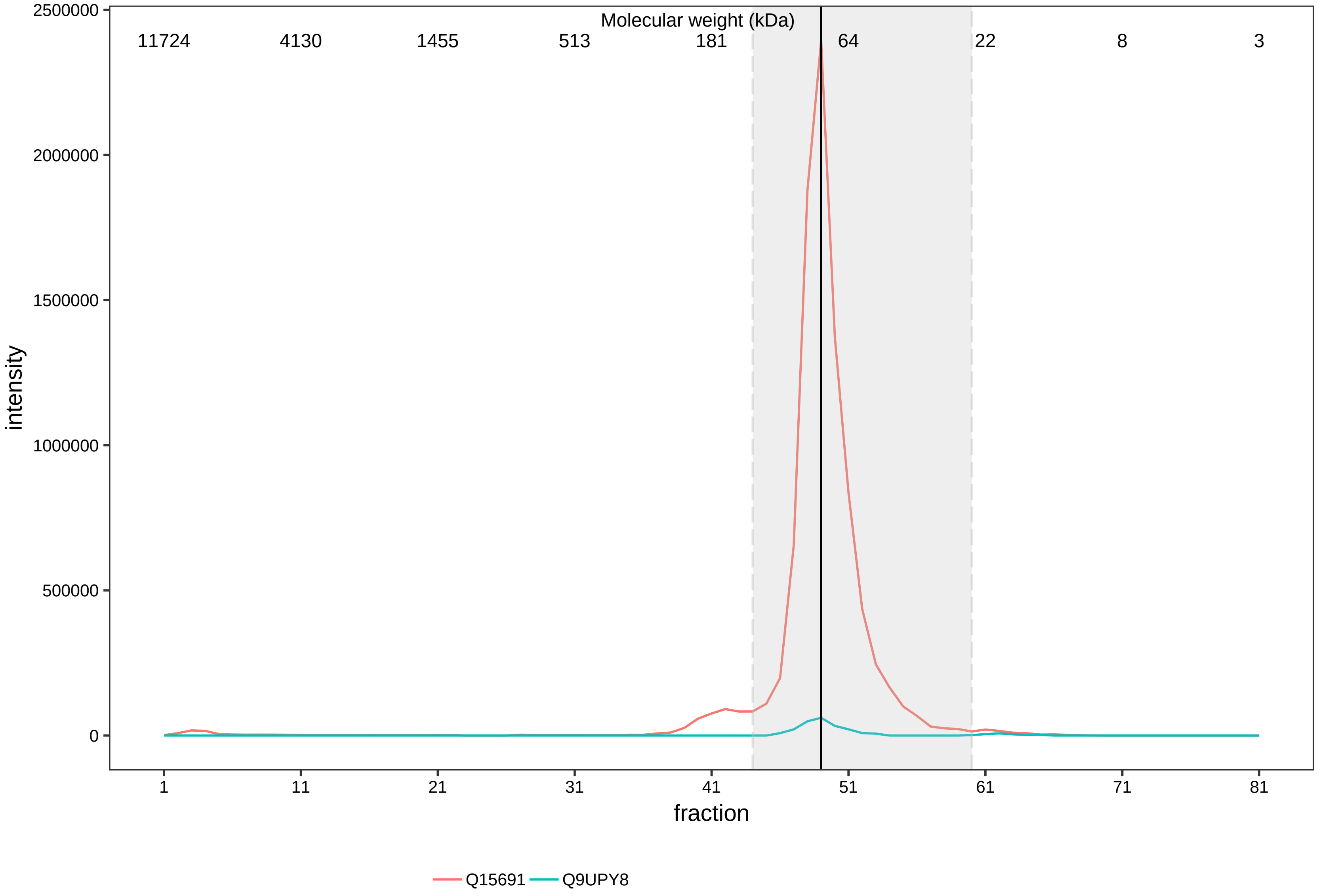

Feature ID 300

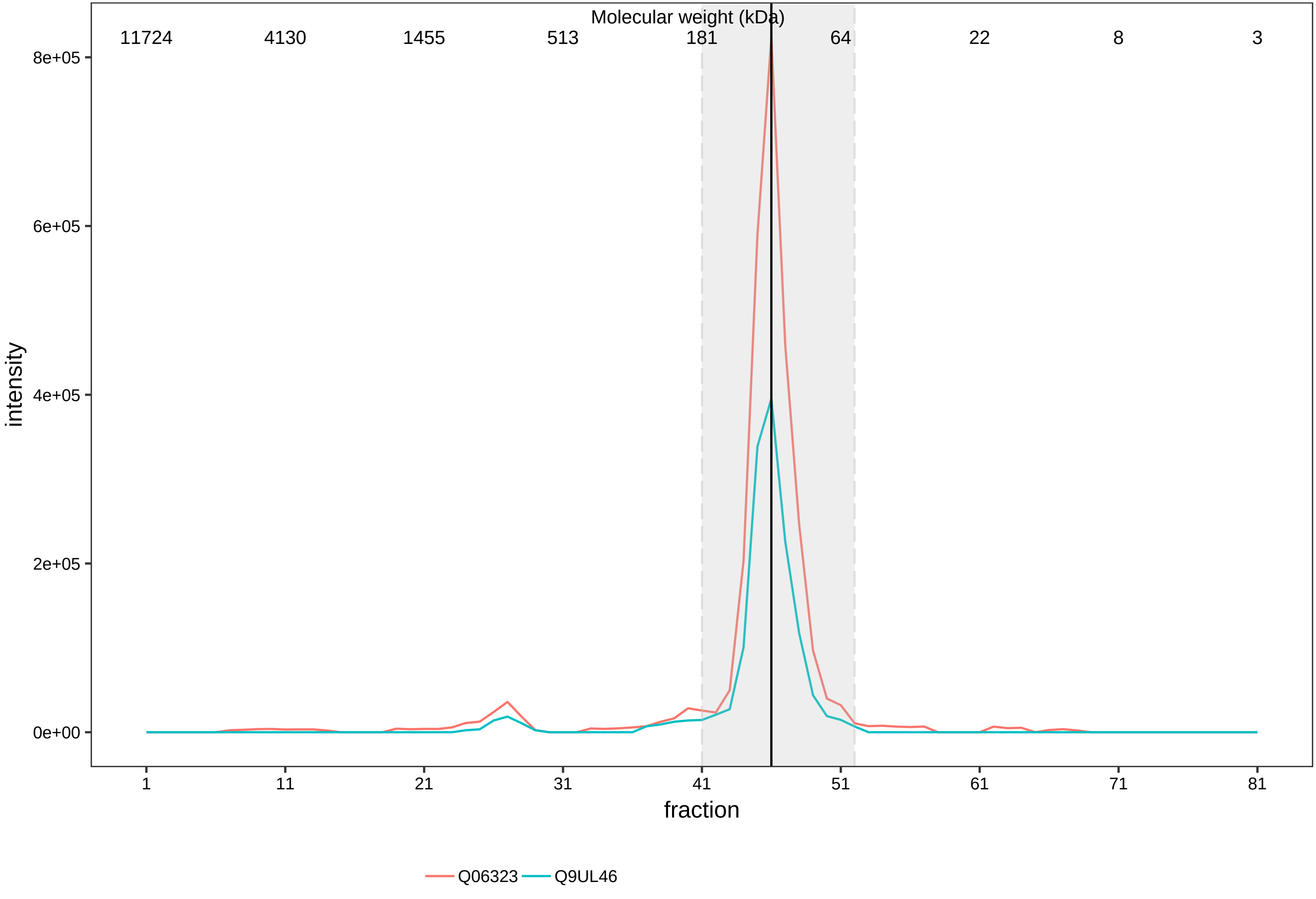

Feature ID 301

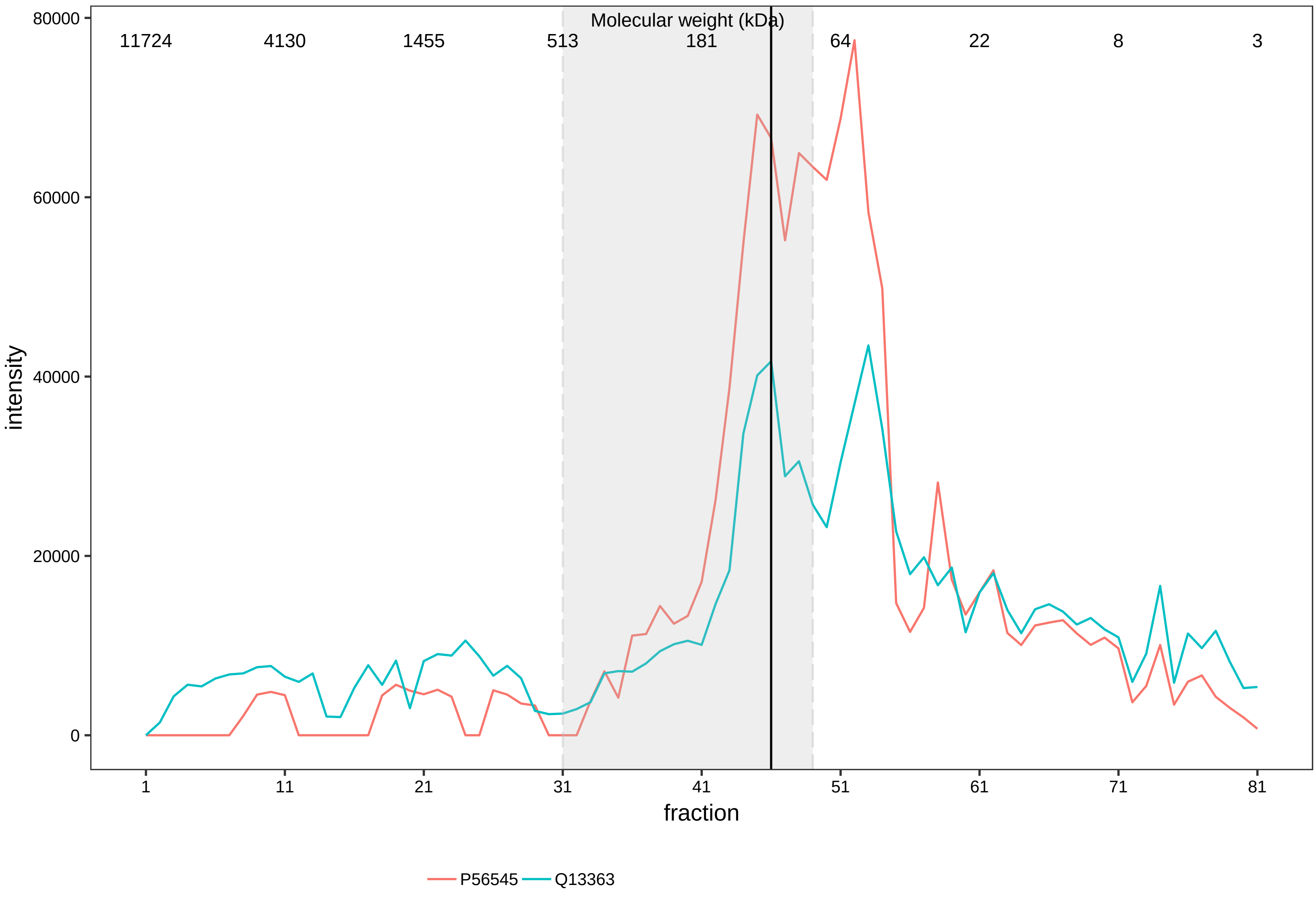

Feature ID 302

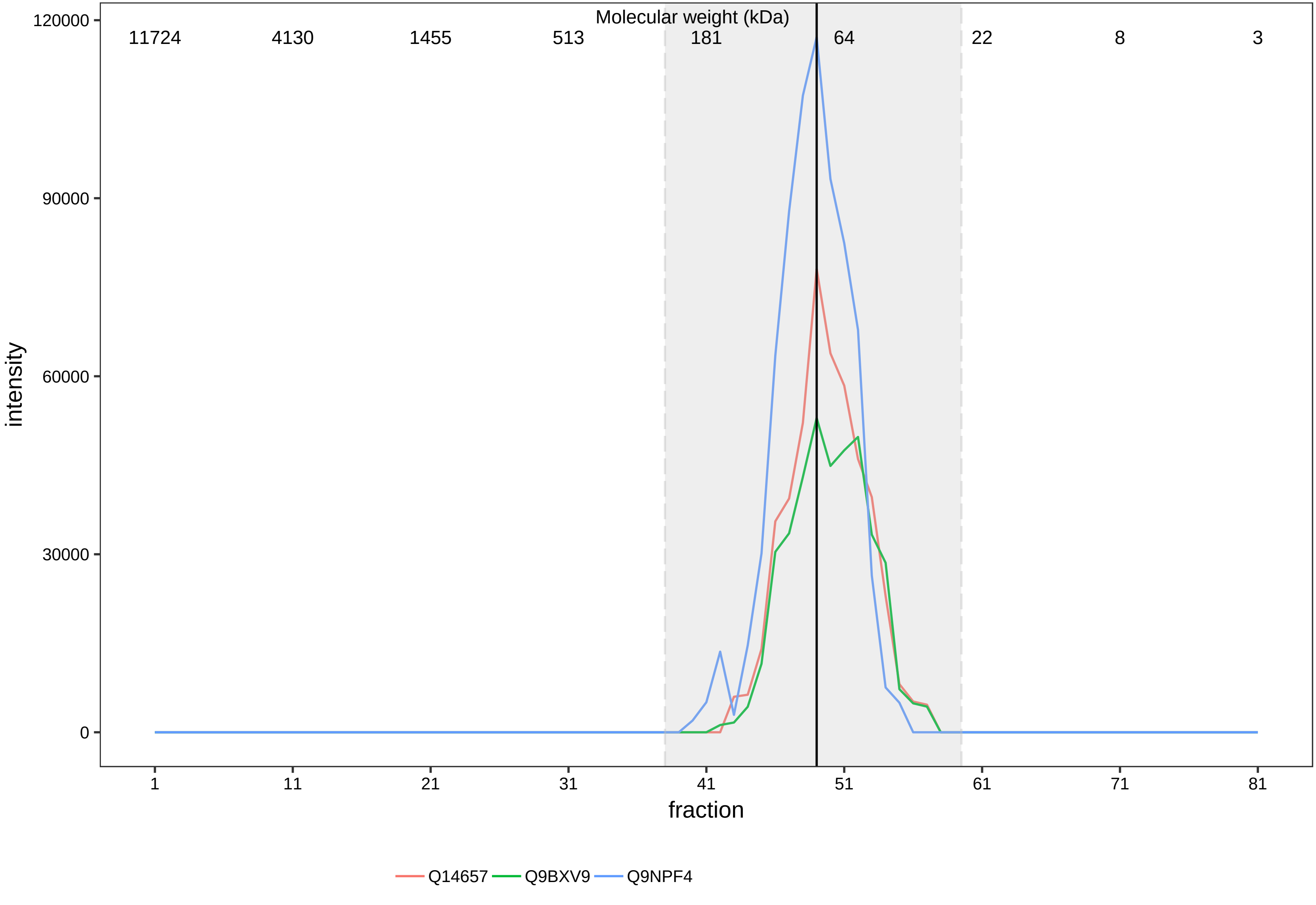

Feature ID 303

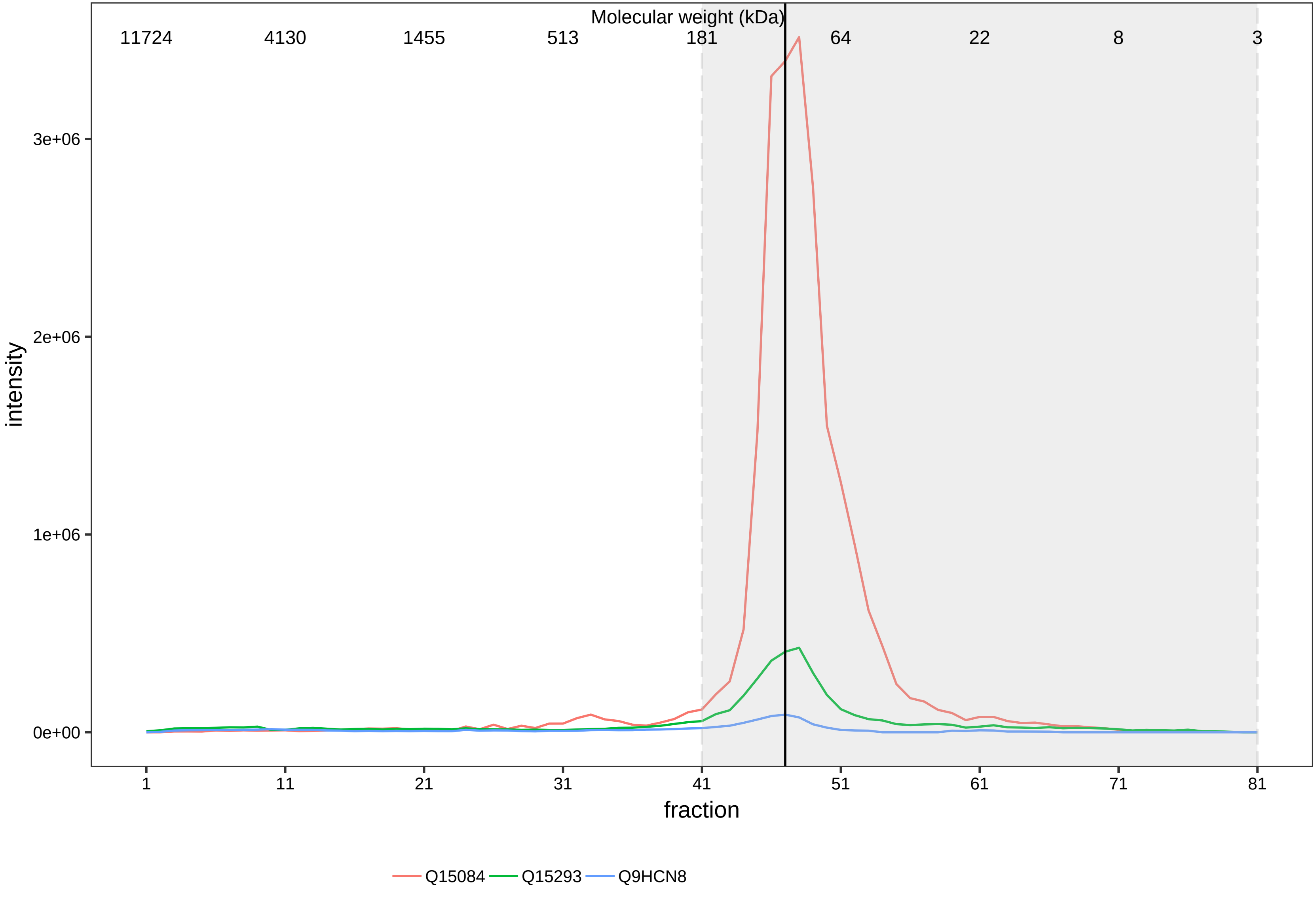

Feature ID 304

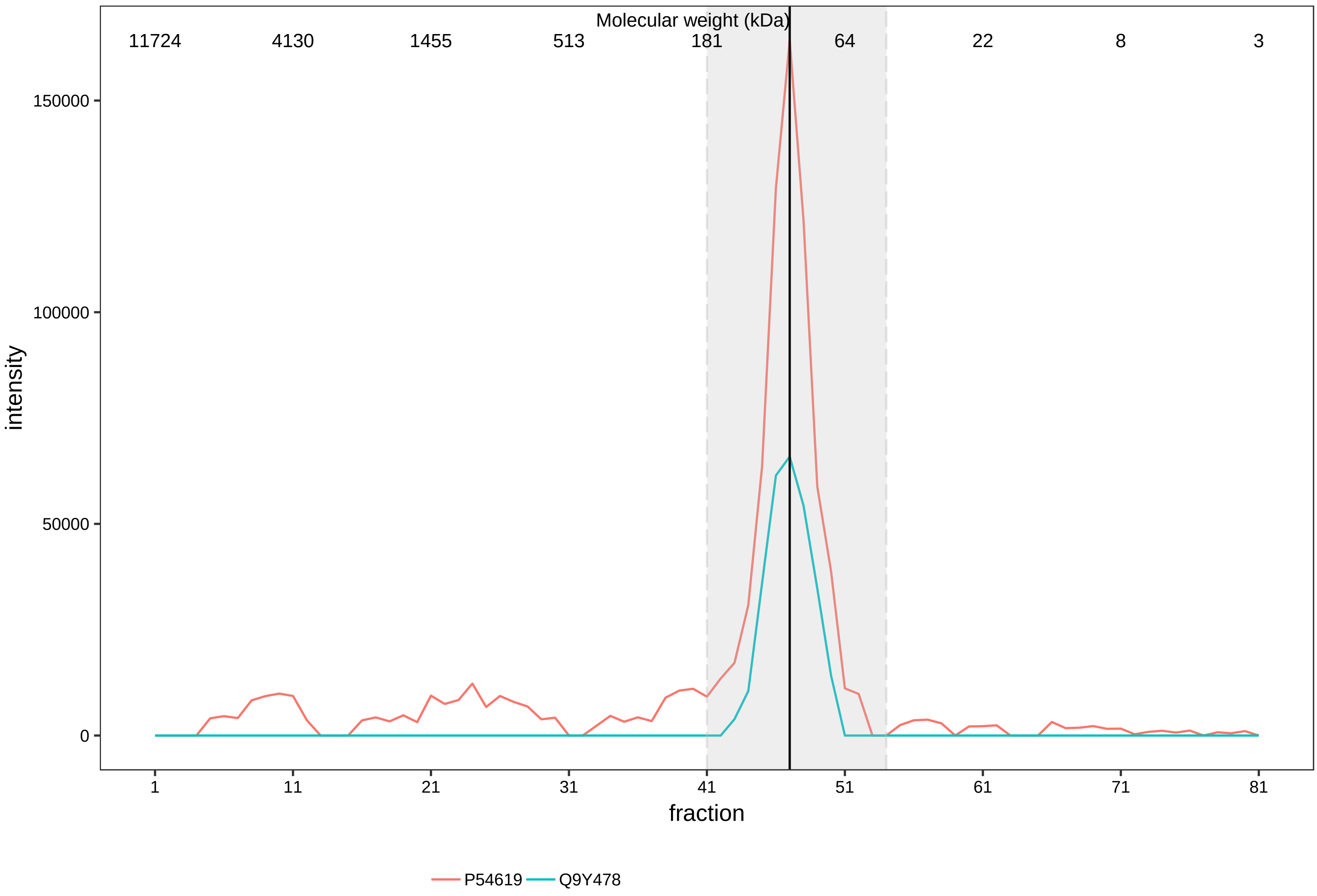

Feature ID 305

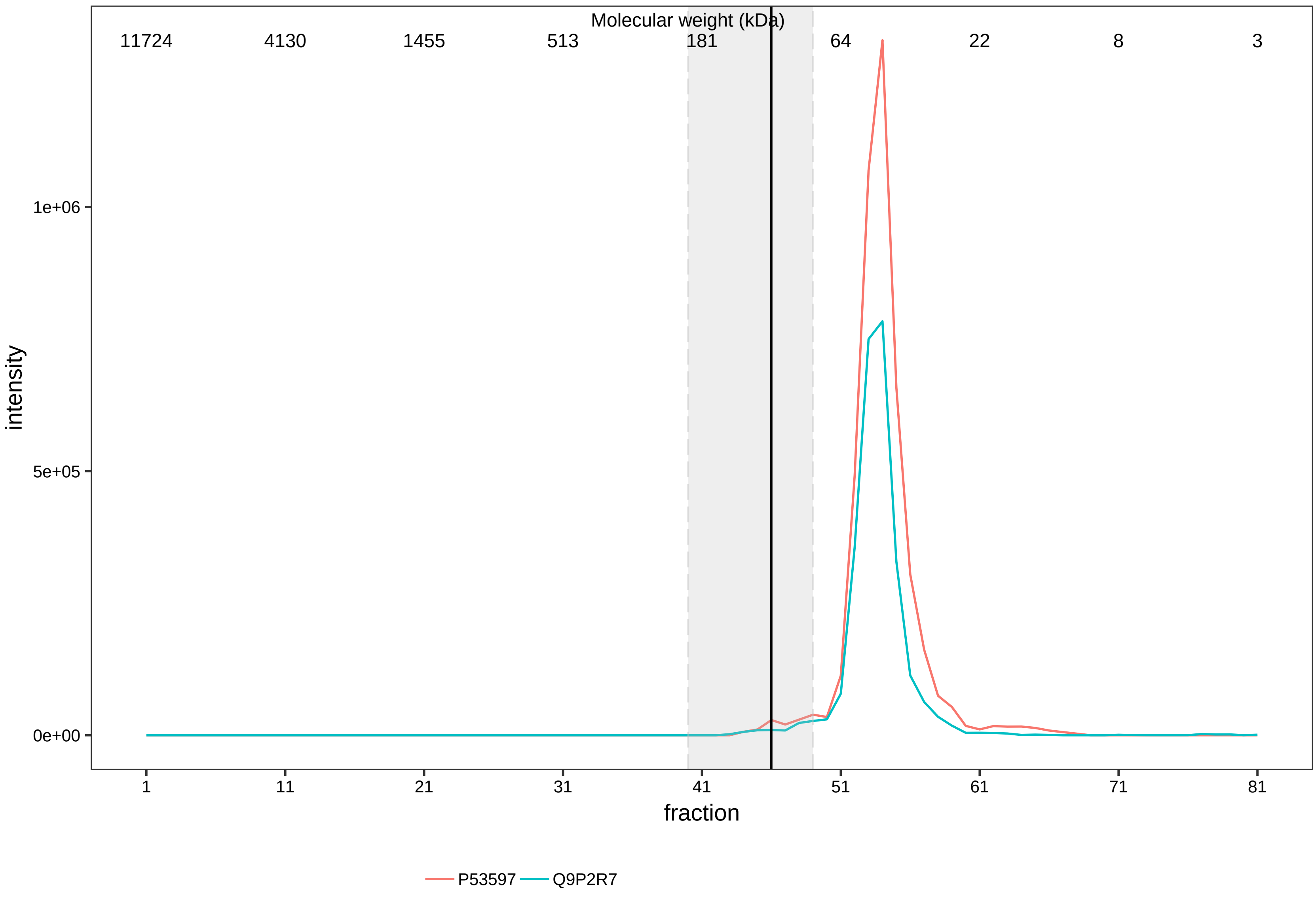

Feature ID 306

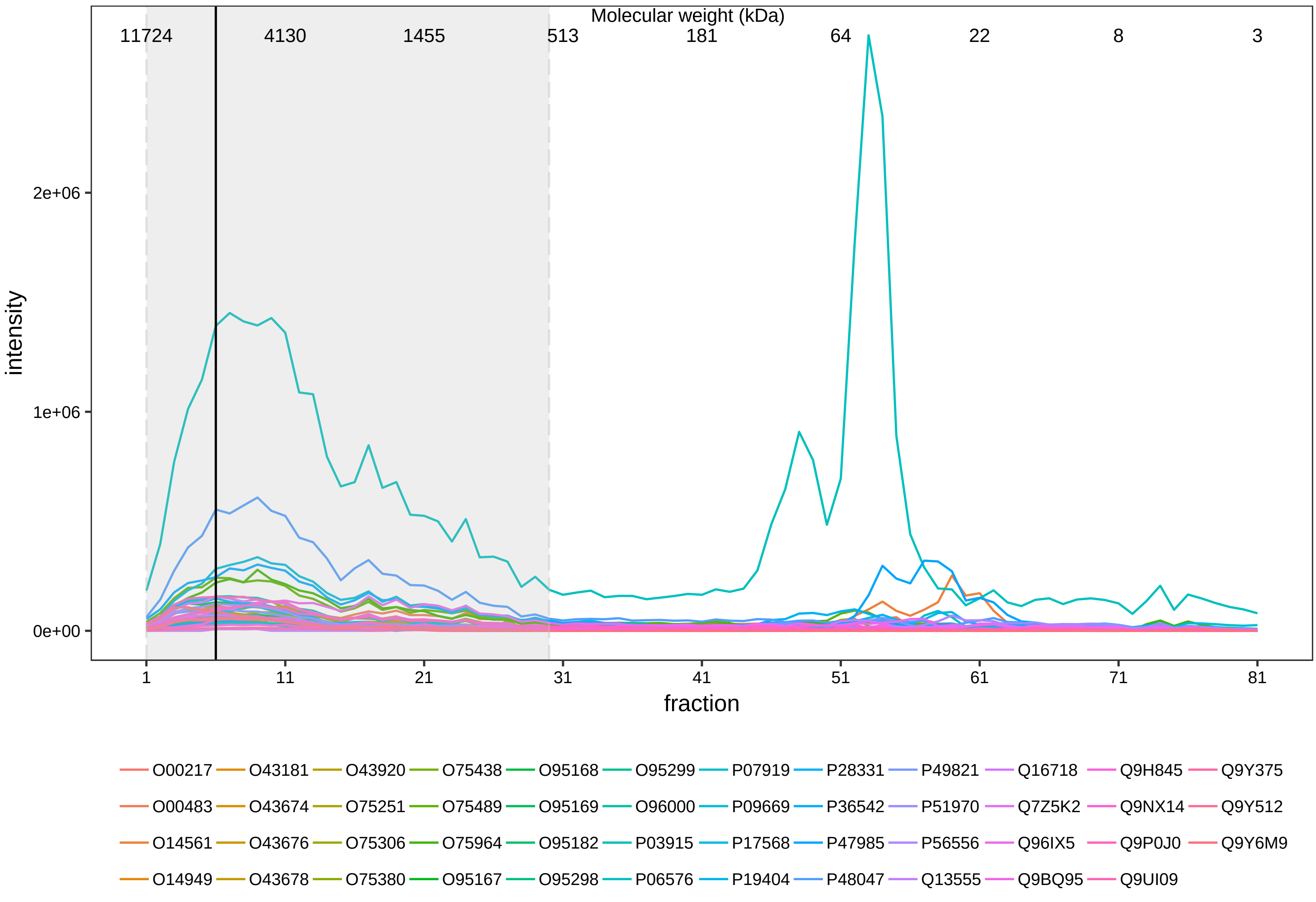

Feature ID 307

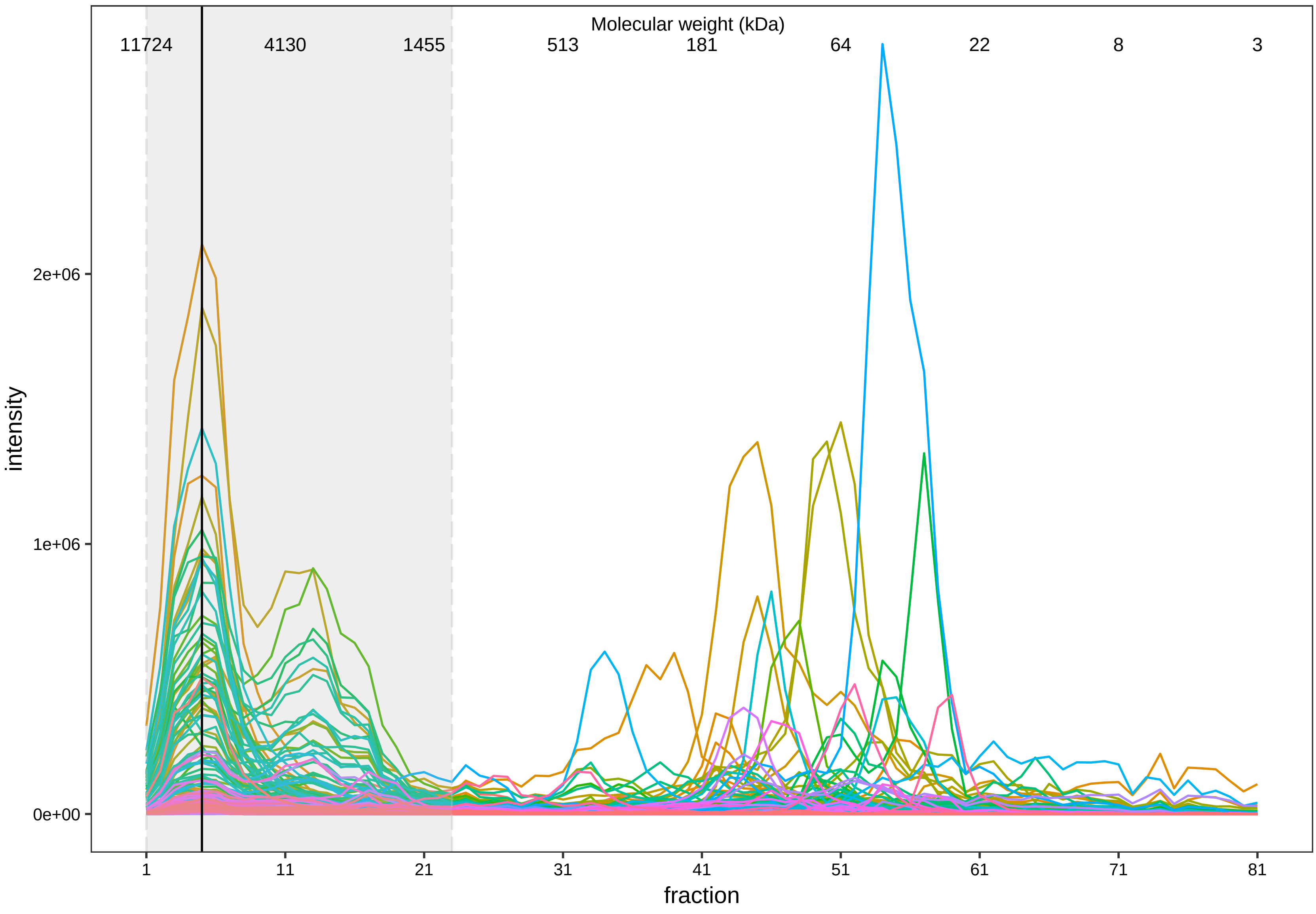

Feature ID 308

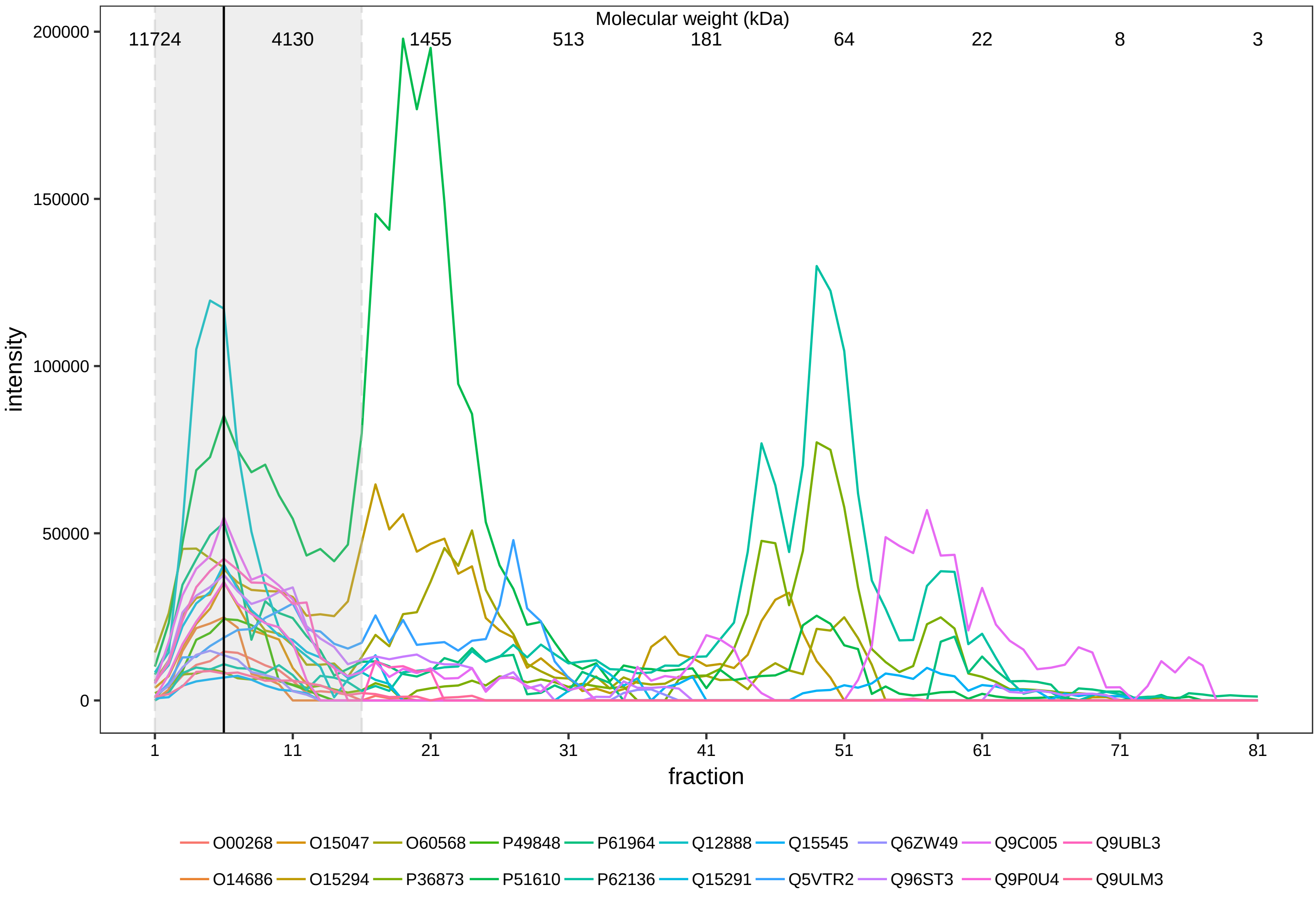

Feature ID 309

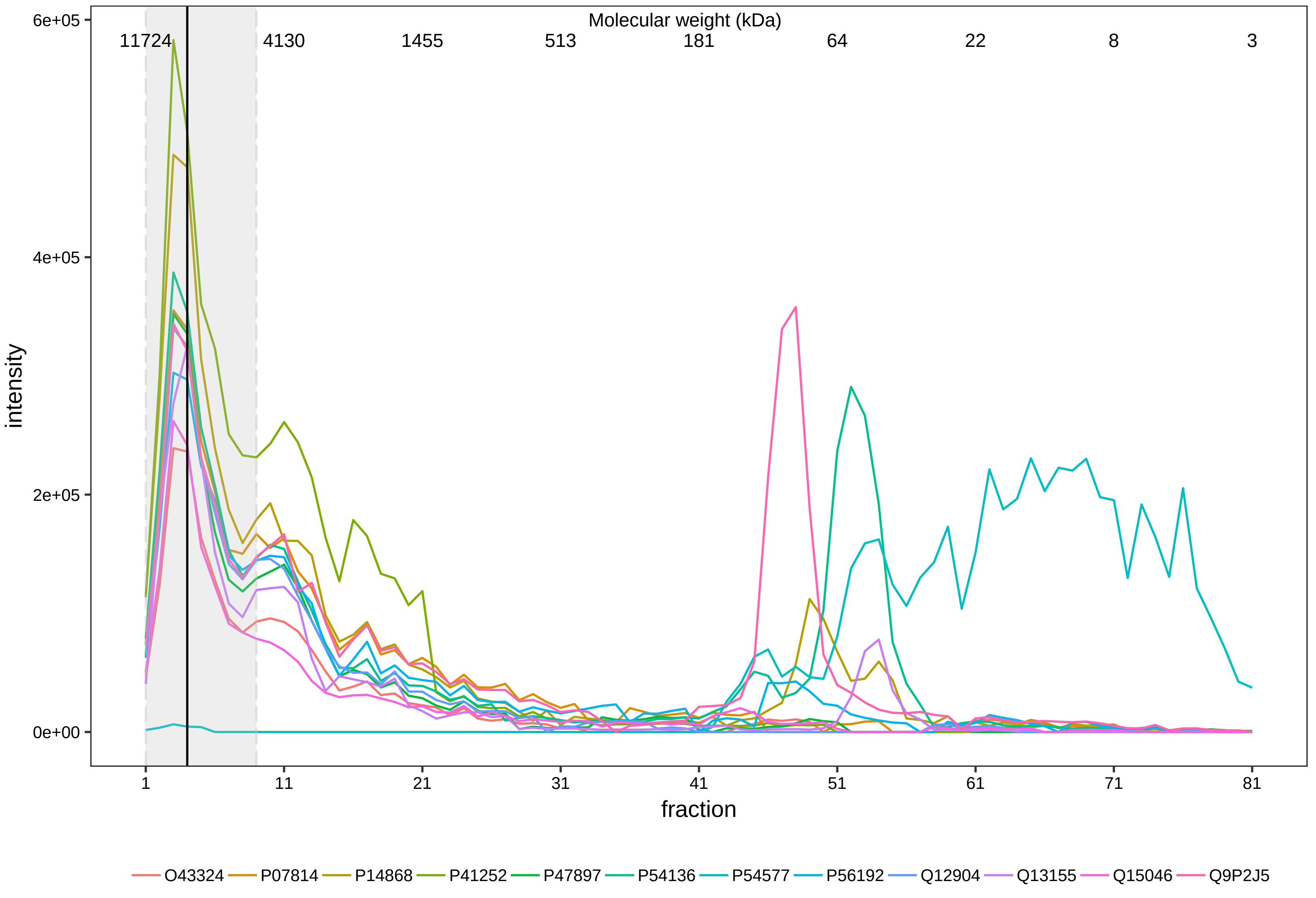

Feature ID 310

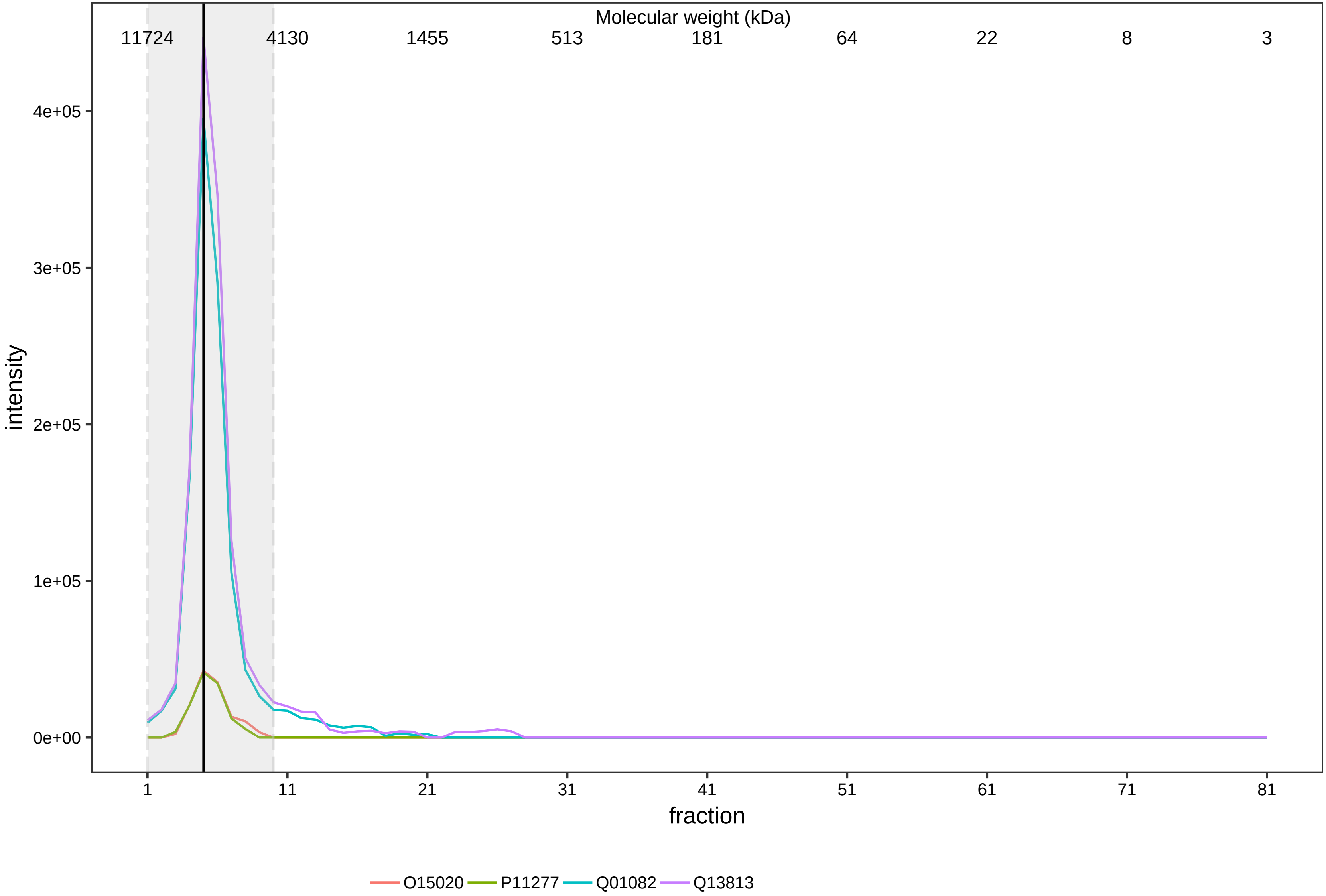

Feature ID 311

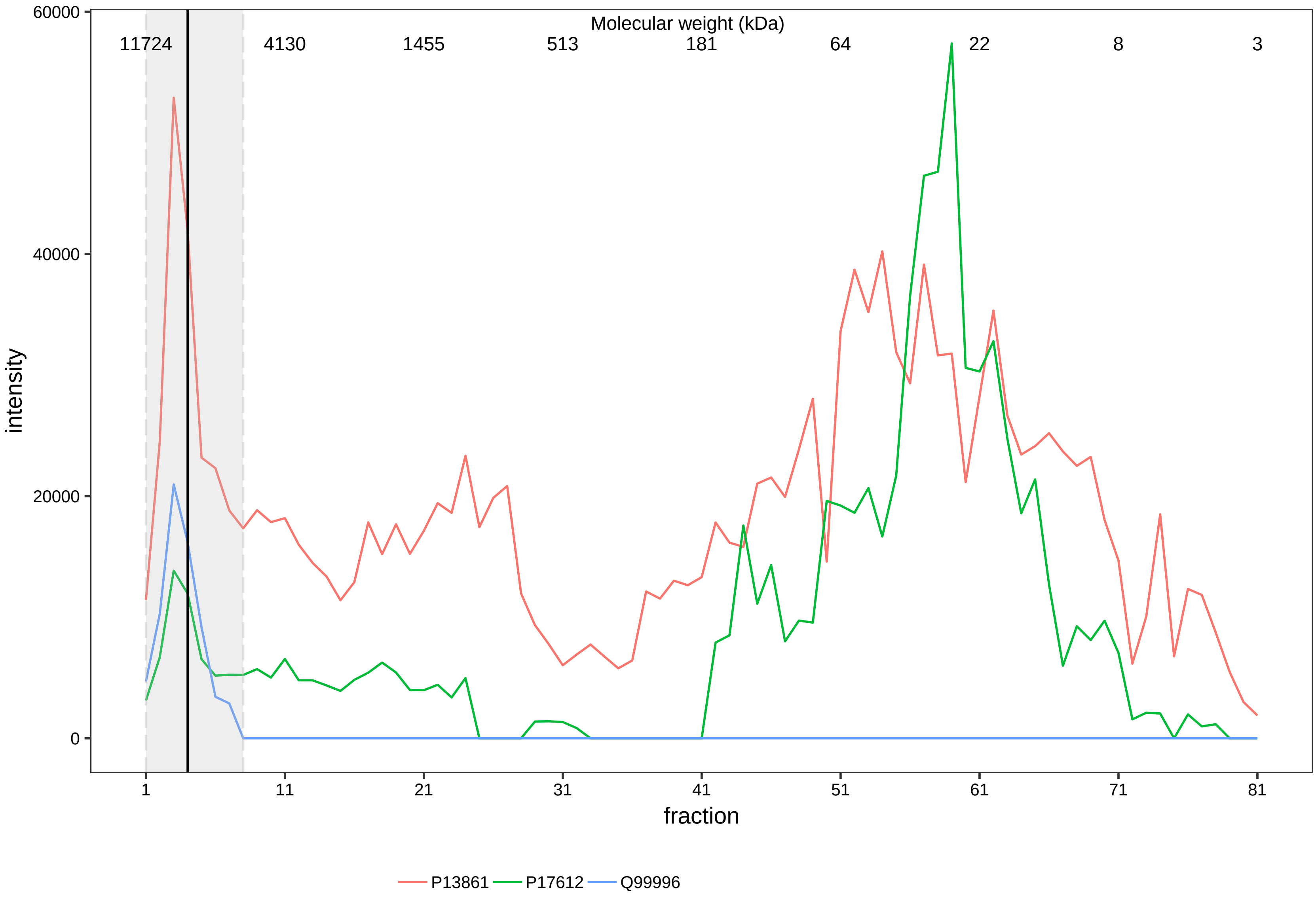

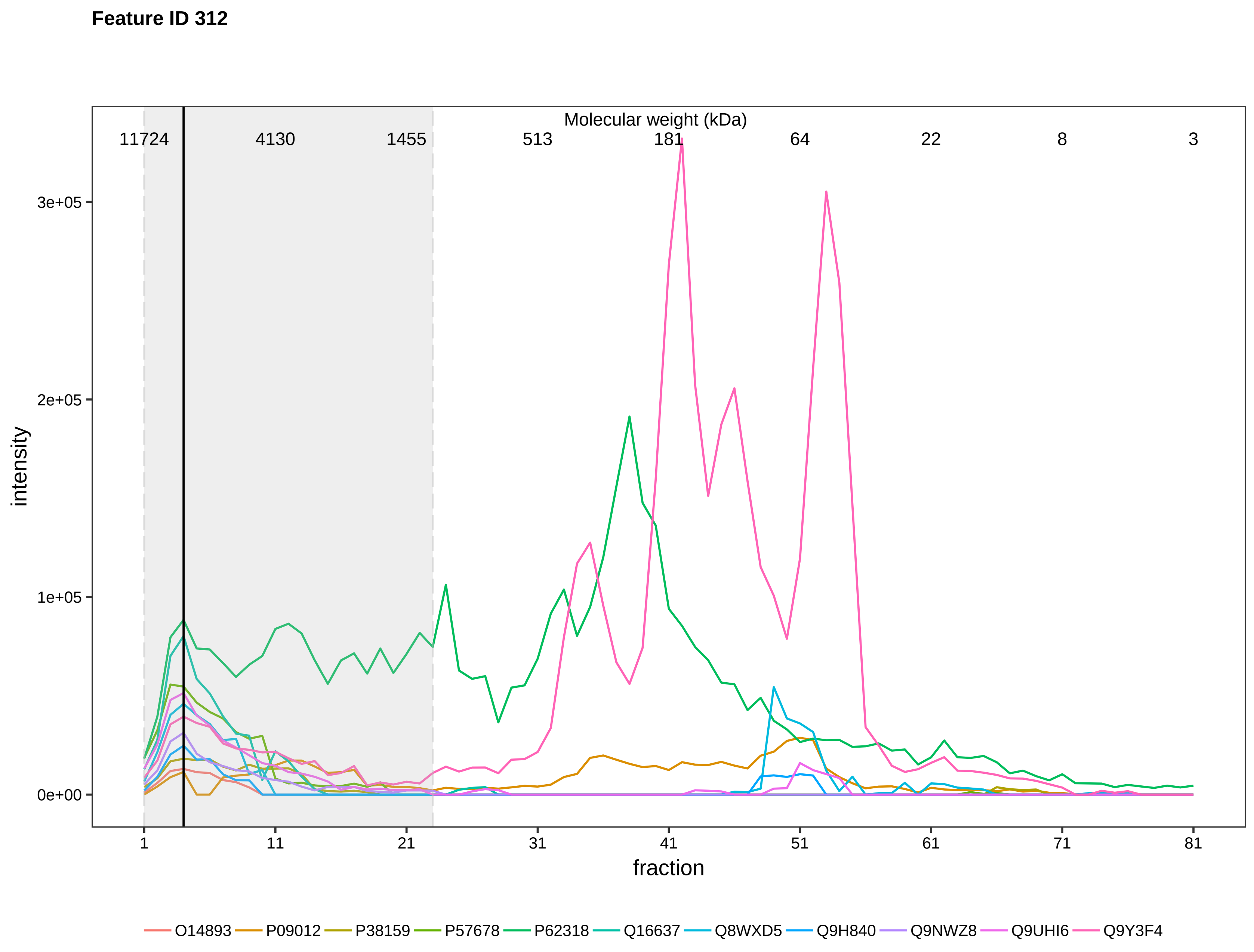

Feature ID 312
Molecular weight (kDa)
11724
4130
1455
513
181
64
22
8
3
3e+05
2e+05
1e+05
0e+00
intensity
1
11
21
31
41
51
61
71
81
fraction
O14893
P09012
P38159
P57678
P62318
Q16637
Q8WXD5
Q9H840
Q9NWZ8
Q9UHI6
Q9Y3F4

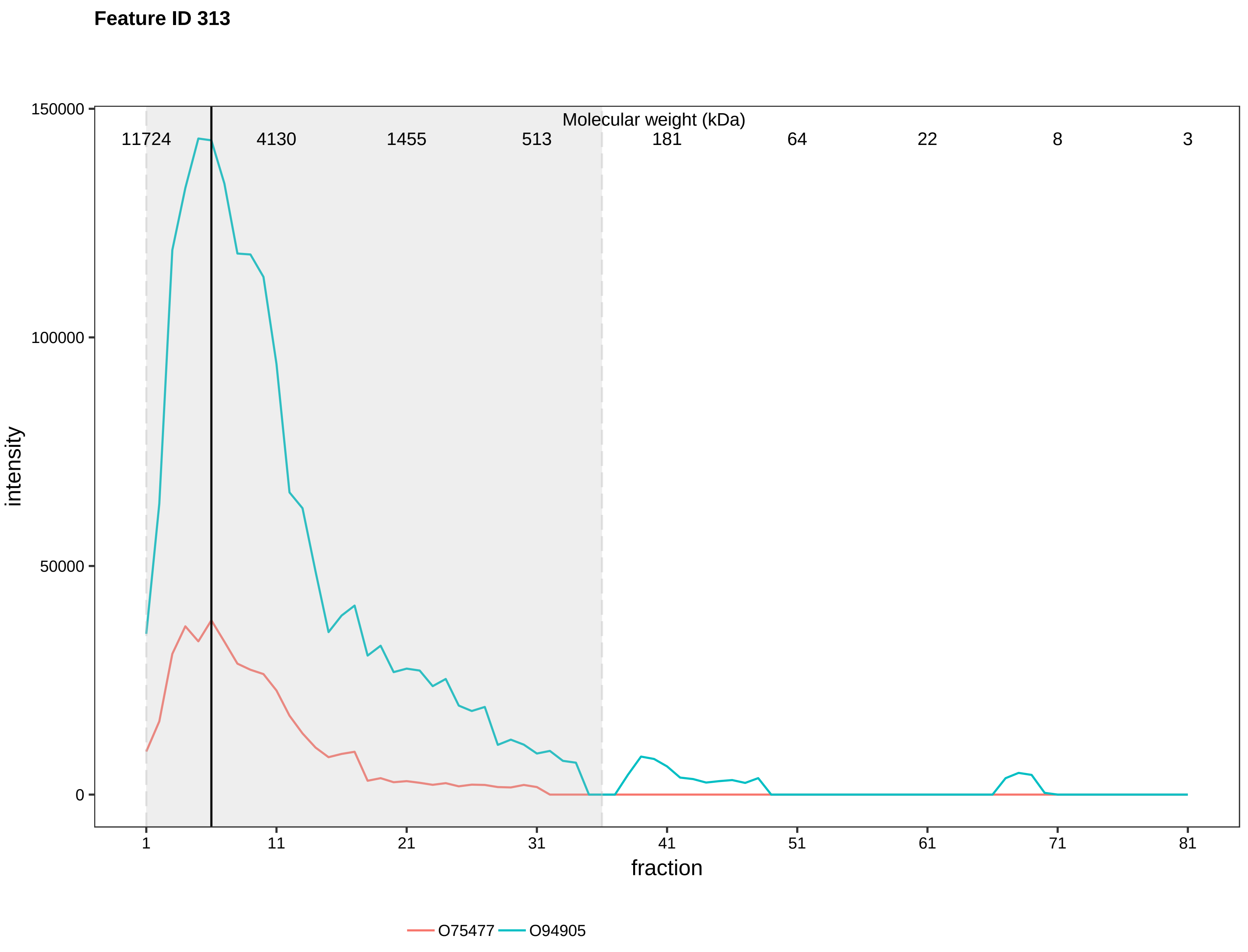
Feature ID 313
Molecular weight (kDa)
11724
4130
1455
513
181
64
22
8
3
150000
100000
50000
0
intensity
1
11
21
31
41
51
61
71
81
fraction
O75477
O94905

Feature ID 314

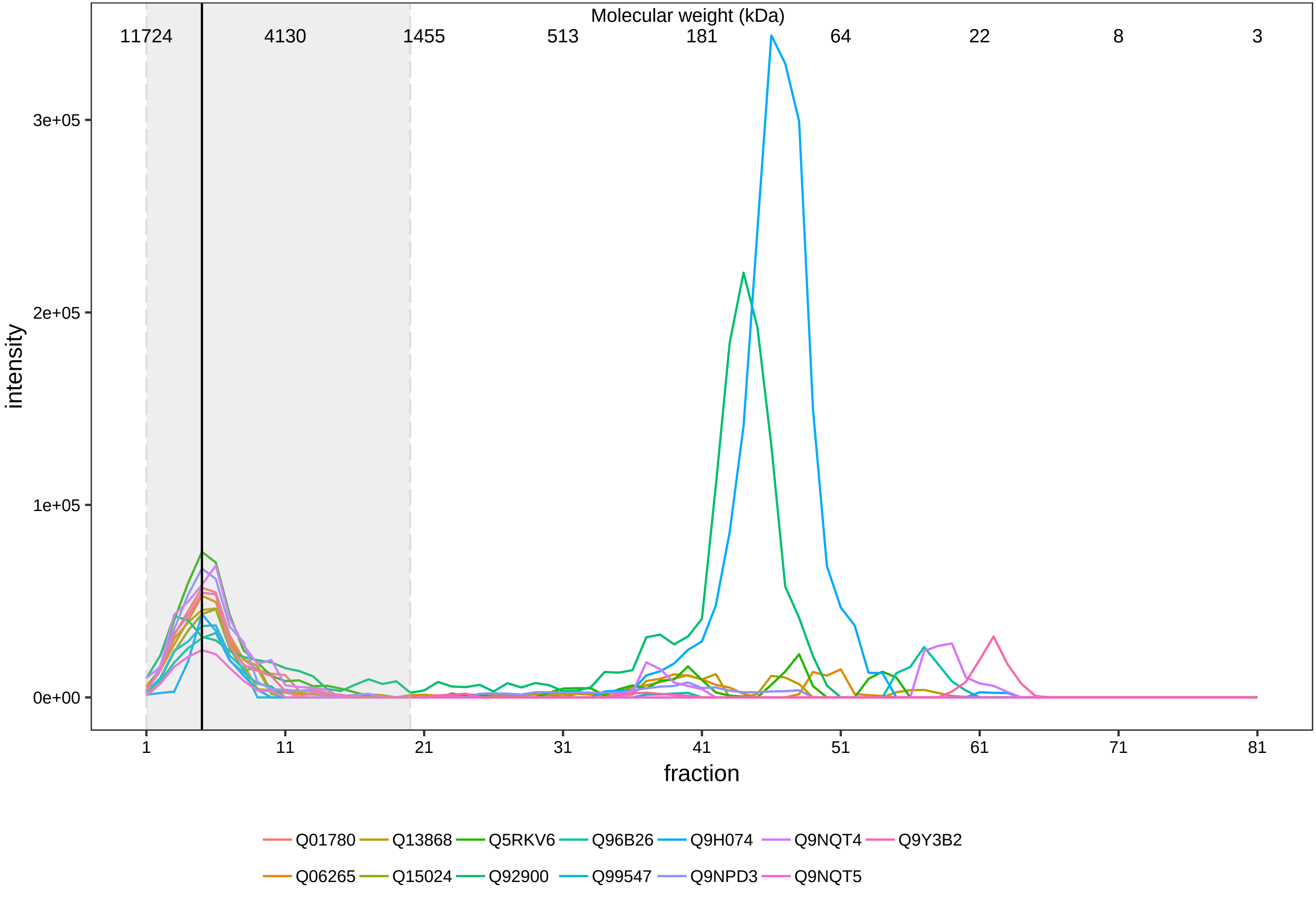

Feature ID 315

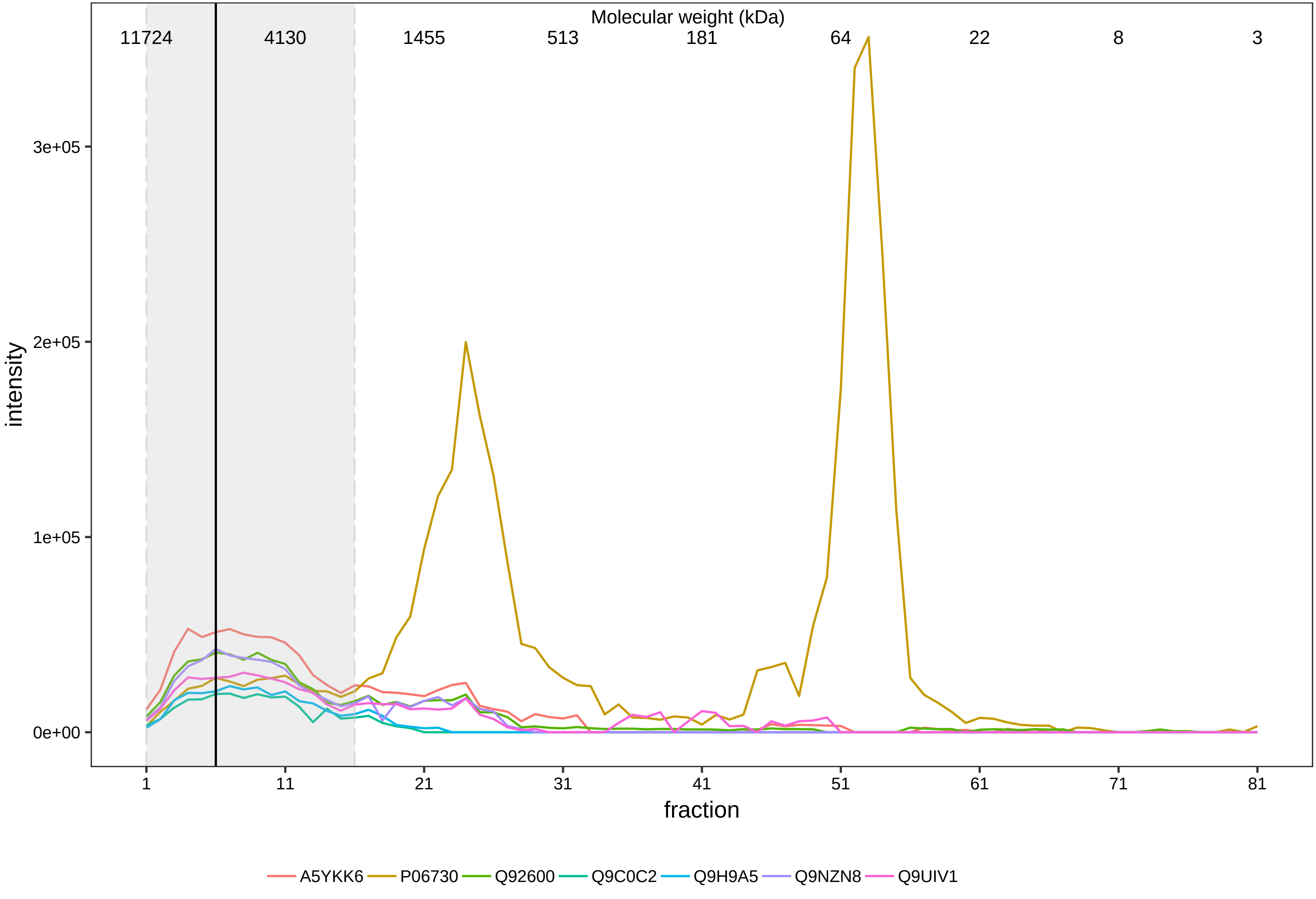

Feature ID 316

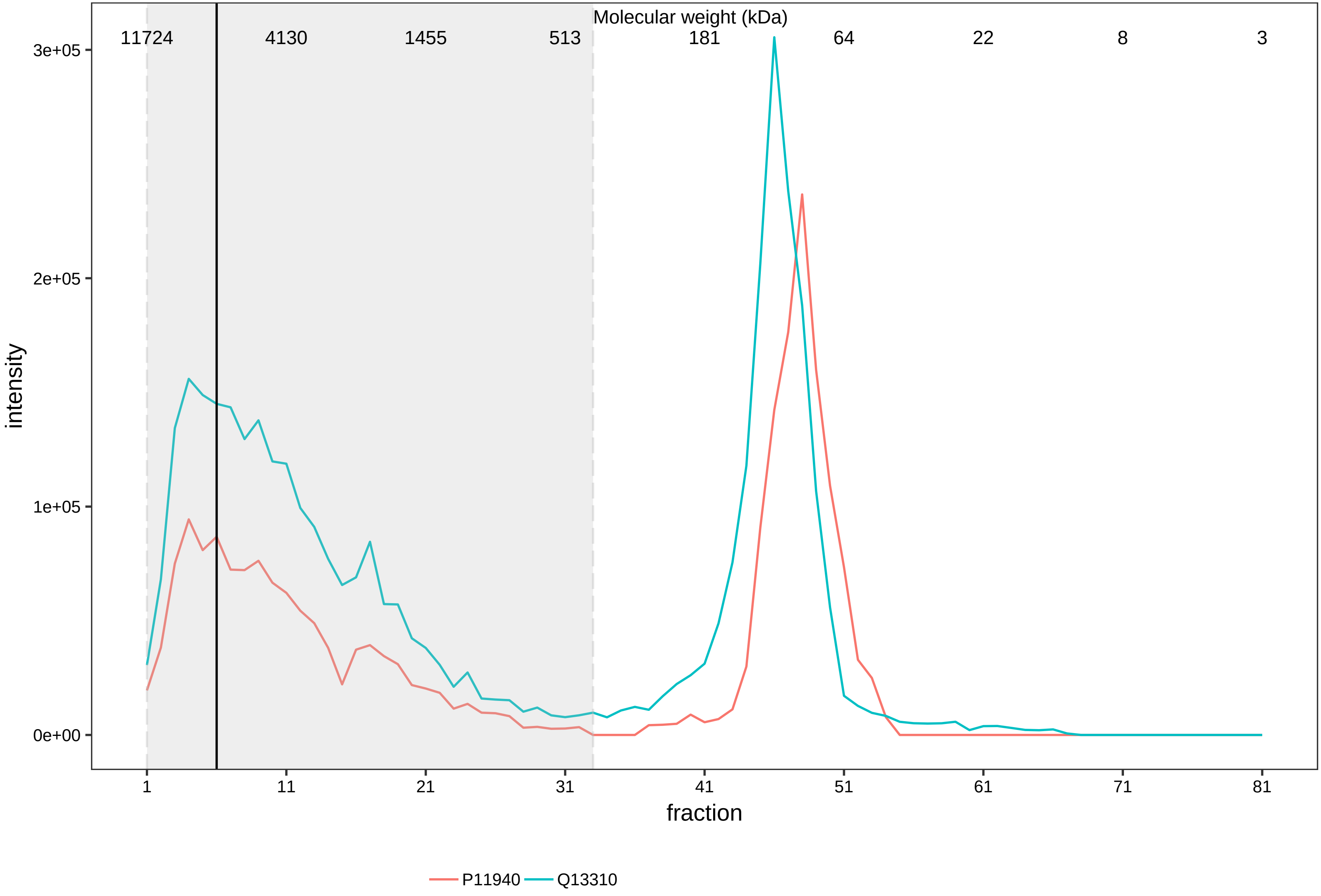

Feature ID 317

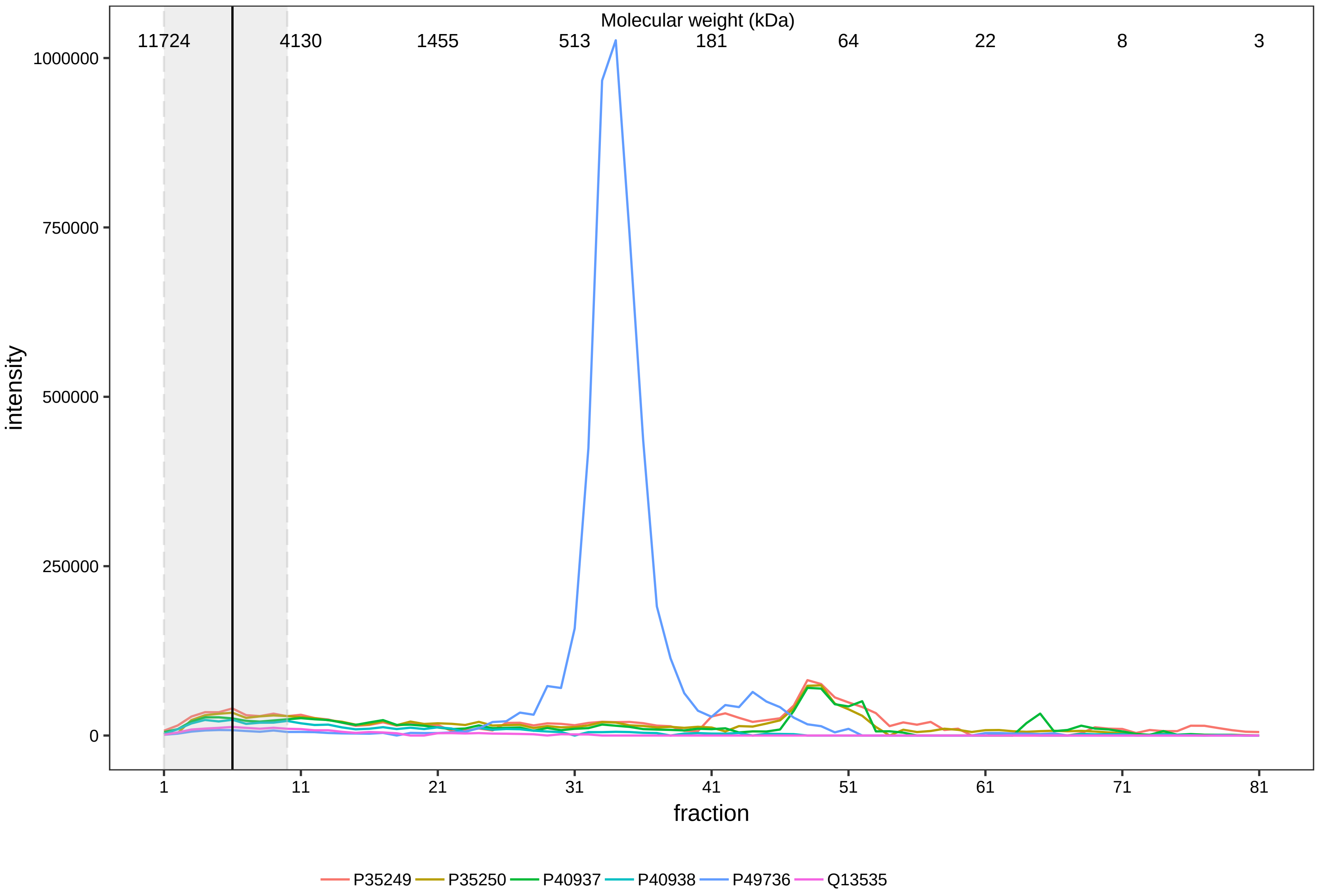

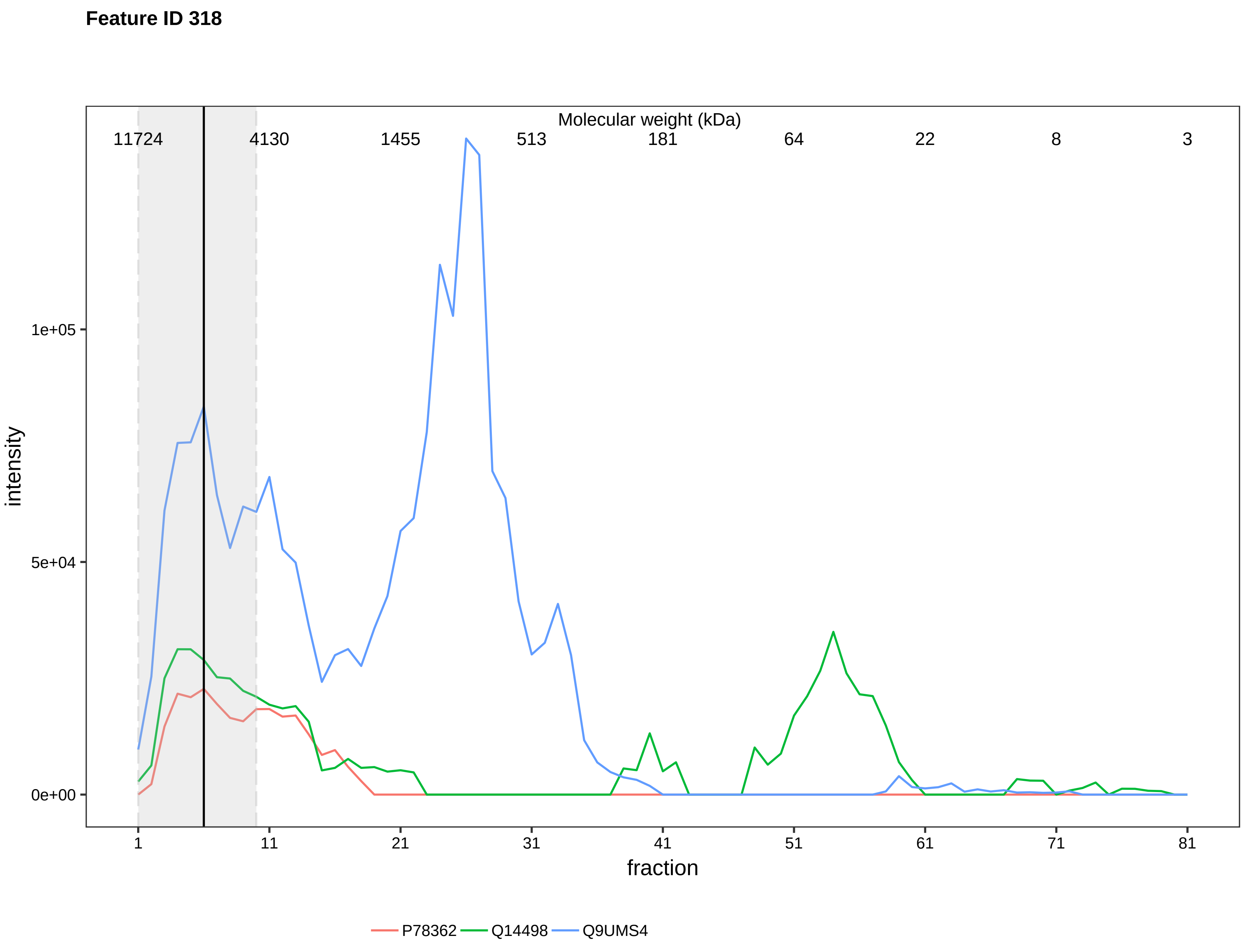

Feature ID 318
Molecular weight (kDa)
11724
4130
1455
513
181
64
22
8
3
intensity
1e+05
5e+04
0e+00
1
11
21
31
41
51
61
71
81
fraction
P78362
Q14498
Q9UMS4

Feature ID 319

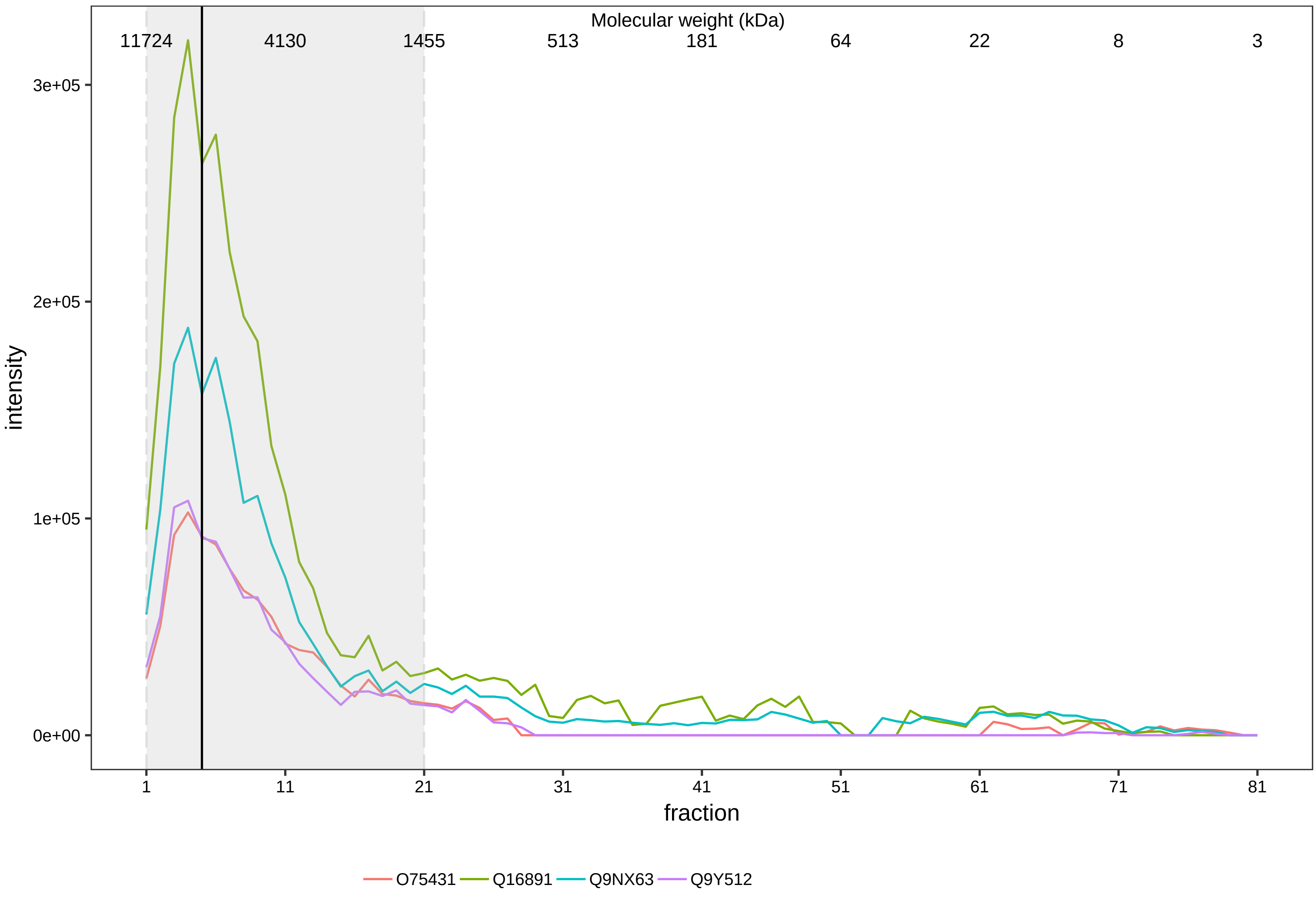

Feature ID 320

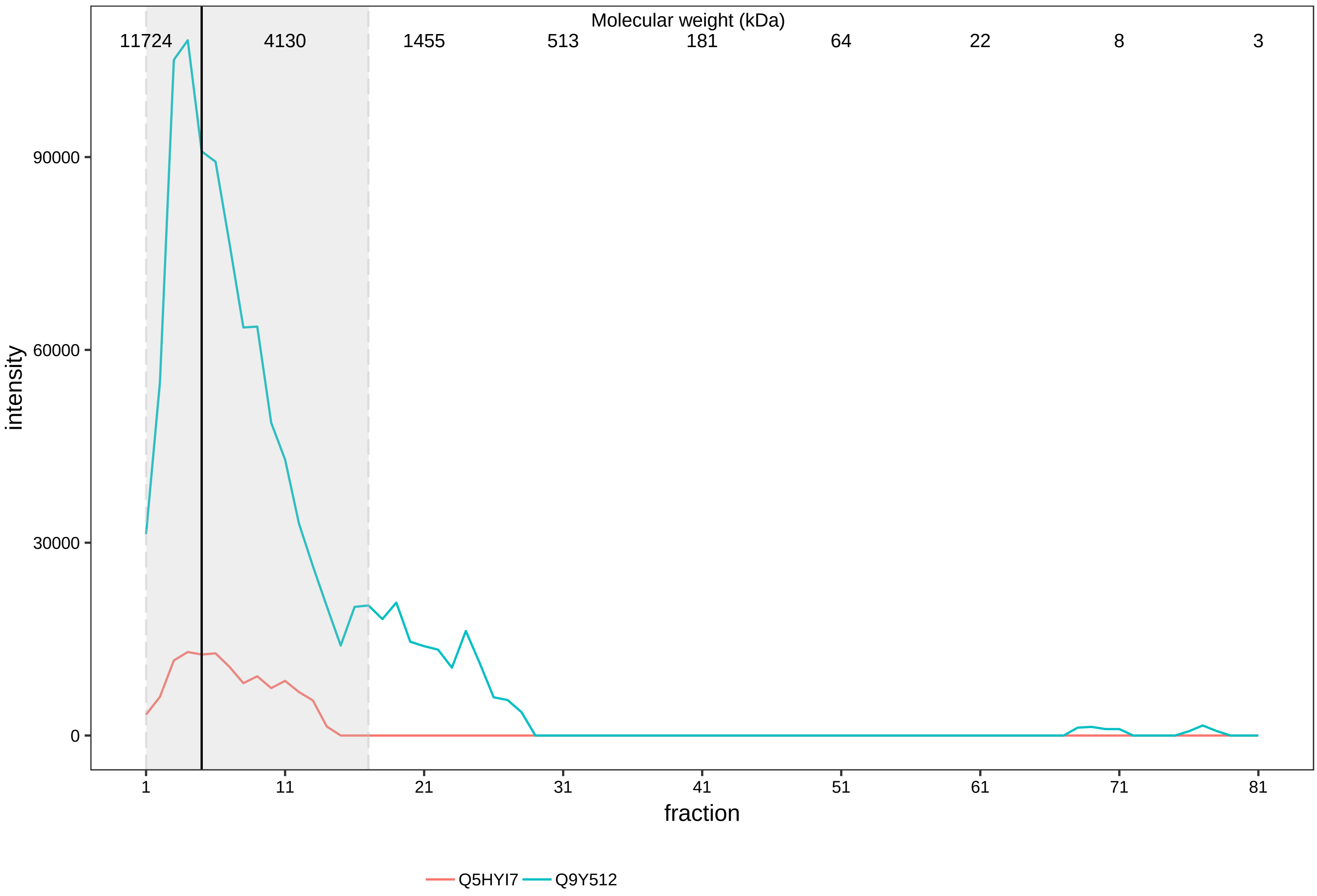

Feature ID 321

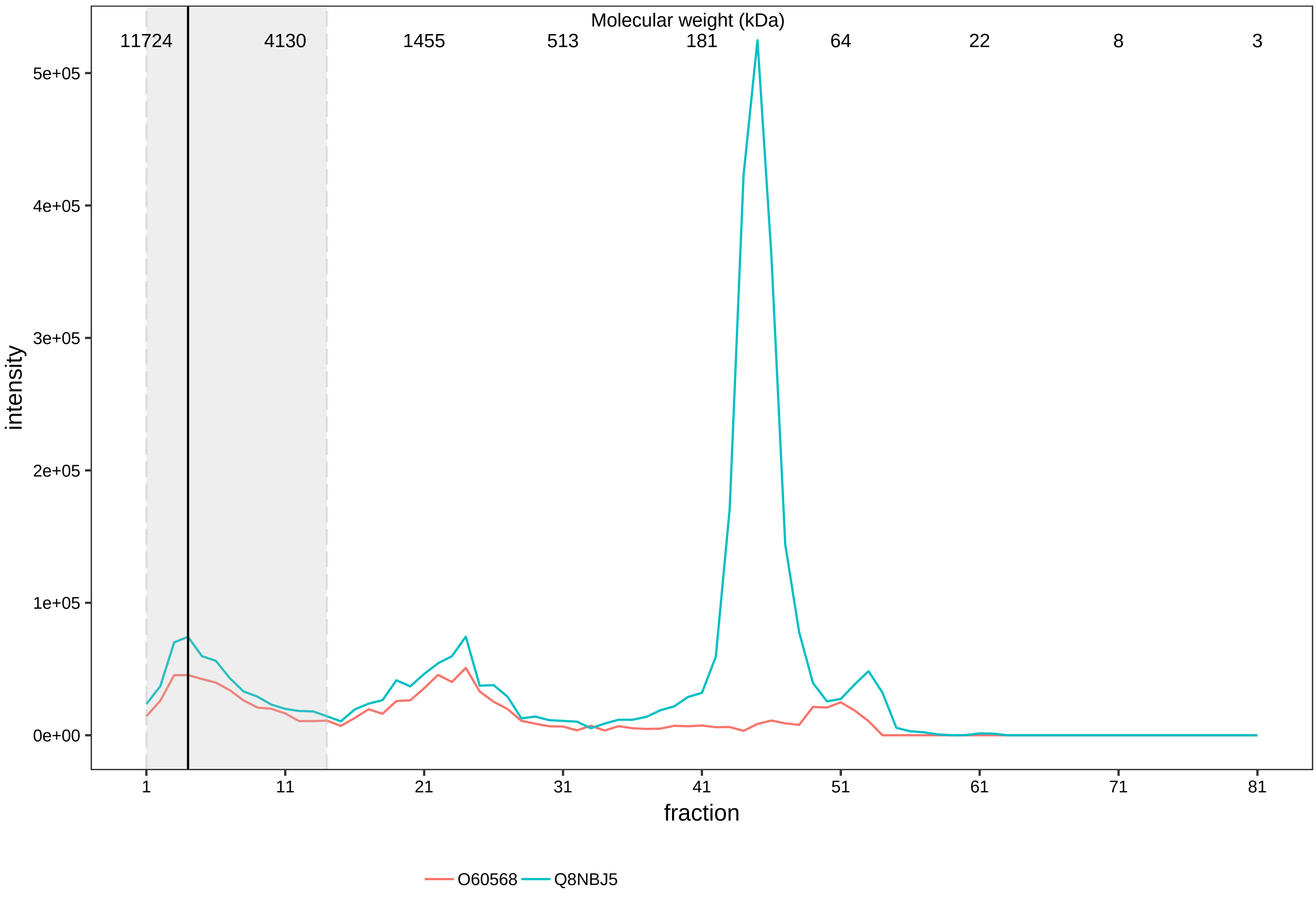

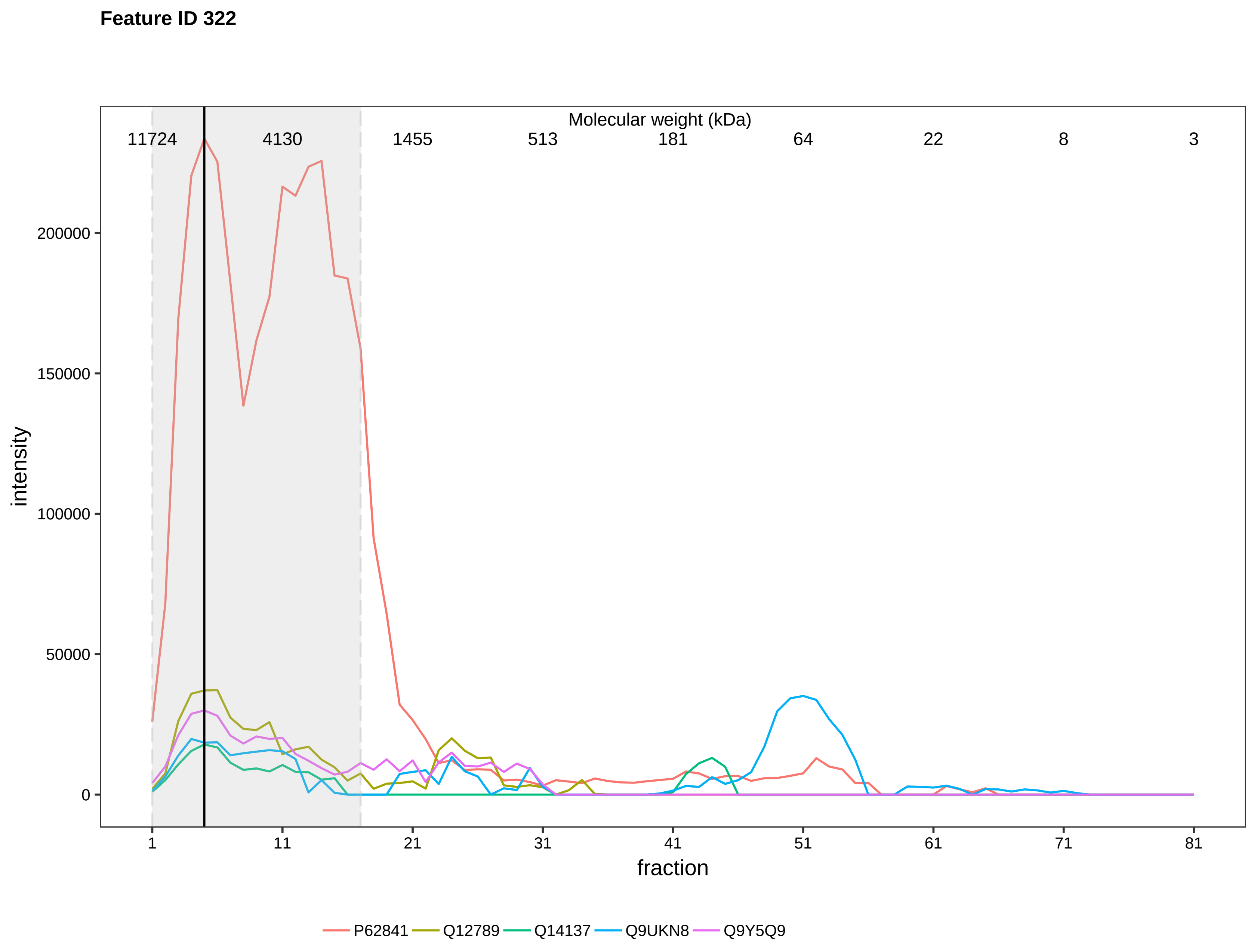
Feature ID 322
Molecular weight (kDa)
11724
4130
1455
513
181
64
22
8
3
intensity
200000
150000
100000
50000
0
1
11
21
31
41
51
61
71
81
fraction
P62841
Q12789
Q14137
Q9UKN8
Q9Y5Q9

Feature ID 323

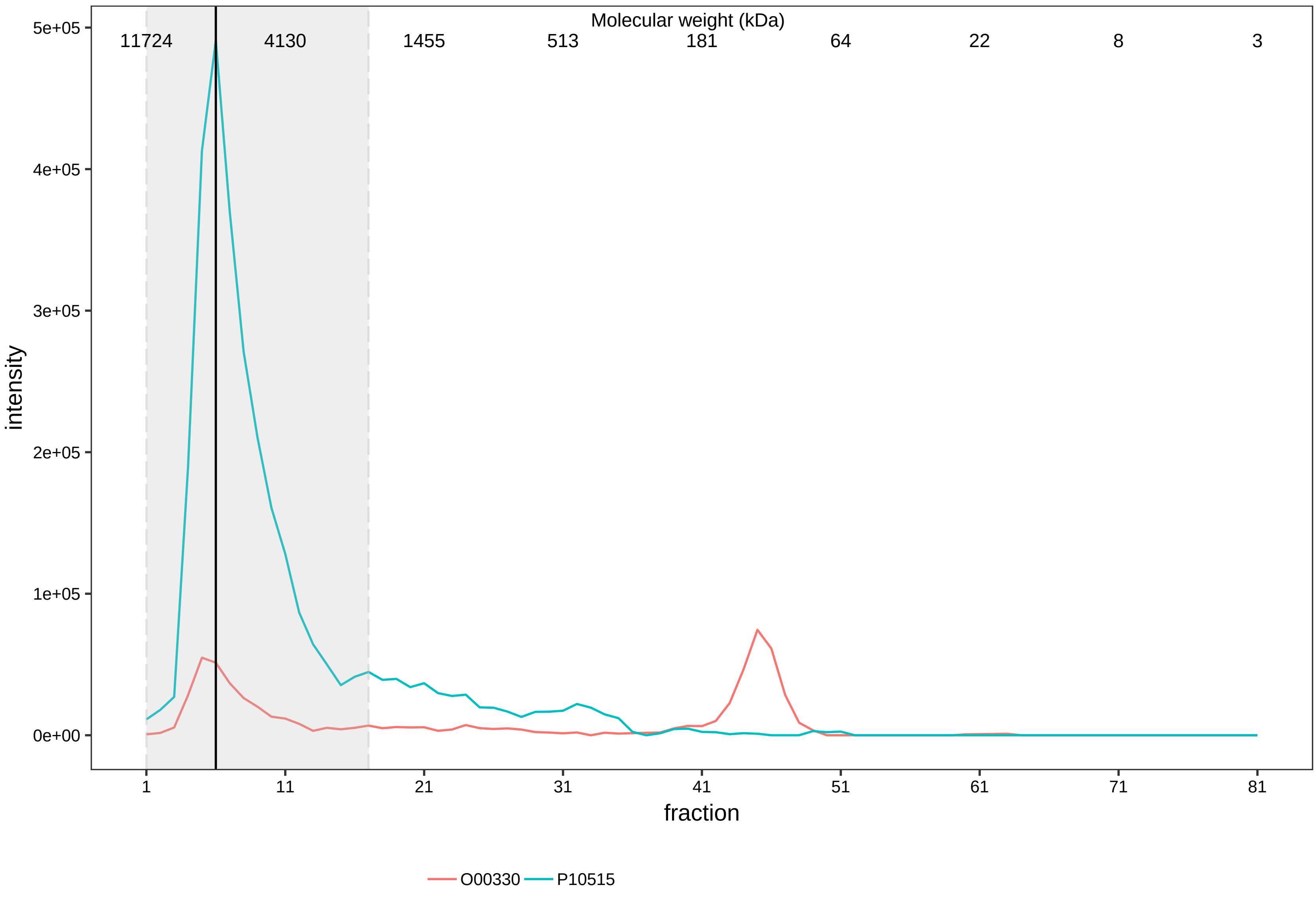

Feature ID 324

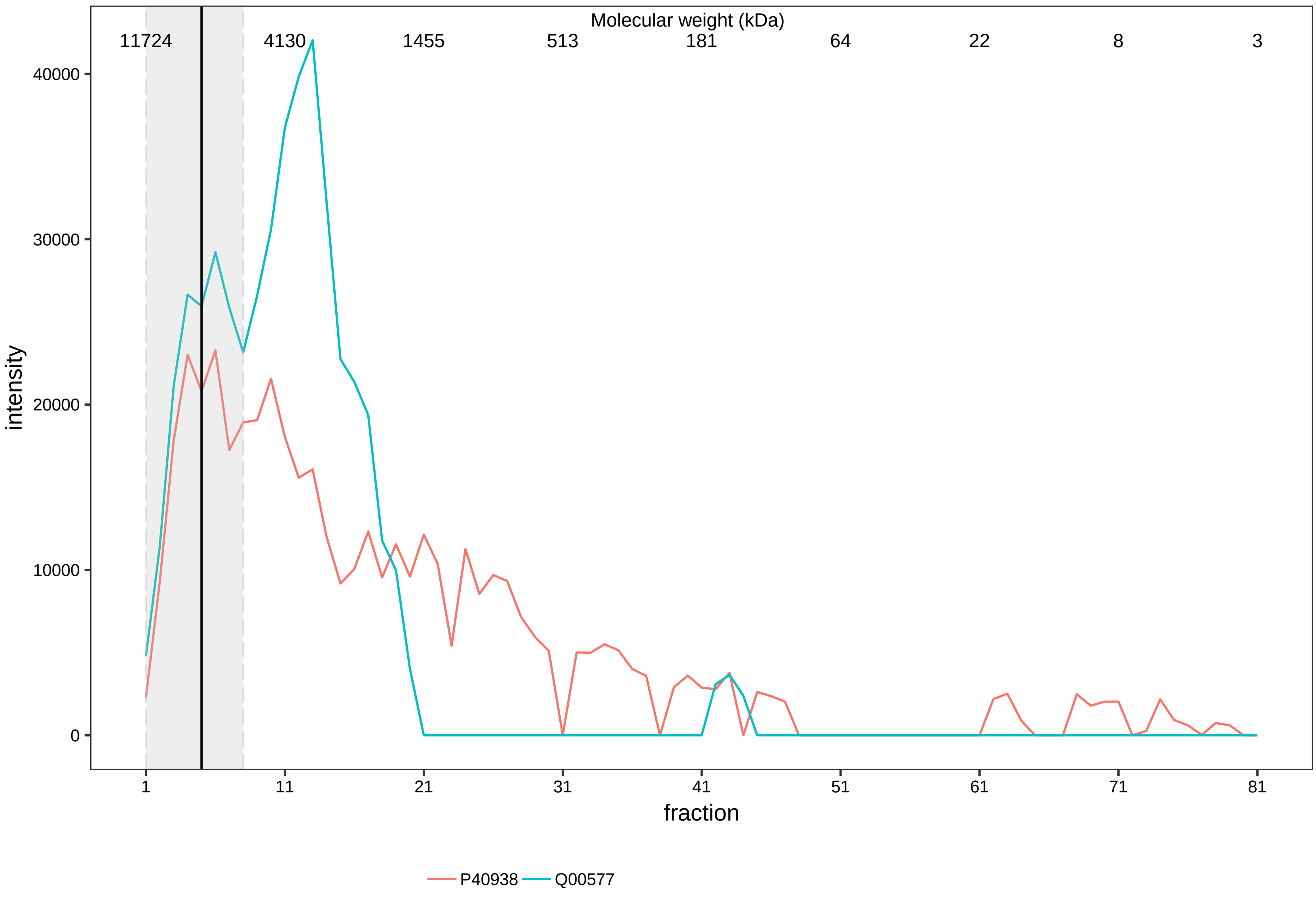

Feature ID 325

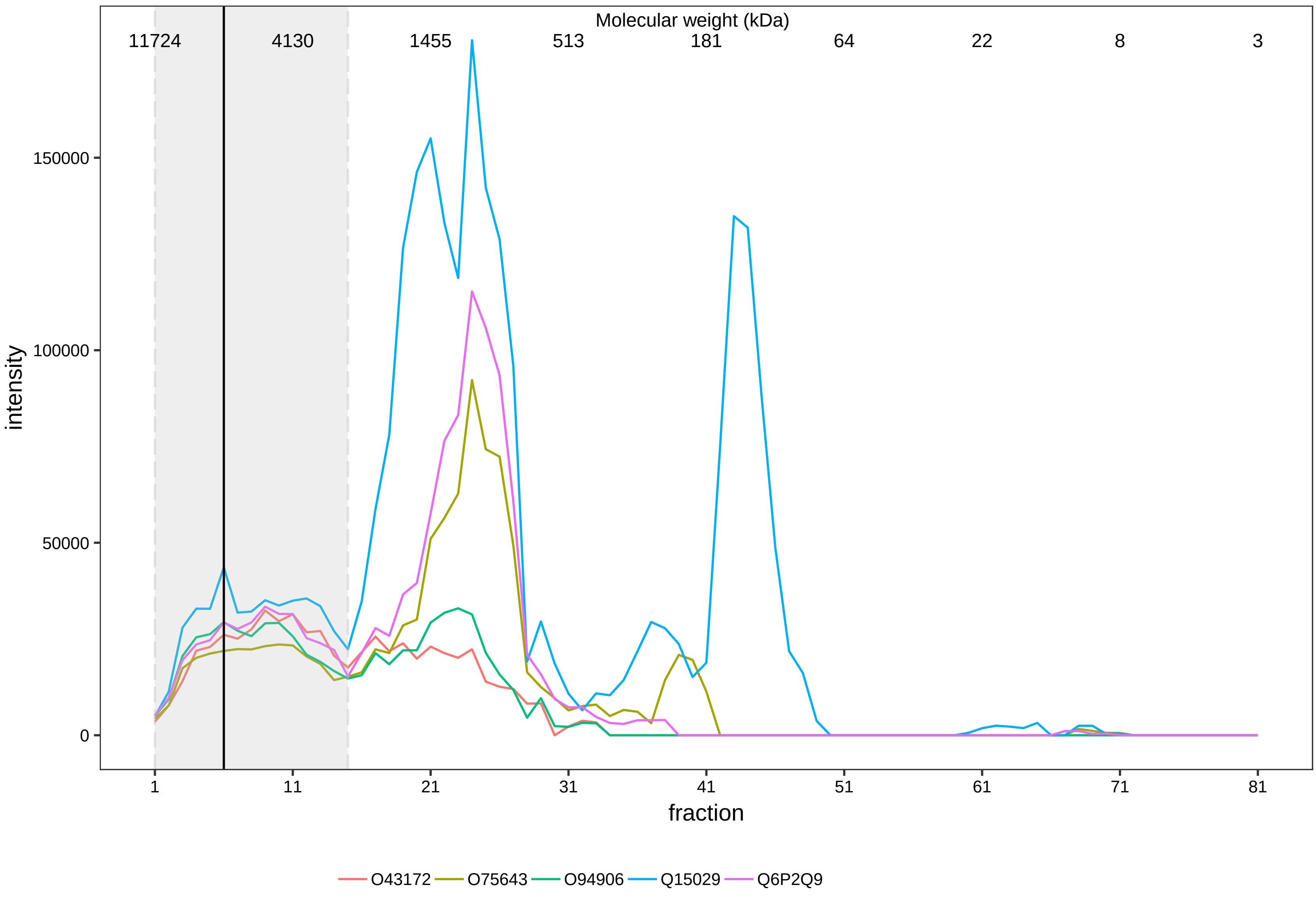

Feature ID 326

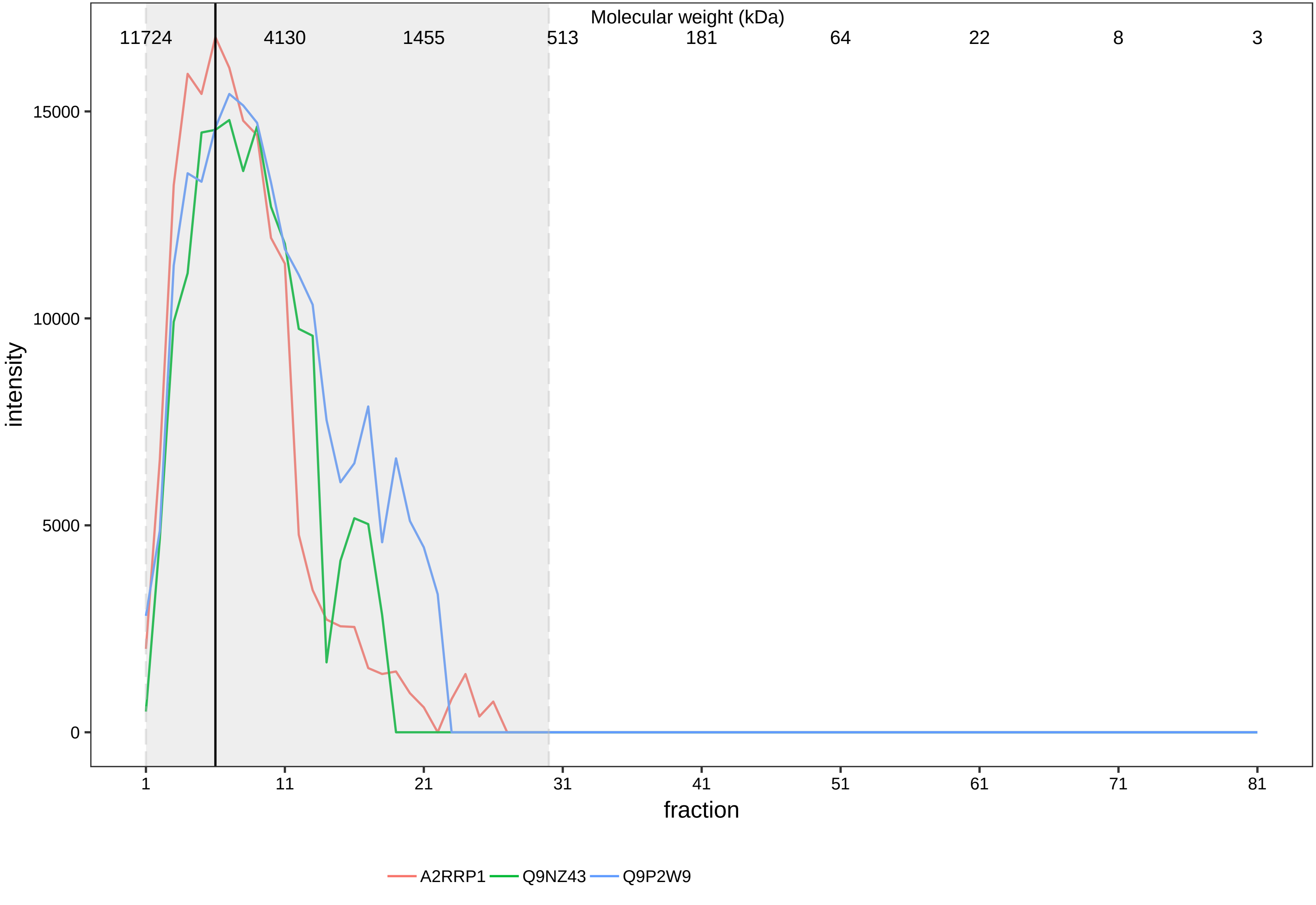

Feature ID 327

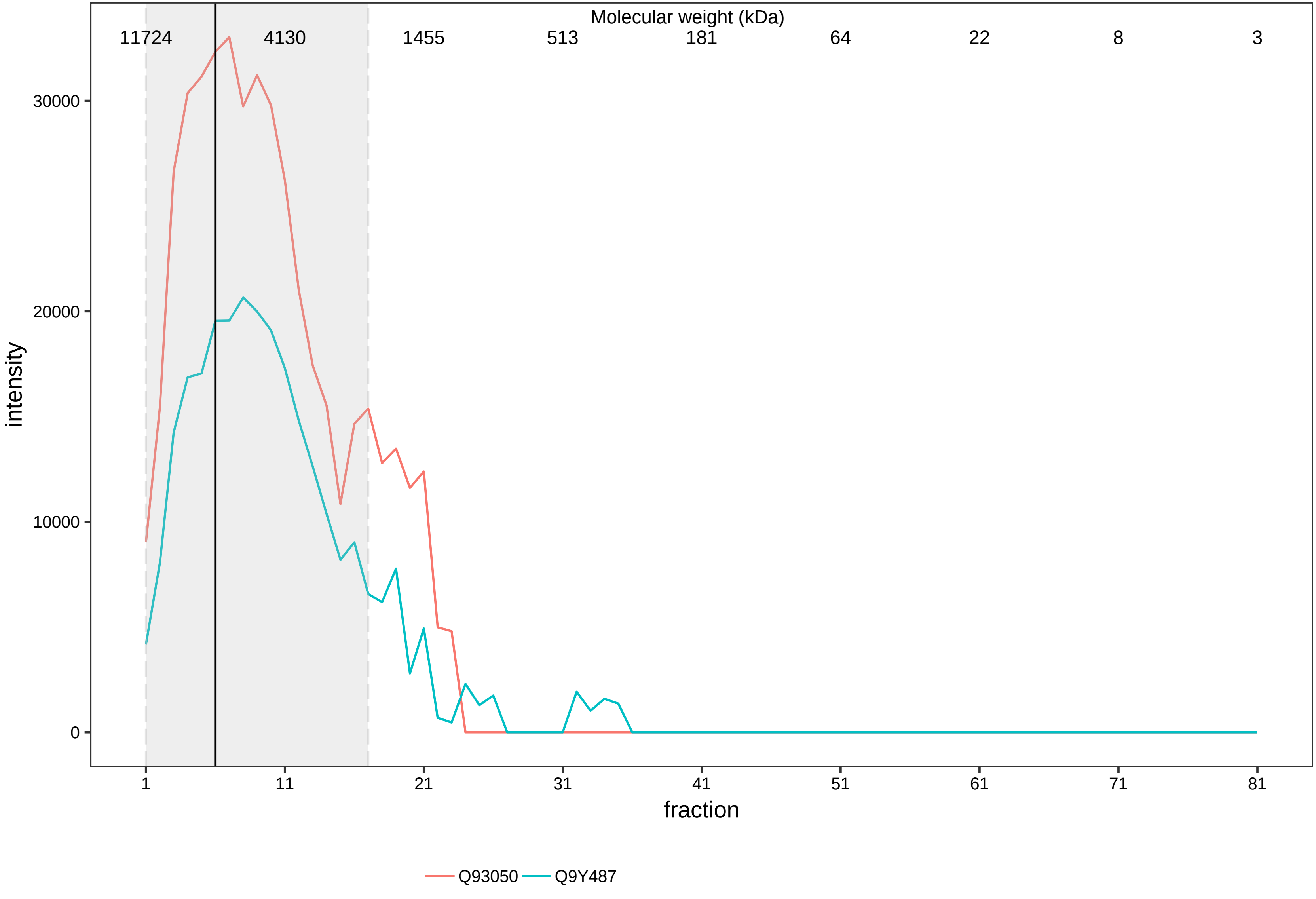

Feature ID 328

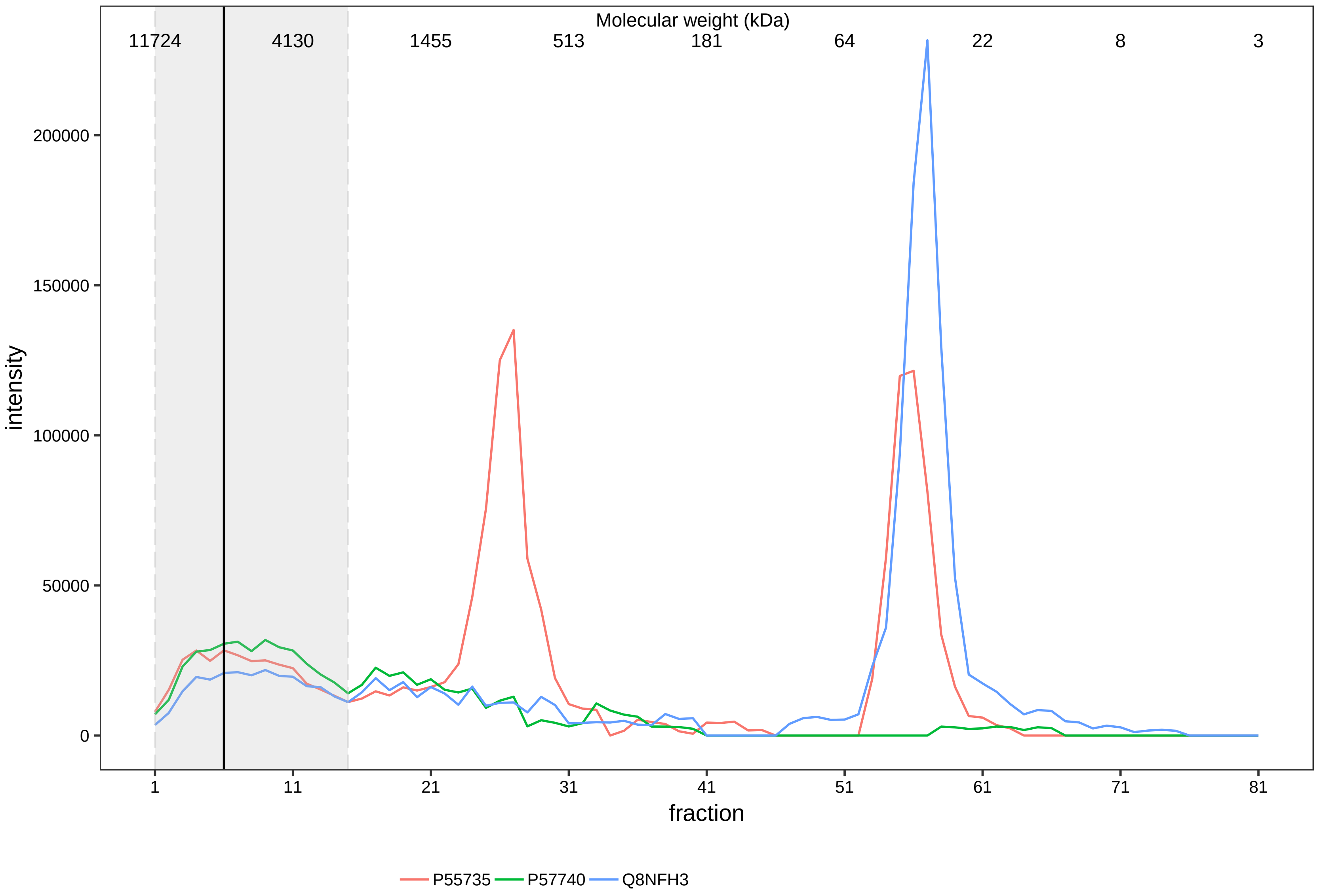

Feature ID 329

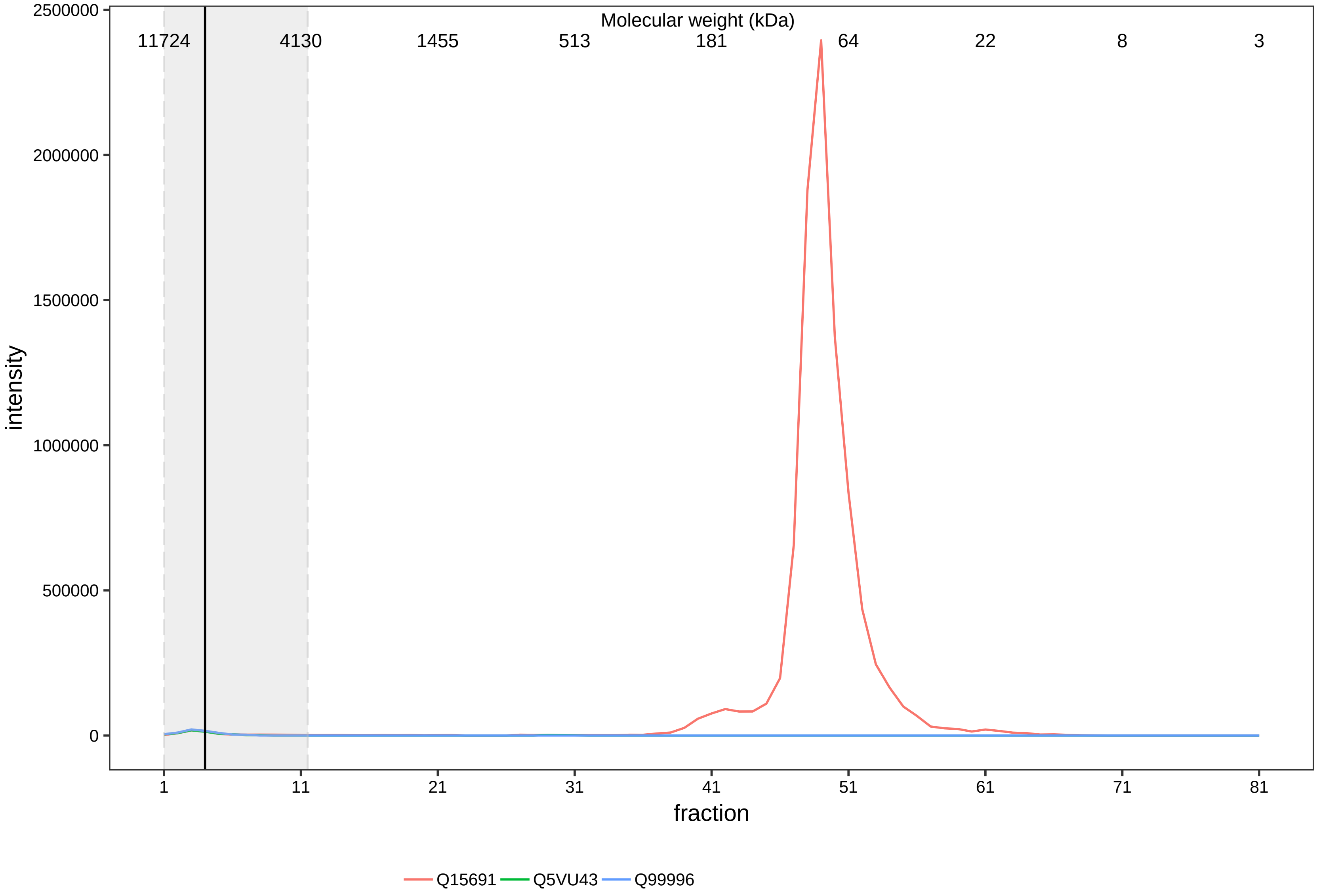

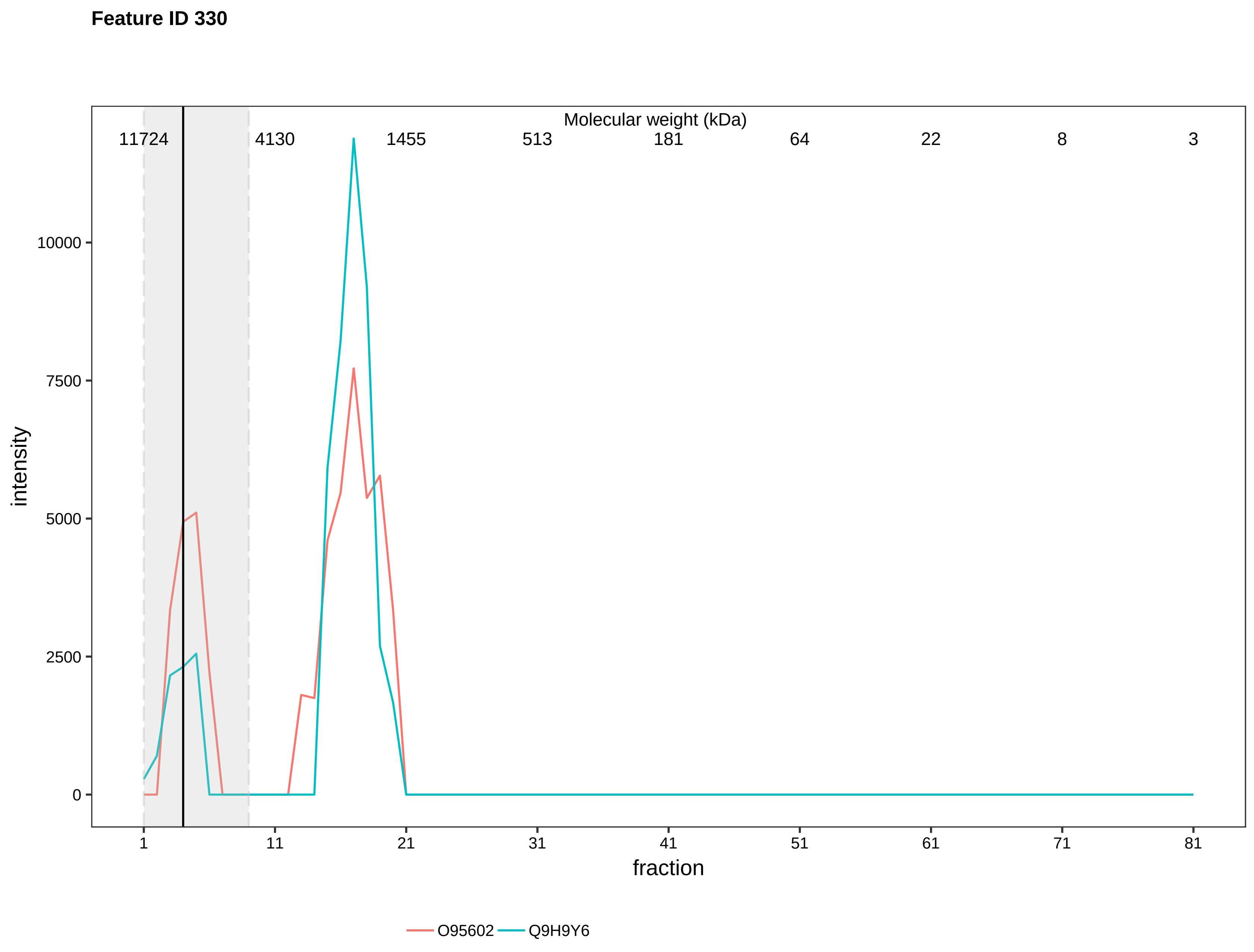

Feature ID 330
Molecular weight (kDa)
11724
4130
1455
513
181
64
22
8
3
10000
7500
5000
2500
0
intensity
1
11
21
31
41
51
61
71
81
fraction
O95602
Q9H9Y6

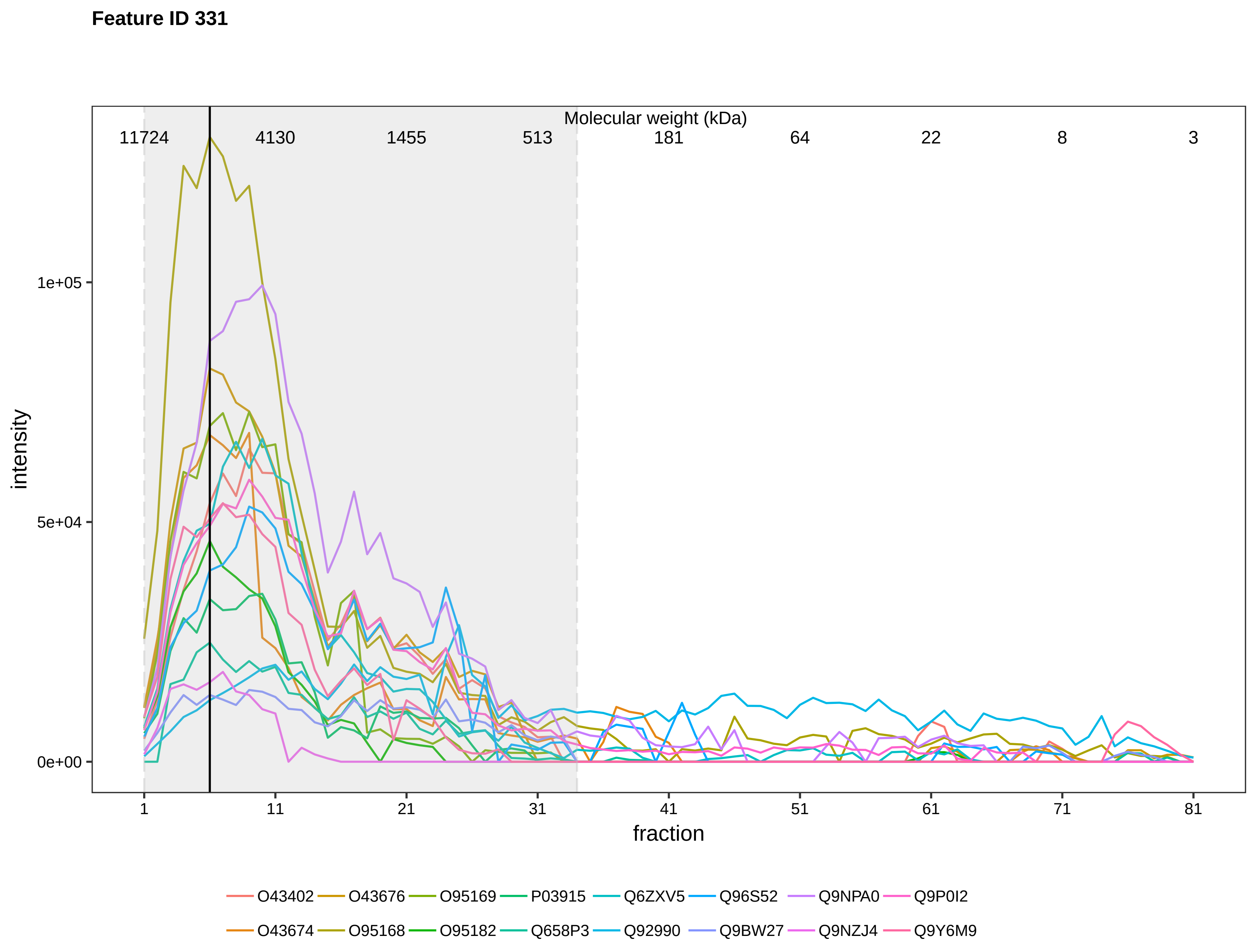

Feature ID 331
Molecular weight (kDa)
11724
4130
1455
513
181
64
22
8
3
1e+05
5e+04
0e+00
intensity
1
11
21
31
41
51
61
71
81
fraction
O43402
O43676
O95169
P03915
Q6ZXV5
Q96S52
Q9NPA0
Q9P0I2
O43674
O95168
O95182
Q658P3
Q92990
Q9BW27
Q9NZJ4
Q9Y6M9

Feature ID 332

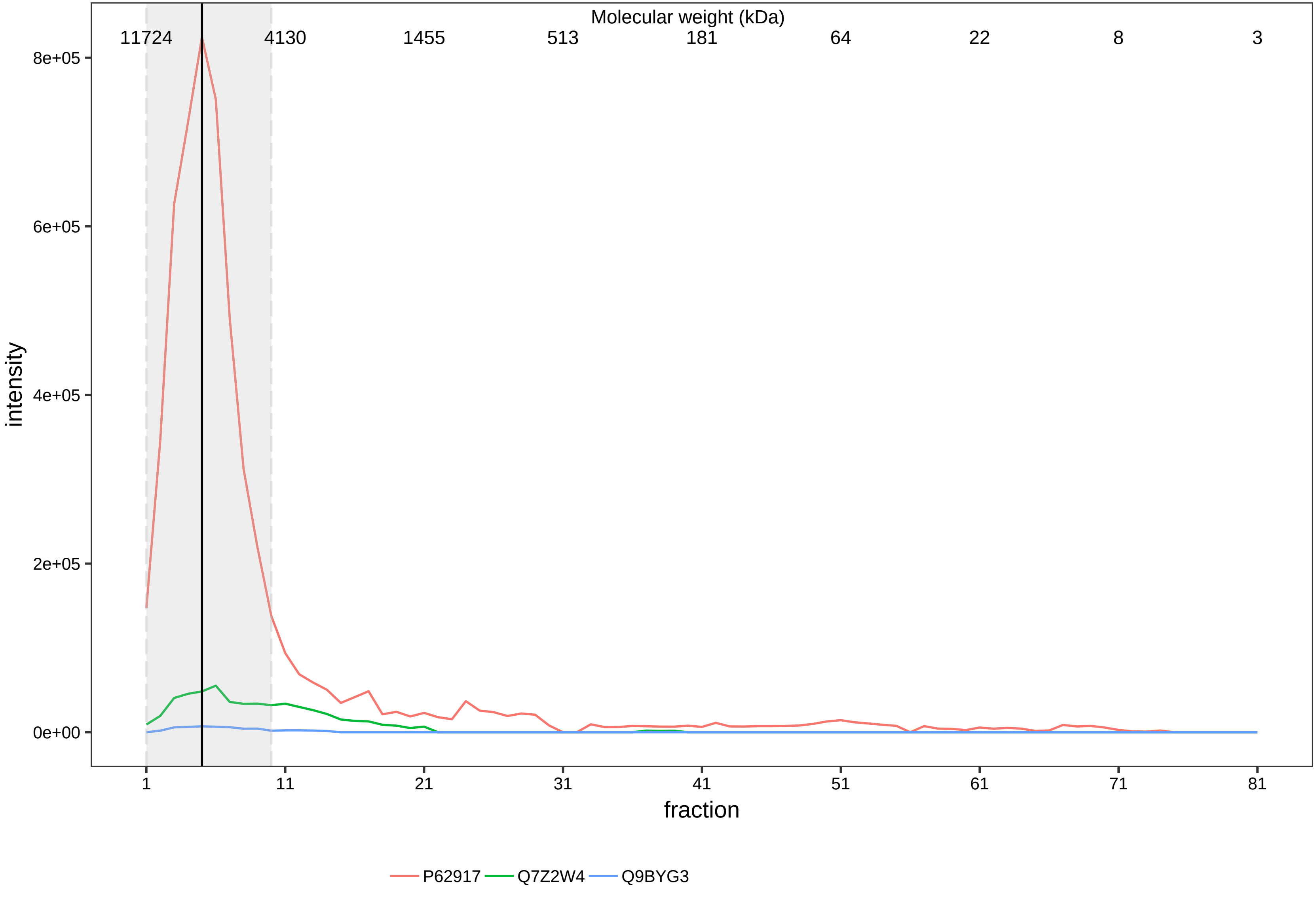

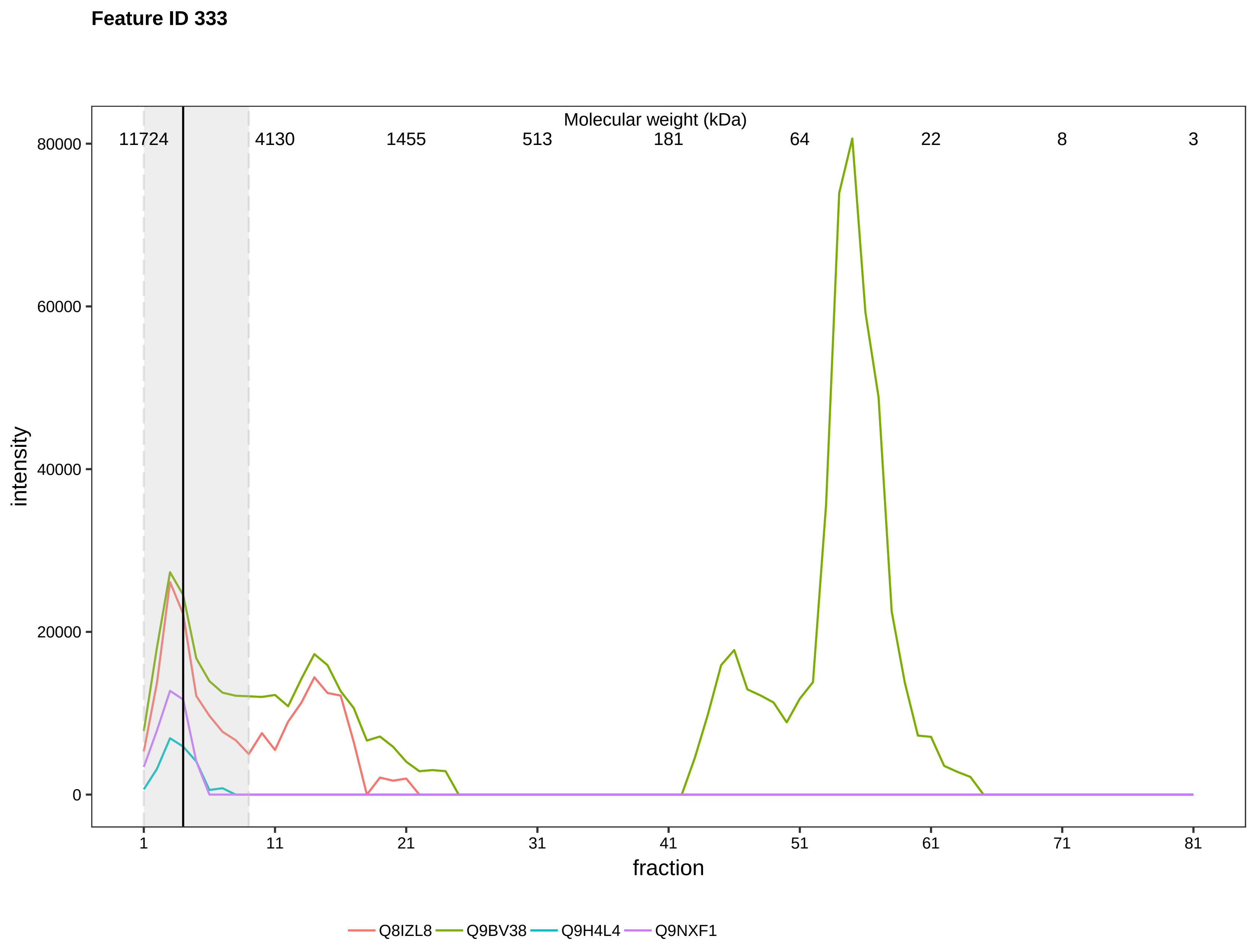

Feature ID 333
Molecular weight (kDa)
11724
4130
1455
513
181
64
22
8
3
80000
60000
40000
20000
0
intensity
1
11
21
31
41
51
61
71
81
fraction
Q8IZL8
Q9BV38
Q9H4L4
Q9NXF1

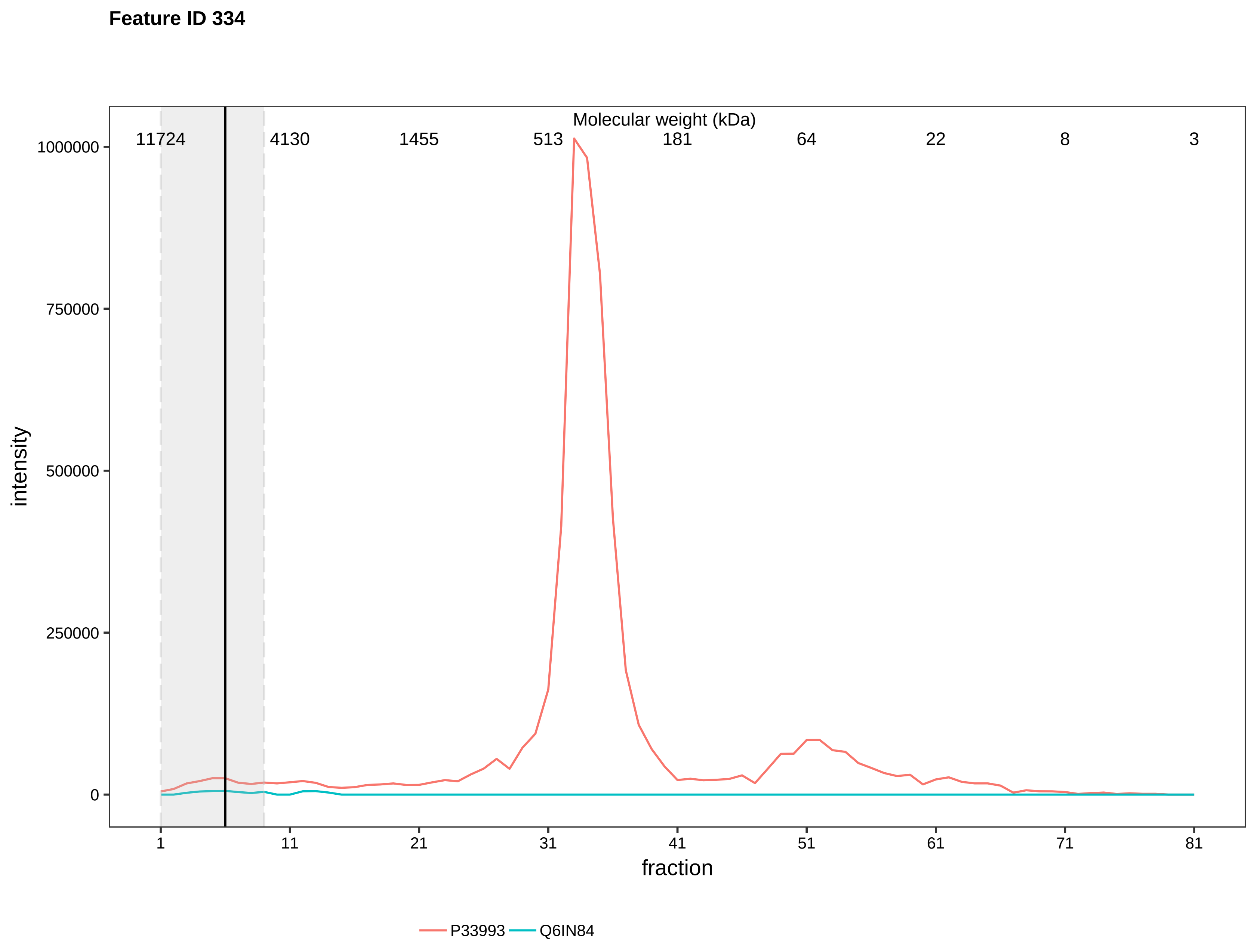

Feature ID 334
Molecular weight (kDa)
11724
4130
1455
513
181
64
22
8
3
1000000
750000
500000
250000
0
intensity
1
11
21
31
41
51
61
71
81
fraction
P33993
Q6IN84

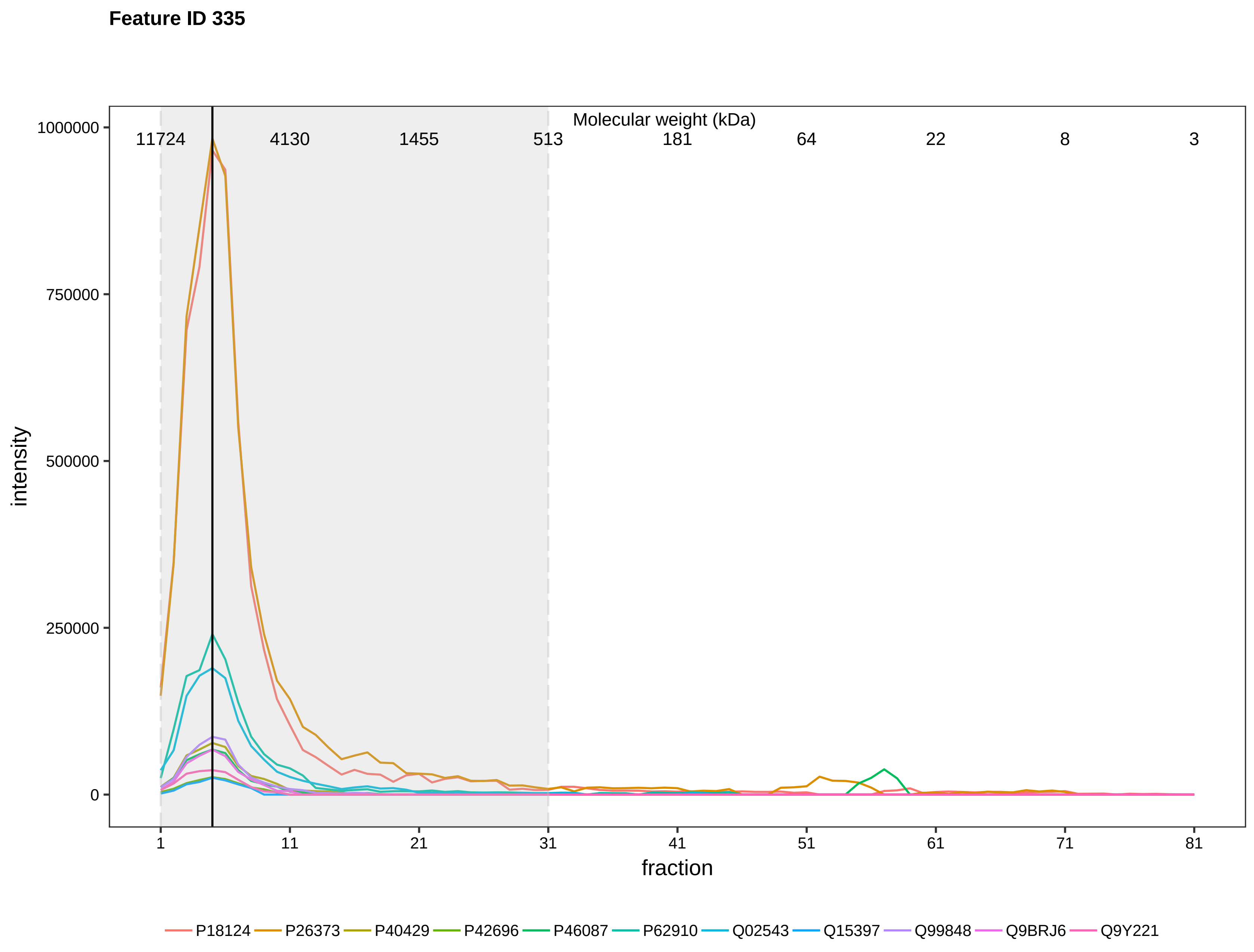
Feature ID 335
Molecular weight (kDa)
11724
4130
1455
513
181
64
22
8
3
1000000
750000
500000
250000
0
intensity
1
11
21
31
41
51
61
71
81
fraction
P18124
P26373
P40429
P42696
P46087
P62910
Q02543
Q15397
Q99848
Q9BRJ6
Q9Y221

Feature ID 336

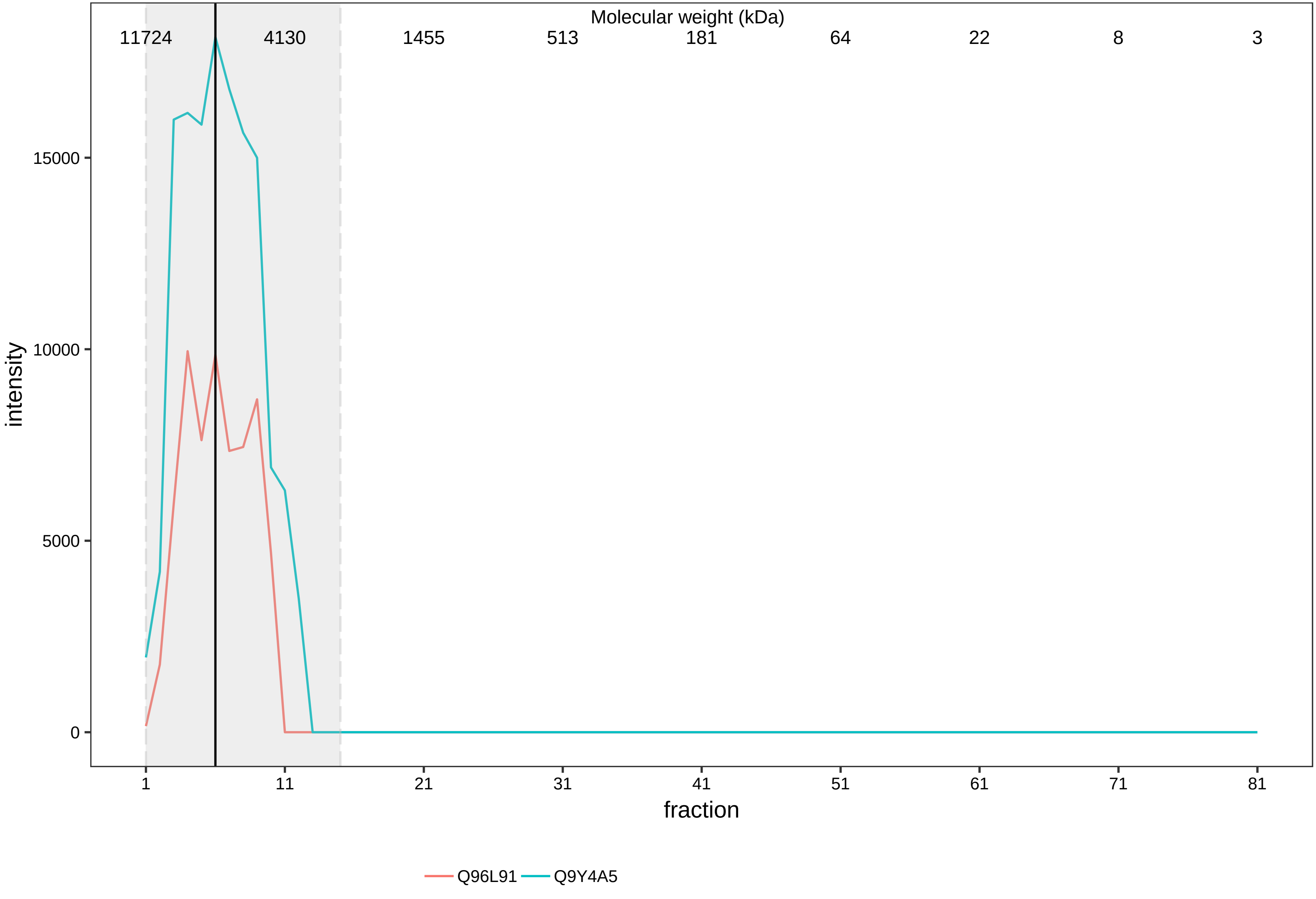

Feature ID 337

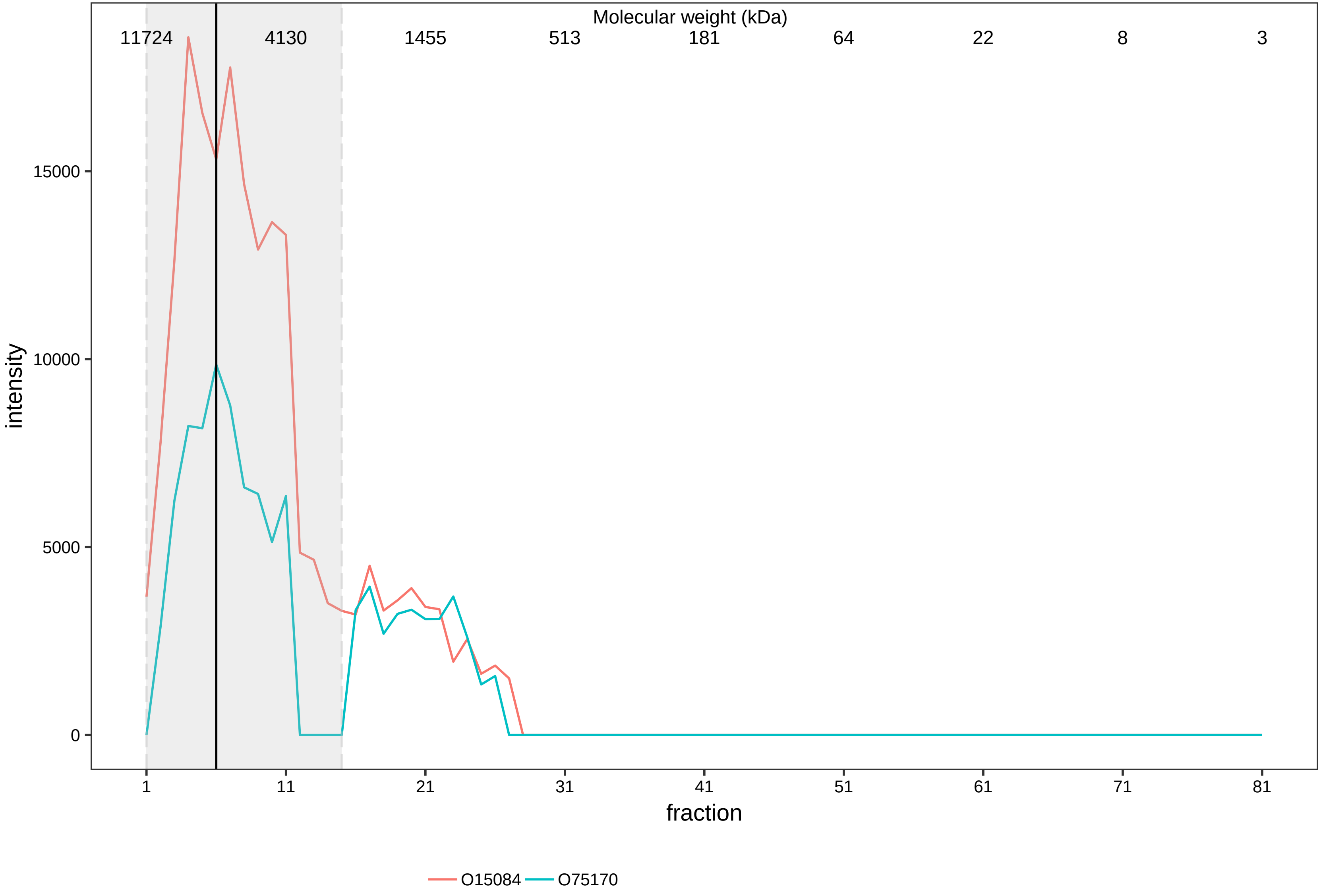

Feature ID 338

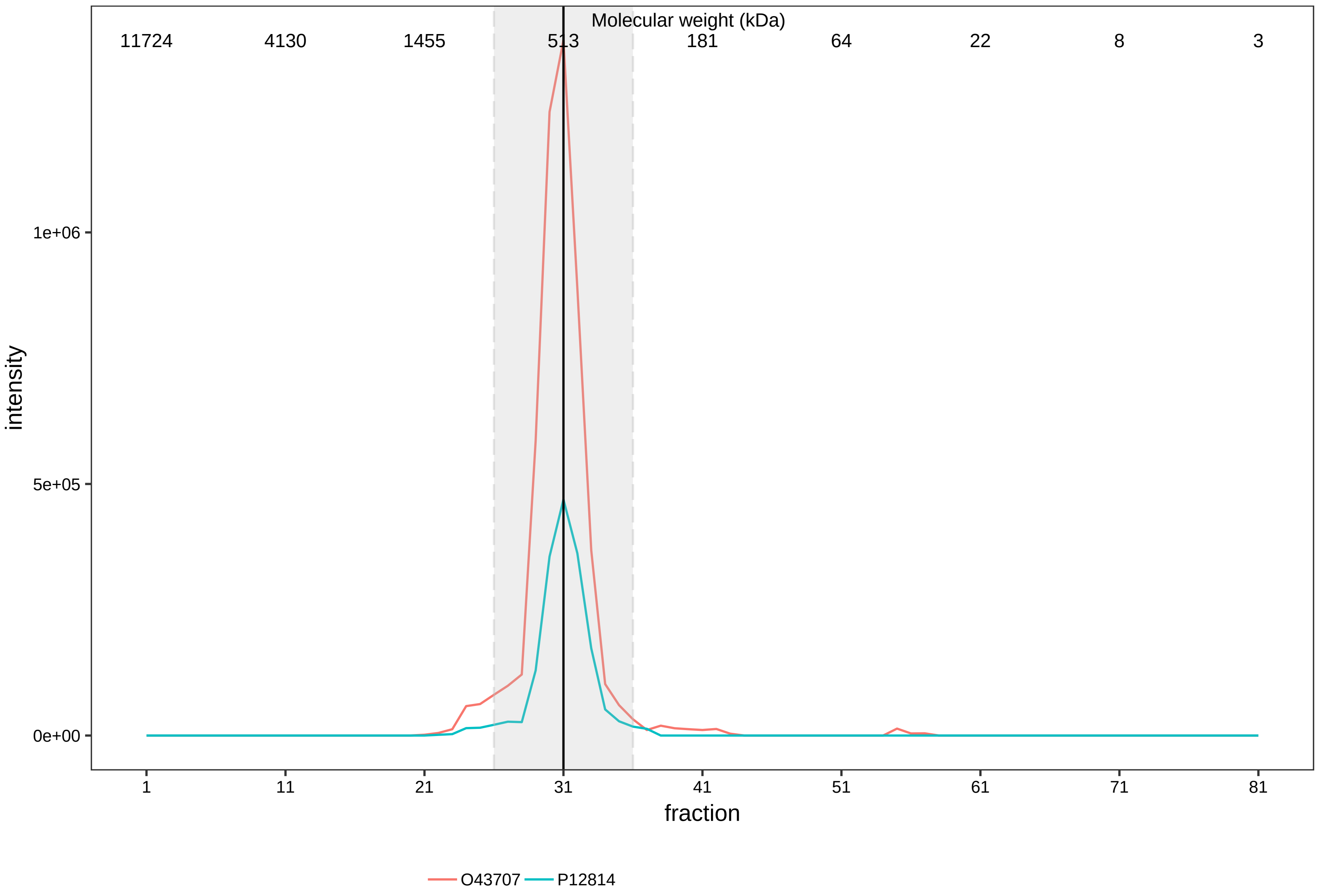

Feature ID 339

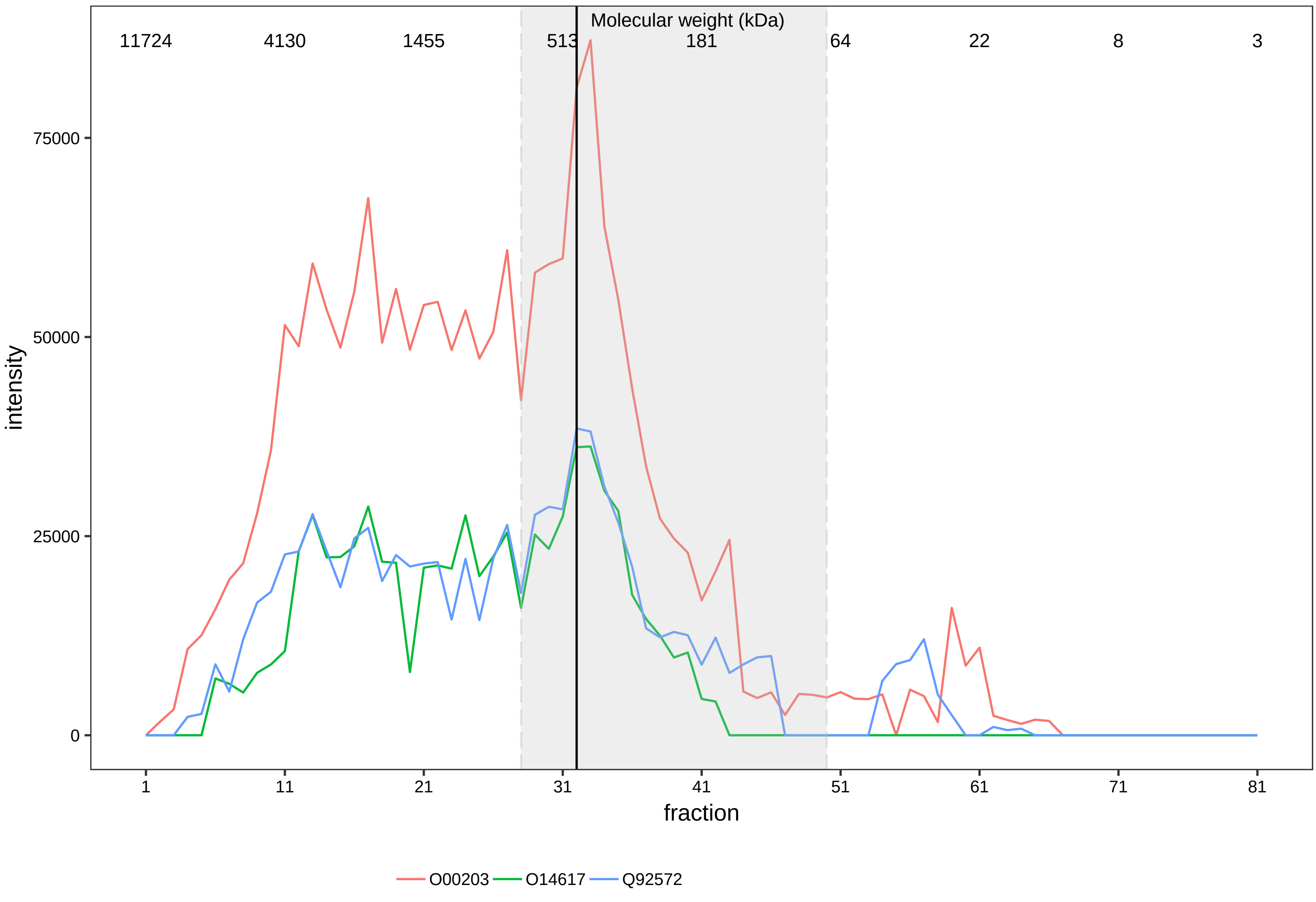

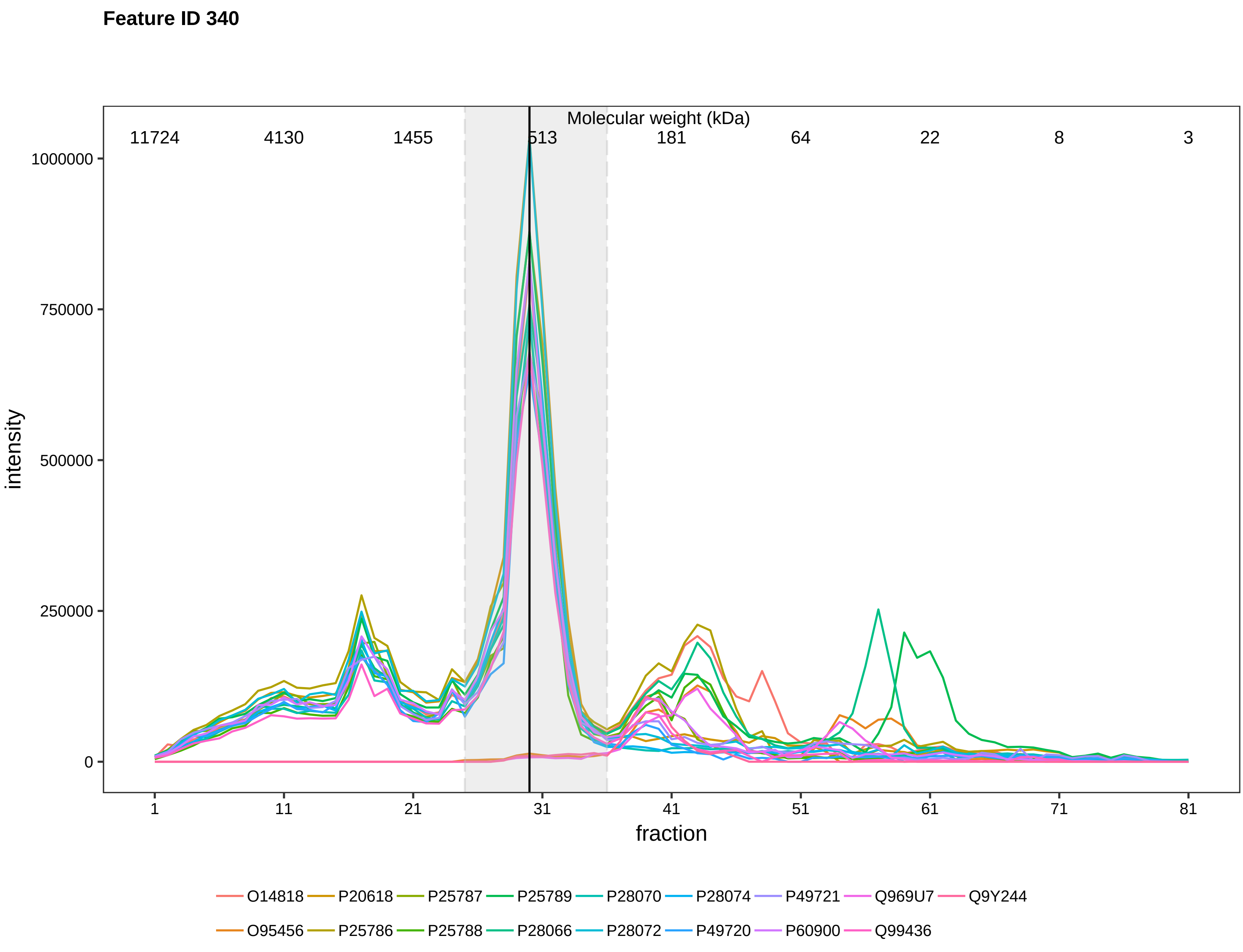

Feature ID 340
Molecular weight (kDa)
11724
4130
1455
513
181
64
22
8
3
1000000
750000
500000
250000
0
intensity
1
11
21
31
41
51
61
71
81
fraction
O14818
P20618
P25787
P25789
P28070
P28074
P49721
Q969U7
Q9Y244
O95456
P25786
P25788
P28066
P28072
P49720
P60900
Q99436

Feature ID 341

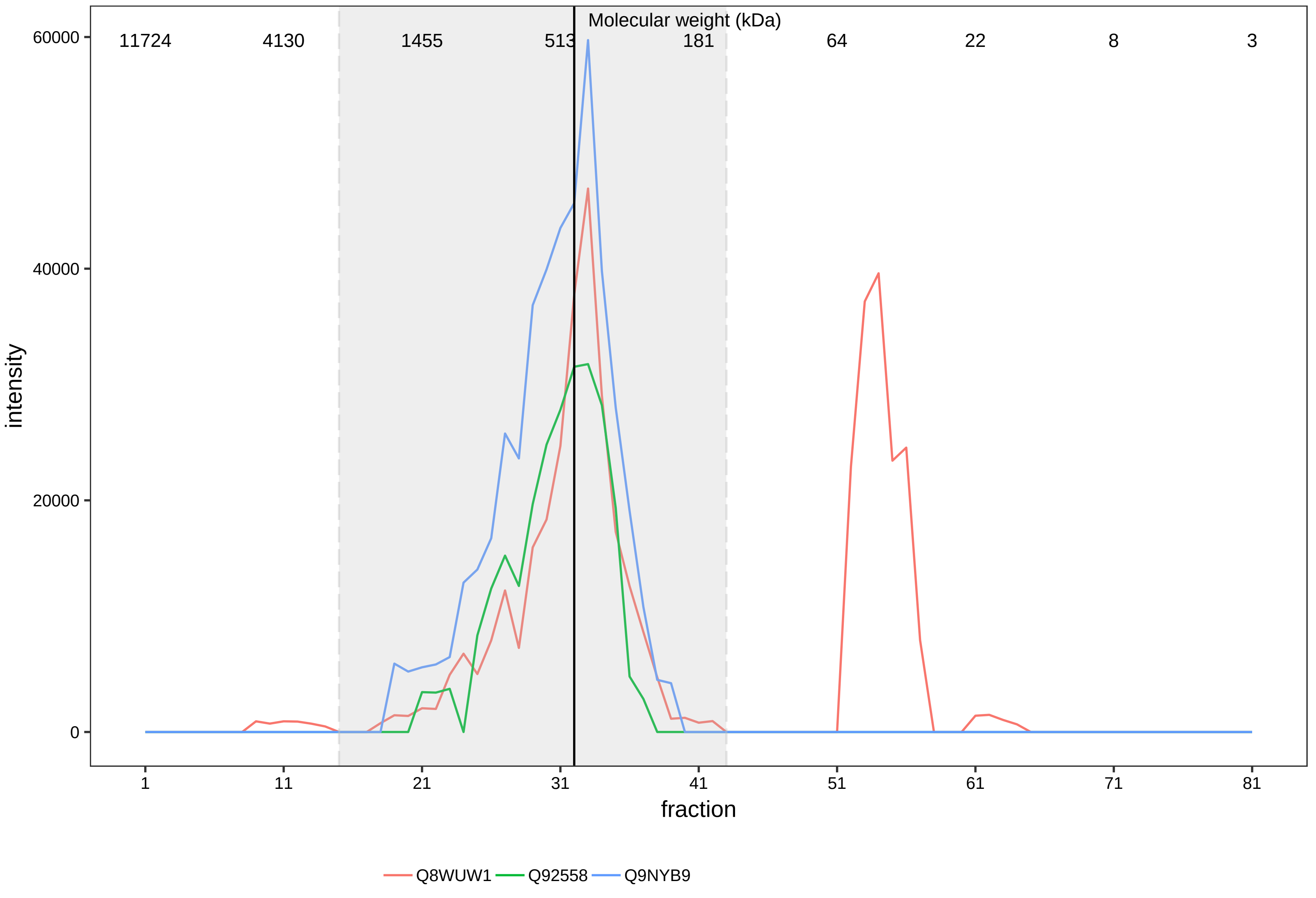

Feature ID 342

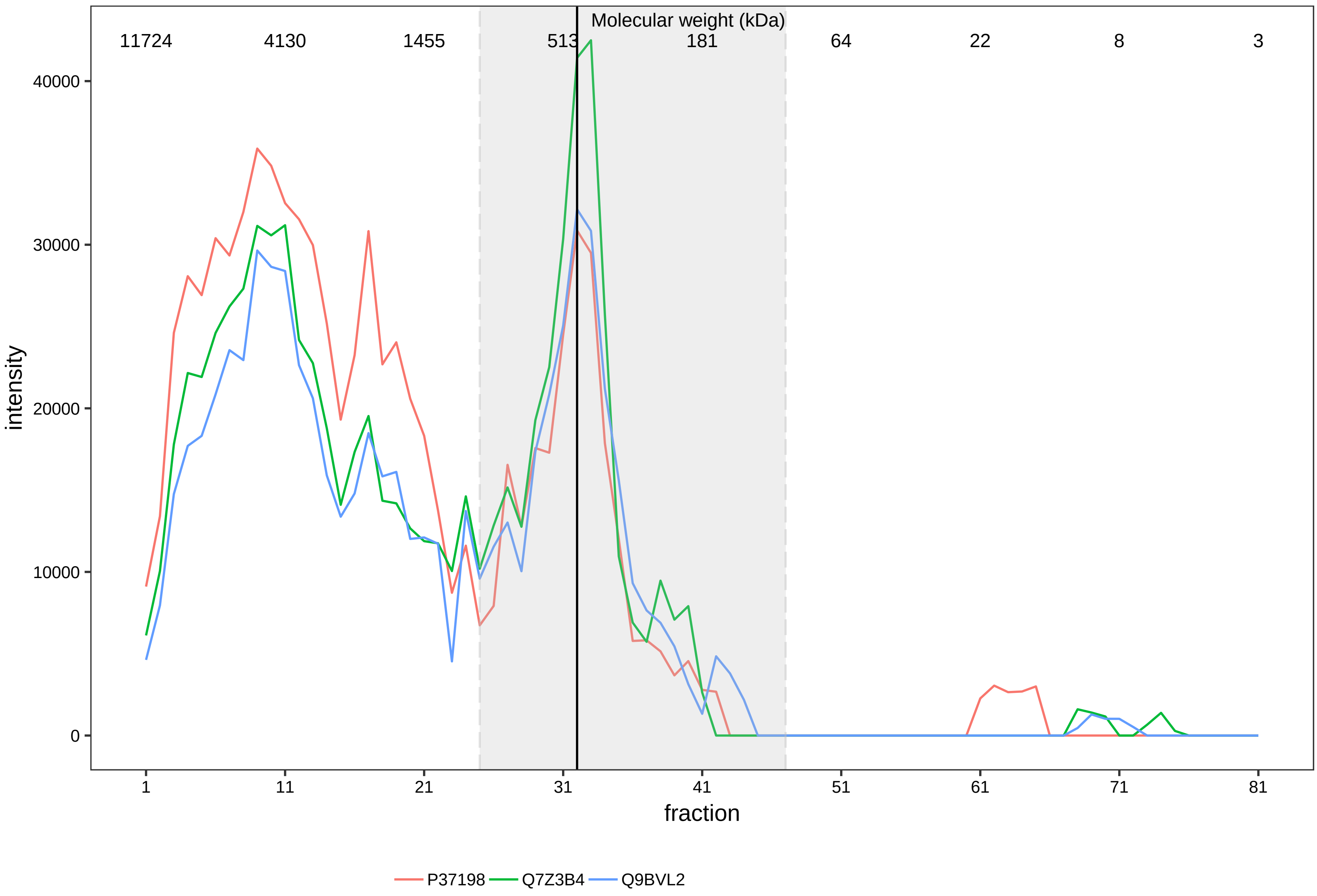

Feature ID 343

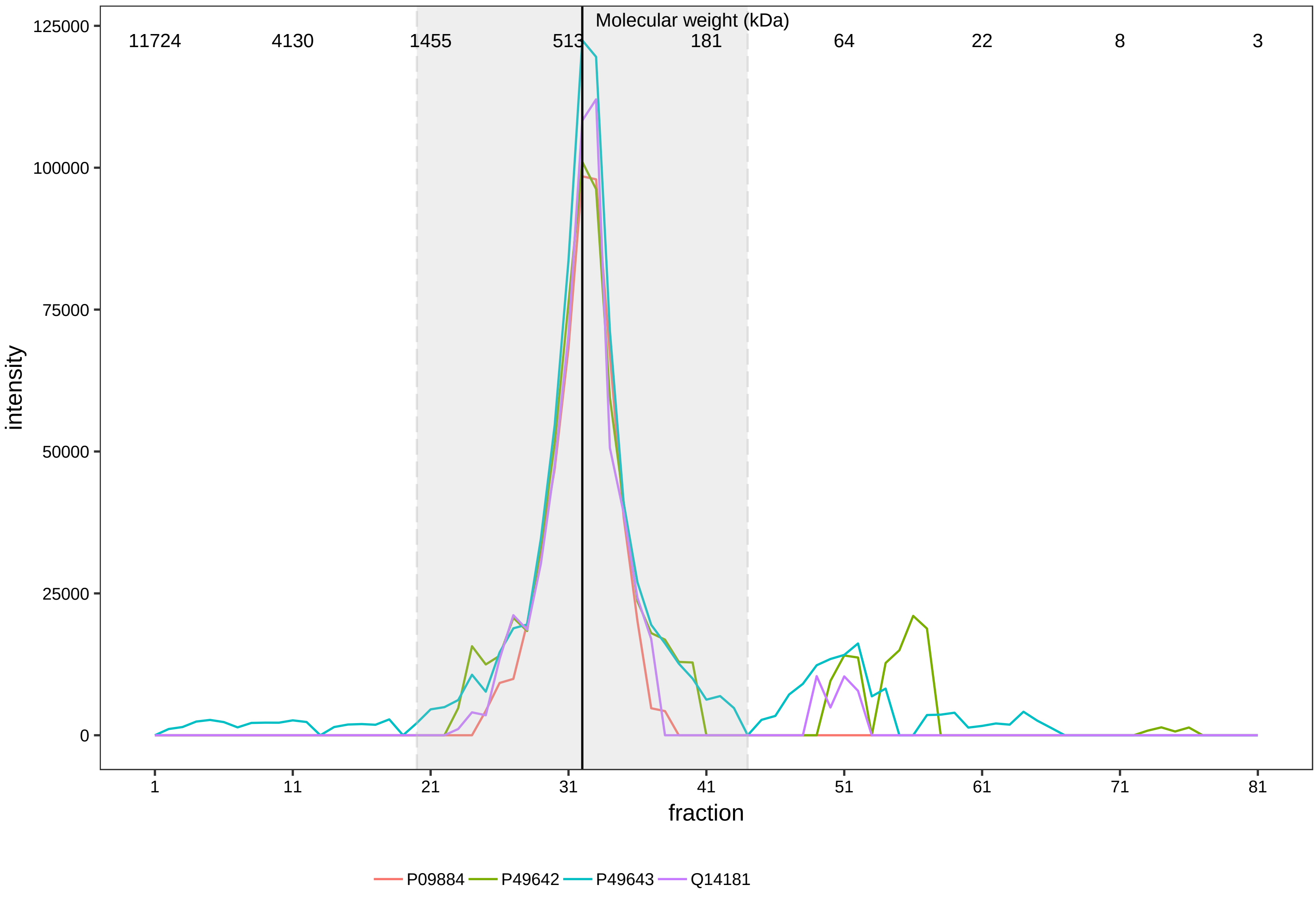

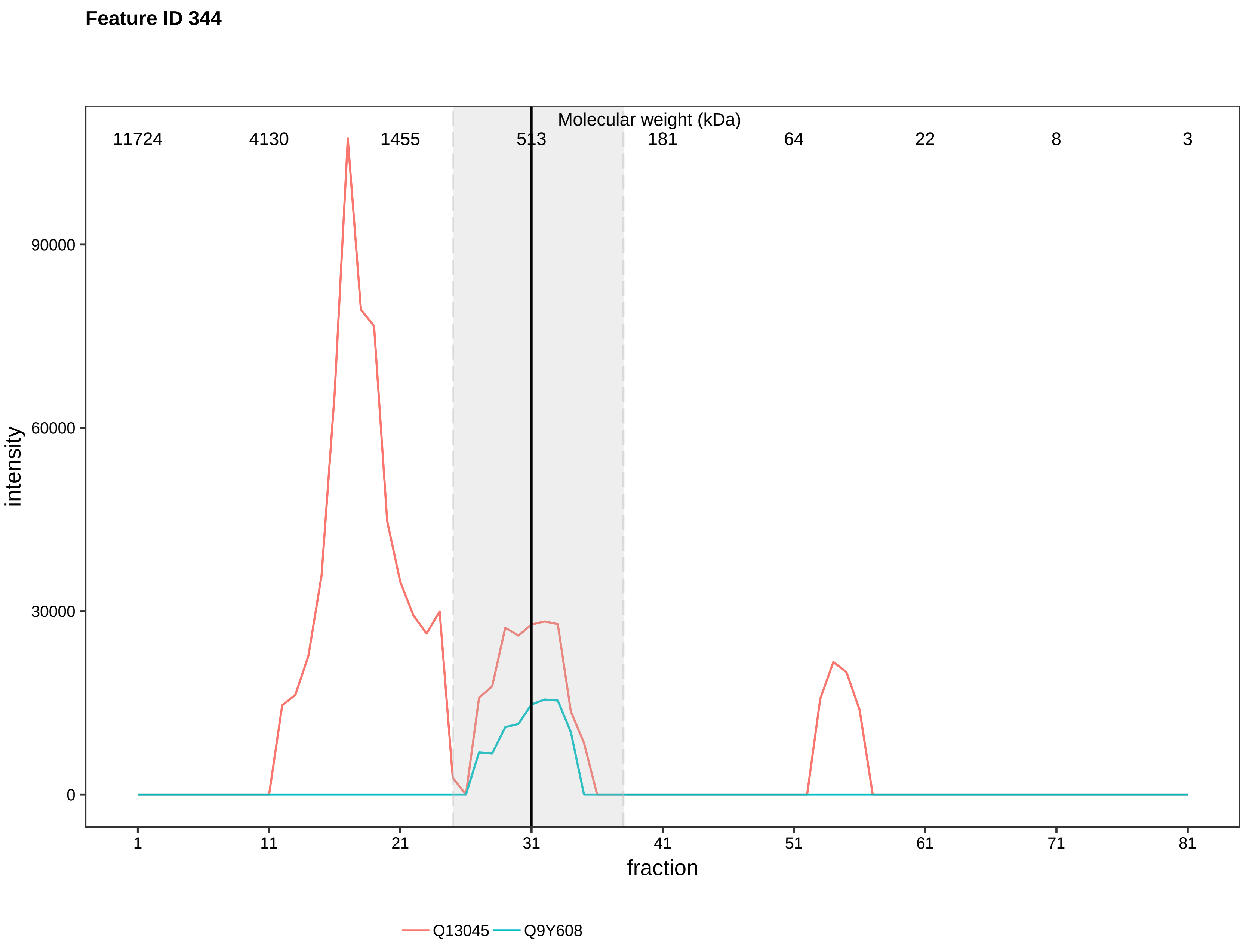

Feature ID 344
Molecular weight (kDa)
11724
4130
1455
513
181
64
22
8
3
90000
60000
30000
0
intensity
1
11
21
31
41
51
61
71
81
fraction
Q13045
Q9Y608

Feature ID 345

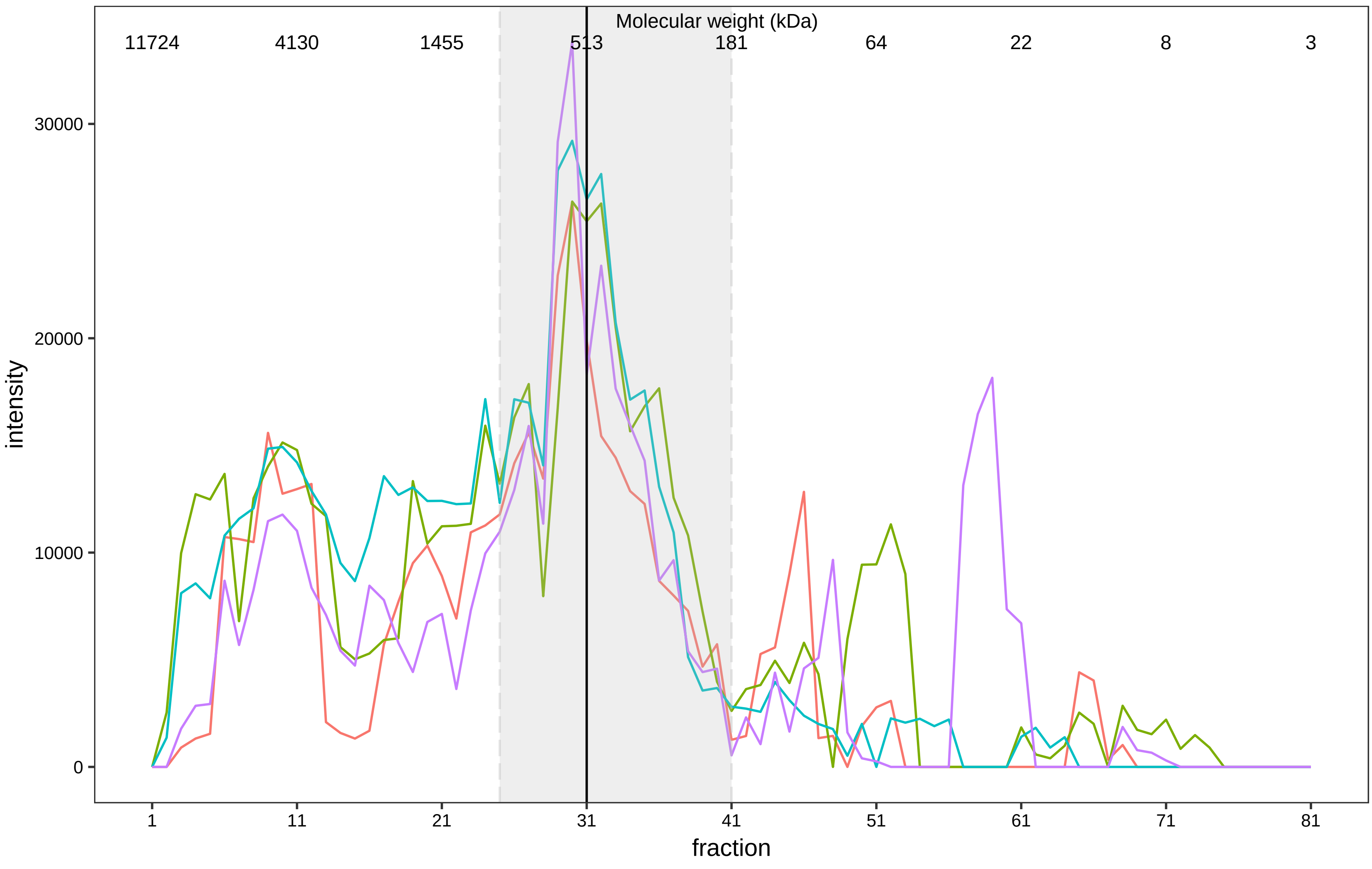

Feature ID 346

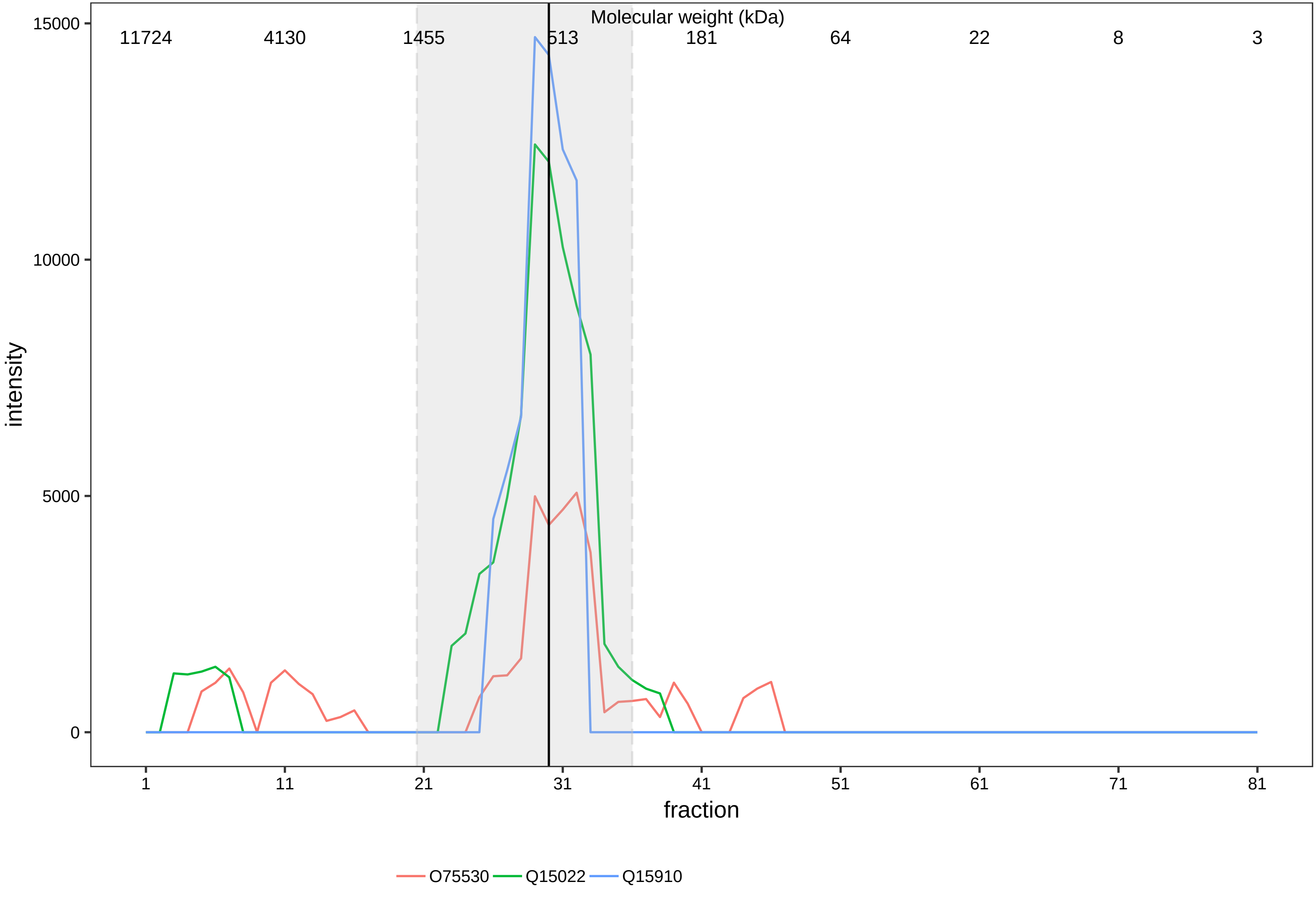

Feature ID 347

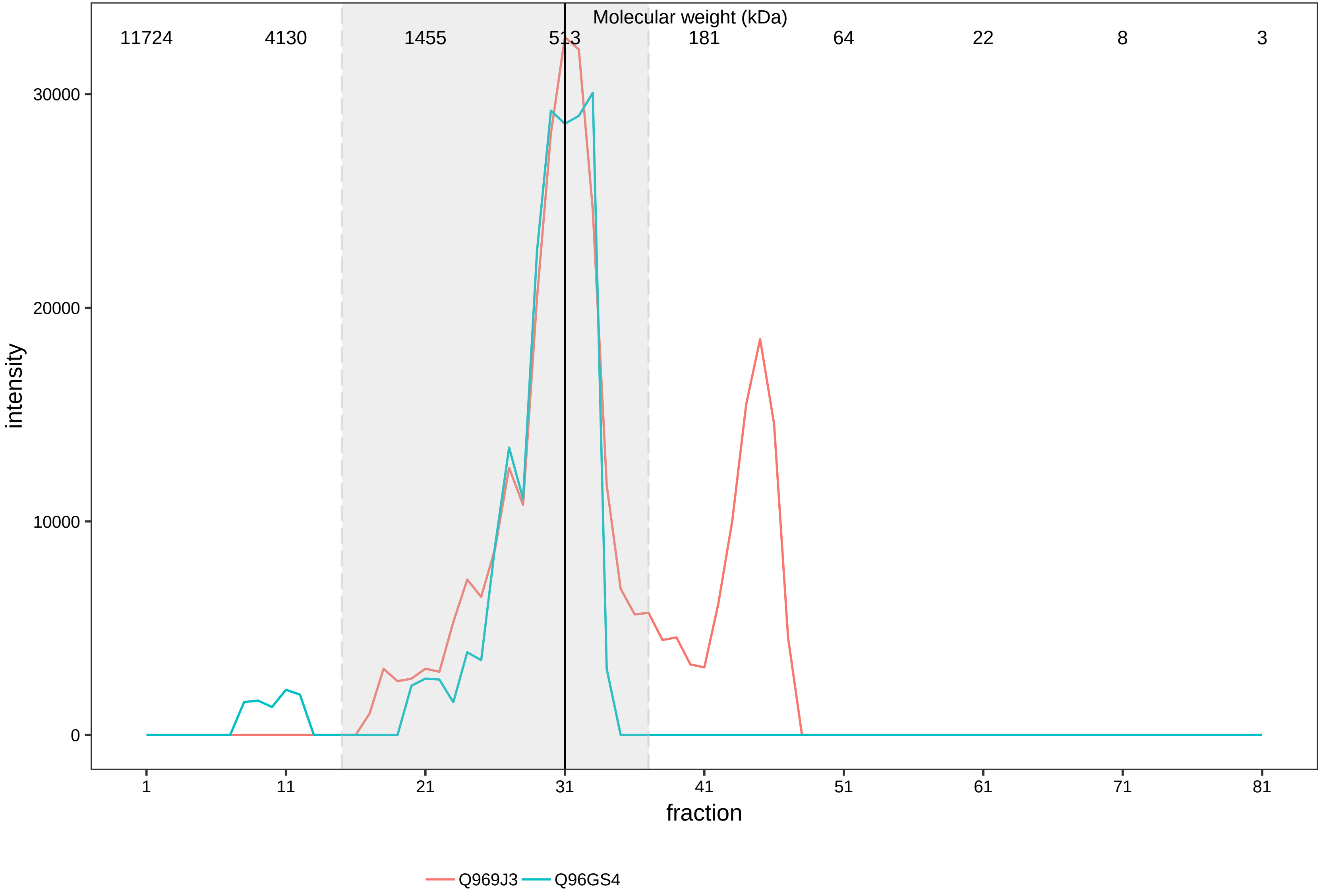

Feature ID 348

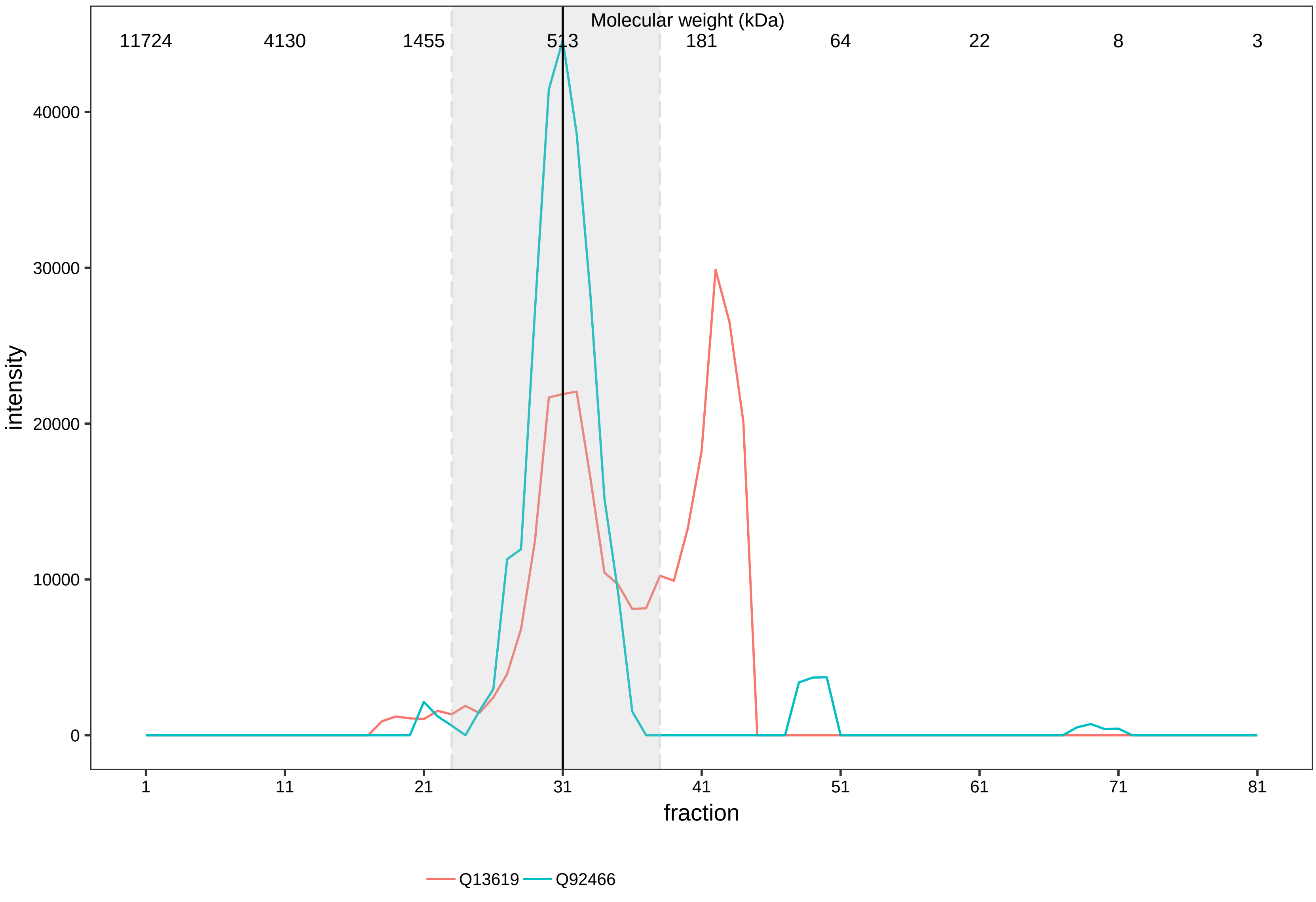

Feature ID 349

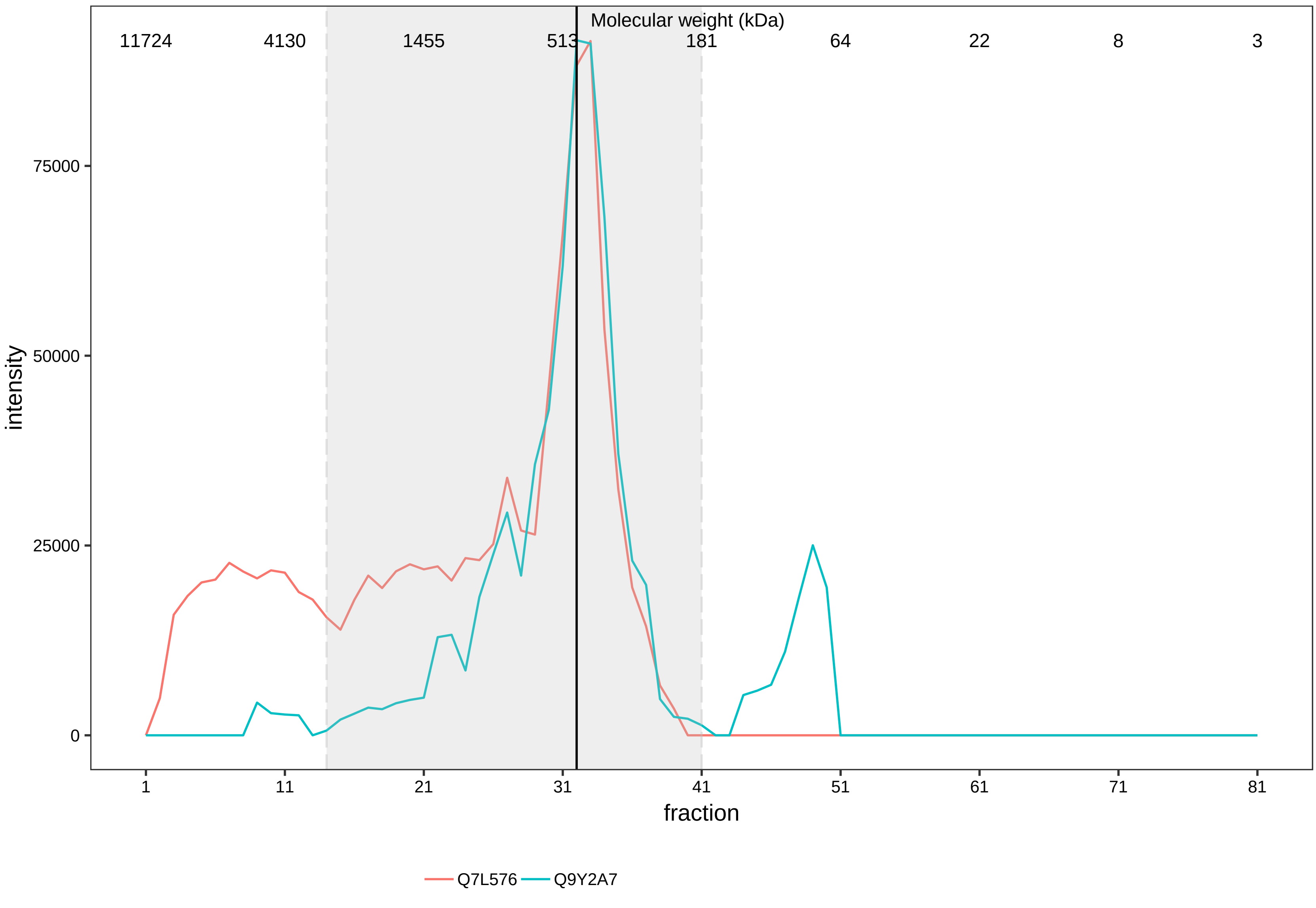

Feature ID 350

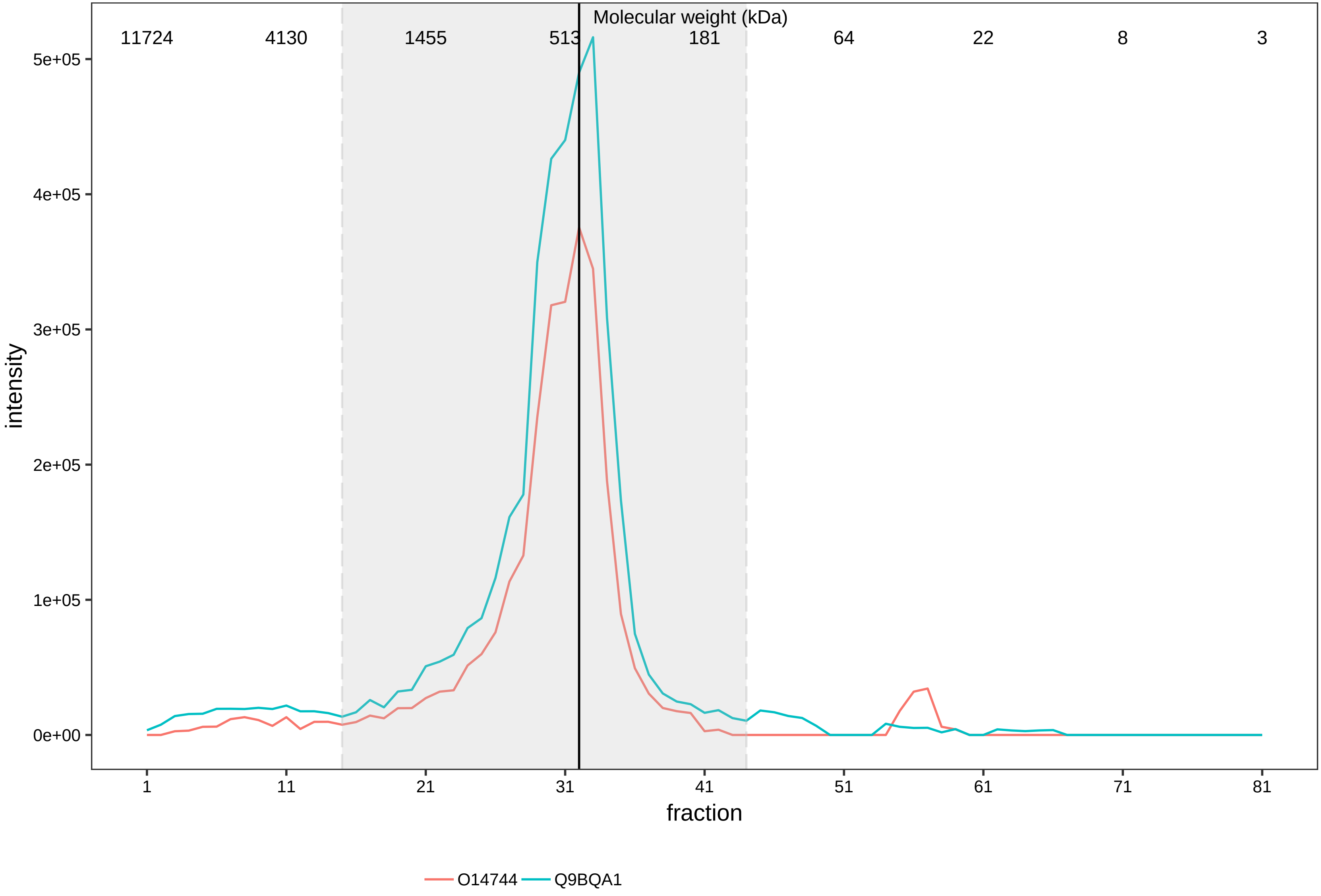

Feature ID 351

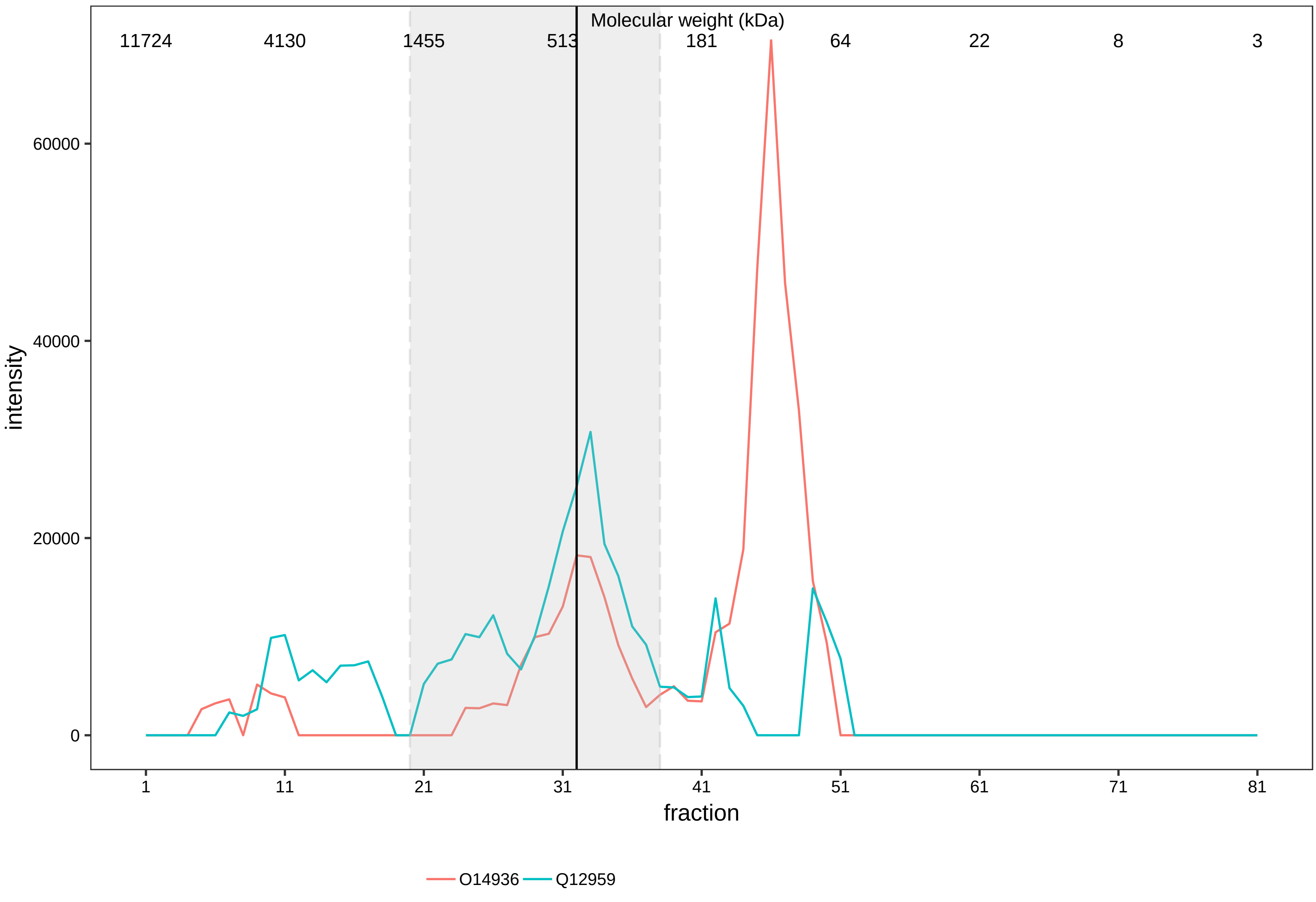

Feature ID 352

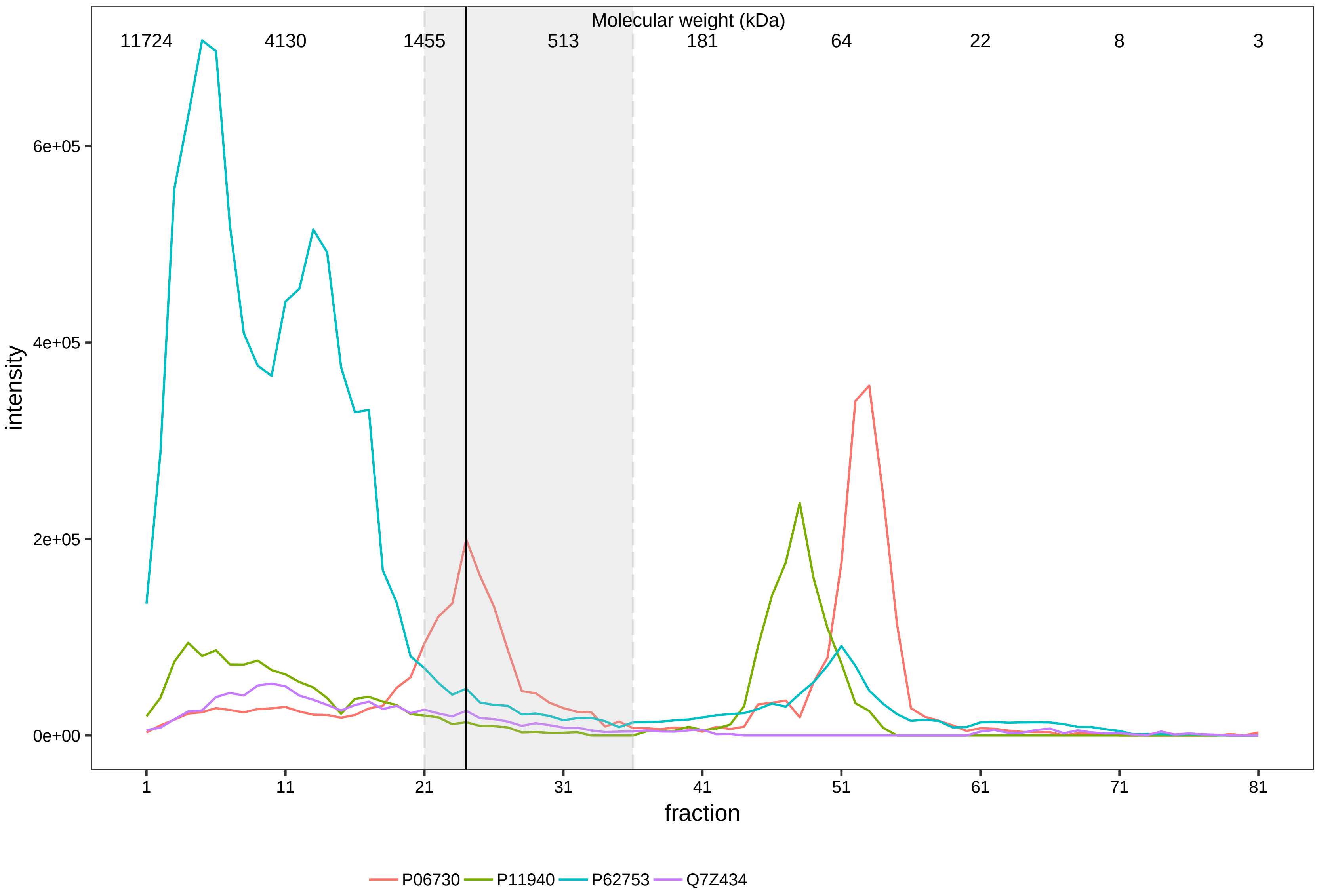

Feature ID 353

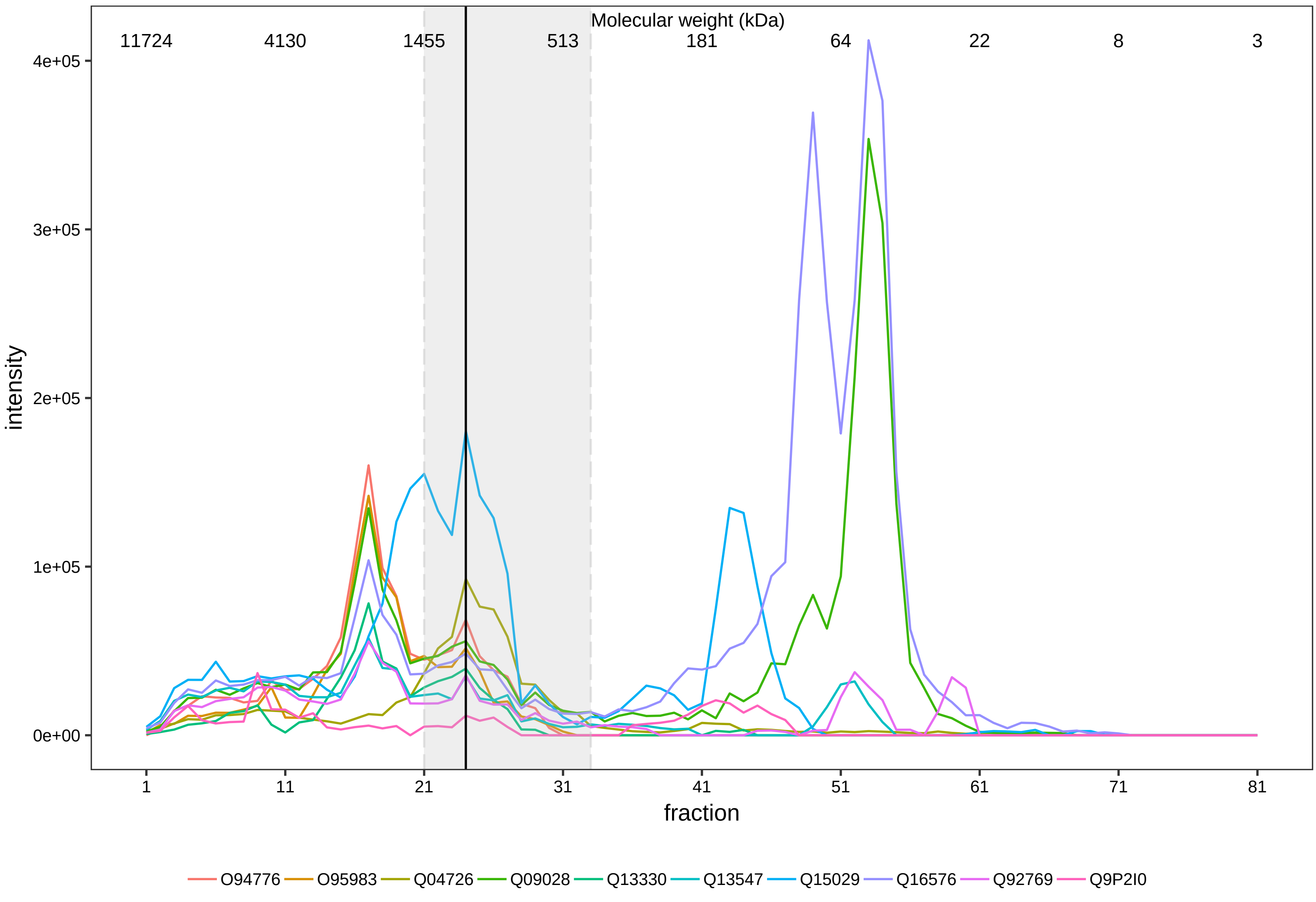

Feature ID 354

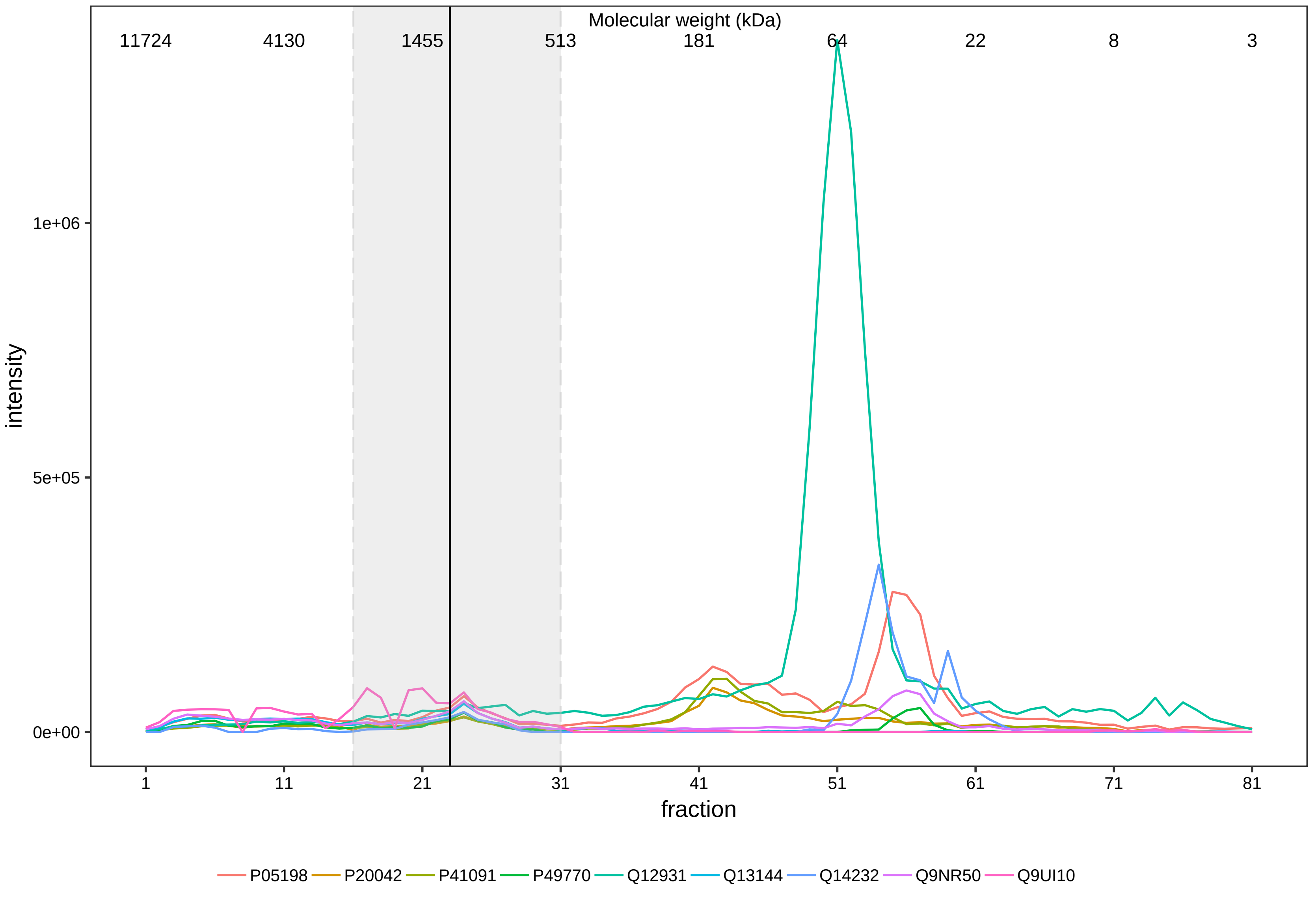

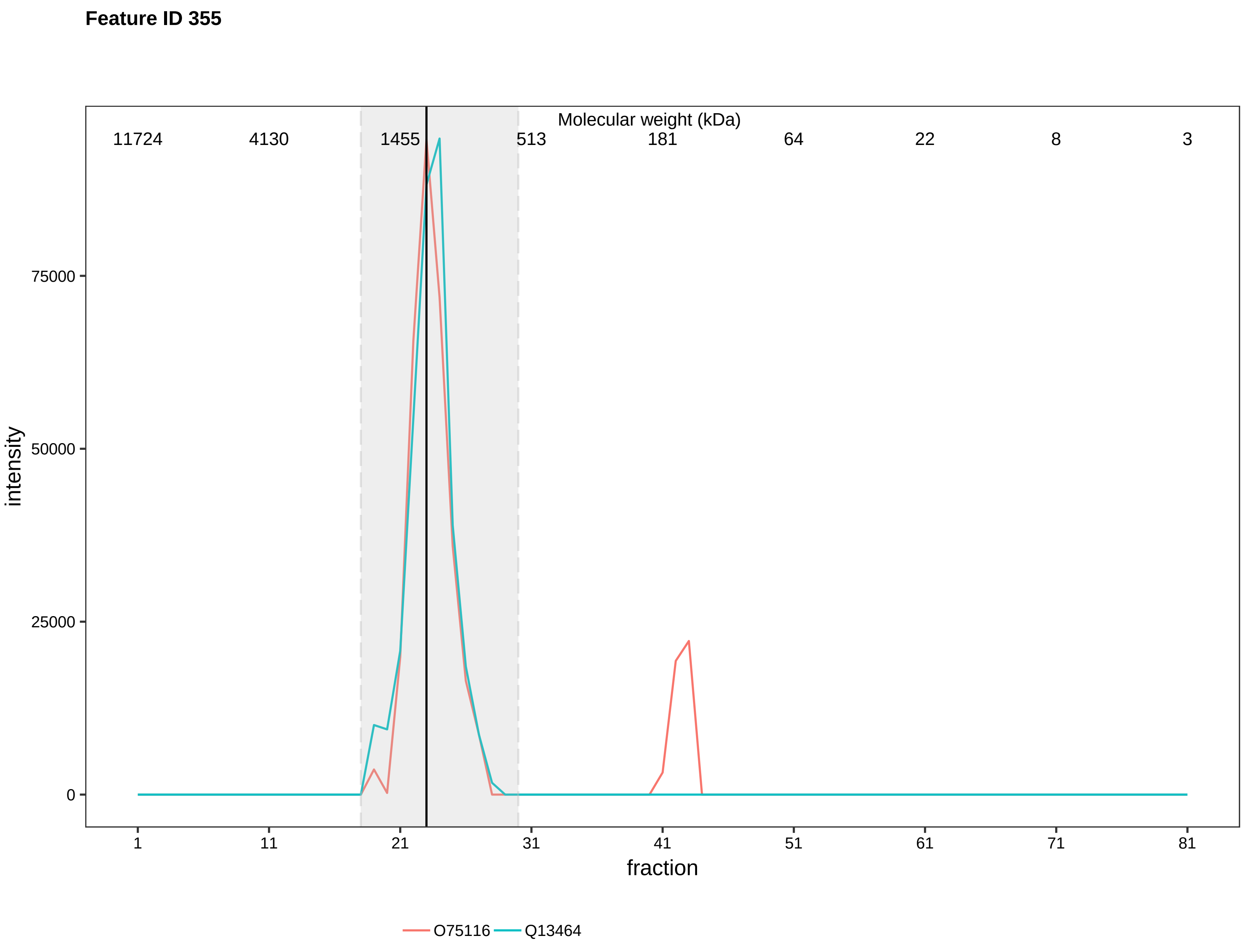

Feature ID 355
Molecular weight (kDa)
11724
4130
1455
513
181
64
22
8
3
intensity
75000
50000
25000
0
1
11
21
31
41
51
61
71
81
fraction
O75116
Q13464

Feature ID 356

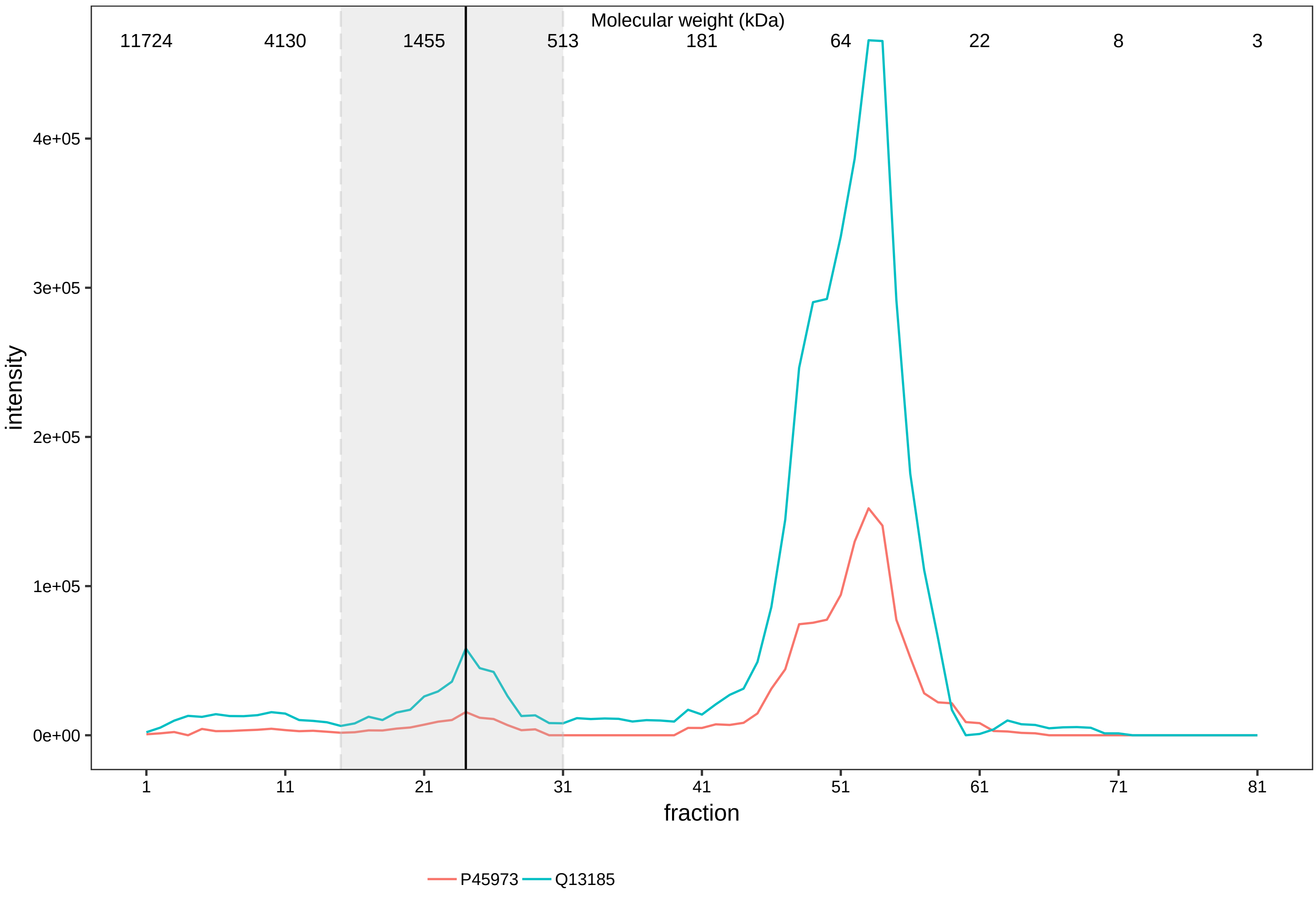

Feature ID 357

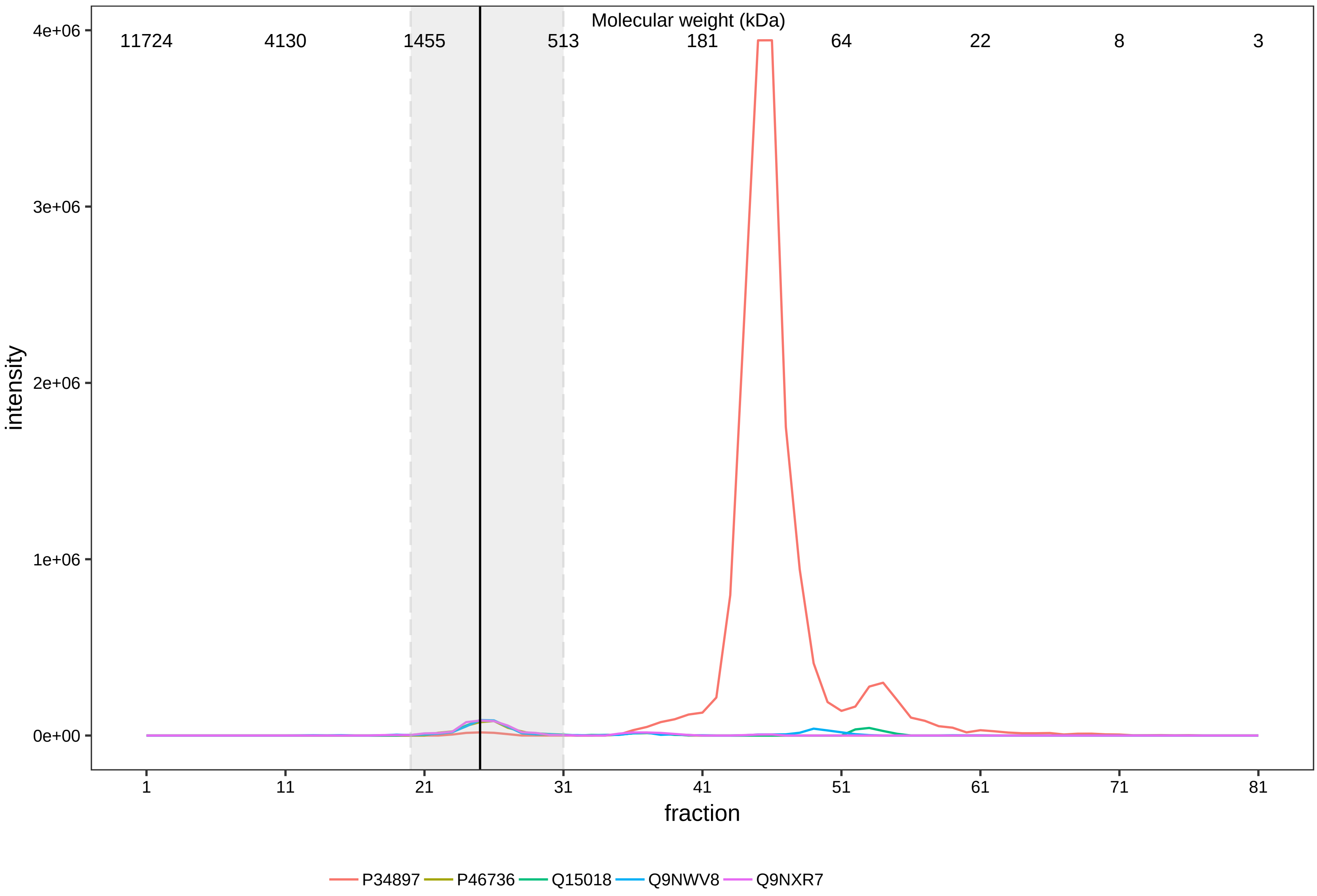

Feature ID 358

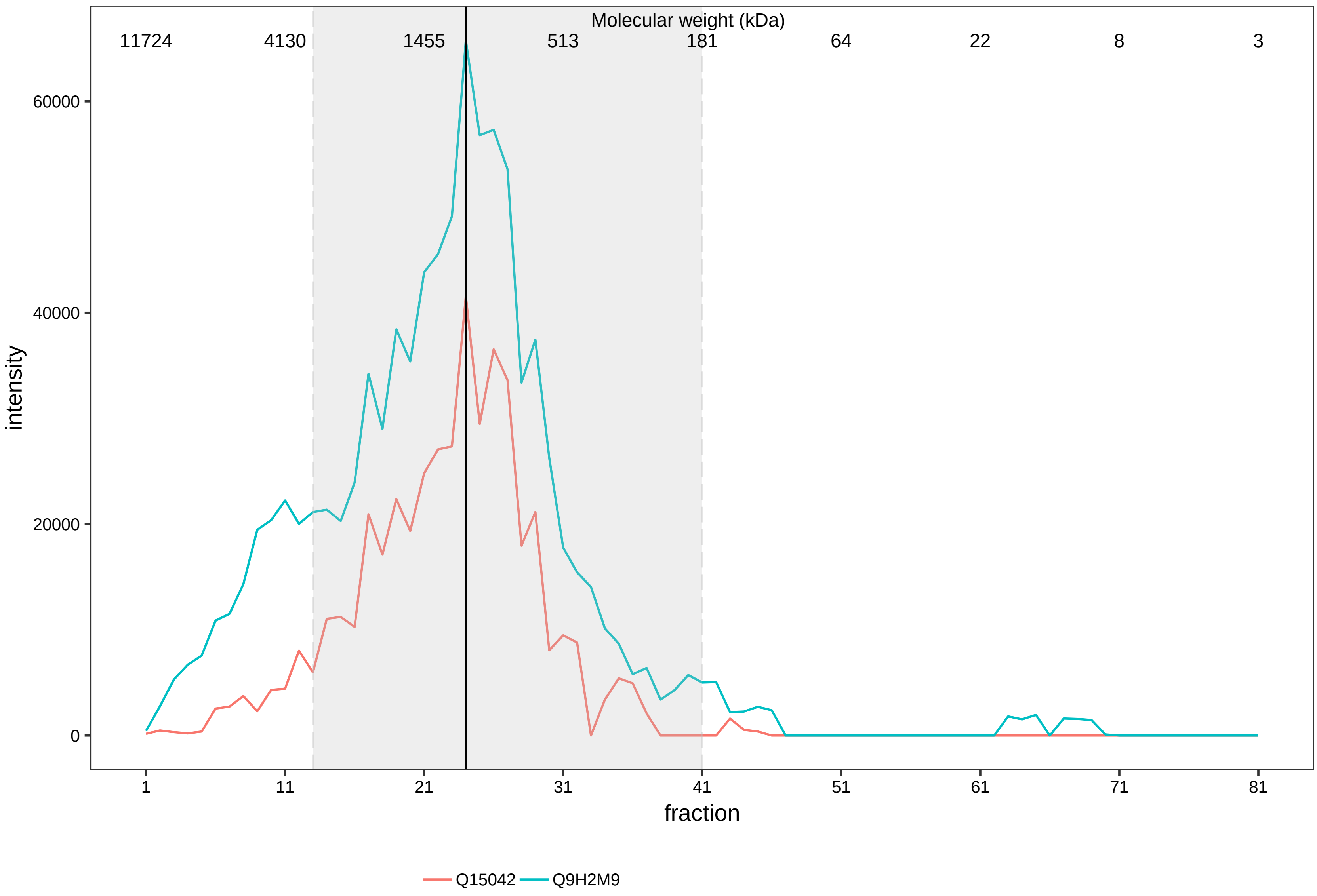

Feature ID 359

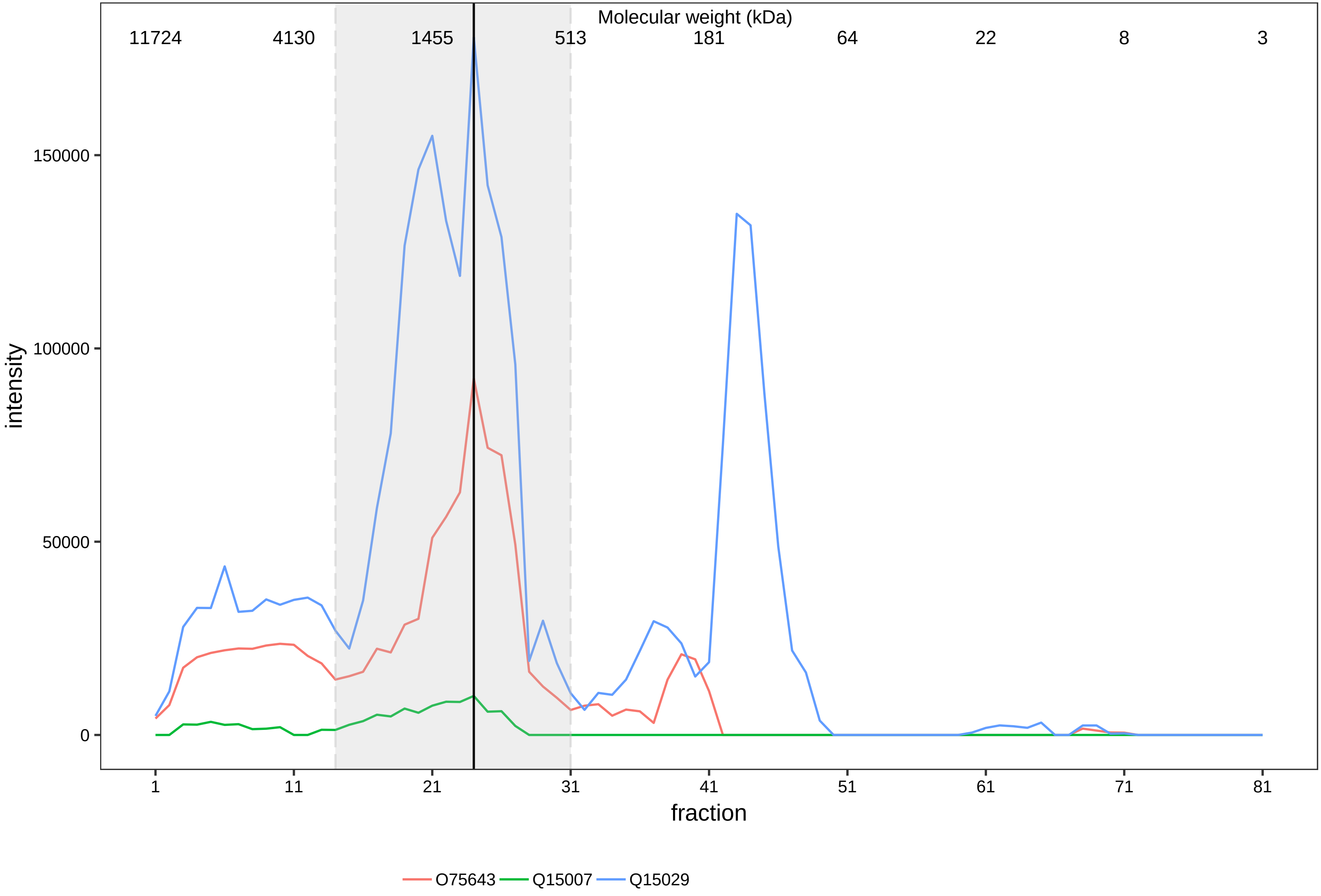

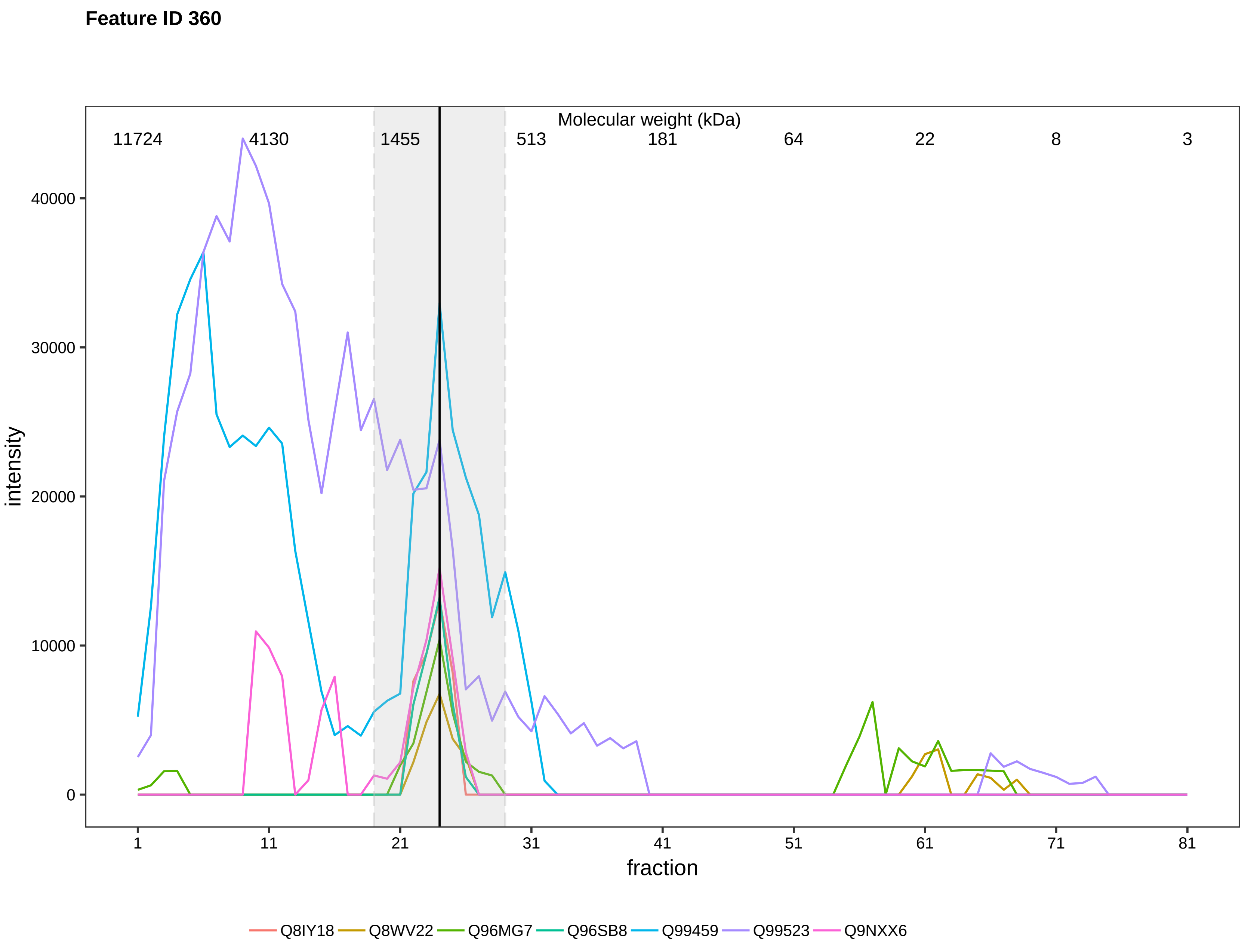
Feature ID 360
Molecular weight (kDa)
11724
4130
1455
513
181
64
22
8
3
40000
30000
20000
10000
0
intensity
1
11
21
31
41
51
61
71
81
fraction
Q8IY18
Q8WV22
Q96MG7
Q96SB8
Q99459
Q99523
Q9NXX6

Feature ID 361

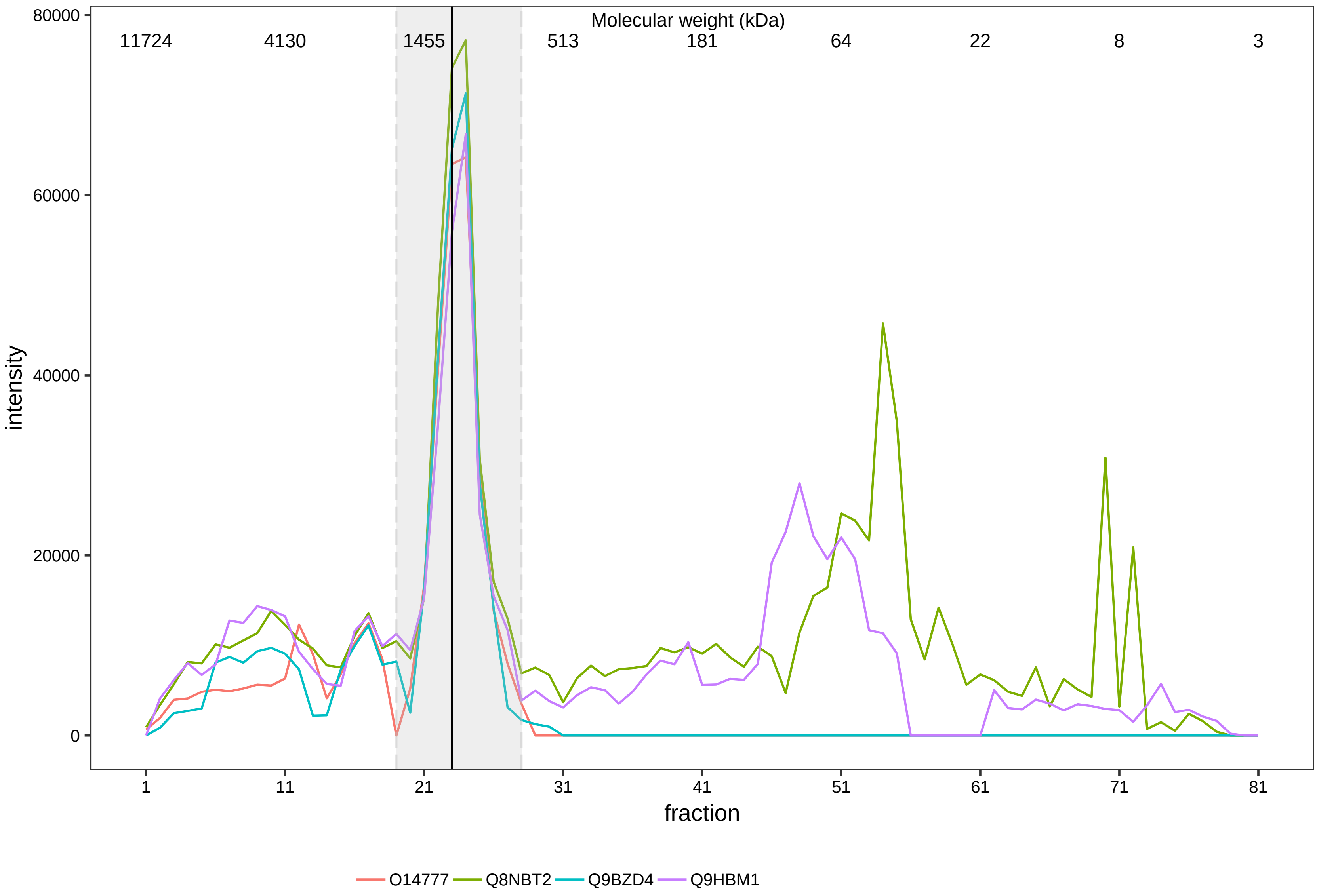

Feature ID 362

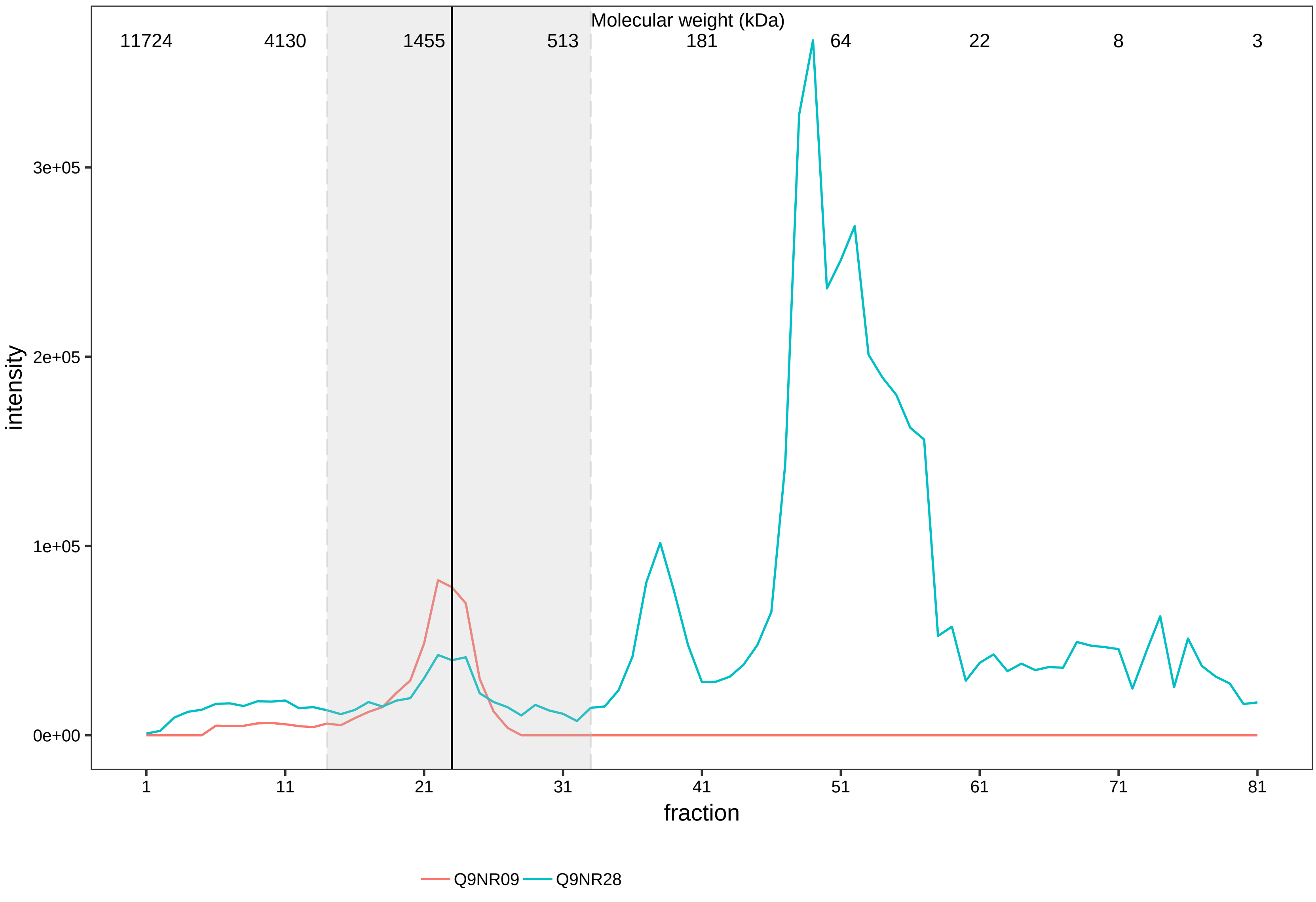

Feature ID 363

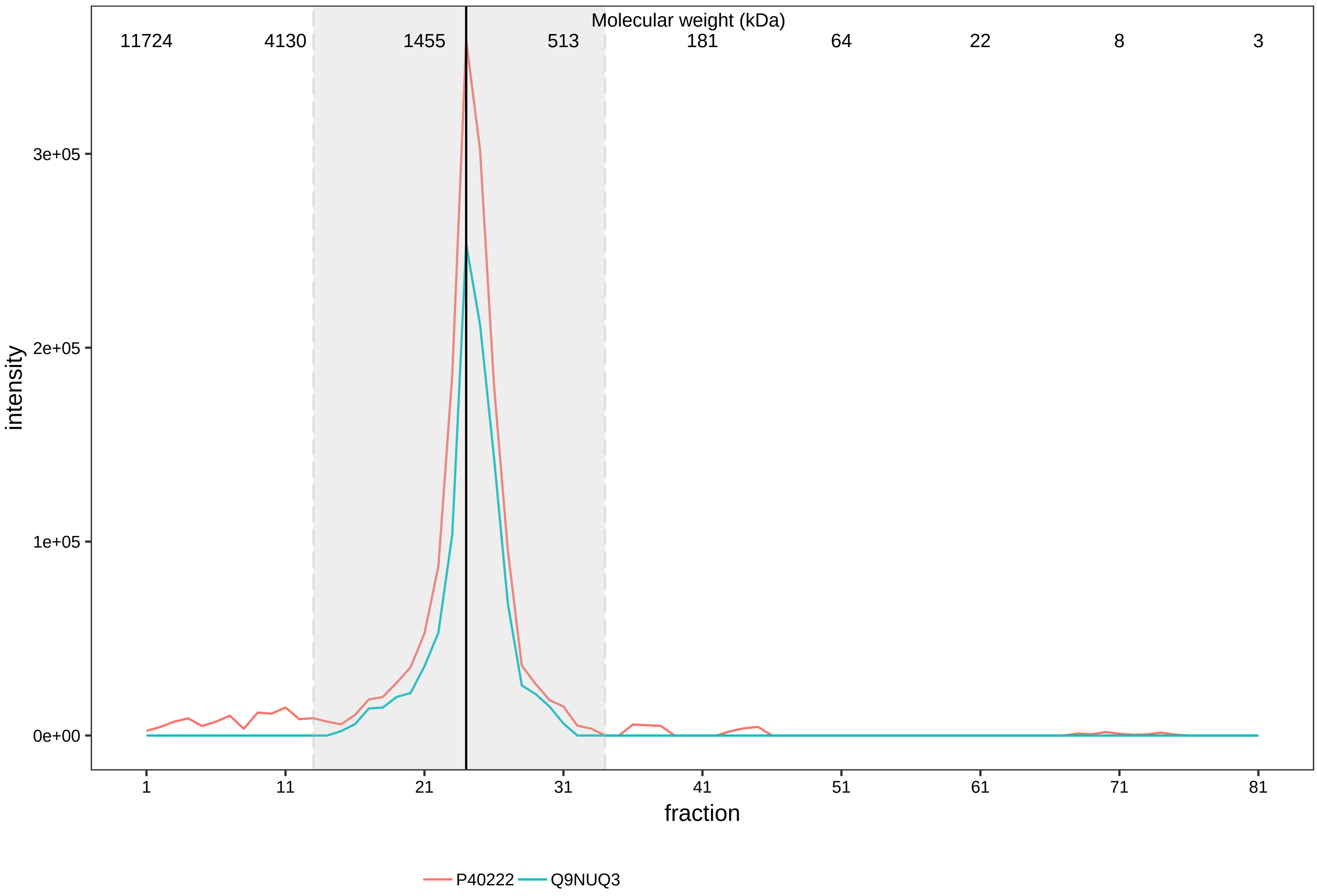

Feature ID 364

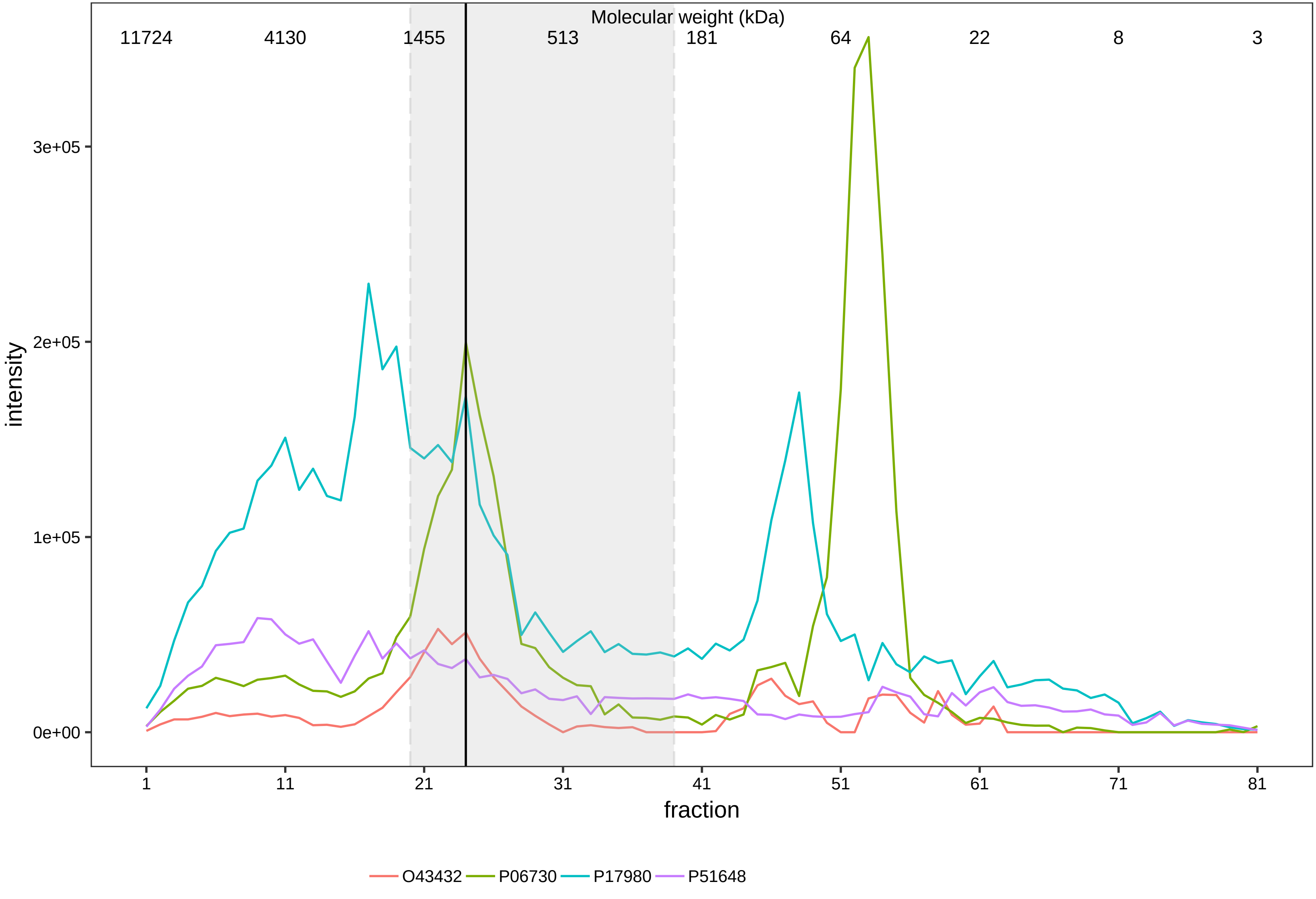

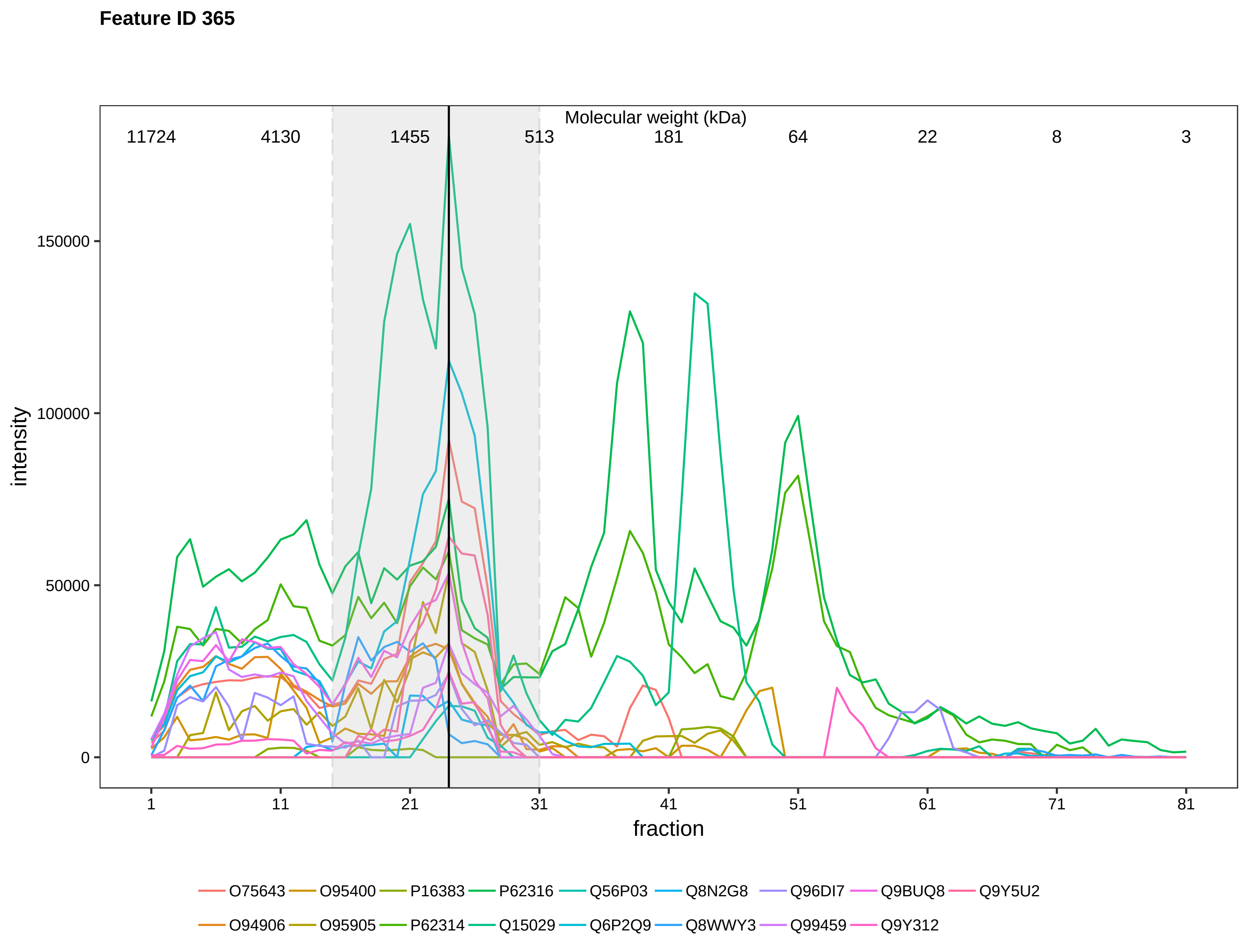
Feature ID 365
Molecular weight (kDa)
11724
4130
1455
513
181
64
22
8
3
intensity
150000
100000
50000
0
fraction
1
11
21
31
41
51
61
71
81
O75643
O95400
P16383
P62316
Q56P03
Q8N2G8
Q96DI7
Q9BUQ8
Q9Y5U2
O94906
O95905
P62314
Q15029
Q6P2Q9
Q8WWY3
Q99459
Q9Y312

Feature ID 366

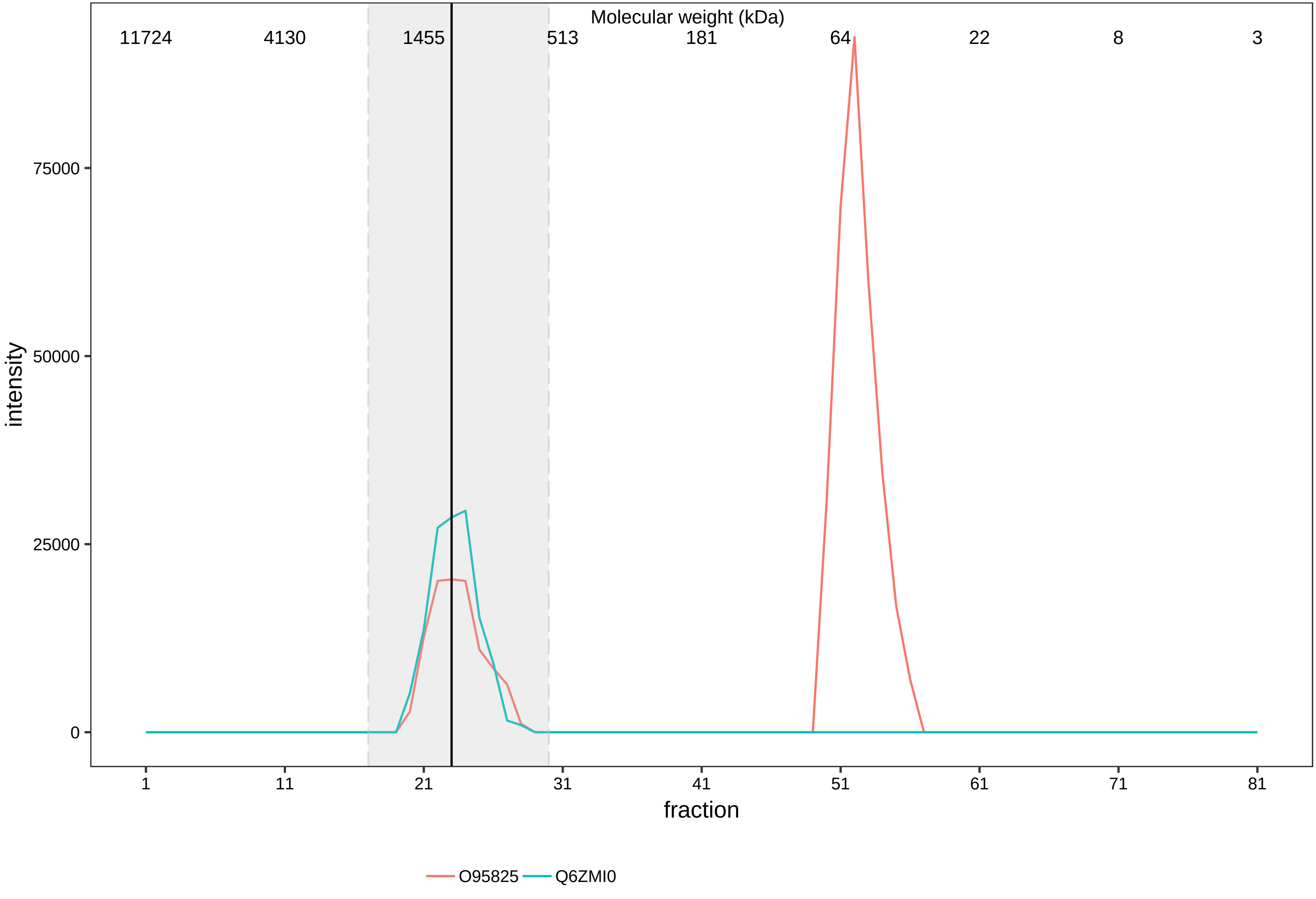

Feature ID 367

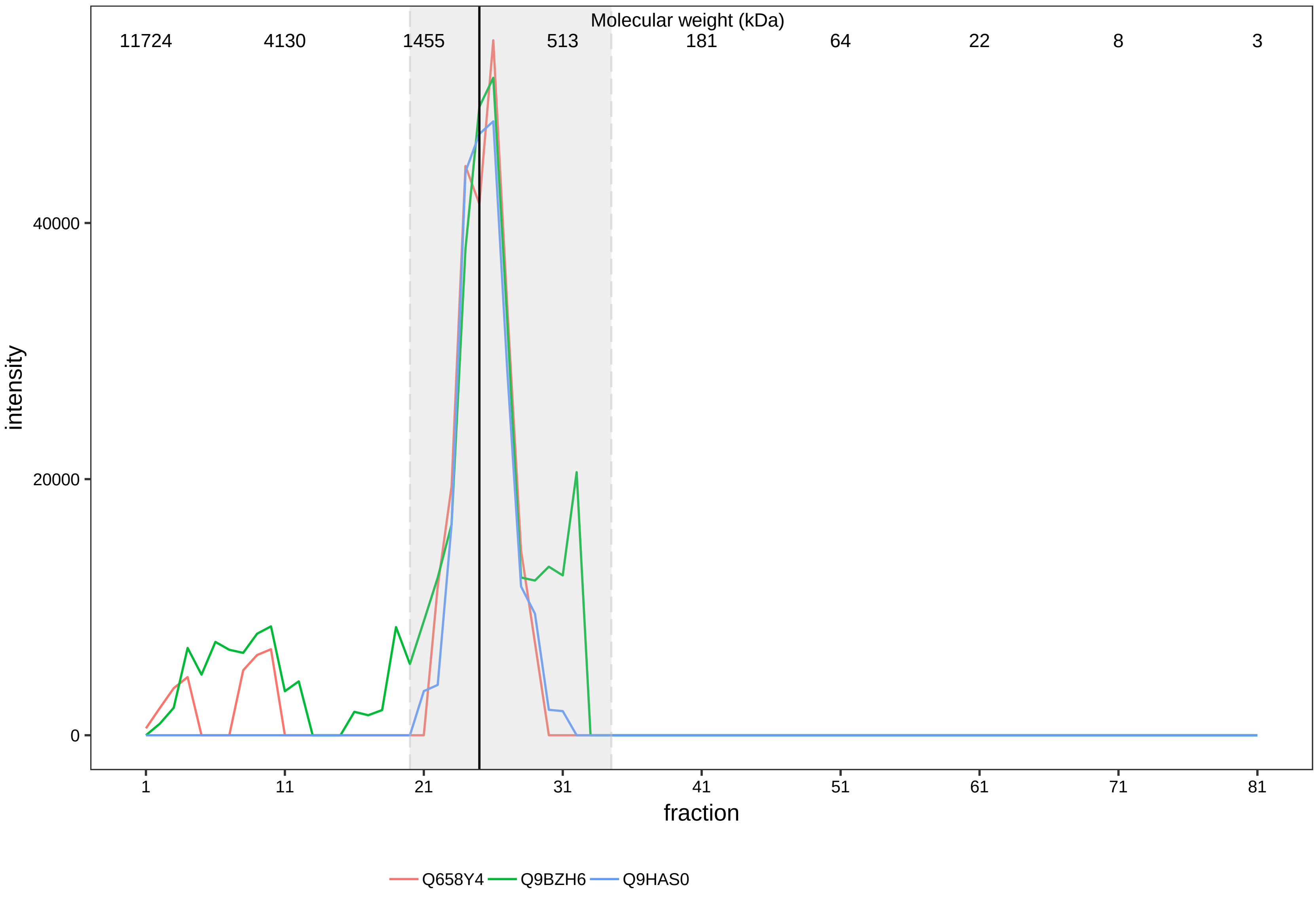

Feature ID 368

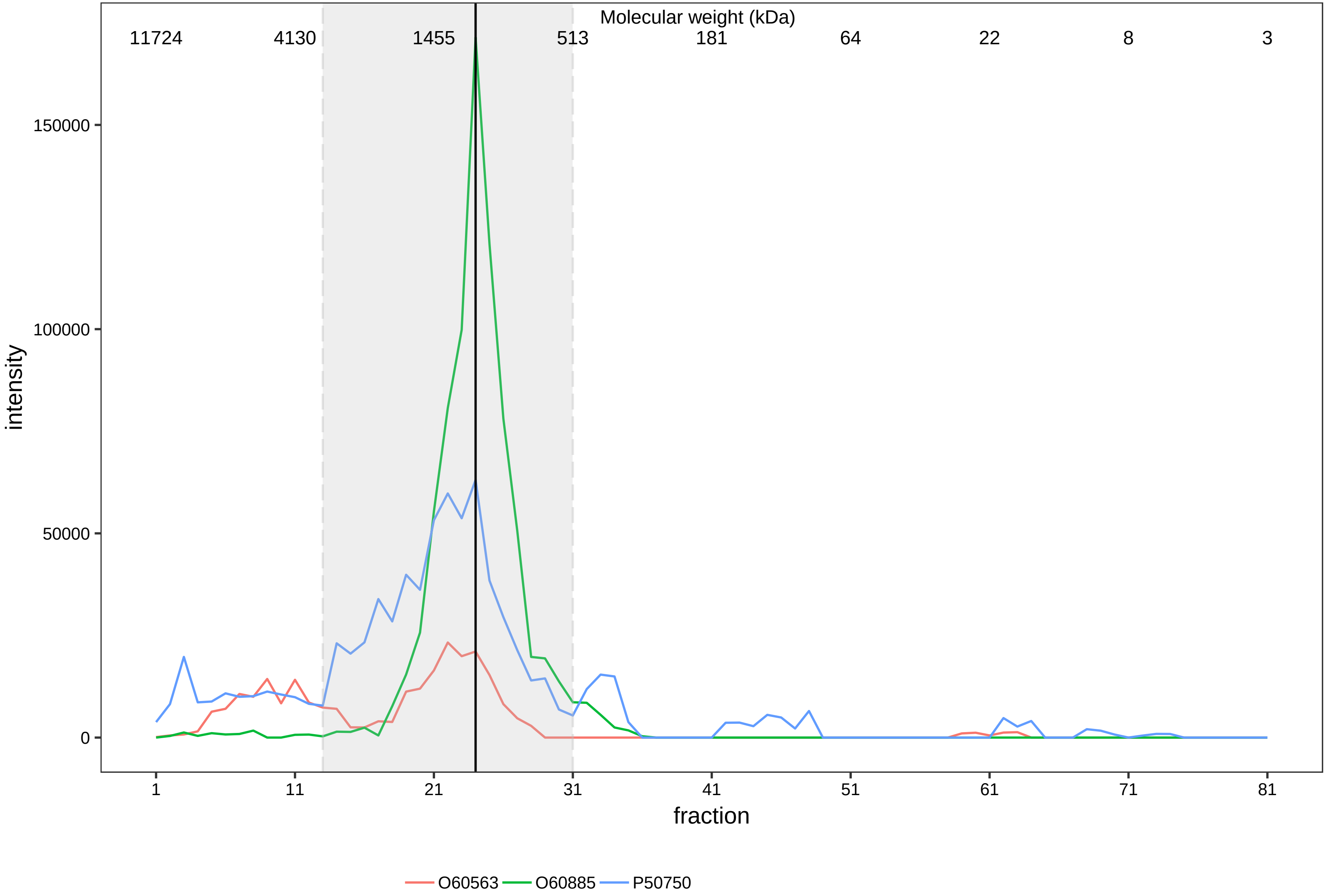

Feature ID 369

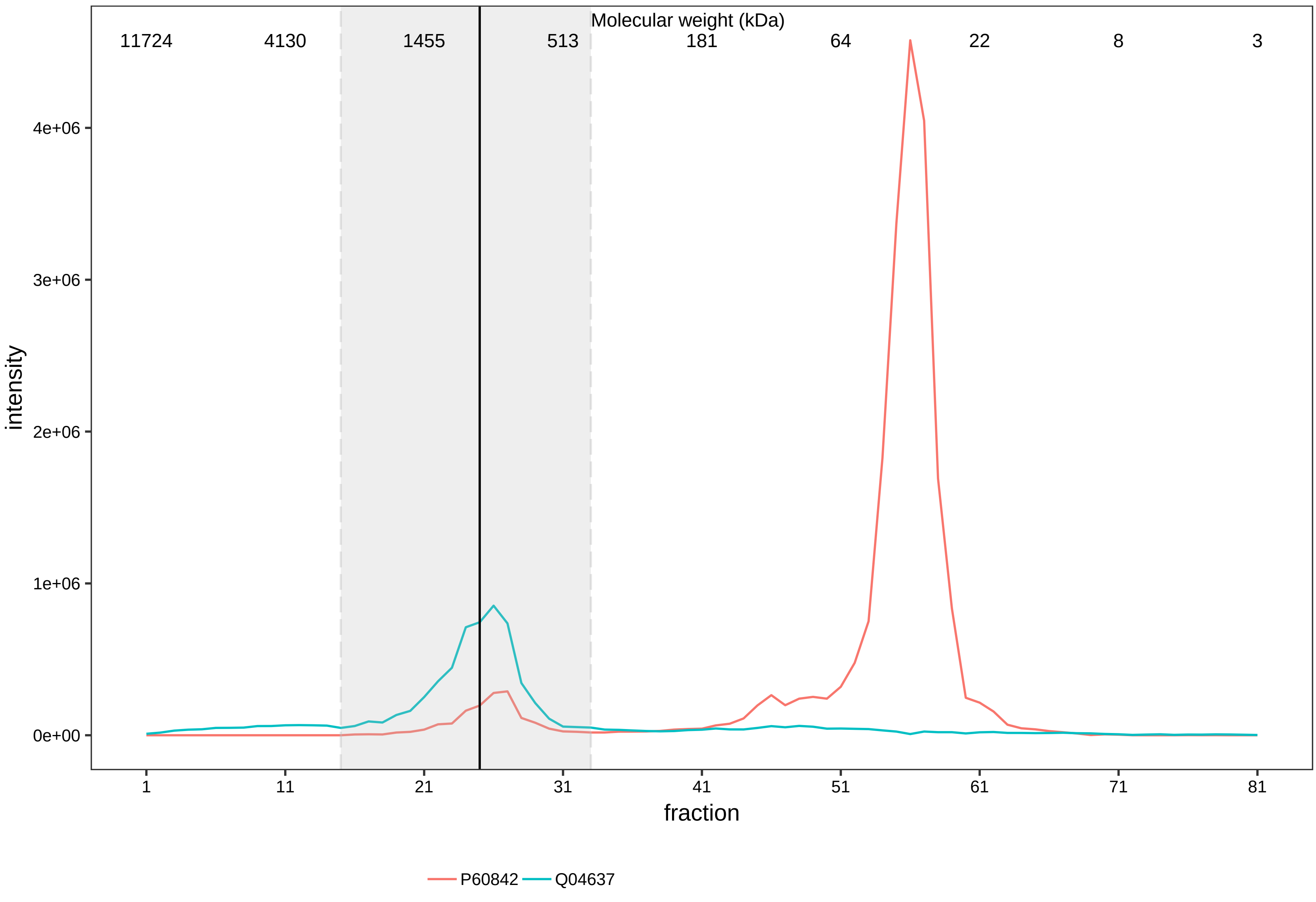

Feature ID 370

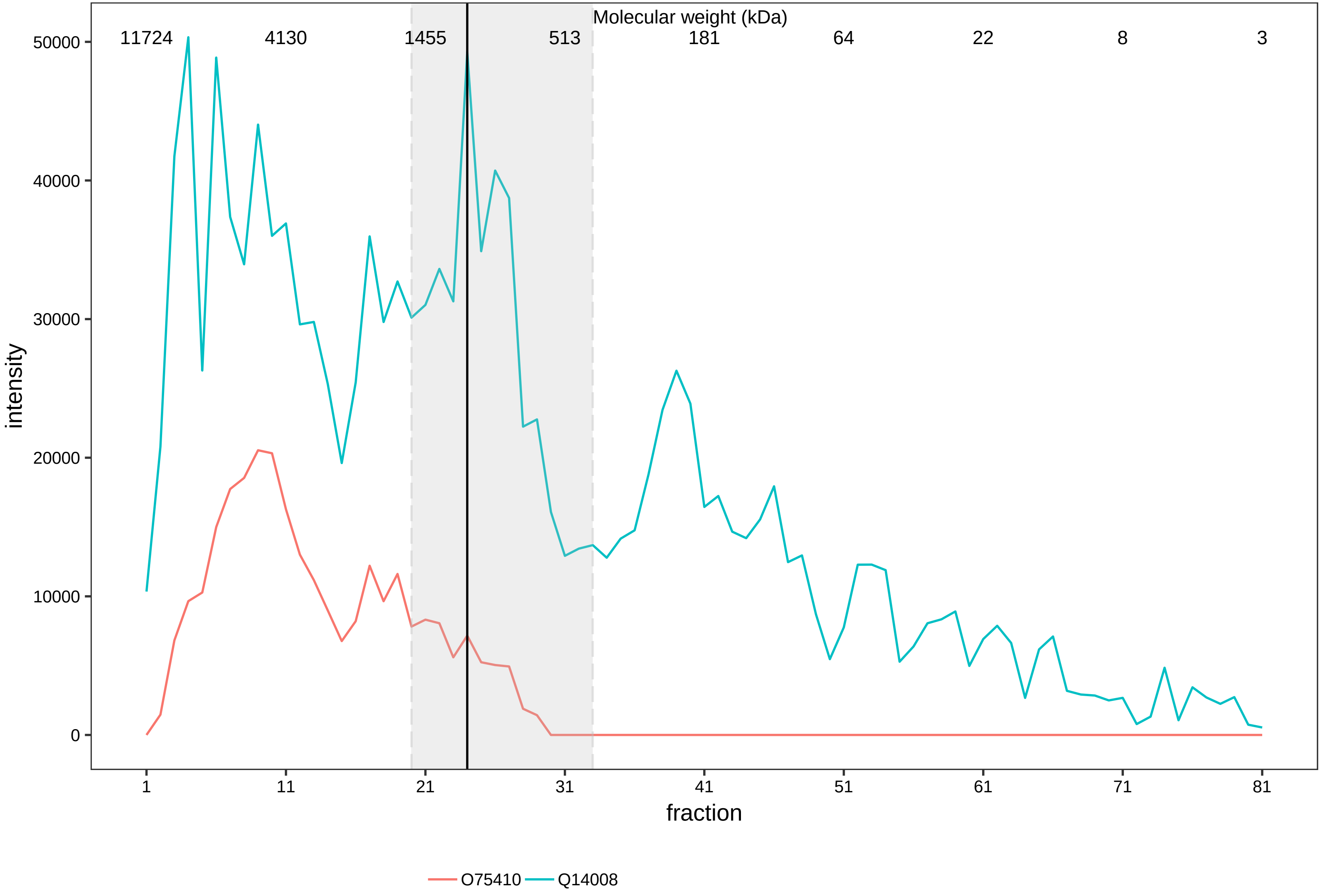

Feature ID 371

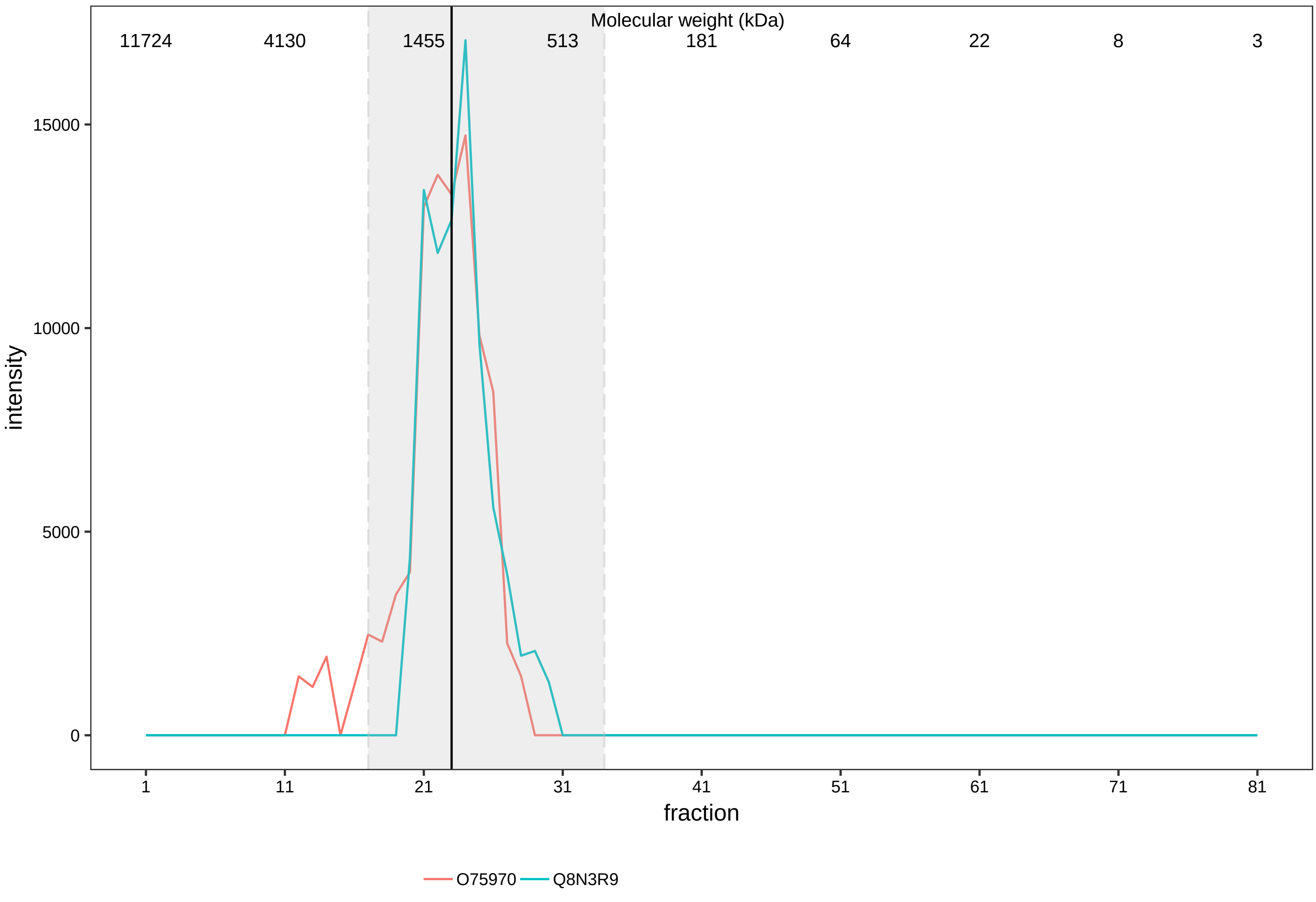

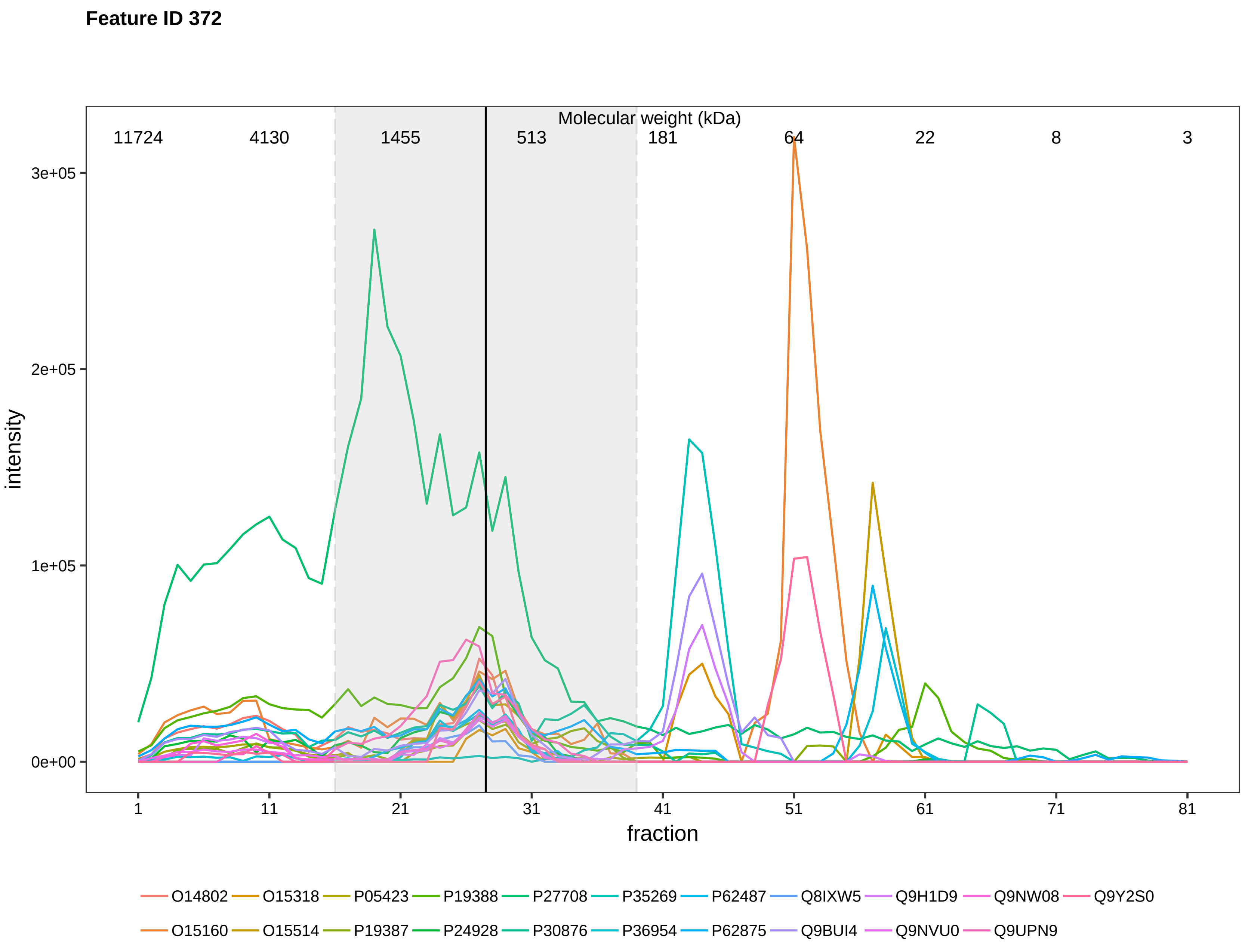
Feature ID 372
Molecular weight (kDa)
11724
4130
1455
513
181
64
22
8
3
3e+05
2e+05
1e+05
0e+00
intensity
1
11
21
31
41
51
61
71
81
fraction
O14802
O15318
P05423
P19388
P27708
P35269
P62487
Q8IXW5
Q9H1D9
Q9NW08
Q9Y2S0
O15160
O15514
P19387
P24928
P30876
P36954
P62875
Q9BUI4
Q9NVU0
Q9UPN9

Feature ID 373

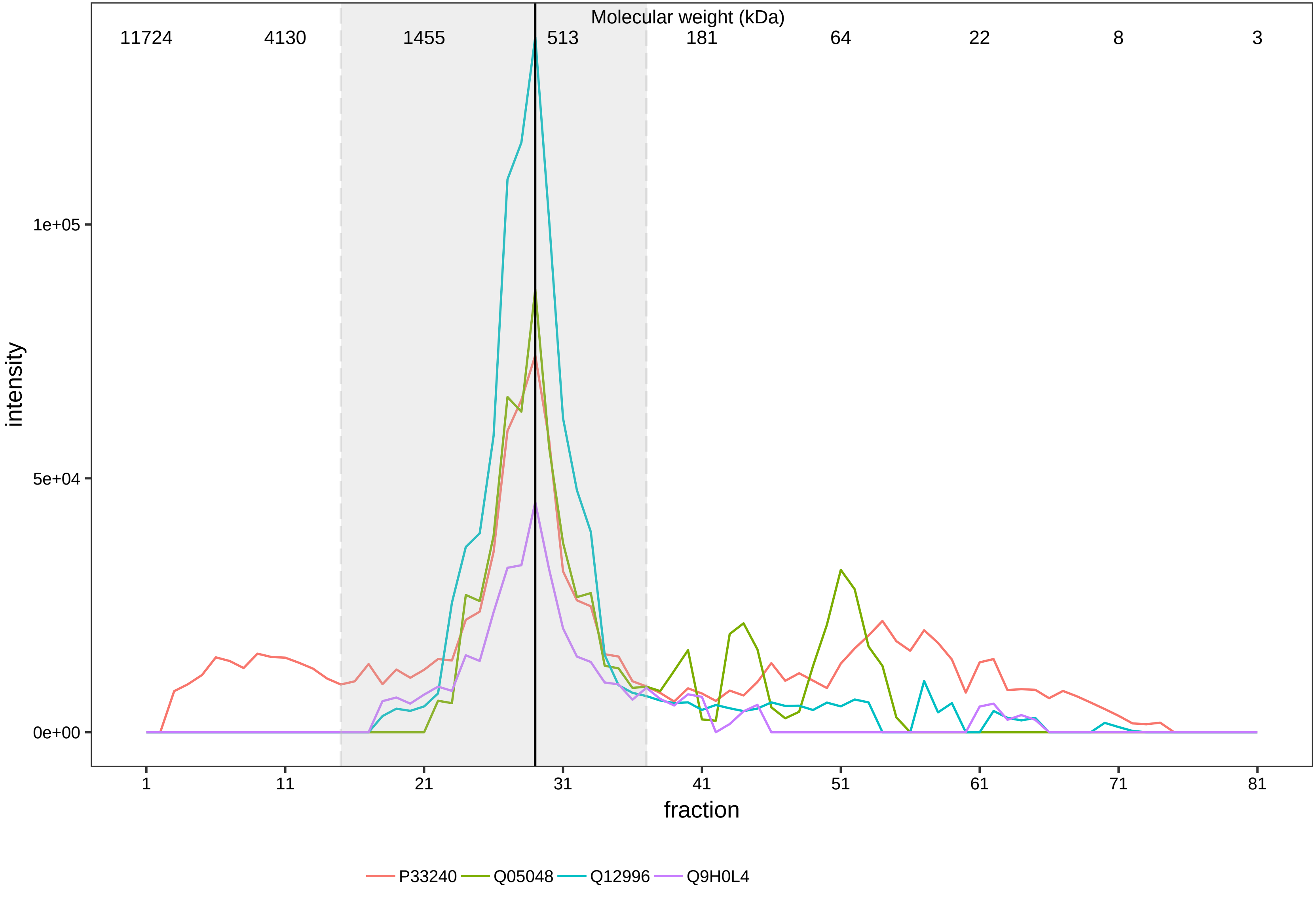

Feature ID 374

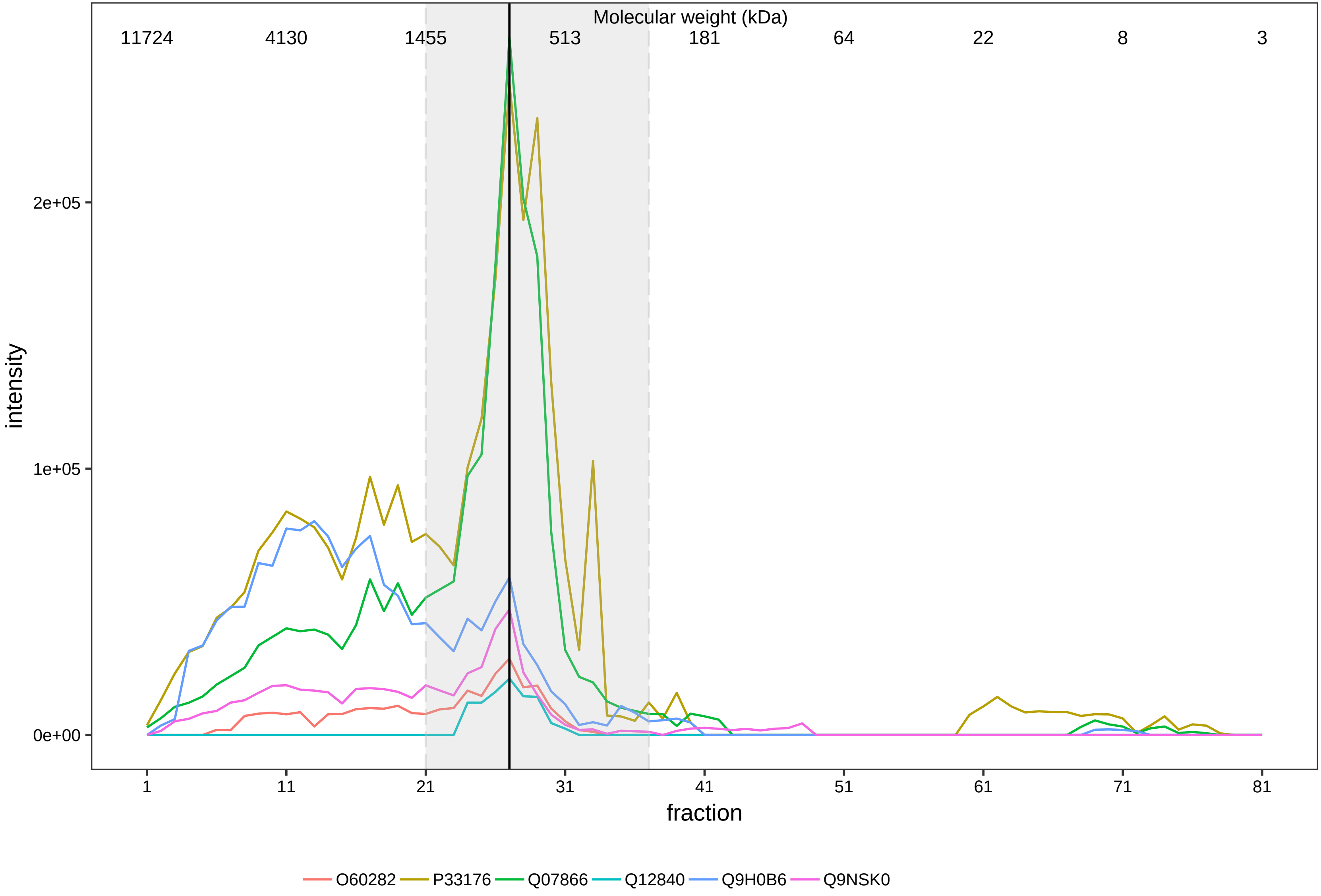

Feature ID 375

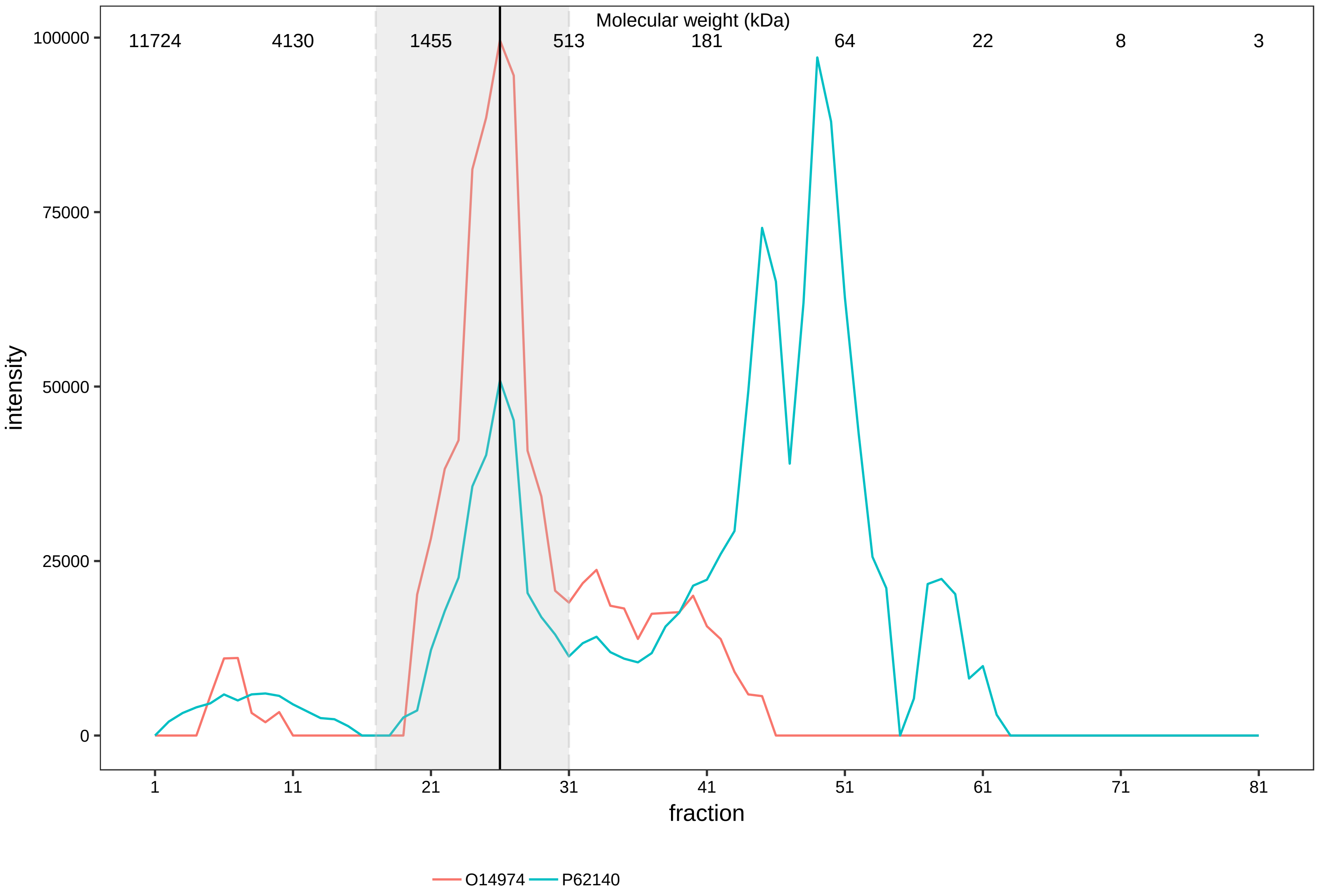

Feature ID 376

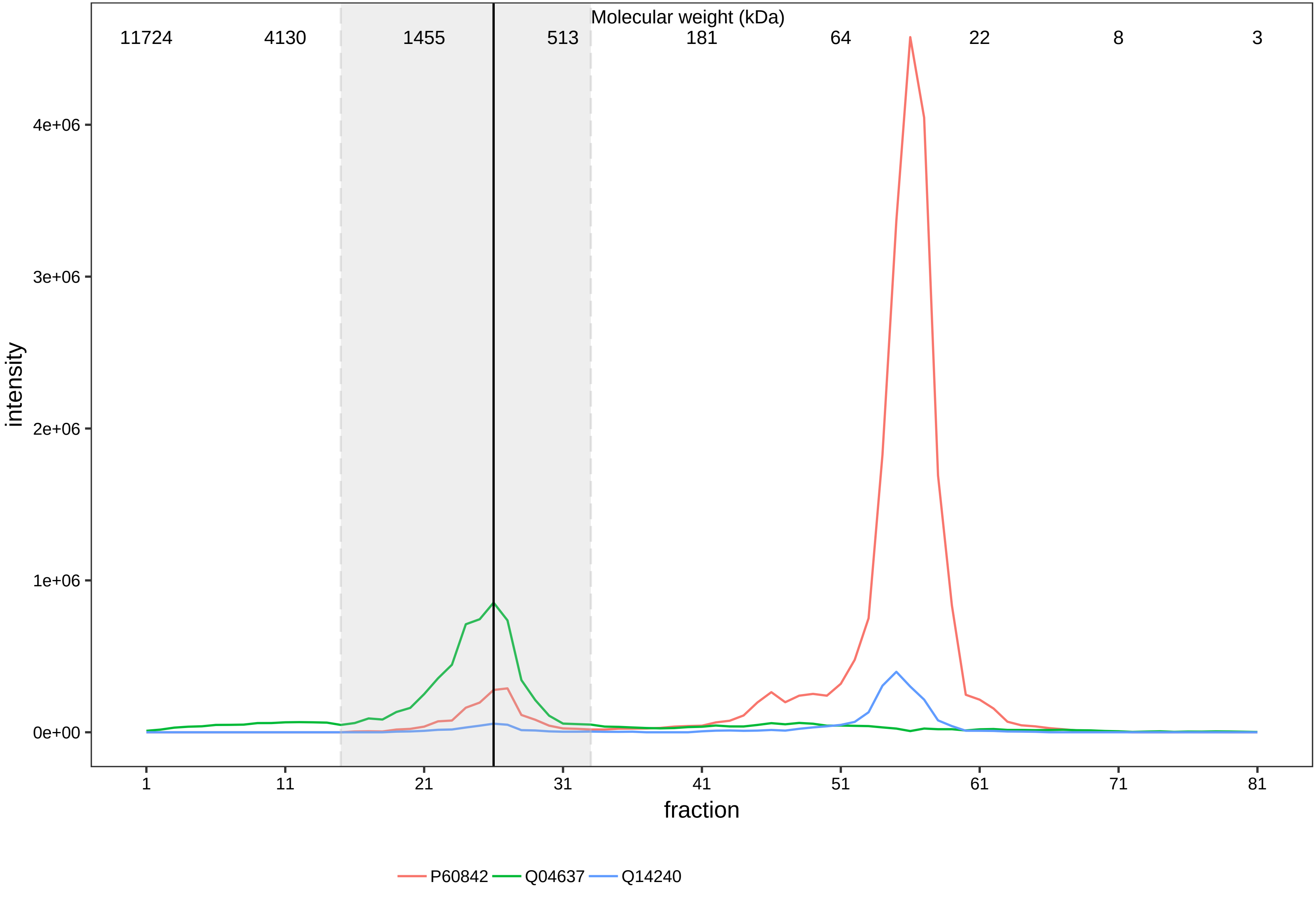

Feature ID 377

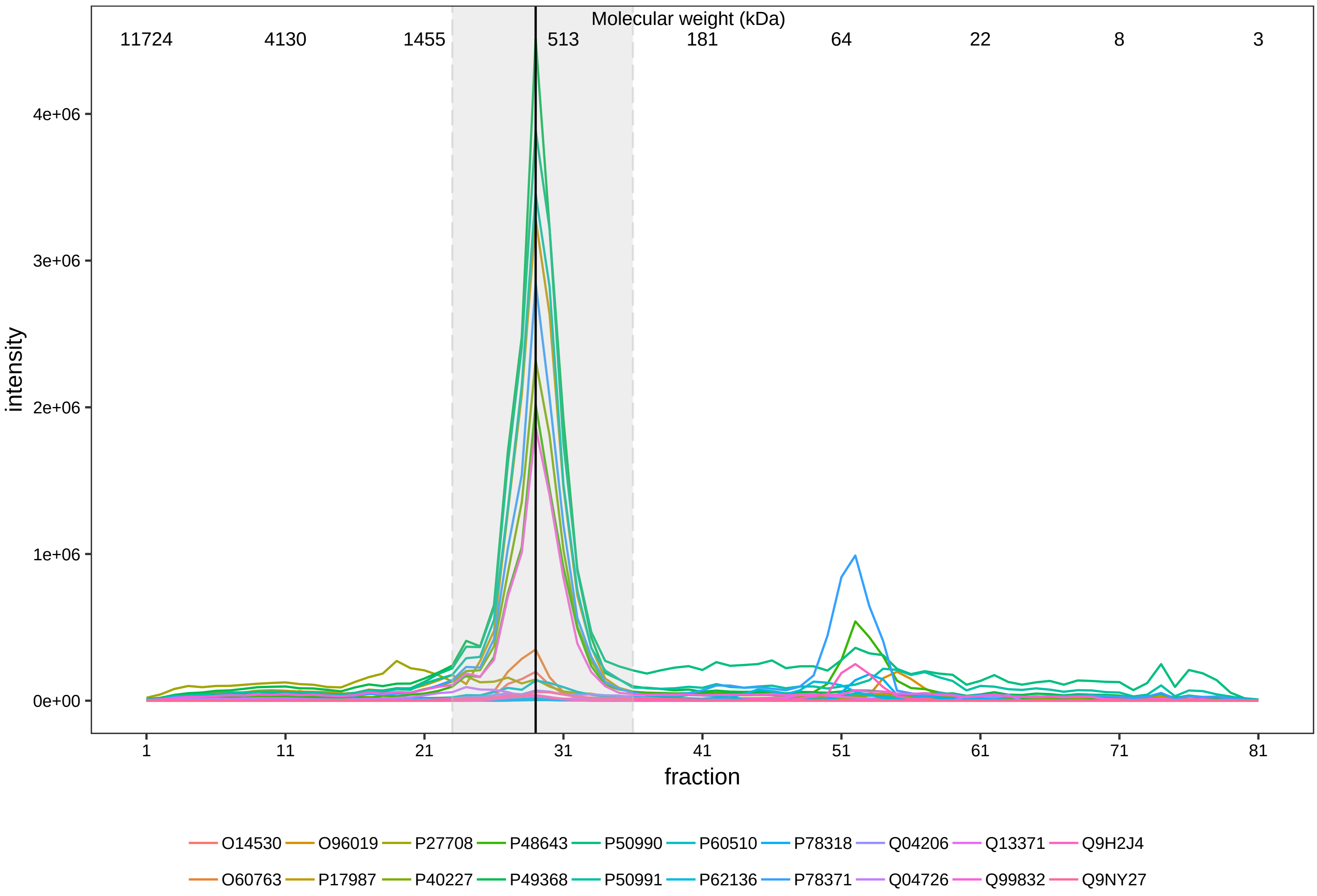

Feature ID 378

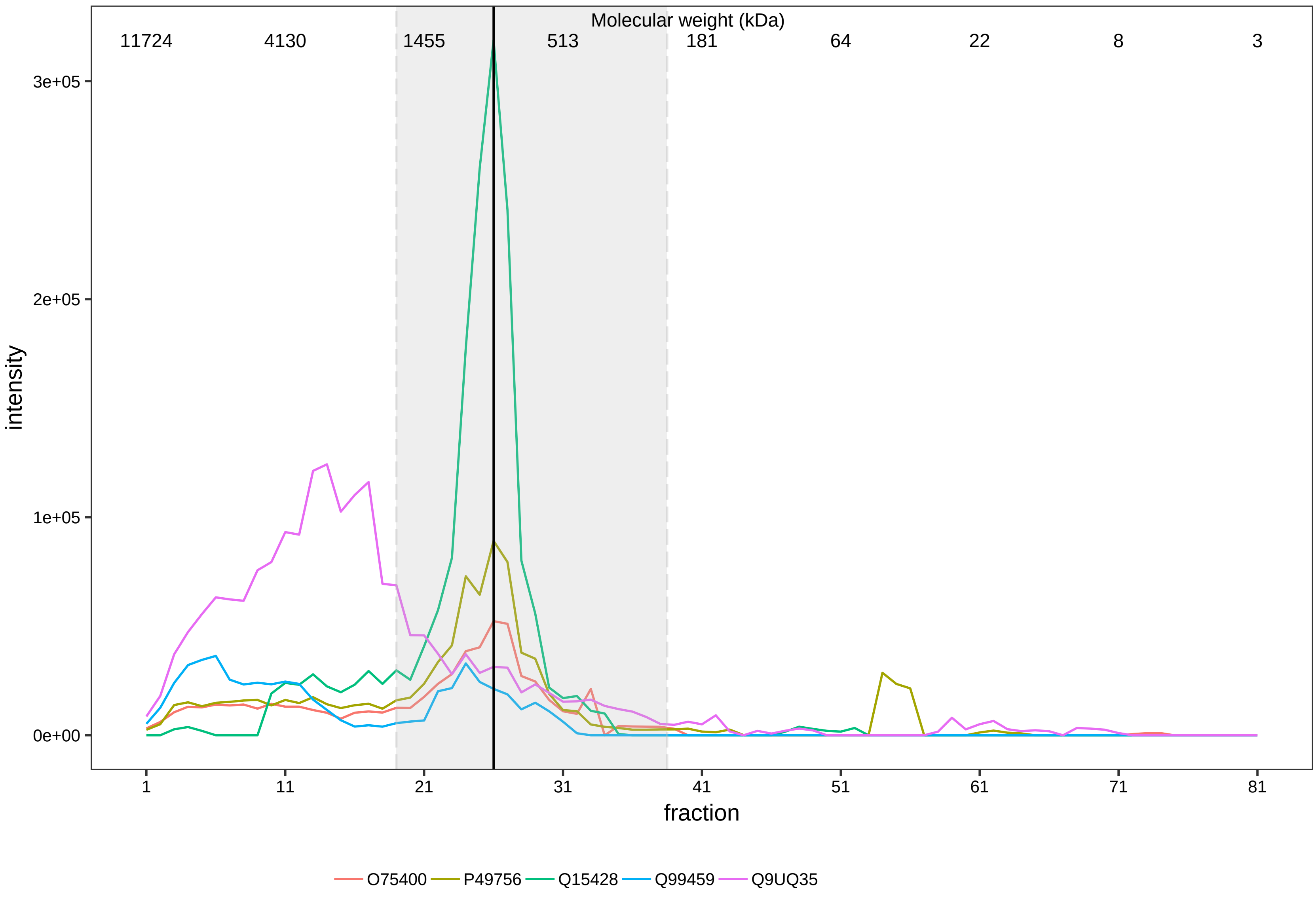

Feature ID 379

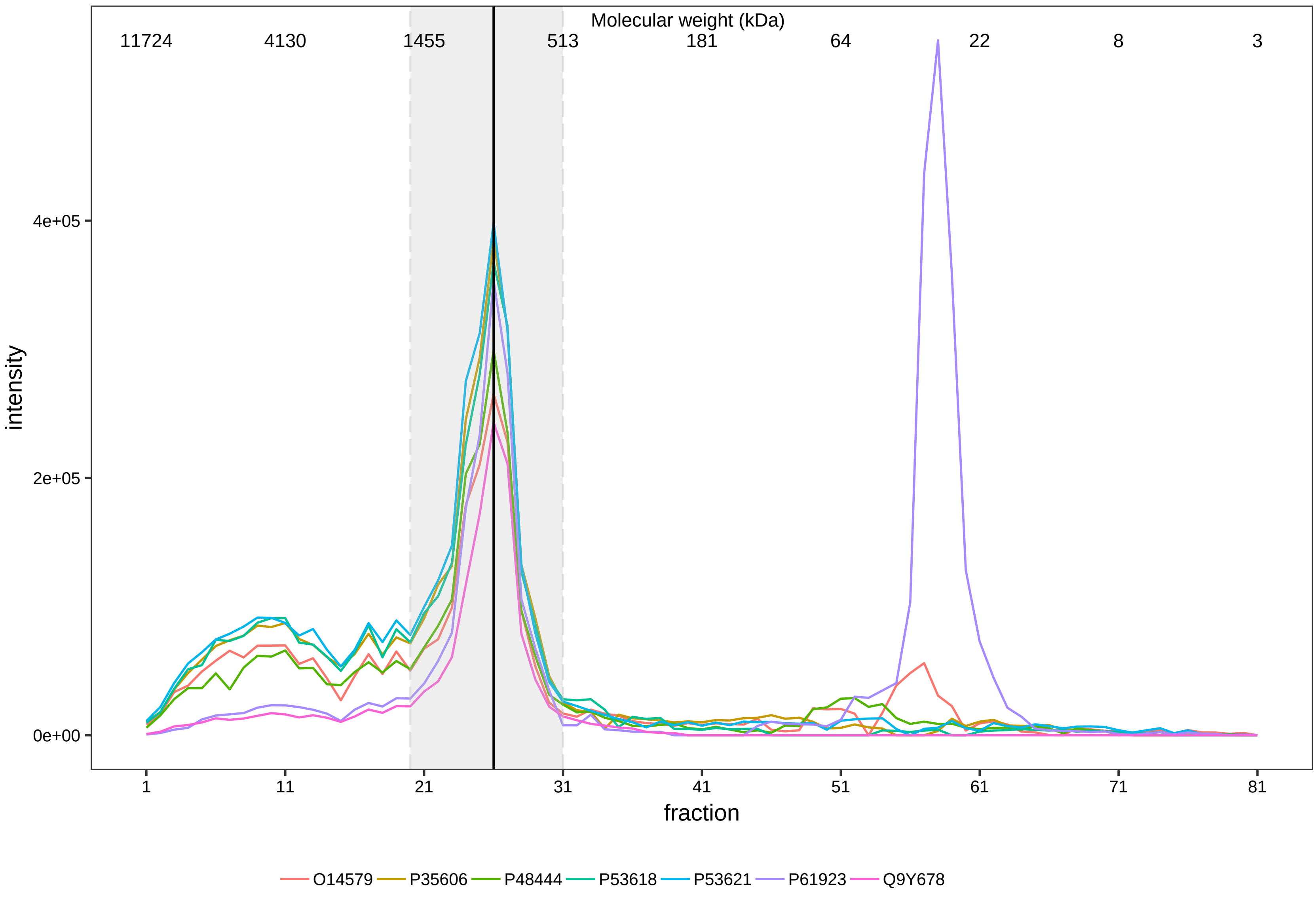

Feature ID 380

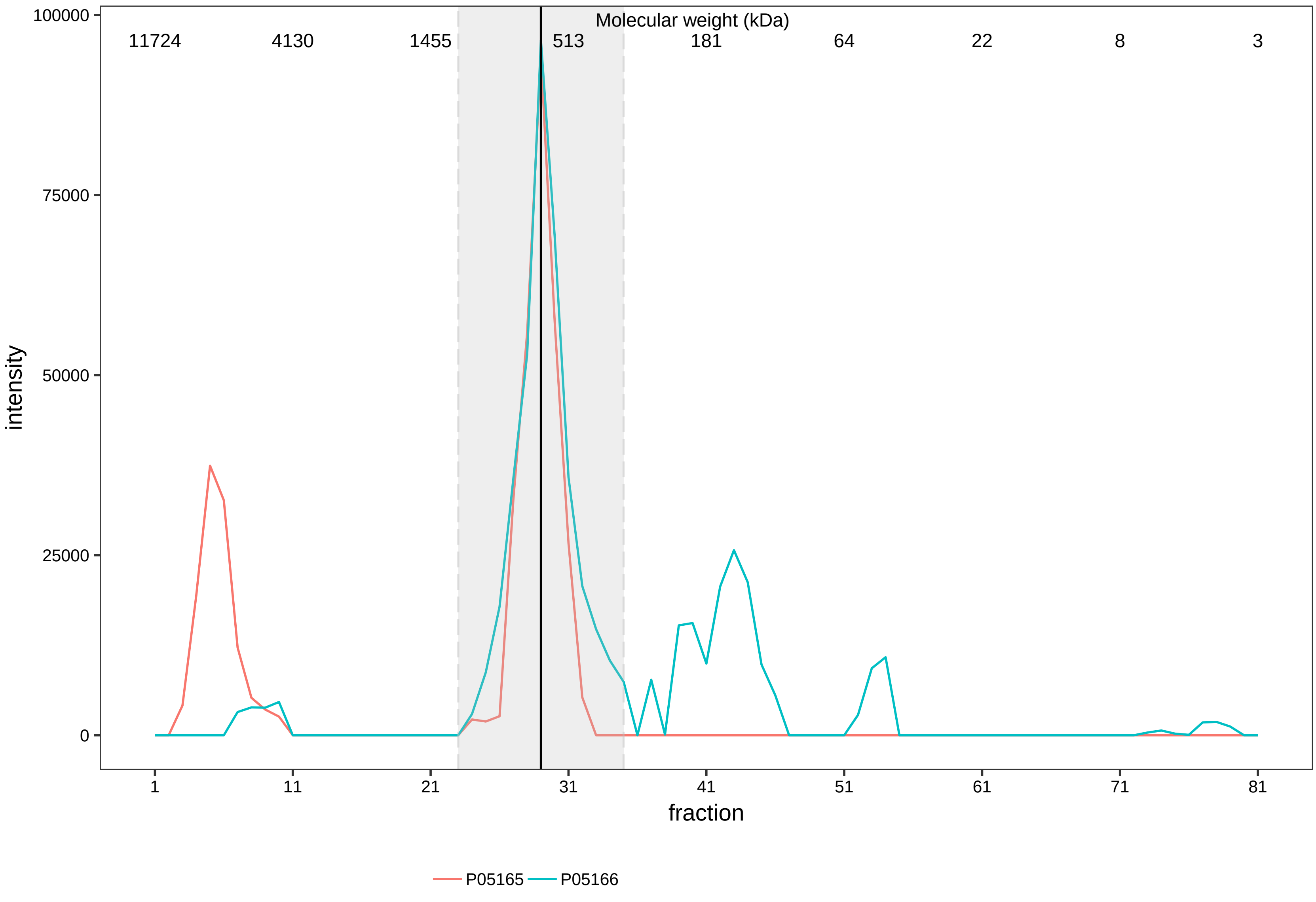

Feature ID 381

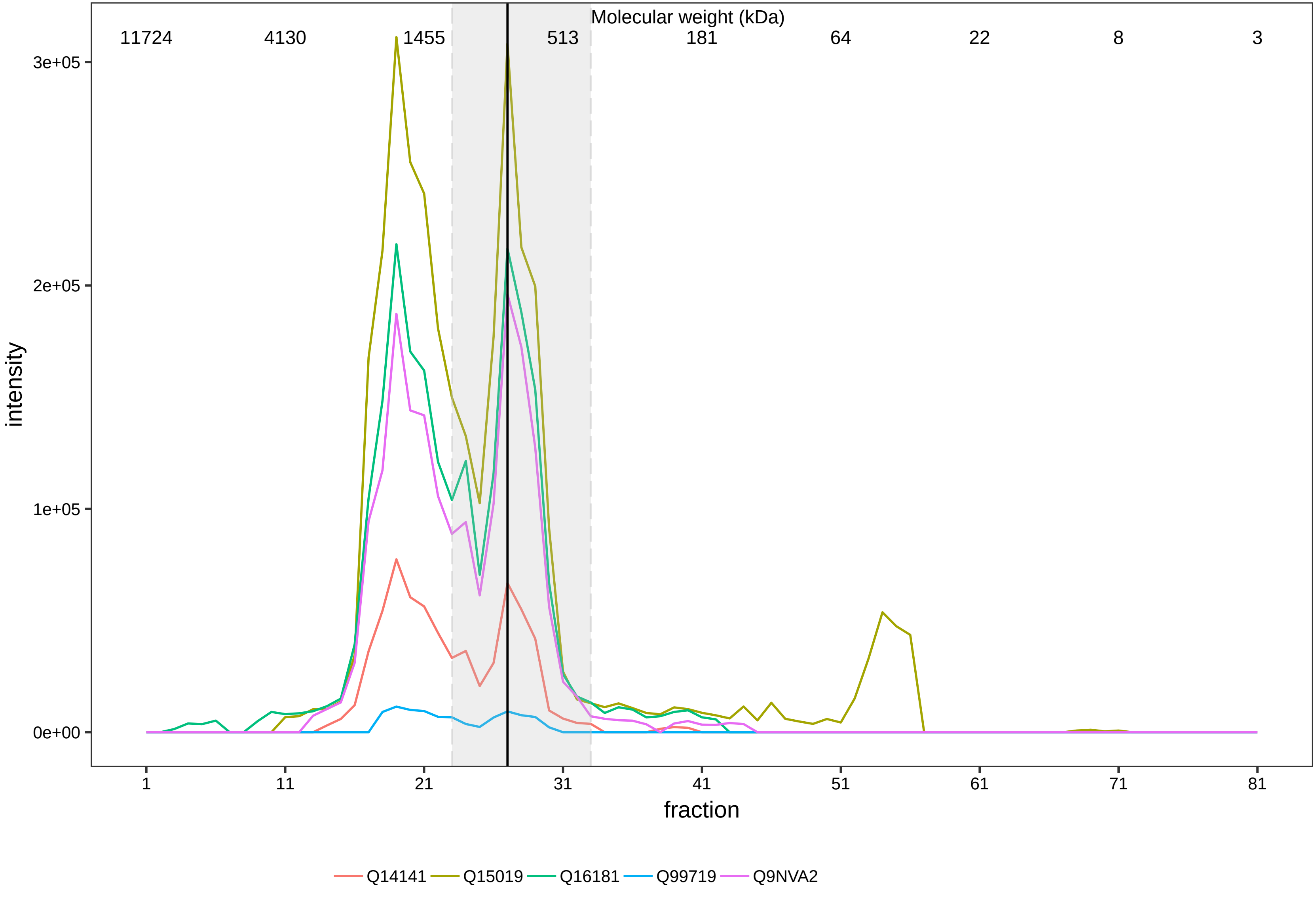

Feature ID 382

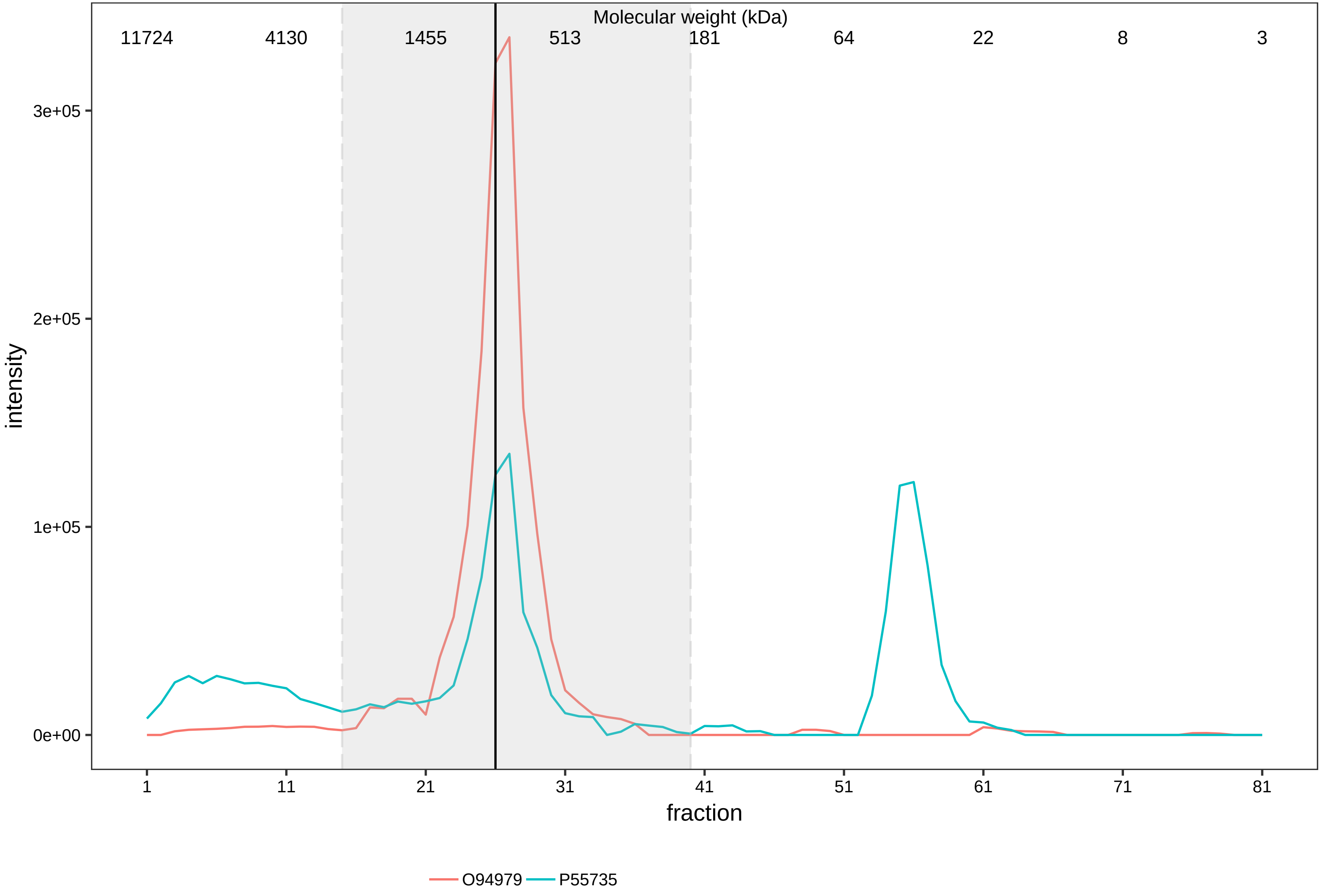

Feature ID 383

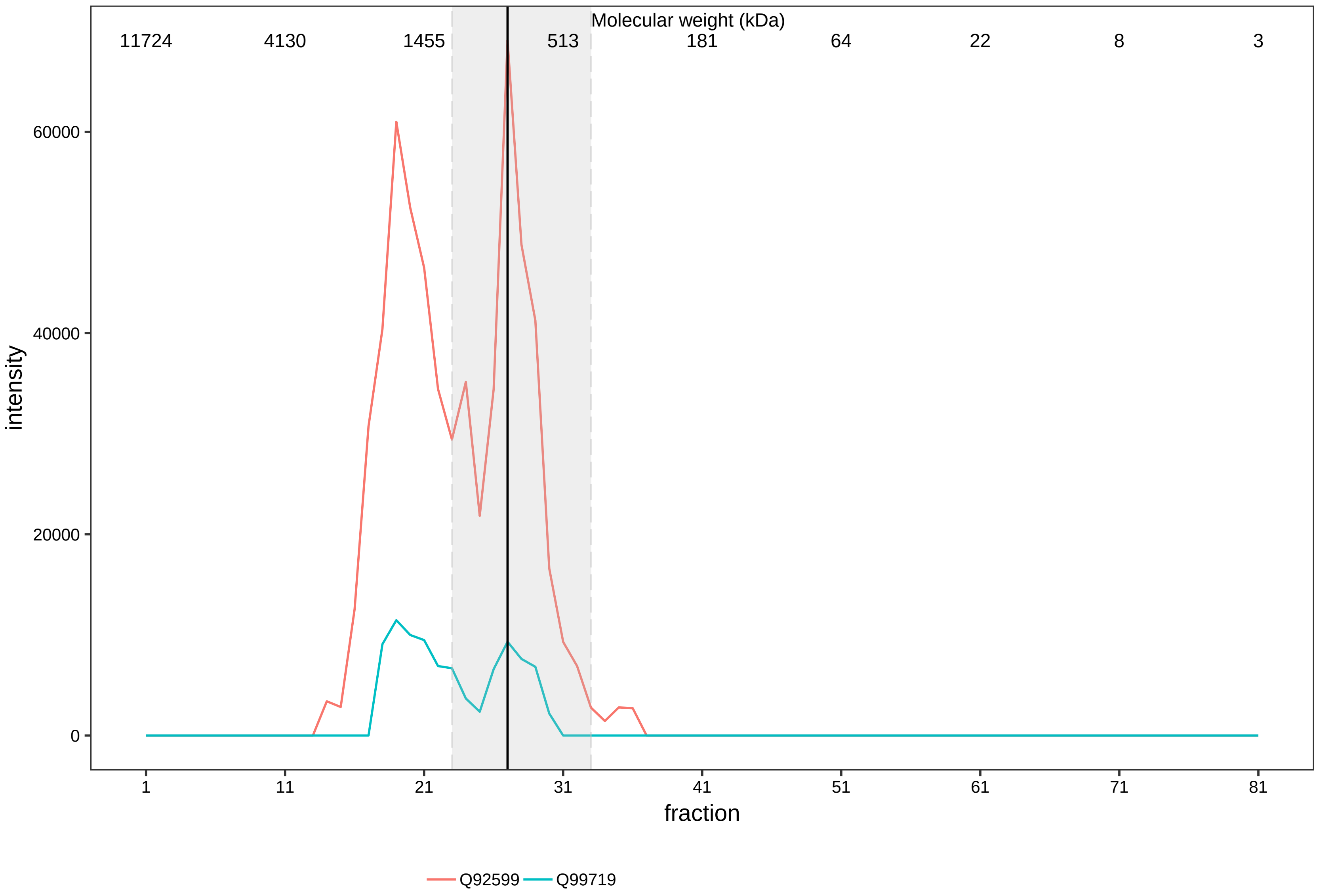

Feature ID 384

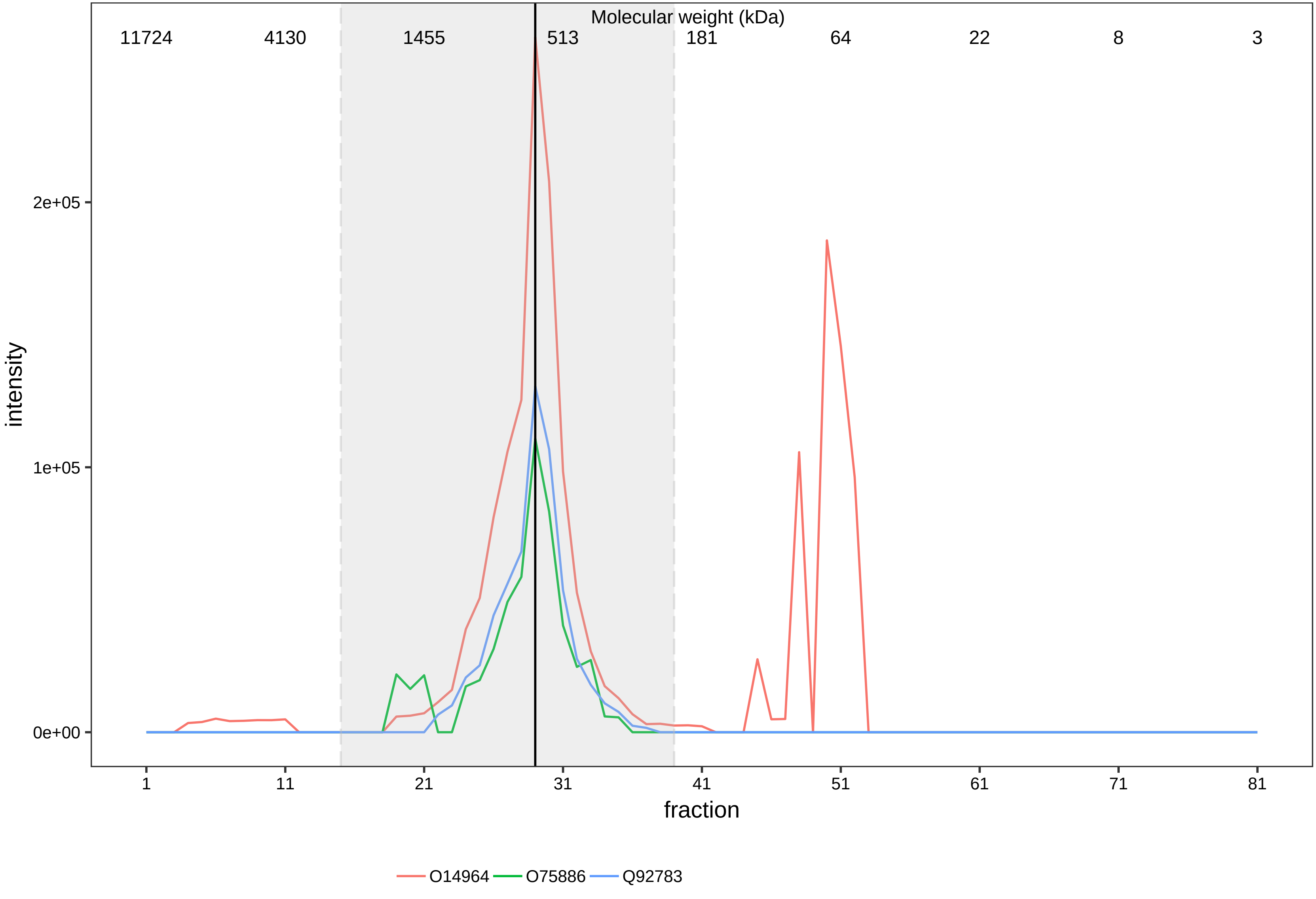

Feature ID 385

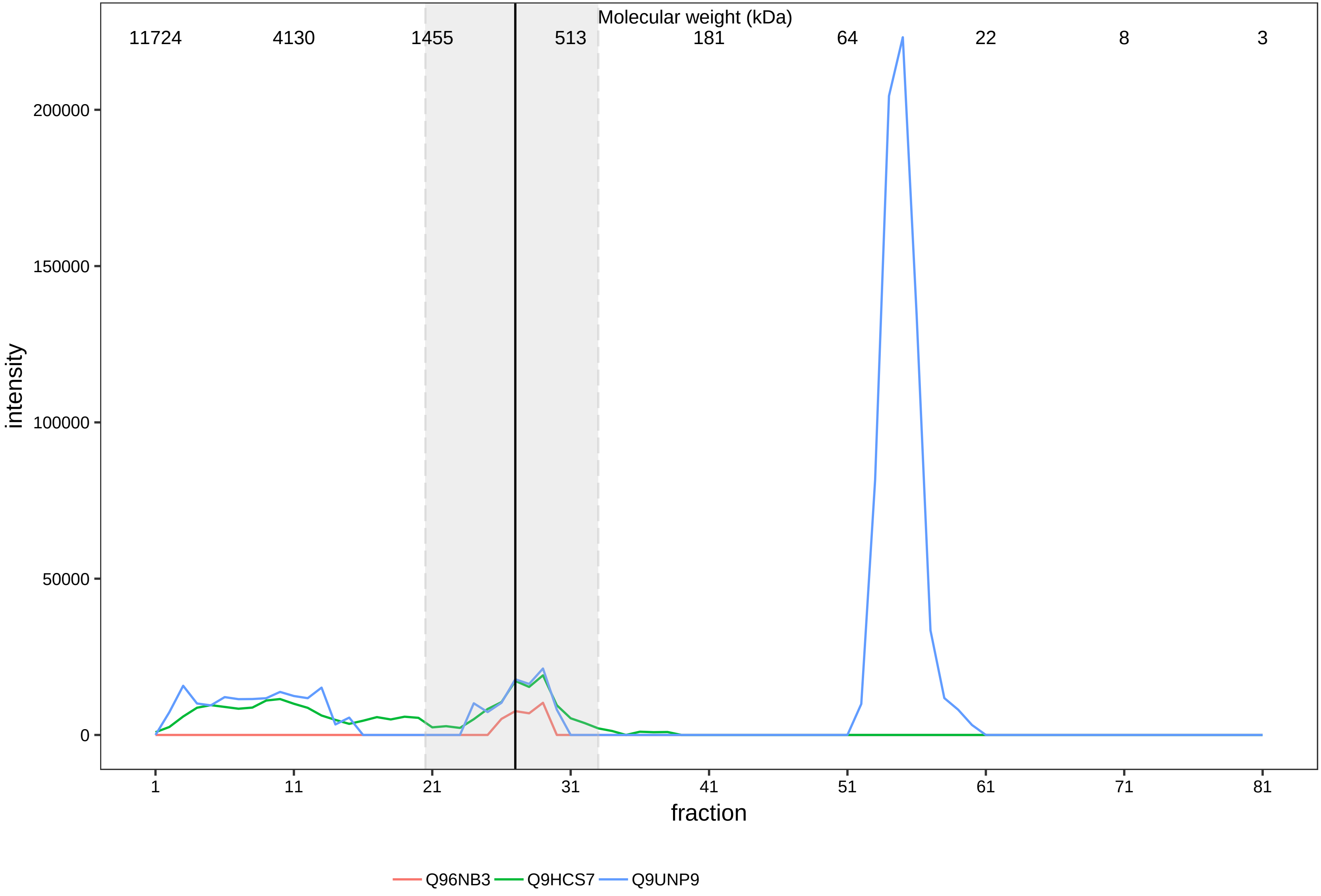

Feature ID 386

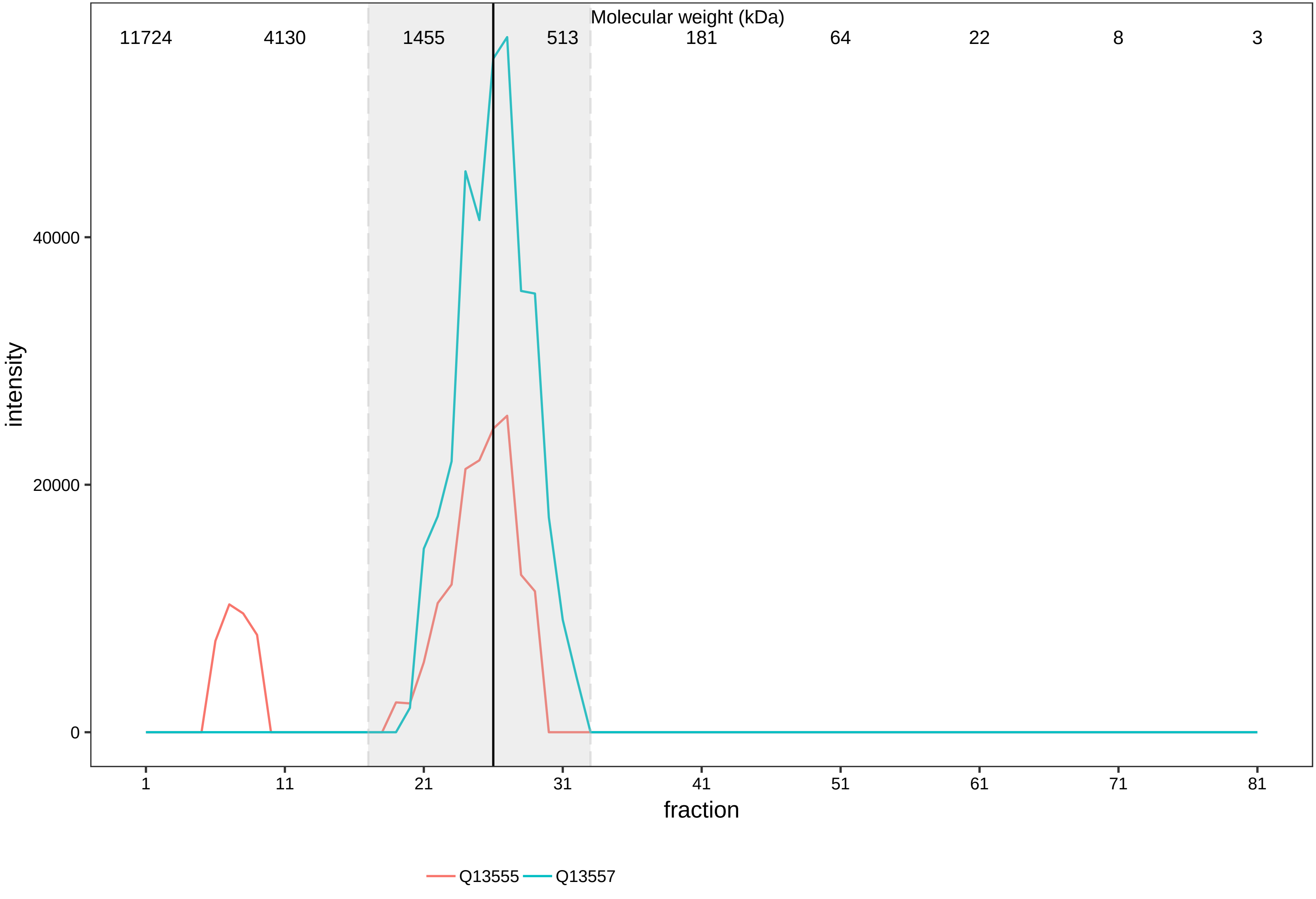

Feature ID 387

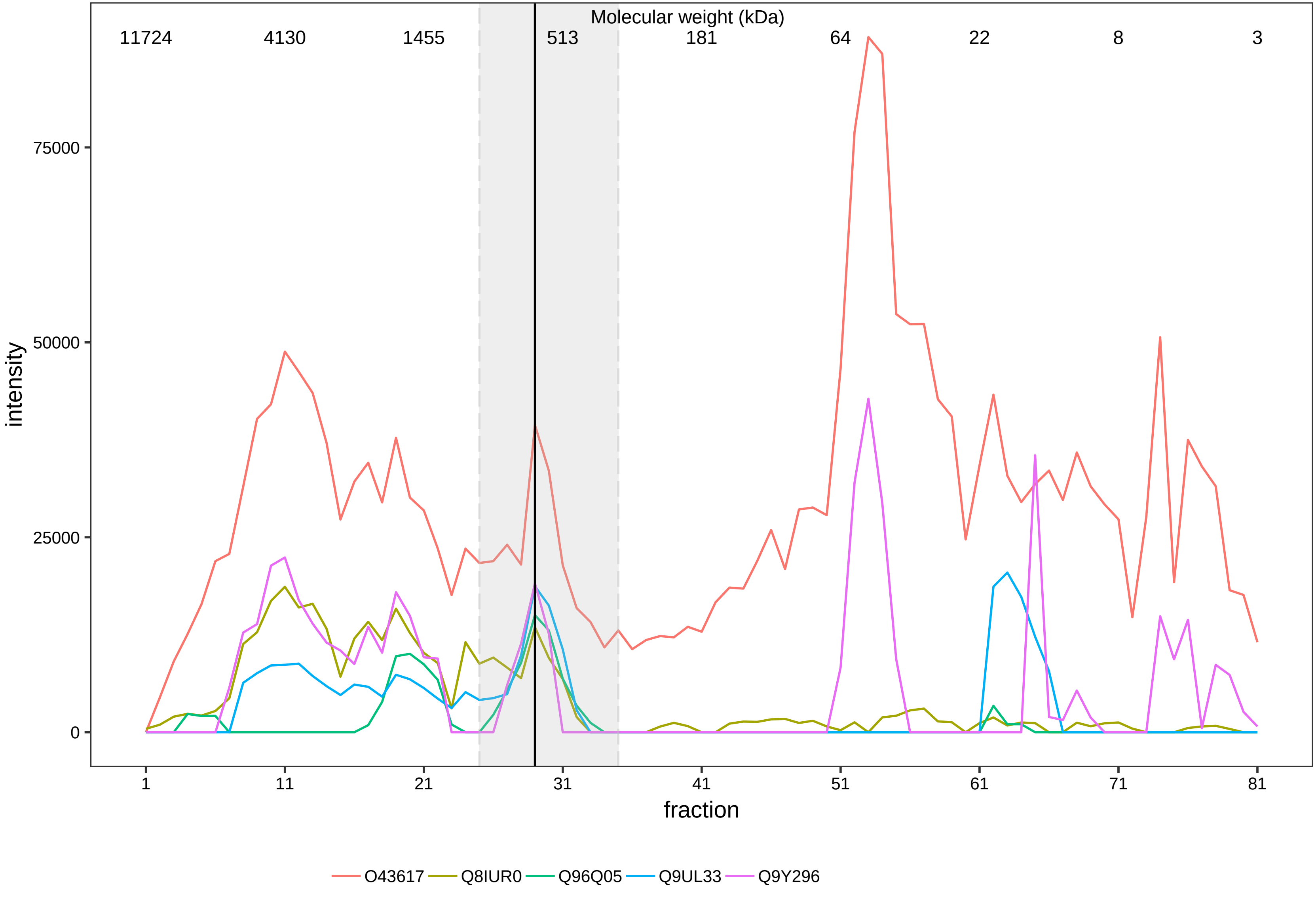

Feature ID 388

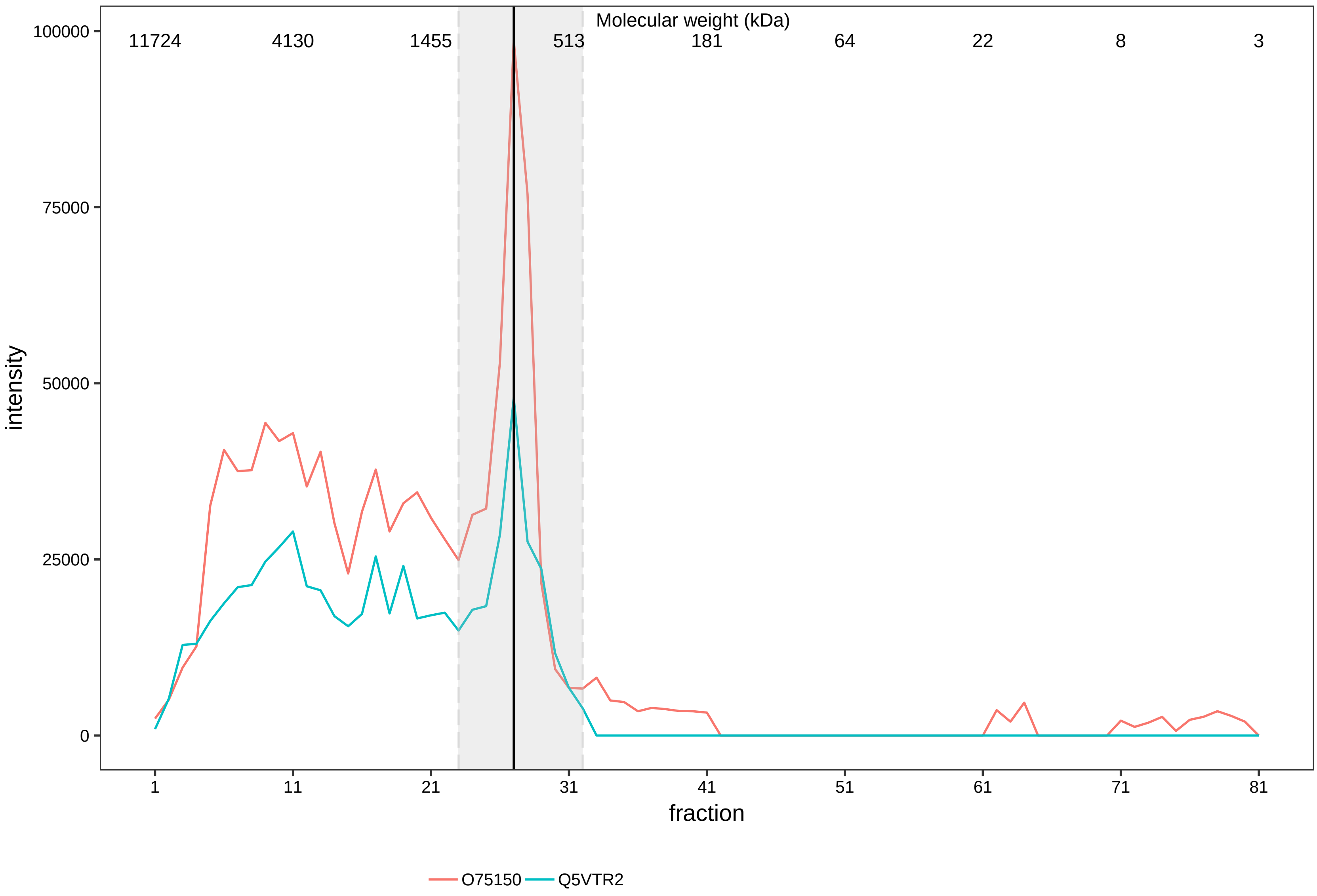

Feature ID 389

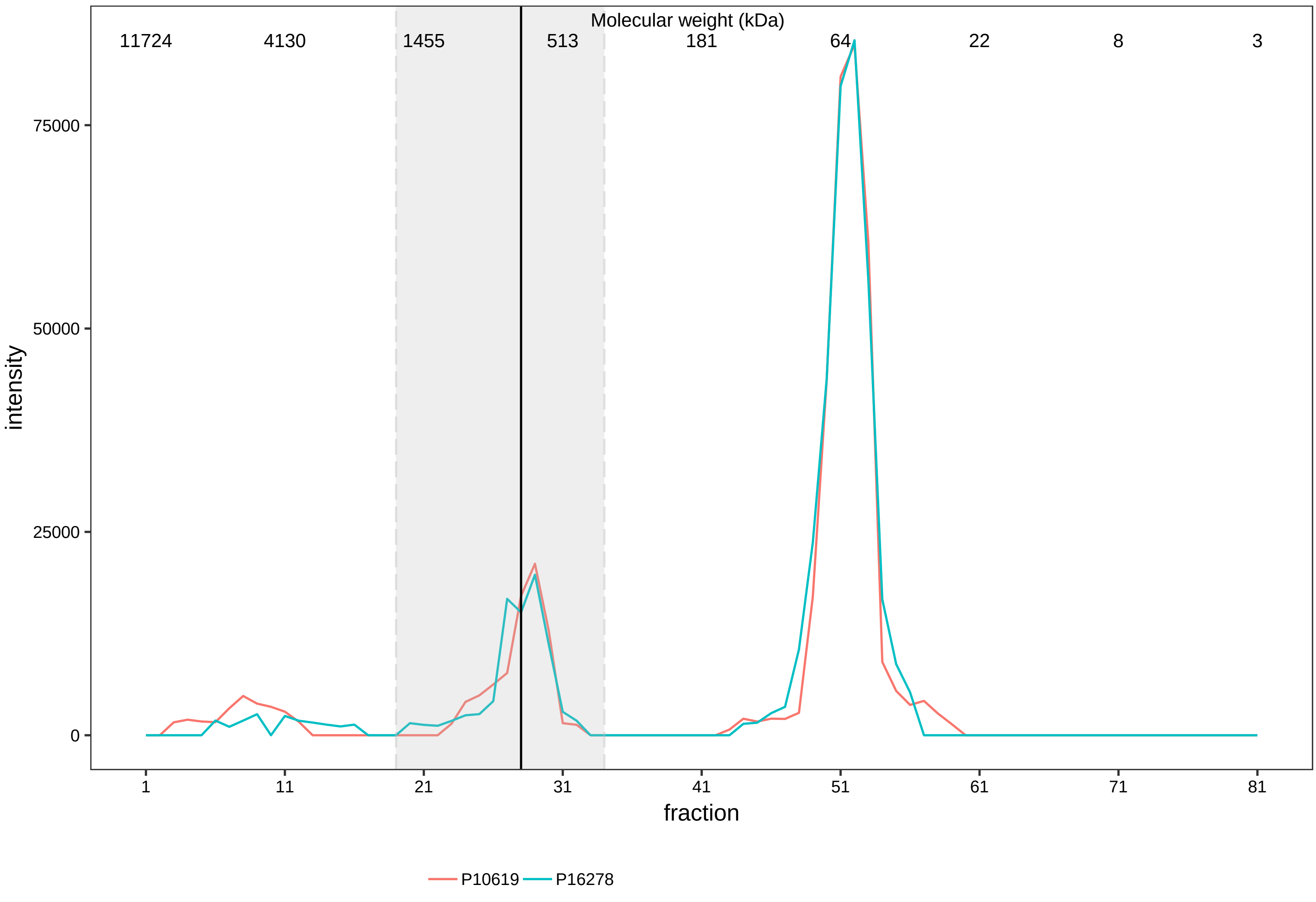

Feature ID 390

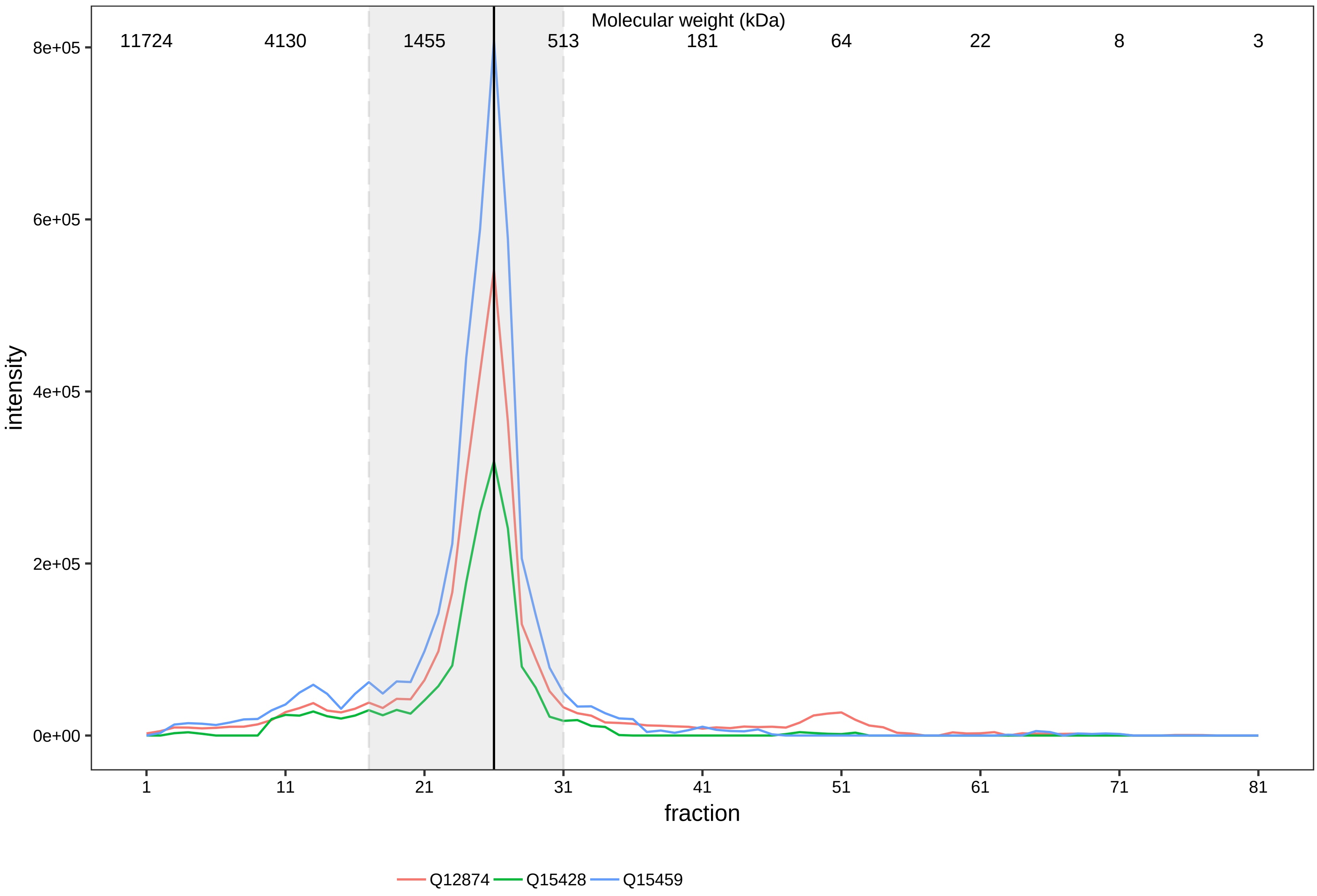

Feature ID 391

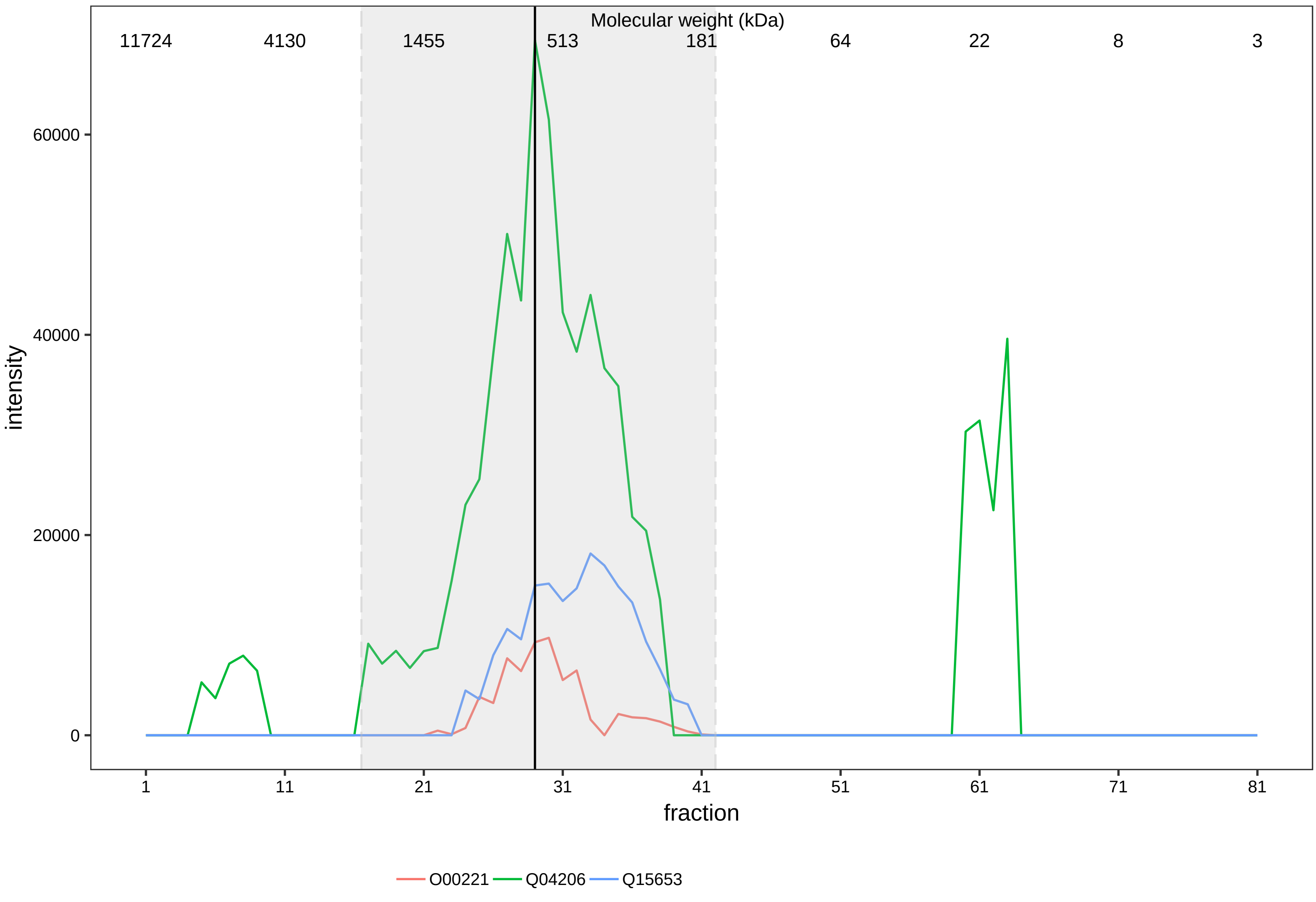

Feature ID 392

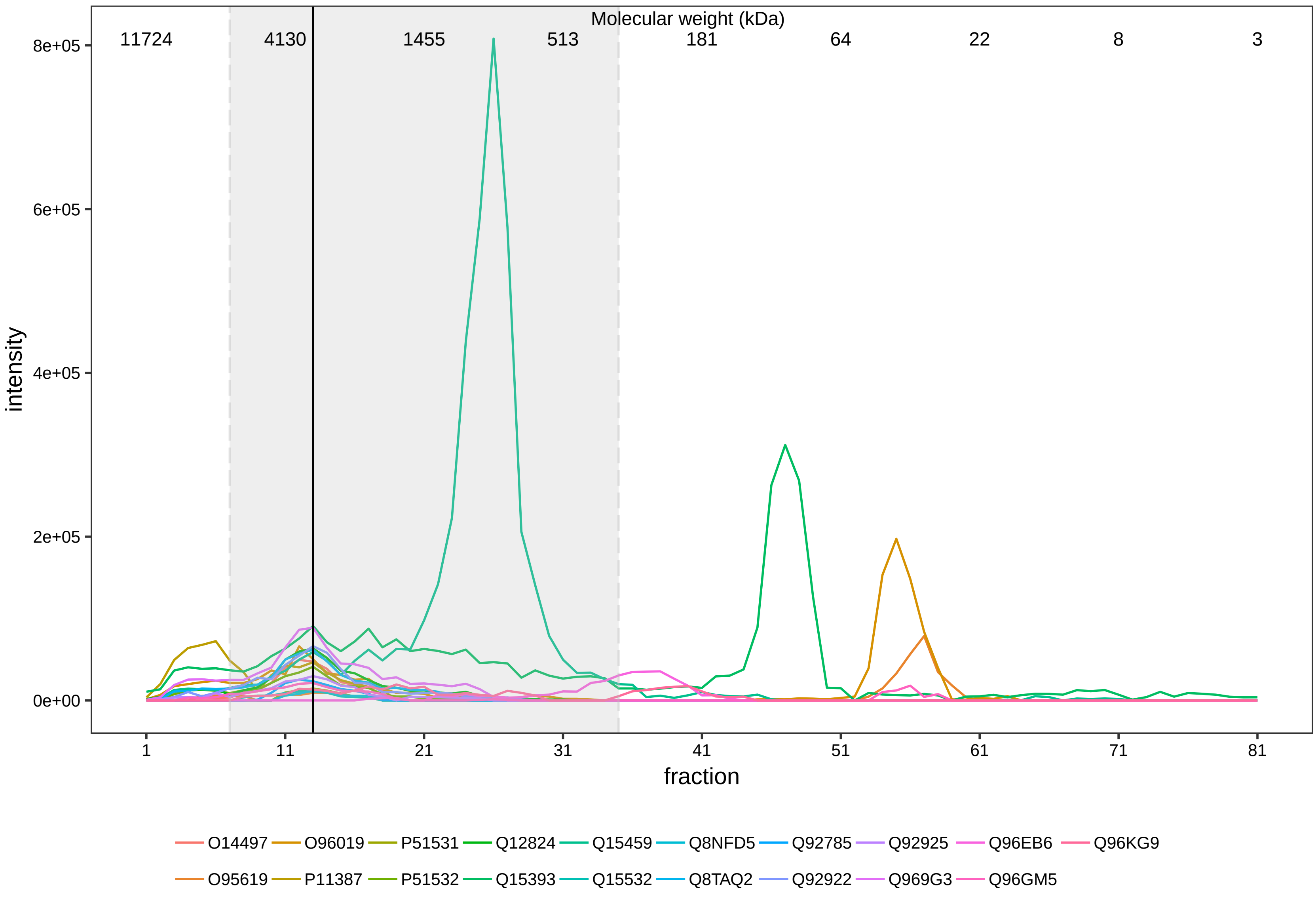

Feature ID 393

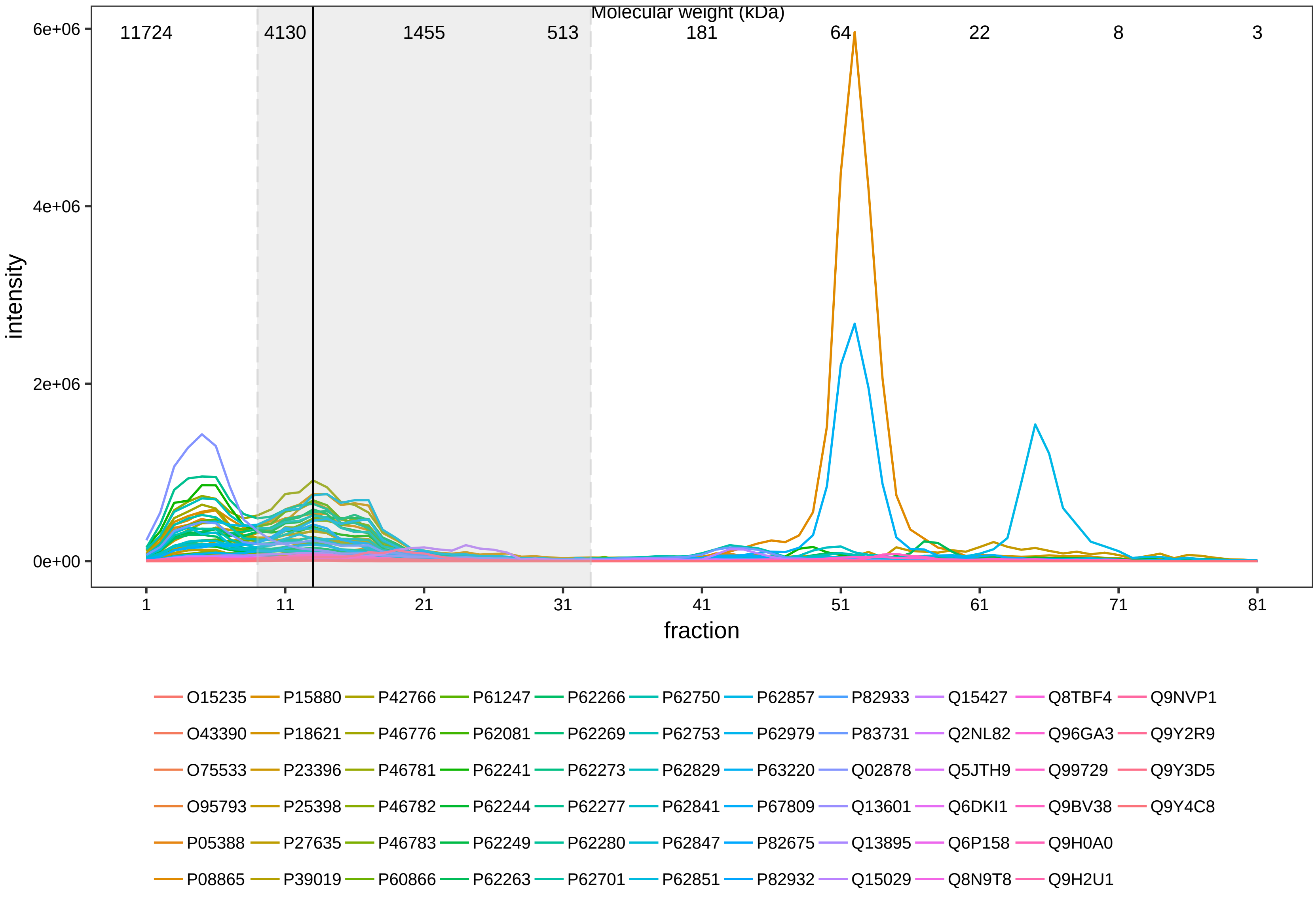

Feature ID 394

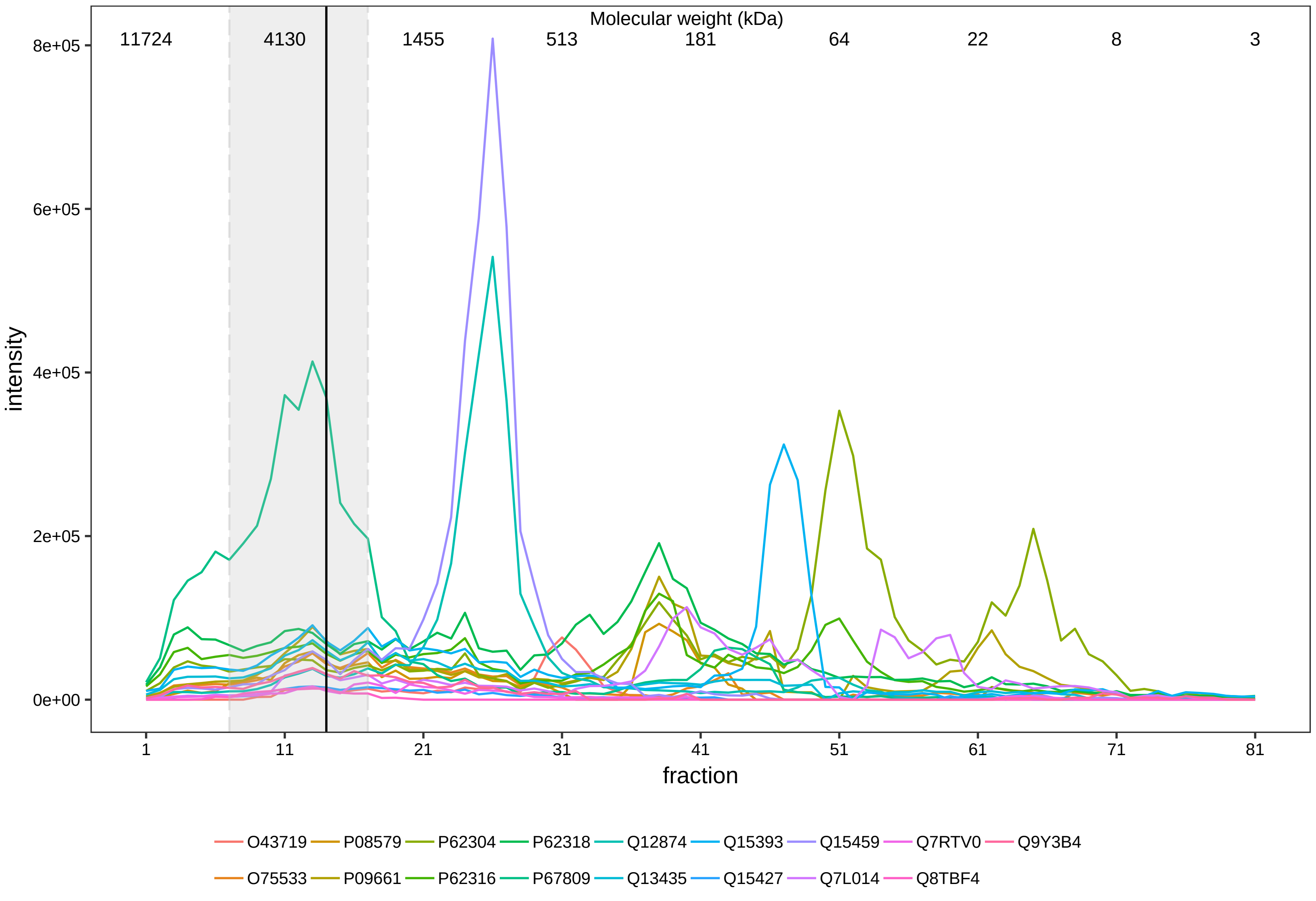

Feature ID 395

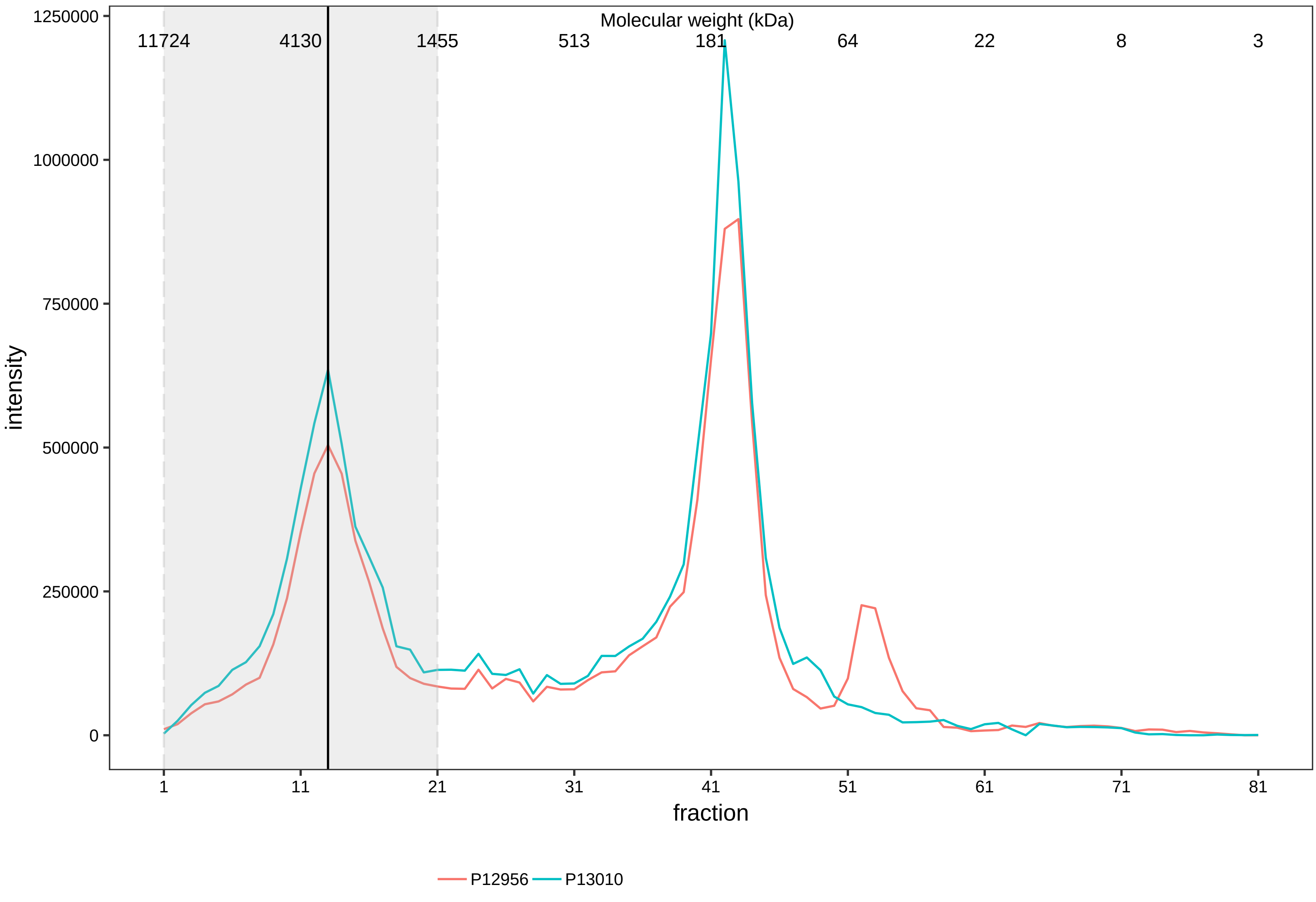

Feature ID 396

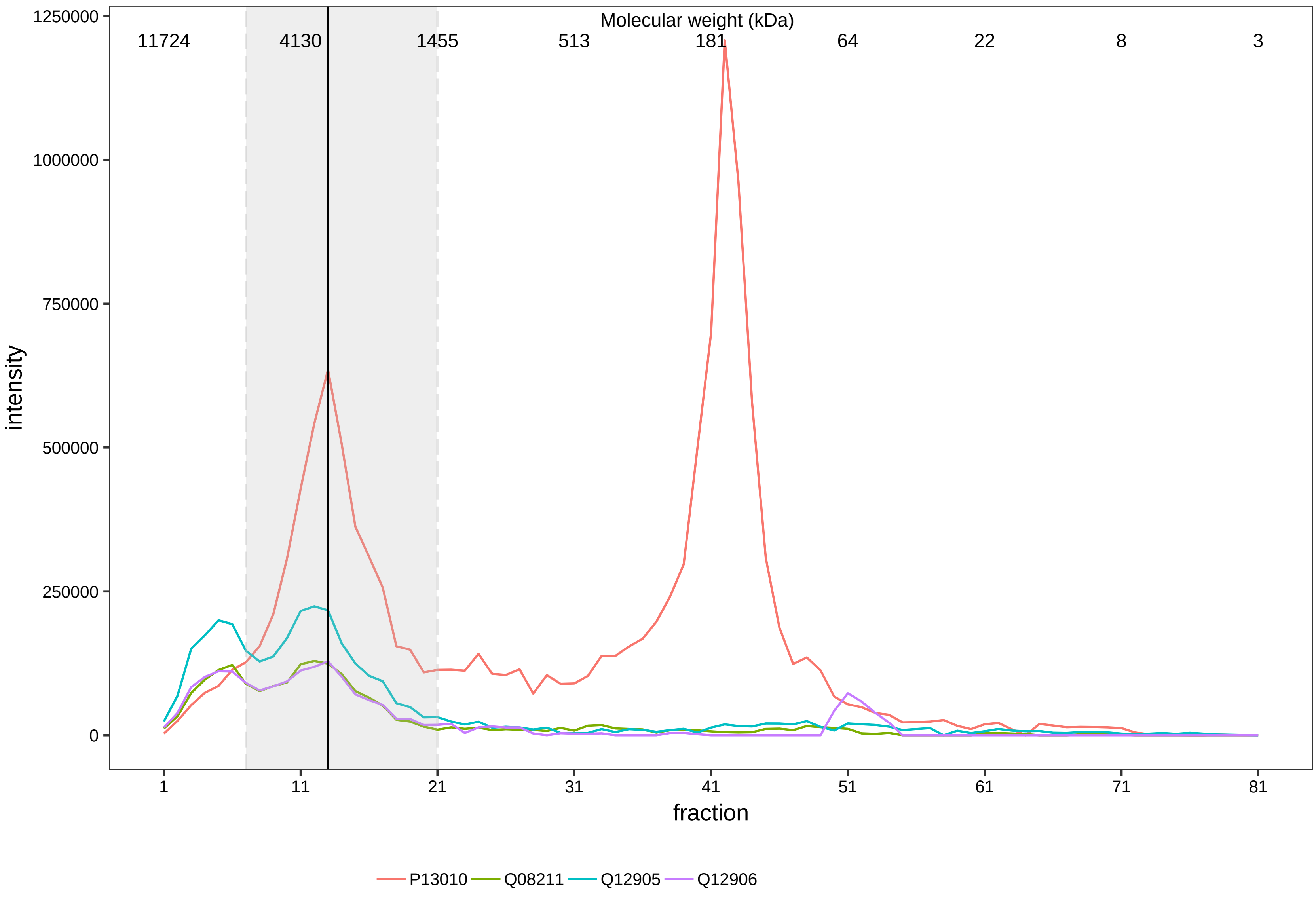

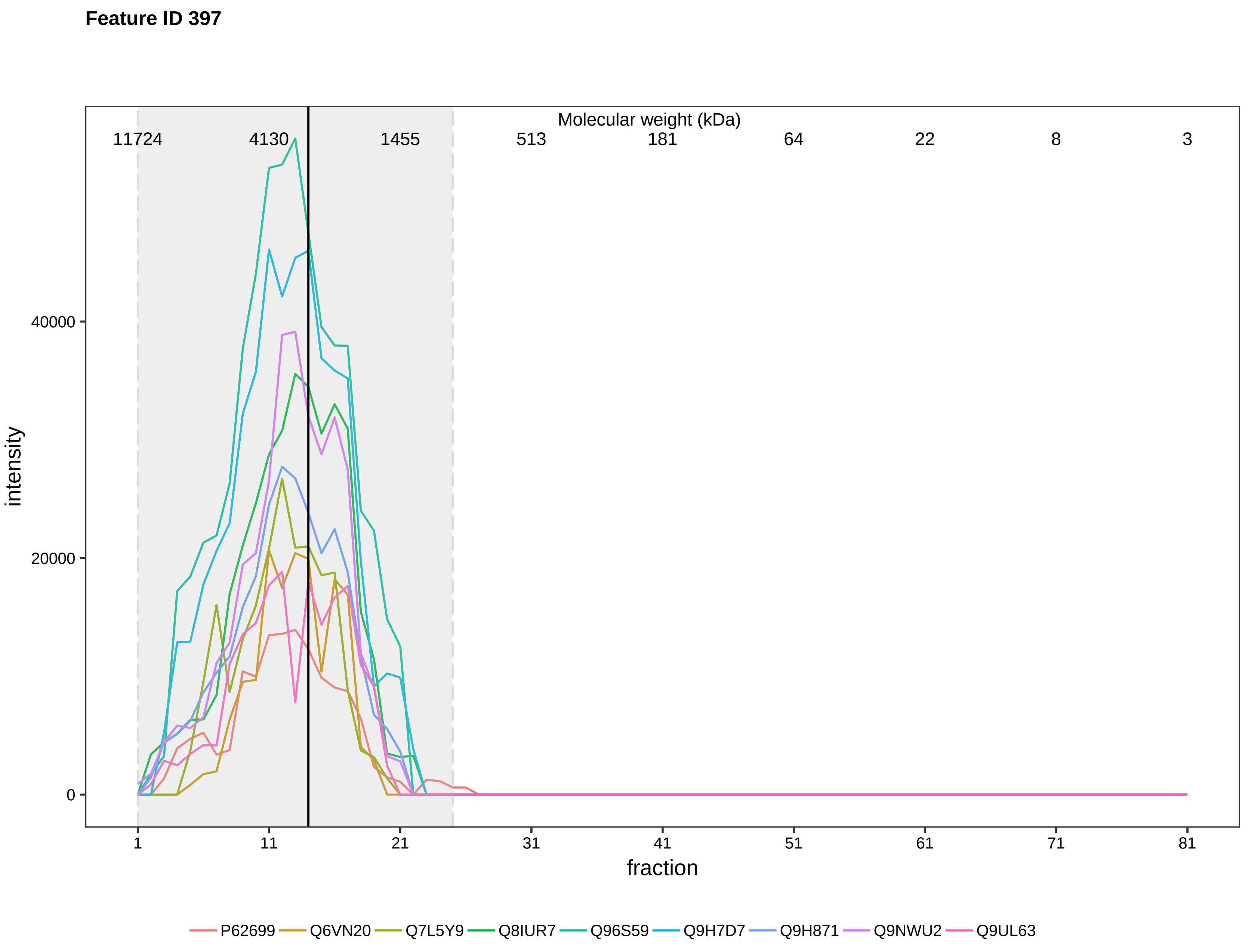

Feature ID 397
Molecular weight (kDa)
11724
4130
1455
513
181
64
22
8
3
intensity
40000
20000
0
1
11
21
31
41
51
61
71
81
fraction
P62699
Q6VN20
Q7L5Y9
Q8IUR7
Q96S59
Q9H7D7
Q9H871
Q9NWU2
Q9UL63

Feature ID 398

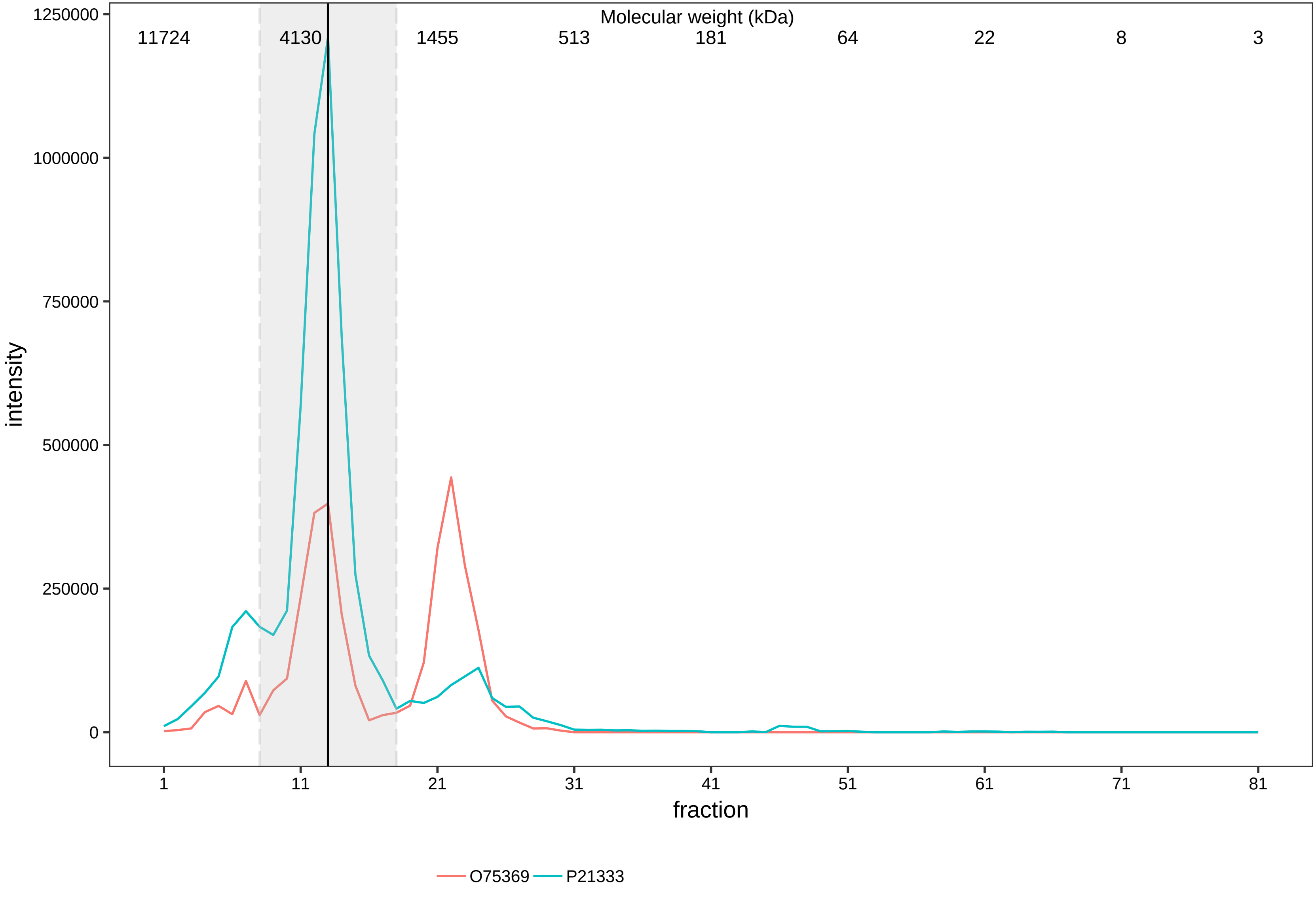

Feature ID 399

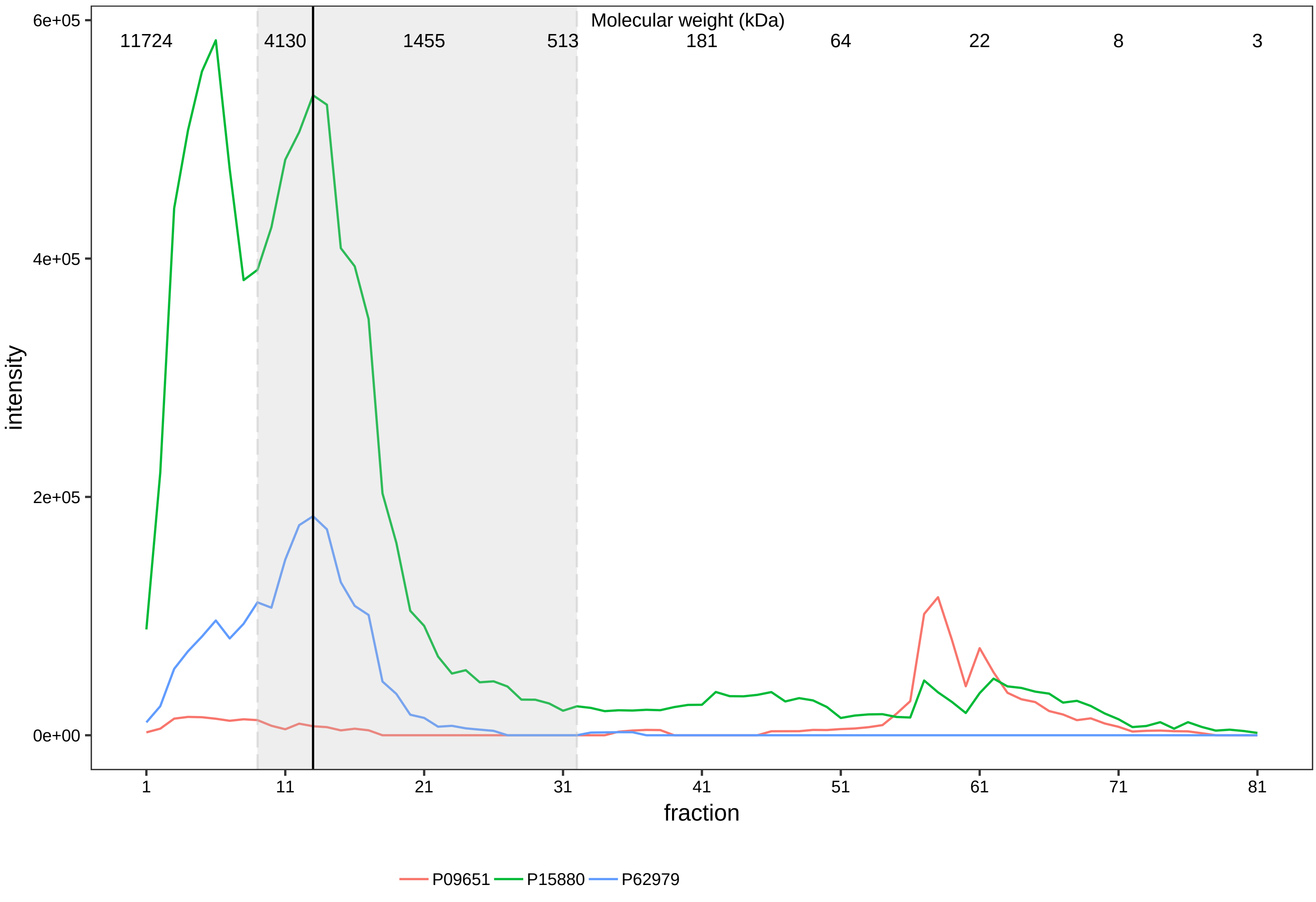

Feature ID 400

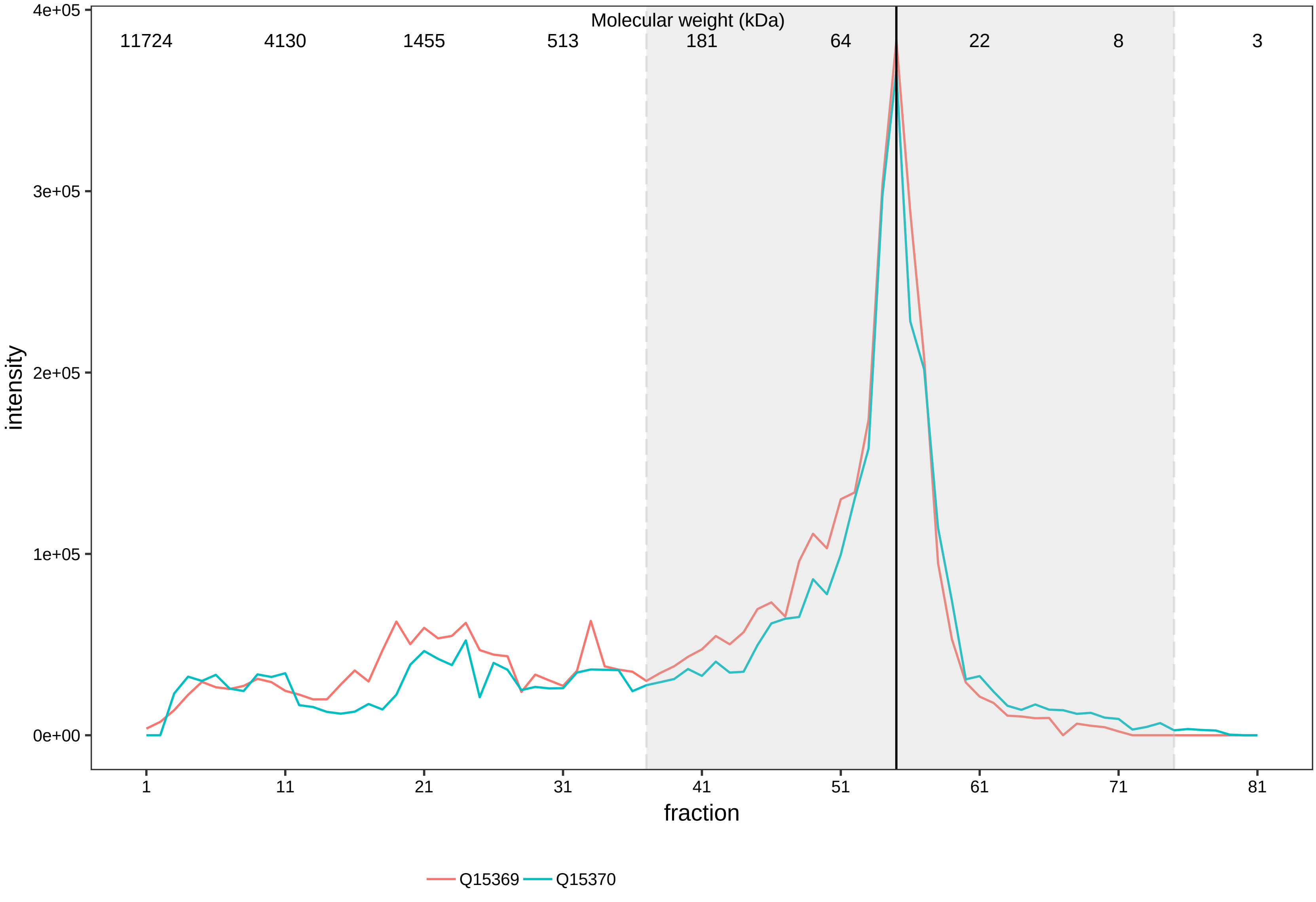

Feature ID 401

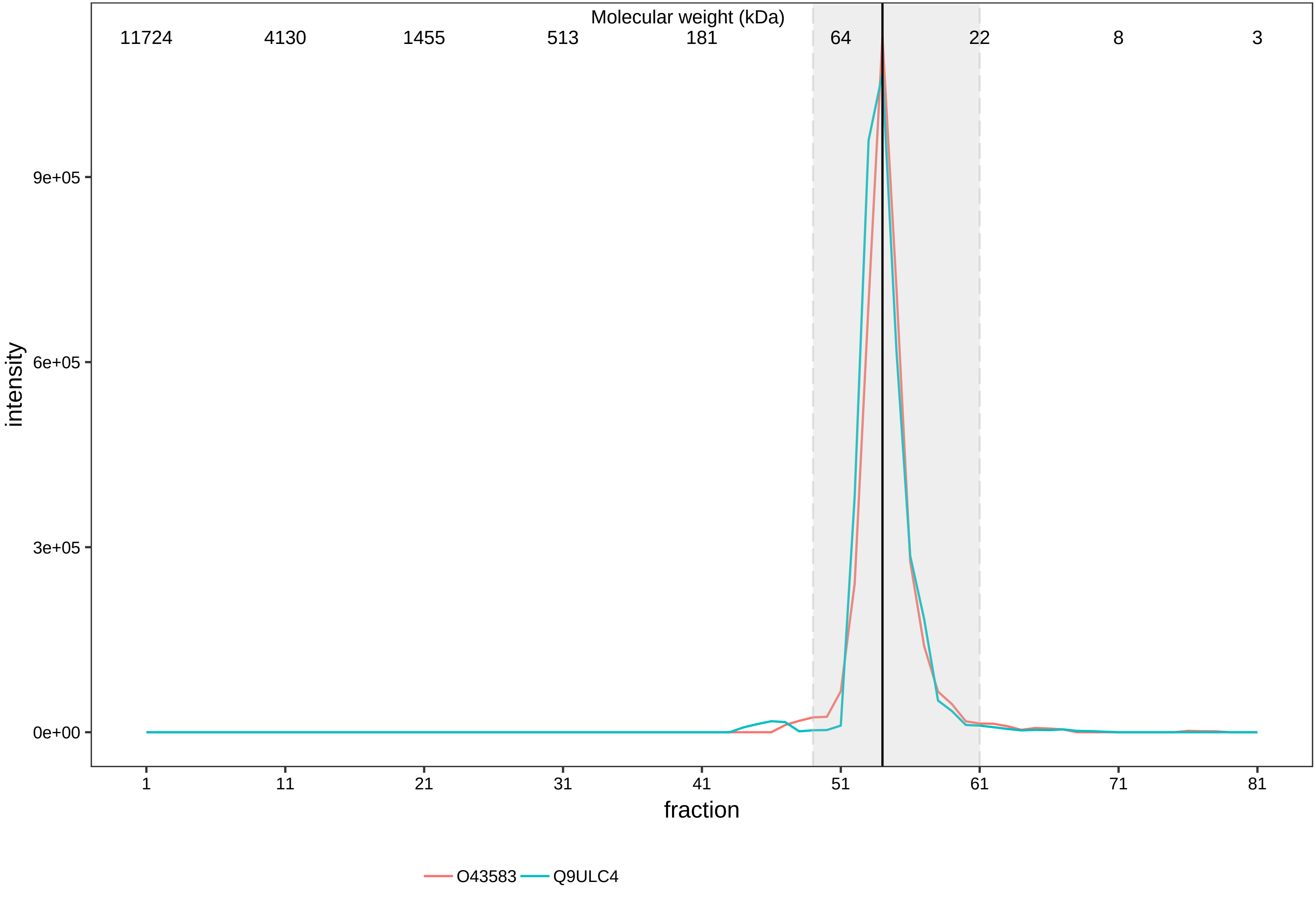

Feature ID 402

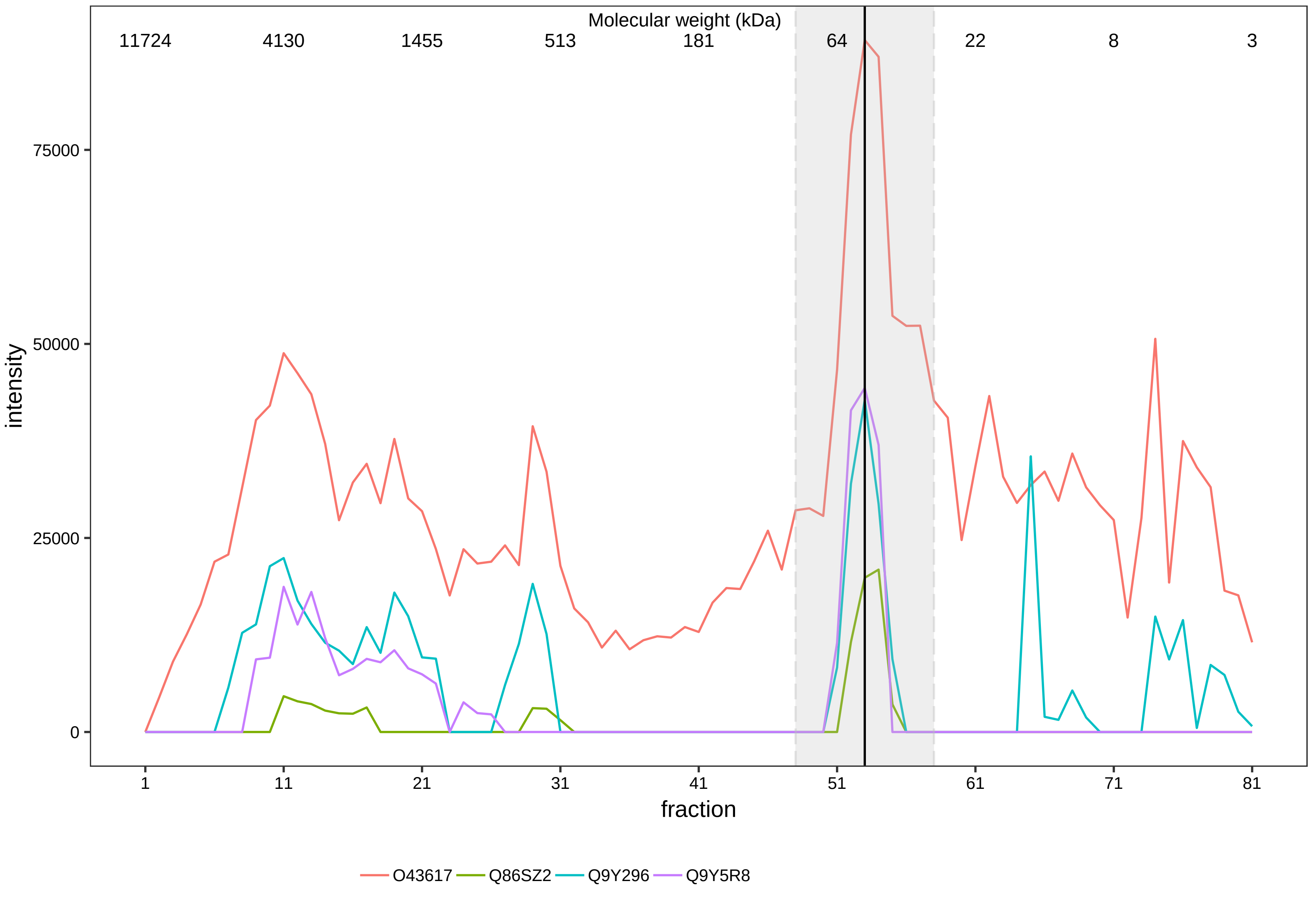

Feature ID 403

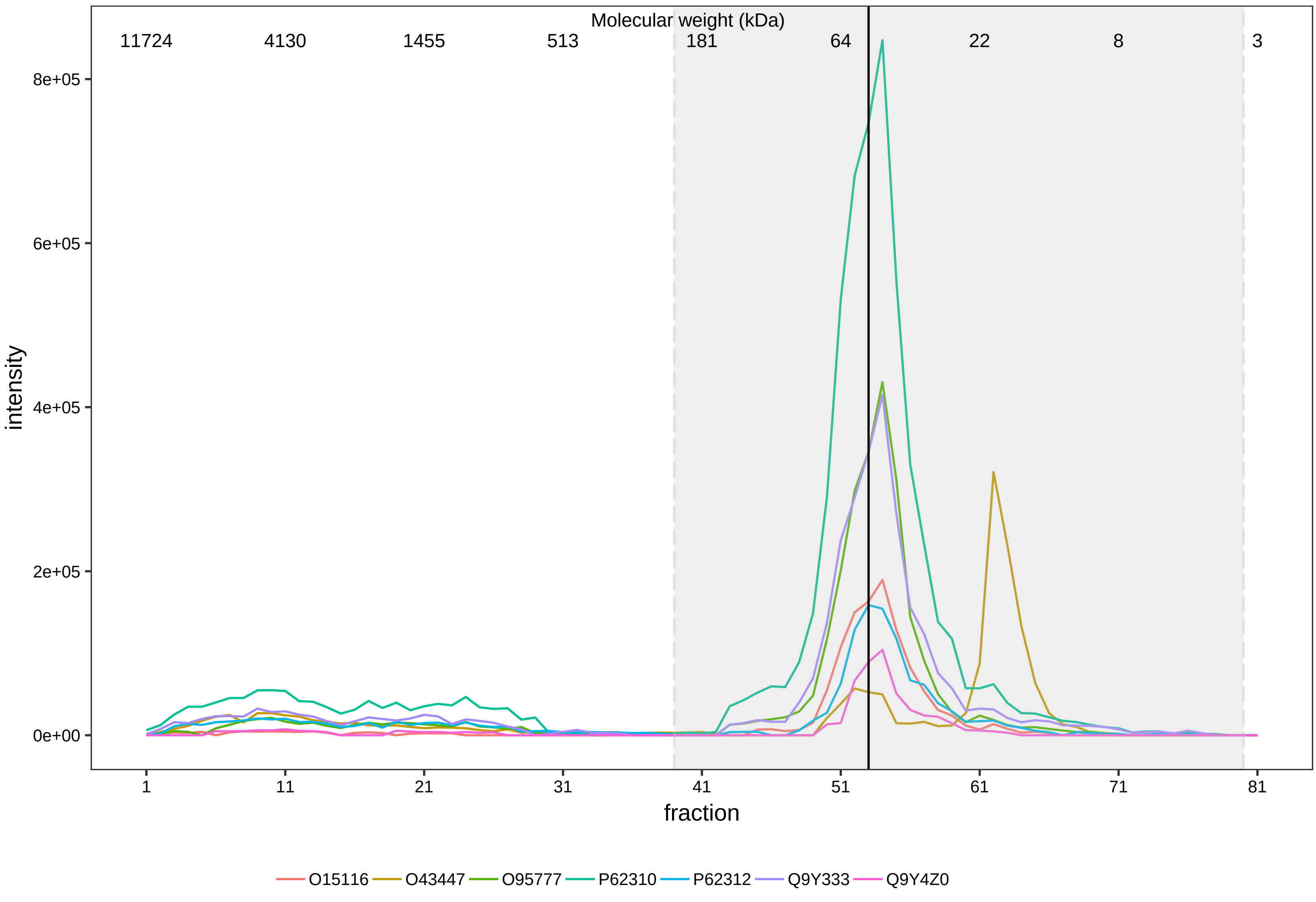

Feature ID 404

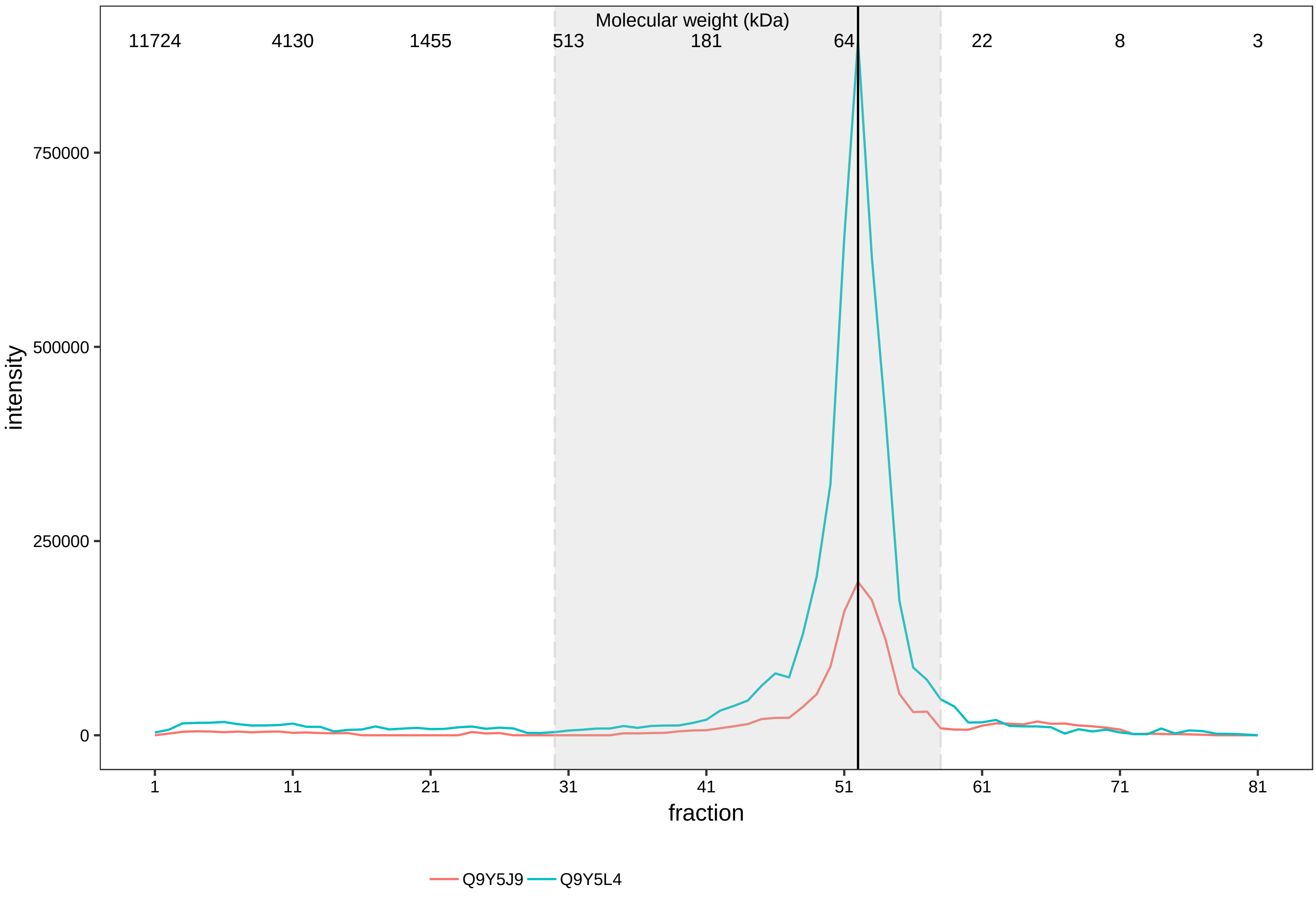

Feature ID 405

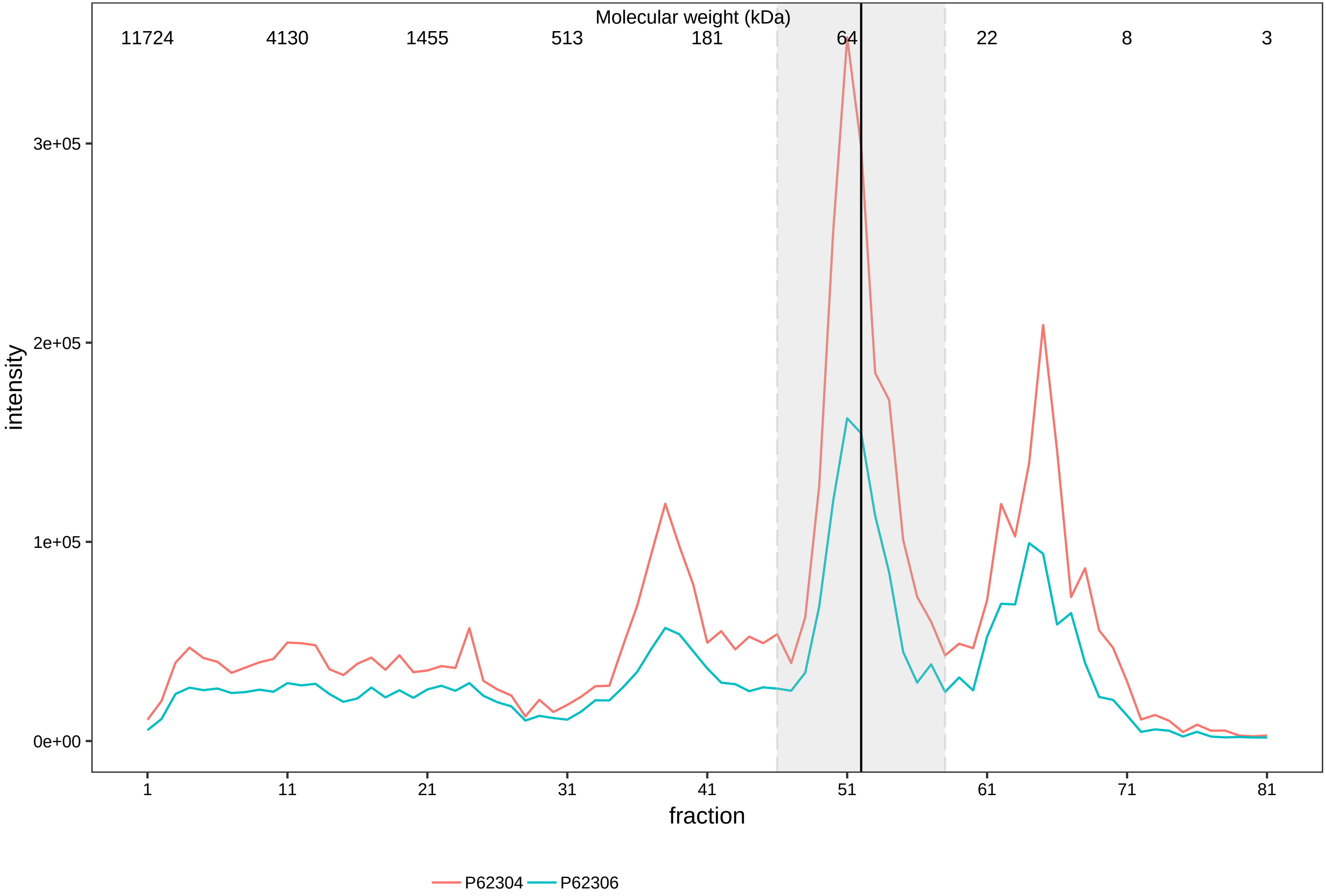

Feature ID 406

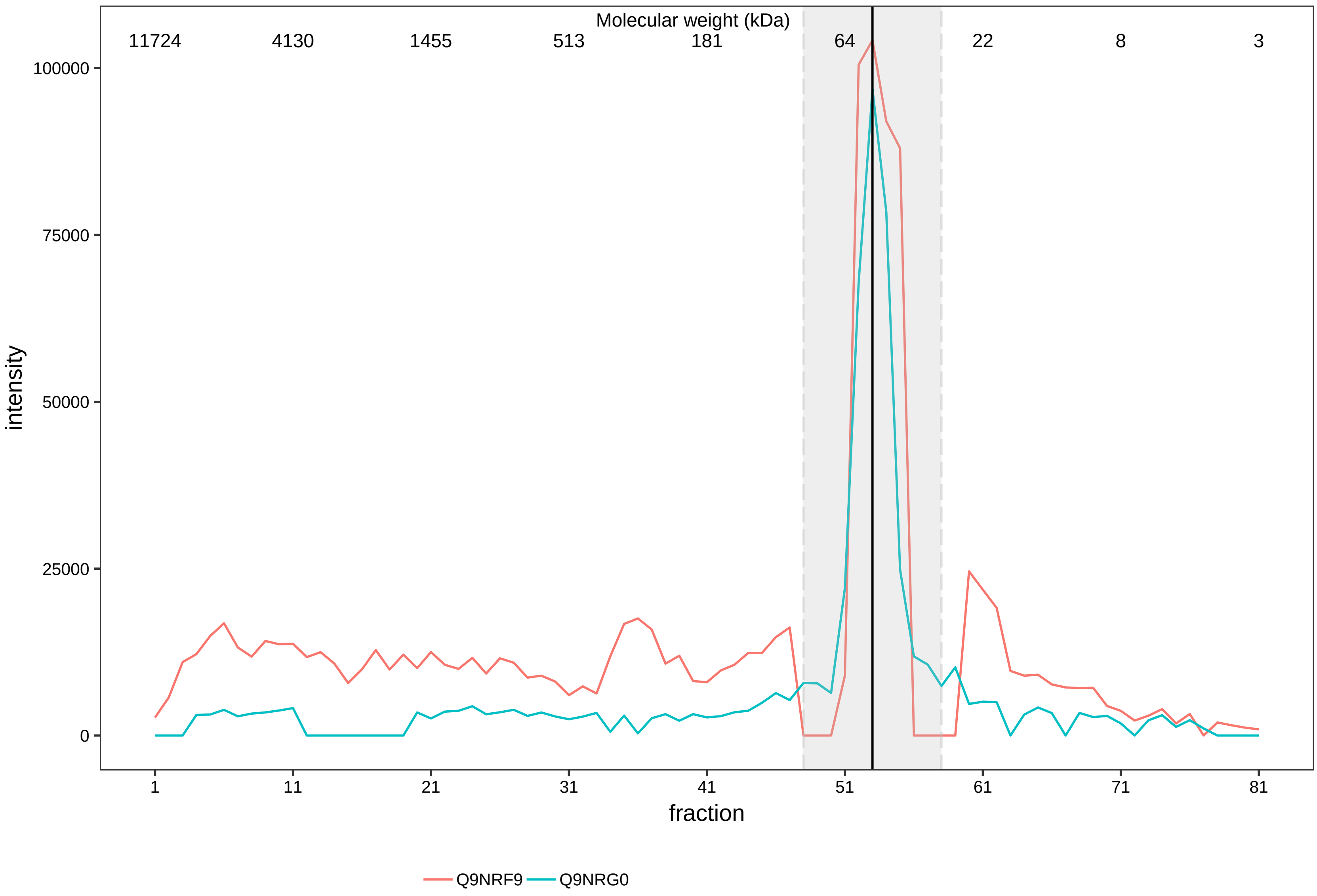

Feature ID 407

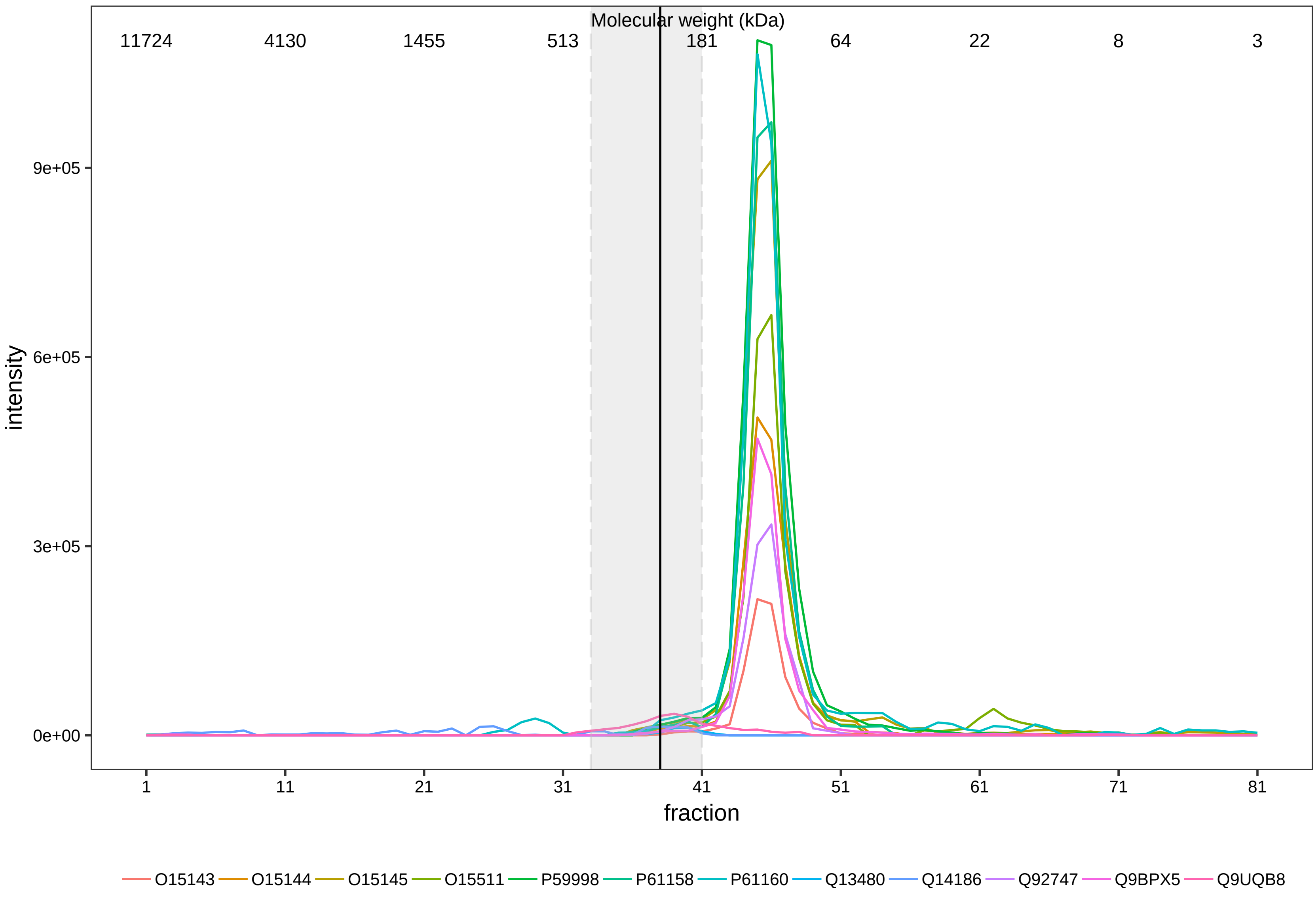

Feature ID 408

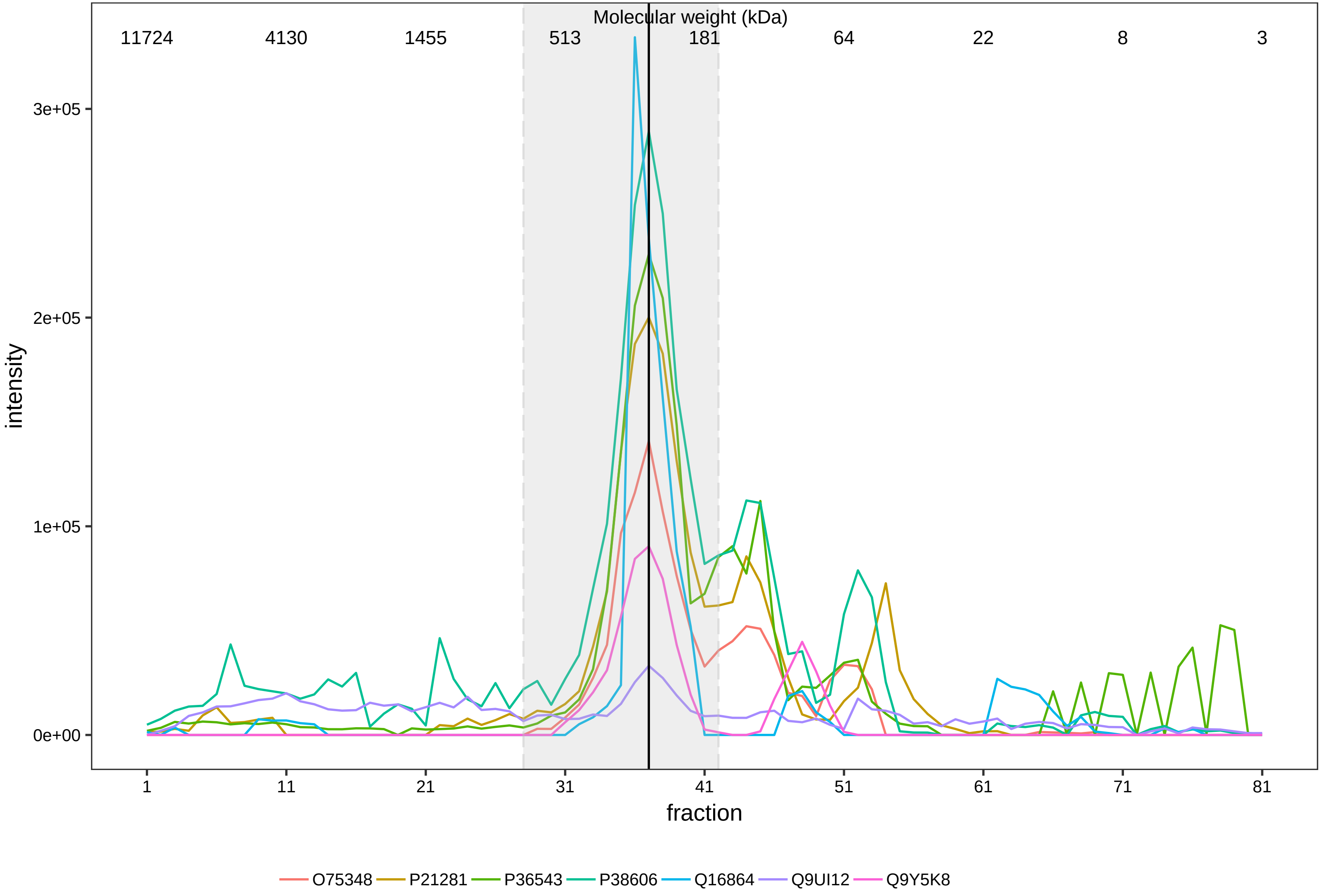

Feature ID 409

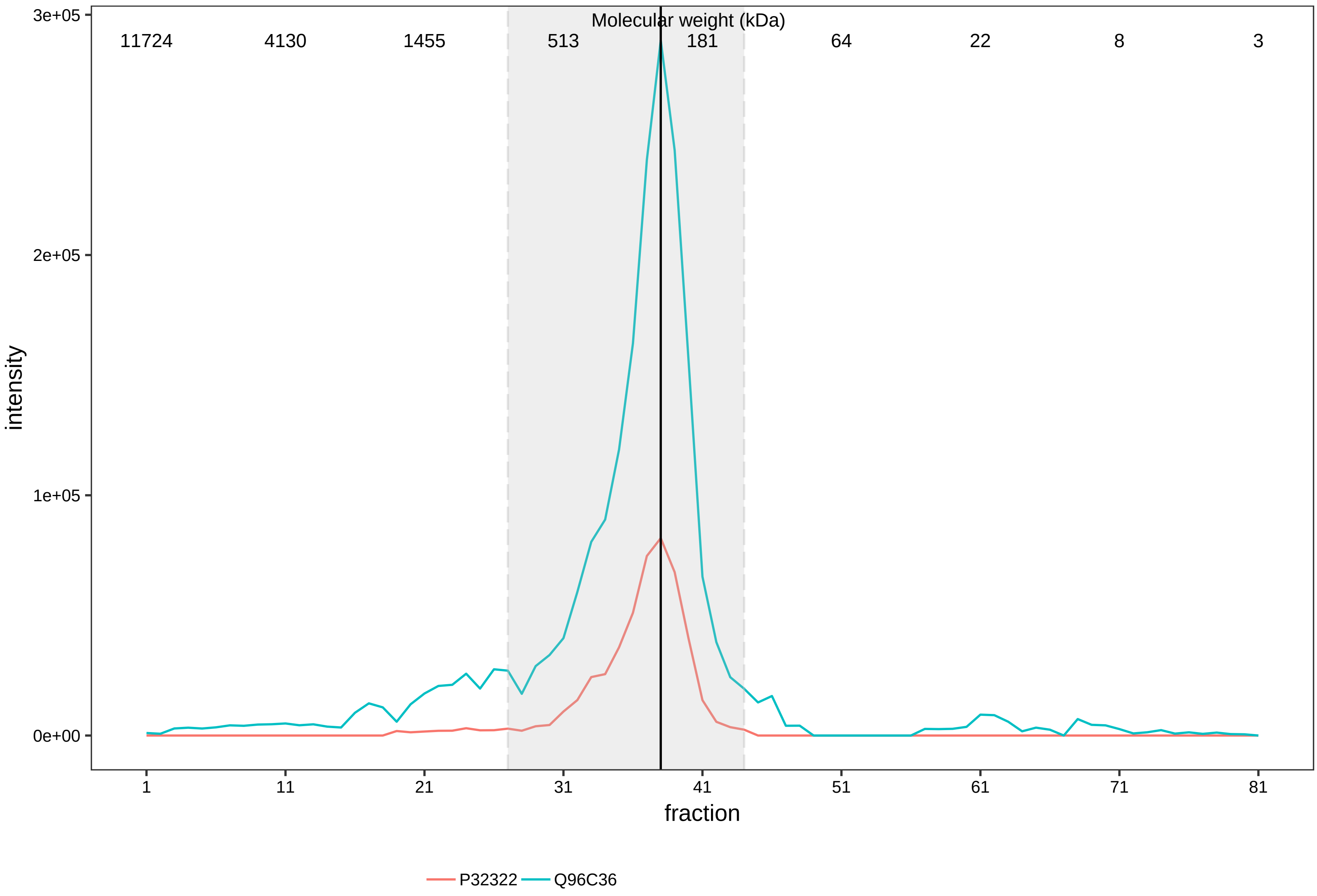

Feature ID 410

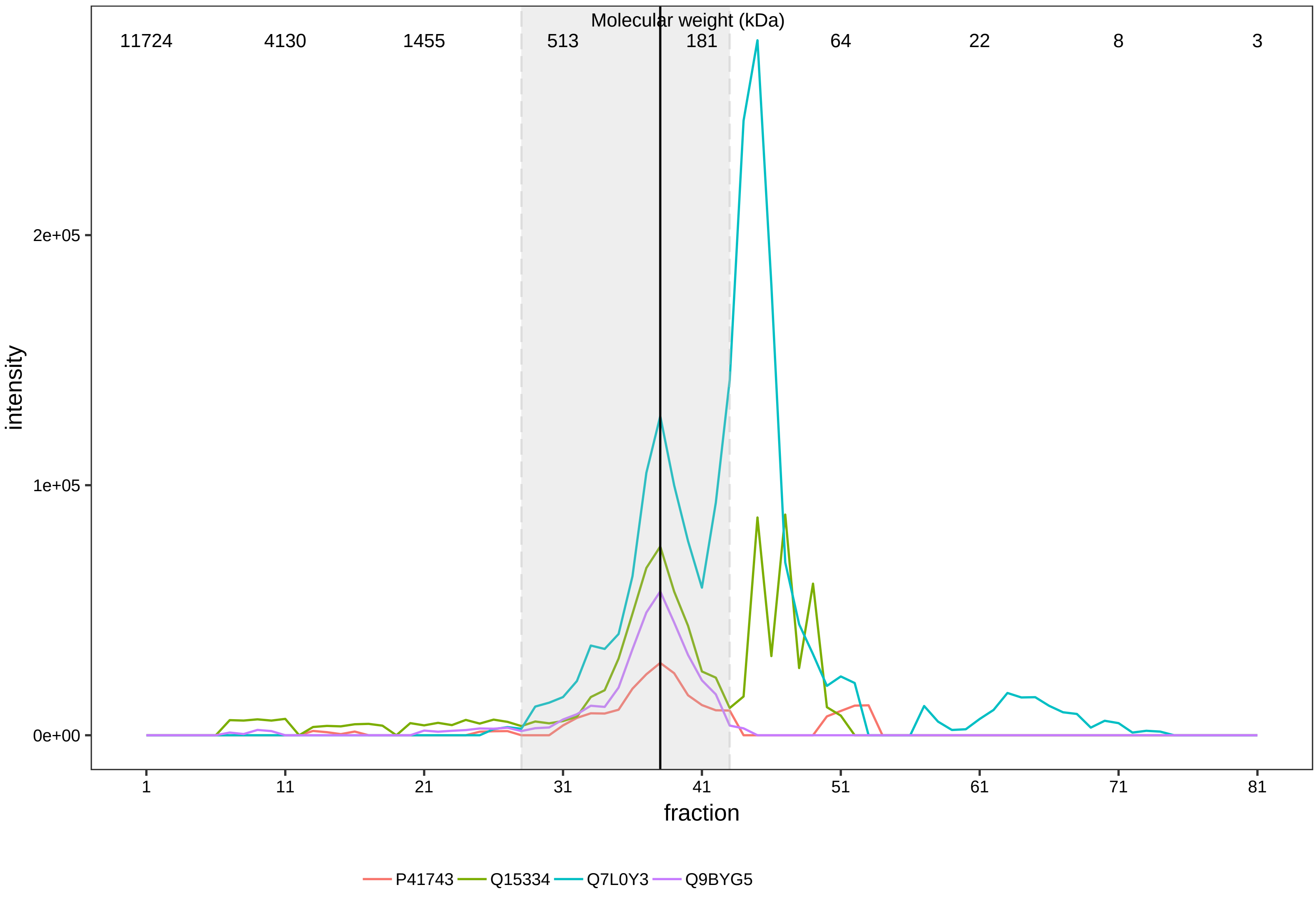

Feature ID 411

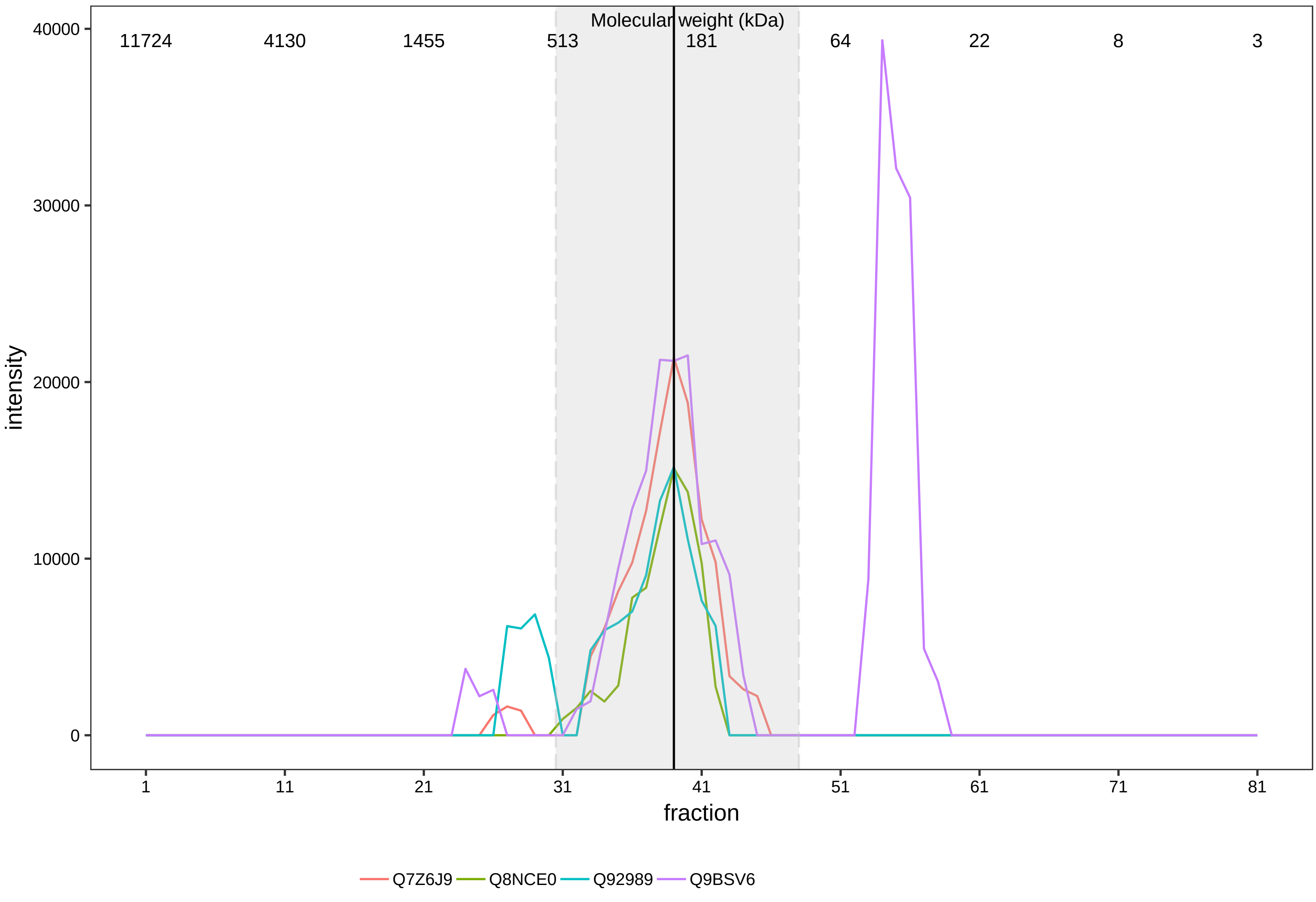

Feature ID 412

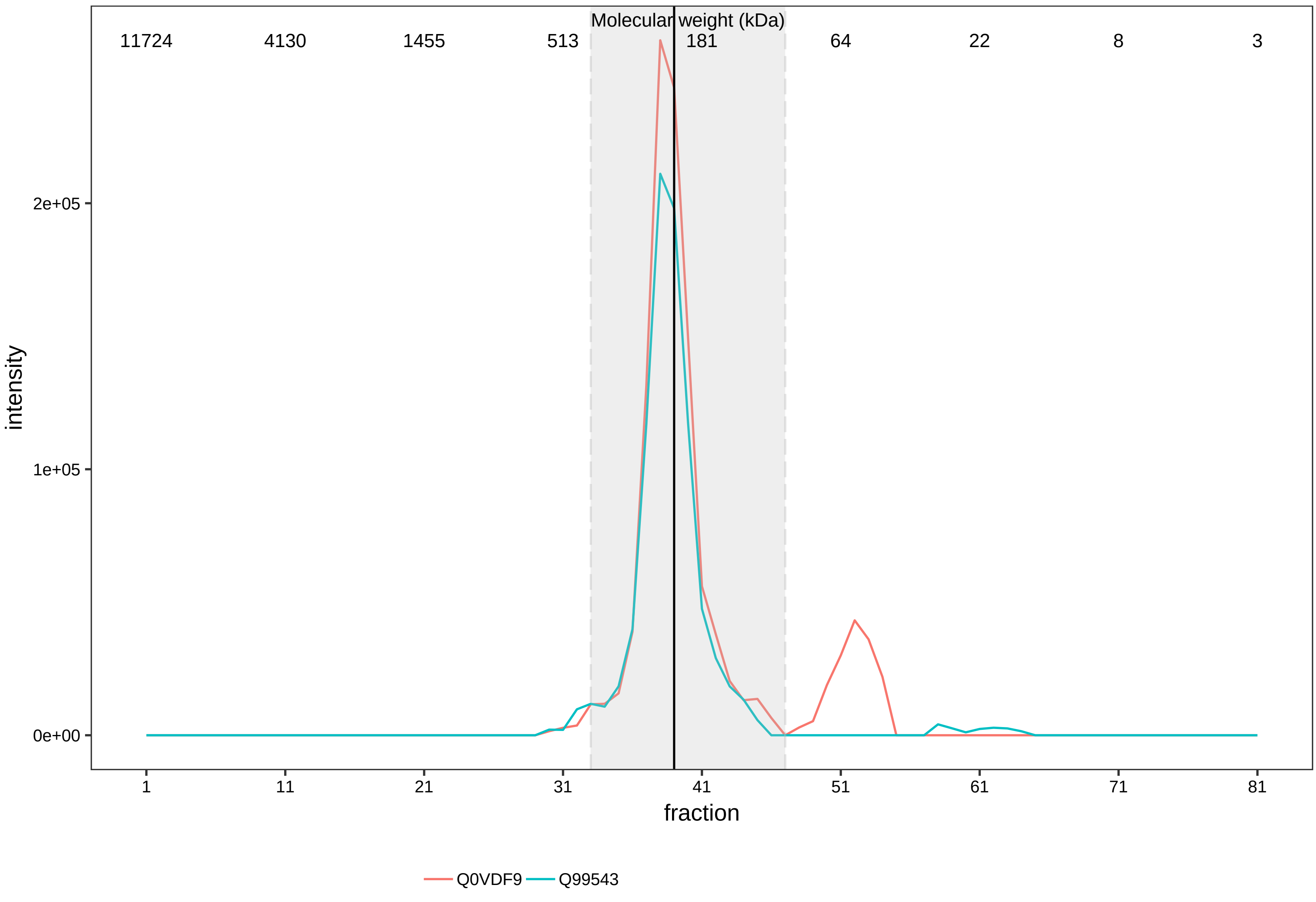

Feature ID 413

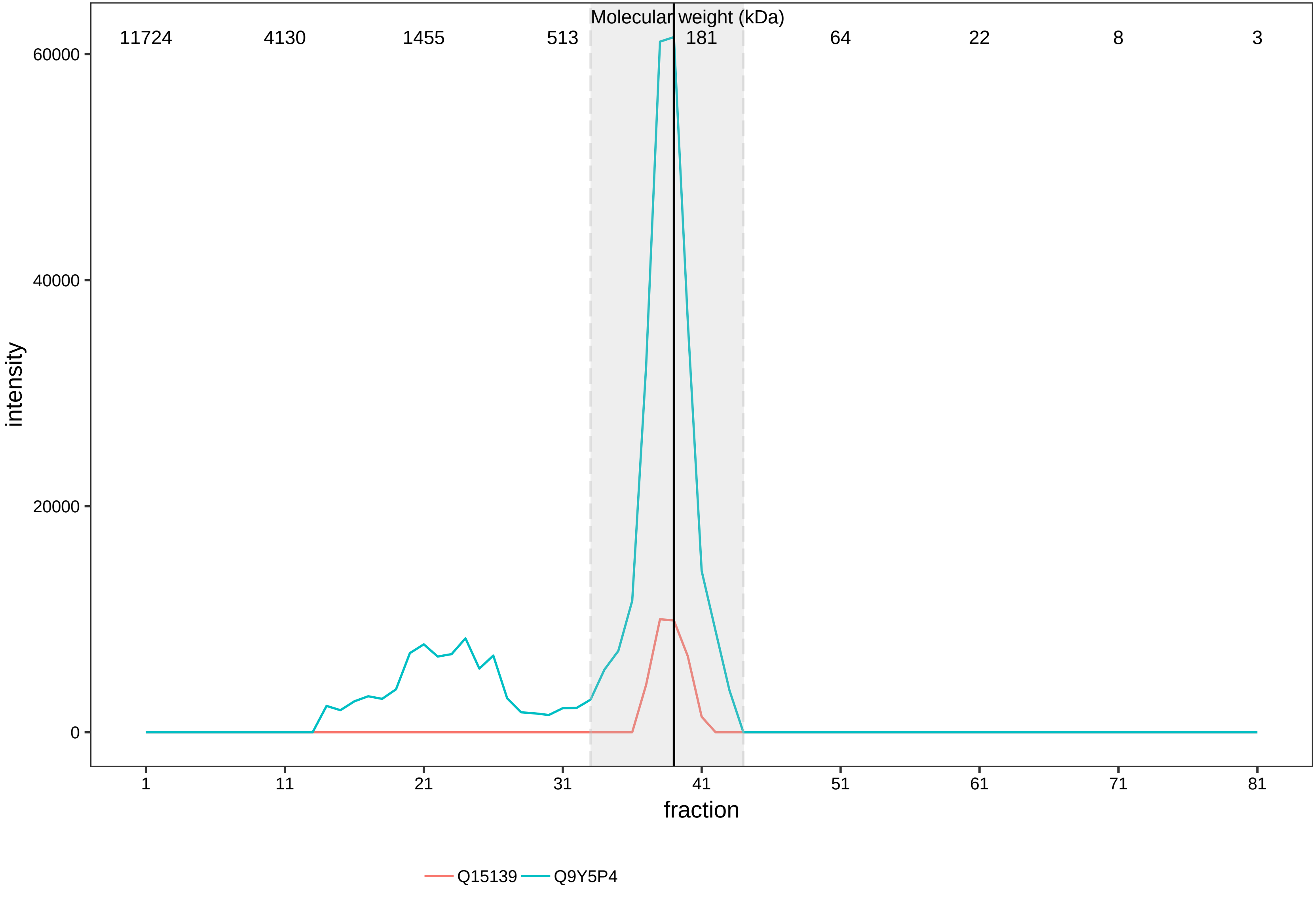

Feature ID 414

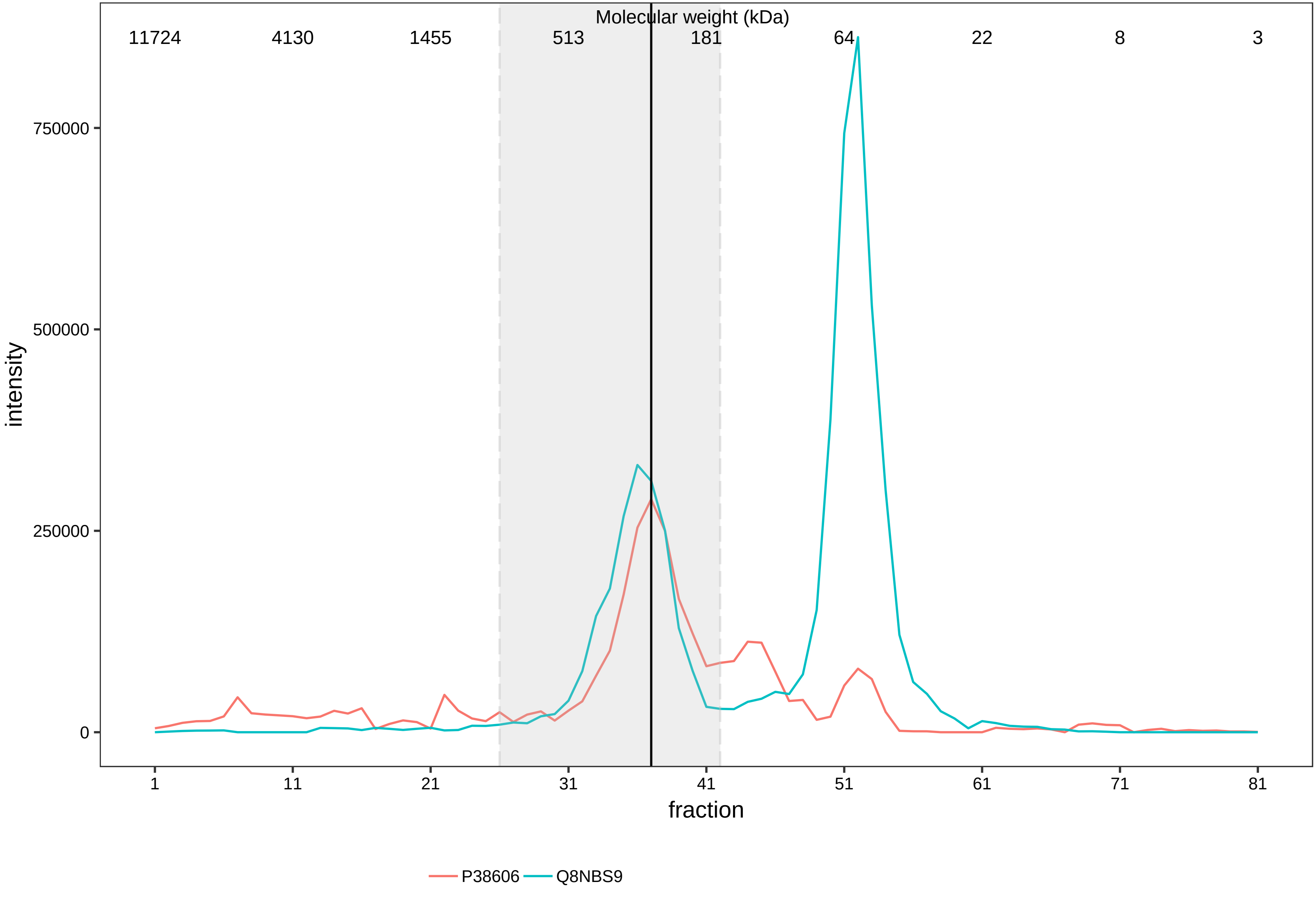

Feature ID 415

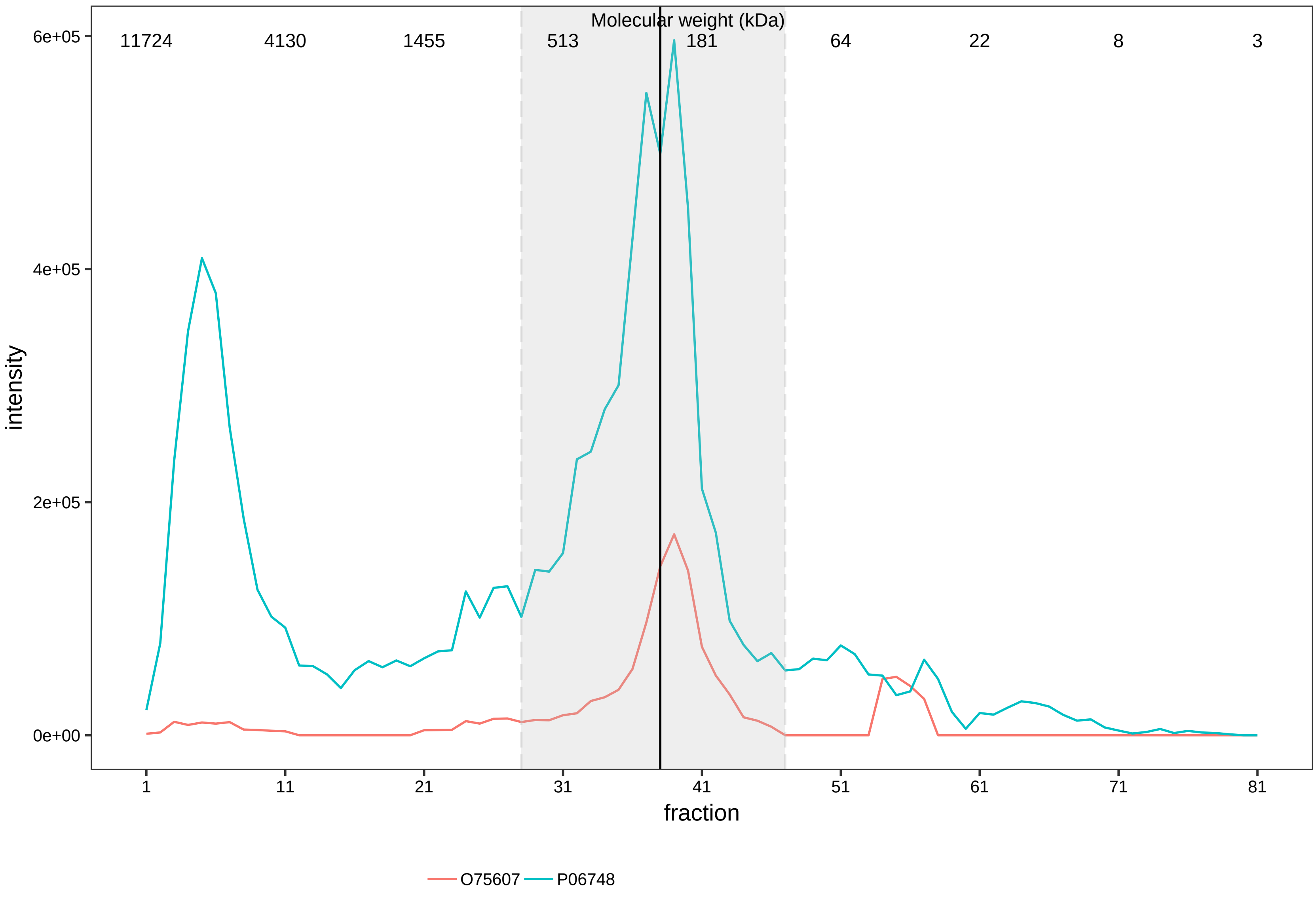

Feature ID 416

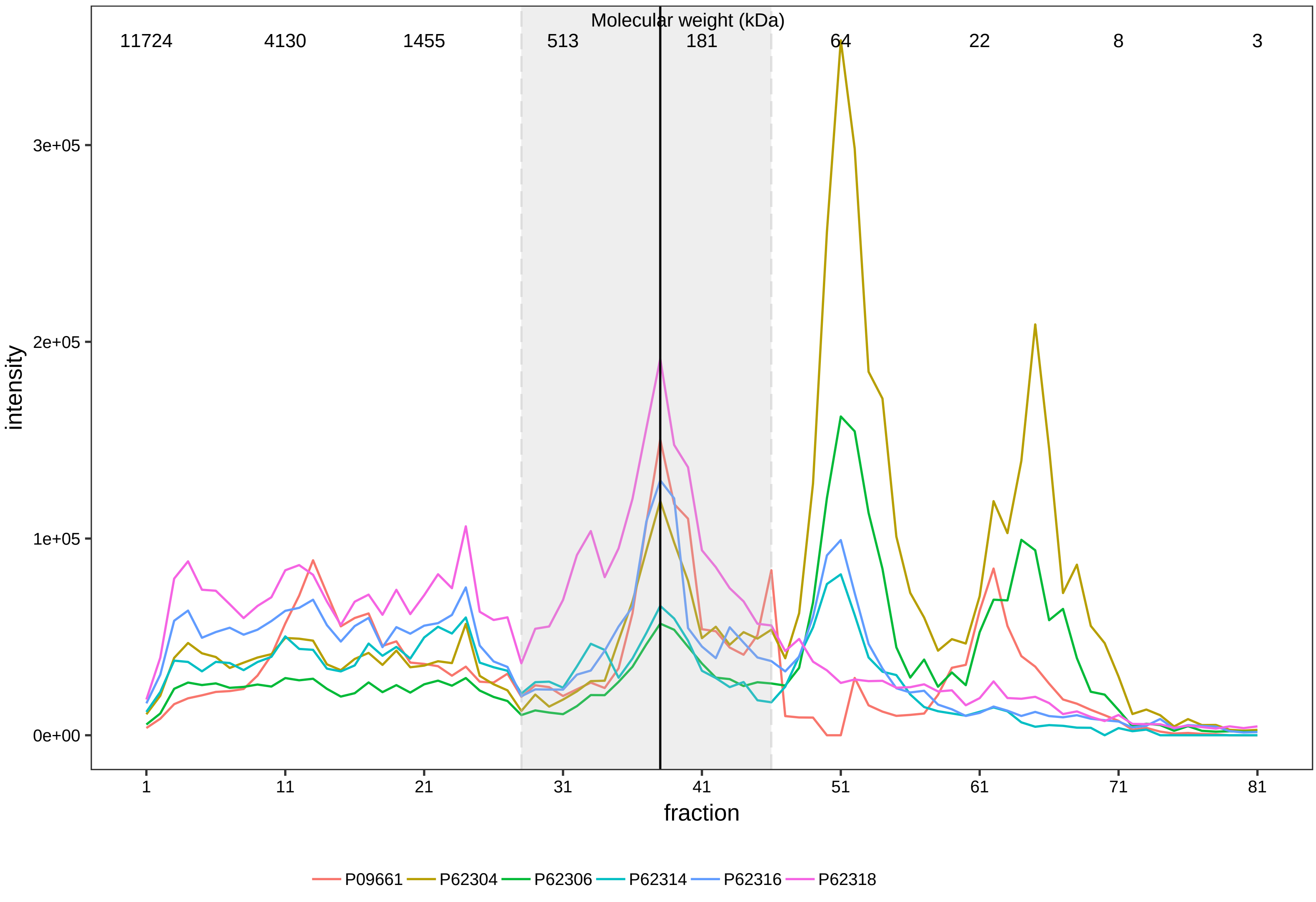

Feature ID 417

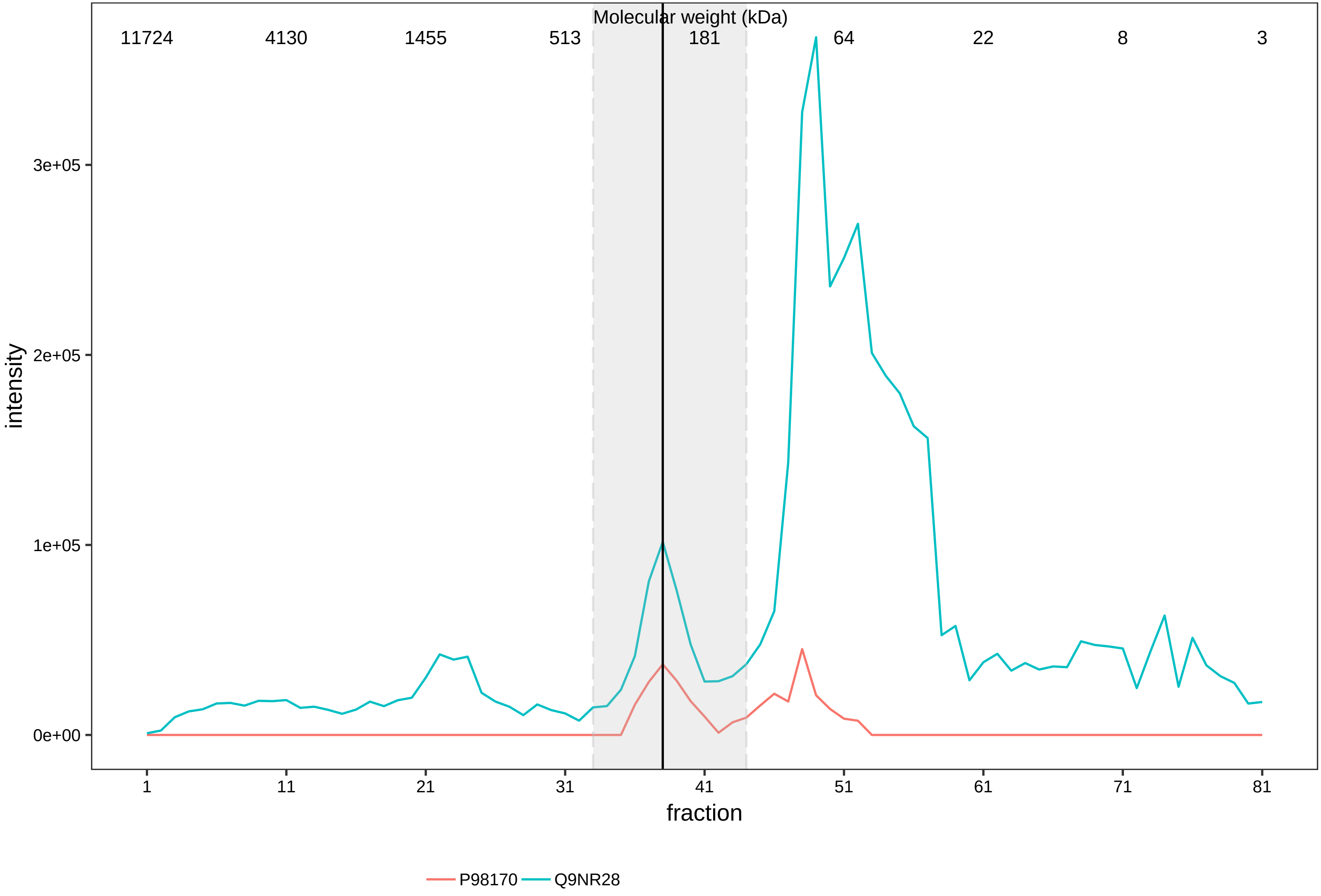

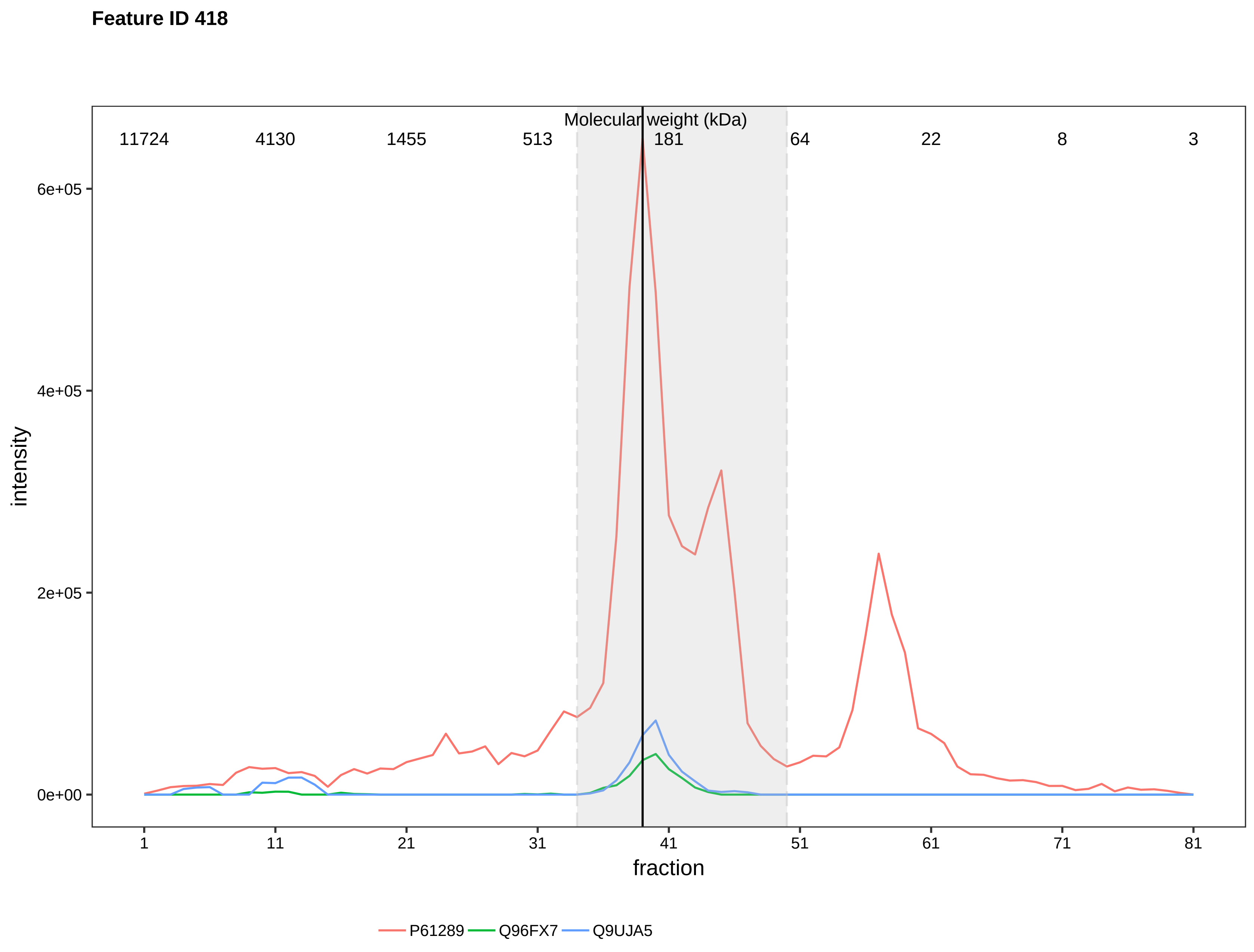

Feature ID 418
Molecular weight (kDa)
11724
4130
1455
513
181
64
22
8
3
6e+05
4e+05
2e+05
0e+00
intensity
1
11
21
31
41
51
61
71
81
fraction
P61289
Q96FX7
Q9UJA5

Feature ID 419

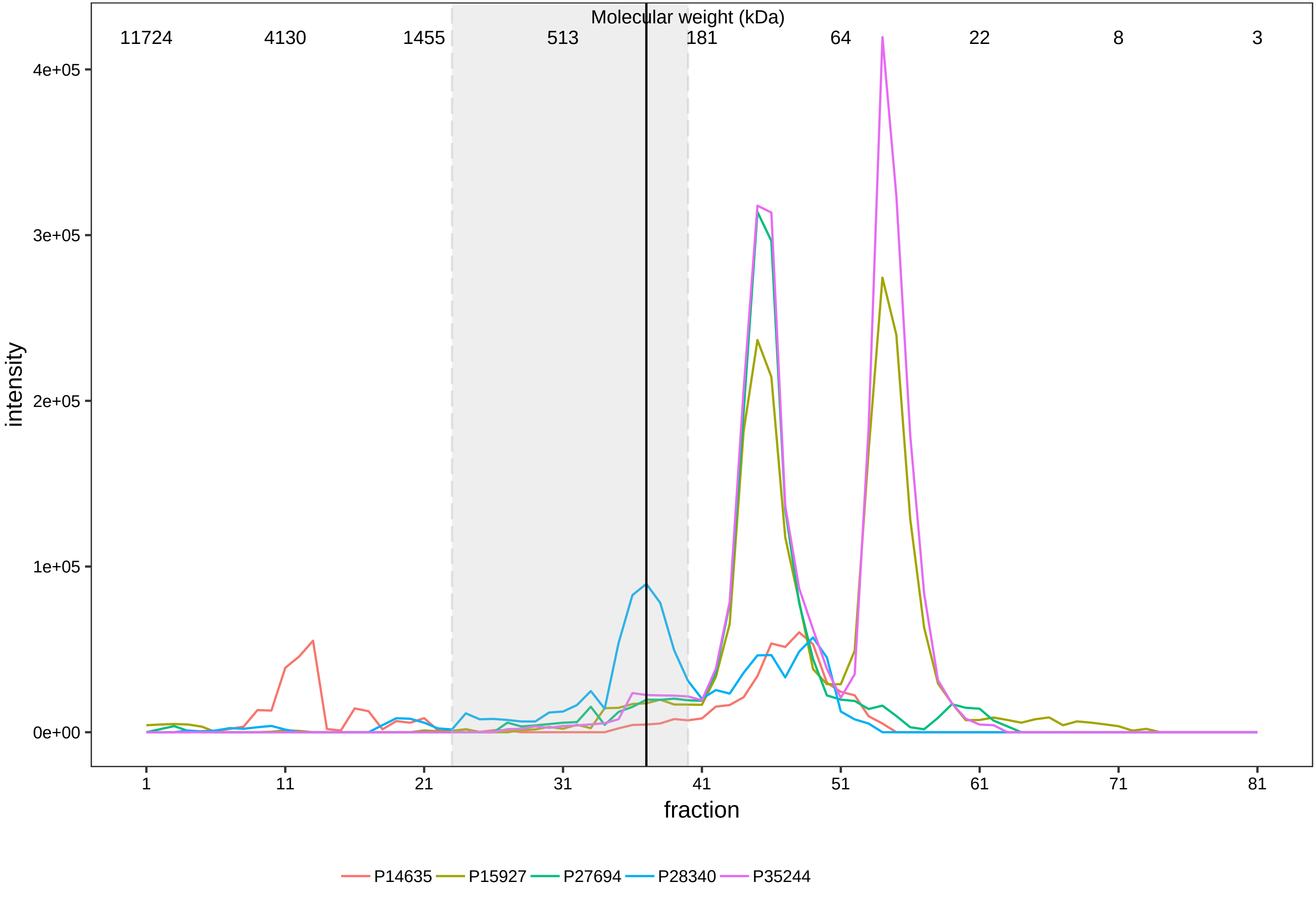

Feature ID 420

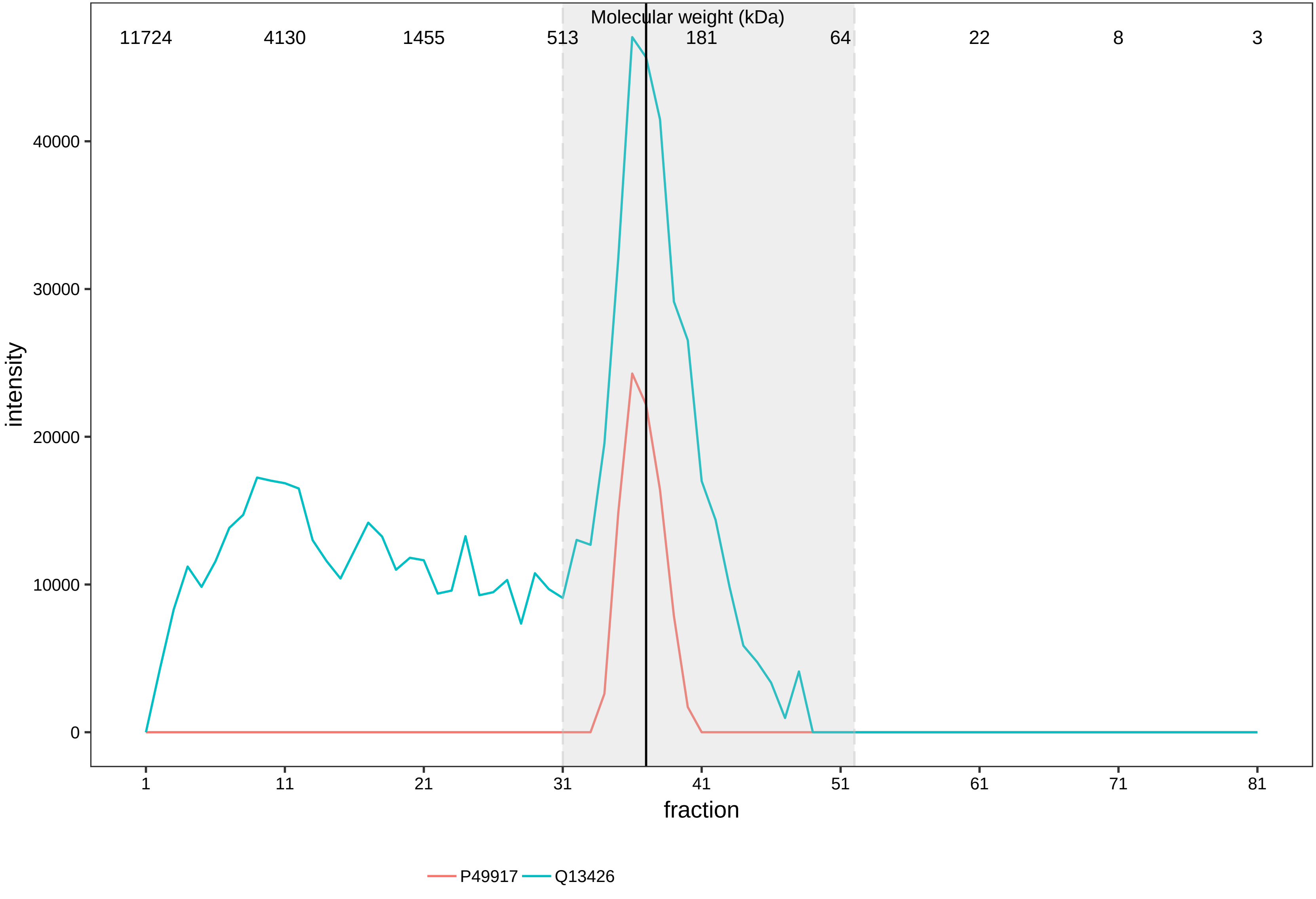

Feature ID 421

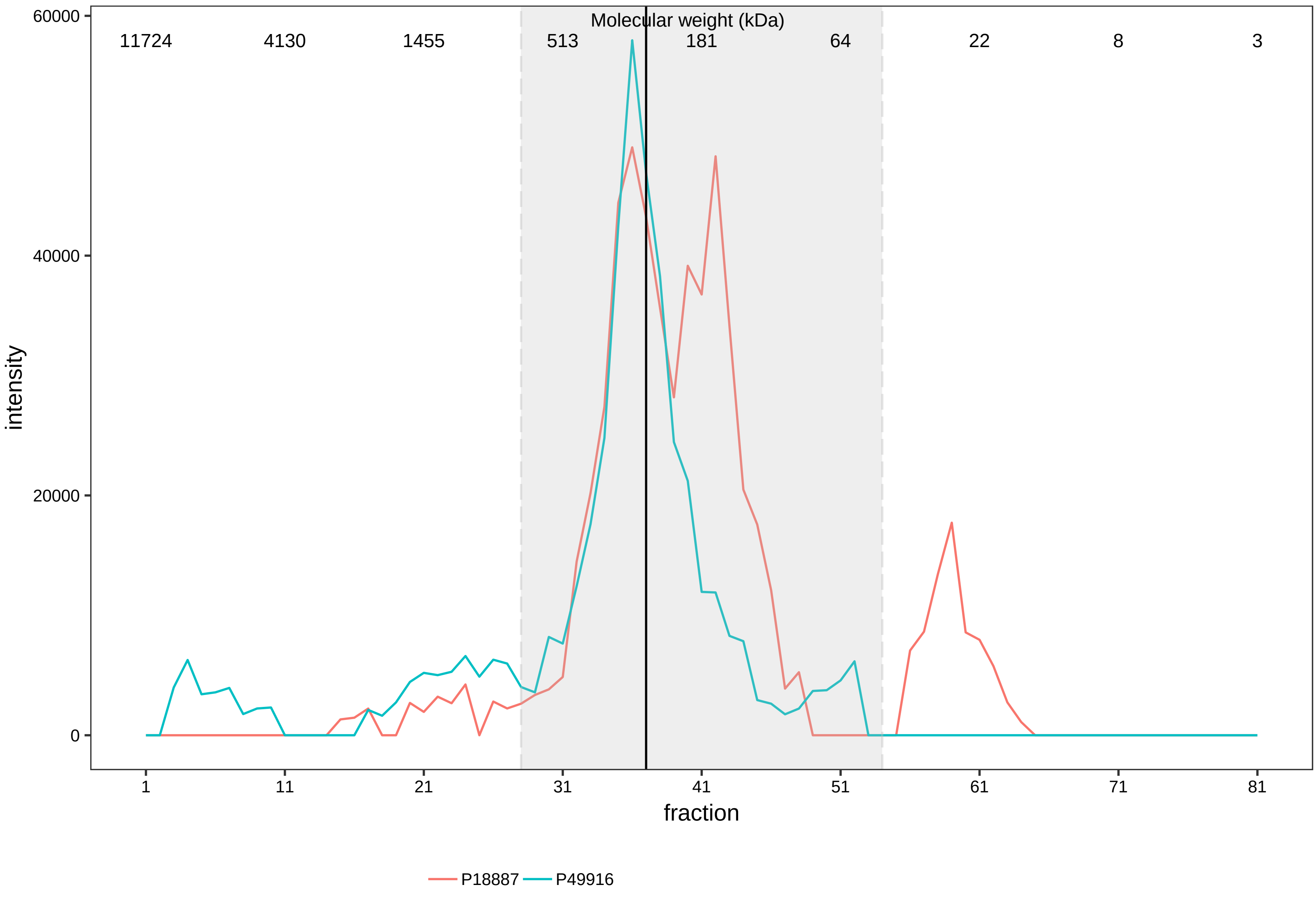

Feature ID 422

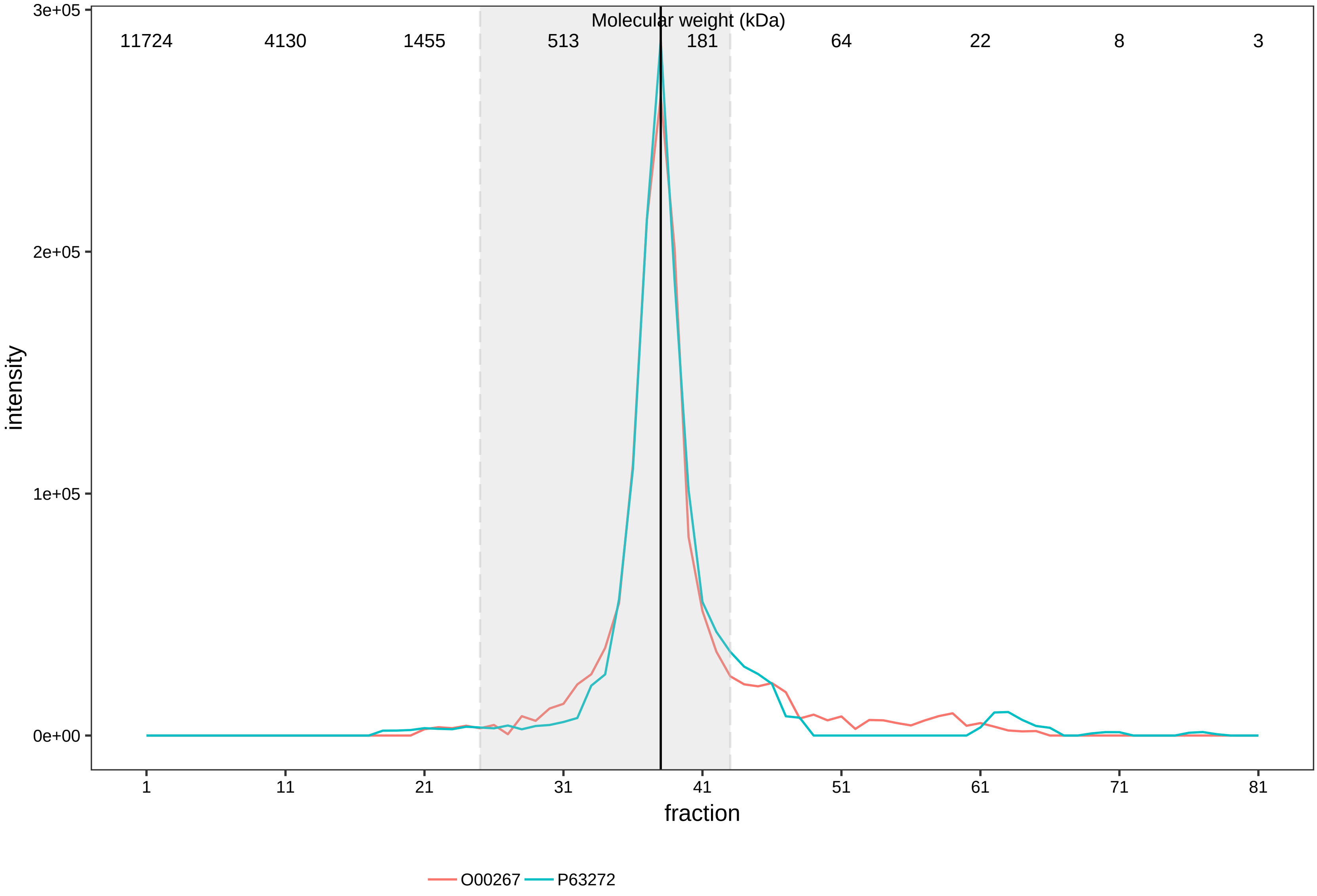

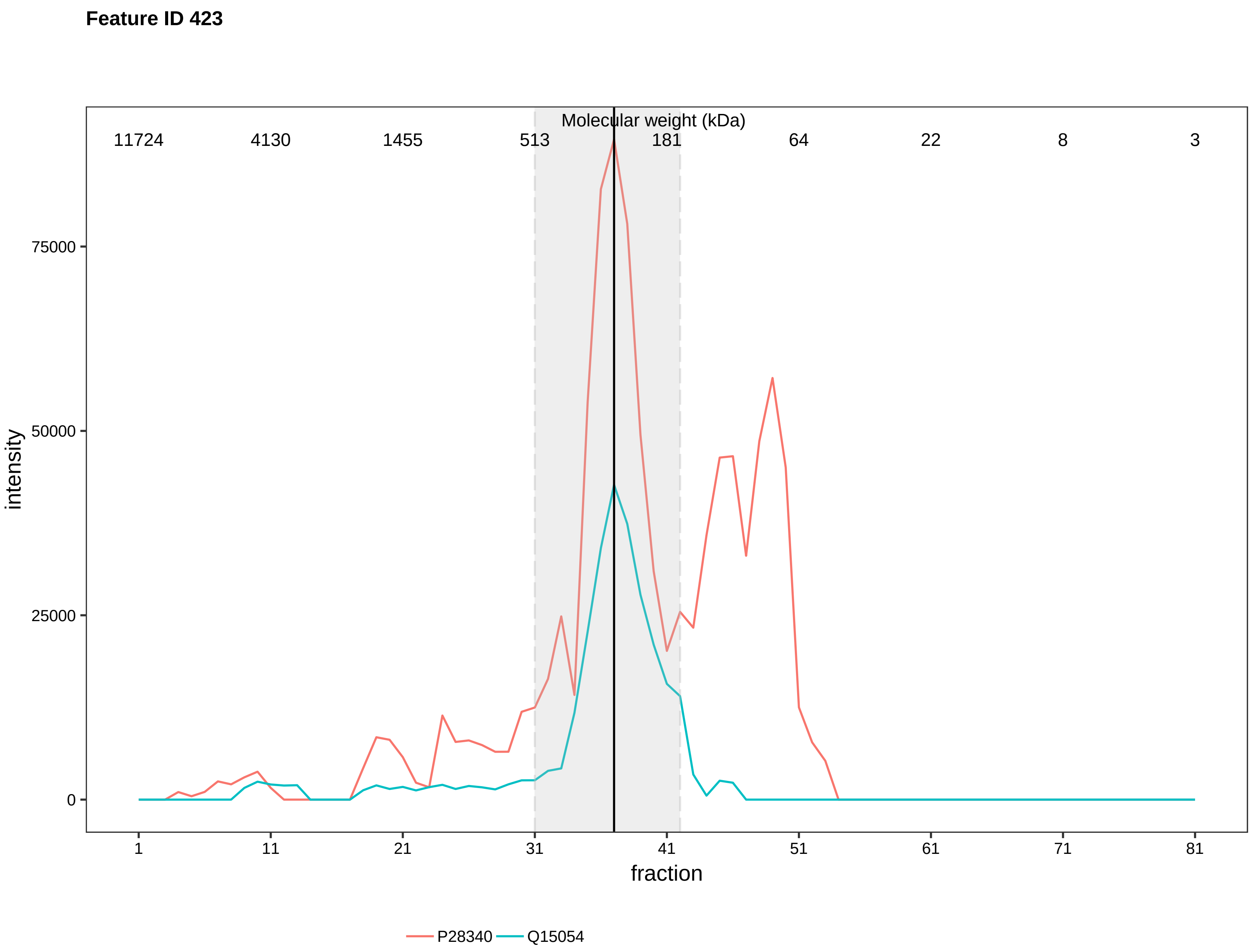

Feature ID 423
Molecular weight (kDa)
11724
4130
1455
513
181
64
22
8
3
intensity
75000
50000
25000
0
1
11
21
31
41
51
61
71
81
fraction
P28340
Q15054

Feature ID 424

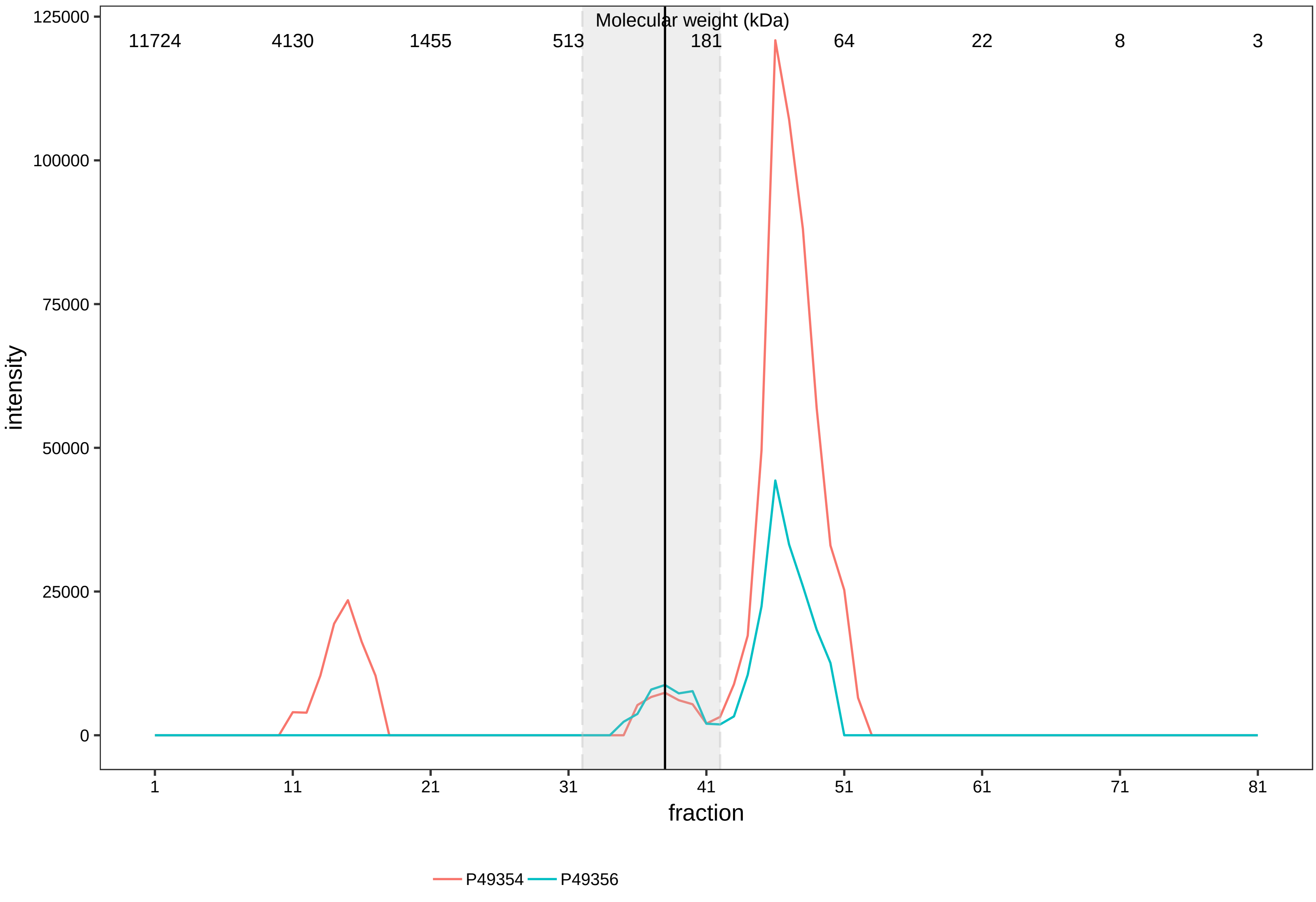

Feature ID 425

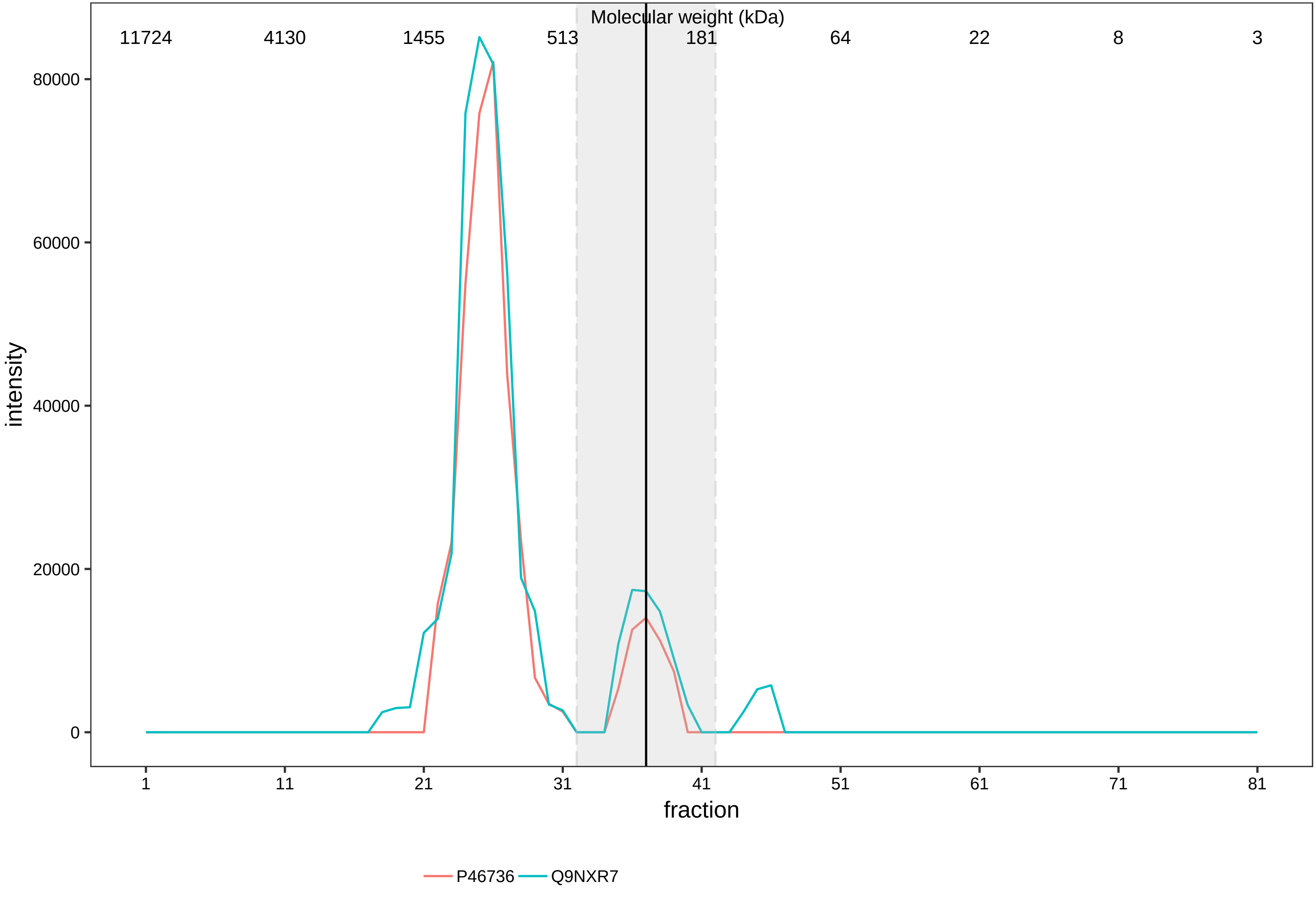

Feature ID 426

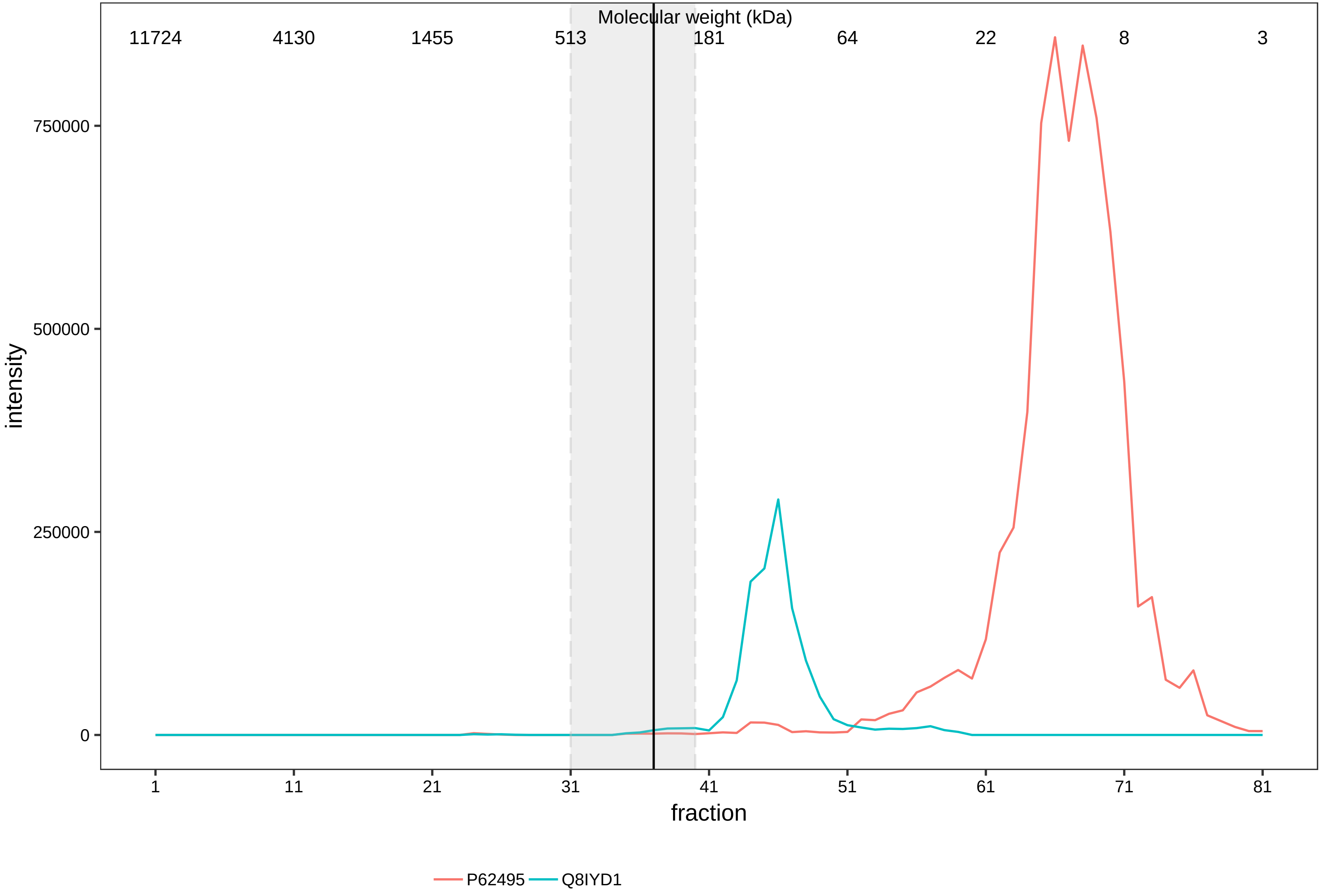

Feature ID 427

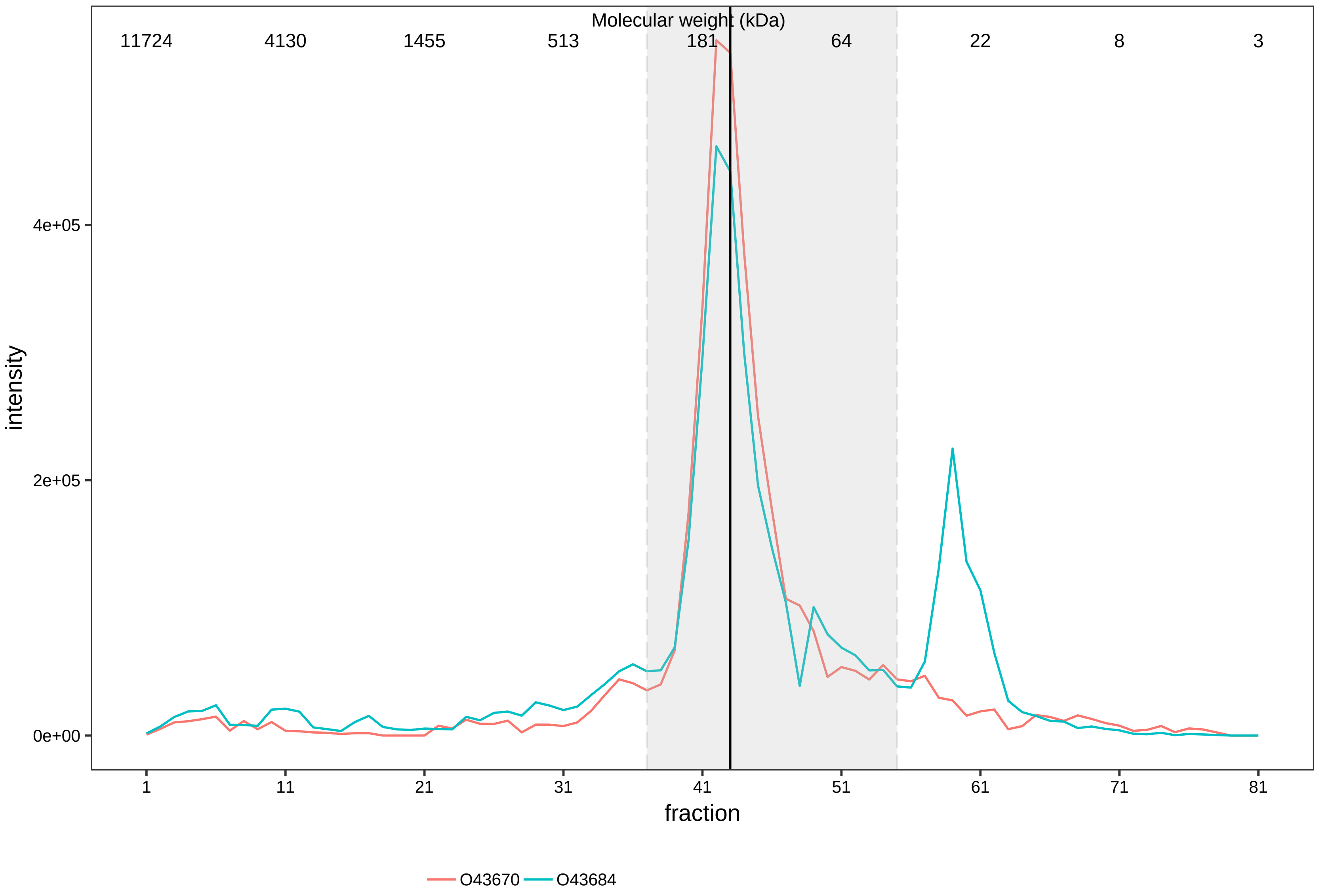

Feature ID 428

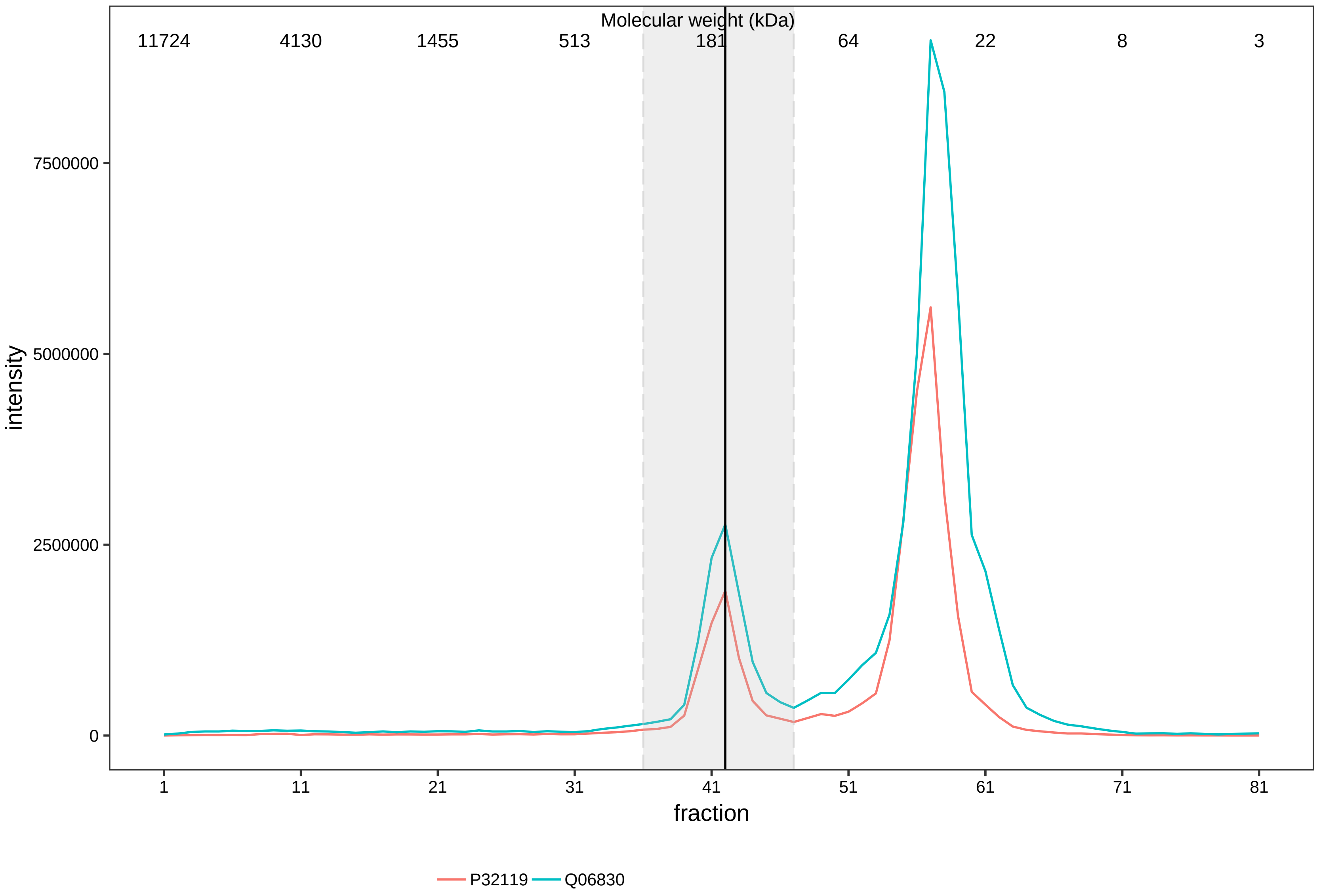

Feature ID 429

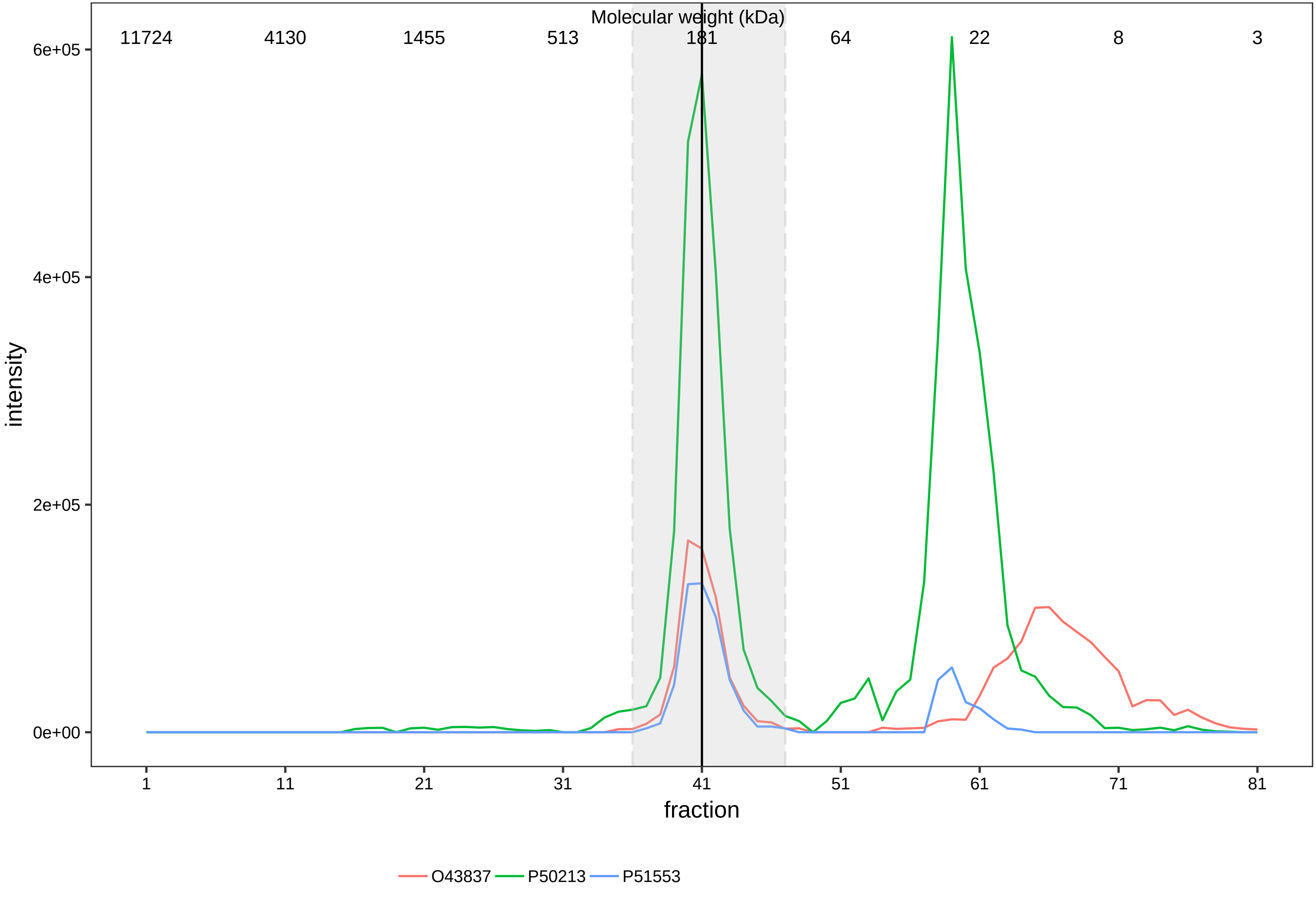

Feature ID 430

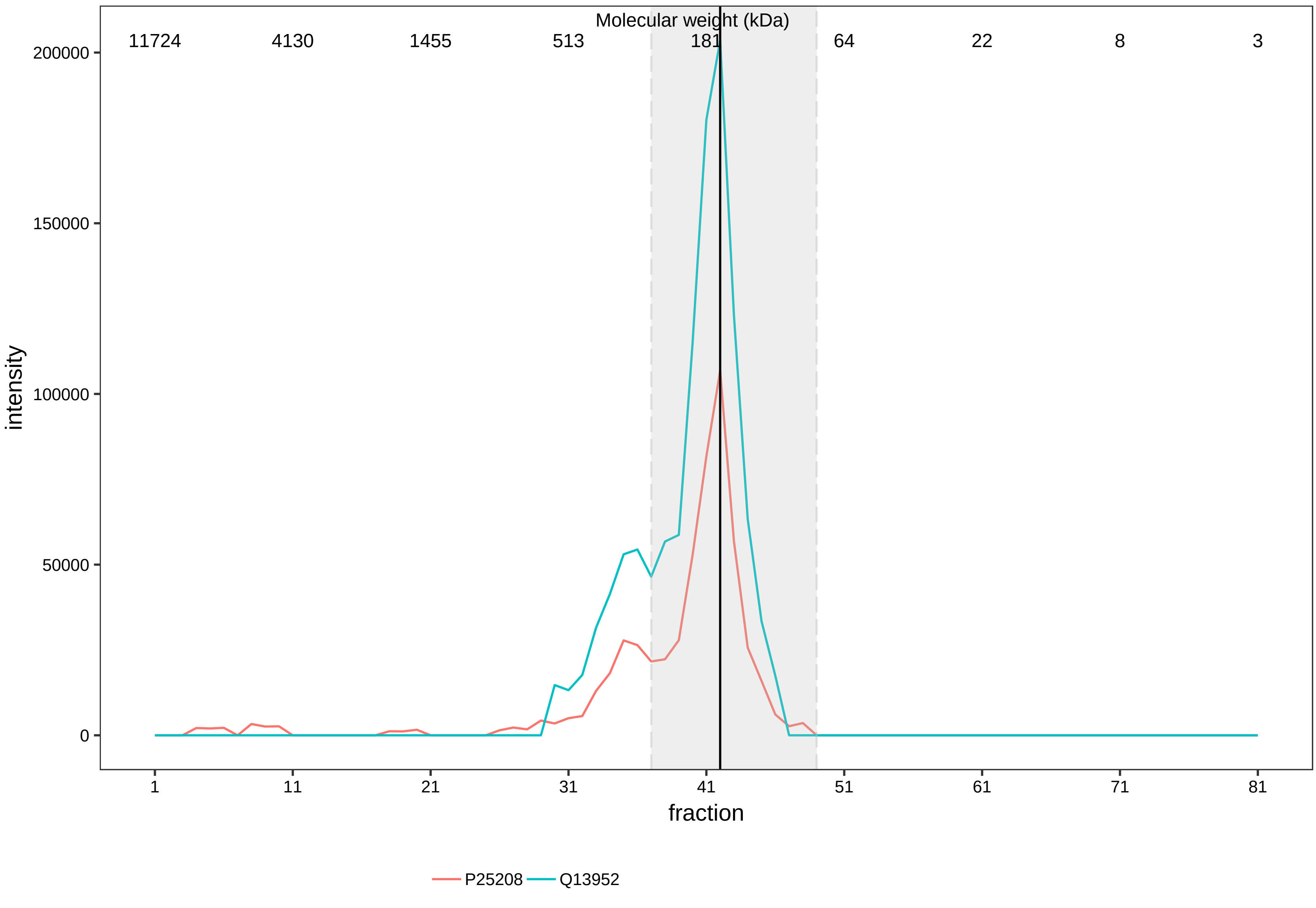

Feature ID 431

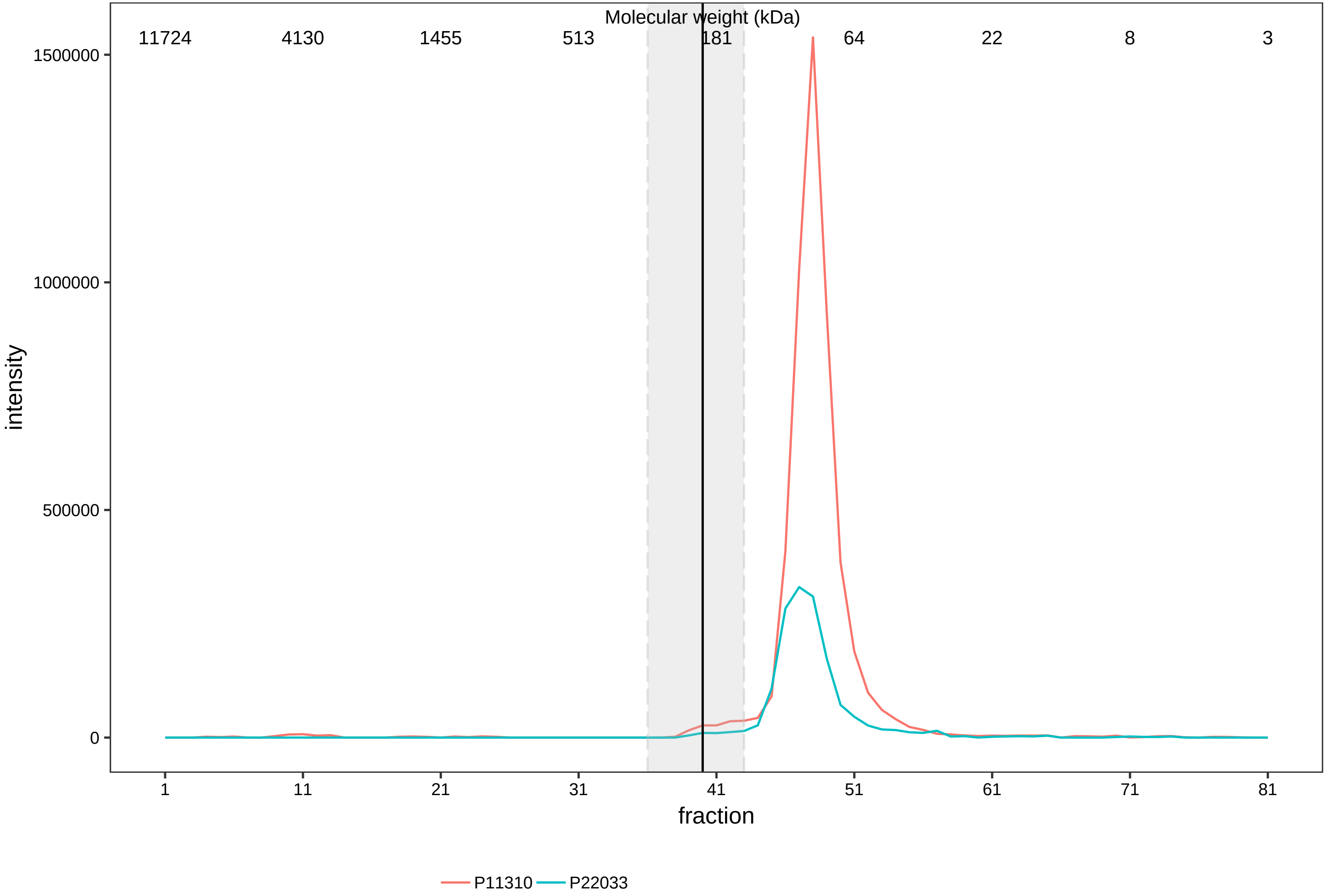

Feature ID 432

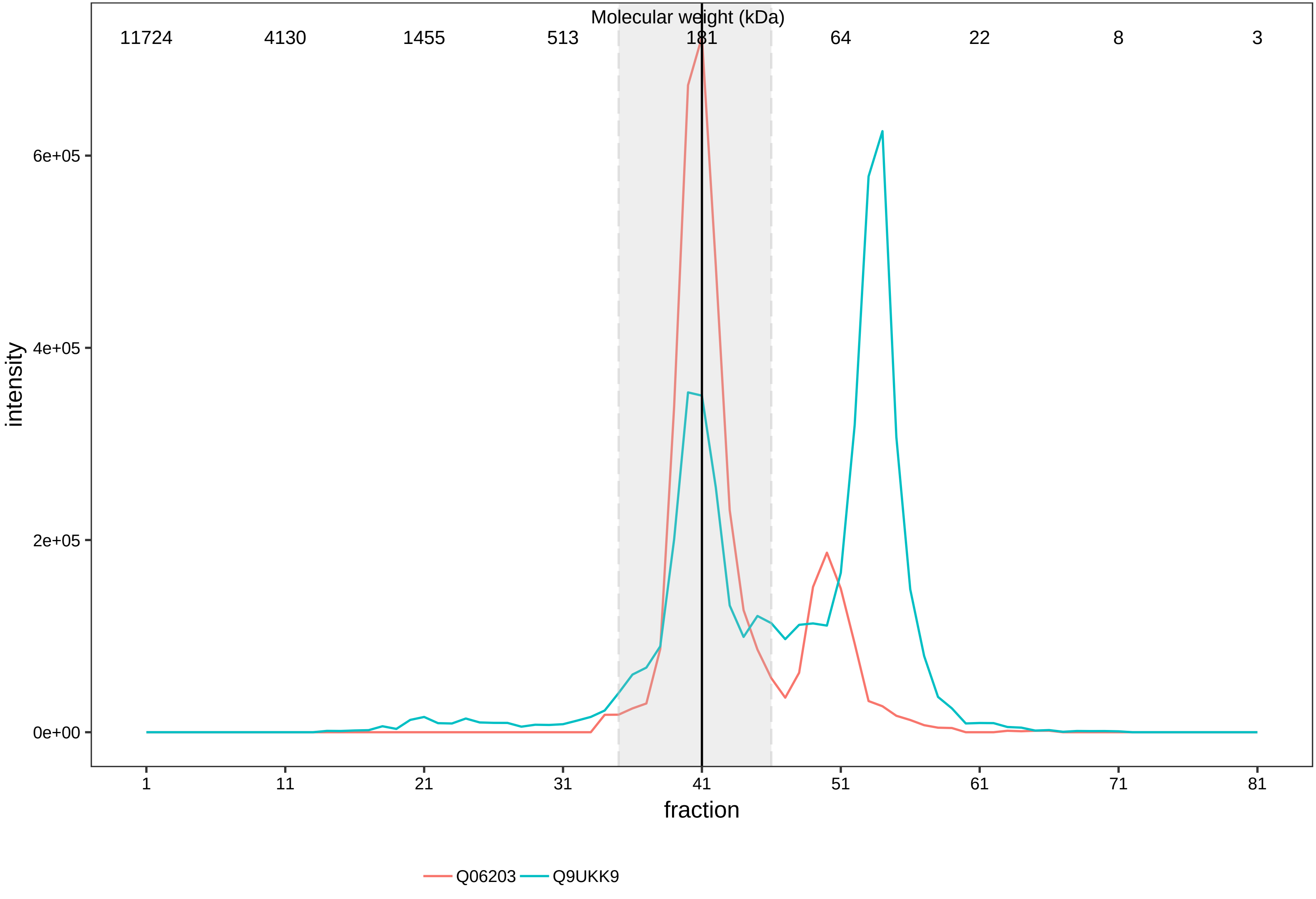

Feature ID 433

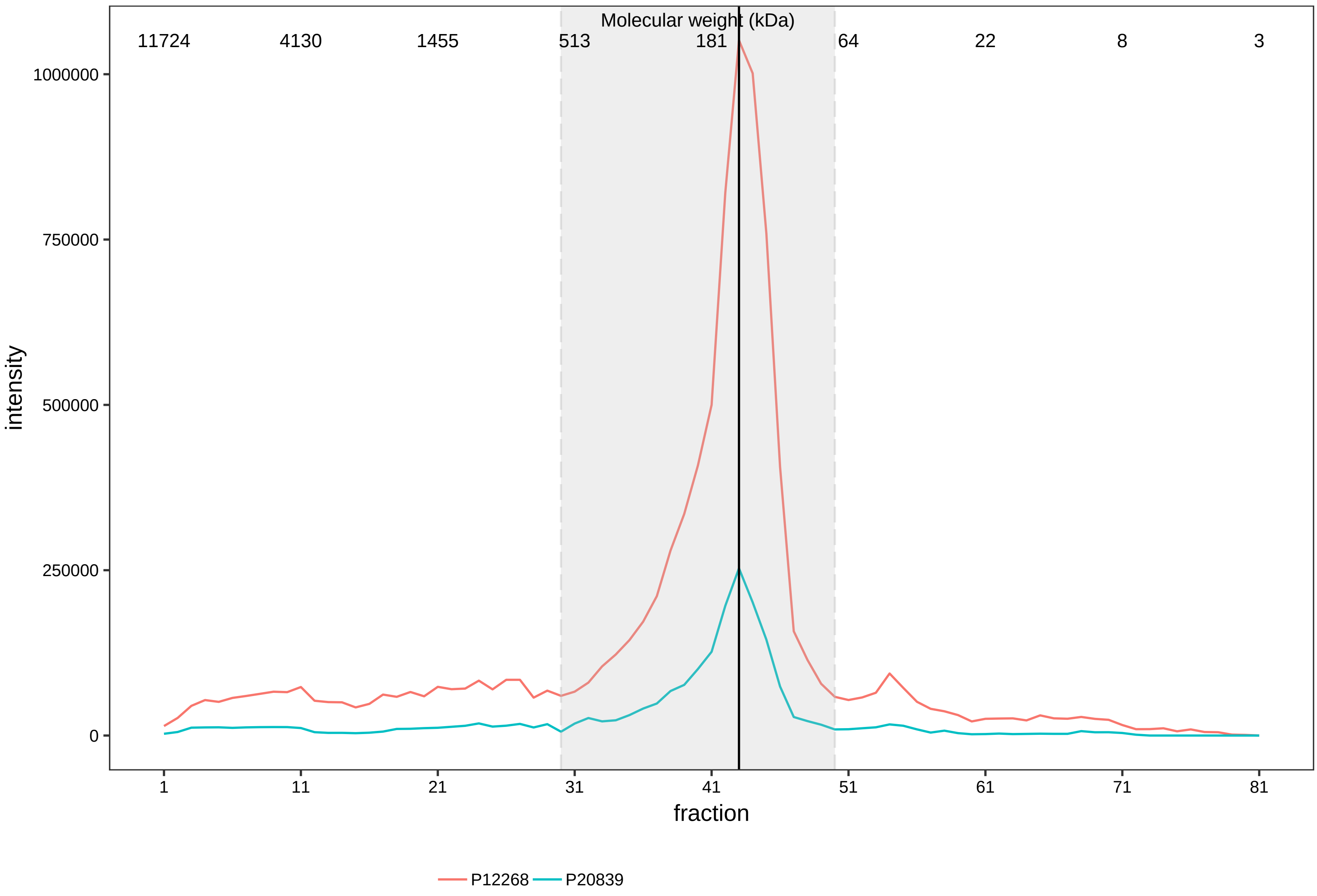

Feature ID 434

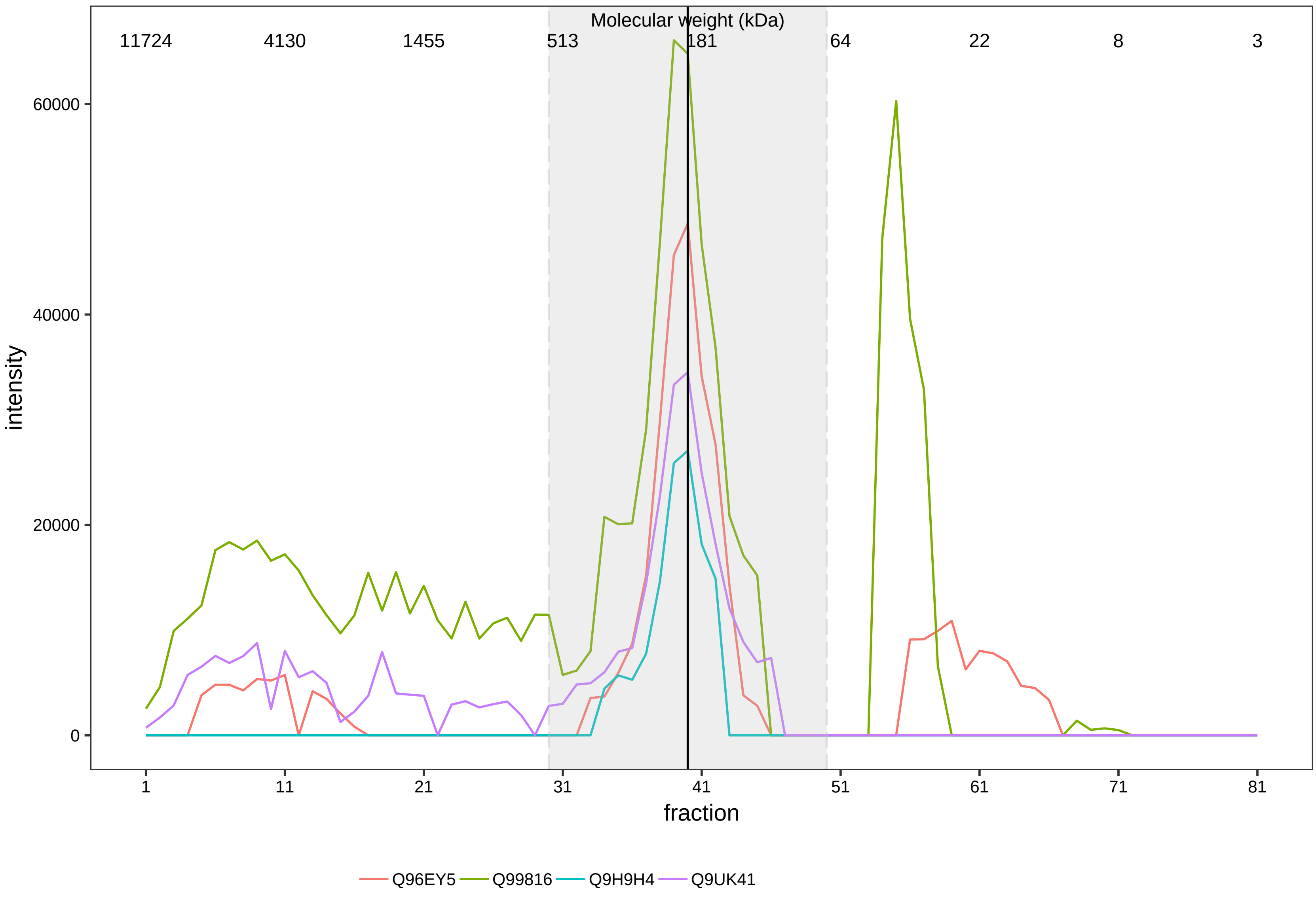

Feature ID 435

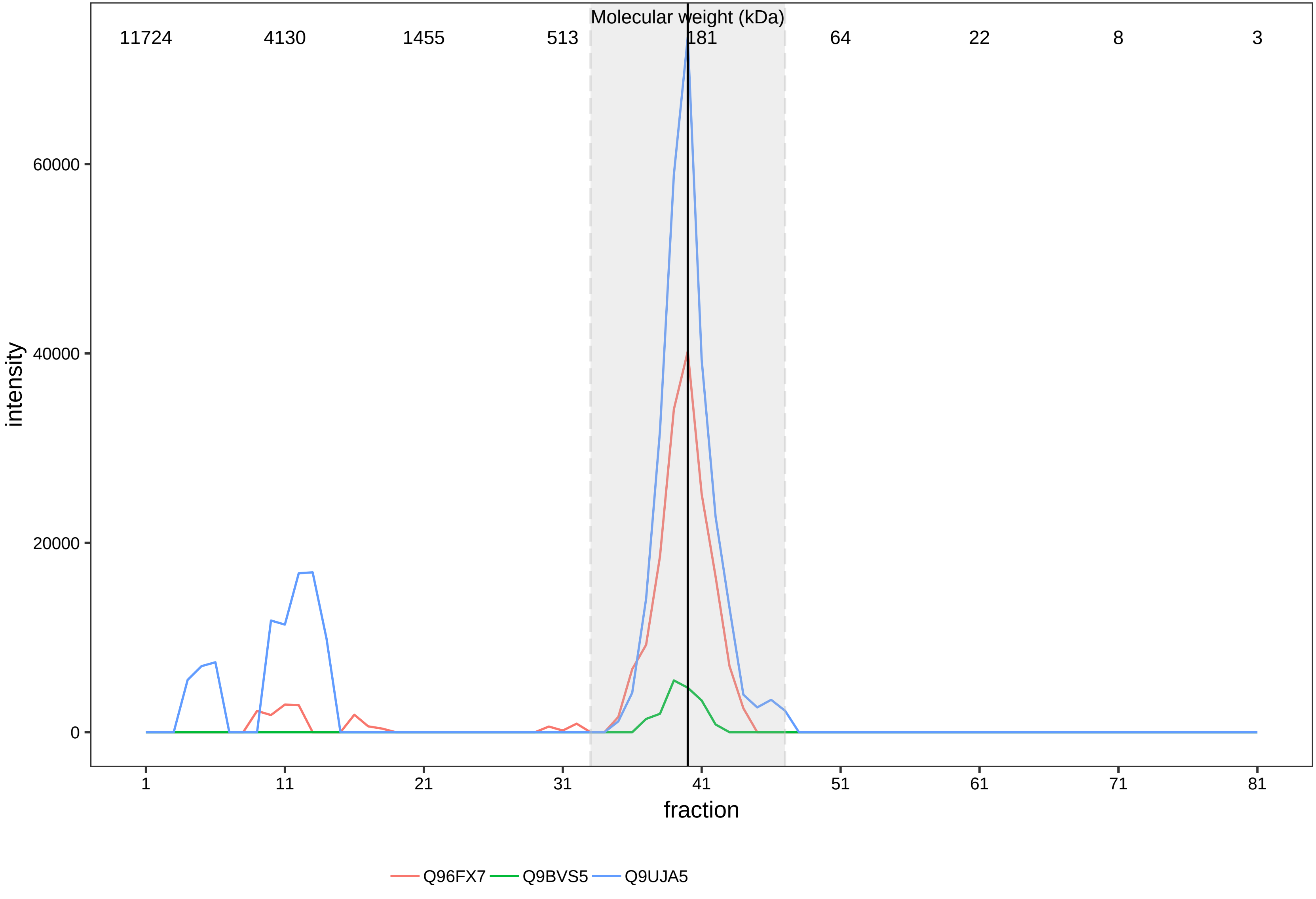

Feature ID 436

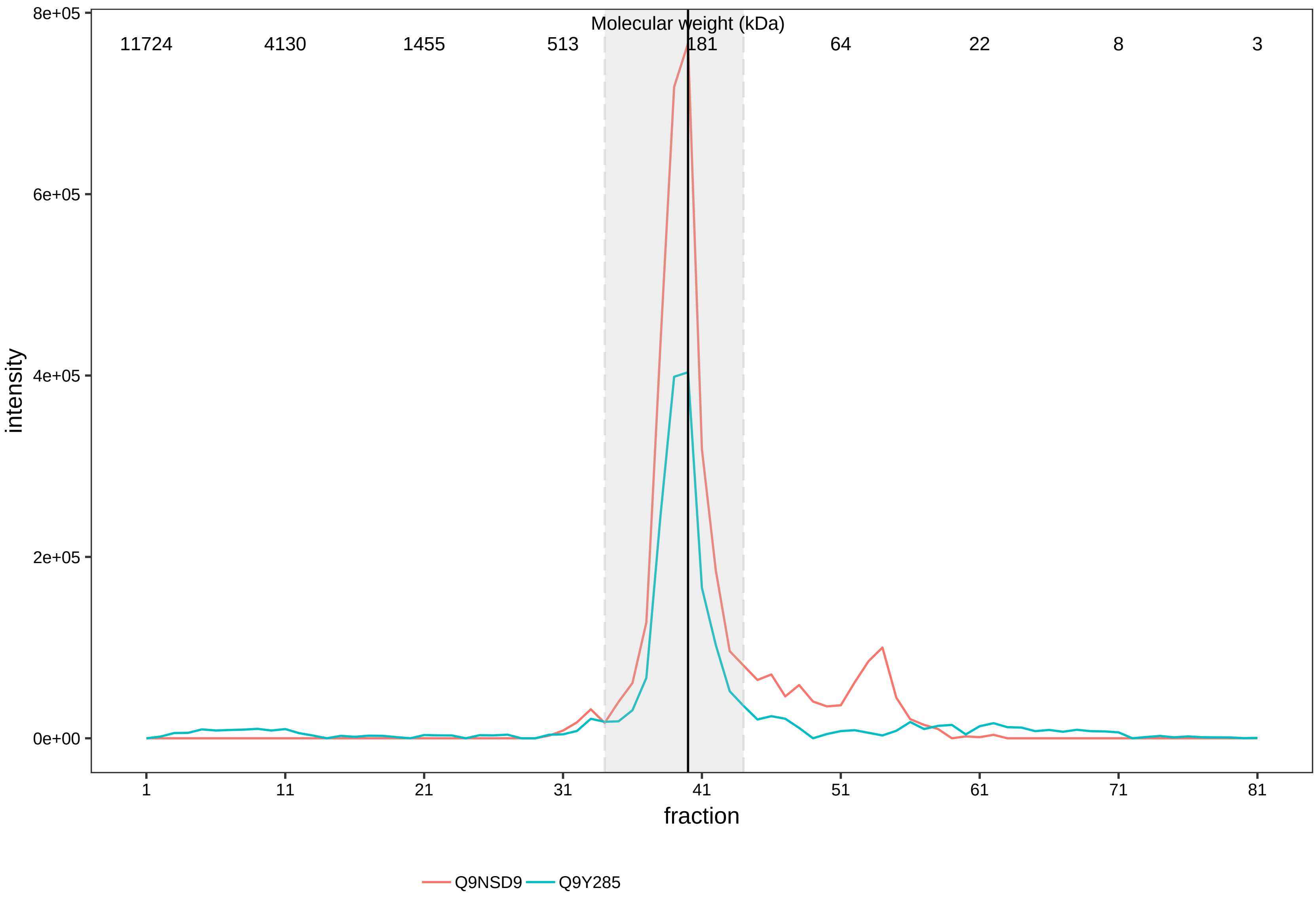

Feature ID 437

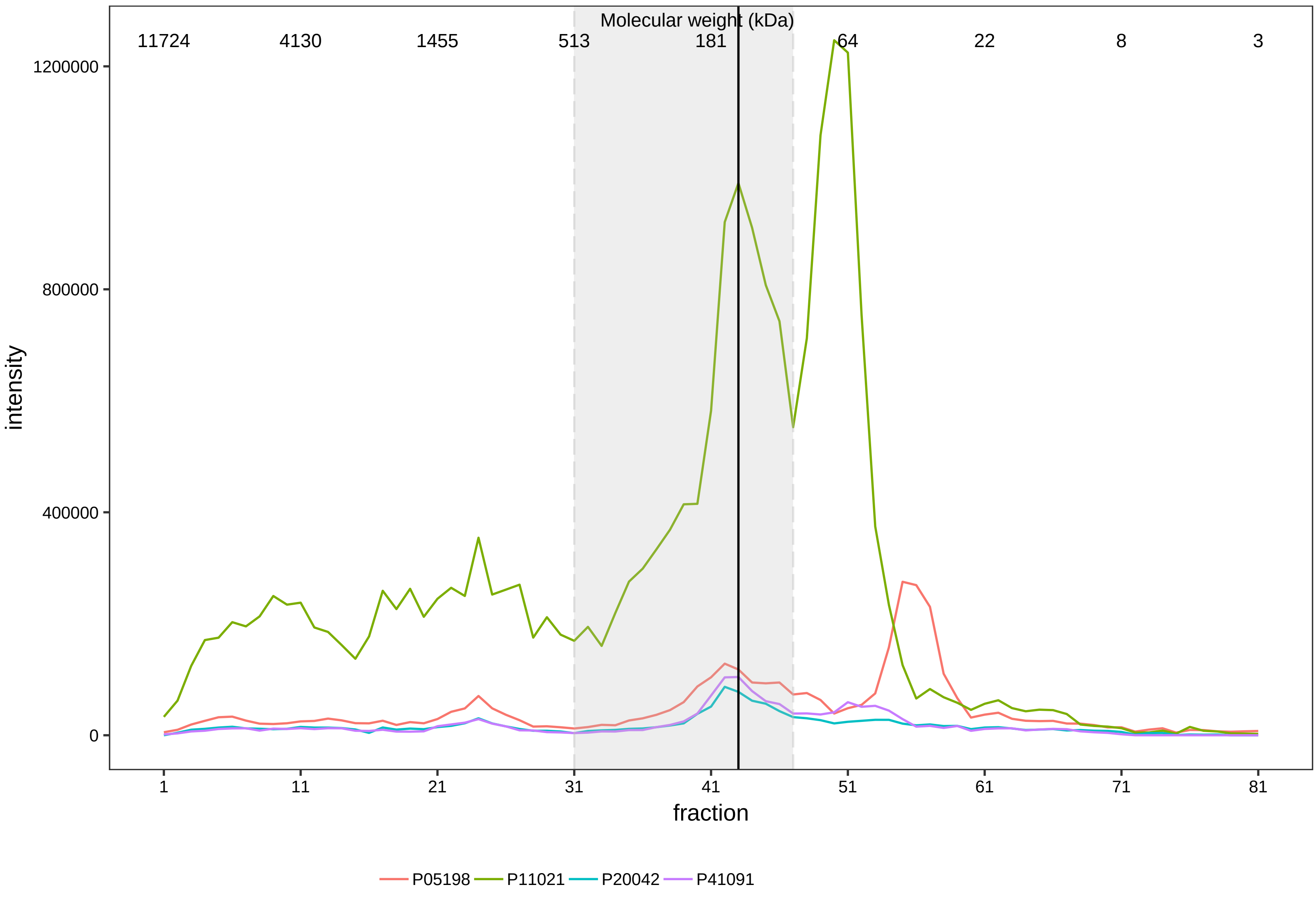

Feature ID 438

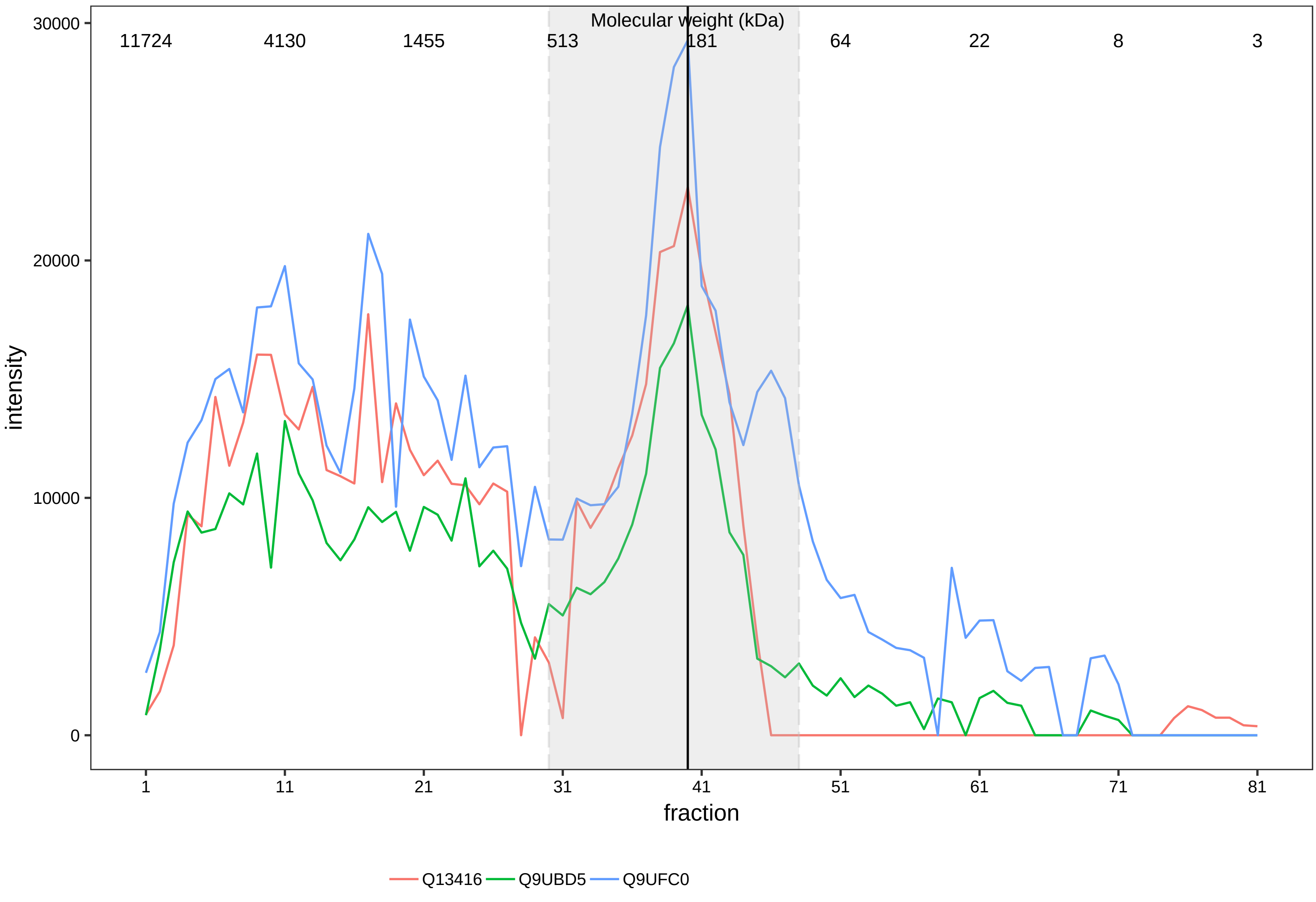

Feature ID 439

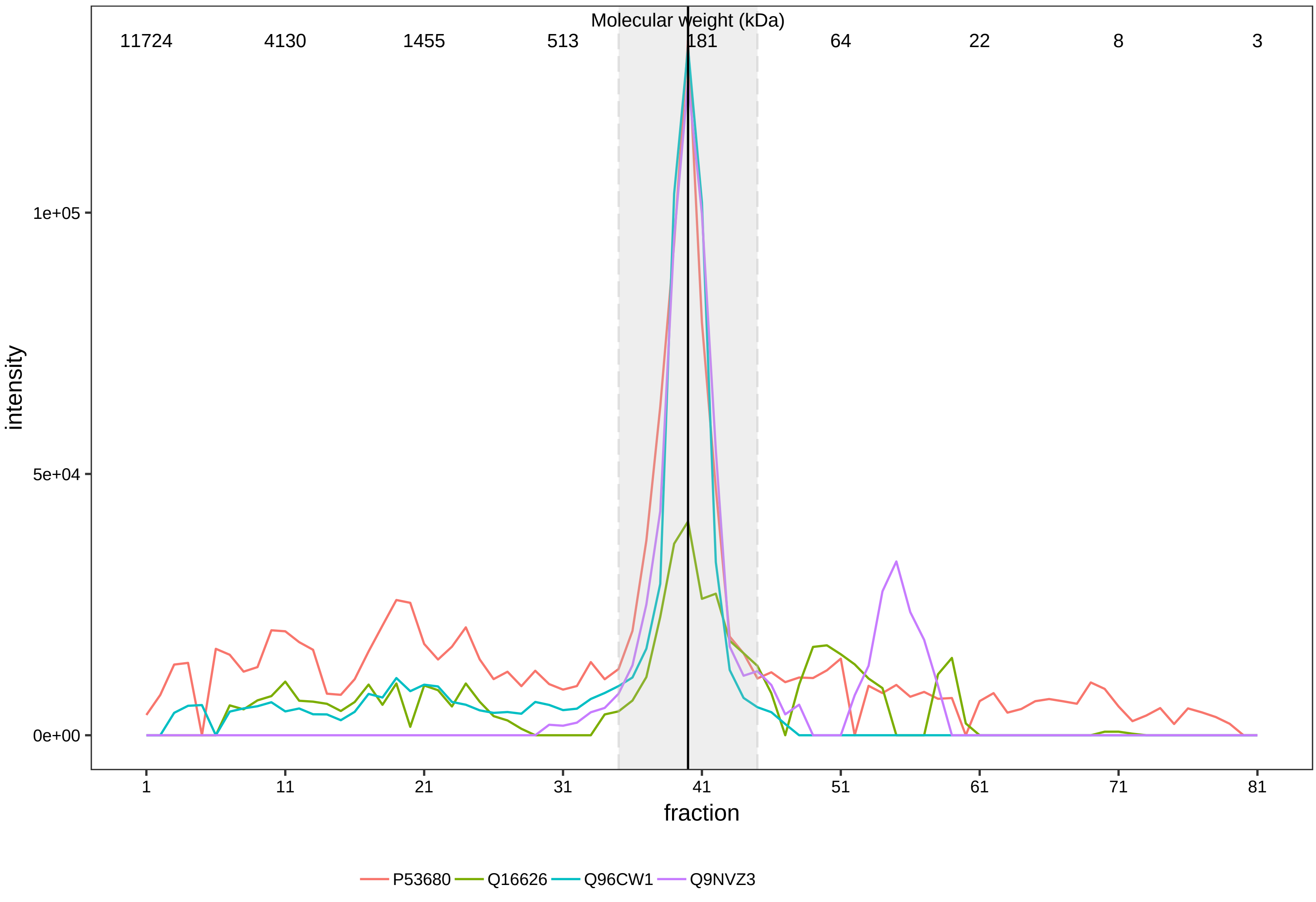

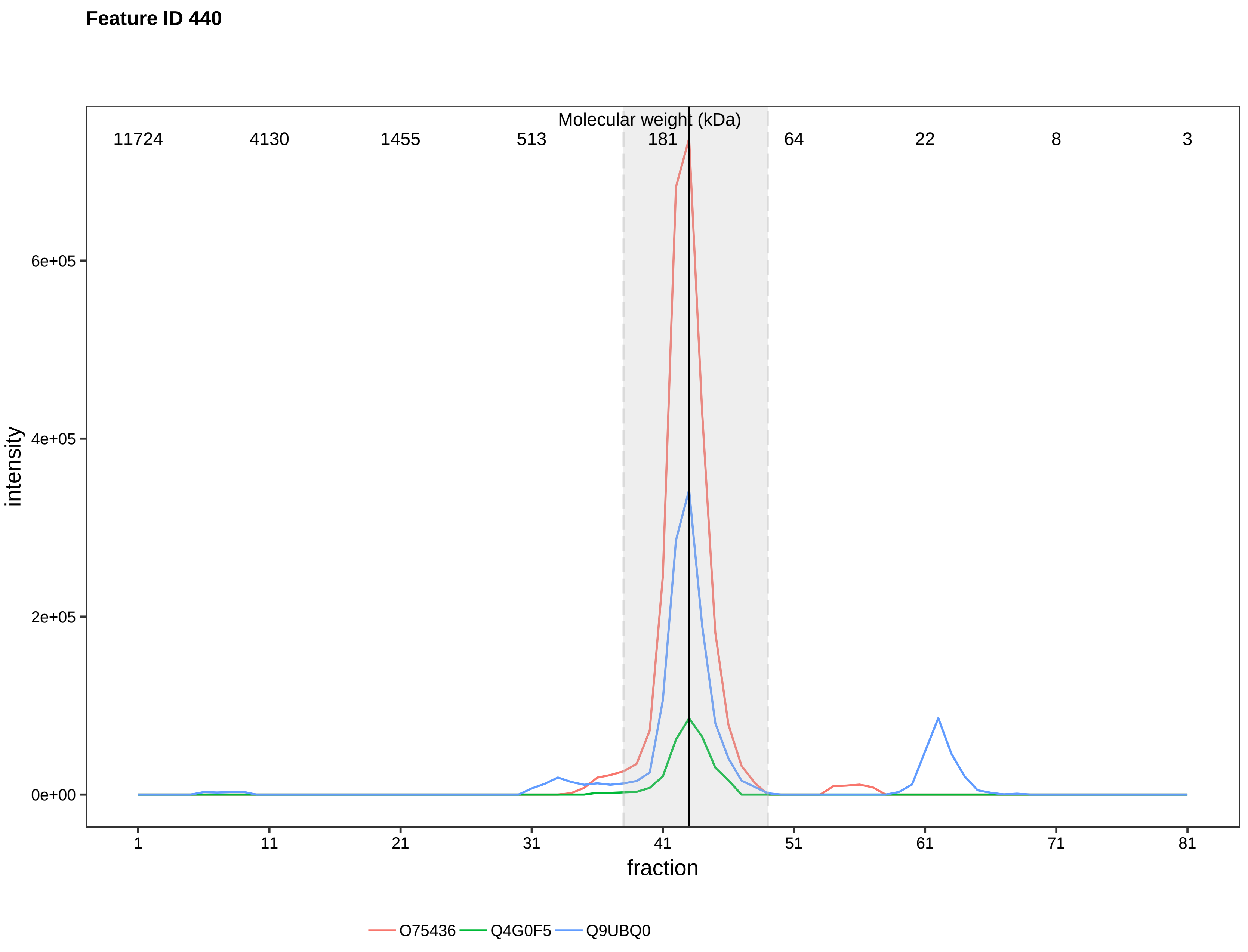

Feature ID 440
Molecular weight (kDa)
11724
4130
1455
513
181
64
22
8
3
6e+05
4e+05
2e+05
0e+00
intensity
1
11
21
31
41
51
61
71
81
fraction
O75436
Q4G0F5
Q9UBQ0

Feature ID 441

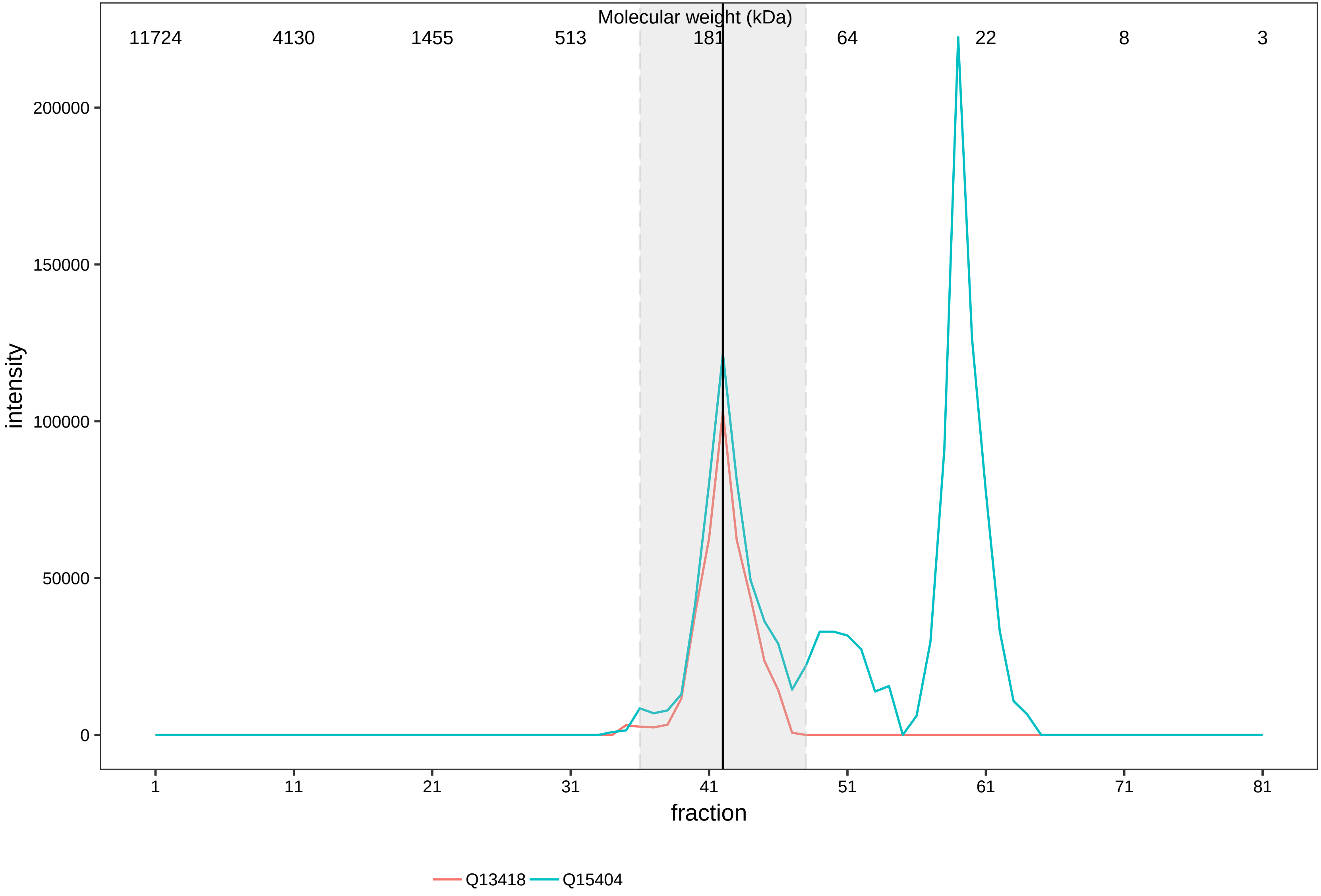

Feature ID 442

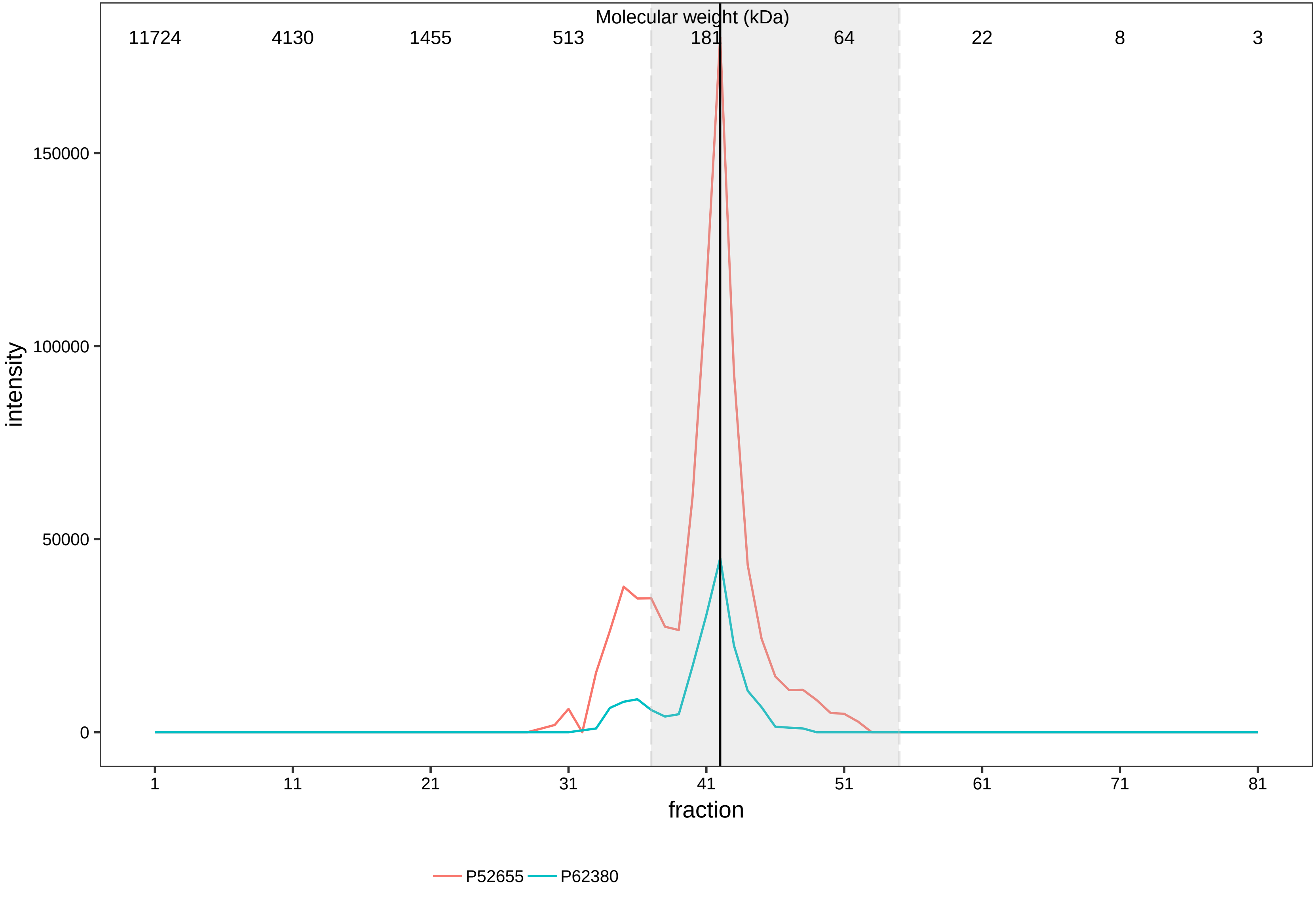

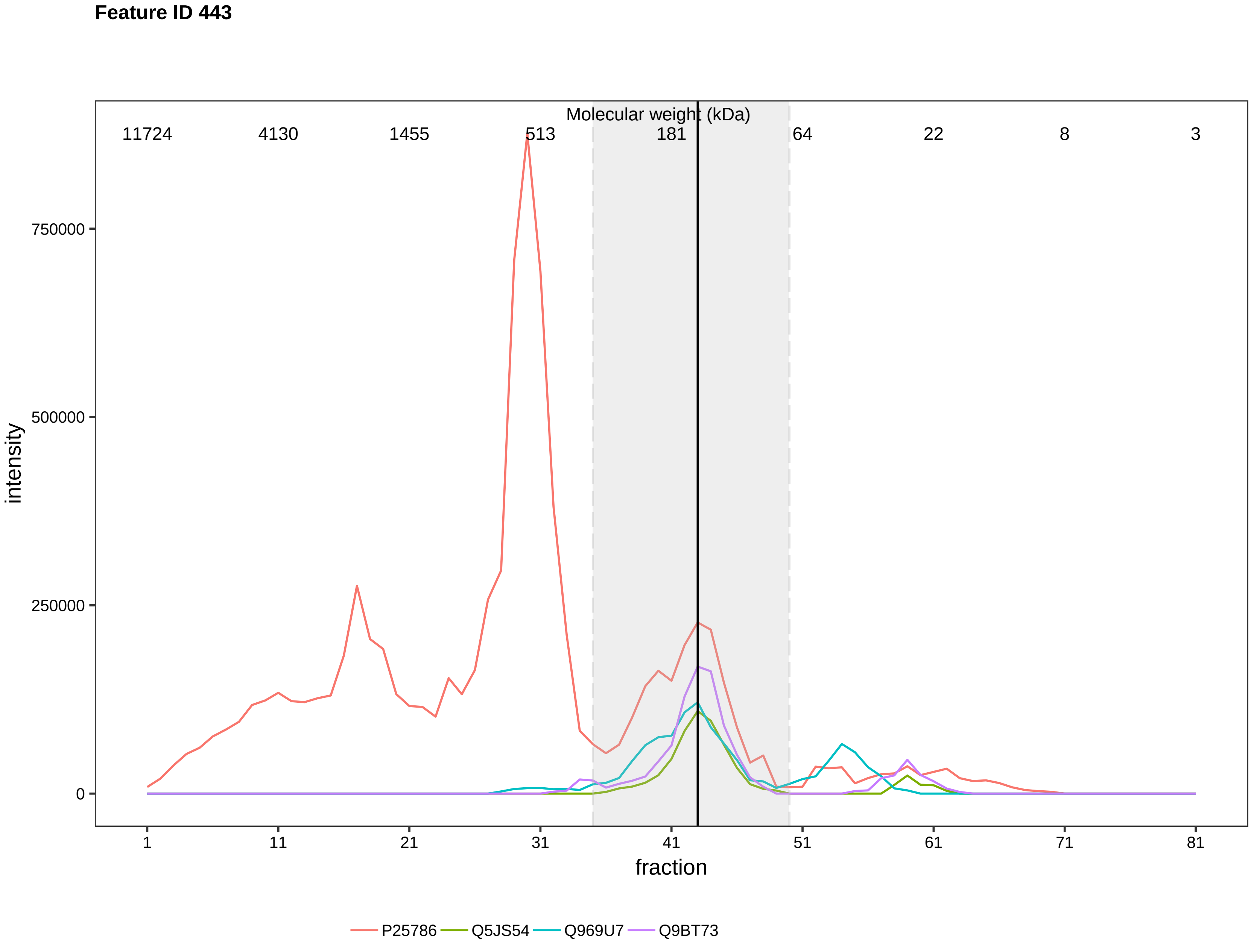

Feature ID 443
Molecular weight (kDa)
11724
4130
1455
513
181
64
22
8
3
750000
500000
250000
0
intensity
1
11
21
31
41
51
61
71
81
fraction
P25786
Q5JS54
Q969U7
Q9BT73

Feature ID 444

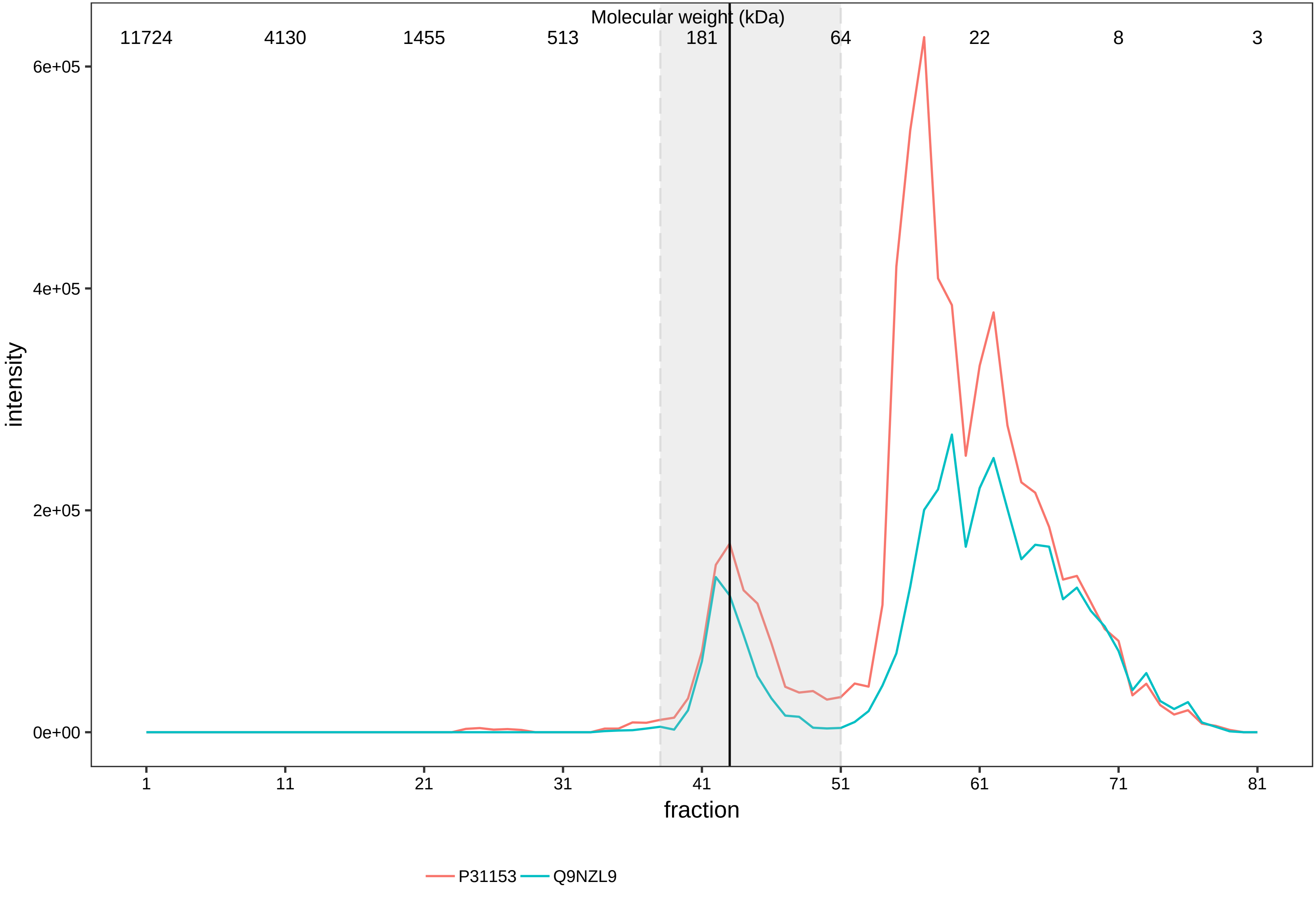

Feature ID 445

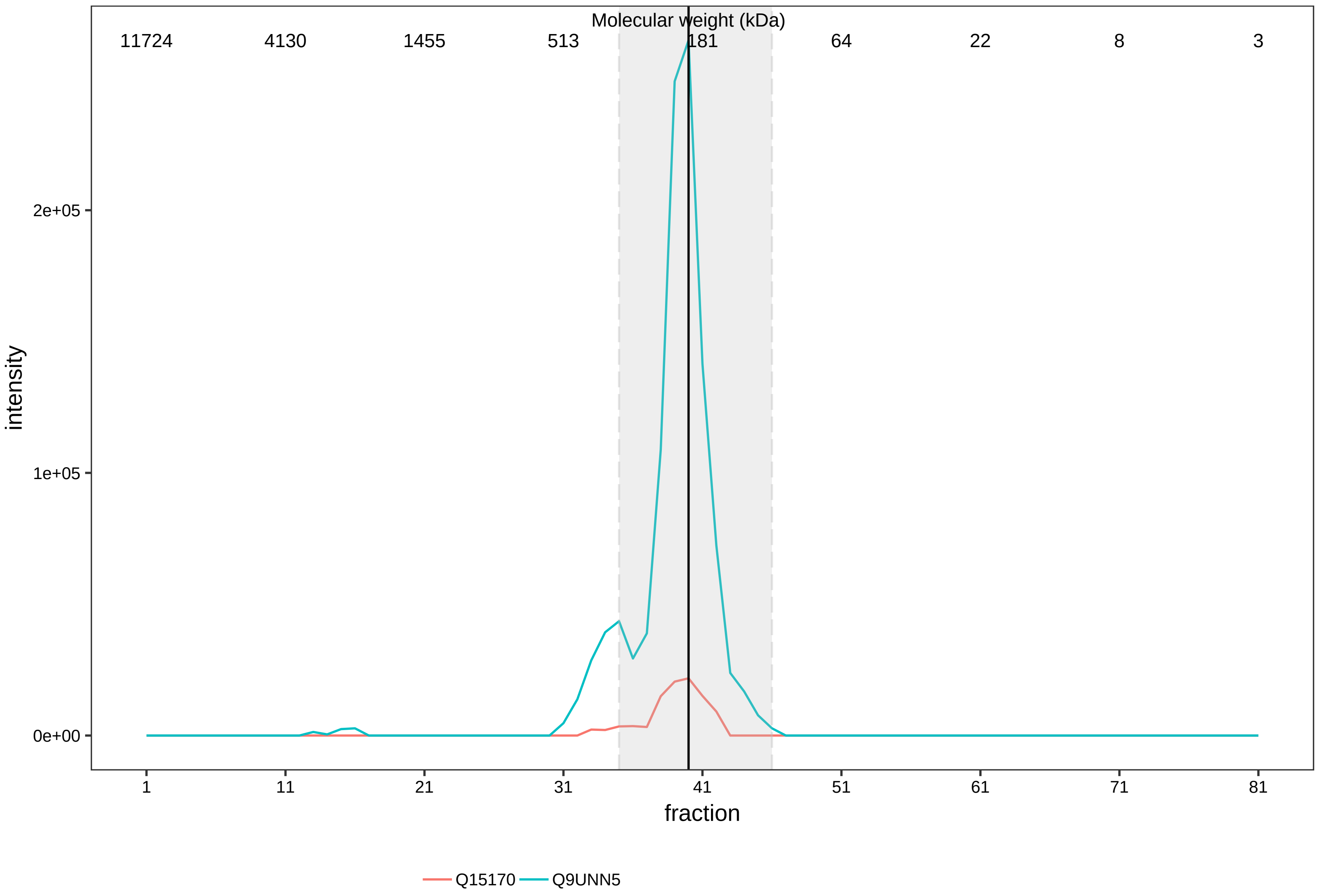

Feature ID 446

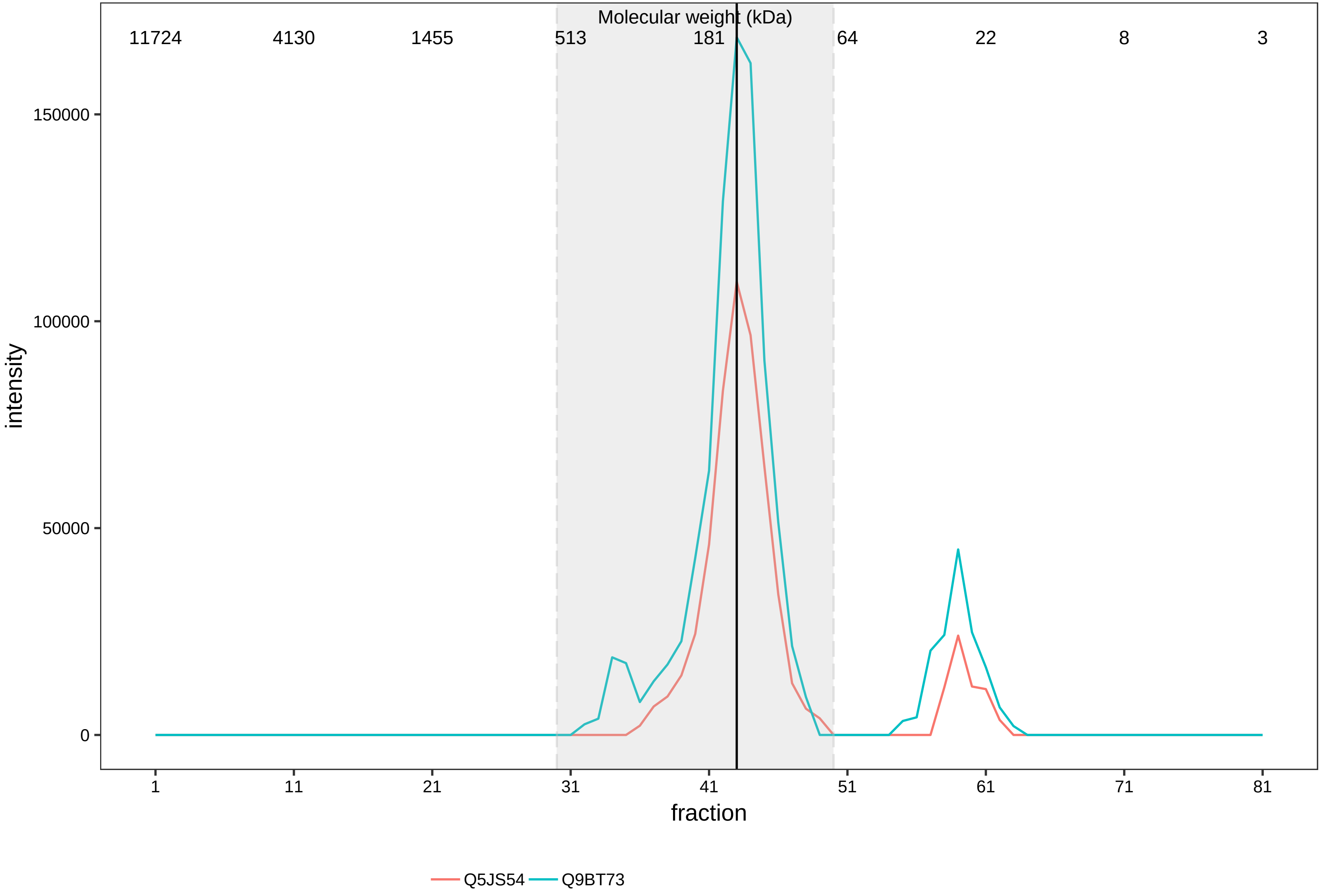

Feature ID 447

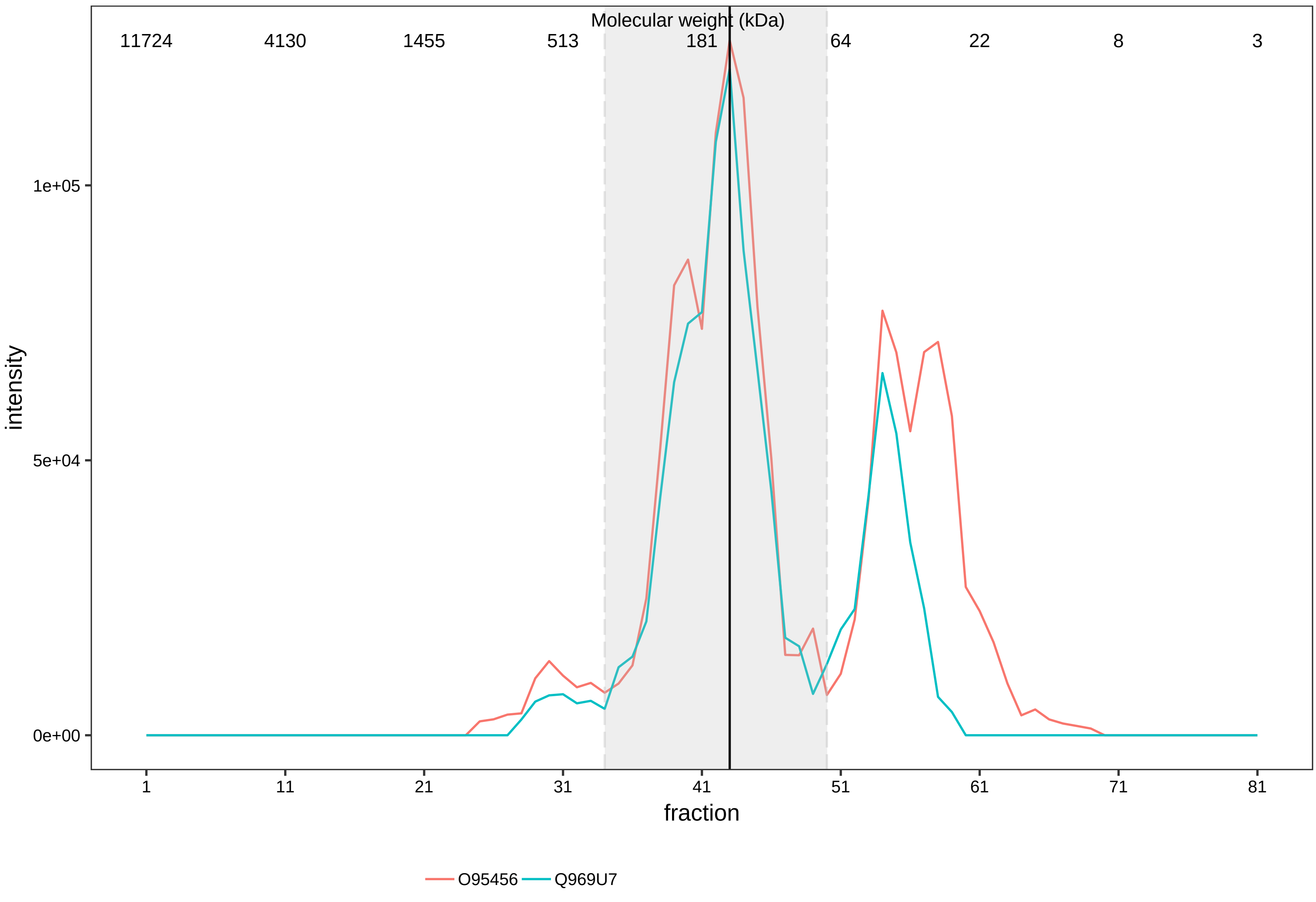

Feature ID 448

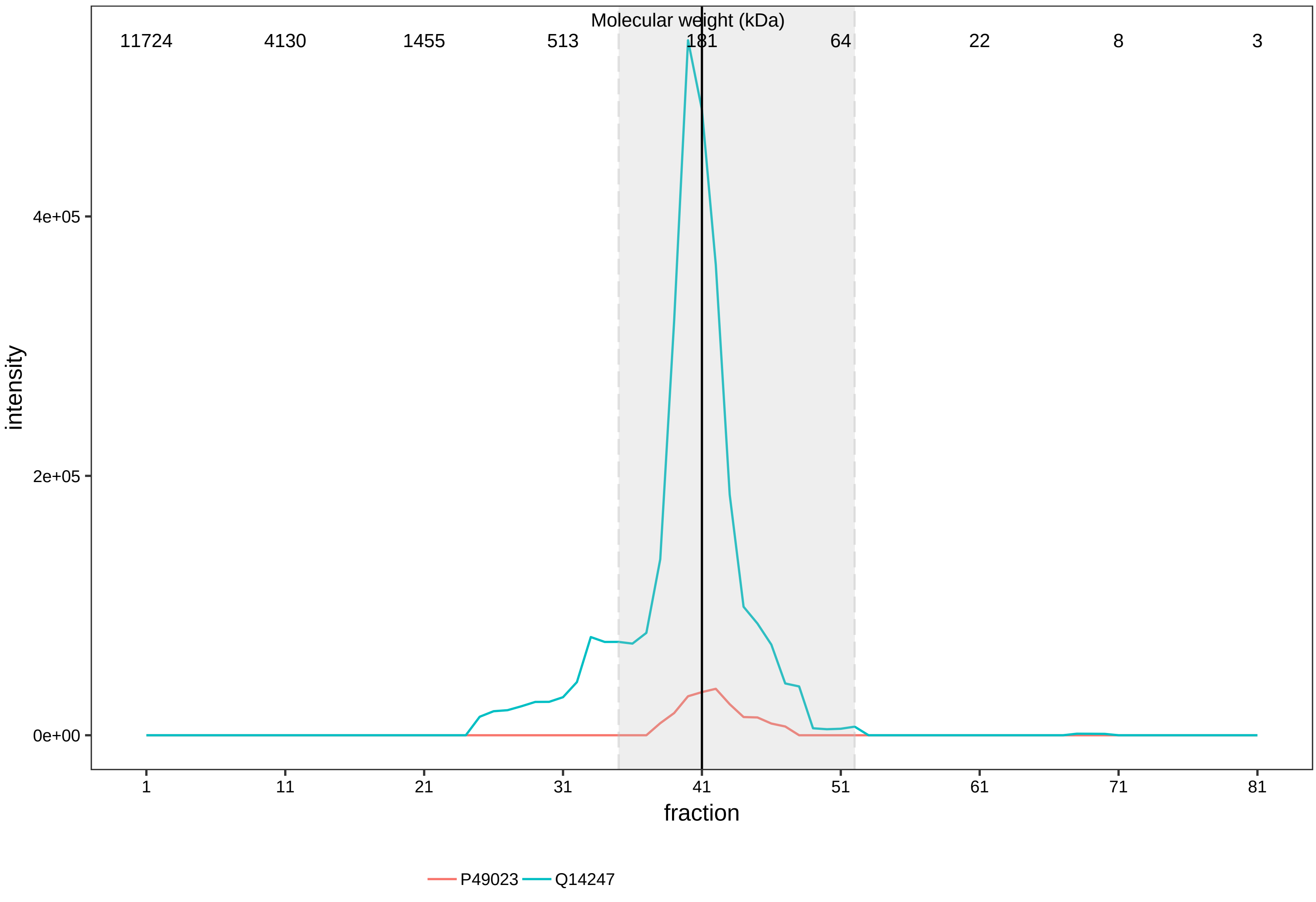

Feature ID 449

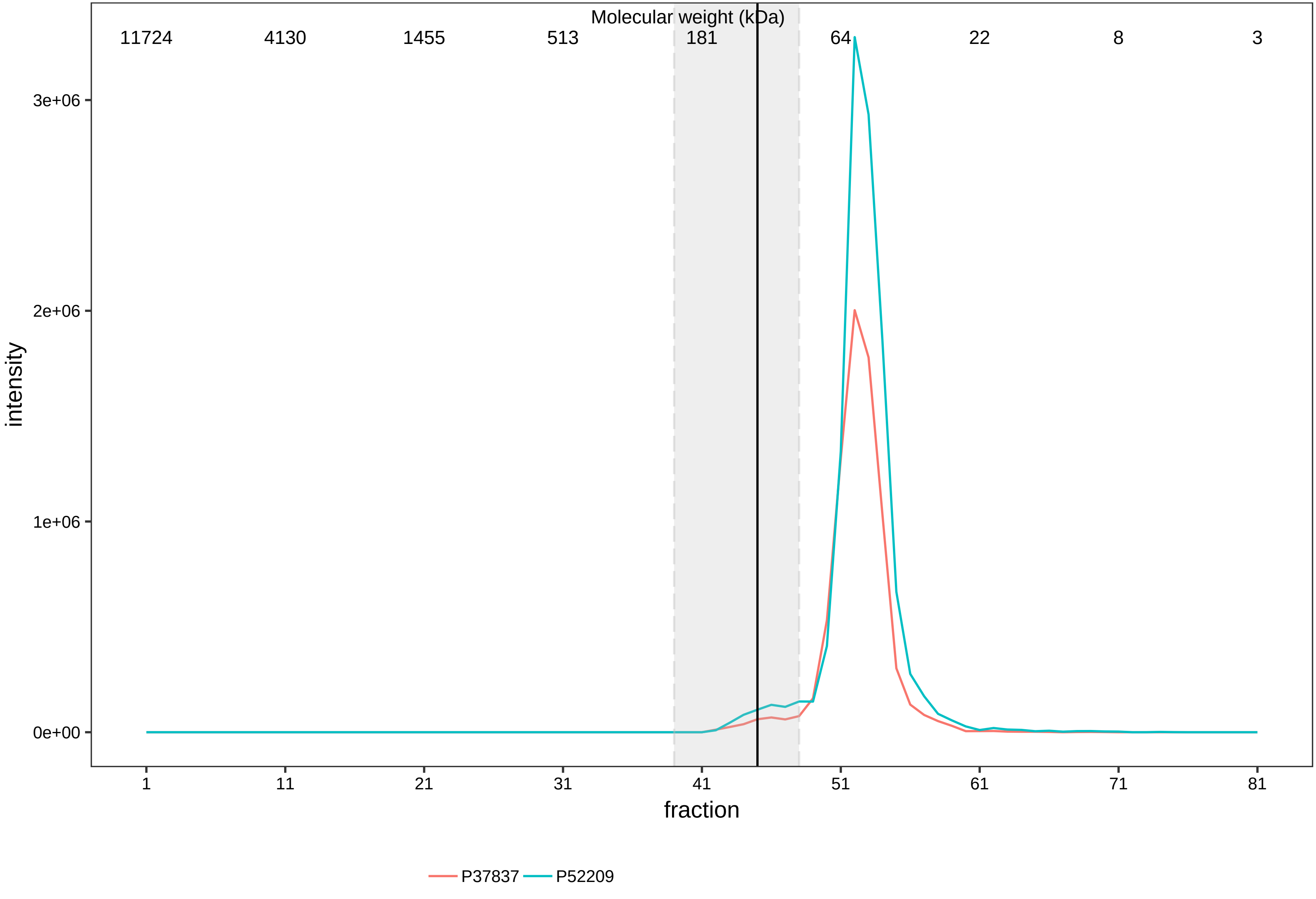

Feature ID 450

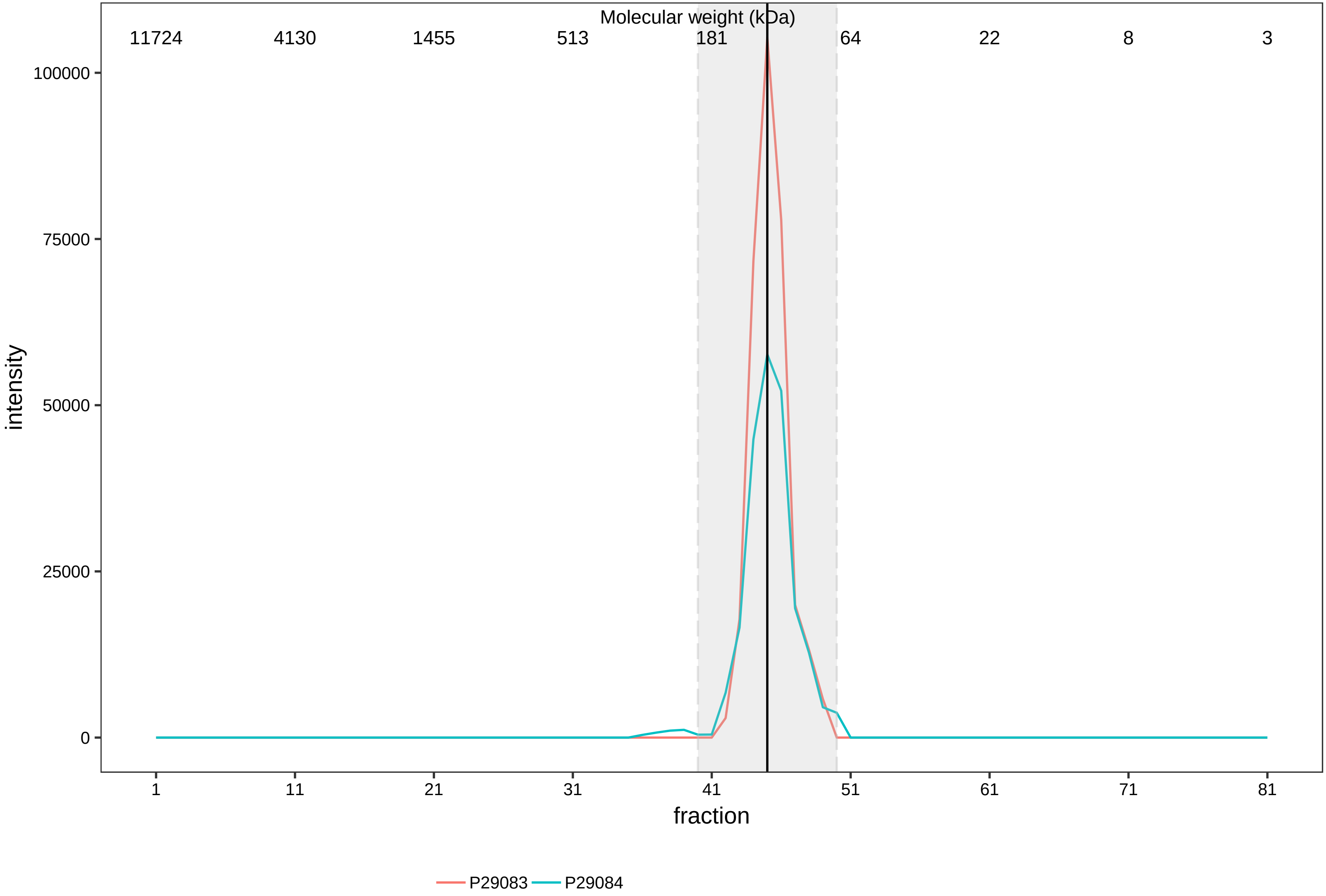

Feature ID 451

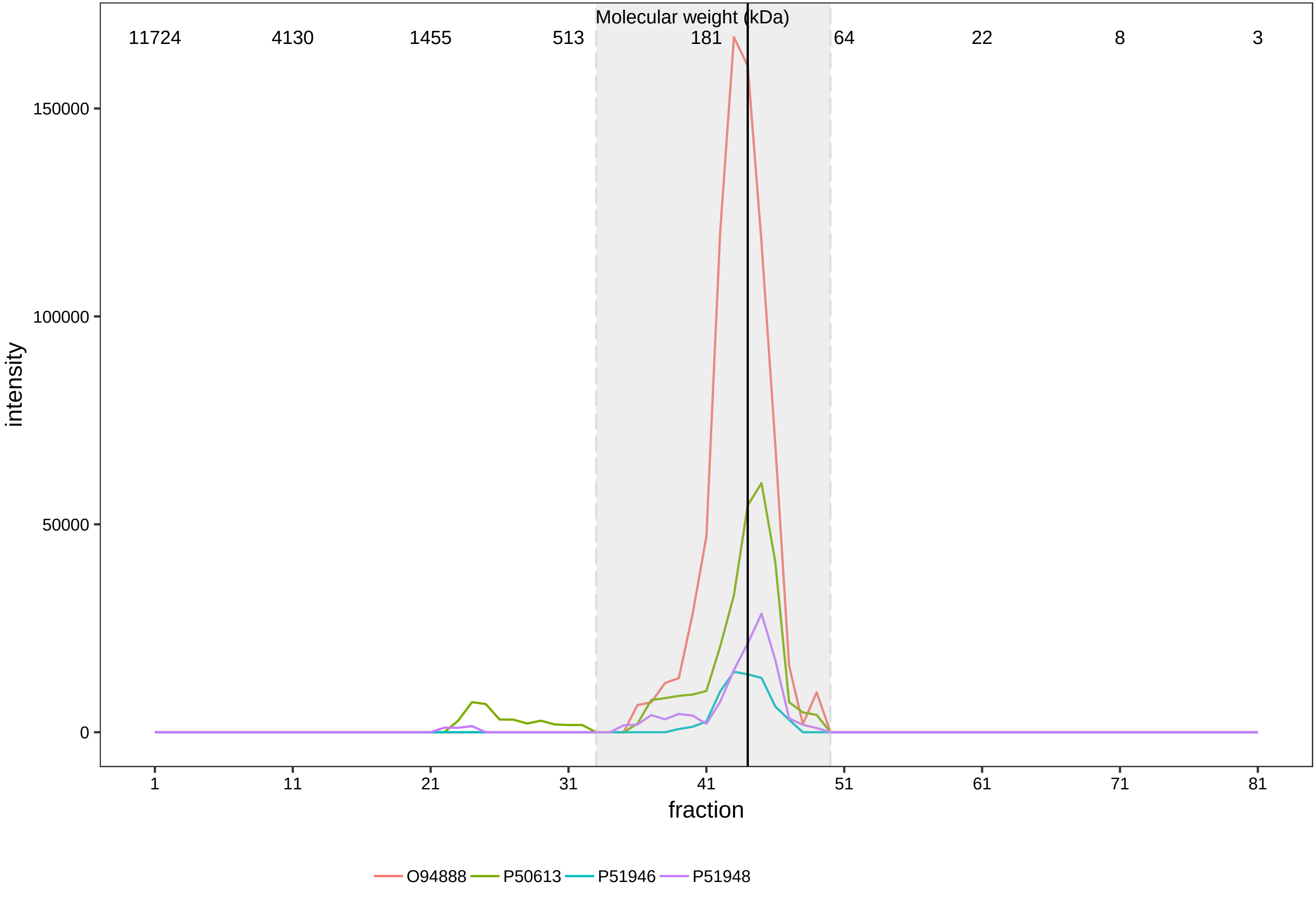

Feature ID 452

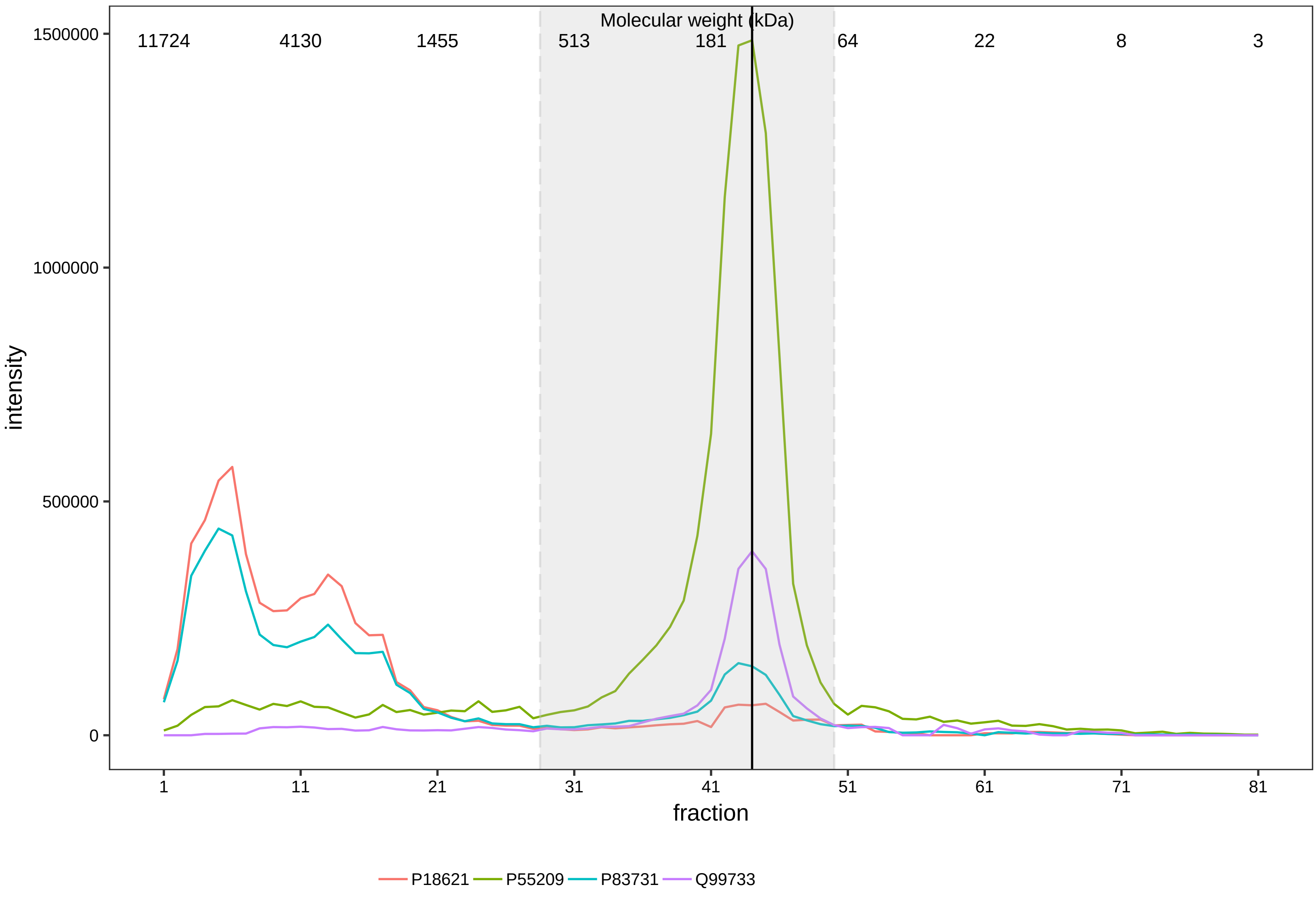

Feature ID 453

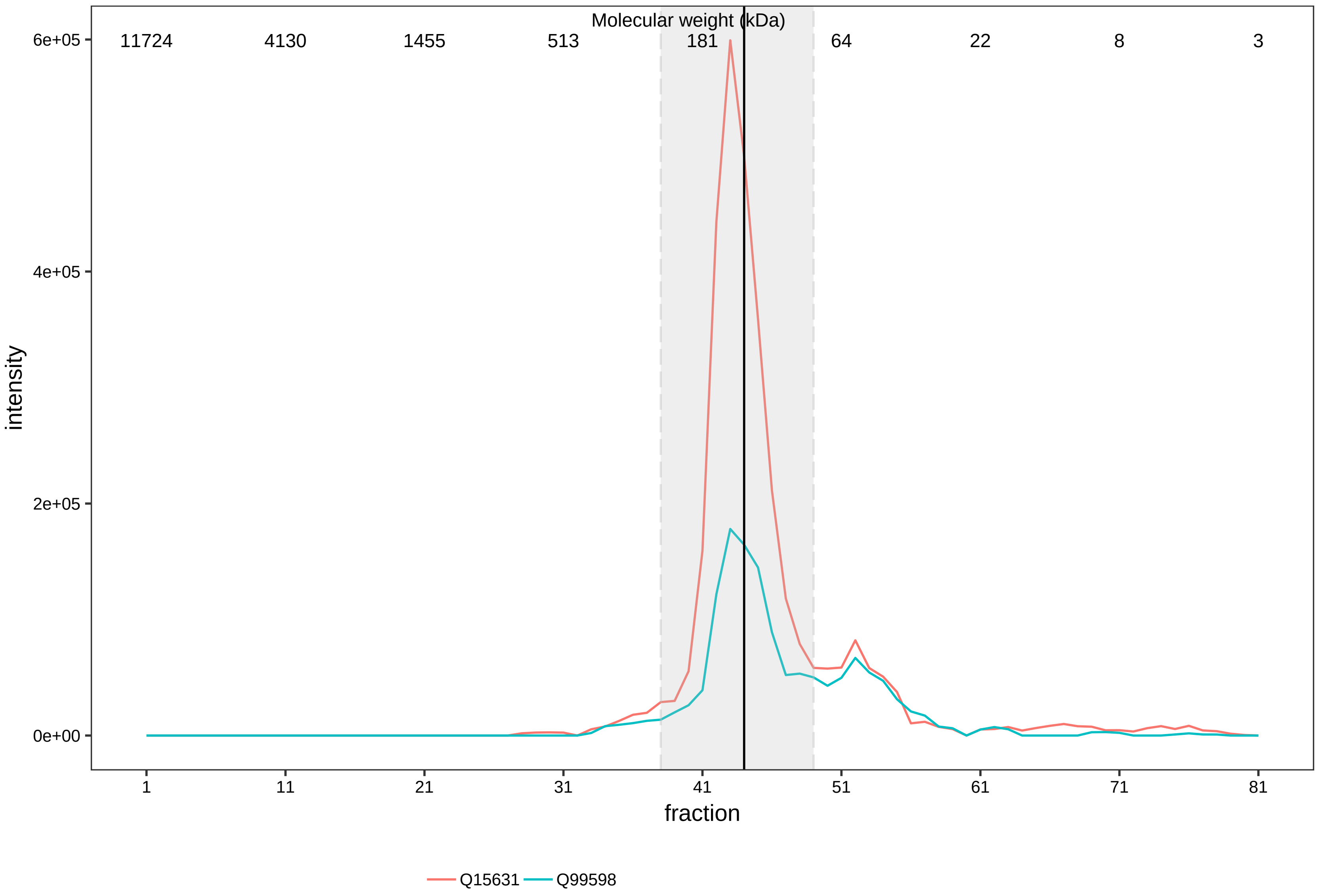

Feature ID 454

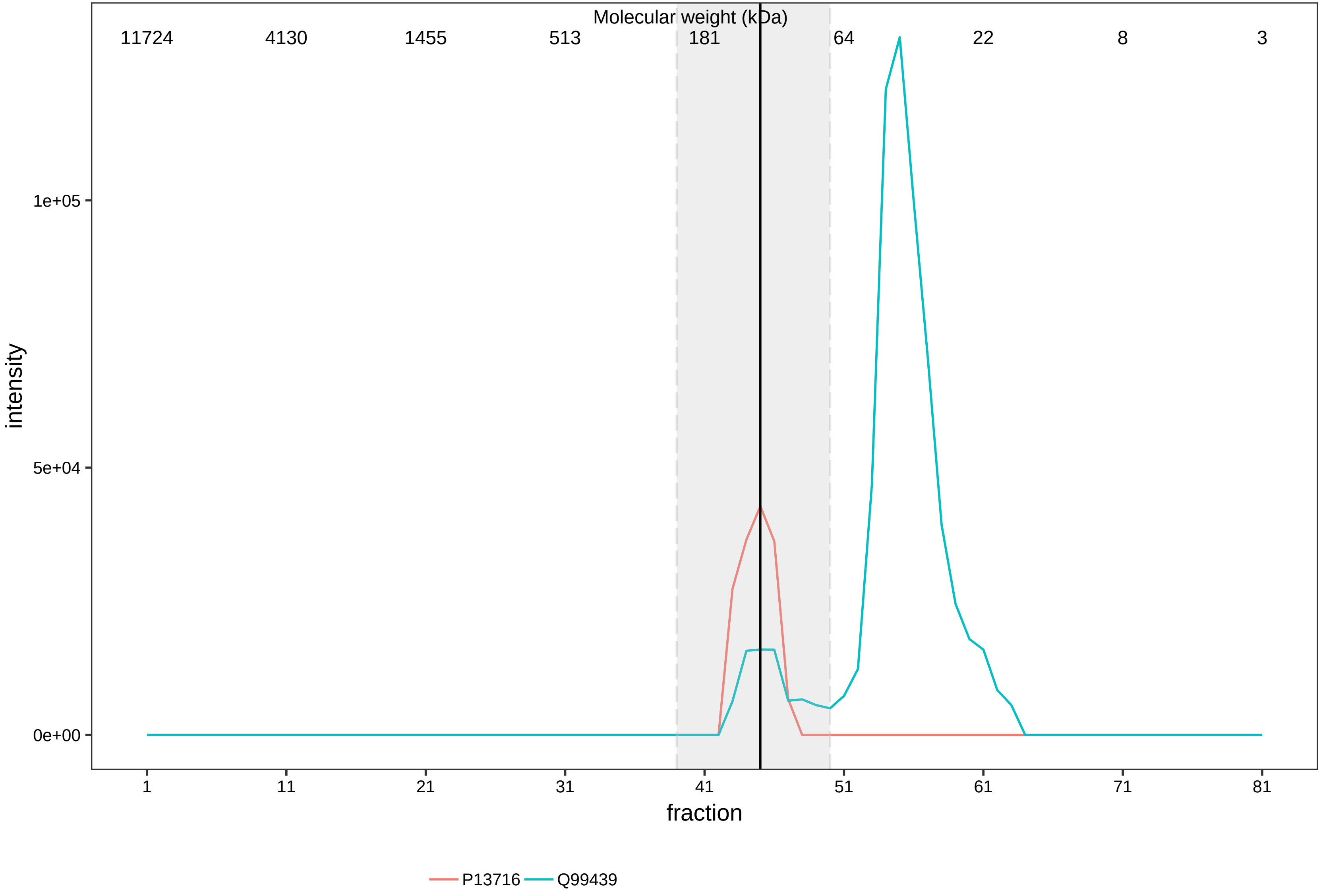

Feature ID 455

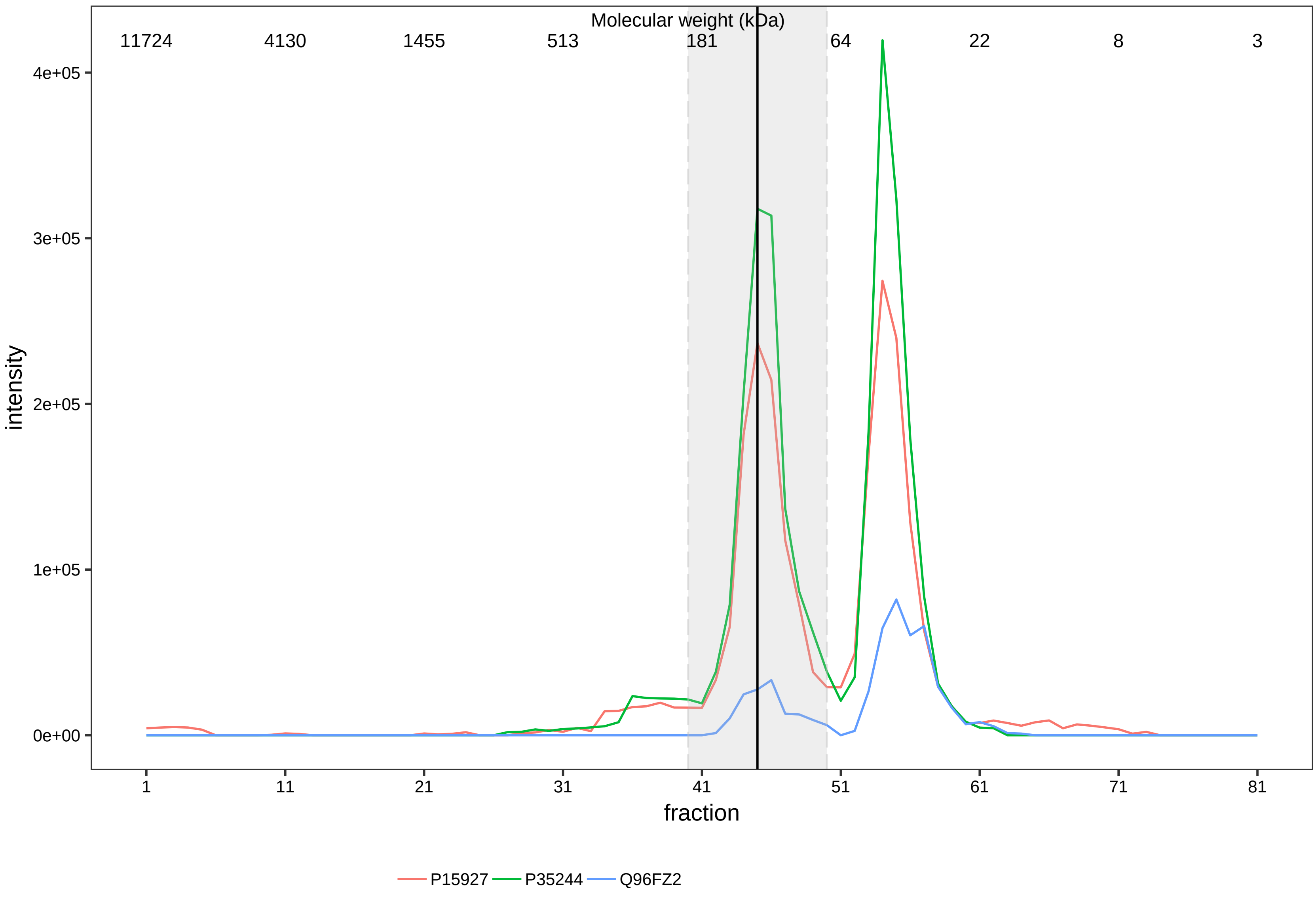

Feature ID 456

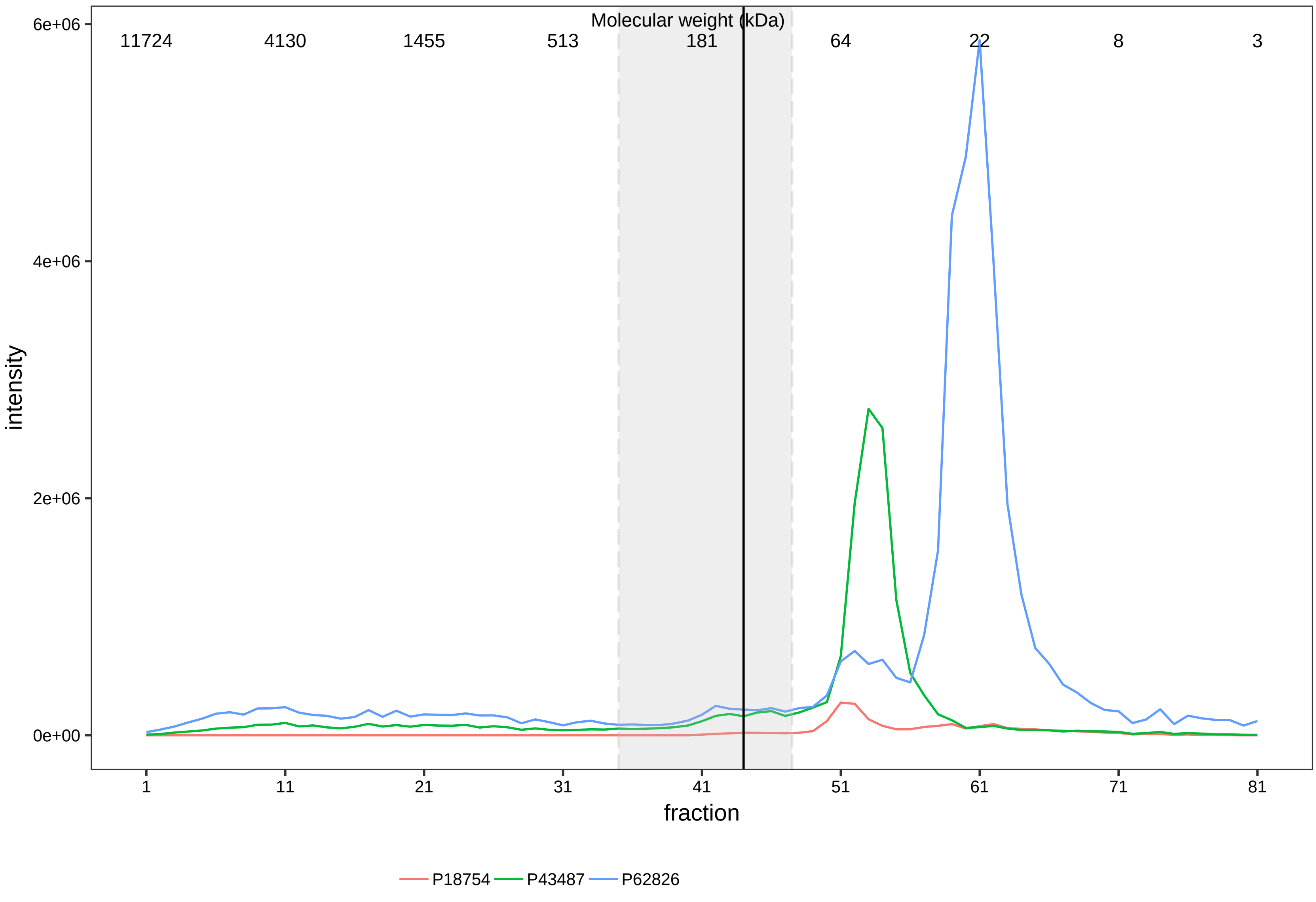

Feature ID 457

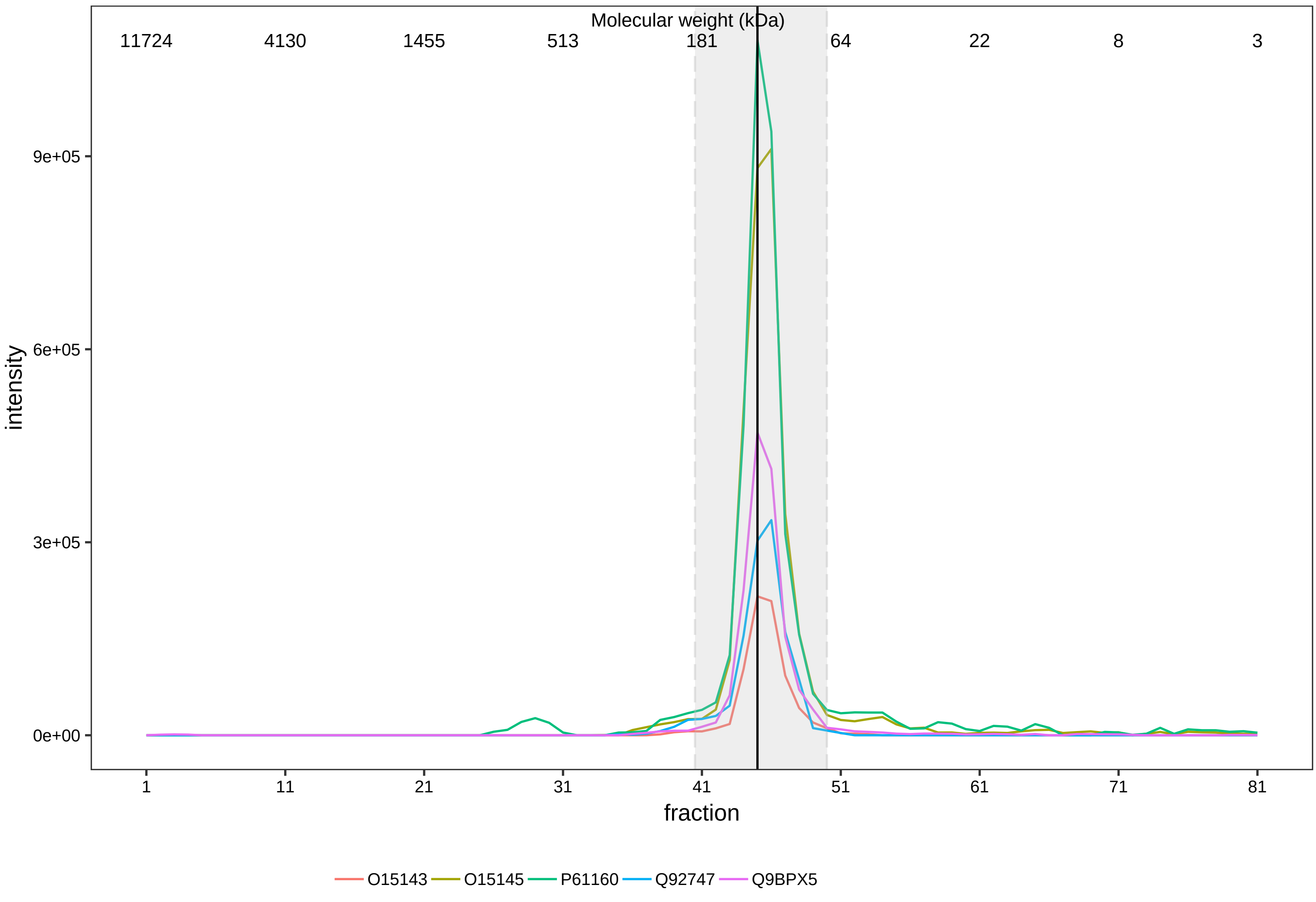

Feature ID 458

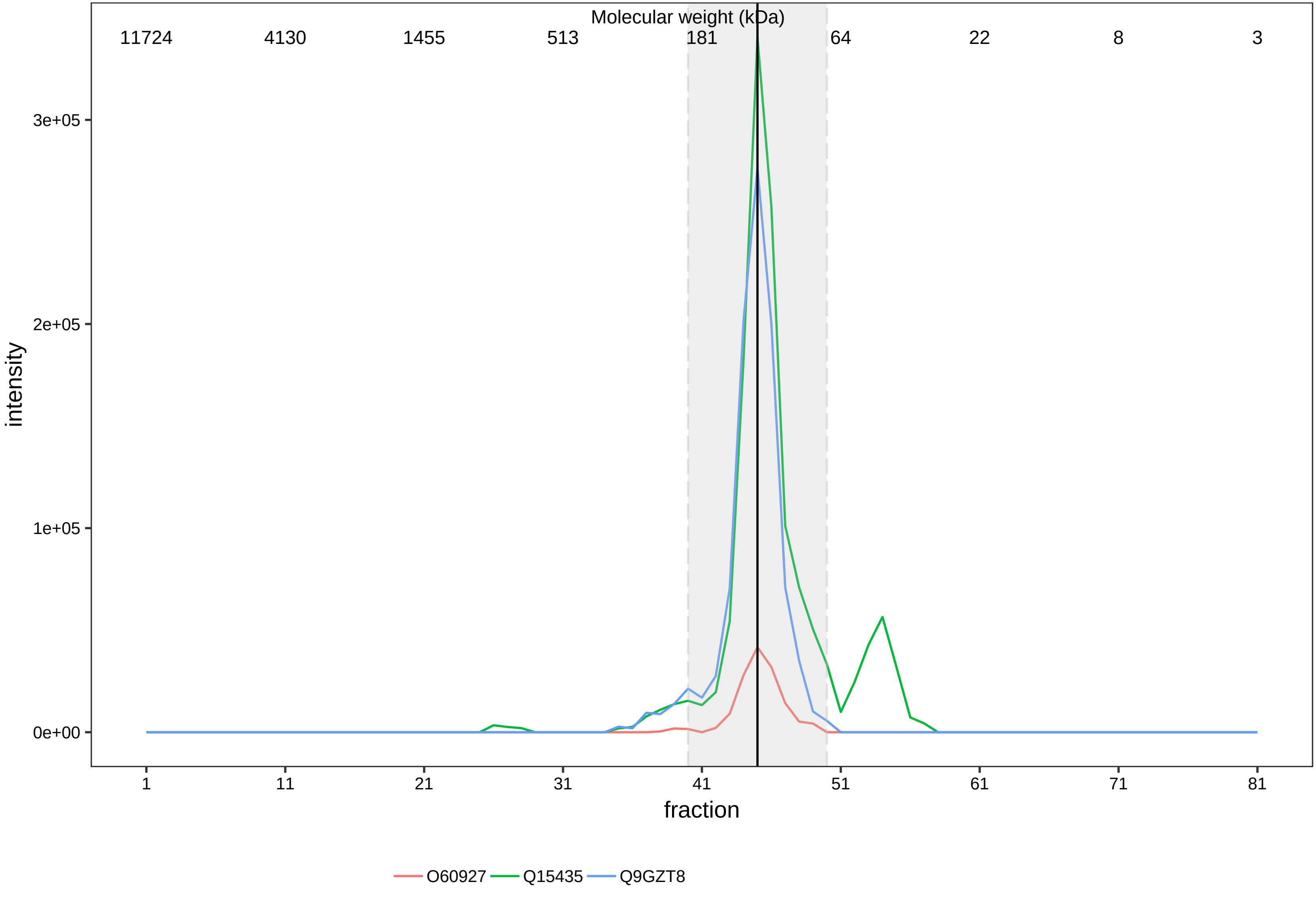

Feature ID 459

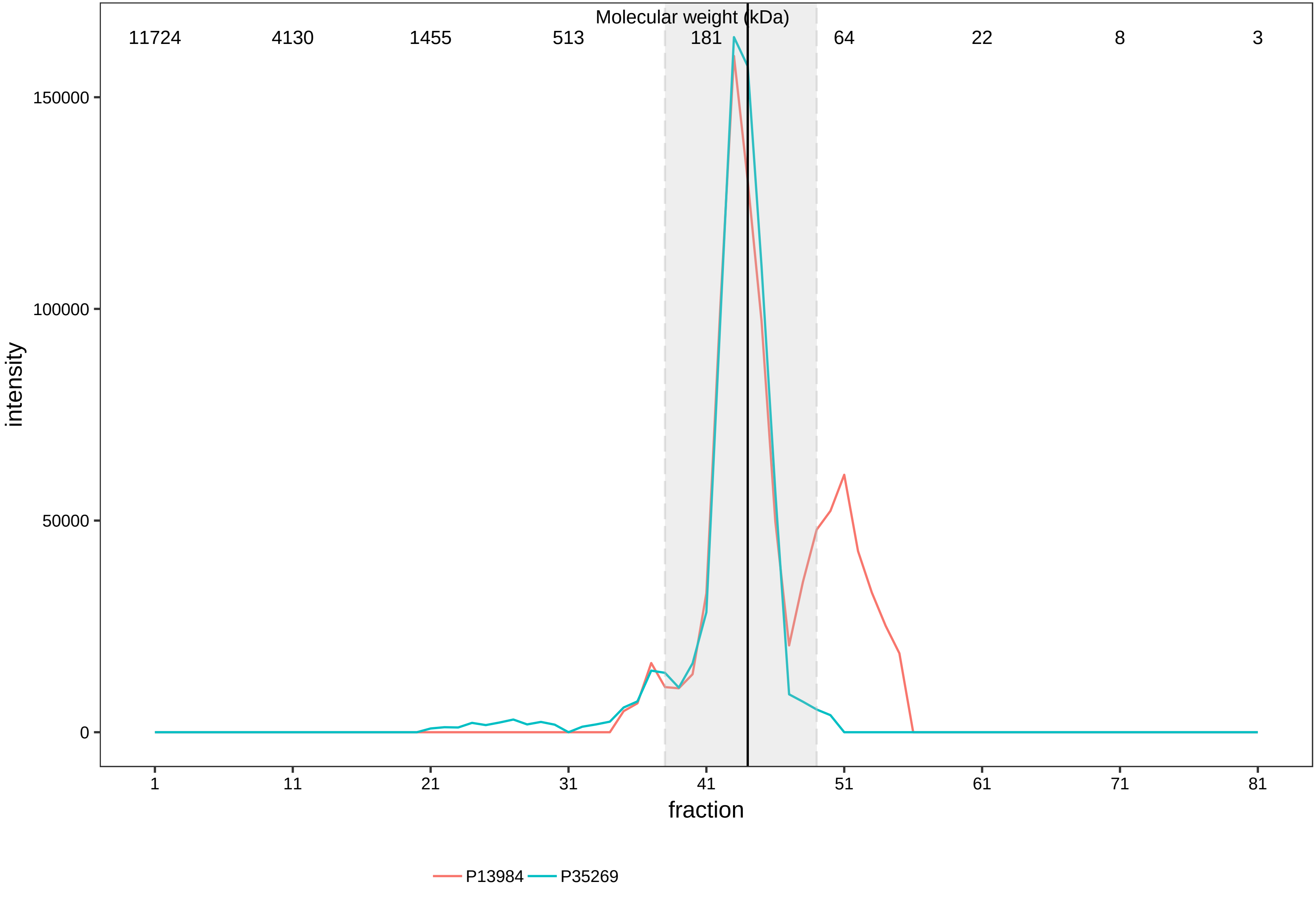

Feature ID 460

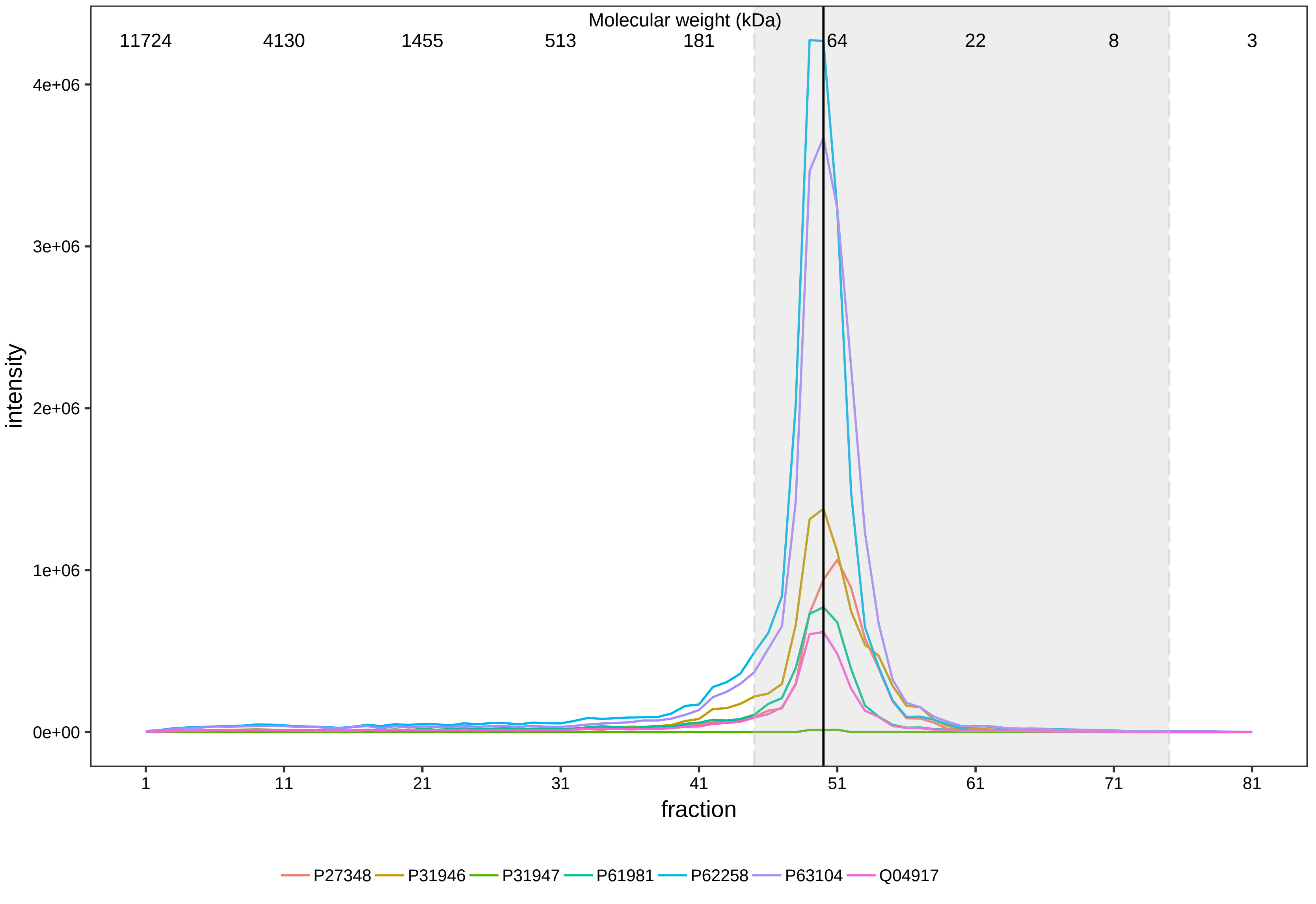

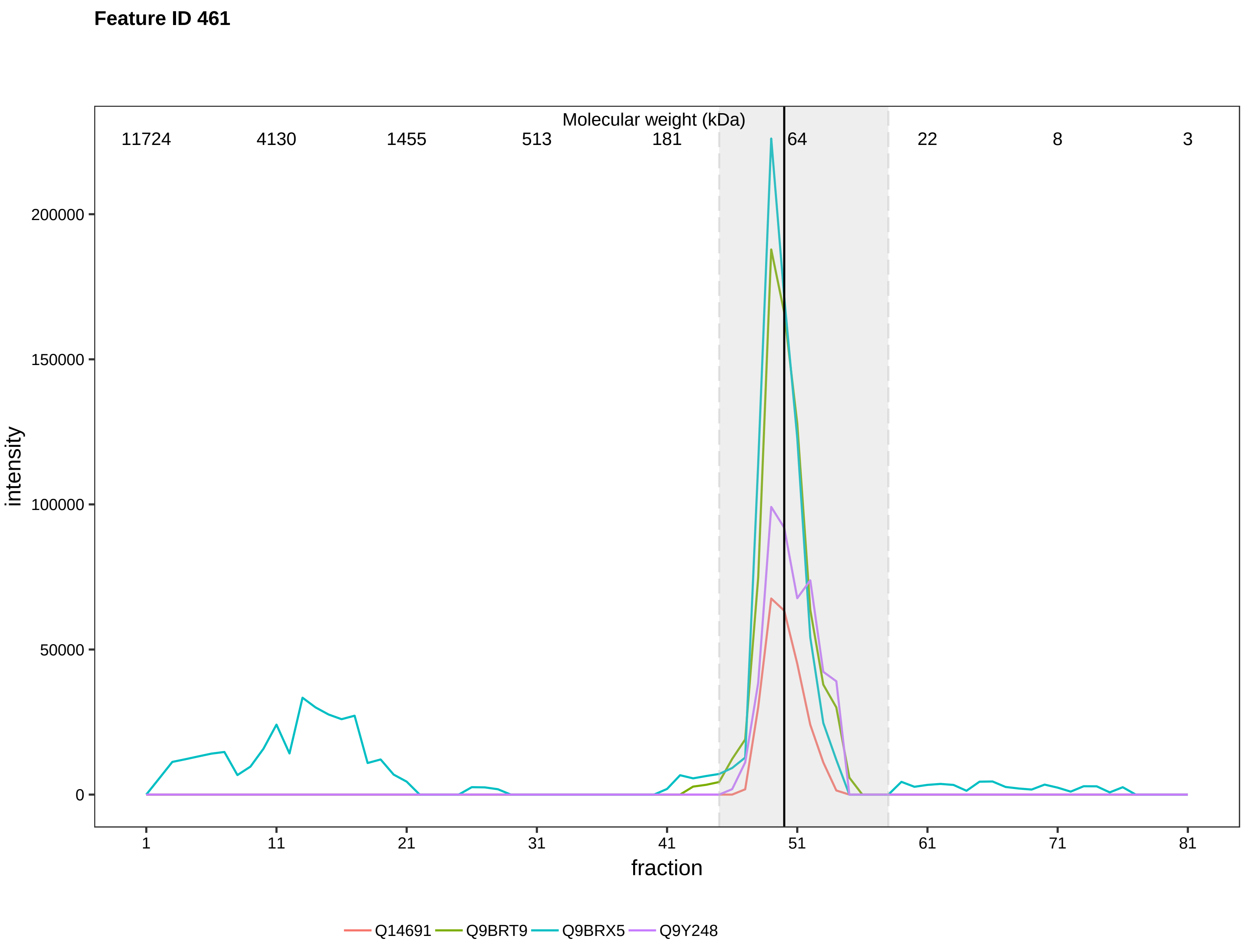

Feature ID 461
Molecular weight (kDa)
11724
4130
1455
513
181
64
22
8
3
intensity
200000
150000
100000
50000
0
1
11
21
31
41
51
61
71
81
fraction
Q14691
Q9BRT9
Q9BRX5
Q9Y248

Feature ID 462

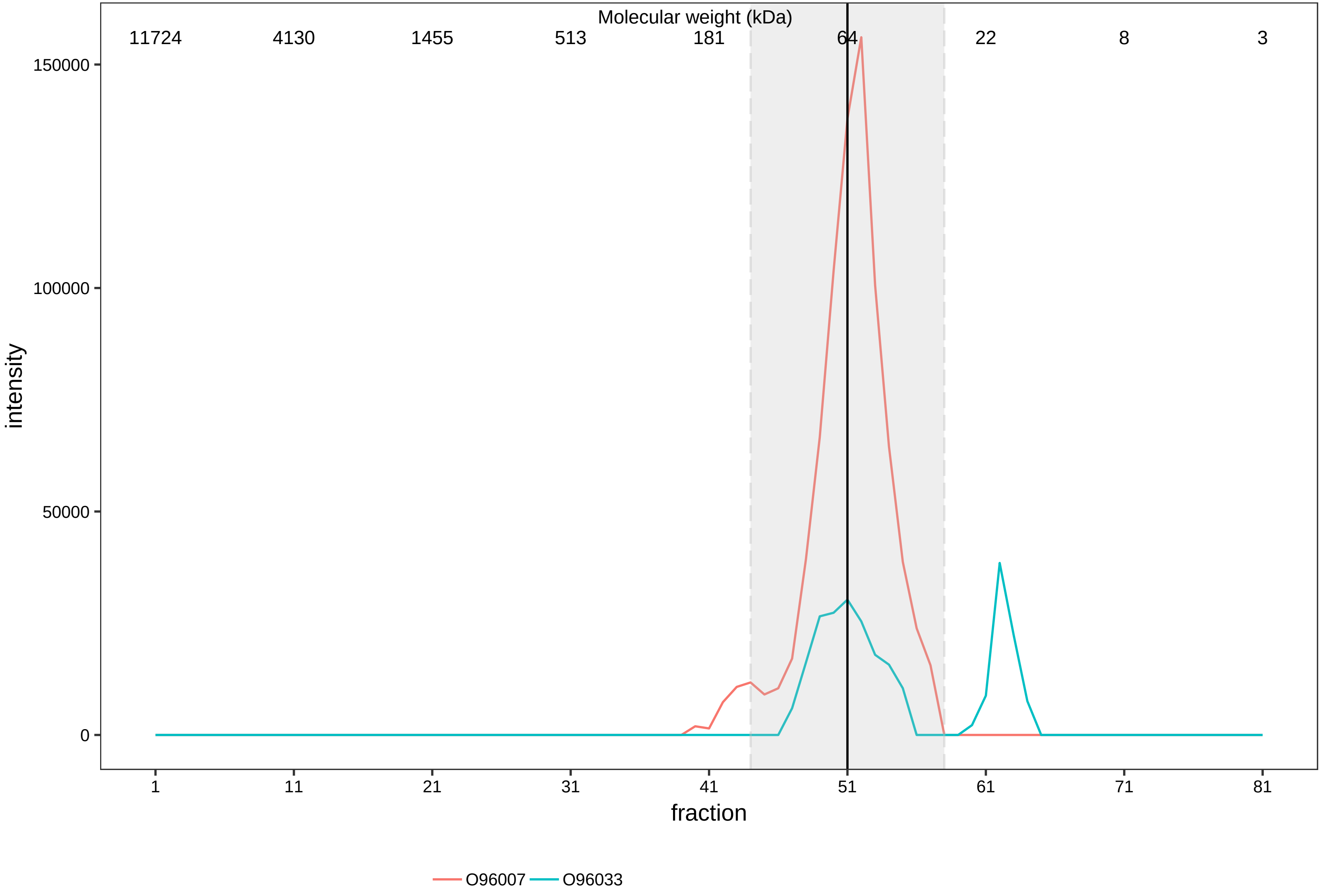

Supplement: Supplementary file 9 — Dataset EV8 [file MSB-15-e8438-s009.zip › Dataset_EV8_complexes_collapsed.pdf]
